# Dietary specialization drives multiple independent losses and gains in the bitter taste gene repertoire of Laurasiatherian Mammals

**DOI:** 10.1186/s12983-016-0161-1

**Published:** 2016-06-29

**Authors:** Zhijin Liu, Guangjian Liu, Frank Hailer, Pablo Orozco-terWengel, Xinxin Tan, Jundong Tian, Zhongze Yan, Baowei Zhang, Ming Li

**Affiliations:** Key laboratory of Animal Ecology and Conservation Biology, Institute of Zoology, Chinese Academy of Sciences, 1-5 Beichen West Road, Chaoyang, Beijing, 100101 China; School of Biosciences, Cardiff University, Sir Martin Evans Building, Museum Avenue, Cardiff, CF10 3AX UK; Institute of Health Sciences, Anhui University, Hefei, 230601 Anhui China; School of Life Sciences, Zhengzhou University, Zhengzhou, 450001 Henan China; School of Life Sciences, Anhui University, Hefei, 230601 Anhui China

**Keywords:** Laurasiatheria, Mammal, Bitter taste receptor, *TAS2R*, Gene gain and loss, Dietary specialization

## Abstract

**Background:**

Bitter taste perception is essential for species with selective food intake, enabling them to avoid unpalatable or toxic items. Previous studies noted a marked variation in the number of *TAS2R* genes among various vertebrate species, but the underlying causes are not well understood. Laurasiatherian mammals have highly diversified dietary niche, showing repeated evolution of specialized feeding preferences in multiple lineages and offering a unique chance to investigate how various feeding niches are associated with copy number variation for bitter taste receptor genes.

**Results:**

Here we investigated the evolutionary trajectories of *TAS2R*s and their implications on bitter taste perception in whole-genome assemblies of 41 Laurasiatherian species. The number of intact *TAS2R*s copies varied considerably, ranging from 0 to 52. As an extreme example of a narrow dietary niche, the Chinese pangolin possessed the lowest number of intact *TAS2R*s (*n* = 2) among studied terrestrial vertebrates. Marine mammals (cetacea and pinnipedia), which swallow prey whole, presented a reduced copy number of *TAS2R*s (*n* = 0-5). In contrast, independent insectivorous lineages, such as the shrew and insectivorous bats possessed a higher *TAS2R* diversity (*n* = 52 and *n* = 20-32, respectively), exceeding that in herbivores (*n* = 9-22) and omnivores (*n* = 18-22).

**Conclusions:**

Besides herbivores, insectivores in Laurasiatheria tend to have more functional *TAS2R*s in comparison to carnivores and omnivores. Furthermore, animals swallowing food whole (cetacean, pinnipedia and pangolin) have lost most functional *TAS2R*s. These findings provide the most comprehensive view of the bitter taste gene repertoire in Laurasiatherian mammals to date, casting new light on the relationship between losses and gains of *TAS2R*s and dietary specialization in mammals.

**Electronic supplementary material:**

The online version of this article (doi:10.1186/s12983-016-0161-1) contains supplementary material, which is available to authorized users.

## Background

The ability to perceive taste provides animals with important dietary information that can be crucial for their survival. Bitter taste perception is mediated by a group of G protein-coupled receptors, which are encoded by the type 2 taste receptor gene family (*TAS2R*s) [1]. Previous studies noted a marked variation in the number of *TAS2R* genes among various vertebrate species, but the underlying cause is not well understood [2, 3]. A study surveying bitter taste receptor genes in 52 vertebrate species (fish, birds, amphibians, reptiles and mammals) [4] reported that the number of *TAS2R* genes per species correlates with the proportion of plants in their diet. Furthermore, carnivores were found to have fewer *TAS2R* genes compared to herbivores and omnivores [5]. Because plant tissues contain more toxic compounds than animal tissues, these findings suggest that dietary toxins are a major selective force influencing the diversity of the *TAS2R* repertoire. Given this finding, it is reasonable to speculate that animals whose diet contains more toxins, besides herbivores, tend to have more functional *TAS2R*s, while animals with less dependence on bitter taste are likely to lose a certain number of functional *TAS2R*s. To test this hypothesis it is necessary to rely on a taxon with highly diversified and specialized dietary niches.

Over an estimated ~80 million years of diversification [6], Laurasiatherian mammals have evolved multiple lineage-specific feeding characteristics, such as the obligate ant-ingesting pangolins, the insectivorous shrews, carnivores (cats, dogs and allies), ungulate herbivores, herbivorous or insectivorous bats, and marine mammals that adopted a purely marine diet [7]. During the process of diet differentiation in Laurasiatheria, bitter taste preferences presumably evolved adaptively to avoid ingestion of indigestible or harmful compounds. In particular, there are several independent insectivorous lineages (shrews, insectivorous bats and so on). Eating insects is similar to herbivory, where detecting toxins is important. Many insects release defensive secretions that are toxic to their predators [8–10]. It could therefore be hypothesized that insectivores may be exposed to a similar amount of toxins as herbivores. Furthermore, there are also several independent lineages (cetaceans, pinnipeds and pangolins), which have evolved specialised foraging behaviour, ingesting prey whole. These species are likely to lose a certain number of functional *TAS2R*s due to less dependence on the taste perception. In summary, Laurasiatheria offers a unique chance to investigate how various feeding niches are associated with copy number variation for bitter taste receptor genes. Since the publication of Li and Zhang [4] additional vertebrate genomes have become available for study. Wang and Zhao [11] have reported a small repertoire of *TAS2R*s in birds based on a genomic survey including 48 birds. Furthermore, Hayakawa et al. [12] have described the frequent expansions of the *TAS2R*s in the Euarchontoglires clade, which is the sister clade of Laurasitherian mammals. However, less research has been carried out concerning the evolutionary history of the repertoire of *TAS2R*s in Laurasitheria. Here we use a comprehensive set of 41 Laurasitherian genomes encompassing all dietary niches across mammals and test the potential impact of all dietary types (herbivory, insectivory, carnivory and omnivory) and foraging behaviours (swallowing the prey whole or not) on the evolutionary scenarios of *TAS2R*s in Laurasiatherian mammals. The results showed that the diverse dietary characteristics in Laurasiatheria have had a larger impact in the *TAS2R*s repertoires than previously acknowledged.

## Results

### *TAS2R*s repertoires in Laurasiatheria

**Fig. 1:**
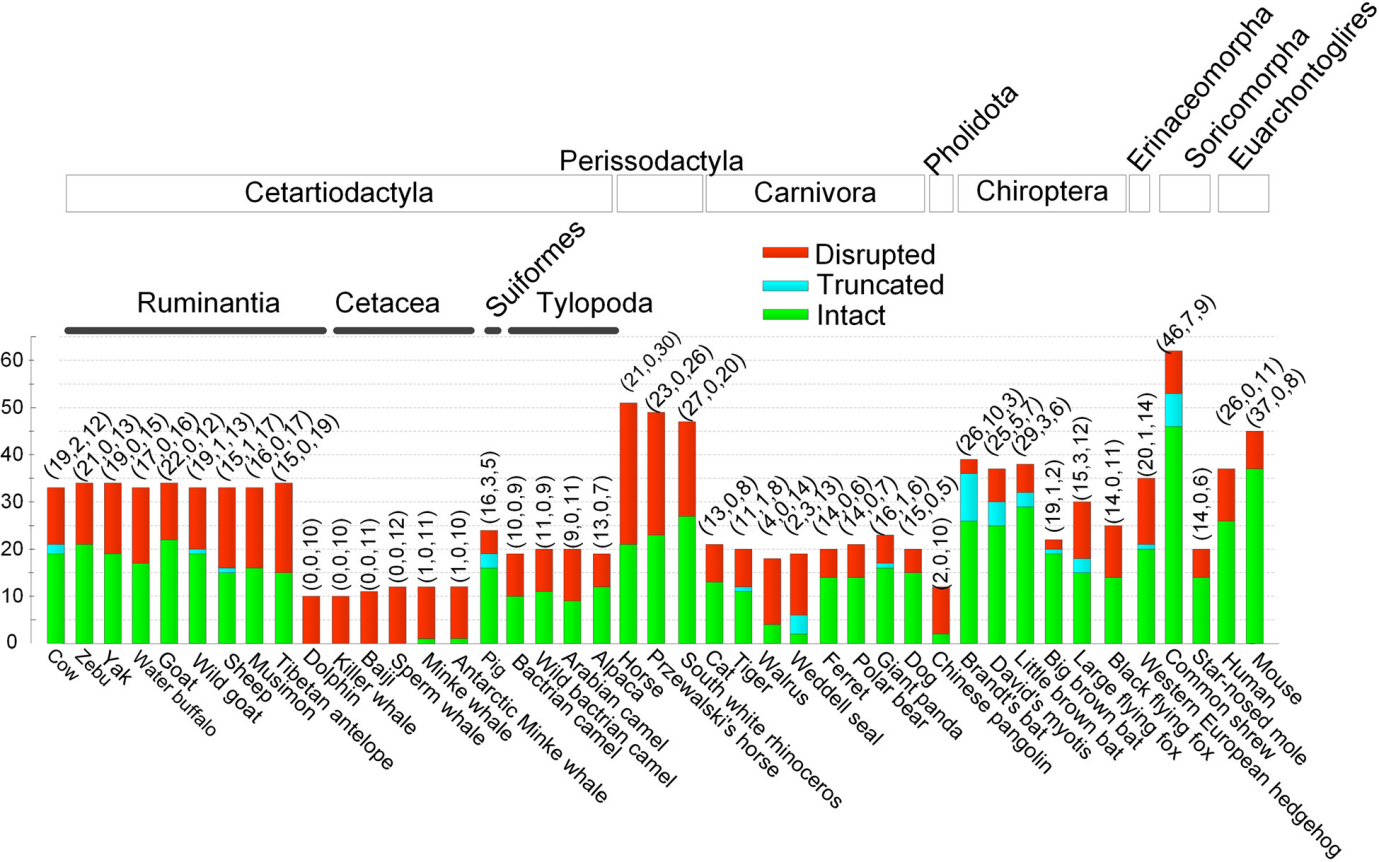
Numbers of complete, partial and disrupted *TAS2R*s in 41 Laurasiatherian mammals

**Fig. 2:**
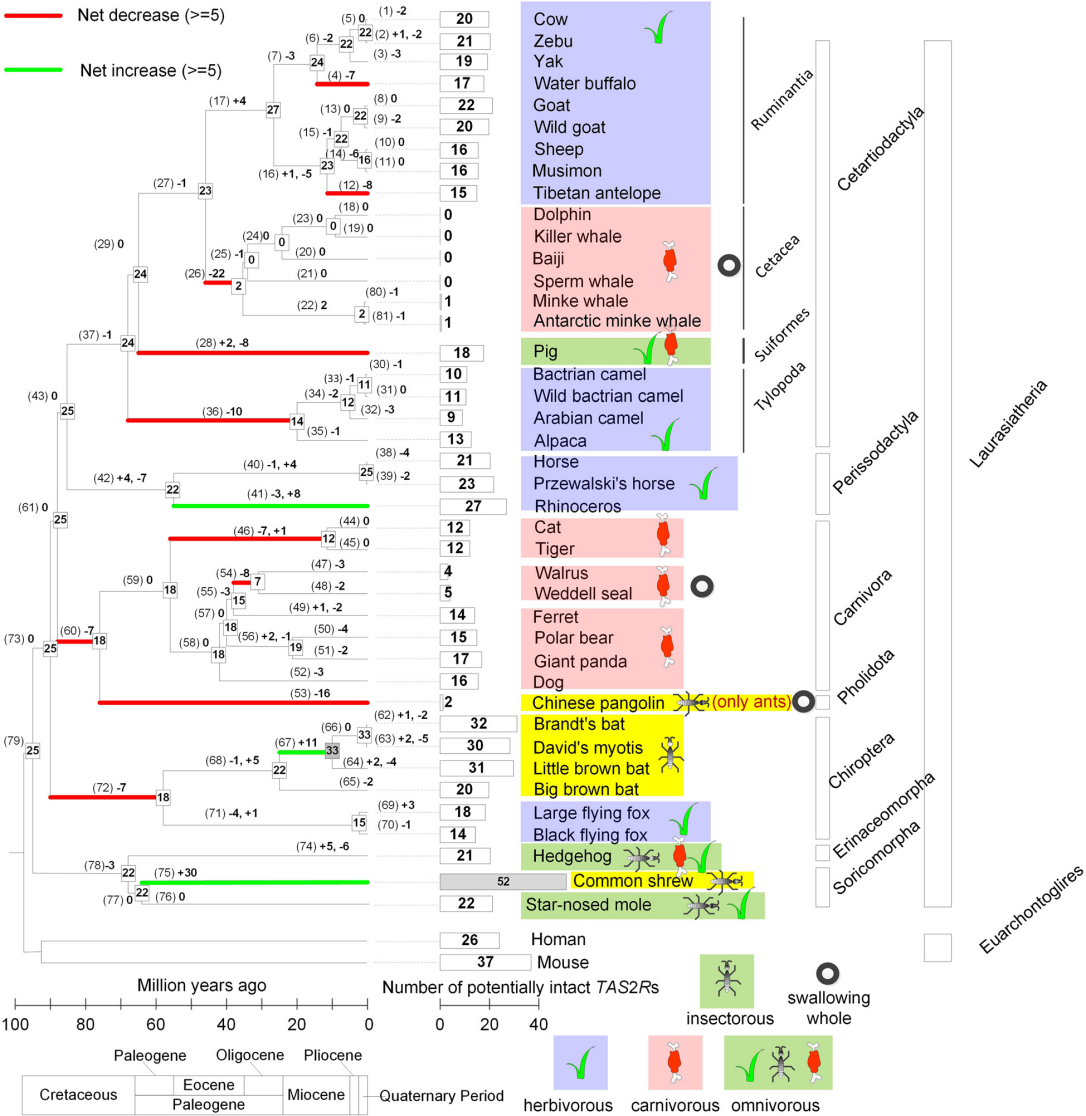
Evolutionary trajectories of *TAS2R*s in Laurasiatheria. Numbers next to nodes denote the number of intact *TAS2R*s (complete and partial ones). Numbers on branches denote the number of increases (caused by duplication of genes) and decreases (caused by pseudogenisation and whole-gene deletion). The bracketed ordinal numbers in this figure are used in electric supplementary material table S4, to describe the details of the birth-and-death evolution. To assess the phylogenetic relationships and divergence times of these species, we referred to TimeTree v3.0 [6] and Zhou et al. [39]

We analysed the genome assemblies of 41 mammals representing all seven orders in Laurasiatheria (Fig. 1, Additional file 1: Table S1). A total of 1,101 *TAS2R*s (594 complete, 43 partial and 473 disrupted *TAS2R*s) in these genome assemblies were annotated (Fig. 1), with the number of intact (both complete and partial copies) *TAS2R*s varying in the range from 0 to 52 (Figs. 1 and 2). The sequences and genomic locations of these *TAS2R*s are listed in the electronic supplementary dataset. Among all studied terrestrial Laurasiatherian mammals the average number of *TASR2s* was 18.7 (standard deviation, sd: 8.6), with the Chinese pangolin presenting the lowest number of intact *TAR2R*s (*n* = 2). Marine cetaceans (whale and dolphin) and pinnipedia (walrus and seal) also showed few intact *TAS2R*s (range: 0–5). The insectivorous shrew (*Sorex araneus*) showed the largest number of intact *TAS2R*s (*n* = 52). Similarly, insect-eating bats also had significantly higher numbers of intact *TAS2R*s (*n* = 20-32) compared to fruit-eating flying foxes (*n* = 14-18; Fishers Exact Test, *P* = 0.008). The numbers of intact *TAS2R*s in herbivorous ruminants, tylopods and perissodactyls ranged from 15 to 20, 9–13 and 21–27, respectively. The terrestrial Carnivora (excluding walrus and seal) had 12–17 functional *TAS2R*s (Fig. 2).

**Fig. 3:**
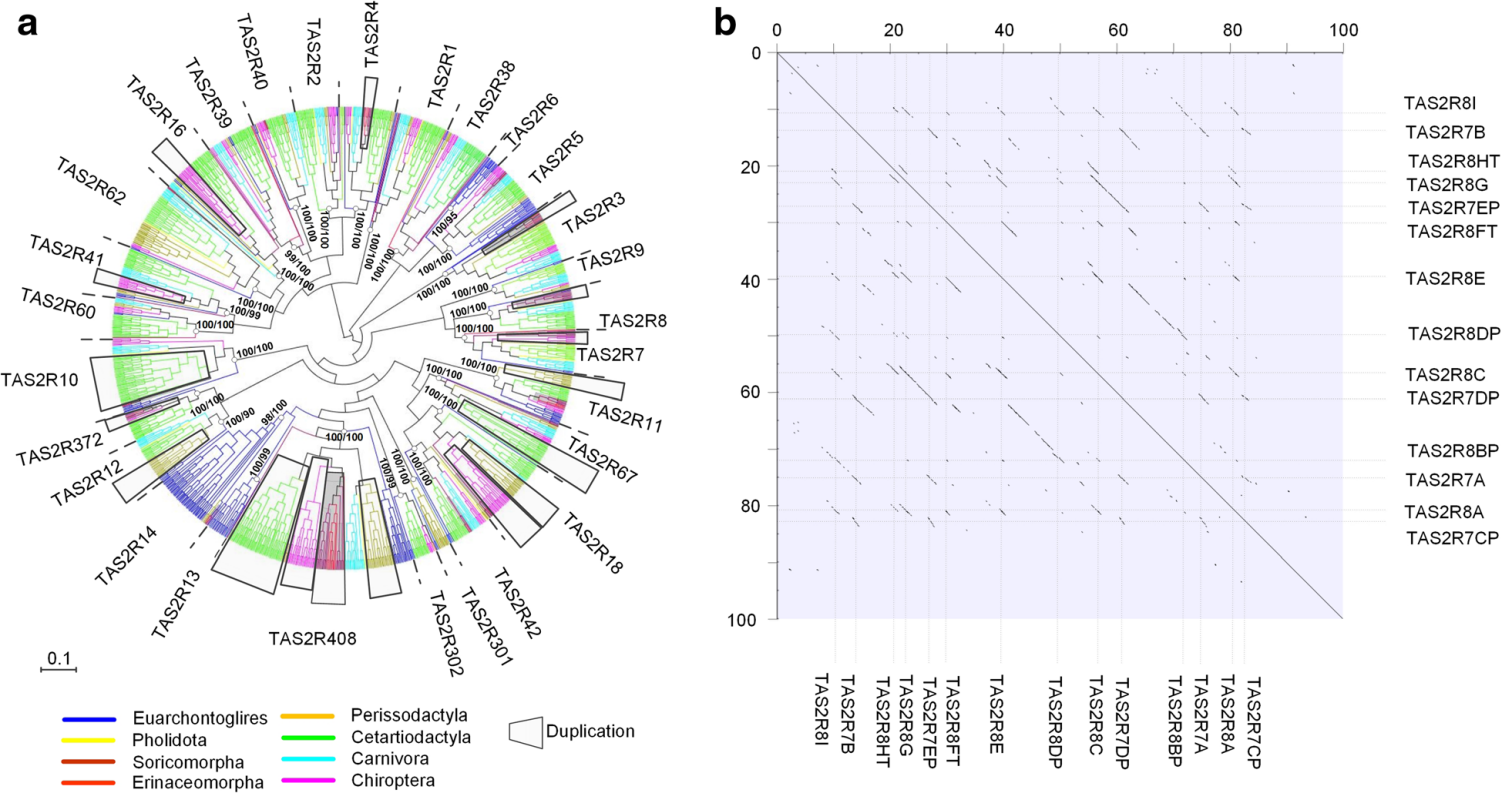
**a** A Neighbour-joining tree based on an alignment of annotated Laurasiatherian *TAS2R*s (with orthologs representing all the 28 clades of Euarchontoglires *TAS2R*s as outgroups) is shown. The clades not found (*TAS2R6* and *TAS2R13*) and the pseudogenized clade *TAS2R*301 are underlined. The massive duplication events in different orders are marked by shadow blocks. **b** The dotplots showing that the duplication in the genomic region NW_004545902.1 of the common shrew resulted in increased gene copy numbers of clades *TAS2R7* and *TAS2R8*

The phylogeny of all alignable *TAS2R*s in the 41 studied Laurasiatherian mammals was constructed, including representative orthologs from all 28 clades of Euarchontoglires *TAS2R*s reported in Hayakawa et al. [12]. The gene tree recovered the same 28 main clades of *TAS2R*s in Laurasiatheria as in Euarchontoglires (hence for all Boreoeutheria), thus we maintained the nomenclature of the *TAS2R*s clades established by Hayakawa et al. [12]. *TAS2R6* and *TAS2R13* were not found in the studied Laurasiatherian mammals (Fig. 3a). Additionally, orthologs of the clade *TAS2R301* in Laurasiatheria were pseudogenized. Conservatively, we conclude that the most recent common ancestor (MRCA) of Laurasiatheria had at least 25 intact *TAS2R*s. The orthologous and paralogous relationships among the annotated Laurasiatherian *TAS2R*s are provided in Additional file 1: Table S2.

### Lineage-specific gene degeneration and expansion in Laurasiatherian *TAS2R*s

Chinese pangolin (*Manis pentadactyla*) only contained two intact bitter taste genes (*TAS2R1* and *TAS2R7*; Fig. 1). In Cetartiodactyla only the minke whale (*Balaenoptera acutorostrata*) and the Antarctic minke whale (*Balaenoptera bonaerensis*) retain a single *TAS2R* gene, although each species retains a different one, i.e. *TAS2R16* and *TAS2R67*, respectively. The remaining whales and dolphins lost all intact *TAS2R*s that our analysis indicates were present in their common ancestor. Similarly, another group of marine mammals, pinnipedia (walrus and Weddell seal, Carnivora), only present 4–5 *TAS2R*s while having lost the rest of them (Fig. 2; Additional file 1: Table S2), which is consistent with the results of a previous genomic analysis on carnivores [13].

The increase in numbers of intact *TAS2R*s in the common shrew was observed and caused by the lineage-specific duplication of *TAS2R7*, *TAS2R8*, *TAS2R372* and *TAS2R408*. These *TAS2Rs* duplications are the outcome of duplications in the genomic region NW_004545902.1 of the common shrew (Fig. 3a and b; Additional file 1: Figure S1). Similarly to the shrew, we found lineage-specific duplication of *TAS2R16*, *TAS2R18* and *TAS2R41* in insectivorous bats (Fig. 3a; Additional file 1: Table S2) corresponding to a genomic duplication that increased the gene copy number of *TAS2R16* and *TAS2R18* in the insectivorous Brandt’s bat (*Myotis brandtii*). Contrastingly, the frugivorous big brown bat (*Eptesicus fuscus*) shows no duplication in its orthologous genomic region (Additional File 1: Figure S1).

### Correlation between diet and *TAS2R*s repertoire

**Fig. 4:**
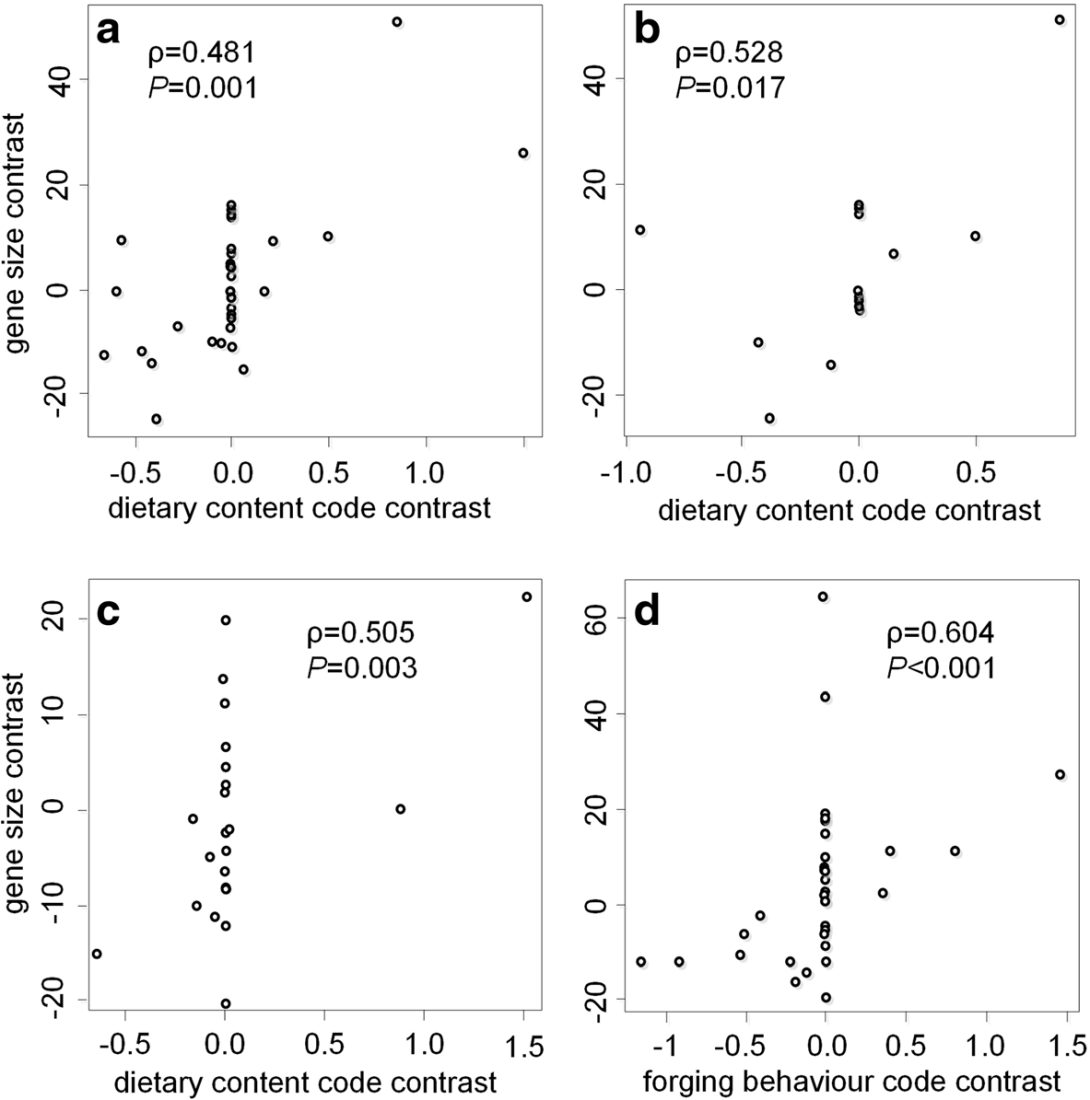
The impact of dietary content and behaviour on the intact *TAS2R* genes repertoire, as revealed by a PIC analysis. **a** significant positive correlation of PICs in total number of intact genes with PICs in diet content code (herbivores and/or insectivores:1, omnivores: 0.5, carnivores: 0). **b** the first sub-dataset (insectivores:1, omnivores: 0.5, carnivores: 0): a significantly positive correlation of PICs in total number of intact genes with PICs in diet content code. **c** the second sub-dataset (herbivores:1, omnivores: 0.5, carnivores: 0; not including tylopods): a significantly positive correlation of PICs in total number of intact genes with PICs in diet content. **d** significant positive correlation of PICs in total number of intact genes with PICs in foraging behaviour code (swallowing food whole: 0, chewing food: 1). The Spearman’s rank correlation coefficient (ρ) with a one-tailed *P* value was used to evaluate the association

To obtain a general trend of the relationship between the evolutionary trajectories of *TAS2R*s and feeding performance in Laurasiatheria, we conducted a correlation and regression analysis between the number of intact *TAS2R*s and the dietary strategy for Laurasiatherian species [14]. However, in order to account for the inherent correlations deriving from the relationships between taxa in a phylogenetic tree (phylogenetic inertia) [15], we performed a phylogenetically independent contrast (PIC) analysis implemented in the package Analyses of Phylogenetics and Evolution to assess the patterns of change in *TAS2R* genes across the Laurasitherian phylogeny [14]. Firstly, we categorize the species as herbivore, insectivore, omnivores and carnivore on the basis of dietary preferences ([4, 16], http://animaldiversity.ummz.umich.edu/). Then, as done previously [3, 4], we coded a species as 1 for herbivores and insectivores, 0.5 for omnivores and 0 for carnivores, indicating the percentage of plant and insect tissue in its diet because plant and insect tissues may have the most abundant potential toxins, whereas non-insect and non-plant tissues have the least. The only exception is the Chinese pangolin with its highly specialized diet. Because pangolins feed almost exclusively on termites and ants that are located by scent, and show a clear feeding preference for particular species of ants and termites, they are unlikely to be exposed to the variety of potential toxins as are other insectivores. Thus we also code the Chinese pangolin as 0 because of their narrow dietary niche. Then we converted the 41 phylogenetically correlated data points into 40 PICs, using the information of the species tree of the 41 species including their divergence times (Additional file 1: Figure S2). After converting the diet codes and the intact *TAS2R* gene numbers into PICs, we conducted a regression analysis. The PICs in the dietary content code positively correlated with that of intact *TAS2R*s numbers (Spearman’s ρ = 0.481, *P* = 0.001, Fig. 4a), supporting the hypothesis that animals whose diet contains more toxins tend to have more functional *TAS2R*s. To compare with an earlier vertebrate-wide study [4], we also coded insectivores as 0, In this case, the correlation between the PICs of diet codes and those of intact gene numbers is not significant (ρ = 0.252, *P* =0.122).

Secondly, to test the impact of insectivory and herbivory on the *TAS2R*s diversity separately, we divided the whole dataset into two sub-datasets. The first sub-dataset includes the insectivores, carnivores and omnivores, while excluding herbivores. The second dataset includes the herbivores, carnivores and omnivores, thereby excluding insectivores from the analysis. Subsequently, we conducted the PICs analysis on the two datasets separately. For the first sub-dataset including insectivores, omnivores and carnivores, the PICs in the dietary code were positively correlated with that of intact *TAS2R*s numbers (ρ = 0.528, *P* = 0.017; Fig. 4b), supporting the hypothesis that insectivores exhibit more intact *TAS2R*s than omnivores and carnivores. For the second sub-dataset including herbivores, omnivores and carnivores, we performed two PIC analyses. One included all the herbivores and another excluded the tylopods. The PICs analysis was based on the data including all the herbivores revealed a marginal significant correlation between the dietary code and intact *TAS2R*s number (ρ = 0.289, *P* = 0.051). Furthermore, the PICs analysis excluding tylopods supported a significant correlation between the dietary code and intact *TAS2R*s number (ρ = 0.505, *P* = 0.003; Fig. 4c). Camels live in a harsh desert environment and mainly feed on xerophilous plants such as camel thorn (*Alhagi spp.*) and plants from the Chenopodiaceae family [17]. Similarly, alpaca live in areas of high altitude and extreme cold, with relatively few food sources [18]. Consequently, in light of the available variety of plants in their environments, it is likely that tylopods adapted to a limited range of plant species [17, 18] and gradually decreased dependence on bitter taste and experienced a reduction of *TAS2R*s numbers. The reduction of *TAS2R*s in tylopods still reflects the changes in dietary preferences, with a correlation between the functional *TAS2R*s number and abundance of potential toxins in the diet, and does not refute the general trend that herbivores evolved more copy numbers of intact *TAS2R*s than omnivores/carnivores.

Thirdly, we coded a species as 0 for swallowing the food whole and 1 for species that chew their food. Then the PICs analysis was implemented again to test the correlation and regression analysis of the numbers of intact *TAS2R*s and the foraging behaviour code for Laurasiatherian. The PICs in the foraging behaviour code also positively correlated with that of intact *TAS2R*s numbers (ρ = 0.604, *P* <0.001, Fig. 4d), supporting the hypothesis that the animals less dependent on bitter taste are likely to pose a lower number of functional *TAS2R*s.

## Discussion

### *TAS2Rs* degeneration in pangolin and marine mammals

Pangolins are highly specialized in their diet, feeding almost exclusively on termites and ants that are located by scent [19]. Different pangolin species typically show a clear feeding preference for particular species of ants and termites, often rejecting all other available prey species [19]. Furthermore, pangolins catch living ants with their tongue, and all food is ingested whole and then crushed in the lower section of the stomach. This feeding behaviour together with our finding of very low *TAS2R* diversity in Chinese pangolin indicates that in this taxon bitter taste perception is highly reduced as a consequence of (a) a hyper-specialization on a very specific feeding resource, and (b) a lack of reliance on bitter taste perception in the identification of prey. Even with no knowledge of the tuning width of the only two *TAS2Rs* in pangolins, the exclusive diet and special feeding behaviour of pangolins might ultimately have rendered their bitter taste receptors redundant resulting in the loss of most *TAS2R*s. On the other hand, it should be interesting to assess the detection range of the pangolins’ only two *TAS2R*s on various bitter compounds.

Examples that illustrate the relationship between *TAS2R* gene number and special foraging behaviours are also found in marine mammals. Feng et al. [20] reported that nine of 10 *TAS2R*s surveyed in 12 whales were pseudogenized. Consistent with Feng et al’s results but based on a larger genomic scale, we show that all but two of the analysed whales and dolphin lost all the 23 intact *TAS2R*s in their most recent common ancestor. Similarly, another group of marine mammals, pinnipedia [walrus (*Odobenus rosmarus*) and the Weddell seal (*Leptonychotes weddellii*)] possess only 4–5 intact *TAS2R*s (Fig. 2). The bitter taste function in these taxa (cetacea and pinnipedia) might have been relaxed during the evolutionary history of both of them following their colonisation of the marine environment, their dietary switch to consuming fish and cephalopods, and swallowing whole prey [20, 21]. Consistent with this speculation, it has been reported that cetacea have lost most of the sweet, umami and sour tastes receptors and pinnipedia have lost sweet tastes receptors [20, 21].

Purifying selection was suggested to be the major selective force shaping the evolution of taste genes [4]. If selection is relaxed in some species, the corresponding taste genes may become pseudogenes. For example, vampire bats feed solely on a single food item (i.e. blood), and thus have less need for sweet, umami and bitter taste function. As expected, it was recently shown that vampire bats have lost the functional sweet and umami taste genes and pose a significantly greater percentage of pseudogenes of bitter taste genes than other bats [22, 23]. In giant panda the pseudogenization of the umami taste gene coincided with a dietary switch to bamboo [24]. Similarly, penguins are carnivorous, swallow food whole and their tongue structure and function suggest they do not need taste perception, and as expected, they have also lost the functional sweet, umami, and bitter taste genes [25]. These examples suggest that dietary composition and foraging behaviour indeed affect the number of intact taste genes.

### *TAS2R*s expansions in insectivorous bats and shrew

Bitter tasting compounds (toxins or not) in insects appear to have strongly impacted the diversity of the *TAS2R*s repertoire in insectivores. Many insects that fly at night have evolved strategies to avoid predation by bats, of which the chemical defence is an important component: the chemical repertoire of nocturnal insects includes cardiac glycosides and pyrrolizidine alkaloids, which are effective feeding deterrents for bats [8, 9]. Diversity of the *TAS2R*s repertoire might thus be of vital importance for insectivorous bats, allowing them to detect toxins and to avoid their ingestion. Similarly, shrews have an insectivorous diet consisting mostly of soil insects [26, 27]. The high metabolic rates of most shrews make them susceptible to food shortage and result in the requirement of a constant food supply [26]. This likely contributes to the critical role of bitter taste for shrews, allowing them to detect toxins and to make prompt decisions while feeding.

### The process of the influence of dietary specialization on *TAS2R* gene number

The influence of dietary specialization on the *TAS2R*s number changes could be an interactive, time-dependent, and step by step process. On the one hand, individuals of a species, which started to lose some *TAS2R* genes caused by dietary specialization may not detect toxins in their ancestral diet, thereby increasing their mortality. Consequently, the loss in *TAS2R* genes could result in the species acquiring a narrow dietary niche. Conversely, a narrow diet might further reduce the strength of selection for a more diverse *TAS2R*s repertoire, accelerating the genomic loss of *TAS2R*s. This kind of evolutionary vortex might explain the process of the *TAS2R*s loss in marine mammals and Chinese pangolin. On the other hand, individual of one species that acquire new *TAS2R* genes may be able to detect a wider range of toxins in new potential diets, thereby extending their diet range. The expanded dietary niche might explain the importance of a more diversified *TAS2R*s repertoire.

## Conclusions

The present study provides the most comprehensive understanding of the *TAS2R*s in Laurasiatheria to date, going  beyond the known correlation between herbivory and *TAS2R*s numbers. Besides herbivores, we show that insectivores tend to have more functional *TAS2R*s‚ while lineages with a narrow dietary niche (pangolins) and with less dependence on bitter taste (pangolins, cetacea and pinnipedia) have lost most functional *TAS2R*s. Our study reveals that the dietary specialization of Laurasiatheria has had a complex influence on bitter taste gene evolution. However, this finding does not automatically refute the potential impact of other factors than dietary niche on the bitter taste gene evolution. The presence/absence of intact taste genes in mammals and other vertebrates is sometimes inconsistent with what is expected from organismal diet, inferring that our understanding of the physiological functions of these tastes and/or their receptor genes is not complete [28]. Both genetic and behavioural studies, suggested by Behrens et al. [29] and Jiang et al. [30], are needed to deepen our understanding of the function and evolution of *TAS2R*s.

## Methods

### Data resource and nomenclature

The genome assemblies of 41 Laurasiatherian mammals were downloaded (Additional file 1: Table S1), and the phylogenetic relationships of these species were assessed using TimeTree v3.0 (http://www.timetree.org/, last accessed June 30, 2016) [6].

### Annotation of *TAS2R*s

BLASTN and TBLASTN in BLAST v2.2.23 [31] were used for sequence similarity searches in the 41 Laurasiatherian genome assemblies based on templates of nucleoid and protein sequences of the orthologs of *TAS2R*s from human, mouse, dog and cow as queries respectively. BLASTN and TBLASTN were performed with filtering off (−F false) and the cutoff E value of 1e-5 (using all the intact and disrupted *TAS2R*s as queries) and 1e-10 (using all the intact genes as queries), respectively. The outputs from one Laurasiatherian genome were used for reciprocal BLASTN against this genome itself. Outputs with a length of less than 100 bp were discarded. Sequences longer than 750 bp with complete open reading frame (ORF) and minimal flanking sequences were regarded as candidate complete *TAS2R*s. Complete genes were verified by the TMHMM method for the presence of a seven transmembrane domains [32]. The remaining outputs larger or equal to 750 bp were regarded as disrupted *TAS2R*s reading frames if they were pseudogenised as indicated by a loss of their start position, a gain of premature stop codons, and/or the presence of indels that disrupted the coding frame. Subsequently, the remaining outputs where the contig end had ≤30 bp in the flanking sequences, which was not disrupted by stop codons or indels according to the multiple alignments with known complete *TAS2R*s, were regarded as partial *TAS2R*s, because the last 30 bp in the end of contig could be sequenced and assembled incorrectly [12].

Partial *TAS2R*s may be complete genes, but they were not sequenced completely or assembled completely during genome assembly. It has been reported that the probability of overestimating the number of partial *TAS2R*s increases when surveying genome assemblies with a contig N50c value of less than 10 kb [12]. However, the N50c values of 40 herein surveyed assemblies are higher than 10 kb (Additional file 1: Table S1). Our data was thus gathered in a way to minimize the probability of such assembly errors, ensuring the reliability of the data. All the overlapping sequences of hits with the same orientations at the contig level were merged (Additional file 1: Table S3). Both complete and partial *TAS2R*s were regarded as intact *TAS2R*s in subsequent analysis.

### Reconstruction of *TAS2R*s repertoire evolution

In this study, we follow the nomenclature of *TAS2R*s in Euarchontoglires (sister clade of Laurasiatheria) reported by Hayakawa et al. [12]. The annotated *TAS2R*s were merged with the *TAS2R* genes representing all the reported 28 Euarchontoglires *TAS2R*s clades [12] into a multiple alignments of nucleotide sequences using E-INS-i in MAFFT v7 [33]. After removal of gap sites, the phylogenetic trees of *TAS2R*s were constructed with MEGA 5 using the neighbour-joining (NJ) method [34] and with RAxML v8.1.X using the maximum-likelihood (ML) method [35, 36]. The orthologous and paralogous relationships with the *TAS2R*s were inferred based on the phylogenetic tree. The evolution of the *TAS2R* repertoires (births and deaths) in Laurasiatherian lineages was reconstructed as in Hayakawa et al. [12]. Dot-plots were constructed using UGENE v1.10.0 [37] to compare the structure of scaffold/chromosomal regions containing *TAS2R* loci to detect duplications and deletions.

### Phylogenetically independent contrasts (PIC) analyses

The PIC analyses were conducted using the *pic* function within the R package of Analyses of Phylogenetic and Evolution (*ape*) in order to identify whether there was any trend in the gain/loss of *TAS2R*s along the phylogenetic tree while taking into account the position of species along the phylogenetic tree [12, 38]. The nonparametric Spearman’s rank correlation coefficient (ρ) was used to assess the correlation. The information of topology and branch length of the tree was shown in Fig. 3, which are reconstructed on the basis of TimeTree v3.0 [6] and Zhou et al. [39].

## Additional files

Additional file 1: Tables S1-S4 and Figures S1-S2. Whole-genome assemblies analyzed in the present study. **Table S2.** Bitter taste receptor genes *TAS*2*R*s in the genome assemblies of Laurasiatherian mammals (Please refer to the excel file Supplementary_Table_S2, Additional file 3). Pseudogenes were listed in red characters. Whales and dolphins (Tutr, Oror, Live, Phma, Baac) which have lost most of the *TAS*2*R*s were marked in pale read shadow, pangolin (Mape) which only have 2 *TAS*2*R*s was marked in pale blue shadow, and the common shrew (Soar) with the most number of 52 *TAS*2*R*s was marked in yellow shadow. **Figure S1.** (A and B) Dotplots comparing the Brandt’s bat and the big brown bat assemblies containing the clade of *TAS2R16*. (C and D) Dotplots comparing the Brandt’s bat and the big brown bat assemblies containing the clade of *TAS2R18*. (E and F) Dotplots comparing horse and cat assemblies containing the clades of *TAS2R11* and *TAS2R12*. (G and H) Dotplots comparing horse and cat assemblies containing the clade of *TAS2R62*. The minimum repeat length was 100 bp and the repeat identity was 90 %. The *TAS2R*s positions are shown by dashed lines. **Figure S2.** Forty PIC values converted from the 41 phylogenetically correlated data. **Table S3.** Classification of Truncated *TAS2Rs* in the genome assemblies of Laurasiatherian mammals. For each species, overlapping truncated *TAS2Rs* with similar orthologies in the multiple alignments were regarded as being derived from different loci. In contrast, non-overlapping *TAS2Rs* were regarded as being derived from the same loci with gap(s). **Table S4.** Birth genes and death genes in each branch of Laurasiatherian mammals. (PDF 0.99 mb) Additional file 2: Data set S1. Annotated sequences of *TAS2R*s (*Tas2r*s) in the present study. The header of each FASTA sequence indicates the *TAS2R* name and its location in the contig, scaffold, or chromosome in the whole-genome assembly. Truncated and disrupted genes are indicated by T and P, respectively. (PDF 796 kb) Additional file 3: Excel file Supplementary_Table_S2. (XLSX 19.2 kb)
