# Supplementary material for: Dietary specialization drives multiple independent losses and gains in the bitter taste gene repertoire of Laurasiatherian Mammals
Source: Front Zool. 2016 Jun 29;13:28. doi: 10.1186/s12983-016-0161-1 (PMC4928315; doi:10.1186/s12983-016-0161-1)
Supplement: Additional file 1: Tables S1-S4 and Figures S1-S2. — Whole-genome assemblies analyzed in the present study. Table S2. Bitter taste receptor genes TAS2Rs in the genome assemblies of Laurasiatherian mammals (Please refer to the excel file Supplementary_Table_S2, Additional file 3). Pseudogenes were listed in red characters. Whales and dolphins (Tutr, Oror, Live, Phma, Baac) which have lost most of the TAS2Rs were marked in pale read shadow, pangolin (Mape) which only have 2 TAS2Rs was marked in pale blue shadow, and the common shrew (Soar) with the most number of 52 TAS2Rs was marked in yellow shadow. Figure S1. (A and B) Dotplots comparing the Brandt’s bat and the big brown bat assemblies containing the clade of TAS2R16. (C and D) Dotplots comparing the Brandt’s bat and the big brown bat assemblies containing the clade of TAS2R18. (E and F) Dotplots comparing horse and cat assemblies containing the clades of TAS2R11 and TAS2R12. (G and H) Dotplots comparing horse and cat assemblies containing the clade of TAS2R62. The minimum repeat length was 100 bp and the repeat identity was 90 %. The TAS2Rs positions are shown by dashed lines. Figure S2. Forty PIC values converted from the 41 phylogenetically correlated data. Table S3. Classification of Truncated TAS2Rs in the genome assemblies of Laurasiatherian mammals. For each species, overlapping truncated TAS2Rs with similar orthologies in the multiple alignments were regarded as being derived from different loci. In contrast, non-overlapping TAS2Rs were regarded as being derived from the same loci with gap(s). Table S4. Birth genes and death genes in each branch of Laurasiatherian mammals. (PDF 0.99 mb) [file 12983_2016_161_MOESM1_ESM.pdf]

Table S1. Whole-genome assemblies analyzed in the present study.

Name	Species	Prefix[a]	Assembly	N50[b]	Source	Reference
Cow	*Bos taurus*	Bota	Bos_taurus_UMD_3.1.1	97kb	NCBI	Zimin et al. 2009
Zebu	*Bos indicus*	Boin	Bos_indicus_1.0	28kb	NCBI	Canavez et al. 2012
Yak	*Bos grunniens*	Bogr	BosGru_v2.0	23kb	NCBI	Qiu et al. 2012
Water buffalo	*Bubalus bubalis*	Bubu	UMD_CASPUR_WB_2.0	22kb	NCBI	Zimin et al. Unpublished
Goat	*Capra aegagrus hircus*	Cahi	CHIR_1.0	19kb	NCBI	Dong et al.2012
Wild goat	*Capra aegagrus*	Caae	Caeg1	52kb	NCBI	Streeter et al. Unpublished
Sheep	*Ovis aries*	Ovar	Oar_v3.1	40kb	Ensembl	Jiang et al. Unpublished
Musimon	*Ovis aries musimon*	Ovmu	Oori1	40kb	NCBI	Streeter et al. Unpublished
Tibetan antelope	*Pantholops hodgsonii*	Paho	PHO1.0	19kb	NCBI	Ge et al. 2013
Dolphin	*Tursiops truncatus*	Tutr	turTru1	10kb	Ensembl	Gnerre et al. 2011
Killer whale	*Orcinus orca*	Oror	Oorc_1.1	70kb	NCBI	Foote et al. 2012
Baji	*Lipotes vexillifer*	Live	Lipotes_vexillifer_v1	32kb	NCBI	Zhou et al. 2013
Sperm whale	*Physeter catodon*	Phca	Physeter_macrocephalus -2.0.2	35kb	NCBI	Walter et al. Unpublished
Minke whale	*Balaenoptera acutorostrata*	Baac	BalAcu1.0	23kb	NCBI	Zheng et al. 2014
Antarctic minke whale	*Balaenoptera bonaerensis*	BaBo	KUjira_1.0	1.7kb	-	Kishida et al. 2015
Pig	*Sus scrofa*	Susc	Sscrofa10.2	69kb	NCBI	Drou et al. Unpublished
Bactrian camel	*Camelus bactrianus*	Caba	Ca_bactrianus_MBC_1.0	139kb	NCBI	Wu et al. Unpublished
Wild Bactrian camel	*Camelus ferus*	Cafe	CB1	90kb	NCBI	Yao et al .2012
Arabian camel	*Camelus dromedarius*	Cadr	PRJNA234474_Ca _dromedarius_V1.0	69kb	NCBI	Wu et al. 2014
Alpaca	*Vicugna pacos*	Vipa	vicugna_pacos-2.0.1	25kb	NCBI	Warren et al. Unpublished
Horse	*Equus ferus caballus*	Eqca	EquCab2.0	112kb	NCBI	Lindblad-Toh et al. 2009
Przewalski's horse	*Equus ferus przewalskii*	Eqpr	Burgud	58kb	NCBI	Huang et al. 2014

Southern white rhinoceros	*Ceratotherium simum*	Cesi	CerSimSim1.0	93kb	NCBI	Di et al. Unpublished
Cat	*Felis catus*	Feca	Felis_catus_6.2	21kb	NCBI	Pontius et al. 2007
Tiger	*Panthera tigris altaica*	Pati	PanTig1.0	30kb	NCBI	Chu et al. 2013
Walrus	*Odobenus rosmarus*	Odro	Oros_1.0	90kb	NCBI	Foote et al. 2015
Weddell seal	*Leptonychotes weddellii*	Lewe	LepWed1.0	24kb	NCBI	Di et al. Unpublished
Ferret	*Mustela putorius furo*	Mufu	MusPutFur1.0	45kb	Ensembl	Di et al. Unpublished
Polar bear	*Ursus maritimus*	Urma	UrsMar_1.0	47kb	NCBI	Liu et al. 2014
Giant Panda	*Ailuropoda melanoleuca*	Aime	AilMel_1.0	40kb	Ensembl	Li et al. 2010
Dog	*Canis lupus familiaris*	Cafa	CanFam3.1	267kb	NCBI	Lindblad-Toh et al. 2005
Chinese pangolin	*Manis pentadactyla*	Mape	M_pentadactyla-1.1.1	29kb	NCBI	Wilson et al. Unpublished
Brandt's bat	*Myotis brandtii*	Mybr	ASM41265v1	23kb	NCBI	Seim et al.2013
David's myotis	*Myotis davidii*	Myda	ASM32734v1	15kb	NCBI	Zhang et al. 2013
Little brown bat	*Myotis lucifugus*	Mylu	Myoluc2.0	64kb	Ensembl	Di Palma et al. Unpublished
Big brown bat	*Eptesicus fuscus*	Epfu	EptFus1.0	21kb	NCBI	Di Palma et al. Unpublished
Large flying fox	*Pteropus vampyrus*	Ptva	Pvam_2.0	22kb	Ensembl	Liu et al. 2014
Black flying fox	*Pteropus alecto*	Ptal	ASM32557v1	32kb	NCBI	Zhang et al. 2013
Western European hedgehog	*Erinaceus europaeus*	Ereu	EriEur2.0	21kb	NCBI	Di Palma et al. 2012
Common shrew	*Sorex araneus*	Soar	SorAra2.0	23kb	UCSC	Di Palma et al. 2012
Star-nosed mole	*Condylura cristata*	Cocr	ConCri1.0	46kb	NCBI	Di Palma et al. 2012
Human	*Homo sapiens*	Hosa	GRCh38.p2	56Mb	NCBI	
Mouse (ALLPATHS-LG)	*Mus musculus*	Mumu	MmusALLPATHS1	16kb	NCBI	Gnerre et al. 2011

[a]The prefix is used to discriminate the name of orthologs in (sub)species. For example, human *TAS2R1* is referred to as Hosa*TAS2R1.*

[b]N50C is the contig length where 50% of the assembled genome lies in blocks of at least $N50_C$.

Reference：

Zimin *et al*. 2009. A whole-genome assembly of the domestic cow, *Bos Taurus*. Genome Biol. 10(4):R42.

Canavez et al. 2012. Genome sequence and assembly of *Bos indicus*. J. Hered. 103(3):342-8.

Qiu et al. 2012. The yak genome and adaptation to life at high altitude. Nat Genet. 44(8):946-9.

Dong *et al*. 2012. Sequencing and automated whole-genome optical mapping of the genome of a domestic goat (Capra hircus). Nat Biotechnol. 31(2):135-41.

Ge et al. 2013. Draft genome sequence of the Tibetan antelope. Nat Commun. 4:1858.

Gnerre et al. 2011. High-quality draft assemblies of mammalian genomes from massively parallel sequence data. *Proc. Natl. Acad. Sci. USA*. 108:1513–1518.

Foote et al. 2015. Convergent evolution of the genomes of marine mammals. Nat. Genet. 47(3):272-275

Zhou et al. 2013. Baiji genomes reveal low genetic variability and new insights into secondary aquatic. Nat. Commun. 4:2708.

Zheng et al. 2014. Minke whale genome and aquatic adaptation in cetaceans. Nat. Genet. 46(1):88-92.

Yao et al .2012. Genome sequences of wild and domestic bactrian camels. Nat. Commun. 3:1202.

Wu et al. 2014. Camelid genomes reveal evolution and adaptation to desert environments. Nat Commun. 5:518.

Lindblad-Toh et al. 2009. Genome sequence, comparative analysis, and population genetics of the domestic horse. Science. 326(5954):865-867.

Huang et al. 2014. Analysis of horse genomes provides insight into the diversification and adaptive evolution of karyotype. Sci. Rep. 4:4958.

Pontius et al. 2007. Initial sequence and comparative analysis of the cat genome. Genome Res. 17(11):1675-1689.

Chu et al. 2013. The tiger genome and comparative analysis with lion and snow leopard genomes. Nat Commun. 4:2433.

Liu et al. 2014. Population genomics reveal recent speciation and rapid evolutionary adaptation in polar bears. Cell. 157(4):785-94.

Li et al. 2010. The sequence and de novo assembly of the giant panda genome. Nature. 463(7279):311-7.

Lindblad-Toh et al. 2005. Genome sequence, comparative analysis and haplotype structure of the domestic dog. Nature. 438(7069):803-19.

Seim et al. 2013. Genome analysis reveals insights into physiology and longevity of the Brandt's bat Myotis brandtii. Nat. Commun. 4:2212.

Zhang et al. 2013. Comparative analysis of bat genomes provides insight into the evolution of flight and immunity. Science. 339 (6118):456-460.

Kishida et al. 2015. Aquatic adaptation and the evolution of smell and taste in whales. Zoological Letters: 1:9.

Table S2. Bitter taste receptor genes *TAS2R*s in the genome assemblies of Laurasiatherian mammals (Please refer to the excel file Supplementary_Table_S2). Pseudogenes were listed in red characters. Whales and dolphins (Tutr, Oror, Live, Phma, Baac) which have lost most of the *TAS2R*s were marked in pale read shadow, pangolin (Mape) which only have 2 *TAS2R*s was marked in pale blue shadow, and the common shrew (Soar) with the most number of 52 *TAS2R*s was marked in yellow shadow.

Table S3. Classification of Truncated *TAS2Rs* in the genome assemblies of Laurasiatherian mammals. For each species, overlapping truncated *TAS2Rs* with similar orthologies in the multiple alignments were regarded as being derived from different loci. In contrast, non-overlapping *TAS2Rs* were regarded as being derived from the same loci with gap(s).

Assembly	Different orthologs	Overlapping *TAS2R*s (potentially intact paralogs)	Non-overlapping *TAS2R*s (potentially intact gene from same locus)	Intact *TAS2R*s	Truncated *TAS2R*s	Truncated *TAS2R* loci	Potentially intact *TAS2R*s
Big brown bat	TAS2R18BT	–	–	19	1	1	20
Brand’t bat	TAS2R16ET	TAS2R18HT, TAS2R18IT, TAS2R18JT	TAS2R18B1T-TAS2R18B2T, TAS2R18E1T-TAS2R18E2T, TAS2R18F1T-TAS2R18F2T,	26	10	7	33
Cow	–	–	TAS2R7AT-TAS2R7BT	19	2	1	20
David’s myotis	TAS2R41CT, TAS2R18KT, TAS2R18HT	TAS2R7AT, TAS2R7BT	–	25	5	5	30
Western European hedgehog	TAS2R5T	–	–	20	1	1	21
Little brown bat	TAS2R41BT	–	TAS2R16ET- TAS2R16FT	29	3	2	31
Large flying fox	TAS2R1BT, TAS2R3BT, TAS2R39T	–	–	15	3	3	18
Giant panda	TAS2R408CT	–	–	16	1	1	17
Pig	TAS2R4BT	–	TAS2R1AT- TAS2R1BT	16	3	2	18
Weddell seal	TAS2R9T, TAS2R41T	TAS2R7AT, TAS2R7BT	–	2	4	3	5
Sheep	TAS2R10AT	–	––	15	1	1	16
Common Shrew	TAS2R372BT	TAS2R8HT, TAS2R8FT	TAS2R408PT-TAS2R408QT, TAS2R408NT-TAS2R408OT	46	7	5	51

Tiger	TAS2R4T	−	−	11	1	1	12
Wild goat	TAS2R67BT	−	−	19	1	1	20

Table S4. Birth genes and death genes in each branch of Laurasiatherian mammals.

Branch	Name	Number of births	Birth genes	Number of deaths	Death genes
1	Cow	0		2	TAS2R2, TAS2R372
2	Zebu	1	TAS2R1A, TAS2R1B	2	TAS2R7, TAS2R408C
3	Yak	0		3	TAS2R4, TAS2R42, TAS2R18A
4	Water buffalo	0		7	TAS2R2, TAS2R3, TAS2R10C, TAS2R13, TAS2R408B, TAS2R408C, TAS2R18A
5	Cow & Zebu	0		0	
6	*Bos*	0		2	TAS2R8, TAS2R40
7	Bovidae	0		3	TAS2R5, TAS2R11, TAS2R62
8	Goat	0		0	
9	Wild goat	0		2	TAS2R40, TAS2R408D
10	Sheep	0		0	
11	Musimon	0		0	
12	Tibetan antelope	0		8	TAS2R11, TAS2R12, TAS2R38, TAS2R41, TAS2R42, TAS2R408D, TAS2R62, TAS2R67A
13	*Capra*	0		0	
14	*Ovis*	0		6	TAS2R408A, TAS2R40, TAS2R42, TAS2R408C, TAS2R62, TAS2R372
15	*Ovis* & *Capra*	0		1	TAS2R5
16	Caprinae	1	TAS2R67A, TAS2R67B	5	TAS2R8, TAS2R10C, TAS2R13, TAS2R408B
17	Ruminantia	4	TAS2R408A, TAS2R408B, TAS2R408D, TAS2R10A, TAS2R10B, TAS2R10C	0	

18	Dolphin	0		0	
19	Killer whale	0		0	
20	Baji	0		0	
21	Sperm whale	0		0	
22	Minke whale	0		0	
23	Delphinidae	0		0	
24	Delphinidae & Lipotidae	0		0	
25	Odontoceti	0		1	TAS2R16
26	Cetacean	0		22	TAS2R1, TAS2R2, TAS2R3, TAS2R4, TAS2R5, TAS2R7, TAS2R8, TAS2R10, TAS2R10C, TAS2R11, TAS2R12, TAS2R13, TAS2R38, TAS2R39, TAS2R40, TAS2R41, TAS2R42, TAS2R60, TAS2R18, TAS2R67, TAS2R372, TAS2R408
27		0		1	TAS2R9
28	Pig	2	TAS2R4A, TAS2R4BT, TAS2R408A, TAS2R408B	8	TAS2R2, TAS2R5, TS2R8, TAS2R13, TAS2R40, TAS2R18, TAS2R67, TAS2R372
29		0		0	
30	Bactrian camel	0		1	TAS2R4
31	Wild Bactrian camel	0		0	
32	Arabian camel	0		3	TAS2R2, TAS2R4, TS2R41
33		0		1	TAS2R42
34	*Camelus*	0		2	TAS2R16, TAS2R40
35	Alpaca	0		1	TAS2R41
36	Tylopoda	0		10	TAS2R5, TAS2R8, TAS2R9, TAS2R11,

					TAS2R12, TAS2R13, TAS2R60, TAS2R18, TAS2R372, TAS2R408
37	Cetartiodactyla	0		1	TAS2R14
38	Horse	0		4	TAS2R16, TAS2R41, TAS2R42, TAS2R62F
39	Przewalski's horse	0		2	TAS2R12D, TAS2R12E
40	Equidae	4	TAS2R408A, TAS2R408D, TAS2R408E, TAS2R11B, TAS2R11C, TAS2R12D, TAS2R12E	1	TAS2R38
41	Southern white rhinoceros	8	TAS2R408A, TAS2R408B, TAS2R408E, TAS2R408G, TAS2R11A, TAS2R11D, TAS2R11E, TAS2R12A, TAS2R12D, TAS2R12B, TAS2R12C	3	TAS2R12E, TAS2R14, TAS2R41
42	Perissodattyla	4	TAS2R12A, TAS2R12B, TAS2R62A, TAS2R62B, TAS2R62C, TAS2R62D	7	TAS2R5, TAS2R10, TAS2R13, TAS2R60, TAS2R18, TAS2R67, TAS2R372
43	Euungulata	0		0	
44	Cat	0		0	
45	Tiger	0		0	
46	Felidae	1	TAS2R408A, TAS2R408B	7	TAS2R5, TAS2R8, TAS2R10, TAS2R39, TAS2R40, TAS2R41, TAS2R62
47	Walrus	0		3	TAS2R9, TAS2R41, TAS2R408
48	Weddell seal	0		2	TAS2R42, TAS2R62
49	Ferret	1	TAS2R408B	2	TAS2R42, TAS2R62
50	Polar bear	0		4	TAS2R3, TAS2R12, TAS2R408B, TAS2R408C
51	Giant Panda	0		2	TAS2R5, TAS2R62
52	Dog	0		3	TAS2R4, TAS2R8, TAS2R9

53	Chinese pangolin	0		16	TAS2R2, TAS2R3, TAS2R4, TAS2R5, TAS2R8, TAS2R9, TAS2R10, TAS2R408, TAS2R38, TAS2R39, TAS2R40, TAS2R41, TAS2R42, TAS2R62, TAS2R67
54	Pinenipedia	0		8	TAS2R1, TAS2R3, TAS2R4, TAS2R5, TAS2R10, TAS2R12, TAS2R38, TAS2R67
55	Pinenipedia & Ferret	0		3	TAS2R39, TAS2R40, TAS2R41
56	Ursidae	2	TAS2R20, TAS2R30, TAS2R43	1	TAS2R67
57		0		0	
58		0		0	
59	Carnivora	0		0	
60	Ferae	0		7	TAS2R11, TAS2R13, TAS2R14, TAS2R16, TAS2R372, TAS2R62, TAS2R18
61	Fereuungulata	0		0	
62	Brandt's bat	1	TAS2R16ET	2	TAS2R408A, TAS2R39
63	David's myotis	2	TAS2R7AT, TAS2R7BT, TAS2R41CT	5	TAS2R5, TAS2R16D, TAS2R18G, TAS2R18I, TAS2R18J
64	Little brown bat	2	TAS2R16ET, TAS2R16FT, TAS2R41BT	4	TAS2R408C, TAS2R18G, TAS2R18H, TAS2R18I
65	Big brown bat	0		2	TAS2R4, TAS2R408C
66	Brandt's bat & David's myotis	0		0	
67	*Myotis*	11	TAS2R16A, TAS2R16B, TAS2R16C, TAS2R16D, TAS2R31, TAS2R43, TAS2R18A, TAS2R18B, TAS2R18C, TAS2R18D, TAS2R18E, TAS2R18F,	0	

			TAS2R18G, TAS2R18H, TAS2R18I, TAS2R18B		
68	Vespertilionidae	5	TAS2R408A, TAS2R408B, TAS2R408C, TAS2R408D, TAS2R41A, TAS2R41B, TAS2R18A, TAS2R18B	1	TAS2R9
69	Large flying fox	3	TAS2R1A, TAS2R1BT, TAS2R3A, TAS2R3BT, TAS2R5A, TAS2R5B	0	
70	Black flying fox	0		1	TAS2R408A
71	Pteropodidae	1	TAS2R43, TAS2R408B	4	TAS2R2, TAS2R10, TAS2R11, TAS2R18
72	Chiroptera	0		7	TAS2R8, TAS2R12, TAS2R13, TAS2R14, TAS2R60, TAS2R62, TAS2R67
73	Scrotifera	0		0	
74	Western European hedgehog	5	TAS2R3A, TAS2R3B, TAS2R3C, TAS2R4A, TAS2R4B, TAS2R19, TAS2R408B, TAS2R408C,	6	TAS2R2, TAS2R8, TAS2R13, TAS2R39, TAS2R42, TAS2R67
75	Common shrew	30	TAS2R3A, TAS2R3B, TAS2R3C, TAS2R4A, TAS2R4B, TAS2R4C, TAS2R7A, TAS2R7B, TAS2R8A, TAS2R8C, TAS2R8E, TAS2R8G, TAS2R8I, TAS2R8HT, TAS2R8FT, TAS2R11A, TAS2R11B, TAS2R11C, TAS2R11D, TAS2R408A, TAS2R408B, TAS2R408C, TAS2R408D, TAS2R408E, TAS2R408PT, TAS2R5408QT, TAS2R408F, TAS2R408G, TAS2R408H, TAS2R408I, TAS2R408G, TAS2R408K, TAS2R408L, TAS2R408NT,	0	

			TAS2R408OT, TAS2R372A, TAS2R372BT, TAS2R372C		
76	Star-nosed mole	0		0	
77	Soricomorpha	0		0	
78	Eulipotyphla	0		3	TAS2R9, TAS2R12, TAS2R16

Supplementary Figure Legend

Fig. S1. (A and B) Dotplots comparing the Brandt's bat and the big brown bat assemblies containing the clade of *TAS2R16*. (C and D) Dotplots comparing the Brandt's bat and the big brown bat assemblies containing the clade of *TAS2R18*. (E and F) Dotplots comparing horse and cat assemblies containing the clades of *TAS2R11* and *TAS2R12*. (G and H) Dotplots comparing horse and cat assemblies containing the clade of *TAS2R62*. The minimum repeat length was 100 bp and the repeat identity was 90%. The *TAS2R*s positions are shown by dashed lines.

Fig. S2. Forty PIC values converted from the 41 phylogenetically correlated data.

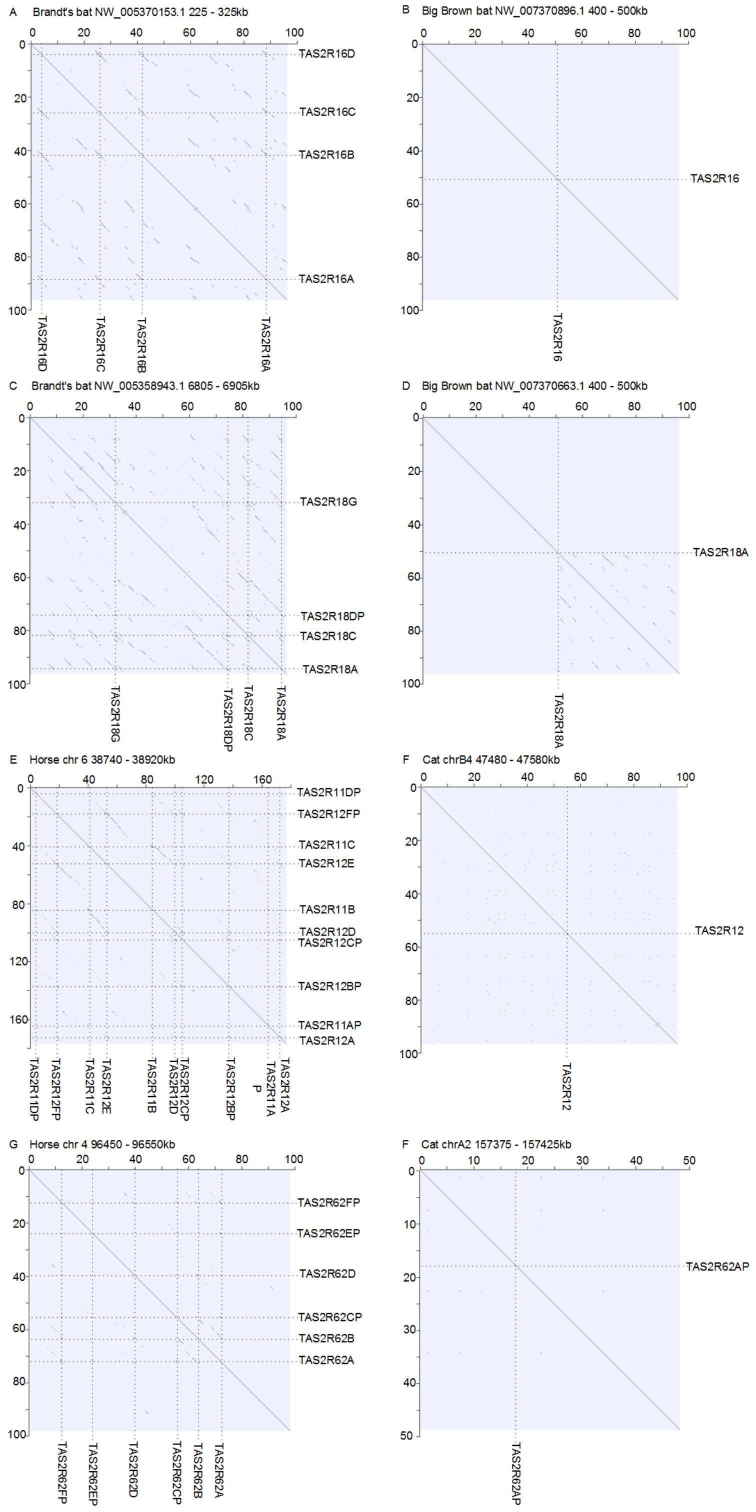
A Brandt's bat NW_005370153.1 225 - 325kb
TAS2R16D
TAS2R16C
TAS2R16B
TAS2R16A
B Big Brown bat NW_007370896.1 400 - 500kb
TAS2R16
C Brandt's bat NW_005358943.1 6805 - 6905kb
TAS2R18G
TAS2R18DP
TAS2R18C
TAS2R18A
D Big Brown bat NW_007370663.1 400 - 500kb
TAS2R18A
E Horse chr 6 38740 - 38920kb
TAS2R11DP
TAS2R12FP
TAS2R11C
TAS2R12E
TAS2R11B
TAS2R12D
TAS2R12CP
TAS2R12BP
TAS2R11AP
TAS2R12A
F Cat chrB4 47480 - 47580kb
TAS2R12
G Horse chr 4 96450 - 96550kb
TAS2R62FP
TAS2R62EP
TAS2R62D
TAS2R62CP
TAS2R62B
TAS2R62A
F Cat chrA2 157375 - 157425kb
TAS2R62AP

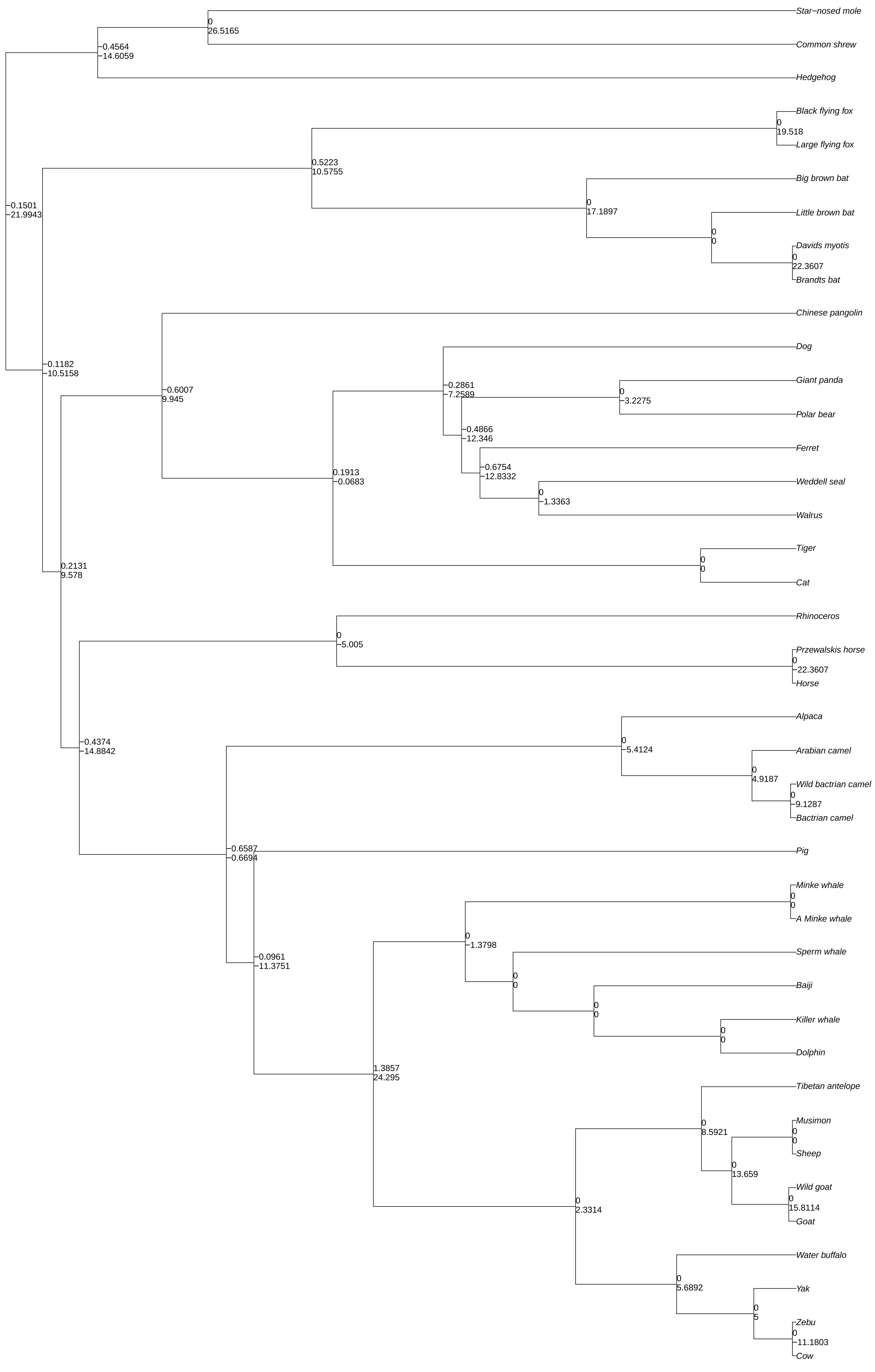
Star−nosed mole
Common shrew
Hedgehog
Black flying fox
Large flying fox
Big brown bat
Little brown bat
Davids myotis
Brandts bat
Chinese pangolin
Dog
Giant panda
Polar bear
Ferret
Weddell seal
Walrus
Tiger
Cat
Rhinoceros
Przewalskis horse
Horse
Alpaca
Arabian camel
Wild bactrian camel
Bactrian camel
Pig
Minke whale
A Minke whale
Sperm whale
Baiji
Killer whale
Dolphin
Tibetan antelope
Musimon
Sheep
Wild goat
Goat
Water buffalo
Yak
Zebu
Cow
0 26.5165
−0.4564 −14.6059
−0.1501 −21.9943
0 19.518
0.5223 10.5755
0 17.1897
0 0
0 22.3607
−0.1182 −10.5158
−0.6007 9.945
−0.2861 −7.2589
0 −3.2275
−0.4866 −12.346
−0.6754 −12.8332
0 −1.3363
0.1913 −0.0683
0 0
0.2131 9.578
0 −5.005
0 −22.3607
−0.4374 −14.8842
0 −5.4124
0 4.9187
0 −9.1287
−0.6587 −0.6694
0 0
0 −1.3798
0 0
0 0
0 0
−0.0961 11.3751
1.3857 24.295
0 8.5921
0 0
0 13.659
0 15.8114
0 2.3314
0 5.6892
0 5
0 −11.1803
