# Supplementary material for: Dietary specialization drives multiple independent losses and gains in the bitter taste gene repertoire of Laurasiatherian Mammals
Source: Front Zool. 2016 Jun 29;13:28. doi: 10.1186/s12983-016-0161-1 (PMC4928315; doi:10.1186/s12983-016-0161-1)
Supplement: Additional file 2: — Data set S1. Annotated sequences of TAS2Rs (Tas2rs) in the present study. The header of each FASTA sequence indicates the TAS2R name and its location in the contig, scaffold, or chromosome in the whole-genome assembly. Truncated and disrupted genes are indicated by T and P, respectively. (PDF 796 kb) [file 12983_2016_161_MOESM2_ESM.pdf]

Data set S1. Annotated sequences of *TAS2R*s (*Tas2r*s) in the present study. The header of each FASTA sequence indicates the *TAS2R* name and its location in the contig, scaffold, or chromosome in the whole-genome assembly.Truncated and disrupted genes are indicated by T and P, respectively.

>VipaTAS2R1_NW_005882712.1:6884786-6883899
ATGCTGGAGTCACAGCTCATTAGCCATCTTCTCTTGACAGTGATTCAGCTTCTCCTTGGGCTTTTTGTAAATGCCATCAT
TGTGGTTGTGCACGGCACGGGCTTGATCAAGCACAGAAAGATGATTCCGTTGGAGGCCCTTGTTTCCTGCCTGGCGATGT
CCAGGATCTGTCTAGAGCTGGCCATCTTCTACCTTAATCTGGCTGTTTTTAACTTGATTGACATCCGTCAGTTTGTCGGG
AAGTTTGTGATTCTCTCCTTTGTAAATGAACTGGGGCTGTGGTTTGCCACATGGCTCAGCGTCTTCTACTGCATTAAGAT
TGCCAGGATCGCTCACCCTCTCTTCCTCTGGTTGAAGCTGAAGATCTCCAAGCTGGTTCCTTGGCTGATTCTGGGGTCCC
TCCTACATGCATCTAGCACCTGTGTCTTCCACAGCAAACAGCCATGGATCTTTTCCGAAGACCTCCTGGGCTTTTCCTCT
CAAAATGCAACAGCTGAAAGCGAAGACATCGCTACTTTGCGGTTCGCCCTTCTCTTCGCTGAGCTCTCCTTGCCGTCACT
CATCTTCCTCATTTCTGCCCTGCTCCTGCTGGTCTCCCTGGGGAGACACACCTGGCAGATGAGAAACACAGCCGCAGGCC
CTTGCCCCCGTGTGCACATCAGGTCCCTCCTGTCCATCTTCGCCTTCCTGGTCCTCTACCTCTGCCACTACCTGACGGTT
CTTTTGCTCTTTTCTCAAGTTTTCAAACTTACAAGCCTCAGATTTCTGTTCTGCATCTCGCTGGTTGGTTCCTGCCACTC
CGGACACTCTATTATCTTAATTTTAGGAAATCCTAAACTGAAACAAAATGTGGAGAAGTTGCTCCTCCATAGAAAGTGCT
GTCAGTGA
>VipaTAS2R2_NW_005883013.1:905570-904641
ATGGCTTCCTCTTGGTCGGCTATTCCTCATGTTATCATCATGTCAGCAGAATTTGTCACAGGGATTACAGTAAATGGATT
TCTTACAATCATCAATTGTAATGAATTGGTCAAAAGCAGAAAGCTAACACCAATACAGCTTCTTTTTATATGTATAGGAA
TGTCTAGATTTGGTCTACAGGTCGTGTTAATGGTACAAAGTTTTTTTTCTGTGTTCTTTCCACTCTTTTATAAAGTAAAA
ATTTATGGCACAGCGATGATATTTTTTTGGATGTTTTTTAGCTCTATCAGTCTCTGGTTTGCCACCTGTCTCTCTGTATT
TTACTGCCTCAAGATATCAGGCTTCACCCAGTCCCATTTTCTTTGGCTGAAATTCAGGATCTCAAAGTTAATGCCTTGGC
TACTTCTGGGAAGTCTGCTGTCGTCTGTGAGCATTTCAACTCTGTGCATCAAGGTGGATTACCCTAAAAACTTGGATAGT
GATGTCCTCAGGAATGCCACACTAAAGAGGACTAAACTCAAGACAAAGCAAATTAATGAACTGTTTCTTGTCAACTTGGC
ATTAATACTTCCTGTAGCCATATTTGTGATGTGTACTGTTATGTTATTCATTTCTCTCTGCAAGCATACTCATCGAATGC
AAAACGGACCTCATGGTTTAGAAATGCCAGCACAGAAGCCCACATTAATGCATTAAGAACAGTGATAACATTCTTCTGC
TTCTTTGTTTCTTATTTGCTGCCTTCATGGCAAATGTAACATTCAATATTCCTCACGGAAGTCAATGGTTCTTTGTGGT
GAAGGACGTAATGGCAGCGTATCCCTCTGGCCACTCAATTATAATGATCTTGAGTAATTCTAAGTTTCAACAACCATTCA
GGAGACTTCTCTGCCTCGAAAAGAATCAAAGAAGAGGAGGACCAATGTAA
>VipaTAS2R3_NW_005882807.1:3794016-3794966
ATGCTGGGGTGCACCGACTGGGGCTTTCTGGTTCTGACTGCCAGTGAGTTCGTCCTGGGAATGCTGGGCAATGGCTTCAT
AGGGCTAGTCAATGGCAGCAGCTGGTTCAAGAGCAAGAGAATCTCTTTGTCCGACTTCATCATCACTAACCTGGCTCTCT
CTAGGATTGTTCTGCTGGGGACTCTCTTGGTTGATGGTGTTTTAATGGTGTTCTCTTCCAAACTACATGATGAAGGGATA
GTAATGCAGATGATTGATATTTTCTGGACGTTTACAAACCATCTGAGCATTTGGCTTTCCACGTGTCTCAGTGTCTTCTA
CTGCCTGAAAGTCGCCAGTTTCTCCCACCCTGCATTCCTCTGGCTCAAGTGGAGAGTTTCCAGGGTGGTCGTGTGGATGC
TGATGAGCACCCTGCTCCTAGCGTGCAGCAGCACCGTGTCTCTGGTCCATGAATTTAAGATCTATTCTGTTCTCAGTGGA
ATTGATGGAACAGGAAATATGACTGAGCAGGTTTTAAAGAAGAGAAACGAATACAAACTGATCCATGTTTTGGGGACTCT
GTGGAACCTCCTTCCCCTAACTGTGTCTCTAGCCTCCTACTTTCTGCTCATCCTCTCCCTGGGAAGGCATACACGGCAGA
TGCAGCAGAACTGTACCGGCTCCAGGGATCCAAGTACTGAGGCCCACAAGAGGGCCATCAAAATCATCCTCTCCTTCCTC
TTTCTCTTCCTACTTTACTTTCTTTCCTTTTTAATTTTATCATCTAGTTATTTCCTACCAGCAACTAAAATGATTAAGAT
GATTGGAGAAGTGATTACAATGTTATATCCTGCTGGCCACTCATATATTCTCATTCTGGGAAACAGCAAGCTGAAGCAGA
CGTTTATGGAGATGCTCTGGTGTGGGCATGGTCATCTGAAGCCTGGATCCAAGGGCCCCTTTTCTCCATAG

>VipaTAS2R4_NW_005882807.1:3803949-3804836
ATGCGTCAGGCGTTCTTTTTTTCTTCTCTTATTATCTCAGCAGTTGTGACTTCTGTTGGAGTCATTGCAAATCTGTTCAT
TGCAGTGACCAATTACAAGAATTGGGTCAAAAGCCACAAGATCTCCTCTTCTGATAGAATCCTGTTCAGCTTGGGCATCA
CCCGAGTTCTCATCCTGGGACTGAGTACTGTTTTCTCCATCTTTTCAAGTATTCGAAGGTCAGTCTCCCCATCCGCTTAT
TTCCTGTTGTGTTGGTTGTTTTTGGACTCTAATAGTCTTTGGTTTGTAACCTTGCTCAACGCCTTGTACTGTGTGAAGAT
TACTAACTACCAACACCCAATTTTTCTCCTGCTGAAACGAAATCTCTCCCCCAAAATGCCCAGGCTGCTGCTGGCCTGTG
TGCTGATTTCTGCCTTTACCGCACTCCTGCATGTTGTGCTCCGAGAAGCATCACGCTCTCTGGAATTGGTGACCGGGAGA
AATGGCACCGTTTGTGACATCAATGAGGAAATCTTCCTTTTGGTGAGCCCTTGGCTCTTGAGCTCATGTCTGCAGTTCTC
CCTTAATGTGACTTCTGCTTCCTTGTTAATAAATTCCTTGAGGAGACACATCCAGAAGATGCAGAGAAGTGCCACTGCTC
TTTGGACTCCCCAGACCGAGGCTCATGTGGGTGCTATGAAGCTGATGATCTGTTTCCTCATACTCTACGTTCCATATTCA
GTTGCTTCCCTGCTCCATTATCTCCCTTCTGTAGCGAGTGATTTGAGACTCAAGTCCATTTATATTATTATTTCCACCTT
TTACCCTCCAGCACATTCTGTTCTCATTATTCTCACACATCCTAAACTGAAAACAAAAGCAAAGAAGATTCTTCACTTCA
ATAAATAG
>VipaTAS2R7_NW_005882720.1:706706-705771
ATGCCAGGTAAGGTGGAGAACACCTTAATCCTCATAGCAGCAGGAGAATTTTCACTGGGGATCTTAGGGAATGTGTTCAT
TGGATTGGTAAACTTTGTGGACTGGATCAAGCATAGGAAGATCGCCTCCATTGATTTAATTCTCACAAGTCTGGCCATAT
CCAGAATTTCTCTATTGTGTATAATACTATTAGACTGTTTTATACTGGTTCAGTATCCAGACGTCTATACTGCTGGTAAA
CAAATGAGAATCATTGACTGCTTCTGGACACTAACCAACCATTTAAGTGTCTGGTTTGCCACCTGCCTGAGCATCTTCTA
TTTCCTCAAGATAGCTAATTTCTTCCATCCCCTTTTCCTCTGGATGAAGCTGAGAATTGACAATGTGATTCCTAAGATCC
TGCTGGGGTGCTTGGCCCTCTCTGTGTTTTTTAGCCTTCCTGTCTCTGGGAATTGGAATGATGATTTCAGGTTTTGTGTC
AAGGCAAAGTTGAAAACAAACTTAACTTTGAGATGCAAAATAAATAAAGCACAATATGCTTCCACCAAGATATATCTCAA
CCTGCTAACACTAGTCCCCTTTTCTGTGTCCCTGATCTCGTTTCTCCTCTTGATTCTCTCCCTGTGGAGACACACCCGAC
GAATGCAGCTCAATGCCACAGGGTCCAGAGACCCCAGCATAGAAGCCCACGTGGGAGCCATGAAGGCGGTCATCTCCTTC
CTCCTCCTTTTCATTGCCTACTACTTGGCCTATCTTGTAGCCACCTCCAGCTACTTTCTACCGGAGACTGAATTAGCTGT
GATGGTTGGTGAGGTGACAGCTCTAATCTGTCCCTCAAGCCATTCACTTATCCTAATTCTGGAGAACAGTAAATTAAGAA
AAGCATTTCTAAGGGTTCTATGGAAAGTAAAGTATGTCCCAACAAGGAGTTGCTAA
>VipaTAS2R10_NW_005882720.1:715676-714747
ATGCTAAGTGTAGGAGAAGGCCTCCTTCTTTTTGTAGCAGTTAGTGAGTCAGTACTGGGGGTTTTAGGGAACGGATTTAT
TGGACTTGTAAACTGCACTGACTGTGTGAGAAACAAGAAATTCTCTATGATCAGCTTTATTCTCACTGGCTTAGCTACTT
CGAGAATTGGTCTGTTATGGATAATAATTACAGACGGATTTGTAAGGATATTCTTTCCAGAAATGTATTCGTCCGGTAAC
CTGGTTGACTATATTAGTTACTCATGGATAATTCTGAATCAATTAAGTGTCTGTCTTGCCACCAGCCTCAGTGTCTTCTG
TTTCCTGAAGATAGCAAAATTTTCCCACCACATTTTTCTCTGGTTGAAGAGGAGACTCAACAGGGTTCTTCTCATTCCGA
TGGGATTATTGCTAATTTCATGGTTATTTACTTTTCCACAGATGGTGAGGATTATTAGTGATAATAGAATAAGAAATGGA
AGTACAACTGGGGTAACCAACATGCCCAAACATAAATACCTTACATACCAGGTTTCTCTCAATCTGGGGACCATTCTCCT
CTTTCTACTCTGCCTGATTACGTGTTTCCTATTGATCATTTCCCTTTGGAGGCACAACAGGAAGATGCAACTGAATGCCA
CAGGATTCAGGGACCCCAGCACAGAAGCACATATCAAAGCAATGAAAGTCTTGATATCTTTTGTCATCTTGTTTTTCTTA
TATTTTGTAGGCGTTGCTATAGAAACATATCACTATACTCAGCCAGAAAACAAAGTGCTATTTACTTTTGGTATGGCAAC
CATAGCCATCTATCCCTGGGGTCACTCATTTATCCTAATTCTAGGAAACCGCAAGCTGAAGCAAGCCTCTTTGAAGGTAC
TGAAGCATGTAAAGTGCTGGGAAAGAGAGAAACTTCTCAGAATTCCATGA
>VipaTAS2R16_NW_005882734.1:3418675-3417788
ATGATACCCATCCAACTCAATGTCTTCTGCATAATCGTCTACATGCTCGAGTCCTTGATAATAATTGTGCAGAGCAGCTT
AACTGCTGTGGTGCTGGGCAGAGAGTGGGTGCGGGTTAAAGGGCTGTCACCTGTGGACAAGATTCTCACCGTTCTGGGCA
TCTGCCGCTTCTGTCAACAGTGGTCATCGATGCTGTTCAATTTCTGCTCCTACCTCCACCCTAACTACGTATTTTGGTAC
TTGGCAATCGTCTGGGAATTTATTAACACTCTTTCGTTCTGGTTAACCTCCTTGCTTGCTGTCTTCTACTGTATCAAAGT

CTCTTCCTTCAGCCATCCCATCTTCCTCTGGCTGAAGTGGAGAATTGTGAAGTTGGTTCCTCGGCTGCTGCTGGGTTGTC
TGCTGATTTCTTGTCTGTCAATCATCTTTTCAGCTATGAGGCATCGCATCACAATTCAGTTAAAGTCTATGAGGCACTTT
GCTAGAAACAGCACTGTGATTGAAAGACTTGAGACATTTCAGTGGGATTTTTCCATATATCAGCAAGTGGCGGTGCTGGT
TATTCCTTTCCTCCTGTTCCTGGTCTCCGTTGTCTTGCTCATGACCTTATTGTCCCAACATCTGAGGCAGATGAAACATG
ATCACACTGGGCACTCCAGCTCCAGCCTGAAAGCTCACGTTACTGCCCTGAGGTCTCTGGCCATCTTCCTCATCATCTTC
ACATCTTATTTTCTGGTCATACTCATCTCCATCATAGGTACCCTCCTGGATAAGGGGTCCTGGTTCTGGGCCTGGGAAGC
TGTCATCTATGCTGTAGTCTCTATTCATTCCATTTTACTGATGCTGACCAGCCCTAAGTTGAAAAAGGCCTTACCGGTAA
GATGCTAG
>VipaTAS2R38_NW_005882807.1:3902848-3901841
ATGTTGACCCCGGCTTCCATCGTGACTGTGTCCTATGAAGTCAAGAATGCTTTTCTGTTCCTTTCAGTCCTGGAGTTTGC
AGTAGGGATCCTGGTCAATGCTTTCATCTTCTTGGTGAATTTTTGGGACGTGGTGAGGAGGCAGCCACTGAGCAGCTGTG
ATCTCATCCTTCTGAGCCTCAGCCTCACCCGGCTGCTCCTGCATGGGCTGCTGTTTCTGGACGCCGTTCAGCTGGCCTGT
TTCCAGCGGATGAAAGACCCACTGAGCGTCAGCTACCAAACCATCATCCTGCTCTGGATGATCGTGAACCAGGCTGGCCT
CTGGCTCACCACCTGCCTTAGCCTCCTCTACTGCGCCAAGATCGTCCGTTTCTCTCATGCCTTCGCGCTCTGCTTGGCGA
GCTGGGTCCCCAGAAAGATGCCTCAGATGCTCCTGGGCGCCATTACCTTCACCTGCGTCTGCGCCACTCTCTGTCTGGGG
GACTTTTTCGGGAGATCTCACTTCTCAGTCACAGCTATGTCTTTCGTAAATAACAGTACAGAGTTCAATCTGCAAATTGC
AAAATTTAGTTTCTTCCATTCCTTCCTCTTCTGCATTGTGGGGTCCATCCCTTCTTTCCTAATTTTTCTGGTTTCTTCTG
GGGTGCTGATTGTCTCCCTGGGAAGGCACATGAGGACGATGAGGGCCCAAACCGAAGACTCTCGGGACCTCAGCCTGGAG
GCCCATATCAAAGCTCTCCGATCTCTCATCTCCTTCCTCTGCCTGTACCTGCTGTCACTCTGCGCCGCCCACCTCTCCGT
GCCGTTGCTGATGCTGTGGCACAGCAAGGTCGGGGTGATGGTCTGCGTAGGCATCCTGGCAGCCTGCCCCTCGGGACATG
CCATCATCCTGATCTCAAGCAACGCCAAGCTGAGGAGAGCCCTGGAGAGCATTGTGCTGTGGGCTCAGAGCGGCCGAAAG
GTCAGGGTGGGCCACGAGGCCGCTTGCGGGACGCCGACTCTGTGCTGA
>VipaTAS2R39_NW_005882807.1:4613643-4614617
ATGACCGAAACCTGCAATCTCACAGAAAATGAATTTTCACCATTTCTCCTCATTTTAACTTGTATAATTATAGGAAGTGA
ATGCACCATTGGTATCATTACAAATGGGTTCATTGTGGCTATAAATACAGCTGAATGGATGCAGACAAAAGCAGTTTCCA
CAAGCGGCAAGATCCTGCTTTTCCTGAGTGTGTCCAGAATAGCTCTACAAAGCTTCATAATCCTAGAAATTATCTTCAGT
TCAACACTCCCATATTTTTATAATGAAGAAATTGTATATGACACATTCAAAGGAAGTTTCATGTTCTTAAATTACTGTGG
CCTCTGGTTTGCTGCCTGGCTCAGTGTCTTCTACTTCGTGAAGATTGCTGATTTCTCCTACCCCCTTTTCCTCAAGCTGA
AGTGGAGAATTTCTGGATGGATGCCCTGGCTTCTGTGGCTATCCGTGTTTATTTCCTTGGGCACCTGTGTGTTCTTCTTC
AAAAACGTCTACACTGTGCACTGTAACAATTACGTTCCTGTCCCCTCCTCCAACTCCACTAAGAAAAAAGTCTCCACTGA
GACCAATGTGACCAACTTGGCTGTTCTCTTTCAAATAGGGATCTTCATTCCTCTGATCATGTTCATCCTTACAGCCACCC
TGCTGATCGTCTCTCTCAGGAGACACACCCGACACATGTCCAGCAATGCCACAGGTTACAGGGACCCCAGCATGGAGGCT
CACCTGGGGGCCATCAAAGCCACCAGCTACTTTCTCATCCTCTACATTTTCAACTCAGTCGCTCTGTTTCTCTATATGTC
CAACATCTTTGATATCAGCAGTTTCTGGAGTATCTTGTGCAAAGCCATCATGGCCGCCTACCCTGCTGGCCACTCCATTC
TCCTGATTCGGGACAACCCTGGGCTGAGAAGAGCCTGGAAGCGGCTCCAGGCTCGAGTTCATCCGTACAGGGTAGACTCC
ATGACCCACATGTGA
>VipaTAS2R40_NW_005882807.1:4643793-4644929
ATGGCCACAGTGAACACAGATGCCACGGATAAAGACACAACCACGTTTAAAATCGTCTTCATCTTGGCGGTCTCCGGAAC
AGAGTGCATCACTGGCATCGCTGGGAACGGCTTCATCACGGCCATCCACGGGGCCGAGTGGGTCAGCGGCAAAGGACTGC
CTGCTGGTGACTGCATTCTACTGATGCTGATCTTTTCCAGGCTCTTGCTACGGGTTTGGATGATGCCAGAGAACACTTAC
AGTCTGCTCTTCCCGGTCACTTATAACCAAAATGCAGTGCATACGCGTTTCAAAGCCATCATCATGTTTCTGAACTATTC
CAACCTCTGGCTTGCCGCATGGCTCAGTATATTCTATTGTCTTAGAATCGCAAACTTCACTCACCCTTTGTTCTTCGTGA
TGAAGAGGAAAATCATGGTGCTGATACCCTGGCTTGTGAGGCTGTCACTGTTAATCTCTTTATGCTCCAGCTTCCCCTTC
TCTGTAGATATCCTCAGTGTGTCTGTGAATACTTCCGAACCTATTCCTTCCTCCAGCTCCCCTGAGAAGGTGTACATCTC

CAAAGCCAACATGGTCAACTTGGTTCTCACCCCTTACCCGGGGGTCTTCATTCCTCTGATCATGTTCCTCCTTGCGGCCA
CCCTGCTGATCGTCTCTCTCAAGAGACACACCCTACACATGGCCAGCAATGCCACAGGGCTCCAGGGACCGACCCCAGCA
TGGAGGCTCACATGGGAGCCCTCAAAGCTATCAGCTGTTTTCTTATTTCTACATTTGAAGTGCCGCTGCTCTATTTCTT
TCCATGTCCAACATCTTTGATGTCAACGGTTCCTGGAGCATTTTGTGCAAAACCGTCATGGCTGCCTACCCTTCCAGCCA
CTCAGTGCTATTGGCCTTGGGCAACCCTGGGCTGGAAAGAGTGTGGAAGCGGTTTCAGCACCCAGTTCATCTTTGCCTGT
AGGGACAGACTCTGTGACTGGAACCTGCAGGGTACCACAGGACCTGACCCAACCAGCTCTGCCTTTCCCTCACTGAGACC
TCTCTCCTCACCCATCTTTTCTTCCCTCATCTGCTCTGACACGTCTCCTCAGGTAACTGGATACTTTCATGAATGTGAAG
CTGATTTTTTAATTTGA
>VipaTAS2R42_NW_005882720.1:752934-752026
ATGTTCACTGGGTTGAGTGCAATCTTCCTGATGCTGTCAGTAGTGGAATTCATAATCGGAATGTTGGGGAATGTGTTCAT
CGGACTGGTAAACTGCTCTGAGTGGGTCAAGAACCAAAAGATCTCTTTATTTGACTTTATCATCACCTCCTTGGCTATCT
TTAGAATTGGTCAACTGTTGGTGTTTTTGTTTCAGTCCTTTTTAATGGGACTAGATCCACATTTACTTTTTACTCTTAAA
CTAGTAAAAACTATTAGTTTGCTTTGGAGAATCACTAATCACTTGGCTACCTGGCTTGCCACCTGCCTAAGTATTTTCTA
CCTCCTTAAGGTAGCTCACTTCTCCCACTCCCTTTTCCTCTGGCTGAAGTGGAGAATGAACAGAGTCATTCTTGTAATAT
TTGTATTTTCTTTGGTCTTTCTGTTTTTTGACTTTCTCTTGCTAGAAACATTTAACGATCTCTTCTGGATGACAGCAGAT
GAAAGTAATCTGACTTTGTATTTAGATGAAAGGAAAATGTTCCACGTACAAAGTGAGATTCTTCTTAGCTTGACCTACCT
CATTCCTGTTGTTCTGTCCCTGATCTCACTGCTGGTTTTATTTCTGTCCTTGGTGAAACACACCAGAAATTTGCAGCTCC
ACTTTGTGGGCTCGAGGGACCTCAGCACAGAGGCCCATAAAAGAGCCATGAGAATGGTGATGTCATTCCTACTCGTCGTC
ATGATTCATTTTTTTTCCATGCAGTTGGCAAACTGGATGTTTTTTATGTTTTTTGATAAGAAGTTCACAAAATTCATCAT
ATTGACAGCATATGTCTTTCCCTCAGGCCATTCGTTTATGTTGATTCTGGGAGACAACAAACTGAGACAGCGAGCTTTGA
AGGTACTGAGTCATCTTAAAGTCTCCTAG
>VipaTAS2R62_NW_005882807.1:4817821-4818753
ATGTTCCCCTTACCTGTGCTGATTTTCATGGCCATCTTTCTCCTGGAGTCACTGGCTGCAATGTTACAGAATGGCTTCAT
GGTTACTGTGCTGAGCAAGGAGTGGGTGCGATGTCGGACACTGCCCACAGGTGACATGATTGTAACCTGCCTGGCTGCTT
CCCGGTTCTGCCTGCATGGGATGGCCCTCCTGAACAACTTCCTATCTTCCTTTGGTTTTTATACCGTAACTAAATATTTC
AGCATCCCCTGGGAGTTCATCAACTCTCTCACTTTCTGGCTTACTGCCTGGCTTGCTCTCTTCTATTGTGTGAAGATCTC
ACTCTTCTCGCACCGCATCTTCTTCTGGCTGAAGTGGAGGGTTTCTCGGTCAGTACCTGGGCTGCTGCTGGGCTCCCTGG
TCCTGTCTGGTATAGCAGTCGTCTCATCAGTCATTAGGAATATAATTCTAACATGGAGGACTGAGACCCAGAGTTCCCAG
GGAAACAGCACCCTGGCTAGTAGACTACAGACCGTCTCTTCATACATTTTTCTATCTCATATTATTCTTACGTGGTTGAT
TCCATTTCTCCTGTTCCTGGTGTCCATCCTCTTGCTCATGTTCTCACTGTGCCGGCACTTGAGGCAGATGATGGACCATA
GACCTGGCCCGAGTGATCCCAGCACACAGGCTCACACTATCGCCCTGAAGTCGCTTGCCTTCTTCCTCATCTTCTACACA
TCATACTTCCTGTCCCTGGTTATTGCTGTTAGTAACATCAAAGCCCTCCAGAGCCACTGGCACTGGGCCTGGGAAGTGGT
GATCTACGCAGGCATCTGTCTGCACTCCAGCATCCTGGTGCTAAGCAGCCCCAAGCTGAGAAAGGTCCTAAAGAAGAGGC
TTTGGAGAGCCCTGGGCAAAGAGCAGTTTGTCTCGAGTTATCAGTGTCAGTAA
>VipaTAS2R67_NW_005882720.1:745706-744765
ATGCCATCTGGAATTGATAATGCTCTTCTAGCAGCCATAATAGGAGAAGTCATAATTGGAACGTTGGGGAATGGGTTCAT
TGTACTAGTTAACTGCATTGGCTGGGTGAAGAGGAACAAGTTCTCATCAGTCGACTGCATCCTCACCGGCCTGGCTATCT
CCAGAATCAGTCAACTTTGGATAACACTGTTTGAATCACTTTTAATGTTGTTTTGGTCACATCTCTATGCCACTGATAAA
TGTTTAATAAGTATTGTTAGCATTTTTTGGACATTGTCCAATCACCTAGCTACCTGGTGCGCTACCTGCCTAAGTGTTTT
CTACTTCTTACAAATAGCCAGGTTCTCCCACCCCTGCTTCACCTGGCTGCGCTGGCGAATTCACAGGGTGATACTTGTCC
TTCTGCTGGGGTCTTTGTTCTTACTGGTTTTCAACTCTAAATTAATACATTCGTTTGGTGAATCCTGGACTAGTATCTAT
AAAATAGATGAAAGAAACTCAACTGGGTCCTCGGGCGAAATGAAAACTCTGTATCTTAATGGGTTGATTGTTTACAGCTT
GATTTGCTTAATGCCCTTTCTTGTGTCCCTGACCTCACTGCTCCTTTTATTCATCTCCTTGAGAAGACACACCAAGAATC
TGAAGCTGAACCCTAGCTCTAGGGACCTCAGCACAGAGGCCCATAAAGGGCCATGAGAATGATAATTTCTTTCCTCCTA

CTCTTCTTGGTCCACTTTTCTTCCATTCTATCAGTGGGCTGGGCATTCATTATACTGCAGAAGCATCAGGTCAACTTGGT
TGTCATGTTAACTTCAATTGTTTTTCCTTCAGGCCACTCATTTATCCTAATTTTGGGAAACAGCAAGCTGAGACAAAATG
CCTTAGGACTACTGTGGTATCTTAATTGTCACCTGAAAAGAATGAAACGTTTAGCTTCATAG
>VipaTAS2R5P_NW_005882807.1:3812869-3813753
ATGCTGACTGCCGTCCTAGGACTGCTGGTGCTGGTGGCAGTAGCTGAATCTCTCACTGGCCTGGTTGGAAGTGGAGTCCT
TGTGGTCTGGCATTTTGGGGAATGGGTCAGAAAACTCGAGGGGTCCTCATATAACCCCATTGTCCTGGGCCCGACTGTCT
GCTGATTTCTCCTGCAGTGCTTGATTGTGGTGGACTTAAGTCTGTTTCTGCTTTTCCAGGGCAGCCATTGGCTTCACTGT
CTCAGTGTCTTCTGGGTCCTGATAAGCCAGGCCAGCCTGTGGTTTGCCACTTTCCTCAGTATCTTCTACTGTAGGAAGAT
CACAGCCTTTGAACACCCTATCTACTTGTGGCTGAAGCAGAGGGCCTGTTGCCTGAGTCGCCGGTGCCTTCTGGTGTACC
GCAGTTTGTTACTTACATTCCACGGTGGCTTAGAGCTCTGCCATTCTTCCAAAGGAAACAGCAGCGTTTTATTCCCCCTT
TCAGACTGGCACCATGTGTATACATTACAGTTCAGTGCAGGAAGTGTGTTGCCTTTCATGATGTTTCTTATTTCCTCTGG
GATGCCGATGGCCTCTTTGTGTAGACACCACAGGAAGATGAAGGTCCACACAGCCAGCAGGAGAGTTCTTCAGGCCACGG
CTCACATCACTGTGCTGAAGTCCTTGGGCTGTTTCCTCATACTTTACATAGTTTACTTCGTGGCCATCCCCTTCTCCATC
ACCTCCAAGTCTTTTCCTGCTAATTTCGCCACTCTCTTCATCTCTGAGACACTCGTGGCTGCCTACACTTCTCTCCATTC
TGTCATCTAGATCACGGGAAATCCCAGGTTGAAGCAGACTTGCCAGAGAATCCTGTGGAAGACAGTGTGTGCTAGGAGAT
CCTGG
>VipaTAS2R11P_NW_005882936.1:2112588-2112163
AACCAGTCTACTGTGGCTAATAATTGTCAATTTGTTTTTAGTGCTATTCTATCAGGAAACTCTGATACCATGGGGAAAAA
ATATATCTTACTGGCATTTGGATACTGGCCAACCACTTGAGCACTTGGTTTGCTACTTGTCTCGCTGTCTTGTATTTCCT
GAAGATCACCAATTTCTCCTATCCCCCTTTCCTTTGGCTAAAATGGAGAATTAACAAGGTAACTGTCATGCTTCTGCTGG
CATCTGTGCCCTTCCTGTTCATAAGCTTTCCTTTGCCATATGGTTTTGATGTCTTTTGGCATTACAGCCTCCCAAAATGT
GAGACAAATATGACTGGGTTATTCAATATAAGCAAAAATAAAAATTTGAAATCTACGATTACCTTCACAGATTTTTATTG
ATTCACCAGTACAGTTCCCGGAGGTC
>VipaTAS2R13P_NW_005882720.1:719835-718983
ATGGTAAATGTCTTGAATATCATCTTCATCATTTTAATACACTCAGAATTAGTAACTGGTATTTTGGGGAATGGATTCAT
AACACTGGTGAACTGCATTGACTGGCTCAAGACTTGAAAGATCTCCTCAGCTGATCGAATCCTCACCACGTTGGCAATCT
TTAGAATTTGTCTGATTTTGGTAATAGTGGTGAGTTGGTTTACAAGGGAATTTTCTCCATCTTTATATATGAATAGAAAG
AATGTTATACTTATTACTATTTCTTTTATTCTCTCAAGATAGCCAATTTTACAAATGCTGTTTTTCTTCACCTAAAGCAT
AGAGTTGAAATGGTAGTTCTAGTAATGTTTCTGGGGGCATTAGTATTGTTGCCTTTATGTCTTATTATGATAAGTATTCT
TATTAATATCCAGATACATCCATATGAAAGAAATGTAACTTTGAGTTCTAAAAGGAGTGACGCTGAAAACTTTGTGAAAC
TGATTATATTCAGTATGGGAGACTTCATACCCTTTATTATATCCCTGATTTTTTCCTCCTGCTAATCTTCTCCTTATTGA
AACATCTCGAGAAGATGAAGCACCATGCAATGGGATTCAGGGATCCCAGCATCAAAGCCCACGTCAGTGCCATGAAAATG
GTGATATCTTTCCTCCTGCTATTTGCTGTTTACTTCCTGTCTATTCTTATGACAGCCTTCCATTCTGATGTGACTCAGAA
CAAATCGACCCTTATGCTTGGTCAGGTTCTTGCAAATGCTTATCCTTCAGTCCACTCATTTGTCCTAATTCTGGGAAACA
GTGAGCTATGAAGGGCTTCACTTTCGGTGTTGCGGCAGCTGAAATGTGGTTTA
>VipaTAS2R41P_NW_005882807.1:4844558-4845484
ATGCATCCAGCACTCACAGCCCTCTTCGTGCTGCTCTTTATCCTGCTGTGTGTCCTGGGAGTCCTGGCCAATGCTTTCAT
TGTGCTGGTGCTGAGCAGAGAGTGGGTGCGACGGGGGAGGCTGCTCCCCTCCGACATGATCCTCATTAGCTTGAGTGCCT
CCCGCTTCTGCCTGCAGTCGGTTGGATGGGGAACAACTTATACTACTTCCTCCACCTGGTCGAGTACCGTCGGGGTCCTG
CCTGGCAGTTATTTGGTCTATTCTGGGACTTTCTGAACTCAGCCACCTTCTGGTTTGGCTCCTGGCTCAGCGTCCTCTTC
TGCATGAAGATTGCTAACTTCACCCACCCCACCTTCCTCTGGCTGAAATGGAGGTTCCCAGGGTTAGTGCCCTCGCTTCT
GCTGGGATCTCTCCTCGTCGCCTTCATTGTCACCCTGCTGTTCTTTTGGGGGAATCACGCTCTGTATCGAGGATTCTTTA
TTGGAACATTTTCTGGAAATATGGCCTATAAGGAGTGGAGCAGGAGGCTGAAAATACACTATTTCCTGCTCCTGAAGCTA
GTCACCCTTTCAATTCCTTGCTCTAGTTTTCTGGTCTCAATTGCTCTGTTGATAAGTTCTCTGAGGAGACACACGAGGAG

GATGCAGCACAGTGCACACAGCCTGCAGGACCCCAACGCCCAGGCTCACACCGCAGCTCTGAAGTCACTCATCTCCTTCC
TTGTTCTTTACCTTCTGTCCTTCCTGTCCCTGGTTATTGATGCTGTAGTGTTTTTCTCCTCAGAGAGTGAATGGTACTAG
ACATGGCAAATTATAACCTACTTGTGCACATCCGTCCATCCCTTTTATCCTCATCTTCAGCAACCTTAGGCTTCGAGGGA
TATTCAGGCAGCTAATTCTGTTGGCCAGGGGCTTCTGGGTGGCCTAG
>VipaTAS2R40BP_NW_005882720.1:725659-724749
ATGATGAATTTACTACCAAGCATTTTTTCTATCCTGTTAATAGCAGACCTTATTCTAGGGAATTTTGCCAGTGGCTTCGT
AGCACTGGTGAACGGCACTGACTGGGTCAAATGACCAAAGATCTCCTCAGCTGATGGAATTCACACTGCTTTGGGAGTCT
CCAGAACTGGCTTACTCTGGGTAATATTAACAAATTGATATGCAAATATGTTTAATCCAGATTTAGACAATTTAAGAGCA
AGAATTACTACTATTGCCTGGACAATAGCAACCATTATAGCATCTAGTTTGCTGCTGCCTTCAGCATATTTTATTTGTTC
AAGATAGCCAATTTCTCCAGCCTTATTTTCCTTCACCTAAGGTGGAGAATTAAAAGTGTACTTCTCGTGACAACGTTGGG
GACTTTGTTCTTTGTGGTTTTTCAGTTTGCGACAGTAAGCATAGATGAAAATATCCAGACAAATGCATATGAAGGAAACA
TTGCTTGGAGGACCAAACTGAGGGCCATTTTACACATTTCAAATATGACTCTGTTCACGCTAATAAACTTCATACCCTTT
ACTATGTCCCTGATATCTTTTCTGCTGTGAGTCTTTTCCCTATGGAAACAGTTCAAGAAGATGCTGCTCAGTAGGAAAGG
ATCCCAAGATCCCAGCACCAAAGTCTATATAAAAGCCATGCAAACTCTCTCTTGTCTCTTGCTATTTGCCCTTTACGTCT
GGGCTCTAATTTTCTCAATTTGGAGTTCCAGTAGGCAGCAGAACACACTAGTTATCATGGCTTGCCAGGCTGTTGGAATC
ATATACCCTATAAGCCACTTGTTTATCCTGATTTGGGGAAATAAGGAGCTAAGACAGGCCTTCCTGTCTTTTCTGTGGCA
GCTGAGGTGTTGGCTAAAAGAAAGGAAATAA
>VipaTAS2R60P_NW_005882807.1:4823533-4824495
ATGGGTAGAGAGGACATAGTTCCAGGACCTAAGGTGACTGATAAAGAGAGCCATCATCTTCGTTATCATTTTATTCCTTT
TGTGCCTGGTAGCAGTGGTGGGTAACGGTTTAATCACTGCAGCACTAGGCATGGGGTGGTGGCTGCAGAGAACGTTGTCA
CCCTGCAATAGGTTATTGGTCAGCCTGGGTCCTCTTGCTTCTGTCAGCAATGGGTGGTGATAAGTAAGAACATGTATATT
TTCCTGTATCCAATGGCCTTCCCACACAACCTTGTATTACAGTCCCCAGCCATTCAGGGGGACTTCTTGAATGCTGTCAC
GTTATGGTTCTCCACCTGGCTCAGTGTCTTCCACTGCATGAAAATTGCAACATTCACCCACCCTGCCTTCCTCTGGCTAA
GGAGCATGGTGTCTGGGTTTGTCGCTTGGATGCTGCTCAGCTCTGTGTGGTTCTCCAGCATCAGCACCAGCACCATTCTA
TTTTTCATAGGCAACCAGAGAATATACCAGAACTATTTAAGGAAAGGTCTGCAATCTTGGAATGTCACTGGGAATACTCT
GAGGAGAACATATGAGAGATTGTACTTCTTCCCTCTGAAAATTGTTACCTGGACAGTCCCTACAGTTGTCTTCATCACTG
ACATGGTTTTGCTCATTACATCTCTGGGAAGACACACCAAGAAGGTCTTCCTGTCCATCTCAGGTTTTCCTGATCCCTGT
GCCCAGGCACACGTCAAGGCTCTCCTGGCTCTCATCTCCTCTGCCATCCTTTTCATTCCCTATTTCTGTCACTGGTACT
CAATGCTGCAGGCATGTTTCCACTCCAGGAATTCAGATGCTGGGTATGGCAGACTCTGATTTATCTGTGCATGGCAGTCT
ACCCCATCATCCTACTCCTGAGTAACCGCAGGCTGAGAGCTGTGCTGAAGAGGTGCTGCTCCTCAAGGTGTGGGGCATCT
TGA
>VipaTAS2R18P_NW_005882720.1:731397-730484
ATGTCAGTGGGAATGAAGGTCTCCATTCTGGGAGTGGCAACAGGAGAACTGATCTTAGGAATGCTGGGAAATGGGCTCAC
TGAACTGGTAGTCTGCATGGAATGGGTCAAGAATGGGAAGGTCTCATCAGCTGATTTCATCCTTACCAGTTTATCTGTGG
CCAGAATCAGTCAGCTGTGGGTAACACTACTGGATTCATTTATAATATGGCATCTCCACATGTGTATGCCACTGGCAAAC
TAGCAAGAGTGTTCTTTGGGCACTAACGAATCACTTAACTACCTGGTTTGCCGCCTACCTGAGCATTTTCGTCTTCCTTA
ATGTGATCAGTTTCTCCCAGTTTTTTTCATCTGGCTGAAGAGGAGAATGAACAGAGTGGCTCTTGTGCTTTTCCTGGGCT
CTTTGTTCTTACTGTCTGTTAACCTCCTAATGCAGGACGGCTCTTGGTGAGTTGTGGGTGAATAGCTACAGAGGACGTGA
GAGAAACACGACTTTGTATTTAGATGGAAGTAAACTTTTCTGTCTTAAAAGCCTTGTTCTTCTTGATCTGTGTTATCCTG
TTCCTTCTCTCCCTGGCCTCTTTGCTGCTGTTATTTCTCTCCTTGGTGAAACATATCAAGAATCTACAGCTCCACCCGAA
TGAATGGAGAGACCCATAAAAGGGCCATGAAAATGGTGACAACCTTCCTCCTTCTCTTCATCATTTATTTTATTTCCACT
CTAATGGCAAGTTGGATTTTTCTTAAGGTACAAAGTTATCAGGGCATGACATTTTGTCATGGTGATTTCAATTCTCTTTC
CCTCAGTTCACTTCTGTGAATTTCAGTTTTTGGACTAGCAAGCTAGGGTAGATTGCCTTGAAACTACTGTGGTACCTTAC
ACTCTCTCTAAAAAAAGCGAAACCTTTAGCTTAA

＞PahoTAS2R1_NW_005807439．1:88767-89657
ATGCTGGAGTCTCACCTTGTCAGCCACCTTGTTTTGACAGTGATACAATCTCTCTTTGGGATTTTAGTAAATGGCATCAT
TCTGATTGTGAACGGTACTGACTTGATCAAGCAGAGAAAGTTGATCCCACTGGATCTCCTTGTTTCCTGCTTGGTGATTT
CCAGGATGGGAATTCAGCTGGCCTTCTTCTACATTAACCTGGCTCTTCTTTCCTTGGTCAAATTCCCTCAGGTTACTGAG
AAGCTTGTAGTTTTCACATTTGTAAATGATTTGGGACTTTGGTTTGCCACCTGGCTCAGTGTCTACTACTGTACCAAGAT
TGCTACCATCGCTCACCCTCTCTTATTTTGGTTGAAGATGAAGATCTCCAAGCTGGTTCCTTGGCTGATTCTTGGGTCCC
TGCTGTATGCAGGTAGTACTTCTGCTGTGCATGTCAAATATAAGTGGGCATTTTACGGAGAAGACTTCCTGGGCCTTTTC
TTCCCAAATGTAACAACTCACATCAAAGTAACCCCGACTTTACAGTTTGCCTTTCTGTTAGCTGAGTTTGCGTTGCCGTT
TTTCATCTTCCTGATTTCTTCTCTGCTCTTGATATTTTCCTTGGGAAGACACACCTGGCAGGTGAGAAACACATGGACAG
GCCCCAGAAACTCTCACACACATGCATACGTCAGGGCCTTTCACTCCATCCTGTCCTTCCTGGCCCTCTATCTCTGCCAC
TACCTGATCGTTGCTTTGATCTTTTTTCAAAGGTTTAAACTTAGAAGCTTCCTGTTTCTGTTCTGCACCTTTGTGGTTGG
TTCATACCACTCCATCCACTCTATTACTTTAATTTTAGGAAACCCGAAAATGAAACAAAATGCAAAGGCGTTGCTCCTCC
TCAAAAAGTGA
＞PahoTAS2R2_NW_005815815．1:607141-606233
ATGATCTCTTTGTCAGTTATTCCGCATGTTATCATCATGTCTGCAGAATTTATTACAGGGGTTACAGTAAATGGATTTCT
TATAATCATCAACTGTAATGAATTGGTCAAAAGCAGAAAGCTAACACCCATGCAACTCCTGTTCGTATGTATAGGGATGT
CTAGATTTGGTCTACAGACTGTGTTAATGGTACAAGGTTTTTTCTCAGTGTTCTTTCCACGCTTTTATAGCACAAAAATC
TATGGCACACCAATGCTCCTTTTTTGGATGTTTTTCAGCTCTGTCAGTCTCTGGTTTGCCACCTGTCTCTCTTTATTTTA
CTGCCTCAAGGTAACAGGCTTCACCCAGTGCTGTTTTCTTTGGCTGAGAGTCAGGATCTCAAAGTTAATGCCTTGGATGC
TCCTGGGAAGCCTGCTGACCTCTGTGAACATTGCAGCTCTGTGTGTCAAGGTGGATTACCCTAAAATTGTGGATATTGAT
GTCCTCGGGAATGCCACAGCTAAGAGGATTAAACTCAACACAAAGCAAATTAATGAAGTTCTTCTCGTCAACTTGGCATT
ACTATTTCCTCTGACCATATTTATAATATGCACTGTTATATTATTCATTTCTCTCTACAAGCACACTCATCGGATGCAAA
ATGGACCTCTTGGTTTCAGAAACACCAGGACTGAAGCCCATATTAATGCATTAAGAACAGTGATAACATTCTTTTGCTTC
TTTATTTCTTACTTTGGTGCCTTCATGGCAAATATGACATTCAATATTCCTTATGGAAGTCATTGCTTCTTTGTGGTGAA
GGATATTATGGCAGCATATCCCTCTGGTCATTCGGTTATAATGATCTGGAGTAATTCTAAGTTCCAGCAACCAATCAGGA
GACTTCTCTGCCTAAGAAGGAGTCAGTGA
＞PahoTAS2R3_NW_005817127．1:1383493-1384443
ATGTTGAGACTCAGCAATTTGGGGTTTCTGGTTCTGACCTCCATTCAGTTCATCCTGGGAATGCTGGGGAATGGTTTCAT
AGGGTGGGTCAATGGCAGCAGCTGGTTCAAGAGCAAGAGGATCTCTTTGCATGACTTCATTATCACTAACCTGGCTGTCT
CCAGGATTGTTTTGCTGTGGATTCTCTTGATTGATGGTGTTTTACTGGTGTTCTCTCCCAAACTACATGATGAAGGGATA
ATCATGCAAATTATTGATGTTTTCTGGACATTTACAAACCATCTGAGCATTTGGCTTACCACCTGTGCCAGTGTCTTCTA
CTGCCTGAAAGTGGCCAGTTTCTCCCATCCTATGTTCCTCTGGCTCAAATGGAGAGTTTCCAGGGTGGTTGTATGGATGC
TGTTGAGTACCCTGCTGTTATCATGTTGCAGTGCCATCTCTCTGATCCGAGAATTTAAGATCTATTCTGTTCTCAGTGGA
ATTGATAGAACCGGGAATATGACTGAGCTCTTTAGAAGGAAAGAAAAAGAATATAAACTGATCCATGTTCTTGGGACTCT
GTGGGACCTCCCTCCCCTAGTCATATCTCTAATCTCCTACTTTCTGCTTATCCTCTCCCTGGGGAGGCATATGCGGCAGA
TGCATCAAGACTGTGGCAGCTCCAGAGATCCCAGTACCGAGGCCCACAGGAGGGCCATCAGAGTCATCCTCTCCTTCCTC
TTTCTCTTCCTACTCTACTATCTTTCCTTTTCTGTTTTAACATCCAGTTATTTCCTACCAGCAACTAAGATGATTGCGAA
GATCGGAGAAGTAATTACAATGTTCTATCTTGCTGGCCACTCTTATGTTCTCATTCTGGGAAATAGCAAGCTGAAGCAGA
TGTTTGTGGCGATGCTCCGGTGTGAGCCTGGTTGTCTGAAGCCTGGATCCAAGGGATGTGTTTATCCATAG
＞PahoTAS2R4_NW_005817127．1:1396769-1397659
ATGCTTCGGATAGTCTTTTTTTCTTCTATCGTTGTCTCTGAAATTTTAACTTTTGTAGGACTCATTGTGAATCTCTTCAT
TGTAGTGGTCAGTTACAAGACTTGCATCAAAAGCCACAGGATCTCTTCTTCTGACAGACTCCTGTTGAGTTTGGGCATAA
CCAGATTTTTTATACTGTTACTGAATGTTGTTGTCATCATCTCTCCAAATGTGGAAAGGTCAGTCTCCTTATCCTCTTTC
TTCCTGTCATGTTGGATGTTTTTGGACTCTAGTAGTCTTTGGTTTGTAACCTTGCTCAACGTCTTGTATTATGTGAAGAT

TGCTAACTACCAACACTCCGTGTTTCTCCTGCTGAAACGAAATCTCACCACCAGGATGCCCCGGCTGCTGCTGGTCTGTA
TGCTCCTTTCTGTCTTCACCACTCTCCTGTATGTTATGCTCAGACAGTTGGCACCCCGTCTTGAATTTGTGACTGTGAGA
AATGGCACAGTATTTGACATCAATGAGGGACTCTTGTCTTTGGTGACTCCTTTGGTCTTGAGCTCATTTCTCCAATTCAT
CATTAATGTGACTTCTGCTTCTTTGTTAATCAATTCCTTGAAGAGACATATACGGAAGATGCAGAGAAGTGCCACTGTTC
TTTGGAATCCCCAGACTGAAGCTCATGTGGGTGCTATGAAGCTGATGATCTGTTTCCTCATACTCTACATTCCATATTCA
GTTGCTACCTTGCTCCATTATCTCCCTTCTTCTATAGGGATGGATTTGAGAACCAAGTCTATTTATGTTATTATGTCCAC
CATTTACCCTCCAGGACATTCTCTTATTATTATTCTCACACATCCTAAACTGAAAACAAAAGCAAAGAATATTCTTTGTT
TCAGTAAATAG
>PahoTAS2R5_NW_005817127．1:1410543-1411442
ATGCCGACTTCTATCCCAGGACTGATGATGCTGGTGGCAGTGGCTGAATCTCTCATTGGCCTCACTGGAAATGGAGTTCT
TGTGGTCTGGAGTTTTGTAGAATGTCTTCAAACGTCCAGGGAGTCCTCGTATAACCTCATTGTCCTGGGCCTGGAGGTCT
GTCGGTTGCTTCTACAATGGTTGATTATGGTGGACTCAAGTCTGTTCCTGCTTTTCCAGAGCAGCCATTGGCTTTGCTGT
TTCAGTGTCTTCAGGGTTCTGGTAAGCCAGGCCAGCCTGTGGTTTGTGAGTTTTCTCAGTGTCTTCTATTGTAGGAAGAT
CATGACCGTTGAACACCCTGTCTCCTTGTGGCTGAAGCAGAGGGCCTGTTACCTGAGTTGTTGGTGTTTTCTGGTGTACT
TCATGATCCATTTGTTACTTATAGTCAGGGGTAGCTTAGACTTCTCCAGTCCTTCCCAAGGAAACAGCAACATCTTATTC
CCCATTTCAAACTGGCACTATATATGTATATTACAGCTCAATACAGGAAGTATGATGCCTTTCATGATGTTTCCTCTTTC
CTCTGGGCTGCTGATTATCTCTTTGTATAGACACCACAGGAAGATGAAGGTCCATACAGCTGGCAGAAGAGATGCTCAGG
CCAAGGCTCGCATCACTGTCCTCAAGTCCTTGGGCTGTTTCCTTGTACTTTACGTGGTCTACACCCTGGCCAGCCCCTTC
TCCATCAGCTCCAAGACTTTTCCTGCGCATCTCATCACTCTCTTCATCTCTGAGACACTCATGGCTGCCTGCCCTTCTCT
TCATTCTGTCATTCTGATCATGGGGAACCTCAGGATGAAGCAGACTTGTCAGAGAATCCTGTGGAAGACTGTATGTGCTT
GGAGAGCCCAGGGCCTGTGA
>PahoTAS2R7_NW_005810531．1:1586741-1585803
ATGTCAAGTGAAGTGCAGGGTATCTTAATGCTCATAGCAGCTGGGGAATTTTCACTGGGGATCTTAGGGAACGCATTCAT
TGGACTGGTAAACTGTGTGGACTGGATCAAGCACAAGAAGATTGCCTCCATTGATTTAATCCTCACAAGCCTGGCCATCT
CCAGAATTTCTCTCTTATGTATAATACTATTGGATTGTAATATATTGGTCCTGTACCCAGATGTCTATACTGGTGGTAAA
CAAATGAGAATCATTGACTACTTCTGGACATTAACCAACCATTTAAGTGTCTGGTTTGCCACCTGCCTCAGCATTTTCTA
TTTCCTCAAGATTGCAAATTTCTTCCATCCATTTTTCCTCTGGATGAAGTGGAGACTTGACAGTGCAATTCCTAGGATCC
TGCTGGGGTGTTTGGTCTTCTCGGTGTTCATTAGCCTTCCTGTCATTAACAATTTGGATGATGATTTCAGGCATTGTGTC
AAGATGAAGTTGAAAACAAATATAAGTCGGAGATGCAGAGTACATAAAGCTCAGCATGCTTCCATCAAGATACGTCTCAA
TCTGTTGACACTATTTCCCTTTTCTGTGTCTCTGATTTCATTTCTCCTCCTGATCCTCTCCCTGTTCAGACACACCAGAC
GAATGCAGCTCCGTGCCCCAGGGAGCAGAGATCCCAGCACGGAAGCTCACGTGAGAGCCATGAAGGCTGTCATCTCCTTC
CTCCTCCTTTTCATTGCCTACTACTTGGCCTATCTTGTGGCCACGTCCAGCTACTTTATGCCAGAGACTGAATTAGCTGT
GATCGTTGGTGAGTTGATAGCTTTAATCTGTCCATCAAGCCATTCACTCTTCCTTATTCTAGAGAACAAAAAATTAAGAC
AAGCATCTCTAAGGGTGCTATGGAAGGTAAACTATATCCTACGAAGAAGGAATTGCTAA
>PahoTAS2R10A_NW_005810531．1:1616799-1615900
ATGCTGAGTATAGTAGAAGGCCTCCTCCTTTTTGTAGCAGTTAGTGAGTCAGTATTGGGGGTTTTAGGGAATGGGTTTAT
TGGACTAGTAAACTGCATTAACTGTGTGAAAAATAAGAAGATCTCTACACTCAGCCTTATTCTCACTGGCTTAGCCTCTT
CCAGATTTTGCCTGATATGGATAATAACTACAGATGCATATGTGAGATTGTTTTCTCCAGATATGTATTTGTCTGGTGAT
CTAAGTCAATATATAGCTTACTTATGGATAATTATGAATCAATCAAGTGTCTGGTTTGCCACCAGCCTCAGCATCTTCTA
CTTCCTGAAGATAGCCAACTTTTCCCACTGCATTTTTCTCTGGCTGAAGGGTCACATCAATAAGATCCTTCTTCTTCTAA
TGGGATGTTTGCCCATTTCATGGTTATTTACTTTTCCAAACATTACAATGCCTTTTATTAATAATATTATGAAGAACAGA
AACACAACCGGGTTGATCACCATGCAGAAAAGTGAATACTTTATAAATCAGATTTTGTTCAGTATCGGAACACTTCTTGT
CTTTATACTGTGCCTGATTACATGTTTCTTATTAATCACTTCCCTTTGGAAGCACAACAGGAGGATGCAATTGAATGCCA
CAGGATTCAGAGACCCCAGTACAGAAGCACATATCAAAGCAATGAAGATCTTGGGGTCTTTTATTATCCTCTTTATCCTG

TATTTTGTAGGCACTGCCATACAAATATCAATTGATACTATGCCTAAAAACAAACTGCTGTATATTTTTGGTATGACAAC
CACTGTCCTCTATCCCTGTGGACACTCATTTATCCTAATTCTTGGAAACAGCAAGCTTAAGCAAGCCTCTTTGAGGGTAC
TGAAGCTATTAAAGTGCTAG
>PahoTAS2R10B_NW_005810531.1:1647825-1646896
ATGCTGAGTATAGTGGAAGGCCTCCTCATTTATGTAGCAGTTAGTGAATCAGTATTGGGGGTCTTAGGGAATGGATTTAT
TGGAGTTGTAAGCTGCATTGACTGTGTGAAAAGCAAGAAGATCTCTACTGTCAGCCTTATTCTCACTGGCTTAGCCTCTT
CCAGATTTTGCCTGATATGGATAATAATTACAGATGCATATGTGAGGATGTTTTTTCCAGATACATATTTGACTGGTAAT
CTAAGTCAAAATATAGCTCACTTGTGGATAATTATGAATCAATCAAGTATCTGGTTTGCCACCAGCCTCAGCATCTTCTA
TTTCCTGAAGATAGCCAATTATTCCCATTGCACTTTTCTCTGGCTGAAGGGTCACATCAATAGGGTTCTTCTCCTTTTCA
TGGGGTCCTTGCTTATTTCATGGCTATTTGCTTTTCCAAGCATTGCAAAGCCTAGTATTAATGATATTATGAAGAACAGA
AGCACAACCTGGCTGATCGCCATGCATAAAAGGGAATACTTGACAAATTATATTCTGCTCAATATTGGAGTCATTCTTGT
CTTTGTACTATGCCTGATTACATGTTTCTTATTAATCACTTCCCTTTGGAGACACAACAGAAAGATGCGACTGAATGCCA
CAGGATTCAGAGATCCCAGCACTGAAGCACATATCAAAGCAATGAAGATTTTGGTGTCTTTTATCATCCTCTTTATCTTG
TATTTTGTAGGCACTGCCATACAAATATCAGGTAGTACTATGCCTGAAAACAAACTGTTGCTCATTATTGGTTTAACAAC
CAGACTCCTCTATCCCTGGGGACACTCATTGATCCTAATTCTAGGAAACAGGAAGCTGAAGCAAGACTTTTTGAGGGTAC
TGAAGCCATTAAAGTGCTGGGAAAAAGAGAAACTTCTTAGAATTCCATGA
>PahoTAS2R16_NW_005816990.1:2645333-2646238
ATGATAACCAGCCAACTCTCTGTCTTCTTCATGCTCATCTATGTGCTCGAGTTCTTGACAATAACTGGGCAGAGCAGCCT
GATTGTCATTGTGCTGGGCAGAGAGTGGGTGCAGACTCAAAGGCTGCCGCCTGTGGACATGATTCTCACCAGCCTGGGCA
TCTGCCGCTTCTGTCAACTGTGGTCATCGATGCTGCACAACTTTGGCTCACACTTCCACCTTAATTACAGTTTTTGGTAT
TTCGGGATCATCTGGCAATTTACTAACATCCTTTCCTTCTGGCTGACCAGCTTGCTTGCTGTCTTCTACTGTGTCAAAGT
CTCCTTCTTCAGCCACCCCATCTTCCTCTGGATGAAGTGGAGAATTGTGAGATGGGTTCCTTGGCTGCTGCTGGGCTCTC
TGCTGGTTTCTTGTGTGTCTACCATCTTTTCAGCTGCTAGTTATTACATCATCATTCAATTCATCTCCATGAAGGATTTC
CCTAGAAACAGCACCATGCTTGAGAGACTGCAGGCGTTCCTGTGGGATTTTTCCACACTGTGGAAAGCAGTTGTGTTGGT
TATTCCTTTCCTCCTGTTCCTGGCCTCCACAGTCTTGCTCATGGCCTTATTATCCCGACATCTGAAGCAGATGAAAGACC
TTCACACAGGCCGCTCCATCTCCAGCCCGGAAGCTCACTCTGCCGCCCTGAGGTCTCTTGGCATCTTCCTCATCTTGTTC
ACCTTTTATTTTCTGACCGTGCTCGTCTCCGTCTTGGATGTCCTATTTAATAAAGAGTCTTGGTTCTGGGCCTGGGAAGC
CATCATCTATGCATTAGTCTCTATTCATTCTACTTTACTAATGCTGAGCAGTGCCAAACTGAAAAGAGTTTTAAAGGCAA
GGTGCTGGAGCCTAGAAGCTGCCTGA
>PahoTAS2R408A_NW_005810531.1:1742236-1741319
ATGATAACTTTAGTATCGAGCATTCTTTCCATTCTAATGGTGACAGAATTTGTTTTGGGAAGTTTTGTGAATGGTTTCAT
AGCATTGGTGAACTGCAATGACTGGGTCAGGAAACAAAAGATCTCCTCAGCTGATGGGATTCTCACTGCTCTGGCAGTCT
GCAGAATTGTTTTGTTCTGGACAATATTAATAAATTGGTATGCAACTATGTATAATCCAGCTCTATATAGTGTAAGAATT
GTTATCCGTGTTGCCTGGACAGTAAGCAACCATTTTAGTAACTGGCTTGCTACTAGCCTCAGTATATTTTATTTGTTCAA
GATAGCTAATTTCTCCAGCTTAATTTTTCTTCACCTGAAGTGGGGAGTTAAAAGTGTAGTTCTCATGATGATGTTGGGGA
CTTCAGTGATTTTGTTTTTTCAGGTTGCAGTGTTAGGTATAGATGAGACTATTCAGACAAGTGAATATGAAAGAAACATC
ACTGAGAAGACCAAATTAAGGGACTTTTTACACCTTTCAAATATGACCCTGCTCACACTAACAAACTTCATACCCTTCAC
TATGTCCCTGACATCTTTTCTGCTGCTAATCTTTTTCCTGTGGAAACATCTCAGGAAGATGCAGCTCAACGGCAAAAGAT
CCCAAGATCCCAGCACCAAGGTCCACATAAAAGCCATGCAAACTGTCATCTCCTTTCTTTTCCTGTTTTCCACTTACATC
CTGACTGTAATTTTAACAATTTGGAATTCTAATGAGCTGCAGAAGGAATCGGTCCAAATGCTTTTCCAGGCCCTTGCAAT
CACCTATCCTTCAATGCACTCATTTATCCTGATTTGGACAAACAGGAACTTAACACAGACCTTTCTGTCATTTCTATGGC
AGCCAAGATGCTGGCTAAAAGTAAAAGGAACTAGGTAG
>PahoTAS2R39_NW_005816769.1:1306053-1307012
ATGATCCAAACCTGCAGTTCCTCAGAAAATGATCCGTCACCATCTCTTGTCACTTTGATGTTAATAATTATCGGCACGGA

ATGCATCCTTGGTATTCTCGCAAATGGGTTCATTGCAGCGATAAACACAGCTGAATGGATTCACAATAAGGTACTCTCCA
CCAGTGGCAAGATCCTGCTTTTCCTGGGTGTATCCAGAATAGTTCTACAAAGCTTCATGATGCTAGAATGTACCTTAAGC
TCAACATCCCCACAGTTTTATAATGACGACATCACGTATCACACATTCAGAGGATGTTTCATGTTCTTAAATCATTGCAG
CCTCTGGTTTGCTGCCTGGCACAGTGTCTTCTACTTCGTGAAGGTGGCGGATTTCTCCTATCCCCCTTTCCTCAAGCTGA
AGTGGAGAATTTCCGGACTGATGCCCTGGCTTCTGCAGCTTTCAGTGTTTGTTTCCTTGGGCCAGAGTGTGCTCTTCTTC
CAAAACATCTATACTATGAATTGTAACAATCTTTTTTCTCTCCCCTCCTTCAGCTCTACTAAGAAAAAGTTCTTCATGGA
GGCCACTGTGATCAACCTGGTTCTTTTCCTTAACCTGGGGATCTTCATCCCTCTGATCATGTTTATCCTGGCAGCCACCC
TGCTGATCATCTCTCTCAAAAGACACACCTTCCACATGAAAAGCAATGCCACTGGCTCCAGAGACCCCAGCATGGAGGCT
CACCTGGGGGCCATCAGAGCCATCAGCTACTTTCTCATTCTCTATATTTTCCAAGTACTTGCTCTCTTTCTCTACATGTC
CAACTTCTTTGACATCAATAGTCCCTTGAATATTTTGTGCAAAATCATCATGGCTACCTACTCTGCGGCCCATTCCATTC
TACTGATTCAGGACAACCCTGGGCTGAAAAGAGCCTGGAAGAGGCTTCAGGCTCAAGTCCACCTTTATTTTAAAAAGTAG
>PahoTAS2R60_NW_005816769.1:1580911-1581864
ATGAGCGGAGAAGATGTGGTTCCAGGACCTCAGGTGGTTGATAAGACAGCCCTCATCTGCATTGTTATTTTATTCCTTTT
GTTCCTGGTGGCATTGGTAGGTAATGGCTTAATCATTGCGGCACTGGGCAGCGAGTGGCTGCTGCGGAGAACGTTGTCAC
CCTGTGATAAGTTATTGGTCAGCCTGGGGACCTCTCGCTTCTGTCTGCAATGGGTGGTAATTAGTAAAAACATTTACATT
TTCCTGAATCCAACGGCCTTCCCATACAGCCCCATGTTCCAGCTCCTGGCCGTTCAGTGGGACTTCTTGAACTCGGCAAC
ACTGTGGTTCTCCACCTGGCTCAGTGTCTTCTACTGTGTGAAAATCGCAACCTTCACCCACCCGGTCTTCCTCTGGCTAA
AGCGGAATGTATCTGGGTTGGTTCCTTGGATGCTACTCAGCTCTCTGGGGTTCTCTACCTTTACCACCGTTCTATTTTTC
ATAGGCAACCAGAGAATGTATCAGAACTATTTAAAGAGGGGTCTGCAATCTTGGAATGTCACTAGGAATGCTGTGAGAAC
GTATGAGAGGTTCTGCCTCTTCCCTTTGAAAATTGTTACCTGGACCGTCCCTACTGTTGTCTTTATTGCGGGCACGGTTT
TGCTCATTACATCTCTGGGAAGACACACCAAGAAGGTCTTCTTCTCCATCTCAGGCTTTCACAGTTCCAGTGCCCAGGCA
CACATCAAGGCCCTCTTGGCTTTTATCTCCTTTGCTATCTTCTTCACTTCCTCTTTTCTGTCACTGGTTCTCATTGCCTC
AGGTATGTTTCCCTTTGGGGAGTTCCGGTTCTGGATATGGCAGATTGTGATTTATCTGGGTCCAGCAATCCACCCCATTC
TTCTTCTCTTAAGTAACCGCAGGCTGAGAGCTCTGCTAGGGAGGGGCTGCTCCTCAGCACATGGGGCATCTTGA
>PahoTAS2R67A_NW_005812598.1:319773-320711
ATGCCATCTGGAATTGAAAATACTTTTCTAGTAGCAACAATAGGAGGATTTGTGATTGGAATGTTGGGGAATGGGTTCAT
CATACTAGTTAACTGCATTGACCTGGTGAAGAGACAAAAGCTCTCATCAGCTGACTGCATCCTCACAGGCCTGGCTATCT
CCAGAATCAGTCAACTTTCAGCAATACTATGTGACTCATTTTTATTGGTACTATGGCCACACCTATATGCCATTGATAAA
CTAACAAAAGTTGTTAGCATTTTTTGGACATTGTCCAATCACCTAGCTACCTGGTTTGCCACTTGTCTAAGTGTTTTCTA
CTTCTTTAAAGTAGCCAACTTCTCCCACCCCTGCTTCACTTGGCTGCGGTGGCGAATTCGTAGTGTGGTACTGGTGCTTC
TCTTGGGGTCTTTGTCCTTACTGTTTTTGAACTTTGAATTAATACATGTGTTTAATAGTGTTTGGACTAATGACTACAAA
ATATATGCAAGAAATTCAACATGGTCCCCAAATGTAAGTGAAACTCATGATCTTCACCAGTTGATTGTTTTTAACCTCAT
CAGCTTAATCCCCCTTCTTCTGTCCCTGACCTCACTGCTCCTCTTAGTCCTCTCCTTGATGAGACACATCAGGAATTTGC
AGCTCAACCCCAGCTCAAAGGATCTCAGCACAGAGGCCCATAAAAGAGCCATGAAGATGGTGATGTCTTTCCTCTTCCTC
TTCGTCATTCATGTTTCTTCCATCCTATTAACAGGTTGGGTTTTCCTTAAACTGCAGGCACATCTGGCCAAATTGGTGGT
TGTGTTAACTTCGACTGTTTTTCCTTCAAGCCACTCGTTTATCCTAATTTTGGGAAATAGCAAGCTGAGACAAAATGCCA
GAGGACGACTGTGGTATCTTAACTGCCGCCTGAAAAGAGGGAAATCTTTAGCTCCATAG
>PahoTAS2R67B_NW_005812598.1:312373-313311
ATGCCATCTGGAATTGAAAATACTTTTCTAGTAGCAACAATAGGAGGATTTGTGATTGGAATGTTGGGGAATGGGTTCAT
CATACTAGTTAACTGCATTGACCTGGTGAAGAGACAAAAGCTCTCATCAGCTGACTGCATCCTCACAGGCCTGGCTATCT
CCAGAATCAGTCAACTTTCAGCAATACTATGTGACTCATTTTTGTTGGTACTATGGCCACACCTATATGCCATTGATAAA
GTAACAAAGGTTGTTAATAGTTTTTGGATACTGTCCAATCACCTAGCTACCTGGTTTGCCACCTGTTTAAGTGTTTTCTA
CTTCTTTAAAATAGCCAACTTCTCCCACCCCTGCTTCACTTGGCTGCGGTGGCGAATTCGTAGTGTGGTACTGGTGCTTC
TCTTGGGGTCTTTGTCCTTACTGTTTTTGAATTCTGAATCAATATATATGTTTAGTCATATCTCAACTAACAGCTACAAA

ATATATGCAAGAAACTCAACGTGGTCCTCAAATGTAAGTGAAACTCATGATCTTCACCAGTTGATTGTTTTTAACTTCAT
CAGCTTAATCCCCTTTCTTCTGTCCCTGACCTCACTGCTCCTCTTAGTCCTCTCCTTGATGAGACACATCAGGAATTTGC
AGCTCAACCCCAGCTCAAAGGATCTCAGCACAGAGGCCCATAAAAGAGCCATGAAGATGGTGATGTCTTTCCTCTTCCTC
TTCGTCATTCATGTTTCTTCCATCCTATTAACAGGTTGGGTTTTCCTTAAACTGCAGGGACGTCTGGCCAAATTGGTGGT
TGTGTTAACTTCGATTGTTTTTCCTTCAAGCCACTCGTTTATCCTAATTTTGGGAAATAGCAAGCTGAGACAAAATGCCA
TAGGACGACTGTGGTATCTTAACTGCTGCCTGAAAAGAGGGAAATCTTTAGCTTCATAG
>PahoTAS2R372B_NW_005810531.1:1624630-1623704
ATGTCAAATGTCATCAAATATGTTTTTCTGATCATTGAAATCTCAGAATTCATAACAGGAATTTGCGGAAATGGATTCAT
TGCACTAGTACTCTGTGCTGACTCTCTCAAAAGCCAGAATATCTCCTTGCTTGACTTCATCTTTATATGCTTGGCCATCT
CCAGAATTGGTATGTTATGCATACTTCTCCTCGATGGCATTAAAATTGTGTTCCATCCAGAAATATTAGATCGTCACCAG
GTAATAGAAGTAACTTTTGATTTCCTCTGGAATCTGAGCAATTCCTTAGGTACCTGGTGTGCTGCCTGCCTCAGCATCTT
CTACTTCCTCAAGCTATCTAGTTTTTCCCACCCCTTCTTTCTCTGGCTAAAATGGAGAAGAAATAGAGTTGTTTTCACCA
TTATGTTGGGATTCTGTCTCTCTTTGTTTTTGAATCTTCTGAACATAAAATTCAATGCTCTCAGGGTCAGTGACCATTTA
GAAATAGAAAGCAACTTGACTTGGAAAAAATGCATGCGTAAAACACAGTCTTACAGCAGTCAAATTCTCCTCCACCTGGG
ATCTCTCATCCCCTTGGCTGTGTCACTCATTTTATTTTTCCTGTTAATATTTTCCTTATGGAGACATACCAGGCAGATGA
CACGCTATGCCAAAGGATCCAGAGACCTCAACACAGGCGTTCTTGTGAGAACAAGAAATACTTTGGCCTCTTTCATCATT
CTCCTAGCTGTGCGCTATTTGGCTACCTTCATGTTAACCTGGTATTATTTCACACTAGAAAATGACATGACTTTTATTGC
TATTCAATCTGTAGCATTTCTCTATCCTTCAATTCACCCTTTTATTTTGATTCTGAGGAGCAGGAAACTGAGACAGATTT
CTGTGAATCTGCTAAGGCAAATTGAATCCTGTATCAAAGGACTGTAA
>PahoTAS2R8P_NW_005810531.1:1592197-1591269
ATGTTCAGTATAGAAGACCACATCTTTCTGACCATAACGACTGCAGAATTCATCATAGGAATGTTTGTGAATGGATACAT
TGGACTAGTAATATATGTTGATTGGATTAAGAAGAAAAAGATCTCCACAACTGACTACATCCTCTCCAGTTTAGCTCACT
CCAGAATTTGTTTGCTTTGTGTAATGACACTCAATGGCACCATACTGGCACTCTACCCAGGTGTTTATGAAAATGAGAAA
ATAAAGGTAGTTCTTAATATCTTCTGGACATTCACCAACTACTTAAGTATGTGGTTTGCCACCTGCCTCAATGTCTTCTG
TCTCTTCAAGATAGCCAATTTCTTCCACCGACTTTTTCTCTGGCTGAAGTGGAGAATCAAGAGAGTGTTTCACTGGAGCC
TGCTGGGGTCCCTGGCCATTTCCATGTTGATCAGCCTTATACAAGCAACATTAACAAACTCTGATTATGAATTTCTTAAA
ATTGAAAACATAAAAGAAACGTCACCGAATTGTTCCATGTGAGTAAAATTCAATACTTCAACCCACTGACACTGTTTAAC
TTGTTAGCAATTATTCCATTTACTGTGTCATTGATCTCATTTTTCTTTTTAATTACATCCCTATGGAGACACATCAAACA
AGTGAAATCCAGTGTTACAGGCACCACAGACTCCAGCACAGAGGCCCACATGGATGCCATGAAAACAGTGACCTCATTTC
TTTTCTTCCTTTTTGTATACTACCTGGCCTGTCTTTTGGCAACATTTATATACTTTATGAAAGAAAGCAGGTTAGCTATG
ATGTCTAGAGAGATTATAGCAATTCTTTATCCCTTAGGTCACTCACTGTTTTTAATTGTTGGAAATAACAAGCTGAGGCT
GGCAGCTGTCGGGATGCTGAGATGTGGGAAAAGAGTCTGCATGATGTAA
>PahoTAS2R9P_NW_005810531.1:1594061-1593130
ATACCAGGTACAATGGAGGCAATATATATGTTCTTGATTACTGGTGAGTGGATGATAGGAATTTGGGGAAGTGGATTGGT
TGTACTGGTAAACTGCAGTGGCTGGCTCAAAAAGAGAGCTGTCTCCTTGACTGACGTCATCCTGGTCAGCCTGGCCACCT
CCAGAATCTGTTTTTTGTGTGTGATATATATAGATGATTTTATTACGGTACTCTTTCCAGATACATACAGGCATAGTGAG
ATGATGAACATTTTGGATATTTTCTGGACAACTTGCAATCATTCAACTGTCTGGTTTACTTCGTGCCTCAGCATCTTCTA
TTTACTCAAGATAGCCAGTATATCCCACCCAGTTTTCCTCTGGCTGAAGCTGAAGATGAACAGGGTATCCTTGGGATTCT
TCCGATGTCCTTTCTCATCTCCTCAATTGTTAGTATTTTACTGAATAATGATTCATTTTATGACGTCAGAATCAATAATG
AAGCAAACATTATTTAGGAATTCAAAGTAAGTAAAATCCCAACTGCTTTCAAATTGATTATCCTGAACCTGGGGGCTATG
GTTCCCTTTATTCTTTGCCTGGTCTCATTTGTCCTTTTATTTTTCTCACTTTAACACACCAAGCAAATGAAACTTCATGC
CACTGGGTCCAGAGACCCTAGCATAGAGGCCCACATGAGGGTCATAAAGACAATAGTCACCTTTCTGGCTCTTTTCATTA
TGTACAATGCAGTTTTTCTCATTGTAACATCTAGCTTTCTGATTCCTCATGGAAAATTGGAGCTGATGTTTGGTGGCCTA
AGAGCTGCCATTTTCCCATTGAGCCATCCATTCATCCTGCTAATGGGAAACAGAAAGTTGAGGGAGGCTTTTCTGAAGGT

GCTGGGGATTGTGAAGGGTTTCCACAAAAGAAGGAAATATTTTGTTCCCCAG

>PahoTAS2R10CP_NW_005810531.1:1605255-1604502

ATGCTGAGTGTACCGGAAGGCCTCCTCATTTTTGTAGCAGTTAGTGAGTCAGTACTGGGGGTTTTAGGGGACGGATTTAT
TGGACTTGCATACTTCATTGAATGTGTGAAGAACAAGAAGTTTTCTATCAGCTTTATTCTCATGGGCTTAGCTACTTCCA
GAATTTGCCTGATAGGGTTAACAACTACCGATGGATTTGTGAAGATTTTTTTCTCCAGAAATGTATTCCTGTGGTTACCT
AATTCACTGTATTACTTACTCATGGATAATTCTGAATCCATCAAGTGTCTTTTTTTGCCACTAGCTTCAGCATCTTCTAT
TTCCTGAAGATAGCCAATTTTTCCCACCACATTTTTCTCTGGTTGAGGAGTGACATCCAAAGGGTTCTTCTCCTTCTGAT
GGGATACTTGCTTATTTCATGGTTAGTTACGTTTCCACTAACTATGAAGATAATTAGTGATACTAGAGCAAAGAATGGAA
GCGTAATCTTTTCAGCTGAAGTGCATAAAGGGGAATTCTTTAGAAACCAGATTTTGCTCAATCTTGGAACCCTTCCCATC
TTCATACTATGCCTGATTACATGTATCTTATTGCTCATTTCCCTTTGGAGGCACAACCAGAGGATGCTATTGAATACCAC
AGGATTCAGAGACCCCAGCACAGAAGCACATATCAAAGCAATGAAAGTTTTGATATCTTTTATCATCCTTTTTATCTTGA
ATTTTATAAGCATTATCATAGAAATATCATGCAC

>PahoTAS2R10DP_NW_005810531.1:1629947-1629023

ATGCTAAGTATAATGGAAAGTCTTCTCATTTTTGTAGCAATTAGTGAGTCAATATTGGAAATTTTAGGGAATGGATTTAT
TGGATTGGTAAGCTGCATTGACCATATGAAAAACAAGATCTCTACTATCAGCTTTATTCTTGCTGGCTTAGCAACTTCCA
GATTTTACCTGATATGGACAATAGTTACTGATGGATTTTTAAAGTTATTCTCTTCAATGTACATTCCTCTGGGAACCTAG
TTGAGTTTAATGGTTACTTATGGATAGTTATGAATCAATCAACTATCTGGTTTGCCACTTGCCTCAGCATCTTCTATTTC
CTGAAGATATCCAGTTTTTCTCACTGCATCTTTCTCTGGTTGAAGGGTAGACTCAACATGGTTCTTTTCCTTCTTTTGGG
ATGCTTGCTTATTTCATGATTAGTTACTTTTCCACATTTTGTGAAGATTGTTAATGATAATAAAAGGAAAATAGAAACAC
AGTCTGATCAATGGATATGCATAAATGTGAACTCTTTGGAAAACAAATTGGGCTCCATCTTGGTGTCATTCTCCTTTTTA
TACTATGCCTGATTACATGTGTCTTGTTGCTCACTTCTTTTTGGAGACACAACAGGAGGATGCAACTGAATGCCACAGGA
TTCAGAGACCCCAGTACAGAAGCACATATCAAAGCAGTGAAAGTCTTGGTGTCTTTTATCATCCTCTTTATCTTGAATTT
TGTAGGTACTGCCATACAAATATCAAGTGTGACAGTGCCTGAAAACAAACTGCTTTTTATTTTTGGTATGACAACCACAG
TCCTCTATCTCTGGGGTCACTCGCTTATCCTAATTCTAGGAAATAGGAAGCTCAAGCAAGCCTCTTTGAGGGTACTGAAG
CGATTAAAGTGTGGGGAAAAAGAGAAAATTCTCAGAACTCCTTGA

>PahoAST2R11P_NW_005810531.1:1674066-1673172

ATGTTGAATATATTGGAGAAGGTTTTCATGGTTGTGACTGGTGTGGAATTTATAATAGGAATTTTAGGGAATGGATTTAT
TGGACTCACAATTTGCATTGCCTGGATTAGGAATCAGAAGTTGAGCTTGGTTGACTTCATTCTTACTAGTCTGGCCTTTG
CCAGAATCAGTCAATTATGGATAACCACTGTCATGTTTTTTTCAGTGATGTTCTATCAGGCAGGCTTTGGTACTGTGGGA
AGAAAATATATCTTTTTTTGTATCTGGATACTGGCCAGTCACTCAAGCACGTGGCTTGCTACTTGCCTTGCTGTCTTTTA
TTTCCTGAAGATCGCCAGTTTCTCCCATCCTCTTTTCCTTTGGCTAAAATGTTAATTAACAAGGTTGTTTTTATGCTTCC
ACTGGTATCTGTGCCCTTCCGAGTCATAAGTTTTCCTTGGCCGTATAATGTTGATGTCTTCTGGTGTCATGTCCAAAAGA
TGCATGAGAGAAATATGACTGAATTATGCAATGTGAATGAATATCAAAATTTAAATTTTATGATTATCTACACTGTGGAT
TCCCTCCCCCACTCCTTCCTTTCCCTGATTTCCTTTCTCCTGTTGCTCCATTCTTTGTGGAAACACAAGGAGAACATTGC
ACACACTGTCAGGGACTCCAGAGACCCCCGTGTGGAGGCCCATTTCAGAGCCATGAAGACTGTGTTTTCCTTTCTCATGC
TCTTTGTCCTGTAGCAATTTGGCCTTTTCATGACATTTGGGGGACATTTTTTCCTACAGAACAAGCTGGTTGTGATGTTT
GGTTATATGTTAGAAATGCTGTATCCTTCAAGTCATTCATATGTTTTAATTTTTGGAAACAGCCAAATGAGGAGATTCTT
CTTGGTGATTCTTAG

>PahoAST2R12P_NW_005810531.1:1686856-1685928

ATGGAGAGAACACTGAACAATATACTTACGATCATTTCTGCTGGAGAATTCTTACTGGGTATTTTGGGAAATGGATTCAT
TGTTCTGGTTAACTGTATTGATTGGATCAGGAGCAGGAAGTTCTCCCTGATTGACTTTATTCTCACCTGCTTGGCTATTT
CCAGAATATTTGTGCTGTGCATAATGATTTCAAGTACAGGTTTATATGTAATCTCTGAGGAAATACAGTACAACAAGAAT
CTCCTGATAAATTTGGGGTTCCTCTGGACAGGATCCAATTATTTCTCCATAGCCTGCACCACCTGCATCAGTGTCTTCTA
TCTCCTCAGAATAGCTAACTTTTCTAATTTCCTTTTCCTCTGGATGAAACGGAGAATTCACAAGGTGCTTCTCATTATTG

CACTGGGGGCTGTCTTCTCTTTCTGCTTGTGCCTTCTTCAGAAGAATATGGCAGTTGAAATCCTGTTCCCAAACCAGGTA
AACAGCAAAAAAATGTGACATTGGACTTTCTAATGATAAAATACGATTTGTTCCTTACCATAATGTTCCTCATCCCCTTT
GTAGTGTCCCTGGCCTCCTTTCTCCTTTCAATCCTCTCCTTATGTGGTCATCTCAGGCGTATGAAGGGTGTAGACTGTAG
CTCGGAAGCCCACGCGAGAGCCCTGAAGGCCATGATTTCATTCCTACTCCTCTTCATTCTATACTGTTTGAGCAATATTA
TGACAATGTGGGCCAATCACATTCTAGGTAGTTTTGTGGCAAAGATTTTTGTGAACATGCTGTTATTTTTCTGTCCTTCT
GGCCACCCTTGGCTTCTGATTTTGTGGAACAGCAAATTGAAAAAGGCTTCATTCTGTGTCCTAAGGAAGCTGAGGGGTTA
CATGAATCTAAGAAAACCTACTCTTCCAAAAATAAGCCTGAAGCAATGA
>PahoTAS2R13P_NW_005810531.1:1702135-1701225
ATGGCAGATTCCTTGGAAAACATCTTTATCATTTTAATAAATTTAGCATTCATAATTGGTATTCTAGGGAATGGATTCAT
AACACTGGTGAACTGCATTGACTGGATCAAGATGCAAAAGGTCTCCTTGGCTGATTGAATCCTCACTGCTTTGGCAATTT
CCAGAATTGGTCTGATTTTGGTAATGATGGTGAGTTGGTTTACAAAGGAGTCTTATCCATTTTCTTCTTTAGACATAAAG
GGAAATAAAGTCATACTTTTTAGTATTGCTGGGCTCTTGGCCAATTATTTTAGTGTCTGGCTTGCCACAGGCCTCAGCCT
CTTTTATATCCTCAAGATAGTCAATTTTTCAAATGCTGTTTTTCTTCACCTAAAGTTTAGAATTGGAATGGTATTTATGG
TAATGTTTCTGGGGACATTAGTATTGCTGCCTTTAAGTCTTACTCTGGTGAGCGTCTATATTAATATCAAGATACATCCA
TATAAAGAAATATGACTTTAAATTCTAAAAGAAGTGACACTGAAACCTTTTCCAAATTGATTATATTCACCATGGGATCT
TTCTTACCCTTTATTTTATCCCTGAGTTGTTTTCCCCTGTTAATGTTCTCCCTACTGAATCAAGTCTAGAAGATGAGGAG
CCATGCAACAGGGTTCAGAGATCCCAGCAGCAAAGCCCATGTCAGAGCCATGATGATGGTGATGTCTTTTCTCACACTAC
TTGCCATTCACTTCCTATCTCATCTCATGACAACTTTTCATCACAATGTGATGCAGAGTGAACTGGCCTTTATGCTTGCT
GAAGCTCTTGGAACTATTTATCCTTCAGTCCACTCATTTGTCTTGATTCTGGGAAATGACAAGCTAAGAAAAGCTTCACT
TTTGGTGCTGTGGCAGTTGAGGTGTGGCTGA
>PahoTAS2R38P_NW_005816769.1:73668-72673
ATGGTGACTCTGACTCACATCGCATCTGTGCCCTCTGAAGTCAGGAACGCAATTCTGTTCTTTTCAGTCCTGGAGTTTGC
AGTAGGGATCCTGGTCAACACCTTCATTTTCTTGGTGAATTTTCAGGACCTGGTGAAGAGGCAGCCACTGAGCCGCTGTG
TTCTTGTCCTGTTGAGTCTCAGCCTCACCCGGCTTGTCCTGCACGGGCTGCTCTTTCTGAAGGCCATCCAGCTTACTCAT
TTCCAGAGAATAAAAGATCCACTGAGCTTCAGCTACGAGACCATCATCGTACTCTGGATGATAGTCCACCAAGCCGGCCT
CTGGCTCACCACGTGCCTTAGTCTCCTTTACCTCCAAGATTGTCCGTTTCTCTCACGCCTTCCTGCTCCGTGTAGCAAGC
TGGATCTCCAGAAAGATCCCCCAGATGCTTCTGGGTGCTGTGGTTCTCTCCTGTGTCTGCACTCTTCTCTGCTTATGGGA
CATTTTTAGTGGATCTCATTTCTCAGCTGTAAATAGGCTACTCACGAATAACAGTACTGAACCCAATTTGAACATTGCAA
AACTCAGTTTCTTTCATTCCTTCCTCTTCTGCTGCCTGGCGTCCATCCCTTCTTTCTTGCTTTTCCTGGTTTCCTCTGGG
ATGCTGGTGTTCTCCCTGGGAGGCACATGAGGGCTAAAACCAGAGGCTCTTGGGACCCCAGCCTGGAGGCTCACACCCGG
GCGCTCAGGTCTCTCGTCTCTTTCTTCTGCCTGTATGTGCTGTCACTCTCTGCTGCCTTAGTCTCGATGCCGTTGCTGAC
CCTGTGGCACAGCAAGGTTGGGGTGATGGTCTGCATAGGGATAATGGCAGCCTGTCCCTCGAGACATGCAGTCATCCTGA
TCTCAGGGAATGCCAAGCTGAGGAGGGCTGTAGACACCATTCTGCTTTGGGCAAAGAGCAGCTTCAGGGTAAGGGTGCAC
CACAAGGCAGATCCTAGGACACCAGATCTGTGTTGA
>PahoTAS2R40P_NW_005816769.1:1364777-1365713
ATGGTGACAGTGAACACGGATGCGATGGATAAAGACACGACCAGGTTCAAGATCATCTTCACCTTGGTGGTCTCTGCAAT
AGAGTGCATCACTGGCATCGTGGGGAACGGCTTCATCACTGTCATCCACTGAGCCAAGTGGGTCAGAGGCAAAAGACTCC
CCATTGGTGACTGCATTCTGCTCATGCTGAACTTTTCCAGGCTCTTGCTACAGATCTGGATGATGCTGGAAAACACGTAC
AGTCTACTGTTCTGGGTCATCTACAATGAGAAAAGAGTATACATACTTTACAAAATCATCATCATGTTTCTGAATTACTC
CAACCTCTGGCTTGCTGCCTGGCTCAATATCTTCTATTGTCTCAGAATCGCAAGCTTTACTCACCCGTAGTTCTCCGTGA
TGAAGAGGAAGGTCATAGGGCTGATGCCTGGGCTTGTGAGGCTATCCTTGTTCTTCTCCTTTTGCTCCAGCTTCCCCTTC
TCTAGAGGTATCTTCAATGTGTACATGAACAATTCCGTCCCCGTCCCCTCTTCCAACTCCACTGAGAAGGTGTACTTCTC
CGAGACAAACCCTTTACCTGGGGATCTTCATCCCTCTGATCATGTTTATGCTGGCGGCCACCCTGCTGATCATCTCTCTC
AAAAGACACACCTTCCACATGAAAAGCAACACCACTGGCTCCAGGGACCCCAGCATGGAGGCTCACCTGGAGGCCATCAA

AGCCATCAGCTATTTTCTCATCTTCTACATTCTCAATGCAGTTGCTCTGTTTCTTTCCATATCCAACATCTTTGCCGCCA
ACAGCTCCTGGAATATTTTGTGCAAAATCATTATGGCTGCCTACCCTGCTGGCCACTCAGTGCTACTGATCTTAGGCAAC
CCTGGGCTGAAAAGGGCATGGAAGCAGTTTCAGCACCAAGTTCATCTCTACCTGTAA
>PahoTAS2R41P_NW_005810862.1:4383368-4382474
ATGCACCCAGCATTCACAGTCCTCTTCATGCTGCTCTTTGTCCTGCTGTGTATCCTGGGCCTCCTGGCCAATGGCTTCAT
TGTGCTGGTGCTGAGCAGAGAGTGGGTGTGACGTGGGAGGCTGCTCCCCTCTGACCTGATCCTCTTTAGCTTGGGACTCT
CCCGCTTCTGCCTGCAGTGGGTTGGAATGGGGAATAACCTCTACTATTTCCTGCATCTGGTCGACTACTGCAGCGGTCCT
GCCCGGCAGTTCTTCGGTCTACCCTGGGTCTTCCTCAACTCCGTCACTTCCTGGTTTGGCTCCTGGCTCAGCGCCCTCTT
CTGCATGAAGATTGCTAACTTCACCCACCCCCTCTTCCTCTGGCTAAAGTGGAGGTTCCCCAGGTGGGTGCCCTGGCTTT
TGCTGGGCTCTCTCCTCACCTCCTTCCTTGTCACCCTGCTGTTTTTTTCAGGGAACCACGCTTTGCATACAGGGTCCTTC
ACTAGAAAACCTTTCAGGAACATGACCTATCATCAGTGGAGCAGGATTCTGGAAATGTACTATTTCCTGCCCCTGAAACT
GATCACTCTTTCAGTTCCTGGCTCTGTTTTTCTGGTCTCGATTGCTCTGTTGATTCACTCTCTGAGGAGACATGCACGGA
GGACGCAGCACAGTGCTCACAGCCTGCAGGATCCCAGTGGCCAGGCTCACACCAGAGCTCTGAAGTCACTAGTCTCCTCC
CTTGTTCTTTATACTCTGTCTTTCGTGACCCTGATCATTGATGGGGCAGGGTTCTGCTCCTTAGAGAGTGACTGGTACTG
GCCATGGCAAATTTTAGTCTACTCGTGCACATCCATCCATCCCTTTATCCTCATCCTTGGCAACCTCAGGCTTCGAGGGG
CATTTGGGCAGCTGA
>PahoTAS2R42P_NW_005812598.1:300712-301642
ATGTTCCCTGGGTTGAGTACAGTCTTTCTGATACTGTCAGGAGTGGAATTCTTAATCGGAATTCTAGGCAATGTGTTCAT
TGGACTGGTACTCTGCTCTGAATGTGTTAAGAACCAAAAGACATCTTTATTTGACTTCATCCTCACTGGCTTGGTTATCT
CCAGAATCAGTCAACTGTTGGTGTTTTTTGTGGAATCACTTATAATGGGACTAGAACCACAGGGATTTGCCATTTTTAAA
CTAGCAAAGCCCATTGCTTTACTTTGGAGAATATCTAATCATTTGACTGCCTGGCTTGTCACCTGCCTAAGTATTTTCTA
TCTCCTTAAGACAGCTCATTTCTCCCACTCTCTTTTTTTCTGGCTGAAGTGGAGAATGAACAGCGTCATTCTTATGATGC
TTGCATTTTCTTTGGTCTTTATGATTTTGGACATTCTTTTGCTAGAAACATTTAATGATCTCTTCTGGAATTTAATAAAT
GAAGGCAATTTGACTTTAGTTGAAAGTAAAACTCATTATATTAAAAGCGAGAGTCTTCTTAGTTTCTCCTATTTCATTCC
TATTGTTCTGTCCCTGCTCTCATTGTTTTTTTTTATTTTGGTCCTTGGTGAAACATACCAGAAATTTGCAGCTCAGTTTT
ATGGGTTCCAGGGACTTCAGCACAAAGGCCCATAAAAGAGCCATGAAAATAGTGACGTCATTCTTCCTCCTTATCATGGT
TCATTTTCTTTTTACACAGTTGGCAAATTGGATGTTTCATAAGTTTTTGGACAATAAATTCACAAAGTTCATCATGTTAG
TACTATATGCCTTTCCTTCAGGTCACTCATTCATGTTGATTCTGGGAAACAACCAGTTAAGACAGATAGCCTTGAAGGTA
CTGAAGCATCTTAAAAGCTCCTTGAAAAGACAAAATCCATTGGCTTTATAG
>PahoTAS2R408BP_NW_005810531.1:1729117-1728184
ATGATAATATTTATGTCAAACATTGTTTCCATTCTATTAATGACAGAATTTGTTCTGGGAAATTTTGCCAATGGCCTCAT
AGCACTGGTGAACTGCAATGACTGGATCAAGAGACCAAAGATCTCAGCTGATGGGATTCTCACTACTCTGGCATTCTGCA
GAACTGTTATGCTCTGGGCAATGTTAATAAATTGGTATGTAATTGTGCATAATCCAACTCTATATAATTCAAAAGTAAAA
ATTATTGTTCATGTTGCCTGGACAGTAAGCAACCATTTTAGTAACTGGCTTGCTTCTAGCCTCAGTATATTTTATTTGTT
GAAGATAGCCATTTCTCCAGCCTAATTTTTCTTCACCTGAAGCGGAGAGTTAAAAGTGTAGTTCTCATGATGATGTTGGG
GACGTCATTAATCTTGTTTTTTCAAGTTGCAGTGTTAAGCATAGGTGAGGCTATTCAGACAAATGAATATGAAGGAAATG
CCACTCAGAAGACCAAACTAAGGGACATTTCACACCTTTCAAATGTGACTCTGTTCACGCTAACAAACTTCATACCCTTC
ACTATGTCCCTGACATCTTTTCTGCTGCTAATCTTTTCCCTGTGGAAACATCTCAGGAAGATGCAGCTCAATGGTAAATG
ATCCCAAGATCCCAGCATCAAGGTAAACATAAAATCCATGCAAACGGTCATCTCCTTTCTTTTCCTGTTTGCTGTTTACA
TTCTGGCTCTAATTTTATCAGTTTGGAATTCTAATGAGCTTCAGAAAGATCAAATGCTTTCTGATGTTCTTTTAATCATG
TATCCTTCAATCCACTCATGTATCTTGATCTGGGGAAACAGGAAATTAACTCAAGCCTTTCTGTCATTTCTGTGTCAGTC
AAGATGCTGGCTGAAGGAAAGGAAATAGGTGGAAACATATGTCTTTTAGCATAA
>PahoTAS2R408CP_NW_005810531.1:1759288-1758096
ATGATAACTTTACTATTGATCATTTTTTCCATCCTAGTATTAACAGAGTTTGTTCCAGGAAATTTTTCCAGTGGCCTCAT

AGCACGGGTGAATGGCATTGATTGGGTCAAGAGACAAAAGATCTCCTCAGCTGATGGGATTCTCACTGCTCTGGCAGTCT
GCAGAATTGTTTTGCTCTGGGTAATGTTAATGAATTGCTACTTAGTTGTGTTGAATCCAGTTCTATATAGTTTAAAAGTG
AGAATTATTGTTCATATTGCCTGGATAGTACACAACCATTATAGCACCTGGCTTGCTACTAGTCTCAGTATATTTTTTTT
TGTTGAAGATAGCCATTTCTCCAGCCTAATTTTTCTTCACCTGAAGTGGCAAGTTAAGTGTCATGCACATAATACTTCTG
GGAACTTCATTCTTCTTGGTTTTTCATGTTGCAGTGATATAGACAGTGAGGCTATCCAGACAAATGAATACGAAGGAAAC
ATTCCTCAGAAGACCACATTGAGGGGCAGTTTATGGCTTCCACATGTGACTCTGCTTATGCTGCTGCTGCTGCTGCTAAG
TCACTTCAGTCATGTCTGACTCTGTGTGACCCCATAGACAGCAGCCCACCAGGCTCCCCCATCCCTGGGATTCTCCAGGC
AAGAACACTGGAGTGGGTTACCATTTCCTTCTCCAATGCATGAAAGTGAAAAGTGAAAGTGAAGTCTCAGTCATATCTGA
CTCTTCGCAACCCCATGGACTGCAGCCTACTAGGCTCCTCCATCCATGGGATTTTCCAGGCAAGAGTACTGGAGTGGGTT
GCCATTGCCTTCTCCGCTGCTTATGCTAGGAAATCTCATACTATGTCCCTGATATGTTTTCTGCTATTAATTGTTTCCCT
GTGGAAACATCTCAAGAAGATGCAGCTCAGTGGTAAAGGATCTCCAGATACTAGCACCAAAGTCCATATAAAAGCCATGC
AAACTGTGATATCCTCTCTCTTGCTGTTTGCCATTCATTTCCTGGCTCTAATGGGATCCATTCGGAGTTTTAAAAAGCAG
CAGAAGGAACCTGTCTTTTTGTTCTTTGAGGCTCTTGGATTCCTCTGTCCTTCAAATGACTCATGTACCCTGTTTTGGGG
AAACAGGAAGTTAACAAAAGCATTTCTGTCATTTCTTTGGCAGCTAAGGTGCTGACTGAGAGAAAAGAAATAG
>PahoTAS2R408DP_NW_005810531.1:1750599-1749683
ATGATAACTCTACTATCAACCATTTTTTCCATCCTAGTAATAATACAATTTGTTCTGGGAAATTTTGCCAATGGCTTTTT
AGCCCTGGTGAACTGCGTTGACTGGGTTAAGAGACAAAAGAGCTCCTCAACTGATGTGATTGTCACTGCTATGGCAGTCT
CCAGAATTGTTTTGCTCTGTGTAATGTTAATACATTGGTATTATATTTTGCTTCATCCAGCTTTATATGGTTTTAAAGTA
AGAACTATTGTTCATGTTGCCTGGACAATAAGCAATCATTATAGCATCTGGCTTGCTACTAGCCTCAGTTTATTTTATTT
GTTGAAGATAGCCAATTTCTCCAGCCTAACTTTTCTTCACCTGAAGTTGAGAGTTAAAAGTGTAATTCTCGTGATGCTTC
TGGAAACTTCATTCATTTTGGTTTTACAAGTTGTAGTTATAAGTGTAAGTGGGACTATGCAGAGAAGTGAATTTGAAAGA
AACTTCACACAGAAGACCAAACTGAGGGATATTTTATGGCTTTCACATGTGACCTTGCTCATTCTAGGAAACCTCACACC
TTTTACTATGTCCTTAATATCTTTTCTGCTACCAATCTTTTCCCTGTGGAAACATCTCAGGAAGATGCAGCTCAATGGCA
AAGGATCCCAAGTTCCCAGGACCAAAATCCATATAAAAGCCATGCAAACTGTCATCTCCTTTCTCTTGCTATTTGCCTTT
TACTTTCTGGTTCTAATCATATAAACTGGAGTCCTAAAAAGCTGCACGAGGAACCGTTTCTCTTGCTTTTCCCAACAGTT
GAAGTCATCTATCCTTCAGTCCACTCATTTATCCCTATTTGGGGAAACAGAAAGTTAACACAGGCCTTTCTGTTGTTTCT
GAGGCAGCTGGGGTGCTGGCTGAAAGACAGGAAATAG
>PahoTAS2R408EP_NW_005810531.1:1711924-1711046
ATGATAACTTTACTACATTTTTTCCATCCTAGCAATAGAATTTATTCTAAGAAATTTTGCCAGTGGTTTCATGTCACTGG
TGAACTGCATTGACTGGTCAAGAGACAAAAAAATCTCTTCAGCAGATGGGATTCTCACTGCTCTGGCAGTCTCCAGAATT
GGTCTTCTCTGGGTAACATTAATAAATTGATATGTAAATGTGTTTAATCCCAGCTTTAGACAATTTAAGAGCAAGAATTA
TTATTATTGCCTGGATAATAAGCAACTATTTTGGCATCTGGCTTGCTGCTATCCTCAGCATATTTTATTTGCTCAAGATA
GCCAATTCTCCAATATTATTTTTCTTTACCTAAAATGGAAAATTAAAAATATTCTTCTTGTTCACTTTGTCCTGTTTGGC
TTTATTAATTCATGGTGTAAACATAAATAAGACTATCCAGGCAAAAGACTATGAAGGAAACATCACTCAGAAGACCAAGC
AGAGGGACACTTTACACCTTTGAAATATGACTCTGTTCATGCTAGTAAACTTCAAACCCTTTGCAGTATCCCTGATGTCT
TTTCTGCTGTTAATCTTTTCCCCAGGGAAACATTTCAGGAAGATGCAGCTCAGTGGTAAAGGACCCCAAGATCCCAGCAC
CAAGGTCCATATAAAAGCCATGCAAACTGTCTTCTATTTTCTATTTGCCCTTTACTTCCTGGTTCTAATTTTCTGTTTGG
AGTTCTAATAGGCAGTGGAACAACTTGGTTATCATGGCATGCTGGGCTTTTGGAATCATATGTCTTTCCCTTATCCTGAC
ATGGGGAAAACAGATACTAAGACAGTCCTTCCTGTCATTTCTGCAGCAACTGAGTTGATGGCTAAAAGAAAGGAAATAA
>PahoTAS2R62P_NW_005816769.1:1570637-1571563
ATGTCCCCTTTGCCCACATTGATCTTCAAGGTCATCTTTTTCCTGGAGTCATTGGTTGCCATGCTGCAGAATGGCTTCAT
AGTTACCATGATGAGCAGGGAGTGGGCGTGAAGCCGGACTCTGCCCGCCAGTGACATGATTGTGGCCTGCCTGGCTGCCT
CCCGGTTCTGTCTGCATAGGATGGACCTCCTGAACAACCTCATGGCCTCCTCTGGCTTTTGTTCCAAAATCTACTATTTC
AGCATCCCCTGGGATTTCATCAACTCCCTCAGTTTCTGGCTGACTGCCTGGCTTGCTATCTTCTACTGCACGAAGATCTC

CCTCTTCTCTCACCTCGTCTTCTTCTGGATAAAGTGGAGGATTTCTCGATCGGTTCCCCAGCTGCTGCTGGGCTCCTTGA
TCTTATCTGGTCTGACTGTCGTCTCCTCAGCTGCTGGGAATACAATTCTTGCCCAGATGACGGCTGCCCAGAGTTCCCAT
GGAAACACCCTGGCTGGTAGAATACACGCTGTCTATTTGCACTGTTTTCTACCTCATGTAATTCTCATGAGGTTGGTTCC
ATTCCTCCTGTTCCTGGTGTCCACCTTCTCACTCATGGTTTCGCTGTGCCGGCACCTCGGGCAGATACAGGACCGCAGAC
CCAGCCCACGTGATCCCAGTACCCGGGCTCACACCATGGCCCTGAAGTCACTTGCCTTCTTCCTCATCTTCTACACCTTG
CACTTCCTGTCCCTGGTTATCATTGTGTACATCCCAGCCTTCCGGAAACACTGGCACTGGGCCTGTGAGGTGGTGACCTA
TGCAGGCATCTGTCTGCACTCCAGCATCTTGATGCACAGCAGCCCCAAGCTGAGAAAGGCCCTGAAGAAGAGGCTTTGGC
GATCCCTGGACAAGGACCAGTTTGTCTCCAGTTATCAGTATCAATAG
>PahoTAS2R18P_NW_005810531.1:1724905-1724268
ATATCAGTTGGAACAAAGGTCTTCTTTCTAGCGGTGTCAACAGGAGAATTGATCTTAGGAGTACTGGGAAATGGGTTCAT
TGGACTGGTAAACTGCATCGAGTGGGTCAAGAATGGAAAGTTCTCATCAGCTGAGTTCATCCTTACTTGCTTGGCTGTGG
CCAGAATCATTCAGCTGTGGGTAACTCTTTTGGATTCACTTATAGTAGGATTAGCTCCACATCTGTATGCCACTGGTAAC
TAGTAAAAGTAGTTATTCTTCTTTGGGCACTAATGAATCACTTAACTACCTGGTTTGCCACCTGCCTAAGCATATTCTAC
TTCCTTAAGACAGCCAATTTCTTTCACTTCTGTTTCATGCGGCTGAAATGGAGAATGAACTGAGTGCTTCTTGTGCTTTT
CCTGGCCTCTTTCTTCTTATTGTCTTTTGACCTCTCAATGCAAGATGCTCTTGGTGAGTTGTGGATGAACACCTTTAGAG
AACCTGAAAGGAACATGACTTTGCATTTAGATGCAAGTAAAATTTTCTATTACTGATTCTTCTCAGCTTGACATATGTTA
TCCCTTTCCTTCTCTTCATGGCTTCTTTGCTGCCTTTCTTTCTTTCCCTGGTGAGACACATCAAGAATTTCCAAGCCA
>PahoTAS2R372AP_NW_005810531.1:1641411-1640967
CAGCAGCAGGTAATAAGAGTTTTTGAGTTCTCCTGGAATCTGAGAAACTCCTTAAGTACTGGATGTGCTGCCTGCCTCAG
CGTCTTCTACTTCCTCAAACTATCTAGTTTTTCTCACCCCTTCTTTCTCTGGCTAAAGTGCAGAAGAGATAGAGTTTTTT
TCACCATTATGTTGCAATTCTGTCTCTCTTTGATTTTTAATCTTCTGAGCATAAAATTGATACTTTTGTGTTCAGCAAGC
ATTTAGAAAAGGAAAGACCTACCTTGGAAAAAAGATCTGCATAAAAATCAGTATCATAGCAGTCAAGTTCTTTTCAGCCT
TGGATCTCTCATTCCCTGGTCTGTATCACTCATTATATTTTTCCTGTTACTCTTTTCCTTATGGGGCCATACCAAGCAGA
TGACACGCCATAACGCAGACCCCCGGGACTTCAGCACAGGGGTTC
>PahoTAS2R372CP_NW_005810531.1:1658168-1657247
ATGTCAAGTGTAATCAAAAAAGTTTTTATAATCATTGAAATCTTAGAATTCATAACAGGAATTTGCAGAAAAGAATTCAT
TGCACTAGTACTCTGTGCTGACTCTCTCAAAAGCAAGAATATCTCCTTGTTTGACTTGATCTTAACATGGTTGGCCATCT
CCAGAACTGGCATGATATTCATAATTCTTGGATGGTGTTAGAATAGTGATCTATCCAGGAATATTTGAAAGTCATCAGGT
AATAGAAGTAATTTTTTATTTCTTCTGGAATCTGAGCAACTCCTTAGGTACCTGGTGTGCTGTCTGCCTCAGCATCTTCT
ACTTCCTCAAGCCATCTAATTTTTCCCACCCTTTCTTTCTCTGGCTGAAGTGCAGAAGAAATAGAGTTGTTTTTACCATT
CTGCTGGGATTCTGTCTTTCTTTGATTTTAATTTTCTGAGCATAAGTTTTCATACATTTGGGGTCAGTGACCATTTAGAA
ATAGAAAACAACTTGCATTGGAAAAAATATGCATAAAATCCAGTCCTATAGCAGTCAAATCCTCCTCCACCTAGGATCTC
TCATCCCCTTGGCTGTGTCACTCATTTTATTTTTCTTGTTAATCTTTTCCTAATGGAAACATACCAGGCAGATGACAAAT
CATGCCAGAGGATCCAGAGATTTCAACACAGGGATTCTTGTGAGAGCCAGAAATACTTTAACTTCTTTCATCATTTTCTT
AGTTGTGCATTATATGGCTACATTCTTGTTAACCTGGTCTTGTTTCGCACTAGAAAATGAAATGACTTTTATTGTTATCA
AGTCTGCAGCATTTCTCTATCCTTCAATTCACCCTTTTATTTTGATTCTAGGAAACGGAAAATGGAGAGAGACTTCTGTG
AATCTGCTAAGGAAAATTGAGTCTTGCATCAAGAGAATGTAA
>CadrTAS2R1_KN271691.1:855741-854854
ATGCTGGAGTCACAGCTCATTAGCCATCTTCTCTTGACAGCGATACAGCTTCTCACTGGGCTTTTTGTAAATGCCATCAT
TGTGGTTGTGCACGGCACGGGCTTGCTCAAGCACAGAAAGATGATTCCGTTGGAGGCCCTTGTTTCCTGCCTGGCGATGT
CCAGGATCTGTCTAGAGCTGGCCATCTTCTACCTTAATCTGGCTGTTTTTAACTTGATTGACATCCGTCAGTTTGTGGGG
AAGTTTGTAATTCTCTCCTTTGTAAATGAATTGGGGCTGTGGTTTGCCACATGGCTCAGCGTCTTCTACTGCATCAAGAT
TGCCAGGATCGCTCACCCTCTCTTCCTCTGGTTGAAGCTGAAGATCTCCAAGCTGGTTCCTTGGCTGATTCTGGGGTCCC
TCCTACATGCATCTAGCACCTGTGTCTTCCACAGCAAACAGCCATGGATCTTTTCCGAAGACCTCCTGGGCTTTTCCTCT

CAAAATGCAACAGCTGAAAGCGAAGACATCGCTACTTTGCGGTTCACCCTTCTCTTCGCTGAGCTCTCCTTGCCATCACT
CATCTTCCTCGTTTCTGCCCTGCTCTTGCTGTTCTCCCTGGGGAGACACACCTGGCAGATGAGAAACACAGCCACAGGCC
CTTGCCCCCGTGCGCGCGTCAGGTCCCTCCTGTCCATCTTCGCCTTCCTGGTCCTCTACCTCTGCCACTACCTGATGGTT
CTTTTGCTCTTTTCTCAAGTTTTCAAACTTACAAGCCTCAGATTTCTGTTCTGCATCTCGCTGGTTGGTTCCTGCCACTC
CGGACACTCTATTATCTTAATTTTAGGAAATCCTAAACTGAAACAAAATGCGGAGAAGTTTCTCCTGCATAGAAAGTGCC
GTCAGTGA
>CadrTAS2R3_KN271672.1:559490-560440
ATGCTGGGATGCACCGAGTGGGGCTTTCTGGTTCTGACTGCCAGTGAGTTCGTCCTGGGAATGCTGGGCAATGGCTTCAT
AGGGCTAGTCAATGGCAGCAGCTGGTTCAAGAGCAAGAGAATCTCTTTGTCCGACTTCATCATCACTAACCTGGCTCTCT
CTAGGACTGTTCTGCTGGGGACTCTCTTGGTTGATGGTGTTTTAATGGTGTTCTCGTCCAAACTACATGATGAAGGGATA
GTAATGCAGATGATTGATATTTTCTGGACGTTTACAAACCATCTGAGCATTTGGCTTTCCACGTGTCTCAGTGTCTTCTA
CTGCCTGAAAGTCGCCAGTTTCTCCCACCCTGCATTCCTCTGGCTCAAGTGGAGAGTTTCCAGGGTGGTCGTGTGGATGC
TGATGAGTACCCTGCTCCTAGCATGTGGCAGCACCGTGTCTCTGGTCCATGAATTTAAGATCTATTCTGTTCTCAGTGGA
ATTGATGGAACAGGAAATATGACTGAGCAGGTTTTAAAGAAGAGAAACGAATACAAACTGATCCATGTTTTGGGGACTCT
GTGGAACCTCCTTCCCCTAACTGTGTCTCTAGCCTCCTACTTTCTGCTCATCTGCTCCCTGGGAAGGCATACACGGCAGA
TGCAGCGGAACTGTACCGGCTCCAGGGATCCAAGTACCGAGGCCCACTATAGGGCCATCAAAGTCATCCTCTCCTTCCTC
TTTCTCTTCCTACTTTACTTTCTTTCCTTTTTAATTTTATCATCTAGTTATTTCCTACCAGCAACTAAAATGATTAAGAT
GATTGGAGAAGTGATTACAATGTTATATCCTGCTGGCCACTCATATATTCTCATTCTGGGAAACAGCAAGCTGAAGCAGA
CGTTTATGGAGATGCTCTGGTGTGGGCATGGTGATCTGAAGCCTGGATCCAAGGGCTCTTTTTCTCCATAG
>CadrTAS2R7_KN271481.1:403684-402749
ATGCCAGGTAAGGTGGAGAACACCTTAATCCTCATAGCAGTAGGAGAATTTTCACTGGGGATCTTAGGGAACGTGTTCAT
TGGATTGGTAAACTTTGTGGACTGGATCAAGCATAGGAAGATCGCCTCCATTGATTTAATCCTCACAAGTCTGGCCATAT
CCAGAATTTCTCTATTGTGTATAATACTATTAGACTGTTTTATACTGGTTCAGTATCCAGACGTCTATACTGCCGGTAAA
CAAATGAGAATCATTGACTGCTTCTGGACACTAACCAACCATTTAAGTGTCTGGTTTGCCACCTGCCTGAGCATCTTCTA
TTTCCTCAAGATAGCTAATTTCTTCCATCCCCTTTTCCTCTGGATGAAGCTGAGAATTGACAATGCGATTCCTAAGATCC
TGCTGGGGTGCTTGGCCCTCTCTGTGTTTTTTAGCCTTCCTGTCTCTGGGAATTTGAATGATGATTTCAGGTTTTGTGTC
AAGGCAAAGTTGAAAACAAACTTAACTTTGAGATGCAAAATAAATAAAGCGCAATATGCTTCCACCAAGATATATCTCAA
CCTGCTAACACTAGTCCCCTTTTCTGTGTCCCTGATCTCGTTTCTCCTCTTGATTCTCTCCCTGTGGAGACACACCCGGC
GAATGCAGCTCAATGCCACAGGGTCCAGAGACCCCAGCATAGAAGCCCATGTGGGAGCCATGAAGGCGGTCATCTCCTTC
CTCCTCCTTTTCATTGCCTACTACTTGGCCTATCTTGTAGCCACCTCCAGCTACTTTCTACCAGAGACTGAATTAGCTGT
GATGGTTGGTGAGGTGACAGCTCTAATCTGTCCCTCAAGCCATTCACTTATCCTAATTCTGGAGAACAGTAAATTAAGAA
AAGCATTTCTAAGGGTTCTATGGAAAGTAAAGTATGTCCTAAAAAGGAGTTGCTAACAACATAAACAGATCTGA
>CadrTAS2R10_KN271481.1:412683-411754
ATGCTAAGTGTAGGAGAAGGCCTCCTTCTTTTTGTAGCAGTTAGTGAGTCAGTACTGGGGGTTTTAGGGAACGGATTTAT
TGGACTTGTAAACTGCACTGACTGTGTGAGAAACAAGAAATTCTCTATGATCAGCTTTATTCTCACTGGCTTAGCTACTT
CGAGAATTGGTCTGTTATGGTTAATAATTACAGACGGATTTGTAAGGATATTCTTTCCAGAAATGTATTCCTCCGGTAAC
CTGGTTGACTATATTAGTTACTCATGGATAATTCTGAATCAATTAAGTGTCTGTCTTGCCACCAGCCTCAGTGTCTTCTA
TTTCCTGAAGATAGCAAAATTTTCCCACCACATTTTTCTCTGGTTGAAGAGAAGACTCAACAGGGTTCTTCTCATTCCGA
TGGGATTATTGCTAATTTCATGGTTATTTACTTTTCCACAGATGGTGAGGATTATTAGTGATAATAGAATAAGAAATGGA
AGTACAACTGGGGTAGCCAACATGCACAAAGATAAATTCCTTACATACCAGATTTCTCTCAATCTGGGGACCATTCTCCC
CTTTCTACTCTGCCTGATTACATGTTTCCTATTGATCATTTCCCTTTGGAGGCACAACAGGAAGATGAAATTGAATGCCA
CAGGATTCAGGGACCCCAGCACAGAAGCACATATCAAAGCAATGAAAGTCTTGATATCTTTTGTCATCTTGTTTTTCTTA
TATTTTGTAGGCGTTGCTATAGAAACATATCACTATACTCAGCCAGAAAACAAAGTGCTGTTTATTTTTGGTATGGCAAC
CATAGCCATCTATCCCTGGGGTCACTCATTTATCCTAATTCTAGGAAACCGCAAACTGAAGCAAGCCTCTTTGAAGGTAC

TGAAGCATGTAAGGTGCTGGGAAAGAGAGAAACTTCTCAGAATTCCATGA

>CadrTAS2R38_KN271672.1:667704-666697

ATGTTGACCCCGGCTTCCATCGTGACTGTGTCCTACGAAGTCAAGAATGCTTTTCTGTTCCTTTCAGTCCTGGAGTTTGC
AGTAGGGATCCTGGTCAATGCCTTCATCTTCTTGGTGAATTTTTGGGACGTGGTGAGGAGGCAGCCACTGAGCAGCTGTG
ATCTCATCCTTCTGAGCCTCAGCCTCACCCGGCTGCTCCTGCATGGGCTGCTGTTTCTGGACGCCGTTCAGCTGGCCTGT
TTCCAGCGGATGAAAGACCCGCTGAGCGTCAGCTACCAAACCATCATCCTGCTCTGGATGATCGTGAACCAGGCTGGCCT
CTGGCTCACCACCTGCCTTAGCCTCCTCTACTGCGCCAAGATCGCCCGTTTCTCTCCCGCCTTCCCGCTCTGCTTGGCGA
GCTGGGTCCCCAGGAAGATGCCCCAGATGCTCCTGGGCGCCATTACCTTCACCTGCGTCTGCGCCGCTCTCTGTCTGGGG
GACTTTTTCAGGAGATCTCACTTCTCAGTCACAGTTATGTCTTTCGTAAATAACAGTACAGAGTTCGATCCGCAAATTGC
AAAACTTAGTTTCTTCCATTCCTTCATCTTCTGCAGTGTGGGGTCCATCCCTTCTTTCCTAATTTTTCTGGTTTCTTCTG
GGGTGCTGATTGTCTCCCTGGGAAGGCACATGAGGACGATGAGGGAACAAACCGAAGACTCTCGGGACCTCAGCCTGGAG
GCCCATATCAAAGCTCTCCGATCTCTCATCTCCTTCCTCTGCCTGTATCTGCTGTCACTCTGCGCCGCCCACCTCTCCGT
GCCGTTGCTGATGCTGTGGCACAGCAAGGTCGGGGTGATGGTCTGCGTAGGCATCCTGGCAGCCTGCCCCTCGGGACATG
CAGTCATCCTGATCTCAAGCAACGCCAAGCTGAGGAGAGCCCTGGAGAGCATTGCGCTGTGGGCTCAGAGCAGCCGAAAG
GTCAGGGCGGGCCACGGGGCTGCTCGCGGGATGCAGACTCTGTGCTGA

>CadrTAS2R39_KN272510.1:75619-74645

ATGACCAAAACCTGCAATCTCACGGAAAATGAATTTTCACCATTTCTCCTCATTTTAACTTGTATAATTATAGGAAGTGA
ATGCATCATTGGTATCATTACAAATGGGTTCATTGTGGCTATAAATACAGCTGAATGGATGCAGACGAAAGCAGTTTCCA
CAAGCGGCAAGATCCTTCTTTTCCTGAGTGTGTCCAGAATAGCTCTACAAAGCTTCATAATCCTAGAAATTATCTTCAGT
TCAACACTCCCATATTTTTATAATGAAGAAATTGTATATGACACATTCAAAGGAAGTTTCGTGTTCTTAAATTACTGTGG
CCTCTGGTTTGCTGCCTGGCTCAGTTTCTTCTACTTCGTGAAGATTGCTGATTTCTCCTACCCCCTTTTCCTCAAGCTGA
AGTGGAGAATTTCTGGATGGATGTCCTGGCTTCTGTGGCTATCCGTGTTTATTTCCTTGGGCACCTGTGTGTTCTTCCTC
AAAAACATCTACACTGTGCACTGTAACAATTACGTTCCTGTCCCCTCCTCCAACTCCACTAAGAAAAAAGTCTCCACTGA
GACCAATGTGACCAACTTGGCTGTTCTCTTTCAAATGGGGATCTTCATTCCTCTGATCATGTTCATCCTTACAGCCACCC
TGCTGATCGTCTCTCTCAAGAGACACACCCAACACATGTCCAGCAATGCCACAGGTTACAGGGACCCCAGCATGGAGGCT
CACCTGGGGGCCATCAAAGCCACCAGCTACTTTCTCATCCTCTACATTTTCAACTCAGTCGCTCTGTTTCTCTATATGTC
CAACATCTTTGACATCAGCAGTTTCTGGAGTATCTTGTGCAAAGCCATCATGGCCGCCTACCCTGCTGGCCATTCCATTC
TCCTGATTCGGGACAACCCTGGGCTGAGAAGAGCCTGGAAGCGGCTTCAGGCTCGAGTTCATCCGTACAGGGTAGACTCC
ATGACCCACATGTGA

>CadrTAS2R42_KN271481.1:448590-447682

ATGTTCACTGGGTTGAGTGCAATCTTCCTGATGCTGTCAGTAGTGGAATTCATAATCGGAATGTTGGGGAATGTGTTCAT
CGGACTGGTAAACTGCTCTGAGTGGGTCAAGAACCAAAAGATCTCTTTATTTGACTTTATCATCACCTCCTTGGCTATCT
TCAGAATTGGTCAACTGTTGGTGTTTTTCTTTCAGTCCTTTTTAATGGGACTAGATCCACATTTATTTTTTACTCTTAAA
CTAGCAAAAACTATTAGTTTGCTTTGGAGAATCACTAATCACTTGGCTACCTGGCTTGCCACCTGCCTAAGCATTTTCTA
CCTCCTTAAGGTAGCTCACTTCTCCCACTCCCTTTTCCTCTGGCTGAAGTGGAGAATGAACAGAGTCATTCTTGTAATAT
TTGTATTTTCTTTGGTCTTTCTGTTTTTTGACTTTCTCTTGCTAGAAACATTTAACGATCTCTTCTGGATGACAGCAGAT
GAAAGTAATCTGACTTTATATTTAGATGAAAGGAAAATGTTCCACGTACAAAGTGAGATTCTTCTTAGCTTGACCTATCT
CATTCCTGTTGTTCTGTCCCTGATCTCACTGCTGGTTTTATTTCTGTCCTTGGTGAAACACACCAGAAATTTGCAGCTCC
ACTTTGTGGGCTCGAGGGACCTCAGCACAGAGGCCCATAAAAGAGCCATGAGAATGGTGATGTCTTTCCTCCTCGTCGTC
ATGATTCATTTTTTTTCCATGCAGTTGGCAAATTGGATGTTTTTTATGTTTTTTGATAAGAAGTTCACAAAATTCATCAT
ATTGGCAGCATATGTCTTTCCCTCAGGCCATTCGTTTATGTTGATTCTGGGAAACAACAAGCTGAGACGGGGAGCTTTGA
AGGTACTGAGTCATCTTAAAATCTCCTAG

>CadrTAS2R62_KN271112.1:235351-234419

ATGCTCCCCTTACCCGTGCTGATTTTCATGGCCATCTTTCTCCTGGAGTCGCTGGCTGCAATGTTACAGAATGGCTTCAT

GGTGACTGTGCTGAGCAAGGAGTGGGTGCGATGTCGGACACTGCCCACAGGTGACATGATTGTAGCCTGCCTGGCTGCTT
CCCGGTTCTGCCTGCATGGGATGGCCCTCCTGAACAACTTCCTGTCTTCCTTTGGTTTTTATACTGAAACTAAATATTTC
AGCATCCCCTGGCAGTTCATCAACTCTCTCACTTTCTGGCTTACTGCCTGGCTTGCTCTCTTCTATTGTGTGAAGATCTC
ATTCTTCTCGCACCGCATCTTCTTCTGGCTGAAGTGGAGGGTTTCTCGGTCAGTGCCTAGGCTGCTGCTGGGCTCCCTGG
TCCTGTCTGGTCTGGCAGCCGTCTCATCAGTCATTAGGAATATGATTCTAACGCGGAGGACTGAGACCCAGAGTTCCCAG
GGAAACAACACCCTGGCTAGTAGACTACAAACCGTCTCTTCATACATTTTTCTATCTCATATGATTCTTATGTGGTTGAT
TCCATTCCTCCTATTCCTGGTGTCCATCCTCTTGCTCATGTTCTCACTTTGCCGGCACTTGAGGCAGATGACGGACCATA
GACCTGGCCCGAGTGATCCCAGCACACAGGCTCACACTGTCGCCCTGAAGTCGCTTGCCTTCTTCCTCATCTTCTACACA
TCATACTTCCTGTCCCTGGTTATTGCTGTTAGTAACATCAAAGCCCTCCAAAGTCACTGGCACTGGGCCTGGGAAGTGGT
GATCTACGCAGGCATCTGTCTGCACTCCAGCATCCTGGTGCTAAGCAGCCCCAAGCTGAGAAAGGTCCTAAAGAAGAGGC
TTTGGAGAGCCCTGGGCAAAGAGCAGTTTGTCTCGAGTTACCAGTATCAGTAA
>CadrTAS2R67_KN271481.1:441278-440337
ATGCCATCTGGAATTGATAATGCTCTTCTGGCAGCCATAATAGGAGAAGTCATAATTGGAACGTCGGGGAATGGGTTCAT
TGTACTAGTTAACTGCATTGGCTGGGTGAAGAGGAACAAGTTCTCATCAGTCGACTGCATCCTCACCGGCCTGGCTATCT
CCAGAATCAGTCAGCTTTGGATAACGCTGTTTGAATCATTTTTAATGTTGTTTTGGTCACATCTCTATGCCACTGATAAA
CATTTAATAAGTATTGTTGGCATTTTTTGGACATTGTCCAATCACCTAGCTACCTGGTGCGCTACCTGCCTAAGTGTTTT
CTACTTCTTACAAATAGCCAGTTTCTCCCACCCCTGCTTCACCTGGCTGCGCTGGCGAATTCACAGGGTGGTACTTGTCC
TTCTGCTGGGGTCTTTGTTCTTACTGGTTTTCAACTCTAAATTAATACATTCGTTTGGTGAATCCTGGACTAGTATCTAT
AAAATAGATCAAAGAAACTCAACTGCGTCCTCGGGCAAAATGAAAACTCTGTATCTTAATGGGTTGATTGTTTACAGCTT
GATTTGCTTAATGCCCTTTCTTGTGTCCGTGACCTCACTGCTTCTTTTATTCATCTCCTTGAGAAGACACACCAAGATTT
TGAAGCTGAACCCCAGCTCTAGGGACTTCAGCACAGAGGCCCATAGAAGGGCCATGAGAATGATAATTTCTTTCCTCCTA
CTCTTCTTGGTCCACTTTTCTTCCATTCTATCGGTGGGCTGGGCATTCATTATACTGCAGAAGCATCAGGTCAACTTGGT
TGTCATGTTAACTTCAATAGTTTTTCCTTCAGGCCACTCATTTACCCTAATTTTGGGAAACAGCAAGCTGAGACAAAATG
CCTTAGGACTTCTGTGGTATCTTAATTGTCACCTGAAAAGAGTGAAACCTTTAGCTTCATAG
>CadrTAS2R2P_KN271242.1:176091-175238
ATGGCTTCCCCTTGGTCGGCTATTCCTCATGTTATCATCATGTCAGCAGAATTTGTCACAGGGATTACAGTAAATGGATT
TCTTATAATCATCAATTGTAACGAATTGGTCAAAAGCAGAAAGCTAACACCAATACAGCTTCTTTTTATATGTATAGGAA
TGTCTAGATTTGGTCTACAAGTCGTGTTAATGGTACAAAGTTTTTTTCTGTGTTCTTTCCACTCTTTTATAAAGTAAAA
ATTTATGGCACAGCGATGATATTTTTTTGGATGTTTTTTAGCTCTGTCAGTCTCTGGTTTGCCACCTGTCTCTCTGTATT
TTACTGCCTCAAGATATCAGGCTTCACCCAGTCCCATTTTCTTTGGCTGAAATTCAGGATCTCAAAGTTAATGCCTTGGC
TACTTCTGGGAAGTCTGCTGTCGTCTGTGAGCATTGCAACTCTGTAAAGAGGACTAAACTCAAGACAAAGCAAATTAATG
AACTGTTTCTTGTCAACTTGGCATTAGTACTTCCTCTAGCCATATTTGTGATGTGTACTGTTACATTATTCATTTCTCTC
TGCAAGCATACTCATCGGATGCAAAACGGATCTCATGGTTTTAGAAATGCCAGCACAGAAGCCCATATTAATGCATTAAG
AACAGTGATAACATTCTTCTGCTTCTTTGTTTCTTATTTTGCTGCCTTCATGGCAAATGTAACATTCAATATTCCTCACG
GAAATCAGTGGTTCTTTGTGGTGAAGGACATAATGGCAGCGTATCCCTCTGGCCACTCAATTATAATGATCTTGAGTAAT
TCTAAGTTTCAACAACCATTCAGGAGACTTATCTGCCTTGAAAAGAATCAAAGA
>CadrTAS2R4P_KN271672.1:569252-570107
ATGCGTCAGGCATTCTTTTTTTCTTCTCTTATTATCTCAGCTATTGTGACTTCTGTTGGAGTCATTGCAAATCTGTTCAT
TGCAGTGATCAATTACAAGAATTGGGTCAAAAGCCACAAGATCTCCTCTTCTGATAGAATCCTGTTCAGCTTGGGCATCA
CCCGAGTTCTCATCCTGGGACTGAGTACTATTTTCTCCATCTCTTCAAGTATTCGAAGGTCAGTCTCCATATCCACTTAT
TTTCTGTCGTGTTGGTTGTTTTTGGACTCTAATAGTCTTTGGTTTGTAACCTTGCTCAACGCCTTGTACTGTGTGAAGAT
TACTAACTACCAACACCCAGTTTTTCTCCTGCTGACTGCTGGCCTGTGTGCTGATTTCTGCCTTTACCACTCTCCTGCAT
GTTGTGCTCCGAGAAGCATCACGCTCTCTGGAATTGGTGACCGGGAGAAATGGCACCGTCTGTGACGTCAATGAGGAAAT
CTTCCTTTTGGTGAGCCCTTGGCTCTTGAGCTCATGTCTACAGTTCTCCCTTAATGTGACTTCTGCTTCCTTGTTAATAA

ATTCCTTGAGGAGACACATCCAGAAGATGCAGAGAAGTGCCACTGCTCTTTGGACTCCCCAGACCGAGGCTCATGTGGGT
GCTATGAAGCTGATGATCTGTTTCCTCATACTCTACGTTCCGTATTCAGTTGCTTCCCTGCTCCATTATCTCCCTTCTGT
AGCGAGTGATTTGAGACTCAAGTCCATTTATATTATTATTTCCACCTTTTACCCTCCAGCACATTCTGTTCTCATTATTC
TCACACATCCTAAACTGAAAACAAAAGCAAAGAAGATTCTTCACTTCAATAAATAG
>CadrTAS2R5P_KN271672.1:578453-579345
ATGCTGCCCGCTGTCCTAGGACTGCTGGTGCTGGTGGCAGTGGCTGAATCTCTCACTGGCCTGGTTGGAAATGGAGTCCT
TGTGGTCTGGCATTTTGGGGAATGGGTCAGAAAACTAGAGGGGTCCTCATATAACCCCATTGTCCTGGGCCCGGCTGTCT
GCTGATTTCTCCTGCAGTGCTTGATTGTGGTGGACTTAAGTCTGTTTCCGCTTTTGCAGGGCAGCCGTTGGCTTCACTGT
CTCAGTGTCTTCTGGGTCCTGATAAGCCAGGCCAGCCTGTGGTTTGCCACTTTCCTCAGTATCTTCTACTGTAGGAAGAT
CACAGCCTTTGAACACCCTGTCTACTTGTGGCTGAAGCAGAGGGCCTGTTGCCTGAGTTGCCGGTGCCTTCTGGTGTACT
GCACAGTTTGTTATTTACATTTCACGGTGGCTTAGATCTCTGCCATTCTTCCAAAGGAAACAGCATTTTATTCCCCCTTT
CAGACTGGCACCATGTGTATACATTACAGTTCAGTGCAGGAAGTGTGTTGCCTTTCATGATGTTTCTTATTTCCTCTGGG
ATGCCAATGGCCTCTTTGTGTAGACACCACAGGAAGATGAAGGTCCACACAGCCAGCAGGAGAGATCTTCAGGCCACGGC
TCACATCACTGTGCTGAAGTCCTTGGGCTGTTTCCTCATACTTTACATAGTTTACTTTGTGGCCATCCCCTTCTCCATCA
CCTCCAAGTCTTTTCCTGCTAATTTCGCCACTCTGTTCATCTCTGAGACACTCGTGGCTGCCTACACTTCTCTCCATTCT
GTCATCTGGATCATGGGGAATCCCAGGTTGAAGCAGACTTGCCAGAGAATCCTGTGGAAGACAGTGTATGCTAGGAGATC
CTGGGGCCTGTGA
>CadrTAS2R11P_KN271290.1:3394561-3395052
AACCAGTCTACTGTGGCTAATAATTGTCAATTTGTTTTTAGTGCTATTCTATCAGGAAACTGATACCATGGGGAGAAAAT
ATATCTTACTGGCATTTGGATACTGGCCAACCACTTGAGCACTTGGTTTGCTACTTGTCTCTCTGTCTTGTATTTCCTGA
AGATCACCAATTTCTCCTATCCCTCTTTCCTTTGGCTAAAATGGAGAATTAACAAGGTAACTGTCATGCTTCCGCTGGCA
TCTGTGCCCTTCCTGTTCATGAGCTTTCCTTTGCCATATGGTTTTGATGTCTTCTGGTATTACAGCCTCCCAAAATGTGA
GACAAATATGACTGGGTTATTCAATGTAAGCAAAAATAAAAATTTGAAATCTACGATTACCTTCACAGATTTTTATTGAT
TCACCAGTACAGTTCCCGGAGGTCAGGCACACTCAAATTTTATACATATTTCTAAAACCCCAGAATTAAAACAACAACA
ACGTTTAAGAAC
>CadrTAS2R13P_KN271481.1:415689-414766
ATGGTAAATGTCTTGAATATCATCTTCATCATTTTAATACACTCAGAATTAGTAACTGGTATTTTGGGGAATGGATTCAT
AACACTGGTGAACTGCATTGACTGGCTCAAGACTTGAAGGATCTCCTCAGCTGATCGAATCCTCACCACGTTGGCAATCT
TTAGAATTTGTCTGATTTTGGTAATAGTGGTGAGTTGGTTTACAAGGGAAATTTCTCCATCTTTATATTTGAATAGAAAG
AATGTTATACTTATTACTATTATTTATTACCATTGCATGGACCTTGGCCAACCATTTTAGCACCTGGCTTGCCACAGACC
TCAGCCTCTTTTATTCTCTCAAGATGGCCAATTATACAAATCCTGTTTTTCTTCACTTAAAGCATAGAGTTGAAATGGTA
GTTCTGGTAATGTTTCTGGGGGCATTAGTATTGTTGCCTTTATGTCTTATTATGATAAGTATTCTTATTAATATCCAGAT
ACATCCATATGAAAGAAATGTGACTTTGAGTTCTAAAAGGAGTGACACGGAAAACTTTTTGAAACTGATTATATTCAGTA
TGGGAGACTTCATACCCTTTATTATATCCCTGATGTTTTTCCTCCTGCTAATCTCCTTATTGAAACACCTCAAGAAGATG
AAGCACCATGCAATGGGATTCAGAGATCCCAGCATCAAAGCCCACGTCAGTGCCATGAAAATGGTGATATCTTTCCTCAT
GCTATTTGCTGTTTACTTCCTGTCTATTCTTATGACAGCCTTCCATTCTGATATGACTCAGAACAAATCTACCCTTATGC
TTGGTCAGGTTCTTGCAAATGCTTATCCTTCAGTCCACTCATTTGTCCTAATTCTGGGAAACAGTGAGCTATGGAGGGCT
TCACTTTCGGTGTTGTGGCAGCTGAAATGCAGTTTAAAAGCTGGTAATGCGTGATGTGAATA
>CadrTAS2R16P_KN271152.1:11296613-11295710
ATGATACCCATCCAACTCACTGTCTTCTGCATAATCGTCTACATGCTTGAGTCCTTGATAATAATTGTGCAGAGCAGCTT
AACTGCTGTGGTGCTGGGCAGAGAGTGGGTGCAGGTTAAAAGGCTGTCACCTGTGGACAAGATTCTCACTGTTCTGGGCA
TCTGCCGCTTCTGTCAACAGTGGTCATCGATGCTGTTCAATTTCTGCTCCTACCTCCACCCTAACTATGTATTTTGGTAC
TTGGCGATCGTCTGGGAATTTACTAACACTTTCGTTCTGGTTAACCTCCTTGCTTGCTGTCTTCTACTGTATCAAAGTCT
CTTCCTTCAGCCATCCCATCTTCCTCTGTCTGAAGCGGAGAATTGGGAAGTTGGTTCCTCGGCTGCTGCTGGGTTGCCTG

CTGATTTCTTGTCTGTCAATCATCTTTTCAGCTACGAGGCATCGCATCACGATTCAGTTAAAGTCTATGAGGCACTTTGC
TAGAAACAGCACTGTGATTGAAAGACTTGAGACATTTCAGCGGGATTTTTCCATATATCAGCAAGTGGCGGTGTTGGTTA
TTCCTTTCCTCCTGTTCCTGGTCTCCGTTGTCTTGCTCATGACCTTATTGTCACAACATCTGAGGCAGATGAAACATGGT
CACACTGGGCACTCCAGCTCCAGCCTGAAAGCTCACGTTACTGCCCTGAGGTCTCTGGCCATCTTCCTCATCATCTTCAC
ATCTTATTTTCTGGCCATACTCATCTCCATCATAGGTACCCTCCTGGATAAGAGGTCCTGGTTCTGGGCCTGGGAAGCTG
TCATCTATGCTGTAGTCTCTATTCATTCCATTTACTGATGCTGACCAGCCCTAAGTTGAAAAAGGCCTTACCAGTAAGA
TGCTAGGACTTAGAGGCTGCCTGA
>CadrTAS2R40P_KN272510.1:45571-44595
ATGGCCACAGTGAACACAGATGCCACGGATAAAGACACAACCACATTTAAAATCATCTTCATCTTGGCGGTCTCCAGAAC
AGAGTGCATCACTGGCATCGCTGGGAACGGCTTCATCACGGCCATCCATGGGGCCAAGTGGGTCAGCGGCAAAGGACTGC
CTGCTGGTGACTGCATTCTGTTGATGCTGATCTTTTCCAGGCTCTTGCTATGGGTTTGGATGATGCCAGAGAACACTTAC
AGTCTACTCTGCCCGGTCACTTATAACCAAAATGCAGTGCATACACTTTTCAAAGCCATCATCATGTTTCTGAACTATTC
CAACCATTGGCTTGCCACATGGCTCAATATCTTCCATTGTCTTAGAATCACAAACTTCACCCACCCTTTGTTCTCCGTGA
TGAAGAGGAAAATCATGGTGCTGATACCCTGACTTGTGAGGCTCTCACTGTTAATCTCCTTATGCTCCAGCTTCCCCTTC
TCTGTAGATATCCTCAGTGTGTCTGTGAATAGTTCCTTTCCTATTCCTTCCTCCAGCTTCCCTGAGAAGGTGTACATGTC
CAAGACCAACGTGGTCAACTTGGTTCTCACCCCTTACCTGGGGGTCTTCATTCCTCTGATCATGTTCATCCTTGCAGCCA
CCCTACTGATCATCTCTCTCAAGAGACACACCCTACACATGGCCAGCAATTCCACAGGGCTCCAGGGACCGACCCCAGCA
TGGAGGCTCACATGGGAGCCCTCAAAGCTATCAGCTCTTTTCTCATTTTCTACATTTTCAGTGCCGTTGCTCTATTTCTT
TCCATGTCCAACATCTTTGATGTCAAAAGTTCCTGGAGCATTTTGTGCAAAACCGTCATGGCTGCCTACCCATCCAGCCA
CTCAGTGCTATTGACCTTGGGCAACCCTGGGCTGAAAAGAGTGTGGAAGCAGTTTCAGCACCCAGTTCATCTTTGCCTGT
AGGGACAGACTCTGTGA
>CadrTAS2R41P_KN271112.1:209161-208236
ATGCATCCAGCACTCACAGCCCTCTTCATGCTGCTCTTTATCCTGCTGTGTGTCCTGGGAATCTGGCCAATGCTTTCATT
GTGTGGGTGCTGAGCAGAGAGTGGGTGCGACGGGGGAGGCTGCTCCCCTCCGACATGATCCTTATTAGCTTGGGTGCCTC
CCACTTCTGCCTGCAGTCGGTTGGAATGGGGAACAACTTCTACTACTTCCTCCACGTGGTCGAGTACCGTCGGGGTCCTG
CCTGGCAGTTATTTGGTCTATTCTGGGACTTTCTGAACTCAGCCACCTTCTGGTTTGGCTCCTGGCTCAGCGTCCTCTTC
TGCATGAAGGTTGCTAACTTCACCCACCCCACCTTCCTCTGGCTGAAGTGGAGGTTCCCAGGGGTAGTGCCCTTGCTTCT
GCTGGGATCTCTCCTCGTTGCCTTCATTGTCACCCTGCTGTTCTTTTGGGGGAACCATGCTCTGTATCGAGGATTCTTTA
TTGAAACATTTTCTGGGAATATGGCCTACAAGGAGTGGAGCAGGAGGCTGGAAATGCACTATTTCCTGCCCCTGAAGCTA
GTCACCCTTTCAATTCCTTGCTCTAGTTTTCTGGTCTCAATTGCGCTGTTGGTAAGTTCTCTGAGGAGACACACGAGGAG
GATGCAGCACAGTGCACACAGCCTGCAGGACCCCAGCGCCCAGGCTCACACCGCAGCTCTGAAGTCACTCATCTCCTTCC
TCGTTCTTTACCTTCTGTCCTTCCTGTCCCTGGTTATTGACGCTGTAGTGTTTTTCTCCTCGGAGAGTGAATGGTACTGG
CCATGGCAAATTGTAACCTACTTGTGCACATCTGTCCATCCCTTTATCCTCATCTTCAGCAACCTTAGGCTTTGAGGGGT
GTTCAGGCAGCTAATTCTGTTGGCCAGGGGCTTCTGGGTGGCCTAG
>CadrTAS2R408P_KN271481.1:421228-420282
ATGATGAATTTACTACCAAGCATTTTTTCTATCCTGTTAATAGCATACCTTATTCTAGGGAATTTTGCCAGTGGCTTCAT
AGCATTGGTGAACTGCACTGACTGGGTCAAGTGACCGAAGATCTCCTCAGCTGATGGAATTCACACTGCTTTGGGACTCT
CCAGAATTGGCTTACTCTGGGTAATATTAATAAATTGATACGCAAATATGTTTAATCCAGATTTAGACAATTTAAGAGCA
AGAATTACTACTATTGCCTGGACAATAGCAATCATTATAGCATCTAGTTTGCTGCTGCCTTCAGCATATTTTATTTGTTC
AAGATAGCCAATTTCTCCAGCCTTATTTTCCTTCACCTAAGGTGAAGAATTAAAAGTGTACTTCTTGTGATAACGTTGGG
TACTTTGTTCTTTTTGGTTTTTCAGTTTGCGGCAGTAAGCATAGATGAAAATATCCAGACAAATGCATATGAAGGAAACA
TCACTTGGAGGACCAAATTGAGGGCCATTTGACACATTTCAAATATGACTCTGTTCATGCTAATAAACTTCATACCCTTT
ACTATGTCCCTGATATCTTTTCTGCTGTGAATCTTTTCCCTATGGAAACACTTCAAGAAGATGCTGCTCAGTAGGAAAGG
ATCCCAAGATCCCAGCACCAAAGTCTATATAAAAGCCATGCAAACTATCTCTTGTCTCTTGCTATTTGCCCTTTACTTCT

GGGCTCTAATTTTCTCAATTTGGAGTTCCAGTAGGCAGCAGAACAAACTAGTTATGATGGCTTGCCAGGCTTTGGAATCA
TATACCCTGTAAGCCACTCATTTATCCTGATTTGGGGAAACAAGAAGCTAAGACAGGCCTTCCTGTCTTTTCTTTGGCAG
CTGAGGTGCTGGCTAAAAGAAAGGAAATAAGTGGGCGTCATGTATCTTCTAGCAGAAAACAAACTGA
>CadrTAS2R60P_KN271112.1:229536-228591
ATGGTTCCAGGACCTAAGGTGACTGATAAAGAGAGCCATCATCTTCGTTATCATTTTATTCCTTTTGTGCCTGGTGGCAG
TGGTGGGTAACGGTTTAATCACTGCAGCACTAGGCATGGAGTGGTGGCTGCGGAGAACGTTGTCACTCTGCGATAAGTTA
TTGGTCAGCCTGGGGGCCTCTTGCTTCTGTCGGCAATGGGTGGTGATAAGTAAGAACATGTATATTTTCCTGTATCCAAT
GGCCTTCCCACACAACCTTGTATTACAGTTCCTAGCCATTCAGGGGGACTTCTTGAATGCTGTCACGTTGTGGTTCTCCA
CCTGACTCAGTGTCTTCCACTGCATGAGAATTGCAACATTCACCCACCCTGCCTTCCTCTGGCTAAAGAGCAAGTTGTCT
GGGTTTGTCCCTTGGATGCTGCTCAGCTCTGTGCGGTTCTCCAGCATCAGCACCAGCACCATTCTATTTTTCATAGGCAA
CCAGAGAATATACCAGAACTATTTAAGGAAAGGTCTGCAATCTTGGAATGTCACTGGGAATACTCTGAGGAGAACATATG
AGAGATTGTACTTCTTCCCTCTGAAAATTGTTACCTGGACAGTCCCTACAGTTGTCTTCATCACTGACATGGTTTTGCTC
ATTACATCTCTGGGAAGACACACCAAGAAGGTCTTCCTGTCCATCTCAGGCTTTCCTGATCCCTGTGCCCAGGCACACGT
CAAGGCTCTCCTGGCTCTCATCTCTTCCATCCTCTTCATTCCCTATTTTCTGTCACTGGTGTTCAATGCTGCAGGCATGT
TTCCACTCCAGGAATTCAGGTGCTGGGTGTGGCAGACTCTGATTTATCTGTGCATGGCATTCTACCCCATCATTCTACTC
CTGAGTAACCGCAGGCTGAGAGCTGTGCTGGAGAGGTGCTGCTCCTCAAGGTGTGGGGCATCTTGA
>CadrTAS2R18P_KN271481.1:426948-426057
ATGTCAGTGGGAATGAAAGTCTCCATTCTGGGAGTGGCAACAGGAGAACTGATCTTAGGAATGCTGGGAAATGGGCTCAC
TGGACTGGTAGTCTGCATGGAATGGGTCAAGAATGGGAAGGTCTCATCAGTTGATTTCATCCTTACCAGTTTATCTGTGG
CCAGAATCAGTCAGCTGTGGGTAACACTATTGGATTCATTTATAGTATGGCATCTCCACACGTGTATGCCACCGGCAAAC
TAGTGAAAGTGTTCTTTGGGCACTAATGAATCGCTTAACTACCTGGTTTGCCGCCTACCTGAGCATTTTCGTCTTCCTTA
ATGTGATCAGTTTCTCCCAGTTTTTTTCATCTGGCTGAAGTGGAGAATGAACAGAGTGGCTCTTGTGCTTTTCCTGGGCT
CTTTGTTCTTACTGTCTGTTACCCTCCTGATGCGGGACGCTCTTGGTGAGTTGTGGGTGAATAGCTACAGAGGACGTGAG
AGAAACACGACTTTGTATTTAGATGGAAGTAAACTTTTCTGTCTTAAAAGCCTTGTTCTTCTTAGCTTGATCTGTGTTAT
CCTGTTCCTTCTCTCCCTGGCCCCTTTGCTGCTTTTATTTCTCTCCTTGGTGAAGCATACCAAGAATCTACAGCTCCACC
TGAATGAATGGAGAGACCCATAAAAGGGCCATGAAAATGGTGACAACCTTCCTCCTTCTCTTCATCATTTATTTTATTTC
CACTCTAATGGCAAGCTGGATTTTTCTTAAGGTACAAAGTTATCAGGGCATTTTCCCTCAGTTCACTTCTATGAATTTCA
GTTTTTGGACTAGCAAGCTAGGGTAGATTGCCTTGAAACTACTGTGGTACCTTACACTCTCTCTAAAAAAAGCAAAACCT
TTAGCTTAATAG
>CabaTAS2R1_KN276197.1:339782-338895
ATGCTGGAGTCACAGCTCATTAGCCATCTTCTCTTGACAGCGATACAGCTTCTCACTGGGCTTTTTGTAAATGCCATCAT
TGTGGTTGTGCACGGCACGGGCTTGCTCAAGCACAGAAAGATGATTCCGTTGGAGGCCCTTGTTTCCTGCCTGGCGATGT
CCAGGATCTGTCTAGAGCTGGCCATCTTCTACCTTAATCTGGCTGTTTTTAACTTGATTGACATCCGTCAGTTTGTGGGG
AAGTTTGTAATTCTCTCCTTTGTAAATGAATTGGGGCTGTGGTTTGCCACATGGCTCAGCGTCTTCTACTGCATCAAGAT
TGCCAGGATCGCTCACCCTCTCTTCCTCTGGTTGAAGCTGAAGATCTCCAAGCTGGTTCCTTGGCTGATTCTGGGGTCCC
TCCTACATGCATCTAGCACCTGTGTCTTCCACAGCAAACAGCCATGGATCTTTTCCGAAGACCTCCTGGGCTTTTCCTCT
CAAAATGCAACAGCTGAAAGCGAAGACATCGCTACTTTGCGGTTCACCCTTCTCTTCGCTGAGCTCTCCTTGCCATCACT
CATCTTCCTCATTTCTGCCCTGCTCTTGCTGTTCTCCCTGGGGAGACACACCTGGCAGATGAGAAACACAGCCGCAGGCC
CTTGCCCCCGTGCGCGCGTCAGGTCCCTCCTGTCCATCTTCGCCTTCCTGGTCCTCTACCTCTGCCACTACCTGATGGTT
CTTTTGCTCTTTTCTCAAGTTTTCAAACTTACAAGCCTCAGATTTCTGTTCTGCATCTCGCTGGTTGGTTCCTGCCACTC
CGGACACTCTATTATCTTAATTTTAGGAAATCCTAAACTGAAACAAAATGCGGAGAAGTTTCTCCTGCATAGAAAGTGCC
GTCAGTGA
>CabaTAS2R2_KN276392.1:897752-896823
ATGGCTTCCCCTTGGTCGGCTATTCCTCATGTTATCATCATGTCAGCAGAATTTGTCACAGGGATTACAGTAAATGGATT

TCTTATAATCATCAATTGTAACGAATTGGTCAAAAGCAGAAAGCTAACACCAATACAGCTTCTTTTTATATGTATAGGAA
TGTCTAGATTTGGTCTACAAGTCGTGTTAATGGTACAAAGTTTTTTTTCTGTGTTCTTTCCACTCTTTTATAAAGTAAAA
TTTTATGGCACAGCGATGATATTTTTTTGGATGTTTTTTAGCTCTGTCAGTCTCTGGTTTGCCACCTGTCTCTCTGTATT
TTACTGCCTCAAGATATCAGGCTTCACCCAGTCCCATTTTCTTTGGCTGAAATTCAGGATCTCAAAGTTAATGCCTTGGC
TACTTCTGGGAAGTCTGCTGTCGTCTGTGAGCATTGCAACTCTGTGCATCAAGGTGGATTACCCTAAAAACTTGGATAGT
GATGTCCTCAGGAATGCCACGCTAAAGAGGACTAAACTCAAGACAAAGCAAATTAATGAACTGTTTCTTGTCAACTTGGC
ATTAGTACTTCCTCTAGCCATATTTGTGATGTGTACTGTTACGTTATTCATTTCTCTCTGCAAGCATACTCATCGGATGC
AAAACGGATCTCATGGTTTTAGAAATGCCAGCACAGAAGCCCATATTAATGCATTAAGAACAGTGATAACATTCTTCTGC
TTCTTTGTTTCTTATTTTGCTGCCTTCATGGCAAATGTAACATTCAATATTCCTCACGGAAATCAGTGGTTCTTTGTGGT
GAAGGACATAATGGCAGCGTATCCCTCTGGCCACTCAATTATAATGATCTTGAGTAATTCTAAGTTTCAACAACCATTCA
GGAGACTTATCTGCCTTGAAAAGAATCAAAGAAGAGGAGGACCAATGTAA
>CabaTAS2R3_KN276478.1:470641-469691
ATGCTGGGATGCACCGAGTGGGGCTTTCTGGTTCTGACTGCCAGTGAGTTCGTCCTGGGAGTGCTGGGCAATGGCTTCAT
AGGGCTAGTCAATGGCAGCAGCTGGTTCAAGAGCAAGAGAATCTCTTTGTCCGACTTCATCATCACTAACCTGGCTCTCT
CTAGGACTGTTCTGCTGGGGACTCTCTTGGTTGATGGTGTTTTAATGGTGTTCTCGTCCAAACTACATGATGAAGGGATA
GTAATGCAGATGATTGATATTTTCTGGACGTTTACAAACCATCTGAGCATATGGCTTTCCACGTGTCTCAGTGTCTTCTA
CTGCCTGAAAGTCGCCAGTTTCTCCCACCCTGCATTCCTCTGGCTCAAGTGGAGAGTTTCCAGAGTGGTCGTGTGGATGC
TGATGAGTACCCTGCTCCTAGCATGTGGCAGCACCGTGTCTCTGGTCCATGAATTTAAGATCTATTCTGTTCTCAGTGGA
ATTGATGGAACAGGAAATATGACTGAGCAGGTTTTAAAGAAGAGAAACGAATACAAACTGATCCATGTTTTGGGGACTCT
GTGGAACCTCCTTCCCCTAACTGTGTCTCTAGCCTCCTACTTTCTGCTCATCTGCTCCCTGGGAAGGCATACGCGGCAGA
TGCAGCGGAACTGTACCGGCTCCAGGGATCCAAGTACCGAGGCCCACTATAGGGCCATCAAAGTCATCCTCTCCTTCCTC
TTTCTCTTCCTACTTTACTTTCTTTCCTTTTTAATTTTATCATCTAGTTATTTCCTACCAGCAACTAAAATGATTAAGAT
GATTGGAGAAGTGATTACAATGTTATATCCTGCTGGCCACTCATATATTCTCATTCTGGGAAACAGCAAGCTGAAGCAGA
CGTTTATGGAGATGCTCTGGTGTGGGCATGGTGATCTGAAGCCTGGATCCAAGGGCCCTTTTTCTCCATAG
>CabaTAS2R7_KN276461.1:1587367-1586396
ATGCCAGGTAAGGTGGAGAACACCTTAATCCTCATAGCAGTAGGAGAATTTTCGCTGGGGATCTTAGGGAATGTGTTCAT
TGGATTGGTAAACTTTGTGGACTGGATCAAGCATAGGAAGATCGCCTCCATTGATTTAATCCTCACAAGTCTGGCCATAT
CCAGAATTTCTCTATTGTGTATAATACTATTAGACTGTTTTATACTGGTTCAGTATCCAGACGTCTATACTGCCGGTAAA
CAAATGAGAATCATTGACTGCTTCTGGACACTAACCAACCATTTAAGTGTCTGGTTTGCCACCTGCCTGAGCATCTTCTA
TTTCCTCAAGATAGCTAATTTCTTCCATCCCCTTTTCCTCTGGATGAAGCTGAGAATTGACAATGCGATTCCTAAGATCC
TGCTGGGGTGCTTGGCCCTCTCTGTGTTTTTTAGCCTTCCTGTCTCTGGGAATTTGAATGATGATTTCAGGTTTTGTGTC
AAGGCAAAGTTGAAAACAAACTTAACTTTGAGATGCAAAATAAATAAAGCGCAATATGCTTCCACCAAGATATATCTCAA
CCTGCTAACACTAGTCCCCTTTTCTGTGTCCCTGATCTCGTTTCTCCTCTTGATTCTCTCCCTGTGGAGACACACCCGGC
GAATGCAGCTCAATGCCACAGGGTCCAGAGACCCCAGCATAGAAGCCCATGTGGGAGCCATGAAGGCGGTCATCTCCTTC
CTCCTCCTTTTCATTGCCTACTACTTGGCCTATCTTGTAGCCACCTCCAGCTACTTTCTACCAGAGACTGAATTAGCTGT
GATGGTTGGTGAGGTGACAGCTCTAATCTGTCCCTCAAGCCATTCACTTATCCTAATTCTGGAGAACAGTAAATTAAGAA
AAGCATTTCTAAGGGTTCTATGGAAAGTAAAGTATGTCCTAAAAAGGAGTTGCTAA
>CabaTAS2R10_KN276461.1:1596364-1595435
ATGCTAAGTGTAGGAGAAGGCCTCCTTCTTTTTGTAGCAGTTAGTGAGTCAGTACTGGGGGTTTTAGGGAACGGATTTAT
TGGACTTGTAAACTGCACTGACTGTGTGAGAAACAAGAAATTCTCTATGATCAGCTTTATTCTCACTGGCTTAGCTACTT
CGAGAATTGGTCTGTTATGGTTAATAATTACAGACGGATTTGTAAGGATATTCTTTCCAGAAATGTATTCCTCCGGTAAC
CTGGTTGACTATATTAGTTACTCATGGATAATTCTGAATCAATTAAGTGTCTGTCTTGCCACCAGCCTCAGTGTCTTCTA
TTTCCTGAAGATAGCAAAATTTTCCCACCACATTTTTCTCTGGTTGAAGAGAAGACTCAACAGGGTTCTTCTCATTCCAA
TGGGATTATTGCTAATTTCATGGTTATTTACTTTTCCACAGATGGTGAGGATTATTAGTGATAATAGAATAAGAAATGGA

AGTACAACCGGGGTAACCAACATGCACAAAGATAAATTCCTTACATACCAGATTTCTCTCAATCTGGGGACCATTCTCCC
CTTTCTACTCTGCCTGATTACATGTTTCCTATTGATCATTTCCCTTTGGAGGCACAACAGGAAGATGAAATTGAATGCCA
CAGGATTCAGGGACCCCAGCACAGAAGCACATATCAAAGCAATGAAAGTCTTGATATCTTTTGTCATCTTGTTTTTCTTA
TATTTTGTAGGCGTTGCTATAGAAACATATCACTATACTCAGCCAGAAAACAAAGTGCTGTTTATTTTTGGTATGGCAAC
CACAGCCATCTATCCCTGGGGTCACTCATTTATCCTAATTCTAGGAAACCGCAAACTGAAGCAAGCCTCTTTGAAGGTAC
TGAAGCATGTAAAGTGCTGGGAAAGAGAGAAACTTCTCAGAATTCCATGA
>CabaTAS2R38_KN276478.1:362373-363380
ATGTTGACCCCGGCTTCCATCGTGACTGTGTCCTACGAAGTCAAGAATGCTTTTCTGTTCCTTTCAGTCCTGGAGTTTGC
AGTAGGGATCCTGGTCAATGCCTTCATCTTCTTGGTGAATTTTTGGGACGTGGTGAGGAGGCAGCCACTGAGCAGCTGTG
ATCTCATCCTTCTGAGCCTCAGCCTCACCCGGCTGCTCCTGCATGGGCTGCTGTTTCTGGACGCCGTTCAGCTGGCCTGT
TTCCAGCGGATGAAAGACCCGCTGAGCGTCAGCTACCAAACCATCATCCTGCTCTGGATGATCGTGAACCAGGCTGGCCT
CTGGCTCACCACCTGCCTTAGCCTCCTCTACTGCGCCAAGATCACCCGTTTCTCTCCCGCCTTCCCGCTCTGCTTGGCGA
GCTGGGTCCCCAGGAAGATGCCCCAGATGCTCCTGGGCGCCATTACCTTCACCTGCGTCTGCGCCACTCTCTGTCTGGGG
GACTTTTTCAGGAGATCTCACTTCTCAGTCACAGTTATGTCTTTCGTAAATAACAGTACAGAGTTCGATCCGCAAATTGC
AAAACTTAGTTTCTTCCATTCCTTCATCTTCTGCAGTGTGGGGTCCATCCCTTCTTTCCTAATTTTTCTGGTTTCTTCTG
GGGTGCTGATTGTCTCCCTGGGAAGGCACATGAGGACGATGAGGGAACAAACCGAAGACTCTCGGGACCTCAGCCTGGAG
GCCCATATCAAAGCTCTCCGATCTCTCATCTCCTTCCTCTGCCTGTACCTGCTGTCACTCTGCGCCGCCCACCTCTCCGT
GCCGTTGCTGATGCTGTGGCACAGCAAGGTCGGGGTGATGGTCTGCGTAGGCATCCTGGCAGCCTGCCCCTCGGGACATG
CAGTCATCCTGATCTCAAGCAACGCCAAGCTGAGGAGAGCCCTGGAGAGCATTGCGCTGTGGGCTCAGAGCAGCCGAAAG
GTCAGGGCAGGCCACGGGGCCGCTCGCGGGATGCAGACTCTGTGCTGA
>CabaTAS2R39_KN276119.1:1205604-1204630
ATGACCAAAACCTGCAATCTCACGGAAAATGAATTTTCACCATTTCTCCTCATTTTAACTTGTATAATTATAGGAAGTGA
ATGCATCATTGGTATCATTACAAATGGGTTCATTGTGGCTATAAATACAGCTGAATGGATGCAGACGAAAGCAGTTTCCA
CAAGCGGCAAGATCCTGCTTTTCCTGAGTGTGTCCAGAATAGCTCTACAAAGCTTCATAATCCTAGAAATTATCTTCAGT
TCAACACTCCCATATTTTTATAATGAAGAAATTGTATATGACACATTCAAAGGAAGTTTCGTGTTCTTAAATTACTGTGG
CCTCTGGTTTGCTGCCTGGCTCAGTTTCTTCTACTTCGTGAAGATTGCTGATTTCTCCTACCCCCTTTTCCTCAAGCTGA
AGTGGAGAATTTCTGGATGGATGTCCTGGCTTCTGTGGCTATCCGTGTTTATTTCCTTGGGCACCTGTGTGTTCTTCCTC
AAAAACATCTACACTGTGCACTGTAACAATTACGTTCCTGTCCCCTCCTCCAACTCCACTAAGAAAAAAGTCTCCACTGA
GACCAATGTGACCAACTTGGCTGTTCTCTTTCAAATGGGGATCTTCATTCCTCTGATCATGTTCATCCTTACAGCCACCC
TGCTGATCGTCTCTCTCAAGAGACACACCCGACACATGTCCAGCAATGCCACAGGTTACAGGGACCCCAGCATGGAGGCT
CACCTGGGGGCCATCAAAGCCACCAGCTACTTTCTCATCCTCTACATTTTCAACTCAGTCGCTCTGTTTCTCTATATGTC
CAACATCTTTGACATCAGCAGTTTCTGGAGTATCTTGTGCAAAGCCATCATGGCCGCCTACCCTGCTGGCCATTCCATTC
TCCTGATTCGGGACAACCCTGGGCTGAGAAGAGCCTGGAAGCGGCTCCAGGCTCGAGTTCATCCGTACAGGGTAGACTCC
ATGACCCACATGTGA
>CabaTAS2R41_KN276119.1:971549-970623
ATGCATCCAGCACTCACAGCCCTCTTCATGCTGCTCTTTATCCTGCTGTGTGTCCTGGGAGTCCTGGCCAATGCTTTCAT
TGTGTGGGTGCTGAGCAGAGAGTGGGTGCGACGGGGGAGGCTGCTCCCCTCCGACATGATCCTTATTAGCTTGGGTGCCT
CCCACTTCTGCCTGCAGTCGGTTGGAATGGGGAACAACTTCTACTACTTCCTCCACCTGGTCGAGTACCGTCGGGGTCCT
GCCTGGCAGTTATTTGGTCTATTCTGGGACTTTCTGAACTCAGCCACCTTCTGGTTTGGCTCCTGGCTCAGCGTCCTCTT
CTGCATGAAGGTTGCTAACTTCACCCACCCCACCTTCCTCTGGCTGAAATGGAGGTTCCCAGGGGTAGTGCCCTTGCTTC
TGCTGGGATCTCTCCTCGTTGCCTTCATTGTCACCCTGCTGTTCTTTTGGGGGAACCATGCTCTGTATCGAGGATTCTTT
ATTGAAACATTTTCTGGGAATATGGCCTACAAGGAGTGGAGCAGGAGGCTGGAAATGCACTATTTCCTGCCCCTGAAGCT
AGTCACCCTTTCAATTCCTTGCTCTAGTTTTCTGGTCTCAATTGCGCTGTTGGTAAGTTCTCTGAGGAGACACACGAGGA
GGATGCAGCACAGTGCACACAGCCTGCAGGACCCCAGCGCCCAGGCTCACACCGCAGCTCTGAAGTCACTCATCTCCTTC

CTCATTCTTTACCTTCTGTCCTTCCTGTCCCTGGTTATTGACGCTGTAGTGTTTTTCTCCTCAGAGAGTGAATGGTACTG
GCCATGGCAAATTGTAACCTACTTGTGCACATCTGTCCATCCCTTTATCCTCATCTTCAGCAACCTTAGGCTTCGAGGGG
TGTTCAGGCAGCTAATTCTGTTGGCCAGGGGCTTCTGGGTGGCCTAG
>CabaTAS2R62_KN276119.1:997854-996922
ATGCTCCCCTTACCCGTGCTGATTTTCATGGCCCTCTTTCTCCTGGAGTCGCTGGCTGCAATGTTACAGAATGGCTTCAT
GGTTACTGTGCTGAGCAAGGAGTGGGTGCGATGTCGGACACTGCCCACAGGTGACATGATTGTAGCCTGCCTGGCTGCTT
CCCGGTTCTGCCTGCATGGGATGGCCCTCCTGAACAACTTCCTGTCTTCCTTTGGTTTTTATACCGAAACTAAATATTTC
AGCATCCCCTGGCAGTTCATCAACTCTCTCACTTTCTGGCTTACTGCCTGGCTTGCTCTCTTCTATTGTGTGAAGATCTC
ATTCTTCTCGCACCGCATCTTCTTCTGGCTGAAGTGGAGGGTTTCTCGGTCAGTGCCTAGGCTGCTGCTGGGCTCCCTGG
TCCTGTCTGGTCTGGCAGCCGTCTCATCAGTCATTAGGAATATGATTCTAACGCGGAGGACTGAGACCCAGAGTTCCCAG
GGAAACAACACCCTGGCTAGTAGACTACAAACCGTCTCTTCATACATTTTTCTATCTCATATGATTCTTATGTGGTTGAT
TCCATTCCTCCTATTCCTGGTGTCCATCCTCTTGCTCATGTTCTCACTTTGCCGGCACTTGAGGCAGATGACGGACCATA
GACCCGGCCCGAGTGATCCCAGCACACAGGCTCACACTGTCGCCCTGAAGTCGCTTGCCTTCTTCCTCATCTTCTACACA
TCATACTTCCTGTCCCTGGTTATTGCTGTTAGTAACATCAAAGCCCTCCAAAGTCACTGGCACTGGGCCTGGGAAGTGGT
GATCTACGCAGGCATCTGTCTGCACTCCAGCATCCTGGTGCTAAGCAGCCCCAAGCTGAGAAAGGTCCTAAAGAAGAGGC
TTTGGAGAGCCCTGGGCGAAGAGCAGTTTGTCTCGAGTTACCAGTATCAGTAA
>CabaTAS2R67_KN276461.1:1624717-1623776
ATGCCATCTGGAATTGATAATGCTCTTCTGGCAGCCATAATAGGAGAAGTCATAATTGGAACGTCGGGGAATGGGTTCAT
TGTACTAGTTAACTGCATTGGCTGGGTGAAGAGGAACAAGTTCTCATCAGTCGACTGCATCCTCACCGGCCTGGCTATCT
CCAGAATCAGTCAGCTTTGGATAACGCTGTTTGAATCATTTTTAATGTTGTTTTGGTCACATCTCTATGCCACTGATAAA
CATTTAATAAGTATTGTTGGCATTTTTTGGACATTGTCCAATCACCTAGCTACCTGGTGCGCTACCTGCCTAAGTGTTTT
CTACTTCTTACAAATAGCCAGTTTCTCCCACCCCTGCTTCACCTGGCTGCGCTGGCGAATTCACAGGGTGGTACTTGTCC
TTCTGCTGGGGTCTTTGTTCTTACTGGTTTTCAACTCTAAATTAATACATTCGTTTGGTGAATCCTGGACTAGTATCTAT
AAAATAGATCAAAGAAACTCAACTGCGTCCTCGGGCAAAATGAAAACTCTGTATCTTAATGGGTTGATTGTTTACAGCTT
GATTTGCTTAATGCCCTTTCTTGTGTCCGTGACCTCACTGCTTCTTTTATTCATCTCCTTGAGAAGACACACCAAGATTT
TGAAGCTGAACCCCAGCTCTAGGGACTTCAGCACAGAGGCCCATAGAAGGGCCATGAGAATGATAATTTCTTTCCTCCTA
CTCTTCTTGGTCCACTTTTCTTCCATTCTATCGGTGGGCTGGGCATTCATTATACTGCAGAAGCATCAGGTCAACTTGGT
TGTCATGTTAACTTCAATTGTTTTTCCTTCAGGCCACTCATTTACCCTAATTTTGGGAAACAGCAAGCTGAGACAAAATG
CCTTAGGACTACTGTGGTATCTTAATTGTCACCTGAAAAGAGTGAAACCTTTAGCTTCATAG
>CabaTAS2R4P_KN276478.1:460921-460083
ATGCGTCAGGCATTCTTTTTTTCTTCTCTTATTATCTCAGCAATTGTGACTTCTGCTGGAGTCATTGCAAATCTGTTCAT
TGCAGTGATCAATTACAAGAATTGGGTCAAAAGCCACAAGATCTCCTCTTCTGATAGAATCCTGTTCAGCTTGGGCATCA
CCCGAGTTCTCATCCTGGGACTGAGTACTATTTTCTCCATCTCTTCAAGTATTCGAAGGTCAGTCTCCATATCCACTTAT
TTTCTGTCATGTTGGTTGTTTTTGGACTCTAATAGTCTTTGGTTTGTAACCTTGCTCAACGCCTTGTACTGTTTCTCTCC
CCCAAAATGCCCAGGCTGCTGCTGGCCTGTGTGCTGATTTCTGCCTTTACCACTCTCCTGCATGTTGTGCTCCGAGAAGC
ATCACGCTCTCTGGAATTGGTGACCGGGAGAAATGGCACCGTCTGTGACGTCAATGAGGAAATCTTCCTTTTGGTGAGCC
CTTGGCTCTTGAGCTCATGTCTACAGTTCTCCCTTAATGTGACTTCTGCTTCCTTGTTAATAAATTCCTTGAGGAGACAC
ATCCAGAAGATGCAGAGAAGTGCCACTGCTCTTTGGACTCCCCAGACCGAGGCTCATGTGGGTGCTATGAAGCTGATGAT
CTGTTTCCTCATACTCTACGTTCCGTATTCAGTTGCTTCCCTGCTCCATTATCTCCCTTCTGTAGCGAGTGATTTGAGAC
TCAAGTCCATTTATATTATTATTTCCACCTTTTACCCTCCAGCACATTCTGTTCTCATTATTCTCACACATCCTAAACTG
AAAACAAAAGCAAAGAAGATTCTTCACTTCAATAAATAG
>CabaTAS2R5P_KN276478.1:451726-450834
ATGCTGCCCGCTGTCCTAGGACTGCTGGTGCTGGTGGCAGTGGCTGAATCTCTCACTGGCCTGGTTGGAAATGGAGTCCT
TGTGGTCTGGCATTTTGGGGAATGGGTCAGAAAACTAGAGGGGTCCTCATATAACCCCATTGTCCTGGGCCCGGCTGTCT

GCTGATTTCTCCTGCAGTGCTTGATTGTGGTGGACTTAAGTCTGTTTCCGCTTTTGCAGGGCAGCCGTTGGCTTCACTGT
CTCAGTGTCTTCTGGGTCCTGATAAGCCAGGCCAGCCTGTGGTTTGCCACTTTCCTCAGTATCTTCTACTGTAGGAAGAT
CACAACCTTTGAACACCCTGTCTACTTGTGGCTGAAGCAGAGGGCCTGTTGCCTGAGTCGCCGGTGCCTTCTGGTGTACT
GCACAGTTTGTTACTTACATTTCACGGTGGCTTAGAGCTCTGCCATTCTTCCAAAGGAAACAGCATTTTATTCCCCCTTT
CAGACTGGCACCATGTGTGTACATTACAGTTCAGTGCAGGAAGTGTGTTGCCTTTCATGATGTTTCTTATTTCCTCTGGG
ATGCCGATGGCCTCTTTGTGTAGACACCACAGGAAGATGAAGGTCCACACAGCCAGCAGGAGAGATCTTCAGGCCACGGC
TCACATCACTGTGCTGAAGTCCTTGGGCTGTTTCCTCATACTTTACATAGTTTACTTTGTGGCCATCCCCTTCTCCATCA
CCTCCAAGTCTTTTCCTGCTAATTTCGCCACTCTGTTCATCTCTGAGACACTCGTGGCTGCCTACACTTCTCTCCACTCT
GTCATCTGGATCATGGGGAATCCCAGGTTGAAGCAGACTTGCCAGAGAATCCTGTGGAAGACAGTGTATGCTAGGAGATC
CTGGGGCCTGTGA
>CabaTAS2R13P_KN276461.1:1599354-1598431
ATGGTAAATGTCTTGAATATCATCTTCATCATTTTAATACACTCAGAATTAGTAACTGGTATTTTGGGGAATGGATTCAT
AACACTGGTGAACTGCATTGACTGGCTCAAGACTTGAAGGATCTCCTCAGCTGATCGAATCCTCACCACGTTGGCAATCT
TTAGAATTTGTCTGATTTTGGTAATAGTGGTGAGTTGGTTTACAAGGGAATTTTCTCCATCTTTATATTTGAATAGAAAG
AATGTTATACTTATTACTATTATTTATTACCATTGCATGGACCTTGGCCAACCATTTTAGCACCTGGCTTGCCACAGACC
TCAGCCTCTTTTATTCTCTCAAGATAGCCAATTATACAAATGCTGTTTTTCTTCACTTAAAGCATAGAGTTGAAATGGTA
GTTCTGGTAATGTTTCTGGGGGCATTAGTATTGTTGCCTTTATGTCTTATTATGATAAGTATTCTTATTAATATCCAGAT
ACATCCATATGAAAGAAATGTGACTTTGAGTTCTAAAAGGAGTGACACGGAAAACTTTTTGAAACTGATTATATTCAGTA
TGGGAGACTTCATACCCTTTATTATATCCCTGATGTTTTTCCTCCTGCTAATCTCCTTATTGAAACACCTCAAGAAGATG
AAGCACCATGCAATGGGATTCAGAGATCCCAGCATCAAAGCCCACCTCAGTGCCATGAAAATGGTGATATCTTTCCTCAT
GCTATTTGCTGTTTACTTCCTGTCTATTCTTATGACAGCCTTCCATTCTGATATGACTCAGAACAAATCGACCCTTATGC
TTGGTCAGGTTCTTGCAAATGCTTATCCTTCAGTCCACTCATTTGTCCTAATTCTGGGAAACAGTGAGCTATGGAGGGCT
TCACTTTCGGTGTTGTGGCAGCTGAAATGCAGTTTAAAAGCTGG
>CabaTAS2R16P_KN276478.1:14020565-14021468
ATGATACCCATCCAACTCACTGTCTTCTGCATAATCGTCTACATGCTTGAGTCCTTGATAATAATTGTGCAGAGCAGCTT
AACTGCTGTGGTGCTGGGCAGAGAGTGGGCGCAGGTTAAAAGGCTGTCACCTGTGGACAAGATTCTCACTGTTCTGGGCA
TCTGCCGCTTCTGTCAACAGTGGTCATCGATGCTGTTCAATTTCTGCTCCTACCTCCACCCTAACTATGTATTTTGGTAC
TTGGCAATCGTCTGGGAATTTACTAACACTTTCGTTCTGGTTAACCTCCTTGCTTGCTGTCTTCTACTGTATCAAAGTCT
CTTCCTTCAGCCATCCCATCTTCCTCTGTCTGAAGCGGAGAATTGTGAAGTTGGTTCCTCGGCTGCTGCTGGGTTGCCTG
CTGATTTCTTGTCTGTCAATCATCTTTTCAGCTACGAGGCATCGCATCACGATTCAGTTAAAGTCTATGAGGCACTTTGC
TAGAAACAGCACTGTGATTGAAAGACTTGAGACATTTCAGCGGGATTTTTCCATATATCAGCAAGTGGCAGTGTTGGTTA
TTCCTTTCCTCCTGTTCCTGGTCTCCGTTGTCTTGCTCATGACCTTATTGTCCCAACATCTGAGGCAGATGAAACATGGT
CACACTGGGCACTCCAGCTCCAGCCTGAAAGCTCACGTTACTGCCCTGAGGTCTCTGGCCATCTTCCTCATCATCTTCAC
ATCTTATTTTCTGCCCATACTCATCTCCATCATAGGTACCCTCCTGGATAAGAGGTCCTGGTTCTGGGCCTGGGAAGCTG
TCATCTATGCTGTAGTCTCTATTCATTCCATTTACTGATGCTGACCAGCCCTAAATTGAAAAAGGCCTTACCAGTAAGA
TGCTAGGACTTAGAGGCTGCCTGA
>CabaTAS2R40P_KN276119.1:1175623-1174647
ATGGCCACAGTGAACACAGATGCCATGGATAAAGACACAACCACATTTAAAATCATCTTCATCTTGGCGGTCTCCAGAAC
AGAGTGCATCACTGGCATCGCTGGGAACGGCTTCATCACGGCCATCCATGGGGCCGAGTGGGTCAGCGGCAAAGGACTGC
CTGCTGGTGACTGCATTCTGTTGATGCTGATCTTTTCCAGGCTCTTGCTACGGGTTTGGATGATGCCAGAGAACACTTAC
AGTCTACTCTGCCCGGTCACTTATAACCAAAATGCAGTGCATACACTTTTCAAAGCCATCATCATGTTTCTGAACTATTC
CAACCATTGGCTTGCCACATGGCTCAATATCTTCCATTGTCTTAGAATCACAAACTTCACCCACCCTTTGTTCTCCGTGA
TGAAGAGGAAAATCATGGTGCTGATACCCTGACTTGTGAGGCTCTCACTGTTAATCTCCTTATGCTCCAGCTTCCCCTTC
TCTGTAGATATCCTCAGTGTGTCTGTGAATAGTTCTGTTCCTATTCCTTCCTCCAGCTTCCCTGAGAAGGTGTACATGTC

CAAGACCAACGTGGTCAACTTGGTTCTCACCCCTTACCTGGGGGTCTTCATTCCTCTGATCATGTTCATCCTTGCAGCCA
CCCTACTGATCATCTCTCTCAAGAGACACACCCTACACATGGCCAGCAATTCCACAGGGCTCCAGGGACCGACCCCAGCA
TGGAGGCTCACATGGGAGCCCTCAAAGCTATCAGCTCTTTTCTCATTTTCTACATTTTCAGTGCCGTTGCTCTATTTCTT
TCCATGTCCAACATCTTTGATGTCAAAAGTTCCTGGAGCATTTTGTGCAAAACCGTCATGGCTGCCTACCCATCCAGCCA
CTCAGTGCTATTGACCTTGGGCAACCCTGGGCTGAAAAGAGTGTGGAAGCAGTTTCAGCACCCAGTTCATCTTTGCCTGT
AGGGACAGACTCTGTGA
>CabaTAS2R42P_KN276461.1:1631964-1631032
ATGTTCAGTGGGTTGAGTGCAATCTTCCTGATGCTGTCAGTAGTGGAATTCGTAATCGGAATGTTGGGGAATGTGTTCAT
CGGACTGGTAAACTGCTCTGAGTGGGTCAAGAACCAAAAGATCTCTTTATTTGACTTTATCATCACCTCCTTGGCTATCT
TCAGAATTGGTCAACTGTTGGTGTTTTTCTTTCAGTCCTTTTTAATGGGACTAGATCCACATTTATTTTTACTCTTAAA
CTAGCAAAAACTATTAGTTTGCTTTGGAGAATCACTAATCACTTGGCTACCTGGCTTGCCACCTGCCTAAGCATTTTCTA
CCTCCTTAAGGTAGCTCACTTCTCCCACTCCCTTTTCCTCTGGCTGAAGTGGAGAATGAACAGAGTCATTCTTGTAATAT
TTGTATTTTCTTTGGTCTCTGTTTTTTGACTTTCTCTTGCTAGAAACATTTAACGATCTCTTCTGGATGACAGCAGATGA
AAGTAATCTGACTTTATATTTAGATGAAAGGAAAATGTTCCACGTACAAAGTGAGATTCTTCTTAGCTTGACCTATCTCA
TTCCTGTTGTTCTGTCCCTGATCTCACTGCTGGTTTTATTTCTGTCCTTGGTGAAACACACCAGAAATTTGCAGCTCCAC
TTTGTGGGCTCGAGGGACCTCAGCACAGAGGCCCATAAAAGAGCCATGAGAATGGTGATGTCTTTCCTCCTCGTCGTCAT
GATTCATTTTTTTTCCATGCAGTTGGCAAATTGGATGTTTTTTATGTTTTTTGATAAGAAGTTCACAAAATTCATCATAT
TGGCAGCATATGTCTTTCCCTCAGGCCATTCGTTTATGTTGATTCTGGGAAACAACAAGCTGAGACGGGGAGCTTTGAAG
GTCTGAGTCATCTTAAAATCTCCTAGAAAAGAGAAAATCCATGACCTTTACAG
>CabaTAS2R408P_KN276461.1:1604891-1603945
ATGATGAATTTACTACCAAGCATTTTTTCTATCCTGTTAATAGCATACCTTATTCTGGGGAATTTTGCCAGTGGCTTCAT
AGCATTGGTGAACTGCACTGACTGGGTCAAGTGACCGAAGATCTCCTCAGCTGATGGAATTCACACTGCTTTGGGACTCT
CCAGAATTGGCTTACTCTGGGTAATATTAATAAATTGATATGCAAATATGTTTAATCCAGATTTAGACAATTTAAGAGCA
AGAATTACTACTATTGCCTGGACAATAGCAATCATTATAGCATCTAGTTTGCTGCTGCCTTCAGCATATTTTATTTGTTC
AAGATAGCCAATTTCTCCAGCCTTATTTTCCTTCACCTAAGGTGAAGAATTAAAAGTGTACTTCTCGTGATAACGTTGGG
TACTTTGTTCTTTTTGGTTTTTCAGTTTGCAGCAGTAAGCATAGATGAAAATATCCAGACAAATGCATATGAAGGAAACA
TCACTTGGAGGACCAAATTGAGGGCCATTTGACACATTTCAAATATGACTCTGTTCATGCTAATAAACTTCATACCCTTT
ACTATGTCCCTGATATCTTTTCTGCTGTGAATCTTTTCCCTATGGAAACAGTTCAAGAAGATGCTGCTCAGTAGGAAAGG
ATCCCAAGATCCCAGCACCAAAGTCTATATAAAAGCCATGCAAACTATCTCTTGTCTCTTGCTATTTGCCCTTTACTTCT
GGGCTCGAATTTTCTCAATTTGGAGTTCCAGTAGGCAGCAGAACAAACTAGTTATGATGGCTTGCCAGGCTTTGGAATCA
TATACCCTGTAAGCCACTCATTTATCCTGATTTGGGGAAACAAGAAGCTAAGACAGGCCTTCCTGTCTTTTCTTTGGCAG
CTGAGGTGCTGGCTAAAAGAAAGGAAATAAGTGGGTGTCATGTATCTTCTAGCAGAAAACAAACTGA
>CabaTAS2R60P_KN276119.1:991960-991015
ATGGTTCCAGGACCTAAGGTGACTGATAAAGAGAGCCATCATCTTCGTTATCATTTTATTCCTTTTGTGCCTGGTGGCAG
TGGTGGGTAACGGTTTAATCACTGCAGCACTAGGCATGGAGTGGTGGCTGCGGAGAACGTTGTCACTCTGCGATAAGTTA
TTGGTCAGCCTGGGGGCCTCTTGCTTCTGTCAGCAATGGGTGGTGATAAGTAAGAACATGTATATTTTCCTGTATCCAAT
GGCCTTCCCACACAACCTTGTATTACAGTTCCTAGCCATTCAGGGGGACTTCTTGAATGCTGTCACGTTGTGGTTCTCCA
CCTGACTCGGTGTCTTCCACTGCATGAGAATTGCAACATTCACCCACCCTGCCTTCCTCTGGCTAAAGAGCAAGTTGTCT
GGGTTTGTCCCTTAGATGCTGCTCAGCTCTGTGCGGTTCTCCAGCATCAGCGCCAGCACCATTCTATTTTTCATAGGCAA
CCAGAGAATATACCAGAACTATTTAAGGAAAGGTCTGCAATCTTGGAATGTCACTGGGAATACTCTGAGGAGAACATATG
AGAGATTGTACTTCTTCCCTCTGAAAATTGTTACCTGGACAGTCCCTACAGTTGTCTTCATCACTGACATGGTTTTGCTC
ATTACATCTCTGGGAAGACACACCAAGAAGGTCTTCCTGTCCATCTCAGGCTTTCCTGATCCCTGTGCCCAGGCACACGT
CAAGGCTCTCCTGGCTCTCATCTCTTCCATCCTCTTCATTCCCTATTTTCTGTCACTGGTGTTCAATGCTGCAGGCATGT
TTCCACTCCAGGAATTCAGGTGCTGGGTGTGGCAGACTCTGATTTATCTGTGCATGGCATTCTACCCCATCATTCTACTC

CTGAGTAACCGCAGGCTGAGAGCTGTGCTGGAGAGGTGCTGCTCCTCAAGGTGTGGGGCATCTTGA
>CabaTAS2R18P_KN276461.1:1610590-1609751
ATGTCAGTGGGAATGAAAGTCTCCATTCTGGGAGTGGCAACAGGAGAACTGATCTTAGGAATACTGGGAAATGGGCTCAC
TGGACTGGTAGTCTGCATGGAATGGGTCAAGAATGGGAAGGTCTCATCAGCTGATTTCATCCTTACCAGTTTATCTGTGG
CCAGAATCAGTCAGCTGTGGGTAACACTATTGGATTCATTTATAGTATGGCATCTCCACACGTGTATGCCACCGGCAAAC
TAGTGAAAGTGTTCTTTGGGCACTAATGAATCGCTTAACTACCTGGTTTGCCGCCTACCTGAGCATTTTCGTCTTTCTTA
ATGTGATCAGTTTCTCCCAGTTTTTTTCATCTGGCTGAAGTGGAGAATGAACAGAGTGGCTCTTGTGCTTTTCCTGGGCT
CTTTGTTCTTACTGTCTGTTACCCTCCTGATGCGGGACGCTCTTGGTGAGTTGTGGGTGAATAGCTACAGAGGACGTGAG
AGAAACACGACTTTGTATTTAGATGGAAGTAAACTTTTCTGTCTTAAAAGCCTTGTTCTTCTTAGCTTGATCTGTGTTAT
CCTGTTCCTTCTCTCCCTGGCCCCTTTGCTGCTTTTATTTCTCTCCTTGGTGAAGCATACCAAGAATCTATAGCTCCACC
CGAATGAATGGAGAGACCCATAAAAGGGCCATGAAAATGGTGACAACCTTCCTCCTTCTCTTTATCATTTATTTTATTTC
CACTCTAATGGCAAGCTGGATTTTTCTTAAGGTTTTCCCTCAGTTCACTTCTATGAATTTCAGTTTTTGGACTAGCAAGC
TAGGGTAGATTGCCTTGAAACTACTGTGGTACCTTACACTCTCTCTAAAAAAAGCAAAACCTTTAGCTTAATAG
>CafeTAS2R1_KB019481.1:2759797-2758910
ATGCTGGAGTCACAGCTCATTAGCCATCTTCTCTTGACAGCGATACAGCTTCTCACTGGGCTTTTTGTAAATGCCATCAT
TGTGGTTGTGCACGGCACGGGCTTGCTCAAGCACAGAAAGATGATTCCGTTGGAGGCCCTTGTTTCCTGCCTGGCGATGT
CCAGGATCTGTCTAGAGCTGGCCATCTTCTACCTTAATCTGGCTGTTTTTAACTTGATTGACATCCGTCAGTTTGTGGGG
AAGTTTGTAATTCTCTCCTTTGTAAATGAATTGGGGCTGTGGTTTGCCACATGGCTCAGCGTCTTCTACTGCATCAAGAT
TGCCAGGATCGCTCACCCTCTCTTCCTCTGGTTGAAGCTGAAGATCTCCAAGCTGGTTCCTTGGCTGATTCTGGGGTCCC
TCCTACATGCATCTAGCACCTGTGTCTTCCACAGCAAACAGCCATGGATCTTTTCCGAAGACCTCCTGGGCTTTTCCTCT
CAAAATGCAACAGCTGAAAGCGAAGACATCGCTACTTTGCGGTTCACCCTTCTCTTCGCTGAGCTCTCCTTGCCATCACT
CATCTTCCTCGTTTCTGCCCTGCTCTTGCTGTTCTCCCTGGGGAGACACACCTGGCAGATGAGAAACACAGCCGCAGGCC
CTTGCCCCCGTGCGCGCGTCAGGTCCCTCCTGTCCATCTTCGCCTTCCTGGTCCTCTACCTCTGCCACTACCTGATGGTT
CTTTTGCTCTTTTCTCAAGTTTTCAAACTTACAAGCCTCAGATTTCTGTTCTGCATCTCGCTGGTTGGTTCCTGCCACTC
CGGACACTCTATTATCTTAATTTTAGGAAATCCTAAACTGAAACAAAATGCGGAGAAGTTTCTCCTGCATAGAAAGTGCC
GTCAGTGA
>CafeTAS2R2_KB018466.1:580588-579659
ATGGCTTCCCCTTGGTCGGCTATTCCTCATGTTATCATCATGTCAGCAGAATTTGTCACAGGGATTACAGTAAATGGATT
TCTTATAATCATCAATTGTAACGAATTGGTCAAAAGCAGAAAGCTAACACCAATACAGCTTCTTTTTATATGTATAGGAA
TGTCTAGATTTGGTCTACAAGTCGTGTTAATGGTACAAAGTTTTTTTCTGTGTTCTTTCCACTCTTTTATAAAGTAAAA
ATTTATGGCACAGCGATGATATTTTTTTGGATGTTTTTTAGCTCTGTCAGTCTCTGGTTTGCCACCTGTCTCTCTGTATT
TTACTGCCTCAAGATATCAGGCTTCACCCAGTCCCATTTTCTTTGGCTGAAATTCAGGATCTCAAAGTTAATGCCTTGGC
TACTTCTGGGAAGTCTGCTGTCGTCTGTGAGCATTGCAACTCTGTGCATCAAGGTGGATTACCCTAAAAACTTGGATAGT
GATGTCCTCAGGAATGCCACGCTAAAGAGGACTAAACTCAAGACAAAGCAAATTAATGAACTGTTTCTTGTCAACTTGGC
ATTAGTACTTCCTCTAGCCATATTTGTGATGTGTACTGTTACGTTATTCATTTCTCTCTGCAAGCATACTCATCGGATGC
AAAACGGATCTCATGGTTTTAGAAATGCCAGCACAGAAGCCCATATTAATGCATTAAGAACAGTGATAACATTCTTCTGC
TTCTTTGTTTCTTATTTTGCTGCCTTCATGGCAAATGTAACATTCAATATTCCTCACGGAAATCAGTGGTTCTTTGTGGT
GAAGGACATAATGGCAGCGTATCCCTCTGGCCACTCAATTATAATGATCTTGAGTAATTCTAAGTTTCAACAACCATTCA
GGAGACTTATCTGCCTTGAAAAGAATCAAAGAAGAGGAGGACCAATGTAA
>CafeTAS2R3_KB017062.1:2514019-2514969
ATGCTGGGATGCACCGAGTGGGGCTTTCTGGTTCTGACTGCCAGTGAGTTCGTCCTGGGAGTGCTGGGCAATGGCTTCAT
AGGGCTAGTCAATGGCAGCAGCTGGTTCAAGAGCAAGAGAATCTCTTTGTCTGACTTCATCATCACTAACCTGGCTCTCT
CTAGGACTGTTCTGCTGGGGACTCTCTTGGTTGATGGTGTTTTAATGGTGTTCTCGTCCAAACTACATGATGAAGGGATA
GTAATGCAGATGATTGATATTTTCTGGACGTTTACAAACCATCTGAGCATATGGCTTTCCACGTGTCTCAGTGTCTTCTA

CTGCCTGAAAGTCGCCAGTTTCTCCCACCCTGCATTCCTCTGGCTCAAGTGGAGAGTTTCCAGAGTGGTCGTGTGGATGC
TGATGAGTACCCTGCTCCTAGCATGTGGCAGCACCGTGTCTCTGGTCCATGAATTTAAGATCTATTCTGTTCTCAGTGGA
ATTGATGGAACAGGAAATATGACTGAGCAGGTTTTAAAGAAGAGAAACGAATACAAACTGATCCATGTTTTGGGGACTCT
GTGGAACCTCCTTCCCCTAACTGTGTCTCTAGCCTCCTACTTTCTGCTCATCTGCTCCCTGGGAAGGCATACGCGGCAGA
TGCAGCGGAACTGTACCGGCTCCAGGGATCCAAGTACCGAGGCCCACTATAGGGCCATCAAAGTCATCCTCTCCTTCCTC
TTTCTCTTCCTACTTTACTTTCTTTCCTTTTTAATTTTATCATCTAGTTATTTCCTACCAGCAACTAAAATGATTAAGAT
GATTGGAGAAGTGATTACAATGTTATATCCTGCTGGCCACTCATATATTCTCATTCTGGGAAACAGCAAGCTGAAGCAGA
CGTTTATGGAGATGCTCTGGTGTGGGCATGGTGATCTGAAGCCTGGATCCAAGGGCCCTTTTTCTCCATAG
>CafeTAS2R4_KB017062.1:2523808-2524695
ATGCGTCAGGCATTCTTTTTTTCTTCTCTTATTATCTCAGCAATTGTGACTTCTGTTGGAGTCATTGCAAATCTGTTCAT
TGCAGTGATCAATTACAAGAATTGGGTCAAAAGCCACAAGATCTCCTCTTCTGATAGAATCCTGTTCAGCTTGGGCATCA
CCCGAGTTCTCATCCTGGGACTGAGTACTATTTTCTCCATCTCTTCAAGTATTCGAAGGTCAGTCTCCATATCCACTTAT
TTTCTGTCGTGTTGGTTGTTTTTGGACTCTAATAGTCTTTGGTTTGTAACCTTGCTCAACGCCTTGTACTGTGTGAAGAT
TACTAACTACCAACACCCAGTTTTTCTCCTGCTGAAACGAAATCTCTCCCCCAAAATGCCCAGGCTGCTGCTGGCCTGTG
TGCTGATTTCTGCCTTTACCACTCTCCTGCATGTTGTGCTCCGAGAAGCATCACGCTCTCTGGAATTGGTGACCGGGAGA
AATGGCACCGTCTGTGACGTCAATGAGGAAATCTTCCTTTTGGTGAGCCCTTGGCTCTTGAGCTCATGTCTACAGTTCTC
CCTTAATGTGACTTCTGCTTCCTTGTTAATAAATTCCTTGAGGAGACACATCCAGAAGATGCAGAGAAGTGCCACTGCTC
TTTGGACTCCCCAGACCGAGGCTCATGTGGGTGCTATGAAGCTGATGATCTGTTTCCTCATACTCTACGTTCCGTATTCA
GTTGCTTCCCTGCTCCATTATCTCCCTTCTGTAGCGAGTGATTTGAGACTCAAGTCCATTTATATTATTATTTCCACCTT
TTACCCTCCAGCACATTCTGTTCTCATTATTCTCACACATCCTAAACTGAAAACAAAAGCAAAGAAGATTCTTCACTTCA
ATAAATAG
>CafeTAS2R7_KB017817.1:5829934-5830869
ATGCCAGGTAAGGTGGAGAACACCTTAATCCTCATAGCAGTAGGAGAATTTTCGCTGGGGATCTTAGGGAATGTGTTCAT
TGGATTGGTAAACTTTGTGGACTGGATCAAGCATAGGAAGATCGCCTCCATTGATTTAATCCTCACAAGTCTGGCCATAT
CCAGAATTTCTCTATTGTGTATAATACTATTAGACTGTTTTATACTGGTTCAGTATCCAGACGTCTATACTGCCGGTAAA
CAAATGAGAATCATTGACTGCTTCTGGACACTAACCAACCATTTAAGTGTCTGGTTTGCCACCTGCCTGAGCATCTTCTA
TTTCCTCAAGATAGCTAATTTCTTCCATCCCCTTTTCCTCTGGATGAAGCTGAGAATTGACAATGCGATTCCTAAGATCC
TGCTGGGGTGCTTGGCCCTCTCTGTGTTTTTTAGCCTTCCTGTCTCTGGGAATTTGAATGATGATTTCAGGTTTTGTGTC
AAGGCAAAGTTGAAAACAAACTTAACTTTGAGATGCAAAATAAATAAAGCGCAATATGCTTCCACCAAGATATATCTCAA
CCTGCTAACACTACTCCCCTTTTCTGTGTCCCTGATCTCGTTTCTCCTCTTGATTCTCTCCCTGTGGAGACACACCCGGC
GAATGCAGCTCAATGCCACAGGGTCCAGAGACCCCAGCATAGAAGCCCATGTGGGAGCCATGAAGGCGGTCATCTCCTTC
CTCCTCCTTTTCATTGCCTACTACTTGGCCTATCTTGTAGCCACCTCCAGCTACTTTCTACCAGAGACTGAATTAGCTGT
GATGGTTGGTGAGGTGACAGCTCTAATCTGTCCCTCAAGCCATTCACTTATCCTAATTCTGGAGAACAGTAAATTAAGAA
AAGCATTTCTAAGGGTTCTATGGAAAGTAAAGTATGTCCTAAAAAGGAGTTGCTAA
>CafeTAS2R10_KB017817.1:5820932-5821861
ATGCTAAGTGTAGGAGAAGGCCTCCTTCTTTTTGTAGCAGTTAGTGAGTCAGTACTGGGGGTTTTAGGGAACGGATTTAT
TGGACTTGTAAACTGCACTGACTGTGTGAGAAACAAGAAATTCTCTATGATCAGCTTTATTCTCACTGGCTTAGCTACTT
CGAGAATTGGTCTGTTATGGTTAATAATTACAGACGGATTTGTAAGGATATTCTTTCCAGAAATGTATTCCTCCGGTAAC
CTGGTTGACTATATTAGTTACTCATGGATAATTCTGAATCAATTAAGTGTCTGTCTTGCCACCAGCCTCAGTGTCTTCTA
TTTCCTGAAGATAGCAAAATTTTCCCACCACATTTTTCTCTGGTTGAAGAGAAGACTCAACAGGGTTCTTCTCATTCCAA
TGGGATTATTGCTAATTTCATGGTTATTTACTTTTCCACAGATGGTGAGGATTATTAGTGATAATAGAATAAGAAATGGA
AGTACAACCGGGGTAACCAACATGCACAAAGATAAATTCCTTACATACCAGATTTCTCTCAATCTGGGGACCATTCTCCC
CTTTCTACTCTGCCTGATTACATGTTTCCTATTGATCATTTCCCTTTGGAGGCACAACAGGAAGATGAAATTGAATGCCA
CAGGATTCAGGGACCCCAGCACAGAAGCACATATCAAAGCAATGAAAGTCTTGATATCTTTTGTCATCTTGTTTTTCTTA

TATTTTGTAGGCGTTGCTATAGAAACATATCACTATACTCAGCCAGAAAACAAAGTGCTGTTTATTTTTGGTATGGCAAC
CACAGCCATCTATCCCTGGGGTCACTCATTTATCCTAATTCTAGGAAACCGCAAACTGAAGCAAGCCTCTTTGAAGGTAC
TGAAGCATGTAAAGTGCTGGGAAAGAGAGAAACTTCTCAGAATTCCATGA

>CafeTAS2R38_KB017062.1:2623155-2622148

ATGTTGACCCCGGCTTCCATCGTGACTGTGTCCTACGAAGTCAAGAATGCTTTTCTGTTCCTTTCAGTCCTGGAGTTTGC
AGTAGGGATCCTGGTCAATGCCTTCATCTTCTTGGTGAATTTTTGGGATGTGGTGAGGAGGCAGCCACTGAGCAGCTGTG
ATCTCATCCTTCTGAGCCTCAGCCTCACCCGGCTGCTCCTGCATGGGCTGCTGTTTCTGGACGCCGTTCAGCTGGCCTGT
TTCCAGCGGATGAAAGACCCGCTGAGCGTCAGCTACCAAACCATCATCCTGCTCTGGATGATCGTGAACCAGGCTGGCCT
CTGGCTCACCACCTGCCTTAGCCTCCTCTACTGCGCCAAGATCGCCCGTTTCTCTCCCGCCTTCCCGCTCTGCTTGGCGA
GCTGGGTCCCCAGGAAGATGCCCCAGATGCTCCTGGGCGCCATTACCTTCACCTGCGTCTGCGCCACTCTCTGTCTGGGG
GACTTTTTCAGGAGATCTCACTTCTCAGTCACAGTTATGTCTTTCGTAAATAACAGTACAGAGTTCGATCCGCAAATTGC
AAAACTTAGTTTCTTCCATTCCTTCATCTTCTGCAGTGTGGGGTCCATCCCTTCTTTCCTAATTTTTCTGGTTTCTTCTG
GGGTGCTGATTGTCTCCCTGGGAAGGCACATGAGGACGATGAGGGAACAAACCGAAGACTCTCGGGACCTCAGCCTGGAG
GCCCATATCAAAGCTCTCCGATCTCTCATCTCCTTCCTCTGCCTGTACCTGCTGTCACTCTGCGCCGCCCACCTCTCCGT
GCCGTTGCTGATGCTGTGGCACAGCAAGGTCGGGGTGATGGTCTGCGTAGGCATCCTGGCAGCCTGCCCCTCGGGACATG
CAGTCATCCTGATCTCAAGCAACGCCAAGCTGAGGAGAGCCCTGGAGAGCATTGCGCTGTGGGCTCAGAGCAGCCGAAAG
GTCAGGGCGGGCCACGGGGCCGCTCGCGGGATGCAGACTCTGTGCTGA

>CafeTAS2R39_KB017062.1:3322834-3323808

ATGACCAAAACCTGCAATCTCACGGAAAATGAATTTTCACCATTTCTCCTCATTTTAACTTGTATAATTATAGGAAGTGA
ATGCATCATTGGTATCATTACAAATGGGTTCATTGTGGCTATAAATACAGCTGAATGGATGCAGACGAAAGCAGTTTCCA
CAAGCGGCAAGATCCTGCTTTTCCTGAGTGTGTCCAGAATAGCTCTACAAAGCTTCATAATCCTAGAAATTATCTTCAGT
TCAACACTCCCATATTTTTATAATGAAGAAATTGTATATGACACATTCAAAGGAAGTTTCGTGTTCTTAAATTACTGTGG
CCTCTGGTTTGCTGCCTGGCTCAGTTTCTTCTACTTCGTGAAGATTGCTGATTTCTCCTACCCCCTTTTCCTCAAGCTGA
AGTGGAGAATTTCTGGATGGATGTCCTGGCTTCTGTGGCTATCCGTGTTTATTTCCTTGGGCACCTGTGTGTTCTTCCTC
AAAAACATCTACACTGTGCACTGTAACAATTACGTTCCTGTCCCCTCCTCCAACTCCACTAAGAAAAAAGTCTCCACTGA
GACCAACGTGACCAACTTGGCTGTTCTCTTTCAAATGGGGATCTTCATTCCTCTGATCATGTTCATCCTTACAGCCACCC
TGCTGATCGTCTCTCTCAAGAGACACACCCGACACATGTCCAGCAATGCCACAGGTTACAGGGACCCCAGCATGGAGGCT
CACCTGGGGGCCATCAAAGCCACCAGCTACTTTCTCATCCTCTACATTTTCAACTCAGTCGCTCTGTTTCTCTATATGTC
CAACATCTTTGACATCAGCAGTTTCTGGAGTATCTTGTGCAAAGCCATCATGGCCGCCTACCCTGCTGGCCATTCCATTC
TCCTGATTCGGGACAACCCTGGGCTGAGAAGAGCCTGGAAGCGGCTCCAGGCTCGAGTTCATCCGTACAGGGTAGACTCC
ATGACCCACATGTGA

>CafeTAS2R41_KB017062.1:3553642-3554568

ATGCATCCAGCACTCACAGCCCTCTTCATGCTGCTCTTTATCCTGCTGTGTGTCCTGGGAGTCCTGGCCAATGCTTTCAT
TGTGTGGGTTCTGAGCAGAGAGTGGGTGCGACGGGGGAGGCTGCTCCCCTCCGACATGATCCTTATTAGCTTGGGTGCCT
CCCACTTCTGCCTGCAGTCGGTTGGAATGGGGAACAACTTCTACTACTTCCTCCACCTGGTCGAGTACCGTCGGGGTCCT
GCCTGGCAGTTATTTGGTCTATTCTGGGACTTTCTGAACTCAGCCACCTTCTGGTTTGGCTCCTGGCTCAGCGTCCTCTT
CTGCATGAAGGTTGCTAACTTCACCCACCCCACCTTCCTCTGGCTGAAATGGAGGTTCCCAGGGGTAGTGCCCTTGCTTC
TGCTGGGATCTCTCCTCGTTGCCTTCATTGTCACCCTGCTGTTCTTTTGGGGGAACCATGCTCTGTATCGAGGATTCTTT
ATTGAAACATTTTCTGGGAATATGGCCTACAAGGAGTGGAGCAGGAGGCTGGAAATGCACTATTTCCTGCCCCTGAAGCT
AGTCACCCTTTCAATTCCTTGCTCTAGTTTTCTGGTCTCAATTGCGCTGTTGGTAAGTTCTCTGAGGAGACACACGAGGA
GGATGCAGCACAGTGCACACAGCCTGCAGGACCCCAGCGCCCAGGCTCACACCGCAGCTCTGAAGTCACTCATCTCCTTC
CTCATTCTTTACCTTCTGTCCTTCCTGTCCCTGGTTATTGACGCTGTAGTGTTTTTCTCCTCAGAGAGTGAATGGTACTG
GCCATGGCAAATTGTAACCTACTTGTGCACATCTGTCCATCCCTTTATCCTCATCTTCAGCAACCTTAGGCTTCGAGGGG
TGTTCAGGCAGCTAATTCTGTTGGCCAGGGGCTTCTGGGTGGCCTAG

>CafeTAS2R62_KB017062.1:3527256-3528188
ATGCTCCCCTTACCCGTGCTGATTTTCATGGCCGTCTTTCTCCTGGAGTCGCTGGCTGCAATGTTACAGAATGGCTTCAT
GGTTACTGTGCTGAGCAAGGAGTGGGTGCGATGTCGGACACTGCCCACAGGTGACATGATTGTAGCCTGCCTGGCTGCTT
CCCGGTTCTGCCTGCATGGGATGGCCCTCCTGAACAACTTCCTGTCTTCCTTTGGTTTTTATACCGAAACTAAATATTTC
AGCATCCCCTGGCAGTTCATCAACTCTCTCACTTTCTGGCTTACTGCCTGGCTTGCTCTCTTCTATTGTGTGAAGATCTC
ATTCTTCTCGCACCGCATCTTCTTCTGGCTGAAGTGGAGGGTTTCTCGGTCAGTGCCTAGGCTGCTGCTGGGCTCCCTGG
TCCTGTCTGGTCTGGCAGCCGTCTCATCAGTCATTAGGAATATGATTCTAACGCGGAGGACTGAGACCCAGAGTTCCCAG
GGAAACAACACCCTGGCTAGTAGACTACAAACCGTCTCTTCATACATTTTTCTATCTCATATGATTCTTATGTGGTTGAT
TCCATTCCTCCTATTCCTGGTGTCCATCCTCTTGCTCATGTTCTCACTTTGCCGGCACTTGAGGCAGATGACGGACCATA
GACCCGGCCCGAGTGATCCCAGCACACAGGCTCACACTGTCGCCCTGAAGTCGCTTGCCTTCTTCCTCATCTTCTACACA
TCATACTTCCTGTCCCTGGTTATTGCTGTTAGTAACATCAAAGCCCTCCAAAGTCACTGGCACTGGGCCTGGGAAGTGGT
GATCTACGCAGGCATCTGTCTGCACTCCAGCATCCTGGTGCTAAGCAGCCCCAAGCTGAGAAAGGTCCTAAAGAAGAGGC
TTTGGAGAGCCCTGGGCGAAGAGCAGTTTGTCTCGAGTTACCAGTATCAGTAA
>CafeTAS2R67_KB017817.1:5792116-5793057
ATGCCATCTGGAATTGATAATGCTCTTCTGGCAGCCATAATAGGAGAAGTCATAATTGGAACGTCGGGGAATGGGTTCAT
TGTACTAGTTAACTGCATTGGCTGGGTGAAGAGGAACAAGTTCTCATCAGTCGACTGCATCCTCACCGGCCTGGCTATCT
CCAGAATCAGTCAGCTTTGGATAACGCTGTTTGAATCATTTTTAATGTTGTTTTGGTCACATCTCTATGCCACTGATAAA
CATTTAATAAGTATTGTTGGCATTTTTTGGACATTGTCCAATCACCTAGCTACCTGGTGCGCTACCTGCCTAAGTGTTTT
CTACTTCTTACAAATAGCCAGTTTCTCCCACCCCTGCTTCACCTGGCTGCGCTGGCGAATTCACAGGGTGGTACTTGTCC
TTCTGCTGGGGTCTTTGTTCTTACTGGTTTTCAACTCTAAATTAATACATTCGTTTGGTGAATCCTGGACTAGTATCTAT
AAAATAGATCAAAGAAACTCAACTGCGTCCTCGGGCAAAATGAAAACTCTGTATCTTAATGGGTTGATTGTTTACAGCTT
GATTTGCTTAATGCCCTTTCTTGTGTCCGTGACCTCACTGCTTCTTTTATTCATCTCCTTGAGAAGACACACCAAGATTT
TGAAGCTGAACCCCAGCTCTAGGGACTTCAGCACAGAGGCCCATAGAAGGGCCATGAGAATGATAATTTCTTTCCTCCTA
CTCTTCTTGGTCCACTTTTCTTCCATTCTATCGGTGGGCTGGGCATTCATTATACTGCAGAAGCATCAGGTCAACTTGGT
TGTCATGTTAACTTCAATTGTTTTTCCTTCAGGCCACTCATTTACCCTAATTTTGGGAAACAGCAAGCTGAGACAAAATG
CCTTAGGACTACTGTGGTATCTTAATTGTCACCTGAAAAGAGTGAAACCTTTAGCTTCATAG
>CafeTAS2R5P_KB017062.1:2533086-2533978
ATGCTGCCCGCTGTCCTAGGACTGCTGGTGCTGGTGGCAGTGGCTGAATCTCTCACTGGCCTGGTTGGAAATGGAGTCCT
TGTGGTCTGGCATTTTGGGGAATGGGTCAGAAAACTAGAGGGGTCCTCATATAACCCCATTGTCCTGGGCCCGGCTGTCT
GCTGATTTCTCCTGCAGTGCTTGATTGTGGTGGACTTAAGTCTGTTTCCGCTTTTGCAGGGCAGCCGTTGGCTTCACTGT
CTCAGTGTCTTCTGGGTCCTGATAAGCCAGGCCAGCCTGTGGTTTGCCACTTTCCTCAGTATCTTCTACTGTAGGAAGAT
CACAACCTTTGAACACCCTGTCTACTTGTGGCTGAAGCAGAGGGCCTGTTGCCTGAGTCGCCGGTGCCTTCTGGTGTACT
GCACAGTTTGTTACTTACATTTCACGGTGGCTTAGAGCTCTGCCATTCTTCCAAAGGAAACAGCATTTTATTCCCCCTTT
CAGACTGGCACCATGTGTGTACATTACAGTTCAGTGCAGGAAGTGTGTTGCCTTTCATGATGTTTCTTATTTCCTCTGGG
ATGCCGATGGCCTCTTTGTGTAGACACCACAGGAAGATGAAGGTCCACACAGCCAGCAGGAGAGATCTTCAGGCCACGGC
TCACATCACTGTGCTGAAGTCCTTGGGCTGTTTCCTCATACTTTACATAGTTTACTTTGTGGCCATCCCCTTCTCCATCA
CCTCCAAGTCTTTTCCTGCTAATTTCGCCACTCTGTTCATCTCTGAGACACTCGTGGCTGCCTACACTTCTCTCCACTCT
GTCATCTGGATCATGGGGAATCCCAGGTTGAAGCAGACTTGCCAGAGAATCCTGTGGAAGACAGTGTATGCTAGGAGATC
CTGGGGCCTGTGA
>CafeTAS2R11P_KB017230.1:3396133-3396811
AACCAGTCTACTGTGGCTAATAATTGTCAATTTGTTTTTAGTGCTATTCTATCAGGAAACTCTGATACCATGGGGAGAAA
ATATATCTTACTGGCATTTGGATACTGGCCAACCACTTGAGCACTTGGTTTGCTACTTATCTCTCTGTCTTGTATTTCCT
GAAGATCACCAATTTCTCCTATCCCTCTTTCCTTTGGCTAAAATGGAGAATTAACAAGGTAACTGTCATGCTTCCGCTGG
CATCTGTGCCCTTCCTGTTCATGAGCTTTCCTTTGCCATATGGTTTTGATGTCTTCTGGTATTACAGCCTCCCAAAATGT

GAGACAAATATGACTGGGTTATTCAATGTAAGCAAAAATAAAAATTTGAAATCTACGATTACCTTCACAGATTTTTATTG
ATTCACCAGTACAGTTCCCGGAGGTCAGGCACACTCAAATTTTATACATATTTTCTAAAACCCCAGAATTAAAACAACAA
CAACGTTTAAGAACAACTCTCCACGTACCAGTTTGTAAAAGCCTGGCTTGTGGGGAAACTGAATGGACTCATTTTCCTGT
GGGTTCCCAAGAACACTTGCAGCGGCTCTGAACTATTCCCCATCCTACATCTTTCTCTCTTCCCCGCTTTTGTTTTCTCT
TTTCTCCACTTCTCATCACCCTAACTTACTTCAGCCCAA
>CafeTAS2R13P_KB017817.1:5817946-5818850
ATGGTAAATGTCTTGAATATCATCTTCATCATTTTAATACACTCAGAATTAGTAACTGGTATTTTGGGGAATGGATTCAT
AACACTGGTGAACTGCATTGACTGGCTCAAGACTTGAAGGATCTCCTCAGCTGATCGAATCCTCACCACGTTGGCAATCT
TTAGAATTTGTCTGATTTTGGTAATAGTGGTGAGTTGGTTTACAAGGGAATTTTCTCCATCTTTATATTTGAATAGAAAG
AATGTTATACTTATTACTATTATTTATTACCATTGCATGGACCTTGGCCAACCATTTTAGCACCTGGCTTGCCACAGACC
TCAGCCTCTTTTATTCTCTCAAGATAGCCAATTATACAAATGCTGTTTTTCTTCACTTAAAGCATAGAGTTGAAATGGTA
GTTCTGGTAATGTTTCTGGGGGCATTAGTATTGTTGCCTTTATGTCTTATTATGATAAGTATTCTTATTAATATCCAGAT
ACATCCATATGAAAGAAATGTGACTTTGAGTTCTAAAAGGAGTGACACGGAAAACTTTTTGAAACTGATTATATTCAGTA
TGGGAGACTTCATACCCTTTATTATATCCCTGATGTTTTTCCTCCTGCTAATCTCCTTATTGAAACACCTCAAGAAGATG
AAGCACCATGCAATGGGATTCAGAGATCCCAGCATCAAAGCCCACCTCAGTGCCATGAAAATGGTGATATCTTTCCTCAT
GCTATTTGCTGTTTACTTCCTGTCTATTCTTATGACAGCCTTCCATTCTGATATGACTCAGAACAAATCGACCCTTATGC
TTGGTCAGGTTCTTGCAAATGCTTATCCTTCAGTCCACTCATTTGTCCTAATTCTGGGAAACAGTGAGCTATGGAGGGCT
TCACTTTCGGTGTTGTGGCAGCTGA
>CafeTAS2R16P_KB016972.1:576533-577436
ATGATACCCATCCAACTCACTGTCTTCTGCATAATCGTCTACATGCTTGAGTCCTTGATAATAATTGTGCAGAGCAGCTT
AACTGCTGTGGTGCTGGGCAGAGAGTGGGCGCAGGTTAAAAGGCTGTCACCTGTGGACAAGATTCTCACTGTTCTGGGCA
TCTGCCGCTTCTGTCAACAGTGGTCATCGATGCTGTTCAATTTCTGCTCCTACCTCCACCCTAACTATGTATTTTGGTAC
TTGGCAATCGTCTGGGAATTTACTAACACTTTCGTTCTGGTTAACCTCCTTGCTTGCTGTCTTCTACTGTATCAAAGTCT
CTTCCTTCAGCCATCCCATCTTCCTCTGTCTGAAGCGGAGAATTGTGAAGTTGGTTCCTCGGCTGCTGCTGGGTTGCCTG
CTGATTTCTTGTCTGTCAATCATCTTTTCAGCTACGAGGCATCGCATCACGATTCAGTTAAAGTCTATGAGGCACTTTGC
TAGAAACAGCACTGTGATTGAAAGACTTGAGACATTTCAGCGGGATTTTTCCATATATCAGCAAGTGGCGGTGTTGGTTA
TTCCTTTCCTCCTGTTCCTGGTCTCCGTTGTCTTGCTCATGACCTTATTGTCCCAACATCTGAGGCAGATGAAACATGGT
CACACTGGGCACTCCAGCTCCAGCCTGAAAGCTCACGTTACTGCCCTGAGGTCTCTGGCCATCTTCCTCATCATCTTCAC
ATCTTATTTTCTGCCCATACTCATCTCCATCATAGGTACCCTCCTGGATAAGAGGTCCTGGTTCTGGGCCTGGGAAGCTG
TCATCTATGCTGTAGTCTCTATTCATTCCATTTTACTGATGCTGACCAGCCCTAAATTGAAAAAGGCCTTACCAGTAAGA
TGCTAGGACTTAGAGGCTGCCTGA
>CafeTAS2R40P_KB017062.1:3353340-3354301
ATGGCCACAGTGAACACAGATGCCATGGATAAAGACACAACCACATTTAAAATCATCTTCATCTTGGCGGTCTCCAGAAC
AGAGTGCATCACTGGCATCGCTGGGAACGGCTTCATCACGGCCATCCATGGGGCCGAGTGGGTCAGCGGCAAAGGACTGC
CTGCTGGTGACTGCATTCTGTTGATGCTGATCTTTTCCAGGCTCTTGCTACGGGTTTGGATGATGCCAGAGAACACTTAC
AGTCTACTCTGCCCGGTCACTTATAACCAAAATGCAGTGCATACACTTTTCAAAGCCATCATCATGTTTCTGAACTATTC
CAACCATTGGCTTGCCACATGGCTCAATATCTTCCATTGTCTTAGAATCACAAACTTCACCCACCCTTTGTTCTCCGTGA
TGAAGAGGAAAATCATGGTGCTGATACCCTGACTTGTGAGGCTCTCACTGTTAATCTCCTTATGCTCCAGCTTCCCCTTC
TCTGTAGATATCCTCAGTGTGTCTGTGAATAGTTCTGTTCCTATTCCTTCCTCCAGCTTCCCTGAGAAGGTGTACATGTC
CAAGACCAACGTGGTCAACTTGGTTCTCACCCCTTACCTGGGGGTCTTCATTCCTCTGATCATGTTCATCCTTGCAGCCA
CCCTACTGATCATCTCTCTCAAGAGACACACCCTACACATGGCCAGCAATTCCACAGGGCTCCAGGGACCGACCCCAGCA
TGGAGGCTCACATGGGAGCCCTCAAAGCTATCAGCTCTTTTCTCATTTTCTACATTTTCAGTGCCGTTGCTCTATTTCTT
TCCATGTCCAACATCTTTGATGTCAAAAGTTCCTGGAGCATTTTGTGCAAAACCGTCATGGCTGCCTACCCATCCAGCCA
CTCAGTGCTATTGACCTTGGGCAACCCTGGGCTGAAAAGAGTGTGGAAGCAGTTTCAGCACCCAGTTCATCTTTGCCTGT

AG
>CafeTAS2R42P_KB017817.1:5784865-5785798
ATGTTCAGTGGGTTGAGTGCAATCTTCCTGATGCTGTCAGTAGTGGAATTCGTAATCGGAATGTTGGGGAATGTGTTCAT
CGGACTGGTAAACTGCTCTGAGTGGGTCAAGAACCAAAAGATCTCTTTATTTGACTTTATCATCACCTCCTTGGCTATCT
TCAGAATTGGTCAACTGTTGGTGTTTTTCTTTCAGTCCTTTTTAATGGGACTAGATCCACATTTATTTTTTACTCTTAAA
CTAGCAAAAACTATTAGTTTGCTTTGGAGAATCACTAATCACTTGGCTACCTGGCTTGCCACCTGCCTAAGCATTTTCTA
CCTCCTTAAGGTAGCTCACTTCTCCCACTCCCTTTTCCTCTGGCTGAAGTGGAGAATGAACAGAGTCATTCTTGTAATAT
TTGTATTTTCTTTGGTCTCTGTTTTTTGACTTTCTCTTGCTAGAAACATTTAACGATCTCTTCTGGATGACAGCAGATGA
AAGTAATCTGACTTTATATTTAGATGAAAGGAAAATGTTCCACGTACAAAGTGAGATTCTTCTTAGCTTGACCTATCTCA
TTCCTGTTGTTCTGTCCCTGATCTCACTGCTGGTTTTATTTCTGTCCTTGGTGAAACACACCAGAAATTTGCAGCTCCAC
TTTGTGGGCTCGAGGGACCTCAGCACAGAGGCCCATAAAAGAGCCATGAGAATGGTGATGTCTTTCCTCCTCGTCGTCAT
GATTCATTTTTTTTCCATGCAGTTGGCAAATTGGATGTTTTTTATGTTTTTTGATAAGAAGTTCACAAAATTCATCATAT
TGGCAGCATATGTCTTTCCCTCAGGCCATTCGTTTATGTTGATTCTGGGAAACAACAAGCTGAGACGGGGAGCTTTGAAG
GTACTGAGTCATCTTAAAATCTCCTAGAAAAGAGAAAATCCATGACCTTTACAG
>CafeTAS2R408P_KB017817.1:5812422-5813331
ATGATGAATTTACTACCAAGCATTTTTTCTATCCTGTTAATAGCATACCTTATTCTGGGGAATTTTGCCAGTGGCTTCAT
AGCATTGGTGAACTGCACTGACTGGGTCAAGTGACCGAAGATCTCCTCAGCTGATGGAATTCACACTGCTTTGGGACTCT
CCAGAATTGGCTTACTCTGGGTAATATTAATAAATTGATACGCAAATATGTTTAATCCAGATTTAGACAATTTAAGAGCA
AGAATTACTACTATTGCCTGGACAATAGCAATCATTATAGCATCTAGTTTGCTGCTGCCTTCAGCATATTTTATTTGTTC
AAGATAGCCAATTTCTCCAGCCTTATTTTCCTTCACCTAAGGTGAAGAATTAAAAGTGTACTTCTCGTGATAACGTTGGG
TACTTTGTTCTTTTTGGTTTTTCAGTTTGCAGCAGTAAGCATAGATGAAAATATCCAGACAAATGCATATGAAGGAAACA
TCACTTGGAGGACCAAATTGAGGGCCATTTGACACATTTCAAATATGACTCTGTTCATGCTAATAAACTTCATACCCTTT
ACTATGTCCCTGATATCTTTTCTGCTGTGAATCTTTTCCCTATGGAAACAGTTCAAGAAGATGCTGCTCAGTAGGAAAGG
ATCCCAAGATCCCAGCACCAAAGTCTATATAAAAGCCATGCAAACTATCTCTTGTCTCTTGCTATTTGCCCTTTACTTCT
GGGCTCGAATTTTCTCAATTTGGAGTTCCAGTAGGCAGCAGAACAAACTAGTTATGATGGCTTGCCAGGCTTTGGAATCA
TATACCCTGTAAGCCACTCATTTATCCTGATTTGGGGAAACAAGAAGCTAAGACAGGCCTTCCTGTCTTTTCTTTGGCAG
CTGAGGTGCTGGCTAAAAGAAAGGAAATAA
>CafeTAS2R60P_KB017062.1:3533107-3534067
ATGGGTGGAGAGGACATGGTTCCAGGACCTAAGGTGACTGATAAAGAGAGCCATCATCTTCGTTATCATTTTATTCCTTT
TGTGCCTGGTGGCAGTGGTGGGTAACGGTTTAATCACTGCAGCACTAGGCATGGAGTGGTGGCTGCGGAGAACGTTGTCA
CTCTGCGATAAGTTATTGGTCAGCCTGGGGGCCTCTTGCTTCTGTCAGCAATGGGTGGTGATAAGTAAGAACATGTATAT
TTTCCTGTATCCAATGGCCTTCCCACACAACCTTGTATTACAGTTCCTAGCCATTCAGGGGGACTTCTTGAATGCTGTCA
CGTTGTGGTTCTCCACCTGACTCGGTGTCTTCCACTGCATGAGAATTGCAACATTCACCCACCCTGCCTTCCTCTGGCTA
AAGAGCAAGTTGTCTGGGTTTGTCCCTTAGATGCTGCTCAGCTCTGTGCGGTTCTCCAGCATCAGCGCCAGCACCATTCT
ATTTTTCATAGGCAACCAGAGAATATACCAGAACTATTTAAGGAAAGGTCTGCAATCTTGGAATGTCACTGGGAATACTC
TGAGGAGAACATATGAGAGATTGTACTTCTTCCCTCTGAAAATTGTTACCTGGACAGTCCCTACAGTTGTCTTCATCACT
GACATGGTTTTGCTCATTACATCTCTGGGAAGACACACCAAGAAGGTCTTCCTGTCCATCTCAGGCTTTCCTGATCCCTG
TGCCCAGGCACACGTCAAGGCTCTCCTGGCTCTCATCTCTTCCATCCTCTTCATTCCCTATTTTCTGTCACTGGTGTTCA
ATGCTGCAGGCATGTTTCCACTCCAGGAATTCAGGTGCTGGGTGTGGCAGACTCTGATTTATCTGTGCATGGCATTCTAC
CCCATCATTCTACTCCTGAGTAACCGCAGGCTGAGAGCTGTGCTGGAGAGGTGCTGCTCCTCAAGGTGTGGGGCATCTTG
A
>CafeTAS2R18P_KB017817.1:5806676-5807643
ATGTCAGTGGGAATGAAAGTCTCCATTCTGGGAGTGGCAACAGGAGAACTGATCTTAGGAATACTGGGAAATGGGCTCAC
TGGACTGGTAGTCTGCATGGAATGGGTCAAGAATGGGAAGGTCTCATCAGCTGATTTCATCCTTACCAGTTTATCTGTGG

CCAGAATCAGTCAGCTGTGGGTAACACTATTGGATTCATTTATAGTATGGCATCTCCACACGTGTATGCCACCGGCAAAC
TAGTGAAAGTGTTCTTTGGGCACTAATGAATCGCTTAACTACCTGGTTTGCCGCCTACCTGAGCATTTTCGTCTTTCTTA
ATGTGATCAGTTTCTCCCAGTTTTTTTCATCTGGCTGAAGTGGAGAATGAACAGAGTGGCTCTTGTGCTTTTCCTGGGCT
CTTTGTTCTTACTGTCTGTTACCCTCCTGATGCGGGACGCTCTTGGTGAGTTGTGGGTGAATAGCTACAGAGGACGTGAG
AGAAACACGACTTTGTATTTAGATGGAAGTAAACTTTTCTGTCTTAAAAGCCTTGTTCTTCTTAGCTTGATCTGTGTTAT
CCTGTTCCTTCTCTCCCTGGCCCCTTTGCTGCTTTTATTTCTCTCCTTGGTGAAGCATACCAAGAATCTATAGCTCCACC
CGAATGAATGGAGAGACCCATAAAAGGGCCATGAAAATGGTGACAACCTTCCTCCTTCTCTTTATCATTTATTTTATTTC
CACTCTAATGGCAAGCTGGATTTTTCTTAAGGTACAAAGTTATCAGGGCATGATGTTTTGTCATGGTGATTTCAATTCTC
TTTCCCTCAGTTCACTTCTATGAATTTCAGTTTTTGGACTAGCAAGCTAGGGTAGATTGCCTTGAAACTACTGTGGTACC
TTACACTCTCTCTAAAAAAAGCAAAACCTTTAGCTTAATAGACGTTTTGAAAACATTTTCTGTATTCTATGGGAAAACCC
CTGAATAG
>LiveTAS2R1P_NW_006789455.1:161548-162440
ATGCTGGAGTCTCACCTCATTAGCCACCTTTGTTTGGCAGTGATACAATTTCTCATTGGGGTTTTAGTAAATGGCATCAT
CGTGGTTGTGAATGGCACTCACTTAATCAAGAGAAAGATGATTCCATTGGATCTTCTTGTTTCCTGCCTGGCGATTTCCA
GGATTTGTCTGCAACTAGCCATCTTCTACATTAGCCTGGCTGTTCTTTCCTTGATTGAATTCCCTCAGCTTGCTGAGAAG
TTTGTAATTCTCACATTTATAAATGAATCGGGACTTTGATTTGCCACATGGCTCAGCCTTTTCTACTGTGCCAAGATTGC
CACCATTGCTCACCCACACTTCTGGTTGAAGATGAGGATATCCAAGTTGGTTCCTTGGCTGATACTTGAGTCCCCTCTAT
ATGCATCTAGCATGGCTGCTTTCCACAGCAAACATAGATGGTTATTTTCCAAAGAACACTTCCTGGGCCTTTTCTCCCCA
AGTGCAACCACTCAAATCACAGAAATACCTGCTTACAGTTTGCCTTTCTTTTTGCTGAGTTCTCATTGCCATTACTTATC
TTCCTTATTTCTTCTCTGCTCTTGATATTTTCCCTGGGGAGACACACCTGACAGAAGAGAAACACAGCAACAGGCCCCAG
GAACCCTCACACACGTGTGAACATCAGCACTCTTCTCTCCATCCTGTCCTTTCTGGTCCTCTATCTCTGCCACTCCATGA
CAGCTGCTTTGCTCTTTTCTCAAATTTTCAACTTTAGAAACTTCATATTTCTGTTCTGCATCTTGCGGGTTGGTCCATAC
CACTCTGGACACTCTATTATCTTAATTTTAGGAAATCCTAAAATGAAACAAAATGCAAAGAAATTGCTCCTCCACAGAAA
GTGCTGTCAGTGA
>LiveTAS2R2P_NW_006769399.1:5144-6052
GTGGCCTACTCTTTGTCAGCTCGTCTTCATGTTATCCTCATGTCAGCAGAATTTATCACAGGGATTACAGTAAATGGATT
TCTTATAATCATCAACTGTAATGAATTGGTCAAAAGCAGAAAGCTAACACCAATGCAACTCCTTTTCATATGCATAGGGA
TGTCTAGATTTGGTTTGCAGGTGGTGCTAATGGTAAAGTTTCTTCTCATGTTCTTTTCACTCTTTTATAGAGTAAAAATT
TATGGTACAGCGATGATTATTTTGGATGTTTTTCAGCTCTGTCAGTGTCTGGTTTGCCACCTGACTCTCTGTATTTTACT
GCCTCAAGATAACACACTTCACCCAGTACTGTTTTCTTTGGCTGAAATTCAGGATCTCAAAGTTAATGCCTTGACTGCTT
CTGGGAAGCCTGCTGACCTCCGTGAGCATTGCAACTCTGTGTGTCAAGGTGGATTACCCTAAAAATGTGGATATTGATGT
CCTCAGGGATGCCATGCTAAAGAGGACTAAACTCAAGACAAAGCAGATTAATGAAGTGCTTCTTGTCAACTTGGCATTAA
TATTTCCTCTGGCCATATCTGTGATGTGAACTGTTATGTGTTTCATTTCTCTCTATAAGCATGCTAATTGGATGCAAAAT
GGACCTCTTGGTTTTAGAAACGCCAGCACTGAAGCCCATATTAATACATTAAGATCAGTGATAACATTCTTTTGCTTCTT
TATTTCTTATTTTGCTGTCTTCATGGCAAATATGACATTCAGTATTCCTTATGGGAGTCAGTGTTTCTTTGTGGTGAAGG
ACATAATGGCAGCATATATCCCTCTGGCCATTCGGTTATAATTATCTTGAGTAATTCTAAGTTCCAACAACCAATCAGGT
GACTTCTCTGCCTCAGAAAGAATCAATGA
>LiveTAS2R3P_NW_006793703.1:7411639-7412769
ATGCTGGGACTCACTGAGTGCGGGTTTCTGGTTCTGACTGCCACTCAGTTCATTCTGGGAATGCCGGGGAATAGTTTCAT
GGGTTGGTCAATGGTAGCAGCTGGTTCAAGAACAAGAGAACCTCTTTGTCTGGCTTCATCATCACTAACCGGGGTCTCTC
CAGGATTGTTCTGCTGTGGATTCTCTCTCTCTCTCTTTTTTTTTTTGCGGTACACGGGCCTCTCACTGTTGCGGTCTCTC
CTGTTGCGGAGCACAGGCTCCGGACACGCAGGCTCAGCGGCCATGGCTCACGGGTCCAGCCGCTCTGCGGCATGTGGGAT
CTTCCCGGACCGGGGCACAAACCCGTGTCCCCTGCATCAGCAGGCGGACTCTCAACCACTGCGCCACCAGGGAAGCCCTC
TGCTGTGGATTCTCTTGATTGATGGTGTTCTCTTCCAAACTCCACGATGAATAATTTGCAATCATGGAGATTAGTGATAT

TTTCTGGACATTTACAAACCGTCTGAGCATTTGGCTTGCCACCTGTCTCAGTGTCTTCTACTGCCTGAAAGTCATCAGTT
TCTCCCATCCTACGTTCCTCTGGCTCAAGTGGAGAGTTTCCAGGTTGGTTGTATGGATGCTGTTGAGTACCCTGCTCTTA
TCATGTAGCAGTGCTATCTCTCTGATCCATGAATTTAAGATCTATTCTGTTCTTAGTGGAATTGATGGAACAGGGAATGT
GACCGAACCCTTTAGAAAGAAAAGAAATGAATATAAGCTGATCCATGTTCTTGGGACTCTGGGACCTCCCTCCCTTAATT
GTATCTCTAGCTTCCTACTTTCTGCTCATCCTCTCCTTGGGGAGGCTCCAGAGATCCAAGTACTGAGGCCCACAAGAGGG
CCATCAAAATCATCCTTTCCTTCCTCTTTCTCTTCCTACTTTACTTTCTTTTCAGTTTTGACATCCAGTTATTTCTTCCA
GCAAATGAGGTGATTATGATGACTGGAGAAGTAATTACAATGTTATATCCTGCTGGCCGCTCATATATTCTCATTCTGGG
AAATAATAAGCTGAAGCAGGTGTTCATGGAGATGCTTTGGTGTGAGCCTGGTTGTCTGAAGCCTGGATCCAAGGAACCCG
TTTTTCCATAG
>LiveTAS2R4P_NW_006793703.1:7430229-7431114
ACACTTCAGACATTCTTTTCTTTTTCTGTTATTGTCTCAGTGATTTTGACTTTTGTAGGACTCATCGTGAATCTCTTCAT
TGTAGTAGTCAGTTACAATACTTGGGTCAAAAGCCACAGAATCTCCTCTTCTGATAGACTCCTGTTCAGCTTGGGCATCA
CCCGATTTCTTATACTGGGACTGAATGCTGTTTTCTTCATCTCTACAAATATGGAAAGGTCAGTCTACATAGCCATTTTT
TTCCCCTGTCATTTTGGATGTTTTTGGACTCTAATAGTCTTTGGTTTGTAACCTTGCTCAATGCCTTGTACTGTGTGAAG
ATTGCTAACTACCAACACTCACTGTTTCACCTGCTGAAACAAAATCCCTCCCCCAAGATGCCCTGGCTGCTGCTGGTATG
TATGCTGATTTCTGTCTTCACCACTCTCCTGTATGTTGTACTCAGACAGAAAGCACCCACTTCTGAATTTGTGGCTGGGA
GAAATGGCACAGTATTTGACATCAATGAGAGAATCTTGTCTTTGGTGACCCCTTTGGTCTTGAGCTCATTTCTCCAATTC
ATCATTAATGTGACATCTGCTTATTTGTTAATCAATTCCTTGAGGAGACTTATACAGACGATGCAGAGAAATGCCACTGT
TCTTTGGAATCCCCAGGCTCCTGTGGGTGCTCTGAAGCTGATGATATATTTCCTCATACTCTACATTCTGTATTCAATTG
CTTCCCTGCTCCATTATCTCCCTTCTTCTGTAGGGATGGATTTGGAAGCCAAGTCCATTTATGTTATCATTTCCACCATT
TACCCTCCAGGACATTCTGTTCTCATTATCCTCACACACCCTAAACTGAAAACAAAGCAAAGAAGATTCTTTGTTTCAAT
AAATAG
>LiveTAS2R5P_NW_006793703.1:7440637-7441384
ATGCTGACTGCTGTCCTAGGACTGTTGATGCTGGTGGCAGTGGCTGAATTTCTCATTGGCCTGGTTGGAAATGGAGTCCT
TGTGGTCTGGAGTTTTGGAGAATGGCTCAGAAAATTCAAGGGGTCCTCATATTAACTTCATTGTCCTGGGCCTGGCTGTC
TGTTGATTTCTTCTGCAGTGGTTGATTATGGTGGACTTAAGTCTGTTTCCACTTTTCCAGAGCAGCCATTGGCTTCACTA
TCTCAATGTCTTCTGGGTCTTAGTAAGCCAGACCAGCCTGTGGTTTGCCACTTTTCTCAGTGTCTTCTACTGCAGGAAAA
TCATGACCTTTGAACACCCTGTCTACTTGTGGTGAAGCAGAGGGCCTGTTGCCTGAGTCACTGGTGGCTTCTGGTGTACA
CTTCATGATCAGTTTGTTACTTATAGTCCAGGGTAGCTTAGAGTTCTCCGATCCTTCCCACGGAAACAGCAGCATTTTAT
ACCCCCCTTCAAACTGGCACTGTCTGTATATATTATGGCTCAATACACGAAGTATAATGCCCTTCATGGCGTTGCTTATT
TCCTCTGGGATGCGGATTGTCTCTTTGTGTAGACACCGCAGGAAGATGAATGTCCATACAGCCGGCAGGAGAGATGCTCA
GGCCAGGGCTCACATCACTGTCCTGAAGTCCTTGGGCTGTTTCCTTATACTTTACACAGTTTACATCCTGACCAGCCCCT
TCTCCATCACCTCCAAGTCTTTTCCTGCNNNNNNTGCTGATCTTACTGCTCTCTTCATCTCTGAGACACTCATGGCTGCC
TACCGTTCTCTTCATTCTATCATATTGATCATGGGGAATCCCAGGATGAAGCAGACTTGTCAGAGAATCCTGTGGAAGAC
AGTATACACTTGG
>LiveTAS2R16P_NW_006799025.1:1180953-1181840
ATGATAACCATCCAACTCTGTCTTCTTCATGATCATCTATATGCTCAAGTTCTTGACAATAATTGTGCAGAGCTGCTTAA
CTGTTGTTGTGCTGGGCACAGAGTGGGTAAGTTTCCAAAGGCTGTCACCTGTGGAAGTGATCCTCAACGGCCTGGGTGTC
TGCTGCTTCTGTCAACTGTGGTCATCGATGCTGTACAACTACTGCTCCCACTTCTGCCCTAATTACGAATTTTGGTACTT
CAGTATCGTCTGGGAATTTACTAGCATTCTGGGTAACCAGCACATTTGCTGTCTTCTACTGTGTCAAAGTCTCCTCCTTC
AGCCACCCCATCTTCTGGCTGAAGCGGAGAATTGTGAGGCTGGGTCCTTGGCTGTTGCTGGGTTCTCTGCTGATTTCTTG
TGTGTCTATCATCTTTGCAGCTGTTGGGCATTACAGCAAGATTCAACTAATCTCCATGAGGCATTTCCCTAGAAACAGCA
CCATGACTGAGAGACTTGAGATATTCCTGTGGGATTTTTCCATGTGTCACAAGTGGTTGTGTTGATTATTCCTGTCCTCC
CGTTCCTGGCCTCCACCGTCTCGCTCATAGCCTTATTATTCCAACACCTGAGGCAGATGAAAGATCATCACACCAGCCAC

TCTCCAGCCTGGAAGCTCACTCTACTGCCCTGAGGTCTCTTGCCGTCTTCCTCATTTTCTTCACCTCTTATTTTCTGACC
CTACTAAGCTCCGTGTGGGGTGTCCTTTTTAATAAGGGGTCCTGGTTCTGGGCCTGGGAAGCTATCATCTGTGCTCTAGT
CTCTATTCATTCGACTTCATGGATGCTGAGCAGCCCTAAACTGAAAAGGGTTTTAAAGGTAAAGTGCTGGGGCCTAGAAG
CTGCCTGA
>LiveTAS2R38P_NW_006793703.1:7499260-7498256
ATGGTGACTCTGACTGCCATTGTAACTGTGCCCTATGAAGTCAGCAATGCATTTCTGTTCTTTTCAGACCTGGAGTTTGC
AGTAGGGATCCAGGTCAATGCCTTCATTTTCTTGATGAATTTTTGGGTCATGGTGAGGAGGTGGCCACTGAGCAACTGTG
ATCTTGTTCTGCTGAATCTCAGCCTCACCTGGCTTTTCCTGCAGGGGCTGCTCTTTCTGGATGCCATCCAGCTTACCCAC
TTCCAGTGGATAAAAGACCCGCTGAGCCTCTGCTACCAGACCATCCTCGTGCTCTGGATGCTCGTGAATCAAGCTGGCCT
CTGGCTCACCACTTGCCTTAGTCTCCTCTACTGCTCCAAGACTGTCCATTTCTTTCACACCCTCCTCCTCCGCTTGGCAA
GCTGGATCTCCAGGAAGATCCCCCAGATTCTCCTGGGCGCTATTTTTTCCTCCTGTGTCTGCACTGTTCTCTATTTGTGG
GACTTTTTCAGTAGATCTCACTTCTCAGTTGCAACCATGCTACTCATGAATAACAATACAGAACTCACTTGAGAAACTGA
GAAAACTCAATTTCTTTCATTCCTTCCTCTTCTGCAGCCTGGGGTTCATCCCTTCTTTCTTGCTTTTTCTGGTTTCTTCT
GGGGTGCTGATTGTCTCCCTGGGGAGGCACATGAGGACAAGGAGGGCCAAAACCAGAGACTCTCGGGACCCCAGCCTGGA
GGCCCACATCAAAGCACTCGAGTCTCGTCTTTCTTCTGCCTGTATGTGGTGTCCTTCTGCGCCACCTTCATCTCGGTGCC
TTTGCTGATGCTGTGGCACAACAAGATCGGGGTCATGGTCTGTGCAGGGATGCTGGCAGCCTGCCCCTCAGGGCACACAG
TCATCCTGATCTCAGGCAATGCCAAGCTGAAGAGAGCTGTGGAGACCACTCTGCTCCGGGCTCAGAGCAGCCTAAAGGTA
AGGGCGGACCGCAAGGCAGATCCCAGGATGCCGGATCTATGTTGA
>LiveTAS2R39P_NW_006793703.1:8108050-8109015
ATGACTGAAACCTGCAATCCCCCAGAAAATCAACTGTCACCATCTCGCATCATTTTGATGTGAAGAATTATAGGCACCGA
ATGCGTCCTTGGTATCACTGCAAATGGGTTCATTGTGGCTATAAATACAGCAGAATGGATTCACAAGAAGGCAGTTTCCA
CAAGTGGTAAGATCCTGCTTTTCCTGAGTGTATCCAGAAGAGCGCTACAAAGCTTCATGATGCTAGAACTCACCTTCAGT
TCAACATCCCCACACTTTTATAATCAAGACATTCATTGTATGTGATACATTCAAAGCAAGTTTCATGTTCATAAATTATT
GTAGCCTCTGGTTTGCTGCCTGGCTTAGATTCTTCTACTTTGTGAAGATTGCGGATTTCTCCTACCCCCTTTTCCTCAAG
CTGAAATAGAGAATTTCTGGATTGATGCCCTGGCCTCTGTGACTATCAGTGTTTGTTTCCTTGGGCCACAGTGTGTTCTT
CCTCAAAAACATCTACACGGTGCATTGCAACCATCCTTTTACTCCTTCAACTCCACTAAGAAAAATTACTTCACTGAGAC
CAATATGATCAGCCTGGTTCTTTTCTTTAACATGGGAATCTTCGTTCCTCTGATCACGTTCATCCTGGCTGCCACCCTGC
TGATCATCCCTCTAAAGAGACACACCCTACACATGGAAAGCAATGCCACTGGCTCCAGGGCCCCCAGCATGGAGGCTCAT
GTGGGGACCACCAAAGCTATCAGCTATTTTCTCATTTTCTAAATTTTCAATGCACATGCTCTGTTTCTTTCCATGTCCGA
CATCTTTGATATCAATAGTTCCTAGAATATTTTGTGCAAAATCATCATGGCTGCCTATCCTGCTGGCCACTCCATTCTAC
TGATACAGGACAACCCTGGGTTGAGAAGAGCCTGGAGGCGGCTTCAGGCTCACGTTCACCTTTACTTAAAAGAGTAGACT
CTATGA
>LiveTAS2R60P_NW_006793703.1:8322548-8323484
ATGGTTCCAGGACCTCAGTTGGCTGATAAGATAGCCTTTATCTTTGCTATCATTTTATTCCTTTTGTGCTTGGTGGCAGT
GGTGGGTAATGGCTTAATCACCGTGGCACTGGGCATGGAGTGGTTGCTGCAGAGAACTTTGTCACCCTGCAATAAGTTAT
TGGTCAGCCTGGGAGCCTCTAGCTTTTATCTGTGATGGGTGGTGATAAGAACATTTATATTTTCCTGAATCCAGTAGCCT
TCCCATACAACCCTGTATTCCAGTTCCTAGCCTTTCAGCGGGACTTCTTGAATGCTGTCACGTTATGGTTGTCCACCTGG
CTCAGTGTCTTCTACTGTGTGGAAATCGCAACCTTCACCCACCCTGTCTTCCTCTGGCTAAAGCAGATGGTGTCTGCATT
GGTTCCATGGATGCGGCTCAGCTCTGTGGGGTTCTCCAGCTTTAGCACCATTCTAGTTTTCATAGGCAACCAGAGAATAT
ATCAGAACTATTTAAAGAGGGCTCTGCAACCTTGGAATGTCACTGGGAATGCTGTGAGAACATATGAGAGACTCTGCTTC
TTCCCTTTGAAAATTGTTACCTGGACAGTCCCTACTGTTGTCTTCATCGCTGGCATGGCTTTGCTCATTAACCTCTGGGA
AGACACACCAAGAAGGTCTCCCTGTCCATCTCAGGCTCTCATGATCCCAGCACCCAGGCACACATCAAGGCTCTCCTGGC
TCTCATCTCCTTTGTCGTCCTCTTCGTTTCCTATTTTCTGTCACTGGTGCTCAGTGCCCCAGGTGTGTTTCCATCACGGG
AATTCAGGCACTGGGTGTGGTGGCAGGCTGTGATTTATCTGTGCACAGTAGTCCACCCCATTGTTCTCTTCTTGAGTAAC

CACAGACTGAGAGCTGTGCTAGAGAGGGGCTGCTCCTCAGGGCATGGGGCATCTTGA
>LiveTAS2R62AP_NW_006793703.1:8316446-8317372
ATGCCCTCCTCACCCATGTTGATCTTCATGGTCATCTTTTTCCTGGAGTTGTTGGCTGCCATGCTGCAGAATGGCTTCAT
AGTTACTGTGTTGATCAGGGAGTGGGTACAATGCCAGACACTGCTTGCAGGCGACATGATTGCGGTGGCCTCCCTGGCCG
CCTCCCGGTTCTGACTGCATGGGATGGCCCTCCTGAACAACCTCGTAGCCTTCTTTGGTTTTGGTCCCAAAATACAGTAT
TTCAGCATCCCCTAGGACTTCATCAACTCTCTTACTTTCTGGCTTACTGCTTGGCTTGCTACATTCTACTGTGTGAAGAT
CTCATTCTTCTCTCACCCCATCTTCTTTGGGCTGAAGTGGAGGATTTCTCGGTCAGTGCCCAGGCTGCTGCTGGGCTCCC
TGATCTTATCTGCTCTGGTAGTCATCCCATTAGGCACTGGGAACACAATTCGTGTGCAGATGGTTGCTTCCCAGAGTTCT
CATGGAAACAGCACCCTGGCTGGTAGAATACAGACTGTCTCTTTGTACTTTTTTCTACCTCATGTAATTATTATGCGGTC
AATTCCATTTCTCCCGTCCCTGGTGTCCACCCTCTCGTGTTCTCGCTGCGCCGGCACTTGGGGCAGATGAGGGACCATAG
ACCTGGCCCCGAGTGATCCCAGCACCCGGGCTCACACCGTGACCCTGAAGTCACTTGCCTTCTTGCTCATCTTCTACCAT
CATATTACCTGTGCCTGATTATTGTTGTTATAAACATCCTAACCCTCTGGAATCACTGGCGCTGGGCCTGGGAAGTGGTG
ACCTGTGCAGGCATCTGTCTGCACTCCAGCATCTCGGTGCACAGCAGCCCCAAGCTGAGAAAGGCCCTGACGAAGAGGCC
TTGGAGAGCCCTGGGCAAGGAGCAGTTTGTCTCATCAGTGTCAGTAA
>LiveTAS2R62BP_NW_006797698.1:1418974-1418077
ATGTGGATCTGCATGGTCACCTTTTTCCTGGAGTCGGTGGCTGCCACGCTGCAGAACGGCTTCACAGTCATCGTGCTGAG
CCGGAGTGGGACGCTGGATGATGCTGGACGCTGGATGATGCTGGACGCTGCCCGCAGGCGACGTGCTTGTGGCCTGCCTG
GCCGCCTCCCGGTTCTGTCTGCAAGGGATGGCCCTCCAGAACAACCTCCTGACTTCCTTTGGTTTTGGTTCCAAATTTTA
TTTCAAAATCTCCTGGAGCTTCATCAATGCTCTCACTTTCTGGCTGACCAGCTACCGTGTGAAGATAGCATCCTTCTCTC
ACCCCGTCTTCTTCTGGCTGAAGTGTAGGATTTCTCGGTCAGTGTCCAGGCTGCTGCTGGGCTCCCTGATCCTGTCTGGT
CTGACATCATCATCAGAAGTCAATTCTTGTGCAGATGGTTGCCACCCAGAGTTCCCATGGAAACGACACCCTGGCTGGTA
GGATACAGACCGTCTCTTTGCGCTTTTTTCTACCTCATGCAATTATCACGTGGTCAGTTCCATTCCTCCTGTTCCCGGTG
TCCACCCTCTCGCTCGTGTTCTCGCTGCGCCGGCACTTGGGGCAGATGAGGGACCAGACCCGGCCCGAGTGATCCCAGCA
CCCGGGCTCACACCGTGGCCCGGAAGTCACTTGCCTTCTTTTTCATCATATTTCCTGTGCCTGATAATTGTCCTTGTGAA
CATCCCAACCCTCCGGAAGCACCGGCACTGGGCCTGGGGAGCGGTGACCTATGCCGGCATCTGTCTGCACTCCAGCATCT
TGGTGCACAGCAGCCCCAAGCCGAGAAAGGCCCCGAGGAAGAGGCTTCGGCGAGCCCTGGGCAAGGAGCAGTTTGTCTTG
AGTTACCAGTATCAATAA
>EpfuTAS2R1_NW_007370720.1:2598823-2597924
ATGTTACAGTTGTACATCATTACCCATCTTATTTTTTCAGTGATTCAGTTTCTCGTGGGGGTTCTAGCCAATGGCTTCAT
TGTGGTTGTGAACGGCACAGACTTGATCAGGCAGAGAAAGATGGTGCCCTTGGACCTCCTCCTGTGCTGCCTGGCGACTT
CCAGGATTGGTCTCCAGATGGTCACCATCTACTTTAATCTGGTTGCTCTTTCCTTGATTGAATTCTCTCCAGTTCCTGAG
AATTTTATCATTTTCATGTATGTACATGCATCGGAAATTTGGTTGGCCACATGGCTCAGCGTTTTCTACTGTGCCAAGAT
CGCCACCATCGCTCACCCGCTCTTCTTTTGGTTGAAGTTGAGGATCTCCAAGTTGATGCCATGGCTGATTTGTGGGACCT
TGATCTATACCTTTCTCACTTCTGTCTTCCACAGAAAACATGCATGGATTATTTCCCAAAAATCCTGGTTGGGCTTTTTC
TCCCAAAATGCAACAACGCAAGTCGAAGACGTACCTGTATTACAATTTGCCCTTCTTGTCATTGAGTTCATAATGCCCTT
ACTTATCTTCCTTATCTCTGCTCTTCTCTTGGTATTTTCCCTGGGGAGGCACACCCAGCAGATGAGGAGCACAGCGACGG
GCACCAGGCACCCTGGCATGAGTGTCTACATCAGCGCACTCCTGTCCATCCTGTCCTTCCTGGTCCTCTACCTCTCCCAG
TACATGATGGCTGCGTTAGTCTTGTCTCAATTTTTCAAGATCAGAAACTTCATCACTCTGTTCTGCATCTTGCTATTTGG
TTCATACCCCTCTGTACATTCTGTTATCTTAATTTTAGGAAATTCTAAGCTGAAACAAAATGCGAAGAAGTTCCTCCTCC
ACAGTAAGTGCTGTCAGTGA
>EpfuTAS2R2_NW_007370653.1:20013295-20012384
ATGACCCCCTCTTTATCAGCTATTCCCCATGCGATCATCATGTCAGCAGAATTTATTACCGGGATTACAGTCAATGGATT
TCTGATAATCATCAGCTGTAACGAAATGATCAAAAGCAGAAAGCTAACACCAATGCAGCTCATTTTAATATGTATAGGGA
TGTCTAGAGTTGGTCTGCTGATGACATTAATGGTACAAAGTTTTTCTCTATCTTCTTTCCACTCTTTTATCGGACAAGA

ATTTATGGTGCAGCGATGGTGTTCGTTTGGATGTTTTTAGCTCTGTCAGTCTCTGGTTTGCCACCTGCCTGTCTGTATT
TTACTGCCTCAAGTTAATAGTCTTCACTCATCCCTGTTTTCTTTGGCTGAAATTCAGGATCTCAAAGTTAATGCCTGGGC
TGCTTCTGGGAAGCTTGCTGGCCTCGGTGAGCACTGCAACTCTGTGTATCGAGGTAGATTACCCTAAAGACGCGGTGGAG
GATGTCCTCAGAAATGCCACACACACCACGTCTAAAATCAAGATAAGGAAAATTAGTGAAGTGCTTCTTGTCAATTTGTC
ACTCCTATTTCCTCTAGCCATATTCCTGATGTGCACTTTCATGTTACTCGTGTCTCTCTACAAGCACACTCATCGGATGC
AAAACGGATCTCGTGGTTTTAGAAGTGTCAGCACAGAAGTCCATATAAACGCCTTAAGAACAGTGCTAACGTTTGTTCTT
TTCTTTATTTCTTACTTTGCCGCCTTCATAACAAACATGACATTCATTATTCCTCACGGAACTCAGCGCTACTTTGTGAT
GAAGGACATAATGGCAGCATATCCCTCTGGCCACTCAGTTATAATAATCTGGAGTAATTCTAAATTCCAACAACCACTCA
GGAGACTTTTCTGCCTCAAAAAGAGTCAGTGA
>EpfuTAS2R3_NW_007370712.1:442893-441943
ATGTCAGGACTCACCAAGTGGGTGTTTCTCTTTCTTTCTGTCACTCTGTTTGTTCTGGGAATGCTGGTGAATGTCTTCAT
TGTGCTGGTCAATGGCAGCAGCTGGGTCAAGAGCAAGAAAATCTCTTTATCTGACTTCATCATCACTAACCTGGCTCTCT
CTAGGATTGTTCAGCTGTGGATTCTCTTGTTCGATTGTGTAAAAAAGATATTACTTTCCAAACTACTTTTTAATGGGGTA
TTACTGCAAGTTACTGATATTTTCTGGACATTTACAAATCATCTGAGCATTTGGCTTGCCACCTGTCTCGGTGTCTTCTA
CTGCCTGAAAATCGCCAATTTCTCCCACCCCACATTCCTCTGGCTCAAGTGGAGAGTTGCCAGGGTGGTAGTATGGATGC
TGTTGGGTGGGCTGCTCTTATCATGTGGTGATGTCATGTCTCTGATTCATGAGTTTAAGATCTATTATGTTCTCCAGGGA
GCTAATAACTCAGGGAATGTGACTGAGCACTTTAGAGAACTAAAGAATGAATATGAGGTGACCCGTGTTCTTGGGACACT
GTGGAAACTCCTTCCCCTAATTGTGTGTTTGGCCTCCTACATCCTGCTCATCCTCTCCCTGGGGAAGCACACGCGGCAGA
TGCAGCAGAACAGAACCAGCCTCAGCGATCCAAGCACCGAGGCCCACAAGAGGGCCATCAAAATGGTCCTCTCCTTCTTC
TTGCTCCTCCTGTTTTACTTTCTTGCCTATTTACTCACATCATTCCAGTATTTCCTACAAGGAACTGTAATGACTGAGCT
GATTACAGAAGTAAGTGCAATGTTTTATCCAGCCTGCCACTCGTGTATTCTCATTCTGGGAAACAGTAAGCTGAAGCAGA
CGTTTGTGGAGCTGCTCTGGTGTAAGTCTGGTCATCTGAAGCCTGGATCCAAGGAACGCTTTTCCCCATAA
>EpfuTAS2R5_NW_007370712.1:428968-428078
ATGCGTATTGCCACCCTAGGACTGCTGATGGTAGTGGCAGTGGCCGAATTTCTCATTGGCCTGGTTGGAAATGGAGTCCT
TGTGGTCTGGAGTTTTGTAGAATGGGTAAGAAAACTCAAGAAGTCCTCCTACAACCTCATTGTCCTAGGCCTGGCTGGCT
GCCGACTTCTCCTGCAGTGCCTGATTATGGTGGACCTAATGCTGTTTTCGATTTTCAAGAGCTGCATCTGGTTTCGCTAT
CTCAGTGTCTTCTGGGTTGTGGTCAGCCAGGCCAGCCTGTGGTTTGCCACTTTCCTCAGTGTCTTCTACTGCAAGAAGAT
CACGACCTTTGAACACCCTGTCTACCTATGGCTGAAGCAGAGGGCCTATAGTCTGAGTGCCTGGTGTCTTCTGGGGTGCT
TCCTGATCAATCTGCTAATTATAGCCGATGTTGGCTTAAAGTCCCAGAGTCCTTTCCAAGGAAACAGCAGCATTCTGTAC
TCCCTTTCAGACTGGCAGTATCTGCATATATTACAGCTCAATGCAGGAAGTGGGTTGCCTTTCTCTGTGTTTCTAATTTC
CTCTGGGATGTTAATTGTCTCTTTGTATAGACACCATAAGAAGATGAAAGTCCATACAGCTGGCCGGAATGATGCTCGAG
CCAAGGCTCACATCACTGTCCTGAAGTCCTTGGTCTGCTTCCTTATACTTTACTTGGTTTACGTCGTGGCCAGCCCCTTC
TCTATCACCTCCAAATCTTCTCCTGTTAATCTCACCACTATCTTCATCTCGGAGACACTCATGGCTGCCTATCCTTCTCT
TCATTCTGTCATATTGATCATGGGGAATCCCAGGATAAAGCAGGCTTGTCAGAGATTCTTGTGGAAGACAGTGCGTGCTT
GGAAGTCCTGA
>EpfuTAS2R7_NW_007370663.1:1740866-1739928
ATGTCAGACGAAGTAATCAGCACCTTAATGATCATAACAGTTGGGGAGTTTTCAGTGGGGATCTTAGGAAATGCATTTAT
TGTATTGATAAACTTCGTGGACTGGATGAAGAGTAAGAAGATTGCTTCCATTGATTTAATCCTCACAAGTCTGGCCATAT
CCAGAATTTGTCTAATGTGTATAATAATGTTAGATTGCTTTATGTTGGTGTTGGATCCAGACGTCTATGCCACTGGTAAA
CAAATGAGAATCATTGACTTCTTCTGGACACTGACCAACCATTTAAGTATCTGGTTTGCCACCTGCCTCAGCATTTTCTA
TTTCCTCAAGATAGCTAATTTCTTCCATCCTCTTTTCCTCTGGATGAAATGGAGAATTGAAAGGGCGATTCCTGGGATCC
TGCTGGTGTGCTTGGCCTTCTCTGTGTTCATTAGCCTTCCTGCCGCTGAGAACTTGAATGATGACTTCAGGCTTTGTGTC
AAGGTGAAGAGGAAAACAAACTTAACTTTGAGATGCAGGGTAAATAAAGCTCAATATGCTTCCAGCAAGGTATATCTCAA
CCTGTTCACGCTGTTCCCCTTTTCTGTGTCCCTCATCTCTTTCCTCCTCTTGATCCTCTCCCTATGGAGACACAACAGGC

GGATGCAGCTCAATGCCACAGGGTGCAGAGACCCCAGCACAGATGCCCACATGGGAGCCATGAAAGCTCTCATCTCCTTC
CTCCTACTTTTCATTGCCTACTATTTGTCCTTTCTCATAGCCACCTCCAGTTACTTCATGCCAGAGACTGAATTAGCTGT
GCTGATCGGTGAACTGATAGCTCTAATCTATCCTTCAAGCCATTCATTTATATTAATTCTGGGGAACAAGAAATTAAGAC
AAGCATCTCTAAGGGGGCTATGTAAAGTAGCACATACACTAAAGAGAAGAAATTTATAA
>EpfuTAS2R10_NW_007370663.1:1748742-1747843
ATGCTTAATATAGCGGAAGGTCTCCTTATTTCTGTAGTAGTTGGTGAATCAATACTGGGGGTTTTAGGAAATGGATTTAT
TGGACTTGTAAACTTCATTGACTGTGTCAAGAACAAGAACTTTTCTGTAATTGGCTTGATTCTCATTGGCTTAGCTACTT
CGAGAATTTTTCTGATATGGATAATAATTATAGATGGATTTATAAAGATATTCTCTCCACATATGTACTTCTCTGGAAGC
CTAATTGAATATATTACTTATTCATGGATAATTATAAATCACTTAAGTATCGGGTTTGTCTCCAGTCTCAGCATATTCTA
TTTCCTGAAGATAGCTAATTTTTCCCACCACATTTTTCTCTGGTTGAAGCATAGAATCAATAGAGTACTTCCTCTTCTGA
TGGGATTGATGTTTATTTCATGGTTATTTACTTTCCCACAAATTGTTAAGATTATTAATGATTATAAAATAGGTAATAGA
AACACAACCTGGCTTCTCACCATAAGTAAAAATGAATACGCTGCTTACCAAATTTTGCTCAATCTGGGAGTCATTTTCCT
CTTTACACTGTGCCTGATTTCATGCCTCTTTTTAATCATTTCTCTTTGGAGACACAACAGGCAAATGCAATCGAGTGCCC
TAGGTGTCAGAGACCCCAGCACAGAAGCACATGTGAAAGCAATGAAAGTGTTGATATCTTTTATCATCCTCTTGATCTTG
CATTTTATAGGCATTACCATAGAAATATCATGCTTTACTGTGCCAGAAAGCAAATTGCTGCTTATTTTTGGTATGGCCAC
CACAATCACCTATCCCTGTGGTCACTCATTTCTCCTAATTCTAGGAAACAGCAAGCTAAAGCAGGCTTTTCTGAAGGTAC
TATGGCCTTTAAATAGCTAA
>EpfuTAS2R11_NW_007370663.1:1764334-1763393
ATGTTGACTATGCTGGAGAAAGTTTTCGTGACTGTAACAAGTGGGGAATTTTTAACAGGAATTTTAGGAAATGGATTCAT
TGGACTCACAAATTGCATTGCCTGGGCTAGAAATCAGAAGTTCTGCTTGGTTGACTTCATTCTTACCAGTTTGGCCTTCA
TCAGAATCAGTCTTTTGTGTCTAACAATTGCCAATTTGTTTTCAGTGCTGTCCCATCAGGAAATCCCTGATACTATGGAA
GGAAACCTTATTTATTCTAGTTTCTGGATACTGGCCTCTCACCTGAGTACGTGGTTAGCTACTTGTCTCACTGTCTTTTA
TTTCCTGAAGATCGCCAATTTCTCCTCTCCTTTTTTTGTCTGGCTAAAATGGAGAATTAACAAGGTAGTTTTCACGCTTC
TGCTGGTATCTTTGCCCTTCCTGCTCCTGAGCCTTCCTTTGCCATATAATTTTGGTATCTGCTGGTATCATTTCCCCCCA
AAACATGAGGGAAATATGACTGGGTTATTCAATGTGAGTAGAAGTAAAAATCTAGATCAGGTTGTTATGTTCATGATTGG
GTCCCTCCCTCCTTTCTCTGTTTCCTTGATTTCCTTTTTCCTATTGCTGCTTTCTTTGTGGAGACACACAAAACAGGTTG
AGCTCAACATTAGGAAATCCAGAGATGCCAGTACAGAGGCCCACTCCAGAGCGATGAAAACTGTGTTTTTCTTTCTTGTG
CTCTTTGCACTGCAGCACTTCGCCTATTTCATGACATTTGGGGGTTATTTTTTGCCACAGAACAAGCTCATTGTGATGCT
TAATTATATGATAGGAATTTTATATCCTTCAGGGCATTCATATGTTGTGATTTTTGGAAACAGCCAAATGAGGAAAGCCT
TCTTGCGGATTCCTTGGCACCTGAAGCGATGCCTGAGAAGAAAGGTACTCTCAGTTACATAG
>EpfuTAS2R16_NW_007370896.1:451954-451025
ATGATACCCAACCAACTCACTGTTTTCTTCATGATCATCTATCTGCTCGAGTCCTTGACAATAATTGTGCAGAGTAGCTT
AATTGTTGCAGTGCTGAGCAGAGAGTGGGTGCAGGTCAAAAGGCTGTCACCTGTGGACATGATTCTCATCAGCCTGGGTG
TCTGCCGCTTCTGTCTACAGTGGTCATCAGTGCTGTACAACTTTTGCTCCTATTTCAACCCTGACAATGAACTTTGGTAC
ATAGGAGTCATCTGGGAATTTACTAATACTCTTACATTCTGGTTAACCAGCTTGCTTGCTGTCGTCTACTGTGTCAAAGT
CTCTTCCTTCACCTACGCCATCTTCCTCTGGCTGAGGTGGAGAATTTTGAGGTTGATTCCCCGGCTGTTGCTGGGCTCTC
TGATGATTTCCTGTGTGACAATCATTGCTTCAGCTATTAGACATCACCTCAAGAGTCAGTTAATCTTCTTGATGCAATTA
CCTGGAAACAACACGGAGATTGAGACACTTAAGACATTTTTGGATAAGTATTACGCACGTCAGCATCTGGCAATGCTGTT
CATTCCTTTCCTCCTGTTCCTGACCGCCACCATCTTGCTCATAGCCTCGTTGTCCCAACACTTGAGGCAGATACGACATC
ACAACGCCGGCCACAGCAAGTCCAGCATGAAAGTTCATGCCACTGCCCTAAAGTTCTTTGCCTTCTTCCTCATCTACTTC
ACCTCTTACTTTTTGACCATAATCGTCTCCACTAAATACACCCTAGGATATAAGAATTCCTGGTTCTGGGCCTGGGAAAC
TATCATCTATGCTACAGTCTCTATTCATTTAACTTCACTCATGCTGAGTAGCTCTACGTTGAAAAAGGGTTTAAAGGTAA
GCTGCAATGGCCCAAAGCTGCCTGAGACTCCGGGTACAACAAGACCTTGA
>EpfuTAS2R408A_NW_007370663.1:1849004-1848054

ATGATGAGTTTATTACCGATCATTCTTTCCACACTAGTTATAGCTGAATTTTTTCTAGGAAATATTGCTAATGGCTTCAT
AGCACTGGTGAACTGCATTGACTGGGTCAAGAAACACAAGATCTCCTGCGCTGATGGAATTCTCACTGCTCTGGCGGTCT
CCAGAATTGGTTTGCTCTGGGTAATAATATTCCATTGGTATGCAACTCTGTTTAATCCAGCTTTGTATAGTTTAAGAGTA
AGAACTTTTGTTTCTATTGCCTGGGTAGTAAGCAACCATTTTAGCCTCTGGCTTGCCACCAGCCTCAGCATATTTTATTT
GCTCAAGATAGCCAATTTCTCCAGCCTATTTTTTCTTCACCTAAAATGGAGAGCTAACAGAGTGGTTCTCACGATACTGT
GGGGGACTTTGGTCTTCCTGGTTTTTCGTCTTGCGGTGTTAAGCACAGATGAAGAAATGCAGATGCATGAATATAAAGGA
AACATCACTTGGAAGACCAACTTGAGGGGTATTGTATACCTTTCAAATTTGACTGTATTTACGCTCGTAAACTTCATACC
CTTTTCTACGTCCCTGACAGCTTTTCTGCTGTTAATCTTTTCCCTGTGTAAACATCTCAAGAGGATGCAGCTCAGTGGTA
AAGAAACTCAAGATCTCAGCACCAAGGTCCATATAAGGGCCATGCAAACTGTGATCTCCTTTCTCTTGCTATTTGCCATT
TACTTTGTGACTCTAATCATCTCGGTTTGGAGTTTCTATAATCCTCGGGACATACCAGTTTTCCTGTGTTTCCAGGTTTG
GGCACTTGCCTATGTTTCAGGCCACTCCCTAATTCTGATTTGGGGAAACAAGAAGCTAAGTCAGGACTTTGTCTCAGCTT
TATGGCAGGTGAGGTGCTGCCTGGAAAAATGGAAACTTTCAACACTAAGAGGGAAATCATTTGTATTTTAG
>EpfuTAS2R38_NW_007370712．1:349854-350861
ATGTTGACTCTGACTCCCAACATAACTGTGTCCTATGAAGTCAAGAGTGTATTTATGGTCCTTTCAGTCCTGGAGTTTGC
AGTGGGGATTCTGGTCAATGTCTTCATTTTCTTGGTGTATTTTCGGGATGTGGTGAGGAGGCAGCCCCTGAGCACCTGTG
ATCTTGTCCTGCTGAGTCTCAGCCTCATCCGGCTTTTCCTGCATGGGCTGCTGTTTCTGGATGCCATCCAGCTTACTCAC
TCCCAGCAGATGAACGACCTGCTGAGCTTCACCTACCAAACCATCATCATGCTCTGGATGATCACAAACCAAGCCGGCCT
CTGGCTCGCCACCTGCCTCAGCCTCCTCTACTGCTCTAAGATCGTCCGTTTCTCTCACGCCTTCCTGCTCCGCTTGGCCA
GCTGGATCTCCAGGAAGATTTCTAGGATGCTCCTGTGTACTGTCCTTTTCACCAGTGTATGCACTATCATCTGTTCATGG
GACTTTTTTAGTAGATCTCACTTCAGAGTCACAACTGTGTTATTCATGAGTAACAATACAGAATTCAATTTGCAAATTGC
AAACCTCAAGTTCTTTCATTCCTTCCTCTTCTGCAGCGTGGGGTCCATCCCACCTTTCTTGTGTTTTCTGGTCTCTTCTG
GGGTTCTGATTGTCTCCCTGTGCCGCCACATGAGGACAATGAGGGCCAAGACCATGGACTCTTGTGACCCCAGCCTGGAG
GCCCACATCAAAGCACTCAAATCCCTCATCTCCTTTCTCTGCCTCTTTGTGGTGTCATTATGCGCTGCCCTCCTCTCGGT
GCCTTTACTAATGCTGTGGTACAACAAGATTGGGGCCATGGTCTGTGTGGCGATAATGGCAGCCTGTCCCTCAGGGCATG
CAGCCATCCTGATCTCAGGCAATGCCAAGCTGAGGAGAGCTGTGGACAGCATTCTACTGTGGGTTCAGAGCAGCCGAAGG
GTAACAGCAGACCACAAGGCAGATCCCAGGACACCAGGTCTATGTTGA
>EpfuTAS2R39_NW_007370745．1:4532999-4532040
ATGACCAACACCTGCAGTCCACCAGAGGATAATTTGTCACCATTTAATATCATCTTAATTTTCACAATAATGGGCCTGGA
ATGCATCATTGGCATCATTGCAAATGGGTTCATTGTGGCTATAAATGCAGCTGAGTGGATTCAGAATAAGACAATCTCCA
CAAGTGGCAGGATCCTGTGTTTCCTGAGCATATCCAGAATTGCTCTCCAAAGCTTGATGATACTAGAAATTACTTTCCAC
TCAATATCCCCACAATTTTATTATAAAGATGGTGTATATGATACCTTTAAAGTGGGTTTCATGTTCTTACATTATTGTAG
CCTCTGGTTTTCTGCCTGGCTCAGTTTCTTCTACTTCGTGAAGATTGCTGATTTCTCCTACCACCTTTTCCTCAAGCTGA
AGTGGAGAATTACTGGATTGATGCCCTGGCTTCTGTGGCTATCAGTGTTTTTTGCCTTGGGCTACAGTATGCTCTTTTCC
TATGGCATATATACTGTTTATTGTAACAATACTTTTCCTATCCCCTCCTCCAACTCTACTAAGAAAATATACTTCGCTGA
GACCAACGTGGTTAACCTGGTTCTTCTCTATAACCTGGGGATCTTCATTCCCCTCATCATGTTCATCCTGGCGGCCACCC
TGCTGATCATCTCTCTCAAGAGGCACACCCTACACATGAAAAGCAATGCCACTGGCTCCAGGGACCCCAGCATGGAGGCC
CACTTGGGGGCCATCAGAGCTATCAGCTACTTTCTCATTCTCTACATTTTCAATGCAGTTGCTCTATTACTCTATATGTC
CAACATCTTCAATGCCAACAGTTCCTGGGATATTTTGTGCAAAATTATCATGGCCGCCTATCCTGCTGGCCACTCCATTC
TACTGATTCAGGAAAACTCTGGGTTGAAAAGAGCCTGGAAGCAGCTTCAGTCTCAAGTTCATCTTTACCTAAAAAAGTAA
>EpfuTAS2R40_NW_007370745．1:4516840-4515884
ATGATGACGGTGAACAGCGATTACACGGATAAAGACATATCCAGGTTTAAAGTAGTCTTCATCTTGGTGGTCTCCGGAAT
CGAGTGCCTCGCTGGCATCGCTGGGAACGGCTTCATCACGGCCATCCACGGGGCCGAGTGGGCCAGACACAAAAGACTCC
CCGTGGGGGACTGCATTGTGCTGATGCTGAGCTTCTCCAGGCTCTTGCTGCAGATTTGGATGATGCTGGAGAATGTGTTC
AGCCTACTATTCTGGGCCACTTACAACCAAAACACAGTGTACACACCTTTCAAAGTCATCGTCCTGTTTCTGAACTACTC

CAACCTCTGGCTCGCCGCCTGGCTCACCATGCTCTACTGTCTTAAAATTGCAAACTTTACGCACCCTTTGTTCGTCAAGA
TGAAGAGGAAAATCACAGTGCTGATGCCCTGGCTTCTGAGACTGTCGCTGCTCATCTCCTTGGGCTTCAGCTTCCCCTTA
TCTAAAGACATCTTCAATGTGTATGTGAATAGTTCCATTCCTACCCCCTCCTGCAACACCACAGAGAAGACGTACTTCGC
TGAGACCAACGTGGTCAACCTGGTTCTTCTCTATAACCTGGGGGTCTTCATTCCCCTCATCATGTTCATCCTGGCGGCCA
CCCTGCTGATCATCTCTCTCAAGAGGCACACCCTGCACATGAAAAGCAGTGCCACTGGCTCCAGGGACCCCAGCATGGAG
GCCCACTTGGGGGCCATCAGAGCTATCAGCTACTTTCTCATCCTCTACATTTTCAATGCAGTTGCTCTATTTCTTTCCAT
GTCCAATGTCTTCAATGCCTACAGTTTCTGGGATATTTTATGCAAACTCATCACGGCCGCCTACCCTGCTGGCCACTCGC
TGCTGCTGATCTTGGGCAATCCTGGGATGAGAAGAGCCTGGAAGCGGCTTCAGCGCCGAGTTCACCTTCACCTATAA
>EpfuTAS2R41A_NW_007370745.1:4274641-4273715
ATGCAGCCAGCATTCACAGCCCTCTTCATGCTGCTCTTTGTTCTGCTGTGTTTCCTGGGAATCCTGGCCAATGGCTTCAT
TGTGCTGGTGCTGAGCAGAGAATGGAGGCGACTTGGGAGGCTGCTCCCTTCTGACATGATCCTCATTAGCTTGGGTGCCT
CCCGTTTCTGCCTGCAGAGGGTTGGAATGGTGCACAACTTTTACTCCTTCTTCCACCTAGGGGAATTCAGCAAGGATCCT
GCATGGCAGCTCTTTGTTCACCAACGGGACTTGCTGAATTCAGCCACCTTCTGGTTCAGTACATGGCTCAGTGTCCTCTT
CTGCATGAAGATTGCTAACCTCACCCACCCTACCTTCCTCTGGCTGAGGTGGAGGTTCCCAAGGTCAGAGCCCTGGCTTC
TGCTGGGCCCTCTCCTGATATCCACCATCGTCATCCTGCTCTTCTTGTGGAGAAACTACGCTATGGATCAAGGTTTCTTT
ATTAGAGAAGTTTATGAGAATATGACCTACATGGAGAGGGTCATGAAGATGGAAATTCACTATTTCCTACCCCTCAAATT
TGTCACGTTGTCAATTCCTTGGTCACTTTTTCTGGTCTCAACTGCATTGTTGATTCATTCTTTGAGGAGACACACTCAGA
CAATGCGGCAAAGTGCCCACAGCCTGCAAGACGCCAACACCGAGGCTCACACCAGAGCTCTGAAGTCACTCATCTTCTTC
CTCATTCTTGACATTCTGTCTTTCATGTCCCTGGTCATTGATACTATAGGTTTCTTTTCCACAGAGAGTGACTGGTTCTG
GCCATGGCAAATTGTAACCTACCTGTGCACATCTGTCCATCCCTTTATCCTCACCCTCAGGAACCCCAGGCTTCGAGAGG
TGTTCAGGCAGCTACTTCTGTTTGCCAGGGTCTTCTGGCTGGTCTAG
>EpfuTAS2R41B_NW_007370896.1:291822-290896
ATGCAGCCAGCCTTCACAGCTCTCTTCATGCTGCTCTTTGTCCTGCTGTGTCTCCTGGGAATCCTGGCCAATGGCTTCAT
TGTGCTGATGCTGAGCAGAGAATGGAGGCGGCTTGGGAGGCTGCTCCCTTCTGACATGATCCTCATTAGCTTGGGTGCCT
CCCGTTTCTGCCTGCAGTGGGTTGGAATGGTGCACAACTTTTACTCCTTCTTCCACCTGGGGGAGTTCAGCAGGGGTCCT
GCACGGCAGCTCTTTGGTCTCCAATGGGACTTCCTGAATTCAGCCACCTTCTGGTTCGGTACCTGGCTCAGTGTCCTCTT
CTGCTTGAAGATTGCTAACCTCACCCACCCTACCTTCCTCTGGCTGAAGTGGAGGTTCCTAGGGTCAGTGCCCTGGCTTC
TGCTGGGCTCTCTCCTGATCTCCACCATCGTCACCTTGCTCTTCTTTTGGGGAAACTACGCTGTGTATCAAGGTTTCTTC
ATTAGAGAAGTTTATGAGAATATGACCTACATGGAGAGGTTCATGAGGATGGAAATTCACTATTTCCTACCCCTCAAATT
AGTCACGTTGTCAATTCCTTGCTCTGTTTTTCTGGTCTCGACTGCATTGTTGATTCATTCTTTGAGGAGACACACTCGGA
CAATGCGGCAAAGTGCCCACAGCCTGCAAGACGCCAGCACCCAGGCTCACACCAGAGCTCTGATGTCACTCATCTCCTTC
CTCATTCTTTACATTCTGTCTTTCATGTCCCTGATCATTGATGCTGTAGGCTTCTTTTCAACAGAGAGTGACTGGTTCTG
GCCATGGCAAATTGTAACCTACCTGTGCACATCTGTTCATCCCTTTATCATTATCCTCAGCAACCCCAAACATCGAGAGG
TGTTCAGGCCACTACTTCTGTTGGCCAGGGGCTTCTGGTTGGTTTAG
>EpfuTAS2R42_NW_007370663.1:1875915-1874920
ATGCCCACTGGATTGGAAATAATCTTTCTGATACTGTCAATAGCAGCATTCATAATTGGAATGTTGGGGAACGTGTTCAT
TGGACTGGTAAATTGCTCTGAATGGGTCAAGAACCAAAACATCTCTTTAGCTGACTTCATCTTTACCTGCTTGGCTATCT
CCAGAATTAGTCAGTTGTTGGTATTACTTGTTGAATCATTTATATTTGGACTACCTTCATATTTATTTTCCACTCATAAA
CTAGCAAAACTTATTACTTTACTTTGGAGAATAACTAATCATTTGACTACCTGGCTTGCTACCTGCCTAAGCATTTTCTA
CCTCCTTAAGATAGCTCACTTCTCCCACTCTCTTTTCCTCTGGCTGAAGTGGAGAATGAACAGAGTGATTCTTGTGATTT
TTGTATTTTCTTTTATCTTTCTGATTGTTGACTTTCTATTGTTAGAAAGCTTTAATGATTTATTCTTGCATGTCTATATA
CAAGATTATAGTAATCTGACTTTGTATATGGAAGAAAGTAAGACTCTCTATTTTGAAACCCTGATTCTTCTTAGCTTGAC
CTGTTTGCTTCCTATTGTTCTGTCCCTGACCTCATTGCTCCTTTTATTTCTGTCTTTGGTAAGACATATCAGAAATTTGC
AGCTCAACTCCATGGGCTCCAGGGACTCCAGCACAGAGGCCCATAAAAGGGCCATAAAAATGGTGATGTCTTTCCTCTTC

CTCTTCATATTTCATTTTTTTTCCACACAAGTGGTAAATTGGACATTTCTTATGTTTCCAAGCCAGATGATTGTAAAGTT
TATCACGTTATTAGTCTATGTCTTTCCCTCAAGTCACTCATTTCTTTTGATCCTGGGAAACAGCAAGCTAAGACAGACAG
CCGTGAAGGTACTGTGGCATCTCAGAAGCCCCTTGAGAAGAGAAAATCTGTTACACCTATACAGACAGATTTCCCAGTGT
CTTTTCAAAGATAATAGCTTAACGAGGAAACTTTGA
>EpfuTAS2R408B_NW_007370663.1:1841963-1841046
ATGATAAGTTTACTACAAAAATTATTTTCTATGCTAATAATGACAGAATTTGTTCTAGGAAATTTTGCCAATGGCTTCAT
AGCGCTGGTGAACTGCATTGACTGGGTCAAGAAACACAAGATCTCCTGCCCTGATCGAATTCTCACTGCTCTGGCGGTCT
CCAGAATTAGTTTGCTCTGGATAATAGTATTCAATTGGTATGCAACTGTGTTTAATCTACCTTTCTGTAGTTCAGATGTA
AGACTTATGGTTTATATTGCATGGATAGTAAACCATCATTTTTGTCTCTGGCTTGCTGCTAGCCTCAGCATACTTTATTT
GCTCAAGATAGCCAATTTCTCCTGTCTTTTATTTCTTCACCTAAAATGGAGAGCTGAAAGAGTGGTTATCATGATACTGT
GGGGGAGTTTATTCATTTTGCTTTGTCATCTTACAGCGCTAAGCATAGTTGCAAAAATGGAAATGAATGATTATGAAGGA
AACATCACTTGGGAGACGAAATTGAGGGACATTATTCACCTTTCAAGTATGACTGTGTTCACGCTTGCAAACTTCATACC
CTTTACTGTGTCTCTGACAGCTGTTCTGCTGTTAATCTTTTCCCTGTGGAAACATCTGAAGAAGATGCAGCTCGGTGGCA
AAGGAAATCAAGATCCCAGCACCAAGGTCCACATAAGAGCCATGCAAACTGTGATCTCCTTTCTCTTGCTGTTTGTCATT
GATTTCCTCGCTCAAATCATCTCAATTTGGAGGTCTATAATTCAGCAGAATAATTTAGTTCTCATGCTTTGTGAGGTTCT
TGGAATCCTGCATCCTTCAGTCCACTCATTGATCCTTATTTGGGGGAACAAAAAGTTAAGACAGGCCTTTCTGTCATTTC
TGTGGCAGCTGAGGTGCTGTCTGAAGGAAAAGAAATAA
>EpfuTAS2R408C_NW_007370663.1:1858828-1857914
ATGGCTTTACTACCAACCATTCTTTCCAGCCTATTAACAATACAATGTGTTCTAGGATATTTTGCCAATGGTTTCATAGC
GCTGGTGAACTGCATTGACTGGGTCAAGAGACAAAAGATTTCCTGCGCTGATGGAATTCTCACTGCTCTGGCGGTCTCCA
GAATTGGTTTGCTCTGTGTACTAGCATTAAATTGGTATGCATCTGTGTTTAATCTACCTTTCTATAGTTCAGAAGAAAGA
CTTATTGTTCATATTGTCTGGATAGTAAGCCACCATTTTAGTTTGTGGTTTGGTACTAGCCTCAGCATATTTTATTTGCT
CAAGATAGCCAATTTCTCCAGCCTTTTATTTCTTCACCTAAAATGGAGAGCTAAAAGAGTGGTTATCATGATACTGTTGG
GGACTTTAGCCTTCTTGATTTGTCATCTTACAGTGCTAAGCATAGATGAAAAAATGGAGATGAATGATTATGAAGGAAAC
ATCACTTGGAAGACCCACTTGAGGGACATTATGCACCTTTCAAATATGACTGTATTCATTCTTGCAAACTTCATACCCTT
TACTATGTCTCTGACAGCTGTTCTGCTATTAATCTTTTCCCTGTGGAAACATCTGAAGAAGATGCAGCTCAATGGCAAAG
GATCCCAAGATCCCAGCACCGAGGTCCACATAAGAGCCATGCAAACTGTGATCTCCTTTCTCTTGCTGTTTGTCATTTTC
TTCGTGGCACAAATCATCTCAGTTTGGAATTCGAGTACTCAGCAGAACAATTCACTTCACATGGTTTGCAAGGTTCTTGG
AATCCTGTATCCGTCAGGCCACTCATTTATCCTGATTTGGGGGAATAAGAAGCTGAGACAGGCCTTTCTGTCATTTCTGT
GGCAGGTGAGGTGCTGGTTGAGGAAAGGGAAATAA
>EpfuTAS2R18A_NW_007370663.1:1771788-1770847
ATGTCGTTTGGAATAAAGGTCTCCATTCTAGTTGTGGAAACAGGAATACTCATCTTAGGAGTGCTAGGAAATGGATTCAT
CGGACTGGTGAACTGCATTGAATGGTTCAGGACTGGGAAAGTTTCCTCAGCTGATTTCATCCTCACCAGCTTGGCTCTGG
CCAGAATCATTCAACTGTTGGTAATACTCTTGGATTCATTTATACTAGGGCTAGCTCCACATCTGTATGCTATTGCTAAA
CTAGCAAAGGTGGTTACTATTCTTTGGGCACTAACTAATCAGCTAGCTGTTTGGATTGCCACCTGCCTCAGCGTTTTCTA
CTTCCTTAAGATCGCCAATTTCCCCCACTCCTTTTTCACGTGGCTGAAGTGGAGAGTCAACAGGGTGCTTCTTCTGCTTT
TCCTGGGGTCTTTCTTCCTACAGTCTCTTAACCTCTTAATGCACGATGCTATTACTGAATTCTGGTTGAATTCCTACAGG
GTACATGAAGTAAATATGACTTTGCGGTTTGAAGAAAATGAAATGCTCTATCTCAAAAGTCTTCTTCTTCTTACTTTGAC
CTATATTATCCCCTTTTTCCTATCCCTGATCTCTTTGCTCCTTTTAGTTCTGTCCTTGGTGAGACACACCAAGAATTTTC
AGCTCAACCTGGCGGGCTCAGGGGACTCAAGCACAGAGGCCCATAGAAGGGCCATGAAAATGGTGACAATGTTCCTCCTC
CTCTTCATCATTTACATTATTTCTATTCTCACAGCATGTTGGGTCTTCAGTAATTCCCAGACATATAAGATCAAGATGTT
TGTCATGATGATTTTTACTACCTTTCCCTCAGGCCACTCTTTCATTATAATTTTTGGAAACAGCAAGCTAAGACAGATCG
CCTTGAGACTACTCTCGCCCTTAAATTATTTGAGAAAAGCATCTAAAAGTCAAGTGAATTAA
>EpfuTAS2R67_NW_007370663.1:1866256-1865327

ATGCCATCTGGAACTGAAACTGTTTTTCTGGTAGCTGCAATGGGAGAATTCATAACTGGAATGTTGGGGAATGGGTTCAT
TGTACTAGTTAATTGCATTGACTGGGTGAGGAGTCAAAAACTCTCAGCAGCTGACTGTATCCTCACCAGCCTGGCTCTCT
CCAGAATCACTCTTCTTTGGATATCACTAGCTGACTCATTTCTAATGGTGTTGTGGCCACATTTTTATGCCATTGATAAA
CTAGCAAAACTTATTGGCATTTCTTGGATACTGTGCAATCACCTAGCTACCTGGTTTGCCACCTGTCTAAGCATTTTCTA
TTTCTTTAAAATAGCCAATTTCTCCCACCCCTGCTTTGCCTGGCTGAGGTGGAGAACTGGCAAAGTGCTACTTGTGCTTT
TACTGGGATCTTTGTTCTTCCTGCTTTTGAAACTTGTGTTAATAGATTCATTCAATGGTTTCTGGATTAAAGTTGAAAAT
ATGTATGAAAGAAACTCAACATGGTCTCCAGGTATAAGTGAAACTCTGTATCTTAACAGCTTGATGGTTTCTAATTTTAT
CTACTTAATTCCCTTTCTTCTGTCCCTGAGTTCACTGCTCCTTTTATTTCTTTCCTTGATGAGACATACCAGGAATGTGC
AGGTGAACTCCAGCTCTAGCGACTTCCGCACAGAAGCCCATAAAAGGGCCATGAAAATAGTGTTATCTTTCCTCCTCCTC
TTCATACTACATTTTTCTTCCGTTGTATTAACAGGTTGGTGTTTCCTTGTAGTGCAGAAGCACCAGGCTAATCTGGCTGT
CATGTTAACATGGACTATTTTCCCTTCGGGCCACTCATTTATCTTAATTTTGGGAAACAGCAAGCTGAGACAAACTGCGT
TGGGACTATTGCGGCATCTTAACTGCCACCTGAAAAAGGGTGAAACCTGA
>EpfuTAS2R13P_NW_007370663.1:1768654-1767757
ATGGTGAGTGCCTTGCATTGCTTCTTCACCATTTTAGTAAACACGGAATTTGTCATTGGTATTTGGGGGAATAGATTCAT
AATACTGGTGAACTGCATTGACTGGTTCAAGAAACGAAAGATCTTCTCAGTTGATGGAATCCTCACTGCTTTGGCGCTCT
CCAGAATTTGTCTGATTTGGGCAATAATGATGAGTTGGTTTGCAAAGGATATTTATCCATCTTCACACATGAATATAATG
GAAGTTGTACTTATTATTCTTAGGGTTGTGGCCAATCATTTTAGTAACTGGTTTGCCACCGGCCTCAGTCTCTTTTATTT
TCTCAAGATAGCCAATTTCTTAAATCCTGTCTTTCTTTGCCTGAAGCGTAGGTTGAAATGGTCGTTCCGGTAATGTCGCT
GCGAGCATTAGTGTTCTTATCTTTAAATCTTACTATAATAAGTCTGTATATTAATATCCAGATACATCCATGTGAAAGAA
GTAGGACTTTGAGTTCTAAAAAGAGCAACACTAAAAACATTTCAAAACTGATTGTACTCACTCTTGGAGCTCTCATACTT
GTTTCCATATCTCTGAATTTTTTTATCCTGTTCATCTTCTCCCTATGGAAACATCTCAAGAATATGAAGCACAGTGCAA
TGGGATTCAGAGATCCTCACCTCAAGGCCCATATAAGAGCCATGAAAAGTGTGATGTCTTTCCTGTTACTATTTGTTGTT
TATTTCCTGTCTCTTTGTATAACAATTTTCCATTCTGAGGTGATGCAGAACAAACTGGTTCTTTTGCTTGGCCAGGCTAT
TATAAATCTTTATCCTTCTGGCCACTCATCCTGATTCTGGGAAATGGTAAGCTCAGAAAGGCTTCCCTTTCGGTGCTGGG
CAGCTGAAGTGTTGTTGA
>EpfuTAS2R62P_NW_007370745.1:4303397-4302487
ATGCCCTCCTTGCTCTCATTGGTCTCCATGGTCATCTTCCTGAGTCATTGGCTGTAGTGTTGCAGAATGGCTTCATGGTC
ACTATGCTCAGTAGGGAGTGAGAACAATGTAAGACACTGCCCGCAGGTGACATGATTGTGGCCTGCCTGGTCACCTCCAG
TTCTGCCTACATGGGATGGCCCTCCTGAACAACCTCCTGGCCTCCTTTGAGTTTTGTTCTAAACTTTACCATTTCAACAT
CCCCTGGGCCTTTATGAACTCTCACTGTCTGGCTACTGCCTGGCTTACTGTCTTCGACTGTGTGAAGATCTCATTCTCTC
ACCCATCTTCTTCTGGCTCAAGTAGAGGATTTCTCAATCAGTGTTCAGGCTGCAGCTGAGCTCCATGATCCTATCTGGTC
TGACAGTCATTTCATCAGCCACTCGGAATACAATTCTGCTGCAGATAATTGCCTCTCAGAGTTCCCATGAAAACGGCACC
CTGGCTGATAGAATACAGACCTCTATTGGTAATTTTTCTACCTCATTAGTGCTGGTGACATTAATTCCATTCCTCCTGTT
ACTGGTGTTCACCCTCTTGTTCATGTTCATGTTCTCATTGCCTCGGCACTTGGGGCACATGAGGGACCACAGGCTTGGCC
TCTATGACCATAGTACCCAGGCCCACATGATGGCCCTGAAGTCACTTGCTTTCTTCTTCATCTTCTACACATTGTATTTC
ATATCTCTGATAATTATTGCTATGAAAATGACAACCTTCCAGAATCACTGGCACTGGACCTTGGAAGTGGTGACTTATGT
GGGTGTCTGCATTCCAGCATCCTGGTGCTAAGCAACCCCAACTTCAGAAAGGCCCTGAAAGATGAAGCTTTGAACAAAAG
GCACTTCATCTGAAGTTATCAGTATCAATAA
>EpfuTAS2R18BT_NW_007370663.1:181567-1815027
GCCACCTGCCTCAGCGTTTTCTACTTCCTTAAGATCGCCAATTTCTCCCACTCCTTTTTCACGTGGCTGAAGTGGAGAGT
CAACAGGGTGCTTCTTCTGCTTTTCCTGGGGTCTTTCTTCCTACTGTCTCTTAACCTCTTAATGCACGATGCTATTACTG
AACTGTGGTTGAATTCCTACAGGGTACATGAAATAAATATGACTTTGCAGTTTGAAGCAAATGAAATGTCCTATCTCAAA
AGTGTTCTTCTTATTATTACTTTGACCTATATTATCCCCTTTCTCCTGACCCTGATCTCTTTGGTCCTTTTATTTCTGTC
CTTGTTGAGACACTCCAAGAATTTTCAGCTCAACCTGATGGGCTCAGGGGACTCAAGCACAGAGGCCCATAGAAGGGCCA

TGAAAATGGTGACAATGTTCCTCCTCCTCTTCATCATTTACATTACTTCTCTTCTAACAGCAAGTTGGATCTTCACTAAT
TTACAGACACATCAAGTCATGATGCTTGTCACAGTGATTTCAACTGCCTTTCCCTCAGGCCACTCTTTTATTATAATTTT
GGGAAACAGCAAGCTAAGACAGATTGCCTTGAGGCCACTGTGGTACTTAAATTGTCTGAAAAAAGCAAAACCTTTGCCTT
TATAG
>PtalTAS2R1_NW_006438869.1:566362-565463
ATGCTAGAGTTTATTGTCATTACCCATCTTCTTTTTGTAATGATACAACTTCTCTTCGGGGTCTTAGCGAACGGCGTGAT
CGTGGTCGTGACCGGCACGGAGCTGACCAGGCCCAGAAAGATGGCGCCTCTGCATCTCCTTCTCTGCTGCCTGGCTGTTT
CCAGAATTTGTCTCCAGATGTTCATCTTCTACAACAGTCTGGTCGTTCTCTCCCTCATCGAATTCTTCTCGCTTGCCGAG
ACCTATACGGTGTTCATGTTTTTTAGTGAACTGTCGCTTTGGCTGGCCACATGGCTCAGCGTGTTCTACTGTGCCAAAAT
CGCCACCTTCGCTCACCCTCTCTTCTTCTGGTTGAAGCTGAGGATTTCGAGGTTGGTGCCATGGCTGATTGTCGGGTCCT
TGCTATACACGTCTCTCACTGCTGTTTTCCACAGAAAACATACATGGATTCTTTCCCAAAAACTCTGGCTGAGCCTTTTC
TCTCAGAATGCCACCACTCAAGGCAATGAAATGTCCACGTTACACTTTGCCATTCTGGTCGTTGAGTTCTTGTTGCCACT
ATTTATCTTCCTACTTTCTGCTCTGCTCCTGATATTCTCCCTGGGGAGACACGCCCAGCAGATGACGAGCATGGCGGCGG
TCAGTGGGCACACGGGGACGAGCGTCTACTCCAGCACGCTTCTATCCATGCTGTCCTTTCTGGTCCTCTACGTCTCTCAG
TATGTGATGGCTGCTTTAATCTTTTCTCAGATTTTCAAGAACAAGAACTTCACCTTTCTGTTCTGCTTCTTGGTGCTTGG
TTTGTACCTCTCTGGGCACTCTATTATCTTAATTTTTGCAAGTCCCAAACTGAAACAAAATGCAAAGAAGCTCTTCTTCC
ACAGCAAGTGCTGTCAGTGA
>PtalTAS2R3_NW_006434839.1:1094981-1094031
ATGGCGGGACTCACAGAGTGGGTATTTCTGGTCTTGTCTGCCACTCAGTTCTCTCTGGGAATGCTGGGGAATGGTTTCAT
CGGGGTGGTCAACGGCAGCGGCTGGTTCAAGAGCAGGAGAATCTCTTTGTCTGACTTTATCACCACTAACCTGGCTCTCT
CCAGGATTGCTCTGCTGTGGATTCTCATGTCTGATGCTGTCATACTGGTGTTCTTTCCCAAAGCACATGATGAAGGGGTG
GTCATGCAAGTGATTGATATTTTCTGGGCATTTACAAACCATCTGAGCATTTGGCTTGCCACCTGTCTCGGTGTCTTCTA
CTGCCTGAAAATCACCAGTTTCTCCCACCCCACCTTCCTCTGGCTCAAGTGGAGAGTTCACAGCGTGGTTGTGTGGATGC
TGCTGGCTGCGCTGCTCCTATCGTGTGGCAGTGCCGTGTCTCTGATTCACAATTTTAAGGTCTATTCTGCCCTCTGTGGA
ATCAATGGCACAGGGAATGTGACTGAGCACTTTAGAAAACTAATGAACGAGTACCAAGTGACCCATGTTCTTGGCACCCT
GTGGAACCTCCCGCCCCTCATCATGTGTCTGGCCTCCTACATTGTGCTCATCCTCTCCCTGGGGAGGCACGGGCGGCGGA
TGCAGCACAACAGCGCCAGCTCCAGCGATGCCAGCACTGAGGCCCACAAGAGGGCCACTAAAATGATGCTCTCCTTCCTC
TTCCTCTTCCTGCTTTACTTTCTTGCCTTTTTAATTACAACATCCAGTTATTTCCTACCAGGAACTAAGATGACTCAGAT
GATTGGAGAACTAATTACAATGATTTATCCTGCCAGCCACTCATTTGTCTTCATTCTGGGCAACAGCAAGCTGAAGCAGA
CATTTGTGGAGATGCTGTGGTGTGAGTCTGGTCGTCTGAAGTCTGGATCCAAGAGACCCTTTTCCCCATAG
>PtalTAS2R4_NW_006434839.1:1083937-1083038
ATGCGCCTAACGCCCTTTACCTCTGCCGTTATTTTCTCAATGGTTTTGAATGTCGCAGGACTCATTGTGAATCTGTTTAT
TGTGGTGGTCAACTACAAGACGTGGGTGGAAAGCCGCAGAAGCTCCTCTTCTGATAAGATCCTGTTCAGCGTGGGCATCA
CCAGGTTTCTGATGCTGGGGCTGTTCGTCCTGTACATTAACTACTTCCTCATTTCTTCAGATGCTGAAAGGTCAGTCCGC
ATATCCAATCTGTACCTGTTCTCTTGGCTGTTTTTGGACTCGAGCGGTCTCTGGTTTGTAACCTTGCTCAACGTCTTGTA
CTGCGTGAAAATCACGAACTTCCGACACTCAGTTTTTCTTCTGCTGAAACGAAATCTCTCCCCAAAGACCCCCAGGCTGC
TGCTGGCCTGCGTGCTGAGTTCCGCCTTTACCACGCTCCTGTACGCTGTGGTCAGTCAGACATTCCTTCCCAAATTGGCG
GCTGGGCGAAATGGCACAGTGTTCGACATCACTGAGGGCATCTTGTCTTTGGTGATCTCTCTGGGCTTGAGCTCGTTTCT
CCAGTTCACCATCAATGTGACTTCCGCCTCCTTGTTAATATATTCCTTGAGGAGACACATACAGACGATGCGGAGAAACG
CCAGTGGTTTCTGGAATCCCCAGACCGAGGCTCACGTGGGCGCCATGAAGCTGATGGTCTGTTTCCTCCTCCTCTACGTT
CCGTATGCAGCCGCTGCCCTCTTCCTCTACCTCCCTTCTGACGTAGAGGTGGGTTTGGAATTCAGATCCGCGTGTCTGAT
CATCTCTACCTTTTACTCTCCGGGACATTCTGTTCTCATTATTCTCACCCACCCGAAGCTGAAAACTAAAGCAAAGAAGA
TTCTCTGTTTCAACAAATAG
>PtalTAS2R5_NW_006434839.1:1071618-1070722

ATGCAGACCACTGCCCGAGGACTGCTGATGGTGGTGGCAGTCGCTGAGTTTCTGATTGGCCTGGTTAGCAACGGAGTCCT
CATATTCTGGAGTTTTAGAGAGTGCGTCAGAAAATGCAAGGGATCTTCGTACAACCTCATTGTCCTGGGCTTGTCCGGCT
GCCGATTACTCCTGCAGTGCCTGATCATGATAGACTTAAGCCTGTTCCCGCTTTTCCAGAGCAGCCTTTGGCTTCGCTAT
CTCAATGTCTTCTGGGTTATGGTAAGCCAGGCCAGCCTGTGGTTTGCCACGTTCCTCAGTGTCTTCTACTGCAAGAAGAT
CACGACCTTTCAACACCCCGTCTACCTGTGGCTGAAGCACAGGATCTACTGCCTGAGTCTATGGTGCCTTCTGGGTGCCC
TCATGATCAATGTGTTGCTTGTCGCCCACATCGACTTAAAGCCCTACAGCGCTTCCCAAGAAAACAGGAGCATGCTGTGC
CCCCTTTCAAGCTGGCACTATCTGTATATATTACAGCTCAATTCAGGAAGCGGGTTGCCTTTCCTGATGTTTCTTTTATC
CTCTGGGATGCTGATTATCTCTTTGTATAGACACCACAGGAAGATGAAGGTCCATATAGCTGGCAGGAAGGATGCTCGGG
CCACGGCTCACATCACCGTCCTGAAGTCCTTGCTTTGCTTCCTTATACTTTACATGGTATACGTAGTGGCCAGCCCCTAC
TCCATCTCCTCCAAGTCTCCTGTTAATCTCACCTCTGTCTTCATCTCGGAGATACTCATGGCTGCCTATCCTTCTCTTCA
TTCTGTCATATTGATTATGGGGAATCCCAGGATGAAACAGGCTTGTCAGAGAATCCTGCAGAAGGTAGTGTGTGCTTGGA
GGGCCTGCGGCCTGTGA
>PtalTAS2R7_NW_006432008.1:183494-182556
ATGTCAGATGAAGTAAAAAACACCTTAATGATCATAGCAGCTGGAGAATTTTCAATGGGAATCTTAGGAAATGCATTCAT
TGGCTTGGTGAACTGTATGGACTGGATCAAGAATAAGAAGATTGCTTCCATTGATTTAATCCTCACAAGTCTGGCCATAT
CTAGAATTTGTCTATTGTGTATAATACTATTAGATTGTTTTATATTGGTGCTGTATCCAGATGTCTATGCCACCGGTAAA
CAAATGAAAATCATTGACTTCTTCTGGACACTAACCAACCATTTGAGTGTCTGCTTTGCCACCTGCCTCAGCATTTTTTA
TTTCCTCAAGATAGCTAATTTCTTTCATCCTCTTTTCCTCTGGATGAAGTGGAGAATTGACAGGGTGATTCCTAGGATCC
TGCTGGTGTGCTTGGCCGTCTCTGTCATTATTAGCTTTCCTGTCATTGAAAATTTGAATGATGATTTCAGGCTTTGTGTC
AAGGCAAAGTGGAAAGCAAACTTAACTTTGAGATGCAGAGTAAATAAAGCTCAGTATGCTTCCATTAAGGTATGTCTCAA
CCTGTTAACACTCTTCCCCTTTTCTGTGTCCCTGATCTCATTTCTCCTCTTGATTCTCTCCCTGCGGAGACACACCAGGC
AGATGCGACTCAATGCCACAGGATGCAGAGACCCCAGCATAGAAGCTCATGTGGGAGCCATGAAAGCTGTCATCTCTTTC
CTCATACTTTTCATTGCCTACTATTTGTCCTTTCTTGTAGCCACCTCCAGCTACTTTATGCCAGAGACTGAATTAGCTGT
GATGATTGGTGAGTTGATAGCTCTAATCTATCCCTCAAGCCATTCTTTTATCCTAATTCTAGGGAACAATAAATTACGAC
AAGCATTTCTAAGGGTGCTATGGAAAGTAACATATATCCTGAAAAGAAGAAATTTCTAA
>PtalTAS2R9_NW_006432008.1:191893-190958
ATGCTAAATACAATGGAGGCAATATATATGATCTTGATTGCTGGTGAAATGACTATAGGAATTTGGGGAAATGGATTCAT
TGTACTGGTTAACTGCATTGGTTGGCTCAAAAAGAGAGATATCTCCTTGATTGATATCATCCTGGTCAGCTTAGCCATTT
CCAGAATCTGTTTGTTGTGTGTAATATTTTTAGATGGCATCATAACGCTTCTTTCTCCAGAAACATATGACCAAGATGAA
CTAATGAACATTTTGGATGTTTTCTGGACACTCTGCAATCATTCAAGTGTCTGGTTTACCTCTTGCCTTAGCATCTTCTA
TTTACTCAGAATAGCCAATATATCCCACCCATTTTTCCTCTGGATGAAGCTAAAGATTAACAGGATCATCCTTGGGATTC
TTCTTGTGTCTTTTCTCATCTCCTTAATTTTTAGTATTTCATTGAATGAGGGCTCCTGGAATTATTTCAAGGTCAATCAT
GAAGAAAACATAACTTGGGAATTCAAAGTGAGTAAAATCGCAAATGCTTTCAAACAGATTACCCTGAATCTGGGTGCTAT
ACCTCCTTTTGTTCTTTGCCTGATCTCATTTCTCTTGCTACTTTTCTCCCTTTTTAGACACACCAAGCAGATGAAATTTC
ATGCCACAGGGTTCAGAGACCCCAGCACAGAGGCCCACATGAGGGCCATAAAGGCAGTGATAATCTTTCTGATGCTCTTC
ATTATGTACTATGTAGCCTTTCTTGTAGTAACCTCTAGTCTTATGATTCCCCAGGGAAAATTAGCAATGATGTTTGGTGG
CATAATAACTGTCATTTTTCCATCAAGCCATTCATTCATCCTGATAATGGGAAACGGTAAGCTGAGGGAGGCTTTTCTGA
AGGTGCTAAGGATTTTGAAATGTTTCCACAAGAGAAGGAGCTTTTTTGTTCCTTAG
>PtalTAS2R16_NW_006434839.1:14259271-14260176
ATGATACCCATCCAACTCACTGTCTTCTTCATGGTCATCTATGCGCTTGAGTCCTTGACAATAATTGTGCAGAGTAGCCT
AATTGTTGCAGTGCTGGGCAGAGAGTGGATGCGGATCAAAAGGATGTCACCTGTGGACATGGTTTTCATCAGCCTGGGTT
TCTGCCGCTTCTGCCAGCAGTGGTCATCGGTGCTGTACAATTTTTGCTCCTATTTCAACCCTAACACCACATTTTGGTAC
ATATCAATCATCTGGGAATTTACTAATACTCTTACGTTCTGGTTAACCAGCTTGCTTGCTATTGTCTACTGTGTCAAAGT
CTCTTCCTTCACCCACCCCGTCTTTCTCTGGCTGAAGTGGAGAATTTTGAGGTTGATTCCCCAGCTACTGCTGGGCTCTC

TGCTGATTTCTTGTGTGGCAATCATCTTTTCAGTCATTAGGAGTCGCATCAAATTTCAGTTAATCTCCATGATGCATTTG
CCTGGAAACAACACTGTGACTGAGAGAATTAAGAAGCTTCTGCAGAATTTGTTGATATTTCAGCAACTGGTGTTGTTGGT
TATTCCTTTCCTCCTATTCCTGGCCTCCACCATCTCGCTCATAGCCTCATTGTGCCAACACTTGGGGCAGATACAACGTC
ACAACATTGGCCACTGCAACTCCAGCTTGAAAGCTCACTTTAGTGCCTTGAGGTATCTTGCCTTCTTCCTCATCTTCTTC
ACCTCTTACTTTCTGGCCATATTTATCACCATAGTAGACAATCCGTTTAATAGGAGACATTGGTTCTGGGCCTGGGAAAC
TGTCATCTATGCTGTGGTCTCTATTCACTCCACTTTACTGATGATGAGCAGCCCTAAATTGAAAAAGGTTTTAAAGGTAA
GGTGCTGGGGCCTAGAGACTGCCTGA
>PtalTAS2R38_NW_006434839.1:1010614-1011621
ATGCTGATGCTGACTCCCGTCGTCACTGTGTCCTACGAAGCCAAGCGCGCATTTCTGTGCCTTTCGATCCTGGAGTTTGT
GGTGGGGATTCTGGCCAATGCCTTCATTTTCTTGGTGAATTTTCGGGACGTAGTGAGGAGGCAGCCCCTGAGCAACTGTG
ACCTTGTCCTGCTGAGCCTCAGCCTCACGCGGCTCTTCCTGCATGTGCTGCTGTTTCTGTATGCCATCCAGCTTACCCAT
TTCCAGCAGATGAAAGACCCGCTGAGTGTCAGTTACCAAACCATCGTCATGCTCTGGATGGTCGCAAACCAAGCTGGCCT
CTGGTTCGCCACGTGCCTCAGTCTCCTCTATTGCTCCAAGATCGTCCGCTTCTCCCGCGCCTTCCTGCTCTGCTTGGCAA
AGTGGGTCTCCAGGAAGATGCCCCAGATGCTCCTGGGTACAACCCTTTTCACCACCGTCTGCACCGTCATCTGTTCTTGG
GACTATTTTAGCAGCTCTCACTTCACAGGCACAGCTATGCTTTTCATGAATAATGATACAGAGTTCCACCTGCAAATTAA
AAATCTCGGTTTCTTTCATTCCTTCCTCTTCTGCAGCCTGGGATCCGTCCCGCCTTTCTTGTGCTTTGCGGTTTCTTCTG
GGGTGCTGATCGTCTCCCTGTGGCAGCACATGAGGACGATGAGGGCCAAAACCAGAGACTCCCGGGACCCCAGCCTGGAG
GCCCACGTCAAAGCGCTCAAGTCTCTCGTCTCCTTCCTCTGCCTCTACGTGGTGTCACTCTGCGCTGCCCTCCTCTCGAT
GCCTTTACTGATACTGTGGCACAACAAGATTGGGGTCATGATCTGTGTGGGGATCATGGCAGCCTGTCCCTCGGGGCACG
CGGCCATCGTGATCTCAGGCAATGCCAAGCTGAGGGCGGCTGTGGACGCCATCCTGCTCTGGGCTCGGACCAGGCTCACG
GTGGGAGCGGAGCACCAGGCAGATCCGCTGGTGCCAGATCGATGCTGA
>PtalTAS2R39_NW_006434839.1:475210-474251
ATGACCAAAACCTGCAGTCCCCCAGAAAATGAGTTGTCATCATTCCACGTCATCTTAACTTTCACAATTATAGGCACTGA
ATGCGTTATTGGTATTGCTGCAAATGGGTTCATTATGGCTGTAAATGCAGCAGAATGGATTCAGAATAAGGCAGTTTCTA
CAAGTGGCAGGATCCTGTTTTTCCTGAGCGTATCCAGAATAGCTCTTCAAAGCTTCATGATGCTAGATATTGCCTGCAGC
TCAAAATTACCACGCCTTTATAATAAAGGTGTTTTATATAATACATTCAAAGTAAGTTCCATATTCTTAAATTATTGTAG
CCTCTGGTTTGCTGCCTGCCTCAGTTTCTTCTACTTTGTGAAGATTGCCAACCTCTCCCACCCACTTTTCCTCAAGCTGA
AGTGGAGAATTGCTAAGCTGATGCCCTGGCTTCTATGGCTATCGATATTCATTTCCTTGGGCTACAGCAGTCTCTTCTGG
AAAGGCATCTACTCTATACACTGTAGCAACTCATTTCCTGGCGCCTCGTCCAACTCCACTAAGAAAAAATTTTTCACTGA
GACCAACGTGGCCAACCTGGCTCTTCTCTACAACCTGGGGGTCTTCATTCCTCTGACCGTGTTCATCGTGGCGGCCACCC
TGCTGATCATCTCTCTCAGGAGACACACGCTACACATGGAGAGCAAGGGCACCGGCTCCGGGGACCCCAGCATGGAAGCT
CACATGGGCGCCATCAGAGCCATCAGCTACTTTCTCATTCTCTACATCTTCAACGCGGTTGCTCTGTTTCTGTCCATGTC
CAACATCTTTGACGCCAACAGTTCCTGGAATATTTTGTGCAAAACCATCATGGCTGCCTACCCTGCTGGCCACTCAGTGC
TACTGATCTTGGGCAATCCTGGCCTGAGAAGGGCCTGGAAGCGGCTTCAACACCAAGTCCATCTTTACCTAAAAGAGTAA
>PtalTAS2R40_NW_006434839.1:452142-451186
ATGTCAACAGTGAACACAGATGCCACGGATAAAGGCATGTCCAGGTTTAAAATCGTCTTCACCCTGGCGGTCTCCGGAAT
AGAGTGCATCACTGGCATTGCCGGGAACAGCTTCATCACGGCCATCCATGGGGCCGAGTGGGTCAGACGCAGAAGGCTTC
CTGTTGGTGACTGCATTCTGTTGATGCTGAGCTTTTCCAGGCTCTTGCTACAGATCTGGATGATGCTGGAGAATACTTAC
AGTCTACTATTCAGGGTCACTTATAACCAAAATGCAGTGTATATACCTTTCAAAGTCATCATCATGTTTCTCAACTATTC
CAACCTCTGGCTCGCCACCTGGCTTAACGTCTTCTATTGTCTTAGAATTGCAAACTTCACTCACCCTTTGTTCTCCCAGA
TGAGGAGGAAGGTCATGGTGCTGATGCCTTGGCTCCTGCGGCTCTCGCTGTTCCTCTCCCTGTGCTTCAGCTTCCCCTTC
TCCGTAGACATCTTCAATGTGTATGTGAACAGCTCCGTTCCAGTCCCCTCGTCCAACTCCACTGAGAAGAAGTACTTCTC
TGAGACCAACGTGGCCAACCTGGCTCTTCTCTACAACGTGGGGGTCTTCATTCCTCTGACCGTGTTCATCGTGGCGGCCA
CCCTGCTGATCATCTCTCTCAGGAGACACATGCTACACATGGAGAGCAAGGCCACCGGCTCCGGGGACCCCAGCATGGAA

GCTCACATGGGCGCCATCAGAGCCATCAGCTACTTTCTCATTCTCTACGTCTTCAACGCGGTTGCTCTGTTTCTGTCCAT
GTCCAACGTCTTTGACACCAACAGTTCCTGGAATATTTTGTGCAAAATCATCATGGCTGCCTACCCTGCTGGCCACTCAG
TGCTACTGATCTTGGGCAATCCTGGCCTGAGAAGGGCCTGGAAGCGGCTCCAGTGCCGAGTGCCTCTTTACCTGTGA
>PtalTAS2R41_NW_006434839.1:282390-281464
ATGCAGCCAGCACTCACGTCCCTCTTCATGCTGCTCTTTTTCCTGCTGTGTCTCCTGGGAATCCTGGCCAATGGGTTCAT
CGTGCTGGTGCTGAGCAGAGAGTGGAGGCAGCGTGGACGGCTGCCCCCCTCGGAACTGATCCTCATTAGCCTGGGCGCCT
CCCGCTTCTGCCTGCAGTGGGTTGGACTGGCGCACAACTTCTACTACTTCCTGCGCCTGGTGGACTATTCCAGGGGCCCA
GCGCAGCAGCTCTTCGGTCTGCACTGGGACTTCCTGAACTCGGCCACCTTCTGGTTCGGCACCTGGCTCAGTGTCCTCTT
CTGCGTGAAGATCGCTAACCTCTCCCACCCCACCTTCCTCTGGCTGAAGTGGAGGTTCCCGGGGTCTGTGCCCTGGCTGC
TGCTGGGTTCCCTCCTGGTGTCCTCCATCGTCACCATGCTCTTCTTCTGGGGCAACCGCGCCGTGTACCTCGGTTTCTTC
ATTGGAAGGTGTTCTGGAAACATGACCTACAAGGAGTGGAGCAGGTGGCTGGAAATTCACTATTTCCTGCCCCTGAAATT
TGTCACGTTGTCAGTCCCTTGCTCTGTCTTCCTGGTCTCGACGGCACTGTTGATTAATTCCCTAAGGAGACACACTCGGA
TGATGCGGCACAATGCCCACAGCCTGCAGGACCCCAGCACCCAGGCTCACACCAGGGCTCTGAAGTCACTCATCTCCTTC
CTTGTTCTCTATGCTCTGTCCTTTGTGTCCATAGTCATCGATGCTGCAGGCTTCTTCTCCTCAGACAGTGACTGGTACTG
GCCGTGGCAAATTTTAATGTACCTGTGTACATCTGTCCATCCCTTTATCCTCATCCACAGCAACCTCAGGCTTCGAGGGG
TGTTCAGGCAGCTACTTCTATTGGTCAGGGGCTTCTGGGTGGCCTAG
>PtalTAS2R42_NW_006432008.1:272209-271262
ATGCCCACAGGATTGGATATAATCTTTGTGATACTGTCAATAGCAGAGTTCACAATTGGAATTTTGGGAAATGTGTTCAT
TGGACTGGTAAACTGCTCTGAATGGGTCAAGAACCAAAAGATCTCTTTAGCAAACTTCATCCTTACCTGCTTGGCCATCT
TCAGAATCAGTCAATTGTTGGTATTATTGTTTAAATCCCTTATACTGGGGCTATCTCTACATTTACATTTAACTTATACA
ATAGCAAAGCTTATGAGTTTGCTTTGGAGAATAACTGATCACTTGACCACTTGGCTTGCTACCTGCCTAAGCATTTTCTA
CCTCCTTAAGATAGCTTACTTCTCCCACCCCCTTTTCCTATGGCTGAAGTTGAGACTGAATAGAGTGATTCCTGTGACTT
TTTTATTTTCTTTGTTTTTCCTGATTGTTGACTTTCTATTGTTAGAAATATTTAATGATTTTTTCTTAAACGTCTATATA
CTAGATAAAAGCAATCTTACTTTATTTATAGTTGAAAGTAAAACTCACTATGTTGAAACCCTGATTCTTCTTAGCTTGAC
CTGTTTCTTTCCCATTGTTTTGTCCCTGACTTCATTGCTCCTTTTATTTCTGTCCTTGGTAAGACACATCAGAAATTTGC
AGCTCAATTCCATGAGCTCAAGGGACTCTAGCACAGAGGCCCATAAAAAGGCCATAAGAATGGTGATGTCTTTCTTTTTC
CTCTTCATAGTTCATTTTTTTTCCATACAAGTAGCAAGTTGGCTATTTCTTATGACGTGGATCAACAAATTTGCAAAGTT
TGCTGTGTTAGCAGTATATATCTTTCCCTCAGGACATCCATTTATTTTGATTGTGGGAAATAGCCAGCTAAGACAGACAA
TCTTGAAGGTACTGTGGCATCTTAAAAGTTTCTCAAAAAGAGAAAATCTGTTACAGATTTACAGATAG
>PtalTAS2R408B_NW_006432008.1:238218-237259
ATGATAAATTTACTACAGAGCATTCTTTCCATCTTTGTAATAGCAGAATTTGTTCTAGGAAATTTTGCCAATGGCTTCAT
AGCACTGGTGAACTGTATTGATTGGGTCAAGGGACAAAAGATCTTCTTAGTTGATGGAATTCTCGCTGCTCTGGCGGTCT
CCAGAATGTGTTTGCTTTGGATATCAGTAATACACTGGTATGCAACTGTGTTTTCTCCAGCTTTATGTAGTTCAGAAGCA
AGACTTATTATTAATGTGATCTGGATAGTAAGCAACCATTTTTGTGTCTGGCTTACAACTAGCCTCAGCATACTTTATTT
ACTCAAGATAGCCAATTTCTCCAACCTTATATTTCTTCACCTAAAGTGGAGAGTTAGAAGAGTGATTCTCACGATACTGT
TGGGGACTTCGGTCTTCTTGGTTTTTCATCTTGTATTGGTAAGTATAAATAGAAAAATGTGGATGAATGAATGTGAAGGA
AACATCACCTGGAATATCAAATCGAGGGACATCATGCCCCTTTCGTATATGACTGTATTCACGCTTGCAAACTTCGTACC
CTTTGCTATGTCCCTGATGTCTTCGCTGCTGCTAATCTTTTCCCTGTGGAAACATCTCAAGAAGATGCGGCTTAGTGGCA
AAGGATCCCAAGATCCCAGGACCGAGGTCCACATAAGAGCCATTCAAACTGTGATCTCCTTTCTCTTGCTATTTCTCATT
CACTTCCTGATTCTGATCTTTGCAGTTTGGTATTTTAATAGTCTGCAGAATGACTCAGTCTTCTTCAGTGGTCAGGTCCT
TGCAATTGTTTATCCTTCAGGCCACTCATTTATTCTGATTTGGGGAAACAAGAAGCTAAAGCAGGACTTTCTCTCAGTTT
TACGGCAGGTGAAGTGCTGGCTGAACAAATGGAAAGCTCAACGCCATAGGTCAATAAGAGGTGCATCGTTTGTGTTCTAG
>PtalTAS2R372_NW_006432008.1:203662-202703
ATGTCAAGTGCAATTGAAAATGTTTTTATAATCATTGAACTTTTGGAATTTATAACAGGTATTTGGGGAAATGGATTCAT

CGTACTTGTAATCTGTGCTGACTGGGTCAAAACCAAGAAAATCTCCCTGTTAGATTTCATCTTCACAATCTTGGCCATCT
CCAGGATTGGCATGATATGCATGCTTTTTGAAGATAGCCTTAAAACAGTGTACTACTCAGGAATATTTGAAAATCACCTA
ATGATGATAGTAGTCAGTGATTTCTTCTGGGATCTGAACAACGACATCAGTACCTGGTGTGCCACCTCCCTCGGTGTCTT
CTATTTCCTCAAACTGTCCAATTTTTCCCACCCCTTCTTTCTCTGGCTGAAGTGGAGACGAGATAGAGTTGTCATCACCA
TTCTGTTGGGTTTCTTTCTCTCTTTGTTTGTTGGTCTTCTGAACATAAAATTTGATGCTTTTAAGGTCAGCGAATATTTA
AAAACAGAAAGAAACTGGACTCGGAAAGAATATATGCGTAAAACCCAGTACTTTAACAATAAAATTCTCTTGAGCCTGGG
ATCTCTCATTCCCATGGTTGTGTCGCTAATCTCTTTTTTCCTGTTAATCCTTTCCTTATGGAGACATATCCGGCAGATGA
TGCATTACGCCAAAGGATCTGGAGACTTTAACACAGAGGTTTATGTGAGAGCCAGAAATACTATGATTTCTTTCATCATT
CTCTTGGTTGTGCACTATTTTTTCACTATCCTGTTACTTTGGTCCTACTCCACAATAGAAAACTCACTAAGTATGATTAT
CTGTGAGACTGTAGTATTGCTGTATCCTTCAATTCACCCTATCCATATGATCCTGGGGAACAGAAAACTGAGACGGACTG
CTGTGAATTTGCTAAGGCAAATGAGTCCTGCATCAAGGGAACGTGATTCTTCACAGCACGCAGGAACTGAGAACTATTGA
>PtalTAS2R2P_NW_006436282.1:8517144-8518051
ATAACCTCTCTTTATCAGCTGTTCCTCATGCTACCATCATGTCAGCAGAATTTATTACGCAGATTACAGTAAATGGATTT
CTTATAATCATCGACTGGAATGAATTGATCAAAAACAGAAGCCTAACGCCAATACAACTCCCTTTAATATGTATCGGGAT
GTCTAGATTTTGTCTGCAGGTGGTATTCATGGTACAAAGCTTTTTCTCTGTGTTTTTTCCAGTCTTTTATCTGACAAAAA
TTTATGATGCAGCAATGATATTCCTCTGGATGATTTTTTTAGTTCTATCCGTCTCTGGTTTGCCACCTGCCTTTCTGTAT
TTTATTGTCTCAAGATTTCAGGCTTCACCCAGTCCTATTTTCTTTGACCGAAATACAGGATCTCAGCGTTAATGCCTTGG
ATGCTTCTGGGAAGCCTGCTGGCCTCCGTGAGCACTGCAGCTCTGTGTCTCTACGTAGATTACCCTAAAAACGAAGAGGA
TGATGTCCTCAGAAATGCCATGCTAATGACTAAAATCAAGATAAGGCCCATTAACGAAGTGCTTCTTGTCAGCTTGACAC
TTGTATTTCCTCTAGCCATATTTGCAATGTGCACTTTTATGTTACTCATTTCCCTCTAGAAGCACATGCTTCTTATGCAA
AACGGATTTGGTTTTAGAAATACCAGCACAGATGTCCATATAAACGCCTTAAGTACAATGGTAACATTCTTTTTCTTCTT
TATTTCTTATTTTGCCACCTTCATAACAAATAACGACATTCACTATTCCTTGCAGAAGTCAGCGCTTCTTTGTGGTGAAG
GACATAATGACAGCGCATCCCTCTGGCCATTCTGTTATAATAATCTTGAGTAGTTCTAAATTCCGACAACCATTCAGGAA
ACTTCTCTGCCTCAAAAAGAATCAATAA
>PtalTAS2R8P_NW_006432008.1:187950-187046
ATACTCAGTATAGAAGATAATGTCTTATCATGATAATAACTGGAGAGTCTATATTAGGAATTTTGGGAAATGGATACATT
AGACTAGTAAACTGGATTGACTGGGTTAAGAAAAAAAAAGATCTCCTCAACTGACTACATCTCCAGAATTCGTTTGCTCT
GTGTAATGGTGCTCAATGGCATCATAATGGTTTTATAGCCAGATGTTTATAAAAATGATAAGCTAAAAATAGTTGATACC
TTGTAGACACTCACCCACGACGTCAGTATGAGAACTGCCACCTGCTTCAACGTCTTCCATTTCCTCAAGATAGCCAATTT
CTGCTACCCATTTTTCTTCTGGCTGGAATGGAGAGTTTAGAGGGTGGTTTGCTGGATCCTGCTGCAATGCTTGCCATTTC
TTTGTTAAGCAGCCTAATGCTAGCAATGAGACCTAATTATGATTTTGAGTTTAAAATTGCAAAACATAAGAGAAACTTCA
CTGAATTGTTTCATGTAAGTGTAATTCAATACTTCAACCTGTTGTCATTCTTTAACCTCATAGCGATTGCCCCATTTACT
GTGTCATTGATCTCATTTTTTCTTTTAATTACGTCCTTATGGAGACACACTAAGTGAATGAAACTGAATGTTACAGGCTG
TAGAGATCCCAGCACAGAGGCCCACGTAGGAGTCATGAAAACTGTTACTTTGTTTCTCTTCCTCTTTTTTGTATACTATC
TTGCTCCTCTTTTGGCAACATTTAGCTACCTTATGAAGGAAAGAAAGTTAGCTGTGATATTCGGAGAGGTTATAGCAATT
CTCTACCCCTCAGGTCTCTCACTTATTTTAATTATTGGAAATAACAGACTGAGGCAGGCATCTGTCAGGATGCTGAGGTG
TGGAAAAACAGCATGTGTGATGTAA
>PtalTAS2R10P_NW_006432008.1:201711-200803
CGCAGTGGAAGGCCTCCTCATTTTTATGGCAGTTAGTGAATCAATACTGGGGATTTAAGGAATGGATTTATTGGACTTGT
AAACTGCACTGACTGTGTCAAGAATAAGGAGTTTTCTGTAATTGGCTTGATTCTCATTGGCTTAGCTCCTTCAAGACTTT
TTCTGATATGATAATAATTACAGATAGATTTATAAAGATATTCTCTCCAGATATATATTTATCTGGAAGTCTAATTGAAT
ATATTAGTTATTTATGGGTGATTATCAATCAATCAAGTATCTGGTTTGTCACCAGCCTCAGCATCTTTTTATTTCCTAAA
GTTACAAATTTTTCCCACCACATTTTTCTCTGATCGAAGGGTAGAATCAACAGGGCTGTTCCTTTTCTGATGGAATTCTT
GCTCATTTCATGGTTGCTTGCTTTCCTACAAAGTATAGAGATTATTAATGATCATAGAATGAGTAATAGAAACACAACGT

GGATGCTCAACATACATAAAAATAAATTCTTTGCTCAATCTGGGAGTCATTTTCCTCTGTGCACTATCCCTGATTACATG
CTTCTTGTTAATCATTTCCCTTTGGAGACACAGCAGGCATTGCAATTGAATGTCACAGGTTTCAGAGACTCCAACACAGA
AGCACATGTGAAAGCAATGAAAGTTTTGATATCTTTTATCATCCTCTTTGTCTTGCATTTTATAGGCATTGCCATAGAAT
TATTGTGTTTTACTGTGTCAGAAAACAAATTGCTGTTTATTTTTAGTATGATAATCACATTCCTCTACCCGTGGGGTCAT
CCGTGTGGTTATCTTAATTCTAGGAAACAGCAAGCTAAAGCAAGCCTCTTTGAAGGTAGTACAGCATTTAAAGTGCTGGA
CAAAAGAAGAACCTCTCAGGACTGCATAG
>PtalTAS2R12P_NW_006432008.1:210762-209819
AGGTCAAGCATACTAGAGACCTTGTTTATAATCATTTTTATAGTAAAATTCATAACAGAAAATTTGGGGAATGGATTCAT
TTTACTGGTAAACAATATTAACTTGATCAGGAACTAGATGGTCTCCATGATTAATTTTTTTTCTCACCTGCTTGACCTTC
TCCAGGATGTTTTCTGTGTCTGTTGATTTTAGATATCTCTTTCTCTATGGTCTATGAGAAAATATTTCACTCTAAGAATC
TAATGCTAAGTTTTGACATCCTCTGGATGAGATCTAACTATTTCTACATGACATATATCATTTGCCTTTGTGTCTTCTAT
TTCTTTAAAATAGCCAGCTTCTCTGACTCCATTTTCCTCTGGATAAAATGGAAAATTCACAAGGTGCTTCTCATTATTAT
ACTGGGAGTGATGCTCTTATTCTGTATGTATCTCCTTTTGAAGAAAATATTAATTAATAGACTGATTGAGAATAGGTAAA
AATGGAAAGAAGCTTGACATTCAAATTTATAGAGAATATATGTGATTTTTTAACATATCAGATTCTCCTGAACATGGTGT
TCATCATCATCTTCGTAGTGTCATTATCCCCTTTTCTCTTTTAATCCCCTCCTTATGGAGCCATACTCGTCAGATACAGG
ATATGTATTCTAAGGATTTTAGCTCGGAAGCCCATATAAAAGCTATGAGAGCTATGGTTCCATTCCTATTGCTCTTTATT
ATGTACTATTTTGAGCAATACCATGATAATGTTGACTCATTTTATTATAGACAATGAGATGGTAAAGATGTTTGACAACG
CATTAACATTTTAAAAATGCTTCTGGCCAATTGTTTGTACCAATTTAATGAAACAGGAAACTGAAACAGATCTCTCTCTG
TGTCCATAGAAAGCTAAAGTGTGCCTGAAAGAAGATAATTTCTCATTCTTATAAATATATCTGA
>PtalTAS2R13P_NW_006432008.1:220730-219831
ATGGTAAGAGCTTTGCATAGCTTCCTCATCATTTTAGTATATACAGAACTTATAATTGGTATTTTGGGAAATGGATTCAT
AACACCGGTGAACTGCATTGACTGGCTCAAGAAATGAAAGATCTCCTCAGCTGATCAAATTTTGACTGCTTTAGCGATCT
CCAAAATTTGTCTCATTTGGGTAATAATGATGAGTTGCTTTTCAAAGGAGTTTCATCTATCTTCATACATAAACAGAATG
GAGATTATACCTATTAGTATTGTTGGGGTTTGTGCCAGTTATTTTAGCAACTGGTTTGCCACAAGCCTCAGTCTCTTTTA
TCTTTTCAAGATAACCAATTTTTTAAATTCTGTCTTTCTTCATCTAAAGCATAGAGTTGAAATGGTGGTTCTGGTAATGC
TGCTGGGAGCATTAGCATTCTTGCCTTTAAATATTATTATGGTAAACATGTATATTAATATGCAGATACATTCCTATGAA
AGAAATATGACTTTGAGTTCTAAACAGAGTAACAAGGAAATCTTTTCAAAATTGATTGTATTCACTACAGGATCTTTTGT
ACCCTTCTCAATATCCCTGAAATTTTTTATCTTGTTAATCTTCTCCCTGTGGAAACATCTCAAGAATATGAAGCACAGTG
CAACGGGATTGAGAGATCCCAATGTCAAGGCCCATATAAGAGCTATGAAAAGTGTGATATCTTTCCTCTTACTATCTGTT
GTTTACTTTCTCATAGAAGTTTTCCATTCTGAGATGATGATACAGGATGAACTGGTCTTTTTGCTTAGTCAGGCTATTGC
AAATGTTTATCCTTCAGTCCACTCATTTATCCTGATTCTGGGAAATGGTAAGCTAAGAAAATCTTCACGTTTGGTGCTGT
GGCAGCTGAAGAGTGTTGAA
>PtalTAS2R408AP_NW_006432008.1:228015-227094
ATGATAAGTTTACTACTGATTATTTTCCCCATCCTACTAATGACAGAATTTGCTCTAGGAAATTTTGCCAATGGCTTCAT
AGCACTGGTGAACCTCATTGACTGGGTCAAGAGACAAAAGATCTTGTCAGCTGATGGAATTCTCACTGCGCTGGTGGTCT
CCAGAATGGGTTTGCTCTGGACAATAGTAATAGATAGATAGGTATGCCCCTGTGTTTTATCCAGCTTTATGTAGTTCAGA
AGCAAGAATTATTTTTAATGTGATCTGGACAGTAAGCAACTATTTTTGTGTCTGGCTTACGACTAGCCTCAGCATACTTT
ATTTGCTCAAGATAGCCAATTTCTCCAGCCTTATATTTCTTCACTTAAAGTGGAGAGTTAAAAGAGTGATTCTCACGATA
CTGTTCGGGACTTTGGTCTTCCTGGTTTTTCATCTTGTATTGGTAAGTATAGATAGAAAAATGTGGATGAATGAATGTGA
AGGAAGCATCACCTGGAATACCAAATCGAGGGACATCGTGCACCTATCGTATATGACTGTATTTACATTTGCAAACTTTG
TATCCTTTTCTATGTCCCTGACGTCTTCTCTGCTGCTAATTTTTTCCCTGTGGAAACATCTCAAGAAGATGCGGCTTAGT
GGCAAAGGATCCCAAGATCCCAGGACCGAGGTCCACATAAGAGCCATTCAAACTGTGATCTCCTTTCTCTTGCTATTTGC
CATTTACTTCCTGACTCTAATCTTCTCAGCTTTGAACATAAATACTCTGAAATATGAACCAGTTCTCATGTTTTGCCAGA
TTTTTGGAATCCTGTATGCTTCAGGCCACTCATTTATCCTGATTTGGGGAAACAAGAAGCTAAGACAGGTCTTTCTGTCG

TTTCTATGGCAGCTGAAGTACTGACTGAAAGAAAGGAAATAA
>PtalTAS2R408CP_NW_006432008.1:250019-249099
ATGATAAGTTTACTAATTATGAGCATTCTTTCCATCTTTGTAATAGCAGAATTTGTTCTAGGAAATTTTGCCAATGGCTT
CCTAGCACTGGTGAACTGTATCAACTGGGTCAAGGGACAAAAGATCTCCTTAGCCGATGGAATTCTCACTGCTCTGGCGG
TCTCCAGAATGGGTTTGCTCTGGGCATTAGTAATAAATTGGTATGCAACTGTGTTTAATCCAGCTTTATATAGCTCAGAA
GTAAAAATTATTGTCTATATGATCTGTACAGTAAGCAACCATTTTAGTGTCTGGCTTACAACTAGCCTCAGCATACTTTA
TTTGCTCAAGATAGCCAATTTCTCCAGCCTTATATTTCTTCACCTAAAGTAGAAAGTTAAAAGAGTAATTCTCATGATAC
TGTTTGAGACTTTGGTCTTCTTGGTTTTTCATCTTGCAGTTGTAAACATAGATGAAAATATGCAAATGAATGAATGTGAA
GGAAACATCACCTGGAATACCAAATCGAGGGACATGGTGCACATTTCAAATATGACTGTATTTATGCTTGTGAACTTCGT
ACCCTTTACTATGTCCCTGATGGCTTTGCAGCTGCTAATCTTTTCCCTGAGGAAACATCTCAAAAAGATGTAGCTCAATG
GCAAAGGATCCCAAGATCCCAGGACTGAGGTCCACATAAGAGCCATTCAAACTGTGATCACCCTTCTCTTGCTTCTTGCT
ATTTACTTCCTGACTCCGATCTTCTCAGTTTGGAATTCTAATATGATGAGGAACAAAACAGATTTCCTGCTTTTCAGAGT
TCTTGAAATCCTGTATCCTTCAGACCACTCATTTATCCTGATTTGGGGAAACAAGAAGCTAAGATAGGCCTTTCTGTCAT
TTCTGTAGCAGCTGAGGTGCTGGCTGAAAGAAAGGAAGTAA
>PtalTAS2R60P_NW_006434839.1:300826-299919
ATGAGGATAGTGTGGTTAAGAGAACCATCATCTTGGCTATCGTTTTATTCCTTTTGTGCCTGGTGGCTGTAGTGAACAAC
AGCTTCATCACTGCACCACTGGGCATGGAGTGGTTGCTGTGGAGAGCAGTGTCATCTTGTGATAAGTTATCCATGCCAGA
GAGCCTGAGGGCCCCTAGCTTCTGTCCGCAGTGGGTGGTGATCAGTAAGAGCTTCTGTGTTTTCCTGTATCCAAGGGCCT
TCCTGTACGCCCTGGGCTCCAGCTCCTAGCCTTCCAGTGGGACTTCTTGAATGCTGCCACCTTATGGTTTTCTAGTTGAC
TCAGTGTCTTCTATTGTATGAAAATTACAACCCTCACTCACTCTGTCTTCCTTTGGCTAAAGCGGAAGGTATTTAGGGGG
TTCCATGGGTGTTGTTCAGCTCTGCGAGGATTCTCTAGCTTGAGCATCATCTTATTTTATATAGGCAACCAGAGCATATA
TCAGAACTATTTAAGGAGAGGTCCACAATCTTGGAATGTCACTGGGAATACTATCAGAGAACTTATGAGAAATTCTACTT
TTTCCCTCTAAAAATGGTTACCTGGGCAGTCTCTGCCATCATCATCCTCATTTGCATGGTTTAGCTCATCACATGTCTGG
GAAGGCACGCCAAGAAGGCCTTCCAGCCATCTCAGGCTCTCACACTCCGGCGCCCAGGCACACATCAGGGCTCTCCAGGC
TCTCATCTCCTTTGCCTCCTCTTTCCTGTCACCGATGCTCGACGCTGCAGGTGGTTCCCCATCTCAGGACTGGGTGTGCA
GGCAGTGTTTTATCTGTGCACAGCGGCCCACCCCATCCTACTGTGAGCAGCCCCCAGGCTGAGAGCTGTGCTGGGCAGGG
CTGCTTCTTAAGGCGTGTGGCATCTTGA
>PtalTAS2R62P_NW_006434839.1:306540-305629
ACGCCCTCCTTGCCCACGGTGGTCTCCAAGGCCACCTTCTTCCTGGAGTCGTGGGCTGCAGTGCGGCTGAATGGCTTCGT
GGTGGCTGTGCTAAGCAGGGAGCGAGTGCAATGTCAGACATTGCCCTCAGGCGACCTGACTGTGGCCAGCCTGGCCACCT
ACCGGTTCTGCCTGCATGGGATGGCCCTCCTAACAAACCTCCTGACCTCCTTTGAGTTTTGTTCCAAAGTTTACTCTTTC
AACATCCCTTGGTCCTTTATCAGCACCCTCACTTTCTGGCTGACTGCCTGGCTGGCTGTCTTCCACCGTGTGAGAAGACC
TCATCCTTCTCTCTCCTACATCTTCTTCTGGCAGAAGAGGAGAACTTCTCGGTCAGGGCCTGGGCCATCGCTGGGCTCCC
TGATCATATCTGGTCTGACAAGCATCTCATCAGCCACTCGGAATATAGTTCTGGTGCAGGTCATTGCCGCCCAGTTCCCA
TGAAAACAGCGCCCTGGCTGATAGCGTACAGACCTTCCATGGGCACGTTTTCCTTCCTCATGTGGTGCTTGTGACATCGA
TCCCGTTCCTCCCGTTCCTGGTGTCCCGTTTTCCTGCTCATGTTCTCACCACACTGGCACTTGGGGCAGATGAGGGACCA
CAGGGCCCGGGGCTCACACCGAGGCCCTGAAATCACTTGCCTTCTTCCTCATCTTCTGCACCTCGTAGTTCCTGTCCCTG
GTTATCGTCGCTGTGAAAATCACAACCTTCCGGAATGACTGGCACCAGGCCTGGGAAGTGGTGACTTATGCAGGCATCTG
TCTGCACTCCAGCGTCCTGGTGCCGAGCAGCCCCACGCTTAGAAAGGCCCCGAAGACAAGGCTCTGGGAAGCCCTGGGCA
AAGGGCGGTTCATCTCAAGTTATCAGTGTCAA
>PtalTAS2R18P_NW_006432008.1:245956-244983
ATGTCAGTTGGAATGGAGGTCTCCTTTCTGGTAGTGGCAACAGGAGAATTCATCTTGGAAATGCTGGGAAATGGGTTTAT
TGAACTGGTAAACTGTATCGAATGGGTCAAGAGTGGGAAGGTCTCCTTGGCAGATTTCATCCTCACCAGCTTGGCTCTGG
CCAGGATCATTCAACCATTCATAACACTATTGGATTCATTCCTAATAGGGCTATCTCCACATCTGTATGCTATTGGTAAA

CTAGTGAAAGTGGTTACTATTCTTTGGGCACTAACTAATCACTTAACAACCTGGTTTGCCACCTGCCTAAGCATTTTCTA
CTTCCTTAAGAAAGCCAGTTTTTCCTACTTCTTTTTCATCTGGGTGAAGTGGAGAGTGAACAGTGGTTCTTATGCTTTTT
CTGGTGTCTTTCTTCTTATTGTCTATTATATCTCTTATGCAGGCTGCTCTTAGTGAGTTGTAGTTGAATACTTCTAGAGT
GCATGAAAGGAACATGACTTTGCATTTAGATGAAAATAAAATTTTCTACCTTAAAAGTCTTCTTCTTCTTAGCTTGACCT
GTGTTATCCCCTTTCTTCAGTCCCTGATGTCTTCACTCCTTTTATTTCTCTCCTTGTTGAGACACACCAAGAATTTACGG
CTCAACCTGATAGGATCAAGGGACTCTGGCACAGAGGCCTGCAGGAGGGCCATGAAAATGGTGACCATCTTCCTCCTCCT
CTTCATCATTTACTTTATTTCCACTCTAATAGCAAGTTGGATCTTTACTAAGGTACAGAGCTATTAGGCCATGATGCTGA
TCTCAACTATCTTTCCCTTGAGTCACTCATTTATTGTAATTTTGGGAAACAGCAAGCTAAGGCAGATCACCTGGAGACTA
CTGTGGCATCTTAAATTCTCTGATAAAAGCAAAACCTTTAGCTTCATGAGCAAAATTTGAAAGAACTTTGTGTATTCTAT
GGGACAGACCTTAA
>PtalTAS2R67P_NW_006432008.1:269668-268748
ATGTCATCTGGAATAGAAAATATTTTTCTGATAGCGGCAAAAGGAGAATTCATAGCTGGCATGTTGGGGAATGGCTTCAT
TGCACTAGTTAACTGCACTGACTGGGTGAAAAGTCAAAAATTCTCCGTAGACAATGGCATCTTCACCAGCTTGGCTATTT
CCAGAATAACTCTTCTTTTGATAGTACTGGTTGATTTACTTCTAACGGCGTTATGGCTACATCTATATGCCATTGGTGAA
CGAGCAAAATTTATTAGTATTTCTTGGGTACTGTCCAATCACCTAGCTACCTGGTTTGCCACCTGACTGTTTTCTACCTC
TTGAAAATAGCCAGTGTCGCTCACCCCTGTTTTGCCTGGCCGACGTGGAGAATTAGCAGAATGCTACTTGTGCTTCCACT
GGGGTTTTTGTTCCTACTGTTTTTCAACATCGCATTAACAGACACATTAAATGATTTCTGGGTTAACGCCTATAAAATAT
ATGAAAGAAACTCAATGTGGTCTTTAGATGTGAGTAAAATCCTGTATTTTAACACCTTGATTGTTTACGATTTTATCTAC
TTAGTTCCCTTTCTTCTGTCCCTGACTTCACTGCTCCTTTTATTTCTCTCCTTGAAGAGACACATCAGGAACGCGCGGCT
GAACTCCAGCTCTGGTGACTTTAGCACAGAGGACCATAAGAGGGCCATGAAAATGGTGATGTCTTTCCTCCTTCTCTTCA
AACTTCATGTTTCTTCCACTATATTTCCTTATGCTGCAGAGAGATCAGGCCAACGCGTTCGCCAAGTTCACATTGAATAT
TTTTCCTTCAGGTCACTCATTTATCCTAATTTTGGGAAACAGCAAGCTGAGACAAACTGGCTTAGGACTACTGTGGTATC
TTAATTGCCACCTGAAAAGGGTGAAACCTTTAGCTTCATAG
>BoinTAS2R1A_AGFL01164380.1:4288-5178
ATGCTGGAGTCTCACCTTGTTAGCCACCTTGTTTTGGCAGTGGTACACCTTCTCTTGGGGATTTTAGTAAATGGCATCAT
TGTGATTGTGAACGGTACTGACTTCATCAAGCAGAGAAAGTTGATCCCGCTGGATCTCCTTGTTTCCTGCTTGGCGATTT
CCAGGATGGGAATTCAGCTGGCCTTCTTCTACACTAACCTGGCTCTTCTTTCCTTGATCAAATTCCCTCAATTTACTGAG
ACGCTTGTAGTTTTCACATTTGTAAATGATTTGGGACTTTGGTTTGCCACCTGGCTCAGTGTCTACTACTGCACCAAGAT
TGCTACCATCGCTCACCCGCTCTCGTTCTGGTTGAAGATGAAGATCTCCAAGTTGGTTCCTTGGCTGATTCTTGTGTCCC
TGCTGTATGCATGTAGTACTTCTGCTATGCATGTCAAATATAAGTGGGTATTTTACGGAGAAGACTTCCTGGGCCTTTTC
TTCCCAAATGTAACAACTCACATCAAAGTAACCCCTACCTTACAGTTTGCCTTTCTGTTTGCTGAGTTTGCATTGCCATT
GTTCATCTTCCTGATTTCTTCTCTGCCCTTGATATTTTCCTTAGGAAGACATGCCTGGCAGGTGAGAAACACATGGACAG
GCCCCAGAAACCCTCACACACGTGCGTACATCAGGGCCTTTCTCTCCATCCTGTCCTTCTTGGCCCTCTATCTCTGCCAC
TACCTGATCATTGCTTTGATCTTTTTTCAAATTTTTAACCTCAGAAGCTTTCTATTTCTGTTCTGCACCTTCGTGGTTGG
TTCATACCACTCCGTCCACTCTATTACTTTAATTTTAGGAAACCCGAAAATGAAACAAAATGCAAAGGCATTGCTCCTCC
TCAGAAAGTGA
>BoinTAS2R1B_AGFL01164364.1:4282-5172
ATGCTGGAGTCTCACCTTGTTAGCCACCTTGTTTTGGCAGTGGTACACCTTCTCTTGGGGATTTTAGTAAATGGCATCAT
TGTGATTGTGAACGGTACTGACTTCATCAAGCAGAGAAAGTTGATCCCGCTGGATCTCCTTGTTTCCTGCTTGGCGATTT
CCAGGATGGGAATTCAGCTGGCCTTCTTCTACACTAACCTGGCTCTTCTTTCCTTGATCAAATTCCCTCAATTTACTGAG
ACGCTTGTAGTTTTCACATTTGTAAATGATTTGGGACTTTGGTTTGCCACCTGGCTCAGTGTCTACTACTGCACCAAGAT
TGCTACCATCGCTCACCCGCTCTCGTTCTGGTTGAAGATGAAGATCTCCAAGTTGGTTCCTTGGCTGATTCTTGTGTCCC
TGCTGTATGCATGTAGTACTTCTGCTATGCATGTCAAATATAAGTGGGTATTTTACGGAGAAGACTTCCTGGGCCTTTTC
TTCCCAAATGTAACAACTCACATCAAAGTAACCCCTACCTTACAGTTTGCCTTTCTGTTTGCTGAGTTTGCATTGCCATT

GTTCATCTTCCTGATTTCTTCTCTGCCCTTGATATTTTCCTTAGGAAGACATGCCTGGCAGGTGAGAAACACATGGACAG
GCCCCAGAAACCCTCACACACGTGCGTACATCAGGGCCTTTCTCTCCATCCTGTCCTTCTTGGCCCTCTATCTCTGCCAC
TACCTGATCATTGCTTTGATCTTTTTTCAAATTTTTAACCTCAGAAGCTTTCTATTTCTGTTCTGCACCTTCGTGGTTGG
TTCATACCACTCCGTCCACTCTATTACTTTAATTTTAGGAAACCCGAAAATGAAACAAAATGCAAAGGCATTGCTCCTCC
TCAGAAAGTGA
>BoinTAS2R2_AGFL01034737.1:8457-9365
ATGATCTCTTTGTCAGGTATTCCTCATGTTATCATCATGTCAGCAGAATTTATCACAGGGGTTACAGTAAATGGATTTCT
TATAATCATCAACAGCAATGAATTGGTCAAAAGCAGAAAGCTAACACCAATGCAACTCCTGTTCGTATGTATAGGGATAT
CTAGATTTGGTCTACAGACGGTGTTAATGGTACAAGGTTTTTCTCAGTGTTCTTTCCACTCTTTTATAGCGCAAAAATT
TATGGTACACCAATGCTGTTTTTTTGGATGTTTTTCAGCTCTGTCAGTCTCTGGTTTGCCACCTGTCTCTCTTTATTTTA
CTGCCTCAAGGTTACAGGCTTTACCCAGTCCTGTTTTCTTTGGCTGAAAGTCAGGATCTCAAAGTTAATGCCTTGGATGC
TTCTGGGAAGCCTGCTGACCTCTGTGAGCATTGCAGCTCTGTGTGTCAAGGTGGATTACCCTAAAATTGTGGATATTGAT
GTCCTCGGGAATGCCACAGCTAAGAGGACTAAACTCAACACAAAGCAAATTAATGAAGTTCTTCTCATCAACTTGGCATT
AATATTTCCTCTGACTATATTTATAATATGCACTGTTATATTATTAATTTCTCTCTACAAGCACACTCATCGRATGCAAA
ATGGACCTCTTGGTTTTAGAAACACCAGGACTGAAGCCCATATTAATGCATTAAGAACAGTGATAACATTCTTTTGCTTC
TTTATTTCTTACTTTGGTGCCTTCATGGCAAATATGACATTCAATATTCCTTATGGAAGTCATTGCTTCTTTGTGGTAAA
GGATATTATGGCAGCATATCCCTCTGGTCATTCAGTTATAATGATTTGGAGTAATTCTAAGTTCCAGCAACCAATCAGGA
GACTTCTCTGCCTAAGAAGGAGTCAATGA
>BoinTAS2R3_AGFL01041397.1:12189-13139
ATGTTGAGACTCAGCAATATGGGGTTTCTGGTTCTGACCACCATTCAGTTCATCCTGGGAATGCTGGGGAATGGTTTCAT
AGGGTGGGTCAATGGCAGCAGCTGGTTCAAGAGCAAGAGGATCTCTTTGCATGACTTTGTTATCACTAACCTGGCTGTCT
CCAGGATTGTTTTGCTGTGGATTCTCTTGATCGATGGTATTTTACTGGTGTTCTCTCCCAAACTACATGATGAAGGGATA
ATCATGCAAATTATTGATGTTTTCTGGACATTTACAAACCATCTGAACATTTGGCTTACCACCTGTCTCAGTGTCTTCTA
CTGCCTGAAAGTGGCCAGTTTCTCCCATCCTATGTTCCTGTGGCTCAAATGGAGAGTTTCCAGGGTGGTTGTATGGATGC
TGTTGAGTACCCTGCTGTTATCATGTTGCAGTGCCATCTCTCTGATCCGGGAATTTAAGATCTATTCTGTTCTTGGTGGA
ATTGATAGAACCGGGAATATGACTGAACTTTTTASAAAGAAAGAAAAAGAATATAAACTGATCCATGTTCTTGSGACTCT
GTGGGACCTCCCTCCCCTAGTCATATCGCTAATCTCCTACTTTCTGCTTATCCTCTCCCTGGGGAGGCATATGCGGCAGR
TGCATCAAGACTGTGCCAGCTCCAGAGATCTCAGTACCGAGGCCCACAGGAGGGCCATCAGAGTCATCCTCTCCTTCCTC
TTTCTCTTCCTACTCTACTATCTTTCCTTTTATGTTTTAACATCCAGTTATTTCTTACCAGCAACTAAGATGATTGCAAA
GATTGGAGAAGTAATTGCAATGTTATATCTTGCTGGCCACTCCTATGTTCTCATTCTGGGAAATAGTAAGCTGAAGCAGA
TGTTTGTGGCGATGCTCCRGTGTGAGCCTGGTTGTCTGAAGCCTGGATCCAAGGGATCTGTTTATCCATAG
>BoinTAS2R4_AGFL01041398.1:8379-9269
ATGCTTCGGATAGTCTTTTTTTCTTCTGTCGTTGTCTCTGAAATTTTAACTTTTGTAGGACTCATTGTGAATCTCTTCAT
TGTAGTGGTCAGTTACAAGACTTGCATCAAAAGCCACAGGATCTCTTCTTCTGACAGACTCCTGTTCAGTTTGGGCATCA
CCAGATTTTTTATACTGTTACTGAATATTGTTGTCATCATCTCTCCAAATGTGGAAAGGTCAGTCTCCTTATCCTATTTT
TTTCTGTCATGTTGGATGTTTTTGGACTGTAGTAGTCTTTGGTTTGTAACCTTGCTCAACGTCTTGTATTGTGTGAAGAT
TGCTAACTACCAACACTCAGTGTTTCTCCTGCTGAAACGAAATCTCTCCACCAAGATGCCCCGGCTGCTGCTGGTCTGTA
TGCTGCTTTCTGTCTTCACCACTCTCCTGTATGTTATGCTCAGACRGTTGGCACCCTCTCTTGAATTTGTGACTATGAGA
AATGGCACAGTATTTGACATCAATGAGGGACTCTTGTCTTTGGTGACTCCTTTGGTCTTGAGCTCATTTCTCCAATTCAT
CATTAATGTGACTTCTGCTTCTTTGTTAATCAATTCCTTGAAGAGACATATACAGAAGATGCAGAGAAGTGCCACTGTTC
TTTGGAATCCCCAGACTGAAGCTCATGTGGGTGCTATGAAGCTGATGATCTGTTTCCTCRTACTCTACATTCCATATTCA
GTTGCTACCCTGGTCCATTATCTCCCTCCTTCTATAGGGATGGATTTGAGAACCAAGTCTATTTATGTTATTATGTCCAC
CATTTACCCTCCAGGACATTCTCTTCTTATTATTCTCACACATCCTAAACTGAAAACAAAAGCAAAGAATATTCTTTGTT
TCAGTAAATAG

>BoinTAS2R10A_AGFL01051160.1:22198-23097
ATGCTGAGTATAGTAGAAGGCCTCCTCCTTTTTGTAGCAGTTAATGAGTCAGTATTGGGGGTTTTAGGGAATGGGTTTAT
TGGACTAGTAAACTGCATTAACTGTGTGAAAAATAAGAAGATCTCTACACTCAGCCTTATTCTCACTGGCTTAGCCTCTT
CCAGATTTTGTCTGATATGGATAATAACTACAGATGCATATGTGAGGGTGTTTTCTCCAGATATGTATTTGTCTGGTAAT
CTAAGTCAATATATAGCTTACTTATGGATAATTATGAATCAATCAAGTGTCTGGTTTACCACTAGCCTCAGCATCTTCTA
CTTCCTGAAAATAGCCAACTTTTCCCACTGCATTTTTCTCTGGCTGAAGGGTCACATTACTGAGATTCTTCTTCTTCTAA
TGGGATGTTTGCCCATTTCATGGTTATTTACTTTTCCAAACATTACAATGCCTTTTATTAATAATATTATGAAGAACAGA
AGCACAACCGGGTTGGTCACCATGCAGAAAAGTGAATACTTTATAAATCAGATTTTGTTCAATCTTGGAACATTTCTTGT
CTTTGTACTATGCCTGATTACATGTTTCTTAATAATCACTTCCCTTTGGAGGCACAACAGGAGGATGCAATTGAATGCCA
CAGGATTCAGAGACCCCAGTACAGAAGCACACATCAAAGCAATGAAGATTTTGGTGTCTTTTATCATCCTCTTTATCCTG
TATTTTGTAGGCACTGCCATACAAATATTAAGTGTGACAGTGCCTGAAAACAAACTGCTATTTATTTTTGGTATGACAAC
CACCATCCTCTATCCCTGTGGACACTCATTTATCCTAATTCTTGGAAACAGCAAGCTTAACCAAGCCTCTTTGAGGGTAC
TGAAGCTATTAAAGTGCTAG
>BoinTAS2R10B_AGFL01051158.1:28485-29417
ATGCTGAATATAGTGGAAGGCCTCCTCATTTATGTAGCAGTCAGTGAATCAGTATTGGGGGTCTTAGGGAATGGATTTAT
TGGAGTTGTAAGCTGCATTGACTGTGTGAAAAGCAAGAACATCTCTACTGTCAGCCTTATTCTCACTGGCTTAGCCTCTT
CCAGATTTTGCCTGATATGGATGATAATTACAGATGCATATATAAGGATATTTTTTCCAGATATATATTTGTCTGGTAAT
ATAAGTCAATATATAGTTTACTTAAGGATAATTATGAATCAATCAAGTACCTGGTTTGCCACCAGCCTCAGCATCTTCTA
TTTCCTGAAGATAGCCAATTATTCCCACTGCATTTTTCTCTGGCTGAAGTGTCACATCAACAGGGTTCTTCTCCTTTTCA
TGGGGTCTTTGCTTATTTCATGGTTATTTGCTTTTCCAAGCATTGCAAAGCCTAGTACCAATAATATTATGAAGAACAGA
AGCACAACCTGGCTGATCACCATGCATAAAAGTGAATACTTGACAAATCAGATTCTGCTCAATATTGGAGTCATTCTTGT
CTTTGTACTATGCCTGATTACATGTTTCTTATTAATCACTTCCCTTTGGAGACACAACAGAAAGATGCGACTGAGTGCCA
CAGGATTCAGAGATCCCAGCACTGAAGCACATATCAAAGCAATGAAGATTTTGGTGTCTTTTATCATCCTCTTTATCTTG
TATTTTGTAGGCACTGCCATACAAATATCAGGTAGTAGTACTATGCCTGAAAACAAACTGTTGTTCATTATTGGTATAAC
AACCAGACTCCTCTATCCCTGGGGACACTCATTGATTCTAATGCTAGGAAACAGGAAGCTGAAGCAAGACTCTTTGATGG
TACTGAAGCCATTAAAGTGCTGGGAAAAAGAGAAACTTCTTAGAATTCCATGA
>BoinTAS2R10C_AGFL01051162.1:1238-2167
ATGCTGAGTGTACTGGAAGGCCTCCTCATTTTTGTAGCAGTTAGTGAGTCAATATTGGGGGTTTTAGGGGATGGATTTAT
TGGACTTGCATACTTCATTGAATGTGTGAAGAACAAGAAGTTTTCTACTATCAGCTTTATTCTCATGGGACTGGCTACTT
CCAGAATTTGCCTGATAGGGTTAATAACTACCGATGGATTTGTGAAGATTTTTTCTCCAGAAATGTATTCCTCTGGTTAC
CTAATTGACTGTATTACTTACTCATGGGTAATTCTGAATCCAACAAGTGTCTTTTTTGCCACCAGCCTCAGCATCTTCTA
TTTCCTGAAGATAGCCAATTTTTCCCACCACATTTTTCTCTGGTTGAGGAGTGACGTCAAAAGGGTTCTTCTCCTTCTGA
TAGGATACTTGCTTATTTCATGGTTAGTTACTTTTCCACTAACTATGAAGATAATTAGTGATTCTAGAGCAAAGAATAGA
AGTGTAGTCTTTTCAGTTGAAGTGCATAAAGGTGAATTCTTTAGAAACCAGATTTTGCTCAATCTTGGAACCCTTACCAT
CTTCATACTATGCCTGATTACATGTATCTTATTGCTCATTTCCCTTCAGAGGCACAACCAGAGGATGCTACTGAATGCCA
CAGGATTCAGAGACCCCAGCACAGAAGCACATATCAAAGCAATGAAAGTTTTGATATCTTTTATCATCCTTTTTATTTTG
TATTTTATAGGCATTACCATAGAAATATCATGCACTACTATGTCAGAAAGCAAGCTGTTGTTTATTTTTGGTCTGACCAT
CACTGCCCTCTATCCCTGGGGACACTCATTTATCCTAATTCTAGGAAACAACAAGCTAAAGCAAGTTTTTTTGAGAGTAC
TGAAGCAATTAAAATGCTGGAAGAAAGAGAAGCTCCTCAGAACTCCTTGA
>BoinAST2R12_AGFL01051154.1:11243-12172
ATGGAGAGAACATTGAACAATATACTTACGATCATTTATGCTGGAGAGTTCTTACTGGGTATTTTGGGAAATGGATTCAT
TGTTCTGGTTAACTGTATTGATTGGATCAGGAGTAGGAAGTTCTCCCTGATTGACTTTATTCTCACCTGCTTGGCTATTT
CCAGAATATGTGTGCTGTGCATAATGATTTCAAGTACAGGTTTATATGTAATCTCTAAGGAAATACGGTACAATAAGAAT
CTCCTGATAAATTTGAGGTTCCTCTGGACAGGATCCAATTATTTCTCCATAGTCTGCACCACCTGCATCAGTGTCTTCTA

TCTCCTCAGAATAGCCAACTTTTCGAATTTCCTTTTCCTCTGGATGAAATGGAGAATTCACAAGGTGCTTCTCATTATTG
CACTGGGGGCTGTCTTCTCTTTCTGCTTGTGCCTTCTTCAAAAGGATGCGGTAGTTGAAAGCCGGCTCCAAAACCAGGTA
AACAGCGAAAACAATGTGACATTGGACTTTCTAATGATAAAATATGATTTGTTCCTTACCATAATGTTCCTCATCCCCTT
TGTAGTGTCCCTGGCCTCCTTTCTCCTTTTAATCCTCTCCTTATGTGGTCATCTCAGGCGTATGAACGGTGTAGACTGTA
GCTCGGAGGCCCATGTGAGAGCCCTGAAGGCTATGATTTCATTCCTACTCCTCTTCGTTCTATACTATTTGAGCACTATT
ATAACTGTGTGGGCCAATCACATTCTAGGTAGTTTCGTGGCAAAGATTTTTGTGAACATGCTGTTATTTTTCTGTCCTTC
TGGCCACACTTTGCTTCTGATTTTGTGGAACAGCAAATTGAAACAGGCTTCACTCTGTGTCCTAAGGAAGCTGAAGGGTT
ACATGAATCTAAGAAAACCTGCTCTTCCAAAAAGAAGCCTGAAGCGATGA
>BoinTAS2R13_AGFL01051153.1:3560-4471
ATGGAAGATTCCTTGGAAAACATCTTTATCACTTTAATAAATTCAGAATTCATAATTGGCATTCTGGGGAATGGGTTCAT
AACACTGGTGAACTGCACTGACGAGATCAAGATGCAAAAGGTCTCCTTGGCTGATCAAATCCTCACTGCTTTGGCAATTT
CCAGAATTGGTCTGATTTTGGTAATGATAGTGAGTTTGTTTACAAAGGAGTCTTATCCATCTTCATCTTTAGACATAAAG
GGAAATAAAGTCATACTTTTTAGTATTGCTGGGCTCTTGGCCAATCATTTTAGTGTCTGGCTTGTCACAGGCCTCAGCCT
CTTCTATTTCCTCAAGATAGTCAATTTTTCAAATGCTGTTTTTCTTCACCTAAAGTTTAGAATTGGAATGGTAGTTATGG
TAATGTTTCTGGGGACATTAGTATTGCTGCCTTTAAGTCTTACTCTGGTGAGTACCTATATTAATATCAAGATACATCCG
TATGAAAGAAATATGACTTTAAATTCTAAAAGGCATGACACTGAAACCTTTTCCAAATTAATTATATTCACCGTAGGATC
TTTCTTACCTTTTATTATATCCCTGAGTTGTTTTCTCCTATTAATGTTCTCCCTACTGAAACATGTCAAGAAGATGAGGA
GCCATGCAACAGGATTCAGAGATCCCAGCAGCAAAGCCTACGTCAGAGCCATGATCATGGTGATATCTTTTCTCATACTA
CTTGCCATTCACTTCCTATCTCATCTCATGACAACTTTTCATCACAATGTGATACAGAGTGAACTGGCCTTTATGCTTGC
TGAAGCTCTTAGAACTATTTACCCTTCAGTTCACTCATTTGTCCTGATTCTGGGAAATGACAAGCTAAGAAAAGCTTCAC
TTTTGGTGCTGTGGCAGTTGAGGTGTGGCTGA
>BoinTAS2R16_AGFL01040016.1:31289-30384
ATGACAACCAGCCAACTCTCTGTCTTCTTCATGATTATCTATATGCTCGAGTTCTTGACAATAACTGGGCAGAGCAGCCT
GATTGTTGTAGCGCTGGGCAGAGAGTGGGTGCAGACTCAAAGGCTGCCACCTGCGGACATGATTCTCATCAGCCTGGGCA
TCTGCCGCTTCTGTCAACTGTGGTCATCGATGCTGTACAACTTTGGTTCCCACTTCCACCCTAATTACAATTTTTGGTAT
TTCGGGATCATCTGGGAATTTACTAACATCCTTTCCTTCTGGTTGACCAGCTTGCTTGCTGTCTTCTACTGTGTCAAAGT
CTCYTTCTTCAGCCACCCCGTCTTCCTCTGGCTGAAGTGGAGAATTGTGAGATGGGTTCCTCGGCTGTTGCTGGGCTCTC
TGCTGATTTCTTGTGTGTCTACCATATTTCCAGCTACTAGTTATTACATTGATATTCAATTCATCGCCATGAAGCATTTC
CCTAGAAACAGCACCATGCTTGAGAGACTTGAGGCGTTCCTGTGGGATTTTTCCACACTGCACAAAGTAGTTGTGTTGGT
TATTCCTTTCCTCCTGTTCCTGGCCTCCACAGTCTTGCTCATGGCCTTATTATCCCGACATCTGAAGCAGATGAAAGACC
TTCACACAGGCTGCTCCAACTCCAGCCGGGAAGCTCACTCTGCCGCCCTGAGGTCCCTTGCCATCGTCCTCATCTTGTTC
ACCTTTTATTTTCTCACCGTGCTCCTCTCCATATTGGATGTCCTATTTAATAAAGAGTCCTGGTTCTGGGCCTGGGAAGC
TATCATCTATGCATTAGTCTCTATTCATTCTACTTTACTAATGCTGAGCAGTGTCAAACTGAAAAGAGTTTTAAAGGCAA
GGTGCTGGAGCCTAGAAGCTGCCTGA
>BoinTAS2R408A_AGFL01051151.1:2196-3113
ATGATAACTTTAATATCGAGCATTATTTCCATTCTAATGTTGGCAGAATTTGTTCTGGGAAATTTTGTGTATGGTTTCAT
AGCACTGGTGAACTGCAATGACTGGCTCAGGAAACAAAAGGTCTCCTTAGCTGATGGGATTCTCACTGCTCTGGCAGTCT
GCAGAATTGTTTTGCTCTGGACAATATTAATAAATTGGTATGCAACTATGTATAATCCAGCTCTATATAGTTTAAGAATT
GTTATCCGTGTTGCCTGGACAGTAAGCAACCATTTTAGTAACTGGCTTGCTACTAGCCTCAGTATATTTTATTTGTTCAA
GATAGCTAATTTCTCCAGCCTAATTTTTCTTCACCTGAAGTGGAGAGTTAAAAGTGTAGTTCTCATGATGATGTTGGGGA
CTGCAGTGATTTTGTTTTTTCAAGTTGCAGTGTTAAGTATAGATGAGACTATTCAGACAAGTGAATATGAAAGAAACATC
ACTGAGAAGACCAAATTAAGGGACATTTTACACCTTTCAAATATGACCCTGCTCACACTAACAAACTTCATACCCTTCAC
TATGTCTCTGATATCTTTTCTGCTGCTAATCTTTTTCCTGTGGAAACATCTCAGGAAGATGCAGCTCAACGGCAAAAGAT
CCCAAGATCCCAGCACCAAGGTCCACATAAAAGCCATGCAAACTGTCATCTCCTTTCTTTTCCTGTTTGCCACTTACATG

CTGACTGTAATTTTAACAATTTGGAATTCTAATGAGCTGCAGAAGGAACTGGTCCAAAGGCTTTTCCAGGCTCTTGCAAT
CACATATCCTTCAATACACTCATTTATCCTGATTTGGACAAACAGGAAATTAACACAGACCTTTCTGTCATTTCTGTGGC
AGCCAAGATGCTGGCTAAAAGTAAAAGGAACTAGGTAG
>BoinTAS2R38_AGFL01041408.1:8346-7339
ATGGTGACTCTGACTCACATCGTATCTGTGCCCTCTGAAGTCAGGAATGCATTTCTGTTCTTTTCAGTCCTGGAGTTTGC
AGTAGGGATCCTACTCAACGCCTTCATTTTCTTGGTCAATTTCCGGGACCTGGTGAGGAGGCAGCCACTGAGCCACTGTG
ATCTTGTCCTATTGAGTCTCAGCCTCACCCGGCTTGTCCTACACGGGCTGCTCTTTCTGAAGGCCATCCAGCTTACTCAT
TTCCAGCGGATGAAAGACCCGCTGAGCTTCAGCTACCAGACCATCATCGTACTCTGGATGATCGTCCACCAAGCCGGACT
CTGGCTCACCATGTGCCTTAGTCTCCTCTACTGCTCCAAGATTGTCCGTTTCTCTCACGCCTTCCTGCTCCATGCAGCAA
GCTGGATCTCCAGAAAGATCCCCCAGATGCTTCTGGGTGCTATGGTTCTCTCCTGTGTCTGCACTCTTCTCTGCTTATGG
GACTTTTTTAGTGGATCTCATTTATCAGCTGTAACTAGGCTACTCATGAATAACAGTACTGAACTCAATTTGAACATTGC
AAAACTCAGTTTCTTTCATTCCTTCCTCTTCTGCAGCCTGGCATCCATCCCTTCTTTCTTGCTTTTCCTGGTTTCCTCTG
GGATGCTGGTGTTCTCCCTGGGGAGGCATATGAGGATGATGAGGGCCAAAACCAGAGGCTCTGGGGACCCCAGCCTGGAG
GCTCACACACGGGCGCTCAGGTCTCTTGTCTCTTTCTTCTGCCTGTATGTGCTGTCACTCTGTGCTGCCTTATTCTCGAT
ACCGTTGCTGATGCTGTGGCACAGCAAGGTCGGGGTGATGGTCTGCATAGGGATAATGGCAGCCTGTCCCTCAGGACATG
CAGTCATTCTGATCTCAGGGAATGCCAAGCTGAGGAGGGCTGTGGACACCATTCTGCTTTGGGCAAAGAGCAGCTTCAAG
GTAAGGGTGGACCACAAGGCATATCCCAGGACGCCAGATCTGTGTTGA
>BoinTAS2R39_AGFL01041560.1:799-1842
ATGAGTGGGAGCTATCACAGACCAGCACACCAAGTGCTAAGGAGACATTTTCCTCCAGACATTGAAGAAAAGCAACCACT
CAGGATGATCCAAACCTGCAGTTCCTCAGAAAATGATCTGTCACCATCTCTTGTCACTTTGATGTTAATAATTATCGGCA
CGGAATGCATCCTTGGTATCCTCGCAAATGGGTTCATTGCAGCGATAAACACAGCTGAATGGATTCACAGTAAGGTACTC
TCCACCAGTGGCAAGATCCTGCTTTTCCTGGGTGTATCCAGAATAGTTCTACAAAGCTTCATGATGCTAGAACTTACCTT
AAGCTCAACATCCCCACAGTTTTATAATGATGACATCATGTATCACACATTCAGAGGATGTTTCATGTTCTTAAATCACT
GCAGCCTCTGGTTTGCTGCCTGGCTCAGTGTCTTCTACTTCGTGAAGGTGGCGGATTTCTCCTACCCCCTTTTCCTCAAG
CTGAAGTGGAGAATTTCCGGACTGATGCCCTGGCTTCTGCAGCTATCAGTGTTTGTTTCCTTGGGCCAGAGTGTGCTCTT
CTTCCAAAACAACTATACTATGAATTGTAACAATCTTTTTTCTCTCCCGTCCTTCAACTCCACTAAGAAAAAGTCCTTCG
CGGAGTCCGCTGTGATCAACCTGGTTCTTTTCCTTAACCTGGGGATCTTCATCCCTCTGATCATGTTTATGCTGGCGGCC
ACCCTGCTGATCATCTCTCTCAAAAGACACATCTTCCACATGAAAAGCAATRCCACTGGCTCCAGAGACCCCAGCATGGA
GGCTCACCTGGGGGCCATCAGAGCCATCAGCTATTTTCTCATTCTCTATATTTTCAAAGTACTTGCTCTCTTTCTCTACA
TGTCCAACTTCTTTGACATCAATAGTCCCTTGAATATTTTGTGCAAAATCATCATGGCTACCTACCYTGTGGGCCATTCC
ATTCTACWGATTCAGGACAATCCTGGGCTGAAAAGAGCCTGGAAGAGGCTTCAGACTCAAGTTCACCTTTATTTTAAAAA
GTAG
>BoinTAS2R41_AGFL01041586.1:46493-47386
ATGCATCCAGCATTCACAGTCCTCTTCATGCTGCTCTTTGTCCTGCTGTGTATCCTGGGCCTCCTGGCCAATGGCTTCAT
TGTGCTGGTGCTGAGCAGAGAATGGGTGCGACGTGGGAGGCTGCTCCCCTCTRACCTGATYCTCTTTAGCTTGGGACTCT
CCCGCTTCTGCCTGCAGTGGGTTGGAATGGGGAATAACTTCTACTATTTCCTGCATCTGGTYGACTACTGCAGTGGTCCC
GCCCGGCAGTTCTTTGGTCTACCCTGGGACTTCCTCAACTCTGTCACCGCCTGGTTTGGCTCCTGGCTCAGCGTCCTCTT
CTGCATGAAGGTTGCTAACTTCACCCACCCTGGCTTCCTCTGGCTAAAGTGGAGGTTCCCCAGGTCAGTGCCCTGGCTTT
TGCTGGGCTCTCTCCTCACCTCCTTCATTGTCACCCTACTGTTTTTTGGGGGGAACCACGCTTTGTATAAAGAGTCCTTC
ACTAGAAAACCTTTCGGGAATATGACCTACTATCAGTGGAACAGGATTCTGGAAATGTACTATTTCCTGCCCCTGAAACT
GATCACCTTTTCAATTCCTGGCTCTGTTTTTCTGGTCTCGATTGCTCTGTTGATTGACTCTCTGAGGAGACACGCATGGA
GGATGCAGCACAGTGCTCACAGCCTGCAGGACCCCAGTGGCCAGGCTCACACCAGAGCTCTGAAGTCACTAGTCTCCTTC
CTTGTTCTTTATACTCTGTCTTTCATGTCCCTGATCATCGATGGTGAAGGGTTCTGCTCCTCAGAGAGTGACTGGTACTG
GCCATGGCAAATTTTACCTACTCGTGCACATCCATCCATCCCTTTATCCTCATCCTTGGCAACCTCAGGCTTCGGGGGGC

ATTTGGGCAGCTGA
>BoinTAS2R42_AGFL01051145.1:44140-45069
ATGTTCCCTGGGTTGAGTACCATCTTTCTGATACTATCAGGAGTGGAATTCTTAATCGGAATTCTAGGCAATGTGTTCAT
TGGACTGGTACTCTGCTCTGAATGCGTTAAGAACCAAAAGACATCTTTATTTGACTTCATTCTCACTGGCTTGGCTATCT
CCAGAATCAGTCAACTGTTGGTGTTTTTTGTGGAGTCACTTATGATGGGACTAGATTCACAGGTATTTGCCATTTTTAAA
CTAGCAAAACCCATTACTTTACTTTGGAGAATATCTAATCATTTGACTACCTGGCTTGTCACCTGTCTAAGTATTTTCTA
TCTCCTTAAGATAGCTCATTTCTCCCACTCTCTTTTTTTCTGGCTGAAGTGGAGAATGAACAGAGTCATTCTTGCGATGC
TTGCATTTTCTTTGGGCTTTCTGATTTTGGATATTCTTTTGCTAGAAACATTTAATGATCTCTTCTGGAATTTAATAAAT
GAAGGCAATTGGACTTTAGTTGAAAGTAAAACTCATTATATTAAAAGCGAGAGTCTTCTTAGTTTCTCCTATTTCATTCC
TATTGTTCTGTCCCTGCTCTCATTGTTTTTTTTATTTTGGTCCTTGGTGAAACACACCAGAAATTTGCAGCTCAATTTTA
TGGGTTCCAGGGACTTCAGCACAAAGGCCCATAAAAGAGCCATGAAAATGGTGACGTCGTTCCTCCTCCTTATTATGGTT
CATTTTCTTTTTACACAATTGGCAAATTGGATGTTTCATAGGTTTTTGGACAATAAGTTCACAAAGTTCATCATGTTAGC
ACTATATGTCTTTCCTTCAGGCCACTCGTTCATGTTGATTCTGGGAAATAGCCAGTTAAGACAGATAGCCTTGAAGGTAC
TGAGGCATCTTAAAAGCTCCTTGAAAAGACAAAATCCATTGGCTTTATAG
>BoinTAS2R408B_AGFL01051149.1:9063-9980
ATGGTAACTCTACTATCAACCATTTTTTCCATCCTAGGAATAATACAATTTGTTCTGGGAAATTTTGCCAATGGCTTCAT
AGCCCTGGTGAACTGCATTGACTGGGTCAAGAGACAAAAAATCTCCTCAACTGATGTGATTGTCACTGCTATGGCAGTCT
CCAGAATTGTTTTGTTCTGTGTAATGTTAATACATTGGTATTATATTTTGCTTCATCCAGCTTTATATAGTCTAAAAGTA
AGAACTATTTTTCATGTTGCCTGGACAATAAGCAATCATTATAGCACCTGGCTTGCTACTAGCCTCAGTATATTTTATTT
GTTGAAGATAGTCAATTTCTCCAGCCTAACTTTTCTTCACCTGAAGTGGAGAGTTAAAAGTATAGTTCTCATGATGCTTC
TGGGAACTTCATTCATTTTGGTTTTACAAGTTGTAGTTATAAGCGTAAGTGGGACTATGCAGAGAAGTGAATTTGAAGGA
AACTTCACACAGAAGACCAAACTCAGGGATATTTTATGGCTTTCACATGTGACCCTGCTCATTCTAGGAAACCTCACACC
CTTTACTATGTTCTTAATATCTTTTCTGCTACCAATCTTTTCCCTGTGGAAACATCTCAGGAAGATGCAGCTCAATGGCA
AAGGATTCCAAGATCCCTGTACGAAGGTCCACATAAAAGCCATGCAAACTGTCATCTCCTTTCTCTTGCTATTTGCCTTT
TACTTTCTGGTTCTAATAATATCAATCTGGAGGCCTAAAAAACTGCATGAGGAACCATTTCTCTTGCTTTTCCCAACAGT
CAAAGTCATCTATCCTTCAGTCCACTCATTTATCCTGATTTGGGGAAACAGAAAGTTAACACAGGCCTTTCTGTTGTTTC
TGTGGCAGCTGGGGTGCTGGCTGAAAGAGAGAAAATAG
>BoinTAS2R60_AGFL01041586.1:16245-17183
ATGGTTCCTGGACCTCAGTTGGTTGATAAGACAGCCCTTGTCTGCATTATTATTTTATTCCTTTTGTTCCTGGTGGCATT
GGTAGGTAATGGCTTAATCATCATGGCACTGGGCAGCAAGTGGCTGCTGCAGAGAACGTTGTCGCCTTGCGATAAGTTAT
TGGTCAGCCTGGGGGCCTCTCGCTTCTGTCTGCAATGGGTGGTGATTAGTAAGAACATTTACATTTTCCTGAATCCCACG
GCCTTCCCATACAACCCCGTGTTCCAGCTCCTGGCCGTTCAGTGGGACTTCTGGAACTCTGCAACACTGTGGTTCTCCAC
CTGGCTCAGTGTCTTCTACTGTGTGAAAATTGCCACCTTCACCCACCCCGTCTTCCTCTGGCTAAAGCGGAATGTATCTG
GCTTGGTTCCTTGGATGCTACTCAGCTCTCTGGGGTTCTCTACCTTTACCACCGTTCTATTTTTCATAGGCAACCATAGA
ATGTATCAGAACTATTTAAAGAAGGGTCTGCAACCTTGGAATGTCACTAGGAATGCTGTGAGAACATATGAGAGGTTCTG
CCTCTTCCCTTTGAGAATTGTTACCTGGACCGTCCCTACTGTTATCTTTATTGTGGGCACAGTTTTGCTCATTACATCTC
TGGGAAGACACACCAAGAAGGTCTTCTTCTCCATCTCAGGCTTTCACAGTTCCAGYGCCCAGGCACACRTCAAGGCTCTC
TTGGCTTTTATCTCCTTTGCTATCTTCYTCACTTCCTCTTTTCTGTCACTGGTTCTCACTGCCTCAGGTATGTTTCCTTT
TGGGGAATTCCGGTTCTGGATATGGCAGACTGTGATTTATCTGGGTACAGCAATCCACCCCCTTATTCTTCTCTTGAGTA
ACCGCAGGCTGAGAGCTCTGCTAGGGAGGGGCTGCTCCTCAGCACATGGGGCATCTTGA
>BoinTAS2R18A_AGFL01051152.1:9228-10115
ATGTCTGACTCTGTGCGACCCCATAGACAGCAGCCCACCAGGCTCCCCCATCCCTGGGATTCTCCAGGCAAGAACACTGG
AGTGGGTTGCCATTTCCTCCTCCAATGCATGAAAGTGAAAAGTCAAAGTGAAGTCGCTCAGTTGTGTCTGACTCCTAGCG
ACCCCATGGACTGCAGCCTACCAGGCTCCTCCATCCATGGGATTTTCCAGGCAAGAGTACTGGAGTGGGGTTGGGCACTA

ACAAATCACTTAACTATCTGGTTTGCCACCTGCCTAAGCATATTCTACTTCCTTAAGATAGCCAGTTTCTCTCACTTCTT
TTTCATGTGGCTGAAATGGAGAATGAACCGAGTGCTTCTGGTGCTTTTCCTGGCCTTTTTCTTTTTATTATCTTTTGACC
TCTTAATGCAGGATGCTCTTGGTGAGTTGTGGATGAACACCTTTAGAGAACCTGAAAGGAACATGACTTTGCATTTAGAT
GCAAGTAAAATTTTCTATCTTAAAAGCCTTATTCTTCTCAGATTGACATATGTTATCCCTTTTCTTCTCTCCTTGGCTTC
TTTCCTGCTTTTCTTTCTTTCCCTGGTGAGACACATCAAGAATTTCCAAGTCAACTTGAACCACTCGAGAGACTTCAGCA
CAGAGGCCGATAAAAGGGCCACGAAAATGGTGACAACATTTCTCCACCTCGTCATCGTTTACTTCATTTCCATTCTAATT
GGAGAATGGATCTTCTTTAAGCTACACTGGTATGAGGTCATGATGTTTGTCATGGTGATTCCAACTCTCTTTTCATCCGG
CCACTCGTTTGTTATAATTTTGGGAAACAGCAAGCTAAGGCAGATTGCTTTCAGACTATTGTGGGGTCTTACGTTCTCTA
AAAACTAA
>BoinTAS2R67_AGFL01051146.1:7113-8051
ATGCCATCTGGAATTGAAAACACTTTTCTAGCAGCAACAATAGGAGGATTCCTGATTGGAATTTTGGGGAATGGGTTCAT
TGTACTAGTTAACTGCATTGACCTGGTGAAGAGACAAAAGCTCTCATCAGCTGACTGCATCCTCACAGGCCTGGCTATCT
CCAGAATCAGTCAACTTTGGGTAATACTATGTGACTCATTTTTATTGGTACTATGGCCACACCTATATGCCATTGATAAA
CTAACAAAAGTTGTTAGTAGTTTTTGGATATTGTCCAATCACCTAGCTACCTGGTTTGCCACCTGTCTAAGTGTTTTCTA
CTTCTTTAAAGTAGCCAACTTCTCCCACCCCTGCTTCACTTGGCTGCGGTGGCGAATTCGTAGTGTGGTACTGGTGCTTC
TCTTGGGGTCTTTGTCCTTACTGTTTTTGAATTATGAATCAATATATACACTTAGTCATATCTTAACTAACAGCTACAAA
ATATATGTAAGAAACTCAACGTGGTCCTCAGATGTAAGTGAAACTCATTATCTTCACCAGTTGATTGTTTTTAACTTCAT
CAACTTAATCCCCTTTCTTCTGTCCCTGACCTCACTGCTCCTCTTAGTTCTCTCCTTGATGAGACACATCAGGAATTTGC
AGTTCAACCCCAGCTCAAAGGATCTCAGCACAGAGGCCCATAAAAGAGCCATGAAAATCGTGATGTCTTTCCTCTTCCTC
TTCATCATTCATGTCTCTTCCATCCTATTAATAGCTTGGGTTTTCCTTAAACTGCAGGGACGTCTGGCCCAATTGGTGGT
TGTGTTAACTTCGACTGTTTTTCCTTCAAGCCACTCCTTTATCCTAATTTTGGGAAATAGCAAGCTGAGACAGAATGCCT
TACGACTACTGTGGTATCTTAACTGCCACCCGAAAAGAGTGAAATCTTTAGCTTCATAG
>BoinTAS2R372A_AGFL01051160.1:11353-12279
ATGTCAAATGTCATCAAATATGTTTTTTTGATCATTGAAATCTCAGAATTCATAACAGGAATTTGCGGAAATGGATTCAT
TGCACTAGTACTTTGTGCTGACTCTCTCAAAAGCAAGACTATCTCCTTGCTTGACTTCATCTTCACATGCTTGGCCATCT
CCAGAATTGGTATGGTATTCATACTTCTCCTGGATGGCATTAGAATAGTGTTCCATCCAGAAATATTAGATAGTCACCAG
GTAATAGAAGTAACTTTTGATTTCTTCTGGAATCTGAGCAATTCCTTAGCTACCTGGTGTGCTGCCTGCCTCAGCATCTT
CTACTTCCTCAAGCTATCTAATTTTTCCCACCCCTTCTTTCTCTGGCTAAAATGGAGAAGAAATAGAGTTGTTTTCACCA
TTATGTTGGGATTCTGTCTCTCTTTGTTTTTTAATCTTCTGAACATAAAGTTCAATACTCTCAGGGTCAGTGACCATTTA
GAAATAGAAAACAACTTGACTTGGGAAAAAATGCATGCCTAAAACACAGTACTACAGCAGTCAAATTCTCCTCCACCTGGG
ATCTCTCATCCCCTTGGCTGTGTCACTCATTTTATTTTTCCTGTTAATMTTTTCCTTATGGAGACATACCAGGCAGATGA
CACATCATGCCAAAGGATCCAAAGACCTCAACACAGGAGTTCTTGTGAGAACAAGAAATACTTTGACTTCTTTCATCATT
CTCTTAGTTGTGCACTACTTGGCTACATTCATGTTAACTTGGTTCTATTTCACACTAGAAAATGACGTGACTTTTATTGC
TGCTCAAACTGTAGCATTTCTCTATCCTGCAATTCACCCTTTTATTCTGATTCTGCGGAGCAGGAAACTGAGACAGATTT
CTGTGAATCTGCTAAGGCAAATTGAATCCTGTGTCAAGCGATTGTAA
>BoinTAS2R5P_AGFL01041398.1:20181-21058
ATGCCCTCTTCTATCCTAGGACTGCTGATGCTGGTGGCAGTAGCTGAATCTCTCATTGGCCTCAYTGGAAATGGAGTTCT
TGTGGTCTGGAGTTTCGGAGAAYGTCTCCGAACGTTCAGGGCGTCCTCGTATAACCTCATTGTCCTGGGCCTGGCGGTCT
GTCGGTTGCTTCTACAATGGTTGATTATGGTGGACTCAAGTCTGTTCSTGCTTTTCCAGAGCAGCCATTGGCTTYGCTGG
CTCAGTGTCTTCAGGGTTCTGGYAAGCCAGGTCAGCCTGTGGTTTGCGAGTTTTCTCAGTGTCTTCTATTGTAGGAAGAT
CATGACCGTTGAACACCCTGTCTCTTTGTGGCTGAAGCAGAAGGYGTTACCTGAGTTGCTGGTGCTTTCTGGTGTACTTC
ACRATCCATTTGTTACTTACAGTCAGGGGTAGCTTAGACTTCTCCAGTCCTTCCCAAGGAAACAGCAGCATCTTATTCCC
CATTTCAAACTGGCACTATATATGTATATTACAGCTCAATACAGAAAGTATGATGCCTTTCACRATGTTTCCTGTTTCCT
CTGGGCTGCTGTGTCTCTTTGTATAGACACTACAGGAAGATGAAGGTCCATACAGCCGGCAGAAGAGATGCTCAGGCCAA

GGCTCATATCACTGTCCTGAAGTCCTTGGGCTGTTTCCTTGTACTTTACATGGTCTACATCCTGGCCAGCCCCTTCTCCA
TCAGCTCCAAGACTTTTCCTGCAGATCTCTTCACTGTCTTCATCTCTGAGACACTCATGGCMACCTACCCTTTTCTTCAT
TCTGTCATACTGATCATGGGGAACCCCAGGATGAAGCAGGCATGTCAGAGAATCCTGTGGAAGACTGTATGTGCTTGA
>BoinTAS2R8P_AGFL01051164.1:4515-5444
ATGTTCAGTATAGAAGACCACATCTTTCTGACCATAATGACTGCATAATTCATCATAGGAATGTTTGTGAATGGATGCAT
TGGACTAGTAATATGTGTTGATTGGATTAAGAAGAAAAAGATCTCCATAGCTGACTACATCCTCACCAGTTTAGCTCTCT
CCAGAATGTATTTGCTTTGTGTAATGACACTCAACGGCACCATACTGGCACTCTACCCAGGTGTTTATGAAAATGAGAAA
ATAAAGGTAGTTCTTAATATCTTCTGGACATTCACCAACTACTTAAGTATGTGGTTTGCCACCTGCCTCAATGTCTTCTG
TCTCTTCGAGATAGCCAATTTCTCCCACCGACTTTTTCTCTGGCTGAAGTGGAGAATTGACAGGGTGGTTCACTGGAGCC
TACTGGGGTCCCTGGCCATTTCCATGTTGATCAGCCTTATACAAGCAACGTTAACAAATTCTGATTATGATTTTCTTAAA
ATTGCAAAACATAAAAGAAACGTCACCGAATTGTTCCATGTGAGTAAAATTCAATACTTCGACCCATTGACATTGTTTAA
CCTGTTTGCTATTATTCCATTTACTGTGTCATTGATCTCATTTTTCTTTTTAATTACATCCCTGTGGAGATACAGTAAAC
AAATGAAATCCAGTGTTACAGGCTCCAGAGACTCCAGCACAGAGGCCCACGTGGAGGCCAGGAAAACAGTGACCTCATTT
CTTTTCTTCCTTTTTGTATACTACCTGGCCTGTCTTTTGGCAACATTTAGCGACTTTATGAAAGAAAGCAAGTTAGCTAT
GATGTCTGGAGAGATTATAGAAATTCTTAATCCCTTAGGTCACTCACTGTTTTTTATTGTTGGAAATAACAAGCTGAGGC
TGGCATCTGTCAGGACGCTGAGATGTGGGAAAACAGCCTGCATGATGTAA
>BoinTAS2R9P_AGFL01051164.1:2631-3568
ATACCAGGTACAATGGAGGCAATATATATGTTCTTGATTACTGGCACGTGGATGATAGGAATTTGGGGAAATCGATTCAT
TGTACTGGTAAACTACAGTGGCTGGCTCAAAAAGAGAGCTGTCTCCTTGACTGATGTCATCCTGGTCAGCCTGGCCACCT
CCAGAATCTGTTTTTTTTTTTGTGTTATATATATGGATGGTTTTATTATGGTACTCTTTCCAGATACATACAGGCATGGT
GAGATGATGAACATTTTGGATATTTTCTGGACAACTTGCAATCATTCAACTGTCTGGTTTACTTTTTGCCTCAGCATCTT
CTATTTACTCAAGATAGCCAGTATATCCCACCCAGTTTTCCTCTGGCTGAAGCTGAAGATGAACAGGGTATCCTTGGGAT
TCTTCCGATGTCCTTTCTCATCTCCTCAATTTTTAGTGCTTTACTGAATAATGATTCATTTTATGACTTCAGAATCAATA
ATGAAGCAAACATTACGTAGGAATTCAAAGTAAGTAAAATCCCAACTGCTTTCAAATAGATTATCCTGAGCCTGGAGGCT
ATGGTTCCCTTTATTCTTTGCCTGGTCTCATTTGTCCTTTTATTTTTCTCCTTACTTCGACACACCAAGCAGATGAAACT
TCATGCCACAGGGTCTAGAGACCCTAGCATAGAGGCCCACATGAGGGCCATAAAGACAATAGTCATCTTTCTGGCTGTTT
TCATTATGTACTATGTAGTTTTTCTCTTTGTAATATCTCGCTTTCTGAATCCTCATGGAAAATTGGAGTTGATGTTTGGT
GGCCTAACAGCTGTCATTTTCCCATTGAGCCATTTGTTCATCCTGCCAATGGGAAACAGCAAGCTGAGGGAGGCTTTTCT
GAAGGTGCTGGGGATTGTGAAGGGTTTCCACAAAAGAAGGAAATATTCTGTTCCCCAG
>BoinTAS2R10DP_AGFL01051160.1:6056-6969
ATGGACTGGTTGGATCTCCTTGCAGTTAGTGAGTCAATATTGGGACTTTAAGGGAATGGATTTATTGGATTTATAAGCTG
CATTGATGGTATGAAAAACAAGAAGATCTCTACTATCAGCTTTATTCTCGCTGGCTTAGCAATTGCCAGAGTTTGCCTGA
TATGGACAATAGTTACTGATGGATTTTTAAAGTTATGCTCTCCAGATGTACATTCCTCTGGGGACCTAATTGAATATAAT
GGTTACTTGTGGATAGTGATGAATCAATCAAGTATCTGGTTTGCTACCTGCCTCAGCATCTTCTATTTCCTGAAGATATC
TAGTTTTTCCCACTGCATCTTTCTCTGGTTGAAGGGTAGACTCAACATGGTTGTTTTCCTTCTTTTGGGATGCTTGCTTA
TTTCATGGTTAGTTACTTTTCCACATTTTGTGAAGATTGTTAATGATGATAAAAGGAAAATAAAAACACAGTCTGGTCAA
TGGATATGCATAAAGGTGAACTCTTTGGAAAACAAATTTGGCTGCATCTTGGTGTCATTCTCCTTTTTATACAATACCTG
ATTATATGTGTCTTGTTGCTCACTTCTCTTTGGAGACACAACAGGAGGATGCAATCGAATGCCACAGGATTCATGGTCCC
CAGTACAGAAGCACATATCAAAGCGATGAAAGTCTTGGTGTCTTTTATCATCCTCTTTATCTTGAATTTTGTAGGTACTG
CCATACAAATATCAAGTGTGACAGTGCCTGAAAACAAACTGCTTTTTATTTTTGGTATGACAACCACAGTCCTCTATCTC
TGRGGTCACTCGCTTATCCTAATTCTAGGAAATAGGAAGCTCAAGCAAGCCTCTTTGAGAGTACTGAAGTCATTAAAGTG
CTGGGAAAAAGAGAAACTTCTCAGAACTCCTTGA
>BoinTAS2R11P_AGFL01051154.1:23706-24600
ATGTTGAATATATTGGAGAAGATTTTCATGGTTGTGACTGGTGGGGAATTTATAATAGGAATTTTAGGGAATGGATTTAT

TGGACTCACAACTTGCATTGCCTGGATTAGAAATCAGAAGTTGAGCTTGCTTGACTTCATTCTTACTAGTTTGGCCTTTG
CCAGAATCAGTCAATTATGGATAACCACTGTCATGTTCTTTTCAATGATGTTCTATCAGGCAGGCTTTGGTACTGTGGGA
AGAAAATATATCTTTTTTTGTATCTGGATACTGACCAGTCACTCAAGCACTTGGCTTGCTACTTGCCTTGCTGTCTTTTA
TTTCCTGAAGATTGCCAGTTTCTCCCATCCTCCTTTCCTTTGGCTAAAATGGAGAATTAACAAGGTTGTTTTCATGCTTC
CACTGGTATCTGTGCCCTTCCTAGTCATAAGTTTTCCTTGGCCAAATAATGTTGATGTCTTCTGGTGTCATGTCCAAAAG
ATGCATGAGAGAAGTATGACTGAGTTATGCAATGTGAATGAATATCAAAATTTAAATTTTATTATTATCTTCACAATGGA
GTCCCTCCCACCTTCTTTCTTTCCCTGATTTCCTTTCTCCTGTTGCTCCATTCTTTGTGGAAACACAAGAAGAACATTGC
ACACACTGTCAGGAATTCCAGAGACCCCCGTGTTGAGGCCCATTTCAGAGCCATGAAAACTGTGTTTTTCTTTCTCATGC
TCTTTGTCCTGTACCAATTTGGCCTTTTCATGACATTTGGGGGGCATTTTTTCCTACAGAACAAGCTGGCTGTGATGTTT
GGTTATATGTTAGGAATGCTGTATCCTTCAAGTCACTCATATGTTTTAATTTTTGGAAACAGTCAAATGAGGAAATTCTT
CTTGGTGATTCTTAG
>BoinTAS2R408EP_AGFL01051152.1:21471-22358
ATGACAACTTTACTACATTTTTTCCATTCTGGTAATAGAATTTATTCTAAGAAATTTTGCCAGTGGTTTCATGTCACTGG
TGAACTGCATTGACTGGTCAAGAGACAAAAAATCTCTTCAGCAGATGGGATTCTCACTGCTCTGGCAGTCTCCAGAATTG
GTTTACTCTGAGTAACATTAATAAATTGGTGTGAAAATGTGTTTAATCCCAGCTTTAGACAATTTAAGAGCAAGAATTAT
TATTATTGCCTGGATAATAAGCAACTGTTTTGGTACCTGGCTTGCTGCTATCCTCAGCATATTTTATTGCTCAAGATAG
CCAATTTTCCAATATTATTTTTCTTTACCTAAAATGGAGAATTAAAAATGTTCTTCTTGTTCACTTTGTCCTGTTTGGTT
TTATTAATTCATGGTGTAAATGTAAACAAGACTATCCAGACAAATGACTATGAAGGAAACATCACTCAGAAGACCAAGCT
GAGGGACATTTTACACCTTTGAAATATGACTCTGTTCATGCTAGTAAACTTCACACCCTTTGCTATGTCCCTGACTTCTT
TTCTGCTGTTTATCTTTTCCCCATGGAAACATTTCAGGAAGATGCAGCTCAGTGGTAAAGGACCCCAAGATCCCAGCACC
AAGGTCCATATAAAAGCCATGCAAACTGTCATCTGTTTTCTATTTGCCATTTGCTTCTGGGTTCTAATTTTCTGTTTGAA
GTTCTAATAGGCAGTGGAACAACTTGGTTATCATGGCATGCCAGGCTTTTGGAATCATATGTCCTTCACTTATCCTGACA
TGGGGAAAGCAGACACTAGGACAGTCCTTCCTGTCATTTCTGCAGCAGCTGAGTTGATGGCTAAAAGAAAGGAAATAAGT
GGGCATGA
>BoinTAS2R408DP_AGFL01051149.1:832-1747
ATGATAACTTTACTATGGACCATTTTTTCCATCCTAGTATTAACAGAATTTGTTCTAGGAAATTTTGCCCATGGCCTCAC
AGCACTGGTGAACTGCATTGATTGGGTCAAGAGACAAAAGATCTCCTCAGCTGATGGGATTCTCACTGCTCTGGCAGTCT
GCAGAATTGTTTTGCTCTGGGTAACGTTAATGAATTGGTACTTAGTTGTGTTGAATCCAGTTCTATATAGTTTAAAAGTA
AGAATTATTGTTCATATTGCCTGGATAGTAAGCAACCATTATAACACCTGGCTTGCTACTAGTCTCAGCATATTTTATTT
GTTGAAGATAGCCATTTCTCCAGCCTAATTTTTCTTCACCTGAAATAGAGTTAAAAGTGTCATGCACATAATACTTCTGG
GAACTTCATTCTCCTTGGTTTTTCATGTTGCAGTGATATACAACGATAAGGCTATCCAGACAAATGAATACAAAGGAAAC
ATTCCTCAGAAGACCATATTGAGGGGCAGTTTATGGCTTCCACATGTGACTCTGCTTATGCCAGGAAATCTCATATGCTT
TACTATGTCCCTGACATGTTTTCTGCTATTAACTGTTTCCCTGTGGAAACATCTCAAGAAGATGCAGCTCAGTGGTAAAG
GATCTCCAGATTCTAGCACCAAAGTCCATATAAAAGCCATGCAAACTGTGATATCCTTTCTCTTGCTGTTTGCCATTCAT
TTCCTGGCTCTAAATGGGATCCATTTGGAGTTTTAAAAGGCAGCAGAAGGAAACTGTCTTTTTGTTCTTTGAGGCTCTTG
GATTCCTCTATCCTTCAAACCACTCATGTATCCTGATTTGGGGAAACAGGAAGTTAACAAAGGCATTTCTGTCATTTCTG
TGGCAGCTAAGGTGCTGACTGAGAGAAAAGAAATAG
>BoinTAS2R40P_AGFL01041566.1:11721-12676
ATGGTGACGGTGAACACAGATGCGATGGATAAAGACACGACCAGGTTTAAGATCGTCTTCACCTTGGTGGTCTCTGCAAT
AGAGTGCCTCATTGGCATTGCTGGGAATGGCCTCATCACCGTCATCCATGGAGCCGAGTGGGTCAGAGGCAAAAGACTCC
CCATTGGACTGCATTCTGCTCATGCTGAGCTTTTCCAGGCTCTTGCTACAGATTTGGATGATGCTGGAAAACACGTACAG
TCTGCTGTTCTGGGTCATCTACAATGAAAAAAGAGTATACATACTTTTCAAAACCATCATCATGTTTCTGAACTACTCCA
ACCTCTGGCTTGCTGCCTRGCTCAATATCTTCTATTGTCTTAGAATCGCAAGCTTTACTCACCCGTGGTTCTCCGTGATG
AAAAGGAAGGTCATGTGGCTGATGCCTGGGCTTGTGAGGCTGTCCTTGTTCTTCTCCTTTTGCTCCAGCTTTCCCTTCTC

TAAAGGTATATTCAACGTGTATGTGAACAATTCCGTCCCCATCCCCTCCTCCAACTCCACTGAGAAGGTGTACTTCTCCG
AGACCAACATGGGCAACTTGGTTACCACCCTTTACCTGGGGATCTTCATCCCTCTGATCATGTCTATGCTGGTGGCCACC
CTGCTGATCATCTCTCTCAAAAGACACACCTTCCACATGAAAAGCRATGCCACTGGCTCCAGGGACCCCAGCATGGAGGC
TCACCTGGGGGCCATCARAGCCATCAGCTATTTTCTCATTTTCTACATTCTCAATGCAGTTGCTCTGTTTTTTTCCATA
TCCAACATCTTTGCYGCCAACAGCTCCTGGAATATTTTATGCAAAATCATCATGGCTGCCTACCCTGCTGGCCACTCAGT
GCTACTGATCTTGGGCARCCCTGGGCTGAAAAGGGCATGGAAGCAGTTTCAGCACCAAGTTCATCTCTACCTGTAA
>BoinTAS2R408CP_AGFL01051152.1:4910-5851
ATGATAATGTTTATGTCAAACATTGTTTCCATTCTATTAATGACAGAATTTGTTCTGGGAAATTCTGCCAATGTCCTCAT
AGCACTGGTGAACTGCAATGACTGGACCAAGAGACCAAAGATCTCAGCTGATGGGATTCTCACTGCTCTGGCATTCTGCA
GAATTGTTATGCTCTGGCCAATGTTAATAAATTGGTATGTAATTGTGTAAAATCTAACTCTATATAATTCAAAAGTAAAA
ATTATTGTTCATGTTGCCTTGACAGTAAGCAATCATTTTAGTAACTGGCTTGCTACTAGCCTCAGTATATTTTATTTGTT
GAAGATAGCCAATTTCTCCAGCCTAATTTTTCTTCACCTGAAGTGGAGAGTTAAAAGTGTAGTTCTCATGATGATGTTGG
GGACGTCATTGTTCTTGTTTTTTCAAGTTGCAGTGTTAAGCATGGATGAGGCTATTCAGACAAATGAATATGAAGGAAAT
ACCACTCAGAAGATCAAACTAAGGGACATTTTACACCTTTCAAATGTGACTCTGTTCACACTAACAAACTTTATACCCTT
CACTATGTCCTTGACATCTTTTCTGCTGCTAATCTTTTCCCTGTGGAAACATCTCAGGCAGATGCAGCTCAATGGTAAAG
GATCCCAAGATCCCAGCACCAAGGTCCACATAAAAGCCATGCAAACTGTCATCTCCTTTCTTTTCCTGTTTGTTATTTAC
ATTCTGGCTCTAATTGTATCAGTTTGGAATTCTAATCAGCTGCAGAAAGAACCAGTCCAAATGCTTTATGATGTTGTTTT
AATCATGTATCCTTCAATCCACTCATGTTATCTTGATCTGGGGAAATGGGAAATTAACTCAAGCCTTTCTGTCATTTCTA
TGGCAGTCAAGATGCTGGCTGAAGGAAAGRAAATAGGTGGAAACACATGTCTTTTAGCATAA
>BoinTAS2R62P_AGFL01041586.1:6334-7244
ATGTTGATATTCAAGGTCATCTTTTTCCTGGAGTCATTGGTTGCTGTGCTGCAGAATGGCTTCATAGTTACTGTGTTGAG
CGGGGAGTGGGTGTGAAGCCGGATGCTGCCCGCTGGTGACATGATTGTGACCTGCCTGGCTGCCTCCTGGTTCTTTCTGC
ATGGGATGGCCCTCTTGAACAACATCATGGCCTCTTCTGGCTTTTGTTCCAAAATCGACTATTTCAGCATTCCCTGGGAT
TTCATCAACTGCCTCAGTTTCTGACTGACTGCCTGGTTTGCTGTCTTCTACTGCGCGAAGATCTCCCTCTTCTCTCATCC
CCTCTTCTTCTGGATAAAATGGAGGATTTCTCGGTCGGTTCCCCAGCTGGTGCTGGGCTCCTTGATCTTATCTGGTCTGT
CCGTCATCTCAGCTGCTGGGAATACAATTCTTGCCCAGATGACGGCTGCCCAGATTTCCCATGGAAACGACACCCTGGCT
GGTAGAATACATGCTACCTATTTGCACTTTTTCTACCTCATGTAATTCTCATGTGGTTGGTTCCATTCCTCCTGTTCCTG
GTGTCCACCCTCTCGCTCATGTTCTCACTGCGCCGGCACCTCTGGCAGATGCAGGACCACAGACCCAGCCCACGTGATCC
CAGTACCTGGGCTCACACCATGGCCCTGACGTCACTTGCCTTCTTCCTCATCTTCTACACCTTGTACTTCCTGTCCCTGG
TTATCATTATGTACATCCCAGCCCTCCGGGAACACTGGCACTGGGCCTGTAAGGTGGTGACCTACACGGGCATCTGTCTG
CACTCCAGCATCTTGGTGCACAGCAGCCCCAAGCTGAGAAAGGGCCTGAAGAAGAGGCTTTGGCGAGCCCTGGACAAGGA
CCAGTTTGTCTCCAGTTATCAGTATCAATAG
>BoinTAS2R18BP_AGFL01051151.1:7295-8237
ATGTCAGTTGGAATGAAGGGCTCTTTCTACTAGTGGCAACAGGAGAACTCATCTTAGGAGTGCTGAGAAATGGGTCAAGA
ACAGGAAAGTCTCATCAGCTGGTTTCATCCTTACCTGCTTAGCTGTGGAGAGAATCATTCAAATGTGGGTAACACTATTG
GGTTCATTTACAGCGGGGCTATTTTCACATCTGTATGCTACCAGCAAACTAGCAGAGGTGATTACTCTTTTTTGGGCACT
AACGAATCACTTAACTACCTGGTTTGCTAACCCCTAAGTGTGTTCCATTTCTTTAAGATAGCCAATTTCTCTCATTTCTT
CTTCACATGGCTGGAGTGGAGAAGGAACAGAGTGATTCTTATACTTTTCCTGGGMTCTTTGCTCTAACTGTCTGTTAACC
TCTTAATGCTCTTGGTGAGTTGTGGATGAGTAGCTATAGAGAGCCTGAAAGAAACACAGCTTTGCATTTAGATGCAAATA
AAATTTTCTATCTTAGATGCCTTATTCTTCTTAGCTTGACCTATGTTACCCATTTTCATCTCTCCCTGGCCTCKTTGTGC
TTTTATTTTTCTCCTTGGTGAGACACACCAAGAATTTCCAACTCAACCTGAATGGCTCAGAAGACACCAGCTCAGAGGCC
CATAAAAGGACCGTGAAAAGGGTGACAACCTTCCTCTTCCTGTTCATCATTTACTTTTTCCACTCCATTAGGGAGCTGGA
TCTTTCTTAAGGTACAGCGGTATCAGGCCATGATGTTTGTCATGAAGGTTTCAACTGTCTTCACTTTGGGTCCTCTTATG
TTTTAATTTCGGGGATTAGCAAGCTAAGAAAGATCACCTTGAGTTTAATTTTGAATCTTATATTCTCTTTGAGAAAACCA

AGATCATTAGTTTCATGGACAGAATTTAAATGTACTTTATGTATTCTGGAGAAAATGCCTTAA
>BoinTAS2R372CP_AGFL01051158.1:34362-35327
TCAGCAGTGACCAAAGTAACCAGGATTTCACTTCTGCAGTCATGTCCAGTGTAATCAAAAGATTTTTATGATCATTGAAA
TGTTATAATTCATAACAGGAATAACTGTGCTGACTTTGTCAAAAGCAAGAATAGTGCCTTGTTTGACTTCATCTTCACAT
GGATTAGCGTGATGTTCATACTTCTCCTAGATTGCATTAAACTAGTGTTCCATCTAGAAATATTAGATGGTCACCAGGTA
ATAAGAGGAGTTTTTGAGTTCTCCTGGAGTCTGAGAAACTCATTAAGTACTGGATGTGCTGCCTGCCTCAGTGTCTTCTA
CTTCCTCAAGCTATCTAGTTTTTCTCACCCCTTCTTTCTCTGGCTGAAGTGCAGAAGAGATAGAGTTGTTTTCACCATTA
TGTTGGGATTCTGTCTCTTTTTGATTTTTAACCTTCTGAGCATAAAATTTATACTTTTGTGTTCAGCAAGCATTTAGAAA
AGGAAAGACTTAACTTGGAAAAAAGATATGCATAAAAATCAGTATTATAACAGTCAAGTTCTCTTCAGCCTTGGATCTCT
CATCCCCTTGTCTGTATCACTCATTATATTTTTCCTGTTAATCTTTTCCTATGGGGACATACCAAGCAGATGACATGCCA
TAACACAGATCCCAGGGACTTCAATGCTGGGAGCCACCACGGGAGATCCCACCCATGACAAAGGTCATGCGGAAGAGACC
TGACAGGCAAAGGAGGATCAGGCCTCAAGGGACCCCCTGAATCTTCTTGAGCATCTACCSCAAAACCAAAATCTGTCTAC
TGTTTATTATATTATGCCTTTCACCAACTCTTCTGTCATTAACAGGGGGCTATCCCCAACCGGGAGAAATATCAATAACC
TCAGATATGCAGATGACACCACCCTTATGGCAGAAAGTGAAGAGGAACTCAAAAGCCTCTTGATGAAAATGAAAGAGGAG
AGTGAA
>BoinTAS2R372BP_AGFL01051158.1:13699-14434
ATGTCAAGTGTAATCAAAAAAGTTTTTATAACCATTGAAATCTTAGAATTCATAACAAGAATTTGCAGAAATGAATTCAT
TGCACTAGTACTCTGTGCTGACTCTCTCAAAAGCAAGAGTGTCTCCTTGTTTGACTTGATCTTAACAGGCTTGGCCATCT
CCAGAACTGGCATGATATTCATAATTTTCTTGGATGGCATTAGAATAGTGTTCTATCCAGGAATATTTGAAAGTCATCAG
GTAATAGATGTAATTTTTTATTTCCCATTCTGTTGGGATTCTGTATTTCTTTGATTTTAATTTTCTGAGTATAAGTTTTC
ATACATTTGGGGTCAGTGACCATTTAGAAATAGAAAATAACTTGACTTGGGAAAAATATGCATAAAATCCGATCCTATAG
CAGTCAAATTCTCCTCCACCTAGGATCTCTCATCCCCTTGGCTGTGTCACTCATTTTATTTTTCCTGTTAATCTTTTCCT
TATGGAAACATACCAGGCAGATGACACGTCATGTCAAAGGATCCAGAGACCTCAACACAGGAGTTCTTGTGAGAGCCAGA
AATACATGACTTCCTTCATCATTTTCTTAGTTGTGCACTATTTGGCTACATTCTTGTTAACTTGATCCTGTTTCACACTA
GAAAATGAAATGACTTTTATTGTTATTAAGTCTGTAGCATTTCTCTATCCTTCAATTCACCCTTTTATTTTGATTCTAGG
AAACGGAAAACTGAGA
>MybrTAS2R1_NW_005362626.1:2672135-2671236
ATGTTAGAGTTGTACATGATTGCCCATCTTATTTTTTCAGTGATTCAGTTTCTTGTGGGGGTTCTAGCCAATGGCTTCAT
TGTGGTTGTGAATGGCACAGACTTGATCAGGCGGAGAAAGATGGTGCCCTTCGACCTCCTCCTATGCTGCCTGGCGACTT
TCAGGATTGGTCTCCAGATGGTCATGATCTTCATTAATCTGGCTGTTCTTTCCTTGATTAAATTCTCTCCAGTTCCTGGG
AATATTACAATTTTCATGTATGTACATGCATCGGAACTTTGGTTGGCCACGTGGCTCAGCGTTTTCTACTGTGCCAAGAT
CGCCACCATCGCTAACCCGCTCTTCTTTTGGTTGAAGTTGAGGATCTCCAAGTTGGTGCCGTGGCTGATTTGTGGGACCT
TGACATATACCTTTCTCACTTCTGTCTTCCACAGAAAACATGCATGGATTATTTCCCAAAAATCCTGGTTGGGCTTTTTC
TCCCAAAATGCAACAACTCAAATTGAAGACATATCTGCATTACAATATGCCCTTCTTTTAATTGAGTTCGTATTGCCTTT
ATTTATCTTCCTTATCTCTGCTCTTCTCTTGATATTTTCCCTGGGGAGGCACACCCAGCAGATGAGGAGCACCGAGACGG
GCACCAGGCACCCTGGCATGAGTGTCTACATCAACGCACTCCTATCCATCCTGTCCTTCCTGATTCTCTACGTCGCCCAG
TACATGATGGCTGCATTAGGTTTTTCTGAAATTTTCAAGATCAGAAACTCCATCACTCTGTTCTGCATCTTGCTGTTTGG
TTCATACCCCTCTGTACACTCTGTTATCTTAATTTTAGGAAATCCTAAGCTGAAACAAAATTCGAAGAAGTTCCTCCTCT
ACAGTAAGTGCTGTCAGTGA
>MybrTAS2R2_NW_005371516.1:299188-300099
ATGACCTCCTCTTTGTCAGCTATTCCTCATGCTATCATCATGTCAGCAGAATTTATTACAGGGATTACAGGCAATGGATT
TCTGATAATCATCAGCTGTAACGAATTGATCAAAAGCAGAAAGCTAACACCAATGCAGCTCATTTTAATATGTATAGGGA
TGTCTAGAGTCGGTCTGCTGATGATGTTAATGGTACAAAGTTTTTTCTCTATGTTCTTTCCACTCTTTTATCAGACAAAA
ATTTATGGTGCAGCGATGGTGTTCCTTTGGATGTTTTTAGCTCTGTCAGTCTCTGGTTTGCCACCTGCCTTTCTGTATT

TTACTGCCTCAAGTTAATAGGCTTCACTCATCCCTGTTTTCTTTGGCTGAAATTCAGGATCTCAAAGTTAATGCCTGGGC
TGCTTCTGGGAAGCTTGCTGGCCTCGGTGAGCACTGCAACTCTGTGTATCGAGGTAGATTACCCTAAAAACGCGGTGGAG
AATGTCCTCAGAAATGCCACACGTACGACGTCTAAATTCAAGCTAAGGAGTATTAATGAAGTGCTTCTTGTCAATTTTTC
ACTCCTATTTCCTCTAGCCATATTCCTGATGTGCACTTTTATGTTACTCATGTCTCTCTACAAGCACACTCACCGGATGC
AAAACGGATCTCGTGGTTTTAGAAGTGTCAGCACAGAAGTCCATATAAACGCCTTAAGAACAGTGCTAACGTTCGTTCTT
TTCTTTATTTCTTATTTTGCCGCCTTCATAACAAACATGACATTCATCATTCCTCATGGAACGCAGCGCTACTTTGTGCT
GAAGGACATAATGGCAGCATATCCCTCTGGCCACTCAGTTATAATAATCTGGAGTAATTCTAAATTCCAACAACTACTCA
GGAGACTTTTCTGCCTCAAAAAGAGTCAATGA
>MybrTAS2R3_NW_005367384.1:2834865-2835815
ATGTCAGGACTCGCCAAGTGGGTGGTTCTGTTTCTTTCTGTCACTCAGTTCCTTCTGGGAATGCTGGGGAATGGCTTCAT
TGTGCTGGTCAATGGCAGCAGCTGGGTCAAGAGCAAGAGAATCTCTGTGTGTGATTTCATCATCACTAACCTGGCTCTCT
CCCGGATTGTTCAGCTGTGGATTCTTTTTTCTGATTTTGTAATAATGATATTCTTTTCCAAACTATTAAATAATGTAGTA
TTCATACAAGTTGCTGATATTTTCTGGACATTTACAAACCATCTGAGCATTTGGCTTGCCACCTGTCTCGGTGTCTTCTA
CTGCCTGAAAATCGCCAATTTCTCCCACCCCACATTCCTCTGGCTCAAGTGGAGAGTTGCCAGGGTGGTCATATGGATGC
TGTTGTGTGGGCTGCTCTTATCGTGTGGTAATGCCATGTCTCTGATTCATCAGTTTAATATGTATTATGTTCTCCGTGGA
GCTGATGACTCAGGGAATGTGACTGAGCACTTTAGAGAGCTAAAGAATGAATATGAGGTGATCCATGTTCTTGGGACACT
GTGGAACCTCCTTCCCCTAATTGTGTGTTTGGCCTCCTACATCCTGCTCATCCTCTCCCTGGGGAGGCACACGCGGCAGA
TGCAGCAGAACAGAACCAGCCCCAGCGATCCAAGCACCGAGGCCCACAAGAGGGCCATCAAAATGGTCCTCTCCTTCCTC
TTTCTCCTCCTGCTTTACTTTCTTGCCTATGTACTCACATCATCCCATTATTTCCTATCAGGAACTGTGTTGACTAAGAT
GATTTCAGAAGTAAGTGCAATGTTTTATCCTGCCTGCCACTCGTTTGTTCTCATTCTGGGAAACAGTAAGCTGAAGCAGA
CGTTTGTGGAGCTGCTCTGGTGTAAGTCTGGTCATCTGAAGCCTGGATCCAAAGAACGCTTTTCCCCATAA
>MybrTAS2R4_NW_005367384.1:2844211-2845110
ATGTTCCAAATATTCTCTGTCTCTGTTCTTACTATCTCAGTAGTTTTGGATTTTATAGGACTCATTGTGAATCTGTTTAT
TGCAGTGATCAATTACAAGACTTGGGTCCAAAGCCACGGAATGTCCTCTTCGGATAGGATCCTGTTCAGCTTAGGCATCA
CCAGATTTCTCATGATGGGAATGTCCCTGGTGAACATCTGCTTCTTCATCTCTCCAAATGTTGAAAGGTTAGTTTACTTA
CCCAAATTTTTCCTGTTGTTTTGGATGTTTTTGGACTCCAGTAGCCTCTGGTTTGTAACTTTGCTCAATGTCTTGTACTG
TGTGACGATTGCTAACTTGAAATACTCAATTTTTCTTCTGCTGAAACAAAATCTCTCCCCAAAGACCCCCAGGCTATTGC
TGGCCTGTGTGTTGCTTTCTGCCTTCACCGTGCTTCTGTATATTGTGCTCAGACAGAAGCTACTCTTTCCTGAATTTGTG
ATGACGGAGAGAAATGGCACAGAATTTAACGCCGATGAGGGCACCTTGTCAGTGGTGATCTCTTTGTTCTTGAACTCATT
TCTCCAGTTCATCATTAATGTGACTTCTGCTTCCTTGTTAATAAACTCCTTGAGGAAACATATACAGAAGATGCAGAGAA
ATGCCACTGGCTTTTGGAATCCCCAGACTGAAGCTCATGTGGGTGCTATAAAGCTGATGGCTTATTTCCTCCTCCTCTAC
ATTCCGTATACAGTTGCCACCCTGTTCCAGTACCTCCTTTCTAAAGAGATGGATTTGGGAACCAGATCCATATGTATAAT
AATTTCCACCTTTTACATTCCAGGACATTCTGTTCTCATTGTTCTCACACATCCTAAACTTAAAAGTAAAGCAAAGAAGA
TTATTTGTTTCAACAAATAG
>MybrTAS2R5_NW_005367384.1:2855189-2856070
ATGCATATTGCCACCCTAGGACTGCTGATGGTGGTGGCAGTGACCGAATTTCTCATTGGCCTGGTTGGAAATGGAGTCCT
TCTGGTCTGGAGTTTTGTAGAATGGGTAAGAAAACTCAAGGAGTCCTCCTACAACCTCATTGTCCTGGGCCTGGCTGGCT
GCCGACTTCTCCTGCAGTGCCTGATTATGGTGGACCTAATACTGTTTTCGATTTTCAAGAGCTGCATCTGGTTTCGCTAT
ATCAGTGTCTTCTGGGTTGTGGTCAGCCAGGCCAGCCTGTGGTTTGCCACTTTCCTCAGTGTCTTCTACTGCAAGAAAAT
CACGACCTTTGAACACCCTGTCTACCTATGGCTGAAGCAGAGGGCCTATAGCCTGAGTGCCTGGTGTCTTCTGGGGTGCC
TCCTGATCAATCTGCTAATTATAGCCGATGTTGGCTTAAAGCCCCACAGTCCTTTCCAAGGAAACAGCAGCATTCTGTAC
TCCTTTTCAGACTGGCAGTATCTGTATATATTACAGCTCAATGCAGGATGTGGGTTTCCTTTCTCGGTGTTTCTAATTTC
CTCTGGGATGTTAATTGTCTCTCTGTATAGACACCATAAGAAGATGAAGGTCCATACAGCTGGCCGGAATGATGCTCGAG
CCAAGGCTCACATCACTGTCCTGAAGTCCTTGGTCTGCTTCCTTATACTTTACTTGGTTTACATCGTGGCCAGCCCCTAC

TCTATCAAATCTAAGACTTCTCCTGTTGATCTCACCACTGTCTTCATCTCGGAGACAGTCATGGCTGCCTATCCTTCTCT
TCATTCTGTCATATTGATCATGGGGAATCCCAGGATAAAGCAGGCTTGTCAGAGAATCCTGTGGAAGACAGTGCGCGTTT
AG
>MybrTAS2R7_NW_005358943.1:6938370-6939308
ATGTCAGATGAAGTAATCAACACCTTAATGATCATAACAGTTGGGGAGTTTTCAGTGGGGATCTTAGGAAATGCATTTAT
TGTATTGGTAAACTTCATGGACTGGATGAAGAATAAGAAGATTGCTTCCATTGATTTAATCCTCACAAGTCTGGCCATAT
CCAGAATTTGTCTAATGTGTATAATAACGTTAGATGGTTTTATGTTGGTGTTGGATCCAGATGTCTATGCCACTGGTAAG
CAAATGAGAATCATTGACTTCTTCTGGACACTAACCAACCATTTAAGTATCTGGTTTGCCACCTGCCTCAGCATTTTCTA
TTTCTTCAAGATAGCTAACTTCTTCCATCCTCTTTTCCTCTGGATGAAATGGAGAATTGAAAGGGCGATTCCTGGGATCC
TGCTGGTGTGCGTGGCCTTCGCTGTGTTCATTAGCCTTCCTGCCGCTGAGGATTTGAATGATGACTTCAGGCTTTGTGTC
AGGGCGAAGTGGAAAACAAACTTAACTTTGAGATGCAGGATAAATAAAGCTCAATATGCTTCCAGCAAGGTATATCTCAA
CCTGTTCACGCTGTTCCCCTTTTCTGTGTCCCTCATCTCATTCCTCCTCTTGATCCTCTCCCTATGGAGACACATCAGGC
GGATGCAGCTCAATGCTACAGGGTGCAGAGACCCCAGCACAGATGCCCACATGGGAGCCATGAAAGCTGTTATCTCCTTC
CTCCTCCTTTTCATTGCCTACTATTTGTCCTTTCTCATAGCCACCTCCAGTTACTTCATGCCAGAGACTGAATTAGCTGT
GCTGATTGGTGAACTGATAGCTCTAATCTATCCTTCCAGCCATTCATTTATATTAATTCTGGGGAACAAGAAATTAAGAC
AAGCATCTCTAAGGGTGCTATATAAAGTAACACATACACTAAAAAGAAGAAATTTCTAA
>MybrTAS2R10_NW_005358943.1:6927369-6928267
ATGCTAAGTATAGCGGAAGGCCTCCTCATTTTTATAGCAGTTGGTGAATCAATACTGGGGGTTTTAGGGAATGGATTTAT
TGGACTTGTAAACTGCATTGACTGTGTCAAGAACAAGAAGTTTTCTGTAATTGGCTTGATTCTCATTGGCTTAGCTACTT
CGAGAATTTTTCTGATATGGATAATAATTACAGATGGATTTATAAAGATACTCTCTCCATATATGTACTCCTCTGGAAAC
CTAAAAGAATATATTAGTTATTCATGGATAATTATAAATCACTTAAGTATCTGGTTTGTCTCCAGTCTCAGCATCTTCTA
TTTCTTGAAGATAGCCAATTTTTCCCACTACATTTTTCTCTGGTTGAAGCATAGAATCAACAGAGTACTTCCTCTTCTGA
TGGGCTTGATGCTTATTTCATGGTTATTTATTTTCCCACAAATTGTTAAGATTATGAATGAATATAAAATAAATAATGGA
AACACAACCCGGCATCACAACGTATCTAAAAGTGAATACATTGCTTACCAAATTTTGCTCAATCTGGGAGTCATTTTCCT
CTTTATACTGTGTTTGATTTCATGCCTCTTGTTAATCATTTCTCTTTGGAGACACAACAGGAACATGCAATCGAGTGCCC
GAGGTGTCGGAGACCCCAGCACAGAAGCACATGTGAGAGCAATGAAAGTGTTGATATCTTTTATCATCCTCTTGATCTTG
CATTTTATAGGCATTGCCATAGAAATAGCATGCTTTTCTGTGCCAGAAAACAAATCGCTGTTTCTTTTTGGTATGGTGAC
CGCAATCATCTATCCCTGCGGTCACTCATTTCTCCTAATTCTAGGAAACAGCAAGCTAAAGCAGGCTTTCCTGAAGGTAC
TACGGCCTTTCAATAGCTAA
>MybrTAS2R11_NW_005358943.1:6913857-6914789
ATGTTGGAGAAAGTTTTCATGATTATAACCAGCGGGGCATTTTTAATAGGAATTTTAGGGAATGGATTCATTGGACTCAC
AAATTGCATTGCCTGGGCTAGAAATCAGAAGTTATGCTTGGTTGACTTCATTCTCACCAGTTTGGCCTTCACCAGAATCA
GTCTTTTGTGGCTAATAATTGTCAATTTGTTTTCAGTGCTGTCCTATCAGGAAATCCCTGCTACTATGGAAGGAAACCTT
ATTTGTTCTAGTTTCTGGATACTGGCCTCTCACCTGAGTACTTGGTTAACTGCTTGCCTTGCCGTCTTTTATTTCCTGAA
GATCGCCAATTTCTCCTCGCATTTTTTTGTTTGGCTAAAATGGAGAATTAACAAGGTAGTTTTCATGCTTCTGCTGGTAT
CTTTGCCCTTCCTGTTCCTGAGCCTTCCCTTGCCGTATCATTTTGGTATCATCTGGTATCATTTTTCCCCAAAACATGAG
GGAAATATGACTGAGTTATTCAATGTGAGTACAAGTAAAAATTTAGATCAGATTATTATGTTCATGATTGGGTCCCTCCC
TCCTTTCTCTGTTTCCTTCATTTCCTTTTTCCTGTTGCTGCTTTCTTTGTGGAGACACACAAAACACGTTGAGCTCAACA
TCAGGAACTCCAGAGATGCCAGTATGGAGGCCCACACCAGAGCAATGAGAACTGTGTTTTTCTTTCTTATGCTCTCTGCA
CTGCAGCAATTTGCCTATTTCATGACATTTGGGGGATATTTTTTGCAAGAGAACAAGCTGGTTGTGATGCTTGGTTATAT
GATAGGAATTTTATATTCTTCAGGGCACTCATATGTTGTGATTTTTGGAAACAGCCAAATGAGGAAAGCCTTCTTGCGGA
TTCCTGGGCACCTGAAGCGAGGTCTGAAAAGAAAGGTACTCTTGGCTACATAG
>MybrTAS2R16A_NW_005370153.1:312891-313814
ATGATACCCAACCAACTCACTCTTTTCTTCATGACCATCTATCTGCTCGAGTCCTTGACAATAATTGTGCAGAGCAGCTT

AATTGTTGTGGTGCTGAGCAGAGAGTGGGTGCAGGTCAAAAGGCTGTCACCTGTGGACATGATTCTCATCAGCCTGGGCG
TCTGCCGCTTCTGTCTACAGTGGTCTTCAGTGCTGCACAACTTTTGCTCCTATTTCAACTCTGACGATGACCTTTGGTAC
ATAGCAATCATCTGGGAATTTACTAATACTCTTGCATTCTGGTTGACCAGCTTGCTTGCTGTCGTCTACTGTGCCAAAGT
CTCTTCCTTCACCTACGCCATCTTCCTCTGGCTGAGGTGGAGAATTTTGAGGCTGATTCCCCAGCTGTTGCTGTGCTCTC
TGATGATTTCCTGTGTGACAATCATTGTTTCAGCTATTAAACATTACATCAAGAGTCAGTTAATCTTGCAATTACCTGGA
AACATCACGAAGACTGAGACACTTAGGACCTTCCTGGAAAAATATTACATAGGTCAGCATCTGGCAATGTTGTTCATTCC
TTTCCTCCTGTTCCTGGCCTCCACCATCTTGCTCATAGCCTCATTGTGCCAACACTTGAGGCAGATACGACATCACGACA
CTGGCCACAGCAACTCCAGCATGAAAGCTCATGCTACTGCCCTGAGGTTTCTTGCCTTCTTCCTCATCTTCTTCACCTCT
TACTTTTTGACCATAATCATCTCCACTAAATACATCTTAAGGCATAAGACTTCCTGGTTCTGGGCCGGCGAAACTATCAT
CTATGCTACAGTCTCTATTCATTTAACTTCACTAATGCTGAATAGCCCTACGTTGAAAAAGGTTTTAAAGGTAAACTGCT
GTGGCCCAAAGCTGCCTGAGTCTCCAGGTACAGCAAGACCCTAA
>MybrTAS2R16B_NW_005370153.1:265995-266927
ATGATACCCAACCAACTCACTGTTTTCTTCATGACCATCTATCTGCTCGAGTCCTTGACAATAATTGTGCAGAGCATCTT
AATTGTTGCGGTGCTGAGCAGAGAGTGGGTGCAGGTCAAAAGGCTGTCACCTGTGGACATGATTCTCATCAGCCTAGGCA
TCTGTCACTTCTGTCTACAGTGGTCATCAGTGCTGTACAACTTATTCTACTATTTCAACCCTGAAGATGACCTTTGGTAC
ATAGCAACCATCTGGGAATTTACTAATACTCTTACATTCTGGTTAACCAGCGTGCTTGCTGTCGTCTACTGTGTCAAAGT
CTCTTCCTTCACCTATGCCATCTTCCTCTGGCTGAAGTGGAGAATTTTGAGGTTGGTACCCTGGCTGTTGCTGGGCTCTC
TGATGATATCCTGTGTGACAATCATTGTTTCAGCTCTTAGAGGTGTCTTCATCAAGAGACATTTAATCGCCATGATGCAA
TTACCTGGAAACAATACGGAGACTGAGTCACTTAGGACATTCATAGAGAAAAATTACGTATATCAGCGTCTGGCAATGTT
GTTCATTCCTTTCCTCCTGTTCCTGGCCTCCACCATCTTGCTCATAGCCTCATTGTGCCAACACTTGAGGCAGATACGAC
ATCATGACACTGGCCACAGCAACTTCAGCATGAAAGCTCATGCCACTGCCCTGAGATTTCTTGCCTTCTTCCTCATATTT
TTCACCTCTTACTTTTTGACCATAATCATCTCCACTACATATTACCTAAGGTATAAGAGTTACTGGTTCTGGGCCGGCGA
AACTATTATCTATGCTACAGTCTCTATTCATTTAACTTCACTAATGCTGAGTAGCCCTACGTTTAAAAAGGTATTAAAGG
TAAGCTGCTGTGGCCCAAAGCTGCCTGAGGCTCCAAGTACAACAAGACCCTAA
>MybrTAS2R16C_NW_005370153.1:249986-250918
ATGATACCCAACCAACTCACTGTTTTCTTCATGACCATCTATCTGCTCGAGTCCTTGACAATAATTGTGCAGAGCAGCTT
AATTGTTGCGGTGCTGAGCAGAGAGTGGGTGCAGGTCAAAAGGCTGTCACCTGTGGACATGATTCTCATCAGCCTGGGCG
TCTGCCGCTTCTGTCTACAGTGGTCTTCAGTGCTGCACAACTTTTGCTCCTATTTCAACCCTGACAATGAACTTTGGTAC
ATAGGAATCATCTGGGAATTCACTAATACTCTTGCATTCTGGTTAACCAGCTTGCTTGCTGTCGTCTACTGTGTCAAAGT
CTCTTCCTTCACCTACGCCATCTTCCTCTGGCTGAGGTGGAGAATTTTGAGGTTGGTTCCCTGGCTGTTGCTGGGCTCTC
TGATGATATCCTGTGTGACAATCATTGATTCAGCTCTTAGAGTTGTCTTCATCAAGAGTCAGTTAATCTCCAGGATGCAA
TTACCTGGAAACAACACGGAGACTGAGACACTTATTTCATTCCTGGAGAAAAATTATGAACATCCACTTCTGGCAATGTC
GTTCATTCCTTTCCTCCTGTTCCTGACCTCCACCATCTTGCTCATAGCCTCATTGTGCCAACACTTGAGGCAGATACGAC
ATCATGACACTGGCCACAGCAACTCCAGCATGAAAGCTCATGCCACTGCCCTAAGGTTTCTTGCCTTCTTCCTCATATTT
TTCACCTCTTACTTTTTGACCATAATCATCTTTAGTACATACAACCTAACGCATAAGAGTTACTCGTTCTGGGCCAGCGA
AACTATCATCTATGCTACAGTTTCTATTCATTTAACTTCACTAATGCTGAGTAGTCCTGCATTGAAAAGGGTTTTAAAGG
TAAGCTGCTGTGGACCAAAGCTGCCTGAGGCTCCAGGTACAACAAGAACCTGA
>MybrTAS2R16D_NW_005370153.1:228297-229229
ATGATACCCAACCAACTCACTGTTTTCTTCATGACCATCTATCTGCTCGAGTCCTTGACAATAATTGTGCAGAGCAGCAT
AATTGTTGCGGTGCTGAGCAGAGAGTGGGCGCAGGTCAAAAGGCTGTCACCTGTGGACATGATTCTCATCAGCCTGGGCG
TCTGCCGCTTCTGTCTACAGGGGACATCAGTGCTGTACAACTTTTGCTACTATTTCAACCCTGATGATGACCTTTGGTAC
ATAGCAACCATCTGGGAATTTACTAATACTCTTACATTCTGGTTAACCAGCGTGCTTGCTGTCGTCTACTGTGTCAAAGT
CTCTTCCTTCACCTACGCCATCTTCCTCTGGCTGAGGTGGAGAATTTTGAGGTTGGTTCCCTGGCTGTTGCTGGGCTCTC
TGATGATTTCCTGTGTGACAATCATTGTTTCAGCTCTTAGAGTTGTCTTCTTCAAGAGTCATTTAATCTCCATGATGCAA

TTACCTGGAAACAACACAAAGACTGAGACACTTAGGACATTCCTGGAGAAAAATTACGTACATCAGTTTCTGGCAACTTC
GTTCATTCCTTTCCTCCTGTTCCTGACCTCCACCATCTTGCTCATAGCCTCATTGTGCCAACACTTGAGGCAGATACGAC
ATCATGACACTGGCCACAGCAACTCCAGCATGAAAGCTCATGCCACTGCCCTGAGATTTCTTGCCTTCTTCCTCATATTT
TTCACCTCTTACTTTTTGACCATAATCATCTCCACTACATATTACCTAAGGTATAAGAGTTACTGGTTCTGGGCCGGCGA
AACTATTATCTATGCTACAGTCTCTATTCATTTAACTTCACTAATGCTGAGTAGCCCTACGTTTAAAAAGGTATTAAAGG
TAAGCTGCTGTGGCCCAAAGCTGCCTGAGGCTCCAGGTACAGCAAGACCCTAA
>MybrTAS2R408A_NW_005358943.1:6809154-6810071
ATGATACACTTATTACCAAGCATTCTTTCCATCCTTATAATAGCACAATATGTTCTAGGAAGTTTTGCCAATGGCTTCAT
AGCACTGGTGAACTGCATTGACTGGGTCAAGAAACACAAGATCTCCTGTGCTGATCGAATTCTCACTGCTCTGGCTTTCT
CTAGAATTTGTTTGCTCTGGATAATAATATTCAATTGGTATGGAACTGTGTTGCATCCAGCGATCTATAGTTCAGAAGTA
AAAACTATTGTTCATATTGCATGGGTAGCAAGCAACCATTTTAGTCTCTGGCTTGCTACTAGCCTCAGCATACTTTATTT
GCTCAAGATAGCCAATTTCTCCTGCCTTTTATTTCTTCACCTAAAATGGAGAGCTGAAAGAGTGGTTATCATGATACTGT
GGGGGACTTCGGTCTTCTTGGTTTTTCATCTTGCAGTGGTAGGCACAGATGAAAAAATGAAGATGAACGTATACAAAGGA
AACATCACCTGGGAGAGCAAATTGAGGGACATCACACACCTTTCAAATGGGATTATATTCGTGCTTGTACACTTCATACC
CTTCACTACGTCCCTGACAGCTGTTCTGCTGCTAATCTTTTCCATGTGGAAACATCTGAAGAAGATGCAGGTCAGTGGCA
AAGGATCCCAAGATCCCAGCACCGAAGTCCACATAAGAGCCATGCAAACTGTGATCTCCTTTCTCTTGCTATTTGTCATT
TACTTCTTCGCTCAAATCATCTCATTTTGGAATTTTAGTATTATGCAGAACAATTCAGTTCCCTTGCTTTGCCAAGTTTT
TGGAATCCTGTATCCATCGAGCCACTCATTTATCCTGATTTGGGGAAATAAGAAGCTGAGACAGGCCTTTCTGTCACTTC
TGAGGCAGTTGAGGTGCTGGCTGAAGGAAAGGAGATAA
>MybrTAS2R38_NW_005367384.1:2942273-2941266
ATGTTGACTCTGACTCCCATCATAACTGTGTCCTATGAAGTCAAGACTGTGTTTCTGGTCCTTTCAGTCTTGGAGTTTGC
AGTGGGGATTCTGGTCAATGTCTTCATTTTCTTGGTGTATTTTTGGGATGTGGTGAGGAGGCAGCCCCTGAGCACCTGTG
ATCTTGTCCTGCTGAGTCTCAGCCTCAACCGGCTTTTCCTGCATGGACTGCTGTTTCTGGATGCCATTCAGCTTACTCAC
TTCCAGCAGATGAACGACCTGCTGAGCTTCAGGTACCAAACCATCATCATGCTCTGGATGATCACAAACCAAGCCGGCCT
CTGGCTCGCCACCTGCCTCAGCCTCCTCTACTGCTCTAAGATCGTCCGTTTCTCTCATGCCTCCCTGCTCTGCTTGGCCA
GCTGGATCTCCGGGAAGATTTCTAGGATGCTCCTGTGTACTGTCCTTTTCACCAGTGTATGCACTATCATCTGTTCTTGG
GACTTTTTTAGTAGATCTCACTTCACAGTCACAACCGTGCTATTCATGAGTAACAATTCAGAATTCAATTTGCAAATTGC
AAACCTCAAGTTCTTTCATTCCTTCCTCTTCTGCAGCGTGGGGTCCATCCCACCTTTCTTGTGTTTTCTGGTGTCTTCTG
GGGTGCTGATTGTCTCCCTGTGCCGCCACATGAGGACAATGAGGGCCAAGACCATGGACTCCTGTGACCCCGGCCTGGAG
GCCCACATCAAAGCACTCAAATCCCTCATCTCCTTTCTCTGCCTCTTTGTGGTGTCATTATGCGCTGCCCTCCTCTCAGT
GCCTTTACTGGTGCTGTGGCACAACAAGATCGGGGCCATGGTCTGTGTGGGGATAATGGCAGCCTGTCCCTCAGGGCACG
CAGCCATCCTGATCTCAGGCAATGCCAAGCTGCGGAGAGCTGTGGACAGCATTCTACTGTGGGTTCAGAGCAGCCGAAGG
GTAACGGCAGACCACAAGGCAGATCCCAGGACACCAGGTCTATGTTGA
>MybrTAS2R40_NW_005356173.1:436887-437843
ATGATGACGGTGAACACCAATTACGCGGATGAAGACATGTCCAGGTTTAAAGTGGTCTTCATCTTGGTGGTCTCTGGAAT
CGAGTGCCTCACTGGCATCGTTGGGAATGGTTTCATCACGGCCATCCACGGGGCCGAGTGGGCCAGACGCAAAAGACTCC
CCGTGGGGGACTGCATTGTGCTGATGCTGAGCTTCTCCAGGCTCTTGCTGCAGATTTGGATGATGCTGGAGAATGTGTAC
AGCCTACTATCCCGGGCCACTTACAACCAAAACACAGTGTATATACCTTTCAAAGTCATCGTCCTCTTTCTGAACTACTC
CAACCTCTGGCTCGCCGCCTGGCTCACCATCTTCTACTGTCTTAAAATTGCAAACTTTACGCACCCTTTGTTCGTCACGA
TGAAGAGGAAAATCACAGTGCTGATGCCCTGGCTTCTGAGGCTGTCGCTGCTCATCTCCTTGTGCTTCAGCTTCCCCTTA
ACTAAAGACATCTTCAATGTGTATGTGAATAGTTCCATTCCTATCCCCTCCCACAATGCCACAGAGAAGACGTACATCGC
TGAGACCAACGTGGTCAACCTGGTTCTTCTCTATAACCTGGGGATCTTCATTCCTCTCATCATGTTCATCCTGGCGGCCA
CCCTGCTGATCATCTCTCTCAAGAGGCACACCCTGCACATGAAAAGCAATGCCACTGGCTCCAGGGACCCCAGCATGGAG
GCCCACTTGGGGGCCATAAGAGCTATCAGCTACTTTCTCATTCTCTACATTTTCAATGCAGTTGCTCTATTTCTTTCCAT

GTCCAATGTCTTCGATGCCTACAGTTTCTGGAATATTTTATGCAAATTCATCATGGCCGCCTACCCTGCTGGCCACTCAC
TGCTGCTGATCGTGGGCAATCCTGGGCTGAGAAGAGCCTGGAAGCGGTTTCAGCACCGAGTTCATCTTCACCTGTAA
>MybrTAS2R41A_NW_005356173.1:691159-692085
ATGCAGCCAGCATTCACAGCCCTCTTCATGCTGCTCTTTGTCCTGCTGTGTCTCCTGGGAATCCTGGCCAATGGCTTCAT
TGTGCTGGTGCTGAGCAGAGAATGGAGGCGGCTTGGGAGGCTGCTCCCTTCTGACATGATCCTCATTAGCTTGGGTGCCT
CCCGTTTCTGCCTACAGTGGGTTGGAATGGTGCACAACTTTTACTCCTTCTTCCACCTGGAGGAGTTCAGCAAGGGTCCT
GCAGGGCAGCTCATTAGATTCCAATGGGACTTCCTGAATTCAGCCACCTTCTGGTTTGGTACCTGGCTCAGTGTCCTCTT
CTGCGTGAAGATTGCTAACCTCACCCACCCTACCTTCCTCTGGCTGAAGTGGAGGTTCCCAGGGTCAGTGCCCTGGCTTC
TGCTGGGCTCTCTCCTGATCTCCACCGTCGTTGCCCTGCTCTTCTTCTGGGGAAACTACTCTGTGAATCAAGGTTTCTTC
ATTAGAGAAGTTTATGAGAATATGACCTACATGGAGAGGGTCATTAGCATGGAAATTCACTATTTCCTACCCCTCAAATT
TGTCACGTTTTCAATTCCTTGCTCTGTTTTTCTGGTCTCGACTGCATTGTTGATTCATTCTTTGAGGAGACACACTCGGA
CAATGCGGCAAAGTGCCCATAGCCTGCAAGACGCCAGCACCGAGGCTCACACCAGAGCTCTGAAGTCACTCATCTTCTTC
CTCATTCTTTACATTCTGTCTTTCATGTCCCTGATCATTGATGCTGTAGGCTTCTTTTCAACAGAGAATGACTGGTTCTG
GCCATGGCAAATTGTAACCTACCTGTGCACATCTGTCCATCCCTTTATCCTTATCCTCAGCAGCCCCAGGCTTCGAGAGG
TGTTCAGGCAGCTACTTCTGTTGGCCAGGGGCTTCTGGCTGGTGTAG
>MybrTAS2R41B_NW_005370153.1:473860-474786
ATGCAGCCAGCATTCACAGCCCTCTTCATGCTGCTCTTTGTCCTGCTGTGTCTCCTGGGAATCCTGGCCAATGGCTTCAT
TGTGCTGGTGCTGAGCAGAGAATGGAGGCGGCTTGGGAGGCTGCTCCCTTCTGACATGATCCTCATTAGCTTGGGTGCCT
CCCGTTTCTGCCTACAGTGGGTTGGAATGGTGCACAACTTTTACTCCTTCTTCCACCCGGAGGAATTCAGCAAGGGTCCT
GCACGGGAGCTCTTTGGTCTCCATTGGGACTTCCTGAATTCAGCCAACTTCTGGTTTGGTACCTGGCTCAGTGTCCTCTT
CTGCATGAAGATTGCTAACCTCACCCACCCGACCTTCCTCTGGCTGAAGTGGAGGTTCCCAAGGTCAGTGCCCTGGCTTC
TGCTGGGCTCTCTCCTGATCTCCACCGTCGTTGCCCTGCTCTTCTTCTGGGGAAACTACTCTGTGAATCAAGGTTTCTTC
ATTAGAGAAGTTTATGAGAATATGACCTACATGGAGAGGGTCATTAGCATGGAAATTCACTATTTCCTACCCCTCAAATT
TGTCACGTTTTCAATTCCTTGCTCTGTTTTTCTGGTCTCGACTGCATTGTTGATTCATTCTTTGAGGAGACACACTCGGA
CAATGCGGCAAAGTGCCCACAGCCTGCAAGACGCCAGCACCCAAGCTCACACCAGAGCTCTGAAGTCACTCATCTTCTTC
CTCATTCTTTACATTCTGTCTTTCATGTCCCTGATCATTGATGCTGTAGGCTTCTTTTCAACAGAGAATGACTGGTTCTG
GCCATGGCAAATTGTAACCTACCTGTGCACATCTATTCACCCCTTTATCCTCATTCTTAGCAACCTCAGGTTTCGAGAGG
TGTTCAGGCCGCTACTTCTGTTGGCCAGGGGCTTCTGGCTGGTCTAG
>MybrTAS2R42_NW_005358943.1:6752518-6753513
ATGCTCACTGGATTGGAAATAATCTTTCTGATACTGTCAATAGCAGAATTCATAATTGGAATGTTGGGGAATGTGTTCAT
TGGACTGATAAACTGCTCTGAATGGGTCAAGAACCAAAACATCTCTTTAGCTGACTTCATCTTTACCTGCTTGGCTATCT
CCAGAATTAGTCAGTTGTTGGCATTACTTTTTGAATCACTTACATTGGGACTATTTTCACATGTATTTTCTACTTATAAA
CTAGCAAAACCTATTACTTTACTTTGGAGAATAACTAATCACTTGACTACCTGGCTTGCTACTTGCCTAAGCATTTTCTA
CCTCCTTAAGATAGCTCACTTCTCCCACTCTCTTTTCCTCTGGCTGAAGTGGAGAATGAAGAGAGTGATTCTTGTGATAT
TTGTATTTTCTTTTATCTTTCTGATTTTTGACTTTCTATTGTTAGAAAGCTTTAATGATTTATTTTTGAAGGCCTATATA
TATGATAATAGTAATCTGACTTTATATATAGAAGAAAGTAAGACTGTCTATTTTGAAACCCTGATTCTTCTTAGCTTGAC
CTGTTTGCTTCCTATTGTTCTGTCCCTGACCTCATTGCTCCTTTTATTTCTGTCTTTGGTAAGACATATCAGAAATTTGC
AGCTCAACTCCATGGGCTCAAGGGACTCCAGCACAGAGGCCCATAAAAGGGCCATAAAAATGGTGATGTCTTTTCTCTTT
CTCTTCGTATTTCATTTTTTTTCCACACAAGTGGTAAATTGGATATTTCTTATGTTTCCAGATTACATGATTGTAAAATT
TATCACGTTATTAGTCTATGTCTTTCCCTCAAGTCACTCATTTCTTTTGATTCTGGGAAACAGCAAGCTAAGACAGACAG
CCTTGAAGATACTATGGCATCTCAAAAGCTCCTTGAAAAGGGAAAATCTGTTACACCTTTACAGACAGATTTCTCAGAGT
CTTTTCAAAGATAATAACTTAACGAGGAAACTTTGA
>MybrTAS2R408C_NW_005358943.1:6760876-6759962
ATGGCTTTACTACCAGCCATTCTTTCCAGCCTATTCACAATACAATTTGTTCTAGGATATTTTGCCAATGGCTTCATAGC

ACTGGTGAACTGCATTGACTGGGTCAAGAGACAAAAGATCTCCTGCGCTGATGGAATTCTCACTGCTCTGGCTGTCTCCA
GAATTTGTTTGCTCTGTGTATTAGTACTAAATTGGTATGCAATTGTATTTAATCTAGCATTTTATAGTTTAGATGTAAAA
CTTATATTTCATATTGCCTGGATGACAAGCCACCATTTTAGTCTGTGGCTTGCTACTAGCCTTAGCATATTTTATTTGCT
CAAGATAGCCAATTTCTCCAGCCTTTTATTTCTTCACCTCAAATGGAGAGCTGAAAGAGTGGTTATCATGATACTGTTGG
GGGCTTTGGTCTTCTTGGTTTTTCATCTTGCAGTGGTAGGCACAGATGAAAAAATGCAGATGAATGAAGATAAAGGAAAC
ATCACTTGGGAGACTAACTTGGGGGACATTATGCACCTTTCAAATCAGACTTTATTCATGCTTGCAAACTTCATACCCTT
TACTATGTCCCTGGCAGCTGTTCTGCTGTTAATCTTTTCCATGTGGAAACATCTGAAGAACATGCAGCTCAGTGGCAAAG
GAACTCAAGATCCCAGCACCGAGGTCCACATAAGAGCCATGCAAACTGTGATCTCCTTTCTCTTGGTATTTTTCATTTTT
TTCTTCACTCAAATCATCTCACTTTGGAATTCGAGTACTCAGCAGAACAATTCGCTTCACATGGTTTGCAAGGTTCTTGG
AATCCTGTATCCGTCAAGCCACTCATTTATTCTGATTTGGGGGAATAAGAAGCTGAGACAGGCCTTTCTGTCATTTCTGT
GGCAGTTGAGGTGCTGGTTGAGGAAAGGGAAATAA
>MybrTAS2R408B_NW_005358943.1:6776412-6777326
ATGGCTTTACTACCAGCCATTCTTTCCAGCCTATTCACAATACAATTTGTTCTAGGATATTTTGCCAATGGCTTCATAGC
ACTGGTGAACTGCATTGACTGGGTCAAGAGACAAAAGATCTCCTGCGCTGATGGAATTCTCACTGCTCTGGCTGTCTCCA
GAATTTGTTTGCTCTGTGTATTAGTACTAAATTGGTATGCAATTGTATTTAATCTAGCATTTTATAGTTTAGATGTAAAA
CTTATATTTCATATTGCCTGGATGACAAGCCACCATTTTAGTCTGTGGCTTGCTACTAGCCTTAGCATATTTTATTTGCT
CAAGATAGCCAATTTCTCCAGCCTTTTATTTCTTCACCTCAAATGGAGAGCTGAAAGAGTGGTTATCATGATACTGTTGG
GGGCTTTGGTCTTCTTGGTTTTTCATCTTGCAGTGGTAGGCACAGATGAAAAAATGCAGATGAATGAAGATAAAGGAAAC
ATCACTTGGGAGACTAACTTGGGGGACATTATGCACCTTTCAAATCAGACTTTATTCATGCTTGCAAACTTCATACCCTT
TACTATGTCCCTGGCAGCTGTTCTGCTGTTAATCTTTTCCATGTGGAAACATCTGAAGAACATGCAGCTCAGTGGCAAAG
GAACTCAAGATCCCAGCACCGAGGTCCACATAAGAGCCATGCAAACTGTGATCTCCTTTCTCTTGGTATTTTTCATTTTT
TTCTTCACTCAAATCATCTCACTTTGGAATTCGAGTACTCAGCAGAACAATTCGCTTCACATGGTTTGCAAGGTTCTTGG
AATCCTGTATCCGTCAAGCCACTCATTTATTCTGATTTGGGGGAATAAGAAGCTGAGACAGGCCTTTCTGTCATTTCTGT
GGCAGTTGAGGTGCTGGTTGAGGAAAGGGAAATAA
>MybrTAS2R408D_NW_005358943.1:6792721-6793698
ATGCGCAGTAATATTACGTTTGGCACTTTCTTTGCCACGCTTGACTTTTCCCCCCAAGATCTGATAAGCTTACTACAAAT
TTTTTTTTCCATGCTAATAATGACAGGATTTATTCTAGGAAATTTTGCCAATGGCTTCATAGCACTGGTGAACTGCATTG
ACTGGATCAAGAAACACAAGATCTCCTGTGCTGACCGAATCCTCACTGCTCTGGCTGTCTCCAGAATTGGTTTGCTCTGG
ATAATAGTATTCAATTGGTATGGAACTGTGTTTAATCTACCTTTCTATAGTTCAGAAGTAAGTACTACTGTTTACATGGT
CTGGATAGTAAACCACCATTTTTGTCTCTGGCTTGCTACTAGCCTCAGCATCCTTTATTTTCTCAAGATAGCCAATTTCT
CCTGTCTTTTATTTCTTCACCTAAAATGGAGAGCTGAAAGAGTGGTTATCATGATACTGTGGGGGAATTTGTTCATCTTG
GTTTGTCATCTTACAGTGCTAAGCATAGATGCAAAAATGAAGAAGAATGATTATGAAGGAAACGTCACTTGGAAGACCAA
CTTGAGGGACATTATGCACCTTTCAAGTATGACTGTATTTATGCTTACAAACTTCATACCCTTCACTATGTCCCTGACAG
CTTTTCTGCTGCTAATCTTTTCCCTGTGGAAACATCTGAAGAAGATGCAGCTCAGTGGCAAAGGAACTGAAGATCCCAGC
ACCGAGGTCCACGTAAGAGCCATGCAAACTGTGATCTCCTTTCTCTTGCTATTTGTCATTTATGTCCTCGCTCAAATCAT
CTCAATTTGGAGACCCCAAACTCTGCAGAATAATTCAGTTCTCATGCTTTGTGAGGTTCTTGGAATCGTGCATCCTTCAA
CCCACTCATTGATCCTGATTTGGGGGAACAAAAAGTTAAGACAGGCCTTTCTGTCATTTCTGTGGCAGCTGAGGTGCTGG
CTGAAGGAAAAGAAATAA
>MybrTAS2R408E_NW_005358943.1:6786034-6786963
ATGATGAGTTTATTCCTGAGCATTCTTTCCACACTAGTTATAGCAGGATTTGTTCTAGGAAACTTTGCCAATGGCTTCAT
AGCACTGGTGAACTGCATTGACTGGGTCAAGAGACAAAAGATCTCCTGCGCTGATGGAATTCTCACTGGTCTGGCGGTGT
CCAGAATTGGTTTGCTCTGGGTAATAATATTCCATTGGTATGCAACTCTGTTTAATCCAGCTTTGTATAGTTTAAGAGTA
AGAACTGTTGCTGCTATTGTCTGGGTAGTAAGCAACCATTTTAGCCTCTGGCTTGCTACCAGCCTCAGCATATTTTATTT
GCTCAAGATAGCAAATTTCTCCAGCCTATTTTTTCTTCACCTAAAATGGAGAGCTAAAAGAGTGGTTCTCATGATACTGT

GGGGGACTTTGGTCTTCTTGGTTTTTCGTCTTGCAGTGTTAAGCATAGATGAAGAAATGAAGATGAGTGAATATAAAGGA
AACATCACTTGGAAGACCAACTTGAGGAACATTATACACCTTTCAAATTTGACTATATTTACGCTCGCAAACTTCATACC
CTTTTCTGTGTCTCTGACAGCTGTTCTGCTGTTAATCCTTTCCCTGTGGAAACATCTCAAGAGGATGCAGCTCAGTGGTA
AAGAAACTCAAGATCTCAGCACCAAGGTCCATGTAAGGGCCATGCAAACTGTGATCTCCTTTCTCTTGCTATTTGCCATT
TACTTTGTGACTCTAATCATCTCAGTTTGGAGTTTCTATAATCCTCAGAATATACCAGTTTTTCTGTGTTTCCAGGTTTT
GGCACTTGTCTATGTTTCAGGCCACTCGCTAATTCTGATTTGGGGAAACAAGAAGCTAAATCAGGACTTTCTCTCAGCTT
TATGGCAGGTGAGATGCTGGCTGAAAGAATGGAAACCTTCAACACTATAG
>MybrTAS2R18A_NW_005358943.1:6899397-6900317
ATGTCCATTGTAATAAAGGTCTCCATTATAGTTGTGGCAACAGGAATACTCATCTTAGGAGTGCTAGGAAATGGATTCAT
CGGACTGGTGAACTGCATCGAATGGTTCAGGACTGGGAAAGTTTCTTCAGTTGATTTCATCCTGACCAGCTTGGCTCTGG
CCAGAATCATCCATCTGTTGCTAACACTATTGGATTCATTTATAATAGGGCTGGCTCCACATCTGTATGCTACTGGTAAA
CTAGCAAAGGTGGTTACTATTCTTTGGGCACTAACTAATCAACTAACTATCTGGTTTGCCACCTGCCTCAGCATTTTCTA
CTTCCTTAAGATAGCCAATTTCTCCCACTCCTTTTTCATGTGGCTGAAGTGGAGAGTCAACAGAGTGGTTCTTCTGCTTT
TCCTTGGGTCTTTCTTCCTACTGTCTCTCAATATCTTAATGCATGATGCTGTTAGTGAATTTTGGTTGAATACCTACAGG
GTACATGAAATAAATATGACTTTGCAGTTAGAGGTAAATGAAATGTTCTATCTCAAAAGTCTTCTTCTTCTTACTTTGAC
CTACATTATCCCCTTTTTCCTGTCCCTGATCTCTTTGCTTCTTTTATTTCTGTCCTTGGTGAGACACACCAAGAATTTTC
AGCTCAACCTGACAGGCTCAAGCACAGAGGCCCATAGAAGGGCCATGAAGATGGTGACAGCCTTCCTCCTGCTCTTCATC
ATTTACATTATTTCTATTCTAACGGCATGTTGGATCTTCACTAATTTACAGACATATCAGGTCAAGATGTTTGTCATGAT
GATTTTGATTACCTTTCCCTCAGGCCACTCATTTATTATAATTTTGGGAAACAGCAAGCTAAGACAGATCTCCTTGAGAC
TACTCAGGAACTTAAGTTCTCTGAGAAAAGCACAACTTTAA
>MybrTAS2R18C_NW_005358943.1:6886657-6887664
ATGTCCACCTGCAACTGTTTCACTATAAAGCTTTCATTGCGGCACAACATTAGAAACATTCTTATACAGGTGTCAACTGA
AATGAAGGCCTCCATTCTAGTTGTGGCAACAGGAATATTCATCTTAGGAGTGCTAGGAAATGGATTCATCGGACTGGTGA
ACTGCATCGAATGGTGCAGGACTGGGAAAGTTTCCTCAGCTGATTTCATCCTCACCAGCCTGGCTCTGGCCAGAATCATT
CAACTGTTGGTAATACTCTTGGATTTATTTATAATGGGGCTGGCTCCACATCTGTATGCTACTGGTAAACTAGCAAAGGT
GGTTCCTATTCTTTGGGCACTAACTAACCACCTAACTATCTGGTTTGCCACCTGCCTCAGCATTTTCTACTTCCTTAAGA
TAGCCAATTTCTCCCACTCCTTTTTCATGTGGCTGAAGTGGAGAGTCAACAGGGTGGTTCTTCTGCTTTTCCTGGGGTCT
TTCTTCCTACTGTCTCTTAACCTCTTAATGCGTGATGCTATTAATGAATTGTGGCTGAACACCTACAGGTTACATGAAAT
AAATATGACTTTGCAGTTAGAGGCAAATGAAATATTCTATCTCAAAAGTCTTCTTCTTCTTAGTTTGACCTATGTTATCC
CCTTTTTCTTGTCCCTGATTTCTTTGCTTCTTTTATTTCTGTCCTTGGTGAGACACACCAAGAATTTTCAGCTCAACTTG
ACGGGCTCGGTGGACTCAAGCACAGAGGCCCATAGAAGGGCCATGAAAATGGTGACAACGTTCCTCCTCCTCTTCATCAT
TAACATATTATCTATTCTAACTGCAATTTGGATCTTCAATAAGGTAGAGACCTATCAGATCACGATGCTTGTCACAGTGT
TGTCAGCTACCTTTCCCTCAGGCCACTCTTTTCTTATAATTTTTGGAAACAGCAAGCTAAGACAGATCGCCTTGAGGCCA
CTGTGGCACTTAAATTCTCTGAGAAAAGCAAAACCTTTGCCTTCATAG
>MybrTAS2R18G_NW_005358943.1:6837073-6837915
ATGTCATTTGAAATGAAGGCCTCCTCTGTGGTTGTGGCAACAGGAATATTCATCTTAGGAGTGCTAGGAAATGGATTCAT
CAGACTGGTGAACTGCATCGAATGGTTCAGGACTGGGAAAGTTTGCTCAGCTGATTTCATCCTCACCAGCTTGGCTCTGG
CCAGAATCATTCAACTGTTGGTAATACTGTTGGATTCATTTATAATGGGGCTAGCTCCACATCTGTATGCTACTGGTAAA
CTAGCAAAGGTGGTTAGTATTCTTTGGGCACTAACTAACCACCTAACTATCTGGTTTGCCACCTGCCTCAGCATTTTCTA
CTTCCTTAAGATAGCCAATTTCTCCCACTCCTTTTTCATGTGGCTGAAGTGGAGAGTCAACAGGGTGGTTCTTCTGCTTT
TCCTGGGGTCTTTCTTCCTACTGTCTCTTAACCTCTTAATGCATGATGCTATTAATGAATTGTGGTTGAATACGTACAGG
GTACATGAAATAAATATGACTTTGCAGTTAGAGGCAAATTTGGTGAGACACACCAAGAATTTTCAGCTCAACCTGACGGG
CTCGGTGGACTCAAGCACAGAGGCCCATAGAAAGGCCATGAAAGTGGTGACAACGTTCCTCCTCCTCTTCATCATTTACA
TATTATCTATTCTAACTGCAATTTGGAACTCCACTAAGGTAGAGACATATCAAATCATGATGCTTGTCATAGTGTTGTCA

GCTACCTTTCCCTCAGGCCACTCTCTTCTTATAATTTTTGGAAACAGCAAGCTAAGACAGATCGCCTTGAGGCCACTGTG
GCACATAAATTCTCTGAGAAAAGCAAAACCTATGCCTTTATAG
>MybrTAS2R67_NW_005358943.1:6767745-6768674
ATGCCATCTGGAGCTGAAAGTATTTTTCTGGTAGCTGCAATGGGAGAATTCATAGCTGGAATGTTGGGGAATGGGTTCAT
TGTACTAGTTAATTGCATTGATTGGGTGAAGAGTCAAAAACTCTCAGCAGCTGACTGCATCCTCACCAGCCTGGCTCTCT
CCAGAATCACCCTTCTTTGGATATCACTAGCTGACTCATTTCTAATGGTGTTGTGGCCACAATTTTATGCCATTGGTAAA
CTAGCAAAACTTATTGGCATTCTTTGGATACTGTGCAATCACCTAGCTACCTGGGTTGCCACCTGTCTAAGCATTTTCTA
TTTCTTTAAAATAGCCAATTTCTCTCACCCCTGCTTTGCCTGGCTGAGGTGGAGAATTGGCAGAGTGCTACTTGTGCTTT
TATTGGGGTCTTTGTTCTTCCTGTTTTTGAAACTTGTGTTAATAGACTCATTCAATGGTTTCTGGATTAATGTCTATAAT
ATACATGAAAGAAACTCAACATGGTCTCCAGGTGTAAGTGAAACTCTGTATCTTAACAGTTTGATTGTTTCTAATTTTAT
CTACTTAATTCCCTTTCTTCTGTCCCTGAGTTCACTGCTCCTTTTATCTTTTTCCTTGATGAGACATACCAGGAATGTGC
GGATGAACTCCAGCTCTAACGACTTCCGCACAGAAGCCCATAAAAAGGCCATGAAAATAGTGTTATCTTTCCTCCTCCTC
TTCATACTTCATTTTTCTTCCTTTGTATTAACAGGTTGGTGTTTCCTTATAGTGCAGAAGCAGCAGGCCAATCTGGCTGT
CATGTTAACATGGAGTATTTTCCCTTCGGGCCACTCATTTATCCTAATTTTGGGAAACAGCAAGCTGAGACAAACTGCAT
TGAAACTATTGTGGCATCTTAACTGCCACCTGAAAAAGGGTGAAACTTAG
>MybrTAS2R39P_NW_005356173.1:416748-417880
ATGAATAGTGAGAGCTACCAACGATCTGCCCATCAAATGCTAGGGAGACAATCTCCAACAGACACCAAAGAGGAGTAACG
ACTCAGGATGACCAACACCTGCAGTCCCCCAGAGGATAATTTGTCACCACTTAATATCATCTTAATTTTCACAGTTATGG
GCACGGAATGCATCATTGGCATCGTTGCAAATGGGTTCATTGTGGCTATAAATGCAGCTGAGTGGATTAAGAATAAGGCA
GTCTCCACAAGTGGCAGGATCCTGTGTTTCCTGAGCATATCCAGAATAGCTCTCCAAAGCTTGATGATGCTAGAAATTAC
TTTCCACTCAACATCCCCACAATTTTATTATAAAGATGGTGTATATGATACCTTGAAAGTGAGTTTTGTGTTCTTACATT
ATTGTAGCCTCTGGTTTTCTGCCTGGCTCAGTTTCTTCTACTTCGTGAAGATTGCTGATTTCTCCTACCGCCTTTTCCTC
AAGCTGAAGTGGAGAATTACTGGATTGATGCCCTGGCTTCTGTGGCTATCAGTGTTTTTTGCCTTGGGCTACAGTATGTT
CTTTTCCTATGGCATATACACTGTTTATTGTAACAATTCTTTTCCTATCCCCTCCTCCAACTCTACTAAGAAAATATACA
TCACTGAGACCAACGTGGTCAACCTGGTTCTTCTCTATAACCTGGGGATCTTCATTCCTCTCATCATGTTCATCCTGGCG
GCCACCCTGCTGATCATCTCTCTCAAGAGGCACACCCTGCACATGAAAAGCAATGCCACTGGCTCCAGGGACCCCAGCAT
GGAGGCCCACTGATCATCTTCAAGAGGCACACCCTGCACATGAAAAGCAATGCCACTGGCTCCAGGGACCCCAGCATGGA
GGCCCACTTGGGGGCCATAAGAGCTATCAGCTACTTTCTCATTCTCTACATTTTCAATGCAATTGCTCTATTACTCTATA
TGTCCAACATCTTCAATGCCAACAGTTCCTGGGATATTTTGTGCAAAATTATCATGGCTGCCTATCCTGCTGGTCACTCC
ATTCTACTGATTCAGGACAACTCTGGGTTGAAAAGAGCCTGGAAGCGGCTTCAGTCTCAAGTTCATCTTTACCTAAAAAA
GTAAACTCTGTGA
>MybrTAS2R60P_NW_005356173.1:657227-658094
ATGGTTTCAGGACTTCCAGTGATGGATAAGAGAGCCGTCACCTTGGATATCATTTTATTCCTTTTGTGTCTGATAGCTGT
GGTGAAGAATGGCTTAATTACTGCAGAACTAGGCATGCAGTGGTTGCTGTGGAGAATATTGTCACCTTGAGATAAATTAT
TGATCAGCCTGGAGCCTCTTGGTTCTGTCTGTAATGGGTGTTGATCAGTGAGAGAATTTGTGGTTTTTCTGTATCCAAAG
TCCTTCCTGTACAATCCTGTACTCCAGTTCCTCGCCTTCCACTGGGACTTCTTGAATGATGCTACCCTATGGTTTTCCTA
CTGGCGCAGTGTTTTCTATCATGTGAAAATTGCAATCCTCACTCACCCTGTCTTCCTCTGCCTAAAGCAGAAGGTGTTTG
GGTTGGGTTGCTCAGCTCTGTGGGGTTCTTCAGCTTGAATGCCATCTTATTTTTCATAAGCAATGAAAGCATATATAGGA
ATTATTTAAGGAGAGGTCTGCAATCTTGGAATGCCACTGGAAATACTATCAGGAGAATATTTGAGAAATTCTACTTCTTC
CCTCTAAAAATTATTACTTGAACAGTCCCTACCATTGTCTTCTTCACTGGCATGTTTTGCTCATCACATCTTTGGGAAGA
CACGACAAAAAGACCTTCCTATATATCTTGGGCTCTCATGCCCCAGTGCTCATGCACACATCAAGGCTCTCACTGGTGCT
CAACACTGCATGTATTTTCCCATCTCAGGAATTTAAATGGAAGGTGGTGATTTACCTGTCCACAGTGGTCCACCCCATCG
TTCTACTGGTGAGCAACCCCGGCTGAGAGCTGGAGAGGGGATGCTCCTTAAGGTGTGGGGCATCTTGA
>MybrTAS2R18DP_NW_005358943.1:6879091-6880020

ATGTCCATTGGAATGAAGGTCTCCATTCTACTTGTGGCAACAGGAATACTCATCTTAGGAGTGCTAGGAAATGGATTCAT
CGGACTGGTGAACTGCATCGAATGGTTCAGGACTGGGAAAGTTTCCTCAGCTGATTTCATCCTCACCAGCTTGGCTCTGG
CCAGAATCATCCATCTGTTGCTAACACGATTGGATTCATTTATAATGGGGCTGGCTCCACATCTGTATGCTACTGGTAAA
CTAGCAAAGGTGGTTCCTATTCTTTGAGCACTAACTAATCACCTCACTATCTGGTTTGCCACCTGCCTCAGCATTTTCTA
CTTCCTTAGGATAGCCAGTTTCTCCCACTCCCTTTTCATGTGGCTAAAGCAAAGAGTCAACAGGGTGGTTCTTCTGCTTT
TCCTGGGGTCTCTCTTCCTATTGTCTTTTAATCTCTTAATGCATAATGCTGCTAGTAAATATTGGTTGAATACCTACAGG
GTATATGAAATAAATATGAGTTTGCAGTTAGAGGTAAATTAAGTGTTCTATCTTAAAAGTCTTCTTCCTCTTAGTTTGAC
CTATGTTATCCCCTTATTCCTATCCCTGATCTCTTTGCTTCTTTTATTCCTGTCCTTGGTGAGACACACCAAGAATTTTC
AGCTCAACCTGACAGACTTAGGAGACTCAAACACAGAGGCCCATAGAAGGGCCATGAAAATGGTGACAGCCTTCCTCCTC
CTCTTTATCATTTACATTATTTCTATTGTAACATCATGTTGGATCTCCGCTAAGGTACTGACATTTCAGGTCAAGATGCT
TGTCATGATGATTTCAACTACCTTTCCCTCAGGCCACTCCTTCATTATAATTTTTGGAAACAGCAAGCTAAGACAGATTG
TCTTGAGACTACTCTGGCACTTAAATTCTCTGAGAAAAGCAAAACTTTAA
>MybrTAS2R16ET_NW_005368699.1:28-477
TCAAGAGTCAGTTCAATTCCATGATGCAATTACCTGGAAACAGCACAGAGACTGAGACACTTATGACATTCCTGGAGAAA
AATTACGTACATCAGCTTCTGGCAACATCGTTCATTCCTTTCCTACTGTTCCTGACCTCCACCATCTTGCTCATAGCCTC
ATTGTGCCAACACTTGAGGCAGAAAAGACATCACGACACTGGCCACAGCAACTTCAGCATGAAAGCTCATGCCACTGCCC
TGAGGTTTCTTGCCTTCTTCCTCATATTCTTCACCTCTTACTTTTTGACCATAATCATCTCCACTACATATTACCTAAGG
TATAAGAGTTACTGGTTCTGGGCCGGCGAAACTATTATCTATGCTACAGTCTCTATTCATTTAACTTCACTAATGCTGAG
TATTCCTGCGTTTAAAAAGGTTTTAAAGGTAAGCTGCTGTGGCCCAAAGC
>MybrTAS2R18B1T_NW_005358943.1:6888604-6888787
ATGTCAATTGCAATAAAGGTCTCCATTCTAGTTGTGGCAACAGGAATATTCATCTTAGGAGTGCTAGGAAATGGATTCAT
CGGACTGGTGAACTGCATCGAATGGTTCAGGACTGGGAAAGTTTCCTCAGCTGATTTCATCCTCACCAGCTTGGCTCTGG
CCAGAATCATTCAACTGTTGGTAA
>MybrTAS2R18B2T_NW_005358943.1:6889212-6889496
GAGACTCAAGCACAGAGGCCCATAGAAGGGCCATGAAAATGGTGACAACGTTCCTCCTCCTCTTCATCATTTACATTATT
TCTATTCTAACTGCAACTTGGATCTTCACTAAGGTACAGACATACCAAGTCATGATGCTTGTCACAGTGTTGTCAGCTAC
CTTTCCCTCAGGCCACTCTTTTCTTATAATTTTTGGAAACAGCAAGCTAAGACAGATTGCCTTGAGACTACTGTGGCACT
TACGTTCCCTGAGAAAATACAAGTTTAACTTTATAGACAGAATTTGA
>MybrTAS2R18E1T_NW_005358943.1:6866952-6867135
ATGTCAATTGCAATAAAGGTCTCCATTCTAGTTGTGGCAACAGGAATATTCATCTTAGGAGTGCTAGGAAATGGATTCAT
CGGACTGGTGAACTGCATCGAATGGTTCAGGACTGGGAAAGTTTCCTCAGCTGATTTCATCCTCACCAGCTTGGCTCTGG
CCAGAATCATTCAACTGTTGGTAA
>MybrTAS2R18E2T_NW_005358943.1:6867918-6868095
TGCAATTTGGAACTCCACTAAGGTAGAGACATATCAAATCATGATGCTTGTTATAGTGTTGTCAGCTACCTTTCCCTCAG
GCCACTCTTTTCTTATAATTTTTGGAAACAGCAAGCTAAGACAGATCGCCTTGAGACTACTGTGGCACTTACATTCCCTG
AGAAAATCGCAAGTTTAA
>MybrTAS2R18F1T_NW_005358943.1:6838855-6839038
ATGCCAATTGAAATAAAGGTCTCCATTCTAGTTGTGGCAACAGGAATATTCATCTTAGGAGTGCTAGGAAATGGATTCAT
CGGACTGGTGAACTGCATCGAATGGTTCAGGACTGGGAAAGTTTCCTCAGCTGATTTCATCCTCACCAGCTTGGCTCTGG
CCAGAATCATTCAACTGTTGGTAA
>MybrTAS2R18F2T_NW_005358943.1:6839611-6839789
GCAATTTGGAACTCCACTAAGGTAGAGACATATCAAATCATGATGCTTGTTATAGTGTTGTCAGCTACCTTTCCCTCAGG
CCACTCTTTTCTTATAATTTTTGGAAACAGCAAGCTAAGACAGATCGCCTTGAGACTACTGTGGCACTTACATTCCCTGA
GAAAATTACAAGTTTAA

>MybrTAS2R18HT_NW_005358943.1:6829492-6830136
ACTATCTGGTTTGCCACCTGCCTCAGCATTTTCTACTTCCTTAAGATAGCCAATTTCTCCCACTCCTTTTTCATGTGGCT
GAAGTGGAGAGTCAACAGGGTGGTTCTTTTGCTTTTCCTGGGGTCTTTCTTCCTACTGTCTCTTAACCTCGTAATGCATG
ATGCTATTAATGAATTGTGGTTGAATACCTACAGGGTACATGGAATAAATATGACTTTGCAGTTAGAGGCAAATGAAATG
GTCTATCTCAAAAGTCTTCTTCTTCTTACTTTGACCTATGTTATTCCCTTTTTTCTGTCTCTGATCTCTTTGCTTCTTTT
ATTCCTGTCCTTGTTGAGACACACCAAGAATTTTCAGCTCAATCTGACGAGCTTGAAAGACTCAAGCACAGAGGCCCATA
GAAGGGCCATGAAAATGGTGACAACGTTCCTCCTCCTCTTCATCATTAACATATTATCTATTCTAACTGCAATTTGGATC
TTCAATAAGGTAGAGACCTATCAGATCACGATGCTTGTCACAGTGTTGTCAGCTACCTTTCCCTCAGGCCACTCTTTTCT
TATAATTTTTGGAAACAGCAAGCTAAGACAGATTGCCTTGAGACTACTGTGGCACTTACGTTCCCTGAGAAAATTACAAG
TTTAA
>MybrTAS2R18IT_NW_005358943.1:6821740-6821889
ATGTCATTTGAAATGAAGGCCTCCTTTCTGGTTGTGGCAACAGGAATATTCATTTTAGGAGTGCTAGGAAATGGATTCAT
CGGACTGGTGAACTGCATCGAATGGTTCAGGACTGGGAAAGTTTCCTCAGCTGATTTCATCCTCACCAGCG
>MybrTAS2R18JT_NW_005358943.1:6813511-6814163
ATGTCATTTGAAATGAAGGCCTCCTTTCTGGTTGTGGCAACAGGAATATTCATTTTAGGAGTGCTAGGAAATGGATTCAT
CGGACTGATGAACTGCATCGAATGGTTCAGGACTGGGAAAGTTTCCTCAGCTGATTTCATCCTCACCAGCTTGGCTCTGG
CCAGAATCATTCAACTGTTGGTAATACTCTTGGATTCATTTATAATGAGGCTAGCTCCACATCTGTATGCTACTGGTAAA
CTAGCAAAGGTGGTTAGTATTCTTTGGGCACTAACTAACCACCTAACTATCTGGTTTGCCACCTGCCTCAGCATTTTCTA
CTTCCTTAAGATAGCCAATTTCTCCCACTCCTTTTTCATGTGGCTGAAGTGGAGAGTCAACAGGGTGGTTCTTCTGCTTT
TCCTGGGGTCTTTCTTCCTACTGTCTCTTAACCTCTTAATGCGTGTTGTTCCTAGTGAATTGTGGTTGAATACGTACAGG
GTACATGAAATAAATATGACTTTGCAGTTAGAGGCAAATGAGATGTTCTCTATTAAATCTCTTCTTATTATTACTTTGAC
CTATATTATCCCCTTTTTCCTGTCCCTGATCTCTTTGCTTCTTTTATTCCTGTCCTTGGTGAGACACACCAAGAATTTTC
AGCTCAACCTGAC
>FecaTAS2R1_chrA1:231771551-231772447
ATGCTAGACTTTTACCTCATTATCCATTTTCTTCTTCCAGTGATACAATGTCTCATCGGAGTTTTAGCAAATGGCATCAT
TGTGATCGTGAATGGCACTGAGTTGATCAAGCAGAGAAAGATGGTTCCGTTGGATCTCCTTCTTTCCTGCCTGGCGATTT
CCAGGATTTGTCTGCAGTCATTTATCTTCTACATTAATCTGGTTATTCTCTCCTTGATCGACTTCCTTCCACTTGTTAAG
AATTTTGCGGTTTTCATGTTTGTAAATGAAACGGGACTTTGGCTGGCCACATGGCTCGGCGTTTTCTACTGCGCCAAGAT
CTCCCCCATCGCTCACCCACTCTTCTTCTGGTTGAAGAGGAGGATATCCAAGTTGGTGCCATGGCTGATCATCGGGTCTC
TGCTTTTTGCCTCCATCCCTTTGGTTTTCTACAGCAAGCATACGTGGGTTCTTTCCCAAGAAGTCTTGTTGAGACTTTTC
TCCCCAAATGCAACAACTCAAATCAAAGAAACATCTGCTTTACAGATTGTCTTTCTTGCTAGGTTTTCACCGCCGTTCAT
TATCTTCCTCACTTCTACTCTGCTCCTGGTGTTTTCTCTGGGGAGACATACGTGGCAGATGAGAAACACAGCGACGGGCA
CCAGGGACGGTAGCACAGGTGTCCATGTGAGTGCGCTTCTGTCCATTCTGTCCTTCTTGGTCCTCTATCTCTCCCACTAC
ATGACAGCTGCTTTGCTCTCTTCTCACATTTTTGAGCTCAGAAGCTTCATGTTTCTGTTCTGTATCTTGGTGTTCGGGTC
CTACCCTTCGGGACACTCTATTATCTTAATTTCGGGAAATCGTAAACTGAAACAAAATGCAAAGAAGTTCCTCCTCCATG
GGCAGTGCTGCCAGTGA
>FecaTAS2R2_chrA2:105909232-105910146
ATGGCCTCCTCTTTGTCAGCGATTCCTCACCTTATCATCATGTCAGCAGAATTTATCACAGGGATTACAGTAAATGGATT
TCTTGTAATCATCAACGGTAAAGAATTGATCAAAAGCAGAAAGCTAACACCAATGCAACTCCTTTGCATATGTATAGGGA
TATCGAGATTTGGTTTGTTGATGGTGTTAATGGTACAAAGTTTTTTCTCTGTGTTCTTTCCACTCTTTTATAGGGTAAAA
ATTTATGGTGCATCAATGTTGTTCTTTTGGATGTTTTTTAGCTCTGTCAGTCTTTGGTTTGCCACCTGCCTTTCTGTGTT
TTACTGCCTCAAGATATCAGGCTTCACTCAATCCTATTTTCTTTGGCTGAAATTCAGGATCTCAAAGTTAATGCCTTGGC
TGCTTCTGGGAAGCCTGCTGGCCTCCATGAGCATTGCCGCTGTGTCTTTGGATGTAGGTTACCCTAAAAACATGAACAAT
AATGATTTCCTCAAGAATGCCACGCTGAAGAAGACTGAACTCAAGATAGGGCCAATTAATGGAGTGCTTCTTGTCAACTT

GGCATTGCTATTTCCACTAGCCATATTTGTGATGTGTACTTTTATGTTATTCATTTCTCTCTATAGGCACACTCATCGGA
TGCAAAACAGATCTCATGGTGTTAGAAATGCCAGCACAGAAGCCCATATAAATGCATTAAAAACAGTGATAACATTCTTT
TGCTTCTTTATTTCTTATTTTGCTGCCTTCATGGCCAATATGACATTCAGTATTCCTTACGGAAGTCAGTGCTTCTTTGT
GGTAAAGGACATAATGGCAGCATTTCCCTCTGGACATTCAGTTATAATCATATTGAATAATTCTAAATTCCAACAACCAT
TCAGGAGACTTCTCTGCCTCAAAAAGAATCAATGA
>FecaTAS2R3_chrA2:156212612-156213562
ATGTCAGGGCTCCACAAGTGGGTGTTTCTGGTTCTGTCTGCCACTCAGTTCATTCTGGGGATGCTGGGGAATGGTTTCAT
AGTGTTGGTCAGTGGCAGCAGTTGGTTTAAGAATAAGACAATCTCTTTGTCTGACTTCATCATCGCTAACCTGGCTCTCT
CCAGGATCGTTCTGCTGTGGATTCTCTTGGTTGATGGTGTTTTAATTGTGTTCTCTTCCAAAGTGCATGATGAAGGGATA
ATAATGCAAATTATTGATATTTTCTGGACATTTACAAACCACCTGAGCATTTGGCTTGCCACCTGTCTCAGTGTCCTCTA
CTGCCTGAAAATTGCCAGTTTCTCTCACCCTACATTCCTCTGGCTCAAGTGGAGAGTTTCCAGGATGGTCGTACAGATGA
TCTTGGGTGCGCTGGTCTTATCGTGTGCCAGTGCCCTGTCTCTGATCCATGAATTTAAGATGTATTCTATTCTCGGTGGG
ATCGATGGCACAGGGAATGTGACTGAGCACTTTAGAAAGAAAAGAAATGAATATAAATTGATCCATGTTCTTGGGACTCT
GTGGAACCTGCCTCCTCTGATTGTGTCTCTGGCCTCCTACTTTCTGCTCATCGTCTCTCTGGGGAGGCACACGCAGCGGA
TGGAGCAAAGCGGCACCAGCTCCGGAGATCCAAGCGCTGAGGCCCACAAGAGGGCCATCAAAATCATCCTCTCCTTCCTC
CTTCTCTTCCTGCTTTACTTTCTTGCCTTTTTAATTACATCATCCAGTTATTTCATACCAGGAACTGAGATGGTGAAGAT
AATTGGAGAACTCATTACCATGTTTTATCCTGCTAGCCACTCATTCATTCTCATTCTGGGAAACAGCAAGCTGAAGCATA
TGTTTGTGGGGATGCTGCGGTGTGAGTCTGGTCATCTGAAGCCTGGATCCAAAGGACCTGTTTCCCTGTAG
>FecaT2R4_chrA2:156222478-156223398
ATGCATCAGATACTCTTCTTATCTGCTCTTACTGTCTCAGCAATTTTGAATTTTGTAGGACTCGTTGTAAATCTGTTTAT
CGTAGTGGTCAACTACAGGACTTGGGTCCAAAGCCACAGAATCTCCTCTTCTAATAGGATCCTGTTCAGCTTGGGCGTCA
CCAGATTTATTATGCTAGGACTGTTTCTCCTGAACATTATCTACCTGTTCACCTCTCCACATGTCGAAAGGTCAGTCCAC
CTATCCACTTTTTTCCTGTTGTGTTGGATGTTTTTGGAGTCTACTAGTCTCTGGCTTGTAACCTTGCTCAATGCCTTGTA
CTGCGTGAAGATTACTGACTTCCAACACTCAGTATTCCTCCTGCTGAAACGAAAGCTGTCCCCAAAGATCCCCAGGCTGC
TGCTGGCCTGCGTGCTGATCTCTGCCTTCTCCACTCTCCTGTATGTTGTGCTCACACAGACATCACCCTTTCCTGAGTTT
CTGACTGGGAGCAATGGTACAGTATGTGACATCAATAAGAGCATCTTGTCTTTGGTGACCTCCTTGGTCCTGAGCTCCTT
TCTCCAGTTCATCATGAATGTGACTTCCGCTTCCTTGTTAATACATTCCTTGAGGAGACATATACAGAAGATGCAGAAAA
ACGCCACTGATTTTTGGAATCCCCAGACTGAAGCTCTTATGGGTGCTATGAAGCTAATGATCTATTTCCTCATCCTCTAC
ATTCCATATTCACTTGCTACCCTGCTACAGTATCTCCCTTCCGTACGGATGGATTTGGGAGCCACATCCATCTGTATGAT
TATTTCCACCTTTTATCCTCCAGGACATTCTGTTCTCATTATTCTCACACATCCTAAACTGAAAACAAAAGCAAAGAAGA
TTCTTTGTTTCAACATATGGTGGAATTTCAGTAGTAAATAG
>FecaTAS2R7_chrB4-47491318-47490383
ATGCTGGATAAAGTGGAGAGCACCTTGATGCTCATAGCAGCTGGAGAATTTGCAATGGGGATTTTAGGGAATGCATTCAT
TGGATTGGTAAACTGCATGAACTGGATCAAGAATAGGAAGATTGCCTCCATTGACTTAATCCTCACAAGTCTGGCCATAT
CCAGAATTTGTCTATTATGTATCATACTATTAGACTATTTTATACTGGGGCTGTATCCAGATGTCTATACTACCGGTAAA
AAAATGAGAATCATTGACTTCTTCTGGACGCTCACCAACCACCTAAATGTCTGGTTTGCCACCTGCCTCAGCGTCTTCTA
TTTCCTCAAGATCGCGAATTTCTTCCATCCCCTTTTCCTCTGGATGAAGTGGAAAATTGACAGTGCGATTCCTAGGATCC
TGCTGGGATGCTTGGCCTTCTCTGTGTTCATTAGCCTTGTTGTCTCTGAGAATCTGAACGATGATTTCAGGTCTTGTGTT
AAGGTAAAGAAGAAAACAAACATAACTGTGAAATGCAGAGTAAATAAAGCCCAATATGCTTCCGTCAAGATTTGCCTCAA
CCTGTTGACGCTATTCCCCTTTTCCGTGTCCGTGATCTCATTTCTCCTCTTGCTCCTCTCCCTGTGGAGACATACCAGGC
AGATGAAGATCAGTGCCACGGGGTGCAGGGACCCCAGCATAGAAGCCCATGTGGGAGCCATGAAAGCTGTCATCTCCTTC
CTCCTCCTTTTCATTGCTTACTATTTGGCTTTTCTCGTAGCCACCTCCAGCTACTTTATGCCAGAGACTGAATTAGCTGT
GATGATTGGTGAGTTGATAGCTCTCATCTATCCAAGCCATTCATTGATTCTAATTCTGGGGAACAATAAATTACGGCAGG
CGTCTCTAAGGGTGCTGTGGAAAGTAAAGTGTATCCTAAAAAGAAGAAATCACTAA

>FecaTAS2R9_chrB4:47499536-47498523
ATGCCAAGTGCAGTGGAGGTAATATATATGGTCTTGATTGCTGGTGAATTGACTATAGGAATCTGGGGAAATGGATTTAT
TGTACTGGTTAACTGCACTGGTTGGCTCCAAAGGCGAGATAGCTCCGTGATTGACATCATCCTGGTGAGTTTGGCCATCT
CCAGAATCTGTGTGTTGTGTGTGGTATCTGCAGAAGGCTTTGTTCTGCTGCTCTCTCCACATGCGTATGCTCAAAATGAG
ACAATAAACACCTTGGATGCTTTCTGGACACTGAGCAACCATTCAAGTGTCTGGTTCACTGCTTGCCTCAGCATTTTCTA
CTTACTGAAGATAGCCAACATATCCCACCCGGTGTTCCTCTGGCTGAAGCTAAACGTTACCAGAGTCGTCCTGGGGCTTT
TTCTGGCGTCCTTCCTCACCTCCATAATTATTAGTGTCTTTTTGAAAGAGGGATCCTGGGGTCACGTCGAAGTCAATCAC
GAGGAAAACATAACTTGGGAATTCAGAGTGAGTAAAGCCCCAAGCGCTTTCAAACTGATTATCCTGAACCTGGGGGCTCT
AGTTCCCTTTGCTCTGTGCCTAATCTCCTTTGTCTTGTTACTTTTCTCCCTCTTTAGACACGCTAAGCAGATGCAACTTT
ACGCCACCGGGTCCAGGGACTGTAGCACAGAGGCACACATGAGGGCCATAAAGGCAGTGACCATCTTTCTGCTTTTCTTC
ATCATGTACTATGCAGTCTTTCTTGTAGTCACTTCTAGCTTCCTGATTCCCCAAGGACGGTTAGTGCTGATGTTTGGTGG
CATAGTCACTGTCATTTTCCCATCAAGCCATTCATTCATCCTGATCATGGGCAACAGCAAGCTGAGGGAGGCCTTTCTGA
AGGTGCTAAGGTGTGTGAAGGGCTTCCACAAAAGAAGGAAACCTCTTGTTCCGCAGAGAATCCTGAATACGGGGAGAAAG
AAATCAACAAAAGACTGTCTCCCTTCTCCCCGGGGGTTACATTCATTTGCTTAA
>FecaTAS2R10_chrB4:47514880-47513837
ATGTTAAGCATAGTGGAAGGCCTTCTCATTTTTATAGCAGTTAGTGAATCAGTACTGGGGGTTTTAGGGAATGGATTTAT
TGGACTTGTAAACTGTATGGACTGTGTGAAGAACAAAAAGTTTTCTATGATTGGCTTCATCTTCACCGGCTTAGCTACTT
CCAGAATTTGTCTGATATTGATAGTAATGGCAGATGGATTTATAAAGATATTCTCTCCAGATATGTACTCTTCTGGTCAC
CTAATTGATTATATTAGTTACTTATGGATAATTATCAATCAATCAAACATCTGGTTTGCCACCAGCCTCAGCACCTTCTA
CTTCCTGAAGATAGCAAATTTTTCCCACCATATGTTTCTCTGGTTGAAGGGTAGAATCAATTGGGTTCTTCCCCTTCTGA
TGGGATCCTTGTTTATTTCATGGCTCTTTACGTTCCCTCAAATTGTGAAGATTCTTAGCGACGGTAAAGTGGGGAATGGA
AACGCAACCTGGCAGCTCAACATGCCGAAGAGTGAGTTCTTAACTAAGCAGATTTTGGTCAACGTAGGAGTCCTTCTCCT
CTTCACGCTATTCCTGATTACATGTTTCCTGTTAATCATTTCCCTTTGGAGACACAGCAGGCGGATGCAATTGAATGTCA
CTGGATTCCAAGACCCCAGTACAGAAGCGCATATGAAAGCCATGAAAGTTTTGATATCTTTCATCATCCTCTTTATCTTG
CATTTTATAGGCCTGGCCATAGAAATAGCATGCTTCACAATGCCAGAAAAAAAATTGCTGTTTATTTTTGGTATGACGAC
CACAGTCTTGTACCCCTGGGGTCACTCATTTATCCTCATTCTCGGAAACAGCAAGCTAAAGCAAGCCTCTCTGAGAGCAC
TGCAGCAGGTCAAGTGCTGTTAA
>FecaTAS2R12_chrB4:47536442-47535513
ATGGCAAGCGTATTGAAGAATGTATTTATGATACTGTTTGCTGGAGAATTCATAATGGGGATTTTGGGAAATGGATTCAT
TATATTGGTTAACTGTATTGACTGGATCAGGAACTGGAAATTCTTCGTAATTGACTTTATTATTACCTGCCTAGCTATTT
CCAGAATAGTTCTGTTGTGCATAATAATTTTAGGCATAGGTTTAGATGTACCTTGTGAAGAAATATGGAACAAGAATAAT
CAACTAATAAGGTTTGAAATCCTCTGGACAGGATCCAATTATTTCTGCATAACCTGTACCACCTGCCTCAGTGTCTTCTA
TTTCTTCAAGATAGCCAACTTTTCCAACCCTCTTTTCCTCTGGATAAAATGGAGAATTCACAAAGTGCTTCTCACGATTG
TACTGGCCGCAGTCTTCTCTTTCTGCTTGTCTCTTCCCTTTAAGGATACAGTGTTCACGAGTCTGATCAAAAACAAGGTA
AACGCGGAAAGAAATTGGACAGTGAGTTTCACAATGAGAACATATGAGTTATTTTTGTCTCATATGCTCCTGAACATAAT
GTTCATCATCCCCTTTGCAGTGTCTCTGGCTTCCTTTGTCCTTTTGATCTGTTCCTTATGGAGCCACACCAGGCAGATGA
AGGGCAGAGGTGGGGATCCTACCACAAAAGTTCACGTGAGAGCCATGAAGGCTATGATTTCATTCCTACTCTTCTTCTTT
ATGTACTATTTGAGCACTATTATGATGAATTTGGCCTACGTCATCCTAGATAGTTTGGTGGCAAAGATTTTTGCTAATAC
ACTAGTATTTTTATATCCATCTGGCCATACATTTCTTCTGATTTTATGGACCAGCAAATTGAAACAGGCTTCTCTCTGTG
TCCTGAAGAAGCTGAAGTGCCTGCATCTAAGGAAACCCACACGCCCATAA
>FecaTAS2R408A_chrB4:47593985-47593089
ATGGTAACCGCGCTACCGAGCATTTTTTCCATCGTGGTAATAGAATTTCTCCTAGGAAATTTTGCCAATGGCTTCATAGC
ACTGGTGAACTTCATTGACTGGACCAAGAGACAAAAGATCTCCTCAGTTGATCACATTCTCACTGCTCTGGCTGTCTCCA
GAATTGGTTTGCTCTGGGTAATATTAATAAATTGGTATGCAACTTTGTTCAGTCCAGATTTCTATAGCTTAGAAGTAAGA

ATTATTTTTCAAACTGCCTGGACAGTAAGCAATCATTTTAGCATCTGGCTGGCTACTAGCCTCAGCATATTTTATTTGTT
CAAAATAGCCAACTTCTCCAGCCTTATTTTTCTTCGCCTCAAGTGGAGAGTTAAAAGCATAGTTCTTGTGATTCTGTTGG
GGTCCTTGTTCTTTTTGGTTTGTCATGTTGTGGCGGTGAGCGTGCGTGAGAAAGTGCAGACTGACGTATATGAAGGAAAC
GGCACTAGGAAGACCAAATTGAGGGACATTTTACAGCTTTCAAATATGACTATATTCACACTAGCAAACTTCATACCCTT
TGGTATGTCCCTGACGTCTTTTGTGCTGTTGATCTTTTCCCTCTGGAAACATCTCAAGAGGATGCAGCTCTGTGATAAGG
GATCTCAAGATCCCAGCACCAAGGTCCACATAAGAGCCATGCAGACCGTGGTCTCCTTTCTCTTGTTCTTTGCCGGTTAC
TTCTTTACTCTGACGATCACAATTTGGAGTTCTAATTGGCCGCAGAACGAGTTCGGCTTCCTCCTTTGCCAGGTTATTGG
AATCCTATATCCTTCAATCCACTCGTTGATGCTGATTCGGGGAAACAAGAAGCTAAGACAGGCCTTTCTGTCATTTTTGT
GGCAGCTGAAGTGCTGA
>FecaTAS2R408B_chrB4:_47572300-47571374
ATGGTAAGCGCGCTACCAAGCATTTTTTCCATCGCGGTAATAATAGAATTTCTCCTAGGAAATTTTGCCAATGGCTTCAT
AGCACTGGTGAACTTCATTGACTGGACCAAGAGACAAAAGATCTCCTCAGTTGATCACATTCTTGCTGCTCTGGCTGTCT
CCAGAATTGGTTTGCTCTGGGTAATGATAATAAATTGGTATGCAACTTGGTTCAGTCCAGATTTCAAGAGCTTAGAAGTA
AGAATTATTTTTCAAATTGCCTGGACAGTAAGCAATCATTTTAGCATCTGGCTGGCTACTAGCCTCAGCATATTTTATTT
GTTCAAAATAGCCAACTTCTCCAGCCTTATTTTCCTTCGCCTCAAGTGGAGAGTTAAAAGCATCGTGCTTGTGATGCTGC
TGGGGTCTTTGTTCTTATTGTTTTCTCATGTGGCGGCAGTGAGCATATATGAGAAAGTGCAGACTAAGGCATATGAAGGG
AATGTCACTTGGAGGACCAAATGGACGGGCATGGCACACCTCTCAAATATGACTGTATTCACACTAGCAAACTTCATACC
CTTTGCTACGTCCCTGACGTCTTTTGTGCTGTTGATCTTTTCCCTCTGGAGACATCTCAAGCGGATGCAGCTCTGTGGCA
AGGGATCCCAAGATCCCAGCACCAAGGTCCACATAAGAGCCATGCAGACGGTGGTCTCCTTTCTCTTGTTCTTTGCCGGT
TACGTTCTGAATCTAATTGTTACAATTTGGAGTTTTAACGGGCTGCAGAAGGAACTGTTCATGTTTTGCCAGGTACTTGC
CTTCGTGTATCCTTCGATCCACTCGCTGATGTTGATTTGGGGAAACAAGAAGCTAAAACAGGCCTTTCTGTCTGTTTTAT
ACCAGGAGAAGTACTGGCTGAAAGAACAGAAACACTCAACTCCATAG
>FecaTAS2R38_chrA2:156379999-156378995
ATGTTGGCTCTGACTCCTGTCATAACTGTGTCCTATGAAGTCAAGAGTGCATTTCTATTCCTTTCAATCCTGGAATTTAC
AGTGGGGGTCCTGGCCAATGCCTTCATTTTCCTGGTGAATTTTTGGGACGTGGTGAGGAAGCAGCCACTGAGCAACTGTG
ATCTTATTCTTCTGAGTCTCAGCCTCACCCGGCTTTTCCTGCACGGGCTGCTGTTTCTGGATGCCCTCCAGCTTACATAC
TTCCAGAGGATGAAAGATCCGCTGAGCCTCAGCTACCAGACCATCATCATGCTCTGGATGATCACAAACCAAGTTGGGCT
CTGGCTCACCACCTGCCTCAGTCTTCTCTACTGCTCCAAGATTGCCCGTTTCTCTCACACCCTCCTGCACTGTGTGGCAA
GCTGGGTCTCCCGGAAGGTCCCCCAGATGCTCCTGGGTGCAATGCTTTTCTCTTGTATCTGCACCGCCATCTGTTTGGGG
GACTTTTTTAGTAGATCTGGCTTCACATTCACAACTATGCTATTCGTGAATAATACAGAATTCAATTTGCAAATTGCAAA
ACTCAGTTTCTATCACTCCTTCATCTTCTGCACACTGGCGTCCATCCCGTCGTTGTTATTTTTTCTGGTTTCTTCTGGGG
TGCTGATTGTCTCCCTGGGGAGGCACATGAGGACAATGAGGGCCAAAACCAAAGACTCCCACGACCCCAGCCTGGAAGCC
CATATCAAAGCCCTCCGATCTCTTGTCTCCTTTCTCTGCCTCTATGTGGTGTCATTCTGTGCTGCCCTCGTTTCAGTGCC
TTTACTGATGCTGTGGCACAACAAGATCGGGGTAATGATCTGTGTGGGGATCCTAGCAGCTTGTCCCTCGATACATGCAG
CAATCCTGATCTCAGGCAATGCCAAGCTGAGGAGAGCTGTGGAGACCATTCTACTCTGGGTTCAGAACAGCCTAAAGATA
GGGGCAGACCACAAGGCAGATGCCAGGACTCCAGGCCTATGTTGA
>FecaTAS2R42_chrB4:47646667-47645699
ATGTTAGCCGGACTGGATAAAATCTTTCTTACGCTGTCAACGGCAGAATTCGTAATTGGAATGTCGGGGAATGTGTTCGT
TGGACTGGTGAACTGCTCTGAATGGATCAAGAACCAAAAAATCTCTTTTGTTGACTTCATCCTCACCTGCTTGGCTCTCT
CCCGAATCACTCAGCTGCTGGTGTCACTGTGGCAATCATTCGTAATGACACTATCTCCGCCTTTCTATTCCACTTGGAAA
TCAGCAAAACTTATTACTTTGCTTTGGAGAATAACGAATCACTGGACTACCTGGTTTACCACCTGCCTGAGCATTTTCTA
CCTCCTTAAAATAGCTCACTTCTCCCACTCTTTCTTCCTCTGGCTGAAGTGGAGAACGAACAGAGTGGTTCTTGCCATTC
TTGTCCTTTCTTTGCCCTTTCTGCTGTTTGACTTCCTGGTGCTAGAATCATTGAATGATTTCTTCTTAAACGTCTATGTG
ATGGATGAAAGTAATCTGACATTACATATAAATGACTGTAAAAGCCTTTATATTAAAACCCTGATTCTTCTTAGTTTTTC

CTATACCATTCCTATTGTTCTGTCCCTGACCTCACTGGTCCTATTGTTTCTGTCCTTGGTAAGACACATCAGAAATTTGC
AGCTCAACGTCATGGGCTCCAGGGACGCCAGCACACAGGCCCATAAGGGGGCCATTAAAATGGTTATGTCTTTCCTCCTC
CTCTTCACGGTTCATTTTTTTTCCATCCAATTGACAAACTGGATGCTTTTGATATTTTGGAACAACAAGGTCACAAAGTT
TATCATGTTGGCCATATATGTCTTCCCCTCAGGCCACTCGTTAATTTTGATTCTGGGAAACAGCAAACTGAGACAGACAG
CCTTGAAGGTACTGCGGCATCTTAAAAGCACCTTGAAAAGAGAAAAAACAGTTTCGTCTTTACAGATAGACGTTCCAGGG
TCTTTCTAA
>FecaTAS2R67_chrB4:47638968-47638030
ATGCCATCTGGAATCGAAAATACTTTTCTGACAGCAGCAGTAGGAGCATTCATGATTGGAATGTTGGGGAATGGTTTCAT
CGCACTCGTCAACTGCATTGACTGGGTGAAGCATCGAAAGCTCTCGCCAGCTGACTGCATCCTCACCAGCCTGGCTGTCT
CCAGAATCATTCTTCTTTGGATGATACTATTCGATTTGCTTGTAATGGTGTTTTGGCCACATCTATATAACATTGAGAAA
CTAGCTACCGCTGTTAATATCTGTTGGACACTGACCAATCACCTAGCTACCTGGTTTGCCACCTGCCTGAGTGTTTTCTA
TTTCTTTAGGATAGCCAATTTCTCCCACCGCTGTTTCACCTGGCTGAGGCGGAGAATTAGCAGGGTGCTCCCTGTGCTTC
CTCTGGGGTCTTTATTCTTACTGGTTTTCAACTACAAATTATTAGTTGGATTTTCTGATCTCTGGGCTACCATCTACCAC
AACTATGAAAGAAACTCAACTCGGCCCCTAGATGTAAGTAAAACTGGGTATCTTAACAGCTTGGTTATTCTCAGTTTCAT
CTACTTAATCCCCTTCCTTCTGTCCCTGACCTCACTGCTCCTTTTATTTCTCTCCTTGATGAGACATACCAGGAACGTGC
AACTGAACTCTAGCTCGAGGGACTTCAGCACGGAGGCCCATAAAAGGGCCATGAAAATGGTGATATCTTTCCTCCTCCTC
TCCACGGTTCATTTTTTTTCCATCCAGTTAACAGGTTGGATTTTCCTTTTACTGAAGAAACATCATGCCAACTTGATGGT
GACGTTGACATCGGCTCTTTTTCCTTCAGGCCACTCATTTATCCTCATTTTTGGAAACAGCAAGCTGAGACAAACTGCTT
TAGGACTACTGTGGCATCTCAATTGCCACCTGAAAATGGTGAAACCTTTAGCTTCATAG
>FecaTAS2R5P_chrA2:156237297-156238311
ATGAAAACTTACCTTGAGTGTGGCATCACCCTCCGGGGATCTGAAGCTCAGTCAGGAGCCACACACATTCATCTGTTGAA
GCAGGATATTTTCTGTGAAGGAAGTGAGAGACTTCTGACCCCAGCCATACTGTCTGCCGCCCTAGCACTGCTGATGGTGG
TGAGAGTGGCCGAATTTCTCATTGGCCTGGTTGGAAATGAAGTCCTTGTGGTTTGGAGTTTTGGAGAATGGGTCAGAAAA
TTCAACGAGTCCTTATACAACCTCATTGTTCCGGGCCTGGCCGTTTGCCGATTTCTCCTGTGGTGGCTGATTATGATGGA
CTTAACCCTGTTTCCGCTTTTCCAGAGCAGCCATTGGCTTCACTACCTCAGTGTCTTCTGGATCCTGGTAAGCCAGGCCA
GCTTGTGGTTTGCCACTTTCCTCAGTATCTTCTACTGTAGGAAGATCACAGCCCTTGAACAGCCTGTCTGTCTGTGGCTG
AAGCAGAGGGCCTATCACCTGAGTCTCTGGTGCCTTCTGGGGTACCTCATGATAAATTCGTTACTTGTGGCCCACATTGG
CTTAACGTCCAAAATCCTTCCCAAGGCAACAACAGCATTCTGTGCACCCTTTCAAACTGGTACTACATGTACATATTAAA
GCTCACTGCAGGAAGCGGGTTGCCTTTGATGGTGTTTCTTGTTCCTTCTGGGATGCTGATTGATTGTCTCTTTGTGTAGA
CACCACAAGAAGATGAAGGTGCATACAGCTGTAGGAGAGATGCTTGGGCCCAGCCTCACATCACTGCCCTGAAGCCCTTG
ACCTGCTTCCTTATCCTTGGCGTGGTTTATATCCTGGCCAGCCCCTTTCCCATCACCTCCAAGTATTCTGCTAATCTCCC
CGTTGTCTTCATTTCCAAGACCCTCATGGCTGCCTATCCTTCTCTTCATTCTGTCGTATTGATCATGGGGAATCCCAGGA
TGAAGCAGACTTGTTAGAGAATCCTGTGGAAGATAGTGTATGCTTGGAGATCCTGA
>FecaTAS2R8P_chrB4:47496422-47495489
ATGCTCTGTACAGAAGACAACATCTTCATGATCATAATAACTGGAGAATTCATAATAGGAATTCTGAGGAATGTATACAC
TGGACTGGTAAACTGGATTGACTGGATAAGAAGAAAAAGATCTCCTCAACTGACTATATCCTCCCCAGTCTAGCCATCTC
CAGAATGTGTTTGCTCTGTATAATGATAGTAAATATCATCATAATGATATCTCTACCCAGATTTTTATGAAAATGCTAAA
CTAAAAAGCCATCATGCATATCGTCTGGACAGTTGCCAACTACTTAAGTATATGGTTTGCCACCTGACTCAATGTTTTCT
ATTTCCTCAAGATAGCCAACTTCTCCCACCCACTTTTTTCTCTGGCTGAAGAGGGAGAATTGACAGAGTGATTCACTGGA
TTCTGCTGGGCTGTTTGGCCATCTCCTCCTTGGTCAGCCTTCTACTGGCAATGACACCACATTATGATTAGGAGTTTCAT
AAAATTGCAGAACATAAAAGCAACTGCCCTGAAATGTTCCATGTGAGTAAGAGTCAATTCTTCAACCCGCTGACCCTCTT
TAACCTGTTGGCAATTGTCCCATGGACTGTGTCATTGATCTCACTTTTTCTTTTAATTGTGTCCCTACAGAGACATGTCA
AGCAAATGAAACCCAGTGTTACAGGCTGTGGAGGTCCCAGCACAGAGGCCCAGGCGGGAGCCATGAAAACTATGACTTCA
TTTCTCTTCCTCCTTTTTGTGTGTTATGAGGTTTCCCTTTTGGTGACTTTTAGCCACCTTATGAAAGAACGCAAGTTCGC

TGTGATGTTTGGAGAAGCTATACCAATTCTCTATCCTTCTGGTCATTCACTCATTTTAATTATTGGGAATAACAAGCTGA
GGCAGGGCATCTATCAGGATGCCAAGGTATGGCAAAACGGTCTGCATGATGTAA
>FecaT2R16P_chrA2:140495344-140494451
ATGCCCCTTGGCTCACTATCTTCTTCATAACCATCTATGTGCTCAAATCCTTGAGAGTAATTATGCAGAGGAGCTTAATG
TTTGCAGTGCTGGGCAGAGTGGGTGCAGGCCAAAAGGCTGTCATCAGTGGACTTGAGTCTCATCTGCCTGGGTATTTGCT
GCTTCTGTCTACAATGGGCATCTGTCCTATACAATTTTTGCTCCTATTTTAACCCTAACTATGTATTTTGGTACTTATCA
ATCACCTGAGAATTTACTAATACTCTTACTTTCTGGTTAACCAGCTTGCTTGCTGTCTTCTAACTGTGTCAAAGTCTCTT
CCTTTACCAGTCCACCTCTTCTAGCTGAGGTGGAGAATGTTGAGGTTTGTTCCTTGGCTGTTGCTGGGTTCTTTGTTGGT
TTTTTGTGTGTCAATCATCTTTTCAACTATTAGGAATTGTGTCAATGTTCGCTTAATCACCGTGGGGTGTTTCTCTACAA
ACAGCACTATGGTTAAGGGACTTAAGACATTCCATTTGTTTACCATATCTCATCTAATGGTTGCATTGGTTATTCCTTTC
CTCCTGTTCCTGGCCTCCACAATCCTGCTCATGGCCTCACTGTTCCAACACATGGAACAATGCAACACCATAGCACTGGT
CACTGCAGCTCCAGCATGAAAGCTCACACCACTGCCCTCAGGTCTCTCACCATTTTCCTCATCCTCTTCACTTCTTACTT
GCTGACCCTACTCATCTCTATTATGAGTATCTCATTGGATAAAAGGTCTTGGTTCGGGGCTTGGGGAGCTGTCATCTGTG
CTATAGTCTCTATTCACGCCACTTAACTAATGCCGAGCATCCCTAAATTGAAAAAGTTTTCAAGATAAGGTGCTGGAGCC
TAGAGACTGACTGA
>FecaT2R39P_chrA2:157194231-157195356
ATGAATAGTGGGAGCTACTGAGGATCTGCCTATCAAACGCTAGAGAGGCATTTTCCTCCAGACACCAAACAGGGGTGACA
ACTCAGGATGACAAAAACCTGCGATCCTGCAGATAATGAATTTTCACCATTTCACATCCTCTCAATTTTAACAATTATAG
GCACTGAATGCATCATTGGTATCATTGCAAATGGGTTCATCATGGCTATAAATATAGCTGAATGTATTAAAAAATAAGGCA
GTTTCCAATAAGCAGGATCCTGTTTTTCTTGAGTGTATCCAGAATACCTCTCCAAAGCTTCATGATGATAGAAATTACCT
TCAGCTCAACATCCCCACATTTTTATAATGAAGATGTTATATATGGTACAAAGTAACTTTCATGTTCTTAAATCATTGTA
GCCTCTGGCTTGCTGCCTGGCTCAGCTTCTACTTCGCGAAGATTGCTGATTTCTCCCACCCCTTTTCCTCAAGCTGAAGT
GGAGAATTTCCGGGACAGATGTCCTGGCTCTGTGGCTATCAATGTTTATTTCCTTAGGCTACAGTGTACTCTTCTCCAAT
GACATCAACACCATGTATTGTAACAATTCTTCTATCCCCTCTCCCAACTCCACTAAGAAAAAATACTTCACTGAGACCAA
TGTGGTCAACCTGGTTCTTCTCTATAACCTGGGGATCTTCATTCCTGTGATCATGTTCATCTTTGCAGCCACCCTGCTGA
TCATCTCTCTCAAAAGACACACCCTACACATGGAAAGCAATGCCACTGGCTCCAGGGACCCCAGCATGGAGACTCACATG
GGGACCATCAAAGTTACCAGCTACTTTCTCATTCTCTACATTTCCAATGCAGTTGCTCTGTTTCTTTATATGTCCAATAT
CTGTGATGCCAACAGTTCCTGGATTATTTTGTGCAAATTCATCATGGCTGCCTACCCTGCTGGTCACTCCATTCTGCTGA
TTCAGGACAACCCTGGGTTGAGAAGAGCTTGGAAGCAGCTTCAGCCTCAAGTTCATCTTCACCTAAAAGAGAAGCACAAT
ACCACAAAACAAGCCCAACCAGCTCTGCCTCCCCCTCACCTAGACCCCTCTCCTCCCACCTCCTTTCTTCCCCAAACCTG
ATCTGA
>FecaTAS2R40P_chrA2:157218362-157219338
ATGGCGACAGTGAACACAGATGCCATGGATAGAGACACGTCCAGGTTTAAAATCGTCCTCACCGTGGTGGTCTCTGGAGT
AGAGTGCATGACTGGCATGATTGGGAATGGCTTCATCACTGCCATCCAGGGGGCCGAGTGGGCCAGAGGCAAAAGGCTCC
CTGTGAGTAACTGTATCCTGTTGAAGCTGAGCTTCTCCAGGCTTTTGCTGCAGATCTGGATGATGCTGTAGAATATTTAC
AGTCTATTCTTCCTGGTCACTTATAACCAAAACACAGTGTTTATAATCTTCAAAGTCATCACCATGTTTCTGAACTATTC
CAACCTCTGGCTTGCGGCCTGGCTCAACATCGTCTCTTGCCTTCCAATTGCAAACTTTGCTCACTGTTTGTTCTCCATGA
TGAAGAGGAAAATCAGAGAGCTGATGCCTCGGCTTCTGGGGCTGTCACTGTTCATCTCCTTATGCTTCAGCTTTCGCTTC
TCTAAAGATATCTTCAATGTGTACGTAAATAGTTCCGTTCCTATCCCCTCCTCCAACACCACTGAGAAGTACTTCTCTGA
GACTAACATGGTCAACCTGGTTCTTCTCTATAACCTGGGGATCTTCATTCCTCTGATCATGTTCATCTTTGCAGCCATTC
TGCTGATCATCTCTCTCAAGAGATACACCCTACACAAGAGATACACCCTACACATGCCACTGGCTTCAGGGACCCCAGCA
TGGAGGCTCACATGGGGGCGATCAAAGCTATCAGCTACTTTCTCATTTTCTATATTTTCAATGCAGTTGCTCTATTCATT
TCCATGTCCAACATCTTTGACATCAACAGTTCCTGGAATATTTTGTGCAAAATCGTCATGGCTGCTTACCCAGCTGGCCA
CTCAGTGCTACTGATCTTGGGCAACCCTGGGCTGAGAAGAGCCTGGAAGAGGTTTTAGCACCGTGTTCATCTTCATCTAT

AAGAGCAGACCCCGTGA
>FecaTAS2R41P_chrA2:157423984-157424995
ATGTCCCCAGGAGAGGTCTAAAGAGGGACTGTGTGCGGGGGTAGGGAGGAGGCCAGGGACCAAGGCGCAAGGCACAGGGG
TGGTGCCCCGAGCGGCCGAAACGCGGCCAGGGCTCTCAGCCTTCCTCATGCTGCTCTTCGTCCTGCCGTGCCTTCTGGGA
ATCCTGGCCAACGGCTCGTTGTGCTGGTGCTGGGCCGGGAAGGGCTGCGGCGGGGGAGGCTGCCTCCCTCTGGCATGATC
CTCCTGAGCTTGGGCGCCTCCCGCTTCTGCCTGCGGTGGGTTGGAACGGTGAACAGCTTCTACTGGCCGAGTACAGCAGA
GGTCCTGCACGGCAGTTCTTTGGTCTCCATGGGGACTTCCTGAACTCAGCCACCTTCTGGTTCAGCTCCTGGCACAGTGT
CCTCTTCTGCATGAAGATCACTAGCTTCACCCACCCCACCTTCCTCTGGCTCAAGTGGAGGTTCCCAGGGTCAGTGCCCT
GGCTCCTCATGGCTCCTCTCCTGATCTCCTTCAGCGTCACTCTGCTCTTCTTTTGGGGAAACCGTGCTGTGTGTGAAGGA
TTCTTAATTAGAAAGTTTCCTGGGAACATGACCTTCGGACCGTGGAGCAGGACACTGGAAGTTCACTACTTCTTGCCCCT
GAAGCTGATCACCCGGTTAGTTCCTTGCTCTGTTTTTCTGGTCTCAGTTGCACTGTTGATTAATTCTCTGAGGAGGCACA
CACCGGGAGGATGCGGCTCAGCGCCCACAGGCCACAGGACCCCAGTGCCCAGGCTCACAGCAGAGCTCTGAAGTCGCTCG
TCTCCTTCCTCATTCCTGATGCTCTGTCCCTCGCATCCCTGGTCATCGATGCTGCAGGTTTCTTCTCAGAGAGTGACTGG
TACTGGCCGTGGCAGATTTTAATCTACCTGTGCGTGTCTGTCCATCCGTTTTTCCTCATCTCCAGCAACCTCAGGCTTCG
AGGGGTGTGCAGACAGCTACTGCTGCTGGCCAGGGGCTTCTGGGTGGCCTAG
>FecaTAS2R60P_chrA2:157399351-157400276
ATGAATGGAGATGACATGATTCCAGGATCTCCAGTGACTGATAAGAGAGCCATCATCTTGGCTATCATTTTATTATTTTT
GTGCCTGGTGGCAGCAGTGAGCAATGGCTTCATCACTGCAGCTCTGGACGTGGAGTGGTTGCTACAGAGAACACTGTTCC
CTTGTGATAAATTATTAGTCAGCCTGGGGGCCTCTTGCCTCTGTCTGCAGTGGGTGTGATGAGGAAGACCATTTATATTT
TCCTGTATCCAGAGGCCTTCCCATCCAACCTTGTACTGCAGTTCCTAGCCTTCTGGCAGGACTGGCCATCTTATGGTTCT
CCACCTACCTAGCTCAGTGTCTTCCATTGCATGAAAATTGCAACGTTCACCCTCCCCATCTTCCTCTGGCTAAAATAGAA
GGTGTCGGGGTTGGTTGCATGGATGCTGTAGGGCTCTCCAACTTGAGCACCATCTTATTTTCATAGGCAACCAGAGCTTG
TAACAGTACTTTGTAAGGAGAAGGTTGCAATCTGGGAATGCCATTGAGAATACCGTAAGACCATATGAGAAATTCTACTT
CTTCTGTTTAAAACTTGTTAGCTGGACAGTCGTTACCGTCGTCTTCCTTTTTGGCATGGTTTGGTCATGTCTCTGGAAAG
ACACACTAAGAAGGTCTGCCTGTCCATTGTGGCTTTTGTGAGCCCAGTGCCCAGGCACACAACCAGGTTCTCCTGGCTCT
CATGTCCTTATCCGTCCTCTCCACCTCCTGTTTTCTATCACCGGTGCTCAATGCTGCAGGTGTTTTTCCATTTTGGGACC
CTAGCTACTGGGTGTGGCAGGCAGTGATTTATCTGTGCTCAGCAGTCCCCATCACTGCACTTGAGCAACCCCTGACCGAG
AGGTGTGGTGGAGAGGGGCTGCTGTGCAGGGTGCTGGGCATCGTGA
>FecaTAS2R62P_chrA2:157393422-157394347
ATGCCCTCACCTGCATTGATCTTCATGGTCATCTTCTTCCTGGAGTCACTGGCTGCAATGTTGCGGAATGGCTTCGTGGT
TACTGTGCTGGGCAGGGAGTGGGTGCGATGCCGGATGCTGCCCGCGAGTGGCATGATTGTGGCCTCCTTGGCCGCCTCCC
GGTTCTGCCTGCATGGGTGGCCATCCTGAACAACCTCTTCACCTTCTTTGATTTTCGTTCCATAATGGACTATTTCAGCA
TCCTCTGGAGCTTCTTCAAGACTCTGTCTTCCTGGCTCACCGCCTGGCTTGCTGTCTTCTACTGTGTGAAGACCGCCATC
TTCTCCCACCCTGTCTTCTCCTGGCTAAGGTGGAGGATTTCTCAGTCAGTGCCCAGGCTGCTGCTGGGCTCCCTGCTCAT
GGCTGGTCTGGTGGTCGTCTCATCAACCATTGGGACTATATTTCCTGTGCAGGTGACTGCCTCCCAGAATTCCCAAGGAA
ACAGGACCCTGGCTGATAGACTACAGAGCTATTGGCACTTTTTTCATCCTCATGCAATGCTTATGTGGTTGATCCCGTTC
CTTCTGTTCTTGGTGTCCATGCTTTTGCtCATGTGCTCCCTGCACCGGCACTTGGGGCAGATGAGGGATCATAGACTGGG
TCCATGTGATCCCAGCACCCAGGCTCACATCATGGCCCTGAAGTCACTTGCCTTCTTCCTTGTCTTCTATACATCATATT
TTCTGTCCCTGATTGTCATTACTATGAAAATAGCAACCTTCCAGGGTCACTGGCGCTGGGCCTGGGAAGTGGTGACCTAC
ACAGGTATCTGTCTGCACTCCAGCATCCTGATGCTAAGCAGCCCCAAGCTGAGAAAGGCCCTGAGGACCAGGCTTTGGAG
AGCTCTGGAAAAAGGCGGGTTTATCTCAAGTTATCAGTATCAATAA
>BotaTAS2R1_chr20:63676557-63677447
ATGCTGGAGTCTCACCTTGTTAGCCACCTTGTTTTGGCAGTGGTACACCTTCTCTTGGGGATTTTAGTAAATGGCATCAT
TGTGATTGTGAACGGTACTGACTTCATCAAGCAGAGAAAGTTGATCCCGCTGGATCTCCTTGTTTCCTGCTTGGCGATTT

CCAGGATGGGAATTCAGCTGGCCTTCTTCTACACTAACCTGGCTCTTCTTTCCTTGATCAAATTCCCTCAATTTACTGAG
ACGCTTGTAGTTTTCACATTTGTAAATGATTTGGGACTTTGGTTTGCCACCTGGCTCAGTGTCTACTACTGCACCAAGAT
TGCTACCATCGCTCACCCGCTCTCGTTCTGGTTGAAGATGAAGATCTCCAAGTTGGTTCCTTGGCTGATTCTTGTGTCCC
TGCTGTATGCATGTAGTACTTCTGCTATGCATGTCAAATATAAGTGGGTATTTTATGGAGAAGACTTCCTGGGCCTTTTC
TTCCCAAATGTAACAACTCACATCAAAGTAACCCCTACCTTACAGTTTGCCTTTCTGTTTGCTGAGTTTGCATTGCCATT
GTTCATCTTCCTGATTTCTTCTCTGCCCTTGATATTTTCCTTGGGAAGACATGCCTGGCAGGTGAGAAACACATGGACAG
GCCCCAGAAACCCTCACACACGTGCGTACATCAGGGCCTTTCTCTCCATCCTGTCCTTCTTGGCCCTCTATCTCTGCCAC
TACCTGATCATTGCTTTGATCTTTTTCAAATTTTAACCTCAGAAGCTTTCTATTTCTGTTCTGCACCTTCGTGGTTGG
TTCATACCACTCCGTCCACTCTATTACTTTAATTTTAGGAAACCCGAAAATGAAACAAAATGCAAAGGCATTGCTCCTCC
TCAGAAAGTGA
>BotaTAS2R3_chr4:105874500-105875450
ATGTTGAGACTCAGCAATATGGGGTTTCTGGTTCTGACCGCCATTCAGTTCATCCTGGGAATGCTGGGGAATGGTTTCAT
AGGGTGGGTCAATGGCAGCAGCTGGTTCAAGAGCAAGAGGATCTCTTTGCATGACTTCGTTATCACTAACCTGGCTGTCT
CCAGGATTGTTTTGCTGTGGATTCTCTTGATCGATGGTATTTTACTGGTGTTCTTTCCCAAACTACATGATGAAGGGATA
ATCATGCAAATTATTGATGTTTTCTGGACATTTACAAACCATCTGAACATTTGGCTTACCACCTGTCTCAGTGTCTTCTA
CTGCCTGAAAGTGGCCAGTTTCTCCCATCCTATGTTCCTCTGGCTCAAATGGAGAGTTTCCAGGGTGGTTGTATGGATGC
TGTTGAGTACCCTGCTGTTATCATGTTGCAGTGCCATCTCTCTGATCCGGGAATTTAAGATCTATTCTGTTCTTGGTGGA
ATTGATAGAACCGGGAATATGACTGAACTTTTTAGAAAGAAAGAAAAAGAATATAAACTGATCCATGTTCTTGGGACTCT
GTGGGACCTCCCTCCCCTAGTCATATCGCTAATCTCCTACTTTCTGCTTATCCTCTCCCTGGGGAGGCATATGCGGCAGA
TGCATCAAGACTGTGCCAGCTCCAGAGATCTCAGTACCGAGGCCCACAGGAGGGCCATCAGAGTCATCCTCTCCTTCCTC
TTTCTCTTCCTACTCTACTATCTTTCCTTTTATGTTTTAACATCCAGTTATTTCTTACCAGCAACTAAGATGATTGCAAA
GATTGGAGAAGTAATTGCAATGTTATATCTTGCTGGCCACTCCTATGTTCTCATTCTGGGAAATAGTAAGCTGAAGCAGA
TGTTTGTGGCGATGCTACGGTGTGAGCCTGGTTGTCTGAAGCCTGGATCCAAGGGATCTGTTTATCCATAG
>BotaTAS2R4_chr4:105887871-105888761
ATGCTTCGGATAGTCTTTTTTTCTTCTGTCGTTGTCTCTGAAATTTTAACTTTTGTAGGACTCATTGTGAATCTCTTCAT
TGTAGTGGTCAGTTACAAGACTTGCATCAAAAGCCACAGGATCTCTTCTTCTGACAGACTCCTGTTCAGTTTGGGCATCA
CCAGATTTTTTATACTGTTACTGAATGTTGTTGTCATCATCTCTCCAAATATGGAAAGGTCAGTCTCCTTATCCTATTTT
TTTCTGTCATGTTGGATGTTTTTGGACTGTAGTAGTCTTTGGTTTGTAACCTTGCTCAACGTCTTGTATTGTGTGAAGAT
TGCTAACTACCAACACTCAGTGTTTCTCCTGCTGAAACGAAATCTCACCACCAAGATGCCCCGGCTGCTGCTGGTCTGTA
TGCTGCTTTCTGTCTTCACCACTCTCCTGTATATTATGCTCAGACAGTTGGCACCCTCTCTTGAATTTGTGACTATGAGA
AATGGCACAGTATTTGACATCAATGAGGGACTCTTGTCTTTGGTGACTCCTTTGGTCTTGAGCTCATTTCTCCAATTCAT
CATTAATGTGACTTCTGCTTCTTTGTTAATCAATTCCTTGAAGAGACATATACAGAAGATGCAGAGAAGTGCCACTGTTC
TTTGGAATCCCCAGACTGAAGCTCATGTGGGTGCTATGAAGCTGATGATCTGTTTCCTCGTACTCTACATTCCATATTCA
GTTGCTACCCTGGTCCATTATCTCCCTCCTTCTATAGGGATGGATTTGAGAACCAAGTCTATTTATGTTATTATGTCCAC
CATTTACCCTCCAGGACATTCTCTTCTTATTATTCTCACACATCCTAAACTGAAAACAAAAGCAAAGAATATTCTTTGTT
TCAGTAAATAG
>BotaTAS2R10A_chr5:99230573-99231472
ATGCTGAGTATAGTAGAAGGCCTCCTCCTTTTTGTAGCAGTTAATGAGTCAGTATTGGGGGTTTTAGGGAATGGGTTTAT
TGGACTAGTAAACTGCATTAACTGTGTGAAAAATAAGAAGATCTCTACACTCAGCCTTATTCTCACTGGCTTAGCCTCTT
CCAGATTTTGTCTGATATGGATAATAACTACAGATGCATATGTGAGGGTGTTTTCTCCAGATATGTATTTGTCTGGTAAT
CTAAGTCAATATATAGCTTACTTATGGATAATTATGAATCAATCAAGTGTCTGGTTTACCACTAGCCTCAGCATCTTCTA
CTTCCTGAAAATAGCCAACTTTTCCCACTGCATTTTTCTCTGGCTGAAGGGTCACATTACTGAGATTCTTCTTCTTCTAA
TGGGATGTTTGCCCATTTCATGGTTATTTACTTTTCCAAACATTACAATGCCTTTTATTAATAATATTATGAAGAACAGA
AGCACAACCGGGTTGGTCACCATGCAGAAAAGTGAATACTTTATAAATCAGATTTTGTTCAATCTTGGAACATTTCTTGT

CTTTGTACTATGCCTGATTACATGTTTCTTAATAATCACTTCCCTTTGGAGGCACAACAGGAGGATGCAATTGAATGCCA
CAGGATTCAGAGACCCCAGTACAGAAGCACACATCAAAGCAATGAAGATTTTGGTGTCTTTTATCATCCTCTTTATCCTG
TATTTTGTAGGCACTGCCATACAAATATTAAGTGTGACAGTGCCTGAAAACAAACTGCTATTTATTTTTGGTATGACAAC
CACCATCCTCTATCCCTGTGGACACTCATTTATCCTAATTCTTGGAAACAGCAAGCTTAACCAAGCCTCTTTGAGGGTAC
TGAAGCTATTAAAGTGCTAG

>BotaTAS2R10B_chr5:99192315-99193247

ATGCTGAATATAGTGGAAGGCCTCCTCATTTATGTAGCAGTCAGTGAATCAGTATTGGGGGTCTTAGGGAATGGATTTAT
TGGAGTTGTAAGCTGCATTGACTGTGTGAAAAGCAAGAACATCTCTACTGTCAGCCTTATTCTCACTGGCTTAGCCTCTT
CCAGATTTTGCCTGATATGGATGATAATTACAGATGCATATATAAGGATGTTTTTTCCAGATATATATTTGTTTGGTAAT
ATAAGTCAATATATAGTTTACTTAAGGATAATTATGAATCAATCAAGTACCTGGTTTGCCACCAGCCTCAGCATCTTCTA
TTTCCTGAAGATAGCCAATTATTCCCACTGCATTTTTCTCTGGCTGAAGTGTCACATCAACAGGGTTCTTCTCCTTTTCA
TGGGGTCCTTGCTTATTTCATGGTTATTTGCTTTTCCAAGCATTGCAAAGCCTAGTACCAATAATATTATGAAGAACAGA
AGCACAACCTGGCTGATCACCATGCATAAAAGTGAATACTTGACAAATCAGATTCTGCTCAATATTGGAGTCATTCTTGT
CTTTGTACTATGCCTGATTACATGTTTCTTATTAATCACTTCCCTTTGGAGACACAACAGAAAGACGCGATTGAGTGCCA
CAGGATTCAGAGATCCCAGCACTGAAGCACATATCAAAGCAATGAAGATTTTGGTGTCTTTTATCATCCTCTTTATCTTG
TATTTTGTAGGCACTGCCATACAAATATCAGGTAGTAGTACTATGCCTGAAAACAAACTGTTGTTCATTATTGGTATAAC
AACCAGACTCCTCTATCCCTGGGGACACTCATTGATTCTAATGCTAGGAAACAGGAAGCTGAAGCAAGACTCTTTGAGGG
TACTGAAGCCATTAAAGTGCTGGGAAAAAGAGAAACTTCTTAGAATTCCATGA

>BotaTAS2R10C_chr5:99242115-99243044

ATGCTGAGTGTACTGGAAGGCCTCCTCATTTTTGTAGCACTTAGTGAGTCAATATTGGGGGTTTTAGGGGATGGATTTAT
TGGACTTGCATACTTCATTGAATGTGTGAAGAACAAGAAGTTTTCTACTATCAGCTTTATTCTCATGGGATTGGCTACTT
CCAGAATTTGCCTGATAGGGTTAATAACTACCGATGGATTTGTGAAGATTTTTTCTCCAGAAATGTATTCCTCTGGTTAC
CTAATTGACTGTATTACTTACTCATGGGTAATTCTGAATCCAACAAGTGTCTTTTTTGCCACCAGCCTCAGCATCTTCTA
TTTCCTGAAGATAGCCAATTTTTCCCACCACATTTTTCTCTGGTTGAGGAGTGACGTCAAAAGGGTTCTTCTCCTTCTGA
TAGGATACTTGCTTATTTCATGGTTAGTTACTTTTCCACTAACTATGAAGATAATTAGTGATTCTAGAGCAAAGAATAGA
AGTGTAGTCTTTTCAGTTGAAGTGCATAAAGGTGAATTCTTTAGAAACCAGATTTTGCTCAATCTTGGAACCCTTACCAT
CTTCATACTATGCCTGATTACATGTATCTTATTGCTCATTTCCCTTCGGAGGCACAACCAGAGGATGCTACTGAATGCCA
CAGGATTCAGAGACCCCAGCACAGAAGCACATATCAAAGCAATGAAAGTTTTGATATCTTTTATCATCCTTTTTATTTTG
TATTTTATAGGCATTACCATAGAAATATCATGCACTACTATGTCAGAAAGCAAGCTGTTGTTTATTTTTGGTCTGACCAT
CACTGCCCTCTATCCCTGGGGACACTCATTTATCCTAATTCTAGGAAACAACAAGCTAAAGCAAGTTTTTTTGAGAGTAC
TGAAGCAATTAAAATGCTGGAAGAAAGAGAAGCTCCTCAGAACTCCTTGA

>BotaAST2R12_chr5:99140568-99141497

ATGGAGAGAACATTGAACAATATACTTACGATCATTTATGCTGGAGAGTTCTTACTGGGTATTTTGGGAAATGGATTCAT
TGTTCTGGTTAACTGTATTGATTGGATCAGGAGTAGGAAGTTCTCCCTGATTGACTTTATTCTCACCTGCTTGGCTATTT
CCAGAATATGTGTGCTGTGCATAATGATTTCAAGTACAGGTTTATATGTAATCTCTAAGGAAATACGGTACAATAAGAAT
CTCCTGATAAATTTGAGGTTCCTCTGGACAGGATCCAATTATTTCTCCATAGTCTGCACCACCTGCATCAGTGTCTTCTA
TCTCCTCAGAATAGCCAACTTTTCGAATTTCCTTTTCCTCTGGATGAAATGGAGAATTCACAAGGTGCTTCTCATTATTG
CACTGGGGGCTGTCTTCTCTTTCTGCTTGTGCCTTCTTCAAAAGGATGCGGTAGTTGAAAGCCGGCTCCAAAACCAGGTA
AACAGCGAAAACAATGTGACATTGGACTTTCTAATGATAAAATATGATTTGTTCCTTACCATAATGTTCCTCATCCCCTT
TGTAGTGTCCCTGGCCTCCTTTCTCCTTTTAATCCTCTCCTTATGTGGTCATCTCAGGCGTATGAACGGTGTAGACTGTA
GCTCGGAGGCCCATGTGAGAGCCCTGAAGGCTATGATTTCATTCCTACTCCTCTTCGTTCTATACTATTTGAGCACTATT
ATAACTGTGTGGGCCAATCACATTCTAGGTAGTTTCGTGGCAAAGATTTTTGTGAACATGCTGTTATTTTTCTGTCCTTC
TGGCCACACTTTGCTTCTGATTTTGTGGAACAGCAAATTGAAACAGGCTTCACTCTGTGTCCTAAGGAAGCTGAAGGGTT
ACATGAATCTAAGAAAACCTGCTCTTCCAAAAAGAAGCCTGAAGCGATGA

>BotaTAS2R13_chr5:99121140-99122051
ATGGAAGATTCCTTGGAAAACATCTTTATCACTTTAATAAATTCAGAATTCATAATTGGCATTCTGGGGAATGGGTTCAT
AACACTGGTGAACTGCACTGACGAGATCAAGATGCAAAAGGTCTCCTTGGCTGATCAAATCCTCACTGCTTTGGCAATTT
CCAGAATTGGTCTGATTTTGGTAATGATAGTGAGTTTGTTTACAAAGGAGTCTTATCCATCTTCATCTTTAGACATAAAG
GGAAATAAAGTCATACTTTTTAGTATTGCTGGGCTCTTGGCCAATCATTTTAGTGTCTGGCTTGTCACAGGCCTCAGCCT
CTTCTATTTCCTCAAGATAGTCAATTTTTCAAATGCTGTTTTTCTTCACCTAAAGTTTAGAATTGGAATGGTAGTTATGG
TAATGTTTCTGGGGACATTAGTATTGCTGCCTTTAAGTCTTACTCTGGTGAGTAGCTATATTAATATCAAGATACATCCG
TATGAAAGAAATATGACTTTAAATTCTAAAAGGCATGACACTGAAACCTTTTCCAAATTAATTATATTCACCGTAGGATC
TTTCTTACCTTTTATTATATCCCTGAGTTGTTTTCTCCTATTAATGTTCTCCCTACTGAAACATGTCAAGAAGATGAGGA
GCCATGCAACAGGATTCAGAGATCCCAGCAGCAAAGCCTACGTCAGAGCCATGATCATGGTGATATCTTTTCTCATACTA
CTTGCCATTCACTTCCTATCTCATCTCATGACAACTTTTCATCACAATGTGATACAGAGTGAACTGGCCTTTATGCTTAG
AGAAGCTCTTGGAACTATTTACCCTTCAGTTCACTCATTTGTCCTGATTCTGGGAAATGACAAGCTAAGAAAAGGTTCAC
TTTTGGTGCTGTGGCAGTTGAGGTGTGGCTGA
>BotaTAS2R16_chr4:88194752-88193847
ATGACAACCAGCCAACTCTCTGTCTTCTTCATGATTATCTATATGCTCGAGTTCTTGATAATAACTGGGCAGAGCAGCCT
GATTGTTGTAGCGCTGGGCAGAGACTGGGTGCAGACTCAAAGGCTGCCACCTGCGGACATGATTCTCATCAGCCTGGGCA
TCTTTTGCTTCTGTCAACTGTGGTCATCGATGCTGTACAACTTTTGTTCCCACTTCCACCCTAATTACAATTTTTGGTAT
TTCGGGATCATCTGGGAATTTACTAACATCCTTTCCTTCTGGTTGACCAGCTTGCTTGCTGTCTTCTACTGTGTCAAAGT
CTCTTTCTTCAGCCACCCCGTCTTCCTCTGGCTGAAGTGGAGAATTGTGAGATGGGTTCCTCGGCTGTTGCTGGGCTCTC
TGCTGATTTCTTGTGTGTCTACCATATTTCCAGCTACTAGTTATTACATTGATATTCAATTCATCGCCATGAAGCATTTC
CCTAGAAACAGCACCATGCTTGAGAGACTTGAGGCGTTCCTGTGGGATTTTTCCACACTGCACAAAGTAGTTGTGTTGGT
TATTCCTTTCCTCCTGTTCCTGGCCTCCACAGTCTTGCTCATGGCCTTATTATCCCGACATCTGAAGCAGATGAAAGACC
TTCACACAGGCTGCTCCAACTCCAGCCCGGAAGCTCACTCTGCCGCCCTGAGGTCCCTTGCCATCGTCCTCATCTTGTTC
ACCTTTTATTTTCTCACCGTGCTCCTCTCCATATTGGATGTCCTATTTAATAAAGAGTCCTGGTTCTGGGCCTGGGAAGC
TATCATCTATGCATTAGTCTCTATTCATTCTACTTTACTAATGCTGAGCAGTGTCAAACTGAAAAGAGTTTTAAAGGCAA
GGTGCTGGAGCCTAGAAGCTGCCTGA
>BotaTAS2R408B_chr5:99072339-99073241
ATGATAACTTTAGTATCGAGCATTATTTCCATTCTAATGGTGGCAGAATTTGTTCTGGGAAATTTTGTGAATGGTTTCAT
AGCACTGGTGAACTGCAATGACTGGCTCAGGAAACAAAAGGTCTCCTTAGCTGATGGGATTCTCACTGCTCTGGCAGTCT
GCAGAATTGTTTTGCTCTGGACAATATTAATAAATTGGTATGCAACTATGTATAATCCAGCTCTATATAGTTTAAGAATT
GTTATCCGTGTTGCCTGGACAGTAAGCAACCATTTTAGTAACTGGCTTGCTACTAGCCTCAGTATATTTTATTTGTTCAA
GATAGCTAATTTCTCCAGCCTAATTTTTCTTCACCTGAAGTGGAGAGTTAAAAGTGTAGTTCTCATGATGATTTTGGGGA
CTTCAGTGATTTTGTTTTTTCAAGTTGCAGTGTTAAGTATAGATGAGACTATTCAGACAAGTGAATATGAAAGAAACATC
ACTGAGAAGACCAAATTAAGGGACATTTTACACCTTTCAAATATGACCCTGCTCACACTAACAAACTTCATACCCTTCAC
TATGTCTCTGGTATCTTTTCTGCTGCTAATCTTTTTCCTGTGGAAACATCTCAGGAAGATGCAGCTCAACGGCAAAAGAT
CCCAAGATCCCAGCACCAAGGTCCACATAAAAGCCATGCAAACTGTCATCTCCTTTCTTTTCCTGTTTGCCACTTACATG
CTGACTGTAATTTTAACAATATGGAATTCTAATGAGCTGCAGAAGGAACTGGTCCAAATGCTTTTCCAGGCTCTTGCAAT
CACATATCCTTCAATACACTCATTTATCCTGATTTGGACAAACAGGAAATTAACACAGACCTTTCTGTCATTTCTGTGGC
AGCCAAGATGCTGGCTAAAAGTAAAAGGAACTAGGTAG
>BotaTAS2R38_chr4:106104045-106103038
ATGGTGACTCTGACTCACATCGTATCTGTGCCCTCTGAAGTCAGGAATGCATTTCTGTTCTTTTCAGTCCTGGAGTTTGC
AGTAGGGATCCTACTCAACGCCTTCATTTTCTTGGTCAATTTCCGGGACCTGGTGAGGAGGCAGCCACTGAGCCACTGTG
ATCTTGTCCTATTGAGTCTCAGCCTCACCCGGCTTGTCCTACACGGGCTGCTCTTTCTGAAGGCCATCCAGCTTACTCAT
TTCCAGCGGATGAAAGACCCGCTGAGCTTCAGCTACCAGACCATCATCGTACTCTGGATGATCGTCCACCAAGCCGGACT

CTGGCTCACCATGTGCCTTAGTCTCCTCTACTGCTCCAAGATTGTCCGTTTCTCTCACGCCTTCCTGCTCCATGCAGCAA
GCTGGATCTCCAGAAAGATCCCCCAGATGCTTCTGGGTGCTATGGTTCTCTCCTGTGTCTGCACTCTTCTCTGCTTATGG
GACTTTTTTAGTGGATCTCATTTATCAGCTGTAACTAGGCTACTCATGAATAACAGTACTGAACTCAATTTGAACATTGC
AAAACTCAGTTTCTTTCATTCCTTCCTCTTCTGCAGCCTGGCATCCATCCCTTCTTTCTTGCTTTTCCTGGTTTCCTCTG
GGATGCTGGTGTTCTCCCTGGGGAGGCATATGAGGATGATGAGGGCCAAAACCAGAGGCTCTGGGGACCCCAGCCTGGAG
GCTCACACACGGGCGCTCAGGTCTCTTGTCTCTTTCTTCTGCCTGTATGTGCTGTCACTCTGTGCTGCCTTATTCTCGAT
ACCGTTGCTGATGCTGTGGCACAGCAAGGTCGGGGTGATGGTCTGCATAGGGATAATGGCAGCCTGTCCCTCAGGACATG
CAGTCATTCTGATCTCAGGGAATGCCAAGCTGAGGAGGGCTGTGGACACCATTCTGCTTTGGGCAAAGAGCAGCTTCAAG
GTAAGGGTGGACCACAAGGCATATCCCAGGACGCCAGATCTGTGTTGA
>BotaTAS2R39_chr4:107396019-107397062
ATGAGTGGGAGCTATCACAGACCAGCACACCAAGTGCTAAGGAGACATTTTCCTCCAGACATTGAAGAAAAGCAACCACT
CAGGATGATCCAAACCTGCAGTTCCTCAGAAAATGATCTGTCACCATCTCTTGTCACTTTGATGTTAATAATTATCGGCA
CGGAATGCATCCTTGGTATCCTCGCAAATGGGTTCATTGCAGCGATAAATACAGCTGAATGGATTCACAGTAAGGTACTC
TCCACCAGTGGCAAGATCCTGCTTTTCCTGGGTGTATCCAGAATAGTTCTACAAAGCTTCATGATGCTAGAACTTACCTT
AAGCTCAACATCCCCACAGTTTTATAATGATGACATCATGTATCACACATTCAGAGGATGTTTCATGTTCTTAAATCACT
GCAGCCTCTGGTTTGCTGCCTGGCTCAGTGTCTTCTACTTCGTGAAGGTGGCGGATTTCTCCTACCCCCTTTTCCTCAAG
CTGAAGTGGAGAATTTCCGGACTGATGCCCTGGCTTCTGCAGCTATCAGTGTTTGTTTCCTTGGGCCAGAGTGTGCTCTT
CTTCCAAAACAACTATACTATGAATAGTAACAATCTTTTTTCTCTCCCGTCCTTCAACTCCACTAAGAAAAAGTCCTTCG
CGGAGTCCGCTGTGATCAACCTGGTTCTTTTCCTTAACCTGGGGATCTTCATCCCTCTGATCATGTTTATGCTGGCGGCC
ACCCTGCTGATCATCTCTCTCAAAAGACACATCTTCCACATGAAAAGCAACGCCACTGGCTCCAGAGACCCCAGCATGGA
GGCTCACCTGGGGGCCATCAGAGCCATCAGCTATTTTCTCATTCTCTATATTTTCAAAGTACTTGCTCTCTTTCTCTACA
TGTCCAACTTCTTTGACATCAATAGTCCCTTGAATATTTTGTGCAAAATCATCATGGCTACCTACCCTGTGGGCCATTCC
ATTCTACTGATTCAGGACAATCCTGGGCTGAAAAGAGCCTGGAAGAGGCTTCAGACTCAAGTTCACCTTTATTTTAAAAA
GTAG
>BotaTAS2R41_chr4:107700102-107700995
ATGCATCCAGCATTCACAGTCCTCTTCATGCTGCTCTTTGTCCTGCTGTGTATCCTGGGCCTCCTGGCCAATGGCTTCAT
TGTGCTGGTGCTGAGCAGAGAATGGGTGCGACGTGGGAGGCTGCTCCCCTCTGACCTGATCCTCTTTAGCTTGGGACTCT
CCCGCTTCTGCCTGCAGTGGGTTGGAATGGGGAATAACTTCTACTATTTCTTGCATCTGGTCGACTACTGCAGTGGTCCC
GCCCGGCAGTTCTTTGGTCTACCCTGGGACTTCCTCAACTCTGTCACCGCCTGGTTTGGCTCCTGGCTCAGCGTCCTCTT
CTGCATGAAGGTTGCTAACTTCACCCACCCTGGCTTCCTCTGGCTAAAGTGGAGGTTCCCCAGGTCAGTGCCCTGGCTTT
TGCTGGGCTCTCTCCTCACCTCCTTCATTGTCACCCTACTGTTTTTTGGGGGGAACCACGCTTTGTATAAAGAGTCCTTC
ACTAGAAAACCTTTCGGGAATATGACCTACTATCAGTGGAACAGGATTCTGGAAATGTACTATTTCCTGCCCCTGAAACT
GATCACTTTTTCAATTCCTGGCTCTGTTTTTCTGGTCTCGATTGCTTTGTTGATTGACTCTCTGAGGAGACACGCATGGA
GGATGCAGCACAGTGCTCACAGCCTGCAGGACCCCAGTGGCCAGGCTCACACCAGAGCTCTGAAGTCACTAGTCTCCTTC
CTTGTTCTTTATACTCTGTCTTTCATGTCCCTGATCATCGATGGTGAAGGGTTCTGCTCCTCAGAGAGTGACTGGTACTG
GCCATGGCAAATTTTACCTACTCGTGCACATCCATCCATCCCTTTATCCTCATCCTTGGCAACCTCAGGCTTCGGGGGGC
ATTTGGGCAGCTGA
>BotaTAS2R42_chr5:99026430-99027359
ATGTTCCCTGGGTTGAGTACCATCTTTCTGATACTATCAGGAGTGGAATTCTTAATCGGAATTCTAGGCAATGTGTTCAT
TGGACTGGTACTCTGCTCTGAATGCGTTAAGAACCAAAAGACATCTTTATTTGACTTCATTCTCACTGGCTTGGCTATCT
CCAGAATCAGTCAACTGTTGGTGTTTTTTGTGGAGTCACTTATGATGGGACTAGATTCACAGGTATTTGCCATTTTTAAA
CTAGCAAAACCCATTACTTTACTTTGGAGAATATCTAATCATTTGACTACCTGGCTTGTCACCTGTCTAAGTATTTTCTA
TCTCCTTAAGATAGCTCATTTCTCCCACTCTCTTTTTTTTCTGGCTGAAGTGGAGAATGAACAGAGTCATTCTTGCGATGC
TTGCATTTTCTTTGGTCTTTCTGATTTTGGATATTCTTTTGCTAGAAACATTTAATGATCTCTTCTGGAATTTAATAAAT

GAAGGCAATTGGACTTTAGTTGAAAGTAAAACTCATTATATTAAAAGCGAGAATCTTCTTAGTTTCTCCTATTTCATTCC
TATTGTTCTGTCCCTGCTCTCATTGTTTTTTTTATTTTGGTCCTTGGTGAAACACACCAGAAATTTGCAGCTCAATTTTA
TGGGTTCCAGGGACTTCAGCACAAAGGCCCATAAAAGAGCCATGAAAATGGTGACGTCGTTCCTCCTCCTTATTATGGTT
CATTTTCTTTTTACACAATTGGCAAATTGGATGTTTCATAGGTTTTTGGACAATAAGTTCACAAAGTTCATCATGTTAGC
ACTATATGTCTTTCCTTCAGGCCACTCGTTCATGTTGATTCTGGGAAATAGCCAGTTAAGACAGATAGCCTTGAAGGTAC
TGAAGCATCTTAAAAGCTCCTTGAAAAGACAAAATCCATTGGCTTTATAG
>BotaTAS2R408C_chr5:99098189-99099130
ATGATAATGTTTATGTCAAACATTGTTTCCATTCTATTAATGACAGAATTTGTTCTGGGAAATTTTGCCAATGTCCTCAT
AGCACTGGTGAACTGCAATGACTGGACCAAGAGACCAAAGATCTCAGCTGATGGGATTCTCACTGCTCTGGCATTCTGCA
GAATTGTTATGCTCTGGGCAATGTTAATAAATTGGTATGTAATTGTGTATAATTTAACTCTATATAATTCAGAAGTAAAA
ATTATTGTTCATGTTGCCTTGACAGTAAGCAACCATTTTAGTAACTGGCTTGCTACTAGCCTCAGTATATTTTATTTGTT
GAAGATAGCCAATTTCTCCAGCCTAATTTTTCTTCACCTGAAGTGGAGAGTTAAAAGTGTAGTTCTCATGATGATGTTGG
GGACGTCATTGTTCTTGTTTTTTCAAGTTGCAGTGTTAAGCATGGATGAGGCTATTCAGACAAATGAATATGAAGGAAAT
ACCACTCAGAAGATCAAACTAAGGGACATTTTACACCTTTCAAATGTGACTCTGTTCACACTAACAAACTTTATACCCTT
CACTATGTCCTTGACATCTTTTCTGCTGCTAATCTTTTCCCTGTGGAAACATCTCAGGCAGATGCAGCTCAATGGTAAAG
GATCCCAAGATCCCAGCACCAAGGTCCACATAAAAGCCATGCAAACTGTCATCTCCTTTCTTTTCCTGTTTGTTATTTAC
ATTCTGGCTCTAATTGTATCAGTTTGGAATTCTAATCAGCTGCAGAAAGAACCAGTCCAAATGCTTTATGATGTTGTTTT
AATCATGTATCCTTCAATCCACTCATGTTATCTTGATCTGGGGAAATGGGAAATTAACTCAAGCCTTTCTGTCATTTCTA
TGGCAGTCAAGATGCTGGCTGAAGGAAAGGAAATAGGTGGAAACATATGTCTTTTAGCATAA
>BotaTAS2R408A_chr5:99065710-99066627
ATGATAACTCTACTATCAACCATTTTTTCCATCCTAGGAATAATACAATTTGTTCTGGGAAATTTTGCCAATGGCTTCAT
AGCCCTGGTGAACTGCATTGACTGGGTCAAGAGACAAAAGATCTCCTCAACTGATGTGGTTGTCACTGCTATGGCAGTCT
CCAGAATTGTTTTGTTCTGTGTAATGTTAATACATTGGTATTATATTTTGCTTCATCCAGCTTTATATAGTTTAAAAGTA
AGAACTATTTTTCATGTTGCCTGGACAATAAGCAATCATTATAGCACCTGGCTTGCTACTAGCCTCAGTATATTTTATTT
GTTGAAGATAGTCAATTTCTCCAGCCTAACTTTTCTTCACCTGAAGTGGAGAGTTAAAAGTGTAGTTCTCATGATGCTTC
TGGGAACTTCATTCATTTTGGTTTTACAAGTTGTAGTTATAAGCGTAAGTGGGACTATGCAGAGAAGTGAATTTGAAGGA
AACTTCACACAGAAGACCAAACTGAGGGATATTTTATGGCTTTCACATGTGACCCTGCTCATTCTAGGAAACCTCACACC
CTTTACTATGTTCTTAATATCTTTTCTGCTACCAATCTTTTCCCTGTGTAAACATCTCAGGAAGATGCAGCTCAATGGCA
AAGGATTCCAAGATCCCTGTACGAAGGTCCACATAAAAGCCATGCAAACTGTCATCTCCTTTCTCTTGCTATTTGCCTTT
TACTTTCTGGTTCTAATCATATCAATCTGGAGGCCTAAAAAACTGCATGAGGAACCATTTCTCTTGCTTTTCCCAACAGT
CAAAGTCATCTATCCTTCAGTCCACTCATTTATCCTGATTTGGGGAAACAGAAAGTTAACACAGGCCTTTCTGTTGTTTC
TGTGGCAGCTGGGGTGCTGGCTGAAAGAGAGGAAATAG
>BotaTAS2R60_chr4:107669854-107670792
ATGGTTCCTGGACCTCAGTTGGTTGATAAGACAGCCCCTTGTCTGCATTATTATTTTATTCCTTTTGTTCCTGGTGGCATT
GGTAGGTAATGGCTTAATCATCATGGCACTGGGCAGCGAGTGGCTGCTGCAGAGAACGTTGTCGCCTTGCGATAAGTTAT
TGGTCAGCCTGGGGGCCTCTCGCTTCTGTCTGCAATGGGTGGTGATTAGTAAGAACATTTACATTTTCCTGAATCCCATG
GCCTTCCCATACAACCCCGTGTTCCAGCTCCTGGCCGTTCAGTGGGACTTCTGGAACTCTGCAACACTGTGGTTCTCCAC
CTGGCTCAGTGTCTTCTACTGTGTGAAAATTGCCACCTTCACCCACCCCGTCTTCCTCTGGCTAAAGCGGAATGTATCTG
GGTTGGTTCCTTGGATGCTACTCAGCTCTCTGGGGTTCTCTACCTTTACCACCATTCTATTTTTCATAGGCAACCACAGA
ATGTATCAGAACTATATAAAGAAGGGTCTGCAACCTTGGAATGTCACTAGGAATGCTGTGAGAACATATGAGAGGTTCTG
CCTCTTCCCTTTGAGAATTGTTACCTGGACCGTCCCTACTGTTATCTTTATTGTGGGCACAGTTTTGCTCATTACATCTC
TGGGAAGACACACCAAGAAGGTCTTCTTCTCCATCTCAGGCTTTCACAGTTCCAGTGCCCAGGCACACATCAAGGCTCTC
TTGGCTTTTATCTCCTTTGCTATCTTCCTCACTTCCTCTTTTCTGTCACTGGTTCTCACTGCCTCAGGTATGTTTCCTTT
TGGGGAATTCCGGTTCTGGATATGGCAGACTGTGATTTATCTGGGTACAGCAATCCACCCCCTTATTCTTCTCTTGAGTA

ACCGCAGGCTGAGAGCTCTGCTAGGGAGGGGCTGCTCCTCAGCACATGGGGCATCTTGA
>BotaTAS2R18A_chr5:99102507-99103394
ATGTCTGACTTTGTGCGACCCCATAGACAGCAGCCCACCAGGCTCCCCCATCCCTGGGATTCTCCAGGCAAGAACACTGG
AGTGGGTTGCCATTTCCTCCTCCAATGCATGAAAGTGAAAAGTCAAAGTGAAGTCGCTCAGTTGTGTCTGACTCCTAGCG
ACCCCATGGACTGCAGCCTACCAGGCTCCTCCATCCATGGGATTTTCCAGGCAAGAGTACTGGAGTGGGGTTGGGCACTA
ACAAATCACTTAACTATCTGGTTTGCCACCTGCCTAAGCATATTCTACTTCCTTAAGATAGCCAGTTTCTCTCACTTCTT
TTTCATGTGGCTGAAATGGAGAATGAACCGAGTGCTTCTGGTGCTTTTCCTGGCCTTTTTCTTTTTATTATCTTTTGACC
TCTTAATGCAGGATGCTCTTGGTGAGTTGTGGATGAACACCTTTAGAGAACCTGAAAGGAACATGACTTTGCATTTAGAT
GCAAGTAAAATTTTCTATCTTAAAAGCCTTATTCTTCTCAGATTGACATATGTTATCCCTTTTCTTCTCTCCTTGGCTTC
TTTCCTGCTTTTCTTTCTTTCCCTGGTGAGACACATCAAGAATTTCCAAGTCAACTTGAACCACTCGAGAGACTTCAGCA
CAGAGGCCGATAAAAGGGCCACGAAAATGGTGACAACATTTCTCCACCTCGTCATCGTTTACTTTATTTCCATTCTAATT
GGAGAATGGATCTTCTTTAAGCTACACTGGTATGAGGTCATGATGTTTGTCATGGTGATTCCAACTCTCTTTTCATCCGG
CCACTCGTTTGTTATAATTTTGGGAAACAGCAAGCTAAGGCAGATTGCTTTCAGACTATTGTGGGGTCTTAAGTTCTCTA
AAAACTAA
>BotaTAS2R67_chr5:99035752-99036690
ATGCCATCTGGAATTGAAAACACTTTTCTAGCAGCAACAATAGGAGGATTCCTGATTGGAATTTTGGGGAATGGGTTCAT
TGTACTAGTTAACTGCATTGACCTGGTGAAGAGACAAAAGCTCTCATCAGCTGACTGCATCCTCACAGGCCTGGCTATCT
CCAGAATCAGTCAACTTTGGGTAATACTATGTGACTCATTTTTATTGGTACTATGGCCACACCTATATGCCATTGATAAA
CTAACAAAAGTTGTTAGTAGTTTTTGGATATTGTCCAATCACCTAGCTACCTGGTTTGCCACCTGTCTAAGTGTTTTCTA
CTTCTTTAAAGTAGCCAACTTCTCCCACCCCTGCTTCACTTGGCTGCGGTGGCGAATTCGTAGTGTGGTACTGGTGCTTC
TCTTGGGGTCTTTGTCCTTACTGTTCTTTAATTATGAATCAATATATACACTTAGTCATATCTTAACTAACAGCTACAAA
ATATATGTAAGAAACTCAACGTGGTCCTCAGATGTAAGTGAAACTCATTATCTTCACCAGTTGATTGTTTTTAACTTCAT
CAACTTAATCCCCTTTCTTCTGTCCCTGACCTCACTGCTCCTCTTAGTTCTCTCCTTGATGAGACACATCAGGAATTTGC
AGTTCAACCCCAGCTCAAAGGATCTCAGCACAGAGGCCCATAAAAGAGCCATGAAAATCGTGATGTCTTTCCTCTTCCTC
TTCATCATTCATGTCTCTTCCATCCTATTAATAGCTTGGGTTTTCCTTAAACTGCAGGGACGTCTGGCCCAATTGGTGGT
TGTGTTAACTTCGACTGTTTTTCCTTCAAGCCACTCCTTTATCCTAATTTTGGGAAATAGCAAGCTGAGACAGAATGCCT
TACGACTACTGTGGTATCTTAACTGCCACCCGAAAAGAGTGAAATCTTTAGCTTCATAG
>BotaTAS2R2p_chr4:20085208-20086116
ATGATCTCTTTGTCAGGTATTCCTCATGTTATCATCATGTCAGCAGAATTTATCACAGGGGTTACAGTAAATGGATTTCT
TATAATCATCAACAGCAATGAATTGGTCAAAAGCAGAAAGCTAACACCAATGCAACTCCTGTTCGTATGTATAGGGATAT
CTAGATTTGGTCTACAGATGGTGTTAATGGTACAAGGTTTTTTCTCAGTGTTCTTTCCACTCTTTTATAGCGCAAAAATT
TATGGTACACCAATGCTGTTTTTTTGGATGTTTTTCAGCTCTGTCAGTCTCTGGTTTGCCACCTGTCTCTCTTTATTTTA
CTGCCTCAAGGTTACAGGCTTTACCCAGTCCTGTTTTCTTTGGCTGAAAGTCAGGATCTCATAGTTAATGCCTTGGATGC
TTCTGGGAAGCCTGCTGACCTCTGTGAGCATTGCAGCTCTGTGTGTCAAGGTGGATTACCCTAAAATTGTGGATATTGAT
ATCCTCGGGAATGCCACAGCTAAGAGGACTAAACTCAACACAAAGCAAATTAATGAAGTTCTTCTCATCAACTTGGCATT
AATATTTCCTCTGACTATATTTATAATATGCACTGTTATATTATTAATTTCTCTCTACAAGCACACTCATCGGATGCAAA
ATGGACCTCTTGGTTTTAGAAACACCAGGACTGAAGCCCATATTAATGCATTAAGAACAGTGATAACATTCTTTTGCTTC
TTTATTTCTTACTTTGGTGCCTTCATGGCAAATATGACATTCAATATTCCTTATGGAAGTCATTGCTTCTTTGTGGTAAA
GGATATTATGGCAGCATATCCCTCTGGTCATTCAGTTATAATGATTTGGAGTAATTCTAAGTTCCAGCAACCAATCAGGA
GACTTCTCTGCCTAAGAAGGAGTCAATGA
>BotaTAS2R5p_chr4:105899673-105900550
ATGCCCTCTTCTATCCTAGGACTGCTGATGCTGGTGGCAGTAGCTGAATCTCTCATTGGCCTCATTGGAAATGGAGTTCT
TGTGGTCTGGAGTTTCGGAGAATGTCTCCGAACGTTCAGGGCGTCCTCGTATAACCTCATTGTCCTGGGCCTGGCGGTCT
GTCAGTTGCTTCTACAATGGTTGATTATGGTGGACTCAAGTCTGTTCGTGCTTTTCCAGAGCAGCCATTGGCTTCGCTGG

CTCAGTGTCTTCAGGGTTCTGGTAAGCCAGGTCAGCCTGTGGTTTGCGAGTTTTCTCAGTGTCTTCTATTGTAGGAAGAT
CATGACCGTTGAACACCCTGTCTCTTTGTGGCTGAAGCAGAAGGCGTTACCTGAGTTGCTGGTGCTTTCTGGTGTACTTC
ACGATCCATTTGTTACTTACAGTCAGGGGTAGCTTAGACTTCTCCAGTCCTTCCCAAGGAAACAGCAGCATCTTATTCCC
CATTTCAAAC
TGGCACTATATATGTATATTACAGCTCAATACAGAAAGTATGATGCCTTTCACGATGTTTCCTGTTTCCTCTGGGCTGCT
GTGTCTCTTTGTATAGACGCTACAGGAAGATGAAGGTCCATACAGCCGGCAGAAGAGATGCTCAGGCCAAGGCTCATATC
ACTGTCCTGAAGTCCTTGGGCTGTTTCCTTGTACTTTACATGGTCTACATCCTGGCCAGCCCCTTCTCCATCAGCTCCAA
GACTTTTCCTGCAGATCTCTTCACTGTCTTCATCTCTGAGACACTCATGGCCACCTACCCTTTTCTTCATTCTGTCATAC
TGATCATGGGGAACCCCAGGATGAAGCAGGCATGTCAGAGAATCCTGTGGAAGACTGTATGTGCTTGA
>BotaTAS2R8p_chr5:99256642-99257571
ATGTTCAGTATAGAAGACCACATCTTTCTGACCATAATGACTGCGTAATTCATCATAGGAATGTTTGTGAATGGATGCAT
TGGACTAGTAATATGTGTTGATTGGATTAAGAAGAAAAAGATCTCCATAGCTGACTACATCCTCACCAGTTTAGCTCTCT
CCAGAATGTATTTGCTTTGTGTAATGACACTCAACGGCACCA
TACTGGCACTCTACCCAGGTGTTTATGAAAATGAGAAAATAAAGGTAGTTCTTAATATCTTCTGGACATTCACCAACTAC
TTAAGTATGTGGTTTGCCACCTGCCTCAATGTCTTCTGTCTTTTCGAGATAGCCAATTTCTCCCACCGACTTTTTCTCTG
GCTGAAGTGGAGAATTGAGAGGGTGGTTCACTGGAGCCTACTGGGGTCCCTGGCCATTTCCATGTTGATCAGCTTATACA
AGCAACGTTAACAAATTCTGATTATGATTTTCTTAAAATTGCAAAACATAAAAGAAACGTCACCGAATTGTTCCATGTGA
GTAAAATTCAATACTTCGACCCATTGACATTGTTTAACCTGTTTGCTATTATTCCATTTACTGTGTCATTGATCTCATTT
TTCTTTTTAATTACATCCCTGTGGAGATACAGTAAACAAATGAAATCCAGTGTTACAGGCTCCAGAGACTCCAGCACAGA
GGCCCACGTGGAGGCCAGGAAAACAGTGACCTCATTTCTTTTCTTCCTTTTTGTATACTACCTGGCCTGTCTTTTGGCAA
CATTTAGCGACTTTATGAAAGAAAGCAAGTTAGCTATGATGTCTGGAGAGATTATAGAAATTCTTAATCCCTTAGGTCAC
TCACTGTTTTTTATTGTTGGAAATAACAAGCTGAGGCTGGCATCTGTCAGGACGCTGAGATGTGGGAAAACAGCCTGCAT
GATGTAA
>BotaTAS2R10Dp_chr5:99214431-99215330
ATGGACTGGTTGGATCTCCTTGCAGTTAGTGAGTCAATATTGGGACTTTAAGGGAATGGATTTATTGGATTTATAAGCTG
CATTGATGGTATGAAAAACAAGAAGATCTCTACTATCAGCTTTATTCTCGCTGGCTTAGCAACTTCCAGAGTTTGCCTGA
TATGGACAATAGTTACTGATGGATTTTTAAAGTTATGCTCTCCAGATGTACATTCCTCTGGGAACCTAATTGAATATAAT
GGTTACTTGTGGATAGTGATGAATCAATCAAGTATCTGGTTTGCTACCTGCCTCAGCATCTTCTATTTCCTGAAGATATC
TAGTTTTTCCCACTGCATCTTTCTCTGGTTGAAGGGTAGACTCAACATGGTTGTTTTCCTTCTTTTGGGATGCTTGCTTA
TTTCATGGTTAGTTACTTTTCCACATTTTGTGAAGATTGTTAATGATGATAAAAGGAAAATAAAAACACAGTCTGGTCAA
TGGATATGCATAAAGGTGAACTCTTTGGAAAACAAATTTGGCTCCATCTTGGTGTCATTCTCCTTTTTATACAATACCTG
ATTATATGTGTCTTGTTGCTCACTTCTCTTTGGAGACACAACAGGAGGATGCAATTGAATGCCACAGGATTCAGTGACCC
CAGTACAGAAGCACATATCAAAGCAATAAAAGTCTTGGTATCTTTTATCATCCTCTTTATCTTGAATTTTGTAGGTACTG
CCATACAAATATCTAGTGTGACAGTGCCTGAAAACAAACTGCTTTTTATTTTTGGTATGACAACCACAGTCCTCTATCTC
TGAGGTCACTTGCTTATCCTAATTCTAGGAAATAGGAAGCTCAAGCAAGCCTCTTTGAGAGTACTGAAGTCATTAAAGTG
CTGGGAAAAAGAGAAACTTCTCAGAACTCCTTGA
>BotaTAS2R11p_chr5:99153031-99153925
ATGTTGAATATATTGGAGAAGATTTTCATGGTTGTGACTGGTGGGGAATTTATAATAGGAATTTTAGGGAATGGATTTAT
TGGACTCACAACTTGCATTGCCTGGATTAGAAATCAGAAGTTGAGCTTGCTTGACTTCATTCTTACTAGTTTGGCCTTTG
CCAGAATCAGTCAATTATGGATAACCACTGTCATGTTCTTTTCAATGATGTTCTATCAGGCAGGCTTTGGTACTGTGGGA
AGAAAATATATCTTTTTTTGTATCTGGATACTGACCAGTCACTCAAGCACTTGGCTTGCTACTTGCCTTGCTGTCTTTTA
TTTCCTGAAGATTGCCAGTTTCTCCCATCCTCCTTTCCTTTGGCTAAAATGGAGAATTAACAAGGTTGTTTTCATGCTTC
CACTGGTATCTGTGCCCTTCCTAGTCATAAGTTTTCCTTGGCCAAATAATGTTGATGTCTTCTGGTGTCATGTCCAAAAG
ATGCATGAGAGAAGTATGACTGAGTTATGCAATGTGAATGAATATCAAAATTTAAATTTTATTATTATCTTCACAATGGA

GTCCCTCCCACCTTCTTTCTTTCCCTGATTTCCTTTCTCCTGTTGCTCCATTCTTTGTGGAAACACAAGAAGAACATTGC
ACACACTGTCAGGAATTCCAGAGACCCCCGTGTTGAGGCCCATTTCAGAGCCATGAAAACTGTGTTTTTCTTTCTCATGC
TCTTTGTCCTGTACCAATTTGGCCTTTTCATGACATTTGGGGGGCATTTTTTCCTACAGAACAAGCTGGCTGTGATGTTT
GGTTATATGTTAGGAATGCTGTATCCTTCAAGTCACTCATATGTTTTAATTTTTGGAAACAGTCAAATGAGGAAATTCTT
CTTGGTGATTCTTAG
>BotaTAS2R40p_chr4: 107447957-99215344
ATGGTGACGGTGAACACAGATGCAATGGATAAAGACACGACCAGGTTTAAGATCGTCTTCACCTTGGTGGTCTCTGCAAT
AGAGTGCCTCATTGGCATTGCTGGGAATGGCCTCATCACCGTCATCCATGGAGCCGAGTGGGTCAGAGGCAAAAGACTCC
CCATTGGACTGCATTCTGCTCATGCT
GAGCTTTTCCAGGCTCTTGCTACAGATTTGGATGATGCTGGAAAACACGTACAGTCTGCTGTTCTGGGTCATCTACAATG
AAAAAAGAGTATACATACTTTTCAAAACCATCATCATGTTTCTGAACTACTCCAACCTCTGGCTTGCTGCCTGGCTCAAT
ATCTTCTATTGTCTTAGAATCGCAAGCTTTACTCACCCGTGGTTCTCCGTGATGAAAAGGAAGGTCATGTGGCTGATGCC
TGGGCTTGTGAGGCTGTCCTTGTTCTTCTCCTTTTGCTCCAGCTTTCCCTTCTCTAAAGGTATATTCAACGTGTATGTGA
ACAATTCCGTCCCCATCCCCTCCTCCAACTCCACTGAGAAGGTGTACTTCTCCGAGACCAACATGGGCAACTTGGTTACC
ACCCTTTACCTGGGGATCTTCATCCCTCTGATCATGTCTATGCTGGTGGCCACCCTGCTGATCATCTCTCTCAAAAGACA
CACCTTCCACATGAAAAGCAATGCCACTGGCTCCAGGGACCCCAGCATGGAGGCTCACCTGGGGGCCATCAGAGCCATCA
GCTATTTTCTCATTTTCTACATTCTCAATGCAGTTGCTCTGTTTTTTTTCCATATCCAACATCTTTGCCGCCAACAGCTC
CTGGAATATTTTATGCAAAATCATCATGGCTGCCTACCCTGCTGGCCACTCAGTGCTACTGATCTTGGGCAACCCTGGGC
TGAAAAGGGCATGGAAGCAGTTTCAGCACCAAGTTCATCTCTACCTGTAA
>BotaTAS2R408Dp_chr5:99057485-99058400
ATGATAACTTTACTATGGACCATTTTTTCCATCCTAGTATTAACAGAATTTGTTCTAGGAAATTTTGCCCATGGCCTCAC
AGCACTGGTGAACTGCATTGATTGGGTCAAGAGACAAAAGATCTCCTCAGCTGATGGGATTCTCACTGCTCTGGCAGTCT
GCAGAATTGTTTTGCTCTGGGTAACGTTAATGAATTGGTACTTAGTTGTGTTGAATCCAGTTCTATATAGTTTAAAAGTA
AGAATTATTGTTCATATTGCCTGGATAGTAAGCAACCATTATAGCACCTGGCTTGCTACTAGTCTCAGCATATTTTATTT
GTTGAAGATAGCCATTTCTCCAGCCTAATTTTTCTTCACCTGAAATAGAGTTAAAAGTGTCATGCACATAATACTTCTGG
GAACTTCATTCTCCTTGGTTTTTCATGTTGCAGTGATATACAACGATAAGGCTATCCAGACAAATGAATACAAAGGAAAC
ATTCCTCAGAAGACCATATTGAGGGGCAGTTTATGGCTTCCACATGTGACTCTGCTTATGCCAGGAAATCTCATATGCTT
TACTATGTCCCTGACATGTTTTCTGCTATTAACTGTTTGCCTGTGGAAACATCTCAAGAAGATGCAGCTCAGTGGTAAAG
GATCTCCAGATTCTAGCACCAAAGTCCATATAAAAGCCATGCAAACTGTGATATCCTTTCTCTTGCTGTTTGCCATTCAT
TTCCTGGCTCTAAATGGGATCCATTTGGAGTTTTAAAAGGCAGCAGAAGGAAACTGTCTTTTTGTTCTTTGAGGCTCTTG
GATTCCTCTATCCTTCAAACCACTCATGTATCCTGATTTGGGGAAACAGGAAGTTAACAAAGGCATTTCTGTCATTTCTG
TGGCAGCTAAGGTGCTGACTGAGAGAAAAGAAATAG
>BotaTAS2R408Ep_chr5:99114750-99115627
ATGACAACTTTACTACATTTTTTCCATTCTGGTAATAGAATTTATTCTAAGAAATTTTGCCAGTGGTTTCATGTCACTGG
TGAACTGCATTGACTGGTCAAGAGACAAAAAATCTCTTCAGCAGATGGGATTCTCACTGCTCTGGCAGTCTCCAGAATTG
GTTTGCTCTGAGTAACATTAATAAATTGGTGTGAAAATGTGTTTAATCCCAGCTTTAGACAATTTAAGAGCAAGAATTAT
TATTATTGCCTGGATAATAAGCAACTGTTTTGGTACCTGGCTTGCTGCTATCCTCAGCATATTTTATTTGCTCAAGATAG
CCAATTTTCCAATATTATTTTTCTTTACCTAAAATGGAGAATTAAAAATGTTCTTCTTGTTCACTTTGTCCTGTTTGGTT
TTATTAATTCATGGTGTAAATGTAAACAAGACTATCCAGACAAATGACTATGAAGGAAACATCACTCAGAAGACCAAGCT
GAGGGACATTTTACACCTTTGAAATATGACTCTGTTCATGCTAGTAAACTTCACACCCTTTGCTATGTCCCTGACTTCTT
TTCTGCTGTTTATCTTTTCCCCATGGAAACATTTCAGGAAGATGCAGCTCAGTGGTAAAGGACCCCAAGATCCCAGCACC
AAGGTCCATATAAAAGCCATGCAAACTGTCATCTGTTTTCTATTTGCCATTTGCTTCCGGGTTCTAATTTTCTGTTTGAA
GTTCTAATAGGCAGTGGAACAACTTGGTTATCATGGCATGCCAGGCTTTTGGAATCATATGTCCTTCACTTATCCTGACA
TGGGGAAAGCAGACACTAGGACAGTCCTTCCTGTCATTTCTGCAGCAGCTGAGTTGATGGCTAAAAGAAAGGAAATAA

>BotaTAS2R62p_chr4:107659943-107660853
ATGTTGATATTCAAGGTCATCTTTTTCCTGGAGTCATTGGTTGCTGTGCTGCAAAATGGCTTCATAGTTACTGTGTTGAG
CGGGGAGTGGGTGTGAAGCCAGATGCTGCCCGCTGGTGACATGATTGTGACCTGCCTGGCTGCCTCCTGGTTCTGTCTGC
ATGGGATGGCCCTCCTGAACAACATCATGGCCTCTTCTGGCTTTTGTTCCAAAATCGACTATTTCAGCATTCCCTGGGAT
TTCATCAACTGCCTCAGTTTCTGGCTGACTGCCTGGTTTGCTGTCTTCTACTGCGCGAAGATCTCCCTCTTCTCTCATCC
CCTCTTCTTCTGGATAAAATGGAGGATTTCTCGGTCGGTTCCCCAGCTGGTGCTGGGCTCCTTGATCTTATCTGGTCTGT
CCGTCATCTCAGCTGCTGGGAATACAATTCTTGCCCAGATGACGGCTGCCCAGATTTCCCATGGAAACGACACCCTGGCT
GGTAGAATACATGCTACCTATTTGCACTTTTTCTACCTCATGTAATTCTCATGTGGTTGGTTCCATTCCTCCTGTTCCTG
GTGTCCACCCTCTCGCTCATGTTCTCACTGCGCCGGCACCTCTGGCAGATGCAGGACCACAGACCCAGCCCACGTGATCC
CAGTACCTGGGCTCACACCATGGCCCTGACGTCACTTGCCTTCTTCCTCATCTTCTACACCTTGTACTTCCTGTCCCTGG
TTATCATTATGTACATCCCAGCCCTCCAGGAACACTGGCACTGGGCCTGTAAGGTGGTGACCTACACGGGCATCTGTCTG
CACTCCAGCATCTTGGTGCACAGCAGCCCCAAGCTGAGAAAGGGCCTGAAGAAGAGGCTTTGGCGAGCCCTGGACAAGGA
CCAGTTTGTCTCCAGTTATCAGTATCAATAG
>BotaTAS2R18Bp_chr5:99077438-99078380
ATGTCAGTTGGAATGAAGGGCTCTTTCTACTAGTGGCAACAGGAGAACTCATCTTAGGAGTGCTGAGAAATGGGTCAAGA
ACAGGAAAGTCTCATCAGCTGGTTTCATCCTTACCTGCTTAGCTGTGGAGAGAATCATTCAAATGTGGGTAACACTATTG
GGTTCATTTACAGCGGGGCTATTTTCACATCTGTATGCTACCAGCAAACTAGCAGAGGTGATTACTCTTTTTTGGGCACT
AACGAATCACTTAACTACCTGGTTTGCTAACCCCTAAGTGTGTTCCATTTCTTTAAGATAGCCAATTTCTCTCATTTCTT
CTTCACATGGCTGGAGTGGAGAAGGAACAGAGTCATTCTTATACTTTTCCTGGGCTCTTTGCTCTAACTGTCTATTAACC
TCTTAATGCTCTTGGTGAGTTGTGGATGAGTAGCTATAGAGAGCCTGAAAGAAACACAGCTTTGCATTTAGATGCAAATA
AAATTTTCTATCTTAGATGCCTTATTCTTCTTAGCTTGACCTATGTTATCCATTTTCATCTCTCCCTGGCCTCGTTGTGC
TTTTATTTTTCTCCTTGGTGAGACACACCAAGAATTTCCAACTCAACCTGAATGGCTCAGAAGACACCAGCTCAGAGGCC
CATAAAAGGACCGTGAAAAGGGTGACAACCTTCCTCTTCCTGTTCATCATTTACTTTTTCCACTCCATTAGGGAGCTGGA
TCTTTCTTAAGGTACAGCAGTATCAGGCCATGATGTTTGTCATGAAGATTTCAACTGTCTTCACTTTGGGTCCTCTTATG
TTTTAATTTCGGGGATTAGCAAGCTAAGAAAGATCACCTTGAGTTTAATTTTGAATCTTATATTCTCTTTGAGAAAACCA
AGATCATTAGTTTCATGGACAGAATTTAAATGTACTTTATGTATTCTGGAGAAAATGCCTTAA
>BotaTAS2R372BP_chr5:99198192-99199157
TCAGCAGTGACCAAAGTAACCAGGATTTCACTTCTGCAGTCATGTCCAGTGTAATCAAAAGATTTTTATGATCATTGAAA
TGTTATAATTCATAACAGGAATAACTGTGCTGACTTTGTCAAAAGCAAGAATAGTGCCTTGTTTGACTTCATCTTCACAT
GGATTAGCGTGATGTTCATACTTTTCCTAGATTGCGTTAAACTAGTGTTCCATCTAGAAATATTAGATGGTCACCAGGTA
ATAAGAGGAGTTTCTGAGTTCTCCTGGAGTCTGAGAAACTCATTAAGTACTGGATGTGCTGCCTGCCTCAGTGTCTTCTA
CTTCCTCAAGCTATCTAGTTTTTCTCACCCCTTCTTTCTCTGGCTGAAGTGCAGAAGAGATAGAGTTGTTTTCACCATTA
TGTTGGGATTCTGTCTCTTTTTGATTTTTAACCTTCTGAGCATAAAATTTATACTTTTGTGTTCAGCAAGCATTTAGAAA
AGGAAAGACTTAACTTGGAAAAAAGATATGCATAAAAATCAGTATTATAACAGTCAAGTTCTCTTCAGCCTTGGGTCTCT
CATCCCCTTGTCTGTATCACTCATTATATTTTTCCTGTTAATCTTTTCCTATGGGGACATACCAAGCAGATGACATGCCA
TAACACAGATCCCAGGGACTTCAATGCCGGGAGCCACCACGGGAGATTCCACCCATGACAAAGGTCATGCGGAAGAGACC
TGACAGGCAAAGGAGGATCAGGCCTCAAGGGACCCCCTGAATCTTCTTGAGCATCTACCCCAAAACCAAAATCTGTCTAC
TGTTTATTATATTATGCCTTTCACCAACTCTTCTGTCATTAACAGGGGGCTATCCCCAACCGGGAGAAATATCAATAACC
TCAGATATGCAGATGACACCACCCTTATGGCAGAAAGTGAAGAGGAACTCAAAAGCCTCTTGATGAAAGTGAAAGAGGAG
AGTGAA
>BotaTAS2R372AP_chr5:99177536-99178271
ATGTCAAGTGTAATCAAAAAAGTTTTTATAACCATTGAAATCTTAGAATTCATAACAAGAATTTGCAGAAATGAATTCAT
TGCACTAGTACTCTGTGCTGACTCTCTCAAAAGCAAGAATGTCTCCTTGTTTGACTTGATCTTAACAGGCTTGGCCATCT
CCAGAACTGGCATGATATTCATAATTTTCTTGGATGGCATTAGAATAGTGTTCTATCCAGGAATATTTGAAAGTCATCAG

GTAATAGATGTAATTTTTTATTTCCCATTCTGTTGGGATTCTGTATTTCTTTGATTTTAATTTTCTGAGTATAAGTTTTC
ATACATTTGGGGTCAGTGACCATTTAGAAATAGAAAATAACTTGACTTGGGAAAAATATGCATAAAATCCGATCCTATAG
CAGTCAAATTCTCCTCCACCTAGGATCTCTCATCCCCTTGGCTGTGTCACTCATTTTATTTTTCCTGTTAATCTTTTCCT
TATGGAAACATACCAGGCAGATGACACGTCATGTCAAAGGATCCAGAGACCTCAACACAGGAGTTCTTGTGAGAGCCAGA
AATACATGACTTCCTTCATCATTTTCTTAGTTGTGCACTATTTGGCTACATTCTTGTTAACTTGATCCTGTTTCACACTA
GAAAATGAAATGACTTTTATTGTTATTAAGTCTGTAGCATTTCTCTATCCTTCAATTCACCCTTTTATTTTGATTCTAGG
AAACGGAAAACTGAGA
>BotaTAS2R7AT_chr5:99262343-99262952
ATGTCAAGTGAAGGGCAGAGTATCTTAATGCTCATAGCAGCTGGGGAATTTTCACTGGGGATCTTAGGGAATGCATTCAT
TGGACTGGTAAACTGTGTGGACTGGATCAAGCACAAGAAGATTGCCTCCATTGATTTAATCCTCACAAGCCTGGCCATCT
CCAGAATTTCTCTCTTATGTATAATACTGTTGGATTGTTACATATTGGTCCTGTACCCAGATGTCTATACTGGTGGTAAA
CAAATGAGAATCATTGACTACTTCTGGACACTAACCAACCATTTAAGTGTCTGGTTTGCCACCTGCCTCAGCATTTTCTA
TTTCCTCAGGATAGCAAATTTCTTCCATCCTTTTTTCCTCTGGATGAAGTGGAGAATTGACAGTGCAATTCCTAGGATCC
TGCTGGGGTGTTTGGTCTTCTCGGTGTTCATTAGCCTTCCTGTCATTAACAATTTGGATGATGATTTCAGGCATTGTGTC
AAGATGAAGTTGAAAACAAATTTAAGTCGAAGATGCAGAGTACATAAAGCTCAGCATGCGTCCATCAAGATACGTCTCAA
TCTGTTGACACTACTTCCCTTTTCTGTGTCCCTGATCTCATTCCTCCTC
>BotaTAS2R7BT_chr5:99263066-99263278
CTTTTCATTGCCTACTACTTGGCCTATCTTGTGGCCACGTCCAGCTACTTTATGCCAGAGACTGAATTAGCTGTGATCGT
TGGTGAGTTGATAGCTTTAATCTGTCCATCAAGCCATTCACTCTTCCTAATTCTAGAGAACAAAAAATTAAGACAAGCAT
CTCTAAGGGTGCTTTGGAAGGTAAAATGTATCCTACGAAGAAGGAATTGCTAA
>MydaTAS2R1_NW_006294876.1:6320407-6321306
ATGTTAGAGTTATACACTATTGCCCATCTTATTTTTTCAGTGATTCAGTTTCTTGTGGGGGTTCTAGCCAATGGCTTCAT
TGTGGTTGTGAATGGCACAGACTTCATCAGGTGGAGAAAGATGGTGCCTTTTGACCTCCTCCTGTGCTGCCTGGCGACTT
TCAGGATTGGTCTCCAGATGGTCATGATCTTCATTAATCTGGCTATTCTTTCCTTGATTGAATTCTCTCCAGTTCCTGGG
AATATTATAATTTTCATGTTTGTACATTCATCGGAACTTTGGTTGGCCACGTGGCTCAGCGTTTTCTACTGTGCCAAGAT
CGCCACCATCGCGAACCCGCTCTTCTTTTGGTTGAAGTTGAGGATCTCCAAGTTGGTGCCGTGGCTGATTTGTGGGACCT
TGACGTATACCTTTCTCACTTCTGTCTTCCACAGAAAACATGCATGGATTATTTCCCAAAAATCCTGGTTGGGCTTTTTC
TCCCAAAATGCAACAACTCAAATTGAAGACATATCTGCATTACAATTTGCCTTTCTTTTAATTGAGTTCGTATTGCCTTT
ATTTGTCTTCCTTATCTCTGCTCTTCTCTTGATATTTTCCCTGGGGAGGCACACCCAGCAGATGAGGAGCACTGAGATGG
GCACCAGGCACCCTGGCATGAGTGTCTACATCAGCGCACTCCTATCCATCTTGTCCTTCCTGATCCTCTACCTCTCCCAG
TACATGATCGCTATATTAGGTTTTTCTGAAATTTTCAAGATCAAAAACTCCATCACTCTGTTCTGCATCTTGCTGTTTGG
TTCATACCCCTCTGTACACTCTATTATCTTAATTTTAGGAAACCCTAAGCTGAAACAAAATGCGAAGAAGTTCTTCCTCT
ACAGTAAGTGCTGTCAGTGA
>MydaTAS2R2_NW_006383016.1:12204518-12203607
ATGACCTCCTCTTTGTCAGCTATTCCTCATGCTATCATCCTGTCAGCAGAATTTATTACAGGGATTACCGGCAATGGATT
TCTGATAATCATCAGCTGTAACGAATTGATCAAAAGCAGAAAGCTAACACCAATGCAGCTCATTTTAATATGTATAGGGA
TGTCTAGAGTCGGTCTGCTGCTGATGTTAATGGTACAAAGTTTTTCTCTATGTTCTTTCCACTCTTTTATCAGACAAAA
ATTTATGGTGCAGCGATGATATTCCTTTGGATGTTTTTAGCTCTGTCAGTCTCTGGTTTGCCACCTGCCTTTCTGTATT
TTACTGCCTCAAGTTAATAGGCTTCACTCATCCCTGTTTTCTTTGGCTGAAATTCAGGATCTCAAAGTTAATGCCTGGGC
TGCTTCTGGGAAGCTTGCTGGCCTCGGTTAGCACTGCAACTCTGTGCATTGAGGTAGATTACCCTAAGGACGCGGTGGAG
GGTGTCCTTAGAAATGCCACACGCACGACGTCTAAATTCAAGCTAAGGAGTATTAATGAAGTGCTTCTTGTCAATTTTTC
ACTCCTATTTCCTCTAGTCATATTCCTGATGTGCAGTTTTATGTTACTCATGTCTCTCTACAAGCACACTCATCGGATGC
AAAACGGATCTCGTGGTTTTCGAAGTGTCAGCACAGAAGTCCATATAAACGCCTTAAGAACAGTGCTAACGTTCATTCTT
TTCTTTATTTCTTATTTTGTCGCCTTCATAACAAACATGACATTCATTATTCCTCACGGAACTCAGCGCTACTTTGTGCT

GAAGGACATAATGGCAGCATATCCCTCTGGCCACTCAGTTATAATAATCTGGAGTAATTCTAAATTCCAACAACTACTCA
GGAGACTTTTCTGCCTCAAAAAGAGTCCATGA
>MydaTAS2R3_NW_006300374.1:342665-341715
ATGTCAGGACTCGCCAAGTGGGTGTTTTTGTTTCTTTCTGTCACTCAGTTCCTTCTGGGAATGCTCGGGAATGGCTTCAT
TGTGCTGGTCAATGGCAGCAGTTGGGTCAAGAGCAAGAGAATCTCTTTGGGTGACTTCATCCTCACTAACCTGGCTCTCT
CCCGGATTGTTCAGCTGTGGATTCTTTCTTCTGATTTTGTAATAATGATATTCTTTTCCAAATTATTAAATAATGTGGTA
CTCATACAAGTTACTGATATTTTCTGGACATTTACAAACCATCTGAGCATTTGGCTTGCCACCTGTCTCGGTGTCTTCTA
CTGCCTGAAAATTGCCAATTTCTCCCACCCCACATTCCTCTGGCTCAAGTGGAGAGTTGCCAGGGTGGTCGTATGGATGC
TGTTGTGTGGCCTGCTCTTATCGTGTAGTAATGCCATGTCTCTGATTCCTCAGTTTAAGATGTACTATATTCTCCGTGGA
GCTGATGATTCAGGGAATGTGACTGAGCACTTTAGAGAGCTAAAGAATGAATATGAGGTGATCCATGTTCTTGGGACACT
GTGGAACCTCCTTCCCCTAATTGTGTGTTTGGCCTCCTACATCCTGCTCATTCTCTCCCTGGGGAGGCACACGCGGCAGA
TGCAGCAGAACAGAACCAGCCCCAGCGATCCCAGCACCGAGGCCCACAAGAGGGCCATCAAAATGGTCCTCTCCTTCCTC
TTTCTCCTCCTGCTTTACTTTCTTGCCTATTTACTCACATCATTCCATTATTTCCTATCAGGAACTGTGTTGACTAAGAT
GATTACAGAACTAGCTGCAATGTTTATCCTGCCTGCCACTCATTTATTCTCATTCTGGGAAACAGTAAGCTGAAGCAGA
CATTTGTGGAGCTGCTCTTGTGTAAGTCTGGTCATCTGAAGCCTGGATCCAAAGAACACTTTTCCCCATAA
>MydaTAS2R4_NW_006300374.1:333072-332173
ATGCTCCAAATATTTCTCTCTCTGTTATTATCTCAGTAGTTTTGGATTTTGTAGGACTCATTGTGAATCTGTTTATTGC
AGTGATCAATTACAAGACTTGGGTCCAAAGCCACGGAATGTCCTCTTCGGATAGGATCCTGTTCAGCTTAGGCATCACCA
GATTTCTCATGATGGGAATATCCCTGGTGTACATCTTCTGCTTCTTCATCTCTCCAAATGTTGAAAGGTTAGTTTACTTA
TCCAAAATTTTCCTGTTGTTTTGGGTGTTTTTGGACTCCAGTAGCCTCTGGTTTGTAACTTTGCTCAATGTCTTGTACTG
TGTGACGATTGCTAACTTGAAATACTCAATTTTTCTTCTGCTGAAACGAAATCTCTCCCCAAAGACCCCCAGGCTATTGC
TGGCCTGTGTGTTGCTTTCTGCCTTCACCATGCTTCTGTATATTGTGCTCAGACAGAAGCTATTCTTTCCTGAATTTGTG
ACGACGGAGAGAAATGGCACAGAATTTAACGCCGATGAGGGCACCTTGTCAGTGATGATCTCTTTGTTCTTGAACTCATT
TCTCCAGTTCATCATTAATGTGACTGCTGCTTCCTTGTTAATAAACTCCTTGAGGAGACATATACAGAAGATGCAGAGAA
ACGCCACTGGCTTTTGGAATCCCCAGACTGAAGCTCATGTGGGTGCTATTAAGCTGATGGCTTATTTCCTCCTCTTCTAC
ATTCCGCATACAGTTGCCACCCTGTTCCAATACCTCCCTTCTAAAGATTTGGATTTGGGAACCAGATCCATATGTATAAT
AATTTCCACCTTTTACATTCCAGGACATTCTGTTCTCATTGTTCTCACACATCCTAAACTGAAAAGCAAAGCAAAGAAGA
TTATTTGCTTCAACAAATAG
>MydaTAS2R10_NW_006286849.1:8341-7442
ATGCTAAGTATAGTGGAAGGCCTCCTCATTTTTATAGCAGTTGGTGAATCAATACTGGGGGTTTTAGGGAATGGATTTAT
TGGATTTGTAAACTGCATTGACTGTGTCAAGAACAAGAAGTTTTCTCTAATTGGCTTGATTCTCATTGGCTTAGCTACTT
CGAGAATTTTTCTGATATGGATAATAATTACAGATGGATTTATAAAGATACTCTCTCCATATATGTACTCCTCTAGAAAC
CTAAATGAATATATTAGTTATTCATGGATAATTATAAATCACTTAAGTATCTGGTTTGTCTCCAGTCTCAGCATCTTCTA
TTTCCTGAAGATAGCCAATTTTTCCCACCACATTTTTCTCTGGTTGAAGCATAGAATCAACAGAGTACTTCCTCTTCTGA
TGGGATTGATGCTTATTTCATGGTTATTTATTTTCCCACAAACTGTTAAGATTATGAATGAATATAAAATAAATAATGGA
AGCACAACCCGGCACCGCGACATATCTAAAAGTGAATACATTGCTTACCAAATTTTGCTCAATCTGGGAGTCATTTTCCT
CTTCACCCTGTGCCTGATTTCATGCCTCTTGTTAATCATTTCTCTTTGGAGACACAACAGGAACATGCAATCGAGTGCCC
GCGGTGGCGGAGACCCCAGCACAGAAGCACACGTGAGAGCAATGAAAGTGTTGATATCTTTTATCATCCTCTTGATCTTG
CATTTTATAGGCATTTCCATAGAAATAGCATGCTTTTCTGTGCCAGAAAACAAATCGCTGTTTATTTTCGGCATGGCCAT
CGCAATCACCTATCCTTGCGGTCACTCATTTCTCCTAATTCTAGGAAACAGCAAGCTAAAGCAGACTTTTCTGAAGGTAC
TACGGCCTTTCAATAGCTAA
>MydaTAS2R11_NW_006286849.1:21051-20119
ATGTTGGAGAAAGTTTTTGTGATTATAATAGGTGGGGAATTTTTAATAGGAATTTTGGGGAATGGATTCATTGGACTCAC
AAATTGCATTGCCTGGGCTAGAAATCGGAAGTTATGCTTGGTTGACTTCATTCTCACCAGTTCGGCCTTCACCAGAATCA

GCCTTTTGTGGCTAACAATTGTCAATTTGTTTTCAGTGCTGTCCTATCAGGAAATCCCTGCTACTATGGAAGGAAACCTT
ATTTGTTCTAGTTTCTGGATACTGGCCTCTCACCTGAGTACGTGGTTAACTACTTGTCTTGCTGTCTTTTATTTCCTGAA
GATCGCCAATTTCTCCTCGCATTTTTTTGTTTGGCTAAAATGGAGAATTAACAAGGTAGTTTTCATGCTTCTGCTGGTAT
CTTTGCCCTTCCTGTTCCTGAGCCTTCCTTTGCCATATCATTTTGATATTATCTGGTATCATTTCCCCCCAAAACATGAG
GGAAATATGACTGAGTTATTCAATGTGAGTACGAGTAAAAATTTAGATCAGATTATCATGTTCATGATTGGGTTCCTCCC
TCCTTTCTCTGTTTCCTTCATTTCCTTTTTCCTGTTGCTGCTTTCTTTGTGGAGACACACAAAACACGTTGACCTCAACA
TTAGGAACTCCAGAGATGCCAGTATGGAGGCCCACACCAGAGCAATGAAAACTGTGTTTTTCTTTCTTGTGCTCTCTGCA
CTGCAGCAATTTGCCTATTTTATGACATTTGGGGGATATTTTTTGCAACAGAACAAGCTGGTTGTGATGCTTGGTTATAT
GATAGGAATTTTATATTGTTCAGGGCACCCATATGTTGTGATTTTTGGAAACAGCCAAATGAGGAAAGCCTTCTTGTGGA
TTCCTTGGCACCTGAAGCGAGGCCTGAAAAGAAAGGTACTCTCAGCTACATAG
>MydaTAS2R16A_NW_006296406.1:752172-751240
ATGATACCCAACCAACTCACTGTTTTCTTCATGACCATCTATCTGCTCGAGTCCTTGACAATAATTGTGCAGAGCAGCTT
AATTGTTGCGGTGCTGAGCAGAGAGTGGGTGCAGGTCAAAAGGCTGTCACCTGTGGACATGATTCTCATCAGCCTGGGCA
TCTGCCGCTTCTTACTACAGGGGACATCAGTGCTGTACAACTTTTGCTATGATTTCAACCCTGACGATTACCTTTGGTAC
ATTGCACTCATCTGGGAATTTACTAATACTCTTGCATTCTGGTTAACCAGCTTGCTAGCTGTCGTGTACTGTGTCAAAGT
CTCTTCCTTCACCTATGCCATCTTCCTCTGGCTGAGGTGGAGAATTTTGAGATTGGTTCCCCAGCTGTTGCTGGGCTCTC
TGATGATTTCCTGTGTGACAATCATTGTTTCAGCTCTTAGATTTGTCTTCATCAAGAGTCAATTGATCTCCATGATGCAA
TTACCTGGAAACAACACAGAGATGGAGACACTTACGACATTCCTGGAGAAAAATTATGCATATCAGCTTCTGGCAATGTT
GTTCATTCCTTTCCTCCTTTTCCTGACCTCCACCATCTTGCTCATAGCCTCATTGTGCCAACACTTGAGGCAGAAAAGAC
ATCACGACACTGGCCACAGCAACTCCAGCATGAAAGCTCATGCCACTGCCCTGAGGTTTCTTGCCTTCTTCCTCATCTTC
TTCACCTCTTACTTTTTTACCATAATTATCTCCACTACATATCATCTAAAGCATAAGAGTTACTGGTTCTGGGCCAGCGA
AACTATCATCTATGCTACAATCTCTATTCATTTAACTTCACTAATGCTGAGTAGTCCTGCATTGAAAAAGGTTTTAAAGG
TAAGCTGCTGTGGCCCAAAGCTGCCTGAGGCTCCAGGTAGAACAAGAACCTAA
>MydaTAS2R16B_NW_006296406.1:732271-731348
ATGATACCCAACCAACTCACTGTTTTCTTCATGACCATCTATCTGCTCGAGTCCTTGACAATAATTGTGCAGAGCAGCTT
AATTGTTGCGGTGCTGAGCAGAGAGTGGGTGCAGGTCAAAAGGCTGTCACCTGTGGACATGATTCTCATCAGCCTGGGCA
TCTGCCGCTTCTTTCTACAGTGGTCATCAGCGTTGCACAACTTTTGCTCCTATTTCAACCCTGACAATGATCTTTGGTAC
ATAGCACTCATCTGGGAATTTACTAATACTCTTGCATTCTGGTTAACCAGCTTGCTAGCTGTCGTGTACTGTGTCAAAGT
CTCTTCCTTCACCTACGCCATCTTCCTCTGGCTGAGGTGGAGAATTTTGAGATTGGTTCCCCGGCTGTTGCTGTGCTCTC
TTATTATTTCCTGTGTGACAATCATTGTTTCAGCTATTAAACATTACATCAAGAGTCAGTTAATCTTGCAATTACCTGGA
AACATCACGAAGACTGAGACACTTAGGACATTCTTGGGAAAATATTACATAGGTCAGCATCTGGCAATGTTATTCATTCC
TTTCCTCCTGTTCCTGACGTCCACCATCTTGCTCATAGCCTCATTGTGCCAACACTTGAGGCAGATACGACATCACGACA
CTGGCCACAGCAACTCCAGCATGAAAGCTCATGCCACTGCCCTGAGGTTTCTTGCCTTCTTCCTCATCTTCTTCACCTCT
TACTTTTTGACCATAATCATCTCCACTAAATACATCCTAAGGCATAAGACTTCCTTGTTCTGGGCCGGCGAAAGTATCAT
CTATGCTACAGTCTCTATCCATTTAACTTCACTAATGCTGAGTAGCCCTACATTGAAAAAGGTTTTAAAGGTAAGCTGCT
GTGGCCCAAAGCTGCCTGAGGCTCCAGGTACAGCAACACCCTAA
>MydaTAS2R16C_NW_006296406.1:746240-745344
ATGTATTTTCTACTTAGAAATTATTGCAGAGATTCTGGGTCTGTGAGCAGGAAGATACTTTGGAGTGGAAGAATGATGCC
CAAAACACTGTCACCTGTGGACATGATTCTCATCAGCCTGGGCGTCTGCCGCTTCTTTCTACAGTGGTCATCAGTGCTGT
ACAACTTTTGCTCCTATTTCAACCCTGACAATGAACTTTGGTACATAGGAATCATCTGGGAATTTTCTAATACTCTTGCA
TTCTGGTTGACCAGCTTGCTAGCTGTCGTGTACTGTGTCAAAGTCTCTTCCTTCACCTATGCCATCTTCCTCTGGCTGAG
GTGGAGAATTTTGAGATTGGTTCCCCAGCTGTTGCTGGGCTCTCTGATGATTTCCTGTGTGACAATCATTGTTTCAGCTC
TTAGATTTGTCTTCATCAAGAGTCAATTAATCTCCATGATGCAATTACCTGGAAACAACACAGAGAATGAGACACTTAGG
ACATTCCTGGAGAAAAATTACATACATCAACTTCTGGCAGCATCGTTCATTCCTTTCCTCCTGTTCCTGACCTCCACCAT

TTTGCTCATAGCCTCATTGTGCCAACACTTGAGGCAGAAAAGACATCACGACACTGGCCACAGCAACTCCAGCATGAAAG
CTCATGCCACTGCCCTGAGGTTTCTTGCCTTCTTCCTCATCTTCTTCACCTCTTACTTTTTGACCATGATCATCTCCACT
ACATATATCCTAATGCATGCGAATTCCTGGTTCTGGGCCTGTGAAACTATCATCTATGCTACAGTCTCTATTCATTTAAC
TTCACTAATGCTGAGTAGTCCTGCATTGAAAAAGGTTTTAAAGGTAAGCTGCTGTGGCCCAAAGCTGCCTGCGGCTCCGG
GTACAACAATACCCTAA
>MydaTAS2R408A_NW_006288361.1:1108860-1109837
ATGAGCAGTAATATTACGTTTGGCACTTTCTTTTCCACACTTGACTTTCCTCCCCAAGATCTGATAAGCTTACTACAATT
TTTTTTTTCCATGCTAATAATGACAGGATTTATTCTAGGAAATTTTGCCAATGGCTTCATAGCACTGGTGAACTGCATTG
ACTGGATCAAGAAACACAAGATGTCCTGTGCTGATCGAATTCTCACTGCTCTGGCTGTCTCCAGAATTGGTTTGCTCTGG
ATAATAGTATTAAACTGGTATGCAACTATGTTTAATTTACCTTTCTATAGTTCAGAAGTACGTACTACTGTTATTATTGC
CTGGATAATAAACCACCATTTTTGTCTCTGGCTTGCTACTAGCCTCAGCATACTTTATTTTCTCAAGATAGCCAATTTCT
CCTGTCTTTTATTTCTTCACCTAAAATGGAGAGCTGAAAGAGTGGTTACCATGATACTGTGGGGGAATTTGTTCATCTTG
GTTTGTCATCTTACAGTGGCAAGCATAGATGAAAAAATGCAGATGAATGATTATGAAGGAAATGCCACTTGGAAGACCAA
CTTGAGGGATATTGTGCGCCTTTCAAATATGACTGTACTCACGCTTGCAAACTTCATACCCTTAACTATGTCCCTGACAG
CTTTTCTGCTGCTAATCTTTTCCTTGTGGAAACATCTGAAGAACATGCAGCTCAGTGGCAAAGGAACTCAAGATCCCAGC
ACCGAGGTCCACATAAGAGCCATGCAAACTGTGATCTCCTTTTTCTTGCTATTTGTCATTTATTTCCTTGCTCAAACCAT
CCCAATTTGGAGGCCCAAAACTCTGCAGAATAATTCATTTCTCACTCTTTGTGAGGTTCTTGGAATCATGCATCCTTCAA
CCCACTCATTGATCCTGATTTGGGGTAACAAAAAGTTAAGACAGGCCTTTCTGTCATTTCTGTGGCAGCTGAGGTGCTGG
CTGAAGGAAAAGGAATAA
>MydaTAS2R408B_NW_006288361.1:1091520-1092434
ATGGCCTTACTACCAGCCATTCTTTCCAGCCTATTCACAATACAATTTGTTCTAGGATATTTTGCCAATGGCTTCATAGC
AGTGGTGAACTGCATTGACTGGGTCAAGAGACAAAAGATTTCCTGTGCTGATGGAATTCTCACTGCTCTGGCAGTTTCCA
GAATTGGTTTGCTCTGTGTACTACTATTAAATTGGTATGCAACTGTATTTAATCGAGCATTTTATAGTTTAGAAGTAAAA
CTTATTGTTCATATTGCCTGGATGACAAGCCACCATTTTAGTCTGTGGTTTGCTACTAGCCTTAGCATATTTTATTTGCT
CAAGATAGCCAATTTCTCCAGCTTTTTATTTCTTCACCTAAAATGGAGAGCTAAACGAGTGGTTCTCATGATACTGTTGG
GGGCTTTGGTCTTCTTGGTTTTTCGTCTTGCAGTGGTAAGCACAGATGAAAAAATGCAGATGAATGAATATAAAGGAAAC
ATCACTTGGGAGACTAACTTGAGGGACATTATGCACCTTTCAAATCACACTTTATTCATGCTTTCAAACATAGTACCCTT
TACTATGTCCCTGACAGCTGTTCTGCTGCTAGTCTTTTCCCTGTGGAAACATCTGAAGAACATGCAGCTCAGTGGCAAAG
GAACTCAAGATCCCAGCACCGAGGTCCACTTAAGAGCCATGCAAACTGGGATCTCCTTTCTCTTGACATTTGTCATTTTT
TTCTTCTCTCAAATCATCTCACTTTGGAATTCGAGTACTCAGCAGAACAACTTAGTTTACATGGTTTGCAAGGTTCTTGG
AATCCTGTATCCGTCAAGCCACTCATTTATCCTGATTTGGGGGAATAAGAAGCTGAGACAGGCCTTCCTGTCATTTCTGT
GGCAGTTGAGGTGCTGGCTGAGGAAAGGGAAATAA
>MydaTAS2R38_NW_006300374.1:237425-238432
ATGTTGACTCTGACTCCCGTCATAACTGTGTCCTATGAAGTCAAGACTGTGTTTCTGGTCCTTTCAGTCCTGGAGTTTGC
AGTGGGGATTCTGGTCAATGTCTTCATTTTCTTGGTGTATTTTCGGGATATGGTGAGGAGGCAGCCCCTGAGCACCTGTG
GTCTTGTCCTGCTGAGTCTTAGCCTCACCCGGCTTTTCCTGCATGGACTGCTGTTTTTGGATGCCATTCATCTTACTCAC
TCCCAGCGGATGAACGACCTGCTGAGCTTCAGGTACCAAACCATCATCATGCTCTGGATGATCACAAACCAAGCCAGCCT
CTGGCTCACCACCTGCCTCAGCCTCCTCTACTGCTCTAAGATCGTCCGTTTCTCTCATGCCTTCCTGCTCTGCTTGGCCA
GCTGGATCTCCAGGAAGATTTCTAGGATGCTCCTGTGTACTCTCCTTTTCACCAGTGTATGCACTATCATCTGTTTTTGG
GACTTTTTTAGTAGATCTCACTTCACAGTCACAACTGTGCTATTCATGAGTAACAATTCAGAATTCAATTTGCAAATTGA
AAACCTCAAGTTCTTTCATTCCGTCCTCTTCTGCAGTGTGGGGTCCATCCCACCTTTCTTGTGTTTTCTGCTGTCTTCTG
GGGTGCTGATTATCTCCCTGTGCCGCCACATGAGGACAATGAGGGCCAAGACCATGGACTCCTGTGACCCCAACCTGGAG
GCCCACATCAAAGCACTCAAATCCCTCATCTCCTTTCTCTGCCTCTTTGTGGTGTCTTTATGCACTGCCCTCCTCTCAGT
GCCTTTACTGGTGCTGTGGCACAACAAGATCGGGGCCATGGTCTGTGTGGGGATAATGGCAGCCTGTCCCTCAGGGCACG

CAGCCATCCTGATCTCAGGCAATGCCAAGCTGCGGAGAGCTGTGGACAGCATTCTACTGTGGGTTCAGAGCAGCCGAAGG
GTAACGGCAGACCACAAGGCAGATCCCAGGACACCAGATCTATGTTGA
>MydaTAS2R39_NW_006294067.1:273958-272999
ATGACCAACACCTGCAGTCCCCCAGAGGATAATTTGTCACCACTTAATATCATCTTAATTTTCACAGTTTTGGGCACGGA
ATGCATCATTGGCATCGTTGCAAATGGGTTCATTGTGGCTATAAATGCAGCTGAGTGGATCCAGAATAAGGCAGTCTCCA
CGAGTGGCAAGATCCTGTGTTTCCTGAGCATATCCAGAATAGCTCTCCAAAGCTTGATGATACTAGAAATTACTTTCCAC
TCAACATCCCCACAATTTTATTATAAAGATGGTGTATATGATACCTTGAAAGTGAGTTTTGTGTTCTTACATTATTGTAG
CCTCTGGTTTTCTGCCTGGCTCAGTTTCTTCTACTTTGTGAAGATTGCTGATTTCTCCTACCGCCTTTTCCTCAAGCTGA
AGTGGAGAATTACTGTATTAATGCCCTGGCTTCTGTGGCTATCAGTGTTTTTTGCCTTGGGCTACAGTATGTTCTTTTCC
TATGGCATATACACTGTTTATTGTAACAATTCTTTTCCTATCCTCTCCTCCAACTCTACTAAGAAAATATACATCACTGA
GACCAACGTGGTCAACCTGGTTCTTCTCTATAACCTGGGGATCTTCATTCCTCTCGTCATGTTCATCCTGGCGGCCACCC
TGCTGATCATCTCTCTCAAGAGGCACACCCTGCACATGAGAAGCAATGCCACTGGCTCCAGGGACCCCAGCATGGAGGCC
CACTTGGGGGCCATCAGAGCTATCAGCTACTTTCTCATTCTCTACATTTTCAATGCAATTGCTCTATTACTCTATATGTC
CAACACCTTCAATGCCAACAGTTCCTGGGATATTTTGTGCAAAGTTATCATGGCTGCCTATCCTGCTGGTCACTCCATTC
TACTGATTCAGGACAACTCTGGGTTGAAAAGAGCCTGGAAGCGGCTTCAGTCTCAAGTTCATCTTTACCTTAAAAAGTAA
>MydaTAS2R40_NW_006294067.1:256296-255343
ATGACGGTGAACACCAATTACGCGGATGAAGGCACGCCCAGGTTTAAAGTGGTCTTCATCTTGGTGGTCTCCGGAATCGA
GTGCCTCACTGGCATCATTGGGAATGGTTTCATCACGGCCATCCACGGGGCCGAGTGGGCCAGACGCAAAAGACTCCCCG
TGGGGGACCGCATTGTGCTGATGCTGAGCTTCTCCAGGCTCTTGCTGCAGATTTGGATGATGCTGGAGAATGTGTACAGC
CTGCTATCCCAGGCCACTTACAACCAAAACACAGTGTATATACCTTTCAAAGTCATCATCCTCTTTCTGAGCTACTCCAA
CCTCTGGCTCGCCGCCTGGCTCACCATCTTCTACTGTCTTAAAATTGCAAACTTTACGCACCCTTTGTTCGTCACGATGA
AGAGGAAAATCACAGTGCTGATGCCCTGGCTTCTGAGCCTGTCGCTGCTCAACTCCTTGTGCTTCAGCTTACCCTTAACT
AAAGACATCTTCAATGTGTATGTGAATAGTTCCATTCCTATCCCCTCCTGCAACGCCACAGAGAAGGCGTACATCGCTGA
GACCAACGTGGTCAACCTGGTTCTTCTCTATAACCTGGGGATCTTCCTTCCTCTCGTCATGTTCATCCTGGCGGCCACCC
TGCTGATCATCTCTCTCAAGAGGCACACCCTGCACATGAGAAGCAATGCCACTGGCTCCAGGGACCCCAGCATGGAGGCC
CACTTGGGGGCCATCAGAGCTATCAGCTACTTTCTCATTCTCTACATTTTCAATGCAATTGCTCTATTTCTTTCCATGTC
CAATGTCTTTGATGCCTACAGTTCCTGGAATATTTTCTGCAAATTTGTCATGGCTGCCTACCCTGCTGGCCATTCACTGC
TGCTGATCGTGGGCAATCCTGGGCTGAGAAGAGCCTGGAAGCGGTTTCAGCATCGAGTTCATCTTCACCTGTAA
>MydaTAS2R41A_NW_006296406.1:574538-573621
ATGCAGCCGGCATTCACAGCCCTCTTCATGCTGCTCTTTGTCCTGCTGTGTTTCCTGGGAATCCTGGCCAATGGCTTCAT
TGTGCTGGTGCTGAGCAGAGAGTGGAGGCGGCTTGGGAGGCTGCTCCCCTCTGACATGATCCTCATGAGCTTGGGTGCCT
CCCGTTTCTGCCTGCAGTGGGTTGGAATGGTGCACAACTTTTACCTCTTCTTCCACCTGGGCGAATTCAGCAAGGGTCCT
GCACGGGAGCTCTTTGGTCTCCATTGGGACTTCCTGAATTCAGCCAACTTCTGGTTTGGTACCTGGCTCAGTGTCCTCTT
CTGCATGAAGATTGCTAACCTCACCCACCCGACCTTCCTCTGGCTGAAGTGGAGGTTCCCAAGGTCAGTGCCCTGGCTTC
TGCTGGGCTCTCTCCTGATCTCCACTGTCGTCACCCTGCTCTACTTCTGGGGAAACTACGCTGTGAATCAAGGTTTCTTC
ATTAGAGAAGTTTTTGGGAATATGACCTACATGGAGAGGGTCATGAGGATGGAAATTCACTATTTCCTACCCCTCAAATT
CATCACGTTGTCGATTCCTTGCTCTGTTTTTCTGGTCGCGACTGCATTGTTGATTCATTCTTTGAGGAGACACGCTCGGA
CAATGCGGCAAAGTGCCCACAGCCTGCAAGACGCCAGCACCCAGGCTCACACCAGAGCTCTGAAGTCACTCATCTTCTTC
CTCATTCTTTACATTCTGTCTTTCATGTCCCTGATCATTGATGCTGTAGGCTTCTTTTCAACAGAGAATGACTGGTTCTG
GCCATGGCAAATTGTAACCTACCTGTGCACATCTGTTCATCCCTTTGTCCTCATCCTCAGCAACCTCAGGCTTCGAGAGG
TTTTCAGGACGCTACTTCTGTTGGGCAGGGGCTTCTAG
>MydaTAS2R41B_NW_006294067.1:4363-3437
ATGCAGCCAGCATTCACAGCCCTCTTCATGCTGCTCTTTGTCCTGCTGTGTCTCCTGGGAATCCTGGCCAATGGCTTCAT
TGTGCTGGTGCTGAGCAGAGAATGGAGGCGGCTTGGGAGGCTGCTCCCTTCTGACATGATCCTCATTAGCTTGGGTGCCT

CCCGTTTCTGCCTACAGTGGGTTGGAATGGTGCACAACTTTTACGCCTTCTTCCACCTGGAGGAGTTCAACAAGGGTCTT
GCAGGGCAGCTCGTTAGACTCCAATGGGACTTCCTGAATTCAGCCACCTTCTGGTTCGGTACCTGGCTCAGTGTCCTCTT
CTGCATGAAGATTGCTAACCTCACCCACCCTACCTTCCTCTGGCTGAAGTGGAGGTTCCCAGGGTCAGTGCCCTGGCTTC
TGCTGGGCTCTCTCCTGACCTCCACCATCGTCACCCTGTTCTTCTTTTGGGGAGACTACACTTTGAATCAAGGTTTCTTT
ATTAGAGAAGTTTATGACAATATGACCTACATGGAGAGAGTCATGAGCATGGAAATTCACTATTTCCTACCCCTCAAATT
TGTCTTGTTTTCAATTCCTTGCTCTGTTTTTCTGGTCTCGACTGCATTGTTGATTCATTCTTTGAGGAGACACGCTCGGA
CAATGCGGCAAAGTGCCCACAGCCTGCAAGACGCCAGCACCCAGGCTCACACCAGAGCTCTGAAGTCACTCATCTTCTTC
CTCATTCTTTACATTCTGTCTTTCATGTCCCCGATCATTGATACTGTAGGTTTCTTTTCCTCAGAGAATGACTGGTTCTG
GCCATGGCAAATTGTAACCTACCTGTGCACCTCTGTCCATCCCTTTATCCATCTCCTCAGCAGCCCCAGGCTTCGAGCGG
TGTTCAGGCAGCTACTTCTGTTGGCCAGGGGCTTCTGGCTGGTCTAG
>MydaTAS2R42_NW_006288361.1:1070168-1071163
ATGCTCACTGGATTGGAAATAATCTTTCTGGTACTGTCAATAGCAGAATTCATAATTGGAATGTTGGGGAATGTGTTCAT
TGGACTAGTAAACTGCTCTGAATGGGTCAAGAACCAAAACATCTCTTTAGCTGACTTCATCTTTACCTGCTTGGCTATCT
CCAGAATTAGTCAGTTGTTGGTATTACTTTTTGAATCACTTACATTGGGACTATTTTCACATGTATTTTCCACTTATAAA
CTAGCAAAATCTATTACTTTACTTTGGAGAATAACTAATCACTTGACTACCTGGCTTGCTACTTGCCTAAGCATTTTCTA
CCTCCTTAAGATAGCTCACTTCTCCCACTCTCTTTTCCTCTGGCTGAAGTGGAGAATGAAGAGAGTGATTCTTGTGATAT
TTGTATTTTCTTTTATTTTTCTGATTTTTGACATTCTATTGTTAGAAAGCTTTAACGATTTATTCTTGAAGGTCTGTATA
TATGATAATAGTAATCTGACTTTATATATAGAAGAAAGTAAGACTCTCTATTTTGAAACCCTGATTCTTCTTAGCTTGAC
CTGTTTGCTTCCTATTGTTCTGTCCCTGACCTCATTGCTCCTTTTATTTCTGTCTTTGGTAAGACATATCAGAAATTTGC
AGCTCAACTCCATGGGCTCAAGGGACTCGAGCACAGAGGCCCATAAAAGGGCCATAAAAATGGTGATGTCTTTTCTCTTC
CTCTTCATATTTCATTTTTTTTCCACACAAGTGGTAAATTGGATATTTCTTATGTTTCCAAATCACACAATTGTAAAATT
TATCATGGTATTAGTCTATGTCTTTCCCTCAAGTCACTCACTTCTTTTGATTCTGGGAAACAGCAAGCTAAGACAGACAG
CCTTGAAGGTACTATGGCATCTCAAAACCTCCTTGAAAAGAGAAAATCTGTTACACCTTTACAGACAGATTTCCCAGAGT
CTTTTCAAAGATAATAACTTAATAAGGAAACTTTGA
>MydaTAS2R408C_NW_006288361.1:1080479-1079565
ATGGCCTTAATACCAGCCATTCTTTCCAGCCTATTGACAATACAATTTGTTCTAGGAAATGTTGCCAATGGCTTTATAGC
AGTGGTGAACTGCGTTGACTGGGTCAAAGGACAAAAGATCTCCTGCGCTGGTGGAATTCTCACTGCTCTGGCAGTTTCCA
GAATTGCTTTGCTCTATGTATTAGTAATAAATTGGTATGCAACTGGATTTAATCAAGCATTTTATAGTTTAGAAGTAAGA
CATATTGTTCATATTTTTCATATAATAACCCACCATTTTAGTCTGTGGTTTGCTACTAGCCTTAGCATATTTTATTTGCT
CAAGATAGCCAATTTCTCCAGCCTATTTTTCCTTCACCTAAAATGGAGAGCTAAAAAAGTGGTTCTCATGATACTGTTGG
GGAGTTTGGTCTTTTTGGTTTGTCATCTTACGGTGCTAAGCATAGATGAAAAAATGCAGATGAATGAATATAAAGGAAAC
ATCACTTGGGAGACCAACTTGGGGGACATTATGCACCTTTCAACCCATTCTTTATTCATGCTTGCAAACTTCATACCCTT
TACTATATCCCTGACGGCTGTTCTGCTGTTAATCTTTTCCATGTGGAAACATCTGAAGAACATGCAGCTCAGTGGCAAAG
GAACTCAAGATCCCAGCACCGAGGTCCACATAAGAGCCATGCAAACTGTGATCTCCTTTCTCTTGACATTTGTCATTTAC
CTCTTCGCTCATATCATCTCACTTTGGAATTCGAGTACTCAGCGGAACAATTTAGTTCACATGGTTTGCAAGGTTCTTGG
AATCCTGTATCCGTCAAGCCACTCATTTATCCTGATTTGGGGGAATAAGAAGCTGAGACAGGCCTTCCTGTCACTTCTGT
GGCAGTTGAGGTGCTGGCTGAGGAAAGGGAAATAA
>MydaTAS2R408D_NW_006288361.1:1123910-1124824
ATGATACATTTATTACCAAGCATTCTTTCCATCCTTATAATAGCTCAATTTGTTCTGGGAAATTTTGCCAATGGCTTCAT
AGCACTGGTGAGCTGCATTGACTGGGTCAAGAAACACAAGCTCTCCTGCGCTGATCGAATTCTCACTGCTCTGGCTGTCT
CCAGAATTAGTTTGCTCTGGGTAATAGTATTCAATTGGTATGGAACAGTATTGCATCCAGCGAGCTATAGTTCAGAAGTA
AGAGCTATTGTTTATATTGCCTGGGTAGCAAGCAACCATTTTTGTCTCTGGCTTGCTACTAGCCTCAGCATACTTTATTT
GCTCAAGATAGCCAATTTCTCCTGTCTTTTATTTCTTCACCTAAAATGGAGAGCTGAAAGAGTGGTTATCATGATACTGT
GGGGGACTTTGGTCTTCTTGGTTTTTCATTTTGCAGTGGTAGGCATAGATGAAAAAATGAAGATGAACGTATATAAAGGA

AACATCACTTGGGAGAGCAAATTGAGGGACATCGCACACCTTTCAAATGGGATTATATTTGTGCTTGTACACCTCATACC
CTTCACTGTGTCCCTGACAGCTGTTCTGCTGCTAATCTTTTCCATGTGGAAACATCTGAAGAAGATGCAGGTCAGTGGCA
AAGGATTCCAAGATCCCAGCACTGAGGTCCACTTAAGAGCCATGCAAACTGTGATCTCCTTTCTTTTGATATTTGTCATT
TACTTCTTCGCTCAAATCATCTCATTTTGGAATTTTACTATGCAGAACAATTCAGTTCCCTTGCTTTGCCAAGTTCTTGG
AATCCTGTATCCATCGAGCCACTCATTTATCCTGATTTGGGGGAACAAGAAGCTGAGACAGGCCTTTCTGTCACTTCTGA
GGCAGTTGAGGTGCTGGCTGAAGGAAAGGAAATAA
>MydaTAS2R408E_NW_006288361.1:1102138-1103067
ATGATGAGTTTATTCCCGAGCATTCTTTCCACACTAGTTATAGCAGGATTTGTTCTAGGAAACTTTGCCAATGGCTTCAT
AGCACTGGTGAACTGCATTGACTGGGTCAAGAGACAAAAGATCTCCTGTGCTGATCGAATTCTCACTGGTCTGGCGGTGT
CCAGAATTGGTTTGCTCTGGGTAATAATATTCCATTGGTATGCGAATCTGTTTAATCCAGCTTTGTATAGTTTAAGAGTA
AGAACTGTTGCTGCTATTGTCTGGGTAGTAAGCAACCATTTTAGCCTCTGGCTGGCTACCAGCCTCAGCATATTTTATTT
GCTCAAGATAGCAAATTTCTCCAGCCTATTTTTTCTTCACCTAAAATGGAGAGCTAAAAGAGTGGTTCTCATGATACTGT
GGGGGAGTTTGGTCTTCTTGGTTTTTCGTCTTGCAGTGTTAAACATCGATGAAGAAATGAAGATGAGTGAATATAAAGGA
AACATCACTTGGAAGACCAACTTGAGGAACATTATAAACCTTTCAAATTTGACTATATTTACACTTGTAAACTTCATACC
CTTTTCTGTGTCCCTGACAGCTTTTCTGCTGTTGATCTTTTCCCTGTGGAAACATCTCAAGAGGATGCAGCTTAGTGGTA
AAGAAACTCAAGATCTCAGCACCAAGGTCCATGTAAGGGCCATGCAAACTGTGATCTCCTTTCTCTTGCTATTTGCCATT
TACTTTGTGACTCTAATCATCTCAGTTTGGAGTTTCTATAATCCTCAGAATATACCAGTTTTTCTGTGTTTCCAGGTTTT
GGCACTTGCCTATGTTTCAGGCCACTCACTAATTCTGATTTGGGGAAACAAGAAGCTAAATCAAGACTTTCTCTCAGCTT
TATGGCAGGTGAGATGCTGGTTGAAAGAATGGAAACCTTCAACACTGTAG
>MydaTAS2R18A_NW_006286849.1:41737-40808
ATGTCCATTGGAATAAAAGTCTCCTTTCTGGTTGTGGCAACAGGAATATTCATCTTAGGAGTGCTAGGAAATGGATTCAT
CGGACTGGTGAACTGCACTGAGTGGGTCAAGACTGGGAAAGTGTCCTTAGCTGATTTCATCCTCACCAACTTGGCTCTGG
CCAGAATCATCCATCTGTTGCTAACACTATTGGATTCATTTATAGTAGGGCTGGCTCCACATCTGTATGCTACTGGTAAA
CTACTAAAGGTGGTTAGTGTACTTTGGGCCCTAACTAACCATTTAACTATCTGGTTTGCCACCTGCCTCAGCATTTTCTA
CTTCCTTAAGATAGCCAATTTCTCCTACCCCTATTTCTTGTGGCTGAAGTGGAGAGTCAACAGGGTGGTTCTTCTGCTTT
TCCTGGGGTCTCTCTTCCTATTCTCTTTAAATATGTTTATGCATAATGCTGCTAGTAAATATTGGTTGAATACCTACAAG
GTTTATGAAATAAATATAAGTTTACAGTTAGAGGAAAATGAAATTTTCTATCTTAAAAGTCTTCTTCCTCTTAGTTTCAC
CTATGTTATCCCCTTATTCCTATCCCTGACTTCTTTGCTTCTTTTATTTCTGTCCTTGGTGAGACATACCAAGAATTTTC
AGCTCAACCTGACAGTGTCAGGCGACTCAAACACAGAGGCCCATAGAAGGGCCATGAAAATGGTGACAGCCTTCCTCCTC
CTCTTTATAATTTACATTATTTCTATTGTAACAGCAAGTTGGATTTCCACTAAGGTACTGACATTTCAGGTCAAGATGCT
TGTCATGATGATTTCAGCTACCTTTCCCTCGGGCCACTCTTTTATTATAATTTTGGGAAACAGCAAGCTAAGACAGATCG
CCTTGAGACTGGTCTGGCACTTAAATTCTCTGAGAAAAGCAAAACGTTAA
>MydaTAS2R18B_NW_006286849.1:32933-31989
ATGTCCATTGTAACAAAGGTCTCCATTCTAGTTGTGGCAACAGGAATACTCATCTTAGGAGTGCTAGGAAATGGATTCAT
CGGACTGGTGAACTGCATCGAATGGGCCAAGACTGGTGAAGTTTCTTCAGTTGATTTCATCCTGACCAGCTTGGCTATGG
CCAGAACCATTCAACTGTTGCTAATTCTATTGGATTCATTTATAGTAGGGCTGGCTCCACATCTGTATGCTACTGGTAAA
CTAGCAAAGGTGGTTACTATTTTTTGGGCACTAACTAATCAACTGACTATCTGGTTTGCCACCTGCCTCAGCATTTTCTA
CTTCCTTAAGATAGCCAATTTCTCCCACCCCTTTTTCATGTGGCTGAAGTGGAGAGTCAACAGGGTGGTTCTTCTGCTTT
TCCTGGGGTCTTTCTTCCTACTGTCTCTCAATATCTTAATGCATGATGCTATTTGTGAATTGTGGTTGAGTACCTACAGG
GTACATGAAATAAATACGACTTTGCAGTTAGAAGTAAATGAAATTTTCTATCTCAAAAATCTTCTTCCTCTTACTTTGAC
CTATGTTATTCCCTTATTCCTATCCCTGATCTCTTTGCTTCTTTTATTTCTGTCCTTGGTGAGACACACCAAGAATTTTC
AGCTCAATCTGACGGGCTCAAGCACAGAGGCCCATAGAAGGGCCATGAAAATGGTGACAGCCTTCCTCCTGCTCTTCATC
ATTTACATTATTTCTATTCTAACAACATGTTGGATCTTCACTAATTTACAGACATTTCAGGTCAAGATGTTTGTCATGAT
GATTTTGATTACCTTTCCCTCAGGCCACTCATTTATTATAATTTTGGGAAACAGCAAGCTAAGACAGATCGTCTGGAGAC

TACTCAGGCACTTCAATTCTCTGAGAAAAGCACAACTTTCACTTTATGAACAGAATCTGAAATAA
>MydaTAS2R18C_NW_006286415.1:988-65
ATGTCCATTGAAATAAAGGTCTCTATTCTAGTTGTGGCAATGGGAATACTCATCTTAGGAGTGCTAGGAAATGGATTCAT
TGGACTGGTGAACTGCATAGAATGGTTCAGGACTGGGAACGTTTCCTCAGCAGATTTCATCCTCACCAGCTTGGCTCTGG
CCAGAATCATTCAACTGTTGGTAATACTCTTGGATTCATTTATAATGGGGCTGGCTCCACATCTGTATGCTACTGGTAAA
TTAGCAAAGGTGGTTAGTATTCTCTGGGCACTAACTAACCACCTAACTATCTGGTTTGCCACCTGCCTCAGCGTTTTCTA
CTTCCTTAAGATAGCCAATTTCTCCCACTCCTTTTTCATGTGGCTGAAGTGGAGAATCAACAGGGTGATTCTTCTGCTAT
TCCTGGGGTCTTTCTTCCTACTGTCTCTTAACCTCGTAATGCATGATGCTGTTAATGATCTGAATACTTACAGGGTACAT
GAAATAAATATGACTTTGAAGTTAGAGGCAAATGAAATGTTCTATCTCAAAAGTCTTCTTCTTCTTAGTTTGACCTACGT
TATCCCCTTTTTCCTGTCCCTGATCTCTTTGCTTCTTTTATTTCTGTCCTTGGTGAGACACACCAAGAATTTTCAGCTCA
CTCTGACGGGCTCAAGCACAGAGGCCCATAGAAGGGCCATGAAAATGGTGACCACGTTCCTCCTCCTCTTCATCATTTAC
ATTATTTCTATTCTTACAGCAAGTTGGATCTTCACTACGGTAGAGACCTATCAAGTCAAGATGCTTGTCACAGTGATTTC
AACTACCTTTCCCTCAGGTCACTCTATTCTTATTATTTTTGGAAACAGCAAGCTGAGACAGATCGCCTTGAGGCCACCGT
GGCACATAAATTCTCTGAGAAAAGCAAAACCTTTGCCTTTATAG
>MydaTAS2R18D_NW_006298959.1:2018-1107
ATGTCCATTGGAATAAAGGTCTCCATTCTAGTTGTGGCAATAGGAATACTCATCTTAGGAGTGCTAGGAAATGGATTCAT
CGGATTGCTAAACTGCATCGAATGGTTCAGGACTAGGAAAGTTTCCTCAGCTGATTTCATCCTCACCAGCTTGGCTCTGG
CCAGAATCATTCAACTGTTGGTAGCACTATTGGATTTATTTATAATGGGGATATCTCCACATCTGTATGCTACTGGCAAA
CTAGCAAAGGTGGTTCCTATTCTTTGGGCACTAACTAACCACCTAACTATCTGGTTTGCCACCTGCCTCAGCATTTTCTA
CTTCCTTAAGATAGCCAATTTCTCTCACCCCTTTTTCATGTGGCTGAAGTGGAGAGTCAACAGGGTGGTTCTTCTGCTTT
TCCTGGGGTCTTTCTTCCTACTGTCTCTTAACCTCTTAATGCGCGTTGTTCCTAGTGAACTGTGGTTGTCTACTTACAGG
GTACATGAAATAAATATGACTTTGCAGTTAGAGGCAAATGAAATGTTTCTATTAAATCTCTTCTTATTATTACTTTGAC
CTATATTATCCCCTTTTTTCTGTCCTTGATCTCTTTGCTTCTTTTATTTCTGTCCTTGGTGAGACACACCAAGAATTTTC
AGCTCAACCTGACGGGCTTGAGAGACTCAAGCACAGAGGCCCATAGAAGGGCCATGAAAGTGGTGACAACGTTCCTCCTC
CTCTTCATCATTAACATTATTTCTATTCTAACTGCAATTTGGATCTTCACTAAGATACAGACATATGAGGTCATGATGCT
TGTCACAGTGTTGTCAGCTACCTTTCCATCAGGCCACTCTTTTCTTATAATTTTTGGAAACAGCAAGCTAAGACAGATCG
CCTTGAGACTACTGTGCACTTACAATCCCTGA
>MydaTAS2R67_NW_006288361.1:1083319-1084293
ATGCCATCTGGAGCTGAAAGTCTTTTCCTGGTAGCTGCAATGGGAGAATTCATAGCCGGAATGCTGGGGAATGGGTTCAT
TGTACTAGTTAATTGCATTGACTGGGTGAAGAGTCAAAAACTCTCAGCAGCTGACTGCATCCTCACCAGCCTGGCTCTCT
CCAGAATCACTCTTCTTTGGATATCACTAGCTGACTCGTTTCTAATGGTGTTGTGGCCACATTTTTATGCCATTGATAAA
CTAGCAAAACTTATTGGCATTCTTTGGATACTGTGCAATCACCTAGCTACCTGGTTTGCCACCTGTCTAAGCATTTTCTA
TTTCTTTAAGATAGCCAATTTCTCCCACCCCTGCTTTGCCTGGCTGAGGTGGAGAATTGGCAAAGTGCTACTTGTGCTTT
TACTGGGGTCTTTGTTCTTCCTGTCTTTGAAACTTGTGTTAATAGACTCATTCAATGGTTTCTGGATTAAGGTCTATAAT
ATACATGAAAGAAACTCAACATGGACTCCAGGTGTAAGTGAAACTCTGTATCTTAACAGTTTGATTGTTTCTAATTTTAT
CTACTTAATTCCCTTTCTTCTGTCCCTGAGTTCACTGCTCCTTTTATCTCTTTCCTTGATGAGACATACCAGGAATGTGG
GCACGAACTCCAGCTCTAAGGACTTCCGCACAGAAGCCCACAAAAAGGCCATGAAGATAGTGTTATCTTTCCTCCTCCTC
TTCATACTTCATTTTTCTTCCTTTGTATTAACGGGTTGGTGTTTCCTTATAGTGCAGAAGCAGCACGCCAATCTGGCTGT
CATGTTAACATGGAGTATTTTCCCTTCGGGCCACTCATTTATCCTCATTTTGGGAAACAGCAAGCTGAGGCAAACTGCGT
TGGGACTATTGTGGCATCTTAACTGCCACCTGAAAAAGGGTGAAATCTTAGCTTCACAGACTTTACCAGGATTGTCTATA
TTCCAGGAAAATTAA
>MydaTAS2R5P_NW_006300374.1:321827-320925
ATGCATATTGCCACCCTAGGACTGCTGATGGTGGTGGCAGTGACCGAATTTCTCATTGGCCTGGTTGGAAATGGAGTCCT
TGTGGTCTGGAGTTTTGTAGAATGGGTAAGAAAATGCAAGGAGTCCTCCTACAAACTCATTGTCCTGGGCCTGGCTGGCT

GCTGACTTCTCCTGCAGTGCCTGATTATGGTGGACCTAATACTGTTTTCAATTTTCAAGAGCTGCATCTGGTTTCGCTAT
ATCAGTGTCTTCTGGGTTGTGGTCAGCCAGGCCAGCCTGTGGTTTGCCACTTTCCTCAGTGTCTTCTACTGCAAGAAGAT
CATGACCTTTGAACACCCTGTCTACCTATGGCTGAAGCAGAGGGCATATAGCCTGAGTGCCTGGTGTCTTCTGGGGTGCC
TCCTGATCAATCTGCTAATTATAGCCGATGTTGGCTTAAAGCCCCATAGTCCTTTCCAAGGAAACAGCAGCATTCTGTAC
TCCCTTTCAGATTGGCAGTATCTGTATATATTACAGCTCAATGCAGGAAGTGGGTTTCCTTTCTTGGTGTTTCTAATTTC
CTCTGGGATGTTAATGGTCTCTCTGTATAGACATCATAAGAAGATGAAGGTCCATACAGCTGGCCGGAATGATGCTCGAG
CCAAGGCTCACATCACTGTCCTGAAGTCCTTGGTCTGCTTCCTTATACTTTACTTGGTTTACATCGTGGCCAGCCCCTAC
TCTATCAAATCCAAGACTTCTCCTGTTGATCTCACCACTATCTTCATCTCGGAGACAGTCATGGCTGCCTATCCTTCTCT
TCATTCTGTCTTATTGATCATGGGGAATCCCAGGATAAAGCAGGCTTGTCAGAGAATCCTGTGGAAGACAGCGGGCCGTG
CTTGGAAGTCCTGAGGCTTGTGA
>MydaTAS2R13P_NW_006286849.1:30427-29999
ATGGTGAGGGCCTTGCATTGCTTCTTCACCATTTCAGTAAACACAGAATTTGTCATTGGTATTTTGGGAAATGGGTTCAT
AATACTGTGAACTATATTGACTGATTCAAGAAACAAAAGACCTTTTCAGTTGACTGAATCCTCACTGCTTTGGCGCTCTC
CAGACTTTGTCTGATTTGGGTAATAATGATGAGTTAGTTTGCAAAGGATATTTATCCATCTTCACAGATGAATATAATGG
AAGTTGTATTTATTATTCTTGTGGTTGCAGCCAATCATTTTAGTGACTGGTTTGCCACAGGCCTCAGCCTCTTTTATTTC
TCAAGATAGCCAGTTTCTCAAATCCTGCTTTTCTTTGCCTGAAGCGTAGGTTGAAATGGTAGTTCTGATAATGTTGCTGG
GAGCATTACTGTTCTTACCTTTAAATCTT
>MydaTAS2R18EP_NW_006291053.1:6490-7263
ATGCCAATTGAAATAAAGGTCTCCATTCTAGTTGTGGCAACAGGAATATTCATCTTAGGAGTGCTAGGAAATGGATTCAT
CGGACTGGTGAACTGCATCGAATGGTTCAGGACTGGGAACGTTTCCTCAGCTGATTTCATCCTCACCAGCTTGGCTATGG
CCAGAATCATCCAACTGTTGGTAATGCTGTTGGATTCATTTATACTGGGGCTAGCTCCACATCTGTATGCTACTGGCAAA
CTAGCAAAGGTGGTTCCTATTCTTTGGGCACTAACTAACCACCTAACTACCTGGTTTGCCACCTGCCTCAGCGTTTTCTA
CTTCCTTAAGATAGCCAATTTCTCCCATTCCTTTTTCATGTGGCTGAAGTGGAGAGTCAACAGGGTGGTTCTTCTGCTTT
TCCTGGGGTCCATCCCCTTTTTCCTGTCTCTGATATCTTTGTTTCTTTTATTTTTGTCCTTGGTGAGACACACCAAGAAT
TTTCAGCTCAACATGATGGGTTCGGTGGACTCAAGCACAGAGGCCCATAGAAGTGCCATGAAAATGGTGACAACGTTCCT
CCTCCTCTTCATCATTTACTTTATTTCTATTCTAACTGCAATTTGGAGCTCCACTAATGTAGAGACCTATCAAATCATGA
TGCTTGTCATAGTGTTGTCATCTACCTTTCCCTCAGGCCACTCTTTTCTTATAATTTTTGGAAACAGCAAGCTAAGACAG
ACTGCCTTGAGACTACTCTGGCAGTTATATTCCCTGAGAAAATCAAAAGTTTAA
>MydaTAS2R18FP_NW_006288361.1:1128723-1129391
ATGTCAATTGAAATAAAGGTCTCCATTCTAGTTGTGGCAACAGGAATACTCATCTTAGGAGTGCTAGGAAATGGATTCAT
CGGACTGGTGAACTGCATCGAATGGGTCAAGACTGGGAAAGTGTCCTTAGCTGATTTCATCCTCACCAGCTTGGCTCTGG
CCAGAATCATCCATCTGTTGGTAACACTATTGGGTTCATTTATAGTAGGGCTAGCTCCACATCTGTATGCTACTGGTGAA
CTACTAAAGGTGGTTAGTGTACTTTGGGCACTAACTAACCATTTAACTATCTGGTTTCCCTGATCTCTTTGCTTCTTTTA
TTTCTGTCCTTGGTGAGACACACCAAGAATTTTCAGCTCAACCTGACGGGCTTGAGGGACTCATGCACAGAGGCCCATAG
AAGGGCCATGAAAATGGTGACCACGTTCCTCCTCCTCTTCATCATTAACATTATTTCTATTCTAACTGCAGGTTGGATCT
TCACTACGGTACAGACCTATCAAGTCATGATGCTTTTCTTAGTGATTTCACCTACCTTTCCCTCAGGCCACTCTTTTCTT
ATAATTTTTGGAAACAGCAAGCTGTAATAGATTGCCTTGAGACTGCTGTGGCACTTAAATTCTCTGAGAAAAGCAAAACC
TTTGACTTTATAGACAGAATAAGAAATAA
>MydaTAS2R18GP_NW_006288361.1:1147149-1147754
ATGTCCATTGGAATAAAGGTCTCCATTCTGGTTGTGGCAATAGGAATACTCATCTTAGGAGTGTTAGGAAATGGATTCAT
CGGATTGCTAAACTGCATCGAATGGTTCAGGACTAGGAAAGTTTCCTCAGCTGATTTCATCCTCACCAGCTTGGCTCTGG
CCAGAATCATTCAACTTTTAGTAATACTGTTGGATTCATTTATGAGACTGACATGCTGCCTGCTGTGCTAAGAGGGTCCC
TACTGTTGGATTCATTTTTAATGGGGCTAGCTCCACATCTGTATGCTACTGGCAATTCTTGGATTCATTTATGAGACTGA
CATGCTGTCTGCTGTGCTAAGAGGGCCCCTACTTTTGGATTCATTATAATGGGGCCAGCTCCACATCTGTATGCTACTGG

CAAACTAGCAAAGGTGGTTCCTATTCTTTGGGCACTAACTAATCACCTAACTACCTGGTTTGCCACCTGCCTCAGCGTTT
TCTACTTCCTTAAGATAGCCAATTTCTCCCATTCCTTTTTCATGTGGCTGAAGTGGAGAGTCAACAGGGTGGTTCTTCTG
CTTTTCCTGGGGTCTTTCTTCCTTCTGTCTCTTAACCTCTTAATGC
>MydaTAS2R18IP_NW_006286849.1:56143-55501
GTGTCATTTGAAATGAAGGCCTCCTTTACCGTTGTGGCAATACGAATACTCATCTTAGGAGTGCTAGGAAATGGATTCAT
CGGACTGGTGAACTGCATCGAATGGTTTAGGACTGGGAACATTTCCTCAGCTGATTTCATCCTCACCAGCTTGGCTCTGG
AATACTGACAGAGTACATGAAATAAATATGACTTTGCAGTTAGAGGCAAATGAAATGTTCTATATCAATTGTCTTCTTCT
TCTTAGTTTTACTTATGTTATCCCCTTTTTCCTGTCTCTGATCTCTTTGCTTCTTTTATTTCTGTCCTTGGTGAGACACA
CCAAGATTTTTCAGCTCAACCTGAAGGGCTTGAGGGACTCAAGCACAGAGGCCCATAGAAGGGCCATGAAAGTGGTGACA
ATGTTCCTCCTCCTCTTCATCATTTACATTATTTCTATTCTAACTGCAACTTGGATCTTCACTAAGGTACAGACATACTA
AGTCATGATGCTTGTCAGTGATTTCAACTACCTTTCCCTCAGGCCACTCTTTTCTTAAAATTTTTGGAAACAGCAAGCTA
AGACATTGCCTTGAGGCCACTGTGGTACGTAAATTGCCTGAGAAAAGGAAAACCTTTGCCTTTATAGACAGAATTTGAAA
TAA
>MydaTAS2R18JP_NW_006291053.1:4876-5583
ATGTCATTTGAAATGAAGGCCTCCTTTCTGGTTGTGGCAACAGGAATATTCATCTTAGGAGTGCTAGGAAATGGATTCAT
CGGACTGGTGAACTGCGTCGAATGGTTCAGGACTGGGAACGTTTCCTCAGCTGATTTCATCCTCACCAGCTTGGCTCTGG
CCAGAATCATTCAACTGTTGGTAATACTGTTAATGCATCATGCTGTTAATGAATTGTGGTTGAATGCGTACAGGGTACAT
GAAATAAATATGACTTTGCCGTTAGAGGCAAATGAAATGTTCTATCTCAAAAGTCTTCTTCTTCTTAGTTTGACCTATGT
TATCCCCTTTTTCCTGTCCCTGATCTCTTTGCTTCTTTTATTTCTGTCCTTGGTGAGACACACCAAGAACATGCAGCTCA
ATGTGACGGGCTCGGTGGACTCAAGCACAGAGGCCCATAGAAGCGCCATGAAAGTGGTGACAACGTTCCTCCTCCTCTTC
ATCATTTACATTATTTCTGTTCTAACTGCAACTTGGAATTCCTCTAATGTACACACATATCAGGTCATGATGCTTGTCAC
AGTGTTGTCATCTACGTTTCCCTCAGGCCACTCTTTTCTTATAATTTTTGGAAACAGCAAGCTAAGACAGATCGCCTTGA
GACTACTCTGGCAGTTACATTCCCTGAGAAAAGCACAAGTTTAACTTTATAGACAGAAATTGAAATAA
>MydaTAS2R7AT_NW_006376196.1:320-1
ATGTCAGATGAAGTAATCAACACCTTAATGATTATAACAGTTGGGGCGTTCTCAGTGGGGCTCTTAGGAAATGCATTTAT
TGTATTGGTAAACTTCATGGACTGGATGAAGAATAAGAAGATTGCTTCCATTGATTTAATCCTCACAAGTCTGGCCATAT
CCAGAATTTGTCTGATGTGTATAATAACGTTAGATGGTTTTATGTTGGTGTTGGATCCCGATGTCTATGCCACTGGTAAG
CAAATGAGAATCATTGACTTCTTCTGGACACTAACCAACCATTTAAGTATCTGGTTTGCCACCTGCCTCAGCATTTTCTA
>MydaTAS2R7BT_NW_006377440.1:1262-615
TGGTTTGCCACCTGCCTCAGCATTTTCTATTTCTTCAAGATAGCTAACTTCTTCCATCCCCTTTTCCTCTGGATGAAATG
GAGAATTGAAAGGGTGATTCCTGGGATCCTGCTGGTGTGCGTGGCCTTCGCTGTGTTCATTAGCCTTCCTGCTGCTGAGG
ATTTGAATGATGACTTCAGGCTTTGTGTCAGGGCGAAGTGGAAAACAAACTTAACTTTGAGATGCAGGGTAAATAAAGCT
CAATATGCTTCCAGCAAGGTATATCTCAACCTGTTCACGCTGTTCCCCTTCTCTGTGTCCCTCATCTCATTCCTCCTCTT
GATCCTCTCCCTATGGAGACACATCAGGCGGATGCAGCTCAATGCCACAGGGTGCAGAGACCCCAGCACAGATGCCCACA
TGGTAGCCATGAAAGCTGTCATCTCCTTCCTCCTACTTTTCATTGCCTACTATTTGTCCTTTCTCATAGCCACCTCCAGT
TACTTCATGCCAGAGACTGAATTAGCTGTGCTGATTGGTGAACTGATAGCTCTAATCTATCCTTCCAGCCATTCATTTAT
ATTAATTCTGGGGAACAAGAAATTAAAACAAGCATCTCTAAGGGTGCTATATAAAGTAACACATACACAAAAAAGAAGAA
ATTTCTAA
>MydaTAS2R41CT_NW_006290031.1:2780011-2779604
ATGCAGCCGGCATTCACAGCCCTCTTCATGCTGCTCTTTGTCCTGCTGTGTTTCCTGGGAATCCTGGCCAATGGCTTCAT
TGTGCTGGTGCTGAGCAGAGAGTGGAGGCGGCTTGGGAGGCTGCTCCCCTCTGACATGATCCTCATGAGCTTGGGTGCCT
CCCGTTTCTGCCTGCAGTGGGTTGGAATGGTGCACAACTTTTACCTCTTCTTCCACCTGGGCGAATTCAGCAAGGGTCCT
GCACGGGAGCTCTTTGGTCTCCATTGGGACTTCCTGAATTCAGCCAACTTCTGGTTTGGTACCTGGCTCAGTGTCCTCTT
CTGCATGAAGATTGCTAACCTCACCCACCCGACCTTCCTCTGGCTGAAGTGGAGGTTCCCAGGGTCAGTGCCCTGGCTTC

TGCTGGGC
>MydaTAS2R18T_NW_006286849.1:53012-52596
GCAAATGAAATGTTCTATACTAAATGTCTTCTTCCTATTACTTTGACCTATGTCATCCCCTTTTTCCTGTCTCTGATATC
TTTGTTTCTTTTATTTCTGTCCTTGGTGAGACACACCAAGAATTTTCAGCTCAACCTGACGGGCTTGAGGGACTCAAGCA
CAGAGGCCCATAGAAGGGCCATGAAAATGGTGACCACGTTCCTCCTCCTCTTCATCATTTACATTATTTCTATTCTAACA
GCATTTTGGATCTTTACTAAGGTAGAGACATATCAGGTCATGATGCTTTTCATAGTGTTGTCAGCTACCTTTCCGTCAGG
CCACTCTTTTCTTATAATTTTTGGAAACAGCAAGCTAAGACAGATCGCCTTGAGACTACTGTGGAACTTACATTCCCTGA
GAAAAGCACAAGTTTAA
>MydaTAS2R18HT_NW_006288361.1:1136905-1137102
ATGTCAATTGAAATGAAGGCCTCTTTTCTGGTTGTGGCAACAGGAATATTCATCTTAGGAGTGCTAGGAAATGGATTCAT
CGGACTGGTGAACTGCGTCGAATGGTTCAGGACTGGGAACGTTTCCTCAGCTGATTTCATCCTCACCAGCTTGGCTCTGG
CCAGAATCATTCAGCTGTTGGTAATACTCTCGGATTCA
>CafaTAS2R1_chr34:4537632-4538525
ATGTTAGAGTTTTACCTTATTATCCATTTTCTTTTCACAGTGATGCAATTTCTCATCGGGGTTTTAGCAAATGGCATCAT
TGTGGTGGTGAATGGCACTGAGTTGATCAAGCAGAGAAAGATGATTCCCTTGGCTCTCCTTCTTTGCTGTCTGGCGATTT
CCAGGATTTGTCTACAATTGATCATCTTCTTCATGAATCTGGGTACTCTCTTCTTGATTGAAGTCCCCCTACTTGCTGAT
AATTTTGTAATTTTCGTGTTTGTAAATGAATTGGGACTTTGGTTCGCCACATGGCTTGGGGTTTACTACTGTGCCAAGAT
CGCCCCCATAACTCACTCATTCTTTTTCTGGTTGAAGATAAGGATATCCAAGTGGATGCCATGGCTGATCCTCGGGTCCA
TGATGTATGCATCCGTCCCTTCTGTTTTCTGCAGCAAACAGATATGGGTTTATTCCCAAAACGTTTTGTCCAGCCTTTTT
TCCCCAAACGCAACTCAAATCAAAGAAACATCTGCTTTACAGATTGCCTTTCTTATTAGGTTATTATTGCCACTGCTTAT
CTTTCTCGGTTCCACCCTACTTTTGATATTTTCCCTGGGGAGACACACCTGGCAGATGAGAAACACAGCAACAGGCCCCA
GGGACCCTAGCACAGGTGTCCACGTGAGCACGATCCTGTCCGTTCTATCCTTCCTGGTCCTCTGCCTCTCCCACTACATG
GCAGCTGCTTTGCTCTCTTTTCAGATCTTTCAGCTCAGAAGCCTCGTCTTTCTGATCTGTCTCTGGGTGTTTGGGTCCTA
TCCTTCTGGACACTCTATGATCTTAATTTTAGGAAATCCTAAATTGAAACAAAATGCAAAGAAGCTCCTCCTCCACGGGA
AGTGCTGCCAGTGA

>CafaTAS2R2_chr14:27345807-27346718
ATGATCTCCTTTTTGTCAGCTCTTCCTCATGTTATTGTTATGTCAGCAGAATTTATCACAGGGATTACAGTAAATGGATT
TCTTATCATCATGAACTGTAAAGAATTGATCAAAAGCAGAAAGCCAACACCAGTGCAACTCCTTTTCATATGTATAGGGA
TGTCGAGATTTGGTCTGCTCATGGTGTTAATGATACAAAGTTTTTCTCTGTGTTATTTCCACTCTTTTATAAGGTAAAC
ATTTTTGGTACAGCAATGTTGTTCTTTTGGATGTTTTTAGCTCTGTCAGTTTCTGGTTTGCCACCTGCCTTTCTGTATT
TTACTGCCTCAAGATAGCAGGCTTCACTCAATCCTGTTTTCTTTGGCTGAAATTCAGGATCTCGAAGTTAATGCCTTGGC
TACTTCTGGGAAGTTTGCTGGCCTCCATGAGCATTGCAGCTCTGTGTATTGAAGCAGATTACCCTAAAAAGGTGGATGAT
GATGCCCTCAAGAATGCCACATTGAAGAGGACTGAACCCAAGATAAGGCAAATTAGTGAAATGCTGCTTGTCAACTTGGC
ATTACTATTTCCTCTAGCCATATTTGTGATGTGCACTTTTATGTTATTCATTTCTCTCTATAAGCACACTCATCGGATGC
AAAATGGATCTCATGGTGTTAGAAATGCCAGCACAAAAGCCCATATAAATGCATTAAAAACAGTGATAACATTCTTTTGC
TTCTTTATTTCTTATTTTGCTGCCTTCATGGCAAATATGACATTCAGTATTCCTTATGGAAGTCATTGCTTCTTTGTAGT
AAAGGACATAATGGCAGCATTTCCCTCTGGTCATTCAATTATAATCCTCCTGAGTAATTCTAAATACCAACAACCTTTCA
GGAGACTTCTCTGCTTCAAAAAGAATCAAT
GA

>CafaTAS2R3_chr16:7436097-7435147
ATGTCAGGGCTGGGGAAATCCGTGTTCCTGGTTCTGTCTGTCACTCAGTTCATTCTGGGGATGCTGGGGAATGGTTTCAT
AGTGTTGGTCAATGGCAGCAGCTGGTTCAAGAACAAGACAGTCTCTTTGTCTGACGTTATCATCACTAACCTGGCTCTCT

CCAGGATTGTTCTGCTGTGGATTCTCTTGGTTGATGGTGTTTTAATGGTCTTCTTTTCCAAAGTACATGATGAAGGGACA
GTAATGGAAATTATTGATATTTTCTGGACATTTACGAACCACCTGAGCATTTGGCTTGCCACCTGTCTCAGTGTCCTCTA
CTGCCTGAAAATTGCCAGTTTCTCCCATCCGACGTTCCTCTGGCTCAAGTGGAGAGTTTCCAGAGTGGTCGTACAGATGA
TTTTGGGTGCACTGCTCTTATCGTGTGCCAGTGCCATGTCTCTGGTCCATGAATTTAAGATCTATTCTATTCTCAGTGGA
ATTGCTGGTACAGGGAATGTGACCGAGCACTTTAGAAAGAAGAGAAATGACTATAAAGTGGCCCATGTTCTTGGGACTCT
GTGGAACCTCCCTCCCCTAATTGTTTCTCTGGCCTCCTACTTTCTGCTCATCTTCTCCCTGGGAAGGCACACACAGCAGA
TGAAGCACAGTGGCACCAGCTCCAGAGATCTGAGCACGGAGGCCCACCAGAGAGCCATCAAAATCATCGTCTCTTTCCTC
TTTCTCTTCCTGCTTTACTTTCTTGCCTTTTTAATTACATCATCCAGTTATTTCATACCAGAAACTGAGATGGTTAAGAG
AGTTGGAGTAGTTGTTACAATGTTTTACCCTGCCAGCCACTCATTCGTTATCATTCTGGGAAACAATAAGCTGAAGCAGA
TGTTTACGGAGATGCTGTGCTGTGAGCCTGGTTATCTGAAGCCTGGATTCAAAAGACCTTTTGCCCCATAA
>CafaTAS2R5_chr16:7407742-7406855
ATGCTGACTGCTGCCCTACCACTGCTGATGGTGGTGGCAGTGGTTGAATTTCTCATTGGCTTGGTGGGAAATGGAGTCCT
TATGGTCTGGAGTTTTGGTGAATGGGTCAGAAAATTCAACGGGTCCTCATACAACCTCATTGTCCTGGGCCTGGCTGTCT
GCCGATTTCTCCTGCAGTGTCTGATTATGATGGACTTAAGCCTGTTTCCATTTTTCCAGAGTAGCCGTTGGCTTCACTAT
CTCAGTATCTTCTGGATCCTGGTAAGCCAGGCCAGCCTGTGGTTTGCCACTTTCCTCAGCGTCTTCTACTGCAGGAAGAT
CATGACCCTTGAACATCCTGTCTGCTTGTGGCTGAAGCAGAGGGCCTATTGCCTGAGTCTCTGGTGCCTTCTGGTGTACC
TCATGATCAGTTTGTTACTTGTAGCACACATTGGCTTAAAGCCCTATAATCCTTCTCAAGGCAACAGCAGCATTCTGTAC
CCCCTTAAAAGCTGGCACTACCTGTATATAGTAAAGCTCAACGCAGGAAGTGGATTGCCTCTCATGGTGTTTCTTGTTTC
TTCTGGGATGCTGATTGTCTCTTTGTATAGACACCACAAGAAGATGGAGGTACATACAGCTGGTAGGAGAGATGCTCAGG
CCAAGGCTCACATCACTGTACTGAAGTCCTTGGGCTGCTTCCTTATCCTTCATGTGATTTATATCCTGGCCAGCCCCTTT
TCCATTACCTCCAAGTCTTCTGCTGATCTCCTCGTTGTCTTCATCTCTGAGACAGTCATGGCTGCCTATCCTTCTCTTCA
TTCTGTCATTCTGATCCTGGGGAATCCCAGGATGAAGCAGACTTGTCAGAGAATTCTGTGGAAGACAGTGTGTGCTTGGA
AATCCTAG

>CafaTAS2R7_chr27:34898964-34899902
ATGCCGGATAAAGTGGAGAGCATCTTAATGCTCGTAGCAGCTGGAGAATTTTCAATGGGGATTTTAGGGAATACATTCAT
TGGATTGGTAAACTGCATAGGCTGGATCAAGAAGAGGAAGATTGCCTCCATTGATTTAATCCTCACAAGTCTGGCCATAT
CCAGAATTTGTCTATTATGTATAATACTATTAGATTGTTTTATATTGGTGCTGTATCCAGATGTCTATGCTACCGGTAAA
CAAATGAGAATAATTGACTTCTTCTGGACACTAACCAACCATTTAAGTGTCTGGTTTGCCACCTGTCTCAGCATTTTCTA
TTTCCTCAAGATTGCGAATTTCTTCCATCCCCTTTTCCTCTGGATGAAGTGGAGAATTGACAGTGCGATTCCTAGGATCC
TGCTGGGATGCTTGGCCCTTTCTGTGTTTATTAGCCTTGTTGTCACTGAGAATTTGAATGATGATTTCAGATGTTGTGTT
AGGACAAAGAAGAAAACAAACTTAACTGTGAGATGCAGAGTAAAGAAAGCTAAATATTCTTCCATCAAGATTTGCCTCAA
CCTGTTAACGCTATTCCCCTTTTCTGTGTCCCTGATCTCATTTCTCCTCTTGATCCTCTCCCTCTGGAGACATACCAGGC
AGATGAAGTTCAATGCCACAGGGTGTAGAGACTTCAGCATAGAAGCCCACATGGGAGCCATGAAAGCTGTCATCTCCTTT
CTCCTCCTTTTCATCGCCTACTATTTGGCCTTTCTTGTAGCCACCTCTAGCTACTTTATGCCAGAGACTGAATTAGCTGT
GATCATTGGTGAGTTGATAGCTCTAATCTATCCCTCGAGCCATTCGTTTATCCTAATTCTGGGGAGCAATAAATTAAGAC
AGGCATCTCTAAGGGTACTATGGAAAGTAAAATATGTCTTAAAAAGAAGAAACTTCTAA

>CafaTAS2R10_chr27:34876090-34877058
ATGCTAAGCATACTGGAAGGCCTCCTCATTTTTATAGCTGTTAGTGAATCAATACTGGGAGTTTTAGGGAATGGATTTAT
TGGACTTGTCAATTGTATTGACTGTGTGAAGAACAAAAAGTTTTCTATGGTTGGCTTTATTCTCACTGGCTTAGCTACTT
CCAGAATTTGTCTGATATTGATAATAATTACAGATGGATTTATAAAGATATTCTCTCCAGATATGTATTCCTCTGGTAAC
TTAATTGATTATATTAGTTACCTATGGGTAATTATCAATCAATCAAGTATCTGGTTTGCCACCAGCCTCAGCATCTTCTA
TTTCCTGAAGATAGCAAATTTTTCCCACCACATTTTTCTCTGGCTGAAGGGTAGAATCAATAGCGTTCTTCCCCTTCTGA

TGGGATCCTTGTTTATTTCATGGTTATTTACTTTTCCACAAATTGTGAAGATTATTAATGATAATAGAATGAAGAGTAGA
AATACAACCTGGCAGCTCAACATGCAGAAAAGTGAATTCTTTACTAAGCAGATTTTACTCAACCTAGGAGTCATTCTTCT
CTTTACTCTATGCCTGATTACATGTTTCTTGCTAATCGTTTCCCTTTGGAGACACAACAGGCACATGCAATTGAATGTCA
CTGGACTCCGAGACCCCAGTACAGAAGCACATGTGAAAGCAATGAAAATTTTGGTATCTTTTATCATCCTCTTTATCTTG
TATTTTATAGGCATTGCCATAGAAATATCATGTTTCATTCTGCCAGAAAACAAACTGCTGTTTATTTTTGGTATGATGAC
CACAGCCATCTATCCCTGGGGTCATTCATTTATCCTAATTCTAGGAAACAGCAAGCTAAAGCAAGCTTCTTTGAAGACCC
TGCAGCAACTCAAGTGCGAGGCAAGGAGACTGCTCACAGCTGCACAGATCCATGTGGGGGGAAATGGATGTTCCAGGAGA
ATAATCTAG

>CafaTAS2R12_chr27:34855685-34856629
ATGGCAGGCACAATGAAGAATGTATTTATGATGATTTTTGCCGGAGAATTCATAATAGGGATTTTGGGAAATGGATTCAT
TATATTGGTTAACTGTATCGATTGGATCAGGAGCTGGAAGTTCTTCCTGATTGACTTTATTCTTACCTGCTTAGCCATTT
CCAGGATATTTCTGCTGTGCATAATAATGTTAGGCATAGGTCTAGATATAATTTGTAAGGAAATATGGTACAATGATAAT
CAACTGATAACCTTTGAAGTCCTCTGGACAGGATGCAATTATTTCTGCACAATCTGTACTGTGTGCCTCAGTGTCTTCTA
CTTCCTCAAGATAGCCAACTCTTCCAATCCCATTTTCTTCTGGCTAAAACGGAGAATTCACAGACTGCTTCTCATTATTG
TCCTGGGAGCAGTCTTCTATTTCTGCTTGTCCCTGCTTTTGAAGGATATAGTATTTAAGAACATGATCAAAACCAAGGTA
AACACTGAAAGCAATGTGACATTAAATTTCACAGCGAGAAAATATGATTTACTAACTTCTAATATATTCCTGAACATGCT
ATTCGTCATCCCCTTTGCAGTGTCTCTGGCTTCCTTTGTCCTTTTGATCCATTCCTTATGGAACCATACCAGGCGGATGA
AGGGCATTGATTCTGGGGATCTTATCACAGAGGCCCATGTAAGAGCCATGAAGTTTATGATTTCATTCCTGCTATTCTTC
TTTATATACTATTTGAGCAATATTATAATATATTTTGCCTATGTTGTTCTGGATAGTCTGGTGGCAAAAATTTTTGCTAA
TATATTAGTATTTTCCTATCCTTCTGGCCATCCATTTCTTCTGATTTTATGGAACTGCAAATTGAAACAGGCTTCTCTCT
ATGTCCTGAGGAAGCTGAAGTGGTGCATGAATCTAAGGAAACCCGCATACATAAAGCATACCTGA

>CafaTAS2R38_chr16:7224077-7225027
ATGTTCCTTTCAGTACTGGAGCTCGCAGTGGGGATCCTGACCAATGCCTTCATTTTCTTGGTGAATTTTTGGGATGTGGT
GAGGAGGCAGCCACTGAGCAACTGCGATCTTATCCTTCTGAGTCTCAGCCTCACTCGACTTTTCCTGCATGGGCTGCTGT
TTCTGGATGCCATCCAGCTTACATACTTCCAGCGGATGAAAGACCCACTGAGCCTCAGCTACCAGACCATCATCATGCTC
TGGATGATCACAAACCAAGCTGGGCTCTGGCTCACCACCTGTCTCAGTCTTTTCTACTGCTCCAAGATTGTCCGTTTCTC
TCATACCCTCCTTCTCTGCTTGGCAAACTGGGTCTCCAGGAAGGCACCCCAGATGCTCCTGGGTGCCATGCTTTTCTCTT
CTGCCTGCACTCTCCTCTGTTTGGGGGACTTCTTTAGTAGATCTGGCTTTGCATTCACAACTGTGCTACTCATGAATAAT
ACAGAATTTAATTCACAAATTGTAAAACTCAATTTCTATTATTCCTCCATCTTCTGTACCCTGGGGTCAATCCCTCCTTT
CATGTTTTTTCTGGTTTCTTCTGGGGTGCTGATTATCTCTCTGGGAAGGCACATGAGAACAATGAAGGCCAACACCAAAG
ACTCCGGTGACCCCAGCCTGGAGGCCCATATCAAAGCACTCATATCTCTCATCTCCTTTCTCTGCCTCTATGTGGTGTCA
TTCTGTGTTGCCCTTATCTCAGTGCCTTTAACCATGGTGTGGCACAACAAGATCGGGGTAATGATCTGTGTAGGGATCCT
AGCAGCTTGTCCCTCTATACATGCAGCCATCCTGATCTCAGGCAATGCCAAGCTGAGGAGAGCTGTGGAGACCATTCTAC
TCTGGGTTCAGAGCAGCCTTAAGGTAAGGGCAGGCCACAGGGCAGATCTCAGGACTCCAGATCTATGTTGA

>CafaTAS2R39_chr16:6466605-6465643
ATGGAAACCTGCAATCCCCCAGAAAATGAATTGTCACCATTTGGCATCCTCTCGATTTTAACAATTACAGGCACTGAATG
CATCGTTGGTATCATTGCAAATGGGTTCATCATGGCTATAAATGCGGCTGAATGGATTAAAAATAAGACAGTTTCCACAA
GTGGCAGAGTCCTGTTTTTCTTGAGTGCATCCAGAATAGCTCTCCAAAGCTTCACAATGCTAGAAATTACCTTCAGTTCA
ACATCCCCACGTTTTTATAATGAAGATGTTATGTATGACACATTCAAAGTAAGTTTCATGTTCTTAAATCATTGTAGCCT
CTGGTTTGCTGCTTGGCTCAGTTTCTTCTACTTCGTGAAGATTGCTGATTTCTCCCACCCCCTTTTTCTCAAGCTGAAGT
GGAGAATTTCCAGACTGATGCCCTGGCTTCTGTGGCTTTCAGTGCTTATTTCCTTGGGCTACAGTATGCTCCTCTCCAAT

GACATCTACACTGTGTATTGTAACAATTCTTCTATCCCCTCTTCCAACTCCACTAAGAAAAAATACTTCACTAAGACCAA
TGTGGTCAACCTGGTTCTTCTCTATAACCTGGGGATCTTCATTCCTCTAATCATGTTCATCCTTTCGGCCACCCTGCTGA
TCATCTCTCTCAAGAGACATACACTACACATGGAAAGCAATGCCACTGGCTGCAGGGACCCCAGCATGGAGGCTCACATA
GGGGCCATCAGAGCGACCAGCTACTTTCTCATTCTCTATATTTTCAATTCAGTTGCTCTATTTCTCTATATGTCCAACAT
CTTTGATATCAACAGCTCCTGGAATATTTTGTGCAAATTCATCATGGCTGCCTACCCTGCTGGTCACTCCATTCTGCTGA
TTCAGGACAACCCTGGGTTGAGAAGAGCCTGGAAGCGGCTTCAGCCTCAAGTTCATTTTTACCTAAAAGAGCAGACTCCA
TGA

>CafaTAS2R40_chr16:6440017-6439097
ATGTCCAGGTTTAAAATCGTCCTCACCTTGGTGGTCCCCGGAATAGAGTGCCTCACTGGCATCGTTGGGAATGGCTTCAT
CACAATCATCCATGGGGCCAAGTGGGCCAGAGGCAAAAGGCTCCCGGTCACTGACTGCATTCTGCTGATGCTCAGCTTTT
CCAGGCTCTTACTGCAGATCTGGATGATGCTGGAGAATATTTACAGTCTACTATTCCGGGTCACTTACAACCAAAGCACA
GTGTTTATAGTCTTCAAAGTCACTGTCATTTTCCTGAACTATTTCAACCTCTGGCTTGCTGCCTGGCTCAACATCTTCTA
TTGTCTGAGAATCACAAACTTGGCTCACCATGTGTTCTTCATGATGAAGAGGAAAATCACGGAGCTGATGCCTCGGCTTC
TGGGACTGTCACTGTTCATCTCCTTATGCTTCAGCTTTCCTTTCTCTACAGATATCTTCCATGTGTACGTAAACAGTTCC
ATCCCTATCCGTTCCTCCAATACCACCGAGAAGAAGTACTTCTCTGAGACCAATGTGGTCAACCTGGTTCTTCTCTATAA
CCTGGGGATCTTCATTCCTCTGATCATGTTCATCCTTTCGGCCACCCTGCTGATCATCTCTCTCAAGAGACACACACTAC
ACATGGAAAGCAATGCCACTGGCTGCAGGGACCCCAGCATGGAGGCTCACTTTGGGGCCATCAGAGCGACCAGCTACTTT
CTCATTCTCTACATTTTCAATGCAGTTGCTCTATTTCTTTCCATGTCCAACATCTTCGACATCAACAGCTCCTGGAATAT
TTTGTGCAAAATTGTCATGGCCGCCTACCCAGCTAGCCACTCAGTGCTACTGATCTTGGGTAACCCTGGGCTGAGAAGAG
CCTGGAAGAGGTTTCAGCACCATGTTCCTCTTCACCTGTAA

>CafaTAS2R41_chr16:6231777-6230851
ATGCAGCCCGCCGTGTCCGCCTTCTTCATGCTGCTCTTTGTCCTGCTGTGTGTCCTGGGGATCCTGGCCAACGGCTTCAT
CGTGCTGGTGCTGAGCAGGGAGAGGATGCGGCGGGGGAGGCTGCTCCCCTCCGACGTGATCCTCCTTAGCCTGGGCGCCT
CCCGCTTCTGCCTGCAGTGCATTGGGATGATGAACAACTTTTACTACTACCTCCACCTGGAGGAGTACAGCACGGGCCCG
GCTCGGCAATTCTTTGGCCTCCACTGGGACTTCCTGAACTCGGCCACCTTCTGGTTCGGCTCTTGGCTCAGCGTCCTCTT
CTGCATGAAGATCGCCAGCTTCACCCACCCCACCTTCCTCTGGCTGAGGTGGCGGCTCCCAGGCTCGGTGCCCTGGCTCC
TCGGGGCTTCCCTCCTGATCTCCTTCCTCGTCACCCTGCTCTTCTTTTGGGGAAACCATGCCGTGTATCAAGGATTCCTA
ATCAGAAAATACCCCGGGAACATGACCTTCCAGCAGTGGAGCAGGAGGCTGGAAATTCACTATTTCTTGCCCCTGAAATT
CATCACCTTGTCAGTGCCTTGCTCTGTCTTCCTGGTGTCCATCGCACTGTTGATTAATTCCCTGAGGCGACACAGGGGGA
GGATGCGGCGCAGTGGCCACGGCCTGCAGGACCCCAGCAGCCAGGCTCACACCAGGGCTCTGAAGTCCCTCGTCTCCTTC
CTCATTCTGTATGCTCTGTCCTTTGCGTCCCTGGTCATCGATGCTGCGGGTTTCTTCTGCTCGCAGAGTGACTGGTACTG
GCCCTGGCAGATTTTAATCTACCTGTGCACCTCTGTCCATCCCTATATCCTCATCCTCAGCAACCTCCGGCTCCGAGGGG
GGTGCAGGCAGCTACTTCTGTTGGTCAGGGGCTCCCAGCTGGCCTAG

>CafaTAS2R42_chr27:34739537-34740508
ATGTTAGCTGGATTGGATATAATCTTTCTTACACTGTCAACAGCAGAATTCATAATTGGAATGTTGGGGAATGCGTTCAT
TGGACTGGTAAACTGCTCTGAATGGGTCAAGAACCGGAAAATCTCTTTAGCTGACTTCATTCTCATCTGCTTGGCTATCT
CCAGAATCGCTCAGCTGTTGGTGTCATGGTTTGAATCATTTATGATGGGACTATCTCCACTTTTCTTTTCCACTTATAAA
CTGGCAAAATCTATTACTTTGCTTTGGAGAATAACTCATCATTTGGCTACGTGGTTTAGTACCTGCCTAAGCATTTTCTA
CCTCCTTAAGATAGCTCAGTTCTCTCATTCCCTTTTCCTCTGGCTGAGGTGGAGAATGAACAGAGTGGTTCTTGCAATTC
TTGTATTTTCTTTGTTCTTTCTACTGTTTGACTTTCTAATGCTAGAAACATTCAATGATCTCTTCTCGAATGTCGATGCA
ATGGATGAAAGTAATCTGACTTTATATATATATGAAAGTAAAACTTTTTATGTTAAAACCTTGATTCTTCTTAGTTTTTC

CTATATCATTCCTATTATTCTGTCCCTGACCTCATTGCTCCTTTTATTTCTGTCCTTGGTAAAACACATCAGAAATTTGC
AGCTCAACTCCATGGGCTCCAGGGATTCCAGCACACAGGCCCATAAAAAAGCCATTAAAATGGTGATGTCTTTCCTCTTC
CTTTTCACAGTTCACTTTTTTTCCATACAATTGTCAAATTGGATGTTTTTTTATTTTGGAACAAGAAGATCACAAAGTT
TATCATGTTGGCCGTTTATGTCTTTCCTTCAAGCCACTCACTAATTTTGATTCTGGGAAACAGCAAGCTGAGACAGACAG
CCTTGAAGGTACTGTGGCATCTTAAAAGCTCCCTGAAAAGAGAAAAACCAAATTCATCTTTACCGATAGACTTTCCAGAA
TCTTTCCAATGA

>CafaTAS2R408A_chr27:34792712-34793761
ATGCTACCTTTACTACAGAGCATTTTTTCCATCCTAGTAATGACAGAATTTGTTCTAGGAAATTTTGCCAATGGCTTCAT
AGTGCTGGTGAACTACATTGCATGGGTCAAGAGACAAAAGATCTCCTCAGCTGATCAAATTCTCACTGGTCTGGCTGTCT
CCAGAATTGGTTTACTCTGGGTAATATTAATAAATTGGTATGCAACTCTGTTGAATCCAGCTTTATATAGCTTAGAAGTA
AGGCTTCTTGTTCATATTGCCTGGACAGCGAACAATCATTTTAGCATCTGGCTTGCTACTAGCCTCAGTGTATTTTATTT
GTTCAAAATAGCCAATTTCTCTAACCTTATTTTTCTTCGCCTAAAGTGGAGAGTTAAAAGTGTAGTTTTTGTGATGCTGT
TGGGGTCTTTGTTCTTTTTGGTTTTTCATGTTGCAGTGGTAAGCATATATGAGCAAATGCAGATGAAGGAATATGAAGGA
AACATCACTAGGCAGACCAAACTGAGGGACATTGCACAGCTTATGAATATGACTGTATTCACGCTAATGAACTTTGTACC
CTTTGCTATATCCCTAACATCTTTTCTGCTGTTAATCTTTTCCCTGTGGAAACATCTCAAGAAGATGCGATCCGGTGGTA
AAAGATATCAAGATTCCAGCACCAAGGTCCACATAAAAGCCATGCAGACTGTGATCTCTTTTCTTTTGTTATTAGTTTGT
TACTTCCTGACTTTAATTGCCATAGTTTGGAGTTCTAATAGGCTGCAGAACAAGTTGATCTTCTTGCTTTGCAAGGCTAT
TGGAATCCTGTATCCTTCAAGCCACTCATTTATCCTGATTTGGGGAAACAAGAAGCTCAGAGAGGACTTTCTGTCATTTC
TGTGGCAGCTGAAGGGCTGGCTGAAAAAAGGATATAAGAGGAGCATCATGTGTCTTCTAGGAGAAAACAAATTGATGGAG
TCTGTAATATTTTTTTCTTCTACTTCTTTTTCTAATGAGTATGTAATTGAGCAATTTCCAAAGATTTACCTAAAAAAGTC
TTTTCTCTGA

>CafaTAS2R62_chr16:6262445-6261539
ATGTCCTCCTCACCTACATTGATCTTCATGGTCATCTTCTTCCTGGAGTCGTTGGCTGCAATGCTGCAGAATGGCTTCAT
GGTTACTGTGTTGGGCAGGGAGTGGGTGCGACGCCGGACGCTGCCTGCAGGTGACATGATTGTGGCCTCCCTGGCTGCCT
CCTGGTTCTGCCTGCATGGGGTGGCCATCCTGAACAACCTCTTGATCTTCTTTGGTTTTCACTTCGTAAGGGATTATTAC
AACACCCTCTGGCACTTTGTCAACACTCTCACTCTCTGGCTCACTGCCTGGCTTGCTGTCTTCTACTGTGTGAAGGTCGC
CGTCTTCTCTCACCCGGTCTTCTTCTGGCTGAAATGGAGGATTTCTCGGTTAGTGCCCAGGCTGCTGCTGGGCTCCCTGG
TCTTAGTTGGCCTGACAGTCATCTCATCAGCCATTGTGACTGGAATTCTGAAACAGATGATTGCCTCCAAGAGTTCCCAA
GGAAACAGCACCTGGGCTGAGAGAGTACAGGCCTTCTATAGGTCTTTTCATCTATTTGATGTAATGCTTATGTGGTCAGT
TCCATTCCTCCTGTTCTTGGTGTCCATGCTCTTGCTTGTGTTCTCACTGTGCCGGCATTTGGGGTTGATGAGGAACTATA
GACAGGACCCATGTGATCCTAGCACCCGGGTTCACACGATGGCCCTGAAGTCACTTGTCTTCTTCCTTGTCTTCTACACA
CCATATTTCCTGTCTCTGGTTGTTGTTGCTATAGAAATAACAAACTTCCAGAGTCACTGGTACTGGGCCTGGGAAGTGGT
AACCTATGCGAGCATCTGTCTGCACTCCAGCATGCTGGTGCTAAGCAGCCCCAAACTGAGAAAGGTCCTGATGACCAGGC
TTTGGAAAGCTCTGGACAAAGGCTGA

>CafaTAS2R67_chr27:34748642-34749580
ATGCCATCTAGAATTGAAAATGCTTTTCTGGTAGCAGCAGCAGGAGAACTCATAACTGGAATGTTGGGGAACGGTTTCAT
TGTACTAGTTAACTGCATTGACTTGGTGAAGAATCTAAAGCTCTCTACTGCTGACTGCATCCTCACCAGCCTGGCTCTTT
CCAGAATCATTCTTCTTTGTATAATACTACTTGATTCACTTTTAATGGTGTTTTGGCAACATCTTTATGCCATTGATAAG
CTAGCAAAATTCATTAGTGTTTTTTGGACACTAAGCAATCACCTAACTACCTGGATTGTTACCTGTCTAAATGTTTTCTA
CTTCTTTAAAATAGCCAATTTTTCCCACCCCTGTTTCACCTGGCTGAGGTGGAGAATTAGCAGAGTGCTACTTGTGCTTC
CACTGGGGTCTTTATTCTTACTGTTTTTCAACTTTGAATTATTAGATACATTTACGAATTTCTGGGTTAATCTCTATCAA

AGACATGAAAGAAACTCAATTTGGTCCCTAGATGTAAGTAAAACTCTGTATCTTAACAGCTTGATTGTTTTCAGTTTCAT
CTACTTAATCCCCTTTCTTCTGTCCCTGGCCTCTTTGCTCCTTTTATTTCTTTCCTTAATGAGACATATCAGGAATGTGC
AACGGAACTCCAGCTCTAGGGACTTCAGAACAGAGGCCCATAAAAGGGCCATGAAAATGGTGATGTCTTCTCTTTTTCTT
TCCATGGTTAATTTTACTTCCATCCTATTAACAGGATGGTTTTCCCTTTTACTGCAGAATCATCAGGCCAATTTGGCTGT
CCTGTTATTATCGACTCTTGTACCCTCAGGCCACTCATTTATTCTAATTTTGGGAAACAACAAGTTGAGACAAGCTGCGT
TAGGTCTACTGTGGCATCTTAATTGCCACCTGAAAATGGTGAAGCCTTTCGCTTCCTAG

>CafaTAS2R8p_chr27:34893770-34894688
ATGCTCAGTATGGAAGACATCATCTTCATGATCGTAATAACTGGAGAATTCATAATAGGAATGTTGGGGAATGCATGCAT
TGGACTAGTAAACTCTATTGACTGGATTAAGAAGAAAAAGATCTCCTCAATTGACTATATCCTCACCAGTCTAGCCAAAA
TTGGTTTGCTCTGTATAATGATACTAAATGGCACCAAAATCGTATTCTGCCCAGATTTTTATAAAAAGGATAAGCTACAA
GCAGTCATTAATATCTTCTGGATACTCACCAACTACTTAAGTATGTGGTTCACCACCTGCCTTAATGTCTTCTATTTACT
CAAGATAGCCAATTTCTCCCACCCACCTTTTTCTCTGGCTAAAGAGGAGAACTGACAGAGTGATTCACTGGATTCTGCTG
GGTTGTTTGGCTCTTTCTTCCTTAATCAGCCTTATACTAGCAATGACACCAAATTATGATTGTGAGTTTTGTAACATTGC
AAAACATAAAGGAAACTGAACTGAAATGCTCTCTGTAAGTAAAAGTCAATACTTCAAGCCATTGACTCTCTTTAACTTGT
TGGCAATTGTCCCATGTACTGTGTCATTGGTCTCATTTTTCCTTTTAATTATGTCCCTATGGAGACATATCAAGCAAATG
AAACTCAACGTTATAGGCTGTGGAGACCTCAGCACAGAGGCGGGAGCCATGAAAACTGTGACTTCATTTCTTTTCCTCCT
TTTTGTGTACTATGGGGCTTCTCTTTTGGTAACTTTTAGCTACCTTATGAAAGAAAGCAAGTTAGCTGTGATGTTTGAAG
AAATTATAGCAACACTCTATCCTTCTGGTCATTCACTTATTTTGGTTATTGGAAATAACAAGCTGAGGCAGGCATTTATC
AGGATGCTGAGATACGGAAGAACAGTCTGCATGATGTAA

>CafaTAS2R4p_chr16:7425302-7424403
ATGCTTCAGATATTCTTTTTATCTGCCATTATTTTCTCAGCAATTTTGAATTTTGTGGGACTCATTGTAAATCTGTTTAT
TGCAGTGGTCAGTTATAGGACTTGGCTCAAAAGCCATAGAATTTCCTCTTCTAATTGGATCCTCTTCAGCTTGGGCATCA
CCAGATGTCTTATGCTGGGACTGTTTCTACTCAACATCATCTACTTCTTCATCTCTCCAAAAATGGAAAGGTCGGTGCAC
CTATCCCACTTTTTCCTGTCGTAGTGGATGTTTTTGGACTCTAATAGTCTCTGGTTTGTAACCTTGCTCATTGCCTTGTA
CTGCGTGAAGATTACGGACTTCCAACTTGGAGTATTTCTCCTGCTGAAGCGAAATCTCTCCCCAAAGATCCCCAGGCTGT
TGCTAGCCTGTGTACTGATTTCTGCCTTCACCACTCTCCTGTATGTTGTGCTCAAACAGACATCATCCCTTCCTGAATTT
GTGACTCAGAGAAATGGTACAGGATGTGGCATCCATGGGAGTGTCTTGTCTTTGGTGACCTCTTTGGTCTTGCGCTCAGT
TCTCCAGTTTATCATTAATGTGACTTCTGCTTCCTTGTTGATACATTCCTTGAGGAGACATATACAGAAGATGCAGAAAA
ACACCACTATTTTTTGGAATCCTCAGACTGAAGCTCATGTGGGCGCTATGAAGCTGATGATCTGTTTCCTCATCCTGTAC
ATTCCTTACTCAGTTGCTACCTTGCTACATTATTTCCCTTATGGTGGGATGGATTTGAGAACCAGATCCATCTGTTTGGT
TATTTCCAGCTTTTACCCTCCAGGACATTCTATTCTCATTATCCTCACACATCCTAAACTGAAAACAAAAGCAAAGAAGA
TTCTTTGTTTCAACAAATAG

>CafaTAS2R9p_chr27:34890746-34891680
ATGCTAAGTACAATGGAGGTAATATACATCTTCTTGATTGCCGGAGAAATGACTATAGGAATTTGGGGAAATGGATTTAT
TGCACTGGTTAACTGCACTAGGTGGCTCAGAAGGAGAGACATCTCCGTGATTGACATCATCCTGGTGATCTTGGCCATCT
CCAGAATCTGTTTGCTGTGTGTGGTATCTTTAGATGGCTTTATTTTGCTGCTCTCTCCAGATATATATGCCAATAGCGAG
CTAATGAATATTGTGGATGTTGTCTGGACACTTAGCAATCATTCAAGTATCTGTTTTACTTCTTGCCTCAGTATTTTCTA
TTTACTGAAGATAGCCAATATATCCCATCCGTTTTTCTCTGGCTGAAGCTAAAGATTAACAGAGTCATTCTGGGGATGT
TTCTGATGTCTTTTCTTACCTGTATAATTATTAGTGTTTCATTGAATGAGGACTTCTGGGATCCCTTCAAAGTCAATCAT
AAGGAAAACATAACTTGGGAATCCAAAGTGAGTAAAATCCCAAGTGCTTTCAAACTGTTTATCCTGAATCTGGGAGCTAT
AGTTCCCTTTGTTCTTTGCCTAATCTCAGTTCTCTTGTTACTTTTCTCCCTATTTAGACACACTAGGCAGATGAAACTTT

ATGCCACAGGGTCCAGAGACCCCAGCACAGAGGCCCACATGAGGGCCATAAAGGCAGTGATGATCTTTCTGCTCCTCTTC
ATTATTTACTATGCAGTCTCTCTTGTAGTAACCTCTAGCTTCCTGATTCCTCACGGAAAATTAGTGGTTATGTTTGGTGG
CGTGGTAGTTGGCATTTTCCCATCGAGCCATCGTTCATACTGATAATGGGCAACAGCAAGCTGAGGGGGGCTTTCCTAAA
AGTGCTTAGGATTGTGAAGGGTTTCCACAAAAGAAGGAAACCTTTTGTTCCATAG

>CafaTAS2R408BP_chr27:34812404-34813330
ATGCTACCTTTACTACAGAGCATTTTTTCCATCCTAGTAATGACAGAATTTGTTCTAGGAAATTTTGCCAATGGCTTCAT
AGTGCTGGTGAACTACATTGCGTGGGTCAAGAGACAAAAGATCTCCTCAGCTGATCAAATTCTCACTGGTCTGGCTGTCT
CCAGAATTGGTTTACTCTGGGTAATATTAATAAATTGGTATGCAACTCTGTTGAATCCAGCTTTATATAGCTTAGAAGTA
AGGCTTCCTGTTCATATTGCCTGGACAGCGAGCAATCATTTTAGCATCTGGCTTGCTACTAGCCTCAGTGTATTTTATTT
GTTCAAAATAGCCAATTTCTCTAACCTTATTTTTCTTCGCCTAAAGTGGAGAGTTAAAAGTGTAGTTTTTGTGATGCTGT
TGGGGTCTTTGTTCTTTTTAGTTTTTTCATGTTGCAATGGTAAGCGTATATGAGCAAATGCAGATGAAGGAATATGAAGG
AAACATCACTAGGCAGACCAAACTGAGGGACATTGCACAGCTTATGAATATGACTGTATTCACGCTAATGAACTTTGTAC
CCTTTGCTATATCCTTAACATCTTTTCTGCTGTTAATCTTTTCCCTGTGGAAACATCTCAAGAAGATGCGATCCAGTGGT
AAAAGATCCCAAGGTTCCAGCACCAAGGTCCACATAAGAGCGCAGACTGTGATCTCTTTTCTTTCGTTATTAGTTTGTTA
ATTCCTGACTTTAATTGCCATAGTTTGGAGTTCTAATAGGCTGCAGAATGGACTATTCTTCATGCTTTGCCAAGTTTTTG
CATATGCATATCCTTCAAGCCACTCATTTATCCTGATTTGGGGAAACAAGAAGCTAAGAGAAGCCTTTCTGTCTGTTTTA
TACCAGGTGAAGTACTGGTTGAAAGACCAAAAACTCTCAACTCAATAA

>CafaTAS2R60p_chr16:6256349-6255251
ATGAATGGAGATGACACGATTCCAGAATCTCCAGTGACTGTTTAGAGACCCATCACCCTGGCTATCATTTTGTCTTATTT
TTTGTTCCTGGTGGCAGCAGTGACCAATAGCTTCATCACTATAGCCCTGGGCATGGTGTGGCTGCTACGGAGAACACTGT
TAGCCTTGTGATAAATTATTAGTCAGCCTGGGGGCCTCTTGCCTCTGTCTGCAGTGGGTGGTGAGAGGGAAGAGCATTTA
TATTTTCCTGTATCCAGGGGCCTTCCCATACAATCCTGTACTGCAGTTCCTGGCCTCCCAGTGGGACTTGTTGAACACTG
CCACCTTATGGTTCTTCACCTAGCTGGGTGCCTTCTGTTGCGTGAAAACCCGCAACCATCATTCATCCTCCCCATCTTTT
CTGGCTAAAACAGAAGGTGTCTGGGTTGGTTCCATGGGTGCTGCTCAGTTCTGTGGGGCTCCAGCTTGAGCACCTATTTT
CATAGGCAATCGGAGCTTACAGCCCTATTTTTTAAAAAGATTTTATTTATTTATTCATGAGAGACAGAGAGAGAGGCAGA
GACACAGGCAGAAGGAGCAAGCTCCCGATGGGGAGCTGGATGTGGGACTCGATCCCAGGATTCCAGGATCATGACCCGAG
CCAAAGGCAGATGCTCAATCACCGAGCCACCCAGGATGCCCCATATCCCTACTTTTTAAGGAGAGGGTTGCAATCTTGGA
AGCCTATTTCTTCTCTTTAAAACTTGTTACCTGGACAGTCTTTGCTGTTGTCTTTCTTGTTGGTATGGCTTTGCTCATGT
CTCTGGAAAGACACACTAAGAAGGCCCTGCTCTCCATTGTGGCCTTTGTGACCCCAGTGCCTGGGCACACACCCAGGTTC
TCCTGGCTCTCATCTCCTTTGGTATCCTTCTCACCTCCTATTCTTCTGTCATTGGTGCTTAGCACTGCAGGTGTTTTTCC
ATCTTGGAATGTAGGCGCTGGGTGTGGCAGGCAGTGATTTATCTGTGCACAGTGGCCCACCCCATCACTGCTCTTGAGCA
ACCCCAGGCTGCGAGGTGTGCTGGAGGGGGGCCGCTATGCAGGGTGCTGAGTATCTTGA
>TutrTAS2R1P_scaffold_93142:21120-22012
ATGCTGGAGTCTCACCTCATTAGCCACCTTTGTTTGGCAGTGATAAAATTTCTCTTTGGGGTTTTAGTAAATGGCATCAT
TGTGGTTGTGAATGACACTCACTTGATCAAGCAGAGAAAGATGATTCCATTGGATCTCCTTGTTTCCTGCCTGGCGATTT
CCAGGATTTGTCTGCAACTAGCCATCTTCTACGTTAACCTGGCTGTTCTTTCCTTGATTGAATTCCCTCAGCTTGCTGAG
AAGTTCGTAATTCTCACATTTATAAATGAATCGGGACTTTGATTTGCCACATGGCTCAGCCTTTTCTACCGTGCCGAGAT
TGCCACCATTGCTCACCCACACTTCCGCTTGAAGGTGAGGATATCCAAGTTGGTTCCTTGGCTGGCACTTGAGTCCCTGC
TATATGCATCCAGCATGGATGTTTTCCACAGCAAACATAGGTGGATATTTTCCAAAGAACACTTCCTGGGCCTTTTCTCC
CCAAATGCAACCACCCAATCAAAGAAATACCTGCTTTACAGTTTGCCTTTCTTTTTGCTGAGTTTTCATTGCCATTACTT
ATCTTCCTTATTTCTTCTCTGCTCTTGATATTTTCTCTGGGGAGACACACCTGACAGATGAGAAACACAGCAACAGGCCC
CAGGAGCCCTCGCACATGCGTGCACATCAGCACTCTTCTCTCCATCCTGTCCTTTCTGGTCCTCTATCTCTGCCACTCCA

TGACAGCTGCTTTGCTCTTTTCCCAAATTTTCAACTTTAGAAGCTTCATATTTCTGTTCTGCATCTTGTGGGTTGGTTCA
TACCACTCTGGACACTCTATTACCTTAATTTTAGGAAATCCTAAAATGAAACAAAATGCAAAGAAATTGCTCCTCCACAG
AAAGTGCTGTCAGTGA
>TutrTAS2R2P_GeneScaffold_691:426433-427341
ATGGCCTCCTCTTTGTCAGCTCGTCTTCATGTTATCCTCATGTCAGCAGAATTTATCACAGGGATTACAGTAAATGGATT
TCTTATAATCATCGACTGTAATGAATTGGTCAAAAGCAGAAAGCTGACACCAATGCATCTCCTTTTCATATGCATAGGGA
TGTCTAGATTTGGTTTGCAGATAGTGTTAATGGTAAAGTTTTTCTCATGTTCTTTCCACTCTTTTATAGAGTAAAAATTT
ATGGTACAGCGATGATTTTTTGGGGATGTTTTTCAGCTCTGTCAGTCTCTGGTTTGCCACCTGTCTCTCTGTATTTTACT
GCCTCAAGATAACACACTTCACCCAGTACTGTTTTCTTTGGCTGAAATTCAGGATCTCAAAGTTAATGCCTTGACTGCTT
CTGGGAAGCCTGCTGACCTCCGTGAGCATTGCAACTCTGTGTGTCAAGGTGGATTACCCTAAAAATGTGGATATTGATGT
CCTCAGGGATGCCATGCTAAAGAGGACTAAACTCAAGACAAAGCAGATTAATGAAGTGCTTCTTGTCAGCTTGGCATTAA
TATTTCCTCTGGCCATATCTGTGAGGTGAACTGTTATGTTATTCAGTTCTCTCTATAAACACGCTAATCGGATGCAAAAT
GGACCTCTTGGTTTTAGAAACGCCAGCACTGAAGCCCATATTAATACATTAAGATCAGTGATAACATTCTTTTGCTTCTT
TATTTCTTATTTTGCTGCCTTCATGGCAAATATGACATTCAGTATTCCTTATGGGAGTCAGTGCTTCTTTGTGGTGAAGG
ACATAATGGCAGCATATATCCCTCTGGCCATTCGGTTATAATTATCTTGAGTAATTCTCAGTTCCAACAACCAGTCAGGA
GACTTCTCTACCTCAGAAAGAATCAATGA
>TutrTAS2R3P_GeneScaffold_3490:375543-376703
ATGCTGGGACTCACCGAGTGCGGGTTTCTGGTTCTGACTGCCACTCAGTTCATTCTGGGAATGCCGGGGAATAGTTTCAT
GGGTTGGTCAATGGTAGCAGCTGGTTCAAGAACAAGAGAACCTCTTTGTCTGACTTCATCATCACTAACCGGGGTCTCTC
CAGGATTGTTCTGCTGTGGATTCTCTTTTTTTTTTTTGCGGTACGCGGTCCTCTCACTGTTGTCGGCCTCTCCCGTTCGN
NNNNCACGAACCCGTGTCCCCTGCATCGGCAGGCGGACTCTCAACCACTGCGCCACCAGGGAAGCCCTCTGCTGTGGAT
TCTCTTGATTGATGGTGTTCTCTTCCAAACTCCACGATGAATAATTTGCAGTCATGCAGATTAGTGATATTTTCTGGACA
TTTACAAACCATCTGAGCATTTGGCTTGCCACCTGTCTCAGTGTCTTCTACTGCCTGAAAGTCGCCAGTTTCTCCCATCC
TACGTTCCTCTGGCTCAAGTGGAGAGTTTCCAGGTTGGTTGTATGGATGCTGTTGGTACCCTGCTCTTATCATGTAGCAG
TGCCGTCTCTCTGATCCATGAATTTAAGATCTAGTCTGTTCTCAGTGGAATTGATGGAACAGGGAATGTGACTGAACCCT
TTAGAAAGAAAAGAAATGAATATAAGCTGATCCATTTTCTTGGCACTCTGTGGGACCTCCCTCCCTTAATTGTATCTCTA
GCTTCCTACTTTCTGCTCATCCTCTCTCTGGGGAGGCGTATGCGGCAGATGCAGCAAAACTTTACCGGCTCCAGATATCC
AAGTACTGAGGCCCAAAAGAGGGCCATCAAAATCATCCTTTCCTTCCTCTTTCTCTTCCTACTTTACTTTCTTTTCTTTG
CAATTTTGACATCCAGTTATTTCTACCAGCAACTGAGGTGATTATGATGACTGGAGAAGTAATTACAATGTTATATCCTG
CTGGCCGCTCATATATTCTCATTCTGGGAAATAATAAGCTGAAGCAGATGTTCATGGAGACGCTTTGGTGTGAGCCTGGT
CATCTGAAGCCTGGATCCAAGGAACCCGTTTTTCCATAG
>TutrTAS2R5P_GeneScaffold_3490:392469-393351
ATGCTGACTGCTGTCCTAGGACTGTTAATGCGGGTAGCAGTGGCTGAATTTCTCATTGGCCTGGTTGGAAATGGAGTCCT
CGTGGTCTGGAGTTTTGGAGAACGGCTCAGAAAATTCAAGGGGTCCTCATATAACCTCATTGTCCTGGGCCTGGCTGTCT
GTTGATTTCTTCTGCAGTGGTTGATTATGGTGGACTTAAGTCTGTTTCCACTTTTCCAGAGCAGCCATTGGCTTCGCTAT
CTCCATGTCTTCTGGGTCTTAGTAAACCAGACCAGCCTGTGGTTTGCCACTTTTCTCAGTGTCTTCTACTGCAGGAAGAT
CATGACCTTTGAACACCCTGTCTACTTGTGGCTGAAGCAGAGGGCCTGTTGTCTTAGTCACTGGTGCCTTCTGGTGTACT
TCATGATCAGTTTGTTACTTATAGTCCAGGGTAGCTTAGAGTTCTCCAATCTTTCCCAAGGAAACAGCAGCATTTTATAC
CCCCCTTCAAACTGGCACTGTCTGTATATATTATGGCTCAATACAGGAAGTATAATGCCCTTCATGGTGTTGCTTATTTC
CTCTGGGATGCAGATTGTCTCTTTGTGTAGACACCGCAGGAAGATGAATGTCCATACAGTCGGCAGGAGAGATGCTCAGG
CCAAGGCTCACATCACTGTCCTGAAGTCCTTGGGCTGTTTCCTTATACTTTACATAGTTTACATCCTGGCCAGCCCCTTC
TCCATCACCTCCAGGTCTTTTCCTGCTGCTCTTACCGCTCTCTTCATCTCTGAGACACTCATGGCTGCCTAGGCTTCTCT
TCATTCTGTCATATTGATCATGGGGAATTCCAGGATGAAGCAGACTTGTCAGAGAATCCTGTGGAAGACAGTGTACGCTT
GGA

>TutrTAS2R16P_scaffold_109169:86664-85768
ATGATAACCATCCAACTCTGTCTTCTTCATGATCATCTATATGCTCAAGCTCTTGACAATAATTATGCAGAGCAGCTTAA
CTGTTGTAGTGCTGGGCACAGAGTGGGTAAGTTTCCAAAGGCTATCACCTGTGGAAATGATTCTCACCGGCCTGGGTGTC
TGCATGCTTCTGTCAACTGTGGTCATCAATGCTGTACAACTTTTGCTCCCACTTCTACCCTAGTTACGAATTTTGGTACT
TCAGTATCGTCTGGGAATTTACTAACATTCTTTCATTCTGGTTGACCAGCATGTTTGCTGTCTTCTACTGTGTCAAAGTC
TCCTCCTTCAGCCACCCCATCTTCTGGCTGAAGTGGAGAATTGTGAGGTTGGTTCCTTGGCTGTTGCTGGGTTCTCTGCT
GACTTCTTGTGTGTCTATCATCTTTGCAGCTGTTGGGCATTACAGCAAGATTCAACTAATCTCCATGACGCATTTCCCTA
GAAACAGCACCATGACTGAGAGACTTGAGATATTCCTGTGGGATTCTTCCATGTGTCACAAGTGGTTGTGTTGATTATTC
CTTTCCTCCTGTTCCTGGCCTCCACCGTCTTGCTCATGGCCTTATTATTCCAACACCTGAGGCAGATGAAAGATCATCAC
ACCAGCCACTCTCCAGCCTGAAAGCTCACTCTACTGCCCTGAGGTCTCTTGCTGTCTTCCTCATTTTCTTCACCTCTTAT
TTTCTGACCCTAATAATCTCCATGTGGGGTGTCCTTTTTAATAAGGGGTCCTGGTTCTGGGCCTGGGAAGCTATCATCTG
TGCTCTGGTCTCTATTCATTTGACTTCACGGATGCTGAGCAGCCCTAAACTGAAAAGGGTTTTAAAGGTAAAGTGCTGGG
ACCTAGAGGCTGCCTGA
>TutrTAS2R38P_scaffold_90586:6346-7344
ATGGTGACTCTGACTGCCACTGTAACTGTGCCCTATGAAGTCAGGAATGCATTTCTGTTCTTTTCAGTCCTGGAGTTTGC
AGTAGGGATCCTGGTCAATGCCTTCATTTTCTTGATGAATTTTTGGGTCGTGGTGAGGAGGTGGCCACTGAGCAACTGTG
ATCTTGTCCTGCTGAATCTCAGCCTCACCTGGCCTTTCCTACACGGGCTGCTCTTTCTGGATGCCATCCAGCTTACCCAC
TTCCAGTGGGTAAAAGACCCGCTGGGCCTCTGCTACCAGACCACCCTCATGCTCTGGATGCTCGTAAATCAAGCTGGCCT
CTGGCTCACCACTTGCCTTAGTCTCCTCTACTGCTCCAGGACTGTCCATTTCTTTCACACCTTCCTCCTCCGCTTGGCAA
GCTGGATCTCCAGGAAGATCCCCCAGATGCTCCTGGGTGCTATTTTTTCCTCCTGTGTCTGCACTGTTCTCTATTTGTGG
GACTTTTTCAATAGATCTCACTTCTCAGTTGCAACCATGCTACTCATGAATAACAATACTCAATTGAGAAACTGAGAAAA
CTCAATTTCTTTCATTCCTTCCTCTTCTGCAGCCTGGGGTCCACCCCTTCTTTCTTGCTTTTTCTGGTTTCTTCTGGGGT
GTTGATTGTCTCCCTGGGGAGGCACATGAGGACAAGGAGGGCCAAAACCAGAGACTCTCGGGACCCCAGCCTGGAGGCCC
ACATCAAAGCACTCGGGTCTCATCTCTTTCTTCTGCCTGTATGTGGTGTCCTGCGCTGGCTTCATCTCGGTGCCTTTGCT
GATGCTGTGGCACAACAAGATCGGGGTCATGGTCTGTGCAGGGATACTGGCAGCCTGCCCCTCGGGGCACACAGTCATCC
TGATCTCAGGCAATGCCAAGCTGAAGAGAGCCGTGGAGACCATTCTGCTCCGGGCTCAGAGCAGCCTAAAGGTAAGGGCG
GACCGCAAGGCAGATCCCAGGATGCCAGATCTATGTTGA
>TutrTAS2R39P_scaffold_98570:44391-45069
ATGACTGAAACCTGCAATCCCCCAGAAAATCAACTGTCACCATCTCGCATCATTTTGATGTGAATCGTTATAGGCACCGA
ATGCGTCCTTGGTCTCACTGCAAATGGGTTCATTGTGGCTATAAATACAGCAGGATGGATTCACAACAAGGCAGTTTCCA
CAAGTGGCAAGATCCTGCTTCTCCTGAGCGTATCCGGAAGAGTGCTACAAAGCTTCATGATGCTAGAACTCACCTTCAGT
TCAACATCCCCACACTTTTATAATCAAGACATTCATCGTATATGATACGTTCAAAGGAAGTTTCATGTTCTTAAATGATT
GTAGCCTCTGGTTTGCTGCCTGGCTTAGATTCTTCTACTTCGTGAAGATGGCGGATTTCTCCTACCCCCTTTTCCTCAAG
CCGAAGTAGAGAATTTCTGGATGGATGCCCTGGTTTCTGTGACTATCAGTGTTTGTTTCCTTTGGGCCACAGTGTGTTCT
TCCTCAAAAACATCTACACTATGCATTGCAACCATCCTTTTTCTAGCCCCTCCTTCAACTCCACTAAGAAAAATTACTTC
ACTGAGACCAACGTGATCAGCCTGGTTCTTTTCTTTAACGTGGGAATCTTCGTTCCTCTGATCACGTTCATCCTACCTGC
CACCCTGCTGATCATCTCTCTAAAGAGACACACCCTACACATGGAAAGCAATGCCACTGGTTCCAGGGACCCCAGCATGG
AGGCTCATGTGGGGACCATCAAAGCTATCAGCTATTTTCTCATTTTCTAAATTTTCAATGCAGATGCTCTATTTCTTTCC
ATGTCCGACATCTTTGATATCAATAGTTCCTAGAATACTTTGTGCAAAATCATCATGGCTGCCTATCCTGCTGACCACTC
CATCCTACTGATACAGGACAACCCTGGGTTGAGAAGAGCCTGGAAGCGGCTTCAGCCTGGAAGCGGAGTTCACCTTTACT
TGAGAGTGGACTCTATGA
>TutrTAS2R60P_scaffold_108147:11082-10178
ATGAGTGGAGAGGACGTGGTTCCAGGACCTCAGTTGGCTGATAAGATAGCCTTTATCTTTGCTATCATTTTATTCCTTTT
GTGCTTGGTGGCAGTGGTGGGTAATGGCTTAATCACCATGGCACTGGGCATGGAGTGGTTGCTGCAGAGAACTTTGTCAC

CCTGCAATAAGTTATTGGTCAGCCTGGGAGCCCCTAGCTTCTATCTGTGATGGGTGGTGATAAGAACATTTATATTTTCC
TGAATCCAATAGCCTTCCCATACAACCGTATTCCAGTTCCTAGCCTTTCAGTGGGACTTCTTGAATGCTGTCACGTTATG
GTTCTCCACCTGGCTCAGTGTCTTCTCCTGTGTGAAAATCGCAACCTTCACCCACCCTGTCTTCCTCTGGCTAAAGCAGA
TAGTGTCTGCGTTGGTTCCATGGGTGCTGCTCAGCTCCGTGGGGGTCTCCAGCTTCAGCACCATTCTAGTTTTCATAGGC
AACCGGAGAATAGATCAGAACTATTTAAAGAGGGTTCTGCAACCTTGGAATGTCGCTGGGAATGCTGTGAGAACATATGA
GAGACTCTGCTTCTTCCCTTTGAAAATTGTTACCTGGACAGTCCCTACTGTTGTCTTCATCGCTGGCATGGCTTTGCTCA
TTCCACCTCTGGGAAGACACACCAAGCAGGTCTCCCTGTCCATCTCAGGCTCTCACGATCCCAGCACCCAGGCACACATC
AAGGCTCTCATCTCCTTTGCTGTCCTCTTTGTTTCCTATTTTCTGTCACTGGTGCTCAGTGCCTCAGGTGTGTTTCCATC
ACGGGAATTCAGGCACTGGGTGTGGCAGGCTGTGATTTATCTGTGCACAGTAGTCCGCCCCATTGTTCTTTTCTTGAGTA
ACAGCAGGCCGAGAGCTGTGCTAGAGAGGGGCTGCTCCTCAGGGCCATGGGGCATCTTGA
>TutrTAS2R62AP_scaffold_108147:16688-15749
ATGCCCTCCTCACCCATGTTGATCTTCATGGTCATCTTTTTCCTGGAGTTGCTGGCTGCCATGCTGCAGAATGGCTTCAT
AGTTACTGTGTTGATCAGGGAGTGGGTACAATGCCAGACACTGCTTGCAGGCGACATGATTGCGGCGGCCTCCCTGGCCG
CCTCCCGGTTCTGACTGCATGGGATGGCCCTCCTGAACAACCTCGTGGCCTTCTTTGGTTTTGGTTTCAGAATTTACTAT
TTCAGCATCCCCTAGGACTTCATCAACTCTCTCACTTTCTGGCTTACTGCTTGGCTTGCTACATTCTACTGTGTGAAGAT
CTCATTCTTCTCTCACCCCATCTTCTTTGGGCTGAAGTTGAGGATTTCTCGGTCAGTGCCCAGGCTGCTGCTGGGCTCCC
TGATCTTATCTGCTCTGGTAGCCATCCCGTTAGACACTGGGAACACAATTCGTGTGCGGATGGTTGCTGCCCAGAGTTCC
CATGGAAACAGCACCCTGGCTGGTAGAACACAGACTGTCTCTTTGTACTTTTTTCTACCTCGTGTAATTATTATGCGGTC
AATTCCATTTCTCCCGTCCCTGGTGTCCACCCTCTCGTGTTCTCGCTGCGCCGGCATTTGGGGCAGATGAGGGACCATAG
ACCTGGCCCCGAGTGATCCCAGCACCTGGGCTCACACCGTGGCCCTGAAGTCACTTGCCTTCTTCCTCACCTTCTACCAT
CACGTTACCTGTGCCTGATTATCGTTGTTATAAACATCCTAACCCTCTGGAATCACTGGCGCTGGGCCTGGGAAGTGGTG
ACCTGTGCAGGCATCTGTCGGCACTCCAGCATCTCGGTGCACGGCAGCCCCAGGCTGAGAAAGGCCCTGATGACGAGGCC
TTGGAGAGCCCTGGGCAAGGAGCAGTTTGTCTCATCAGAGTCAGTAACTGCTGTCACTGA
>TutrTAS2R62BP_GeneScaffold_59:355654-356498
GCGTCCCCCTCACCCACGTGGACCTGCACGGTCACCTTTCTCCTGGAGTCGGTGGCTGCCAGGCTGCAGAACGGCCTCAC
AGTCGTCGTGCTGAGCCGGACTGGGACGCTGGACGCTGCCGGACGCTGTGCGCAGGCGACGTGATTGTGGCCCGCCTGGC
CGTCTCCCAGTTCTGTCTGCAAGGGATGGCCCTCCAGCGCAACCTCCTGGCTTCCTTTGGTTTTGGTTCCCAATTTTATT
TCAGCATCTCCTGGAGCTTCATCAACACTCTCACTTTCTGGCCGACCAGCTGGCTTGCTGTCTTCTACCGTGTGAAGGTA
GCATCCTTCTCTCACCCCATCTTCTTCTGGCTGAAGTGCAGGATTTCTCGGTCAGTGCCCGGGCTGCTGCTGGGCTCCCT
GATCCTGTCTGGTCTGACATCATCAGCAGCCACCGGGAAGTCAATTCTTGTGCAGATGGTTGCCACCCAGGGTTCCCATG
GCAACGACACCCTCACGCAATTATCATGCGGTCAGTTCCATTCCTCCTGTTCCTGGTGTCCACCCTCTCGCTCGTGTTCT
CGCTGCACCGGCACTTGGGGCAGATGAGGGACCACAGACCCGGCCCGAGTGATCCCAGCACCCGGGCTCACACCGTGGCC
CGGAAGTCACTTGCCTTCTTTTTCATCCTATTTCCTGTGCCTGAGAATTGTCCTTGTGAACATCCCAACCCTCCGGAAGC
ACCGGCACTGGGAAGCGGTGACCTACGCCGGCATCTGTCTGCACGCCAGCATCTTGGTGCACAGCAGCCCCAAGCCGAGA
AGGGCCCCAAAGAAGAGGCTTCGGCGAGCCCTGGGCAAGGAGCAG
>MufuTAS2R1_GL896975.1:8104774-8105670
ATGCTAGAGTTTTACCTTATTATCCATTGCCTTTTTTCGACAGTACAATTTCTCATCGGGGTTTTAGCAAACGGCCTCAT
CCTGGTTGTGAACGGCACCGAGTTGATCCAACAGAGAAAGATGGTTCCGTTGGCTCTTCTTCTTTCCTGCCTAGCGATGG
CCCGGATCTGTCTGCAATTGGTCATCTTCTACATCAACCTGGCTATCCTCCTCTGGATCGAAGTCCCTCTACTTATTGAG
AATTTTTCAATCTTCTTGTTTGTAAATGAATTGGGACTGTGGTTTGCCTCGTGGCTCGGCGTTTTCTATTGTGCCAAGAT
CGCCCCCATAGCTCACCCACTCTTCTTCTGGTTGAAGATGAGGATATCGAAGCTGGTGCCGTGGCTGATCCTAGGGTCCC
TGCTGTACACATCCGCCCCCTCTATCTTCTACAACAAACATATATGGGTTTTTTCCCAACAAGTTTTTTTGGGTTTTTTC
TCCCCAAACACAACAACCCAAATCAAAGAGAAGTCTGCCTTACAGATCGCCTTTCTTGTGAGGTTATCATTGCCACTACT
CATCTTCCTCGCGTCTTCCCTGCTTTTGCTATTTTCCCTGGGGAGACACACCTGGCAGATGAGAAACACGGCCATGGGCA

CCAGGGTCCCTAGCACAGGTGTCCACGTGAGATCGCTGCTGTCGGTTCTGTCCTTCCTGGTCCTCTGCGTCTCCCACTAC
ATGACAGCCGCTTTGCTCTCTTCTCAGATTTTTAAGCTCAGGAGTCTCATGTTTTTGTTCTGTGTCTGGGTGTTTGGGTC
CTTTCCTTCTGGACACTCTATGATCTTAATTTTAGGCAGTCCTAAACTGAAACAAAATGCAAAGAAGTTCCTCCTCCGCG
GGAAATGCTGCGAGTGA
>MufuTAS2R2_GL896907.1:25461110-25460196
ATGGCCTCCTCCTTGTCAGCAATTCCTCATCTTATTGTTATGTCAGCAGAATTTATCACAGGGATTACAGTAAATGCATT
TCTTATCCTCATCAACTGTAAAGAACTGATCAAAAGCAGAAAGCTAACACCAATGCAACTCCTTTTCCTATGTATAGGGA
TGTCGAGATTTGGTCTGCTGATGGTGTTAATGGTGCAAAGTTTTTTCTCTGTGTTCTTTCCACTCTTTTATAGGGTAAAA
GTTTATGGTGCAGCCATGTTGTTCTTCTGGATGTTTTTCAGTTCTGTCAGTCTCTGGTTTGCCACCTGCCTTTCTGTATT
TTACTGTCTCAAGATATCAGGCTTCACTCAGTCCTATTTTCTTTGGCTGAAATTCAGGATCTCAAAGTTAATGAGCTGGC
TGCTTTTGGGAAGCTTTCTGGCCTCCATGAGCACAGCAGCTCTGTGTATTGAGGCTGATTACCCTAAAGAAGCAAACGAT
GATGATGTCCTCAAGAATGCCACACTGAAGAGGACTGAACCCAAGATAAGGCAAATTAACGAAGTACTTCTTGTCAACTT
GGCATTACTATTTCCTCTAGCCCTATTTGTGATGTGCACTTTTATGTTACTCATTTCTCTCTACAAGCACACTCATCGGA
TGCAAAATGGATCTCATGGTGTTAGAAATGCCAGCACAGAAGCCCATATAAATGCATTAAAAACAGTGATGACGTTCTTT
TGCTTCTTTATTTCTTATTTTGCTGCTTTCATGGCAAATATGACATTCAGTATGCCTTATGGAAGTCATTGCTTCTTTGT
ACTAAAGGATATAATGGCAGCATTTCCCTCCGGCCATTCAATTATAATCATCTTGAGTAATTCAAAATTTCAACAACCTT
TCAGGAGACTTCTCTGCTTCAAAAAGAATCAATGA
>MufuTAS2R3_GL896904.1:451353-450403
ATGTCAGGGCTGGAGAAATGGATGTTCCTGGTTCTGTCTGCCGCCGAGTTCATTCTGGGGATGCTGGGGAATGGGTTCAT
AGTGTTGGCCAATGGCAGCAGCTGGTGCAAAAACAAGACAGTCTCTCTGTCTGACTTCATCATCACTAACCTTGCTCTCT
CTAGGATCGTTCTCCTGTGGATTCTCTTGGTTGACGGTGTTTTAATGGTGTTCTCTTTCAAAGTACGTGATGAAGGGATA
GTGATGCAAATTATTGATATTTTCTGGACATTTACAAACCACCTGAGCATTTGGCTCGCCACCTGTCTCAGTGTCCTCTA
CTGCCTGAAAATTGCCACTTTCTCCCATCCTGCATTCCTCTGGCTCAAGTGGAGAGTTTCTAGGATGGTTGTATGTATGA
TTTTGGGTGCCCTGTTCTTATCGTGTGTCAGTGCCCTATCTCTGATCCAGGAATTTAAGATCTATTCTGTTCTCAGGGGG
ATCGATGGCACAGGGAATGTGACCGAGCACTTTAGAAAGAAAAGAAATGAATACAAAGTGTCCCATGTTCTTGGGACTCT
GTGGAACCTGCCTCCCCTAATTGTGTCTGTGGCCTCGTACTTTCTGCTCATCCTCTCCCTGGGGAGACACGTGCAGCAGC
TGCAGCAAAGCGGCATCAGCTCCAGAGATCCAAGCACCGAGGCCCACCAGAGAGCCATCAAAATCATCGTCTCCTTCCTC
TTTCTCTTCCTGCTTTACTTTTTGGCCTTTCTGATTACATCCTGCAATTATTTCATACCAGGAACTGAGATGGTCATTAT
GATCGGAGAAGCAGTTACAATGTTTATCCTGCTGGCCACTCATTCATTCTCATTCTGGGAAACAACAAGCTGAAGCAGA
CGTTCGTGGAGATGCTGTGGTGTGAGCCTGGCCTTCGGAAGCCTGGATTCAAGGGACCTTATGCCCCCTAG
>MufuTAS2R4_GL896904.1:442789-441887
ATGCTTCAGATATTCTTTTTCTTGCCATTATTGTCTCAGCAATTTTGAATTTTGCGGGACTCATTGTAAATCTCTTTTT
TGCAGTGGTCAGTTTTCGGACGTGGCTCAAAAGCCCCAGAATCTCCTCTTCAAATAGGATCCTCTTCAGCTTGGGCGTCA
CCAGATTTCTTACGCTGGGACTGTTTTTACTCAACATCAGTTACTTCTTCATCTCTCCAAATGTGGCAAGGTCAGTGCAC
TTATCTACTTTTTTCCTGTTGTGTTGGATGTTTTTGGACTCGAATAGTCTCTGGCTTGTCACCTTGCTCAATGCCTTGTA
CTGTGTGAAGATTACGGACATGCAACACGCGGTATTTCTCCTGCTGAAACGAAATCTCTCCCCAAAGATCCCCAGGCTCG
TGCTAGCCTGTGCGCTGATTTCTGCTTTCACCACTCTCCTGTATGTGGCGCTCAGACAGACGTCGTGCTTTCCCGAATTT
GTGCCTGGGAGGAATGGGACAGGATGTGACATCAACGAGGGCGTCTTGTCGTTGGTGATCTCTTTGGTCTTGTGCTCGTT
TCTCCAGTTCATCATTAATGTGACTTCTGCTTCCTTGTTAATTCATTCCTTGAGGAGACATATACAGAAGATGCAGAAAA
ACGCCACTCTTTTTTGGACTCCCCAGACTGAAGCTCACCTGGGCGCTATGAAGCTCATGATCTGTTTCCTCATCTTGTAC
ATTCCCTACTCGCTCGCTTCTCTACCGCACTATTTCCCCTTTGTCGGGATGGATTTGGGAGCCAGATCTATCTGCATGGT
TATTTCCACCATTTACGCCCTAGGACATTCCGTTCTCATTATTCTCACACACCCTACACTGAGAACAAAAGCAAAGGCGA
TTCTTTGTTTCAACAAGGAGTAG
>MufuTAS2R5_GL896904.1:430537-429650

ATGCTGAGTGCTGCCCTAGCACTGCTGATGGTGGTGGCGGTGGCCGAATTTCTCATTGGGCTGGTTGGAAATGGTGTCCT
TGTGGTTTGGAGCTTTGGAGAATGGGTCCGAAAATTCAATGGGTCCTCATACAACCTCATTGTCCTGGGCCTGGCTGTCT
GCCGATTTCTCCTGCAGTGGCTGATTATGATGGATTTAAGCCTGTTTCCACTTTTCCACAGTAGCCACTGGCTTCGGTAT
CTCAGTGTCTTCTGGATCCTGGTAAGCCAGGCCAGCCTGTGGTTTGCTGCTTTCCTCAGTGTTTTCTATTGCAGGAAGAT
CATGACCCTTGAGCACCCTGTCTGCATGTGGCTGAAGCAGAGAGCCTATTGCCTGAGTCTCTGGTGCCTTCTGGGGTACT
TCATGATCAGTTTGTTACTTGTAACCCTCATTGGCTTAAAGCCCTATGATCCTTCCCAAGGCAACAGCAGCATTCTGTAC
CCCTTTGAAAGCTGGCACTACCTGTATGTATTAAAGCTTAATGCAGGAAGTGGGTTGCCTCTGATGGTATTTCTTGTTTC
TTCCGTGATGCTGATTATCTCTTTGTACAGACACCACAAGAAGATGAAGGTATATACAGCTGGTAGGAGAGATGCTCGGG
CCAAGGCTCACATCACTGTCCTGAAGTCCTTGGGCTGCTTCCTTATCCTTCATGTGGTTTACATCCTGGCCAGCCCTTTT
TCCATCATCTACAAGTCTTCTGCTAATCTCCTCATTATCTTCATCTCTGAGACAGTCATGGCTGCCTATCCTTCTCTTCA
TTCTGTCATATTGATCATGGGGAATCCCAGGGTGAAGCAGACTTGTCAAAGAATTCTCTGGAAGATGGTGTGCGCTTGGA
GATCCTAG
>MufuTAS2R7_GL896948.1:12161144-12162082
ATGCCGGATAAAGTGGAAACTACCTTAATGCTCATAGCAGCTGGAGAGTTTTCAATGGGGATTTTAGGGAATGCATTCAT
TGGATTGGTAAACTGCATGGGCTGGATCAAGAATAGGAAGATCACCTCCATTGATTTAATCCTCACAAGTCTGGCCATAT
CCAGAATTTGTCTATTATGTATAATACTATTAGATTGTTTCATATTGGTGCTGTATCCAGATGTATATACTGCCGGTAAA
CAAATGAGAATCATTGACTTCTTCTGGACACTAACCAACCATTTAAGTGTCTGGTTTGCCACCTGCCTCAGCATTTTCTA
TTTCCTCAAGATTGCAAACTTTTACCATCCCCTTTTCCTCTGGATGAAGTGGAGAATTGACAGCATGATTCCTAGGATCC
TGCTGGGGTGCTTGGCCGTCTCTGCATTTATTAGCCTTGTTGTGACTGAGAATTTGAATGATGATTTCAGGTATTGTGTT
AAGACAAAGAGGAAAGCAAATGTAACTGTGAGATGCAGAGTAAATAAAGCTCAGTATGCTTCCATCAAGATTTGCCTCAA
CCTGTTAACGCTCCTCCCTTTTTCTGCATCCCTGATCTCATTTCTCCTCTTGATCCTCTCCCTCTGGAGACATAACAGGC
AGATGAAGGTCAGTGCCACCCGGTGCAGAGACTTCAACATAGAAGCCCACGTGGGAGCCATGAAAGCTGTCATCTCCTTT
CTCCTCCTTTTCATTGCCTACTATTTGGCCTTTCTCGTAGCTACCTCTAGCTACTTTATGCCAGAAACTGAATTAGCAGT
GATCATTGGTGAGTTAATAGCTCTAATCTATCCCTCAAGCCATTCATTTATCCTAATTCTGGGGAGCAAAAAACTAAGAC
AGACATCACTAACAGTGCTATGGAAAGTAAAGTATATTCTGAACAGAAGAAATTTTTAA
>MufuTAS2R9_GL896948.1:12153680-12154675
ATGCTAAGTACAATGGAGGTAATATATACCATCTTGATCGCTGGTGAACTGACAATAGGAGTTTGGGGAAATGGATTTAT
TGTACTGGTTAACTGTACTGGCTGGATCAAAAGGAGAGATATTTCCATGATAGACGTCATCTTGGTGAGCTTAGCCTTCT
CCAGAATCTGTTTGTTGTGTGTGATATCTTTAGATGGCCTTCTTATATGCATCTCTCCAGATACATATGCCAATAGCAAG
TTAACGAGCATTGTGGATGTTTTCTGGACACTTAGCAATCATTCAAGTGTCTGGTTTACTTCTTGCCTCAGCATCTTCTA
TTTACTGAAGATAGTCAATATATCCCACCCACTTTTCCTCTGGCTGAAACTAAAGATTAACAGAATCATCCTGGGGATTT
TTCTGATGTCCTTCCTTAGCTGTATAATCATTAGTGTTTCAATGAATGAGGACTTCTGGGATCCCTTCGAAGTCAATCAT
AAGGAAAACACAACTTGGGAACTGAAAGTGAGTAAAATCCCAAGTGCTTTCAAACTGCTTTTCCTGAACCTGGGGGTTAT
CATTCCCTTTGTTCTTTGCCTAACCTCGTTTCTCTTATTACTTTTCTCTCTAATTAGACACACCAAGCAGAGAAAACTTT
ATGCCACAGGGTCCAGAGACCCCAGCACAGAGGCCCACATGAGGGCCATAAAGGCACTGATCATCTTTCTGCTTCTCTTC
CTTATGTACTATGCAGTCTTTATTATACTAACCTCTAGCTTACTGATTCCTCAGGGAGAATTAGTGGTGATGTTTGGTAG
TGCTATAGCTGTCATTTTTCCATCAAGCCATTCATTCATCCTGATAATGGGGAACAGCAAACTGAGGAAGGCTTTTCTGA
AAGTGCTAAGGGTTGTGAAAAGTTTGCACAGAGGAAGAAAACCTTTTGTTCTGCAGAGAATCCTGAATACAAGGGGAAAG
AAATCAACAAAAGATCCTCTCCCTTCTCCCAATTAA
>MufuTAS2R10_GL896948.1:12142458-12143423
ATGATAAGCATACTGGAAGGCCTTCTCATTTTTATAGCAGTTAGTGAAGCAGTACTGGGAGTTCTAGGGAATGGATTTAT
TGGACTTGTCAACTGTGTAGACTATGTGAAGAACAAAAAGTTTTCGATGATTGGCTTTATTCTCACTGGCTTAGCTACTT
CCAGAGTTTTTCTGATATTGTTAATAATTACAGATGGACTTATAAAGCTATTCTCTCCAGATATGTATTACTCTGGCAAA
CTAATTGATTTTATTAGTTACTCATGGATAATTATCAATCAATCAAGTATCTGGTTTGCCACCAGCCTCAGTATCTTCTA

TTTCCTGAAGATAGCAAATTTTTCCCACCATATTTTTCTCTGGCTGAAGGGTAGGATCAATAGGGTTCTTCACCTTCTGA
TGGGATCCTTGTTTATTTCATGGTTATTTACTTTTCCACAAATTGTGAGGATTATTGATGATAACAGAATGAGGAGTGGA
AATACAACCTGGGACTTCAACATGCCAAAAAGTATGTTCTTTACTAAGCAGATTTTGGTCAACCTAGGAGTCATTCTTCT
CTTTATACTCTGCCTGGTTACATGTTTCTTGTTAATCGTTTCCCTCTGGAGACACAGCAGGCGCATGCAAATGAAGGTCA
CTGGACCCCGAGACCCCAGTACAGAAGCACACGTGAAAGCAATGAAAGTTTTAATATCTTTTATCTTTCTCTTCATCTTG
TATTTTATAGGCATTGTCATAGAGATAGTATGTTTCAATATGCCAGAAAACAGACTACTGTTTATTTTTGGCATGACGAC
TGCAGCCATCTATCCCTGGGGTCACTCCCTTATCCTAATTCTAGGAAACAGCAAGCTGAAGCGAGCCTCTTTGAAGGCCC
TGCAGAGATGCAAGTGCTGTGAGGCCGGGACACGGCTCACAGCTGACAGACCCACGTGGGGCCAAATGGATGTTCTAGGA
GAATAA
>MufuTAS2R12_GL896948.1:12121967-12122902
ATGGCAAGCACATTCAAGAATGTATTTACGATGATTTTTGCTGGAGAATTCATAGCGGGGCTTTTGGGAAATGGGTTCAT
TATATTGGTTAACTGTATTGATTGGATCAGGAGCTGGAAGTTCTTCTTGATTGACTTTATTCTTACCTGCTTAGCTATTT
CCAGAATGCTTCTTCTCTGTATAATAATGCTAGGCATAGGTATAGACATAATTTGTGAGGAAATATGCTACAATGACAAT
CAACTGATGATTTTTGAAATCCTCTGGACAGGATCGAATTATTTCTGCATAACCTGTACCGCCTGTCTCAGTGTCTTCTA
TTTGCTTAAGATAGCCAACTTTTCCAATCCCATTTTCTTCTGGATAAAACAGAGAACTCACAGACTGCTTCTCATTATTG
TCCTTGGAGCGGTCCTCTCTTTCTGTTTGTCCCTGCTTTTTAAGGATACAGTATTTAAGAACCTGATCAAAACCTGGGTA
AACCCTGACAGCAATCAGACATTGAACTTCACAGTAAGAAAATATGATTTATTAACTTCTAATATAGTCCTGAACATAAT
GTTCATCATCCCCTTTGGAGTGTCTCTGGCTTCCTTTGTCCTTTTGATCCATTCCTTATGGAAACACACCAGGCGGATGA
AGGGCACAGGTTCTGGGGATCTTATCACCAAGGCCCATGTGCGAGCCATGAAGTCTATGATTTCATTCCTACTCTTCTTC
TTTATGTACTGGTTGAGCAATATTATAATATATTTGGTCTATGTCATGCTAGACAGTTTGGTGGCAAAAATGTTTGCTTA
TATGTTTGTATTTTCCTATCCATCTGGCCATCCATTCCTTCTGATTTTATGGAACAGCAAATTGAAACAGGCTTCTCTCT
GTGTCCTGAGGAAGCTGAGGTGGTGCAGGAATGTCAGGAAATCTGCAAATCCATAA
>MufuTAS2R38_GL896904.1:261720-262724
ATGTTGACTCTGACTCCTGTCATAACTGTGTCCTATGAAGTCAAGTGTGCATTTCTATTGCTTTCAGCCCTGGAGTTTGC
AGTGGGGATTCTGACCAATACCTTCATTTTCTTGGTGAATATTTGGGACGTGGTGAGGAGGCAGCCACTGAGCAACTGTG
ATCTTATCCTTCTGAGTCTCAGCCTCACCCGGCTTTTCCTGCATGGGCTGCTGTTCCTGGATGCCCTTCAGCTTATATAC
TTCCAGCGCATGAAGGACCCACTGAGCCTCAGCTACCAGATCATCGTCATGCTCTGGATGGTCACAAACCAAGCTGGTCT
GTGGCTCACCACCTGTCTCAGCCTTCTCTACTGCTCCAAGATTGTCCGTTTCTCTCACACCATCCTGCTCTGCTTGGCAA
GCTGGGTCTCCAGGAAGGTCCCCCAGATGCTCCTGGGCGCCATGGTTTTCTCTTCCATCTGCACTCTCATCTGTTTGGGG
GATTTTTTTAGTAGATCAGGCTTTGGGTTCACAACTACGCTCTTCATGAATAATACAGAACTCAATTTGCAAATTACAAA
ACTCAATTTCTATTATTCCTTCATCTTCTGCACCTTGGGGTCCATCCCGCCTTTCTTGCTTTTTCTGGTTTCTTCTGGGG
TGCTGATTGTCTCTCTGGGGAGGCACATGAGGACAATGAAGGCCAAATCTAAGGACTCCCATGACCCCAGCCTGGAGGCC
CATATCAAAGCACTCAGATCTCTCATCTCCTTTCTCTGCCTCTACGTGGTGTCCTTCTGTGCGGCCCTCATTTCAGTGCC
TTTACTGATGCTGTGGCGCAACAAGATCGGGGTAATGATATGTGTAGGGATCTTAGCAGCCTGTCCCTCGATACACGCAG
CAATCATAATCTCAGGCAATGCAAAGCTAAGGAGAGCTGTGGAAACCATTCTACTCTGGATTCAGAGCAGCCTAAAGGTA
AGGGCAGACCACAGGGCAGATCCCAGGACTCCAGATCTATGCTGA
>MufuTAS2R408A_GL896948.1:12104620-12105543
ATGGTAACTTTTCTACTGAACATTTTAGAAATCCTCATAATAACAGAATTTGTTCTAGGAAATTTTGCCAATGGCTTCAT
AGTGGTGGTGAACTGCATTGACTGGGTCAAGAGACAAAAGATGTCCTCAGCTGATCAAATTCTCACTGCTCTGGCGATCT
CCAGAATCGGTTTGCTCTGCGTAATGTTAATAAATTGGTATGGAACTGTGTTGAATCCAGATTTATTTAGATTGGAAACA
AGACTTCTGGTTCATATTGCGTGGACGGCAAGCAATCATTTAGTGTCTGGCTTGCTACTTGCCTCAGCATATTTTATTT
GTTCAAAATAGCCAATTTCTCTAGCTTTATTTTTCTTCACCTCAAGTGGAGAGTTAAAAGTGTAATTCTTGTGATACTGT
TGGTGTCTTCGTTCTTTTTGGTTTTTCATGTTGCGGTGGTAAGCATATATGAGCTGACGAAGGAATATGAAGGAAACATC
ACTAGACAATCCAGATTGGTGAACATTGCATGCCTTTCAAATATGACTGTATTCACGTTAGCAAACTTTGTGCCCTGTGC

TATATCGCTGGCATCTTTTCTGCTGTTAATCTTTTCCCTATGGAAACATCTCAAGAAGATGCAATCTGGTGGTAAAAGAT
GCCAAGATCCCAGCACCAAGGTCCACATAAGAGCCATGCAGACTGTGATCTCCTTTCTCTTGCTATTAGCTGGTCACTTC
CTGACTCTAATTGTCACTGTCTGGAGTTCTGAGTGGCTGCCGACCAAACTGTTCTACGTGTTTTGCCAAACTTTTGGATT
TGTGTATCCTTCAAGCCACTCATTTATCCTGATCTGGGGAAACAAGAAGCTAAAACAGGTCTTTCTGTCTATTTTGTATC
AGGGGAAGTACTGGCTGAAAGAACAGAAACTCTCAACTCCATAG
>MufuTAS2R408B_GL896948.1:12075997-12076908
ATGGTAACTTTGCTACCTGACATTTTCTCCATCTTTGTAATAACAGAATTTATTCTAGGAAATTTTGCCAATGGCTTCAT
AGTGGTGGTGAACTGCATTGACTGGGTCAAGAGACAAAAGATGTCCTCAGCTGATCAAATTCTCACTGCTCTGGCGATCT
CCAGAATCAGTTTGCTCTGCGTATTGTTAATAAATTGGTATGTAGCTGTATTGAATCCAGATTTATTTAGATTGGAAACA
AAACTTCTGGTTCATATTGCGTGGACGGCAAGCAATCATTTTAGCGTCTGGTTTGCTACTTGCCTCAGCGTATTTTATTT
GTTCAAAATAGCCAATTTCTCTAGCTTTATTTTTCTTCGCCTCAAGTGGAGAGTTAAAAGTATAATTCTTGTGATACTGT
TGGTGTCTTCGTTCTTTTTGGTTTTTCATGTTGCGGTGGTAAGCATATATGAGCTGATGAAGGAGTATGAAGGAAACATC
ACTAGACAATCCAGATTGGTGAACATTGCACGCCTTTCAAATATGACTGTATTCACGTTAGCAAACTTTGTGCCCTGTGC
TATATCGCTGGCATCTTTTCTGCTGTTAATCTTTTCCCTGTGGAAACATCTCCAGAAGATGCAATCCAGTGGTAAAAGAT
GCCAAGATCCCAGCACCAAGGTCCACATAAGAGCCATGCAGACTGTGATCTCCTTTCTCTTGCTATTAGTAGGTTACTTC
CTGACTCTAACTGTCACAATCTGGACTTCTAAGTGGCTGCCGAACAAGTCAGCCCTCCTGTTTAGCAAGGCTATTGGAAT
CTTGTATCCTTCAAGCCACTCATTTATCCTGATTTGGGGAAACAAGAAGCTGAGAAAGGCCTTTCTGTCATTTCTGTGGC
AGCTGAGGTACTGGCTGAAAGAAAGGATGTAA
>MufuTAS2R42_GL896948.1:12030812-12031783
ATGTTAGCTGGATTGGATACAATCTTTCTCACACTGTTGACATCAGAATTCATAATTGGAATGTTGGGGAATGTGTTTAT
TGGACTGGTAAACTGCTCTGAATGGGTCAGGAACCGAGAGATCTCTTTAGCTGACTTCATCCTCACTTGCTTGGCAATCT
CCAGAATCTCGCAGCTGTTGGTGTCATCGTTTGAATCATTTATGATGGGACTAAGTCCACCTTTCTATCCCACTCATAAA
CTAGCAAAACCTGTTACTTTGCTTTGGAGAATAACTAATCATTTGTCCACCTGGTTTACTACCTCCCTAAGCATTTTCTA
CCTCTTTAAGATAGCTCAGTTCTCCCATTCCCTTTTCCTCTGGCTGAGGTGGAGAATGAACAGAGTGGTTCTTGCAATTC
TTGTATTTTCTTTGTTCATTTTACTGTTTGACCTTCTATTGCTAGAAACATTTAATAATTTCTTCTGGAATATCCATGCA
ATGGATGAAAGTAATCTGACCTTACATATAAATGAAAGTAAAACTTCTTATATTAAAACCCTAGTTCTTCTTAGTATTTC
CTATATCATTCCTATTGTTCTGTCCCTGATCTCATTGCTTCTTTTATTTCTGTCGTTGGTAAAACACATCAGGAATTTGA
AGCTCAACTCGATGGGCTCCAGGGACTTCAGCATACAGGCCCATAAAAAGGCCATTAAAATGGTGGTGTCTTTCCTCTTC
CTTTTCACAGTTCATTTTTTGTCTATACAGTTGTCAAATTGGATCCTTTTTTTATTTTGGAACAACAAGAGCGCAAAGTT
TATCATGTTGGCTGTATACATCTTTCCCTCAGGCCACTCATTAATTTTGATTCTGGGAAACAGCAGGCTAAGACAGACAG
CCTTGAATGTTCTGTGGCATCTTAAAAGCTTCCTGAAAAGAGAAAAAGCAATTTCATCTTTACAGATAAACCTTCCAGAG
CCTTTCCAATGA
>MufuTAS2R67_GL896948.1:12039519-12040454
ATGCCATCTGGAATTGAAAATGCTTTTCTGATAGCAGCCACAGGCGGATTCCTAACTGGAATGTTGGGGAACAGTTTCAT
TGTACTAGTTAACTGCATTGACTGGGTGAAGGGTCAAAAGCTCTCATCAGCTGACTGCATTCTCACCAGCCTGGCTATCT
CCAGAATCGTTCTTCTTTGCATAATACTGTTAGATTCATATTTAATGGTGGTGTGGCCGCATCTGTATATCATTGATAAT
ATAGCAAAATTCGTTAATAGTTTTTGGACACTAAGCAATCACCTAGCTACTTGGTTTGCCACCGGTCTAAGTGTCTTCTA
CTTCTTTAAAATAGCCAATTTCTCCCACTCCTGTTTCACCTGGCTGAGGAGGAGAATTAGCAGAGTCCTACTCGTGCTTC
CCCTGGGGTCTTTACTCTTACTGCTTTGCAACTTTGAATTAACAGATACATTTAGTAATTTCTCGGTTAATGTCTATCAA
AGATACGAAAGAAACTCAACTTGGTCCCTAAATGTAAGTAAAACTCTGAATCTTAACAGCTTGATTGTTTTCAGTTTCAC
CTACTTAATCCCCTTTCTTCTGTCCCTGGCTTCCCTACTCCTTTTGTTTCTTTCCCTGAGGCGACATACCAGGAATGTGC
AACAGAACTGCTCTAGGGACTTCAATACAGAGGCCCATCAAAGGGCCATGAAAATGGTGATGTCTTTCCTTTTCCTCTCC
ACAGTTCATTTTTCTTCCATCCTATTAACAGGTTGGATTTTTGTTTTGCTGCAGAATTCTCAGGTCAATTTGGTGGCCAT
ATTATTGTCAACTCTTTTTCCTTCAAGCCACTCATTTATTCTGATTTTGGGAAACAGCAAGTTGAGAAAAACTGCTTTAG

GACTACTGTGGCATATTAATCACCACCTGAAAACAGTGAAACCTTTCATTCCATAG
>MufuTAS2R8P_GL896948.1:12156521-12157449
ATGCTCAGTATGGAAGACAACATCTTTGTGATCATTATAGCTGAAGAATTCATAATAGGAATGTTGGGGAATGTATATAT
TGGACTAGTAAACTGGATTGACTGGATTCAGAAGAAAAAGATCTCCTCAGTTGACTATATCCTCACCAGTCTAGCCATCT
CCAGAATTTGTTTGCTCTGTATATTGATACTAAATGTCATCATAATGATATGCTACCCAGATTTTTATGAAAGTGATAAA
CTACAGACATTCATTAGTATCTTCTGGACACTCACCAACTATCTAAGTACGTGGTTTGCCACCTGCCTCAACGTCTTCTA
TTTGCTCAAGATAGCCAATTTCTCCCATCTGTTTTTCTCTGGCTAAAGCGAAGAATAGACAGAGTGATTCATTGGATTC
TCCTGGGTTGTTTGGCCGTTTCGTCTTTGATCAGCCTTATACTAGCAATGACACCAGATTATGATCATGAGTTTCATAAA
ATTGTAAATCCTAAAAGAAACTGCACTGAAATGTTCAATGTGAGTAAAAGTCAGTTCTTCCACTCATTGATTCTCTTCAA
CCTGTTGGCAGTTGTCCCATGTACTGTGTCATTGATCTCATTTTTTCTTTTAGTTATGTCCCTATGGAGACATATCAAGC
AAATGAAACTCAACGTTACAGGCTGCAGAGACCCTAGCACAGAGGCCCATGTGGGAGCCATGAAAACTATGACTTCATTT
CTCTTCCTCCTTTTTGTATACTATGGGGCTTCTCTTTTGGCGACTTTTAGCTAGCTTATGAAAGAAAGGAAGTTAGTTGT
GATGTTAGGAGAAATGATAGCAATTCTCTATCCTTCTGGTCATCACTTATTTTAATTATTAGAAATAACAAGCTGAGGCA
GGCATCTATCAGGATGCTGAGATATGGGAGAACATTCTGCATGTTGTAA
>MufuTAS2R16P_GL896904.1:15676008-15676901
ATGGCCTTCCCCCTCCAACTCACTGTTTCTTCATAATCATCTATGTGCTCGAATCCTTGACAGTAATTATGCAGAGCAAC
TTAATTTTTGCAGTGCTGAGCAGAGAGGCTGTCATCGATGGACTTGTTTCCCACCTGCCTGGGCATCTGCTGCTTCTGTC
TACAGTGCGTGTCAGTGCTGAACAATTTTTGCTCCTATTTTAACCCTAACTATGTATTTTGATACCGATCAATCACCTGG
GAATTTACTAGTACTCTTACGGGTTAACCAGCTTGCTCGCTGTCTTCCACTGCATCAAGGGTCAACTCTTTTACCCACCC
ATCTTCCTCTGGCCAACGTGGAGACTTTTCAGGTTCGTTCTGCGGCTGTTGCTAAATGCTCTGCTGATTTCTTGTGTGTC
AATAATCACCATCTCAGCTACTAGGAATTATATGAATGTTCACTTAATCACTGTGGGGCTTTTCTCTATAAACAGCCCTA
GGATTGAGAGACTTAAGACGTTCCACCTGTATTTTACCATATCTTATCAAACAGTTGCAATTTTTATTTCTTTCCTCCTA
TCCTTGGCCTCCACCATGTCACTCAGCCTCCCTGTTCCAACACATGGAGTAGATGCAACACCACAGCTCTGGCCACTAGA
ACTACAGTTCTAGCATGGAAGCTCACACCACTGCCCTAAAGTCTTTTGCCATCTTCCTCATTTTCTTCACCTCTTACTTT
CTGACCCTACTCATCTCTGTTATGAGCATCTCATTGGACAAGAGGTCTTGGTTCTGGGTCTGGGAAGTTGTCATCTATGC
AATAGTCCCTATTCACTCCTCTTCACTAATGCTGAGCAGCTCTAAATTGAAAGAGGTTTTAAAAGTAAGGTGCTGGGGCC
TAGAGGCTGCCCGA
>MufuTAS2R39P_GL897291.1:40743-39785
ATGACCAAAACCTGCAATCCCCCAGAAAATGAACTGTCACCATTTCAGATCTTACCGATTTTAACAATTATAGGCACTGA
ATGCATCACTGGTATCATTGCAAATGGGTTCATTATGACTGTAAATGCAGCTGAGTGGATTAAAAATAAGGTAATTTCCA
CCTGTGGCAGAATCCTGTTTTTCTTGAGTGTATCCAGAATAGCTCTCCAAAGCTTCATGATGCTAGAGTTTACCTTCAGC
TCAACATTCCCACGTTTTTATAATGAAGACCTTATATATGACATGTTCAAAGTAAGTTTCATGTTCTTAAATCATTGTAG
CCTCTGGTTTTCTGCCTGGCTCAGTTTCTTCTACTTCGTGGAGATTGTTGATTTCTCCCACCCCCTTTTCCTCAAGCTGA
AGTAGAGAATTTCCAGATGGATGCCCTGGCTTCTGTGGCTTTCAGTGTCTATTTCCCTGGGCTACGGTGGGCTCTTCCCT
AAAGACATCTACAGCGTGTATCACAATTCTTCTATCCCTTCCTCCAACTCAAGAAAAAATACTTCACTGAGACCAATATG
GTCAACCTGGTTCTTCTCTATAACTTGGGGGTCTTCATTCCTCTGATCATGTTCATCCTTTCAGCCACCCTGCTGATCAT
GTCTCTCAAGAGACACACCCTACACTTGAAAAGCAATGCCACTGGCTCCAGGGATCCCAGCATGGAGGCTCACATGGGGG
CCATCAAAGCTACCAGCTACTTTCTCATTCTCTACATTTTCAATGCAGTTGCTCTATTTCTCTATATGTCCAACATCTTT
GACATCAACAGTTACTGGAATATTTTGTGCAGATTTATCATGGCTGCCTACCCTGCTGGACACTCCATTCTGCTGATTCG
GGACAACCCTGGGTTGAGAAGAGCTTGGAAGCAGGTTCAATCTCAAGTTCATCTTTACCTAAAAGAGCAGACTCCATGA
>MufuTAS2R40P_GL897291.1:15393-14439
ATGGCCACGGTGAGCACAGATGCCACAGATAGAGACACATCCAGGTTTAAAATCATCCTCACCCTGGTGGTCTCTGGAAT
AGAGTGCATCACTGGCATCATTGGGAACGGCTTCATTACAGCTATCCACGGGGCCAAGTGGGCCAGAGGCAAAAGACTCC
CTGTCAGTGACTGCATTCCGCTGATGCTCGGCTTTTCCAGGCTCTTGCTGCAGATCTGGATGATGCTGGAGAATACTTAC

AGTCTACTCTTCCTGGTCACTCATAACCAAACCACAGTGTTTATACTCTTCAAAGTCGTCGTCATGTTTCTGAGCTATTT
CAACCTCCGGCTTGCTGCCTGGCTCAGCATCTTCTGTTGTCTTAGAATTGCAAACTTTGCTCACCCTTTGTTGGCTTCTG
GGACTATCACCATTCATCTCCTTATGCTTCAGCTTTCCCTTCTCTATAGATACCTTCAAAGTGTATGGAAGTAGTTCTAT
TCCAGAGAGGAAATGGTAGTAGAAATGGTAGTAGTTCCATTTCCACTGAGAGGAAATACTTCTCTGAGACCAATATGGTT
AACCTGGTTCTCTATAACCTGGGGGTCTTCATTCCACTGATCATGTTCATCCTTTCAGCCACCCTGCTGATCCTCTCTCT
CAAGACACGTACTCTACACATGGAAAGCCATGCCACTGGCTCCAGGGACCCCAGCATGAAGGCTCACATGGGGGCCAACA
AAGCTATCAGCTGCTTTCTCGTTCTCTACATTTTCAATGCAGTTGCTATTTATTTCCATGTCCAACATCTTCAACGTCAA
CAGTTCCTGGAATATTTTGTGCAAAAAGCGTCATCATGGACGCTTCTCCAGCTGGCCACTCAGTGCTACTGATCTTGGGT
AACCCTGAGCTGAGAAGAGCTTGGAAGAGGTTTCAGCACCATGTTCATCCTCACCTGTAAGAGCAGACTCTGTGA
>MufuTAS2R41P_GL897067.1:175632-176543
ATGTAGCCAGCACTCTCAGCCTTCTTCATGCTCCTCTTTGTCCTTCTGTGTCTCCTGGGAATCCTGGCCAATGGCTTCAT
TGTGCTGGTGCTGAGCAGGGAAATGATGCGGCGTGGGAGGCCGCTTCCCTCTGACATGATTCTCATTAGCTTGGGTGCCT
CTCGCTTCTGCCTGCAGTGGTTGGGATGGTGAACAGCTTCTACTACTTCCTCTACCTGAATGAGTACAGCAGTGGTCCTA
CGCGGCAGCTCATTGGTCTCCACTGGGACTTCCTGAACTCGGCCTCCTTTTGGTTCAGCTCTTGGCTCAGTGTCCTCTTC
TGTATGAAGATTGTTAACTTCACCCACCCGACCTTCCTTTGGCTGAAGTGGAGGTTGCCAGGGTCAGTGCTCTTCATGGC
TTCTCTCCTGATCTCTTTCAGCATCACCCTGCTCTTCTCTTGGGGAGACCACGCTGTGTATCAAAGATTTTTAATTAGAA
AATTTCCTGGGAACATGACCAGGAGTGGAGCAGGAGGCTGGAAGTTCACTATTTCTTGCCCCTGAAACTTATCACCTTGT
CGGTTCCCTGCTCTGTCTTTCTGTTCTCAATTGCGCTGTTGATTCTCTGAGGTGACATACAGGGAGAATGCAGCACAGCG
CCCATAGCCCACAGGACCCCAGTGGCCAGGCTCACACCAGAGCTCTGAAGTCTCTCATCTCCTTCCTCATTCTTTATGCT
CTGTCTTTTTCATCCCTGGTCATTGATGGTGTGGGTTTCTTCTTCTAAGAGAATGACTGGTACTGGCCATGGCAGATTTT
AACCTACCTGGGCACATCCGTCCATCGCTACATCCTCATTCTCAGCAACCTCCAGCTTTGAAGGTTGTGCAGGTAGCTAC
TTCTGTTAGCCAGGGGCTTCCAGCTGTCCTAG
>MufuTAS2R62P_GL897067.1:142035-142937
ATGTCCTCCTCACCTGCATTGATCTTCATGGTCATCTTCTTCCTGGAGTTGATGATTGCGATGTTGCAGAATGGCTTCAT
GGTTATTGTGCTGGGCATGGAGTGAGTGTGATGTGGGTGCTGCCTGCAGGTGACATGATTGTGGCCTCCCTGGCTGCCTC
CCAGTTCTGCCTGTATGGGGTAGCCATCTTGAATAACCTCTTGACCTTCTCTGAAATAGACTATTACCAGATCCCCTGGA
ACTTCATCAATACTCTCACTTCCTGGCTCACTGTGTGGCTTGCCGTCTTCTACTGTGTGAAGATCACTCTCTTCTCCTAC
CCTGTCTTCTTCTGCCTGAAGTGAAGGATTTCTCCGTCAATGCCCAGGCTGCTGCTGGGCTCTCTGGTCTTAGCTGGCCT
GAGAGTCATCTCATCAGCCACTGGAATGAGAATTTTTATGCAGATGCTTACCCCCCAGAGTTCCCAAGGAAATAGCACCC
TGGCTGACAGTACAGCCCTTCTATTGGTCTCTTGTTCTACCTCATACAGTGCTCACATGGTTGATCCCATTCTTCCTATT
CTTGGTGTCCATGCTCTTGTTCATGTTCTCACTGTACCAGCACTTGGGGCGGATGAGGGAATGTAGACTGGGAGCATGTG
ATCCTAGTACCCAGGCTCACACCATGCCCCCGAAAGTCACTTGTTTTCTTCCTTGTCTTTTATATATCATATTTCCTGTC
TCTGATTGTTGTTGCTATGAAAATCAGTCTTCACTGATACTGGTCCTGGGTCACTGGTACTGGTCCTGGGAAGTGGTGAC
CTATGCAAGCATCTGTTTGCACTCCAGCATCCTGGTGCTAAGCAGCCCCAAGCTGCGAAAAGTCCTGAAGACCATGCTTT
GGAAAGCTCTGGACAAAGGCTGG
>CahiTAS2R1_chr20:63009951-63010841
ATGCTGGAGAGTCACCTTGTCAGCCACCTTGTTTTGACAGTGATACAATCTCTCTTTGGGATTTTAGTAAATGGCATCAT
TCTGATTGTGAACGGTACTGACTTGATCAAGCAGAGAAAGTTGATCCCACTCGATCTCCTTGTTTCCTGCTTGGCGATTT
CCAGGATGGGAATTCAGCTGGTCTTCTTCTACATTAACCTGGCTCTTCTTTCCTTGGTCAAATTCCCTCCAGTTATTGAG
AAGCTTGTAGTTTTCACATTTGTAAATGATTTGGGACTTTGGTTTGCCACCTGGCTCAGTGTCTACTACTGCATCAAGAT
TGCTACCATCGCTCACCCTCTCTTATTCTGGTTGAAGAGGAAGATCTCCAAGCTGGTTCCTTGGCTGATTCTTGCGTCCC
TGCTGTATGCATGTAGTACTTCTGCTGTGCATGTCAAATATAAGTGGGCATTTTATGGAGAAGGCTTCCTGGACCTTTTC
TTCCCAAATGTAACAACTCACATCAAACTAACCCCTACTTTACAGTCTGCCTTTCTGCTTGCTGAGTTTGCATTGCCGTT
TTTCATCTTCCTGATTTCTTCTCTGCTCTTGATATTTTCCCTGGGGAGACATGCCTGGCAGGTGAGAAACACATGGACAG

GCCCCAGAAACCCTCACACACATGCATACCTCAGGGCCTTTCACTCCATCCTGTCCTTCCTGGCCCTCTATCTCTGCCAC
TACCTGATCGTTGCTTTGATCTTTTTTCAAATTTTTAACCTTAGAAGCTTCCTATTTCTGTTCTGCACCTTCATGGTTGG
TTCATACCACTCCATCCACTCTATTACTTTAATTTTAGGAAACCCCAGAATGAAACAAAATGCAAAGGCGTTGCTCCTCC
TCAGAAAGTGA
>CahiTAS2R2_chr4:18432591-18433499
ATGATCTCTTTGTCAGTTATTCCACATGTTATCATCATGTCTGCAGAATTTATTACAGGGGTTACAGTAAATGGATTTCT
TATAATCATCAACTGTAATGAATTGGTCAAAAGCAGAAAGCTAACACCCATGCAACTCCTGTTCGTATGTATCGGGATGT
CTAGATTTGGTCTACAGACTGTGTTAATGGTACAAGGTTTTTTCTCAGTGTTCTTTCCACGCTTTTATAGCACAAAAATC
TATGGCACACCAATGCTGCTCTTTTGGATGTTTTTCAGCTCTGTCAGTCTCTGGTTTGCCACCTGTCTCTCTTTATTTTA
CTGCCTCAAGGTAACAGGCTTCACCCAGTGCTGTTTTCTTTGGCTGAAAGTCAGGATCTCAAAGTTAATGCCTTGGATGC
TCCTGGGAAGCCTGCTGACCTCTATGAACATTGCAGCTCTGTGTGTCAAGGTGGATTACCCTAAAATTGTGGATATTGAT
GTCCTCGGGAATGCCACAGCTAAGAGGATTAAACTCAACACAAAGCAAATTAATGAAGTTCTTCTCGTCAACTTGGCATT
ACTATTTCCTCTGACTATATTTATAATATGCACTGTTATATTATTCATTTCTCTCTACAAGCACACTCATCGGATGCAAA
ATGGACCTCTTGGTTTCAGAAACACCAGGACTGAAGCCCATATTAATGCATTAAGAACAGTGATAACATTCTTTTGCTTC
TTTATTTCTTACTTTGGTGCCTTCATGGCAAATATGACATTCAATATTCCTTATGGAAGTCATTGCTTCTTTGTGGTGAA
GGATATTATGGCAGCATATCCCTCTGGTCATTCGGTTATAATGATCTGGAGTAATTCTAAGTTCCAGCAACCAATCAGGA
GACTTCTCTGCCTAAGAAGGAGTCAGTGA
>CahiTAS2R3_chr4:102113723-102114673
ATGTTGAGACTCAGCAATTTGGGGTTTCTGGTTCTGACCGCCATTCAGTTCATCCTGGGAATGCTGGGGAATGGTTTCAT
AGGGTGGGTCAATGGCAGCAGCTGGTTCAAGAGCAAGAGGATCTCTTTGCATGACTTCATTATCACTAACCTGGCTGTCT
CCAGGATTGTTTTGCTGTGGATTCTCTTGATCGATGGTGTTTTACTGGTGTTCTCTCCCAAACTACACGATGAAGGGATA
ATCATGCAAATTATTGATGTGTTCTGGACATTTACAAACCATCTGAGCATTTGGCTTACCACCTGTGTCAGTGTCTTCTA
CTGCCTGAAAGTGGCCAGTTTCTCCCATCCTATGTTCCTCTGGCTCAAATGGAGAGTTTCCAGGGTGGTTGTATGGATGC
TGTTGAGTACCCTGCTGTTATCATGTTGCAGTGCCATCTCTCTGATCCGAGAATTTAAGATCTATTCTGTTCTCGGTGGA
ATTGATAGAACCAGGAATATGACTGAGCTCTTTAGAAGGAAGGAAAAAGAATATAAACTGATCCATGTTCTTGGGACTCT
GTGGGACCTCCCTCCCCTAGTCGTATCTCTGATCTCCTACTTTCTGCTTATCCTCTCCCTCGGGAGGCACGTGCGGCAGA
TGCATCAAGACTGTGGCAGCTCCAGAGATCCCAGTACCGAGGCCCACAGGAGGGCCATCAGAGTCATCCTCTCCTTCCTC
TTCCTCTTCCTACTCTACTATCTTTCCTTTTCTGTTTTAACATCCAGTTATTTCCTACCAGCAACTAAGATGATTGCGAA
GATTGGAGAAGTAATTACAATGTTCTATCTTGCTGGCCACTCTTATGTTCTCATTCTGGGAAATAGCAAGCTGAAGCAGA
TGTTTGTGGCGATGCTTCGGTGTGAGCCTGGTTGTCTGAAGCCTGGATCCAAGGGATCTGTTTATCCATAG
>CahiTAS2R4_chr4:102127001-102127891
ATGCTTCGGATAGTCTTTTTTTCTTCTATCGTTGTCTCTGAAATTTTAACTTTTGTAGGACTCATTGTGAATCTCTTCAT
TGTAGTGGTCAGTTACAAGACTTGCATCAAAAGCCACAGGATCTCTTCTTCTGACAGACTCCTGTTCAGTTTGGGCATCA
CCAGATTTTTTATACTGTTACTGAATGTTGTTGTCATCATCTCTCCAAATGTGGAAAGGTCAGTCTCCTTATCCTCTTTC
TTCCTGTCATGTTGGATGTTTTTGGACTCTAGTAGTCTTTGGTTTGTAACCTTGCTCAACGTCTTGTATTGTGTGAAGAT
TGCTAACTACCAACACTCCGTGTTTCTCCTGCTGAAACGAAATCTCTCCACCAGGATGCCCCGGCTGCTGCTGGTCTGTA
TGCTCCTTTCTGTCTTCACCACTCTCCTGTATGTTATGCTCAGACAGTTGGCACCCCGTCTTGAATTTGTGACTGTGAGA
AATGGCACAGTATTTGACATCAATGAGGGACTCCTGTCTTTGGTGACTCCTTTGGTTTTGAGCTCATTTCTCCAGTTCAT
CATTAATGTGACCTCTGCTTCTTTGTTAATCAATTCCTTGAAGAGACATATACGGAAGATGCAGAGAAGTGCCACTGTTC
TTTGGAATCCCCAGACTGAAGCTCATGTGGGTGCAATGAAGCTGATGATCTGTTTCCTCATACTCTACATTCCATATTCA
GTTGCTACCTTGCTCCATTATCTCCCTTCTTCTATAGGGATGGATTTGAGAACCAAGTCTATTTATGTTATTATGTCCAC
CATTTACCCTCCAGGACATTCTCTTCTTATTATTCTCACACATCCTAAACTGAAAACAAAAGCAAAGAATATTCTTTGTT
TCAGTAAATAG
>CahiTAS2R7_chr5:91079349-91080287

ATGTCAAGTGAAGTGCAGGGTATCTTAATGCTCATAGCAGCTGGGGAATTTTCACTGGGGATCTTAGGGAACGCATTCAT
TGGACTGGTAAACTGTGTGGACTGGATCAAGCACAAGAAGATTGCCTCCATTGATTTAATCCTCACAAGCCTGGCCATCT
CCAGAATTTCTCTCTTATGTATAATACTATTGGATTGTAATATATTGGTCCTGTACCCAGATGTCTATACTGGTGGTAAA
CAAATGAGAATCATTGACTACTTCTGGACATTAACCAACCATTTAAGTGTCTGGTTTGCCACCTGCCTCAGCATTTTCTA
TTTCCTCAAGATAGCAAATTTCTTCCATCCATTTTTCCTCTGGATGAAGTGGAGACTTGACAGTGCAATTCCTAGGATCC
TGCTGGGGTGTTTGGTCTTCTCGGTGTTCATTAGCCTTCCTGTCATTAACAATTTGGATGATGATTTCAGGCATTGTGTC
AAGATGAAATTGAAAACAAATATAAGTCGGAGATGCAGAGTACATAAAGCTCAGCATGCTTCCATCAAGATACGTCTCAA
TCTGTTGACACTATTTCCCTTTTCTGTGTCTCTGATTTCATTTCTCCTCCTGATCCTCTCCCTGTTCAGACACACCAGAC
GAATGCAGCTCCGTGCCCCGGGGAGCAGAGATCCCAGCACGGAAGCTCACGTGAGAGCCATGAAGGCTGTCATCTCCTTC
CTCCTCCTTTTCATTGCCTACTACTTGGCCTATCTTGTGGCCACGTCCAGCTACTTTATGCCAGAGACTGAATTAGCTGT
GATCGTTGGTGAGTTGATAGCTTTAATCTGTCCATCAAGCCATTCACTCTTCCTAATTCTAGAGAACAAAAAATTAAGAC
AAGCATCTCTAAGGGTGCTATGGAAGGTAAAATATATCCTACGAAGAAGGAATTGCTAA
>CahiTAS2R10A_chr5:91050078-91050977
ATGCTGAGTATAGTAGAAGGCCTCCTCCTTTTTGTAGCAGTTAGTGAGTCAGTATTGGGGGTTTTAGGGAATGGGTTTAT
TGGACTAGTAAACTGCATTAACTGTGTGAAAAATAAGAAGATCTCTACACTCAGCCTTATTCTCACTGGCTTAGCCTCTT
CCAGATTTTGCCTGATATGGATAATAACTACAGATGCATATGTGAGATTGTTTTCTCCAGATATGTATTTGTCTGGTGAT
CTAAGTCAATATATAGCTTACTTATGGATAATTATGAATCAATCAAGTGTCTGGTTTGCCACCAGCCTCAGCATCTTCTA
CTTCCTGAAGATAGCCAACTTTTCCCACTGCATTTTTCTCTGGCTGAAGGGTCACATCAATAAGATCCTTCTTCTTCTAA
TGGGATGTTTGCCCATTTCATGGTTATTTACTTTTCCAAACATTACAATGCCTTTTATTAATAATATTATGAAGAACAGA
AACACAACCGGGTTGATCACCATGCAGAAAAGTGAATACTTTATAAATCAGATTTTGTTCAGTATTGGAACACTTCTTGT
CTTTATACTGTGCCTGATTACATGTTTCTTATTAATCACTTCCCTTTGGAAGCACAACAGGAGGATGCAATTGAATGGCA
CAGGATTCAGAGACCCCAGTAGAGAAGCACATATCAAAGCAATGAAGATCTTGGTGTCTTTTATCATCCTCTTTATCCTG
TATTTTGTAGGCACTGCCATACAAATATCAATTGATACTATGCCTAAAAACAAACTGCTGTATATTTTTGGTATGACAAC
CACTATCCTCTATCCCTGTGGACACTCATTTATCCTAATTCTTGGAAACAGCAAGCTTAAGCAAGCCTCTCTGAGGGTAC
TGAAGCTATTAAAGTGCTAG
>CahiTAS2R10B_chr5:91018465-91019394
ATGCTGAGTATAGTGGAAGGCCTCCTCATTTATGTAGCAGTTAGTGAATCAGTATTGGGGGTCTTAGGGAATGGATTTAT
TGGAGTTGTAAGCTGCATTGATTGTGTGAAAAGCAAGAAGATCCCTACTGTCAGCCTTATTCTCACTGGCTTAGCTTCTT
CCAGATTTTGCCTGATATGGATAATAATTACAGATGCATATGTGAGGATGTTTTTTCCAGATACATATTTGTCTGGTAAT
CTAAGTCAAAATATAGCTCACTTTTGGATAATTATGAATCAATCAAGTATCTGGTTTGCCACCAGCCTCAACATCTTCTA
TTTCCTGAAGATAGCCAATTATTCCCACTGCATTTTTCTCTGGCTGAAGGGTCACATCAACAGGGTTCTTCTCCTTTTCA
TGGGGTCCTTGCTTATTTCATGGTTATTTGCTTTTCCAAGCATTGCAAAGCCTAGTATTAATGATATTATGAAGAACAGA
AGCTCAACCTGGCTGATCGCCCTGCATAAAAGGGAATACTTGACAAATCATATTCTGCTCAATATTGGAGTCATTCTTGT
CTTTGTGCTATGCCTGATTACATGTTTCTTATTAATCACTTCCCTTTGGAGACACAACAGAAAGATGCAATTGAATGCCA
CAGGATTCAGAGATCCCAGCACTGAAGCACATATCAAAGCAATGAAGACTTTGGTGTCTTTTATCATCCTCTTTATCTTG
TATTTTGTAGGCACTGCCATACAAATATCAGGTAGTACTATGCCTGAAAACAACCTGTTGCTCATTATTGGTATAACAAC
CAGACTCCTCTATCCCTGTGGACACTCATTGATCCTAATTCTAGGAAACAGGAAGCTGAAGCAAGACTTTTTGAGGGTAC
TGAAGCCATTAAAGTGCTGGGGAAAAGAGAAACTTCTTAGAATTCCATGA
>CahiTAS2R11_chr5:90986578-90987545
ATGTTGAATATATTGGAGAAAGTTTTCATGGTTGTGACTGGTGTGGAATTTATAATAGGAATTTTAGGGAATGGATTTAT
TGGACTCACAATTTGCATTGCTTGGATTAGAAATCAGAAGTTGAGCTTGGTTGACTTCATTCTTACTAGTTTGGCCTTTG
CCAGAATCAGTCAATTATGGATAACCGCTGTCATGTTTTTTTCAATGATGTTCTATCAGGCAGGCTTTGGTACTGTGGGA
AGAAAATATATCTTTTTTTGTATCTGGATACTGGCCAGTCACTCAAGCACTTGGCTTGCTACTTGCCTTGCTGTCTTTTA
TTTCCTGAAGATCGCCAGTTTCTCCCATCCTCTTTTCCTTTGGCTAAAATGGAGAATTAACAAGGTTGTTTTTATGTTTC

CACTGGTATCTGTGCCCTTCCTAGTCATAAGTTTTCCTTGGCCATACACTGTTGATATCTTCTGGTGTCATGTCCGAAAG
ATGCATGAGAGAAATATGACTGAATTATGCAATGTGAATGAATATAAAAATTTAAATTTTATGATTATCTACAGTGTGGT
GTCCCTCCCACCCTTCTTCCTTTCCCTGATTTCCTTTCTCCTGTTGCTCCATTCTTTGTGGAAACACAAGAAGAACATTG
CACACACTGCCAGGGATTCCAGAGACCCCCATGTGGAGGCCCATTTCAGAGCCATGAAAACTGTGTTTTTCTTTCTCATG
CTCTTTGTCCTGTACCAATTTGGCCTTTTCATGACATTTGGGGGACATATTTTCCTACAGAACAAGCTGGTTGTGATGTT
TGGTTATCTGTTAGAAATGCTGTATCCTTCAAGTCATTCATATGTTTTAATTTTTGGAAACAGCCAAATGAGGACATTCT
TCTTGGTGATTCTTAGGCACCTGAAGTGTGGCCTGAAAGGAAGAAGGCACTGTTGGCTGCGTAGGTAG
>CahiAST2R12_chr5:90973912-90974823
ATGGAGAGAACACTGAACAATATACTTATGATCATTTCTGCTGGAGAATTCTTACTGGGTATTTTGGGAAATGGATTCAT
TGTTCTGGTTAACTGTATTGATTGGATCAGGAGCAGGAAGTTCTCCCTGATTGACTTTATTCTCACCTGCTTGGCTATTT
CCAGAATATTTGTGCTGTGCATAATGATTTCAAGTACAGGTTTATATGTAATCTCTGAGGAAATACAGTACAACAAGAAT
CTCCTGATAAATTTGGGGTTCCTCTGGACAGGATCCAATTATTTCTCCATAGCCTGCACCACCTGCATCAGTGTCTTCTA
TCTCCTCAGAATAGCTAACTTTTCTAATTTCCTTTTCCTCTGGATGAAACGGAGAATTCACAAGGTGCTTCTCATTATTG
CACTGGGGGCTGTCTTCTCTTTCTGCTTGTGCCTTCTTCAAAAGAATATGGCAGTTGAAATCCTGTTCCAAAACCAGGTA
AACAGCAAAAAAAATGTGACATTGGACTTTCTAATGATAAGATACGATTTGTTCCTTACCATAATGTTCCTCATCCCCTT
TGTAGTGTCCCTGGCCTCCTTTCTCCTTTTAATCCTCTCCTTATGTGGTCATCTCAGGCATATGAAGGGTGTAGACTGTA
GCTCGGAAGCCCATGTGAGAGCCCTGAAGGCTATGATTTCATTCTTGCTCCTCTTCATTCTACACTATTTGAGCAATATT
ATGACAATGTGGGCCAATCACATTCTCGGTAGTTTTGTGGCAAAGATTTTTGTGAACATGCTGTTATTTTTCTGTCCTTC
TGGCCACCCTTTGCTTCTGATTTTGTGGAACAGCAAATTGAAAAAGGCTTCACTCTGTGTCCTAAGGAAGCTGAGGGGTT
ACATGAATCTAAGAAAACTCTTCCAAAAATAA
>CahiTAS2R16_chr4:84884479-84883574
ATGATAACCAGCCAACTCTCTGTCTTCTTCATGCTCATCTATATGCTCGAGTTCTTGACAATAACTGGGCAGAGCAGCCT
GATTGTCATAGTGCTGGGCAGAGAGTGGGTGCAGACTCAAAGGCTGCCGCCTGTGGACATGATTCTCGCCAGCCTGGGCA
TCTGCCGCTTCTGTCAACTGTGGTCATCGATGCTGCACAACTTCGGCTCCCACTTCCACCTTAATTACAATTTTTGGTGT
TTCGGGATCATCTGGCAATTTACCAACATCCTTTCCTTCTGGTTGACCAGCTTGCTTGCTGTCTTCTACTGTGTCAAAGT
CTCCTTCTTCAGCCACCCCATCTTCCTCTGGATGAAGTGGAGAATTGTGAGATGGGTTCCTCGGCTGCTGCTGGGCTCCC
TGCTGGTTTCTTGTGTGTCTACCATCTTTTCAGCGACTAGTTATTACATCATCATTCAATTCATCTCCATGAAGGATTTC
CCTAGAAACAGCACCATGCTTGAGAGACTGGAGGCGTTCCTGTGGGATTTTTCCACACTGCGGAAAGTGGTCGTATTGGT
TATTCCTTTCCTCCTGTTCCTGGCCTCCACAGTCTTGCTCATGGCCTTATTATCCCGACATCTGAAGCAGATGAAAGACC
TTCACACAGGCCGCCCCATCTCCAGCCCGGAAGCTCACTCTGCCGCCCTGAGGTCTCTTGGCATCTTCCTCATCTTGTTC
ACCTTTTATTTTCTGACCGTGCTCGTCTCCATCTTGGATGTCCTATTTAATAAAGAGTCTTGGTTCTGGGCCTGGGAAGC
TATCATCTATGCATTAGTCTCTATTCATTCTACTTTACTAATGCTGAGCAGTGCCAAACTGAAAAGAGTTTTAAAGGCAA
GGTGCTGGAGCCTAGAAGCTGCCTGA
>CahiTAS2R408A_chr5:90916199-90917116
ATGACAACTTTAGTATCGAGCATTCTTTCCATTCTAACGGTGACAGAATTTGTTTTGGGAAATTTTGTGAATGGTTTCAT
AGCACTGGTGAACTGCAATGACTGGGTCAGGAAACAAAAGATCTCCTCAGCTGATGGGATTCTCACTGCTCTGGCAGTCT
GCAGAATTGTTTTGCTCTGGACAATATTAATAAATTGGTATGCAACTATGTATAATCCAGCTCTATATAGTTTAAGAACT
GTTATCCGTGTTGCCTGGACAGTAAGTAACCATTTTAGTAACTGGCTTGCTACTAGCCTCAGTATATTTTACTTGTTCAA
GATAGCTAATTTCTCCAGCTTAATTTTTCTTCACCTGAAGTGGAGAGTTAAAAGTGTAGTTCTCATGATGATGTTGGGGA
CTTCAGTGATTTTGATTTTTCAGGTTGCTGTGTTAGGTATAGATGAGACTATTCAGACAAGTGAATATGAAAGAAACATT
ACTGAGAAGACCAAATTAAGGGACTTTTTACACCTTTCAAATATGACCCTGCTCACACTAACAAACTTCATACCTTTCAC
TATGTCCCTGACATCTTTTCTGCTGCTAATCTTTTTCCTGTGGAAACATCTCAGGAAGATGCAGCTCAACGGCAAAAGAT
CCCAAGATGCCAGCACCAAGGTCCACACAAAAGCCATGCAAACTGTCATCTCCTTTCTTTTCCTGTTTGCCACTTATATC
CTGACTGTAATTTTAACAATTTGGAATTCTAATGAGCTGCGGAAGGAACCGGTCCAAATGCTTTTCCAGGCCCTTGCAAT

CACCTATCCTTCAATGCACTCATTTATCCTGATTTGGACAAACAGGAACTTAACACAGACCTTTCTGTCATTTCTATGGC
AGCCAAGATGCTGGCTAAAAGTAAGAGGAACTAGGTAG
>CahiTAS2R38_chr4:102284523-102283516
ATGGTGACTCTGACTCACATCGCATCTGTGCCCTCTGAAGTCAGGAATGCATTTCTGTTCTTTTCAGTCCTGGAGTTTGC
AGTAGGGATCCTGGTCAACGCCTTCATTTTCTTGGTGAATTTCCGGGACCTGGTGAGGAGGCAGCCACTGAGCCACTGTG
ATCTTGTCCTGTTGAGTCTCAGCCTCACCCGGCTTGTCCTGCACGGGCTGCTCTTTCTGAAGGCCATCCAGCTTACTCAT
TTCCAGCGAATAAGAGACCCACTGAGCTTCAGCTACCAGACCATCATCGTGCTCTGGATGATCGTCCACCAAGCCGGCCT
CTGGCTCACCACGTGCCTTAGTCTCCTTTACTGCTCCAAGATTGTCCGTTTCTCTCACGCCTTCCTGCTCCGTGCAGCAA
GCTGGATCTCCAGAAAGATCCCCCAGATGCTTCTGGGTGCTGTGGTTCTCTCCTGTGTCTGCACTCTTCTCTGCTTATGG
AACTTTTTTAGTGGATCTCGTTTCTCAGCTGTAACTAGGCTACTCACGAATAACAGTACTGAACTCAATTTGAACATTGC
AAAACTCAGTTTCTTTCATTCCTTCCTCTTCTGCAGCCTGGCGTCCATCCCTTCTTTCTTGCTTTTCCTGGTTTCCTCTG
GGATGCTAGTGTTCTCCCTGGGGAGGCACATGAGGATGATGAGGGCTGAAACCAGAGGCTCTCGGGACCCCAGCCTGGAG
GCTCACACCCGGGCACTCAGGTCTCTCGTCTCTTTCTTCTGCCTGTGTGTGCTGTCACTCTCCGCTGCCTTAGTCTCGGT
GCCGTTGCTGACGCTGTGGCACAGCAAGGTTGGGGTGATGGTCTGCATAGGGATAATGGCAGCCTGTCCCTCGGGACATG
CAGTCATCCTGATCTCAGGGAATGCCAAGCTGAGGAGGGCTATGGACACCATTCTGCTTTGGGCAAAGAGCAGTTTCAGG
GTAAGGATGGACCACAAGGCAGATCCGAGGACACCAGATCTGTGTTGA
>CahiTAS2R39_chr4:103009425-103010471
ATGACAAGTGGGAGCTATCACAGACCCGCACATCAAGCGCTAAGGAGCCGTTTTCCTCCAGACATCGAAGAAGAGCAACC
ATTCAGGATGATCCAAACCTGCAGTTCCTCAGAAAAGGATCTGTCACCATCTCTTGTCACTTTGATGTTAATAATTATCG
GCACGGAATGCATCCTTGGTATTCTCGCAAATGGGTTCATTGCAGCGATAAACACAGCTGAATGGATTCACAATAAGGTA
CTCTCCACCAGTGGCAAGATCTTGCTTTTCCTGGGTGTATCCAGAATAGTTCTACAAAGCTTCATGATGCTAGAACTTAC
CTTAAGCTCAACATCCCCACAGTTTTATAATGACGACATCACGTATCACACATTCAGAGGATGTTTCATGTTCTTAAATC
ATTGCAGCCTCTGGTTTGCTGCCTGGCTCAGTGTCTTCTACTTCGTGAAGGTGGCGGATTTCTCCTACCCCCTTTTCCTC
AAGCTGAAGTGGAGAATTTCCGGACTGATGCCCTGGCTCCTGCAGCTTTCAGTGTTTGTTTCCTTGGGCCAGAGTGTGCT
CTTCTTCCAAAACATCTATACTATGAATTGTAACAATCTTTTTTCTCTCCCCTCCTTCAACTCCACTAAGAAAAAGTCCT
TCTCGGAGGCCACTGTGATCAACCTGGTTCTTTTCCTTAACCTGGGGATCTTCATCCCTCTGATCATGTTTATCCTGGCA
GCCACCCTGCTGATCATCTCTCTCAAAAGACACATCTTCCACATGAAAAGCAATGCCACTGGCTCCAGAGACCCCAGCAC
GGAGGCTCACCTGGGGGCCATCAGAGCTATCAGCTACTTTCTCATTCTCTATATTTTCCAAGTACTTGCTCTCTTTCTCT
ACATGTCCAACTTCTTTGACATCAATAGTCCCTTGAATATTTTGTGCAAAATCATCATGGCTACCTACCCTGTGGCCCAT
TCCATTCTACTGATTCAGGACAACCCTGGGCTGAAAAGAGCCTGGAAGAGGCTTCAGGCTCAAGTCCACCTTTATTTTAA
AAAGTAG
>CahiTAS2R40_chr4:103068808-103069761
ATGGTGACAGTGAACACGGATGCAATGGATAAAGACACGACCAGGTTCAAGATCGTCTTCACCTTGGTGGTCTCTGCAAT
AGCGTGCATCATTGGCATCGCGGGGAACGACTTCATCACCATCAACCATGGAGCCGAGTGGGTCAGAGGCGAAAGACTCC
CCATTGGTGACTGCATTCTGCTCATGCTGAGCTTTTCCAGGCTCTTGCTACAGATCTGGATGATGCTGGAAAACACGTAC
TGTCTGCTATTCTGGGTCATCTACAATGAGAAAAGAGTATACATACTTTTCAAAACCATCGTCATGTTTCTGAACTACTC
CAACCTCTGGCTTGCTGCCTGGCTCAATATCTTCTATTGTCTCAGAATCGCAAGCTTTACTCACCCGTGGTTCTCCGTGA
TGAAGAGGAAGGTCATGGGACTGATGCCTGGGCTTGTGAGGCTGTCCTTGTTCTTCTCCTTTTGCTCCAGCTTCCCCTTC
TCTAGAGGCATCTTCAATGTGTACGTGAACAATTCCGTCCCCGTCCCCTCTTCCAACTCCACTGAGAAGGTGTACTTCTC
CGAGACCAACGGCAACTTGGTTACCACCCTTTACCTGGGGATCTTCATCCCTCTGATCATGTTTATGCTGGCGGCCACCC
TGCTGATCATCTCTCTCAAAAGACACACCTTCCACATGAAAAGCAACGCCACTGGCTCCAGGGACCCCAGCATGGAGGCT
CACCTGGGGGCCATCAAAGCCATCAGCTATTTTCTCATCTTCTACATTCTCAACGCAGTTGCTCTGTTTCTTTCCATATC
CAACATCTTTGCCGCCAACAGCTCCTGGAATATTTTGTACAAAATCATCATGGCTGCCTACCCTGCTGGCCACTCAGTGC
TACTGATCTTAGGCAACCGTGGGCTGAAAAGGGCATGGAAGCGGTTTCAGCACCAAGTTCATCTCTACCTGTAA

>CahiTAS2R41_chr4:103311989-103312924
ATGCACCCAGAATTCACAGTCCTCTTCATGCTGCTCTTTGTCCTGCTGTGTATCCTGGGCCTCCTGGCCAATGGCTTCAT
TGTGCTGGTGCTGAGCAGAGAATGGGTGCAACGTGGGAGGCTGCTCCCCTGTGATCTGATCCTCTTTAGCTTGGGACTCT
CCCGCTTCTGCCTGCAGTGGGTTGGAATGGGGAATAACTTCTACTATTTCCTGCATCTGGTCGACTACTGCAGCGGTCCC
GCCCGGCAGTTCTTCGGTCTACCCTGGGTCTTCCTCAGCTCCGTCACTTCCTGGTTTGGCTCCTGGCTCAGCGTCCTCTT
CTGCATGAAGATTGCTAACTTTACCCACCCTGCCTTCCTCTGGCTAAAGTGGAGGTTCCCCAGGTGGGTGCCCTGGCTTT
TGCTGGGCTCTCTCCTCACCTCCTTCACTGTCACCCTGCTTTTTTTTTCAGGGAACCACGCTTTGTATAAAGGGTCCTTC
ACTAGAAAACCTTTCAGGAACATGACCTATCATCAATGGAGCAGGATTCTGGAAATGTACTATTTCCTGCCCCTGAAAAT
GATCACTCTTTCAGTTCCTGGCTCTGTTTTTCTGGTCTCGATTGCTCTGTTGATTCACTCTCTGAGGAGACACGCATGGA
GGATGCAGCACAGTGGTCACAGCCTGCAGGATCCCAGTGGCCAGGCTCACACCAGAGCTCTGAAGTCACTAGTCTGCTTC
CTTGTTCTTTATATTCTGTCTTTCGTGTCCCTGATCATTGATGCTGCAGGGTTCTGCTCCTCAGAGAGTGACTGGTACTG
GCCATGGCAAATTTTAGTCTACTCGTGCACTTCCATCCATCCCTTTATCCTCATCCTTGGCAACCTCAGGCTTCGAGGGG
CATTTGGGCAGCTGATTTTGTTGGCCAGGGGCTTCTGGATGGCCGAGGTGGTGTGA
>CahiTAS2R42_chr5:90857263-90858192
ATGTTCCCTGGGTTGAGTACAGTATTTCTGATACTGTCAGGAGTGGAATTCTTAATCGGAATTCTAGGCAATGTGTTCAT
TGGACTGGTACTCTGCTCTGAATGTGTTAAGAACCAAAAGACATCTTTATTTGACTTCATCCTCACTGGCTTGGCTGTCT
CCAGAATCAGTCAACTGTTGGTGTTTTTTGTGGAATCACTTATAATGGGACTAGAACCACAGGTATTTGCCATTTTTAAA
CTAGCAAAGCCCATTGCTTTACTTTGGAGAATATCTAATCATTTGACTACCTGGCTTGTCACCTGCCTAAGTATTTTCTA
TCTCCTTAAGATAGCTCATTTCTCCCACTCTCTTTTTTTCTGGCTGAAGTGGAGAATGAACAGCGTCATCCTTGTGATAC
TTGCATTTTCTTTGGTCTTTCTGATTTTGGACATTCTTTTGCTAGAAACATTTAATGATCTCTTCTGGAATTTAATAAAT
GAAGGCAATTTGACTTTAGTTGAAAGTAAAACTCATTATATTAAAAGCGAGAGTCTTCTTAGTTTCTCCTATTTCATTCC
TATTGTTCTGTCCCTGCTCTCATTGTTTTTTTTATTTCTGTCCTTGGTGAAACACACCAGAAATTTGCATCTCAATTTTA
TGGGTTCCAGGGACTTCAGCACAAAGGCCCATAAAAGAGCCATGAAAATGGTGACGTCATTCCTGCTCCTTATCATGGTT
CATTTTCTTTTTACACAATTGGCAAATTGGATGTTTCATAGGTTTTTGGACAATAAGTTCACAAAGTTCATCATGTTAGC
ACTATATGTCTTTCCTTCAGGCCACTCGTTCATGTTGATTCTGGGAAACAACCAGTTAAGACAGATAGCCTTGAAGGTAC
TGAAGCATCTTAAAAGCTCCTTGAAAAGACAAAATCCATTGGCTTTATAG
>CahiTAS2R408D_chr5:90907931-90908845
ATGATAACTCTACTATCAACCATTTTTTCCATCCTAGTTATAATACAATTTGTTCTGGGAAATTTTGCCAATGGCTTTTT
AGATCTGGTGAGCTGCGTTGACTGGGTTAAGAGACAAAAGATCTCCTCAACTGATGTGATTGTCACTGCTATGGCAGTCT
CCAGAATTGTTTTGCTCTGTGTAATGTTAATACATTGGTATTATATTTTGCTTCATCCAGCTTTATATGGTTTTAAAATA
AGAACTATTGTTCATGTTGCCTGGACAATAAGCAATCATTATAGCACCTGGCTTGCTACTAGCCTCAGTATATTTTATTT
GTTGAAGATAGCCAATTTCTCCAGCCTAACTTTTCTTCACCTGAAGTTGAGAGTTAGAAGTGTAGTCCTCATGATGCTTC
TGGGAACTTCATTCATTTTGGTTTTACAAGTTGTAGTTATAAGTGTAAGTGGGACTATGCAGAGAAGTGAATTTGAAAGA
AACCACACAGAAGACCAAACTGAGGGATATTTTATGGCTTTCACATGTGACCCCACTTATTCTAGGAAACCTCAAACCCT
TTACTATGTCCTTAATATCTTTTATGCTACCAATCTCTTCTCTGTGAAACATCTCAGGAAGATGCAGCTCAATGGCAAAG
GATTCCAAGTTCCCAGGACCAAGATCCATACAAAAGCCATGCAAACTGTCATCTCCTTTCTCTTGCTATTTGCCTTTTAC
TTTCTGGTTCTAATCATATCAATCTGGAGTCCTAAAAAGTTGCATGAGGAACCGTTTCTCTTGCTTTTCCCAACAGCTGA
AGTCATCTATCCTTCAGTCCACTCATTTTTCCTGATTTGGGGAAACAGAAAGTTAACACAGGCCTTTCTGTTGTTTCTGA
GGCAGCTGGGGTGCTGGCTGAAAGACAGGAAATAG
>CahiTAS2R60_chr4:103283243-103284196
ATGAGGGGAGAGGACGTGGTTCCAGGACCTCAGGTGGTTGATAAGACAGCCCTCATCTGCGTTGTTATTTTATTCCTTTT
GTTCCTGGTGGCATTGGTAGGTAATGGCTTAATCATCGCGGCACTGGGCAGCGAGTGGCTGCTGCGGAGAACGTTGTCAC
CCTGCGATAAGTTATTGGTCAGCCTGGGGACCTCTCGCTTCTGCCTGCAATGGGTGGTAATTAGTAAGAACATTTACATT
TTCCTGAATCCAACGACCTTCCTTTATAGCCCTGTGTTCCAGCTCCTGGCCGTTCAGTGGGACTTCTTGAACTCGGCAAC

ACTGTGGTTCTCCACCTGGCTCAGTGTCTTCTACTGTGTGAAAATCGCAACCTTCACCCACCCCGTCTTCCTCTGGCTAA
AGCGGAATGTATCTGGGTTGGTTCCTTGGATGCTACTCAGCTCTCTGGGGTTCTCTACCTTTACCACCGTTCTATTTTTC
ATAGGCAACCAGAGAATGTATCAGAACTATTTAAGCAGGGGTCTGCAATCTTGGAATGTCACTAGGAATGCTGTGAGAAT
GTATGAGAGGTTCTACCTCTTCCCTTTGAAAATTGTTACCTGGACCGTCCCTACTGTTGTCTTTATTGCGGGCACGGTTT
TGCTCATTACATCTCTGGGAAGACACACCAAGAAGGTCTTCTTCTCCATCTCAGGCTTTCACAGTTCTAGTGCCCAGGCA
CACATCAAGGCCCTCTTGGCTTTTCTCTCCTTTGCTATCTTCTTCACTTCCTCTTTTCTGTCACTGGTTCTCACTGCCTC
AGGTATGTTTCCCTTTCGGGAGTTCGGGTTCTGGATATGGCAGATTGTGATTTATCTGGGTACAGCAATCCACCCCATTA
TTCTTCTCTTAAGTAACCGCAGGCTGAGAGCTCTGCTAGGGAGGGGCTGCTCCTCAGCACATGGGGCATCTTGA
>CahiTAS2R62_chr4:103273254-103274177
ATGTCCCCTTCGCCCACATTGATCTTCACGGTCATCTTTTTCCTGGAGTCATTGGTTGCCATGCTGCAGAATGGCTTCAT
AGTTACCATGATGAGCGGGGAGTGGGCGTGTAGCCGGACTTTGCCCGCCAGTGACATGATTGTGGCCTGCCTGGCTGCCT
CCCGGTTCTGTCTGCATGGGATGGCCCTCCTGAACAACCTCATGGCCTCCTCTGGCTTTTGTTCCAAAATCTACTATTTC
GGCATCCCCTGGGATTTCATCACCTCCCTCAGTTTCTGGCTGACTGCCTGGCTTGCTGTCTTCTACTGCAGGAAGAGCTC
CCTCTTCTCTCACCTCGTCTTCTGGATAAAGTGGAGGATTTCTCGATCGGTTCTCCAGCTGCTGCTGGGCTCCTTGATCT
TATCTGGTCTGACTGTCGTCTCCTCAGCTGCTGGGCATACAATTCTTGCCCAGATGACGGCTGCCCAGAGTTCCCATGGA
AACACCCTGGCTGGTAGCATACACGCCGTCTATTTGCACTGTTTTCTACCTCATGTAATTCTCATGAGGTTGGTTCCATT
CCTCCTGTTCCTGGTGTCCACCTTCTCGCTCATGGTCTCGCTGCGCCGGCACCTCGGGCAGATACAGGACCGCAGACCCA
GCCCACGTGATCCCAGTACCTGGGCTCACACCATGGCCCTGAAGTCACTTGCCTTCTTCCTCATCTTCTACACCTTGCAC
TTCCTGTCCCTGGTTATCATTGTGTACATCCCAGCCTTCTGGAAACACTGGCACTGGGCCTGTGAGGTGGTGACCTATGC
AGGCATCTGTCTGCCCTCCAGCATCTTGATGCACAGCAGCCCCAAGCTGAGAAAGGCCCTGAAGAAGAAGCTTTGGCGAG
CTCTGGACAAGGACCAGTTTGTCTCCACTTATCAGTATCAATAG
>CahiTAS2R67A_chr5:90876605-90877497
ATGCCATCTGGAATTGAAAATACTTTTCTAGTAGTAACAATAGGAGGATTTGTGATTGGAATGTTGGGGAATGGGTTCCT
TGTACTAGTTAACTGCATTGACCTGGTGAAGAGACAGAAGCTCTCATCAGCTGACTGCATCCTCACAGGCCTGGCTATCT
CCAGAATCAGTCAACTTTGGGCAATACTATGTGACTCATTTTTATTGGTACTATGGCCACACCTATATGCCATTGATAAA
CTAACAAAGATTGTTAATAGTTTTTGGATACTGTCCAATCACCTAGCTACCTGGTTTGCCACCTGTCTAAGTGTTTTCTA
CTTCTTTAAAGTAGCCAACTTCTCCCACCCCTGCTTCACTTGGCTGCGGTGGCGAATTCGTAGTGTGGTACTGGTGCTTC
TCTTGGGGTCTTTGCCCTTACTGTTTTTGAATTCTGAATCAATATATATGTTTAGTCATATCTCAACTAACAGCTACAAA
ATATATGCAAGAAACTCAACACGGTCCCCAAATGTAAGTGAAACTCATGATCTTCACCAGTTGATTGTTTTTAACTTCAT
CAGCTTAATCCCCTTTCTTCTGTCCCTGACCTCGCTGCTCCTCTTAGTCCTCTCCTTGATGAGGCACATCAGGAATTTGC
AGCTCAACCCCAGCTCAAAGGATCTCAGCACAGAGGCCCATAAAAGAGCCATGAAAATGGTGATGTCTTTCCTCTTCCTC
TTCGTCATTCATGTTTCTTCCGTCCTATTAACAGGTTGGGTTTTCCTTAAACTGCAGGGACGTCTGGCCAAATTGGTGGT
TGTGTTAACTGCAACTGTTTTTCCTTCAAGCCACTCATTTATCCTAATTTTGGGAAATAGCAAGCTGAGATAA
>CahiTAS2R67B_chr5:90869316-90870254
ATGCCATCTGGAATTGAAAATACTTTTCTAGTAGTAACAATAGGAGGATTTGTGATTGGAATGTTGGGGAATGGGTTCAT
TGTACTAGTTAACTGCATTGACCTGGTGAAGAGACAGAAGCTCTCATCAGCTGACTGCATCCTCACAGGCCTGGCTATCT
CCAGAATCAGTCAACTTTGGGCAATACTATGTGACTCATTTTTATTGGTACTATGGCCACACCTATATGCCATTGATAAA
CTAACAAAGATTGTTAATAGTTTTTGGATACTGTCCAATCACCTAGCTACCTGGTTTGCCACCTGTCTAAGTGTTTTCTA
CTTCTTTAAAGTAGCCAACTTCTCCCACCCCTGCTTCACTTGGCTGCGGTGGCGAATTCGTAGTGTGGTACTGGTGCTTC
TCTTGGGGTCTTTGCCCTTACTGTTTTTGAATTCTGAATCAATATATATGTTTAGTCATATCTCAACTAACAGCTACAAA
ATATATGCAAGAAACTCAACGCGGTCCCCAAATGTAAGTGAAACTCATGATCTTCACCAGTTGATTGTTTTTAACTTCAT
CAGCTTAATCCCCTTTCTTCTGTCCCTGACCTCGCTGCTCCTCTTAGTCCTCTCCTTGATGAGGCACATCAGGAATTTGC
AGCTCAACCCCAGCTCAAAGGATCTCAGCACAGAGGCCCATAAAAGAGCCATGAAAATGGTGATGTCTTTCCTCTTCCTC
TTCGTCATTCATGTTTCTTCCGTCCTATTAACAGGTTGGGTTTTCCTTAAACTGCAGGGACGTCTGGCCAAATTGGTGGT

TGTGTTAACTTCGACTGTTTTTCCTTCAAGCCACTCGTTTATCCTAATTTTGGGAACTAGCAAGCTGAGACAAAATGCCA
TAGGACTACTGTGGTATCTTAACTGCCGCCTGAAAAGAGTGAAATCTTTAGCTTCATAG
>CahiTAS2R372A_chr5:91042000-91042926
ATGTCAAATGTCATCACATATGTTTTTTTGATCATTGAAATCTCAGAATTCATAACAGGAATTTGCGGAAATGGATTCAT
TGCACTAGTACTTTGTGCTGACTCTCTCAAAAGCAAGAATATCTCCTTGCTTGACTTCATCTTCACATGCTTGGCCATCT
CCAGAATTGGTATGATATTCATACTTCTCCTGGATAGCATTAAAATTTTGTTCCATCCAGAAATATTAGATCGTCACCAG
GTAATAGAAGTAACTTTTGATTTCCTCTGGAATCTGAGCAACTCCTTAGGTACCTGGTGTGCTGCCTGCCTCAGCGTCTT
CTACTTCCTCAAGCTATCTAGTTTTTCCCACCCCTTCTTTCTCTGGCTAAAATGGAGAAGAAATAGAGTTGTTTTCACCA
TTATGTTGGGATTCTGCCTCTCTTTGTTTTTTAATCTTCTGAACATAAAATTCAATGCTCTCAGGGTCTGTGACCATTTA
GAAATAGAAAACAAGTTGACTTGGAAAAAATGCATGCGTAAAACACAGTCCTATAGCAGTCAAATTCTCCTCCAGCTGGG
ATCTCTCATCCCCTTGGCTCTGTCACTCGTTTTATTTTTCCTGTTAATATTTTCCTTATGGAGACATAGCAGGCAGATGA
CACGCTATGCCAAAGGATCCAAAGACCTCAACACAGGAGTTCTTGTGAGAACAAGAAATACGTTGGCCTCTTTCATCCTT
CTCCTAGTTGTGCACTATTTGGCTGCTTTCATGTTAACTCGGTCCTATTTCACACTAGAAAATGACATGACTTTTATTGC
TATTCACACTGTAGCATTTCTCTATCCTTCAATTCACCCTTTTATCTTGATTCTGAGGAGCCGGAAACTGAGACAGATTT
CTGTGAATCTGCTAAGGCAAATTGAATCCTGTATCAAAGGACTGTAA
>CahiTAS2R5P_chr4:102140445-102141319
ATGCTGACTTCTATCCCAGGACTGCTGATGCTGGTGGCAGTGGCTGAATCTCTCATTGGCCTCACTGGAAATGGAGTTCT
TGTGGTCTGGAGTTTTGGAGAATGTCTTCGAACGTCCAGGGAGTCCTCGTATAACCTCATTGTCCTGGGCCTGGCGGTCT
GTCGGTTGCTTCTACAATGGTTGATTATGGTGGACTGAAGTCTGTTCCTGCTTTTCCAGAGCAGCCATTGGCTTCGCTGT
CTCAGTGTCTTCAGGGTTCTGGTAAGCCAGGCCAGCCTGTGGTTTGTGAGTTTTCTCAGTATCTTCTATTGTAGGAAGAT
CATGACCGTTGAACACCCTGTCTCCTTGTGGCTGAAGCAGAGGGCCTGTTACCTGAGTTTCTGGTGCTTTCTGGTGTACT
TCATGATCCATTTGTTACTTATAGTCAGGGGTAGCTTAGACTTCTCCAGTCCTTCCCAAGGAAACAGCATCTTATTCCCC
ATTTCAAACTGGCACTATATATGTATATTACAGCTCAATACAGTATGATGCCTTTCATGACGTTTCCTCTTTCCTCTGGG
CTGCTGATTGTCTCTTTATATAGACACGACAGGAAGATGAAGGTCCATACAGCTGGCAGAAGAGATGCTCAGGCCAAGGC
TCGCATCACTGTCCTCAAGTCCTTGGGCTGTTTCCTTGTACTTTACATGGTCTATACCCTGGCCAGCCCCTTCTCCATCA
GCTCCAAGACTTTTCCTGCGGATCTCATCACTCTCTTCATCTCTGAGACACTCATAGCTGCCTGCCCTTCTCTTCATTCT
GTCATTCTGATCATGGGGAATCCCAGGATGAAGCAGACATGTCAGAGAATCCTGCGGAAGACTGTATGTGCTTGA
>CahiTAS2R8P_chr5:91074257-91075185
ACATTCAGTATAGAAGACCACATCTTTCTGACCATAACGACTGCAGAATTCATCATAGGAATGTTTGTGAATGGATACAT
GGGACTAGTAATATATATTGATTGGATTAAGAAGAAAAAGATCTCCACAACTGACTACATCCTCTCCAATTTAGCTCACT
CCAGAATTTGTTTGCTTTGTGTAATGACACTCAACGGCACCATACTGGCACTCTACCCAGGTGTTTATGAAAATGAGAAA
ATAAAGGTAGTTCTTAATATCTTCTGGACATTCACCAACTACTTACATATGTGGTTTGCCACCTGCCTCAATGTCTTCTG
TCTCTTCAAGATAGCCAATTTCTTCCACCGACTGTTTCTCTGGCTGAAGTGGAGAATCGAGAGGGTGTTTCACTGGAGTC
TGCTGGGGTCCCTGGCCATTTCCATGTTGATCAGCCTTATACAAGCAACGTTAACAAATTCTGATTATGAATTTCTTAAA
ATTGGAAACATAAAAGAAACATCACCAAATTGTTCCATGTGAGTAAAATTCAATACTTCAACCCATTGACACTGTTTAAC
TTGTTAGCAATTATTCCATTTACTGTGTCATTGATCTCATTTTTCTTTTTAATTACATCCCTGTGGAGACACAGCAAACA
AGTGAAATCCAGTGTTACAGGTGCCACAAACTCCAGCACAGAGGCCCACGTGGATACCATGAAAACAGTGACCTCATTTC
TTTTCTTCCTTTTTGTATACTACCTGGCCTGTCTTTTGGCAACATTTAGCTACTTTATGAAAGAAAGCAAGTTAGCTATG
ATGTCTAGAGAGATTATAGCAATTCTTTATCCCTTAGGTCACTCACTGTTTTTAGTTGTTGGAAATAACAAGCTGAGGCT
GGCAGCTGTCGGGATGTTGAGATGTGGGAAAACAGTCTGCATGATGTAA
>CahiTAS2R9P_chr5:91072394-91073325
ATACCAGGTACAATGGAGGCAATATATATGTTCTTGATTACTGGTGAGTGGATGATAGGAATTTGGGGAAATGGATTCAT
TGTATCGGTAAACTGCAGTGGCTGGCTCAAAAAGAGAGCTGTCTCCTTGACTGAGGTCATCCTGGTCAGCCTGGCCACCT
CCAGAATCTGTTTTTTGTATGTGATATATATGGATGGTTTTATTATGGTACTCTTTCCAGATACATACAGGCATGGTGAG

ATGATGAACATTATGGATATTTTCTGGACAACTTGCAATCATTCAACTGTCTGGTTTACTTCGTGCCTCAGCATCTTCTA
TTTACTCAAGATAGCCAGTATATCCCACCCAGTTTTCCTCTGGCTGAAGCTGAAGATGAACAGGGTATCCTTGGGATTCT
TCCGATGTCCTTTCTCATCTCCTCAATTGTCAGTGTTTTACTGAATAATGATTCATTTTGTGACGTCAGAATCAATAATG
AAGCAAACATTATTTAGGAATTCAAAGTAAGTAAAATCCCAACTGCTTTCAAATTGATTATCCTGAACCTGGGGGCTATG
GTTCCCTTTATTCTTTGCCTGGTCTCAATTGTCCTTTTATTTTTCTCACTTTAACACACCAAGCAAATGAAACTTCATGC
CACTGGGTCCAGAGACCCTAGCATAGAGGCCCACATGAGGGTCATAAAGACAATAGTCATCTTTCTGGCTCTTTTCATTA
TGTACTATGCAGTTTTTCTCATTGTAACATCTAGCTTTCTGATTCCTCATGGAAAATTGGAGTTGATGTTTGGTGGCCTA
AGAGCTGCCATTTTCCCATTGAGCCATCCATTCATCCTGCTAATGGGAAACAGAAAGCTGAGGGAGGCTTTTCTGAAGGT
GCTGGGGATTGTGAAGGGTTTTCACAAAAGAAGGAAATATTTTATTCCCCAG
>CahiTAS2R10CP_chr5:91061360-91062105
ATGCTGAGAAGGCCTCCTCATTTTTGTAGCAGTTAGTGAGTCAGTACTGAGGGTTTTAGGCGATGGATTTATTGGACTTG
CATACTTCATTGAATGTGTGAAGAACAAGAAGTTTTCTATCAGCTTTATTCTCATGGGCTTAGCTACTTCAAGAATTTGC
CTGATAGGGTTAACAACTACAGATGGATTTGTGAAGGTTTTTTTCTCCAGAAATGTATTCCTGTGGTTACCTAATTCACT
GTATTACTTACTCATGGATAATTCTGAGTCCATCAGGTGTCTTTTTTTGCCACTAGCCTCAGCATCTTCTATTTCCTGAA
GATAGCCAATTTTTCCCACCACATTTTTCTCTGGTTGAGGAGTGATATCAAAAGGGTTCTTCTCCTTCTGATGGGATACT
TGCTTATTTCACGGTTAGTTACTTTTCCACTAACTATGAAGATAATTAGTGATACTAGAGCAAAGAATAGAAGCGTAATC
TTTTCAGTTGAAGTGCATAAAGGGGAATTCTTTAGAAACCAGATTTTGCTCAATCTTGGAACCCTTCCCATCTTCATACT
ATGCCTGATTACATGTATCTTATTGCTCATTTCCCTTTGGAGGCACAACCAGAGGATGCTATTGAATACCACAGGATTCA
GAGACCCCAGCACAGAAGCACATATCAAAGCAATGAAAGTTGTGATATCTTTTATCATCCTTTTATCTTGAATTTTATAA
GCATTATCATAGAAATATCATGCACC
>CahiTAS2R10DP_chr5:91037001-91037923
ATGCTAAGTATAATGGAAAGTCTCCTCATTTTTGTAGCAATCACTGAGTCAATATTGGAACTTTTAGGTAATGGATTTAT
TGGATTGGTAAGCTGCATTGACTGTAAGAAAAACAAGATCTCTACTATCAGCTTTATTCTTGCTGGCTTAGCAACTTCCA
GATTTTGCCTGATATGGACAATAGTTACTGATGGATTTTTAAAGTTATACTCTTCAATGTACATTCCTCTGGGAACCTAG
TTGAATATAATGGTTACTTATGGATAGTTATGAATCAATCAAGTATCTGGTTTGCCACTTGCCTCAGCATCTTCTATTTC
CTGAAGATATCCAGTTTTTCTCACCGCATCTTTCTCTGGTTGAAGGGTAGACTCAACATGGTTCTTTTCCTTCTTTTGGG
ATGCTTGCTTATTTCATGATTAGTTACTTTTCCACATTTTGTGAAGATTGTTAATGATAATAAAAGGAAAATAGAAACAC
AGTCTGATCAATGGATATGCATAAACGTGAACTCTTTGGAAAACAAATTGGGCTCCATCTTGGTGTCATTCTCCTTTTTA
TACTATGCCTGATTACATGTGTCTTGTTGCTCACTTCTTTTTGGAGACACAGCAGGAGGATGCAATTGAATGCCACAGGA
TTCAGAGACCCCAGTACAGAGGCACATATCAAAGCAGTGAAAGTCTTGGTGTCTTTTATCATCCTCTTTATCTTGAATTT
TGTAGGTACTGCCATACAAATATCAAGTGTGACAGTGCCTGGAAACAGACTGCTTTTTATTTTTGGTATGACAACCACAG
TCCTCTATCTCTGGGGTCACTTGCTTATCCTAATTCTAGGAAATAGGAAGCTCAAGCAAGCCTCTTTGAGGGTACTGAAG
CAATTAAAGTGTGGGGAAAAAGAAACTTCTCAGAACTCCTTGA
>CahiTAS2R13P_chr5:90958522-90959513
ATGGCAGATTCCTTGGAAAACATCTTTATCATTTTAATAAATTCAGCATTCATAATTGGTATTCTGGGGAATGGATTCAT
AACACTGGTGAACGGCATTGACTGGATCAAGATGCAAAAGGTTTGCTTGGCTGATCAAATCCTCACTGCTTTGGCAATTT
CCAGGATTCGTCTGATTTTTGGATTGACTGGATCAAGATGCAAAAGGTCTCCTTGGCTGATTGAATCCTCACTGCTTTGG
CAATTTCCAGGATTCGTCTGATTTTGGTAATGATGGTGAGTTGGTTTACAAAGGAGTCTTATCCATTTTCTTCTTTAGAC
ATAAAGGGAAATAAAGTCATACTTTTTAGTATTGCTGGGCTCTTGGCCAATTATTTTAGTGTCTGGCTTGCCACAGGCCT
CAGCCTCTTTTATTTCCTCAAGATAGCCAATTTTTCAAATGCTGTTTTTCTTCACCTAAAGTTTAGAATTGGAATGGTAT
TTATGGTAATGTTTCTGGGGACATTAGTATTGCTGCCTCTAAGTCTTACTTTGGTGAGCATCTATATTAATATCAAGATA
CATCCATATGAAAGAAATATGACTGTAAGTTCTAAAAGAAGTGACACTGAAACCTTTTCCAAATTGATCATATTCACCAT
GGGATCTTTCTCACCCTTTATTATATCCCTGAGTTGTTTTCTCCTGTTAATGTTCTCCCTACTGAATCATGTCTAGAAGA
TGAGGAGCCAGGGTTCAAGAGATCCCAGCAGCAAAGCCCACGTCAGAGCCATGATCATGGCGATGCCTTTCCTCATACTA

CTTGCCATTCACTTCCTATCTCATCTCATGACAACTTTTCATCACAATGTGATGCAGAGTGAACTGGCCTTTATGCTTGC
TGAAGCTCTTGGAACTATTTATCCTTCAGTCCACTCATTTGTCTTGATTCTGGGAAATGACAAGCTAAGAAAAGCTTCAC
TTTTGGTGCTGTGGCAGTTGAGGTGTGGCTGA
>CahiTAS2R408BP_chr5:90926429-90927327
ATGATAATATTTAGGTCAAACATTATTTCCATTCTATTAATGACAGAATTTGTTCTGGGAAATTTTGCCAGTGGCCTCAT
AGCACTGGTGAACTGCAATGACTGGATCAAGAGACCAAAGATCTCAGCTGATGGGATTCTCACTGCTCTGGCATCTGCAG
AATTGTTATGCTCTGGACAATGTTAATAAATTGGTATGTAATTGTGCATAATCCAACTCTATATAATTCAAAAGTAAAAA
TTATTGTTCATGTTGCCTGGACAGTAAGCAACCATTTTAGTAACTGGCTTGCTACTAGCCTCAGTATATTTTATTTGTTG
AAGATAGCCAACTTCTCCAGCCGAATTTTTCTTCACCTGAAGTGGAGAGTTAAAAGTGTAGTTCTCATGATGATGTTGGG
GGCGTCATTGTTTTTGTTTTTTCAAGTTGCAGTGTTAAGCATAGGTGAGGCTATTCAGACAAATGAATATGAAGGAAATA
CCACTCAGAAGACCAAACTAAGGGACATTTTACACCTTTCAAATGTGACTCTGTTCACGCTAACTTCATACCCTTCACTA
TGTCCTAGACATCTTTTCTGCTGCTAATCTTTTCCCTGTGGAGACATCTCAGGAAGATGCAGCTCAATGGTAAAGGATCC
CAGCACCAAGGTCCACATAAAAGCCATGCAACTGTCATCTCCTTTCTTTTCCTGTTTGCTGTTTACATTCTGGCTCCAGT
TTTATCAGTTTGGAATTCTAATGAGCTCCAGAAAGCTTTATGATGTTCTTTTAATCATGTATCCTTCAATCCACTCATGT
ATCTTGATCTGGGGAAACAGAAAATTAACTCAAGCCTTTCTGTCATTTCTGTGGCAGTCAAGATGCTGGCTGAAGGAAAG
GTGCCGGGGGCCAGCGTGA
>CahiTAS2R408CP_chr5:90899441-90900636
ATGATAACTTTACTATTGACCATTTTTTCCATCCTAGTATTAACCAGAGTTTGTTCTAGGAAATTTTGCCGGTGGCCTCA
TAGCACGGGTGAATGGCATTGATTGGGTCAAGAGACAAAAGATCTCCTCAGCTGATGGGATTCTCACTGCTCTGGCAGTC
TGCAGAATTGTTTTGCTCTGGGTAATGTTAATGAATTGGTACTTAGTTGTGTTGAATCCAGTTCTATATAGTTTAAAAGC
AAGAATTATAGTTCATATTGCCTGGATAGTAAGCAACCATTATAGCACCTGGCTTGCTGCTAGTCTCAGTATATTTTATT
TGTTGAAGATAGCCATTTCTCCAGCCTAATTTTCTTCACCTGAAGTGGAAAGTTAAAAGTGTCATGCACATAATACTTCT
GGGAACTTCATTCTTCTTGGTTTTTCATGTTGCAGTGATATAGACAATGAGGCTATCCAGACAAATGAATACGAAGGAAA
CATTCCTCAGAAGACCACATTGAGGGGCAGTTTATGGCTTCCACGTGTGACTCTGCTTACGCTGCTGCTGCTGCTGCTAA
GTCGCTTCAGTCGTGTCCGACTCTGCGTGACCCCATAGACAGCAGCCCACCAGGCTCCCCCATCCCTGGGATTCTCCAGG
CAAGAACACTGGAGTGGGTTGCCATTTCCTTCTCCAATGCATGAAAGTGAAAAGTGAAAGTGAAGTCTCTCAGTCGTATC
TGACTCTTCACAACTGCATGAACTGCAGCCTACTAGGCTCCTCCGTCCATGGGATTTTCCAGACAAGAGTACTGGAGTGG
GTTGCCATTGCCTTCTCTGCTGCTTATGCTGGGAAATCTCATACTATGTCCCTGATATGTTTCTGCTATTAATTGTTTC
CCTGTGGAAACATCTCAAGAAGATGCAGCTCAGTGGTAAAGGATCTCCAGATCCTAGCACCAAAGTCCATATAAAAGCCA
TGCAAACTGTGATATCCTCTCTCTTGCTGTTTGCCATTCATTTCCTGGCTCTAATGGGATCCATTCGGAGTTTTAAAAGG
CAGCAGAAGGAACCTGTCTTTTTGTTCTTTGAGGCTCTTGGATTCCTCTGTCCTTCAAATGACTCATGTACCCTGTTCTG
GGGAAACAGGAAGTTAACAAAAGCATTTCTGTCATTTCTTTGGCAGCTAAGGTGCTGACTGAGAGAAAAGAAATAG
>CahiTAS2R408EP_NW_005168073.1:402-1
ATGGTAACTTTATTACATTTTTTCCATCCTAGTAATAGAATTTATTCTAAGAAATTTTTCCAGTGGTTTCATGTCACTGG
TGAACTGCACTGACTGGTGAAGAGACAAAAAATCTCTTCAGCAGATGGGATTCTCACTGCTCTGGCAGTCTCCAGAATTG
GTCTGCTCTGGGTAACATTAATATATTGATATGTAAATGTGTTTAATCCCAGCTTTAGACAATTTAAGAGCAAGAATTAT
TATTATTGCCTGGATAATAAGCAACTATTTTGGCATCTGGCTTGCTGCTATCCTCAGCATATTTTATTGCTCAAGATAG
CCAATTCTCCAATATTATTTTTCTTTACCTAAAATGGAAAATTAAAAATATTCTTCTTGTTCACTTTGTCCTGTTTGGCT
TT
>CahiTAS2R408FP_NW_005160425.1:1-396
AGGGACACTTTACACCTTTGAAATATGACTTTGTTCATGTTAGTAAACTTCACACCCTTTGCAATGTCCCTGACGTCTTT
TCTGCTGTTAATCTTTTCCCCGTGGAAACATTTCAGGAAGATGCAGCTCAGTGGTAAAGGACCCCAAGATCCCAGCCCAA
GGTCCATATAAAAGCCATGCAAACTGTCTTCTATTTTCTATTTGCCATTTACTTCCTGGTTCTAGTTTTCTGTTTGGAGT
TCTAATAGGCAGTGGAACAACTTGGTTATCATGGCATGCCAGGCTTTTGGAATCATATGTCTTTCACTTATCCTGACATG

GGGAAAACAGATACTAAGACAGTCCTTCCTGTCATTTCTGCAGCAACTGAGTTGATGGCTAAAAGAAAGGAAATAA
>CahiTAS2R18P_chr5:90932314-90932971
TTTGGGCACTAATGAATCACTTAACTACCTGGTTTGCCACCTTCCAAAGCATATTCTGCTTCCTTAAGATAGTCAATTTC
TCTCACTGCTTTTTCACGTGGCTGAAATGGAGAATGAACCGAGTGCCTCTTGCGCTTTTCCTGGCCTCTTTCTTCTTATT
ATCTTTTGACCTCTTCATGCAAGATGCTCTTGGTGAGCTGTGGATGAACACCTTTAGAGAACCTGAAAGGAACATGACTT
TGCATTTAGGTGCAAGTAAAATTTTCTGTCTTAAAAGCCTGATTCTTCTCAGCTTGACATATGTTATCCCTTTCATTCTC
TTCATGGCTTCTTTGCTGCCTTTCTTTCTTTTCCTGGCGAGACACATCAAGAATTTCCAAGTCAACTTGAACCACCGAGA
GATTTCAGCACAGAGGCCGATAAAAGGGCCTTGAAAATGGTGACAACATTTCTCCTCCTCTTCATCGTTTACTTTATTTC
TACTCCAACTGGAAATTGGATCTTCCTTAAGCTACACTGGTATGAGGTCATGATGTTCGTCATGGTGATTTCAACTCTCT
TTTGGTCAGGCCACTCATTTGTTATAATTTTGGGGAACAGCAAGCTAAGGCAGGTTGTCTTCAGACTACTGTGGGGTCTT
AAGTTCTCTAAAAACTAA
>CahiTAS2R372P_chr5:91008874-91009796
ATGTCAAGTGTAATCAAAAAAGTTTTTATAATCATTAAAATCTTAGAATTCATAACAGGAATTTGCAGAAAAGAATTCGT
TGCACTAGTACTCTGTGCTGACTCTCTCAAAAGCAAGAATATCTCCTTGTTTGACTTGATCTTAACATGGTTGGCCGTCT
CCAGAACTGGCATGATATTCATAATTCTTATGTGGTGTTAGAATAGTGATCTACCCAGGAATATTTGAAAGTCATCAGGT
AATAGAAGTAATTTTTTATTTCTTCTGGAATCTGAGCAACTCCTTAGGTACCTGGTGTGCTGTCTGCCTCAGTGTCTTCT
ACTTCCTCAAGCTATCTAATTTTTCCCACCCTTTCTTTCTCTGGCTGAAATGCAGAAGAAAAGAGAGTTGTTTTTACCAT
TCTTCTGGGATTCTGTCTTTCTTTGATTTTAATTTTCTGAACATAACTTTTCATATATTTGGGGTCAGTGACCATTTAGA
AATAGAAAACAACTTGCATTGGAAAAAATATGCATAAAATCTGGTCCTCGAGCAGTCAAATTCTCCTCCACCTGGGATCT
CTCATCCCCTTGGCTGTGTCACTCAGTTTATTTTTCTTGTTAATCTTTTCCTTATGGAAACATACCAGGCAGATGACAAA
TCATGCCAAAGGATCCAGAGACTTCAACACAGGGATTCTTGTGAGAGCCAGAAATACTTTAACTTCCTTCGTCATTTTCT
TAGTTGTGCACTATTTGGCCACATTCTTGTTAACCTGGTCCTGTTTCACACTAGAAAATGAAATGACTTTTATTGTTATT
AAGTCTGTAGCATTTCTCTATCCTTCAATTCACCCTTTTATTTTGATTCTAGGAAATGGAAAACGGAGAGAGACTTCTGT
GAATCTACTAAGGAAAATTGAATCTTGCATCAAGAGAATGTAA
>EreuTAS2R1_NW_006804048.1:3771445-3770507
ATGAAAGAGCCTCAGGTCATTATCGAGCTCATTTTGTTAGTGATACAATTCCTGGTTGGGATTTCAGCCAATGGTTTCAT
TGTGGTTGTGAACGGCATTGGCTTGATTAGACAGAAGAAGATGATTCCACTGAACCTGCTGCTTTCCTGCCTGGCAATTT
CTAGGATCTTTCTGCAGCTGATCATGTTGACCTTTACTCTGACTGTTCTGTCCTTGGTTCAAATCCCAGTTACTGCTGAG
GCTTTTGCCATATTCATGTTTGTTTATGAGTCAGGGATTTGGTTTGCCACACTGCTTGGTGTTTTCTACTGTGTCAAGAT
TGCCACTATCGTCCACCCACTCTTCTTCTGGTTGAAGATGAGGATTTCCAAGATGGTGCCCTGGCTCATCCTTGGGATTT
TGCTCTACACATTTGGCACTTCTGTTTATCATGGTAAACAAACATGGCTGGTTTCCAAAGATTCCTTTTTAAGGTTTATC
TCCGCAAATGTAACAATTCAAACCGATGGCACATTTACATTTCATATTGCCTTTGTTTTCATGGGGATCTCATTTCCACT
ACTTATCTTCCTGGTTTCTGTCCTGTTCTTAACATTTTCCCTGTGCAGACACACCCAGCAGATGAGAAATACAGCAAGGG
GCACTGGGGAAGCTGGAACAAGCGTCCACTACAGTGCGCTGATGTCCATCCTCTCCTTCCTGGTCCTCCACATGTCCCAT
TACATGACAGAGCTTTTACTCTCCTTTCAACTTTTCCACAGTCAAAGCTATATCTTTTGGTTCTTTATCTTGGTGATTGG
TACCTACCCTTGTGGACACTCCATTATCTTAATTTTAGGAAATTCTAAACTGAGACAAAGTGCAAGGAAGCTCCTTCTGT
ATGGACAGTGCTGTCGAAGACAGAACTTTAACCTCATCAAAGAGCTGGCAGTAAAATGA
>EreuTAS2R3A_NW_006804098.1:62595-63530
ATGTTGGACCTCACCAGCTGTGTGTTCTTTGTTCTCTTTGTTGCTGAGTTCATTCTGGGAATGCTGGGGAATGGTTTCAT
TGGCTGGGTCAATGGCAGGATCTGGTTGATGGGCCAAAGACTCTCTTTATCTGACTTTGTCATCACTCTCTTGATTTTAT
CCAGGATCATTCTGCTGTGCATCCTCTTCGCAGATTCAATTTTAGTGGTTTTACTTTCTACAGTGCCTGCTCATGATCTA
GGCAAACAACTTTTTGAAATTATCTGGACATTTACCAATGACTTGAGCATTTGGCTGGTCACCTGCCTCAGTGTCCTCTA
CTGCCTGAAAATCGCCAGTTTCTCACACCCCATGTTCCTCTGGCTCAAATGGAGGGTGTCCCGGGTGGTGGTGTGGATGC
TGCTGGGTGCACTGCTCCTGTCCTGTGCCAGCACCATGTCTCTGGCCTATGAATTTAAGATCTACTCTGTTCTCAGTGAC

ATTGATGGTACAAGGAATATGACTGATCACAGTAGATGGAAAAGCTATTATGAACTGATCCATGGTATTGGGATTCTGTG
GAATGTCCCTTCCCTGCTAGTGGCTGTGGCCTCTTCCTTTCTACTTATCCTCTCCCTGGGAAGGCACACAAGGCAGATGC
AGCAAAATGGTAGCAGCTCCAGAGACCCCAGCACTGAAGCCCACTGGAGGGCCATCAAAATCATCCTCTCCTTCCCCATC
CTCTTCCTACTTTACTTTCTTGCCCTTTTGTTGATATCATCTAGACCTCTCCTACCTGAGACTACAACCATTGAGATGAC
TGCCAAACTAATTAAAATGTTTTATCTTTTTGCCCACTCATTCATTCTCATCTTGGGACAAAACAAGCTGAAGCAGATAT
TTTTGGCAATGCTCAGATGTCAGCCTGGAGAGCTCACAGAATTTATTTCTTCCTAG
>EreuTAS2R3B_NW_006804498．1:1466316-1467230
ATGTCAGGTTTCACAGAGTGGGTATATCTGGTTCTGACTGTTACTCTGTTCCTGTTTGGGATGCTGGGGAATGGCTTTAT
TGGGTTGGTCAATGGCAGCAGCTGGCTGAAGAGCAGGAGGATCTCTTTGTCTGACATCATCACCACTCTGCTGGCTCTCT
GCAGCATCATCCTGCTGTGGACTCTCTTGGTTGATGTTTGCTTAATGCAGTTCCCTTTCAAGACACATGATGAAGTGAGA
ATGAATCATATTCTTGGGATTTTATGGGCATTCATGCATCATCTGGTCCTTTGGCTTCTGGCCTGCCTCAGTATCCTCTA
CTGCCTGAGAATCGCCAGTTTCTCGCACCCCGCATTCCTCTGGCTCAAGTGGAGGGTGTCCCGGGTGGTGGTGTGGATGC
TGCTGGGTGCGCTGCTCCTGTCCTGTGCCAGGGCCATGTTTTTGATCCATGAACTTAAGATCTATACTGTTTTCTTTGGA
ATTTACGAGAAAGGAAACACGACGGAGAACTCTAACAAGGGAAGTCAATATAAACTGACTCATATTCTTGGAACTCTGTG
GGACCTCCCTCCTCTGATGGCATCTCTGGTCTCCTACTTTCTGCTCCTCCACTCCCTGAGGAAGCACTCCAGGCAGATTC
AGCAATATGGTAGCAGCCCCAGAGATCCCAGCACCGAGGCCCACGAGAGAGCCATCAAGATCATCTTTTCCTTCTTCTTT
CTCTTCCTATTTTTCCTACTGGCTCTTATATTTGCATCATCCAGGCATTACCTCCCAGGAGCTGAGAGGATCAAGATGAT
TGGAGTAGCAGTTGTAATGTTTCATTGTGTTGGTCACTCATTTATTCTTATTTTTGGGAACAGCAAGCTGAAGCAATTGT
TTGTGCAGATGATGAGATGTCAACCTGCCATCTGA
>EreuTAS2R3C_NW_006804498．1:1445770-1446723
ATGGCAGGATTCCTGGAATCCTTATTTCTGGCTCTGACCGTCATTTTCTTCATCCTGGGAATGCTGGGAAATGGTTTCAT
CGGGTTGGTCAATGGCAGCCGCTGGGTAAAGAGCAAGAGGATCTCTTTGTCTGACTTCATTATCACGGCCCTAACTCTCT
CTAGGGTCATCCTGCTGTGGATTCTCTTGGCCGATAGCACTTTAATAGTGTTCTTTTACAGAGTACACAATGATGGTATG
GGAATGAAAATTATCGACATTTCCTGGACCTTTATAAACCACCTGAGCATTTGGCTGGTTACCAGTCTCAGTGTCCTTTA
CTGCTTGAAAATTGCCAGTTTCTCGCATCCCATGTTCCTCTGGCTCAAGTGGAGGGTGTCCCGGGTGGTGGTGTGGATGC
TGCTGGGTGCGCTGCTCCTGTCCTGTGGCAGCACCATGTCTCTGGTCCGTGAATTTGAGTTCTACTCTCTATTCTATGGA
TTCAACGGTACTGAGGATCCAGCGGAACACCTTAGAAAGAAGACTGACTATGAAGTGACCCATACGCTGGGGATCCTATG
GGATCTCTTTCCCTTCATCGTGTCTCTGGCCTCTAACACTCTGCTCATCCTCTCCCTGAGAAGACACACACGCCAGATGC
AGCAGAATGGAAGCTGCTCCAGAGACACAACGACCGAGGCCCACCAGAGGGCCATCAGAATCATCCTCTCCTATCTCCTT
CTCTTCCTGTTTTACTATCTAGCCTTTCTAATTACCTACTGCAGCCGTTTCCTACCAGAAACGAAGATGATCAAGATGTT
TGGAGAAGCAGTCACATTGCTTTATCCCGCTGGCCACTCATTCATTCTCATTATGGGAAACAAGAAACTGAAGCAGATGT
TTGTACAGATACTCAGGTGTCAGCCTGGTCATCTGAAACCTGGACCCAAGGGCACCGTGGACTCCCTGAAGTGA
>EreuTAS2R4A_NW_006804498．1:1456654-1457556
ATGCCTCACATCCTTTTTATCTTTGCTGTTGTCATTGCTACGACTTCAAGCTTTGTGGGTCTTCTTACAAATCTGTTTAT
TGCAGTGGTCAATTATAGGAGTTGGATGAGAAGTCACAGAATGTCTTCTTCCGACAGGATCCTGTTCAGCTTGGCCATCA
CCAGATTTCTGATGCTGGGACTGTTTCTCCTGAATGTTATCTTCTTCTTTAGCTCTCCAACTCTTCTAAGGTCAGTCCGC
GTAGTCATATTTTTTGTGTTGTGTTGGATGTTTTTGGACTCTACTAGTCTCTGGTTCGTGACCTTGCTCAACACCTTGTA
CTGTGTGAAGATTACTAACTTCCAACACTCAGTGTTCCTCCTGCTCAAACGCAATCTGTCTTCAGTAGTCCCTGGGCTGT
TGCTGGTATGTGTGCTGATCTCTGCCTTTACCACTCTGCTTTATTCTGTGCTCAGACTGACCTCACCCTTTGCTCCACAT
GTGACTAGGGAAAATGGCACGAGGATTGACAAGAATGGTGACACCTGGTCTTTGCTGATCTCTTTGGTCTTGAGGTCGTT
CCTGCAGTTCATCATTAATGTGACCTCTGCTTCCTTGCTCATCAATTCCTTGAGGAAACACATCCAGAAGATGCAGAGAA
GTGCCACTGGATTCTGGAACCCCCAGACGGAAGCTCACGTGGGTGCCATGAAGCTGATGATCTATTTCCTTGTCCTTTAC
ATCCCATATTCAGTTACAACCATGTTGCTTTACTTGCCTTCTTCTGTAGGGATGAGTTTGAAAGCCAGATCTATTTGTGT
GATTATTTCTAGCCTTTGCCATCCGGGACATTCTATTCTCATTATTCTCACACATCCTAAACTGAAAACAAAAGCCATGA

AGTTTCTTTGTTTCAACAGGTAG
>EreuTAS2R4B_NW_006804098.1:39208-40128
ATGCTTCAGTTTATTTCGTCTGTTCTTATTGCCTCTGCAGTTTTTAATTTTGTTGGTCTCCTTGCAAATCTGTTTATTGC
AGTGGTCAATTACAAGTCTTGGATGCAAAACCATAGAATCTCTTCATCTGACAGGATCCTATTCAGCTTGGCCATCACCA
GATTTCTGATGCTGGGATCATTTCTCCTGTTAGTTATCTACCAGTTTCTCTTTCCACTTGTTCTAAGGTCAGTCTTCGTC
CTCATTTTTTCAAGGCTGTGTTGGGTGTTTCTGGAGTCTACTAGCCTCTGGTTCATGACCTTGCTCAACACCTTGTACTG
TGTGAAGATTACAAACTTTCAACACTCAGTGTTTCTCTTGCTGAAACGGAATCTATCTCCAAAGACTCCTTGGCTGCTGC
TGGCTTGTGTGCTGATCTCTTTCTTTATCACTTTGCTTTACTTTGTGCTCAGACTGACCTCACCCATTCCTCCCTATATG
CTTGGGAGAAATAACACCGAGATTGACTTGAATGCTGAAGTCTTGCCTATACTGATGTCTGTAGCCTTGAGTTGTTGTCT
ACAGTTCATCCTTAATGTGGCCTCTACTTCCTTGGTAATCAAGTCCTTATGGCAACACATAAAGATGATGCAGAAAAACA
ATACAAAGTTTTGGAACCCCCGGACAGAAGCTCTTGCAGGTGCTATGAAGCAGATGATCTATTTCCTCATGGCATATGTT
CCACATTCTGTTCTGATGCTGCTCTTTTACCTAAATATCGCCATAGGAGAGACTGTTGAACACTTAACCATTTATTCATT
TTTTTCTACTTTGTACCACCCAGGGCATTCTGTTCTCATTGTCCTCACACATCCTAGGTTGAAAGCAGAAGCAAAGAAGA
TGCTTTGTTTCAGCAGGTCATGCAAGTTTGATTGTAAATAG
>EreuTAS2R7_NW_006803966.1:4490926-4489988
ATGTCAGAGAAAGTGAACAGAATCTTAATGTTTGTTGTGACTGGAGAATTTGTAGTGGGGATCCTAGGGAATGCATTCAT
TGGACTAGTAAACTGCATGGACTGGATCAAGAAAAGGAAAATTGCCTCCATTGATATGATCCTCACAAGTCTGGCCATTT
CCAGAATTTGTCTCTTGTGTATAATATTATTGGATTGTTTCACGTTGGTGCTATATCCAGATGTCTATGCTACTGGTAAA
CAAATGAGAATCATTGACTTTTTCTGGACACTAACCAACCATTTAAGTGTCTGGTTTGCCACCTGCCTCAGCATCTTCTA
TTTCCTCAAGATTGCGAATTTCTTCCATCCTCTTTTCTTCTGGCTGAAGTGGAGAATTGACCGCCTGGTTCTAAAGATCC
TACTGGGGTGCTTGGCCATCTCCGTTTTTATTAGCCTTCCTGTCACAAGGAATCTGAACAATGATTTCAGGCTTTGTGTC
AAGGCCAAGTGGAAAAGAAACTTAACGTTGAAATGCAGAGACAGTAAAGCTCAATATGCCTCCATTAAGATCTATCTCAA
CCTGTTAACGTTTTTCCCTTTTACTGTGTCCCTAATCTCATTTCTGCTCTTGATCCTCTCCCTGTGGCGACACACCAAGC
AGATGCAGTTCACTGCCACCGGCTGCCAAGACACAAGCATGCAAGCCCACGTGGGAGCCATGAAAGCTGTCATCTCCTTC
CTCCTTCTCTTCATTGCATACTATTTGGCCTTTCTTGTGGCCACATCCAGCTACTTTCTACCAGAGACTGAATTAGCTGT
GATGGTTGGTGAGTTGGTAGCTCTCATTTACCCCTCCAGCCACTCATTTATACTAATTATTGGAAACACTAAATTAAGAC
AAGCATCTCTACAGGTGCTATGGAAAGTAAAGACTACCTTCAAAAGAAGAAATTTCTAA
>EreuTAS2R372_NW_006803966.1:4527878-4526952
ATGTCAACCTCAGCCAAAACAGTGTTTGTCACCATAGAAATTTTAGAATCGGTGGCAGGTGTTTGGGGAAACGGTTTCAT
TGTAGTAGTGCTCTGTGCTGACTGGATCAAAACCAAGAAAATCACCTTGTTTGATTTCATCTATACTTGCTTGGCTATCT
CCAGGATTGTTATGATATACCTACTCTTGGAAGACAGTATTGCACTTGCATTCAATCCAGAATTAATTGCAAAGCACCCC
AAATTAATCGTCATTGCTGATTTTTTCTGGAATCTGAACAGCTCCTTAAGTAGTTGGTGTGCTACCGGCCTAGGTGTCTT
CTATTTCCTCAAGCTGTCCCAGTTTGCCCACCCCTTCTTTCTCTGGCTGAAGTGGAGAAGGGATCAAATCGTCTTGGCCA
TTCTACTAGGGTTCTTTCTTTCATTGCTTTCTCATCTTCTAAGCATAAAATTTTATATACTTTGGATCAACCAGTATGAA
AGAGAGGGAAGAAACCTGAGCTGGAGAGGATGGGCACATAAAAGCCAATACTTCAGCAAACAAGTTCTCCTCAATGGGGG
ATCCCTCATGGCTTTCCCCATGTCCTTCATTTCATTTTTCCTGTTAATAATATCCCTGGGGAGACATACCAGGCAGATAA
TGCATCATGCCAAGGGATTGAGAGGCTTCAACACAGAGGTTCATGTGAGAGCCAGAAATACTATGATTTCCTTCATCATT
TTCTTAGTGGTGCACTACACATCCACTTTGCTGATAATCTGGACCTATAATGAAGTAGAAGACTTCTTCGTTGTAGTCAT
TACTGAGACTATAGCATTACTTTATTTTTCAGTGCATCCTTATAGTATGATTCTGGGGAATGGAAAACTGAGGCAGACCT
TTGTGAATCTCATAAAGAGAACTGAATGCTGTATAAAGAGAGACTAA
>EreuTAS2R10_NW_006803966.1:4520205-4519273
ATGCTAAGTGTTTTGGAAGGCCTATTCATTTTTGTAGCAGCAAGTGAATCGGTTCTGGGGGTCTTAGGGGATGGATTCAT
TGGAACTGTATATTTCATTGAGTTTGTGAAGAACAAAAAGGTCTCCATGATTGGCTTTATTCTCACTGGCTTAGCTATTT
CCAGAATTTGTCTGATACTGTTGATCATTATCGATGGATTTAACAAGATCTTCTTTCCAGATATGTATGCCTCCAGTACC

CTAATTGACCATATTAGTTACGGGTGGACAATTATCAATCAATCCAGTGTCTGGTTTGCCACCAGCCTCAGTATGTTCTA
TTTCCTAAAGATAGCAAATTTTTCCCAGCGTGTTTTCCTCTGGCTGAAAAGTCGGACCAATAAGGTTCTTCCCCTTCTGA
TGGGATCTTTGCTTATTTCATGGTTAATTACTTTTCCACAAGTCATAAAGGTTATTAATGATAATAGAATGAAGAAGGTG
AACACAACCTGGCAGTTCAGTGAGGAGGCTAGAAATCAGTTCCTTACCAATCAATTCTTATTCAGCCTGGGAGTTTTTGT
CTTCTTTCTCCTCACCTTGATCACAAGCTTCTTGTTGATCATTTCCCTCTGGAAGCACCGTAGACAGATGCAGCTGAATA
TCACAAGATTCAGAGACCCCAGCACTGAAGCACATATGAAAGCCATGAAAATTTTAATCTCTTTCATCATCCTCTTTATC
TTGTATTTTATAGGCATTACCATCGAAATTTTATGCTCTACAGTAACACGAAACAAATTGTTGTTTCTTTTTGGCTTGTC
AACCACAACAATCTATCCATGGGGTCACTCATTGATCTTAATTCTAGGAAACAGCACGTTAAAGCAATATTTTTTAAGGG
CACTGCAGCAATTTAAGCGTCATGAGCAAGAGAATCCTTCTAGAACCCAATAG
>EreuTAS2R11A_NW_006803966.1:4547928-4548869
ATGCTCAGTGCAGTGGAGAAAGTTTTCATGGTATTATCAATTGGGGAATTTATAATAGGAATTTTGGGGAATGGATTCAT
CGGACTTGTATATTGCATTGCCTGGATTAGAAATCAGAAGCTATACTTTGTTGACTTCATTCTTATTAGTTTGTCCTGCT
CCAGATTCAGTCAACTGTGTATAGCAAATATGGATTTCTACTTAGTATTGTCCTCTCAGAAAATCAGTGATGTTAAGGGA
AAAAATCCAGTTCTTAATCTTCTCTGGCTACTGAACAACCACCTGAGTATTTGGCTTGCTACTTGCCTTGCTGTGTTTTA
TTTCCTGAAGATCGCCAATTTTTCCCATCCTCTCTTCCTCTGGCTGAGATGGAGAGTCAACAAGGTCATCTTCATGCTTT
TTCTGGGGTCTGTGCTTTTCCTGTTCATGACCTTTCCTTCACCGTATGGTTTTGAAGTCTTCTGGTGTCACATCCCAAAC
AATTATGATAACAATGTGACTGGGTCATTTGATGTGAGTAAAGTTAATTTAAACTATATGATTATTTTTATAATTGCCTC
CATCCCTCCTTTCTCTCTCTCCCTGACCTGCTTTTTGCTGTTGCTTATTTCTTTGTGGAGACATGTGAAGCAGCAACTTG
AGCTCAATGTTAGATATTCCAGAGACCCCAGCATGGAGGCTCACTTCAGAGCCATGAAGACTGTATTTTTCTTTCTCCTC
TTCTTTGTATTGTACCAATTTTCCCTTGTGATGACATTTGGGGGACATTTGTTGCTGCATAATAAGCTTATTGTGATGTT
TGGTTATACCATAGGGCTCTTATATCCTTCAGGGCACTCATATATTGTGATTTTTGGAAACAGTCAAATGAGGACAGTCT
TCTTAAGGATGCTTTGGTACCTGCAATGTGGCCTGAAAAGACAGGTTCTCTCAGGTACCTAA
>EreuTAS2R14_NW_006803966.1:4564252-4563323
ATGGCCAGTGTCGTAAACAGCACATTTCTAGTTATAATCAGTGCTGAAATCATACTGGGAAGTCTAGGAAATGGCTTCAT
AGTGCTGGTGAACTGCATCAACTGGATTCAGAAAAGAAAGGTCTCCTCACTGGATCAAATTCTCACTGGTCTGGCACTCT
CCAGAATTGGTTTGTTCTGGGTAGTACTAATAAATTCATTCATGCTTGAATTTGGCCCATCATTATTTGTAATAGAAAAA
GTGTTAGAAGCAGTTGTCTTTACTTGGGCAGTCACCAATCATTTCAGCATCTGGTTTGCTACAAGCCTCAGCATCTTCTA
TTTTCTCAAGATCTCCAATTTTACTAACTCTGTTTTTCTTTACCTGAAGTGGCGGGTCAAAAAGGTAGTCATGATGGTAC
TACTGTTGACCTTGGTCTTCTTGATTTTTAATGTGGCACTGATGAAGATGTATATTAATTACTGGTCCAACAGGCTTAAG
AGCAACATGACTCACAATTCCAGGATGAGAAACTTCGTGAAATTTTCCAATCTTTTTGTATTCACCAATGCCATGTTCAC
ATGCTTACCCTTTGCTGTGTCCCTGATAATTTTTCTCTTGCTCATCTTATCCCTGTGGAAACATCTCAAGAAGGTACAAC
AAAGCGCCAAAGAGTCCAGAGACACCAGCAGCAAGGCCCACATCAAAGCCCTGCAAACCATCATTACCTTCCTTCTACTC
TATGCCATTTTCTTTTTGTCACTTCTTGTGTCAGCTTGGAGCTCTGATTGGGTAGAGAAAATTCAGATTACTCTGCTTTG
CCAGGCTATAGGAAATCTCTATCCCTTAGGTCACTCTTGTGTTTTGATCTTGGTGAACAGCAAGCTGAAACAGACTTTTC
TTTCAGTTCTGAGGGTCAGGTTCAGTGATGGGGCACATGCTGGCCCATAA
>EreuTAS2R18_NW_006803966.1:4631066-4630125
ATGTCAGTTGGAATGAATGTCTCTTTTCTGGTGGTGACAACAGGAGAATTCCTCTTAGGAATGCTGGGCAATGGGTTTAT
TGGAGTGGTCTACTGTATTGAATGGGTCAAGAATGGAAAGATCTCATTGGCTGATTTCATCCTTACCAACTTGGCTATGG
CCAGAATCATTCAACTGTGGCTAATTCTGATGGATGCATTCATAATGGTGCTGTCTCCAAATCGGTATGGTGCTGGTAAT
CTAGCAAAGGTGATTTCTGTTCTGTGGACATTAAGTAATCACCTAACTACTTGGCTTGCCACTTGCCTAAGTGTTTTCTA
CTTCCTTAGGATTGCCACTTTTTCCCATTTCTTTTTCATCTGGCTGAAGTGGAGGGTGAACAAGGTTATTATTGTGATCT
TTCTGGGGTCTTTCTTTTTCTTGTCTGTTAATCTCTTCACGCAGGATGTTATCAGTGAGCTGTGGATAAGTACTTACAGA
ATCCATGAGAGAAACACAACGTTGCCCTCTGGTGAGAACAAAACTTTGTATCTGTATACCCTTCCTTTTCTTAGCCTGGT
CTATATTATTCCCTTTTTCCTGTCTCTGACAGCTTTGCTTCTTTTATTTCTGTCCTTAGTGAGACACACCAAGAATTTTC

AATCCAGCCAAATGGGCTTGAGGGACTCCAGCACAGAGACCCATAAAAGGGCTTTGAAAACAGTGGCAACATTTGTTCTC
CTCTTTATCATTTACTTTATGGCGTCCCAAACAGCTTGCTGGATCTTCCTTAAGATAGAGAGGTATCAGGTCATTATGCT
CATCATAATGCTTTCAGCTGTCTTTCCCACAGGCCATTCTTTCATTATAATTTTTGGAAACAGCAAGTTAAGACAGATTG
CCTTGAGATTACTGTGGTGTCTTAAGTTATCTGGAAGAGAAACAGAACATCTAGCTTTATAG
>EreuTAS2R408A_NW_006803966.1:4606330-4605413
ATGAGGAATCTACTGCTGAACCTGCTTTTCTTCCCAGTAACAGTAGAATTTGTGATAGGAAATGTTGTAAATGGCTTCAT
AGTGCTGATGAACTGTATTGATTGGGTCAAGAGACATAAGATCTCTTCAGTGGACCAGATTCTCACTGCTCTGGCACTCT
CCAGAATTGGCTTATACTGGGCCATGGTAACAAATTGGTATATAGCAAGATTTTATTCATCACTCTGTACATCAGAAGCA
ATAATCACCATTCACATTGCCTGGATATTAAGCAACCATTTCAGCATCTGGTTTGCTACGAGTCTCAGCATATTTTATTT
GTTCAAGATAGCTAATTTCTCCAATTCTTTCTTTCTTTATTTCAAGAGGAGAGTGAAAGGTGTAGTTGTGGCAATACTGT
TGGGGAGTTTGACTGTGTTAGGCACTCAGCTTGCAGTGGCCACCTTAGATAACAATGTCAGGGTGACTGAATATAAAAGA
AACATGACTTTGAAGACCGCCTTTGAGGACCAGATACGTCTTTCAAATATGGCTGTATTCACGCTAGAGAATTGCATACC
CTTTACTATATCCCTGATGTGTTTTGTGCTGTTAATTTTTTCCCTGATGAGACATCTCCGGAAGATGCAGCTCAGTGGCA
GAGGAGACCGGGACCCCAACACCCAGGTCCACATCAGAGCACTGAAAACTGTCATCTCCTTTCTCCTGCTGTTTGCCTGT
TACTTTCTGGCTCTCATCATCTCAGCTTGGGGTTCAAACAGGTTGCGCAACAAATATGTTCTTCTGTTTGGTGAGATTAT
TTTTGAGCTGTATCCTTCAGGTCACTCCTTTATCCTAATCTGGGGAAACAAAAAGCTGAAAGAGGCCTTTCTGGGGTTTC
TGTGGTCGCTGATGTGCTGCCTAAGAGGGAGGGAATAA
>EreuTAS2R408C_NW_006803966.1:4611358-4610441
ATGCTAACTTCAATACCAAACTTTTTCCCCATCCTATTGACAACAGAATTTGTTCTAGGAATTCTTGTCAATGGCTTCAT
TGTGCTGGTGAACTGCATTGACTGCATCAAGAGACACAAGATCTCCCTAGTGGATCGTATTCTCACTGGCCTGGCATTTT
CCAGAATTGCCTTGCTCTGCGTTATAATAATAAATCTGTATGCAACTTGCTTTGACTCAACTTTTTATGGTTTTGAAGTC
CACATTCTTGTTAATATTGCCTGGACAGCAAGCAACCATTTTAGCATTTGGTTCGCTACGAATCTCAACATATTTTATTT
TCTCAAGATCGCCAACTTCTCTCATTTCTTATTTCTTTATTTAAAAAGGAGACTTAATAGTGTACTTCTTGTGATGATAT
TGGGAACATTAGTCTTCTTGTTTACTCATCTTTCAGTGATAAACATAGATGAAAACATGCTGAAGAATGGCTATAAGGGC
AACATCACCTGGGAGACCAAGTTAAAAAACATTGCACGTCTTTCAAATATGACTATATTCACACTAACAAACTTCATATC
CTTTGCTATGTCCCTGATGTCTTTTGCGCTGTTAATTATTTCCCTTGGGAAACATCTTAAGAAGATGCAACTGAATGCCA
AAGGAGCCCAAGACCCTAGCACCAAGGTCCACACAAGAGCCCTCCAAACTATGATCTCCTATTTTGTGTTATTTGCTGTA
TATACGTTGGCTCTAATTATCTCCGTTTGGATTTCAAACAGGCTGCAGAACAAACCTGCTGTCTTGTTTTGTCAGGCTCT
ACTAAGCCTGTATCCTTCCAATCACTCTTTTTTCTTAATCTGGGGCAACAAGAAGCTGAAAGAGGCCTTTCTGTCATTTC
TGGGGCAGCTGGGGTGCTGGGAAAAAGAAAGAAAGTAG
>EreuTAS2R38_NW_006804098.1:194899-193901
ATGCTGACTCTGAGTCCTGTCATCACCGTGTCCTATGGAGTCAAGAGTGCCTTTCTGGTCCTCTCCATCCTGGAGTTCTC
CCTGGGGATCCTGGTTAACAGCTTCATTGTCCTGGTCAATCTCTGGGACGTGGTGAGAAGACAGCCGCTGAGTCACTGCA
ACCTGGTCCTGCTGTGTCTGAGCCTCACCCGGCTCTTCCTGCATGGGCTGCTCTTCCTGGAGGCCATCCAGCTCATCTAC
TTCCAGAAGATGAAAGACCCACTGACCCTGAGCTACCAGACCATCATCTTGCTCTGGATGATTGTCGAGCAAGCCAGCCT
CTGGCTGGCCACCTGCCTCAGTCTCCTCTACTGCTCCAAGATCATCCGCTTCTCTCATATCTGCCTGGTCCACCTGGCCC
GCTGGGTCTCCCAGAGGATGGGGCAGCTTGTCCTTGGGGCCGTCTTCTCCTCTGCAGTCTGCACTGTCTTCTGCTTGGGG
TGTTACTTCAACCAAACTGGCTTCTTGGACAACAGTGTGTTTTTCCTGAATGGCAGTGCCGAGCAGGCCTTGCAGGTGCC
CAAGCTCAACTTGTTCTTCCACTCCTTCCTCTACTGCAGCCTGAACACTGTCCCCACATTCCTGACTCTTCTGGGCTCCT
CGGGGTTGCTGATTGTCTCCCTGGGGAGGCATCTGAAGACCATGAGGGCCAGCTCCAGAGGTGCTGGTGACCCCAGCCTG
GAGGCCCACATCAGAGCGCTCAAGTCCCTGGTGCCCTTTCTCTGCCTCTACATCGTGTCTTTTGGAGCAGCACTGCTCTC
CTTACCTCTGCTGTTGCTGTGGCACAAGAAGGTTGGAGTCATGGTCTGTGTGGGCGTCATGGCAGCCTGTCCCTCAGGAC
ACGCCATCATCTTGATCACAGGGAACCCCAAGCTGAGGAGGGCTGTGAGGAGCCTGCTGGTCTGGGCTCAGAGCATCAGG
AAGGTGAGGGTGGAGTCCAGGAAATCAGTGCTCTGCTGA

>EreuTAS2R40_NW_006804098.1:1555964-1556935
ATGGCTACAGTCAACAGTGATGCCACAGATAAAGACACGTCCCCATTCAAAATCCTTCTCACCTTGGTGGTCTCCGGAGT
CGAGTGCCTCACAGGTGTGATTGGGAATAGCTTCATCACGGCCATGCATGGTGCTGAGTGGGCCAGAGGCAAAAGACTTC
AAGTAAGTGACTACACCTTGTTGTTCTTGAGTTTCTCCAGGCTCTTGCTCCAGATTTGGATGATGGTGGAAATTACCTGC
AGCCTCCTCTTCCGGGTCACCTATAACCAAAACAAGGTGTACATCCCCTTCAAGGTCATCCTGATGTTCCTGAACTATTG
CAACCTCTGGATGGCCGCCTGGCTCAATATCTACTACTGCCTGAGGATTGCCCATTTCCCGCGTCCTCTGTTCCACTGGA
TGAGGAGAAAAATCGTGGTGCTGATGCCTTGGCTTCTGAAGGGGTCTCTGGTCATCTCCTTAGGTGGTGGCCTTTCCTTC
TGTAAAGACATATTCAACGTGTACGTGAATAACTCCACCCCGATTCCTGCTTCCAACTCCACAGAGAACAGGTACATCTC
TGAGACCAATGTGACGAACCTGGTTCTCCTCCTTTATCTGGGCATCTTCGTGCCTTTGATCCTGTTCATCTTGGCTGCCA
CCCTGCAGATCATCTCGCTCCGGAGACATGTCTTGCACATGGGGAGCAAAGCCACAGGCTCCAGGGACCCCAGCATGGAG
GCCCACGTGGGGGCCATCAAAGCCATCAGCTATTTCCTCATCCTATACCTCTTCAATGCAGTGGCTCTGTTTCTTTCTCT
GTCCAACAAGTTCGACACCAACAGCTCCTGGAATATTCTCTGCAAATTGATCATGGCTGCCTACCCTGCTAGCCACTCCG
TACTCCTGATTGCAAGCAACCCTGGGCTCAGAAGAGCCTGGAGACGAGTTCAGCACCGAGCCCATCTTTATCTCAAATGG
CAGAATCTGTGA
>EreuTAS2R41_NW_006804098.1:2005017-2005943
ATGCAAGTAGCACTGGCATGCTTCTTCATGTTGCTCTTTGTTCTACTGTGTCTCCTGGGAATCCTGGGCAATGGCTTCAT
CGTGTTGGTGCTGAGCAGAGAGTGGAGGCGCCATGGGAGGTTGCTTCCCTCTGACAAGATCCTTCTCAGCCTGGGTGCCT
CCCGTTTCTGCCTGCAGTGGGTTGGGATGGCAAACAACTTCTACTACTTCCTCCACCTGATGGAATACAACAGGGGACCT
GCCCGGCAGGTCTTTGGTCTCTACTGGGACTTCCTGAACTCAGCCACCTACTGGTTTGGTACTTGGCTCAGCGTCCTTTT
CTGCATAAAAATTGCCAACTTCTCCCACCCTGTCTTCCTGTGGCTGAAGTGGAGGTTCCCAGGATCAGTGACCTGGCTTC
TGTTGGGTTCCCTCCTCATCTCCTGCATGGTCACCTTTCTCTTCTTTTGGGGAAACCACTTTATGTATCAAGGATTGAGG
ACTAGAACATTTTCTGGAAACTTAACCTACAAAGAGTGGACCAAGAAGATGGAAATTTGCTATTTCCTGCCCCTGAAATT
TGTCACTTTGTTTGTGCCCTGCTCTATCTTTCTGGTCTCCATTGCATTCTTGATTCACTCTCTGAGGATGCACCTGTGGA
AGATGAGTCACCATGCCCACAACCTGCAGGACCCTCTCACCCAGGCTCACACCCAGGCTCTGAAGTCACTCATCTGCTTC
CTCCTCCTTTACACTCTATCCTTTGTGACCCTGGTCATTGATGCAGCAGGCTTCTTCTCCTCTGAGAGTGACTGGTACTG
GCCATGGCAGATTTTGATTTATCTGTGCACATCTGTCCATCCCTTGATCCTCATTCTCAGCAATCTGAGGCTCCGGGGGG
TGTTCAGGCAGCTTCTCCTATTGGCCAAATACCCCTGGGTAGCCTAG
>EreuTAS2R408B_NW_006803966.1:4665446-4664529
ATGGTAAGTTTGCTAACAGGCATCCTTTACATGATAGTCATAGCAGAATTTGTCACAGGAAATGTTGCCAATTGCTTCAT
AGCACTGGTGAACTGCACTGACTGGGTTAAGAGACAGAAGATGTCCCTAGTGGATCATATTCTCACTGCTCTCGCGCTTT
CCAGAATTGGCTTGCTCTGGACAATGTTAATTTATTCTCATGCAGTGGTATTTGATTCAGCTTCATACAGTCTTGAAGTG
AACATGTCATTTCTTACTTTCTCAATAATAAGTAACCATTTTAGCCTCTGGTTTGCTACAAGTCTCAGCATATTTTATTT
ATTCAAGATAGCCAATTTTTCTGGTTTTGTATTTCTGTATCTGAAGTGGAGAATTAAGAAAGTGTTGCTTAGTTTAATGT
TAGGGAGTCTGGCCATTTTGATTTCTCAGATTGCAGTGGAAGGCATATATGACAAGATGCTGGTCTATGAAGATGAAGGA
AACCTCATGTGGAAGGGCACACTGAAAGCCAGTTTACTTCTTTCAAGAATGACTATCTTCACCCTAACAAACTTCATCCC
CTTTATTCTGTCCCTGATAGCTTTTGTACTATTAATCTTTTCCCTGGGGAAACATCTCTGGAAGATGCAGTTCAGTGGCA
AAGGAACTAGAGATGCCAGCACCCAGGTCCACATTAGAGCCCTGCAAACTGTCATCTCATTTCTCCTGGTGTTTGCTGGG
TATTTCCTGACTCTAACTGGCTCACTTTGGAGCTTTTACAAGAAGCAGAGCAGATCACACCTTCTAGTCTGCCTGGCCAT
AGAAATCCTGTGTCCTTCAAGCCATTCATTTGTGCTGATCTGGGGGAACAAGAAGTTGAAAGAGGCCTTTCTATCTTTTC
TGTGGCAGCTGAAGTGCTGGCTGAGAGAAAAAGTGTAG
>EreuTAS2R60_NW_006804098.1:1963716-1964672
ATGCATGGAGACAGTTCTGACCCACGACCACTGGCAATTGACAAGGGTACTATCACCCTGACTGTCTTCTTACTTCTTTT
GTGCCTGGTGGCGGTGGTGGGTAATGGCCTCATCATCGCAGCCCTGAGCGTGCAGTGGTTGCTTCGGAGAATGCTGTCAC
CTTGTGACAAGTTGCTGGTGAGCCTGGGAGTCTCTCGCTTCTGTCTGCAGTGGATGGTGATCACTAAGACCATTTATATT

TTCCTCCACCCCCAGGCCTTCCCATATAACCCTGTGTGGCAGTGTCTCTCCTTCCAGTGGGACTTCTTGAATGCTGTCAC
CCTGTGGCTCTCGGCCTGGCTCAGCATCTACTACTGTGTGAAAATTGCCACCTTCACCCAGCCAGTCTTCCTCTGGCTGA
AGCGTAAGGTGTCCGGGCTGGTTCTGTGGATGCTTCTTAGCTCTGTGGGCTTCTCCAGCTTCAGCGCTGTGCTGTTTTTA
ATTGGTAATCATCAGGTATATCAGAACTACTTAAAGAGAAATCCTCAGATTTGGAATGTGACCGGAGATGCTGTAAGAAG
ATCCTACGAGAGATTCTACTTCCTTCCCCTCAAACTTGTCACCTGGACGGTGCCAGTAATGGTCTTCCTGGTGGGCATGG
GTTTGCTCCTTTCATCTCTGGGAAGACACACTTGGAAGACCATCCCGTTCATCTTGGGTGGTGACAACCTCAGCACCCAG
TCTCACGTCAAGGCCCTGCTGGCTCTGGGTTCCTTTGCTGTGCTCTTTGTTTCGTACTTCCTGTCACTGCTGCTCAGTGC
TGCAGGGATTTTTCCTTCTGAGGAGCTTAGATACTGGGTGTGGCAAGCAGTGATTTACCTGTGCGCAGCCATCCATCCCA
TCGTCCTGCTCCTGAGCAGCCCCAGACTGAGAGCTGTGCTGAGGAAGGTCCACTTCTCCAGAAGGTGGCCATCTTGA
>EreuTAS2R62_NW_006804098.1:1954038-1954961
ATGAGATCTGTACCCACGTTGATCTTCATGACCGTCTTTGTCGTAGGGTCATTGGCTGCCATGTTGCAAAATGGCTTCAT
GGTCTTTGTGCTGGGCTGGGAACGGGTGCAATGTGGGAAGCTGGCTGCTGGGGACATGATTGTGAGCTGTCTAGCTGCGT
CCCGGTTCTGCCTGCATGGGATATCTCTCTTGAACAACCTGCTGACCTTCCTTTCTGTTTGTCCAGAAGTGATCTATTTT
AACATCCCCTGGGACTTTATCAACACTCTCATGTTCTGGCTCAGTAGCTGGCTTGCATTCTTCTACTGTGTGAAGATCTC
ATCTTTCTCTCATCCCATCTTCTTCTGGCTCAAGTGGAGGATTTCTTGGTTGGTGCCCAGGCTGTTGTTGAGCTCCCTGA
TCCTTGCTTCTCTGACAACCATCTCATCCACCATTGGGAATATAAGACTTGTCAGGATGCTCACCTTGCTGAGTTTCTGT
GGAAATGATACCCTGTATAGTAGTAGACTACAAGACTTTTCTCAACATGTTTTTCTGCCTTCTTTGCTGCTGGCGTTATC
TCTTCCTTTCCTATTGATCCTGGTGTCCACCCTTCTGCTTATGTTTTCACTGCACCAACATTTGCAGAACATGAGGGATT
GCAGATCTGGTTCCCAGGATCTGAGCACTCGGGTTCACACGGTGGCCCTGAAGTCACTTACCTTCTTCCTTATCTTCTAC
ATGTCATATTTTCTGTCCTTGGTATTTGTGACATTAAAAATCACCATCTTCAAGAACCACTGGCACTGGATCTGGGAAGC
GATTACCTACATAGGCATCAGCCTCCATTCTAGCATCTTGGTGCTCAACAACTCCAAGCTGAGAAAGGCCCTACAGATGA
ACCATTGGAAACACTCTGTTCAAAGTGTGTTTTGTCTTGAGTGA
>EreuTAS2R4CP_NW_006804098.1:17786-18688
ATGCTTCACATTTTATTTTTATTTACTCTTCTCTTCTCCTTAGCATTTCATTTTGTTGGACTCGGTGCCAGTCTGTTCAT
TGCAGTGGTCAGTTACAAGAGTTGGATGCAAAGCCATAAGATCTCTTCATCTGACAGGATCCTGTTCAGCTTGGCCATCA
CCAGATTTCTGATGCTGGGACTGCTCCTAGTGGACATTTTTTGCTTTTGCATCTTTCCAACTTTTATAAGGTCAGTCCAT
GTCAACACTTTCTTAATGTTGTGTTGGATGTTTTTGGACTCTACTAGTCTCTGGTTCGTGACCTTGCTCAACACCTTGTA
CTGTGTGAAGATTACTAGCTTCCAACACTCAGTGTTTCTCTTGCTGAAACGGAATCTGTCCCCAAAGACCCCCAGGCTGC
TGCTGGCCTGTGTGCTGATCTCTTTCTTTACCACTCTCCTTTACTTTGTGCTCAGACTGACCTCACCCTTTGCTCCACAT
GTGTCTGGGGAAAATGGCACAAAGACTGACACGAATGGTGACACCTGGTCTTTGCTGACCTCTTTGGTCTTTAGCTCATT
CCTGCAGTTCATCATTAATGTGACCTCTGTTTCCTTGCTCATCAATTCCTTGAGGTAACACATCCAGAAGATGCAGAGAA
GTGCCACTGGATTCTGGAACCCCCAGACAGAAGCTCACGTGGGTGCCATGAAGCTGATGATCTATTTCCTTGTCCTCTAT
GTTCCATACTCAGTTACAACCATACTCATTCATCTCCCTTCCTCTGCTGGCATGGGTTTGGGATGGTTAGCCGTTTGTGT
AATTTTTTCTAGTCTTTACCCTACTGGTCATTCTCTTCTCACTATACTCACACATCCTAAACTGAAAACAAAAGCAAGGA
AGATGATTTGTTTCAACAAGTAG
>EreuTAS2R11BP_NW_006803966.1:4533960-4533062
ATGGTAAATACATTTGACAAAGTTTTCATGCTCTTAGTTGGAGACTTTATAGTAGGTATTTTAGGAAATGTATTCATTGG
ACTCATATATTGCATTACCTGGATCAGAAGCCGGAAGTTATACTTGGTTGACTTCATGCTTATTAGCTTGGCCTCTGCCA
GGTTCAGTCTACTGTGTCTACTAATTGTCAGTTTCTATTTATTGCTGACTTCAAATCAAATTTATGATGTTACAGGTCAA
TACCATGGCTATCATAGTCTGTGGATACTGAACAGTCACTTGAGTATTTGGTTTGCTGCTTGCCTTACACTGTTTTATTT
CCTGAAAATTGTCAGTTGTTCCTACCCTCCTCTCCTCTGCCTGAGATGGAGAATTAACAAGGTCATTTTCATGCTGCTGC
TGGGGTCTGTGCCTTTCCTGTTTATGAGCTTTGCTCCACAATATGGTTTTCACTTCTGCTGGCATTGCGTTCCAAACAAA
AATAACAGAAACATGGTTGAGTCATTTCATGAAAGCAAAATTAACAATTTAAATGCTTTGATTTGTTGCATAGTTGTGTC
CATCTCTCTGATTTTTCTCTCCTTGATTTGCCTTTTGCTCTTGCTCCTATCTTTACAGACACTGAAAGCACATTAAGAGC

AATGCTAATATCTCCAGGGACTCCAGTATGGATGCCCACATCAGAGCCATGAGGACTGTGTTCTCTTTTCTTGTTTTATT
TGTATTGCATCAGATTGCCCTTTTCATGATATTTTCAGGGTATTACTTGCTACAGAATAAGTTTATTGTGGTGTTTGGCT
ATGTGATAAGGCTCTTATATCCTCCAGGGCATTCATATGTTGTGATTTTTGGGAACAGCCAAATGAGAACAACATTCTTT
GGTGTGCTTTGGTACCTGA
>EreuTAS2R11CP_NW_006803966.1:4552580-4553487
ATGTTGAATATACTGAATAAAGTTTTCCTGGTTGTAGCCAGTGGGGGATTTATAGCAAGAATTATAGGGAATGAATTCAT
TGGACTCACAAATTGCATTGCTTAGATTAGACATCAGAAGCTATACTAGGTTGACTTAATTCTTACCACACTGGCGTGCA
TCAGAATCACTTAAATATGGCTATCACTCATTGATCTCTGTTCCATGGTATCCTCTCAGGAAAACTGTGATGCTCTGGAA
AGAAAGTGTATGCTTGTGACTATCTGGATACTGACGGATTACTTAAGCCCTTGGCTTGCCACTTCTCTTGCTGCATTTTA
CTTCCTGAAAATAGCCACTTTCCCCATCCTCTCTTCCTCTGGCTGAGATGGAGAATGAACATGGTCATTTTTGCACTCCT
GCTGGGGTCTGTGCCTTTCCTGTTCATAAGTCTTCCATTGCCATATGGTTTCGATGTCTTCTGGCATTATGAAAAATAAG
TTATAGCAGAAATATGACTGGGATAACAAATGTAGCTGTAGTTAGCAACTTTAATTACATGATCAGTTTTACAGTTGTAT
CCATCCCTCCTTTCTGCCTCTCCTCCATTTCCTTTGTGCAGTTGCTTGTATCTTTGAGGAAACATACAAAGCACCTTGGC
CCCAATGCCATGGGTTCCAGGGACCCCAGTATGGAGGCCCACTTTAGAGGTGTGAAGACTGTGATTTTCTTTCTCAAGCT
CTATGCACTGCACCATGTTTCCCTACAGGGGAACATACAGGGGGGGGGACATTATTTACTCCATAGCAAACTGACTGTGA
TGTCTGGTTATTTGCTTGGGCTTCTGTACCCTTCAGGTCACTGTTATGTTGTGATTTTTGGAAACAGTCACATGAAGATG
ACTTTCTTGGGGATACTTGGGCGCCTGA
>EreuTAS2R408DP_NW_006803966.1:4574694-4574026
GAAGTGAGAATCTTTGCTCACTTTGTCTGGATCACAACCCATCATTTTAGCACCTGGCTTGCAACTTGCCTCAGCATATT
TTACTTGCTCAAGATCGCCAATTTCTCTAGCTTTCTGTTCCTTTATTTGAAGTGGAGAGTTAAAAGTGTCATTCTAACAA
TTCTCTTGGGGAGTCTGGTCTTTTTGGTTTCATATCTTGCAGTGGTAAAGAGGGTAATGACAAATAAAGTAAACATGACT
TGGAATTTCAAATCATGGGGCTTTCCACAAGTTTGGAATATGAGTGCATTTACACTAGGAAGTTTCATCTCCTTGGTGAT
GTCCCTGATATCTTTTGTGTTGCTAACATTTTCCATGTGGAAACATCTCAGGAAGATGCAATGTAGTGGCAACGGAGCCC
AAAATTCCAGTTACAAAGTCCACATTAGAGCCCTGCAAACGGTCTTCTCTTATATCTTGCCTCTTACTGCTTATATCCTG
GCTCTAATGATCTCAGTCTGGAATTCTAATAGCCTGTCGAATAAACCAGTCATCCTGTTTTGCCAGACTCTTGGAGTTTT
GTATCCCTCGATTCATTCAGTTTTCCTGATTTGGGGAAATAAGAAGCTGAAAGAGGCCTTTCTGTGGTTCCTGTGGCAGC
TAATGTGCTGGCTGAGAGAAAGGGAGTGA
>EreuTAS2R408EP_NW_006803966.1:4593116-4594037
ATGAGTGGTCTTCTACTGTGCATTCTTTCCACCATAATAGTGACAGGCTTTGTTGTAGGGAGTGTTGCCAATGGTTTCAT
AGTGTTGGTGAACTGTCTTGACTGCACCAAGAAAACACAAGATCACCTCAGTGGATGGGCTCCTGACTGCTCTAGCGCTC
TCCAGAATTGGTTTGCTTTGGCTAGCGCTACTCTATTGGCACCAAATTCTCTTTGATTCAGTTTCTTACCACTTGGAATG
GAGTATTTTTATTCACATTTCCTGGGTTTTAAGCACCTATTTTAGCATCTGGTTTGCTGTGAGCCTCAGTATATTTTACT
TGCTGAAGATAGCCAATTTCTCTAACCTTGTCTTTCTTTACCTAAAGTGGAGAGTTATTAAGGTACTTCTCATATTAATA
CTAGGAAGTCTGCCCTTTTTGATCTCTCAGTTTACAGTGATAAGCATGCATGACAATATGTTGATAAATGAATGTTAAAG
AAACGTCACTTGGAACACCTAATTGAAGGATATTGTGAACCTTCCAGTTATAACTGTCTTTACACTTGCAAACTTCATAC
CTTCTACTGTGTTGCTGATATGTTTTGTGCTGTCAATTTTTTCCCTAAGGAGACATCTCAAGAAGATGCAACTCAGTGGC
AAAAGAGCCCGGGACCCCAGCTCCCAGGTCCACAGAAGAACCCTGCAGCAAACAGTCATCTCCTTTCTCCTGCTGTTTGC
CTGTTACTTTCTGGTGCTGATGATCTCAGTTTGGAGTTCAAAAAGGGAACCAAAGAAACTGATCTTCATTTTCTGCTTGG
CTGTAGGGATTCTGTATCCTTCAGGTCACACATTTGTGCTGATTTGGGAGAACAAAAAGCTGAAAGAGTCCTTTTTGACA
TTTCTGTGGCAGTTGAGGTGCTGGTTGAGAGAAAAAGTGTAA
>EreuTAS2R408FP_NW_006803966.1:4586762-4587684
ATGATCTATTTCCTGGAAAATATTCTTTTTACCCTAATAATAACAGAATTGATTATAGGAAATGTTGTCAATGGCTTCAT
ATCTCTGATGAACTGCATCATCTTGGTCAGAAGACAACAGATTTCCCCAGTGGACAGAATTCTCACTGCCTTAGCATTCT
CCAGAATTGCTTTGCTCTGGGCAATGTTTAAAAACTGGTATGATTTATTGTTCAATGCAACTTTATACAATGTAGAAGAA

TCGCTTTTTGTTCATGTCACCTGGGTAATATGCAACTATTTTAGCACCTGGTTGGCTACAAGTCTCAGCATATTTTATTT
GCTCAAGATTGCCAATTTCTCAAACTTTGTATTTCTTTATTTAAAGAGGAGAGGTCAACGTGTAGTTGTCATAACACCAT
TGGGGAGTTTGAGCTTGTTAATTACTCAGCTTGCTCAGATCAGAGTACAGGGCAATGTAACCTATATGGGGGAGGGACAC
AAGACAAACTTGACTTGGAAGAATGCATTACAGGACCAAAGAAGCCTTTTAAATATGACTATATTCACTATAGAAAGCTT
AAAACCTTTTACTATATCTCTTATGTGTTTTGTGTTGCTAATCTTTTCTATGGGGAAACATCTCAAGAAAATGTGGCTGA
GTGGAAAAAGAGCCCAAGACCCCAGCACCCAGGTCCACATTCGAGCCCTGCAAACTGTCATCTCCTTTCTCATTTTATTT
ACTGGGTACTTCCTGACTCAATAATCTCAACATGGAACTTAACCTGGCAGCAGAACAAAACATTTTTGCTGCTTTGCCAA
ACTCTTTTACATCTGTATCCTTCAGTTCACTCTTTTATCCTGATCTGGGGAAACAAGAAGCTGAAAGAGGCCTTTCTGTG
GTTTCTGTGCCAGCAGAGATGCTGGTTGAGAGAAAGGGAGTGA
>EreuTAS2R39P_NW_006804098.1:1489072-1490008
ATGCAACAATCCTACACTTTCCCCAAAGATGGACTATCACGCTTAATTCTAACCATCCTAGGTTCTGAATGCATCTTTGG
TATCTTTGCCAATGGCTTCATTGTGGCCATCCATGCAGCTAAGTGGATTCAGTTTCCACCAGTGGCAGAATCCTGCTCTT
CCTGGATGGATCCAAAATTGCTCTCCAGAGCTTTATGATGCTAGAAATAACCTTCAGTTCAATACTTCCACACTGTTATA
ATGAAGATGTCATATATGAAGCCTTCAAAGTAAGTTTCATGGTGTTAAATTATTGTACCCTCTGGTTTGCCACCTGCCTG
GGTTTCTTCTACTTTGTGAAGATTGCTGACTTCACCTACCCTCTTTTCCTCAAGCTGAAGTGGAGAATTTCAGCATGGAT
GCCCTAGATTCTGTACCTGTCTGTGTTTATTTCCTTGTGCTACAGTAGGCTCTTTGTTAATGGCATCTACCATGTGTAAT
TCAATGATTCTTTTCCTATTCCCTCCTCCAACTCCACCAGGAAAAATTACTTCACTAGGACTGATGTAGTCAGCCTGGTT
CTTCTCTTTACCTCAAGACTCTTCTTTCCCATGACCCTGTTCATCCTTGTAGCCTCACTGATGATCATCTCTTTCAAGAG
ACACACCCTACACATGGAGAGCAAAACCACAGGCTCCAGGGACCCCAGCATGGAGGCCCACAGGGGGGCCATCAAGGCCA
CCACTTACATCCTCATTCTTTATGTGTTCAATGCAACTGCTCTATTGCTTCACATGTCTAACATCTTTGATGTTGACAGT
TTCTGGAATGCTTCGTGCAAGATCATCATGGCTACCTCCCTTACTAGCCACTCAGTGCTACTGATTCTGGACAGCCCTGG
GCTGAGAAGAGCCTGGGAGCAATTTCAGCACCAGGTCATCTTTACCTGAAAGAATAA
>EreuTAS2R42P_NW_006803966.1:4695554-4694613
ATGATTACTGGATTGGACAGTATCTTTATGATACTGTCATCAGTAGAATTCATAATTGGAATAGTAGGGAATATGTTCAT
TGCACTGGTAAACTGTTCAGAGTGGCTCAAGAACCGAAAGCTCTCTTTATCTGACTGCAACCTCACCTGCTTGGCTATCT
CCAGAATCAACCGTTTGTCTGTTTCTTTGAATCCTTTGTCATGGGACCATATCCACAACTATACACAACTTATAAGCTAG
CAAAGTCTTCTAGTTTGCTTTGGAGACTGACTAGCCATATGACTATCTGGCTGGCTACCTGTCTAAGGACTTTCTACCTC
CTTAAGATAGCTCACTTCTCACACTCCCTTTTCCTTTGGTTGAAGTGGAGAATACACAGAGTGATTCTTGTGATTTTTGT
GTTTTCTATCTTCTTTCTGATTATTGACTTTCTGTTGTTAGAAACATTTAATGATATTTTCTGGCAAAATCAAGTACTTG
ATAAAAATAACCTGAGTTTCCGTTCAGAAGAAAGACAGAATTCCTATGTGAGATCTTTGATTTTTCTCACCTTGACCAAT
TGTGTTCCTATTGCTCTGTCCTTGACCTCACTGCTCCTTTTATTTCTGTCCTTGGAGAGACACACCAAAAATCTTCAGCT
CAATTCCATGAGCTCCAGGGACATCAGCATATTGGCCTACAAAAGGGCCATAAATATGGTGATGTATTTCCTTTTCCTCT
TCATAGTTAATTTCATTTCCATACAGTTGGCAAATTGGATTTTTCTTATGTTTTGGAAAAGCACATTTACAAAATTTATC
CTGCTGGCAGTATATATCTTTCCCTCCAGCCACTCATATATTTTAATTCTAGGAAACATCAAACTAAGGCAGATAGCTTT
GAAAGTTTTAAAAAATATATATCTTAAAAGTATCTTGATCAGGAAATTCATTGGCTTTATGT
>EreuTAS2R408GP_NW_006803966.1:4582732-4583650
ATGAAGGAATTGTTGCTGAATATCCTTTCCATCCTGATAACAGCTGGGTTTGTGGCAGGCAATTTTGTCAATGGCTTCAT
AGTGTTGTTGAGCTGTATGGATTGGGCCAAGAAACAAGAAATCTCCCTGGTGGATGGAATCCTCACTGCTCTGGCTCTCT
CCAGAATTGGCTTGCTCTGGATGACTTTAATAAATTGGTACGCTGGGTTGTTTTACTTTTCATGCTCTAACTTAGCGGGA
GGCACTGTTGTGAATACTACCTGGGCTTTATTCTACCACCTTAGCCTGTGGCTTACTACAAGTCTCAGTATATTTTACCT
GCTGAAAGTTGCTAATTTCTCTTGCTTTCCATTTCTTTACTTAAAGAGAAGGATCAAAAGCGTGGTTTTTTATAATACTT
CTGGGAAGTCTATCCCTTTTTGTTACTCATATTGCTGTGACGGGCAAAGATGAAAACATGTGGACAAAGGAATACAAAAG
AAACAGCACTCAGAAGACCCAGTTGATGAACCAAACACGTCTTTTAGGTATGACTATCTTCATGATGGAAAACTTCATTC
CCTTTACTGTGTCTCTGACATGTTTTCTGCTGTTAATCTTTTCTCTGGTGAAGCATCTCAAGAAGATGCAGCTCAGTGGC

AAAGGAACCAGAGACACCAGCACCCAGGTCCACATAAGAGCCCTGCAAATTGTTATCTCCTTTCTCCTGCTATTTGCTTG
TTACTTCCTGGCTGTAACCCTTTTAATTGGGGGTTCAAATAGAATGGAGAACAAGCCTGCTGTCCTGTTTGCCCAGATCC
TCATTGAATTGTATCCTTTGGTTCACTCCTTTATCTTGATTTGGGGGAACAAGAAACTGAAAGAGGCCTTTCTGTGGTTT
CTGTGGCAGCTGAGGTGCTGGCTGAGAGAAAGGGAGTGA
>EreuTAS2R408HP_NW_006803966.1:4589124-4590034
ATGATCCATTTCCTGGCAAACATTCTTCTCATCCTAGTAATAACAGAGTTTGTTGTAGGAACTGTTGTCAATAGCTTCAC
AGTGTTGGTATATTGCGTTGATTGGGTCAAGAGCCAGGAGATCTACCAAGTGGATGAAATTCTCACAACTCTGGCACTCT
TCAGAATTGTTTGCTCTGGCCAAAATTGAAGAGCTGGTTTGTAGTACTGTTTAACGTAGTTTTCTATGTCTTAGAAGAAG
TATCCATTATTGCTAATGTTTTCTGGATATCAAACAACCATATTAACATCTGATTGGTTACAAATCTCAGCATATTTTAT
TTGGCCCATGTAGTGAATTTCTCTAACATTGTATTTCTTTACTTAAAGAGAAGAGTTAAAGGTGTGATTGCCATAATGCT
CTTGGGGAGTTTGACTTTGTTACTCAGCTTGCAATGTCCAGCACCAGATGACAATGTATGAATGAATGGATATACAAGGA
ACATGACTAGACAAATCATATTAAAGGACCAAATATTGTTTTCAAGTTTGACTATATTCATGATAGCAAACATCATACCA
TTTACTATGTCCCTGATATCTTGCATGTTGTTAATTTTTTTTCCTTGATGAGACATCTCAAAAAGATGCAGTTCAGTGAA
AATGGAGCCTGAGACCCCAGCACCCAGGTCCACACAGGAGCTCTGCAAATTGCCACCTTCTTTGTCCTGCTATCTGCCAG
TTACTTTCTGGTTGCAGTCATCTCAAGTTGGTTTTTCACATGGCTTCACAACAAATCAGTATTCCTGCTTGGGAAGATTT
TTTTTTTCAACTGTGCCCTTCATTTACTGTGATTTGGGGCAACAAGAAGTTGCAAGAGGCCTTTCTATCATTTTATGGCA
GCTGAGGCGCTGGCTGAGAGAAAGGAAGTAA
>EreuTAS2R408IP_NW_006803966.1:4647857-4647160
ATGTTCCTATCTCAAATTATGTCTATCCCAGTATTGGCAGGATTTGTTCTTGGAAGTTTTGCCAATGGATTCATAGCATT
AGTGAGTGGCACTGACTGGATGAAGGGTCACAAGATCTCCCCAGTGGATAGGATCCTCACTGCTCTGGCTTGCTCCAGAA
TAATCTTGCTTTGGGTTATGTTATTTCATTGGTATGCGACTATGTTTAACTCAGCTTTTTACTGCTTAAAAGCATTTCAA
TTTACTGTTCTCTTTTCCTGGACAGTAAGCAATTACTTCAGCATCTGGTTTGTGGCATGCCTCAGCATCTTTTATTTGCT
CAAGATTGCCAGTTTCTTTAACCTGCTGTTTCTTTACCTCAAGTGGAGAGTGAAAAATGTACTTGTTATAACCCTGTTGG
GGAGTGTTCCTCTTTTGGTTTCTCAGTTTACAGTGGCCAGCATAAATGAGACCAATTTCATAAGAGAATATGTAGGAGAA
GTGACTTGAAAGACCAAATACAAAGATATTCTATATTTTTCAAATGTTACTGTGTTCATTCTCACAAACTGCATTCCTTT
CACTATGTCGCTATTATCTTTTGTGCTATTAATCTTTTCCCTGGGGAAAGATCTCCAGAAAATGCAGCTCAGTGGCAAAG
GAGCCGGAGACACCAGCACCCAGGTCCATGTAAGAGCCTTGCAAACAGTCATCTTCTT
>EreuTAS2R408JP_NW_006803966.1:4668904-4667988
ATGTTAACCCTACTACTAGGTATTCTTTCCATGCTTCTAATAACAGGATTTGTTCTGGGAAATCTTGCCAATGGCTCCAT
AGTACTGACAAACTGCATTAACTGGGCCAAGAAACACAAACTCTCTGCTGCAGACCAATTTCTCACTTTTCTGGCACTCT
CTAGAATTGGCTTGCTTTGGGTGATATTAATAAAGTGGTACAGAGTTGTGTTAAATTCAGCCTCTTATGGGTTTGAAGTC
TTTAGAATTATCATTCTTGTTTCCTGGATAATCAGCAACCATTTCAGCATCTGGTTTGGTACCACTCTCAGCATATTTTA
TTTGCTTAAGATAGCCAATTTCTTCAGTTGTATGGTTTTTTTTTTTTTTTTTTTTTTTTTTTTACCTAAAGAGACAAGTT
CATATCATACTTTTCATAACACAGCTAGGGAGTTTGGTCTTTCTGATTTCTTATCTTGCAGTGGTAAGCTTACAAGTGAA
TTTTCTGCTAAGTGATTACAAAGGAAACCTCACTGGGAAAATGAGCTGCATGTCATACCGTTCAAAAAGGACAGTATTCA
CACTAGAAATCTTCATACCCTTTACTATGTCCCTGGCGTCTTTTGTTCTGCTAATCATTTCCCTGGAGAAACATCTTAAG
AAGATGCAGCTCAGTGGCAAAGGAGCCAGAAATCCCAGCACAGATGTCCATATAAGAACTCTGCGAACCACCATCCCCTT
TCTCCTGCTCTTTGCTGGCTACTTCGTGGTTGAAATCATCTCAGTTTGGGATTTTAAAACACAACATACCAAACGTCCGC
CTCCTTTGCAAGACTTTTGGAATGCTGTATCCTTCAGCTCACTCATGGGTGCTGATTTGGGGAAAGAGGAAGCTGAAAGA
GACCTCTCTGTGGTTTCCGTGGCAGCTGAAGGGCTGG
>EreuTAS2R408KP_NW_006803966.1:4567650-4566780
ATGATACTGATGAGTCTTCTTTCCATTCTAGTAATATCAGAATTCCTCCTAGGAACTTTTGCCAATGGTTTCATAGTATT
GGTCAACTACATTGACTGGGTCAGGCAACAATAGATCTAACTCTTCAGTGGACTGGATTCTTTTACCCTGGAATTCTCCA
GAATTGGTTTGCTCTGGGTAACAATAATAAGTTGGCATACAGCTATATTTAATCCAGCCATATATACTTTAAAAGCAAGA

ATTATTGCTAATATTGCCTGGATAACAAGCAATCATTTTAGTGTTTGGCTTGCTACAAACCTCAGCATATTTTATTTGCT
CAAGGTCACCAATTTCTCTAGCTTTGTATTTCTTTATTTAAAGTGGAGAGTAAAAGTGTACTTCCTATAATCCTTTTGGG
TACTTCGGTCTTTTGGATTTTACATATTCCAGTGTCCATCCTAGATGAAAATATATTTGTGAATGAATATAAAGAGAACA
TCACTTGGAACTGAGGGACACTGTATTCTATTCACATATGATTGTATTCATGGTAACAAACTTCAAACCCTTTACTGTGT
CTCTGATATGTTTTCTTTTGTTAATCTTAAGAAGCTGCAGCTCAGTGACAAAGGAGCCCGAACCCCAAGCACCCAAGTTC
ACATACGAGCCCTGCAAACTATCATCTCCTTTTTCCTGCAGTTTGCCCTTTATTTCCTCTCTCTAATCATCTCAACTTGG
AGTTCTAACAAATTGCAGAACAAACAGACCCTTGTGTTTTGTCAGGCTCTTGGCATCCTGTACACTTCAAACCACTCATT
TGTACTGATCTGGGGAAACCAGAAACTAAAACTGTTCTTTCAGTCTTCATGCAGGCAAGGAGCTGGCTTAA
>EreuTAS2R67P_NW_006803966.1:4684615-4683903
ATGCCATCTGGATTTGAAAATACTTTCCTGGTGGTGACAATAGGGGAATTCATAGTTGGAATGCTAGGGAATGAGTTCAT
TGCATTTGCCAACTGCATAGACTGGGTGAAGACTAAAAAGCTCTTATCAGGTGACTGTGTCCTCACCAGCTTGGCCAACT
CAAGAATCATCCATCTTTGGTTATTGCTATTTGAATCACTCATAATGGTGTTGTGGCCACATCTATATGATAATGGTAGA
CTAGTAACATTTATTAGTGTTTCATGGACTCTGACCAATCATTTCAATGGCCTGGTTTGCTACCTGCTTAAGTGTTTTCT
ACTTCTTTAAAGTAGCCAACTTCTCCCAACCCTGCTTTCTTTGGTTGAGGTGGAGAATCAGCTGAGTGCTGCTTGCACTT
CTACTGGGGTCTTTGATCTTACTGATCATCAACTTTGCCCTAATAGATACATTAAATAGTTCTTGGATTGGTGGCTACCA
AAGACACGAGAGAAACTCGACTTGGTCCTCAGATGTAAATAAAATTCCATATATTAGCAGTGTGGCTGTTTTCAACTTGA
TCTACTTTATCCCCTTTCTTCTCTCCCTCACCTCACTGGGCCTTCTGTTTCTCTCTTTGAGGAGGCACATCAAGAATCTG
CAAGTCAACCGTGGCTTAAGGGACCTCAGCACAGAGACCTATACAAGGGCCATGAAAATAGTGATGTCATTCC
>EreuTAS2R5T_NW_006804098.1:78685-79527
GAATTTCTCATTGGACTTATTGGAAATGGAATCCTTGCTGTCTGGAGTTTTGGAGAATGGGCCAGGAAATTCAAGAGGTC
CTCATACAACCTCATTGTCTTGGGCCTGGCTGTCTGCAGATTTCTTCTTCAGTGGATGATAATGATAGACTTAAGCCTGT
TTCCTTTTATTCAGGACAGGCATTGGCTTCAATATCTCAATATCTCCTGGGTTTTAATCAGCCAGGCTAGTCTGTGGTTT
GCTACTTTCCTCAGTGTCTTCTACTGCAGGAGGATTGCAACCTTTGAACATCCTGTCTGCCTGTGGCTGAAGCAGAGGGC
CAACTGCCTGAGTCTCTGGTGCTTTATGGGGTACCTCCTGATCAGTCTGTTCCTTGTAACCCACATTAGCTTAAAATACA
GCAGTCCTTCTCTGGGGAACAGCAGCTTTCTCTACCCACTTTCAAATGCGCACTATATGTCTATATTACGGCTCAATTCA
GGATGCTGGCTGCCTTTCCTGGTGTTTCTTGTTTCCTCTGGGATGCTGATTATCTCTCTGTATAGTCATCACAAGAAGAT
GCAGGTCCATATAGCTGATAGGAGGGATGCCAGAGCCAAGGCTCACATCACTGTGCTGAAGTCCCTGGGATGCTTTCTCA
TACTTCATGTGGTTTACATCTTAGCCAGTCCCTTGTCTATCACCTCCAAGTCATCTGCTAATCTCTCTACTGTCTTTATT
TCCAAGACTCTCATGGCAGCCTACCCTTCTCTTCATTCTGTCATATTGATCATGGGGAATGCTAGGATAAAGCAGATTTG
CCAGAGAATTCTATGGAAAACCATCAGTGCTTGGAGATCCTGA
>EqcaTAS2R1_chr21:49882403-49883302
ATGCTTGAGACTTACCTTTTTGTCTATCTTCTTTCTGCAGTAATTCAATTGCTCGTTGGAGTTTTAGCAAATGGCATCAT
TGTGGTTGTGAATGGCACTGGCTTGATCAAGCAGAGAAAGATGATTCCATTGGACCTCCTTCTTTCCTGCCTGGCGATTT
CTAGAATTTGTCTGCAGTTGGTCATCTTCTGCATTAATCTGAATGTTCTCTCCTTGACTGAATTTACTATATTTCCGGAC
AATTTTGCAATTTTCACGTTTGTAAATGAATTGGGACTTTGGTTTGCCGCATGGCTCAGCGTTTTCTACTGTGCCAAGAT
TGCCTGCATCGCTCACCCACTCTTCTTCTGGTTGAAGATGAGGATAGCCAAGTTGGTGCCATGGCTGATCTTCGGGTCCC
TGCTATATGCATCGATCATTTCTGTTTTGCATAGCAAACATACAGGGATTCTGTTCCAAAAAATCTGGTTGGACCTTTTC
TCCAATAACACAACAGCTCAAATCAGAGAACTATCTGTTTTACAGCGTTCCTTTCTTGTCATTGAGTTCTCATTACCGTT
TCTTATCTTCCTTTTTTCTACTCTGCTCTTGATATTTTTCCTGGGGAGACACACCTGGCAGATGAGAAACACAGTGACAG
GCACCAGGAATGCTAGCATGCGTATCCACATCAGTGCACTTCTGTCCATCCTGTCCTTCCTGGTCCTCTACCTCGCCTAC
TATGTGATGCCTGCTTTATTCTTTTCTCAAATTTTTAAGCTCAGGAACCCCATCTTTCTATTCTGCCTCTTTGTGGTTGG
ATCATACCCCGGTGGACACTCTGTTATCTTAATTTTAGGAAATCCTAAACTGAAACAAAATGTGAAGAAGACCCTCCTTC
ACAGTAAGTACTGTCAGTGA
>EqcaTAS2R2_chr4:45437715-45438620

ATGGTCTCCCCTTTATCAGCTATTCTTCATGTTCTCATCATGTCAGCAGAATTTATCACAGGGATTACAGTAAATGGATT
TCTTATAATCATCAACTGTCATGAATTGATCAAAAGCAGAAAGCTAACACCAATGCAACTCCTTTTTGTATGTATAGGGA
CGTCTAGATTTGGTCTGCAGATAGTGTTAATGGTACAGAGTTTCTTCTCTATCTTCTTCCCACTCTTATATGCTGTAAAA
ATTTATGGTCCAGTGATGATCTTCCTTTGGATGTTTTTTAGCTCTGTCAGTCTCTGGTTTGCCACCTGCTTGTCTGTATT
TTACTGCCTCAAGATAGCAGGCTTCACTCAGTCCTATTTTCTTTGGCTGAAATTCAGAATCTCAAAGTTAATGCCTTGGC
TGCTTCTGGGAAGCCTGCTGGCCTCCGTGAGCATTGCAGCTCTGTGTACGGAGGTAGATTACCCTCTACACGTGAGTGAT
ATCCTCAGGAACACCACGCTAAAGAGAACGGAACTCAAGATAAAGCAAATTAGTGAAGTGCTTCTTGTCAACTTGGCATT
GATATTTCCTCTGGCCATATTTGTGATGTGCACTTTTGTGTTATTCATTTCTCTCTATAAGCACACTCGTCGGATGCAAA
AAGGACCTCATGGTTTCAGCGATGCCAGCACAGCAGCCCATATAAATGCATTAAGAACAGTAATAACATTCTTTTGCTTC
TTTATTTCTTATTTTGCTGCCTTCATGACAAATATAACATTCAGTGTTCCTTACAGAAGTCAGAACTTCTTTGTGTTGAA
GTTCATAATGGCAGCATATCCCTCTGGCCATTCGGTTATTATAATCTTGAGTCATTCTAAGTTCCAACAACCATTCAGGA
GACTTCTCTGCCTCAAAAAGAATTAA
>EqcaTAS2R3_chr4:94566379-94567326
ATGTTGGGACTCACTGAGTGGGTGTTTCTGGTTCTATCTGCCACTCAGTTCCTTCTGGGAATGCTGGGGAATGGTTTTAT
AGAGTTGGTCAATGGCAGCAGCTGGTTCAAGAACAAGAGAATCTCTTTGTCTGACTTCATCATCACTAACCTGGCTCTCT
CCAGGATCGTTCTGCTGTGGGTTCTCTTGGTTGATGGTGTTTTAATGGTGTTCTCTTCCAAAGTACGTGAGGAAAGGATA
GTAATGCAAATTATTTATGTTTCCTGGACATTTACAAACCATCTGAGTATTTGGCTTGCCACCTGTCTCAGTGTCCTCTA
CTGCCTGAAAATTGCCAGTTTCTCCCACCCTACATTCCTCTGGCTCAAGTGGAGAGTTTCCAGAGTCGTCATATGGATGC
TCTTGGGTGCGCTGCTCTTATCATGTGGCAGTGCCGTGTCTCTGACCCATGGATTTAAGATTTATTCTGTTTTCCGTGGA
ATCAATGGCACAAGGAATGTGACTGAGCACTTTAAAAAGAGAAATGAATATGGATTGATCCATGTTCTTTGGACTCTGTG
GAACCTCCCTCCGTTAATTGTGTCTCTGGCCTCCTACTTTCTGCTCATCCTCTCCCTGGGAAGGCACATGTGGCAGATGC
AGCAAAATGGTACCAGTGCCGGAAATCTAAGCACTGAAGCTCACAAGAGGGCCATCAAAATCATCCTCTCCTTCCTCTTT
CTCTTCCTACTTTACTTTCTTGCCTTTATAATTACAACAGCCAGTGATTTCCTACCAGGAACTAAGATGGTTAAGATGAT
TGGAGAAATAATTACAATGTTTTATCCTGCTGGCCACTCATTTATTCTCATTCTGGGAAACAGCAAGCTGAAGCAGATGT
TTGTGGAGATGCTCTGGTGTAAGCCTGGTCATCTGAAGTCTGGATCCAAGGGCTCCTTTTCCCCATAG
>EqcaTAS2R4_chr4:94571727-94572647
ATGCTTCGGATATTTTTTATCTGTTCTGTTATTGTCTCAGTAATTTTGACCTGTGTAGGACTCATTGTGAACCTGTTTAT
TGCAGTAGTCAATTATAAGACTTGGGTCAAAAGCCACAGAATCTCCTCTTCTGATAGGATCCTGTTCAGCTTGGGCATCA
CCAGATTTCTTATGCTGGGAATTCTTCTACTGAATAATGTCTACATCAACTCTCTGAAAGTTGAAAGGTCAGTCTACTTA
TCCACTTTTTTCCTGTTGTGTTGGATATTTTTGGACTCTAATAGTCTCTGGTCTGTAACCTTGCTCAACGCCTTGTACTG
TGTGAAGATTACTAACTTCCAACACTCCATGTTTCTCCTGCTGAAACGAAATCTCTCCCCAATGATCCCCAGGCTACAGC
TGGCCTGTGTGCTGATTTCTGCCTTCACCACTCTCCTGTATGTTTTGCTCAGACAGACATCACCCGTCCCTGAATTTGTG
ACTGGGAGAAACAGCACAGTATTTAACATCAATGAGGGCGTCTTGTTTTTGGTGACCTCTTTGGTCTCGAGCTCATTTCT
CCAGTTCATGATTAATGTGACGTCTGCTTCCTTGTTAATAAATTCCTTGAGGAGACATATACAGAAGATGCAGAGAAATG
CCACTGGCTTTTGGAATCCCCAGACTGAAGCTCATGTGGGTGCAATGAAGCTGATGATCTGTTTCCTCATCCTCTACATT
CCATATTCAGTTGCTGCCCTACTCCATTATGTCCCTTCTTCTGTAGGGATGAATTTAGGAGCCAGATCCATTTGTGTGAT
TGTTTCCGCCTTATACCCTCCAGGACATTCTGTTCTTATTATTCTCACACATCGTAAACTGAAAACAAAAGCAAAGAAGA
TTCTTTGTTTCAACAAATGGTGGAATTTCAGTAGTAAATAG
>EqcaTAS2R7_chr6:38706526-38705588
ATGACAATTGACATGAAGAGCACCTTAATGATCATAGCAGCTGGAGAGTTCTCGATGGGGATCTTAGGGAATGCATTCAT
TGGATTGGTGAACTGTATGGACTGGATCAAGAACAGAAAGATTGCCTCCATTGATATAATCCTCACGAGTTTGGCCATAT
CCAGAATTTGTCTATTGTGTATTATACTATTAGATTGTTTTATATTGGTGCTGTATCCAGATGTCTATACCAGTGGTAAA
CAAATGAGAATCATTGATTTCTTCTGGACACTAACCAACCATTTAAATGTCTGGTTTGCCACCTGCCTCAGCATTTTCTA
TTTCCTCAAGATAGCAAATTTCTTCCATCCCCTTTTCCTCTGGATGAAGTGGAGAATTAACAGTATGATTCCTAGGATTC

TGCTGGGATGTTTGGCCCTCTCTGTGTTTATTAGCCTTCCTGTCCCTGAGAATCTGAATGATGATTTCAGGAGCTGTGTC
AAGAGAAAGTGGAAAACAAACTTAACTTTGAGATGCAGAGTAAATAAAGCTCAATATGCTTTCATAAAGCTATTTCTCAA
CCTGTTAACACTATTCCCCTTTTCTGTGTCCCTGATCTCATTTTTCCTCTTGATTCTTTCCCTGTGGAGACACACCAGGC
GGATGCAACTCAATGCCACAGGGAGCAGAGACCCCAGCCTGGAAGCCCACGTGGGAGCCATGAAAGCTGTCATCTCCTTC
CTCCTCCTTTTCATTGCCTACTATCTGGCCTTTCTTGTGGCCACCTCCAGCTACTTCATGCCAGAGACTGAATTAGTTGT
TATGGTTGGTGAGGTGATAGCTTTAATCTATCCCTCAAGCCATTCATTTATCCTAATTCTGGGGAACAACAAATTAAGAC
AAGCATCTCTAAGGGTGCTATGGAAAGTAAAGTATCTTCTAAAAAGAAGAAATTTCTGA
>EqcaTAS2R8_chr6:38711035-38710115
ATGCTCAGTACAGAAGAAAACATCTTTATGGTCATAATAACTGGTGAATTCACAGTTGGAATGTTGGGGAATGGATACAT
TGGACTAGTAAACTGGATTGACTGGATTAAGAAGAAAGAGACCTCTTCAATTGACTACATCTTCACCAGTTTAGCTATCT
CCAGAATTTGTTTGATTTGTGCAATGGTGCTGAATGCCCTTATAATAGTATTCTACCCAGAGGTTCATGAAAATGATAAA
ATAAAGATAGTCAACATCTTCTGGACACTCACCAACTACTTAAGTATGTGGTTTGCCACCTGCCTCAATGTCTTCTATTT
CCTCAAGGTAGCTAACTTCTCCCATCCACTTTTTCTCCAGCTGAAGTGGAGAATTGACAGGGTGGTTCACTGGATCCTGC
TGGGGTGCTTGGCCATTTCCTTGTTGATCAGCCTTATATTTGCAATGACCCCAAAATATGAGTTGCTGAAAATTGCAAAA
CATAAAAGAAACTTCACTGAATCATTTCATGTGAGTAAAATTCAATACTTCAGCCCAGTGACAATCTTTAGCCTGTTGGC
AACTGTCCTATTTACTGTGTCATTGATTTCATTTTTCCTTTTAATTATGTCCCTATGGAAACATATTAAGCAAATGAAAC
TCAATGTTACAGGCTGCAGAGACCCCAGCACAGAGGCCCATGTGAGACCCATGAAAACTGTGACTTCATTTCTCTTTCTT
CTTTTTGTATACTATCTGGCCTCTCTTTTAATGACGTTTAGCTACCTGATGAAAGAAAGAAAGTTAGCTGTGATGTTTGA
AGAGGTTATAGCAATTTTCTATCCCTCAGGTCACTCACTTATTTTAATTATTGGAAACAACAAGCTGAGGCAGGCATTTG
TCAGAATGCTGAGATGTGGAAAAACAGCCTGCATGATGTAA
>EqcaTAS2R9_chr6:38714239-38713304
ATGCCAAATACAATGGAGACAATATACATGATCCTGATTGCTAGCGAATTGAGTATAGGAATTTGGGGAAATGGATTTAT
TGTACTGGTTAACTGCACTGGCTGTTTCAAAAGGAGCATTATCTCCTTGATTGACATCATCCTGGTCCCCTTAGCCATCT
CCAGAATCTGTTTGTTGTGTGTGATATCTTTAGATGGCTATGTTATGCTGCTCTCTCCAGATACATATGACCATGGGGAG
CTAATGAACATTTTGGATGTTTTGTGGACATTTAGCAATCATTCAAGTGTCTGGTTTACTTCTTGCCTCAGCATCTTCTA
CTTACTCAAGATAGCCAATATAGCCCACCCATTTTTCCTCTGGCTGAAGCTAAAGATTAACAGGGTCATCCTTGGGATTC
TTCTGGCATCCTTTCTCCTCTCCTTAATTATTAGTGTTGCACTGAATGAAGATTCCTGGTATAACTTCAAGGTCAATCAT
GAAGGAAATATAACTTTGAAATTCAAAGTGAGTAAAGTCTCAAATGCTTTCAAACAGATTATCCTGAACCTGGGGGCGAT
TGTTCCCTTTATACTCTGCCTGATATCATTTCTGTCGCTGCTTTTCTCCCTATTTAGACACACCAAGCAGATGAAATTTC
ATGTCACAGGGTCCAGAGACACCAGCACAGAGGCCCACATGAGGGCCCATAAAGACAGTGCTGATCTTTCTGCTCTCTTC
ATTGTGTACTATGCCGTCTTTCTTGTAATGACCTCTAGCTATCTGATTCCTCAGAGAAAATTACGGATAATGTTTGGTGG
CATAATAGCTGTCATTTTCCCATCTAGCCATTCTTTTATCCTGATAATGGGGAACAGCAAGCTGAGGGAGGCTTTTCTGA
AGGTGTTAAGGACTGTAAAGGGTTTCCACCAAAAAAGGAAATCTTTTGTTCTCTAG
>EqcaTAS2R11B_chr6:38824663-38823722
ATGTTGAATACGTTGGAGAAAATTTTCATGATTGTAACAGGTGGAGAATTTATAATAGGAATTTTAGGGAATGGATTTAT
TGGACTCACAAATTGCATTGCCTGGATTAGAAATCGGAAGTTATGCTTGGTTGACTTCATTCTTACCAGTTTGGCCTTCG
CCAGAATCAGTAAATTATGGCTAACAATTGTCAATTTGGTTTTAGTGCTGGTCTATCAGGAAATCCCTGAAACTATGAAA
ACAAACAACATCCTTACCAGCATCTGGATACTGGTCAACCACTTGACCACTTTGTTGGCTGCTTGTCTCGCTGTCTTTTA
TTTCCTGAAGATCTCCAGTTTCTCCCATCCTCTTTTCCTTTGGCTGAAACGGAGAATTAACAAGGTAATTTACATGGTTC
TGCTGTCATCTTTGCCCTTCCTGTTGATCAACTTTCCTTTGCCAGTTAATATTGATGTCGTCTGGTATCATGTCCAAAAG
AAATATGAAAGAAATATGACTGGGTTAGTCAATGTGAGTAAAAGCAAACATTTAAGAGTCATGGTAGTCTTCATTATTGG
GTCCTTCCCTCCTTTCTCTCTTTCTTTGATTTCCTTTTTCCTTTTGCTCTTTTCTTTGTGGAGACACACGAAACACAATT
TGCTCAACTTCAAGGATTCCAGAGACCCCAGTATGGAGGCCCATGTCAGAGCCATGAAAACTGTATTTCTCTTTCTTGTC
CTCTTTGCTGTGTACCAGTTATCTATTTTCATGACATTTTGGGGGTATTTTTCACTACAGAACAAGCTGGTTGTGATGTT

TGCTTATATGATAGAAATTCTCTATCCTTCTGGTCACTCATATGTTGTGATTTTTGGAAATAGTCAAATGAGGAAAGCCT
TCTTGGGGTTTCTTTGTCACTTGAAGTGTGACTTGAAAGGAAAGGCACTGTCAGCTGCATAG
>EqcaTAS2R11C_chr6:38780857-38779916
ATGCCGAATACATGGGAGAAAGTTTTCATGATTGTAACAGGTGGAGAATTTATAATAGGAATTTTAGGGAATGGATTTAT
TGGACTCACAAATTGCATTGCCTGGATTAGAAATCGGAAGTTGTGCTTGGTTGACTTCATTCTTACCAGTTTGACCTTGG
CCAGAATCAGTCAATTATGCCTAACAATTATCAATTTGTTTTTAGCACTGGTCTATCAGAAAATCCCTGAAACTATGAAA
ACAAACAATATCCTTACCATCATGGGGATACTGATCAACCACTTGACCACTTGGTTGACTGCTTGTCTCGCTGTCTTTTA
TTTCCTGAAGATCTCCAGTTTCTCCCATCCTTTTTTCCTTTGGCTGAAGTGGAGAATTAACAAGGTAGTTTACATGGTTC
TGCTGTCATCTTTGCCCTTCCTGTTGATCAACTTTCCTTTGCCACTTAATTTTGATGTCTTCTGGTATCATGTCCAAAAG
AAACATCAAAGAAATATGACGGGATTAGTCAATGTGAGTAAAAGCAAACATTTAAGTGCCAGGACCGTCTTCATTATTGG
ATCCATCCTTCCTTTCTCTCTTTCCTTGATTTCCTTTTTCCTTTTGCTCTTTTCCTTGCGGAGACACATGAAACGCAATT
TGCTCAACTTCAAGGATTCCAGAGACCCCAGCATGGAGGCCCATGTCAGAGCCATGAAAACTGTGCTTCTCTTTCTTGTG
CTCTTTGCTCTGTACCAATTATCATTTTTCATGACATTTTGGGGATATTTTTCACTACAGAACAAGCTGGTTGTGATGTT
TGCTTATATGATAGGAATTCTCTATCCTTCCGCTCACTCATATGTTGTGATTTTTGGAAATAGTCAAATGAGGAAAGCCT
TCTTGGGGCTTCTTTGTCACGTGAAGTGTGTCCTGAAAGGAAGAGCAATCTCAGCTGCATAG
>EqcaTAS2R12A_chr6:38913168-38912230
ATGGAAAGCACATTGAAGAATATATTTATGATCATTTTGGCTGGAGAATTGTTAATGGGGATTTTGGGAAATGGATTCAT
TGTGCTGGTTAACTGTATTGATTGGATCAGGAGCTGGAAGTTCTCCCTGATTGACTTTATTCTCACCTGCTTGGCTAGTT
CCAGAATATTTCTGCTGTGCATAATAATTTTAGGTATAGCTTTAGATGTAAACTTTGAGGAAATATGGTACAATAATAAT
AATCTACTGAGAAGTTTGGAAATCCTCTGGACGGGATCCAATTATTTCTGCATGATCTCTACCACCTGCCTCTGTGTCTT
CTATTTCCTCAAGATAGCCAACTTCTCTAATTCCATTTTCTTCTGGATGAAGTGGAGAATTCACAAGATTCTTCTCATTA
TTGTGTTGGGGGCAACCCTCTCTTTCTGCTTGTGCATTCTTTTTAAGGATACATTAGTTAGGAGCCTGATCAAATACCAG
GTAAACGCTGAAAGAAATTTTACATGTAACTTCATAGAGAGAAAATATGATTTATTAACTCCTCAAAAGCTCATTAACAT
AATGTTCATCATCCCCTTTTTAGTGTCTCTGGCCTCCTTAGTCCTTTTAATCCTCTCTTTATGGAGTCATGCCAGGCAGA
TGGAAGACACAGGTTCTAGGGATCCTAGCAGAGAGGCCCATGTGAGAGCTATGAAGTCTATGATTTCATTCCTACTCCTC
TTCTTCATATACTATTTGAGCCATATTATAATAAATTCAGCCAATACCACTCTAAACACTTTTGTGGCAAAGATTTTTGC
TCACGTGCTGTTATATTTGTACCCATCTGGCCATCCGTTTCTTCTGATTTTGTGGAACAGCAAATTGAAACAGGCTTCCC
TCAGTGTCCTGAAGAAGCTGAAGAGTTGCATGAACCTAAGGAAACCTACAATCCCATAA
>EqcaTAS2R12D_chr6:38840820-38839885
ATGGCAAGCACACTCAGCAATATATTTGTGATCCTTTATGCTGGAGCATTCACAATGGGGATTTTGGGAAACGGATTCAT
TGTACTGATTAACTGTATTGACTGGATCAGGAGCTGGAAGTTCTCCCTGATTGGCTTTATTCTCACCTGCTTGGCTATTT
CCAGAATATTTCTGCTGTGCATAACAATTTTAGGTATAGGCTTAGATGTAAACTTTGAGAAAATATTGTACACTAATAAT
AATCTACTGATAAATTTGAAAAACCTCTGGATAGGATCCAATTATTTCCGCATGACCTGTACCACCTGCCTCAGTGTCTT
CTATTTCCTCAAGATAGCCAACTTTTCTAATCCCGTTTTCCTCTGGATGAAATGGAGAATTCACAAGATCCTTCTCATTA
TTGTGTTGGGAGCAACCTTCTCTTTCTGCTTGTGCCTGATTTTTAAGGAGGCAGTATTTAAGAGCCTGATCAAAAACAAG
GTAAATGCTGAAAGAAATTTGACATGGAACTTCACAATGAGAAAATATTCATTAACTTCTCAAATGCCCTTTGACATAAT
GTTCATCATTCCCTTTTTAGTGTCACTGGCCTCTTTATTTCCTTTAATCCTCTCCTTGTGGCGCCATATCAAGAAGATGA
AGGGCACAGGTTCTAGGGATCCCAGGACAGAGGCCCATGTGAGGGCCATGAAGTGTATGATTTCATTCCTACTCCTCTTC
TTCTTGTACTATTTGAGCCATATCATAAAATATTCTGCCGATGCGGTTGTAGATACTTTTGTAGCAAAGATATTTGGTAA
TGTGCTAATATTTTTGGATCTGTCTGGCCATCCATTTCTTCTGATTTTGTATAACAGCAAATTGAAACAGGCTTCTCTCA
GTGTCCTGGAGAAGCTGAAGTGTTGCATGAATCTAAGGAAACCTACAATCCCATAA
>EqcaTAS2R12E_chr6:38793720-38792785
ATGGCAAGCACACTCAGCAATATATTTATGATCCTTTATGCTGGAGCATTCACAATGGGGATTTTGGGAAATGGATTCAT
TGTGCTGGTTAACTGTATTGACTGGATCAGGAGCTGGAAGTTCTCCCTGATTGACTTTATTCTCACCTGCTTGGCTATTT

CCAGAATATTTCTGCTGTGCATAATAATTTTAAGAATAGCCGTAGTTGTAAACTATGAGAAAACATTGTACACTAATAAT
AATCTACTGATAAGTTTGGAAACCCTCTGGACAGGATCCAATTATTTCTGCATGACCTGTACCACCTGCCTCAGTGTCTT
CTATTTCCTCAAGATAGCCAACTTTTCTAATCCCGTTTTCCTCTGGATGAAATGGAGAATTCACAAGATGCTTCTCATCA
TTGTACTGGGAGCAACCTTCTCTTTCTGCTTGTGCCTTCTTTTTAAGAATACACTAGTTAAGAGCCTGATCGTAAACCAG
GTAAATGCTGAAAGAAATTTGACTTGGAACTTCACAGTGAGAAAATATTTATTAACTTCTCAAATTCTCCTTGACATAAT
GTTCATCACCCCCTTTGTAGTGTCACTGGCCTCCTTACTTCCTTTAATCCTCTCCTTATGGAGCCATACTAAGCAGATGA
AAGGTACAGGTTCTAGGGATACTAGGACAGAGGCCCACGTGAGAGCCATGAAGTGTATGATTTCATTCCTACTCCTCTTC
TTTATGTACTATTTTAACCATATTATAAAATATTCAGTCTATGCTGTTGTAGATACTTTTGTAGTAAACACTTTTGCTAA
TGTGCTAGTGTTTTTTGATCCATCTGGCCATCCATTTCTTCTGATTTTGTGGAACACCAAATTGAAACAGGCTTTTCTCA
GTGTCCTGGAGAAGCTGAAGTGTTGCGTGAATCTAAGGAAACCTACAATCCCATAA
>EqcaTAS2R14_chr6:38982115-38981153
ATGGTCAGTGTCGTACAGAGCACACTTACAATCATTCTAAGTGCTGAATTCATAATTGGAAATTTAGGAAATGGATTCAT
AGCACTCGTGAACTGCATTGACTGGGTCAAGAGGAGAGAGATCTCTTCAGCTGATCAAATCCTCACTGCTTTGGCAATCT
CCAGAATTGGTCTGCTCTGGTTAGTATCTATAAACTGGTATATATCTGTGTTTTTTACAGTTTTACTTGTGCCTGGAAAA
CTGTTAAGAGTGAATAGTATTGGCTGGACAGTGACCAATCATTTCAGCAACTGGCTTGCTACAAGCCTCAGCATCTTTTA
TTTTCTCAAGATAGCCAGTTTTTCTAACTCTATTTTTCTTTACCTAAAGTGGAGAGTTAAAAAGGTGATTTCAATGATAC
TGCTGGTGACCTTGGTCCTCTTGATTTTTAATATTGCACTGATGAACATGCATATTAATGTCTGGATCAATGAACATAAA
GTAAACATGACCGGCACTTCTAGGATGAGCAACTTTGTACAACTTTCCACTCGTACTTTATTCATTAACACTCTGTTCAC
TATCATACCCTTTGCTGTGTCCCTGATAATTTTTCTTCTGCTTATCTTCTCCTTGTGGAAACACCTCAAGAAGATACAGC
ACAATGCCAAAGACTCCAGAGATGCCAGCACCGAGGCCCACATAAAAGCCATGAAAAGCATGATTGCTTTCCTCCTACTA
TTTGCCATTTACTTTCTGTCTCTTTTTGTGTCAATTTGGAGCTTTAAATTTCCAGAGAGAAAGCAGATCATTATGTTTTG
CCAGGTTATCGGAATTAGCTATCCTGCAGGTCACCCATATGTCCCGATTCTGGGATACAATAAGCTGAGACAAGCCTTTC
TTTCAGTGCTGTGCTGGCTTAGGTCGAAGATGGAGAATCTTCAGGCCCGTAGACCATTTAGAGATTCATCTTGCATATCC
TAG
>EqcaTAS2R39_chr4:96257267-96258226
ATGATGAAAACCTGCGGTTCCCCAGAAAATAAATTGTCACCTTTTTGGATCACCTTAGTTTTCACAATTATAGGCGCTGA
ATGCATCATTGGTATCTTTGCAAATGGATTCATCGTGGCTATAAATGCAGCTGAATGGATTCAGAATAAGGCAGTTTCCA
CAAGTGGCAGGATCCTGCTTTTCCTGAGCGTATCCCGAATAGCTCTCCAAAGCTTCCTGATGCTAGAACTTAACTTCAGC
TCAACATCCCCAAGTTTTTATAATGAAGATGTTGTATATGACACATTCAAAGTAAGTTTCATGTTCTTAAATTATTGTAG
CCTCTGGTTTGCTGCCTGGCTCAGTTTCTTCTACTTTGTGAAGATTGCTGATTTCTCCTACCCCGTTTTCCTCAAGCTGA
AGTGGAGAATTTCTGGATTGATGCCCTGGCTTTTATGGCTATCAATGCTTATTTCCTTGGGCAACAGTGTGGTCTTCTTC
AAAGACATCTACACCGTGTATTCTAACAATTCTTTTCCTATCCCCTCCTCCAACTCCACTGAGAAACAATACTTCACTGA
GACAAATGTGTTCAATCTGGTTCTTTTCTATTACCTGGGGATCCTCATTCCTCTGATCATGTTCATCCTTGCAGCCACCC
TGCTGATCATCTCTCTCAAGAAACACACCCTACACATGGAAAGTAATGCCACTGGCTTCAGGGACCCCAGCATGGAGGCT
CACATGGGGGCCATCAAAGCTACCAGCTACTTTCTCATTCTCTACGTTTTCAATGCAATTGCTCTGTTTCTCTATATGTC
CAACATCTTTGACATCAACAGTTCCTGGAACATTTCGTGCAAAATCATCATGGCGGCCTACCCTGCTGGTCACTCCATTC
TACTGATTCAGGACAACCCTGGGTTGAGAAGAGCCTTGAAGAGGCTTCAGGCTCGAGTTCATCTTTACCTAAAAGACTAG
>EqcaTAS2R40_chr4:96285617-96286573
ATGGCGACAGTGAACACAGATGCCATGGATAAAGACACGTCCAGGTTTAAAATCATCTTTACCTTGCTGGTCTCCGGAAT
AGAGTGCACCATTGGCATCGTTGGGAATGGCTTCATCACAGCTATCTATGGGGCGGAGTGGGCCAGAGGCAAAAGACTAC
CCATTGGTGACCGCATCCTTTTGATGCTGAGCTTTTCCAGGTTCTTGCTACAGATCTGGATGATGCTGGAGAATACTTAC
AGTCTACTATTGCGGGTCATTTATACCCAAAACGCAGTGTATAAACTTTTCAAAGTCATCATCATGTTTCTGAACTATTC
CAACCTCTGGCTTGCTGCCTGGCTCAATGTCTTCTATTGTCTTAGAATTGCAAGCTTTACTCATCCTTTGTTCTCCATGA
TGAAGAGGAAAATCATGGTGCTGATGCCTTGGCTTCTGAGGCTGTCACTCCTCATCTCTTTATGCTTCAGCTTTCCCTTC

TTTAAAGATATCTTCACTGTGTATGTGAATAGTTCCATTCCTATCCCCTCCTCCAACTCCACTGAGAAGAAGTACTTCAC
TGAGACCAATGTCTTCAATCTGATTCTTCTTTATTATCTGGGGATCCTCATTCCTCTGATCATGTTCATCCTTGCAGCCA
CCCTGCTGATCATCTCTCTCAAGAGACACACCCTACACATGGAAAGCAATGCCACTGGCTTCAGGGACCCCAGCATGGAA
GCTCACATGGGGGCCATCAAAGCTATCAGCTACTTTCTCATTCTCTACACTTTCAATGCAGTTGCTCTATTTCTTTCTAT
GTCCAACATCTTTAATGCTGATAGTTCCTGGAATATTTTGTGCAAAATCATCATGGCTGCCTACCCAGCTGGCCACTCAG
TGCTACTGATCTTGGGAAATCCTGGGCTGAGAAGGGCCTGGAAGAGGTTTCAGAACCGAGTTCATCTTTACCTATAA
>EqcaTAS2R408C_chr6:39117394-39116486
ATGATAACTTTACTACCAAGCATTTTTTCCATCCTAATAACAACAGAATTTTTTCTTGGCAATTTTGCCAATGGCTTCAT
AGCACTGGTGAACTGCATTGACCGGGTCAAGAGACAAAAGTCGTCCTCAGCTGATCAAATTCTCACAGCTCTGGCGGTCT
CCAGAATTGGTTTACTCTGGGTAATATTAATAAATTGGTATGTGACTGTGCTTCCTTCAGTTTTTTGTAGTTTAGAAGTA
AGAATTATTGTTTGTGTTGCCTGGACAGTAAGCAACCATTTTAGCATCTGGCTTGCTACTAGCCTCAGCATATTTTATCT
GCTCAAGATAGCTAATTTCTCTTGCTGTATATTTCTTTACCTAAAGTGGAGAGTTAATAGTTTACTTCTTGTAATACTGT
TGGCAACTTTGGTCTTTTTGGTTCCTCACTTTGCAGTGCTGTGCGTAGATGAGACTATGCAGACTAAAGAGTATGAAGGA
AACGTCACTCGGAAGACCAAATTGAGAGATGTTGTAGGCCTTTCAAATATGACTCTATTCACGCTAATACACTTCATACC
CTTTACTATGTCCCTGACATCTTTTCTGCTGTTAATCGTTTCCCTATGGAAACATCTCAAGAAGATGCAGCTTAATGGCA
AAGGATACCAAGATCCTAGCACCAAGGTCCACATAAGAGCCATGCAAACTGTGCTCTCTTTTCTCCTGCTATATTCCGGT
TACTTCTTGGCTCTAGTTATCTCAGTTTGGAGTTCTAATCGACTGAAGAATGAACTAGTTCTCATGATTTGCCAGGCTCT
TGGAATGCTGTATCCTTCAAGCCACTCATTCATCCTGATTTGGGGAAACCAGAAGCTAAGAAAGGCCTTGAAAAATGTAA
GACAGATTATAAGATTACCACACGATTAA
>EqcaTAS2R408D_chr6:39112295-39111387
ATGGTAGCTTTACTACCAAACATTTTTTCTGTTCTAATAATGACAGAATTTATTCTGGGAAATTTTGCCAATGGCTTCAT
AGCACTGGTAAACTGCATTGACTGGGTCAAGAGACAAAGGATGTCCTCAGCTGATCAAATTCTCACAGCTCTGGCGATCT
CCAGAATTGGTTTGCTCTGGGTAATATTAATAAATTGGTATACAGCTGTGCTCCGTACAGGTTTATATAGTTTAGAAGTA
AGAACTGTTGTTCATGTTGCCTGGGCAGTAAGCAACCATTTTAACATCTGGTTTGCTACTAGCCTCAGCATATTTTATTT
GGTCAAGATAGCTAATTTCTCTAGCTTTATATTTCTTTACCTAAAGCAGAGAGTTAAAAGTATACTTCTCGTAATAATCT
TGGGGACTCCAGTCTTTTTGGTTCCTCATATTACAGTGCTATGCTTAGATGAGAATATGCGGACTAATGAATATGAAGGA
AACATCACTCAGAAGACCAAATTGAAGGGCGTTTTTCACCTTTCATTTATGACTCTATTCACACTAGTAAACTTCATACC
ATTTTCTATTTCCCTGACATCTTGTCTGCTGTTAATCATTTCCCTGTGGAAACATCTCAAGAAAATTCAGCTGAATCTAA
AAGGATCCCAAGATCTCAGCACCAGGGTCCACATAAGAGCCATGCAAACTGTGGTTTCCTTTCTCCTGCTACATGTCAGT
TACTTCCTGGCTCTAATTATCTCAGTTTGGAATTCTGAAAGGCTGAAGAATCAACTATTTGTCATGCTTTGTGAGGTTCT
TGCAATGTTGTATCCTCTAAGCCACTCATTTATCCTGATTTGGAAAAACAAGAAGCTAAGATCATACTTGAAAAACATAA
GAAAGATTATAAGGTTACCATACCATTAA
>EqcaTAS2R408G_chr6:39018737-39017829
ATGGTAAATTTACTACCAAGCATTTTTTCTGTCTTAATAACGACGGAATTTATTCTGGGAAATTTTGCCAATGGCATCAT
AGCACTGGTGAATTGCATTGACTGGGTCAAGAGACATAAGATGTCCTCAGCTGATCAAATTCTCACTGCTCTGGCGGTCT
CCAGAATTGTTTTGCTCTGGGTACTATTAATGAATTGGTATACAGTTGTGCTCCATCCGGGTTTATATAGTTTGGAAGTA
AGAATTTTTGTTCGTATTGCCTTGACAGTAAGCAACCATTTTAACATCTGGTTTGCTGCTAGCCTCAGCATATTTTATTT
GCTCAAGGTAGCTAATTTCTCTAGCTTTATATTTCTTTACCTAAAGCGGAGAGTTAAAAGTGTACTTCTCATAATACTGT
TGGGGACTCTGGTCTTTTTGGTTCCTCATCTTGCAATTCTATGCATATATGAGAATATTCAGACTAATGAGTATGAAAGA
AACATCACTCAGAAGACCAAATTGAGGGACATTTTTCACTTCTCATATATGAATCTATTCATGCTAGTAAACTTCACACC
ATTTTCTATGTCGCTGACATCTTTTCTGCTGTTAATCATTTCCCTGTGGAATCATCTCAAGAAGATGCAGCTCAGTGGCA
AAGGATCCCAAGATATCAGCACCAAGGTCCACATAAGAGCCATGCAAACTGTGGTCTCCTTTCTCTTCTTGTATGTCAGT
TACTTCCTAGCTCTGGTTACTTTAGTTGAGAGTTATAATAGGCTGCATAATACACTGCTTGTCATGCTTTCTGAGGCTCT
TGCAATGCTCTATCCTTTAAGCCACTCATTTATCCTGATTTGGGGAAACAAGAAGCTAAGACAGGCCTTGAAAACCATAA

GAAAGATGATAAGATTCCCATACCATTAG
>EqcaTAS2R62A_chr4:96520654-96521559
ATGCCCTTCTCACCCATGTTGATCTTCGTGGTCATCTTTTTCCTGGAGACCTTGGCTGCGATGTTGCAGAATGGCTTCAT
GGTTGCTGTGCTGGGCAGGGAGTGGGTGAGATGCCTCACACTGCCTGCATGTGACATGATTGTGGCCTGTCTAGCTGCCT
CTAGGTTCTGCCTGCATGGGCTGGCCCTCCTAAACAACCTCATTGACTCCTTTAACTTTTGTTCCAAAGTTTACTATTTC
AACATCCTCTGGAACTTTATCAACATTCTCACTTACTGGCTTACTGCCTGGCTTGCTGTCTTCTACTGTGTGAAGATCTC
ATCCTTCTCTCATCCCATCTTCTTCTGGCTGAAGTGGAGGATTTCTCGGTCAGTGCCCAGGCTGCTGCTGGGCTCCCTGA
TCATCTCTGCTGTGACAGTCATTCCAGCAGCAGCTAGCAATGTAATTCTTATACAGATGATTGCCTCGCAGAGTTCCCAT
GGAAACCACACTCTGGCTGATAGAGCACAGACCTTCCATAGGTACTTTTCTCTGTCTAATTTAGTGCTTGTATTGTTGAT
TCCCTTCCTGCTGTTCCTGGTATCCACTCTTTTGCTCATATTCTCACTGCACCAGCACTTGGGACAGATGAGGGCCCGCA
GACCCGGCTCACGTGATCCCAGCACCCAGGCTCACATCATGGCCTTGAAGTCACTTACCTTCTTTCTTGTGTTCTACGCA
TCGTATTTCCTGTCCCTGATTATTGCGTTTATGAAAATCACAGCCCTGCGGCGTCAGTGGCACTGGGTCTGGGAAGTGGT
GACCTATGCAGGCATCTGTCTGCACTCCAGCATCCTGGTGCTAAGCAGCCCTAAGCTGAGAAATACCCTGAAGACAAAGC
TTTGGAAAGCCCTGGACAAAAGGTGA
>EqcaTAS2R62B_chr4:96511767-96512723
ATGCCCTCTTCACTCATGTTGATCTTCATGGCCACTTTTTCCCTGGAGACGTTGATTGCAATGTTGCAGAATGGCTTCAT
TGTTGCTGTGCTGGGCAGGGAGTGGGTACAAGGCTGCACACCCTCCTCGGGGGATGTGATTGTGGCCTGCCTGGCTGCGT
CCCGGTTCTGCCTGCATGGGCTGGCCCTCCTGAACAGCTTCCTGGGCTTCTTTAAGTTTTCTTCCAAAATTTACTATTTC
AGCATCCCCTGGGACTTTATCAACACTCTCAATTTCTGGCTGACTGCCTGGCTTGCTGTCTTCTACTGTGTGAAGATCTC
AACCTTCTCTCATCCCACCTTCCTCTGGCTGAAGTGGAGGATTTCTCGGTCAGTGCCCAGGCTTCTGCTGGGCTCCCTGA
TCATATCTGGTGTGACAGTCATCTCATCAGCTACTGGGAATAGCATCGCTGTGCTGAGGAGTACCTCCCAGAGTTCCCCT
GGAAACCACACTTTGGCTGATAGAATAAGCCCCTTCTTTCGGCACTTTTTTCTGAGTCAAGAGCTACTTGTGTTGTTGCT
TCCTTTTCTCCTGTTCCTGGTGTCCACCCTCTTGCTCATGTTCTCACTGCACCAGCACCTGCAGCAGATGAGGGCCCACA
GACCCAGCCCACATGATCCCAGCACCCAGGTTCACATCACGGCCCTGAAGTCACTTTCCTTCTTCTTTGTGTTCTACACA
TCATATTTCCTGTCCCTGATTATTGTTTCTATGCAAATCACAGCCCTGCAGCATCAGTGGCACTGGGCCTGGGAAGTGGT
GACCTATGCAGGCATTTGTCTGCATTCCAGCATCCTGGTGCTAAGCAGCCCCAAGCTGAGAAAGGCCCTGAAGACAATCT
TTGGAAAGCCCTTGACAAAAGATGCTTCATCTCAAGTTATCAGTATCAATAACCAGTATCAATGGACAAGCCCATGA
>EqcaTAS2R62D_chr4:96488512-96489468
ATGCCTTCCTCACTCACATTGATTTTCATGGTCATCTTTCTTCTAGAGTCCTTGGCTGCAATGTCGCAGAATGGCTTCAT
TGTTGTTGTGCTAGGCAGGGAGTGGGTGCGATGCCGCACACTGCCCTCAGGTGACATGATTGTGTCCTGCCTGGCTGCCT
CCCAGTTCTGCCTGCATGGGATGGCTCTCCTAAGCAACTTTATGTCGTTATTTAATTTTTGTTCCCAAGTTTCCTATTTG
GGCGCTCTTTGGGAGTTTATCAACAGTCTCACTTTCTGGCTTACTGCCTGGCTTGCTGCCTTCTACTGTGTGAAGATCTC
ATCCTTCTCTCATCCCATCTTCTTCTGGCTGAAGTGGAGACTTTCTCGGTCAGTGCCCAGGCTGCTGCTGGGCTCCCTGA
TCATATCTGTTGTGACAGGCATCTCATCAGTCAGTGGGAATATAATTCTTATGCAGATGATTGCCTCCCTGAGGTCCCAT
GGAAACCACACTCTGGCTGATAGAATAAGGACCTTCTCTTGGCACTTTTTTCTACCTCAAGATGTGCTTGCAATGTCAAT
TCCCTTCCTCCTGTTCCTGGTATCCACTCTCTTGCTCATGTTCTCACTGCGCCGGCACTTGCGGCAGATGAGGGCCCATA
GACCCGGCCCACATGATCCCAGCACCCAGGCTCACACCATGGCCCTTAAGTCACTTACCTTCTTCCTCGTGTTCTACACA
TCATATTTCCTGTCCCTGATTATTACTGTTATGAAAATCACAACTCTGCAGGATCAGTGGCACTGGGCCTGGGAAGTGGT
GACCTATGCAGGCATCTGTTTGCACTCCAGCATTCTTGTGCTAAGCAGCCCCAAGCTGAGAAAGGCCCTGAAGATGAGAC
TTTGGAAAGCCCCGGAGAAAAGGTGGTTCATCGCAAGTATCAATATCAATAATCAGTATCAACAGACAAGCCCTTAA
>EqcaTAS2R5P_chr4:94578995-94579889
ATGCTGATTGCTGCCCTAAGACTGCTGATGCTGGTGGCAGTGGCTGAATTTCTCATTGGCCTGGTTGGAAATGGAATTCT
TGTGGTATGGAGTTTTGGAGAATGGGTCAGAAAATCCAAGGGGTCCTCATACAACCTCATTGTGCTGGGCCTGGCTGTTT
GCCGATTTCTCCTGCAGTGGTTGATTATGATGGACTTAATCCTGTTTCCGCTTTTCCAGAGCAGCTGTTGGCATCGCTAT

CTCAGTGTCTTCTGGGTTCTGGTAAGCCAGGACAGCCTGTGGTTTGCCACTTTCCTCAGATTCTTCTACTGCAGGAAGAT
CACGACCTTTGAACACCCCATTTACTTGTGGCTGAAGCAGAGGGCCTATTGCCTGAGTCTCTGGTCTGGGGTACCTCATG
ATCAGTTTGTGACTTGTGGTCCACATTGGCTTAAAGCCTTGCAATCCTTCCCATGGAAACAGCAGCATTCTATACCCCTT
TTCAAACTGGCACTATCTGTGTATTTTACATCTCAGTGCAGGAAGTGTGGTGCCTTTCATGGTGTTTCTGGTTTCTTCTG
GGATGCTGATCGTCTCTTTGTATAGACACCACAGGAAGATGAAGGCCCATACAGCTGGTAGGAGGGATGCTCGGGCTCAG
GCTCACATCACTGTCCTGAAGTCCTTGGGTTGCTTCCTTGTACTTTACGTGGTTTATGTTCTGGCCAGCCCCTTCTCCAT
CACCTCCAAGTATTCTCCTGCTAATCTCACTACTGTCTTCATCTCTGAGACACTCATGGCTGCCTATCCTTCTCTTCATT
CTGTCATATTGATCATGGAGAATCCCAGGGTGAAGCAGATTTGTCAGAGAATTTTGTGGAAGATAATGTGTGCTTGGAGA
TCTTGGGGCCTGTGA
>EqcaTAS2R11AP_chr6:38904990-38904079
ATGTTGAATACATTGGAGAAAGTTTTCATGCTGTAGTAGGTGAGGAATTTATAACAGGAATTTTAGGGAATGGGTTTATT
GCACTCACAAATTGCATTGCCTGCACTAGAAATCAGAAGTTATGCTTGGTTAACTTCATTCTTACTAGTTTTTCCTTTGC
CATAATTAGTCAATGATGGCTAACAATTATGTGTCTTAATAAATCTCTGATACTATGAAAAGAAACCATATCCATACTGG
TATCTGGATACTGGACAATCACTTGAGCACTTGGTTTCCTACTTGTCTCACTGTCTTTTATTTCCTGAAGATCGCCAGTT
TCTCGTTCCCTTTTTCCTTTGACTAAAATGGAGAATTAATGAGATAATTTTCATACTTCTGCTGTTATCTGTGCCCTTCC
TGTTCACCAACTTTCCTTTGCCATATAGTTTGATGTCTTCCAGTATCGTGCCCCCCAAAAAATATGAAAGTAATATGAC
TGGGTTATTTAATGTGAGAAAAAATAAATATTTAAAAGCCATGATACTCTTCATTACTGGGTCTCTCCCTCCCTTCTCTC
CTTCCTTGATTTCCTGTTTCCTTTTCTTTGTGGAGACACACGAGACACAATTTGCTCAGCATCCAGGATTCCAGAGACTC
CAGTATGGATGCCTGTTTCAGAGCCATGAAAACTCTTTTTTGCTTTTGCGCTTTTTGCTCCGTACCAGCTTTCATTTTTC
CTGACATTGTTGAGATATTTTTCACTACAGAACAAGCTGGCTGTGATGTTTGCTTATATGATACAAATTCTCTATCCTTC
AGGTCACTCATATGTTGTGATTTTTGAAAGCAGCCTAATGAGGAAAGCCTTCTTGGGGATTCTCTGGCACCTGAAGTGTG
GCCTGAAAGGAAAGGTAGTCTTGGCTGCACAG
>EqcaTAS2R11DP_chr6:38744162-38743235
GTGTTGAAAACATTGGAGAAAGTTTTCATGATTGTAGCAGGTGGGGAATTTATAGCAGGAATTTTAGGGAATGGATTTAT
TGGACTCACAAATTGCATTGCCTGGATTAGAAATCGGAAGTTATGTTTGGTTGGCTTCATTCTTACCAGTTCGGCCTTCA
CCAGAATCAGTCAATTACGGCTAACAATTGTCAATTTGTTTTCAGTGGTGGTCTATCAGGAAATCCCTGATACTAAGAAA
AGAAATCATATACATACTGGTATCTGGATACTGGCCAACCACTTGAGCACTTGGTTTGCTACTTGTCTCACTGTCTTTAA
TTTTCTGAAGATCAACAATTTCTCCTATCCCCTTTTCCTTTGGCTGAAATGGAGAATTAATCAGGTAGTTTTCATGCTTC
TGCTGTTACCTGTGCCCTTCCTGTTCATCAACTTTCCTTTCCCATACAGTTTTGATGTTTTCTGGTGTCATGTCCAAAAA
AATATAAAAGAGATATGACTGGGTTATTCAATGTGAGTAAAAATAAACATGTAAGTGCCATGACAGTCTTCATTACTGGG
TCCCTCCCTCCTTTCTCTCTTTCCTTGATTTCCTTTCTCCTGTTGCTCCTTTTCTTTGTGGAGACACATGAAACACAATT
TGCTCAAAGTCAGGGTTTCCAAGGACCCCAGTATGGAGGCCCATGTCAGAGCCATGTTTTCTTTCTTGAGCTCTTTTTTC
TGTACCAGTTTGCCTTTTCCTGATATTTTTGGGTTATTTTTCACTACAGAACAAGCTGGTTGTGATGTTTGGTTACATGT
TAAGAATTCTATATCTTTGGGGTCACTCAAATGTCATGATTTTCCTCAGCCAAGTGAGGAAAGCCTTCTTGGGGATTCTC
TGGCACCTCAAGTGTAGCCTCAAAGGAAAGGCACTCTCAGCTGCATAG
>EqcaTAS2R12BP_chr6:38878229-38877274
ATGGCAAGCACACTGAAGAATATATTTATGATAATTTCTGTTGGAGCATTCACAATGGGGATTTTGGGAAACGGATTCAT
TGTACTGATTAACTGTATTGACTGGATCAGGAGCTGGAAGTTCTCCCTGATTGACTTTATTCTCACCTGCTTGGCTATTT
CCAGAATATTTCTGCTGTGCATAATAATTTTAGGTATAGGCTTACATTTCATCAATGAGGAAATATGGTACAATGATAAT
AATCTACTGAGAAGTTTGAGAATTCTCTGGACAGGATCCGATTATTTCTGCATGACCTGTACCACCTGCCTCTGTGTCTT
TTATTTCCTCAAGATAGCCAACTTTTCTAATCCCATTTTCCTCTGGATGAAGTGGAGAATTCACAAGGTGCTTCTCATTA
TTGTGCTCGGGGCATCCGTCTCTTTCTGCTTGTGTATTTTTTTAAGGATACAGAAACTAGGAGCCTGATCAAAAACCAGG
TAAACACGGAACAAAATTTGACATGGAATATTGCAGTGAGAAAATATAATTTATCAACTTCTCATATGCTTATTAACATA
ATGTTCATCATCCCCTTTTTAGTGTCACTGGCCTCCTTACTCCTTTTAATTCTCTCTTTATGGAGCCACACCAGGCAGAT

GAAGGGCACAGGTTCTAGGGATCCTAGCACAGAGGCCCACGTGAGGGCCACGAAGTCTATGATTTCATTCCTACTCTTCT
TCTTCTTGTACTATTTGAGTAATATTATGCTAAAGTCAATCTATGCCAATCTAGACAGTTTTACAGTAAAGATTTTTGCT
AATGTGCTAGTGTTTTTCTTTCTATCTGGCCATCCATTTCTTCTGATTTTGTGGAATAGCAAATTGAAAAAGGCTTCTCT
CAGTGTCCTGAGGAAGCTGAAGTGTTGCATGAATCTAAGGAAACCTACAATCCCGTAAACATGCCTGAAAGGATTG
>EqcaTAS2R12CP_chr6:38845972-38845033
ATGGCAAGCACATTGAAGAATATATTTGTGATCCTTTACACTGGAGCATTCACAATGGGGATTTTGGGAAATGGATTCAT
TGTACTGATTAACTGTATTGACTGGATCAGGAGCTGGAAGTTCTCCCTGATTGACTTTATTCTCACCTGCTTGGCTATTT
CCAGAATATTTCTGCTGTGCATAATAATTTTAGGTATAGGCTTACATTTCATCAATGAGGAAATATGGTACAATGATAAT
AATCTACTGAGAAGTTTGAGAATGCTCTGGATAGGATCCAATTATTTCTGCATGACCTGTACCACCTGCCTCAGTGTCTT
CTATTTTCTCAAGATAGCCAACTTTTCTAATTCCATTTTCCTCTGGATGAAGTGGAGGATTCACAAGGTGCTTCTCATTA
TTGTACTAGGGGCAGCCATTTCTTTCTGCTTGTGCCTTTTTTTTTAAGGAGACAGTATTTAAGAGCCTGATCAAAAACCA
GGCAAACACTGAAAGAAATTTGACATATAACTTATTAGTGAGAAAATATCATTTATTAACTCCTCATATGCTCATTAACG
TAATGTTCATCATCCCCTTTGTGGTGTCGCTGGCCTCCTTACTCCTTTTAGCTCTCTCCTTATGGAGCCATACCAGGCAG
ATGAAGGGCACCGGTTCTAGGGATCTCAGCGCAGAGGCCCATGTGAGGGCCATGAAGTGTACGATTTCATTCCTACTCCT
CTTCTTCTTGTACTATTTGAGTAACATTATGCTAAATTCAGCCTATGCTATTCTAGACAGTTTTACGGCAAAGATTTTCG
CTAATGTGCTAGTGTTTTTCTATCCATCTGGCCATCCATTTCTTCTGATTTTGTACAACAGCAAATTGAAACAGGCTTCT
CTCAGTGTCCTGGAGAAGCTGAAGAGTTGCATGAATCTAAGGAAACCTACATTCCCATAA
>EqcaTAS2R12FP_chr6:38759055-38758104
ATGGCAAGCACATTGAAGAATATATTTAAGATCCTTTATGCTGCAGCATTCACAATGGGGTTTTGGGAAACGGATTCATT
GTACTGATTAACTGTATTGACTGGATCAGGAGCTGGAAGTTCTCCCTGATTGACTTTATTCTCACCTGCTTGGCTATTTC
CAGAATATTTCTGCTGTGCATAACAATTTTAGGTATCGGCTTAGATGTAAACTCTGAGAAAATATTGTACACTAATAATA
ATTGACTGATAAGTTTGGAAACCCTCCGGGTAGGATCCAATTATTTCTGCATGACCTGTACCACCTGCCTCAGTTTCTTC
TGTTTCCTCAAGATAGCCAACTTTTCTAATCCCATTTTCCTCTGGATGACATGGAGAATTCATAAGGTGATTCTCAGTGC
TGTGCTGGGGGAAGCCCACTCTTTCTGCTTGTGCCTTTTTTTAAGGATACAGTACTTAAGAGCCTGATCATAAACCAGGT
AAATACTGAAAGAAATTTGATATGGAACTTCACAGTGAGAAAATATTCATTAACTTCTCAAATGCACTTTGACATAACGT
TCATCACCCCCTTTGTAGTGTCACTGGCCTCCTTACTTCCTTTAATCCTCTCCTTATGGAGCCATACTAAGCGGATGAAG
AGTACAGGTTCTAGGGATCCCAGGACAGAGGCCCATGTGAGGGCCATGAAGTGTATGATTTCATTCCTACTCTTCTTCTT
GTACTATTTGAACCATCTGATAATAAATCCAGCCTGTGCCCTTCTAGACACTTTTGTGGCAAAGACTTATGCTAATGTGC
TAGTATTTTTTGATCCATCTGGCCATCCATTTCTTCTGATTTTGTGGAACAGCAAGTTGAAACAGGCTTCTCTCAGTGTC
CTGGAGAAACTAAAGTGTTGCATGAATCTAAGGAAACCTACATTCCTATCAAGATGCCTGAAAACACTGTGA
>EqcaTAS2R13P_chr6:38965588-38965194
ATGGTAAACGCCTTGCATAGCTTCATCTTCATTTTAATAAATATAGAAATAATAACTGGTAGTTTGGCGAATGGATTCAT
AGCGCTGGTGAGCTGCATTGACTGACTCAAGAAACAAAAGATCTCCTCAGCAGATCGAATCATCACTGGTTTGGCGATCT
CCAGAATTTGTCTGATTTTGGTAATAACGGTGACCTGGTTTACAAAGGAGTTTTATCCATCTTCATATACAAATAGAAAG
AAAGTTACGCTTATTAGTATTGCTGGGACCATGGGCAATCATTTTAGCTTCTGGCTTGCCACAGGCCTCAGCTTCTTTTG
TTTTCTCAAGATAGCCAATTCTTCAAATTCTGTTTTTCTTTACCTAAAGCATAGAGTATATCCTTAAATTTTTTT
>EqcaTAS2R14P_chr6:38972880-38971926
ATGATAAGTTCACTACGGAGCATTTTTTCCAACTAGCAACTGAAGAATTTGTTCTAGGAAATTTGGCCAATGTCTTCATA
GCATGGGTGCATAGAACTGCATTGACTGAGTGAAGAGACAAAAGATGTCCTCAGCTGATTAAATTCTAACTTCTCTGACA
GTCTCCAGGATTGGTCTACTTTGGGTAATATTAATAAATTGGTATTCAATTGTTTAACCCAGCTTTACATAGTTTAGAAG
CAAGACTATTTATATTGCCTGGTCAATAACCAACCATTTTGGCATCTGGCTTGCCACTAGCCTCAGTATATTTTATTTGT
TCAAGATAGCAAATATCTCCAGCTTTATTTTTCTTCACTTAAAGTGGAAAGTTAAAAGAGTGGTTCCCGTGATACTGTTG
AGGACTTGGATCCTTTGGTTTTTCATCTAGTAGTGGTAAGCATAGATGACAGTATGTGCATGAATGACTACAAAGGATGC
ATCACTCGGAAGACCAAATTGAGGGACATTTTACGCCTTTCCAATTTAAGTATATTCACCCTAGCAAACTTCATACCCTT

TATATGTCACTGACATCTTTTCTGCTGTTAATATTTTATCTATGGAAACATCTTAAGAAGATGCAGCTCAATGGCAACGA
TCCCAACATCCCAGTACCAAGGTCCACAAAAGAGCCATGCAAACTGCAGTCTCCTTTCTCTTGCTATATGCTGGTTACTT
CCTGGCTCTAGTTATCTCAGTTTGGAGTTCTAATAGGCTGCATAACAGACCACTCATTTATCTTGATTTCAGGAAAGGAG
AAGATAAGACAGGCCTTTCTGTCATTTCTGTGGCAGCTGAGGTGCTGGCTGAAAAAAAGAGAAGTAAGTGGGCCAGTATG
TGTCTTCTAGCAAGAAACAAACTATCAGTCTTTATAAGCTTTATATATATGTGACAATAAATAATAATGACTTGA
>EqcaTAS2R16P_chr4:78959205-78958301
ATGATTCCCATCCAACTCACTGTCTTCTTCATGGTCATCTATGTGGTCGAGTCCTTGACAATAATTATGCAGAGTGGCTT
AATTGTTGCAGTGCTGGGCAGAGAGTGGGTGCAGGTAAAGAGGCTGTCACCTGTGGACGTGATTCTCACCAGCCTGGGCA
TCTGCCGCTTCTGTCTCAGTGGGCATCGATGCTGTATAATTTTTGCTCCTATTTCAACCCTAACTATGTATTTTGGTACT
ACTCGATCACCTGGGACTTTTTAAATACTCTTACATTCTGGTTAACCAGCTTACTTGCTGTCATCTACTGCGTCAAAGTC
TCTTCCTTCACCCACCCCGCCTTCCTCTGGTTGAGGTGGAGAATTTTGAGGTTGGTTCCCTGGCTGTTACTGGCTTCTCT
GCTGATTTCTTGTGTGACTCTTATCTCTTCAGCTATTAGGAATCACATGAAGATACAGATAATCTCCATGGAGCATTTCC
CTAGAAACAACACTATGGTTGAAAGACTTAAGATGCTTCTGAAGAAATTTACCTTATCTCAGCCAGTGGTTGTGTTGATT
ATTCCTTTCCTCCTGTTCCTGGCCTCCACCATCTTGCTCATGACCTCATTGTCCCAACACTTGGAGCAGATGCAACATCA
CAACACTGACCACAGCAACTCCAGCGTGAACGCTCACTCCACTGCCCTGAGGTCTCTTGCTATCTTCCTTCTCTTCTTCA
CCTCTTATTTCTGACCATACTCATCTCCATTCTGAGCATCCTATATGATAAGAGATCCTGGTTCTGGGTCTGGGAAGCT
GTTATCTATGCTATAGTCTCTGTTCAATCCACTTCACTAATGCTGAGCAGCCCTACATTGAAAAAGGTTTTAAAGGTAAG
GTGCTGGGGCCTGGAGGCTGCCTGA
>EqcaTAS2R18AP_chr6:39168572-39167648
ATGTCAGTTGGAATTAAGGTCTCCTTTCTGGTCATGGCAACAGGAGAACTCATCTTACGAATGCTGGGAAATGGGTTCAT
TGGACTGGTAAACTGCATCGAATGGGTCAAGAATGGGAAAATCTCATCAGCTGATTTCATCCTTACCAGCTTGGTATGGC
CAGAATCATTCAACTGTGGATAACACTATTTGGTTCATTTAATGTAGGGCTATTTCCACATCTGAATGCCACTAGTAAAT
TAGCAAAAGCGATTACTATTCTTTGGGCACTAACTAATCACTTGCTTGCCACCTGCCTAAGCATTTTCTGCTTCCTTAAG
ATTGCCGGTTTCTCCCACTTCTTGTTCATCTGGCTGAAGTGGAGAGTGAACAGAGTGGGTCTTGTACTTTTCCTGAGGTC
TTTCTTCTTATCTCCTGCTAGTCTCTTAATGCAGGATGCTCTTATTGAGTTGTGGATGAATACGTATGGAGTACATGAAA
GAAACATGACTTTGCATTTACACGTAAATAAAATGTTCTATCTTAAAAGCCTTCTTCTTAGTTGACCTATGTTATCCCCT
TTCTTCTGTCCCTGACCTTTTTGCTCCTTTTATTTCTGTCTTTGGTGATACACACCAGGAATTTGTAGCTCAACCTGAAG
GGCATGAGGGACTCCAGCACAAAGGCTCTTAAAGGGTCATGAAGATGGTGACAACCTTCTTCCTCCTCTTCATCATTTAC
TTTATTTCCACTCTAATAGCAAGTTAGATCTTCCTTAAGGTACAAAGGTTTCAGGTTATGATGTTGTTGTGGTGTTTTCC
ACCATCTTTCCCTCAGGCCACTCATTTATTATAATTTAGGAAACAGCAAGCTAAGATAGATCACTAGAGATTACTGTAGC
ATCTTAAATTCTCTCTTGAGAAAAGCAAAACTTTTAGCTTCATAG
>EqcaTAS2R18BP_chr6:39141363-39140424
ATGTCAGTTGGGATGAAGATCTACTTTCTGATCATGGTAACAGGAGAACTCATTTCAGGAAAGCTTGGAAATCAGTTCAT
TGGACTAGTAATCTGCATTGAATGAGTCAAGAATGGGAAGATCTCAACAAGTGATTTCATCCTTACCAGATGATTTCATC
CTTACCAGGGTGGCCATGGGCAGAATCATTCAACTGTGGGTAACACTATTTGATTCATTTATAATGTGGCTATCTCCACA
TCTGTATGCCACCAGTAAACCAGCAAAAGTGGTTACTATTCTTTGGGCACTAATCGATTACTTAACTACCTGGTTTGCCA
TCTGCCTAAGCGTTTTCTGCTTCCTTGAGATAGCCAATTTCTCCCACTTCTTTTTCATCTGGCTGAAGTGGAGAGTGAAC
AGAGTGGTTCTTGTGCTTTTCCTGGTGTCTTTGTCTGTTAACCTCTTAATGTAGAATGCTCTTAAACAGTTGCGAATGAA
TACCTATAGAGTACATGAAAGAAGCATGACTTTGCATTTAGATGTAAATAAAATTTTCTATCTTAAAAGCCGTCTTCTTC
TTAGCTTGACCTATGTTATCACCTTTCTTCTGTCCCTGACCTCTTTGCTCCTTTTGTTTCTGTACTTAACGAAACACAGC
AAGAATTTGCAGCTCAGCCTGTAGAAAGGAGGGACTCCAGTACAGAGGCCCAGAAAAGACCATGGAAGTGAGGACAACCT
TCTTCCTCTTCTTCATCATTTACTTCATTTCCACTCTAATAGCAATTTGGATTTTCCTTAAGGTCCAGAGGTATCAGTTT
GTCATCGTGATTTCATTCATCTTTCCCTCAGTCCACTCATTAATTATAATTTTGGGAAACAGCTAGATAAGACAGGCCAC
CTGAGACTACAGTAGCATTTTAAATTCTCTCTGAGAAAAGCCAAACTTTTGTCTTCACAG

>EqcaTAS2R18CP_chr6:39108627-39107694
ATATGAGTTGGAATGAAGGTCTCCTTTCTTGTCATGGCAACAGGAGAACTCATCTTAGGAATACTGGGAAATGGGTTCAT
TGGACTGGTAAACTGCATCGAATGGGTCAAGAATGGGAAGGTCTCATCAGCTGATTTCATCCTTACCAGCTTGGTATGGC
CAGAATCACTCAACCGTGGGTGACACTATCTGATTCATTTATAATGGCACTGTCTTCACATCTGTATGCCACCAGGAAAC
CAATAAAAGTGGTTACTATTCTTTGGGCACTAACTGATCACTGAACTACCTGGTTTGCCACCTGCCTAAGCATCTTCTGC
TTCCTTAAGATGGCCAGTTTCGCCCACTTGTTTTTCATCTAGCTGTAGTGGAGAGTGAACAAAGTGGTTCTTATGCTTTT
CCTGGGAACTTTCTTCTTGTTGTCTGTTAACCTCTTAATGCAGGATGCTCTTAGGAGTTGTGGATGAATATCTGTAGAGT
ACACGAAAGAAACATGACTTTGCATTTAGATGTAAATAAAATGCTCTATCTTAAAAGCTTTCTTCTTAGTTTGACCTATG
TTATCCCCTTTCTTCTGTCCCTGACTTCTTTGCTCCTTTTGCTCCTGTCCTTGATGAGACACACCAAGAATTTGCAGGTC
AACTTGATGGGCATGAGGGACTCCAGCACAGAGGCCATAAAAGGGCCATAAAAATGGTGAAAACCTTATTCCCCCTCTTG
ATAATTTGCTTTATTTCCACTCTAATAGCAAGTTGGATCTTCCTTAAGGTACAGAGGTATCATGTTATGATGTTTGTCAT
GGTGATTTCAACCATCTTTCCCTCAGGCCACTCATTAATTATAATTTTGGGAAACAGTGAGCTGAGACAGATCATCTGAG
ACTACAGTAGCATCTTAAATTCTCTATGGGAAAAGCCAAACTTTTTCTCCATAG
>EqcaTAS2R18DP_chr6:39056374-39055430
ATGTCAGTTGGAATGAAGGTCTACTTTCTGATGTGGCAGCAGGAGAACTCATTTTAGGAATGCTGGGAAATGGGTTCATT
GGAGTGATAAACACTATCAGATGAGTCAAGAATGGGAAAGTCTCATCAGCTGACTTCAACCTTACTAGCTTGGCTCTGCC
TAGAATCATTCAACTGTGGTTAACACTATTTGATTCAGTTATAATGGGGCTCTCTCCACATCTGTATGCCACCAATAAAC
TAGCAAAAGTGGTTAGTATTCTTTAGGCACTAACTCATCACTTAACTACCTGGTTTACCACCTGCCTAAGCATTTTCTGC
TTCCTTAAGATAACCAGTTTCTCCCACTTCTTTTTCATCCTGCTGAAGTGCAGAGTGAACTGAGTGGTTTTTGTGCTTTT
CCTGGGATCTTTCTTCTTGTTGTCTGTTACCTCTTAATGCAGGATCAGGAAGCTCTTAATGAGTGGTGGATGAACACCTC
TAGAGTATATGAAAGAAACATGACTTTGTATTTAGATGTAAATAAAATTTTCTATCTTACAAACCTTCTTCTTCTTAATT
TGACCTATGTTATCACCTTTCTTCTGTCCCTGACCTCTTTGTTGGTTTTATTTCTGTCCTTGGTGAGGCCCACCAAGAAT
TTATAGCTCAGCCTGATGGGCATGAGGGACTCCAGCACAGAGGCCCATAAAAGACCATGAAAATGGTGACAACCTTCTTC
CTCCTGTTCATCATTTACTTTATTTCCACTCTAATAGCAAGCTGGATCTTCCTTAAGGGACATAAGTATTAGGTTATGAT
GTTTGTCATGGTGATTTCAACCATCTTTCTCTCGGGCCACTCATTTATTGTAATTTTGGGAAATAGCAAGGTAAGACAGA
TAACGTGAGACTACTGTGGTGTCTAAAATTCTCTCTCGAGATATGCAAAACTTTCAGCTTCATAG
>EqcaTAS2R18EP_chr6:39075326-39074407
ATGTCAATTGGAATGAAGGTCTCCCTTTTTGTTGTAGCAACAGGAGAACTCATTTTAGAAATGCAGGGAAATGGGTTCAT
TGGACTGGTGAACTCATCAAATGCATCAACAATGGGAAGATCCTATCAGCTGATTTCAACCTTACCAGCATGCTATGACC
AGAATCATTCAACTGTGGGTAACAGCATTTGGTTTATTTAATGTGAGGGCTCTTTCCACATCTGTAAGCCACCAGTAAAT
CAGCAAAAGCAGTTACTATCCTTTGGGCACTAACCAATCACTTAACTACCTGGTTTGCCACCTGCCTAAGCATTTTCTGC
TTCCTTAAGATAACCAGTTTCTCCCACTTGTTCATCTGGCTGAAGTGGAGAGTGAACAGAATGTTTCTTCTGCTTTTTCT
GGGGTCTTCCTTCTTGATGTCTGTTAACCTCTTAATGGAAGATGCTCTTAATGAGTTGTTGATTAGTACCTATTGCATAT
ATGAAAGGAACATGATTTCACTTTTATTTAGATGTAAATAAATTTTCTATATCAAAAGACTTCTTCTTCTTAGCTTGACC
TATGTCCTTTCTTCTGTCACTGACCTCTTTGCTGCTTTGCTGGTGAGACACACCAAGAATTTTCAGCTCAATCAGATGGG
CCGGAGGGACTCCAGCACAGCAACCCATAAAAGGGCCATGAAAATGTTGACAACCTTCTTTCTCCTCTTCAGCATTTGCT
TTATTTTCACTCTAATAACAAGTAGGATCTTCCTTAAGGTACAGAGGTATCAGGTTATGGTGTTTATCATGGTGATTTCA
CCCTTCTTTCCCTCAGGCCACTCATTCATTATAATTTTTGGAAACAGTGAGCTAAGACAGATCACTTGAGACTACTGTAG
CATTTTAAATTCTAAGAAAAGAAAACTTTTATCTTCAGAG
>EqcaTAS2R18FP_chr6:39015042-39014114
ATGTCGATTGTAATGAGGGCTTCCTTTCTGCTCATGGCAACAGGAGAACTCATCTTAGGAATGCTGGGAAATGGGTTCAT
TGGGCTGGTAACCCGCATCAAATGGATCAAGAATGGGAAGAGCTCATCAGCTGATTTCATCCTACCAGCTTGGCTATGGC
CAGAGTCATCCAACAGTAGGTAACACTATTTGGTTCATTGAATGTGGAGCTATCTCCACATCTGTATGCCACCAGGAAAC
TAGCAAAAGCAGTTACTATCCTTTGAGCACTAACTGATCACTTAACTACCTGGTTTACAACCTGCCTAAGCATCTTCTGC

TTCCTTAAGATCGCCAATTTCTCCCACTTCTTTTTCATCTGGCTGAAGTGGAGAGAGAATAGAATGGTTCTTGTGCTTTT
CCTGGGGTCTTTCTTCTGATTGTCTGTTAACCTTTTAATGCAGGATGCTCTTAGTGAGTTGTGGATGAATATCCATAGAG
GACGTGAAAGAAACATGACTTTGCATTAAGTGAAAATAAAATTTTCTATCTTAAATGCCTTCTTCTTCTTACTTGACCTA
TATCATCCCCTTTCTTCTGTTCCTGACCTCTTTGTTCCTTTGTTTCTGTCCGTGATGAGACACACCGAGAATTTGTACCT
AAACCTGATGGATATAAGGGACTCCAGCACAGAGGTCCATAAAAGGACCGTGAAAATGGTGACAACCTTCTTCCTCCTCT
TCATCATTTACTTTATTTCCACTCTAAGAAAAAGTTAGATCTTCCTTAAGGTACAGAGGTTTCAGGTTATGATGTTTGTT
ATGGTGATTTCAACAATCTTTCCTTCAGGCTACACATTAATTATAATTTTGGGAAATAGCAATCTAAGAAAAATCATCTG
AGATTACTGTAAATTCTCTGTGAGAGAAGCAAAAGTTTTATCATCATAG
>EqcaTAS2R18GP_chr6:39004815-39003883
ATGTCACCTGGAATGAAGGTCTCCTTTCTTGTCGTGGCAATAGGATAACTCATCTAAGGAATGCTAGGAAATGGGTTCAT
TGGACTGGTAAACGTCATCGAATGGATCAAGAATGGAAAGGTCTCATCAGGTGATTTCATCCTTTCCAACTTGGCTGTGG
CCAAAGTCATTCAACTGTGGGTAACTCTATTTGGTTCATTTAATGTGGGTCTATCTCCACATCTGTATGCCACCAGGAAA
CTAGCAAAAACGCGGTCACATTCTTTGGACACTTACTGATCACTTTACCTGGTTTGCAACCTGCCTAAGCATTTTCTGCT
TCCTTAAGTTGGCCAATTTCTCCCACTTCTTTTTCATCTGGCTGAAGTGGAGAGTGAACAGAGTGGTTCTTGCAATTTTC
CTGAGGTCTTTTTTCTTACTGTTAATCTCTTAATGCAGGATGCTCTTAGTGAGTTGTGGGTGAATACCTGTAAAGTACAG
GAAAGAAATATGACTTTGTACTTAGACAGTAATAAAATGTTCTGTCTCAAAAGCCTGTTCTTCTCAGGTTGACCTATGTT
TTCCCCTTTCTTCTGTCTCTGACCTCTTTGTTCCTTTTATTTCTGTCCTTGCTGAGACACACCAAGAATTTGCTGCTCAA
CCTGATGGGTATGAGGGACTCCAGCATAGCGGCCCGTAAAAGGGCTGTGAAGATGGTGACAACCTTCTTCCTCCTCTTCA
TCATTTACTTTGTTTTCACTCTACTAGCAAGTTGGATCTTCCTTAAGGTATAAAGTATCAGGTTATGATGTTTCTCATCG
TAATTTCAACCATCTTTCCCTCAGGCCACTCATTAATTATAATTTTGGGAAACAACGAGCTAAGACAGATGACCTGAGAC
TACTGTAGCATCTTACATTCTCTCTGGGAAAAGCAAAACCTTTATCTTCATAG
>EqcaTAS2R38P_chr4:94760210-94759209
ATGTTGACTCTGCCTTCCATCATAATTGTGTCCTACAAAGTCAAGAATGCATTTCTATTACTTTCATTCCTGGAGTTTGC
TCCGGGAATCCTGGCCAATGCCTTTATTTTCTTGGTGAATTTTTGGTGAGGAAGCAGCCACTAAGCAACTGTGATCTTAT
CCTGCTATGTCTCAACCTCTCTTGGCTTTTCCTGCATGGGCTCTGTTTCTGGGTGCCATACAGCTTACCCATTTCCAGCA
GATGGAAGACCCACTGAGCCTCAGCTACCAAACTATCATCATGCTCTGAATGATCACAAATCAAGCTGGCATCTGGCTTG
CCACTTGCCTCAGTCTCCTCTACTGCTCCAAGATTGTCCATTTCTCTCACACCTTCCTGCTCTGCTTGGCAAGCTGCATC
TCCAGGGAGATCCCCAAGATGCTCCTGGGTGCTATTCTTTTCTCCTGTGTCTGCACTGTCCTCTGTTTGCGGGACTTTTT
CAGTACATCTCACTCCACAGTCTCAACTATGCTATTCATGAATAACAATTCACAACTCAATTTGCAAATTGCAAAACTCA
ATTTCTTTCATTCCTTCCTCTTCCACAGCCTGGCGTCCATCCCACCTTTCTTATTTTTTTCTGTTTCTTCTGGTATGTTA
ATTATCTTCCTGGGGAGGCACATGAGGACAATGAGGGCCAAAACTAGAGAGCCTTGCCATCCCAGCCTGGAGGCCCAGAT
CAAAGCACTCAAATATCTCTCATCTCCTTTCTCTGCCTCTATGTGGTGTCGTTCTGCGCTGTCCTCATCTCAATACCTCT
ACTGATGCTGTGACATAGCAAGATTGTCGTAATGGTCTCTGCATGGATAATGGCAGTTTGTCCCTCGGGACATGCAGCCA
TCCTGATCTCAGGCAATGTTAAGCTGAGGGGAGCTGTGGAGACCATTCCACTCTAGGCTCAGAGCAGCCTAAAGGTAAGG
GCAGACTACAAGGCAGATCCCAGGACACCAGATCTATGTTGA
>EqcaTAS2R41P_chr4:96551272-96552199
ATGAAGCCAGGGCTCACAGCCTTCTTTATACTGCTTTTTGCCCTCCTCTGTGTCCTGGGAATCCTGGCCAATGGCTTCAT
TGTGCTGGTGCTGAGCAGAGAATGGATGCGGAGTGGGAGGCTGCTTCCCTCTGACATGATCCTTATTAGCTTGGGTGCCT
CCCGCTTCTGCCTGCAGTGGGTTGGAATGGTGAGAAACTTCTACTTCTTCCTCCATCTGGTCGAGTACTGCAGGGGTACC
GCATGGCAGTTCTTTGGTCTACACTGGGACTTCCTGAACTCAGCCACCTTCTGGTTCGGCACCTGGCTCAGTGTCCTCTT
CTGCGTGAAGATTGCTACCTTCACCCACCCCACCTTCCTCTGGCTGAAGTGGAGGTTCCCAGGGTCAGTGCCCTGGCTCC
TCCTAGGCTCTCTCCTGGTCGCTTTCATTGTCACCCTGCTCTTCTTTTGGGGGAACTACAGTATGTATCAAAGGATTCTT
CATTAGAAAATTTTCTGGAAACATGACCTACGAGCAATGGAGCAGGAGGCTGGAAATTTACTATTTCCTACCCTTGAAAT
TTATCACGTTGTCAATTCCTTGCTCTATTTTCCTGATCTCAATTGCACTGTTGATTACTTTTCTGAGGAGACACACACGG

AGAATGTGGCATAATGCCCACAGCCTGCAGGACTCTAGCACCCAGGCTCTCACCAGCGCTCTGAGGTCAATCATCTCCTT
CCTTGTTCTTTATGTGATGTCCTTTGTGTCACTGGTCATCGATGCTGCACTGTTTTTGTCCTCAGACAGTGACTGGTACT
GGCCATGGCAAATTTTAACTTACCTGGGCACATCTGTCCATTCCTTTATTCTCATCCTCAGCAACCTCAGGCTTCGAAGG
GTGTTCAGGCAGTTACTTCTGTTGGCCAGGGGCTTCTGGGTGGCCTAG
>EqcaTAS2R42P_chr4:39214038-39213058
ATGTTCACTGTATTGGATAAAATCTTTCTGACGCTGGCAGGAGTGTAATTCATAATCGGAATGTTAGGGAATGTGTTCAT
GGGGCTGGTAACCTTCTCTGAATGGGTCAAGAACCAAAAGATTTCCTTAGCTGACTTCATCCTTACCTGCTTGGCTATCT
CCAGAATCACTCAACTGTTGGTTTTATTGTTTGAATCATTTATGCTGGGACAACCTTCGTGTTTCTATGCCACTTATAAA
CTAGCAAAACCTATCACTTTGCTTTGGAGAATGACTAATCACTTAACCACATGGTTTGCTACCTGCCTAAGCATTTTCTA
CCTCCTTAAGATAGCTCACTTCTCCCATTCCCTTTTCCTCTGGCTGAAGTGGAGAATGAACAGAGTGGTTCTTGTGATTT
TTGTATTTTCTTTGTTGTTTCTGATTTTTGACTTTCTATTGCTAGAAACATTTAACGATCTCTTCTTCAATATCTGTAAA
ATAGATCAAAGTAATCTGACTTTATATTTAGATGAAAGAAAAATTCTTTATGTTAAAACCCAGATTCTTCTTAGCTTGAC
CTATTTCATACCTATTGCTCTGTCTCTGATTTCGTTGCTCCTTTTATTTCTGTCCTTGGGAAGACACAGCAGAAATTTGA
AGCTCAACTCCATGGGCTCAAGAGATTCCAGTACAGAGGCCCACAAAAGGGCCATGAAAATGGTGATGTCCTTCCTCTTC
CTTTTCGTAGTTCATTGTTTTTCACACAATTGACACATTGGATAATTATGTTTTGTAACAGCAAATTCACAAAGTTTGT
CTTATTAGCATTATATGTCTTTCCTTCAGGCCACGCATTTATTTTGATTCTGGGAAATAACAAGCTAAGACAGACAGCCT
TGAAGGTACTGTGGCATCTTAAAAGCTCCTTAAAAAGAGAAAATCCATTAGCTTTACAGGATAGACTTTCCAGAGCCTTT
TCAAAGATAGTAACTCAGTGA
>EqcaTAS2R408AP_chr6:39172524-39171366
ATGATAACTTTACTACCAAGCATTTTTCTGTCCTAATAACGACAGAATTTGTTCTGGGAAATTTCGCCAATGGCTTCAT
AGCACTGGTGAACTGCATTGACTGGGTCAAGAGACAAAAGATGTCCTCAGCTGATCAAATTCTCACAGCTCTGGCGGTCT
CCAGAATTGGTTTGCTCTGGGTAATCTTTTTTTTTTTTTTTTTAAAGATTTTATGTTTCCCTTTTTCTCCCCAAAGCCC
CCCGGTACATAGTTGTGTATTCTTCGTTGTGGGTTCTTCTAGTTGTGGCATGTGGGACGCTGCCTCAGCGTGGTCTGATG
AGCAGTGCCATGTCCGCGCCCAGGATTTGAACTGACGAAACACTGGGCCGCCTGCAGCGGGGTGCGCGAACTTAACCACT
CGGCCACGGGGCCAGCCCCTGCTCTGGGTAATCTTAATAAATTGGTATATGACTGTGCTTCCTTCAGTTTTTCATAGTTT
AGAAGTAAGAATTATTGTTTTTATTGCCTGGACAGTAAGCAACCATCTTAACATCTGGCTTGCTACTAGCCTCAGCATAT
TTTTTTACTTGCTAAAGATAGCTAATTTCTCTAGCCTTATATTTCTTTACCTAAAGTGGAGAGTTAAAAGTGTACTTCTC
GTAATACTGTTGGGCGCTTCGGTCTTTTGGGTTTCTCATCTTGCAGTGCTATGGGTAAATAATAATGTGCAGACTAATGA
ATTTGAAGGAAACATCACTCAGAAGACCAAATTGAGGGATATTGTAGCTCTTTCGAATTTGACTCTATTCACGCTAGTAA
ACTTCATACACTTTTCTATGTCTCTGACATGTTTTCTGCTGTTAATCATTTCCCTGTGGAAACATCTCAAGAAGATTCAG
CTTAATGGCAAAGGATCCCATGATCCCAGCACCAAGGTCCACATAAGAGCCATGCAAACTGTGGTCTCCTTTCTCTTGCT
ATATGCTGTTTACTTCTTGGCTCTAGTTATCTTAGTTTGGAGTTCTAATAGGCTGGAGAGTCAACTGCTTGTCATGCTTT
GCCGGGCTTTTGAAATACTCTATCCTTCAAGCCATTCATTTATTCTGATTTGGGGAAACAAGAAGCTAAGACAGGCCTTG
CAAAATATACGAAATATTATAAGATTACCATACTATTAA
>EqcaTAS2R408BP_chr6:39145041-39144132
ATGGTAACTTTACTACCCAGCATTTTTCCGTCATAGAAATGACAGAATTTATTCTGGGAAATTTCGCTAATGGCTTCAT
AGCACCGCTGAACTTCATTGCCTGGGTCAGGAGACGAAAGATGTCCTCAGCTGATCAAATTCTCATGGCTCTGGCGGTCT
CCAGAATTGGTTTGCACTGGGTAATATTAATAAATTGGTATACAACTGTGCTCCATCCAGTTTTATATAGTTTTGAAGTA
AGAATTTTTGTTCATATTATCTGGGCAGTAAGCAACCATTTTAACATCTGGCTTGCTACTAGCCTCAGCATATTTTATCT
GCTCAAGATCGCTAATTTCTCTAGCCTTATATTTCTTTACCTAAAGTGGAGAGTTAAAAGTGTAATTCTGGTAACACTAT
TAGGGACTTTGGTCTTTTTGGTTTTTTTATTTTGCAGTGATATGCATAGATGAGAAAACACAGACTAATGTGTATGAAGG
AAACATCACTGGGAAGACCAAACTGAGGGATATTTTACGTGTTTCAAATATGACTGTATTCGTGCTAGTACGCTTATTAC
CATTTTCTACGTCCCTGACATGTTTTCTGCTGTTAATCATTTCTCTATGGAAACATCTCAAGAAGATGCAGCTTAATGGC
AAAGGATACCAAGATCCTAGCACCAAGGTCCACATAAGAGCCATGCAAACTGTGTTCTCCTTTCTCTTGCTATATGCCAG

TTACTTACTGGCTCTAGTTATCTCAGTTTGGAGTTCTAATAGGCTGCAGAATGAACTGCTTCTCATGCTTTGTGAGGTTC
TTGCAGTACTGCATCCATTAAGCCACTTGTTTATCCTGATTTGGGGAAACAAGAAGCTAAGACAGGCCTTGAAAAACATA
AGAAAGATTACAAGATTACCATACCATTAA
>EqcaTAS2R408EP_chr6:39078968-39078060
ACAATAACTTTACTACCAATCATTTTTTCCGTCCTGAAAATGACAGAATTTATTCTAGGAAATTTTGCCAATGGCTTCAT
AGCACTGGTGAACTGCATTTACGGGGTCAAGAGATGAAAGATGTCCTCAGCTGATCAAATTCTCATGGCTCTTTCCATCT
CCAGAACTGGTTTGCTGTGGCTCATGTTAATAAATTGGCATGCAACTGTACTTACTCCAGTTTTATATAGTTTAGAAGTA
AGAATGATTGTTCGTATTGCCTGGGCAGTGAGCAACCATTTTAGCATGTGGCTTGCTACTAGCCTCAGCATATTTTATTT
GCTAAAGATAGCTAATTTCTCAAGCTGTATATTTCTTTACTTAAAGTGGAGAGTTAAAAGTGTACTTCTCGGAATACTGT
TGGGGACATCGGTCTTTTTGGTTTCTTATCTTCCAGTGCTATGCATAGATGAGAATATACAGACTAATGAGTATGAAGGA
AAGATCACTTGGAAGACCAAATTGAGGCACACGGTATACCTTTCAAATATGACTCTATTCATGCTAATAAACTTTGTACC
CTTTGTTATGTCCTTGACATGTTTTCTGCTGTTAATTATTTCCCTATGGAAACATCTCAGGAAGATGCAGCTCAATGACA
AAGGATCCCAAGATCCCAGCACCAAGGTCCATCTAAGAGCCATGCAAACTGTGGTCTCCTTTCTCTTCCTATTGTCCTGT
TACTTCCTGACTCTAGTTATCTCAGTTTGGAATTCTAATAGGCTCCAGAATGAATGGCTTCTCATGCTTTGCCAGGCTCT
TGGAATGTTGCATCCTTCAAGCCATTCGTTTATCCTGATTTGGGGAAACAAGAAGCTAAGACAGGCCTTGAAAAATATAA
GAAAGATGATAAGATTACCATACCATTAA
>EqcaTAS2R408FP_chr6:39060005-39059152
ATGAGAAGTTTACTACAAAGCATTTTTTTTTTATCCTAATAATGACAGAATTTATTCTGGGAAATTTTGCCAGTGGCTTC
ATAGCACTGGTGAACTGCACTGGCTGAGTCAAGAGATGAAAGATGTCCTCAGCTGATCAAATTCTCACTGCTCTTGTGGT
CTTGAGAATTGGTTTGCTCTGGGTAATATCAATAAATTGGTATACAACTGTACTTACTCCAGTTTTATATTTTAGAAGTA
AGAATTATTGTTTATGTTGCCTTCACAGTAAGCAGCCATTTTAGCATGTGGCTTGCTACTAGGCTCAGCATATTTTATTT
GCTCAAGATAGCTAATTTCTCTAGCTTGAGAGTTAAAATTTTATTTCTCAGAATACTGCTGGGGAGTTTGGTCTTTTTCT
TATCTTGCAGTGCTATTCATAGATGAGAATACACAGACTAATGCACATGAAGGAAACATCACTGGGAAGACCAAATAGAG
GGATTTTGTAGGCCTTTCAAATATGACTCTACTCACGCTAGTAAACTTGATACCCTTTACCATGTCCCTGACATCTTTTC
TGCTGTTAATCATTTCCCTCAATGGCAAAGGATCCCAAGATGCCAGGACCAAGGTCCACATAAGAGCCATGCAAACTGTG
GTCTCCTTTCTCTTGCCATATGCCAGTTACTTCCTGGCTCTAGTTATTTCAGTTTGGAACCTAACAGGTGGCAGAATGAA
CCAGTTTTTATGCTTTGCCAAGCTCTTAGAATGCTGTATCCTTGAAGCTACTTATTTATACTGATTTGGGGAAACAAAAG
TTAAGACAGGCCCTGAAAAATATGAGATGGATTATAAGATTACCATATCATTAA
>EqcaTAS2R408HP_chr6:39008977-39007992
GTGGCAACTTTACCACCAAGCATTTTTTTCTGTCCTAATAACGACAGAATTTATTCTGGGAAATTTTGTCAAGGCTTCAT
AGCACTGGTGAACTGCATTGACTGGGTCAAGAGACAAAAGATGTCCTCAGCTGATCAAATATTCACACTCTGGTGGTCTC
CAGAATTGGTTTGCTCTGGGTACTATCAATCACTTGGTATACAACTGTGCTCCTTCAAGTTTTATATAGTTTAGAAGTAA
AAATTATTGCTTGTATTGACTGGACAGTGAGCAACCATTTTGATGTCTGGCTTGCTACTAGTCCCAGCATACATTTTTTT
TTTTTTTTAAAGATTTTATTTTTTTCCTTTTTCTCCCCAAAGGTCCCCCGGTACATAGTTGTATATTCTTAGTTGTGGGT
CCGTGAGGTGTGGTATGTGGGATGCCGCCTCAGCGTGGCTCAATGAATGGTGCCACGTCCGCGCCCAGGATTCGAACTGA
CGAAACACTGGGCCGCCTGCAGCGCAGCGCGCGAACTTAACCACTCTGCCACGGGGCCGGCCCCAGTCCCAGCATACATT
TTTGTTCAAGATAGATAATTTCTCTAGCTTTGTATTTCTTTACCTAAAGTAGAGAGTTAAAAGTGTACTTCTCGTTATGT
TGTTAGGGACTTTGGTCTTTTTGGTTCCTCATCTTGCAGTGCTATGCATGGATAATAATATGCAGACTAATGAGTATGAA
GGAAACATCACTCAGAAGGCCAAATTGAGGGACATTTTATACCTTCCAAATGTCACTCTATTCACACTAATACACTTCAT
ACCCTTTACTATGTCCCTGACATCTTTTCTGCTGTTAATCATTTCCCTGTGGAAACATCTCAAGAACATGCAGCTCAATG
GCAAAAGATTCCAAGATCCCAGCACCAAGGTCCACATAAGAGCCATGTAAACTGTGCTCTCCTTTCTCTTTCTATATGCT
GGTTACTTCCTGATTCTAGTTATCTC
>EqcaTAS2R45P_chr6:38987530-38987241
ATGATACATTTACTACAAAGTATTTTTTCCATCCTAGTAATAGTAGAATTTCTTCTAGGAAATTTTGCCAATGGCTTCAT

AGCACTAGTAAACTGCATTAACTAGGTCAAGAGACAAAAGATCTCCTCAACTGATCAAATTCTCCTGGCTCTGGAAGTCT
CTAGAATTGGTTCACTCTAGGTAATATCAATAAATTGGTATACAGCTGTGTTTAATCCAGCTTTATATAGTTTAGAAGTA
AGAACTGATGTTTACATTGCCTGGATAATAGTCAATCATTTTAGCATTT
>EqcaTAS2R60P_chr4:96526366-96527308
ATGGTTTCAGGATGTTGGTGATTGATAGGAGAGCCATCACCTTGGCTATCATTTTATTCCTTTTGTGCCTGGTGGCAGAG
ATGGGCAATGGCTTCATCACTGTGGCACTGGGCATGGAGTGGTTACTATGGAGAACATTGTCACCTTGTGATGTTATTGG
TCAGCCTGGGAGTCTCTTGCTTCTATGTGCAGTGGGTGGTGATGAGTAAGAACATTTACGCTAGAATTGTGTGTCCACTG
GTCCTTCCATACAACCCTGTACTATAGTTTCTAGCCTTATAGTGGGACTTCTTGAACACTACCACCTTTTGGTTCTCTAC
CTGGCTCAGTGTCCTCTATTGCAGGAAAATTTCAACCTTCACTCACCCTGTCTTCCTCTGGCTAAAACAGAAGGTGTCTG
GGTTGGTTCCCTTGATGCTGCTCAGCTCCATGGGGTTCTCTAGCTTGAGCACCATCCTATTTTTCACAGGCAACCAGAGC
CAATATCAGAACTTTTTAAGGAGAGGTCTGCAATATTGGAATATCATCAGGAATCCTATAAGGAGATCACATGAGAAATT
CTACTTCTTTTGTTTAAAATTCGTTACTTAGGCAGTTCCTGCTGTTGTCTCCCTCACTGGTAGGATTTTAGCTCATCATG
TCTCTCGGAAGACACACCAATAAGCCCTTTCTGTCTGTCTCGGGCTTTTGCCATCCTAGTGCCCAGGCACACATCAGGAC
TTTCCTGGCTCTCATCTCCTTTGCTATCTTCTTCAATTCCTATTTTCTGTCACTGGTGCTCAGTGCTGCAGTTATTTTTC
CATCTTAGGAATTTAGGTACTGGGTGTGGCAGACAGTGATTTATCGGTGCACAGTAGTCCACCCCATCAGTCTACTCTTG
AGCAACACCAGGCTGAGAGTGGTGCCGGAGAGGAGCTGCTCCTCAAGGCATGGGGCATCTTGA
>EqcaTAS2R62CP_chr4:96504466-96505418
ATGCCCTCCTCATCCATGTTGATCTTCATGGTCATCTTTTTCATGGAGACCTTGGCTACAATGTTGCAGAATGGCTTCAT
TGTTGCTGTACTGGGCAGGGAGTGGGTGAGATGCTGCACACTGCCTTCAGGTGACATGATGGTATCCTGCCTGGCTGCCT
CCTTATTCTGCCTGCATGGGATGGCCCTCCTAAACAATCTCATTGACTCCTTTAACTTTTGTTCCAAAGTTTACTATTTC
AACACTTCCTGGGGCTTTATCAACGCTCTTACTTTCTGGCTTACTGCCTAGCTTGCTGCCTTCTACTGTGTGAAGATCTC
ATCCTTCTTTCATCCTGTCTTCCTCTGGCTAAAGTGGAGGATTTCTCGGTCAGTGCCCAGGCTGCTGCTGGGCTCCATGA
TTATATCTATCGTGCCAGTCATTCCATTAGCCACTTGGAATAGCATTCTTGTGCAGCTGAGTGCCTCCCAGAGTTCCCAT
GGAAACGGCACCCTATGTGAAAGAATACAGACCATCTCTCTCTACTCTTTTTGCCTACAGTGCTTATGTTGTTGATTCC
CTTTCTCCTGTTCCTTGTGTCCACCCTCTTGCTCATATTCTCACTGTACAGGCACTTGGGGAAAATGAGGGACCACAGAT
CCGGCCCATGTGATCCCAGCACCCAGTCTCACACCATGGCCCCGAAGTCATTTACTGTCTTCCTTGTCTTCTACACATCA
TATTTCCTCTCCCTGATTATTGCTTTTATGAAAATCACAACACTGAAGAATCAGAGCCATGGGGCCTGGGAAGTGGTGAC
CTATGCAGGCATCTGTCTGCATTCTAGCATCCTGGTGCTAAGCAGCCCAAAGCTGAGAAAGGCCCTGAAGAGAATCCTTT
GCAAGTCCCTGGACAAAAGATGGTTCATCACAAGTATCAATATCAATAATCAGTATCAATAGATAAGCCCTGA
>EqcaTAS2R62EP_chr4:96473408-96474309
ATGTCCTCCTCACCCATGTTGATCTTCATGGTCATCTTTTTCTTGGAGTCCTTGGCTGCAAATTTGCAGAATGGCTTCAT
GGTTGCTGTACTGGGCAGGGATTGGATGCAGTGCCACACATTGCTCACAGATGACATGACTGTGGCCTGCTTAGCTGCCT
CCAGGTTCTTCCTGCATTGGATGATTCCCCTGAATAAACTCCTAGCCTCCTTTGATTTTTGCTCCAAAATTCACTATTTC
AACATCCCTTGGAGCTTCATCAACACACTTTCTGCCTCCCTGCCTGGCGTGCTGTCTTCTACTGTGTGAAGATCTCGTCC
TTCTCTCATCCAGTCTTCTTCTTGCTGAAGTGGAGGATTTCTCAGTCACTGCCAAGGCTGCTGCTGGGTTCCCTGATCAT
ATCTGGTGTGACAGTCATCTCATTAGCCACTGGGCATAGCATTCTTGTGTAGATGGTTGCCTGCCATATTTCCTATGGAA
ACAGCACTGTGGCTGATATAACAGAGCACATCTCTTTGTACATTTTTCTGGCTAAGATACTGCTTGTGTTATTGATTTCC
TTCCTCCTGTTCCTGGTGTTCACTCTCTTGCTCATGTTCTCACTTCACCGGTCCTTGAGGCAGATGAGGGATCATAGACC
TGGCCCACACGATCCCAGCACCCAGGCTCACACTATGGCCCCGAAGTCACTTGCTGTCTCTCTCGTCTTCTACACATAAT
ATTTCTTGTCCCTCATTATTGTTTCTATGCATATCACAACACTTCATAATCACTGGAACTGGGTCTAGGATGTGGTGATC
TATGCAGGCATCTGTCTATGCTCCAGCATCCTGGTACAAAGCAGCCTCAAGCTGAGAAAGGCCCTGAAGATGATGTTGTG
GAAATCCCTGGACAAAAGGTGA
>EqcaTAS2R62FP_chr4:96462009-96462917
ATGCCCTCCTCATCCACCCTGATCTTCGTGGTTATCTTTTTCTGGAGACCTTGGCTGCAATGTTGCAGAACGGCTTCAT

GGTTGCTGTGCTGGGCAGGGAGTGGATGCGATGCTGCACACTGCCTGCAGGTGACATGATTGTGGCCTGCCTGGCTGCCT
CCAGGTTCTGCCTGCATGGGATGGCCCTCCTGAACAACCTCCTGGACTCCTTTAATTTTCGTTCCATCGTTTCCTATTTC
AACATCCCTTGGAACTTTATCAACACTCTCACTTACTGGCTGACTGCCTGGCTTGCTGTCTTCTACTGTGTGAAGATCTC
ATCCTTCTCTCATCCCATCTTCTGCTGGCTGAAGTGGAGGATTTCTCGGTCAGTGCTCAGGCTGCTGCTGGGTTCCCTGA
TCATATCTGGTGTGACAGTCATCCCAGCAGTCACCAGAGATATAATTCTTATACAGATGATTGCCTCCCAGAGTTCCCAT
GGAAACTGCACTCTGGCTGATAGAATAAGGACCTTCCATAGGTATTTTTTGATGCCTATATAGTTCTGGTATTGTTGATT
CCCTTCCTGCTGTTCCTGGTATCCACTCTTTTGCTCATATTCTCACTGCACCAGCACTTGGGACAGATGAGGGCCCACAG
ATCCAGCCCACGTGATCCCAGCACCCAGGCTCACACCATGGCCCTGAAGTCACTTACCTTTCTTCCTCGTGTTCTACACA
TCATATTGCCTGTTCCTGATTATTGCTTTTATGAAAATCACAACCCCTGCAGGATCAGTGGCACTGGGCCTGGGAAGTGG
TGACCTATGAGCCAGTAAAGAGGTGCACTCCAGCATCCTGGTGCTAAGCAGCCCCAAGTTGAGAAAGGCCCTGAAGATGA
GGCTTTGGAAAGCCCTGGACAAAAAGCAG
>EqcaTAS2R67P_chr6:39205784-39204906
CCGCCATCTGGAATTGAAAGCACTTTTCTGATAGTGGCAATAGGAGAATTCATAACTGGAATGTTGGGGAATGCGTGCAT
TGTACTGGTTAACTGCATTGACTAGGTGAAGAGTCAGCAGCTCTCATTAGCGGACTGCATCCTCACCAGCCTGGCTATCT
CCAGAACCAGTCATCTTTGGGTAATACTACTTGGTTAATTTGTCATATTGCCACATCTATATGCCAATGATAACTAGCAA
ACTTGGTTGGTATATTTTGGACACTGACCAATCACCTAGCTACCTGGTTTGCCGTCTATCTAAGCTTTTTCTACTTCTTT
AAAATAGCCAGTTTCTCTCACCCCTGCTTCGCCTGGCTGAGGTGGAGAATTAGCATAGTGTTACTTGTGCTTCCACTGGA
GTCTTTGTTCTGTTTTTCAACCTTGAACTGAAAGGTACAATTAATGGTTTCTCAATTTAGTCCTCAGATGTAAGTGAACT
TCTGTATCTTAACAGCTCGTTTGTTGTCAGTTTGATCTACTTAATCCCCCTTCTTCTGTCCCTGAACTCACTGCTCCTTT
TATTTCTCTCCTTGATGAGACATACCAGGAATTTGCCGCTGGACTCTAGCTCTTAGGGACCTCAGCTCAGAGACCCATAA
AAGGGCCACGAAAATGGTGATGTCTTTCCTCCTCCTCCTCATGGTTCACTTTTCTTCCACTCTATTAACAGGGTGGGTTT
TCCTTATACTGAAGAAACATTGGGCCAATTTGGTTAATGTCAACTCTTTTTCCTTCAGGCCACACATTTATCCTAATTTT
AGGAAACAGCAAGCTGAGGCAAAATGCCTTAGGACTATTGTGGCATCTTAATTGTCACTGGAAAGGGTGAAACTTTTAG
>EqcaTAS2R301AP_chr6:39176685-39175745
ATGGTAATTTTCCACTCAGAAGAAACTCAAGTGCTGAGGACAACATCTTTTTGGTTGTCTTAACAATGGAATTCATACTT
GGTACTTAGGGAAATGGATTAATTGCTATAATCAACTTCATTGACAATGCAAGACACAGGAAAATAAGCTTCATTAACCT
CATTCTCACTAACTTATTCATAAGCAGAATATGCTTCTTAACCTGATACTGATGGATTGATTTCTACTGGTGCTGTATGC
AGATCCACATAACATTGGTCAGCTTGGAAAAACTAATACTCTGTTTTGCATAATGATCAAATATTAAGTGTCTGGTTTAA
CATCAGCCTCAGCATCTCCTATTTCCTGAAGATAGCTATGTTTTTTACAAACCTTTTCTTGATTAAAGTGGAGAATTAAT
AGGGTGTAATATTTTTCTAAGGTCTTTGCTCTTTTTTTCTTTTTGCATCCCAGTGATAGACCATGTAATTCCAGATATTT
TAAATCTAGAAACAAACAAACCAGACTTGGAAGATTAATATGCATGAAACAAAACTATTATTTTTGTAGTGCTTATCAGT
GTTGAGATCACTACACCCATAAATATTTCCATAGCGTTGGTTCTTCTGTTACTTCCTTCTTCCTGGAGGTATATCTAGCG
GATGAAGTTCAGTATCTCAGGGTCCCAAGATCCCAGCATGAAGCTCATGGAAAAGCCATGAGAATGGTGATTTTGTTCTT
CTTTCTCTTTTTCACGTATATTGTGTTGATTCTAATAATAGTTACAGCCATTTATTGTCCCCAGACAAGCTGATATTGAC
ATTTGGTAAAGTAATAGCATCTGCCCATCCTTTAGGTGATTCATTTATCCTAGTTTTGAGAAACACCAAGCTGAAGCAAC
CTTCTCTTAGGATTTGGGGGCAGCTGAAGTGCTTTCTTAAAGGAAGGAAAAAACTCAATAA
>EqcaTAS2R301BP_chr6:39150930-39149983
ATGGTGATTTTCCACTCAGAAAAAATGTCAAGTGAACATGACAACCTGTTTTTTGTTGTCCTAAGTATGAAATTAGTACG
TGGTGCTTAGGGAAATGGATTGATTGGAATAACGAACTATATACTCTGGGTAAGACACAGGAAAATCAGCTTCATTAACC
TCATCCTCATGAGCTTATCCGTAAGCAGAATGTGCTTCTTGATTATGATATTGTTTGATTCGTTTCTGCTGCTGCTGTCT
TCAAATCCACATAGCATTAATCAAATTGGAAAACCTAATATTCTGTCATGCATAATGATCAAGCTCTTAAACGTCTGGTT
TGATACCTGCCTCAGCATCTTCTGTTTCCTGAAGATAGCTACCTTTTTTCTGTCTTTTTCTTTGATTAAAGTGGAGAATT
AACAGAGTCGTTATTATTATTTCTCTGAGACCCTTGCTATTTTTTCTTTTTAGCATCCTACTGATAGTTCATCTAATTCC
TGATATTTTACATCTCGCAACAAACAAGACTTGGAGGATTAATATACATGAAACACAATCCATTATTTTCTTCGTGCTCA

TCAGTGTTGAGATCACCATGCCCATAACTATTTCCGTAGCATCAGTTCTTCTGTTACTTCTCTTTTTGGAGGTATAGCTA
AAGGATGGAGTTCAGTCTCACAGGGTCCCAAGATCCCAGGAGTTAAGCTCATGGAAAAGCCATGGGAATGGTGACTTCGT
TCTTCTTTCTCATTTGGATTTCTGTTGTGTTGACTTTATAATAATTACAGCCATTTATTGTCCCCTAACTAGCTGATATT
GACATTTGGTAAAGTAATAACATCTGCCCGTCCTTTAGGCCATTCATTTATCCTAATTTTGAGAAACAGCAAGCTGAAGT
AAGCTGCTGTTAGGATTTGGGGGCAGCTGGAGTGCTTTCTAAAAGGAAGGAAAACATAAACTCAATAA
>EqcaTAS2R301CP_chr6:39121181-39120264
ATGTCAAGTGCACAGGACAATGTCTTTTTGGTTGTTTTAACTATGGAATTCATACTTGATACTTAGGGAAGTGGATTGAT
TGGAATAATGAACTGCATTATCTGGGTAAGACACAGGAAAATCATCTTCATTAACCTCATCCTCATGAGCTTATCTATAA
ACAGAACGTGTTTCTTGATCATGGGGTTGATTGATTCATTTCTGCTGGTGCTGTCTGCATAGCCACATAGAATGGTCAAA
TTGGAAAAGCCAATCTTCTGTTTTATGTCACGATCAACCACTTAAATGTCTGGTTTGACACCTGCTTCAGCATCTTCTGT
TTCCTGAAGATTACTACATTTTACATCTTCTTTTTCTTTGATTAAAGTGGAGAATTAACAGGGTGGTTAGCATTATTTAT
CTGCAGTATTTGCTCTTTTTACTTTTTAGCATCCCAGTGATAGATCATATAGTTGCTGATATTTTAAATTTAGAAATAAA
CAAGACTTAGAGGATTAATGTGTATAAAACACAAACTACTATTTTCTTAGTGTTCATCAGTGTTGAGTTCACCATGCCCA
TAACTCTTTCCATAGCATTGGCTCTTCTGTTACTTCTTTCTCTTGGGAGGCGCATCTGGAGGATGGAGTTCAGTATCGCA
GGGTCCCAAGGTCCCAGAATGAAGCTCATGGAAAAGCCATGAGAATGGTGACTTTGTTCATCTTTTTCTTTTTGATTTAT
ATGGTGTTCATTCTTATAATAGTTTACAGCCATTTATTGTCCCCTAACAAGTTGATGTTGACATTTGGTAAATTAACAGC
ATCTACCCATTCTTTAGGCCATTTATTTATCCTAATTTAGAGAAACAGCAAGCTGAAGCAAGCTGCTGTTAGGATTCGGG
GGCAGCTGAAGTGCTTTTTAAAAGGAAGGAAAACATAA
>EqcaTAS2R301DP_chr6:39089539-39088592
ATGGTAATCTTTCACTCAGAAGAAATGTCAAGAGCACAGGACATCTTATTGGTTGTCTTAACCATGGGATTCATAATAGG
TATTTGGGGAAATGGATTGTTTGGAATAATCAACTTCACCAACTGGGTAAGACACAGGGCAATCATCATCATTAACCTCA
TCCTCAACAGCTTATCCACAAGTAGAATATGTTTCTTGATCCTGATGCTGATTGAATCATTTCTACTGGTGCTGTCTGTA
CATCCACATAGCATTGGTCCATTTTGAAAAACTAATAATTTGTTTCACACACTGATAAAGCACTTAAGTGTCTGATGACG
TCTGCCTCAGAATCTTGTTTCCTGAAGATAGCTACTTTTTTTCATCTGCTTTTTCTTTGATTAAAGTGGAGAATCAACAG
GGTGATTATTACTATTTTTCTAGGGTCTTTGTTTTCTGTTGTTTTTAGAATCCTGGTTATAGGCCATATAATTCCTGATA
TTTTAATTCAAGAAACAAACAATACTTGGAAGATTAATAGGTATGAAACACAAACCATTATTTTCTTAGTGCACATCAGT
GTTGAGATCATCACGCCCATAGCTATTTCCATAGCATCAGTTCTTCTGTTACTTCCTTCTCTTTAGAAGCATACCTAGAG
GATGAAGTTCAGTATCATAGGGTCCCAAGGTCCCAGCAGTGAAGCTCATGGAAAAGCCATGAGAATGGTGACTTCCTTCT
TATTTCTCTTTTTGATTAATATTGTGTTGATTCTTATAACAGTTTATAGGCACTTATTGTCCCCTAACAAGCTGATATTG
ACATTTGGTAAATTAACAGCATTTACCCATCCTTTAGGCCACTTTTTTTATCCTAATTTTGAGAAAGAGTAAGCTCAAGC
AAGCTGCTCTTAGAATTTTGGGGCAGCTGAAGTGTTTTCTGAAAGGAAGGAAAACATTAACTCAATAA
>EqcaTAS2R301EP_chr6:39036142-39035217
ATGGTAATATTTCACTCAGAAGAATTTTAAAGTGCACAGGAAAACATTTTTTTGTTGTCTTAACATGGAATTCATAATAG
GTATTTGGAGAAATGATTGGTTGGAATAATCAACTTCACTGACAAAGTAAGACACAGGAAAGTCAGTTTCATAAACCTCA
TCCTCACTAGCTTGTTCACAAGCAGAATATTTTTCTTGATCCTGATACTGACTGAATCATTTCTAGTGGTGTTATCTGCA
CATCCACATAGCATTGGTGCATTTTGAACAACTAATCTTCTGTTTTGCATAATGATCAACCACGTAAGTTTCTGGTGTGA
CACTTGCCTCAGTATCTTCTATTTCCTGACGATTACTACTTGTTTTGCATTCTCTCTTTTTCTGATTAAAGTGGGGAATT
AGTAAGGTGGTTATATCTATTTTTCTGGAGTCTTTGTTCTTTTTAGCATTCCAGTGTTAGACCATAGAATTCCTCATATT
TTAAATCTAGAAAAAAACAAGACTTGGAAGATTAATATGTATGAAACACAAACCATTTTCTTAGTGCTCATCAGTGTTGA
GATCACCATGCCCATAACTACTTCCATACCATCGGTACTTCTGTTACTTCTTTCTCTTTGGAGGAACATCTAGAGAACGA
AGTTCAGTATCACAGGGTCCCAAGATCCAAGTAGTGAAGCTTATGGAAGAGCAATGAGAATGGTAATTTCTTTCTTCTTT
CTCTTTTTGATTAATATTGTGTTGATTCTTATAACAGTTACAGCCATTTATTGTACCCTAACAAGCTTATGTTGACATTT
GGTAAATTCATAGCAACTGCTTATCCTTTAGACCATTCGTTCATCTTAATCTTGCAAAACAGCAAGCAGAAGCAAGCTAC
TCTTAGGATTTGGTGCAGTTGAAATACTTTCTAAAAGGAAGGAAAA

>EqcaTAS2R301FP_chr6:39022610-39021662
ATGGTAACTTTTCACCCAGAAGAATTGTCAAGTGCACAGGACAACATGTTTTTGGTTTCTTAATTATGGAATTCATACTT
GGTACTTGGGGAAATGGATCTATTGGAATAATGAGCTATATTGTCTGGGTAAGACACAGGAAAAACAGCTTCGTTAGCTT
CATCCTACTGAGCTTATCCATAAGCAGAATGTACTGGATCATCATACTGATTGATTCACTTCTGATGGTGCTGTCTGCAG
ATATACATAGCCTGGCCAATTGGAAAAATTAATATTCTCTTTTATGTAATGATCAACTACTTAAATGTCCGGTTTGAAAC
CTGCTTCAGCATCTTCTTTTCCCTGAAGATCGTTACTTTTTTTTCCCCATCCTCTTTTTCTTTGATTAAAGTGGAGAACT
AACAGATAGTTATTATTATTTTCCTGAGGTCCCTGCTCATTTTTCTTTTCAGCATCATGGTGGTAGTCCACATAATTCCT
GATATTCTAGATCTAGAAACAAACAAGACTTGGAAGATTAATACATATCAAACAAAACCCACCATGTTCTCAGTGCTCAT
CAGTGTTGAGATCACCCTGCCCATAACTCTTCCCACAGCATTGGTTCTTCCATTATTTCTTTCTCTTTGGAGGCATAGCT
AGAGGATGGAGTTCAGTATCACAGGGTCCCAAGATCCCAGAAGTGAAGCTCATGGAAAAGCCATGAGAACGGTGAATTCT
TTCTTCTTTCTCTTTCTGCTTTATATTGTGTTGATTCTGATAACAGTTACATCCATTATTGTCTCCTAACAAGCTGATAT
TGACGTTTGGTAAATTAATAGCATCTGCCCATCCTTTAGGCCATTCATTTATCCTAATTTTGAGAAACAGCAAGCTGAAG
CAAGCTGCTGTTAGGATTTGGGGGCAGCTGAAGTGCTTTCTAAAAGGAATGAAAGCTTAAACTCCATAA
>OrorTAS2R1P_NW_004438476.1:7234566-7235457
ATGCTGGAGTCTCACCTCATTAGCCACCTTTGTTTGGCAGTGATAAAATTTCTCTTTGGGGTTTTAGTAAATGGCATCAT
TGTGGTTGTGAATGGCACTTACTTGATCAAGCAGAGAAAGATGATTCCATTGGATCTCCTTGTTTCCTGCCTGGCGATTT
CCAGGATTTGTCTGCAATTAGCCATCTTCTACGTTAACCTGGCTGTTCTTTCCTTGATTGAATTCCCTCAGCTTGCTGAG
AAGTTCGTAATTCTCACATTTATAAATGAATCGGGACTTTGATTTGCCACATGGCTCAGCCTTTTCTACTGTGCCAAGAT
TGCCACCATTGCTCACCCACACTTCCGCTTGAAGGTGAGGATATCCAAGTTGGTTCCTTGACTGGTACTTGAGTCCCTGC
TATATGCATCCAGCATGGATGTTTTCCACAGCAAACATAGGTGGATATTTTCCAAAGAACACTTCCTGGGCCTTTTCTCC
CCAAATGCAACCACCCAATCAAAGAAATACCCGCTTTACAGTTTGCCTTTCTTTTAGCTGAGTTCTCATTGCCATTACTT
ATCTTCCTTATTTCTTCTCTGCTCTTGATATTTTCTCTGGGGAGACACACCTGACAGATGAGAAACACAGCAACAGGCCC
CAGGAACCCTCGCACATGCATGCACATCAGCACTTTTCTCTCCATCCTGTCCTTTCTGGTCCTCTCTGCCACTCCATGAC
AGCTGCTTTGCTCTTTTCTCAAATTTTCAACTTTAGAAGCTTCATATTTCTGTTCTGCATCTTGCGGGTTGGTTCATACC
ACTCTGGACACTCTATTACCTTAATTTTAGGAAATCCTAAAATGAAACAAAATGCAAAGAAATTGCTCCTCCAAAGAAAG
TGCTGTCAGTGA
>OrorTAS2R2P_NW_004438428.1:730968-730060
ATGGCCTCCTCTTTGTCAGCTCGTCTTCATGTTATCCTCATGTCAGCAGAATTTATCACAGGGATTACAGTAAATGGATT
TCTTATAATCATCAACTGTAATGAATTGGTCAAAAGCAGAAAGCTGACACCAATGCATCTCCTTTTCATATGCATAGGGA
TGTCTAGATTTGGTTTGCAGATAGTGTTAATGGTAAAGTTTTTCTCATGTTCTTTCCACTCTTTTATAGAGTAAAAATTT
ATGGTACAGCGATGATTTTTTGGGGATGTTTTTCAGCTCTGTCAGTCTCTGGTTTGCCACCTGTCTCTCTGTATTTTACT
GCCTCAAGATAACACACTTCACCCAGTACTGTTTTCTTTGGCTGAAATTCAGGATCTCAAAGTTAATGCCTTGACTGCTT
CTGGGAAGCCTGCTGACCTCCGTGAGCATTGCAACTCTGTGTGTCAAGGTGGATTACCCTAAAAATGTGGATATTGATGT
CCTCAGGGATGCCATGCTAAAGAGGACTAAACTCAAGACAAAGCAGATTAATGAAGTGCTTCTTGTCAGCTTGGCATTAA
TATTTCCTCTGGCCATATCTGTGAGGTGAACTGTTATGTTATTCATTTCTCTCTATAAACACGCTAATCGGATGCAAAAT
GGACCTCTTGGTTTTAGAAACGCCAGCACTGAAGCCCATATTAATACATTAAGATCAGTGATAACATTCTTTTGCTTCTT
TATTTCTTATTTTGCTGCCTTCATGGCAAATATGACATTCAGTATTCCTTATGGGAGTCAGTGCTTCTTTGTGGTGAAGG
ACATAATGGCAGCATATATCCCTCTGGCCATTCGGTTATAATTATCTTGAGTAATTCTCAGTTCCAACAACCAGTCAGGA
GACTTCTCTACCTCAGAAAGAATCAATGA
>OrorTAS2R3P_NW_004438464.1:2452522-2451362
ATGCTGGGACTCACCGAGTGCGGGTTTCTGGTTCTGACTGCCACTCAGCTCATTCTGGGAATGCCGGGGAATAGTTTCAT
GGGTTGGTCAATGGTAGCAGCTGGTTCAAGAACAAGAGAACCTCTTTGTCTGACTTCATCATCACTAACTGGGGTCTCTC
CAGGATTGTTCTGCTGTGGATTCTCTTTTTTTTTTTTTGCGGTACGCGGTCCTCTCACTGTTGCGGCCTCTCCCGTTGCG
GAGCACAGGCTCCGGACGCGCAGGCTCAGCAGCCATGGCTCACGGGCCCAGCCGCTCCGCGGCATGTGGGATCTTCCCGG

ACTGCGGCACGAACCCGTGTCCCCTGCATCGGCAGGCGGACTCTCAACCACTGCGCCACCAGGGAAGCCCTCTGCTGTGG
ATTCTCTTGATTGATGGTGTTCTCTTCCAAACTCCACGATGAATAATTTGCAGTCATGCAGATTAGTGATATTTTCTGGA
CATTTACAAACCATCTGAGCATTTGGCTTGCCACCTGTCTCAGTGTCTTCTACTGCCTGAAAGTCGCCAGTTTCTCCCAT
CCTACGTTCCTCTGGCTCAAGTGGAGAGTTTCCAGGTTGGTTGTATGGATGCTGTTGGTACCCTGCTCTTATCATGTAGC
AGTGCCGTCTCTCTGATCCATGAATTTAAGATCTAGTCTGTTCTCAGTGGAATTGATGGAACAGGGAATGTGAATGAACC
CTTTAGAAAGAAAAGAAATGAATATAAGCTGATCCATTTTCTTGGCACTCTGTGGGACCTCCCTCCCTTAATTGTATCTC
TAGCTTCCTACTTTCTGCTCATCCTCTCTCTGGGGAGGCGTATGCGGCAGATGCAGCAAAACTTTACCGGCTCCAGATAT
CCAAGTACTGAGGCCCAAAAGAGGGCCATCAAAATCATCCTTTCCTTCCTCTTTCTCTTCCTACTTTACTTTCTTTTCTT
TGCAATTTTGACATCCAGTTATTTCTACCAGCAACTGAGGTGATTATGATGACTGGAGAAGTAATTACAATGTTATATCC
TGCTGGCCGCTCATATATTCTCATTCTGGGAAATAATAAGCTGAAGCAGATGTTCATGGAGATGCTTTGGTGTGAGCCTG
GTCATCTGAAGCCTGGATCCAAGGAACCCGTTTTTCCATAG
>OrorTAS2R5P_NW_004438464.1:2435485-2434604
ATGCTGACTGCTGTCCTAGGACTGTTAATGCTGGTAGCAGTGGCTGAATTTCTCATTGGCCTGGTTGGAAATGGGGTCCT
CGTGGCCTGGAGTTTTGGAGAATGGCTCAGAAAATTCAAGGGGTCCTCATATAACCTCATTGTCCTGGGCCTGGCTGTCT
GTTGATTTCTTCTGCAGTGGTTGATTATGGTGGACTTAAGTCTGTTTCCACTTTTCCAGAGCAGCCATTGGCTTCGCTAT
CTCAATGTCTTCTGGGTCTTAGTAAGCCAGACCAGCCTGTGGTTTGCCACTTTTCTCAGTGTCTTCTACTGCAGGAAGAT
CATGACCTTTGAACACCCTGTCTACTTGTGGCTGAAGCAGAGGACCTGTTGTCTGAGTCACTGGTGCCTTCTGGTGTACT
TCATGATCAGTTTGTTACTTATAGTTCAGGGTAGCTTAGAGTTCTCCAATCTTTCCCAAGGAAACAGCAGCATTTTATAC
CCCCCTTCAAACTGGCACTGTCTGTATATATTATGGCTCAATACAGGAAGTATAATGCCCTTCATGGTGTTGCTTATTTC
CTCTGGGATGCAGATTGTCTCTTTGTGTAGACACCGCAGGAAGATGAATGTCCATACAGTCGGCAGGAGAGATGCTCAGG
CCAAGGCTCACATCACTGTCCTGAAGTCCTTGGGCTGTTTCCTTATACTTTACATAGTTTACATCCTGGCCAGCCCCTTC
TCCATCACCTCCAGGCCTTTTCCTGCTGATCTTACCGCTCTCTTCATCTCTGAGACACTCATGGCTGCCTACGCTTCTCT
TCATTCTGTCATATTGATCATGGGGAATCCCAGGATGAAGCAGACTTGTCAGAGAATCCTGTGGAAGACAGTGTACACTT
GG
>OrorTAS2R16P_NW_004438427.1:19163088-19163982
ATGATAACCATCCAACTCTGTCTTCTTCATGATCATCTATATGCTCAAGCTCTTGACAATAATTATGCAGAGCAGCTTAA
CTGTTGTAGTGCTGGGCACAGAGTGGGTAAGTTTCCAAAGGCTGTCACCTGTGGAAATGATTCTCACCGGCCTGGGTGTC
TGCTGCTTCTGTCAACTGTGGTCATCAATGCTGTACAACTTTTGCTCCCACTTCTACCCTAGTTACGAATTTTGGTACTT
CAGTATCGTCTGGGAATTTACTAACATTCTTTCATTCTGGTTGACCAGCATGTTTGCTGTCTTCTACTGTGTCAAAGTCT
CCTCCTCAGCCACCCCATCTTCTGGCTGAAGTGGAGAATTGTGAGGTTGGTTCCTTGGCTGTTGCTGGGTTCTCTGCTGA
CTTCTTGTGTGTCTATCATCTTTGCAGCTGTTGGGCATTACAGCAAGATTCAACTAATCTCCATGAGGCATTTCCCTAGA
AACAGCACCATGACTGAGAGACTTGAGATATTCCTGTGGGATTCTTCCATGTGTCACAAGTGGTTGTGTTGATTATTCCT
TTCCTCCTGTTCCTGGCCTCCACCGTCTTGCTCATGGCCTTATTATTCCAACACCTGAGGCAGATGAAAGATCATCACAC
CAGCCACTCTCCAGCCTGAAAGCTCACTCTACTGCCCTGAGGTCTCTTGCTGTCTTCCTCATTTTCTTCACCTCTTATTT
TCTGACCCTAATAATCTCCATGTGGGGTGTCCTTTTTAATAAGGGGTCCTGGTTCTGGGCCTGGGAAGCTATCATCTGTG
CTCTGGTCTCTATTCATTTGACTTCACGGATGCTGAGCAGCCCTAAACTGAAAAGGGTTTTAAAGGTAAAGTGCTGGGAC
CTAGAGGCTGCCTGA
>OrorTAS2R38P_NW_004438464.1:2374863-2375864
ATGGTGACTCTGACTGCCACTGTAACTGTGCCCTATGAAGTCAGGAATGCATTTCTGTTCTTTTCAGTCCTGGAGTTTGC
AGTAGGGATCCTGGTCAATGCCTTCATTTTCTTGATGAATTTTTGGGTCGTGGTGAGGAGGTGGCCACTGAGCAACTGTG
ATCTTGTCCTGCTGAATCTCAGCCTCACCTGGCTTTTCCTGCACGGGCTGCTCTTTCTGGATGCCATCCAGCTTACCCAC
TTCCAGTGGGTAAAAGACCCGCTGGGCCTCTGCTACCAGACCACCCTCATGCTCTGGATGCTCGTAAATCAAGCTGGCCT
CTGGCTCACCACTTGCCTTGGTCTCCTCTACTGCTCCAGGACTGTCCATTTCTTTCACACCTTCCTCCTCCGCTTGGCAA
GCTGGATCTCCAGGAAGATCCCCCAGATGCTCCTGGGTGCTACTTTTTCCTCCTGTGTCTGCACTGTTCTCTATTTGTGG

GACTTTTTCAATAGATCTCACTTCTCAGTTGCAACCATGCTACTCATGAATAACAATACTCAATTGAGAAACTGAGAAAA
CTCAATTTCTATCATTCCTTCCTCTTCTGCAGCCTGGGGTCCACCCCTTCTTTCTTGCTTTTTCTGGTTTCTTCTGGGGT
GCTGATTGTCTCCCTGGGGAGGCACATGAGGACAAGGAGGGCCAAAACCAGAGACTCTCGGGACCCCAGCCTGGAGGCCC
ACATCAAAGCACTCGGGTCTCGTCTCTTTCTTCTACCTGTATATGGTATCTTTCTGCGCTGGCTTCATCTCGGTGCCTTT
GCTGATGCTGTGGCACAACAAGATCGGGGTCATGGTCTGTGCAGGGATACTGGCAGCCTGCCCCTCGGGGCACACAGTCA
TCCTGATCTCAGGCAATGCCAAGCTGAAGAGAGCCGTGGAGACCATTCTGCTCCGGGCTCAGAGCAGCCTAAAGGTAAGG
GCGGACCGCAAGGCAGATCCCAGGATGCCAGATCTATGTTGA
>OrorTAS2R39P_NW_004438464.1:1778529-1777523
ATGCTAGGGAGACATTTTCCTCCAGACACCAAAGAGAAGCAACAACTCAGGATGACTGAAACCTGCAATCCCCCAGAAAA
TCAACTGTCACCATCTCGCATCATTTTGATGTGAATCGTTATAGGCACCGAATGCGTCCCTCGTATCACTGCAAATGGGT
TCATTGTGGCTATAAATACAGCAGGATGGATTCACAACAAGGCAGTTTCCACAAGTGGCAAGATCCTGCTTCTCCTGAGC
GTATCCAGAAGAGTGCTACAAAGCTTCATGATGCTAGAACTCACCTTCAGTTCAACATCCCCACACTTTTATAATCAAGA
CATTCATCGTATATGATACGTTCAAAGGAAGTTTCATGTTCTTAAATGATTGTAGCCTCTGGTTTGCTGCCTGGCTTAGA
TTCTTCTACTTCGTGAAGATGGCGGATTTCTCTTACCCCCTTTTCCTCAAGCTGAAGTAGAGAATTTCTGGATGGATGCC
CTGGTTTCTGTGACTATCAGTGTTTGTTTCCTTGGGCCACAGTGTGTTCTTCCTCAAAAACATCTACACTATGCATTGCA
ACCATCCTTTTTCTAGCCCCTCCTTCAACTCCACTAAGAAAAATTACTTCACTGAGACCAACGTGATCAGCCTGGTTCTT
TTCTTTAACATGGGAATCTTTGTTCCTCTGATCACGTTCATCCTGCCTGCCACCCTGCTGATCATCTCTCTAAAGAGACA
CACCCTACACATGGAAAGCAATGCCACTGGTTCCAGGGACCCCAGCATGGAGGCTCATGTGGGGACCATCAAAGCTATCA
GCTATTTTCTCATTTTCTAAATTTTCAATGCAGATGCTCTATTTCTTTCCATGTCCGACATCTTTGATATCAATAGTTCC
TAGAATACTTTGTGCAAAATCATCATGGCTGCCTATCCTGCTGGCCACTCCATTCTACTGATACAGGACAACCCTGGGTT
GAGAAAAGCCTGGAAGCGGCTTCAGGCTTGCGTTCACCTTTACTTAA
>OrorTAS2R60P_NW_004438464.1:1563119-1562185
ATGGTTCCAGGACCTCAGTTGGCTGATAAGATAGCCTTTATCTTTGCTATCATTTTATTCCTTTTGTGCTTGGTGGCAGT
GGTGGGTAATGGCTTAATCACCATGGCACTGGGCATGGAGTGGTTGCTGCAGAGAACTTTGTCACCCTGCAATAAGTTAT
TGGTCAGCCTGGGAGCCTCTAGCTTCTATCTGTGATGGGTGGTGATAAGAACATTTATATTTCCTGAATCCAATAGCCT
TCCCATACAACCCTGTATTGCAGTTCCTAGCCTTTCAGTGGGACTTCTTGAATGCTGTCACGTTATGGTTCTCCACCTGG
CTCAGTGTCTTCTACGGTGTGAAAATCGCCATCTTCACCCACCCTGTCTTCCTCTGGCTAAAGCAGATGGTGTCTGCGTT
GGTTCCATGGGTGCTGCTCAGCTCCGTGAGGTTCTCCAGCTTTAGCACCATTCTAGTTTTCATAGGCAACCAGAGAATAG
ATCAGAACTATTTAGAGAGGGTTCTGCAACCTTGGAATGTCGCTGGGAATGCTGTGAGAACATATGAGAGACTCTGCTTC
TTCCCTTTGAAAATTGTTACCTGGACAGTCCCTACTGTTGTCTTCATCGCTGGCATGGCTTTGCTCATTCCACCTCTGGG
AAGACACACCAAGCAGGTCTCCCTGTCCATCTCAGGCTCTCACGATCCCAGCGCCCAGGCACACATCAAGGCTCTCCTGG
CTCTCATCTCCTTTGCTGTCCTCTTTGTTTCCTATTTTCTGTCACAGGTGCTCAGTGCCTCAGGTGTGTTTCCATCACGG
GAATTCAGGCACTGGGTGTGGCAGGCTGTGATTTATCTGTGCACAGTAGTCCACCGCATTGTTCTTTTCTTGAGTAACAG
CAGGCTGAGAGCTGTGCTAGAGAGGGGCTGCTCCTCAGGGCATGGGGCATCTTGA
>OrorTAS2R62AP_NW_004438464.1:1568960-1568035
ATGCCCTCCTCACCCATGTTGATCTTCATGGTCATCTTTTTCCTGGAGTTGCTGGCTGCCATGCTGCAGAATGGCTTCAT
AGTTACTGTGCTGATCAGGGAGTGGGTACAATGCCAGACACTGCTTGCAGGCGACATGATTGCGGCGGCCTCCCTGGCCG
CCTCCCGGTTCTGACTGCATGGGATGGCCCTCCTGAACAACCTCGTGGCCTTCTTTGGTTTTGGTTCCAGAATTTACTAT
TTCAGCATCCCCTAGGACTTCATCAACTCTCTCACTTTCTGGCTTACTGCTTGGCTTGCTACATTCTACTGTGTGAAGAT
CTCATTCTTCTCTCACCCCATCTTCTTTGGGCTGAAGTGGAGGATTTCTCGGTCAGTGCCCAGGCTGCTGCTGGGCTCCC
TGATCTTATCTGCTCTGGTAGCCATCCCGTTAGACACTGGGAACACAATTCGTGTGCGGATGGTTGCTGCCCAGAGTTCC
CATGGAAACAGCACCCTGGCTGGTAGAACACAGACTGTCTCTTTGTACTTTTTTCTACCTCATGTAATTATTATGCGGTC
AATTCCATTTCTCCTGTCCCTGGTGTCCACCCTCTCGTGTTCTCGCTGCGCCGGCATTTGGGGCAGATGAGGGACCATAG
ACCTGGCCCGAGTGACCCCAGCACCCGGGCTCACACCGTGGCCCTGAAGTCACTTGCCTTCTTCCTCACCTTCTACCATC

ACGTTACCTGTGCCTGAATATCGTTGTTATAAACATCCTAACCCTCTGGAATCACTGGCGCTGGGCCTGGGAAGTGGTGA
CCTGTGCAGGCATCTGTCGGCACTCCAGCATCTCGGTGCACAGCAGCCCCAGGCTGAGAAAGGCCCTGATGAAGAGGCCT
TGGAGAGCCCTGGGCAAGGAGCAGTTTGTCTCATCAGTGTCAGTAA
>射rorTAS2R62BP_NW_004438555.1:961117-961971
ACGTGGACCTGCACGGTCACCTTTCTCCTGGAGTCGGTGGCTGCCAGGCTGCAGAACGGCCTCACAGTCGTCGTGCTGAG
CCGGACTGGGACGCTGGACGCTGCCAGACGCTGTGCGCAGGCGACGTGATTGTGGCCCGCCTGGCCGTCTCCCAGTTCTG
TCTGCAAGGGATGGCCCTCCAGCGCAACCTCCTGGCTTCCTTTGGTTCTGGTTCCCAATTTTATTTCAGCATCTCCTGGA
GCTTCATCAACACTCTCACTTTCTGGCCGACCAGCTGGCTTGCTGTCTTCTACCGTGTGAAGGTAGCATCCTTCTCTCAC
CCCATCTTCTTCTGGCTGAAGTGCAGGATTTCTCGGTCAGTGCCCGGGCTGCTGCTGGGCTCCCTGATCCTGTCTGCTGA
CATCATCAGCAGCCACCGGGAAGTCAATTCTTGTGCAGATGGTTGCCACCCAGGGTTCCCATGGCAACGACACCCTCACG
CAATTATCATGCGGTCAGTTCCATTCCTCCTGTTCCTGGTGTCCACCCTCTCGCTCGTGTTCTCCCTGCACCGGCACTTG
GGGCAGATGAGGGACCACAGACCCGGCCCGAGTGATCCCAGCACCAGGGCTCACACCGTGGCCCGGAAGTCACTTGCCTT
CTTTTTCATCCTATTTCCTGTGCCTGAGAATTGTCCTTGTGAACATCCCACCCCTCCGGAAGCACCGGCACTGGGAAGCG
GTGACCTACGCCGGCATCTGTCTGCACGCCAGCATCTTGGAGCACGGCAGCCCCAAGCCGAGAAGGGCCCCAAAGAAGAG
GCTTCGGCGAGCCCTGGGCAAGGAGCAGTTTGTCTTGAGTTACCGGTATCAATGA
>MyluTAS2R1_GL429920:2510928-2510029
ATGTTAGAGTTGTACAGGATTGCCCATCTTATTTTTTCAGTGATTCAGTTTCTTGTTGGGGTTCTAGCCAATGGCTTCAT
TGTGGTTGTGAACTGCACAGACTTGATCAGGCAGAGAAAGATGGTGCCCTTCGACCTCCTCCTGTGCTGCCTGGCGACTT
CCAGGATTGGTCTCCAGATGGTCATCATCTTCATTAATCTGGCTGTTCTTTCCTTGATTGAATTCTCTCCAGTTCCTGGG
AATATTATAATTTTCATGTATGTACATGCATCGGAACTTTGGTTGGCCACGTGGCTCAGCGTTTTCTACTGTGCCAAGAT
CGCCACCATCGCTAACCCGCTCTTCTTTTGGTTGAAGTTGAGGATCTCCAAGTTGGTGCCGTGGCTGATTTGTGGGACCT
TGACATATACCTTTCTCACTTCTGTCTTCCACAGAAAACATGCATGGATTATTTCCCAAAAATCCTGGTTGGGCCTTTTC
TCCCAAAATGCAACAACTCAAATTGAAGACATATCTGCATTACAATATGCCCTTCTTTTAATTGAGTTCGTATTGCCCTT
ATTTATTTTCCTTATCTCTGCTTTTCTGTTGATATTTTCCCTGGGGAGGCACACCCAGCAGATGAGGAGCACTGAGATGG
GCACCAGGCACCCTGGCATGAGTGTCTACATCAGCGCACTCCTATCCATCCTGTCCTTCCTGATCCTCTACCTCTCCCAG
TATATGATGGCTGCATTAGGTTTTACTCAAATTTTCAAGATTAAAAACACCATCACTCTGTTGGGCATCTTGCTGTTTGG
TTCATACCCCTCTGTACACTCTGTTATCTTAATTTTAGGAAATCCTAAGCTGAAACAAAATGCAAAGAAGTTCCTCCTCT
ACAGTAAGTGCTGTCAGTGA
>MyluTAS2R2_GL429782:8762423-8763334
ATGACCTCCTCTTTGTCAGCTATTCCTCATGCTATCATCTTGTCAGCAGAATTTATTACAGGGATTACAGGCAATGGATT
TCTGATAATCATCAGCTGTAACGAATTGATCAAAAGCAGAAAGCTAACACCAATGCAGCTCATTTTAATATGTATAGGGA
TGTCTAGAGTCGGTCTGCTGCTGATGTTAATGGTACAAAGTTTTTTCTCTATGTTCTTTCCACTCTTTTATCGGACAAAA
ATTTATGGTGCAGCGATGGTGTTCCTTTGGATGTTTTTTAGCTCTGTCAGTCTCTGGTTTGCCACCTGCCTTTCTGTATT
TTACTGCCTCAAGTTAATAGGCTTCACTCATCCCTGTTTTCTTTGGCTGAAATTCAGGATCTCAAAGTTAATGTCTGGGC
TGCTTCTGGGAAGCTTGCTGGCCTCGGTGAGCACTGCAACTCTGTGTATCGAGGTAGATTACCCTAAAACCGCGGCGGAG
GATGTCCTCAGAAATGCCACACGCACGGCGTCTAAATTCAAGCTAAGGAGTATTAATGAAGTGCTTCTTGTCAATTTTTC
ACTCCTATTTCCTCTAGCCATATTCCTGATGTGCACTTTTATGTTACTCATGTCTCTCTACAAGCACACTCACCGGATGC
AAAACGGATCTCGTGGTTTTAGAAGTGTCAGCACAGAAGTCCATATAAACGCCTTAAGAACAGTGCTAACGTTCGTTCTT
TTCTTTATTTCTTATTTGCCGCCTTCATAACAAACATGACATTCATTATTCCTCATGGAACGCAGCGCTACTTTGTGCT
GAAGGACATAATGGCAGCATATCCCTCTGGCCACTCAGTTATAATAATCTGGAGTAATTCTAAATTCCAGCAACTACTCA
GGAGACTTTTCTGCCTCAAAAGGAGTCAATGA
>MyluTAS2R3_GL429908:1173073-1174023
ATGTCAGGACTCGCCAAGTGGGTGGTTCTGTTTCTTTCTGTCACTCTGTTCCTTCTGGGAATGCTGGGGAATGGCTTCAT
TGTGCTGGTCAATGGCAGCAGCTGGGTCAAGAGCAAGAGAATCTCTTTGTGTGACTTCATCATCACTAACCTGGCTCTCT

CCCGGATTGTTCAGCTGTGTATTCTTTTTTCTGATTTTGTAACAATGATATTCTTTTCTAAAATATTAAATAATGTAGTA
TTCATACAACTTGCTGATATTTTCTGGACATTTACAAACCATCTGAGCATTTGGCTTGCCACCTGTCTCGGTGTCTTCTA
CTGCCTGAAAATCGCCAATTTCTCCCACCCCACATTCCTCTGGCTCAAGTGGAAAGTTGCCAGGGTGGTCGTATGGATGC
TGTTGTGTGGGCTGCTCTTATCGTGTGGTAATGCCATGTCTCTGATTCCTCAGTTTAAGATGTATTATGTTCTCTGTGGA
GCTGATGACTCAGGGAATGTGACTGAGCACTTTAGAGAGCTAAAGAATGAATATGAGGTGATCCATGTTCTTGGGACACT
GTGGAACCTCCTTCCCCTAATTGTGTGTTTGGCCTCCTACATCCTGCTCATCCTCTCCCTGGGGAGGCACACGCGGCAGA
TGCAGCAGAACAGAACCAGCCCCAGCGATCCAAGCACTGAGGCCCACAAGAGGGCCATCAAAATGGTCCTCTCCTTCCTC
TTTCTCCTCCTGCTTTACTTTCTTGCCTATTTACTCACATCGTCCCATTATTTCCTATCAGGAACTGTGTTGACTAAGAT
GATTTCAGAAGTAAGTGCAATGTTTTATCCTGCCTGCCACTCGTTTGTTCTCATTCTGGGAAACAGTAAGCTGAAGCAGA
CGTTTGTGGAGCTGCTCTGGTGTAAGTCTGGTCATCTGAAGCCTGGATCCAAAGAACGCTTTTCCCCATAA
>MyluTAS2R4_GL429908:1183934-1184836
ATGTTCCAAATATTCTCTTTCTCTGTTTTTACTATCTCAGTAGTTTTGGATTTTGTAGGACTCATTGTGAATCTGTTTAT
TGCAGTGATCAATTACAAGACTTGGGTCCAAAGCCACGGAATGTCCTCTTCGGATAGGATCCTGTTCAGCTTAGGCATCA
CCAGATTTCTCATGATGGGAATGTCCCTGGTGAACATCTTCTGCTTCTTCATCTCTCCAAATGTTGAAAGGTTAGTTTAC
TTACCCAAATTTTTCCTGTTGTTTTGGATGTTTTTGGACTCCAGTAGCCTCTGGTTTGTAACTTTGCTCAATGTCTTGTA
CTGTGTGACGATTGCTAACTTGAAATACTCAATTTTTCTTCTGCTGAAACGAAATCTCTCCCCAAAGACCCCCAGGCTAT
TGCTGGCCTGTGTGTTGCTTTCTGCCTTCACCATGCTTCTGTATATTGTGCTCAGACAGAAGCTACTCTTTCCTGAATTT
GTGACGACGGAGAGAAATGGCACAGAATTTAGCGCCGATGAGGGCACCTTGTCAGTGGTGTTCTCTTTGTTCTTGAACTC
ATTTCTCCAGTTCATCATTAATGTGACTTCTGCTTCCTTGTTAATAAACTCCTTGAGGAGACATATACAGAAGATGCAGA
GAAACGCCACTGGCTTTTGGAATCCCCAGACTGAAGCTCATGTGGGTGCTATAAAGCTGATGGCTTATTTCCTCCTCCTC
TACATTCCGTATACAGTTGCCACCCTGTTCCAATACCTCCTTTCTAAAGAGATGGATTTGGGAACCAGATCCGTATGTAT
AATAATTTCCACCTTTTACATTCCAGGACATTCTGTTCTCATTGTTCTCACACATCCTAAACTTAAAAGCAAAGCAAAGA
AGATTATTTGCTTCAACAAATAG
>MyluTAS2R5_GL429908:1195036-1195926
ATGCATATTGCCACCCTAGGACTGCTGATGGTGGTGGCAGTGACTGAATTTCTCATTGGCCTGGTTGGAAATGGAGTCCT
TGTGGTCTGGAGTTTTGTAGAATGGGTAAGAAAACTCAAGGAGTCCTCCTACAACCTCATTGTCCTGGGCCTGGCTGGCT
GCCGACTTCTCCTGCAGTGCCTGATTATGGTGGACCTAATACTGTTTTCGATTTTCAAGAGCTGCATCTGGTTTCGCTAT
ATCAGTGTCTTCTGGGTTGTGGTCAGCCAGGCCAGCCTGTGGTTTGCCACTTTCCTCAGTGTCTTCTACTGCAAGAAGAT
CACGACCTTTGAACACCCTGTCTGCCTATGGCTAAAGCAGAGGGCCTATAGCCTGAGTGCCTGGTGTCTTCTGGGGTGCC
TCCTGATCAATCTGCTAATTATAGCCGATGTTGGCTTAAAGCCCCACAGTCCTTTCCAAGGAAACAGCAGCATTCTGTAC
TCCTTTTCAGACTTGCAGTATCTGTATATATTACAGCTCAATGCAGGATGTGGGTTTCCTTTCTCGGTGTTTCTAATTTC
CTCTGGGATGTTAATTGTCTCTCTGTATAGACACCATAAGAAGATGAAGGTCCATACAGCTGGCCGGAATGATGCTCGAG
CCAAGGCTCACATCACTGTCCTGAAGTCCTTGGTCTGCTTCCTTATACTTTACTTGGTTTACATCGTGGCCAGCCCCTTC
TCTATCAAATCTAAGACTTCTCCTGTTGATCTCACCACTGTCTTCATCTCGGAGACAGTCATGGCTGCCTATCCTTCTCT
TCATTCTGTCATATTGATCATGGGGAATCCCAGGATAAAGCAGGCTTGTCAGAGAATCCTGTGGAAGACAGTGCGCGCTT
GGAAATCCTGA
>MyluTAS2R7_GL429777:10747986-10747048
ATGTCAGATGAAGTAATCAACACCTTAATGATCATAACAGTTGGGGAGTTTTCAGTGGGGATCTTAGGAAATGCATTTAT
TGTATTGGTAAACTTCATGGACTGGATGAAGAATAAGAAGATTGCTTCCATTGATTTAATCCTCACAAGTCTGGCCATAT
CCAGAATTTGTCTAATGTGTATAATAACGTTAGATGGTTTTATGTTGGTGTTGGATCCAGATGTCTATGCCACTGGTAAG
CAAATGAGAATTATTGACTTCTTCTGGACACTAACCAACCATTTAAGTATCTGGTTTGCCACCTGCCTCAGCATTTTCTA
TTTCTTCAAGATAGCTAACTTCTTCCACCCTCTTTTCCTCTGGATGAAATGGAGAATTGAAAGGGCGATTCCTGGGATCC
TGCTGGTGTGCGTGGCCTTCGCTGTGTTCATTAGCCTTCCTGCCGCTGGCGATTTGAATGATGACTTCAGGCTTTGTGTC
AGGGCGAAGTGGAAAACAAACTTAACTTTGAAATGCAGGATAAATAAAGCTCAATATGCTTCCAGCAAGGTACATCTCAA

CCTGTTCACGCTGTTCCCCTTTTCTGTGTCCCTCATCTCATTCCTCCTCTTGATCCTCTCCCTGTGGAGACACATCAGGC
GGATGCAGCTCAATGCCACAGGGTGCAGAGACCCCAGCACAGATGCCCACATGGGAGCCATGAAAGCTGTCATCTCCTTC
CTCCTCCTTTTCATTGCCTACTATTTGTCCTTTCTCATAGCCACCTCCAGTTACTTCATGCCAGAGACTGAATTAGCTGT
GCTGATTGGTGAACTGATAGCTCTAATCTATCCTTCCAGCCATTCGTTTATATTAATTCTGGGGAACAAGAAATTAAGAC
AAGCATCTCTAAGGGTGCTATATAAAGTAACACATACACTAAAAAGAAGAAATTTCTAA
>MyluTAS2R10_GL429777:10758721-10757822
ATGCTAAGTATAGCAGAAGGCCTCCTCATTTTTATAGCAGTTGGTGAATCAATACTGGGGGTTTTAGGGAATGGATTTAT
TGGACTTGTAAACTGCATTGACTGTGTCAAGAACAAGAAGTTTTCTGTAATTGACTTGATTCTCATTGGCTTAGCTACTT
CGAGAATTTTTCTGATATGGATAATAATTACAGATGGATTTATAAAGATACTCTCTCCATATATGTACTCCTCTGGAAAC
CTAAATGAATATATTAGTTATTCATGGATAATTATAAATCACTTAAGTATCTGGTTTGTCTCCAGTCTCAGCATCTTCTA
TTTCCTGAAGATAGCCAATTTTTCCCACCACATTTTCCTCTGGTTGAAGCATAGAATCAACAGAGTACTTCCTCTTCTGA
TGGGCTTCATGCTTATTTCATGGTTATTTATTTTCCCACAAATTGTTAAGATCATGAATAAATATGAAATAAATAATGGA
AACACAACCCGGCATCGCAACATATCTAAAAGTGAATACATTGCTTACCACATTTTGTTCAATCTGGGAGTCATTTTCCT
CTTTACACTGTGCCTGATTTCATGCCTCTTGTTAATCATTTCTCTTTGGAGACACAACAGGAACATGCAATCGAGTGCCC
GAGGTGTCGGAGACCCCAGCACAGAAGCACATGTGAGAGCAATGAAAGTGTTGATATCTTTTATCATCCTCTTGATCTTG
CATTTTATAGGCATTGCCATAGAAATAGCATGCTTTTCTGTGCCAGAAAACAAATCGCTGTTTATTTTTGGTATGGTCAC
CGCAATCATCTATCCCTGCGGTCACTCATTTCTCCTAATTCTAGGAAACAGCAAGCTAAAGCAGGCTTTCCTGAAGGTCC
TACGGCCTTTCAATAGCTAA
>MyluTAS2R11_GL429777:10771926-10770994
ATGTTGGAGAAAGTTTTCGTGATTATAACAAGTGGGGCATTTTTAATAGGAATTTTAGGGAATGGATTCATTGGACTTAC
AAATTGCATTGCCTGGGCTAGAAATCAGAAGTTATGCTTGGTTGACTTCATTCTCACCAGTTTGGCCTTCACCAGAATCA
GTCTTTTGTGGCTAACAATTGTCAATTTGTTTTCAGTGCTGTCCTATCAGGAAATCCCTGCTACTATGGAAGGACACCTT
ATTTATTCTAGTTTCTGGATACTGGCCTCTCACCTGAGTACTTGGTTAACTACTTGTCTCGCTGTCTTTTATTTCCTGAA
GATCGCCAATTTCTCCTCGCATTTTTTTGTTTGGCTCAAATGGAGAATTAACAAGGTGGTTTTCATGCTTCTGCTGGTAT
CTTTGCCCTTCCTGTTCCTGAGCCTTCCTTTGCCGTATCATTTTGGTATCATCTGGTATCATTTTCCCCCAAAACATGAG
GGAAATATGACTGAGTTATTCAATGTGAGTACAAGTAAAAATTTAGATCAGATTATTATGTTCATGATTGGGTCCCTCCC
TCCTTTCTCCGTTTCCTTCATTTCCTTTTTCCTGTTGCTGCTTTCTTTGTGGAGACACACAAAACACGTTGAGCTCAACA
TTAGGAACTCCAGAGATGCCAGTATGGAGGCCCACACCAGAGCAATGAAAACTGTGTTTTCCTTTCTTGTGCTCTCTGCA
CTGCAGCAATTTGCCTATTTCATGACATTTGGGGGATATTTTTTGCAACAGAACAAGCTGGTTGTGATGCTTGGTTATAT
GATAGGAATTTTATATTCTTCAGGGCACTCATATGTTGTGATTTTTGGAAACAGCCAAATGAGGAAAGCCTTCTTGCGGA
TTCCTTGGCACCTGAAGCGAGGCCTGAAAAGAAAGGTACTCTCGGCTACATAG
>MyluTAS2R16A_GL429840:3972666-3973589
ATGATACCCAACCAACTCACTGTTTTCTTCATGACCATCTATCTGCTTGAGTCCTTGACAATAATTGTGCAGAGCAGCTT
AATTGTTGCGGTGTTGAGCAGGGAGTGGGTGCAGGTCAAAAGGCTGTCACCTGTGGACATGATTCTCATCAGCCTGGGCG
TCTGCCGCTTCTGTCTACAGTGGTCATCAGTGCTGCACAACTTTTGCTCCTATTTCAACCCTGACGATGACCTTTGGTAC
ATAGCAATCATCTGGGAATTTACTAATACTCTTGCATTCTGGTTAACCAGCTTGCTTGCTGTCGTCTACTGCGTCAAAGT
CTCTTCCTTCACCTACGCCATCTTCCTCTGGCTGAGGTGGAGAATTTTGAGGTTGATTCCCCAGCTTTTGCTGGGCTCTC
TGATGATTTCCTGTGTGACAATCATTGTTTCAGCTATTAAACATTACATCAAGAGTCAGTTAATCTTGCAATTACCTAGA
AACATCACGAAGACTGAGACACTTAGGACATTCTTGGAAAAATATTACATAGGTCAGCATCTGGCAATGTTGTTCATTCC
TTTCCTACTGTTCCTGACCTGCACCATCTTGCTCATAGCCTCATTGTGCCAACACTTGAGGCAGATACGACATCACGACA
CTGGCCACAGCAACTCCAGCATGAAAGCTCATGCCACTGCCCTGAGGTTTCTTGCCTTCTTCCTCATTTTCTTCACCTCC
TACTTTTTGACCATAATTATCTCCACTAAATACATACTAAAGCATAAGACTTCCTGGTTCTGGGCCTACGAAACTATCAT
CTATGCTACAGTCTCTATTCATTTAACTTCATTAATGCTGAGTAGCCCTACGTTGAAAAAGGTTTTAAAGGTAAGCTGCT
ATGGCCCAAAGCTGCCTGAGGCTCCAGGTACAGCAAGACCCTAA

>MyluTAS2R16B_GL429840:3958278-3959210
ATGATACCCAACCAACTCACTGTTTTCTTCATGACCATCTATCTGCTTGAGTCCTTGACAATAATTGTGCAGAGCAGCTT
AATTGTTGCGGTGCTGAGCAGAGAGTGGGTGCAGGTTAAAAGGCTGTCACCTGTGGACATGATTCTCATCAGCTTGAGCG
TCTGCCACTTCTGTTTACAGTGGTCATCAGTGCTGCACAACTTTTGCTCCTATTTCAACCCTGACGATGATCTTTGGTAC
ATAACAACCATCTGGGAATTTACTAATACTCTTGCATTCTGGTTAACCAGCTTGCTTGCTGTCGTCTACTGTGTCAAAGT
CTCTTCCTTCACCTACGCCATCTTCCTCTGGCTGAGGTGGAGAATTTTGAGGTTGGTTCCCCGGCTGTTGCTGGGCTCTC
TGATGATTTCCTGTGTGACAATCATTGTTTCAGCTCTTAGAGTTGTCTTCATCAAGAGTCAGTTAATATCCATGATGCAA
TTACCTGGAAACAACACAGAGACTGAGACACTTAGGACATTCCTGGAGAAAAATTACGTACATCAGCTTCTGGCAACGTC
GTTCATTCCTTTCCTACTGTTCCTGACCTCCACCATCTTGCTCATAGCCTCGTTGTGCCAACACTTGAGGCAGATACGAC
ATCACGACACTGGCCACAGCAACTTCAGCATGAAAGCTCATGCCACTGCCCTGAGGTTTCTTGCCTTCTTCCTCATCTTC
TTCACCTCTTACTTTTTGACCATAATCATCTCCACTACCTATAACCTAACGCATAAGAGTTACTGGTTCTGGGCCAGCGA
AACTATCATCTATGCTACAGTCTCTATTCATTTAACTTCACTAATGCTGAGTAGTCCTGCATTGAAAAAGGTTTTAAAGG
TAAGCTGCTGTGTCCCAAAGCTACCTGAGGCTCCAGGTACAACAAGAATCTAA
>MyluTAS2R16C_GL429840:3915183-3916115
ATGATACCCAACCAACTCACTGTTTTCTTCATGACCATCTATGTGCTCGTGTCCTTGACAATAATTGTGCAGAGCAGCAT
AATTGTTGTGGTTCTGAGCAGAGAGTGGGTGCAGGTAAAAAGGCTGTCACCTGTGGACATGATTCTCATCAGCCTGGGCG
TCTGCCGCTTCTGTCTACAGGGGACATCAGTGCTGTACAACTTTTGCTACTATTTCAACCCTGCGGATGACCTTTGGTAC
ATAGCAATCATCTGGGAATTTACTAATACTCTTGCATTCTGGTTAACCAGCTTGCTTGCTGTCGTCTACTGCTTCAAAGT
CTCTTCCTTCGCCTACGCCATCTTCCTCTGGCTGAGGTGGAGAATTTTGAGGTTGGTTCCCCAGCTGTTGCTGGGCTCTC
TAATGATTTCCTGTGTGACAATCATTGTTTCAGCTCTTAGAGTTGTCTTCTTCAAGAGTCAGTTAATCTCCAGGATGCAA
TTACCTGGAAACAACACGGAGACTGAGACACTTAGGACATTCCTGGAGAAAAATTATGTACATCAGCTTCTGGCAACGTC
GTTCATTCCTTTCCTCCTGTTCCTGACCTCCACCATCTTGCTCATAGCCTCATTGTGCCAACACTTGAGGCAGATACGAC
ATCACGACACTGGCCACAGCAACTCCAGCATGAAAGCTCATGCCACTGCCCTGAGGTTTCTTGCCTTCTTCCTCATTTTC
TTCACCTCTTACTTTTTGACCATAATCATCTCCACTACATATTACCTAAGGTATAAGAATTACTGGTTCTGGGCCGGCGA
AACTATTATCTATGCTACAGTCTCTATTCACTTAACTTCACTAATCCTGAGTAGCCCTACGTTGAAAAAGGTATTAAAGG
TAAGGTTCTGTGGCCCAAAGCTGCCTGAGGCTCCAGGTACAGCAATACCATAA
>MyluTAS2R16D_AAPE02071117:1798-2730
ATGATACCCAACCAACTCACTGTTTTCTTCATGACCATCTATCTGCTCGAGTCCTTGACAATAATTGTGCAGAGCAGCAT
AATTGTTGTGGTTCTGAGCAGAGAGTGGGTGCAGGTCAAAAGGCTGTCACCTGTGGACATGATTCTCATCAGCCTGGGCG
TCTGCCGCTTCTGTCTACAGTGGTCATCAGTGCTGCACAACTTTTGCTACTATTTCAACCCTGACAATGACCTTTGGTAC
ATAGGAATCCTCTGGGAATTTACTAATACTCTTGCATTCTGGTTAACCAGCTTGCTTGCTGTCGTCTACTGCGTCAAAGT
CTCTTCCTTCACCTACGCCATCTTCCTCTGGCTGAGGTGGAGAATTTTGAGGTTGGTTCCCTGGCTGTTGCTGGGCTCTC
TGATGATTTCCTGTGTGACAATCATTGTTTCAGCTCTTAGAGTTGTCTTCATCAAGAGTCAGTTAATCTCCAGGATGCAA
TTACCTGGAAATAACACGAAGACTGAGACACTTAGGACGTCCATGGAGAAAAATTACATACATCAACTTCTGGCAACGTC
GTTCATTCCTTTCCTACTGTTCCTGACCTCCACCATCTTGCTCATAGCCTCATTGTGCCAACACTTGAGGCAGAAAAGAT
ATCACGACACTGGCCACAGCAACTCCAGTATGAAAGCTCATGCCACTGCCCTGAGGTTTCTTGCCTTCTTCCTCATCTTC
TTCACCTCTTACTTTTTTACCATAATCATCTCCACTACATATATCCTAACGCATGAGAATTCCTGGTTCTGGGCCTGCGA
AACTATCATCTATGCTACAGTCTCTATTCATTTAACTTCACTAATGCTGAGTAGTCCTGCCTTGAAAAAGGTTTTAAAGG
TAAGCTGCTGTGGCCCAAAGCTGCCTGAGGCTCCGGGTACAACAATACCCTAA
>MyluTAS2R408A_GL429777:10892453-10891524
ATGATGAGTTTATTCCTGAGCATTCTTTCCACACTAGTTATAGCAGGATTTGTTCTAGGAAACTTTGCCAATGGCTTCAT
AGCACTGGTGAACTGCATTGACTGGGTCAAGAGACAAAAGATCTCCTGCGCTGATGGAATTCTCACTGGTCTGGCGGTGT
CCAGAATTGGTTTGCTCTGGGTAATAATATTCCATTGGTATGCAACTCTGTTTAATCCAGCTTTGTATAGTTTAAGAGTA
AGAACTGTTGCTGCTATTGTCTGGGTAGTAAGCAACCATTTTAGCCTCTGGCTTGCTACCAGCCTCAGCATATTTTATTT

GCTCAAGATAGCAAATTTCTCCAGCCTATTTTTTCTTCGCCTAAAATGGAGAGCTAAAAGAGTGGTTTTCATGATACTGT
GGGGGACTTTGGTCTTCTTGGTTTTTCGTCTTGCAGTGTTAAGCATAGATGAAGAAATGAAGATGAGTGAATATAAAGGA
AACATCACTTGGAAGACCAACTTGAGGAACATTATACACCTTTCAAATTTGACTATATTTACGCTCGTAAACTTCATACC
CTTTTCTGTGTCTCTGACAGCTGTTCTGCTGTTAATCCTTTCCCTGTGGAAACATCTCAAGAGGATGCAGCTCAGTGGTA
AAGAAACTCAAGATCTCAGCACCAAGGTCCATGTAAGGGCCATGCAAACTGTGATCTCCTTTCTCTTGCTATTTGCCATT
TACTTTGTGACTCTAATCATCTCAGTTTGGAGTTTCTATAATCCTCAGAATATACCAGTTTTTCTGTGTTTCCAGGTTTT
GGCACTTGTCTATGTTTCAGGCCACTCACTAATTCTGATTTGGGGAAACAAGAAGCTAAATCAGGACTTTCTCTCAGCTT
TATGGCAGGTGAGATGCTGGCTGAAAGAATGGAAACCTTCAACACTATAG
>MyluTAS2R408B_GL429777:10911754-10912671
ATGGCTTTACTACCAGCCATTCTTTCCAGCCTATTCACAATACAATTTGTTCTAGGATATTTTGCCAACGGCTTCATAGC
ACTGGTGAACTGCATTGACTGGGTCAAGAGACAAAAGATCTCCTGCGCTGATGGAATTCTCACTGCTCTGGCTGTCTCCA
GAATTTGTTTGCTCTGTGTATTAGTACTAAATTGGTATGCAACTGTACTTAATCTAGCATTTTATAGTTTAGAAGTAAAA
CTGATTGTTCATATTGCCTGGACGACAAGCCACCATTTTAGTCTGTGGCTTGCTACTAGCCTTAGCATATTTTATTTGCT
CAAGATAGCCAATTTCTCCAGCCTTTTATTTCTTCACCTAAAATGGAGAGCTGAAAGAGTGGTTATCATGATACTGTTGG
GGGCTTTGGTCTTCTTGGTTTTTCATCTTGCCGTGGTAGGCACAGATGAAAAAATGCAGATGAATGAAGATAAAGGAAAC
ATCACTTGGGAGACTAACTTGGGGGACATTATGCACCTTTCAAATCAGACTTTATTCGTGCTTGAAAACTTCATACCCTT
TACTATGTCCCTGACAGCTGTTCTGCTGTTAATCTTTTCCCTGTGGAAACATCTGAAGAACATGCAGCTCAGTGGCAAAG
GAACTGAAGATCCCAGCACCAAGGTCCACGTAAGAGCCATGCAAACTGTGATCTCCTTTCTCTTGCTATTTGTCATTTAT
TTCTTCAGTCAAATCATCCTACTTTGGAATTTGAGTACTCAGCAGAACAATTCGCTTCACATGGTTTGCAAGGTTCTTAG
AATCCTGTATCCGTCAAGCCACTCATTTATTCTGATTTGGGGGAATAAGAAGCTGAGACAGGCCTTTCTGTCATTTCTGT
GGCAGTTGAGGTGCTGGTTGAGGAAAGGGAAATAA
>MyluTAS2R38_GL429908:1276850-1275843
ATGTTGACTCTGACTCCCATCATAACTGTGTCCTATGAAGTCAAGACCGTGTTTCTGGTCCTTTCAGTCCTGGAGTTTGC
AGTGGGGATTCTGGTCAATGTCTTCATTTTCTGGGTGTATTTTCGGGATATGGTGAGGAGGCAGCCCCTGAGCACCTGTG
ATCTTGTCCTGCTGAGTCTCAGCCTCAACCGGCTTTTCCTGCATGGACTGCTGTTTCTGGATGCCATTCAGCTTACTCAC
TCCCAGCGGATGAACGACCTGCTGAGCTTCAGGTACCAAACCATCGTCATGCTCTGGATGATCACAAACCAAGCCGGCCT
CTGGCTCGCCACCTGCCTCAGCCTCCTCTACTGCTCTAAGATCGTCCGTTTCTCTCATGCCTTCCTGCTCTGCTTGGCCA
GCTGGATCTCCAGGAAGATTTCTAGGATGCTCCTGTGTACTGTCCTTTTCACCAGTGTATGCACTATCATCTGTTCTTGG
GACTTTTTTAGTAGATCTCACTTCACAGTCACAACTGTGCTATTCATGAGTAACAATTCAGAATTAAATTTGCAAATTGC
AAACCTCAAGTTCTTTCATTCCTTCCTCTTCTGCAGCGTGGGGTCCATCCCACCTTTCTTGTGTTTTCTGGTGTCTTCTG
GGGTGCTGATTGTCTCCCTGTGCCGCCACATGAGGACAATGAGGGCCAAGACCATGGACTCCTGTGACCCCAGCCTGGAG
GCCCACATCAAAGCACTCAAATCCCTCATCTCCTTTCTCTGCCTCTTTGTGGTGTCATTATGCGCTGCCCTCCTCTCAGT
GCCTTTACTGGTGCTGTGGCACAACAAGATCGGGGCCATGGTCTGTGTGGGGATAATGGCAGCCTGTCCCTCAGGGCACG
CAGCCATCCTGATCTCAGGCAATGCCAAGCTGCGGAGAGCTGTGGACAGCATTCTACTGTGGGTTCAGAGCAGCCGAAGG
GTAACGGCAGACCACAAGGCAGATCCCAGGACACCAGGTCTATGTTGA
>MyluTAS2R39_GL429908:2122391-2123350
ATGACCAACACCTGCAGTCCCCCAGAGGATAATTTGTCACCACTTTATATCATCTTAATTTTCACAGTTATAGGCACGGA
ATGCATCATTGGCATCGCTGCAAATGGGTTCATTGTGGCTATAAATGCAGCTGAGTGGATTCAGAATAAGGCAGTCTCCA
CAAGTGGCAGGATCCTGTGTTTCCTGAGCATATCCAGAATAGCTCTCCAAAGCTTGATGATGCTAGAAATTACTTTCCAC
TCAACATCCCCACAATTTTATTATAAAGATGGTGTATATGATACCTTGAAAGTGAGTTTCGTGTTCTTACATTATTGTAG
CCTCTGGTTTTCTGCCTGGCTCAGTTTCTTCTACTTCGTGAAGATTGCTGATTTCTCCTACCGCCTTTTCCTCAAGCTGA
AGTGGAGAATTACTGGATTGATGCCCTGGCTTCTGTGGCTATCAGTGTTTTTTGCCTTATGCTACAGTATGTTCTTTTCC
TATGGCATATACACTGTTTACTGTAACAATTCTTTTCCTATCCCCTCCTCCAACTCTACTAAGAAAATATTCGTCACTGA
GACCAACGTGGTCAACCTGGTTCTTCTCTATAACCTGGGGATCTTCCTTCCTCTCATCATGTTCATCCTGGCGGCCACCC

TGCTGATCATCTCTCTCAAGAGGCACACCCTGCACATGAAAAGCAATGCCACTGGCTCCAGGGACCCCAGCATGGAGGCC
CATTTGGGGGCCATCAGAGCTATCAGCTACTTTCTCATTCTCTACATTTTCAATGCAATTGCTCTATTAATCTATATGTC
CAACATCTTCAATGCCAACAGTTCCTGGGATATTTTGTGCAAAATTATCATGGCTGCCTATCCTGCTGGTCACTCCATTC
TACTGATTCAGGACAACTCTGGGTTGAAAAGAGCCTGGAAGCGGCTTCAGTCTCAAGTTTATCTTTACCTAAAAAAGTAA
>MyluTAS2R40_GL429908:2140277-2141197
ATGTCCAGGTTTAAAGTGGTCTTCATCTTGGTGGTCTCTGGAATCGAGTGCCTCACTGGCATCGTTGGGAATGGTTTCAT
CACGGCCATCCACGGGACCGAGTGGGCCAGACGCAAAAGACTCCCCGTGGGGGACTGCATTGTGCTGATGCTGAGCTTCT
CCAGGCTCTTGCTGCAGATTTGGATGATGCTGGAGAATGTGTACAGCCTACTATTCCAGGCCACTTACAACCAAAACACA
GTGTATATACCTTTCAAAGTCATCATCCTCTTTCTGAACTACTCCAACCTCTGGCTCGCCGCCTGGCTCACCATCTTCTA
CTGTCTTAAAATTGCAAACTTTACGCACCCTTTGTTCGTCACGATGAAGAGGAAAATCACAGTGCTGATGCCCTGGCTTC
TGAGGCTGTCGCTGCTCATCTCCTTGTGCTTCAGCTTCCCCTTAACTAAAGACATCTTCAATGTGTACGTGAATAGTTCC
ATTCCTATCCCCTCCTGCAACGCCACAGAGAAGACGTACATCGCTGATACCAACGTGGTCAACCTGGTTCTTCTCTATAA
CCTGGGGATCTCCATTCCTCTCGTCATGTTCATCCTGGCAGCCACACTGCTGATCATCTCTCTCAAGAGGCACACCCTGC
ACATGAAAAGCAATGCCACTGGCTCCAGGGACCCCAGCATGGAGGCCCACTTGGGGGCCATCAGAGCTATCAGCTACTTT
CTCATTCTCTACATTTTCAATGCAATTGCTCTATTTCTTTCCATGTCCAATGTCTTCGATGCCTACAGTTTCTGGAATAT
TTTATGCAAATTCATCATGGCCGCCTACCCTGCTGGCCACTCACTGCTGCTGATCGTGGGCAATCCTGGGCTGAGAAGAG
CCTGGAAGCGGTTTCAGCATCGAGTTCATCTTCACCTGTAA
>MyluTAS2R41A_GL429840:4153083-4154009
ATGCAGCCAGCATTCACAGCCCTCTTCATGCTGCTCTTTGTCCTGCTGTGTTTCCTGGGAATCCTGGCCAATGGCTTCAT
TGTGCTGGTGCTGAGCAGAGAATGGAGGCGGCTTGGGAGGCTGCTCCCTTCTGACATGATCCTCATGAGCTTGGGTGCCT
CCCGTTTCTGCCTGCAGTGGATTGGAATGGTGTACAACTTTTACTCCTTCTTCCACCCGGGCGAGTTCAGCAAGGGTCCT
GCACAGGAGCTCTTTGGTCTCCAATGGGAATTCCTGAATTCAGCTACTTTCTGGTTCGGTACCTGGCTCAGTGTCCTCTT
CTGCATGAAGATTGCTAACCTCACCCACCCGACCTTCCTCTGGCTGAAGTGGAGGTTCCCAAGGTCAGTGCCCTGGCTTC
TGCTGGGCTCTCTCCTGATCTCCACTGTCGTCTCCCTGCTCTTCTTCTGGGGAAACTACGCTGTGAATCAAGGTTTCTTC
ATTAGAGAAGTTTATGAGAATATGACCTACATGGAGAGGGTCAATATGATTGAAATTCACTATTTCCTACCCCTCAAATT
GGTCACATTGTCGATTCCTTGCTCTGTTTTTCTGGTTGCAACCTCACTGCTGATTCATTCTTTGAGGAGACACACTCGGA
AAATGCGGCAAAGTGCCCATAGCCTGCAAGACGCCAGCACCGAGGCTCACACCAGAGCTCTGAAGTCACTCATCTTCTTC
CTCATTCTTTACATTCTGTCTTTCATGTCCCTGATCATTGATACTGTAGGTTTCTTTCTCTCAGAGAATGACTGGTTCTG
GCCATGGCAAATTGTAACCTACCTGTGCACATCTCTTCATCCCTTTATCCTCATCCTCAGCAACCTCAGGTTTCGAGAGG
TGCTCAGGCCGCTACTTCTGTTTGCCAGGGGCTTCTGGCTGGTCTAG
>MyluTAS2R41C_GL429908:2388713-2389639
ATGCAGCCAGCATTCACAGCCCTCTTCATGCTGCTCTTTGTCCTGCTGTGTCTCCTGGGAATCCTGGCCAATGGCTTCAT
TGTGCTGGTGCTGAGCAGAGAATGGAGGCGGCTTGGGAGGCTGCTCCCTTCTGACATGATCCTCATTAGCTTGGGTGCCT
CCCGTTTCTGCCTACAGTGGGTTGGAATGGTGCACAACTTTTACTCCTTCTTCCACCTGGAAGAGTTCAGCAAGGGTCCT
GCAGGGCAGCTCATTAGATTCCAATGGGACTTCCTGAATTCAGCCACCTTCTGGTTTGGTACCTGGCTCAGTGTCCTCTT
CTGCATGAAGATTGCTAACCTCACCCATCCTACCTTCCTCTGGCTGAAGTGGAGGTTCCCAGGGTCAGTGCCCTGGCTTC
TGCTGGGCTCTCTCCTGATCTCCACCATCGTCACCCTGTTCTTCTTTTGGGGAGACTACGCTTTGAATCAAGGTTTCTTT
ATTAGAGAAGTTTATGAGAATATGACCTACATGGAGAGGGTCATGAGCATGGAAATTCACTATTTCCTACCCCTCAAATT
TGTCACGTTGTCAATTCCTTGCTCTGTTTTTCTGGTCTCAACTGCATTGTTGATTCATTCTTTGAGGAGACACACTCGGA
CAATGCGGCAAAGTGCCCACAGCCTGCAAGACGCCAGCACCCAAGCTCACACCAGAGCTCTGAAGTCACTCATCTTCTTC
CTCATTCTTTACATTCTGTCTTTCATGTCCCTGATCATTGATACTGTAGGTTTCTTTTTCTCAGAGAATGACTGGTTCTG
GCCATGGCAAATTGTAACCTACCTGTGCACATCTGTCCATCCCTTTATCCTCATCCTCAGCAGCCCCAGGCTTCGAGAGG
TGTTCAGGCAGCTACTTCTGTTGGCCAGGGGCTTCTGGCTGGTCTAG
>MyluTAS2R42_GL429777:10921922-10920927

ATGCTCACTGGATTGGAAATAATCTTTCTGATACTGTCAATAGCAGAATTCATAATTGGAATGTTGGGGAATGTGTTCAT
TGGACTGATAAACTGCTCTGAATGGGTCAAGAACCAAAACATCTCTTTAGCTGACTTCATCTTTACCTGCTTGGCTATCT
CCAGAATTAGTCAGTTGTTGGCATTACTTTTTGAATCACTTATATTGGGACTATTTTCACATGTATTTTCTACTTATAAA
CTAGCAAAATCTATTACTTTACTTTGGAGAATAACTAATCACTTGACTACCTGGCTTGCTACTTGCCTAAGCATTTTCTA
CCTCCTTAAGATAGCTCACTTCTCCCACTCTCTTTTCCTCTGGCTGAAGTGGAGAATGAAGAGAGTGATTCTTGTGATAT
TTGTATTTTCTTTTATCTTTCTGATTTTTGACTTTCTATTGTTAGAAAGCTTTAATGATTTATACTTGAAGGCCTATATG
TATGATAATAGTAATCTGACTTTATATATAGAAGAAAGTAAGACTGTCTATTTTGAAACCCTGATTCTTCTTAGCTTGAC
CTGTTTGCTTCCTATTGTTCTGTCCCTGACCTCATTGCTCCTTTTATTTCTGTCTTTGGTAAGACATATCAGAAATTTGC
AGCTCAACTCCATGGGCTCAAGGGACTCCAGCACAGAGGCCCATAAAAGGGCCATAAAAATGGTGATGTCTTTTCTCTTT
CTCTTCATATTTCATTTTTTTTCCACACAAGTGGTAAATTGGATATTTCTTATGTTTCCATATTACATGACTGCAAAATT
TGTCATGTTATTAGTCTATGTCTTTCCCTCAAGTCACTCATTTCTTTTGATTCTGGGAAACAGCAAGCTAAGACAGACAG
CCTTGAAGATACTCTGGCATCTCAAAAGCTCCTTGAAAAGGGAAAATCTGTTACACCTTTACAGACAGATTTCCCAGAGT
CTTTTCAAAGATAATAACTTAACGAGGAAACTTTGA
>MyluTAS2R408C_GL429777:10901122-10900205
ATGGCTTTACTACCAGCCATTCTTTCCAGCCTATTCACAATACAATTTGTTCTAGGATATTTTGCCAATGGCTTCATAGC
ACTGGTGAACTGCATTGACTGGGTCAAGAGACAAAAGATCTCCTGCGCTGATGGAATTCTCACTGCTCTGGCTGTCTCCA
GAATTTGTTTGCTCTGTGTATTAGTACTAAATTGGTATGCAACTGTACTTAATCTAGCATTTTATAGTTTAGATGTAAAA
CTGATTGTTCATATTGTCTGGATGACAAGCCACCATTTTAGTCTGTGGCTTGCTACTAGCCTTAGCATATTTTATTTGCT
CAAGATAGCCAATTTCTCCAGCCTTTTATTTCTTCACCTAAAATGGAGAGCTGAAAGAGTGGTTATCATGATACTGTTGG
GGGCTTTGGTCTTCTTGGTTTTTCATCTTGCAGTGGTAAGCACAGATGAAAAAATGCAGATGAATGAAGATAAAGGAAAC
ATCACTTGGGAGACTAACTTGGGGGACATTATGCACCTTTCAAATCAGACTTTATTCATGCTTGTAAACTTCATACCCTT
TACTATGTCCCTGACAGCTGTTCTGCTGTTAACCTTTTCCATGTGGAAACATCTGAAGAACATGCAGCTTAGTGGCAAAG
GAACTGAAGATCCCAGCACCGAGGTCCACATAAGAGCCATGCAAACTGTGATCTCCTTTCTCTTGGTATTTGTCTTTTTT
TTCTTCACTCAAGTCATCTCAGTTTGGAATTCGAGTACTCAGCAGAACAATTTAGTTCACATGGTTTGCAAGGTTCTTGG
AATCCTGTATCCGTCAAGCCACTCATTTATTCTGATTTGGGGGAATAAGAAGCTGAGACAGGCCTTTCTGTCATTTCTGT
GGCAGTTGAGGTGCTGGTTGAGGAAAGAGAAATAA
>MyluTAS2R408D_GL429777:10858475-10857558
ATGAAACACTTATTACTGAGCATTCTTTCCATCCTTATAATAGCTCAATATGTTCTAGGAAGTTTTGCCAATGGCTTCAT
AGCACTGGTGAACTGCATTGACTGGATCAAGAAACACAAGATCTCCTGTGCTGATCGAATTCTCACTGCTCTGGCTGTCT
CTAGAATTTGTTTGCTCTGGATAATAGTATTCAATTGGTATGGAACTATGTTGCATCCAGCAATCTATAGTTTAGAAGTA
AGAACTATTGTTCATATTGCATGGGTAGCAAGCAACCATTTTAGTCTCTGGCTTGCTACTAGCCTCAGCATACTTTATTT
GCTCAAGATAGCCAATTTCTCCAGCCTTTTATTTCTTCACCTAAAATGGAGAGCTGAAAGAGTGGTTATCATGATACTGT
GGGGGACTTCGGTCTTCTTGGTTTTTCATCTTGCAGTGGTAAGCACAGAAGAAAAAATGCAGATGAATGAAGATAAAGGA
AACATCACCTGGGAGAGCACATTGAGGGACATCGCACACCTTTCAAGTGGGATTATATTCGTGCTTGTACACTTCATACC
CTTCGCTATGTCCCTGACAGCTGTTCTGCTGCTAATCTTTTCCATGTGGAAACATCTGAAGAAGGTGCAGGTCAGTGGCA
AAGGATCCCAAGATCCCAGCACCGAGGTCCACATACGAGCCATGCAAACTGTGATCTCCTTTCTCTTGCTATTTGTCATT
TACTTCTTCGCTCAAATCATCTCATTTTGGAATTTTACTATTACGCAGAACAATTTAGTTCCCTTCGTTTGCCAAGTTTT
TGGAATCCTGTATCCATCGAGCCACTCATTTATCCTGATTTGGGGAAATAAGAAGCTGAGACAGGCCTTTCTGTCACTTC
TGAGGCAGTTGAGGTGCTGGCTGAAGGAAAGGAAATAA
>MyluTAS2R18A_GL429777:10854388-10853459
ATGTCATTTGAAATGAAGGCCTCCTTTCTGGTTGTGGCAACAGGAATATTCATTTTAGGAGTGCTAGGAAATGGATTCAT
CGGACTGGTGAACTGCATCGAATGGTTAAGGACTGGGAAAGTTTCCTCAGCTGATTTCATCCTCACCAGCCTGGCTCTGG
CCAGAATCATTCAACTGTTGGTAATACTCTTGGATTCATTTATAATGGGGCTAGCTCCACATCTGTATGCTACTGGTAAA
CTAGCAAAGGTCGTTAGTATTCTTTGGGCACTAACTAACCACCTAACTATCTGGTTTGCCACCTGCCTCAGCATTTTCTA

CTTCCTTAAGATCGCCAGTTTCTCCCACTCCTTTTTCATGTGGCTGAAGTGGAGAGTCAACAGGGTGGTTCTTTTGCTTT
TCCTGGGGTCTTTCTTCCTACTGTCTCTTAACCTCTTAATGCATGATGCTGTTAATGATCTGAATACTTACAGGGTACAT
GGAATAAATTTGACTTTGCAGTTAGAGGCAAATGAAATGTTCTATCTCAAAAGTCTTCTTCTTAGTTTGACCTATGTTAT
CCCCTTTTTCCTGTCCCTGATTTCTTTGCTTCTTTTATTTCTGTCCTTGGTGAGACACACCAAGAATTTTCAGCTCAACT
TGACGGGCTCGGTGGACTCAAGCACAGAGGCCCATAGAAGGGCCATGAAAATGGTGACAACGTTCCTCCTCCTCTTCATC
ATTTACATTATTTCTATTCTAACAGCAACTTGGATCTTCACTAAGGTAGAGACCTATCAAGTCAAGATGCTTTTCTTAGT
GATTTCAACTACCTTTCCCTCAGGTCACTCTTTTCTTATTATTTTTGGAAACAGCAAGCTAAGACAGATCGCCTTGAGGC
CACTGTGGCACTTAAATTCTCTGAGAAAAGGAAAACCTTTGCCTTTATAG
>MyluTAS2R18B_GL429777:10845245-10844316
ATGTCATTTGAAATGAAGGCCTCCTTTCTGGTTGTGGCAACAGGAATACTCATCTTAGGAGTGCTAGGAAATGGGTTCAT
CGGACTGGTGAACTGCAGCGAGTGGTTCAGGACTGGGAAAGTTTCCTCAGCTGATTTCATCCTCACCAGCCTGGCTCTGG
CCAGAATCATTCAACTGTTGGTAATACTCTTGGATTCATTTATAATGGGGCTAGCTCCACATCTGTATGCTACTGGTAAA
CTAGCAAAGGTGGTTACTATTCTTTGGGCACTAACTAATCACCTCACTATCTGGTTTGCCACCTGCCTCAGCATTTTCTA
CTTCCTTAAGATAGCCAATTTCTCCCACTCCTTTTTCATGTGGCTGAAGGGGAGAGTCAACAGAGTGGTTCTTCTGCTTT
TCCTGGGGTCTTTCTTCCTACTGTCTCTCAATATCTTAATGCATGATGCTATTAGTGAATTGTGGTTGAATACGTACAGG
GTACATGAAATAAATATGACTTTGCAGTTAGAGGCAAATGAAATGTTCTATACTAAATGTCTTCTTCCTATTACTTTGAC
CTATATTATCCCCTTTTTCCCGTCTCTGATCTCTTTGCTTCTTTTATTTCTGTCCTTGGTGAGACACACCAAGAATATTC
AGCTCAACCTGACGGGTTTGAGGGACTCAAGCACAGAGGCCCATAGAAGGGCCATGAAAATGGTGACAACGTTCCTCCTC
CTCTTCATCATTAACATTATTTCTATTCTAACTGCAATTTGGATCTTCAATAAGGTAGAGACCTATCAGATCACGATGCT
TGTCACAGTATTGTCAGCTACCTTTCCCTCAGGCCACTCTTTTCTTATAATTTTTGGAAACAGCAAGCTAAGACAGATCG
CCTTGAGATTACTGTGGCACTTACATTCCCTAAGAAAATCACCAGTTTAA
>MyluTAS2R18C_GL429777:10834140-10833202
ATGTCAATTGAAATGAAGGCCTCCATTCTAGTTGTGGCAACAGGAATATTCATCTTAGGAGTGCTAGGAAATGGATTCAT
CGGACTGGTGAACTGCATCGAGTGGGTCAGGACTGGGAAAGTTTCCTCAGCTGATTTCATCCTCACCAGCCTGGCTCTGG
CCAGAATCATTCAACTGTTGGTAATACTCTTGGATTTATTTATAATGGGGCTAGCTCCACATCTGTATGCTACTGGTAAA
CTAGCAAAGGTAGTTAGTATTCTTTGGGCACTAGCTAATCACCTAACTATCTGGTTTGCCACCTGCCTCAGCATTTTCTA
CTTCCTTAAGATCGCCAATTTCTCCCACTCCTTTTTCATGTGGCTGAAGTGGAGAGTCAACAGGGTGGTTCTTCTGCTTT
TCCTGGGGTCTTTCTTCCTACTGTCTCTTAACCTCTTAATGCGTGATGCTATTAATGAATTGTGGTTGAACATCTACAGG
GTACATGAAATAAATATGACTTTGCAGTTAGAGGCAAATGAAATATTCTATCTCAAAAGTCTTCTTCTTCTTAGTTTGAC
CTATGTTATCCCCTTTTTCCTGTCTCTGATCTCTTTGCTTCTTTTATTTCTGTCCTTGGTGAGACACACCAAGAATTTTC
AGCTCAATCTGACGGGTTTGAGGGACTCAAGCACAGAGGCCCATAGAAGGGCCATGAAAGTGGTGACAACGTTCCTCCTC
CTCTTCATCATTTACGTTATTTCTGTTCTAACTGCAACTTGGATCTTCACTAAGGCACAGACATACCAAGTCATGATGTT
TTTCTTAGTGATTTCAACTACCTTTCCCTCAGGCCACTCTTTTCTTATAATTTTTGGAAACAGCAAGCTAAGACAGATCG
CCTTGGAGCCACTGTGGCACTTAAATGTTCTGAGAAAAGCAAAACCTTTGCCTTTATAG
>MyluTAS2R18D_GL429777:10832265-10831336
ATGCCAATTGAAATAAAGGTCTCCATTCTAGTTGTGGCAACAGGAATATTCATCTTAGGAGTGCTAGGAAATGGGTTCAT
CGGACTGGTGAACTGCATCGAGTGGTTCAGGACTGGGAAAGTTTCCTCAGCTGATTTCATCCTCACCAGCTTGGCTCTGG
CCAGAATCATTCAACTGTTGGTAATACTCTTGGATTTATTTATAATGGGGCTAGCTCCACATCTGTATGCTACTGGTAAA
CTAGCAAAGGTGGTTCCTATTCTTTGGGCACTAGCTAATCACCTAACTATCTGGTTTGCCACCTGCCTCAGCATTTTCTA
CTTCCTTAAGATCGCCAATTTCTCCCACTCCTTTTTCATGTGGCTGAAGTGGAGAGTCAACAGGGTGGTTCTTCTGCTTT
TCCTGGGGTCTTTCTTCCTACTGTCTCTTAACCTCTTAATGCATGATGCTATTAATGAATTGTGGTTGAATACGTACAAG
GTACATGAAATAAATATGACTTTGCAGTTAGAGGCAAATGAAATGTTCTATACTAAATGTCTTCTTCCTATTACTTTGAC
CTATATTATCCCCTTTTTCCTGTCTCTGATCTCTTTGCTTCTTTTATTTCTGTCCTTGGAGAGACACACCAAGAATTTTC
AGCTCAATCTGACGGGTTTGAGGGACTCAAGCACAGAGGCCCATAGAAGGGCCATGAAAATGGTGACAACCTTCCTCCTC

CTCTTCATCATTAACATTATTTCTATTCTAACTGCAATTTGGATCTTCAATAAGGTAGAGACCTATCAGATCACGATGCT
TGTCACAGTATTGTCAGCTACCTTTCCCTCAGGCCACTCTTTTCTTATAATTTTTGGAAACAGCAAGCTAAGACAGACCG
CCTTGAGACTACTGTGGTACTTACATTCCCTGAGAAAATCGCAAGTTTAA
>MyluTAS2R18E_GL429777:10800830-10799901
ATGTCAATTGAAATGAAGGCCTCCATTCTAGTTGTGGCAACAGGAATATTCATCTTAGGAGTGCTAGGAAATGGGTTCAT
CGGACTGGTGAACTGCATCGAGTGGGTCAGGACTGGGAAAGTTTCCTCAGCTGATTTCATCCTCACCAGCCTGGCTCTGG
CCAGAATCATTCAACTGTTGGTAATACTCTTGGATTTATTTATAATGGGGCTAGCTCCACATCTGTATGCTACTGGTAAA
CTAGCAAAGGTCGTTAGTATTCTTTGGGCACTAACTAACCACCTAACTATCTGGTTTGCCACCTGCCTCAGCATTTTCTA
CTTCCTTAAGATCGCCAATTTCTCCCACTCCTTTTTCATGTGGCTGAAGTGGAGAGTCAACAGGGTGGTTCTTCTGCTTT
TCCTGGGGTCTTTCTTCCTACTGTCTCTTAACCTCTTAATGCGTGTTGCTGCTAGTGAATTGTGGTTGAACACCTACAGG
GTACATGAAATAAATATGACTTTGCAGTTAGAGGCAAATGAAATGTTTTCTATTAAACATCTTCTTCCTATTACTTTGAC
CTATGTTATCCCCTTTTTCCTGTCTCTGATCTCTTTGCTTCTTTTATTTCTGTCCTTGGTGAGACACACCAAGAATTTTC
AGCTCAACTTGACGGGCTCAGTGGACTCAAGCACAGAGGCCCATAGAAGGGCCATGAAAGTGGTGACAACGTTCCTCCTC
CTCTTCATCATTTATGTTATTTCTATTCTAACTGCAACTTGGATCTTCACTAAGGTACAGACATTTCAGATCACGATGCT
TGTCACTGTGTTGTCAGCTACCTTTCCCTCTGGCCACGCTTTTCTTATAATTTTTGGAAACAGCAAGCTAAGACAGATCG
CCTTGAGACTACTGTGGCACTTACATTCCCTGAGAAAATCGCAAGTTTAA
>MyluTAS2R18F_GL429777:10786871-10785948
ATGTCCATTGGAATAAAGGTCTCCATTCTAGTTGTGGCAACAGGAATAGTCATTTTAGGAGTGCTAGGAAATGGGTTCAT
CGGACTGGTGAACTGCATCGAATGGTTCAGGACTGGGAAAGTTTCCTCAGCTGATTTCATCCTCACCAGCTTGGCTATGG
CCAGAATCATCCATCTGTTGCTAACACTATTGGATTCATTTATAATAGTGCTGGCTCCACATCTGTATGCTACTGGTAAA
CTAGCAAAGGTGGTTACTATTCTTTGGGCACTAACTAATCAACTGACTATTTGGTTTGCCACCTGCCTCAGCATTTTCTA
CTTCCTTAAGATAGCCAGTTTCTCCCACTCCTTTTTCATGTGGCTGAAGTGGAGAGTCAACAGGGTGGTTCTTCTGCTTT
TCCTGGGGTCTTTCTTCCTACTGTCTCTCAATATCTTAATGCATGATGCTGTTAGTGAATTTTGGTTGAGTACCTACAGG
GTACATGAAATAAATATGACTTTGCAGTTAGAGATAAATGAAATGTTCTATCTCAAAAGTCTTCTTCTTCTTCTTACTTT
GACCTACATTATCCCCTTTTTCCTGTCTCTGATCTCTTTGCTTCTTTTATTTCTGTCCTTGGTGAGACACACCAAGAATT
TTCAGCTCAACCTGACAGGCTCAAGCACAGAGGCCCATAGAAGGGCCATGAAAATGGTGACAGCCTTCCTCCTGCTCTTC
ATCATTTACATTATTTCTATTCTAACAGCATGTTGGATCTTCACTAATTTACAGACATTTCAGGTCAAGATATTTCTCAT
GATGATTTTGATTACCTTTCCCTCAGGCCACTCATTTATTATAATTTTTGGAAACAGCAAGCTAAGACAGATCTCCTTGA
GACTACTCAGGAACTTAAGTTCTCTGAGAAAAGCACAACTTTAA
>MyluTAS2R67_GL429777:10909280-10908351
ATGCCATCTGGAGCTGAAAGTGTTTTTCTGGTAGCTGCAATGGGAGAATTCATAGCTGGAATGTTGGGGAATGGGTTCAT
TGTACTAGTTAATTGCATTGATTGGGTGAAGAGTCAAAAACTCTCAGCAGCTGACTGCATCCTCACCAGCCTGGCTCTCT
CCAGAATCACTCTTTTTTGGATATCACTAGCTGACTCATTTCTAATGGTGTTGTGGCCACAATTTTATGCCATTGGTAAA
CTAGCAAAACTTATTGGCATTCTTTGGATACTGTGCAATCACCTAGCTACCTGGTTTGCCACCTGTCTAAGCATTTTCTA
TTTCTTTAAAATAGCCAATTTCTCCCACCCCTGCTTTGCCTGGCTGAGGTGGAGAACTGGCAAAGTGCTACTTGTGCTTT
TACTGGGGTCTTTGTTCTTCCTGTTTTTGAAACTTGTGTTAATAGACTCATTCAATGGTTTCTGGATTAATGTCTATAAT
ATACATGAAAGAAACTCAACATGGTCTCCAGGTGTAAGTGAAACTCTGTATCTTAACAGTTTGATTGTTTCTAATTTTAT
CTACTTAATTCCCTTTCTTCTGTCCCTGAGTTCACTGCTCCTTTTATCTCTTTCCTTGATGAGACATACCAGGAATGTGC
GGATGAACTCCAGCTCTAACGACTTCCGCACAGAAGCCCATAAAAAGGCCATGAAAATAGTGTTATCTTTCCTCCTCCTC
TTCATACTTCATTTTTCTTCCTTTGTGTTAACAGGTTGGTGTTTCCTTATAGTGCAGAAGCAGCAGGCCAATCTGGCTGT
CATGTTAACATGGAGTATTTTCCCTTCGGGCCACTCATTTATCCTAATTTTGGGAAACAGCAAGCTGAGACAAACTGCAT
TGAAACTATTGTGGCATCTTAACTGCCACCTGAAAAAGGGTGAAACCTAG
>MyluTAS2R12P_GL429777:10777486-10777027
ATGTCAAGCACATGGAGACCCTGTTTATAATCATTCCCGTAGTACAATTCATAATAGGAATTTTGGGGAATAGATTCACT

GCATTGGTAGACAGCATTGACTGGATCAGGAACTGGAAATTCTCCCTGATGTATTTTATTCTCACCTGCTTGGCCATCTC
CAGGGTATGTTTTCTGTGGGTATTGATTTTAGCTCTCTTTTAATGTGGTCAATGAGTAAATATTTTACTCTAAGAACCTA
AAGCTAAGTTTTGACAACCTCTGGACAGGCTCTATTTCTATATGACTGGTACCACCTGCCTAAGTGTTCTCTATTTCCTT
AAGATAATCAACTTTTCTAAGCTAATTTTCCTCTGAATAGAACAGAAAATTCACAAAGTGCCTCTCATTAATACACTGGG
GGCAATACTCTCATTCTGCACATGTCTCCTTTTGAAGGAAATATTAATTAATAGACTGAG
>MyluTAS2R13P_GL429777:10783747-10782653
ATGGTGAGTGCCTTGCATTGCTTCTTCACCATTTCAGTAAACACAGAATTTGTCATTGGTATTTTGGGGAATGGATTCAT
AATACTGTGAACTACATTGACTGATTCAAGAAACGAAAGATCTTCTCAGTTGATGGAATCCTCACTGCTTTGGCGCTCTT
CAGACTTTGTCTGATTTGGGTAATAATGATGAGTTGGTTTGCAAAGGATATTTATCCATCTTCACACATGAATATAATGG
AAGTTGTACTTATTATTCTTGCGGTTGTGGTCAATCATTTTAGTCACTGGTTTGCCACAGGCCTCAGCCTCTTTTATTTT
CTCAAGATAGCCAGTTTCTCAAATCCTGTCTTTCTTTGCCAAGCATAGGTCGAAATGGTAGTTCTGTTAATGTTGCTGAG
AGCATTAGTGTTCTTATCTTTAAATCTTCTATATATATAAAAGCCTATGTGATCAAATGACCGTTTGACCGGTCACTATG
ATGTGCACTGACCACCAGGGGGCAGACACTCAATGTAGGAGCTGCCCCCTGGTGGTCAGTGTGCTCCCACAGTGGGAGCC
TGCTCAGCTGGCTGATGGGCTCACGGCGAGTGCAGCTGTGGCAGTGCGAGCCTTTTCCTCCTTGGCGGCAGTGGAGGTAG
GCGCAGCGGGCTGGATGAGCGGGATTGGTGCAGTGCCCTGCCCAGGCTAGGGCTCCTCCCCAGCTGCCTGCTGCTTCGAA
CACTATTACACCCCCCAGGGGTCCTGGGTTGCCAGAGGGCGCAGGCCAGACTGAGAGACCCCACTGGTGCACAAATCCGT
GTACCAGGTCTCTAGTACTATAATAAGTATGTATATTAATATCCAGATACATCCATGTGAAAGAAATAGGACTTTGAGTT
CTAAAAGGAGCAACACTAAAAACGTTTCTAAACTGATTGTACTCACTCTTGGAGCTCTCATACCCTTTTCCATATCTCTG
AATTTTTTTATCCTGTTCATCTTCTCCCTATGGAAACATCTCAAGAATATAAAGCACAGTGCAATGGGATTCAGAGGTTC
TTGCCTCGAGGTCCATATAAGAGCAATGAAAAGTGTGATATCTTCCCTGTTACTA
>MyluTAS2R45P_GL429777:10884859-10883961
ATGCTAATAATGACAGGATTTATTCTAGGAAGTTTTCCAATGGCTTCATAGCACTGGTGAACTGCATTGACTGGGTCAAG
AAACACAAGATCTCCTGTGCTGAGCGAATTCTCACTGCTCTGGCTGTCTCCAGAATTGGTTTGCTCTGGGTAATAGTATT
AAATTGGTATGGAACTGTGTTTAATCTACCTTTCTATAGCTCAGAAGTAAGTACTACTGTTTACATGGCCTGGATAGTAA
ACCACCATATTTGTCTCTGGCTTGCTACTAGCCTCAGCATCCTTTATTTTCTCAAGATAGCCAATTTCTCCTGTCTTTTA
TTTCTTCACCTAAAATGGAGAGCTGAAAGAGTGGTTCTCATGATACTGTGGGGGAATTTGTTCATCTTGGTTTGTCATCT
TACAGTGCTAAGCATAGATGCAAAAATGCAGAAGAATGATTATGAAGGAAACGTCACTTGGAAGACCAACTTGAGGGACA
TTATGCACCTTTCAAGTATGACTGTATTCACGCTTACAAACTTCATCCCCTTCACTATGTCCCTGACAGCTTTTCTGCTG
CTAATCTTTTCCCTGTGGAAACATCTGAAGAACATGCAGCTCAGTGGCAAAGGAACTGAAGATCCCAGCACCAAGGTCCA
TGTAAGAGCCATGCAAACTGTGATCTCCTTTCTCTTGCTATTTGTCATTTATTTCCTCGCTCAAATCATCGCAATTTGGA
GACCCCAAACTCTGCAGAATAATTCAGTTCTCATGCTTTGTGAGGTTCTTGGAATCATGCATCCTTCAACCCACTCATTG
ATCCTGATTTGGGGGAACAAAAAGTTAAGACAGGCCTTTCTGTCATTTCTGTGGCAGCTGAGGTGCTGGATGAAGAGAAA
TAAGTGGGGCACCTAGTGT
>MyluTAS2R60P_GL429908:2359186-2360053
ATGGTTTCAGGACTTCCAGTGATGGATAAGAGAGCCATCACCTTGGATATCATTTTATTCCTTTTGTGTATGATAGCTTT
GGTGAAGAATGGCTTAATTACTGCAGAACTAGGCATGCAGTGGTTGCTGTGGAGAATATTGTCACCTTGAGATAAATTAT
TGATCAGCCTGGAGCCTCTTGGTTCTGTCTGTAATGGGTGTTGATCAGTGAGAGAATTTGTGGTTTTTCTGTATCCAAAG
TCCTTCCTGTACAATCCTGTACTCCAGTTCCTCGCCTTCCACTGGGACTTCTTGAATGATGCTACCTTATGGTTTTCCTA
CTGGCGCAGTGTTTTCTATCATGTGAAAATTGCAATCCTCACTCACCCTGTCTTCCTCTGCCTAAAGCAGAAGGTGTTTG
GGTTGGGTTGCTCAGCTCTGTGGGGTTCTTCAGCTTGAATGCCATCTTATTTTTCATAAACAATGAAAGCATATATAGGA
ATTATTTAAGGAGAGGTCTGCAATCTTTGAATGCCACTGGAAATACTATCAGGAGAATATTTGAGAAATCCTACTTCTTC
CCTCTAAAAATTATTACTTGAACAGTCCCTACCATTGTCTTCTTCACTGGCATGTTTTGCTCATCAAATCTTTGGGAAGA
CACGACAAAAAGACCTTCCTATATATCTTGGGCTCTCATGCCCCAGTGCTCATGCACACATCAAGGCTCTCACTGGTGCT
CAACACTGCATGTATTTTCCCATCTCAGGAATTTAAATGGAAGGTGGTGATTTACCTGTCCACAGTGGTCCACCCCATCG

TTCTACTGGTGAGCAACCCCGGCTGAGAGCTGGAGAGGGGATGCTCCTTAAGGTGTGGGGCATCTTGA
>MyluTAS2R18GP_GL429777:10814357-10813419
ATGTCCATTGGAATGAAGGTCTCCATTCTAGTTGTGGCAACAGGAATACTCCTTTTAGGAGTGCTAGGAAATGGATTCAT
CGGACTGGTGAACTGCATCGAATGGTTCAGGACTGGGAAAGTTTCTTCAGTTGATTTCATCCTCATCAGCTTGGCTCTGG
CCAGAATCATCCATCTGTTGCTAACACTATTGGATTCATTTATAATAGGGCTGGCTCCACATCTGTATGCTACTGGTAAA
GTAGCAAAGGTGGTTCCTATTCTTTGAGCACTAACTAATCATCTAACTATCTGGTTTGCCACCTGCCACAGCATTTTCTA
CTTCCTTAAAATAGCCAATTTCTCCCACTCCCTTTTCATGTGGCTGAAGTGGAGAGTCAACAGGGTGGTTCTTCTGCTTT
TCCTGGGGTCTCTCTTCCTATTGTCTTTTAATCTCTTAATGCATAATGCTGCTAGTAAATATTGGTTGAATACCTACAGG
GTATATGAAATAAATATGAGTTTGCAGTTAGAGGTAAATGAAATGTTCTATCTTAAAAGTCTTCTTCCTCTTAGTTTGAC
CTATGTTATCCCCTTATTCCTATCCCTGATCTCTTTGCTTCTTTTATTCCTGTCCTTGGTGAGACACACCAAGAATTTTC
AGCTCAACCTGATTGGCTCAGGAGACTCAAACACAGAGGCCCATAGAAGGGCCATGAAAATGGTTATAGCCTTCCTTCTC
CTCTTCATCATTTACATTATTTCTATTGTAACATCATGTTGGATCTCCACTAAGGTACTGACATTTCAGGTCAAGATGCT
TGTCATGATGATTTCAACTACCTTTCCCTCAGGCCACTCCTTTATTATAATTTTTGGAAACAGCAAGCTAAGACAGATCG
CCTTGAGACTACTCTGGCACTTAAATTCTCTGAGAAAAGCAAAACTTTATCATTATTGG
>MyluTAS2R18HP_AAPE02067993:2853-1916
ATGTCCATTGGAATGAAGGTCTCCATTCTAGTTGTGGCAACAGGAATACTCATCTTAGGAGTGCTAGGAAATGGATTCAT
CGGACTGGTGAACTGCATCGAATGGGCCAAGACTGGGAAAGTTTCTTCAGTTGATTTCATCCTCATCAGCTTGGCTATGG
CCAGAATCATCCATCTGTTGCTAACACTATTGGATTCATTTATAATAGGGCTGGCTCCACATCTGTATGCTACTGGTAAA
CTAGCAAAGGTGGTTCCTATTCTTTGAGCACTAACTAATCACCTAACTATCTGGTTTGCCACCTGCCTCAGCATTTTCTA
CTTCCTTAAAATAGCCAATTTCTCCCACTCCATTTTCATGTGGCTAAAGCAGAGAGTCAACAGGGTGGTTCTTCTGCTTT
TCCTGGGGTCTCTCTTCCTATTGTCTTTTAATCTCTTAATGCATAATGCTGCTAGTAAATATTGGTTGAATACCTACAGG
GTAAATGAAATAAATATGAGTTTGCAGTTAGAGGTAAATGAAATGTTCTATCTCAAAAGTCTTCTTCCTCTTAGTTTGAC
CTATGTTATCCCCTTATTCCTATCCCTGATCTCTTTGCTTCTTTTATTCCTGTCCTTGGTGAGACACACCAAGAATTTTC
AGCTCAACCTGACGGGCTCAGGAGACTCAAACACAGAGGCCCATAGAAGGGCCATGAAAATGGTGACAGCCTTCCTCCTC
CTCTTTATCATTTACATTATTTCTATTGTAACATCATGTTGGATCTCCACTAAGGTACTGATATTTCAGGTCAAGATGCT
TGTCATGATGATTTCAACTACCTTTCCCTCAGGCCACTCCTTTATTATAATTTTTGGAAACAGCAAGCTAAGACAGATCG
CCTTGAGACTACTTTGGCACTTAAATTCTCTGAGAAAGCAAAACTTTACCATTACTGG
>MyluTAS2R16ET_GL429840:3934939-3935052
ATGATACCCAACCAACTCACTGTTTTCTTCATGACCATCTATCTGCTCGAGTCCTTGACAATAATTGTGCAGAGCAGCTT
AATTGTTGCGGTGCTGAGCAGAGAGTGGTGCAGG
>MyluTAS2R16FT_GL429840:3935397-3935932
CTCTGATGATTTCCTGTGTGACAATCATTGTTTCAGCTCTTAGAGTTGTCTTCATCAAGAGTCAGTTAATATCCATGATG
CAATTACCTGGAAACAACACAGAGACTGAGACACTTAGGACATTCCTGGAGAAAAATTACGTACATCAGCTTCTGGCAAC
GTCGTTCATTCCTTTCCTACTGTTCCTGACCTCCACCATCTTGCTCATAGCCTCGTTGTGCCAACACTTGAGGCAGATAC
GACATCACGACACTGGCCACAGCAACTTCAGCATGAAAGCTCATGCCACTGCCCTGAGGTTTCTTGCCTTCTTCCTCATC
TTCTTCACCTCTTACTTTTTGACCATAATCATCTCCACTACCTATAACCTAACGCATGAGAGTTACTGGTTCTGGGCCAG
CGAAACTATCATCTATGCTACAGTCTCTATTCATTTAACTTCACTAATGCTGAGTAGCCCTAAGTTGAAAAAGGTTTTAA
AGGTAAGCTGCTGTGGCCCAAAGCTGCCTGAGGCTCCATGTACAACAAGACCCTAA
>MyluTAS2R41BT_GL429840:4102002-4101684
ATGCAGCCAGCATTCACAGCCCTCTTCATGCTGCTCTTTGTCCTGCTGTGTTTCCTGGGAATCCTGGCCAATGGCTTCAT
TGTGCTGGTGCTGAGCAGAGAATGGAGGCGGCATGGGAGGCTGCTCCCTTCTGACATGATCCTCATGAGCTTGGGTGCCT
CCCGTTTCTGCCTGCAGTGGGTTGGAATGGTGCACAACTTTTACTCCTTCTTCCACCCGGGGGAGTTCAGCAAGGGTCCT
GCACGGGAGCTCTTTGGTCTCCATTGGGACTTCCTGAATTCAGCTTCTTTCTGGTTCGGTACCTGGCTCAGTGTCCTCT
>MapeTAS2R1_KN005827.1:402320-403219

ATGCTAGAGTCTTACCATATCGTCCTTCTTCTTTTTGCAGTGATACAGTTTCTCATCGGAGTTTTAGCAAATGGCATCAT
TGTGGTTGTGAACAGCACTGACTTGATCAAGCAGAGGAAGATGATGCCCTTGGATCTCCTTCTTTGCTGCCTGGCAATTT
CTAGAATTTGTTTTCAGTTGGACATCTTCTACATTAACCTGGCTGTTCTCTCCTTGACTAAATTCCCTCCACTTGCTGAG
AATTTTACAATTCTCATGTTTTTAAATGAATCAGGACTTTGGTTTGCCACTTGGCTCAGTGTTTTCTACTGCGCCAAGAT
CGCCACTGTCGCTCATCCAACCATCTTCTGGCTGAAGATGAGGATATCCAAGTTGGTGCCATGGCTGATCCTTGGGTCAC
TGCTTTATGTACCTATCACCTCTGTTTTCCACAGCAAACATACATGGATTCTTCAAAAAAATATGTGGTTGGGCCTTTTC
TCCAAAAAATGCAACAACTCCAATCAAAGAAATGGATGCTTCTCAGTTTGCCTCTCATATTGTTAATCTTTCATTGCCATT
GATTATCTTCCTTATTTCTGTTCTGCTCTTGATATTTTCCCTGGGGAGACACACCCAGAAGATGAGAAATACAGCGGTGG
GAACCAGGAACCTCAGCATGAGTGTCCATGTCAGCACACTTGTGTCCATCCTGTCTTTCCTGGTCCTCTATCTCTCCCAC
TACCTGATTATTGTTGTGATCATTTTGCAAAATTTTAAGCTCAGAAGGCTCATCTTTCTGTTCTGCATCTTGGTGGTTGG
TTCATACCCCTCTGGACACTCAATAATCTTAATTTTAGGAAATCCTAAACTGAAACAAAATGCAAAGAAGTTCTTCCTCC
TCAAGAAGTGCTGTCAGTGA
>MapeTAS2R7_KN007244.1:197259-196327
ATGCCAGATATAGTGGAGAATAGCTTAATGCTCATAGCAGCTGTAGAATTTTCCATGGGGATCTTAGGGAATGTATTCAT
TGCAGTGGTAAATTTCATGGACTGGATCAAGAAGAGGAAGATTGCCTCCATTGATTTAATCCTCACAAGTCTGGCCATAT
CCAGAATTTGTCTACTATGTATAATACTACTTGATTGTTTTATTTTGGTGCTGTATCCTGATATCTATACTGCCGGTAAA
CAAATGCGAATCATTGACTTCTTCTGGACACTAACCAACCATCTAAGTGTGTGGTTTGCCACCTGCCTCAGCATTTTCTA
TTTCCTCAAGATAGCTAACTTCTTCCATCCCCTTTTCCTCTGGATGAAGTGGAGAATTGGCAGTGTGATTCCTAGGATCC
TGCTGGGCTGCTTGGCCCTCTCTGTGTTTATTAGCCTTCCTGTCATTGATAATTTGAATGATGATTTCAGGGGTTGTGTC
AAGGCAAAGTGGAAAAGAAACTTGACTTTGAGATGCAGAGTAAATAAAGCTCAATACGCTTCCATCAAGATATGTCTTAA
CCTGTTGACGCTATTCCCCTTGTCTGTGTCCCTGATCTCATTTTTCCTCTTGATTTTCTCCCTGTGGAGACATACCAGGC
AGATGCAGCTCAATACTACAGGAGACCCCAGCATAGAAGCTCACGTGGGAGCCATGAAAGCTATCATCTCCTTCCTCCTC
CTTTTCCTGGTCTACTATTTAGCCTTTCTCATCGCCACCTCTAGCTATTTTATGCCAGAGACTGAATTAGCTGTGATGAT
TGGTGAGTTGATCGCTTTAATCTATCCTTCAAGCCATTCATTTATCCTAATTCTAGGGAACAATAAATTAAAACAAGCAT
CCCTAAGGGTGCTATGGAAAATAAAGTATATCTTAAAAAGAAGACATTTCTAA
>MapeTAS2R2P_KN016421.1:2635-3539
ATGGCTTCCTCCTTGTCATCTATTGCTCATGTTTTCATCATGACTGCAGAATTTATCACAGGGATTACAGTAAATGGATT
TCTTACAATCACCAACTGTAAGAAACCGATCAAAAACAGAACGCTAACACCAGTGCAACTCCTTTTTTTATGTATAGGGA
TGTCTAGGTTTGGTTTTCAGATGGTGTTAACGGTACAAAGTTTTTCCTCTGTGTTCTTTCTGCTCTTTAATAGAGTAAAA
ATTCATGGTGCAGCCATGATATTCCTCTGGATGTTTTTTAGCTCTGTCAGTCTCTGGTTTGCCACCTGCCTTTCTGTAT
ATTATAGCCTCAAGATACCAGGCTTCCTATTTTCTTTGGCTAAAATGCAGGATCTCAAAGTTAATGACTTGGCTGCTTCT
GGGAAGCCTGCTCACCTCCATAAGCATTGCAGGTTTGTGTACTGAGGTGGACTATCTTAAAAACATGAATGACAATCTCC
TCAGGAATGGCACGCTGAAAAGGACTAAACCCAAGATAAAGAAAATGAATGAAGTGCTTCTTGTCAACTTGGCATTAGTA
TTACCTCTAGTCATATTTATGATGTGCACTTTTATGTTATCCATTTCTCTTTATAAGCACATTCATCGGATGCAAAACGG
ATTTCATGGTTTTAGAAATGCCAGTACAGAAGCCCATATAAATGCATTAAAAACAGTGATAACATTCTTTTGCTCTTTCA
TTTCTTATTTTGCTGCCTTCATGACAAATATGACATTCAATATTCTTTATGGAAGTTGGTGCTTCTTTGTGGTGAAGGAC
ATAATGGCAGCATTTCCCTCTGGTCATTCAGTTATAATAATCTTGAATAATTTCAAGTTCCAACAACCTTTCAGAAGACT
TCTCTCCCTCAAAAAGAAATGAAGA
>MapeTAS2R4P_KN025394.1:21482-20580
ATGCCTCCAATATCCTTTTTATTTGCTGTTATTGTCTCGACAATTTTGAGTACTGTAGGACTCATTGTGAATCTGTTTAT
TGCGGTGGTCAATTGCAAGACTTGGGTCAAAAACCACAGAATCTCCTCTTCTGATAGGATCCTATTCAGCTTGGGTATCA
CCAGATTTCTTACACTGGGACTGTTTCTACTGTACATGATCTACTTCATCTCTCCACATGCTGGAAAGTCAGTCTACTTA
GCCACTTTTTTCATGTTGTGTTGGATGTTTTTGGACTCTAATAGTCTCTGGTTTGTAACCTTGCTCAATGCCTTGTACTG
TGTGAAGATTACTAACTTCCACTACTAAGTGTTTCTCCTGCTGAAACGAAATCTCTCTGCAAAGATCCCCAGACTGCTGC

CAGCCTGTGTGCTGATTTCTGCCTTCACCACTCTCTTCTTGTATGTTGTGCTCAGACAGACATCATCATTTCCTGAATTT
GTAACTGGGATAAATGGCACAGCATTTCACATCAATAAGAGCGTCTTATATTTGATGACCACTTTAGTCTTGAACTCATT
TCTCCAGTTCATCATTAATCTGACTTCTGCTTCCTTGTTAATATATTCCTTGAGGAGACACATACAGAAGATGCAGCAAA
ATGCCACTGGTTTTTGGAATCCCCAGACTGAAGCTCATGTGGGTGCTATGAAGCTGATGATCTTTTTCCTCATCCTCTAC
ATTCCATATTCAGTTGCCACCATGCTCTTTTATCTCTCTTCTTCTGCAGAGATGAATTTGAGAACCAGATCCATGTGTGC
GATTATTTCCACCATTTACACTCCGGGACATTCAGTTCTCATTATTCTCTCACATCCTAAGCTGAAAACAAAAGCAACGA
AGATTCTTTGTTTCAACAAATAG
>MapeTAS2R10P_KN007244.1:211034-210079
AAGGCCTCCTCATTTTTATAGCTGTTAGTGAATCAATACTCGGGGGTTTAGGGAATGGATTTATTGGACTTGTAAACTGC
ATTGACTATGTGAAGAACAAGAAGGTCTCTATGATTGGCTTTATCTTCACTGGCTTAGCTACTTCCAGAATTTGTCTGAT
ATGGATAATAATTATGGATGCAATTATAAAGATACTCTCTCCAGATTGTATTCATTTGGTAATCTAATTGAATATATTAG
TTACTTATGGGTGATTATCAATCAGTCAAGTGTCTGGTTTGCCCCTAGCCTCAGCATCTTCTATTTCCTGAAGATAGCAA
ATTTTTCCCACCACATTTTTCTCTGGTTGAAAGATAGAATCATTAGGGTTCTTCCCCCTTCTCATGGGATCCTTGTTTAT
TTCATGGTTACTGACTTTTCCACAAATCGTGAAGATTATGATTGACTATAGAATGAAGAATAGAAACACAACGTGGCAGC
TGACCATGCATAAAAGTGAATTCTTTACTAACCAGCTTTTGCTTAATCTAGGAGTCATTTTCCTCTTTACACTGTCCTTG
ATTCCATGTTTCTTGTTAAACATTTCCCTTTGGAGACAGAGCAGGCAGATGCAACTGAAGGCCACAGGATTCAGAAACTC
CTGCACAGAAGCATGTATGAAAGCAATCCAAGTTTTGATATCTTTTATCACCCTCTTTACCTTCCATTTTATAGGCATTG
CCATAGAAATACCATGTTTTACTGTACCAGACAGAAAACTGCTGTTTATTTTTGTCGTGACAACCACAGCCGTCTATCCC
TAGGACCACTCATTTATCCTAATTCTCGGCAGCAGCAGGCTACAGCACGCCTCTTTGAGGATATTGCAGCAATTCAAGTG
CTGTGACAAAGGGATGCCGCTCAGAGCTCCACAGACATGTGTGTGTCAAAATGGGTGTTGTCTGGGAATAATCTAG
>MapeTAS2R8P_KN007244.1:201605-200687
ATGCTCAGTAATATCTTTCTGATCATAGTACCTGGAGAATTCATAATAAGAATGCTGGGGAATGTATGCGTCGGACTGAT
ACACTGGATTGACTGGATTAAGAAGACAAAGATCTCTTCAGCAGACTACATCCTCACCAGTCTAGCTATCGCCTTAATTT
CTTTGCTCAGTGTAATGATACTTGATGGCATCATATTGGTACTCTACGCAGATTTTTATGAAGGTGAGAAACTAATCAGT
ATCATTAATATTTTCTGGACACTCACCCACTACTTAAGTACGTGGTTTACCCCCCTCCTTAACATCTTCTATCTACTCAA
GAAGGCCAATTTCTCCACCCACTTTTTCTCTGGCTGAAGTAGAGAATAGGCAGGGTGGTGCCCTGGTCCTGCTGGGGTCT
TTGGCCGTGTCCTCTTTGGCCAGCCTCACACTAGCAACGACACTTAGTTATGACTGTGAGTTTCATACAATCGCAATACA
TAGGAGGAACTTCACTGAAATGTTCCATGTGAGTAACATTCGCGATACTTCAACCCCTTGACTCTCTTTACCCTCTTGGG
GATTGTCCCATTAACTGCGTCACTTAATCTCATTTTTCCTTTTAATTAGGTCCCTATGGAGACATGCCAAGCAAATGAAA
CTCACGGTTTCATCTGAGCACTGGAGACCCCAGCACAGAGGCCCACGTGGGAGCCCTGAAAACTGTGACTTCATTTCTCT
TCCTCCTTTTTGTATACTATGGGGCTTCTCTTTTGGCGACTTAGCTACCTTATGAAAGAAAGCAAGTTAGCCATTATGTT
TGGAGAGATTATAGCAATTCTCTATCCCTCAGGTCACTTATTTTAATTATTGGAAACAAGAAGCAGGGGAGGCATCTGCC
AGGATGCTGAGATGTGGGGAAACAGCCTGCATGCTGTAA
>MapeTAS2R12P_KN012951.1:13677-12737
ATGGTAAATCCACTGAAGAACATGTTTATGATCATTTCTGCTGGAGGATTTTCAATGGCGATTTTGGGAAATGAATTCAT
TGTATTAATTAACTGAACTGATTGTATCAGGAGCTGGACGTTCTCCCTGATGATTTTATTCTCACCTGCTTGGCTATTCC
AGAATATTCCTGCTGCGCATAATAATTTTAAGTATAGGCTTAGATATAATCTGTGAGGAAATACTTATTGTGCAATAATA
ATCTGATAAATTTGGAAATTCTCTGGACAGGATCCAGTTATTTCTGCATGTCCTGTACCACCTGCCTGTGTCTTCTATCT
CCTCAGGACAGCCAATTTATCTAATCCCATTTTCCTCTGGATGAATGGAGAATTCATAAAGTGCCTCTCTTTATTGTACT
GGGGGAAGCCCTCTCTTTCTGCTTGTACCTTGTTCAGGATACAGTAGTTAAGAGTCTGATCAAACCCAGGTAAACACTGA
AAAGGAATTTGACATTGCACTTAATGGTGAGAAATATAACTGACTTCTCATATGTGCCTTCACATGATGTTCACCGTCCC
CTTTGCAGTGTCACTTGCCCCTTTTCTCCTTTTCCTCCTCTCCTTAGGAGCCACATCAGGCAGATGGAGGGTACAGGTTC
TAAAGATCCTGGCACAGAGGCCCATGTGAGAGCTGTGAAGTCCATGATTTCATTCTTACTCTTCTTCTTTAGGTACTATT
TGAACAATACTATGATAAATGTGGCCTATCTCATACTAGACAGTTTGGGAGCAAAGATTTTTGCTAATGTGCTGGTATTT

TTCTATCCAGCCCTCCATCCAGTTCTTCTGATTTTAACCGCAAATTGAAATAGGATTCTCTCTGAAGTGTTGCATGAATT
TTCTCATAAAACATGCCTGAAATGATTATGATTTTCTCAAGAATGAAGACAACAAAAAGGA
>MapeTAS2R16P_KN015488.1:45736-46623
ATGCCTATCCAACTCACATGGCCTCTTCATTATCACCCATGAGCTTGAGTCCTTGGGAACAATTGTGCAGAGCAGGTTAA
TTGCCGCAGTGCTGGGCAGAGAGTGGGTGCAGGTCAAATGGCTGTCACCTGTGGGTGTGATTCTCACCAGCCTGGACTTC
TGCCTTTGGTGGGCATTGATGCTGTACAATTTTTGCTCCTATTTCAACCCTAACTATGTATTTGGTGCTTATCAATGATG
TGGGAACTTACTAAAACTCTTACTTGCTGGTTAACCAGCTAGTTTACTGCCTCCTACTGTGTCAGTCTCTTCTTTCACCC
ACCTTCTCTTTTCTGGCTCAGGTAAAGAATTTTGAGGTTGACTCCCTGGCCATTGCTGACTTTTCTGTTGATTTCTTGTG
TGTGAATCATCCCTTCAGCTATTAGGAATCACATCGTAGTTCAGTTCATCATTACGGGGTATTTCTCTACAAACAGGACT
GTGATTGAACGACTGAAGATATTCCAGCCGGTTTTGCTATGTATCAGCAAATGACTGCAGTGGCCACTCCTTTCCTCCCA
TTGCTGGCCTTCACTGTCTCGCAGTTGGCCTCACTGTCCCAGCACTTGCGGTAAATGCTACCCCACAGTGCTGGCCACTG
CACCTCCAGCATGAGAGCTCAGTCCCCTGCCCTGATGTCTCACACCATCTTCCTCAACTTCTTCCCCTCTTGCTTCCTGA
CCCTCCTCATCTCTGTTATAAGCATCCCATTTGATGAGAAGTCCTGGTTCTGTGCCTGGGAAGCTGTCGTCTGTGCTAGA
GTCTATATTGTTCCACTTCACTAATGTTGAGTGGCCCTACATTGATTAAGGCTTTAAAGGTAAGGTGCTGGGCCTAGAGG
CTGACTGA
>MapeTAS2R40P_KN011172.1:479-1426
ATGGCGACGGTAAAAACAGATGTCACAGGTAAAGACATGTCCAGGTTTAAAACTATCTTCAACATGGGAGTCTCTCGAGC
TGGGTGCATCACTGGCAACGCTGGGAATGCCTCATCCTGGCCATACATGGGGCCAGGTGGGCCAGGAGCAAAAGACTCCC
TGTTAGTGACTGCATTCGGTTTGTGTTGAGCTTTTCCAGGCTCTCGTTACAGGTATGGATAATGCTGGAGAATACTTACA
GTCTACTTTTCTGGATCACTTATAACCAAAACACAGTGTATATACTTTTCAAAGTCATCATCATTTCTCTCAGCCATTCC
AAACTCTGGCTTGCTGCCTGGCTCAGTTTTTTGCCTTTGAATTACAAACTTTATTCCCCTTTGTTCTCCATGATGACGAG
GACAGTCATGGTGCTGATGCCTTGGCTTCTGAGGATATCCCCATTCATCTCCTTATGCTTCAGCTTCCCCTTCTCCACAG
ACACCCTCAGTGCATATGGAAACAGTTCCACTCCTAGCCCCTCCTCCAACACCACTGAGAAGTACTTTTCTGAGACCAGT
GTGGTCAACCTGGTTCTTCTCTATAACTTGAGGATTGTCATTCCTCTGATCATGTTATCCATGTGGTCACCCTGCTGATC
ATCTCTCTCAGGAGACAGACACGCTATACACATGGAAAGCAATGGCACAGGCTTCAGGGCCCACAGCACGGAGGCTCACA
CGGGAGCCATCAAAGCTATCACCTGCTTTTTCATTCTGTACATTTTCAATGCAGTCGCTCTATTTCTTCCATGTCCAGCA
TCTTTGACATTGACAGTTCCTGGAATATTTTGTGCAAAATCATCAAGGCTGCCTACCCTGCTAGCCACTCGCTGCTACTG
ACCTTGGGCAACCCCAGGCTGAGAAGAGCCTGGAAGCAGTTCCAGCACGGAGTTCACCTTACCTATCA
>MapeTAS2R42P_KN012951.1:42833-41902
ATGTTCCCTGGATTGGATGTAATCTTTGTGATACTGTCAATAGTGGAAATCATAATTGAAATGTTGGGGAATGTGTTCAT
GGACTGGTAAACTGCTCTGAATAGGTCAAGAACCAAAAGACCTCTTTCGCTGACCTCATCCTCACCTGCCTGGCTATCTC
CAGAATCGGTCAGCTGTTCGTGTCATTGTTTGAATCATTAATTACGAGATTGTATCTACATTTATTTTCCACTTACGGAC
AAGCTAGCAAAACCTGTTACTTTGTTTTGGAGAATAATCACTTGACTGCCTGTTGGGCTACCTGCTTAAGCATTTTTTAC
CTCCTTAAGATAGTGGAGAAATGACTCCACTCACTTTTCCTCTTGCTGAAGGGGAGAAGAAACAGAGCGATTCTTGTGAT
TCTTTATATTTGTTCTTTCTAAGTTTTGACTTTCTATTGCTAGAAACATTTAAGGATCTCTTCTTGAATGTCTCTGCAGT
AGATGCCAGCAATCTGACAGCCATATTTGGGTGAAAATAAACTATCTATGTCAAAACCGTGGTCATTCGTAGCTTGACCT
GTTTCATTCCTATTGTCCTGTCCCTCATCTCATTGCTCCTTTTATTTCGGTACTTGTAAGAAACATCAGAGATTTGCAGC
TCAACAACATGGGCTCAAGGGACTCCAGCACAGAAGCCTATAAAAAGGCCACGAAAATGGGGATGTCTTTTCTTTCCCTC
TTCAGGGCTCATTTTTTCCCCCACATACTTGGCAAATTGTATGTTTCTTGTATTTTGGAACAAGTTTACCAAGTTTGTCA
TTTTAGCAGTCTATGTTTTTCCCTCAGGCTACCCATTTATTTTGATTCTGGGAAACAGCAAGCTAAGACAGCCTTGAAGG
TACTATGGCAACTTAAAATCTTGAAAAGAGAACATCCTTTGCAGCTCCACAG
>MapeTAS2R46P_KN012951.1:29776-28844
ATGGTAACTTTTACTAGAGACCATTATTTCCATCCTAGTAGTGGTAGTATTTGTTCTGGGAAATCTTGCCAATGGCCTCA
TAGTTCTGGCAAACTGCAATGACTGGGTCAAGAGACTAAAGATCTCCTCAGTGGATCGAATTCTTACTGCTCTGGCTGTC

TCCAGGATCAGTTTGCTCTGGGTAATAGTAATAAATTGGTATACAGCTATGTTTAATCCAGCTTTATATTGTTCAGAAGT
AAGAATTACTGTGTCTATTGCTTGGGCAGTAAGCAACCATTTCAGCATCTGGCTTGCTACCAGCCTCAGCATATTTTATT
TGCTCAAGATAGCCAATTTCTCCGGCCTGATATTTCTTTACGTCAAGGGGAGAGTTAACAGTGTACTTCTTGGAATACTG
TTGGGGCCTTTGGTCTTTTTGTTTTCTCTTATTGCATTGGCCAGCCTAGATGATAAAATGCCGCCTAATGAATGTGAAGG
AAACATGACTTGGAAGACAAAAGTGAGGGACATTGTGCACCTTTCAAATATGAGTATACTCACACTGGCAAACGCCATTC
TCTTTGCTATGTCCCTGGCATCTTTTCTACTGTTAATCTTTTCTCTGTGGAAACATGTCAAGAAGATGAAGCTCCATGGT
AAAAGATCCCAAGACACCAGCATGCAGGTGTCCACCTGAGAGCCATGCAGACTGTGATCTCCTTCCTCTTGCTATTTGCC
AGTTACTTCTTGAGTCTAATCCTCACAGCTTGGAGTTCAAATAGGAAGCAGAACAAGCTGTTCTTCCTGCTTTGTGATGC
TCTTGAATTGGTCTATCCTTCAAGCCACTCATTTATTCTGATTTGGGGAAACCAGAAGCTAAAACAGGCCTTTCTGCTGG
TGTTATGGCAGGTGAACTCCTGGCTCAAAGAATGGATACCTTCAACTGCATAG
>MapeTAS2R62P_KN022215.1:21113-20242
TTCTGGAGTCATTGACTGCTATGTTGCAGAATGGCTTCATGGTTACTGTGCTGGGCAGAGAGTGGGCACCATGCCAGACC
CTCCCTGCAGGCAACATGACTGTGTTCTGCCTGGCTGCTCCTGGTTCTGCCTCCACGGCTGCCTCCACAGCCATCCTGAA
TAACCTCCTGGCTTTCTTTGAATTTTGTTCCAGAGTTTATTATTTCAACATTATCTGGGGCATTATCAACACTCTTTTCT
TTGTGGCTTCCTGTCTTCTACTGTAGATCTCATTCTTCTCTCACCCCGTCTTCTGGCTGAAGTGGAGGATTCCTCAGTCA
GTGCTAAGGGTGCTCCTGGGCTCCCTGATCACGTCTGGCCTGACACTCATCAGCTGCTGAGAATGTGACTCTTGTGCACA
TGACTACCTCCCAGGGTTCCCATGGAAACAGCACCCTGGGCTGATGGAATGCAGACCCTCTGCTGGCAACTTTTTCTACA
TCATATAATACTTAGGCTGTTTTCCATTCCTCCTTTTCCTGGAATCCGTCCTCTTGCTCATGTTCTCACTGCACTGACAC
TTGTGGCAGATGAGGGACCATAGACCCAGCCCATGTGATCCCAGCACCCAGGCTCACATCATAGCCCTGAAGTTATGTGT
CTTCTTCCCTGTCTTCTACACTTCATATTTCCTGTCCCTGCTTATTGTTATGAAATCACAACCTCCCAGAGCCACAGGCA
CTGGGGCCTGGGAAGTGGTGAGCTGTGCAGGCATCTGTCTGCCTTCCAGCATCCTGGTGCTGATCAGTCCCAAGCTGAGA
AAGGCCCTGAAGATAAGGCCTCGGGAAGCCCTGGGAAAAGGACAATTTGTCTCAAGTTGTCAGCACCACTGT
>PtvaTAS2R1A_KN525907.1:1358823-1357924
ATGCTAGAATCTATTGTCGTTACCCATCTTCTTTTTGTAATGATACAACTTCTCTTCGGGGTCTTAGCGAACGGCGTGAT
CGTGGTCGTGACCGGCACGGAGCTGACCAGGCCCAGAAAGATGGCGCCTCTGCATCTCCTTCTCTGCTGCCTGGCTGTTT
CCAGAATTTGTCTCCAGATGTTCATCTTCTACGACAGTCTGGTCGTTCTCTCCCTCATCGAATTCTTCTCGCTTGCCGAG
ACCAATACGGCGTTCATGTTTTTTAGTGAACTGTCGCTTTGGCTGGCCACTTGGCTCAGCGTGTTCTACTGTGCCAAAAT
CGCCACCTTCGCTCACCCTCTCTTCTTCTGGTTGAAGCTGAGGATTTCGAGGTTGGTGCCACGGCTGATTGTCGGGTCCT
TGCTCTACACGTCTCTCACTGCTGTTTTCCACAGAAAACATACATGGATTCTTTCCCAAAATCTCTGGCTGAGCCTTTTC
TCTCAGAATGCCACCACTCAAGGCAATGAAATGTCCACGTTACACTTTGCCATTCTGGCCGTTGAGTTCTTCTTGCCACT
ATTTATCTTCCTACTTTCTGCTCTGCTCCTGATATTCTCCCTGGGGAGACACACCCAGCAGATGACGAGCATGGCGGCGG
TCAGTGGGCACACAGGGACGAGCGTCTACTTCAGCACGCTCCTATCCATGCTGTCCTTTCTGGTCCTCTACGTCTCTCAG
TATGTGATGGCTGCTTTAATCTTTTCTCACAGTTTCAAGATCAAGAACTTCACCTTTATGTTCTGCTTCTTGGTGCTTGG
TTTGTACCTCTCTGGGCACTCTATTATCTTAATTTTTGCAAGTCCCAAACTGAAACAAAATGCAAAGAAGCTCTTCTTCC
ACAGCAAGTGCTGTCAGTGA
>PtvaTAS2R3A_KN525765.1:265536-264586
ATGGCGGGACTCACAGAGTGGGTGTTTCTGGTCTTGTCTGCCACTCAGTTCTTTCTGGGAATGCTGGGGAATGGTTTCAT
CGGGGTGGTCAACGGCAGCGGCTGGTTCAAGAGCAAGAGAATCTCTTTGTCTGACTTTATCACCACTAACCTGGCTCTCT
CCAGGATTGCTCTGCTGTGGATTCTCATGTCTGATGCTGTAATACTGGTGTTCTTTCCCAAAACACATGATGAAGGGGTG
ATCATGCAAGTGATTGATGTTTTCTGGGCATTTACAAACCATCTGAGCATTTGGCTTGCCACCTGTCTCGGTGTCTTCTA
CTGCCTGAAAATCACCAGTTTCTCCCACCCCACCTTCCTCTGGCTCAAGTGGAGAGTTAACAGCGTGGTTGTGTGGATGC
TGCTGGCTGCGTTGCTCCTATCGTGTGGCAGTGCTGTGTCTCTGATTCACAGTTTTAAGGTCTATTCTGCCCTCTGTGGA
ATCGATGGCACAGGGAATGTGACTGAGCACTGTAGAAAACTAACGAACGAGTACCAAGTGACCCATGTTCTTGGCACCCT
GTGGAACCTCCCGCCCCTCATCATGTGTCTGGCCTCCTACATCGTGCTCATCCTCTCCCTGGGGAGGCACGGGCGGCGGA

TGCAGCACAACAGCGCCAGCTCCAGCGATGCCAGCACTGAGGCCCACAAGAGGGCCACTAAAATGATGCTCTCCTTCCTC
TTCCTCTTCCTGCTTTACTTTCTTGCCTTTTTAATTACAACATCCAGTTATTTCCTACCAGGAACTAAGATGACTCAGAT
GATTGGAGAACTAATTACAATGATTTATCCTGCCAGCCACTCGTTTGTCTTCATTCTGGGAAACAGCAAGCTGAAGCAGA
CATTTGTGGAGATGCTGTGGTGTGAGTCTGGTCGTCTGAAGTCTGGATCCAAGAGACCCTTTTCCCCATAG
>PtvaTAS2R4_KN525765.1:256749-255850
ATGCTCCTAACGCCCTTTACCTCTGCCGTTATTTTCTCAATGATTTTGGATGTCGCAGGACTCATTGTGAATCTGTTTAT
TGTGGTGGTCAACTACAAGACTTGGGTGGAAAGCCGCAGAAGCTCCTCTTCTGATAAGATCCTGTTCAGCGTGGGCATCA
CCAGGTTTCTGATGCTGGGGCTGTTTGTCCTGTACATTAACTACTTCCTCATTTCTTCAGATGCTGAAAGGCCAGTCCGC
ATATCCAATTTGTACCTGTTCTCTTGGCTGTTTTTGGACTCTAGCAGTCTCTGGTTTGTAACCTTGCTCAACGTCTTGTA
CTGCGTGAAAATCACGAACTTCCGACACTCAGTTTTTCTTCTGCTGAAACGAAATCTCTCCCCAAAGACCCCCAGGCTGC
TGCTGGCCTGCGTGCTGAGTTCTGCCTTTACCACGCTCCTGTACGCTGTGGTCAGACAGACATTCCTTCCCAAATTTGCG
GCTGGGCGAAATGGCACAGTGTTTGACATCACTGAGGGCATCTTGTCTTTGGTGATCTCTCTGGGCTTGAGCTCGTTTCT
CCAGTTCACCATCAATGTGACTTCCGCCTCCTTGTTAATATATTCCTTGAGGAGACACATACAGACGATGCGGAGAAACG
CCAGTGGTTTCTGGAATCCCCAGACCGAGGCTCACGTGGGCGCCATGAAGCTGATGGTCTGTTTCCTCCTCCTCTACGTT
CCGTATGCAGCCGCTGCCCTCTTCCTCTACCTCCCTTCTGACATAGAGGTGGGTTTGGAATTCAGATCTGCGTGTCTGAT
TATCTCTACCTTTTACTCTCCGGGACATTCTGTTCTCATTATTCTCACCCACCCGAAGCTGAAAACTAAAGTAAAGAAGA
TTCTCTGTTTCAACAAATAG
>PtvaTAS2R5A_KN525765.1:244917-244021
ATGCAGACCGCTGCCCGAGGACTGCTGATGGTGGTGGCAGTCACTGAATTTCTCATTGGCCTGGTTAGCAACGGAGTCCT
CATATTCTGGAGTTTTAGAGAATGCGTCAGAAAATGCAAGGGATCTTCGTACAACCTCATTGTCCTGGGCTTGTCTGGCT
GCCGATTACTCCTGCAGTGCCTGATCATGATAGACTTAAGCCTGTTCCCGCTTTTCCAGAGCAGCCTTTGGCTTCGCTAT
CTCAATGTCTTCTGGGTTATGGTAAGCCAGGCCAGCCTGTGGTTTGCCACGTTCCTCAGTGTCTTCTACTGCAAGAAGGT
CACGACCTTTGAACACCCTGTCTACCTGTGGCTGAAGCACAGGATCTACTGCCTGAGTCTATGGTGCCTTCTGGGTGCCC
TCATGATCAATCTGTTGCTTGTAGCCCACATCGACTTAAAGCCCTACAGCGCTTCCCAAGGAAATAGGAGCATGCTGTGC
CTCCTTTCAAGCTGGCACTATCTGTATATATTACAGCTCAATTCAGGAAGCGGGTTGCCTTTCCTGATGTTTCTTTTATC
CTCTGGGATGCTGATTATCTCTTTGTATAGACACCACAGGAAGATGAAGGTCCATTTAGCTGGCAGGAAGGATGCTCGGG
CCATGGCTCACATCACCGTCCTGAAGTCCTTGCTTTGCTTCTTTATACTTTACATGGTATACGTAGTGGCCAGCACCTAC
TCCATCTCCTCCAAGTCTCCTGTTAATCTCACCTCTGTCTTCATCTCAGAGATACTCATGGCTGCCTATCCTTCTCTTCA
TTCTGTCATATTGATTATGGGGAATCCCAGGATGAAACAGGCTTGTCAGAGAATCCTGCAGAAGGTAGTGTGTGCTTGGA
GGGCCTGCGGCCTGTGA
>PtvaTAS2R5B_KN525765.1:243417-242521
ATGCAGACCGCTGCCCGAGGACTGCTGATGGTGGTGGCAGTCACTGAATTTCTCATTGGCCTGGTTAGCAACGGAGTCCT
CATATTCTGGAGTTTTAGAGAATGCGTCAGAAAATGCAAGGGATCTTCGTACAACCTCATTGTCCTGGGCTTGTCTGGCT
GCCGATTACTCCTGCAGTGCCTGATCATGATAGACTTAAGCCTGTTCCCGCTTTTCCAGAGCAGCCTTTGGCTTCGCTAT
CTCAATGTCTTCTGGGTTATGGTAAGCCAGGCCAGCCTGTGGTTTGCCACGTTCCTCAGTGTCTTCTACTGCAAGAAGGT
CACGACCTTTGAACACCCTGTCTACCTGTGGCTGAAGCACAGGATCTACTGCCTGAGTCTATGGTGCCTTCTGGGTGCCC
TCATGATCAATCTGTTGCTTGTAGCCCACATCGACTTAAAGCCCTACAGCGCTTCCCAAGGAAATAGGAGCATGCTGTGC
CTCCTTTCAAGCTGGCACTATCTGTATATATTACAGCTCAATTCAGGAAGCGGGTTGCCTTTCCTGATGTTTCTTTTATC
CTCTGGGATGCTGATTATCTCTTTGTATAGACACCACAGGAAGATGAAGGTCCATTTAGCTGGCAGGAAGGATGCTCGGG
CCATGGCTCACATCACCGTCCTGAAGTCCTTGCTTTGCTTCTTTATACTTTACATGGTATACGTAGTGGCCAGCACCTAC
TCCATCTCCTCCAAGTCTCCTGTTAATCTCACCTCTGTCTTCATCTCAGAGATACTCATGGCTGCCTATCCTTCTCTTCA
TTCTGTCATATTGATTATGGGGAATCCCAGGATGAAACAGGCTTGTCAGAGAATCCTGCAGAAGGTAGTGTGTGCTTGGA
GGGCCTGCGGCCTGTGA
>PtvaTAS2R7_KN526254.1:241835-240897

ATGTCAGATGAAGTAAAAAACACCTTAATGATCATAGCAGCTGGAGAATTTTCAATGGGAATCTTAGGAAATGCATTCAT
TGGCTTGGTGAACTGTATGGACTGGATCAAGAATAAGAAGATTGCTTCCATTGATTTAATCCTCACAAGTCTGGCCATAT
CTAGAATTTGTCTATTGTGTATAATACTATTAGATTGTTTTATATTGGTGCTGTATCCAGATGTCTATGCCACCGGTAAA
CAAATGAAAATCATTGACTTCTTCTGGACACTAACCAACCATTTGAGTGTCTGCTTTGCCACCTGCCTCAGCATTTTTTA
TTTCCTCAAGATAGCTAATTTCTTTCATCCTCTTTTCCTCTGGATGAAGTGGAGAATTGACAGGGTGATTCCTAGGATCC
TGCTGATGTGCTTGGCCGTCTCTGTCGTTATTAGCTTTCCTGTCATTGAAAATTTGAATGATGATTTCAGGCTTTGTGTC
AAGGCAAAGTGGAAAGCAAACTTAACTTTGAGATGCAGGGTAAATAAAGCTCAGTATGCTTCCATTAAGGTATGTCTCAA
CCTGTTAACACTCTTCCCCTTTTCTGTGTCCCTGATCTCATTTCTCCTCTTGATTCTCTCCCTGCGGAGACACACCAGGC
AGATGCGGCTCAATGCCACAGGATGCAGAGACCCCAGCATAGAAGCTCATGTGGGAGCCATGAAAGCTGTCATCTCTTTC
CTCATACTTTTCATTGCCTACTATTTGTCCTTTCTCGTAGCCACCTCCAGCTACTTTATGCCAGAGACTGAATTAGCTGT
GATGATTGGTGAGTTGATAGCTCTAATCTATCCCTCAAGCCATTCTTTTATCCTAATTCTAGGGAACAATAAATTACGAC
AAGCATTTCTAAAGGTGCTATGGAAAGTAACATATATCCTGAAAAGAAGAAATTTCTAA
>PtvaTAS2R9_KN526254.1:249153-248218
ATGCTAAATACAATGGAGGCAATATACATGATCTTGATTGCTGGTGAAATGACTATAGGAATTTGGGGAAATGGATTCAT
TGTACTGGTTAACTGCATTGGTTGGCTCAAAAAGAGAGATATCTCCTTGATTGATATCATCCTGGTCAGCTTAGCCATTT
CCAGAATCTGTTTGTTGTGTGTAATATTTTTAGATGGCATCATAGAGCTTCTTTCTCCAGAAACATATGACCATGATGAA
GTAATGAACATTTTGGATGTTTTCTGGACACTCTGCAATCATTCAAGTGTCTGGTTTACCTCTTGCCTTAGCATCTTCTA
TTTACTCAGAATAGCCAATATATCCCACCCATTTTTCCTCTGGATGAAGCTAAAGATTAACAGGATCATCCTTGGGATTC
TTCTTGTGTCTTTTCTCATCTCCTTAATTTTTAGTATTTCATTGAATGAGGGCTCCTGGAATTATTTCAAGGTCGATCAT
GAAGAAAACATAACTTGGGAATTCAAAGTGAGTAAAATCCCAAATGCTTTCAAACAGATTACCCTGAATCTGGGTGCTAT
ACCTCCTTTTGTTCTTTGCCTGATCTCATTTCTCTTGCTACTTTTCTCCCTTTTTAGACACACCAAGCAGATGAAATTTC
ATGCCACAGGGTTCAGAGACCCCAGCACAGAGGCCCACATGAGGGCCATAAAGGCAGTGATAATCTTTCTGATGCTCTTC
ATTATGTACTATGTAGCCTTTCTTGTAGTAACCTCTAGTCTTATGATTCCCCAGGGAAAATTAGCAATGATGTTTGGTGG
CATAATAACTGTCATTTTTCCATCAAGCCATTCATTCATCCTGATAATGGGAAACGGTAAGCTGAGGGAGGCTTTTCTGA
AGGTGCTAAGGATTTTGAAATGTTTCCACAAGAGAAGGAGCTTTTTTGTTCCTTAG
>PtvaTAS2R16_KN525750.1:4159979-4159074
ATGATACCCATCCAACTCACTGTCTTCTTCATGGTCATCTATGCGCTTGAGTCCTTGACAATAATTGTGCAGAGTAGCCT
AATTGTTGCAGTGCTGGGCAGAGAGTGGATGCAGATCAAAAGGATGTCACCCGTGGACATGGTTTTCATCAGCCTGGGTT
TCTGCCGCTTCTGCCAGCAGTGGTCATCGGTGCTGTACAATTTTTGCTCCTATTTCAACCCTAACACCACATTTTGGTAC
ATAGGAATCATCTGGGAATTTACTAATACTCTTACGTTCTGGTTAACCAGCTTGCTTGCTATTGTCTACTGTGTCAAAGT
CTCTTCCTTCACCCACCCCGTCTTTCTCTGGCTGAAGTGGAGAATTTTGAGGTTGGTTCCCCAGCTACTGCTGGGCTCTC
TGCTGATTTCTTGTGTGGCAATCATCTTTTCAGTCATTAGGAGTCGCATCAAATTTCAGTTAATCTCCATGATGCATTTG
CCTGGAAACAACACTGTGACTGAGAGAATTAAGATGCTTCTGCAGAATTTTTTGATATTTCAGCAACTGGTGTTGTTGGT
TATTCCTTTCCTCCTATTCCTGGCCTCCACCATCTCGCTCATAGCCTCATTGTGCCAACACTTGGGGCAGATGCAACGTC
ACAACATTGGCCACTGCAACTCCAGCTTGAAAGCTCACTTTAGTGCCTTGAGGTATCTTGCCTTCTTCCTCATCTTCTTC
ACCTCTTACTTTCTGGCCATATTTATCACCATAATAGACAATCTGTTTAATAGGAGACATTGGTTCTGGGCCTGGGAAAC
TGTCATCTATGCTGTGGTCTCTATTCACTCCACTTTACTGATGATGAGCAGCCCTAAATTGAAAAAGGTTTTAAAGGTAA
GGTGCTGGGGCCTAGAGACTGCCTGA
>PtvaTAS2R38_KN525765.1:174688-175695
ATGTTGATGCTGACTCCCGTCGTCACTGTGTCCTACGAAGCCAAGCGCGCATTTCTGTGCCTTTCGATCCTGGAGTTCGT
GGTGGGGATTCTGGCCAATGCCTTCATTTTCTTGGTGAATTTTCGGGATGTCGTGAGGAGGCAGCCCCTGAGCAACTGTG
ACCTTGTCCTGCTGAGTCTCAGCCTCACGCGGCTCTTCCTGCACGTGCTGCTGTTTCTGTATGCCATCCAGCTTACCCAT
TTCCAGCAGATGAAAGACCCGCTGAGTGTCAGTTACCAAACCATCGTCATGCTCTGGATGGTCGCAAACCAAGCTGGCCT
CTGGTTCGCCACGTGCCTCAGTCTCCTCTATTGCTCCAAGATCGTCCGCTTCTCCCGCGCCTTCCTGCTCTGCTTGGCAA

AGTGGGTCTCCAGGAAGATGCCCCAGATGCTCCTGGGTACAACCCTTTTCACCACCGTCTGCACTGTCATCTGTTCTTGG
GACTATTTTAGCAGCTCTCACTTCACGGGCACAGCTATGCTTTTCATGAATAATGATACAGAGTTCCACCTGCAAATTAA
AAATCTCGGTTTCTTTCATTCCTTCCTCTTCTGCAGCCTGGGATCCGTCCCGCCTTTCTTGTGCTTTGCGGTTTCTTCTG
GGGTGCTGATCGTCTCCCTGGGGCAGCACATGAGGACGATGAGGGCCAAAACCAGAGACTCCCACGACCCCAGCCTGGAG
GCCCACGTCAAAGCGCTCAAGTCTCTCGTCTCCTTCCTCTGCCTCTATGTGGTGTCACTCTGCGCTGCCCTCCTCTCGAT
GCCTTTACTGATACTGTGGCACAACAAGATTGGGGTCATGATCTGTGTAGGGATCATGGCAGCCTGTCCCTCGGGGCACG
CGGCCATCGTGATCTCAGGCAATGCCAAGCTGAGGGCGGCTGTGGACGCCATCCTGCTCTGGGCTCGGACCAGGCTCACG
GTGGGAGCGGAGCACCAGGCGGATCCGCTGGTGCCAGATCGATGCTGA
>PtvaTAS2R40_KN526046.1:530832-529876
ATGTCAACAGTGAACACAGATGCCACGGATAAAGGCATGTCCAGGTTTAAAATCGTCTTCACCCTGGCGGTCTCCGGAAT
AGAGTGCATCACTGGCATTGCCGGGAACAGCTTCATCACGGCCATCCATGGGGCCGAGTGGGTCAGACGCAGAAGGCTTC
CTGTTGGTGACTGCATTCTGTTGATGCTGAGCTTTTCCAGGCTCTTGCTACAGATCTGGATGATGCTGGAGAATACTTAC
AGTCTACTATTCAGGGTCACTTACAACCAAAATGCAGTGTATATACCTTTCAAAGTCATCATCATGTTTCTCAACTATTC
CAACCTCTGGCTCGCCACCTGGCTTAACGTCTTCTATTGTCTTAGAATTGCAAACTTCACTCACCCTTTGTTCTCCCAGA
TGAGGAGGAAGGTCATGGTGCTGATGCCTTGGCTCCTGCGGCTATCGCTGTTCCTCTCCCTGTGCTTCAGCTTCCCCTTC
TCCGTAGACATCTTCAACGTGTATGTGAACAGCTCCATTCCAGTCCCCTCGTCCAACTCCACTGAGAAGAAGTACTTCTC
TGAGACCAACGTGGCCAACCTGGCTCTTCTCTACAACGTGGGGGTCTTCATTCCTCTGACCGTGTTCATCGTGGCGGCCA
CCCTGCTGATCATCTCTCTCAGGAGACACACGCTACACATGGAGAGCAAGGCCACCGGCTCCGGGGACCCCAGCATGGAA
GCTCACATGGGCGCCATCAGAGCCATCAGCTACTTTCTCATTCTCTACATCTTCAACGCGGTTGCTCTGTTTCTGTCCAT
GTCCAACGTCTTTGACGCCAACAGTTACTGGAATATTTTGTGCAAAATCATCATGGCTGCCTACCCTGCTGGCCACTCAG
TGCTACTGATCTTGGGCAATCCTGGCCTGAGAAGGGCCTGGAAGCGGCTTCAGTGCCGAGTCCATCTTTACCTGTGAGGA
CAGAGTCTGTGA
>PtvaTAS2R41_KN526046.1:347853-346927
ATGCAGCCAGCTCTCACATCCCTCTTCATGCTGCTCTTTTTCCTGCTGTGTCTCCTGGGAATCCTGGCCAATGGGTTCAT
CGTGCTGGTGCTGAGCAGAGAGTGGAGGCAGCGTGGACGGCTGCCCCCCTCGGACCTGATCCTCATTAGCCTGGGCGCCT
CCCGCTTCTGCCTGCAGTGGGTTGGACTGGTGCACAACTTCTACTACTTCCTGCGCCTGGTGGACTATTCCAGGGGCCCA
GCGCAGCAGCTCTTCGGTCTGCACTGGGACTTCCTGAACTCGGCCACCTTCTGGTTCGGCACCTGGCTCAGTGTCCTCTT
CTGCGTGAAGATCGCTAACCTCTCCCACCCCACCTTCCTCTGGCTGAAGTGGAGGTTCCCAGGGTCTGTGCCCTGGCTGC
TGCTGGGTTCCCTCCTGGTGTCCTCCATCGTCACCATGCTCTTCTTCTGGGGCAACCGCGCCGTGTACCTCGGTTTCTTC
ATTGGAAGGTGTTCTGGGAACATGACCTACAAGGAGTGGAGCAGGTGGCTGGAAATTCACTATTTCCTGCCCCTGAAATT
TGTCACGTTGTCAGTCCCTTGCTCTGTCTTCCTGGTCTCGACGGCACTGTTGATTAATTCCCTAAGGAGACACACTCGGA
CGATGCGGCACAATGCCCACAGCCTGCAGGACCCCAGCACCCAGGCTCACACCAGGGCTCTGAAGTCACTCATCTCCTTC
CTTGTTCTCTATGCTCTGTCCTTTGTGTCCATAGTCATCGATGCTGCAGGCTTCTTCTCCTCAGACAGTGACTGGTACTG
GCCGTGGCAAATTTTAATGTACCTGTGTACATCCGTCCATCCCTTTATCCTCATCCACAGCAACCTCAGGCTTCGAGGGG
TGTTCAGGCAGCTACTTCTATTGGTCAGGGGCTTCTGGGTGGCCTAG
>PtvaTAS2R42_KN526254.1:334976-334029
ATGCCCACAGGATTGGATATAATCTTTGTGGTACTGTCAATAGCAGAATTCACAATTGGAATTTTGGGAAATGTGTTCAT
TGGACTGGTAAACTGCTCTGAATGGGTCAAGAACCAAAAGATCTCTTTAGCAAACTTCATCCTTACCTGCTTGGCCATCT
TCAGAATCAGTCAATTGTTGGTATTATTGTTTAAATCCCTTATACTGGGGCTATCTCTACATTTACATTTAACTTATACA
ATAGCAAAGCTTATGAGTTTGCTTTGGAGAATAACTGATCACTTGACCACTTGGCTTGCTACCTGCCTAAGCATTTTCTA
CCTCCTTAAGATAGCTTACTTCTCCCACCCCCTTTTCCTATGGCTGAAGTTGAGACTGAATAGAGTGATTCCTGTGACTT
TTTTATTTTCTTTGTTCTTCCTGATTGTTGACTTTCTATTGCTAGAAATATTTAATGATTTTTTCTTAAACGTCTATATA
CTAGATAAAAGCAATCTTACTTTATTTATAGATGAAAGTAAAACTCACTATGTTGAAACCCTGATTCTTCTTAGCTTGAC
CTGTTTCTTTCCCATTGTTTTGTCCCTGACTTCATTGCTCCTTTTATTTCTGTCCTTGGTAAGACACATCAGAAATTTGC

AGCTCAATTCCATGAGCTCAAGGGACTCTAGCACAGAGGCCCATAAAAAGGCCATAAGAATGGTGATGTCTTTCTTTTTC
CTCTTCATAGTTCATTTTTTTTCCATACAAGTAACAAATTGGCTATTTCTTATGACGTGGATCAACAAATTTGCAAAGTT
TGCCATGTTAGCAGTATATATCTTTCCCTCAGGACATCCATTTATTTTGATTGTGGGAAATAGCCAGCTAAGACAGACAA
TCTTGAAGGTACTGTGGCATCTTAAAAGTTTCTCAAAAAGAGAAAATCTGTTACAGATTTACAGATAG
>PtvaTAS2R408A_KN526254.1:290313-289477
ATGATAAGTTTACTACTGATTATTTTCCCCATCCTACTAATGACAGAATTTGCTCTAGGAAATTTTGCCAATGGCTTCAT
AGCCCTGGTGAACCTCATTGACTGGGTCAAGAGACAAAAGATCTTGTCAGCTGATGGAATTCTCACTGCGCTGGTGGTCT
CCAGAATGGGTTTGCTCTGGACAATAGTAATAGATAGGTATGCCCCTGTGTTTTATCCAGCTTTATGTAGTTCAGAAGCA
AGAATTATTTTTAATGTGATCTGGACAGTAAGCAACCATTTTAGTGTCTGGCTTACGACTAGCCTCAGCATACTTTATTT
GCTCAAGATAGTCAATTTCTCCAGCCTTATATTTCTTCACTTAAAGTGGAGAGTTAAAAGAGTAATTCTTATGATACTGT
TGGGGACTTTGGTCTTCTTGGTTTTTCATCTTGTATTGGTAAGTATAGATAGAAAAATGTGGATGAATGAATGTGAAGGA
AGCATCACCTGGAATACCAAATCGAGGGACATCGTGCACCTATCGTATATGACTGTATTTACATTTGCAAACTTTGTATC
CTTTTCTATGTCCCTGACATCTTCTCTGCTGCTAATTTTTTCCCTGTGGAAACACCTCAAGAAGATGCGGCTTAGTGGCA
AAGGATCCCAAGATCCCAGGACCGAGGTCCACATAAGAGCCATTCAAACTGTGATCTCCTTTCTCTTGCTATTTGCCATT
TACTTCCTGACTCTAATCTTCTCAGCTTTGAACATAAATACTCTGAAATATGAACCAGTTCTCATGTTTTGCCAGATTTT
TGGAATCTGTATCCTTCAGGCCACTCATTTATCCTGA
>PtvaTAS2R408B_KN526254.1:300521-299362
ATGATAAATTTACTACAGAGCATTCTTTCCATCTTTGTAATAGCAGAATTTGTTCTAGGAAATTTTGCCAATGGCTTCAT
AGCACTGGTGAACTGTATTGATTGGGTCAAGGGACAAAAGATCTTTTTAGTTGATGGAATTCTCGCTGCTCTGGCGGTCT
CCAGAATGTGTTTGCTTTGGATATCAGTAATACACTGGTATGCAACTGTGTTTTCTCCAGCTTTATGTAGTTCAGAAGCA
AGACTTATTATTGATGTGATCTGGATAGTAAGCAACCATTTTTGTGTCTGGCTTACAACTAGCCTCAGCATACTTTATTT
GCTCAAGATAGCCAATTTCTCCAGCCTTATATTTCTTCACCTAAAGTGGAGAGTTAGAAGAGTGATTCTCACGATACTGT
TGGGGACTTCGGTCTTCTTGGTTTTTCATCTTGTATTGGTAAGTATAAATAGAAAAATGTGGATGAATGAATGTGAAGGA
AACATCACCTGGAATATCAAATCGAGGGACATCGTGCCCCTTTCGTATATGACTGTATTCACGCTTGCAAACTTCGTACC
CTTTGCTATGTCCCTGATGTCTTCGCTGCTGCTAATCTTTTCCCTGTGGAAACATCTCAAGAAGATGCGGCTTAGTGGCA
AAGGATCCCAAGATCCCAGGACCGAGGTCCACATAAGAGCCATTCAAACTGTGATCTCCTTTCTCTTGCTATTTCTCATT
CACTTCCTGATTCTGATCTTTGCAGTTTGGTATTTTAATAGTCTGCAGAATGACTCAGTCTTCTTCAGTGGTCAGGTCCT
TGCAATCGTTTATCCTTCAGGCCACTCATTTATTCTGATTTGGGGAAACAAGAAGCTAAAGCAGGACTTTCTCTCAGTTT
TACGGCAGGTGAAGTGCTGGCTGAACAAATGGAAAGCTCAACGCCATAGGTCAATAAGAGGTGCATCGTTTGTGTTCTAG
>PtvaTAS2R372_KN526254.1:260941-259972
ATGTCAAGTGCAATTGAAAATGTTTTTATAATCATTGAACTTTTGGAATTTATAACAGGTATTTGGGGAAATGGATTCAT
CATACTTGTAATCTGTGCTGACTGGGTCAAAACCAAGAAAATCTCCCTGTTAGATTTCATCTTCACAAGCTTGGCCATCT
CCAGGATCGGCATGATATGCATGCTTTTTGAAGATAGCCTTAAAACAGTGTTCTACTCAGGAATATTTGAAAATCACCTA
ATGATGATAGTAGTTAGTGATTTCTTCTGGGATCTGAACAACAACATCAGTACCTGGTGTGCCACCTCCCTCGGTGTCTT
CTATTTCCTCAAACTGTCCAATTTTTCCCACCCCTTCTTTCTCTGGCTGAAGTGGAGACGAGATAGAGTTGTCATCACCA
TTCTGTTGGGTTTCTTTCTCTCTTTGTTTGTTGATCTTCTGAACATAAAATTTGATGCTTTTAAGGTCAGCGAATATTTA
AAAACAGAAAGAAACTGGACTTGGAAAGAATATATGCGTAAAACCCAATACTTTAACAATAAAATTCTCTTGAACCTGGG
ATCTCTCATTCCCATGGTTGTGTCGCTAATCTCTTTTTTCCTGTTAATCCTTTCCTTATGGAGACATATCCGGCAGACGA
TGCATTACGCCAAAGGATCTGGAGACTTTAACACAGAGGTTTATGTGAGAGCCAGAAATACTATGATTTCTTTCATCATT
CTCTTGGTTGTGCACTATTTTTTCACTATCCTGTTACTTTGGTCCTACTCCACAAGAGAAAACTCACTAAGTATGATTAT
CTGTGAGACTGTAGTATTGCTGTATCCTTCAATTCACCCTATCCATATGATCCTGGGGAACAGAAAACTGAGACGGACTG
CTGTGAATTTGCTAAGGCAAATGCGTCCTGCATCAAGGGAACGTGATTCTTCACAGCACGCAGGAACTGAGAACTATTGA
>PtvaTAS2R2P_KN525721.1:7286579-7285672
ATAAGCTCTCTTTATCAGCTGTTCCTCATGCTACCATCATGTCAGCAGAATTTATTACACGGATTACAGTAAATGGATTT

CTTATAATCATCGACTGGAATGAATTGATCAAAAACAGAAGGCTAACGCCAATACAACTCCCTTTAATATGTATCGGGAT
GTCTAGATTTTGTCTGCAGGTGGTATTCATGGTACAAAGCTTTTTCTCTGTGTTTTTTCCAGTCTTTTATCTGACAAAAA
TTTATGATGCAGCAATGATATTCCTCTGGATGATTTTTTTAGTTCTATCAGTCTCTGGTTTGCCACCTGCCTTTCTGTAT
TTTATTGTCTCAAGATTTCAGGCTTCACCCAGTCCTATTTTCTTTGACCGAAATACAGGATCTCAGCGTTAATGCCTTGG
ATGCTTCTGGGAAGCCTGCTGGCCTCCGTGAGCACTGCAGCTCTGTGTCTCTACGTAGATTACCCTAAAAACGAAGAGGA
TGATGTCCTCAGAAATGCCATGCTAATGACTAAAATCAAGATAAGGCCCATTAACGAAGTGCTTCTTGTCAGCTTGACAC
TTGTATTTCCTCTAGCCATATTTGCAATGTGCACTTTTATGTTACTCATTTCCCTCTAGAAGCACATGCTTCGTATGCAA
AACGGATTTGGTTTTAGAAATACCAGCACAGATGTCCATATAAACGCCTTAAGAACAATGGTAACATTCTTTTTCTTCTT
TATTTCTTATTTTGCCACCTTCACAACAAATAACGACATTCACTATTCCTTGCAGAAGTCAGCGCTTCTTTGTGGTGAAG
GACATAATGACAGCTCATCCCTCTGGCCATTCTGTTATAATAATCTTGAGTAGTTCTAAATTCCGACAACCATTCAGGAA
ACTTCTCTGCCTCAAAAAGAATCAATAA
>PtvaTAS2R8P_KN526254.1:246297-245394
ATGCTCAGTATAGAAGATAATGTCTTATCATGATAATAACTGGAGAGTCTATATTTAGGAATTTTGGGAAATGGATACAT
TGGACTAGTAAACTGGATTGACTGGGTTAAGAAAAAAAAGATCTCCTCAACTGACTACATCTCCAGAATTCGTTTGCTCT
GTGTAATGGTGCTCAATGGCATCATAATGGTTTTATATCCAGATGTTTATAAAAATGATAAGCTAAAAATAGTTGATACC
TTCTAGACACTCACCCACGACGTCAGTATGAGAACTGCCACCTGCTTCAACGTCTTCCATTTCCTCAAGATAGCCAATTT
CTGCTACCCATTTTTCTTCTGGCTGGAATGGAGAGTTTAGAGGGTGGTTTGCTGGATCCTGCTGCAATGCTTGCCATTTC
TTTGTTAAGCAGCCTAATGCTAGCAATGAGACCTAATTATGATTTTGAGTTTAAAATTGCAAAACATAAGAGAAACTTCA
CTGAATTGTTTCATGTAAGTGTAATTCAATACTTCAACCTGTTGTCACTCTTTAACCTCATAGCGATTGCCCCATTTACT
GTGTCATTGATCTCATTTTTTCTTTTAATTACGTCCTTATGGAGACACACTAAGTGAATGAAACTGAATGTTACAGGCTG
TAGAGATCCCAGCACAGAGGCCCACGCAGGAGTCATGAAAACTGTTACTTTGTTTCTCTTCCTCTTTTTTGTATACTATC
TTGCTCCTCTTTTGGCAACATTTAGCTACCTTATGAAGGAAAGAAAGTTAGCTGTGATATTCGGAGAGGTTATAGCAATT
CTCTACTCCTCAGGTCTCTCACTTATTTTAATTATTGGAAATAACAGCTGAGGCAGGCATCTGTCAGGATGCTGAGGTGT
GGAAAAACAGCATGTGTGATGTAA
>PtvaTAS2R10P_KN526254.1:259002-258073
ATATCAGGGCTTCGCAGTGGAAGGCCTCCTCATTTTTATGGCAGTTAGTGAATCAATGCAATCAATACTGGGGATTTAAA
GAATGGATTTATTGGACTTGTAAACTGCACTGACTGTGTCAAGAATAAGAAGTTTTCTGTAATTGGCTTGATTCTCATTG
GCTTAGCTCCTTCAAGACTTTTTTGGATATGATAATAATTACAGATAGATTTATAAAGATTTTCTCTCCAGATATATATT
TATCTGGAAATCTAATTGAATATATTAGTTATTTATGGGTGATTATCAATCAATTAAGTATCTGGTTTGTCACCAGCCTC
AGCATCTTTTTATTTCCTAAAGTTACAAATTTTTCCCGCCACATTTTTCTCTGATCGAAGAGTAGAATCAACAGGGCTGT
TCCCTTTCTGATGGAATTCTTGCTCATTTCATGGTTACTTGCTTTCCTACAAAGTATAGAGATTAGTAATGATCATAGAA
TGAGTAATAGAAACACAACGTGGATGCTCAACATACATAAAAATAAATTCTTTGCTCAATCTGGGAGTCATTTTCCTCTG
TGCACTATCCTTGATTACATGCTTCTTGTTAATCATTTCCCTTTGGAGACGCAGCAGGCATTGCAATTGAATGTCACAGG
TTTCAGAGACTCCAACACAGAAGCACATGTGAAAGCAATGAAAGTTTTGATATCTTTTATCATCCTCTTTGTCTTGCATT
TTATAGGCATTGCCATAGAATTATTGTGTTTTCCTGTGTCAGAAAACAAATCGCTGTTTATTTTTAGTATGATAATCACA
TTCCTCTATCCATGGGGTCATCCTTGTGGTTATCTTAATTCTAGGAAACAGCAAGCTAAAGCAAGCCTCTTTGAAGGTAG
TACAGCATTTAAAGTGCTGGATGAAAGAGGAACCTCTCAGGACTGCATAG
>PtvaTAS2R12P_KN526254.1:268088-267145
AGGTCAAGCATACTAGAGACCTTGTTTATAGTCATTTTTATAGTAAAATTCATAACAGAAAATTTGGGGAATGGATTCAT
TTTACTGGTAAACAATATTAACTAGATCAGGAACTAGACGGTCTCCATGATTAATTTTTTTTCTCACCTGCTTGACCTTC
TCCAGGATGTTTTCTGTGTCTGTTGATTTTAGATATCTCTTTCTCTATGGTCTATGAGAAAATATTTCACTCTAAGAATC
TAATGCTAAGTTTTGACATCCTCTGGATGAGATCTAACTATTTCTATATGACATATATCATTTGCCTTTGTGTCTTCTAT
TTCTTTAAAATAGCCAGCTTCTCTGACTCCATTTTTCTCTGGATAAAATGGAAAATTCACAAGGTGCTTCTCATTATTAT
ACTGGGAGTGATGCTCTTATTCTGTATGTATCTCCTTTTGAAGAAAATATTAATTAATAGACTGATTGAGAATGGGTAAA

AATGGAAAGAAGCTTGACATTCAATTTTATAGAGAATATATGTGATTTTTTAACATATCAGATTCTCCTGAACATGGTGT
TCATCATCATCTTCGTAGTGTCATTATCCCCTTTTTTCTTTTAATCCCCTCCTTATGGAGCCATACTTGTCAGATACAGG
ATATATATTCTAAGGATTTTAGCTCGGAAGCCCATATAAAAGCTATGAAAGCTATGGTTCCATTCCTATTGCTCTTTATT
ATGTACTATTTTGAGCAATACCATGATAATGTTGACTCATTTTATTATAGACAATGAGATGGTAAAGATGTTTGACAACG
TGTTAGCATTTTAAAAATGCTTCTGGCCAATCGTTTGTACCAATTTAAGGAAACAGGAAACTGAAACAGGTCTCTCTCTG
TGTCCAGAGAAAGCCAAAGTGTGCCTGAAAGAAGATAATTTCTCATTCTTATAAATATATCTGA
>PtvaTAS2R13P_KN526254.1:283011-282115
ATGGTAAGAGCTTTGCATAGCTTCCTCATCATTTTAGTATATACAGAACTTATAACTGGCATTTTGGGAAATGGATTCAT
AACACTGGTGAACTGCATTGACTGGCTCAAGAAATGAAAGATCTCCTCAGCTGATCAAATTTTGACTGCTTTAGCGATCT
CCAAAATTTGTCTCATTTGGGTAGTAATGATGAGTTGCTTTTCAAAGGAGTTTCATCTATCTTCATACATAAACAGAATG
GAGATTATACCTATTAGTATTGTTGGGGTTTGTGCCAGTTATTTTAGCAACTGGTTTGCCACAAGCCTCAGTCTCTTTTA
TCTTTTCAAGATAACCAATTTTTTAAATTCTGTCTTTCTTCACCTAAAGCATAGAGTTGAAATGGTGGTTCTGGTAATGC
TGCTGGGAGCATTAGCATTCTTGCCTTTAAATATTATTATGGTAAACATGCATATTAATATGCAGATACATTCCTATGAA
AGAAATATGACTGAGTTCTAAACAGAGTAACAAGGAAATCTTTTCAAAATTGATTGTATTCACTACAGGATCTTTTGTAC
CCTTCTCAATATCCCTGAAATTTTTTATCTTGTTAATCTTCTCCCTGTGGAAACATCTCAAGAATATGAAGCATAGTGCA
ATGGGATTCAGAGATCCCATTGTCAAGGCCCATATAAGAGCTATGAAAAGTGTGATATTTTTCCTCTTACTATCTGTTGT
TTACTTTCTCATAGCAGTTTTCCATTCTGAGATGATGATACAGGATGAACTGGTCTTTTTGCTTAGTCAGGCTATTGCAA
ATGTTTATCCTTCAGTCCACTCATTTATCCTGATTCTGGGAAATGATAAGCTAAGAAAATTTTCACATTTGGTGCTGTGG
CAGCTGAAGAGTGTTGA
>PtvaTAS2R60AP_KN526046.1:369146-368238
ATGAGGATAGTGTGGTTAAGAGAGCCATCATCTTGGCTGTCGTTTTATTCATTTTGTGCCTGGAGGCTGTGGTGAGCGAC
AGCTTCATCACTGCACCACTGGGCATGGAGTGGTTGCTGTGGAGAGCAGTGTCATCTTGTGATAAGTTATCCATGCCAGA
GAGCCTGAGGGCCCCTAGCTTCTGTCCGCAGTGGGTGGTGATCAGTAAGAGCTTCTGTGTTTTCCTGTATCGAAGGGCCT
TCCTGTATGCCCTGGGCTCCAGCTCCTAGCCTTCCAGTGGGACTTCTTGAATGCTGCCACCTTATGGTTTTCTAGTTGAC
TCAGTGTCTTCTATTGTGTGAAAATTACAACCCTCGCTCACTCTGTCTTCCTTTGGCTAAAGCGGAAGGTATTTAGGGGG
TTCCATGGGTGTTGTTCAGCTCTGCGAGGATTCTCTAGCTTGAGCATCATCTTATTTTATATAGGCAACCAGAGCATATA
TCAGAACTATTTAAGGAGAGGTCCACAATCTTGGAATGTCACTGGGAATACTATCAGAGAACTTATGAGAAATTCTACTT
TTTCCCTCTAAAAATGGTTACCTGGGCAGTCTCTGCCATCATCATCCTCATTTGCATGGTTTAGCTCGTCACATCTCTGG
GAAGGCACGCCAAGAAGGCCTTCCAGTCCATCTCAGGCTCTCACACTCCGGCGCCCAAGCACACATCAGGGCTCTCCTGG
CTCTCATCTCCTTTGCCTCCTCTTTCCTGTCACCGATGCTCAACGCGGCAGGTGGTTCCCCATCTCAGGACTGGGTGTGC
AGGCAGTGTTTTATCGGTGCACAGCGGCCCACCCCATCCTACTGGGAGCAGCCCCCAGGCTGAGAGCTGTGCTGGGCAGG
ACTGCTTCTTAAGGCGTGTGGCATCTTGA
>PtvaTAS2R60BP_KN526046.1:366282-365374
ATGAGGATAGTGTGGTTAAGAGAGCCATCATCTTGGCTGTCGTTTTATTCATTTTGTGCCTGGAGGCTGTGGTGAGCGAC
AGCTTCATCACTGCACCACTGGGCATGGAGTGGTTGCTGTGGAGAGCAGTGTCATCTTGTGATAAGTTATCCATGCCAGA
GAGCCTGAGGGCCCCTAGCTTCTGTCCGCAGTGGGTGGTGATCAGTAAGAGCTTCTGTGTTTTCCTGTATCGAAGGGCCT
TCCTGTATGCCCTGGGCTCCAGCTCCTAGCCTTCCAGTGGGACTTCTTGAATGCTGCCACCTTATGGTTTTCTAGTTGAT
TCAGTGTCTTCTATTGTGTGAAAATTACAACCCTCGCTCACTCTGTCTTCCTTTGGCTAAAGCGGAGGGTATTTAGGGGG
TTCCATGGGTGTTGTTCAGCTCTGCGAGGATTCTCTAGCTTGAGCATCATCTTATTTTATATAGGCAACCAGAGCATATA
TCAGAACTATTTAAGGAGAGGTCCACAATCTTGGAATGTCACTGGGAATACTATCAGAGAACTTATGAGAAATTCTACTT
TTTCCCTCTAAAAATGGTTACCTGGGCAGTCTCTGCCATCATCATCCTCATTTGCATGGTTTAGCTCGTCACATCTCTGG
GAAGGCACGCCAAGAAGGCCTTCCAGTCCATCTCAGGCTCTCACACTCCGGCGCCCAGGCACACATCAGGGCTCTCCTGG
CTCTCATCTCCTTTGCCTCCTCTTTCCTGTCACCGGTGCTCAACGCTGCAGGTGGTTCCCCATCTCAGGACTGGGTGTGC
AGGCAGTGTTTTATCGGTGCACAGCGGCCCACCCCATCCTACTGTGAGCAGCCCCCAGGCTGAGAGCTGTGCTTGGCAGG

GCTGCTTCTTAAGGTGTGTGGCATCTTGA
>PtvaTAS2R62P_KN526046:374569-373651
ACGCCCTCCTTGCCCACGGTGGTCTCCAAGGCCACCTTCTTCCTGGAGTCGTGGGCTGCAGTGCGGCTGAATGGCTTCGT
GGTGGCTGTGCTAAGCAGGGAGCGAGTGCAATGTCAGACATTGCCCTCAGGCGACCTGACTGTGGCCGGCCTGGCCACCT
ACCGGTTCTGCCTGCATGGGATGGCCCTCCTAACAAACCTCCTGACCTCCTTTGAATTTTGTTCCAAAGTTTACTCTTTC
AACATCCCTTGGTCCTTTATCAGCACCCTCACTTTCTGGCTGACTGTCTGGCTGGCTGTCTTCCACCGTGTGAAGACCTC
ATCCTTCTCTCTCCTACATCTTCTTCTGGCAGAAGAGGAGAACTTCTCGGTCAGGGCCTGGGCCATCGCTGGGCTCCCTG
ATCATATCTGGTCTGACAAGCATCTCATCAGCCACTTGGAATATAGTTCTGGTGCAGGTCATTGCCGCCCAGTTCCCATG
AAAACAGCGCCCTGGCTGATAGCGTACAGACCTTCCGTGGGCACGTTTTCCTTCCTCATGTAGTGCTTGTGACATCGATC
CCGTTCCTCCCGTTCCTGGTGTCCCGTTTTCCTGCTCATGTTCTCATCACACTGGCACTTGGGGCAGATGAGGGACCACA
GGGCCCGGGGCTCACACCGTGGCCCTGAAATCCCTTGCCTTCTTCCTCATCTTCTGCACCTCGTAGTTCCTGTCCCTGGT
TATCGTTGCTGTGAAAATCACAACCTTCCGGAATGACTGGCACTGGGCCTGGGAAGTGGTGACTTACGCAGGCGTCTGTC
TGCACTCCAGCGTCCTGGTGCTGAGCAGCCCCACGCTTAGAAAGGCCCCGAAGACAAGGCTCTGGGAAGCCCTGGGCAAA
GGGCGTTTCGTCTCGAGTTATCAGTGTCAACAACCACTG
>PtvaTAS2R18P_KN526254.1:307394-306420
ATGTCAGTTGGAATGGAGGTCTCCTTTCTGGTAGTGGCAACAGGAGAATTCATCTTGGGAATGCTGGGAAATGGGTTTAT
TGAACTGGTAAACTGTATCGAATGGGTCAAGAGTGGGAAGGTCTCCTTGGCAGATTTCGTCCTCACCAGCTTGGCTCTGG
CCAGGATCATTCAACCGTTCATAACACTATTGGATTCATTCCTAATAGGGCTATCTCCACATCTGTATGCTATTGGTAAA
CTAGTGAAAGTGGTTACTATTCTTTGGGCACTAACTAATCACTTAACAACCTGGTTTGCCACCTGCCTAAGCATTTTCTA
CTTCCTTAAGAAAGCCAGTTTTTCCTACTTCTTTTTCATCTGGCTGAAGTGGAGAGTGGACAGTGGTTCTTATGCTTTTT
CTGGTGTCTTTCTTCTTATTGTCTATTATATCTCTTAATGCAGGATGCTCTTAGTGAGTTGTAGTTGAATACTTATAGAG
TGCATGAAAGGAACATGACTTTGCATTTAGATGAAAATAAAATTTTCTACCTTAAAAGACTTCTTCTTCTTAGCTTGACC
TGTGTTATCCCCTTTCTTCGGTACCTGATGTCTTCACTCCTTTTATTTCTCTCCTTGTTGAGACACACCAAGAATTTACG
GCTCAACCTGATAGGATCAAGGGACTCTGGCACAGAGGCCCGCAGGAGGGCCATGAAAATGGTGACCATCTTCCTCCTCC
TCTTCATCATTTACTTTATTTCCACTCTAATAGCAAGTTGGATCTTTACTAAGGTACAGAGCTATTAGGCCATGATGCTG
ATCTCAACTATCTTTCCCTTGAGTCACTCATTTATTGTAATTTTGGGAAACAGCAAGCTAAGACAGATCACCTGGAGACT
ACTGTGGCATCTTAAATTCTCTGATAAAAGCAAAACCTTTAGCTTCATGAGCAAAATTTGAAAGAACTTTGTGTATTCTA
TGGGACAGACCTTAA
>PtvaTAS2R67P_KN526254.1:332439-331515
ATGTCATCTGGAATAGAAAATATTTTTCTGATAGTGGCAAAAGGAGAATTCATAGCTGGCATGTTGGGGAATGGCTTCAT
TGCACTAGTTAACTGCACTGACTGGGTGAAAAGTCAAAAACTCTCCGTAGACAATGGCATCCTCACCAGCTTGGCTATTT
CCAGAATAACTCTTCTTTTGATAGTACTGGTTGATTTACTTCTAACGGCGTTATGGCTACATCTATATGCCATTGGTGAA
CGAGCAAAATTTATTAGTATTTCTTGGGTACTGTCCAATCACCTAGCTACCTGGTTTGCCACCTGACTAAGTGTTTTCTA
CCTCTTGAAAATAGCCAGTGTCGCTCACCCCTGTTTTGCCTGGCCGACGTGGAGAATTAGCAGAATGCTACTTGTGCTTC
CACTGGGGTTTTTGTTCCTACTGTTTTTCAACATCGCATTAACAGACACATTAAATGATTTCTGGGTTAACGCCTATAAA
ATATATGAAAGAAACTCAATGTGGTCTTTAGATGTGAGTAAAATCCTGTATTTTAACACCTTGATTGTTTACGATTTTAT
CTACTTAGTTCCCTTTCTTCTGTCCCTGACTTCACTGCTCCTTTTATTTCTCTCCTTGAAGAGACACATCAGGAACACGC
AGCTGAACTCCAGCTCTGGTGACTTTAGCACAGAGGACCATAAGAGGGCCATGAAAATGGTGATGTCTTTCCTCCTTCTC
TTCAAACTTCATGTTTCTTCCACTCTATTTCCTTATACTGCAGAGAGATCAGGCCAACGCGTTCGCCAAGTTCACATTGA
ATATTTTTCCTTCAGGTCACTCATTTATCCTAATTTTGGGAAACAGCAAGCTGAGACAAACTGGCTTAGGACTACTGTGG
TATCTTAATTGCCACCTGAAAAGGGTGAAACCTTTAGCTTCATAG
>PtvaTAS2R408DP_KN526254.1:311460-310539
ATGATAAGTTTACTAATTATGAGCATTCTTTCCATCTTTGTAATAGCAGAATTTGTTCTAGGAAATTTTGCCAATGGCTT
CCTAGCACTGGTGAACTGTATCAACTGGGTCAAGGGACAAAAGATCTCCTTAGCTGATGGAATTCTCACTGCTCTGGCGG

TCTCCAGAATGGGTTTGCTCTGGGCATTAGTAATAAATTGGTATGCAACTGTGTTTAATCCAGCTTTATATAGCTCAGAA
GTAAAAATTATTGTCTATATGATCTGTACAGTAAGCAACCATTTTAGTGTCTGGCTTACAACTAGCCTCAGCATACTTTT
TTTGCTCAAGATAGCCAATTTCTCCAGCCTTATATTTCTTCACCTAAAGTGGAAAGTTAAAAGAGTAATTCTCATGATAC
TGTTTGAGACTTTGGTCTTCTTGGTTTTTCATCTTGCAGTTGTAAACATAGATGAAAATATGCAAATGAATGAATGTGAA
GGAAACATCACCTGGAATACCAAATCGAGGGACATGGTGCACATTTCAAATATGACTGTATTTATGCTTGTGAACTTCGT
ACCCTTTACTATGTCCCTGATTGATGGCTTTGCAGCTGCTAATCTTTTCCCTGAGGAAACATCTCAAAAAGATGTAGCTC
AGTGGCAAAAGATCCCAAGATCCCAGGACTGAGGTCCACATAAGAGCCATTCAAACTGTGATCACCTTTCTCTTGCTTCT
TGCTATTTACTTCCTGACTCCGATCTTCTCAGTTTGGAATTCTAATATGATGAGGAACAAAACAGATTTCCTGCTTTTCA
GAGTTCTTGAAATCCTCTCAATAAACCACTCATTTATCCTGATTTGGGGAAACAAGAAGCTAAGATAGGCCTTTCTGTCA
TTTCTGTAGCAGCTGAGGTGCTGGCTGAAAGAAAGGAAGTAA
>PtvaTAS2R408CP_KN526254.1:312962-312041
ATGATAAGTTTACTAATTATGAGCATTCTTTCCATCTTTGTAATAGCAGAATTTGTTCTAGGAAATTTTGCCAATGGCTT
CCTAGCACTGGTGAACTGTATCAACTGGGTCAAGGGACAAAAGATCTCCTTAGCTGATGGAATTCTCACTGCTCTGGCGG
TCTCCAGAATGGGTTTGCTCTGGGCATTAGTAATAAATTGGTATGCAACTGTGTTTAATCCAGCTTTATATAGCTCAGAA
GTAAAAATTATTGTCTATATGATCTGTACAGTAAGCAACCATTTTAGTGTCTGGCTTACAACTAGCCTCAGCATACTTTT
TTTGCTCAAGATAGCCAATTTCTCCAGCCTTATATTTCTTCACCTAAAGTGGAAAGTTAAAAGAGTAATTCTCATGATAC
TGTTTGAGACTTTGGTCTTCTTGGTTTTTCATCTTGCAGTTGTAAACATAGATGAAAATATGCAAATGAATGAATGTGAA
GGAAACATCACCTGGAATACCAAATCGAGGGACATGGTGCACATTTCAAATATGACTGTATTTATGCTTGTGAACTTCGT
ACCCTTTACTATGTCCCTGATTGATGGCTTTGCAGCTGCTAATCTTTTCCCTGAGGAAACATCTCAAAAAGATGTAGCTC
AGTGGCAAAAGATCCCAAGATCCCAGGACTGAGGTCCACATAAGAGCCATTCAAACTGTGATCACCTTTCTCTTGCTTCT
TGCTATTTACTTCCTGACTCCGATCTTCTCAGTTTGGAATTCTAATATGATGAGGAACAAAACAGATTTCCTGCTTTTCA
GAGTTCTTGAAATCCTCTCAATAAACCACTCATTTATCCTGATTTGGGGAAACAAGAAGCTAAGATAGGCCTTTCTGTCA
TTTCTGTAGCAGCTGAGGTGCTGGCTGAAAGAAAGGAAGTAA
>PtvaTAS2R1BT_ABRP02205990.1:463-1335
ATGCTAGAATCTATTGTCATTACCCATCTTCTTTTTGTAATGATACAACTTCTCTTCGGGGTCTTAGCGAACGGCGTGAT
CGTGGTCGTGACCGGCACGGAGCTGACCAGGCCCAGAAAGATGGCGCCTCTGCACCTCCTTCTCTGCTGCCTGGCTGTTT
CCAGAATTTGTCTCCAGATGTTCATCTTCTACAACAGTCTGGTCGTTCTCTCCCTCATCGAATTCTTCTCGCTTGCCGAG
ACCTATACGGCGTTCATGTTTTTTAGTGAACTGTCGCTTTGGCTGGCCACATGGCTCAGCGTGTTCTACTGTGCCAAAAT
CGCCACCTTCGCTCACCCTCTCTTCTTCTGGTTGAAGCTGAGGATTTCGAGGTTGGTGCCATGGCTGATTGTCGGGTCCT
TGCTCTACACGTCTCTCACTTCTGTTTTCCACAGAAAACATACGTGGATTCTTTCCCAAAAACTCTGGCTGAGCCTTTTC
TCTCAGAATGCCACCACTCAAGGCAATGAAATGTCCACGTTACACTTTGCCATTCTGGTCGTTGAGTTCTTCTTGCCACT
ATTTATCTTCCTACTTTCTGCTCTGCTCCTGATATTCTCCCTGGGGAGACACGCCCAGCAGATGACGAGCATGGCGGCGG
TCAGTGGGCACACGGGGACGAGCGTCTACTTCAGCAAGCTTCTATCCGTGCTGTCCTTTCTGGTCCTCTACGTCTCTCAG
TATGTGATGGCTGCTTTAATCTTTTCTCAGAATTTCAAGATCAAGAACTTCGCCTTTCTGCTCTGCTTCTTGGTGCTTGG
TTTGTACCTCTCTGGGCACTCTATTATCTTAATTTTTGCAAGTCCCAAACTGAAACAAAATGCAAAGAAGCTC
>PtvaTAS2R3BT_KN525765.1:263944-263253
TTTTCTGGGCATTTACAAACCATCTGAGCATTTGGCTTGCCACCTGTCTCGGTGTCTTCTACTGCCTGAAAATCACCAGT
TTCTCCCACCCCACCTTCCTCTGGCTCAAGTGGAGAGTTAACAGCGTGGTTGTGTGGATGCTGCTGGCTGCGTTGCTCCT
ATCGTGTGGCAGTGCTGTGTCTCTGATTCACAGTTTTAAGGTCTATTCTGCCCTCTGTGGAATCGATGGCACAGGGAATG
TGACTGAGCACTGTAGAAAACTAACGAACGAGTACCAAGTGACCCATGTTCTTGGCACCCTGTGGAACCTCCCGCCCCTC
ATCATGTGTCTGGCCTCCTACATCGTGCTCATCCTCTCCCTGGGGAGGCACGGGCGGCGGATGCAGCACAACAGCGCCAG
CTCCAGCGATGCCAGCACTGAGGCCCACAAGAGGGCCACTAAAATGATGCTCTCCTTCCTCTTCCTCTTCCTGCTTTACT
TTCTTGCCTTTTTAATTACAACATCCAGTTATTTCCTACCAGGAACTAAGATGACTCAGATGATTGGAGAACTAATTACA
ATGATTTATCCTGCCAGCCACTCGTTTGTCTTCATTCTGGGAAACAGCAAGCTGAAGCAGACATTTGTGGAGATGCTGTG

GTGTGAGTCTGGTCGTCTGAAGTCTGGATCCAAGAGACCCTTTTCCCCATAG
>PtvaTAS2R39T_KN526046.1:558795-557978
ATGACCAAAACCTGCAGTCCCCCAGAAAATGAGTTGTCATCATTCCGCGTCATCTTAACTTTCACAATTATAGGCACTGA
ATGCGTTATTGGTATTGCTGCAAATGGGTTCATTATGGCTGTAAATGCAGCAGAATGGATTCAGAATAAGGCAGTTTCTA
CAAGTGGCAGGATCCTGTTTTTCCTGAGCGTATCCAGAATAGCTCTTCAAAGCTTCATGATGCTAGATATTGCCTGCAGC
TCAAAATTACCACGCCTTTATAATAAAGGTATTTTATATAATACATTCAAAGTAAGTTCCATGTTCTTAAATTATTGTAG
CCTCTGGTTTGCTGCCTGCCTCAGTTTCTTCTACTTTGTGAAGATTGCCAACCTCTCCCACCCACTTTTCCTCAAGCTGA
AGTGGAGAATTGCTAAGCTGATGCCCTGGCTTCTATGGCTATCGATATTCATTTCCTTGGGCTACAGCAGTCTCTTCTGG
AAAGGCATCTACACTATACACTGTAACGACTCATTTCCTGGCGCCTCGTCCAACTCCACTAAGAAAAAATACTTCACTGA
GACCAACGTGGCCAACCTGGCTCTTCTCTACAACCTGGGGGTCTTCATTCCTCTGACCGTGTTCATCGTGGCGGCCACCC
TGCTGATCATCTCTCTCAGGAGACACACGCTACACATGGAGAGCAAGGGCACCGGCTCCGGGGACCCCAGCATGGAAGCT
CACATGGGGGCCATCAAGACTATCAGCTACTTTCTCATTCTCTACATCTTCAACGCGGTTGCTCTGTTTCTGTCCATGTC
CAACATCTTTGACGCCAA
>BaacTAS2R16_NW_006726354.1:16155113-16156015
ATGATAACCATCCAACTCTCCGTCTTCTTCATGATCATCTATATGCTCAAGTTCTTGACAATAACTGCACAGAGCAGCTT
AACTGCTGTAGTGCTGGGCACCGAGTGGGTGAGTTTCCAAAGGCTGTCACCAGTGGAAATGATTCTCACCAGCCTGGGTG
TCTGCTGCTTCTGTCAACTGTGGTCATCGATGCTGTACAACTTTTGCTCCCACTTCCACCCTAGTTATGAATTTTGGTAC
TTCAGGATCGTCTGGGAATTTACTAACATTCTTTCATTCTGGTTGACCAGCTTGCTTGCTGTCTTCTACTGTGTCAAAGT
CTCCTCCTTCAGCCACCCCACCTTCTGGCTGAAGTGGAGAATTGTGAGGTTGGTTCCTCGGCTGTTGCTGGGTTCTCTGC
TGATTTCTTGTGTGTCTATCATCTTTGCAGCTGTTGGGCATTACAGCAAGATTCAATTAATCTCCAAGAGGCATTTCCCT
AGAAACAGCACCACGACTGAGAGACTTGAGATATTCCTGTGGGATTTTTCCATGTGCCAGCAAGTGGTTGTGTTGATTAT
TCCTTTCCTCCTGTTCCTGGCCTCCACCGTCTTGCTCATGGCCTTATTATTCCAACACCTGAGGCAGATGAAAGATCATC
ACACCAGCCACTCCAACTCCAGCCTGGAAGCTCACTCTACTGCCCTGAGGTCTCTTGCCATCTTCCTCATTTTCTTCACC
TCTTATTCTCTGACCCTACTAATCTCCATCTGGGGTGTCCTTTTTAATAAGGGGTCCTGGTTCTGGGCCTGGGAAGCTAT
CATCTATGCTCTAGTCTCTATTCATTTGACTTCACTGATGCTGAGCAGCCCTAAATTGAAAAGGGTTTTAAAGGTAAGGT
ATTGGGACCTAGAGGCTGCCTGA
>BaacTAS2R1P_NW_006728240.1:158717-159613
ATGCTGGAGTCTCACCTCACTAGCCACCTTTGTTTGGCAGTGATACAATTTCTCGTTGGGGTTTAGTAAATGGCATCAT
TGTGGTTGTGAATGGCACTCACTTGATCAAGCAGCGAAAGGTGATTCCATTGGATCTCCTTCTTTCCTGCCTGGCGACTT
CCAGGATTTGTCTGCAGCTAGCCATCTTCTATGTTAACCTGGCTGTCCTTTCCTTGATTGAATTCCCTCAGCTTGCTGAG
AAGTTCATAATTCTCACATTTATAAATGAATCGGGACTTTGGTTTGCCACATGGCTCAGCCTTCTCTGGTGTGCCAAGAT
GGCCACCATTGCTCACCCACTCTTCTGGTTGAAGATGAGGATATCCAAGTTGGTTCCTTGGCTGATACTTGAGTCCCTGC
TATATGCATCTAGTACGGCTGTTTTCCACAGCAAACACAGATGGATATTTTCCAAAGAACACTTCCTGGGCCTTTTCTCC
CCAAATGCAACCACTCAAATCAAAGAAATACCTGCTTTACAGTTTGCCTTTCTTTTTGCTGAGTTCTCATTGCCATTACT
TATCTTCCTTATTTCTTCTCTGCTCTTGATATTTTCCCTGGGGAGACAGATCTGACAGATGAGAAACACAGCAACAGGCC
CCAGGAACCCTCGCACACGCGTGCACATCAGCCCTCTTCTCTCCATCCTGTCCTTTCTGGTCTTCTATCTCTGCCACTCC
ATGACAGCTGCTTTGCTCTATTCTCAAATTTTCAACTTTAGAAGCTTCATATTTCTGTTCTGCATCTTGGGGGTTGGTTC
ATACCACTCTGGACACTCTATTACCTTAATTTTAGGAAATCCTAAAATGAAACAAGATGCAAAGAAATTGCTCCTTTACG
GAAAGTGCTGTCAGTGA
>BaacTAS2R2P_NW_006724571.1:7313574-7312366
ATGGCCTCCTCTTTGTCAGCTTGTCTTCATGTTATCCTCATGTCAGCAGAATTTATTACAGGGATTACAGTAAATGGATT
TCTTATAATCATCAACTGTAATGAATTGGTCAAAAGCAGAAAGCTAACACCAATGCAACTCCTTTTCATATGCATAGGCA
TGTCTAGATTTGGTTTGCAGATGGTGTTAATGGTAAAGTTTTTTCTCATGTTCTTTCCACTCTTTTTTTTTTTTTTTAAA
GATTGATTGATTGATTGATTGCTATGTTGGGTCTTCGTTTCTGTGCTAGGGCTTTCTTTAGTTGCGGCAAGCGGGGGCCA

CGCTTCATCGCGGTGCACGGACCTCTCACTGTCGTGGCCTCTCTTGTTGCGGAGCACAGGCTCCAGACGCGCAGGCTCAG
TAGTTGTGGCTCACGGGCCTAGTTGCTCCGCGGCATGTGGGATCTTCCCAGACCAGGGCGCGAACCCGTGTCCCCTGCAT
TAGCAGGCAGACTCCCAACCACTGCACCACCAGGGAAGCCCCTTTCCACTCTTTTATAGAGTAAAAATTTATGGTACAGC
GATGATTTTTTGTGGATGTTTTTCAGCTCTGTCAGTCTCTGGTTTGCCACCTGTCTCTCTGTATTTTACTGCCTCAAGAT
AACACACTTCACCCAGTACTGTTTTGTTTGGCTGAAATTCAGGATCTCAAAGTTAATGCCTTGGCTGCTTCTGGGAAGCC
TGTTGACCTCCGTGAGCATTGCAGCTCTGTGTGTCAAGGTGGATTACCCTAAAAATGTGGATATTGATGTCCTCAGGGAT
GCCATGCTAAAGCGGACTAAACTCAAGACAAAGCAGATTAATGAAGTGCTTCTTGTCAACTTGGCATTAATATTTCCTCT
GGCCATATCTGTGATGTGAACTGTTATGTTATTCATTTCTCTCTATAAGCACGCTCATCGGATGCAAAATGGACCTCTTG
GTTTTAGAAACGCCAGCACTGAAGCCCATATTAATACATTAAGATCAGTGATAACATTCTTTTGCTTCTTTATTTCTTAT
TTTGCTGGCTTCATGGCAAATATGACATTCAGTATTCCTTATGGGAGTCAGTGCTTCTTTGTGGTTAAGGACTTAATGGC
AGCATATATCCCTCTGGCCATTCGGTTATAATGATCTTGAGTAATTCTAAGTTCCAACAACCAATCAGGAGACTTCTGTG
CCTCAGAAAGAATCAATGA

>BaacTAS2R3P_NW_006726354.1:421966-421019

ATGCTGGGACTCACTGAGTGCGGGTTTCTGGTTCTGGCTGCCACTCAGTTCATTCTGGGAATGCTGGGGAATAGTTTCAC
GGGCTGGTCAATGGTAGCAGCTGGTTCAAGAACAAGAGAACCTCTTTGACTTCCTCATCACTAACCTGGGTCTCTCCAGG
ATTGTTCTGCTGTGGATTCTCTTGATTGGTGGTGTTTTAATGGTGTTCTCTTCCAAACTCCACGATGAATAATTTGCAAT
CATGCAAATTAGTGATATTTTCTGGACATTTACAAACCGTCTGAGCATTTGGCTTGCCACCTGTCTCAGTGTCTTCTACT
GCCTGAAAGTCGCCAGTTTCTCCCATCCTACGTTCCTCTGGCTCAAGTGGAGAGTTTCCAGGTTGGTTGTATGGATGCTG
TTGAGTACCCTGCTCTTATCATGTAGCAGTGCCGTCTCTCTGATCCATGAATTTAAGACCTATTCTGTTCTCAGTGGAAT
TGATGGAACGGGGAATGTGACTGAACCCTTTAGAAAGAAAAGAAATGAATATAAACTGATCCATGTTCTTGGGACTCTGT
GGGACCTCCCTCCCTTAATTGTATCTGTAGCTTCCTACTTTCTGCTCATCCTCTCCCTGGGGAGGCATATGCGGCAGATG
CAGCAAAACTTTACCAGCTCCAGAGATCCAAGTACTGAGGCCCACAGGAGGGCCATCAAAATCATCCTTTCTTTCCTCTT
TCTCTTCCTACTTTACTTTCTTTTCTCTTCAATTTTGACATCCAGTTATTTCTACCAGCAACTGAGGTGATTATGATGAC
TGGAGAAGTAATTACAATGTTATATCCTGCTGGCCGCTCATATATTCTCATTCTGGGAAATAATAGGCTGAAGCAGATGT
TCATGGAGATGCTTTGGTGTGAGCCTGGTCATCTGAAGCCTGGATCCAAGGAACCCGTTTTTCCATAG

>BaacTAS2R31P_NW_006733678.1:4233428-4234311

ATGTTAACTTTACTACTGGACATTTTTTCCATTCTAGTAATGACAGAATTTGTTCTGGGAAATTTTGCCAGTGGCTTCAT
AGCACTGGTGAACTGCATTGACTGGGTCAAGAGACAAAAGGTCTCTTCAGCTGATGGAATTCTAACTGCTCTGGCAGTCT
CCAGAATTGGTTTGCTCTGGGTAATTTTACTAAATTGGTATTTAATTATGTTTAATCCAGTTCTACATAGTTTAAAAGTA
AGAATTATTGTTTATGTTGCCTGGACAGTAAGCAACCATTATAGCATCTGGCTTGCTACTAGCCTCAGCATATTTTATTT
GTTCAAGATAACCAATTTCTCCAGCCTAATTTTTCCTCACCTGAAGTGGAGAGTTAAAAGTGTAGTTCTCATGCTGATGT
TGGGGACTTCTTTCGTTTTGGTTTTTCAAGTTGTGGTGGTAGCATAAATGAGGCTATCCAGACAAATGAATACGAAGGAA
ATATCACTCAGAAGACCAAATTGAGGGACATTTTACACCTTTCAAATTTGACTCTGTTCACGCTAACAAACCTCACACCC
TTTACTATGTCCCTGACAACTTTTCTGCTGCTAATCTTTTCCCTGTGGAAACATCTCAGGAAGATGCAGTTCAATGGCAA
AGGATCCCAAGATCCCAGCACCAAGGTCCATATAAAAGCCATGCAAACTGTCATCTCCATTCTTTTGCTATTTGCCATTT
ACTTCCTGGCTCTAATCGTATCAGTTTGGAGTCCTAATGAGCTGCAGAAGGAATGGTCCTCATGCTTTTCCACACTCTTG
GAACCAGTTGTCCTTCAGTCCACTCATTTATCCTGATTTGGGGAAAGAGGAAATTAACACAGGCCTTTTTGTCATTTCCG
TGGCAGCCAAGATGCTGGCTGAAAGAAAGGAAATAA

>BaacTAS2R38P_NW_006726354.1:349295-350405

ATGGTGACTCTGACTGCCATCGTAACTGTGCCCTATGAAGTCAGGAATGCATTTCTGTTCTTTTCAGTCCTGGAGTTTGC
AGTAGGGATCCTGGTCAATGCCTTCATTTTCTTGATGATCTTGTCCTGCNNNNNNTGCTGAATCTCAGCCTCACCTGGCT
CTTCCTGCACGGGCTGCTCTTTCTGGATGCCATCCAGCTTACCCACTTCCAGTGGATAAAAGACCCGCTGAGCCTCTGCT
ACCAGACCATCCTCATGCTCTGGATGCTCGTAAATCAAGCTGGCCTCTGGCTCACCACTTGCCTTAGTCTCCTCTACTGC
TCCAAGACTGTCCATTTCTTTCACACCTTCCTCCTCCGCTTGGCAAGCTGGATCTCCAGGAAGATCTCCCAGATGCTCCT

GGGTGCTATTTTTTCCTCCTGTGTCTGCATTGTTCTCTATTTGTGGGACTTTTTCAGTAGATCTCCCTTCTCAGTTGCAA
CCATGTTACTCATGAATAACAATACAGAACTCAACTGAGAAATTGAGAAAACTCAATTTCTTTCATTCCTTCCTCTTCTG
CAGCCTGGGGTCCATCCCTTCTTTCTTGCTTTTTCTGGTCTCTTCTGGGGTGCTGATTGTCTCCCTGGGGAGGCACATGA
GGACAAGGAGGGCCAAAACCAGACAGTCTCGGGACCCCAGCCTGGAGGCCCATATCAAAGCACTCAGGTCTCGTCTCTTT
CTTCTGCCTGTATGTGGTGTCCTTCTGCGCTGCCTTCATCTCGGTGCCTTTGCTGATGCTGTGGCACAACAAGATCGGGG
TCGTGGGCTGTGCAGAGATACTGGCAGCCTCCCCTCGGGGCACACAGTCATCCTGATCTCAGGCAATGCCAAGCTGAAGG
GAGCTGTGGAGACCATTCTTCTCCGGGCTCAGAGCAGCCTAAAGGTAAGGGCGGACCGCAAGGCAGATCCCAGGATGCCA
GATCTATGTTGA
>BaacTAS2R39P_NW_006727908.1:5592836-5591779
ATGAATAGTGGGAGCTATCACAAATCTGCCCATCACACGCTAGGGAGACATTTTCCTCAGACACCAAAGAGAAGCAACAA
CTCAGGATGACCGAAACCTGCAATCCCCCAGAAAATCAATTGTCACCATCTCGCATCATTTTGATGTGAATATTTATAGG
CACTGAATGCGTCCTTGGTATCACTGCAAATGGGTTCATTGTGGCTATAAATACAGCAGAATGGATTCACAACAAGGCAG
TTTCCACAAGTGGCAAGATCCTGCTTTTCCTGAGTGTATCCAGAAGAGCGCTACAAAGGTTCATGATGCTAGAACTCACC
TTCAGTTCAACATCCCCACAGTTTTATAATCAAGACATTCATTGTATATGATACATTCAAAGGAAGTTTCATGTTCATAA
ATTATTGTAGCCTCTGGTTTGCTGCCTGGCTTAGATTCTTCTACTTCGTGAAGACTGCGGATTTCTCCTACCCCCTTTTC
CTCAAGCTGAAGTAGAGAATTTCTGGATTGATGCCCTGGCTTCTGTGACTATCAGTGTTTGTTTCCTTGGGCCACAGTGT
GTTCTTCCTCAAAAACATCTACACTGTGCATTGCAACAATCCTTTTTCTAGCCCTTCCTTCAACTCCACTAAGAAAAATT
ACTTTTCTGAGACCAGTGTGATCAGCCTGGTTCTTTTCTGTAACATGGGCATCTTCGTTCCTCTCATCACGTGCATCCTG
GCTGCCACCCTGCTGATCATCTCTGTCAAGAGACACACCCTAAACATGGGAAGCAATGCCACTGGCTCCAGGGACCCCAG
CATGGAGGCTCATGTGGGGACCATCAAAGCAATCAGCTATTTTCTCATTTGCTAAATTTTCAATGCAGATGCTCTATTTC
TTTCCATGTCCAACGTCTTTGATATCAACAGTTCCTAGAATATTTTGTGCAAAATCATCATGGCTGCCTACCCTGTTGGC
CACTCCATTCTACTGATACAGGACAACCCTGGGTTGAGAAGAGCCTGGAGTGGCTTCAGGCTCATGTTCACCTTTACTTA
AAAGAGTAGACTCTATGA
>BaacTAS2R41P_NW_006727908.1:5344246-5343364
CTGCACCCAGCATTCGCAGTCCTCTTCGTGCTGCTCTTTGTTCTGCTCTGTGTGCTGGGACTCCTGGCCAGTGGCTTCAT
TGTGCTGGTGCTGAGCAGAGAATGGGTGTGACCAGGGAGGCTGCTCCCCTCCAGCGTGATCCTCTTTAGCTTGGGTGTCT
CCTGCTTCTGCCTGCAGTGGGTTGGAATGGCGAACAACTTCTACTACTTTCTCCATCTGGTCGAGTACCGCGGGGGTCCC
GCCTGGCAGTTCTTTGGTCTACGCTGAGACTTCCTGAACTCGTTCACCTTCTGGTTTGGCTCCTGGCTCAGCGACCTCTT
CTTCATGAAGATTGCTAACTTCACCCACCCCACCTTCCTCTGGCTGAAGTACAGGTTCCCAGGGTTAGTGCCCTGGCTTC
TGCTGGGCTCTCTCCTCATCTCCCTCATCATCACCCTGTTGTTCTTTTGGGGGAACCACGATTTGTATAAAGGTTTCCTT
ATTAGAAAATTTTCTGGTAACCTGACCTACAATCAGCGGAGCAGGAGGCTGGAAATTCACCATTTTCTACCCCTGAAACT
GGTCACCCTTTCAATTCCTTGCTCTCTTTTTCTGGTCTCGATGGCTGTGTTGATGAACTCTCTGAGGAGACACATGTGGA
GGATGCGGCACAGTGCCCACAGCCTGCAGGAGCCCAGCGCCCAGGCTCACACCAGAGCTCCGAAGTCACTCATCTCCTTC
CTCGTTCTTTATGCTCTGTCCTTCATGTCCCTGATCATCGATGCTGCAGGGTTCTACTCCTCAGAAAGTGACTGGTACTG
GCCATGGCAGATTTTAATCTACTCGTGCACATCCATCCATCCCTTTATCCTCATCCTCAGCAACCTCAGGCTTCGAGCGG
TGTTCAGGCAGCTAATTTTGTTGGCCAGGGGCTTCTGGGTGGCCTAA
>BaacTAS2R67P_NW_006733678.1:4207540-4208469
CTGCCATCTGGAGTTGAAAATACTTTTCTGGTAGAGGTAATAGGAGAATTCATGATTGGAATGCTGGGGAATGGGTTCAT
TGTACTAGTTAACTGCATTGACTGGGTGAAGAGACCGAAGTTCTCATCAGCTGACTGCATCCTCACCGGCCTGGCAATCT
CCAGAATCAGTCAACGTTGGAAAATGCTATTTGACTCGTTTGTACTGGTGTTATGGCCACATCTATATGCCATTGATAAA
CTGGCAAAAATTTTTTGGACCCTGTCCAATCACCTAGCTACCTGGTTTGCTACCTGTCTAAGTGTTTTCTGCCTCTTTAA
AGTAGCCAGTTCCTCCCACCCCTGCTTCACCTGGCTGCGGTGGCAAATTCGTAGAGTGGTACTTGTGCTTCTGTTGGGGT
CTTTGTTCTTACTGTTTTTGAACTTTGAATTAGTAGATGCGTTTAATGGTGTCTGGACTAACGTCTACAAAATATATGAA
AGAAACTCAACAGGGCCCTCAGATGTAAGTAAAACTCTGTATCTTGACGTGTTGATTGTTTTCACCTTCATCTGCCTAAT

CCCCTTTCTTCTGTCCCTGACCTCATTGCTCCTTTTATTCCTCTCCTTGATGAGACACACCAGGAATTTGCAGCTCAACC
CCAGCTCAAAGGACTTCAGCATAGAGGCCCATAAAAGAGCCATGAAAATGGTGATGTCTTTCCTCCTCCTCTTCACGGTT
CACGTTTCTTCTGTCCTATTAACAGGTTGGGTTTTCCTTAAACTGCAGAAACATCAAGCCAATTTGGTTGTCATGTTAAC
TTTGACTCTTTTTCCTTCAAGCCACTCATTTATCCTAGTTTTGGCAAACAGCAAGCTGAGACAAAATGCCTTAGGACTAC
TGTGGTATCTTAACTGCCACCTGAAAAGAGTGAAACCTGTAGCTTCATAG
>BaacTAS2R60P_NW_006727908.1:5373867-5372929
ATGGTTCCAGGACCTCAGTTGGCTGATAAGAGAGCCTTTATCTTTGCTATCATTTTATTCCTTTTGTGCTTGGTGGCAGT
GGTGGGTAATGGCTTAATCACCGTGGCACTGGGCATGGAGTGGTTGCTGCAGAGAACTTTGTCGCCCTGCAATAAGTTAT
TGGTCAGCCTGGGAGCCTCTAGCTTCTGTCTGTGATGGGTGGTGATGAGTAAGAACGTTTATATTCTCCTGAATCCAATA
GCCTTCCCATACAACCCTGTATTCCAGTTCCTAGCCTTTCAGTGGGACTTCTTGAATGCTGTCACGTTATGGTTCTCCAC
CTGGCTCAGTGTCTTCTACTGTGTGAAAATCGCAACCTTCACCCCCCCTGTCTTCCTCTGGCTAAAGCAGATGGTGTCTG
CATTGGTTCCATGGATGCTGCTCAGCTCTGTGGGGCTCTCCAGCTTTAGCGCCATTCTATTTTTCATAGGCAACCAGAGA
GTATAGCAGAACTATTTAAAGAGGGCTCTGCAATCTTGGAATGCCACTGGGAATGCTATGAGAACATATGAGAGACTCTA
CTTCTTCCCTTTGAAAATTGTTACCTGGAGAGTCCCTACTGTTGTCTTCATCGCTGGCACGGCTTTGCTCATTACATCTC
TGGGAAGACACACCATGAAGGTCTCCCTGTCCATCTCAGGCTCTCATGATCCCAGCACCCAGGCACACATCAAGGCTCTC
CTGGCTCTCATCTCCTTTGCTGTCCTCTTCGTTTCCTATTTTCTGTCACTGGTGCTCAGTGCCTCAGGTGTGTTTCCATC
CCTGGAATTCAGGTACTGGGTGTGGCAGGCTGTGATTTATCTGTGCACAGTAGTCCGCCCCATTGTTCTTTTCTTGAGTG
ACCGCAGGCTGAGAGCTGTGCTAGAGAGGGGCTGCTCCTCAGGGCATGGGGCATCTTGA
>BaacTAS2R62AP_NW_006727908.1:5379748-5378812
ATGCCCTCCTCACACATGTTGAACTCCATGGTCATCTTTTTCCTGGAGTTGTTGGCTGCCATGCTGCAGAATGGCTTCAT
AGTTACTGTGTTGATCAGGGAGTGGGTACGATGCCAGACACTGCTTGCAGGCGACATGATTGCAGCGTCCTCCTTGGCCG
TCTCCTGGTTCTGTCTGCATGGGATGGCCCTCCAGGACAACCTCGTGGCCGCCTTTGATTTGGGTTCCAAAATTTACTAT
TTCAGCATCCCCTAGGACTTCATCAACTCTCTCACTTTCTGGCTTACTGCTTGGCTTGCTACATTCTACTGTGTGAAGAT
CTCATTCTTCTCTCACCCCATCTTCTTTGGGCTGAAGTGGAGGATTTCTCGGTCAGTGCCCAGGCTGCTGCTGGGCTCCC
TGATCTTATCTGGTCTGGTAGTCATCCCATTAGACACTGGGAATACAATTCTTGTGCAGATGGTTGCTGCCCAGAGTTCC
CATGGAAACAACACCCTGGCTGGTAGAATACAGACTGTCTCTTTGCACTTTTTTCTACCTCATGTAATTATTATGTGGTC
AATTCCATTTCTCCCGTTCCTGGTGTCCACCCTCTCACTCGTGTTCTCGCTGTGCCGGCACTTGGGGCAGATGAGGGACC
ATAGACCTGGCCCCGAGTGATCCCAGCACCCTGGCTCACACCATGGCCCTGAAGTCACTTGCCTTCTCCCTCGTCTTCTA
CACGTCGTATTACCTGTGCCTGATTATTGTTGTTATAAACATCCTAACCCTCCGGAATCACTGGCGCTGGGCCTGGGAAG
TGGTGACCTATGCAGGCACCTGTCTGCACTCCAGCATCTTGGTGCAAAGCAGCCCCGATCTGAGAAAGGCCCTGAAGAAG
AGGCTTTGGAGAGGGGCAAGAGGGACAAGGAGCAGTTTTTCTCACCAGTGTCAGTAA
>BaacTAS2R62BP_NW_006725237.1:1017289-1018186
ATGGTCATCTTCTTCCTGGAGTCGGTGGTTGCCAGGCTGCAGATCGGCTTCACAGTCATCGTGCTGCGCCGGAGCGGGAC
GCTGGATGATGCCGGACGCTGCCCGCAGGCGACATGACTGTGGCCTGCCCGGCCGCCTCCCGGTTCCGTCCGCAAGGGAT
GGCCCTCCAGAACAACCTCCTGACTTCCTTTGGTTTTGGTTCCAGATTTTATTTCAGAATCTCCTGGAGCTTCATCAACA
CTCTCACGTTCTGGCTGACCACCTGGCTTGCTGTCTTCTACTGTGTGAAGATAGCATCCTTCTCTCACCCCATCTTCTTC
TGGCTGAAGCGGAGGATTCCTCGGTCAGTGTCCCAGCTGCTGCTGGGCTCCCGGATCCTGTCTGGTCTGACCGTCGTCTC
ATCAGCCACCGACAAGTCAATTCTTGTGCAGATGGTTGCCACCCAGGGTTCCCATGGAAACGACACCCTGGCTGGTAGAA
CACAGACCGTCTCTTTGCGCTTTTTTCTACCTCATGCAATTATCATGTGGTCAGTTCCATTCCTCCTGTTCCTGGTGCCC
ACCCTCTCGCTCATGTTCTCGCTGCGCCGGCACTTGGGGCAGATGAGGGACCGCAGACCCGGCCCGAGTGATCCCAGCAC
CCGGGCTCACACCATGGCCCTGAAGTCACCTGCCTTCTTTCTCATCATATTTCCTCTGCCTGATTATCGTCGTTGTGAAC
ATCCCAACCCTCCGGAAGCACCGGCACTGGGCCTGGGAGGCGGTGACCTATGCCGGCATCTGTCTGCACTCCAGCATCTT
GGGGCACAGCAGCCCCAAGCCGAGAAAGGCCCTGAAGAAGAGGCTTCGGCGAGCCCTGAACAAGGAGCAGTTTGTCTTGA
GTTGTCAGTATCAAGAA

>CocrTAS2R3A_NW_004567106.1:68050509-68051456
ATGTCGGGCCTCCCGGAGTGTGTGTTCCTGCTTCTGTCTACCACTCAGTTCATTCTGGGGGTGCTGGGGAACAGCTTCAT
TGTGTGTGTCCACGGCCGCAGCTGGCTCCAGAGCAGGAGGATCTCTTTGTCTGACTTCATCGTCACTACTCTGGCTCTCT
CCAGGCTTATTTTGCTGTTTTCTCTCTTGGCTGACGGTGTTTTAATGGTGTTTCCTTCTCAAGTATGTGACAGGGGAGTA
GAAATGCATTTTCTTGAGATTATCTGGACTTTCACAAATGACCTGAGCATTTGGCTTGTCACCTGTCTCAATGCCCTCTA
CTGCCTGAGAATCGCCAGTTTCTCTCACCCCACATTCCTGTGGCTCAAGTGGAGAGTCTCCAGGTTGGTCGCATGGATGC
TGCTGGGAGCCCTGCTCCTGTCGTGCGCCAGCACCATGTCCCTGATCCATGAATTTAAGATCTGTTCTGTTTTGATTGGA
ATTGATGCCACAAGGAATATGACTGAGCACTTCAGAAAGAAACATGAATATGAACTGGTCCACGGCCTCGGCATTCTGTG
GAACGTCCCTCCCCTCGTCGTGTCTCTGACCTCCTGCCTCGTGCTCCTCCTCTCCCTGGGGAGGCACACGCGGGAGATGC
GGCAGCATGGCGGCAGCGCCGGGGACCCCAGCACCGAGGCCCACCAGAGGGCCATCAGAGCCATCCTCTCCTTCCTCCTC
CTCTTTCTGCTTTACTTCCTTGCCCTTTTACTGATATCATCCAGTCATTTCCTTCCAGGAACTGAGATGATTAGGATGAC
TGGAGTTTTAATTAAAATGTTCTATCCCACTGGCCACTCATTTATTCTCATTCGGGGACACAACAAGCTGAAGCAGATGT
TTGCGGAGATGCGGTCCTGTGAGCCTTGTCGTCTGAGATGTGGGACCAAAGCGGCCTTTTCTCCCTAG
>CocrTAS2R3B_NW_004567106.1:68033238-68034176
ATGTCGGGTCCCCCGGAGTGCGTGTTCCTGCTTCTGTCTACCACTCAGTTCATTCTGGGGGTGCTGGGGAACGGCTTCAT
TGTGTGTGTCCACGGCCGCAGCTGGCTCCAGAGCAGGAGGATCCCTTTGTCTGACTTCATCGTCACTACTCTGGCTCTCT
CCAGGATTATTTTGCTGTCTATTCTATTTTTTGATAGCGTGTTAGCGATATTCTCTTACAAAGTGCATAATGAAGGCATA
CTAATGCAAATTACCGATGTCTTCTGGACATTTACAAATCATCTGAGCATTTGGCTTGTCACCTGTCTCAATGCCCTCTA
CTGCCTGAGAATCGCCAGTTTCTCTCACCCCACATTCCTGTGGCTCAAGTGGAGAGTCTCCAGGTTGGTCGCATGGATGC
TGCTGGGAGCCCTGCTCCTGTCGTGTGCCAGCACCATGTCCCTGATCCATGAATTTAAGCTCTACTCTATTTTGATTGGA
ATTGATGCCACAAGGAATATGACCGAGCACTTCAGAGCAAAAAGTGAATACAATGTGATCCATGTTCTTGGAACTTTGTG
GAACGTCCCTCCCCTCGTCGTTTCTCTGACTTCCTGCTTCGTGCTCATCCTCTCCCTGGGGAGGCACACGCGGGAGATGC
AACAGCATGGCAGCAACGCCGGGGACCCCAGCACCGAGGCCCACGAGAGGGCCATCAAAGTCATCCTCTCCTTCCTCTTT
CTTTTCCTACTTTATTTGTTACTTTTTAATTGCATATTCTAGTCGCTTCCTACCAGGAAATAAAATGATTGGGTTGAT
TGGAGAAACAATGGCAATGTTCTATCCCACTGCCCACACATTTATTCTCATCCTGGGAAACAGTAAACTGAAGCAGGTGT
TTGTGAATGTGATGTGGCGTAAGCCTCCTAATGGAACCAAGACGTCCTTCTCTCCCTAA
>CocrTAS2R4A_NW_004567106.1:68038535-68039455
ATGCGCCACTCAGTGTTTTTCTCGGCTGTTATCGTCTCCACAAGTGTTAATTCTGTCGGACTCTTTGCCAGCCTGTTTAT
TGCAGTGGTCAGTTACAAGACTGGGATGCAAAACCATAGACTCTCCAGTTTCGACAGGCTCCTGGTTAGCCTGGGCATCA
ACCGCTTCCTGTTGCTGGGATTGTTTCTTCTGAATGTTATTTGCTTTTTCATTTTCCCAAATGTTGTAAGGCCGGTGCCT
GTCGCCACTTTTACCCTGTTGTGCTGGCTCTTCTTGGACTCTAACAGCCTGTGGTCTGTAACCTTGCTCAACGCCTTGTA
CTGTGTGAAGATTACTAACTACCAGCACTCAGCGTTTCTGCTGCTGAAGCGCAACCTCCCTGCCAAGGCCCCCCGGCTGC
TGCTGGTCTGTGTGCTGCTTTCTGCCTTCTCCACTCTCCTGTATCTTGTGCTCCTACAGACATCACCCCTTCCTATGTTT
GTGGTTGGGAGGAATGGCACCGAGTTTCACATTCATGCGGACCTCTTGTTTTTGGTGACCTCTTTGGTGTTGAGTTCATG
TCTGCAGTTCCTCATCAACGTGACGTCTGCGTCCCTGTTAATCAGCTCCTTGAGGAGACATGTGCAGACGCTGCAGAGAA
GCACTACTACTCTTTGGAATCCCCAGATTGAAGCTCATGTGGGCGCTATGAAGCTGATGATCTTCTTCCTGGTCCTGTAT
ATTCCCTATTCCATGGCTACCCTGTTCCTTTACCTGCCTTCTCTAGGCATCGGCCTGGAGGCCAGATCTGTTTGTATGAT
TCTCTCCACCCTTTACCACCCGGGACATTCTGTGCTCATTATTCTCACACATCCTAAACTGAAAACTAAAGCCAAGAAGT
GTCTTTGTTTCAACAAACAGTGGAATTCGAACGTTCGCTAG
>CocrTAS2R372_NW_004567137.1:14383634-14384560
ATGTCAGGTGCATTTGAAAATGTTCTTATCATCATTGAAATTGTAGAATTCCTGGTAGGTACCTGGGGCCATGGATTCAT
TGTCTTAGTCATCTGTGCCGACTGGATCAAAACCAAGAAAATCACCTTGCTTGACTTTATCTTCATGTGCTTGGCTGTCT
CCAGGATTAGCATGACATGCCTGCTTCTCCAAGATAGTATCATCATGGCTTTCTATCCAAAAATGTATGAAATCAACCTA
GTAGTCGGAATGACTCTTGGTTTCCTATGGAGTCTCAACAACTCCTTGAGCAACTGGTGCGTGACCTGCCTAAGTGTCTA

CTACTTCCTCAAGCTTTCCAATTTTACCCATCCCTTCTTTCTCTGGCTGAAGTGGAGAAGAGACAGAGTTCTTTTCACCA
TTCTTTTGGGTTTCTTTTTCTCTTTCTTTGCTAATCTTCTGAGCATAAAATGGAATACTTTTTGGATCCATGAATATTTA
AAAATGGAAAAAAATAAGACCTGGAAAGAATGGCTGTATAAAAACTCAAGCTTCAGCCATCAAATTTTCCTGAACTTGGA
TGCCCTCATCCCATTTTTTGTGTCGGTCACTTCATTTTTCCTGTTAATCTTTTCCTTGTGGAGACATATCAGACAGATGA
TGCATCACACCAAAGAATCTGGACACCTCAACACAGAGGTTCCTGTGAGGGCCAGAAATATGATGATTTCTTTCATCATT
CTCTTAGTGGTGCATTATTTGGCCAGTGTCTTGCTAATATGGTGCTACATCAAACTCAACAACTCAGCGAGTATTCTTAT
TGTTGAGATGGTAGCGTTACTCTATCCTTCAATTCACTCTTTCAATATAATTTTGGGGAACAGAAAAGTGAGACAGACTT
TCACGAATCTGCTTAAGCAAATGGAATCTTACATCAAGAGAATGTAA
>CocrTAS2R8_NW_004567137.1:14392601-14393527
ATGCCCAGCACAGAGGACACCATCTTCATAGTCGTAGTCACTGGAGAATCCGTCACAGGACTGCTGGGGAATGGATACAT
TGCACTCGTTAGCTGGAGACACTGTAGTAAGAAGAAAAAGATCTCCTCGATTAACTGCATCCTTCTAAGTTTAGCTGTCT
CCAGAATCTGCTTGGTCTTTTTTATAGCAACAGATAGTATCCTCAGGGTCCTCCTCCCAGACTTTTATGAAAATGATCAA
CTACAGGTAGTCTTCAGTACCTTCTGGACACTCATCAACTACTTAAGTATATGGGTTGCCACCTGCCTCAATTTCTTCTA
TTTACTCAAGATAGCCAATTTCTCCCACCCGCTGTTTCTCTGGCTGAAGTGGAGGATCGACAGGGCCATTCTCTGGATCT
TACTGGGGTGTTTGGCCATTTCTTCTCTGATCAGCCTTATTTTAGCAGTGATTCCAAAGGATGATACATTTCACATGATT
GTAACACAGAGAAGAAACATCACTGAACTATCCCATGTGAGTAAAAGGCAGTACTTCAACTCCATGACTCTCTTTAACCT
GTTCATAATTGTCCCATTTTCTGTGTCATTGATGTCATTTTGCCTTTTAATCATGTCCCTGTGGAGACACACCAAGCAAA
TGAAACTCAGTGGCACTGATTACAGAGACCCCAGCACAGCCGCCCACATAGGAGCCATGAAAAACATCGCTTTCTCTCTT
GTCCTTCTTTTTGTTTACTGTGGGGCTTGTCTTTTGGTGACTTTTAGCTACCTTCTGAAAGAGAAAATGTTAGGTGTGAT
GTTTGGAGAAGCAACATCAATTCTCTACCCCTTAGGCCACTCCCTGATTTTAATTATTGGAAATAACAAGCTGAAACAGT
CCTTGGCCAGGAGGCTGAAATGTATAAAAATAGCATGCATGATGTAA
>CocrTAS2R10_NW_004567137.1:14389242-14390150
ATGTTAAATACAGTGGAAGTTTTCCTAATTTTTGTAGTGATTAGTGAGTCAATATTGGGGGTTCTAGGGAATGGGTTCAT
TGGAATTGTCTATTGCATTGACTGTGTGAAGAAAAGGAAGTTTTCTATGATTGGTTTTATTCTCATAGGCTTAGCAACTT
CCAGAATTTGCCTGATAGTGATGATCACTACGGATGGACTTATAAAGCTATTCTTTCCAGATATGTATGCCTCTGGTAAA
TTAATTGACTATGTTAGTTACTTGTGGGTAATTATCAGTCAATTAAATATCTGGTTTACTACCAGCCTCAACATGTTCTA
CTTCCTGAAGGTAGCAAATTTTTCCCACCACATTTTTGTCTGGTTGAAGAGTAGAATTAATAGGGTTCTTCTCCTTGTGA
TGGGATCCTTGCCTATTTCTTTGTTAATTACTTTTCCCCAAGCTGAGAAGATTTTGAAAGATTATAGAATGATGAATAGC
AGCATGGCCTGGCAGATCAACTTTCCTAAAGATGAATACATCACTAAGCAGATATTGTTCAATCTGGGAGTCATAGTCCT
CTTTACACTAACCCTGATTACATGTTTCTTGCTAATCATTTTCCTCTGGAAGCACAGCAGACAGATGCAACTTAATGTGA
CAGGACTCAGAGACCCCAGCACCGAAGCACATATGAAAGCCATGAAAATTTTGATCTCCTTTGTCATCCTCTTCATCTTA
TTTTTTATAGGTGTTGTCATAGAATTATCATATTATATGAGGCCAGAAAACAAATTGTTGTTTATTTTTGGTATGATAAT
CACAGTACTCTATCCTTGGGGCCACTCATTTATCTTAATCCTAGGAAACAAAAAGATAAAGCAAGCCTACTTGGTGATAC
TGCAGCAATTAAAGTGTCGCAAGAAATAG
>CocrTAS2R408A_NW_004567137.1:14368994-14369953
ATGCTGAGTTCACTCCTGAGCATTCTTTTCACCCTACTAGTAATAGGATTTATTGTAGGGAATTTTGCCAATGGCTTCAT
AGCGCTGGTGAACTGCGTTGACTGGGTCAAAAGACAGCAATTCTCCTTGTTGGATCAAATGCTCACTGCTCTGGCGGTCT
CCAGAATTGGGTTGCTCTGGATAACATTAACGAGTTGGTATGGGATTGCACTGGATCCAGCTTTATATAGTTTTGAAGTC
AGAATAATTGTTCATGTTCTCTGGACGGTAACCCATCATTTTAGCCTCTGGTTTGCTACTAGCCTCAGCATATTTTATTT
GCTCAAGCTGGCCAACTTCTCTAGCCTTAAATTTCTTTACCTAAAATGGAGAGTGAAAAGGGTAATTCACATCATTTTGT
TGGGGAGTTTTATGGTCTTGGTTTCTCATCTTGCACTGGTAAGCATCAGTGAAAATACGTTGGCAGAGGATTATGAAGGA
AACACCACTTGGAACTCCCATTTGAGGCATGATGTATACCTTTCCAACCTCATTGTGTTCACCTTAGTAAATTTCATCCC
TTTTACTCTGGCCCTGATAGCTCTGCTGTTGTTATCTTTTTCTCTGTGGAAGCATCATAGGAGGATGCAGCTCGGGGCCA
GAGGCACCCAGGACCCCAGCACCAGGGTCCACGTCAGAGCCTTGCAGACCGTGGTCTCGTTCCTCTTGCCGTTTGCCTGT

TACATCCTGTCTCTAGTCATCTCAGTTTGGAGTTCACCAAACTTACGGAATAAACACACCGGCTTGTTTTGTCAGTTTCT
TGGAGTCCTGTATCCTTCAAGCCACTCATGTATCCTCATCTGGGGAAACAAGAAGCTAAAGCAGGCTTTCGTGTCATTTC
CGAGGCAGCTGAGGTGCCGGCAGAAAGAAAGGAAGAGGGTGGGAGCTCTGGCATCTTCGTGCAGAAAACAAATTGGTTGA
>CocrTAS2R38_NW_004567106.1:68106742-68105750
ATGCTGACGCTGACCCCTGCCACCACCGTGTCCTATGACGTGAAGCGTGTATTTCTGGCCCTTTCGGTCCTGGAGTTTGC
GGTGGGCCTCCTGGCCAACGCTTTCATTTTCTCAGTGAACCTCCGGGATGTGGTGAGGAGGCAGCCGGTGAGCACCTGCG
ACCTTGTCCTGCTGGGTCTCAGCCTCACCCGGCTCTTCCTGCAAGGGCTGCTGTTCCTGGATGCCATCCAGCTGACCTAC
ATCCAGCAGATGAAAGACCCGCTGAACCGCAGCTACCAAGCCATCTTCCTGCTCTGGATGATCGCAAGGCAAGCCAGCCT
CTGGCTCGCAACCTGCCTCAGTCTCCTCTACTGCTCCAAGATCGTCCGCTCCTCATACACCTTCCTGGCCTGCCTGGTGA
GCTGGGTGCCCCGGAAGATCCCCCAGGTGCTCCTGGCTGCTCTGCTCATCTCCTGTGTCTGCGTCGCCTTCTGTCTGCAG
GGCTACTTCAGTCAGTCTGGCTTCCTTGTCGCAGCTCCCGGAGTCACGAACCACAGCAAGATCAGCATGCGAGTCGTGCA
ACTGCATTTCTTCCATTCCTTCCTCATCTGCAGCCTGGGGGACGTCCCCCCTTTCCTCATTTTTGTTGTTTCTTGTGGGG
TGTTGATTGTCTCCCTAGGGAGGCACCTGAGGACAATGAGGACCCTAACCAGCCACGTTGGGGACCCCAGCCTGGAGGCT
CACATCAGGGCCCTCCAGTCTCTGGTCTCCTTCCTCTGCCTGTACGTGGTGTCGTTCTGTGCGGCCCTGGCATCGGTGCC
TTTACTGATGCTGTGGCACCACAAGCTGGGGGTGATGGTCTGCGTGGGGGTCATGGCCGCCTGTCCCTCGGGACACGCAG
TCATCTTGATCTCAGGCAACCCCAAGCTGAGGAGAGCTGTGGGGACCATGCTATGCTGGGCCCAGAGTAGCAGACGGGGC
AGAGCGGGCCCCAGGACACCAAATCCCTGCTGA
>CocrTAS2R18A_NW_004567137.1:14352175-14353116
ATGTGGACTGCAGCGAAGGCCTCCTTCCTGGTGCTGATCACAGGGGAAATCACCTTAGGGATGCTGGGAAATGGGTTCAT
TGCGCTGGTCCACTGCAGGGAGTGGGTCAAGAAGGGCAAGATCTCAGCAGCTGACTTCATCCTGACCAGCTTGGCTGCGG
CCAGAATCCTTCAACTGGGGGTAACACTACTGGACTCATTTATCGTGGGGCTCTTTCCCCTCATGTATGGCTCTGGTACG
GCAGCAAGAGTGGTGACTTTGCTCTGGTCACTAAGTAATCACTTAGCTACCTGGGCAGCCACCTGCCTCAGCGTGTTGTA
CCTCCTGAAGATCGCCACCTTCTCCCACTTCTTTTTCCTCTGGCTGAAGTGGAGAATGAACAGAGTGGTCCTTGTCATTT
TCCTGGGGTCCTTGTCTTTCCTGTGCGCAGACGCCCTCCTGCAGGATGCTCTCACTGAGCTCTGGATGCTCTCCTATAGA
GGACAAGAAAGAAATGCCACTGTGTGTGAGGATGCAAATCATCCTGCCTACCTGAGAAGCCTGATCCTCCTTAGCATGAC
CTATGTTATCCCCTTTCTCCTGTCCCTGATCTCCTTGGTCCTTTTATTTCTGTCCTTGGTGAGACACACCAGGAATTTGC
GGCGCGGCTGTGTGGGTTCCCGGGACCCCAGCACTGAGGCCCACAAAAGGGCCATGACAATGGTGATGACCTTCCTCCTC
TTCAGTGTCTTCTACTTTGTCTCCACTCTGATAGCAAATTGGGTCTTCCTTAAGCTACAGAGATATCAACCCATGATGTT
TTTCGTGGTGCTTTCTTCTGTCTTTCCTTCAGGTCACTCATTTATCATAATTTTGGGAAGCAACAAGCTAAGGCAGATTG
CCTCGAGACTACTGTGGCATCCGCCATTCTCTGGAAGAAGAGCACAGCCTTTAGCCTCATAG
>CocrTAS2R18B_NW_004567137.1:14342227-14343168
ATGTGGACTGCAGCGAAGGCCTCCTTCCTGGTGCTGATCACAGGGGAAATCACCTTAGGGATGCTGGGAAATGGGTTCAT
TGCGCTGGTCCACTGCAGGGAGTGGGTCAAGAAGGGCAAGATCTCAGCAGCTGACTTCATCCTGACCAGCTTGGCTGCGG
CCAGAATCCTTCAACTGGGGGTAACACTACTGGACTCATTTATCGTGGGGCTCTTTCCCCTCATGTATGGCTCTGGTACG
GCAGCAAGAGTGGTGACTTTGCTCTGGTCACTAAGTAATCACTTAGCTACCTGGGCAGCCACCTGCCTCAGCGTGTTGTA
CCTCCTGAAGATCGCCACCTTCTCCCACTTCTTTTTCCTCTGGCTGAAGTGGAGAATGAACAGAGTGGTCCTTGTCATTT
TCCTGGGGTCCTTGTCTTTCCTGTGCGCAGACGCCCTCCTGCAGGATGCTCTCACTGAGCTCTGGATGCTCTCCTATAGA
GGACAAGAAAGAAATGCCACTGTGTGTGAGGATGCAAATCATCCTGCCTACCTGAGAAGCCTGATCCTCCTTAGCATGAC
CTATGTTATCCCCTTTCTCCTGTCCCTGATCTCCTTGGTCCTTTTATTTCTGTCCTTGGTGAGACACACCAGGAATTTGC
GGCGCGGCTGTGTGGGTTCCCGGGACCCCAGCACTGAGGCCCACAAAAGGGCCATGACAATGGTGATGACCTTCCTCCTC
TTCAGCGTCATCTACTTTTTCTCCATTCTATTAGCAAGCTGGGATGTGTTTAAGATACATAGGTTTTGGGCCAAGATGTG
TTTTGTGATGTTTTCAAGTACTTTTCTCTCTGGTCACTCATTTATCATAATTTTTGAAAACAGCAAACTAAGGCAGGTTG
CCTCAAGATTATTGTGGCATCTTAAGTTTTTTGGAGGAATAGCAAAGCCCTTAGCTTCATAG
>CocrTAS2R42C_NW_004567137.1:14320216-14321172

ATGTTTGAAGGTCTGAATGAAGTCTTTCTGATACTGTCAATAGTGGAGGGGCTCATTGGAATACTGGGGAATGTGTTCAT
TGGAGTCGTCAACTGCTATGAATGGGTCAAGGACCACAAGATCTCCCTGGCTGACTTCATCCTCACCTGCTTGGCTCTCT
CCAGAATCATTCAACTGCTGGTATATTTGCTTGAGTCTTTTCTAATGGGACCAGCTGGGTATAGGTACCTCACATATGAA
CTAACAAGGCTTTTTAGCTTGCTCTGGAGAGTAACTAATCACTTGACTACCTGGCTGGCTACCTGTCTAAGCATTTTCTA
CTTCCTGAAGATCGCTAAGTTCTCCCACTCTTTTTTCCTTTGGCTGAAGTGGAGAATGAATAAAGTGGTTCTTATGATTT
TTATCTCTTCTTCCGCTATTCTGATTTTTGACTTTCTTTTGCTAGAAAGTTTTTTCTATAATGAATTTTTGGGAAGGAAT
CTGACTTCGTATTCAAATGACAGAAAAATTGTCAATGTTAACATTCTGATTGTTCTTAGCATGACCTATGTTATCCCCTT
CCTCCTGTCCCTGACCTCCTTGGTCCTTTTATTTCTGTCCCTGCTGAGTCACACCAGGAATCTACAGCGCAACTGCATGA
CTTCCCGGGACTCCAGCACCCAGGCCCATAAAAGGGCCATGACAGTGATACTGTCTTTCCTCCTCCTCTTTATGGTTCAT
TTTATTTCCATGCAATCGGCAAATTGGTTGTTTTCTATGTTCTGGAGCAACATGTTTACAAGGTTTCTCATGTTACTAAT
ATATGTCTTTCCCACTGGCCACTCGTTTATTTTGATTCTGGGAAACAAGAAGCTCAGACAGACAGTTCAGAAAGTACTGT
GTTACCTTAAAACCTTATTGAAGAGAAAATCTGTCAGCTTTACAGATAGACTTTCCAGAGTCTTTTCAAAGACTTAA
>CocrTAS2R408B_NW_004567137.1:14365414-14366325
ATGATAATGTTGAGGATTCTTTTCATCCTAGTAATGACCGGATTTGTTCTGGGAAATTTGGCCAATGGCTTCATAGTGCT
GGTGAACTGCATTGACTGGGTCAAAAGACAGAAGTTCTCCTGGACGGACGGAATCCTAACTGGTTTGGCGGTCTCCAGAA
TTGGTTTGCTTTGGGTAATATTATTTTATTCTTATGCATCTGTGTATAATTCAGTTCTACTAATCTTAGGAATAAGAACT
ATTGTTAATATTATGTGGATAGTAAGCAACCATTTTAATAACTGGCTTGTTACAATGCTCAGCATATTATATTTGCTCAA
GATAGCCAACTTCTCTAGTCATATTTTTCTTTATTTAAAGTGGAGAGTTAAAAGTGTACTTCTCACCATAATGTTAGGGA
GTTTGCTCTTTCTGGTGACTCACTTTGCAATGGAAAGCGTGTGTGAGAATATGCTGACAAACACGTCTGAGGGAAACATG
ACTTGGAAGATCAAATTCAGAGCCGTCATCTGCCTGTCAAGTACGACTGTTTTCACTTTAGTCATTGCCATACCCTTTAC
CATGTCCCTGATATCTTTTCTGTTACTAATTTTCTCCCTGTGGAGACATCTCAAGAGGATGCAGCTTAGAGGTGAAGGGT
CCCAAGACCTCAGCACCAAGATCCACCTCAGAGCTTTGCAAACCATCATCTCATTTCTCTTGATGTTTGCTCTTTTCTTC
ATGGCTCTCATCATCTCTGTTTGGAGCCCTAGTCACGTCCAGAGCAAACCCATCCATCTGTTATGCCTGGCTGCAGGAAC
TCTGAATCTTTTGATCCACTCACTTATCTTGATTTGGGGAAACCAGAAGCCAAAGCAGGCCTGTGTGTCATTCCTGAGGC
AGCTGAGGTGCTGGCTGAAGAAAAGGAAATAG
>CocrTAS2R46_NW_004567137.1:14358077-14359036
ATGACCTTCTTACTGAACATTCATTTCATCCTAATAATGATAGTATTTATTGCAGGAAATTTGGCCAATGGCTTCATAGC
TCTGGTGAACTGCATTGACTGGGTCAAGAGGCAAAGGTTCTCCTCAGCGGATGGAATTCTCACTGGTCTGGCGGTCTCCA
GAATTGGTTTGATCTGGATAACATTAATAAATAGGTATGCAACTGTGTTTGTTTCAGCTTCCTATACTTTAGAAGTGAAC
GTTATTGTTAATCTTGCCTGGATAACTTGCAACCATCTTAGCACCTGGCTTGCTACATGCCTCAACATATTTTATTTGTT
CAAGATAGCCAATTTCTCTAACCTTCTATTTTTTTATTTGAAGAGGAGAATTAAAAGAGTGGTTCTAGCAATACTGTTGG
GGAGTTCGGTCTTTTGGATTATGCATGTTGCTGTGACGAGTGTAGAAGAAGATGTATGGACAAGTGAGTATGAAGGGAAC
ACCACTGGGATGAGCACTGGGAAGGAAATGATACGCTTTTCCCACATGACTGTCGTTACAGTGGTGAACTTCATACCCTT
CCTTATGTCCTTGGCATCTTTTCTGCTGTTAATCTTCTCTCTGTGGAAACACCTCAAGAAGATGCAACTCAGAGGCAAAG
GGTCCCCAGACCCCAGCACCAAGGTCCACATCAGAGCCATGCAGACTATGGTCTCCTTTCTCCTGCTCTTCACCATTTTC
GTCCTGGCTCTCATCATCTCCCTTTGGAGCTCAAACAAGCTCCAAAACAAACTATCCTTAGTGTTTTGTGAGAGTCTTTT
AGTCCTGTATCCTTCAAGCCACTCATGTATCCTGATTTGGGGAAACCAGAAGCTAAAGCAGGTCTGTATGTCACTTCTGA
GGCAGCTGAACTGCTGGCTGGAGGAAAGGAAGCAGGTGGGAGCTCCCGAGTCTTTGTGCAGAAAGCAAACTGGAGTCTAA
>CocrTAS2R67_NW_004567137.1:14324170-14325102
ATGCCATCTGAAATGGAAACTACTTTTCTGGTGGTGGCAATAGGAGAATTTGTGATTGGAATGCTGGGGAAAGGCTTTAT
TGTGCCGGTGAACAGCAGGGACTGGGTGAAATGTAAAAAGCTCTCTTTAGCGGACTGTATCCTCACCGGGCTCGCTATCT
CCCGAATCGGCCATCTCTCCATAATAGTACTTGATGCATTTGTAATGGTGTTATGGCCACATGTCTATGGCGATGATAAA
CTAGCCACGTGTATGGCTATTGCTTGGACTCTGAACAATCACCTCGCTACCTGGTCGGCTACCTGTCTAAGTGTTTTCTA
CTTCTTTAAAATAGCCAATTTCTCCCAGCTCTGCTTTGTCTGGCTGAAGCAGAGAATTAGCACAGTGCTGTTGGTACTCC

CGCTGGGGTCTTTGTCCTTCCTGATGGCCCACCTGGCATTAACAGATAAGTTTAGTGGCTGCTGGAATGTTTGCGACACT
TACTTAAGAAACTCAACTTGGCTGTCAGGTGTAAGATTGCTATGTACTAAGAGCTTGGTGGCTGACAGTTTGATCCAGTT
AATCCCCTTTCTTCTTTCTGTAATCTCACTGCTCCTTTTATTTCTCTCCTTGATGAGACACACCAGGAAGCTGGAACTCA
TCTCCAGACCCAGGGACCCCAGCACTGTGGCCCATAAACGGGCCATGAAAATGATAACATTGTTCCTCCTTCTCTTCATG
GTTCACTTTTCTTCCTCTGTGTTAACAGGATGGACTTTCCATGTGTCGCAGAAAATTCAAGATGGTTTTGCTGTCACGCT
AGCAGTCACTCTTTTTCCTTCAGGCCACTCCTTTATCCTAATTTTGGGAAACAGAAAGCTGCACCAAGCTGCCTTGCAAC
TACTGAGGCATCTTAAATGCCACCTGAAAAGGGTGAAAACTCTTGCTTCATAG
>CocrTAS2R2P_NW_004567106.1:16615976-16617170
ATGATTTCTTCCTTATCAGGTATTCCTCATGTTATTCTTATGCAGTAGAATTTATCACCGGCATTACAGTAAATGGATTT
CTTATAATCATCAACTGTAAAGAATTGATCAAAAGCAGAAGGCTAATGCCAATACAACTCCTTTTCATATGTATAGGATT
GTCTAGATTTGCTCTGCAGATGGTGTTAATGATACAGAGTTTTTGCTCTGTGTTCTTTCCACTCTTTTTTTTATTTTTTT
TATTTAAAGATTTATTTATTTATTCGTACAGAACTGGGGTACAGGCCAGAGATGCGGAGGGTGAGAGGATTCACCAGAGA
CAGAGCAGGGAGCAGAACAGGCAGATCGAGGAAGTACACTGCTGAGGGGAGCAGTGGGCGGGTCAGGAGAGGTCTGCCTC
GCCATGTGGTCGCCTCCGGGGCAGACAGGGATCGAACTCATGCGGAAGTGGGTTTAGTTTCTTAGTGCAGTGCTTCAACC
CGCTGCGCCACCAGGGAGGGCCCCTCTTTCCACTCTTTTATCGAGTGAAAATTTATGGTTCAACAATGATGTTCCTTTGG
ATATTTTTTAGCTCTGTCAGTCTCTGGTTTGCCACCTGCCTATCCGTGTTTTACTGTTTCAAGATAGCACACTTCTCTCA
GAACTACTTTCTTTGGCTGAAATACAGGATATCAAAGCTGATGCCTTGGCTGCTTCTGAAAAGCCTGCTGACCTCCATGG
GCACTGCCGCTCTGTGTATCATGGTAGATTACCCTAAAAACGTGGATGATGATGTTCTCAGGAATGCTACTCGAAGAGGG
ACTAAATTCAAGCAAATTAATGAAGTACTTTTTGTCAACGTGACATTAATATTTCCTTTAGCCATATTTGTGATATGCAC
TTTTATGTTACTCATTTCTCTCTACAAGCACACTCACCGGATGCAGAACGGATCTCATGGTTTCAGAAATGCTAGCACAA
AAGCCCATATAAATGCATTAAGGACAGTGATAACATTCTTTTGCTCTTTATTTCTTATTTTGCTGTTTTCATGGTGAATA
TAACATTCAGCATCCCTTACAGAAGTCAGCGTTTCTTTGTGATGAAAGATATAATGGCTGCTTACCCCTCTTTTCATTCA
GTAGTAATAATCTTGAGTAATCCTAAATTACAACAATCATTCAGGAGACTTTGCTGCCTAAAAAGGAACTGATGA
>CocrTAS2R4BP_NW_004567106.1:68055091-68056014
ATGCGCCACTCAGTGTTTTTCTCGGCTGTTATCGTCTCCACAAGTGTTAATTCTGTCGGACTCTTTGCCAGCCTGTTTAT
TGCGGTGGTCAGTTACAAGACTGGGATGCAAAACCATAGACTCTCCAGTTTCGACAGGCTCCTGGTTAGCCTGGGCATCA
ACCGCTTCCTGTTGCTGGGATTGTTTCTTCTGTATATGATATACACATTTGTCTTTGCAAATGTTGAAAAGTCAGGGCAT
GTATTTACCGTCTTAACACCGTGTTTCATATTTTTGGACTCTAACAGCCTGTGGTCTGTAACCTTGCTCAACACCTTGTA
CTGTGTGAAGATTACTAATTACCAGCACTCAGCGTTTCTGCTGCTGAAGCGCAACCTCCCTGCCAAGGTTCCCCGGCTGC
TGCTGGTCTGTGTGCTGCTTTCTGCCTTCTCCACTCTCCTGTATCTTGTGCTCCTACAGACATCACCCCTTCCTACGTAA
GTGACTGGGAGGAATGGCACCGAGTTTCACATCAGTGTGGACACTGTATCTTTGGTGACCTCTTTGGTGTGGAGTTCGTT
TCTGCAGTTCCTCATCAACGTGACATCTGCGTCCCTGTTAATCAGCTCCTTGAGGAGACATGTGCAGACGCTGCAGAGAA
GCACTACTATTCTTTGGAATCCCCAGATTGAAGCTCATGTGGGCGCTATGAAGCTGATGATCTTCTTCCTGGTCCTGTAC
ATTCCCTACTTCCTGAGTACCCTGTTGTTTTTTCTGCCTTCTCCTGTTAGCATGGGTTTGGAGGTGGGAATTATTTGTGT
GATCATTCCTGCTCTTTACCACCCAGGACATTCTGTGCTCATTATTCTCACACATCCTAAACTGAAAACTAAAGCCAAGA
AGTGTCTTTGTTTCAACAGCTAGTGAAATCGCACTATTCAGTAA
>CocrTAS2R5P_NW_004567106.1:68059069-68059925
ATGCTGACTGCCACTGCCCTGGCACTGCTGATGCTGGTGGCAGTGGCTGCATTTCTCATTGGCCTGCCCGCAAATGGAAC
CCTTGCTGTCTGGAGTTTTGGAGAGTTGGTCAGGAAATTCAAGAGCTCTATATTCAATCTCCTTATCCTGGGTCTGGCTG
CCTGCAGGTTTCTCCTTCCATGGCTGACCAGGATGGACTTAAGCCTGTTTCCATTTTCCGAGAGCAGACGTTGGTCCCTT
CGTCTCAGGGTCTTCTGTGTCCTGATAAGCCAGCGCAGCCTGTGCAGAAAGATCACCACTGTTGAGCCCCCTGTCTGCTT
GTGGCTGAAAAAGAAGACGAAGACTTATTGCCTGAGTCTCTGGTGCCTTCTGGGGTGCCACGTGATTGATTTGTTGGTTG
CAGCCACAGTGACATAAAGTTCTTCACAAGGAAACAACAGCGTTGTGTACCCCCGTTCAGCATGGCGCTGTGTGTATTTA
TATATGTCACAGCTCATTCAAGGAAGTGGGGTGCTGTTCATGGTGCTGCTTCTTTCCTCTGGGATGCTGATCGTGTCTTT

AATAGATATCACAGGAAGATGCAGGTGTGCACACCTGGCAGGCAGGGTGCTCGGGCCAGGGCTCCAATCACCGCCGTGAG
GTCTCTGGTTGCTTCTCTGTCCGCACGTGCTGGCTGTCCCCCTTTGCTGTCAGCTCCAGGTCTTCCGCTCCTCTTACCTC
AGTCTTCATCTCTGAGACACTCATGGCTGCTCATCCCTCTCTTCATTCTGATCATGGGTGATTAATCCCAAGATGAAGCA
GAATTGCCAGAGAATCCTGTGGAAGGCAGTGTGTGCTTGGAGGGCCTGGGCCAGTGA

>CocrTAS2R14P_NW_004567137.1:14374068-14375218
ATGGTCAGTGTCTTAGACAGCACGCTTGTGGTCATTGTAAGTGCTGACTTGTCATTGGTAAGCTAGGAAACAGCTTCATA
GCCCTGGTGAACTGCATCGACTGGGTCCAGACAAGAAAGATCTTTTTCATGGACAGATTTCTCATGGCTCTGGCAGTCTC
CAGAATTGGTTTGCTCTGGGTAATGTTAATGAATTCGTATGTATCTATGTTTAATTTAGATTTTTTTATGACTCAAAAAA
TGTTAACGACAATTGTAATCTTCTGGACAGTGAGCAATCATTTCAGCCTCTGGTTTGCTACAAGCCTCAGCATCTTTTAT
TTTCTTAAGATAGCCAATTTTTCTGACTCTATTTTTCTCTATCTCAAGTGGAGAGTTAAAAAGGTGGTCTCCAGGCGAGA
AAATAACAAGAATAATGAACAGCCATATACCAGAGAGANNNNNNNNNNCAGGGACTACAATTGAGATGGAAAAAAAAAAG
GTGGTGATGGTCTCACTGACCATACCGATGACTTGGTCCTCTTGATGCTTAATCTTGCATTGGGAGCACACATGTGAATG
CCTGGCATGGTGAATATAAAAGAAACATGACTTGCAGTTCCGTGATGAGGGACATCGCCCAAATTTTCAAGCCTCTTGTC
TTCACAAGCACACTGTTTACGCTCGTGCCCTTTGCCGTGACCCTGATAGTTTTTCTCCTGCTAATCTTCTCCCTGGGGAA
TTATCTCAGGAGAGTACAGCTCAATGCCAAGGGCCCCAGAAGTGCCAGCGCCACATAAAAGCCTTGCCAAGTGGGGTTGC
CTTCCTGCTACTCTGCTGTTTTCTTTCTGTCTCTTTCTAGGTCGTTTTGGTGCTTGGAGCTTCAAGTCAAAAATCGGATC
ACTATGTTTTACCAAGATGTTGGAGCTGTTTATCCTTCGGGTCACCCGTTTGTGCTGATTCTGGGACACCATAAGCTGAG
ACAGGCCTTTCAATCAGTGCTGGGCTGGCTGAGGTCTCGGTTCAAAGATGAGAACCCTGTAAGCTCCTACACTAGAGAGA
GGCTCATCCTGC

>CocrTAS2R39P_NW_004567143.1:289765-290722
ATGACTGAAACCTGCAATCCCCAGAGAATGGATTATCACCATTTCACACCATCTTGGTTTTAACAGTTATAGTAACTGAA
TGCATCATTGGTATCATTGCAAATGGGTTCATCATGGCTGCCCATGCAGCTGAATGGATTCAGAGTAAGGCAGTTTCCAC
AAGCAGCAGGAGCCTATTCTTCCTAAGTATATCCAGAATGGTTCTCCAAGGCTTCATGATGCTAGAAATTACTTTAATTC
AACATCTCCACATTTTTATAATGGTGATGTTGCATACAGTATATTCAAAGTAAGTCTCATGTTCTTAAATTATTGTAGCC
TCTAGTTTGTGCCTGGCTGAGTTTCTGCTACTTTGTGAAGATCACAAAGATTTCCCCATCCCTTTTTCCTGAAGCTGAAG
GGGAGGATTTCTGGGTTGATGCCCTGGTTTCTGTGGCTATCAGTATTTATTTCCTTGGCTTACAGTGTGCTGTTCTCCAG
TGACATTTACATTGTGCATTGTGACACTTCTTTTTCTAACCCCTTTTCCAGCTCCACTAAGAGAAAACACTTCACCGAGA
CCAATGTGGTCAGCCTGCTTCTTCTCTATAATCTGGGGATCTGTGTTCCTCTGACCATGTTCATCCTTGCAGCCACTCTG
ATGAGCATCTCTCTGAGGAGACATACCCTGCACATGGAGAGCAATGCCTGTGGCTCTAGGGATCCCAGTATGGATGCTCA
CTTGGGGACCATCAAAGCTACCAGCTACTCTCTAGTTCTCTGTATGTTCAATACAGTTGCTCTATTTCTTCATATGTCCA
ACATCTTTGGTGCCAACAGTTCCTGGAATATTTTGTGCAAAAGAATCATGGCTGCCTACCCTGCTGGCCACTCATTGCTA
CTGACTGTGGACAACCCTGGCCTGAGAAGAAGTTGGAGCCAGTTTCAGCACCAAGTACATTTTTATCTAAAAGAGTAC

>CocrTAS2R62P_NW_004567143.1:452971-453662
CTTTGACTCCAAAGAGAGCTATTTCAACATCCGCTGGGACTTTATCAACACACTCACCTTCTGGCTAGCGGTCTGGCTGG
CCACCTTCTATTGCGTGAAGGTCTCCTTCTCCCACCCCATCTTCTTCTGGCTCAAGTAGCGGATTTCTCCGTCAGTGTCC
AGGATGCTACCAGGCTTCCTGGTCATATCTAGTCTGACGACCAGCTCCTCAGCCATTGGGAATGGAAATCTCATGTCCTT
GATTGCGTCCCCCGGCTCTCAGGGGAACCCCACCCTGGCTGATAGGATAGTGACATTCCATCGCCGCTTCTTTCTTCTGA
ATTTAATGCTTTGATGTCAACTCCCTTCCTCCTGTTCGTGGTGTCCACCCTCCTACTCATATTCTCCCTGCAGCAGCCCC
TGGGCCAGATGAGAGGCCATCGGTCCAGCCAGCACGATCCCAGCACCTGGGCCCACACCACACTCCTGAAGTCACCCTCC
TTTCTCATCTTCCACATCTTGTACTTCCTGTCCCTGCTGTTTGTCATGATGACAATCATCACCTTCCAGAATCACTGGCA
CTGGGCCTGGGAGGTGGTGACCTATGTGGGCATCTGTCTGCACGCCAGCATCCAAATGCCCTTGAAGACCAGGCTGTGGA
AGGCACTGCTCAGAGGGCAGCCTAACTCCACCGGTCAGGGTCAATGACCAGC

>OvmuTAS2R1_HG925181.1:917666-916776
ATGCTGGAGTGTCACCTTGTCAGCCACCTTGTTTTGACAGTGATACAATCTCTCTTTGGGATTTTAGTAAATGGCATCAT

TTTGATTGTGAACGGTACTGACTTGATCAAGCAGAGAAAGTTGATTCCACTGGATCTCCTTGTTTCCTGCTTGGCGATTT
CCAGGATGGGAATTCAGCTGGCCTTCTTCTACATTAACCTGGCTCTTCTTTCCTTGGTCAAATTCCCTCAGGTTACTGAG
AAGCTTGTAGTTTTCACATTTGTAAATGATTTGGGACTTTGGTTTGCCACCTGGCTCAGTGTCTACTACTGCACCAAGAT
TGCTACCATTGCTCACCCTCTCTTATTCTGGTTGAAGATGAAGATCTCCAAGCTGGTTCCTTGGCTGATTCTTGGGTCCC
TGCTGTATGCATGTAGTACTTCTGCTGTGCATGTCAAATATAAGTGGGCATTTTATGGAGAAGGCTTCCTGGACCTTTTC
TTCCCAAATGTAACAACTCACATCAAAGTAACCCCTACTTTACAGTCTGCCTTTCTGCTTGCTGAGTTTGCATTGCCGTT
TTTCATCTTCCTGATTTCTTCTCTGCTCTTGATATTTTCCTTGGGGAGACACACCTGGCAGGTGAGAAACACATGGACAG
GCCCCAGAAACTCTCACACACGTGCATACATCAGGGCCTTTCACTCCATCCTGTCCTTCCTGGCCCTCTATCTCTGCCAC
TACCTGATCGTTGCTTTGATCTTTTTTCAAATTTTTAACCTTAGAAGCTTCCTATTTCTGTTCTGCACCTTCATGGTTGG
TTCATACCACTCCATCCACTCTATTACTTTAATTTTAGGAAACCCAAAAATGAAACAAAATGCAAAGGCATTGCTCCTTC
TCAGAAAGTGA
>OvmuTAS2R2_HG925067.1:1214179-1215087
ATGATCTCTTTGTCAGTTATTCCACATGTTATCATCATGTCTGCAGAATTTATTACAGGGGTTACAGTAAATGGATTTCT
TATAATCATCAACTGTAATGAATTGGTCAAAAGCAGAAAGCTAACACCCATGCAACTCCTGTTCGTATGTATCGGGATGT
CTAGATTTGGTCTACAGACTGTGTTAATGGTACAAGGTTTTTTCTCAGTGTTCTTTCCACGCTTTTATAGCACAAAAATC
TATGGCACACCAATGCTGCTCTTTTGGATGTTTTTCAGCTCTGTCAGTCTCTGGTTTGCCACCTGTCTCTCTTTATTTTA
CTGCCTCAAGGTAACAGGCTTCACCCAGTGCTGTTTTCTTTGGCTAAAAGTCAGGATCTCAAAGTTAATGCCTTGGATGC
TCCTGGGAAGCCTGCTGACCTCTATGAACATTGCAGCTCTGTGTGTCAAGGTGGATTACCCTAAAATTGTGGATATTGAT
GTCCTCGGGAATGCCACAGCTAAGAGGATTAAACTCAACACAAAGCAAATTAATGAAGTTCTTCTTGTCAACTTGGCATT
ACTATTTCCTCTGACCATATTTATAATATGCACTGTTATATTATTCATTTCTCTCTACAAGCACACTCATCGGATGCAAA
ATGGACCTCCTGGTTTCAGAAACACCAGGACTGAAGCCCATATTAATGCATTAAGAACAGTGATAACATTCTTTTGCTTC
TTTATTTCTTACTTTTGTGCCTTCATGGCAAATATGACATTCAATATTCCTTATGGAAGTCATTGCTTCTTTGTGGTGAA
GGATATTATGGCAGCATATCCCTCTGGTCATTCGGTTATAATGATCTGCAGTAATTCCAAGTTCCAGCAACCAATCAGGA
GACTTCTCTGCCTAAGAAGGAGTCAGTGA
>OvmuTAS2R3_HG925097.1:371945-370995
ATGTTGAGACTCAGCAATTTGGGGTTTCTGGTTCTGACCGCCATTCAGTTCATCCTGGGAATGCTGGGGAATGGTTTCAT
AGGGTGGGTCAATGGCAGCAGCTGGTTCAAGAGCAAGAGGATCTCTTTGCATGACTTCATTATCACTAACCTGGCTGTCT
CCAGGATTGTTTTGCTGTGGATTCTCTTGATCGATGGTGTTTTACTGGTGTTCTCTCCCAAACTACACGATGAAGGGATA
ATCATGCAAATTATTGATGTGTTCTGGACATTTACAAACCATCTGAGCATTTGGCTTACCACCTGTGTCAGTGTCTTCTA
CTGCCTGAAAGTGGCCAGTTTCTCCCATCCTATGTTCCTCTGGCTCAAATGGAGAGTTTCCAGGGTGGTTGTATGGATGC
TGTTGAGTACCCTGCTGTTATCATGTGGCAGTGCCATCTCTCTGATCCGAGAATTTAAGATCTATTCTGTTCTCGGTGGA
ATTGATAGAACCGGGAATATGACTGAGCTCTTTAGAAGGAAGGAAAAAGAATATAAACTGATCCATGTTCTTGGGACTCT
GTGGGACCTCCCTCCCCTAGTCGTATCTCTGATCTCCTACTTTCTGCTTATCCTCTCCCTGGGGAGGCACGTGCGGCAGA
TGCATCAAGACTGTGGCAGCTCCAGAGATCCCAGTACCGAGGCCCACAGGAGGGCCATCAGAGTCATCCTCTCCTTCCTC
TTCCTCTTCCTACTCTACTATCTTTCCTTTTCTGTTTTAACATCCAGTTATTTCCTACCAGCAACTAAGATGATTGCGAA
GATCGGAGAAGTAATTACAATGTTCTATCTTGCTGGCCACTCTTATGTTCTCATTCTGGGAAATAGCAAGCTGAAGCAGA
TGTTTGTGGCGATGCTCCGGTGTGAGCCTGGTTGTCTGAAGCCTGGATCCAAGGGATCTGTTTATCCATAG
>OvmuTAS2R4_HG925097.1:358577-357687
ATGCTTCGGATAGTCTTTTTTTCTTCTATCGTTGTCTCTGAAATTATAACTTTTGTAGGACTCATTGTGAATCTCTTCAT
TGTAGTGGTCAGTTACAAGACTTGCATCAAAAGCCACAGGATCTCTTCTTCTGACAGACTCCTGTTCAGTTTGGGCATCA
CCAGATTTTTTATACTGTTACTGAATGTTGTTGTCATCATCTCTCCAAATGTGGAAAGGTCAGTCTCCTTATCCTCTTTC
TTCCTGTCATGTTGGATGTTTTTGGACTCTAGTAGTCTTTGGTTTGTAACCTTGCTCAACGTCTTGTATTGTGTGAAGAT
TGCTAACTACCAACACTCCGTGTTTCTCCTGCTGAAACGAAATCTCTCCACCAGAATGCCCCGGCTGCTGCTGGTCTGTA
TGCTCCTTTCTGTCTTCACCACTCTCCTGTATGTTATGCTCAGACAGTTGGCACCCCGTCTTGAATTTGTGACTGTGAGA

AATGGCACAGTATTTGACATCAATGAGGGACTCCTGTCTTTGGTGACTCCTTTGGTCTTGAGTTCATTTCTCCAATTCAT
CATTAATGTGACTTCTGCTTCTTTGTTGATCAATTCCTTGAAGAGACATGTACGGAAGATGCAGAGAAGCGCCACTGTTC
TTTGGAATCCCCAGACTGAAGCTCATGTGGGTGCTATGAAGCTGATGATCTGTTTCCTCATACTCTACATTCCATATTCA
GTTGCTACCTTGCTCCATTATCTCCCTTCTTCTATAGGGATGGATTTGAGAACCAAGTCTATTTATGTTATTATGTCCAC
CATTTACCCTCCAGGACATTCTCTTCTTATTATTCTCACACATCCTAAACTGAAAACAAAAGCAAAGAATATTCTTTGTT
TCAGTAAATAG

>OvmuTAS2R7_HG926251.1:310688-309750

ATGTCAAGTGAAGTGCAGGGTATCTTAATGCTCATAGCAGCTGGGGAATTTTCACTGGGGATCTTAGGGAACGCATTCAT
TGGATTGGTAAACTGTGTGGACTGGATCAAGCACAAGAAGATTGCCTCCATTGATTTAATCCTCACAAGCCTGGCCATCT
CCAGAATTTCTCTCTTATGTATAATACTATTGGATTGTAATATATTGGTCCTGTACCCAGATGTCTATACTGGTGGTAAA
CAAATGAGAATCATTGACTACTTCTGGACATTAACCAACCATTTAAGTGTCTGGTTTGCCACCTGCCTCAGCATTTTCTA
TTTCCTCAAGATAGCAAATTTCTTCCATCCATTTTTCCTCTGGATGAAGTGGAGACTTGACAGTGTAATTCCTAGGATCC
TGCTGGGGTGTTTGGTCTTCTCGGTGTTCATTAGCCTTCCTGTCATTAACAATTTGGATGATGATTTCAGGCATTGTGTC
AAGATGAAGTTGAAAACAAATATAAGTCGGAGATGCAGAGTACATAAAGCTCAGCATGCTTCCATCAAGATACGTCTCAA
TCTGTTGACACTATTTCCCTTTTCTGTGTCTCTGGTTTCATTTCTCCTCCTGATCCTCTCCCTGTTCAGACACACCAGAC
GAATGCAGCTCCGTGCCCCGGGGAGCAGAGATCCCAGCACGGAAGCTCACGTGAGAGCCATGAAGGCTGTCATCTCCTTC
CTCCTCCTTTTCATTGCCTACTACTTGGCCTATCTTGTGGCCACGTCCAGCTACTTTATGCCAGAGACTGAATTAGCTGT
GATCGTTGGTGAGTTGATAGCTTTAATCTGTCCATCAAGCCATTCACTCTTCCTAATTCTAGAGAACAAAAAATTAAGAC
AAGCATCTCTAAGGGTGCTATGGAAGGTAAAATATATCCTACAAAGAAGGAATTGCTAA

>OvmuTAS2R10A_HG926251.1:340905-340006

ATGCTGAGTATAGTAGAAGGCCTCCTCCTTTTTGTAGCAGTTAGTGAGTCAGTATTGGGGGTTTTAGGGAATGGGTTTAT
TGGACTAGTAAACTGCATTAACTGTGTGAAAAATAAGAAGATCTCTACACTCAGCCTTATTCTCACTGGCTTAGCCTCTT
CCAGATTTTGCCTGATATGGATAATAATTACAGATGCATATGCGAGATTGTTTTCTCCAGATATGTATTTGTCTGGTGAT
CTAAGTCAATATATAGCTTACTTATGGATAATTATGAATCAATCAAATGTCTGGTTTGCCACCAGCCTCAGCATCTTCTA
CTTCCTGAAGATAGCCAACTTTTCCCACTGCATTTTTCTTTGGCTGAAGGGTCACATCAATAAGATCCTTCTTCTTCTAA
TGGGATGTCTGCCCATTTCATGGTTATTTACTTTCCCAAACATTACAATGCCTTTTATTAATAATATTATGAAGAACAGA
AATACAACCGGGTTGATCACCATGCAGAAAAGTGAATACTTTATAAATCAGATTTTGTTCAGTATTGGAACACTTCTTGT
CTTTATACTGTGCCTGATTACATGTTTCTTATTAATCACTTCCCTTTGGAAGCACAACAGGAGGATGCAGTTGAATGCCA
CAGGATTCAGAGACCCCAGTACAGAAGCACATATCAAAGCAATGAAGATCTTGGTGTCTTTTATCATCCTCTTTATCCTG
TATTTTGTAGGCACTGCCATACAAATATCAATTGATACTATGCCTAAAAACAAACTGCTGTATATTTTTGGTATGACAAC
CACTATCCTCTATCCCTGTGGACACTCATTTATCCTAATTCTTGGAAACAGCAAGCTTAAGCAAGCCTCTTTGAGGGTAC
TGAAGCTATTAAAGTGCTAG

>OvmuTAS2R10B_HG926251.1:370190-369261

ATGCTGAGTATAGTGGAAGGCCTCCTAATTTATGTAGCAGTTAGTGAATCAGTATTGGGGGTCTTAGGGAATGGATTTAT
TGGAGTTGTAAGCTGCATTGATTGTGTGAAAAGCAAGAAGATCCCTACTGTCAGCCTTATTCTCACTGGCTTAGCTTCTT
CCAGATTTTGCCTGATATGGATAATAATTACAGATGCATATGTGAGGATGTTTTTTCCAGATACATATTTGTCTGGTAAT
CTAAGTCAAAATATAGCTCACTTTTGGATAATTATGAATCAGTCAAGTATCTGGTTTGCCACCAGCCTCAACATCTTCTA
TTTCCTGAAGATAGCCAATTATTCCCACTGCATTTTTCTCTGGATGAAGGGTCACATCAACAGGGTTCTTCTCCTTTTCA
TGGGGTCTTTGCTTATTTCATGGTTATTTGCTTTTCCAAGCATTGCAAAGCCTAGTATTAATGATATTATGAAGAACAGA
AGCTCAACCTGGCTGATCGCCCTGCATAAAAGGGAATACTTGACAAATCATATTCTGCTCAATATTGGAGTCATTCTTGT
CTTTGTGCTATGCCTGATTACATGTTTCTTATTAATCACTTCCCTTTGGAGACACAACAGAAAGATGCAATTGAATGCCA
CAGGATTCAGAGATCCCAGCACTGAAGCACATATCAAAGCAATGAAGACTTTGGTGTCTTTTATCATCCTCTTTATCTTG
TATTTTGTAGGCACTGCCATACAAATATCAGGTAGTACTATGCCTGAAAACAACCTGTTGCTCATTATTGGTATAACAAC
CAGACTCCTCTATCCCTGTGGACACTCGTTGATCCTAATTCTAGGAAACAGGAAGCTGAAGCAAGACTTTTTGAGGGTAC

TGAAGCCATTAAAGTGCTGGGGAAAAGAGAAACTTCTTAGAATTCCATGA

>OvmuTAS2R11_HG926251.1:401489-400545

ATGTTGAATACATTGGAGAAAGTTTTCATGGTTGTGACTGGTGTGGAATTTATAATAGGAATTTTAGGGAATGGATTTAT
TGGACTCACAATTTGCATTGCTTGGATTAGAAATCAGAAGTTGAGCTTGGTTGACTTCATTCTTACTAGTTTGGCCTTTG
CCAGAATCAGTCAATTATGGATAACCACTGTCATGTTTTTTTCAATAATGTTCTATCAGGCAGGCTTTGGTACTGTGGGA
AGAAAATATATCTTTTTTTGTATCTGGATACTGGCCAGTCACTCAAGCACTTGGCTTGCTACTTGCCTTGCTGTCTTTTA
TTTCCTGAAGATCGCCAGTTTCTCCCATCCTCTTTTCCTTTGGCTAAAATGGAGAATTAACAAGGTTGTTTTTATGTTTC
CACTGTTATCTGTGCCCTTCCTAGTCATAAGTTTTCCTTGGCCATATACTGTTGATGTCTTCTGGTGTCATGTCCAAAAG
ATGCATGCGAGAAATATGACTGAATTATGCAATGTGAATGAATATAAAAATTTAAATTTTATTATTATCTACACTGTGGT
GTCCCTCCCACCCTTCTTCCTTTCCCTGATTTCCTTTCTCCTGTTGCTCCATTCTTTGTGGAAACACAAGAAGAACATTG
CACACACTGTCAGGGATTCCAGAGACCCCCATGTGGAGGCCCATTTCAGAGCCATGAAAACTGTGTTTTTCTTTCTCATG
CTCTTTGTCCTGTACCAATTTGGCCTTTTCATGACATTTGGGGGACATATTTTCCTACAGAACAAGCTGGTTGTGATGTT
TGGTTATATGTTAGAAATGCTGTATCCTTCAAGTCATTCATATGTTTTAATTTTTGGAAACAGCCAAATGAGGAAATTCT
TCTTGGTGATTCTTAGGCACCTGAAGTGTGGCCTGAAAGGAAGGCACTGTTGGCTGCGTAGGTAG

>OvmuAST2R12_HG926251.1:414284-413362

ATGGAGAGAACACTGAACAATATACTTATGATCATTTCTGCTGGAGAATTCTTACTGGGTATTTTGGGAAATGGATTCAT
TGTCCTGGTTAACTGTATTGATTGGATCAGGAGCAGGAAGTTCTCCCTAATTGACTTTATTCTCACCTGCTTGGCTATTT
CCAGAATATTTGTGCTGTGCATAATGATTTCAAGTACAGGTTTATATGTAATCTCTGAGGAAATACAGTACAACAAGAAT
CTCCTGATAAATTTGGGGTTCCTCTGGACAGGATCCAATTATTTCTCCATAGCCTGCACCACCTGCATCAGTGTCTTCTA
TCTCCTCAGAATAGCTAACTTTTCTAATTTCCTTTTCCTCTGGATGAAACGGAGAATTCACAAGGTGCTTCTCATTATTG
CACTGGGGGCTGTCTTCTCTTTCTGCTTGTGCCTTCTTCAAAAGAATATGGCAGTTGAAATCCTGTTCCAAAACCAGGTA
AACAGCAAAAAAAATGTGACATTGGACTTTCTAATAAGATACGATTTGTTCTTTGCCATAATGTTCCTCATCCCCTTTGT
AGTGTCCCTGGCCTCCTTTCTCCTTTTAATCCTCTCCTTATGTGGTCATCTCAGGCGTATGAAGGGTGTAGACTGTAGCT
CGGAAGCCCATGTGAGAGCCCTGAAGGCTATGATTTCATTCCTACTCCTCTTCATTCTACACTATTTGAGCAATATTATG
ACAGTGTGGGCCAATCACATTCTCGGTAGTTTTGTGGCAAAGATTTTTGTGAACATGCTGTTATTTTTCTGTCCTTCTGG
CCACCCTTTGCTTCTGATTTTGTGGAACAGCAAATTGAAAAAGGCTTCACTCTGTGTCCTAAGGAAGCTGAGGGGTTACA
TGAATCTAAGAAAACTCTTCCAAAAATAA

>OvmuTAS2R16_HG925256.1:1192041-1192946

ATGATAACCAGCCAACTCTCTGTCTTCTTCATGCTCATCTATATGCTCGAGTTCTTGACAATAACTGGGCAGAGCAGCCT
GATTGTCATAGTGCTGGGCAGAGAGTGGGTGCAGACTCAAAGGCTGCCACCTGTGGACATGATTCTCACCAGCCTGGGCA
TCTGCCGCTTCTGTCAACTGTGGTCATCGATGCTGCACAACTTCGGCTCCCACTTCCACCTGAATTACAATTTTTGGTGT
TTCGGGATCATCTGGCAATTTACTAACATCCTTTCCTTCTGGTTGACCAGCTTGCTTGCTGTCTTCTACTGTGTCAAAGT
CTCCTTCTTCAGCCACCCCATCTTCCTCTGGATGAAGTGGAGAATTGTGAGATGGGTTCCTCGGCTGCTGCTGGGCTCTC
TGCTGGTTTCTTGTGTGTCTACCATCTTTTCAGCGACTAGTTATTACATCATCATTCAATTCATCTCCATGAAGGATTTC
CCTAGGAACAGCACCATGCTTGAGAGACTAGAGGCGTTCCTGTGGGATTTTTCCACACTGCGGCAAGTAGTTGTATTGGT
TATTCCTTTCCTCCTGTTTCTGGCCTCCACAGTCTTGCTCATGGCCTTATTATCCCGACATCTGAAGCAGATGAAAGACC
TTCACACAGGCCACCCCATCTCCAGCCCGGAAGCTCACTCTGCCGCCCTGAGGTCTCTTGGCATCTTCCTCATCTTGTTC
ACCTTTTATTTTCTGACCGTGCTCGTCTCCATCTTGGATGTCCTATTTAATAAAGAGTCTTGGTTCTGGGCCTGGGAAGC
TATCATCTATGCATTAGTCTCTATTCATTCTACTTTACTAATGCTGAGCAGTGCCAAACTGAAAAGAGTTTTAAAGGCAA
GGTGCTGGAGCCTAGAAGCTGCCTGA

>OvmuTAS2R38_HG925097.1:205794-206801

ATGGTGACTCTGACTCACATCGCATCTGTGCCCTCTGAAGTCAGGAACGCATTTCTGTTCTTTTCAGTCCTGGAGTTTGC
AGTAGGGATCCTGGTCAACGCCTTCGTTTTCTTGGTGAATTTCCGGGACCTGGTGAGGAGGCAGCCACTGAGCCACTGTG
ATCTTGTCCTGTTGAGTCTCAGCCTCACCCGGCTTGTCCTGCACGGGCTGCTCTTTCTGAAGGCCATCCAGCTTACTCAT

TTCCAGCGAATAAGAGACCCACTGAGCTTCAGCTACCAGACCATCATCGTGCTCTGGATGATCATCCACCAAGCCGGCCT
CTGGCTCACCACGTGCCTTAGTCTCCTTTACTGCTCCAAGATTGTCCGTTTCTCTCACGCCTTCCTGCTCCGTGCAGCAA
GCTGGATCTCCAGAAAGATCCCCCAGATGCTTCTGGGTGCTGTGGTTCTCTCCTGTGTCTGCACTCTTCTCTGCTTATGG
GACTTTTTTAGTGGATCTCGTTTCTCAGCTGTAACTAGGCTACTGACGAATAACAGTACTGAACTCAATTTGAACATTGC
AAAACTCAGTTTCTTTCATTCCTTCCTCTTCTGCAGCCTGGCGTCCATCCCTTCTTTCTTGCTTTTCCTGGTTTCCTCTG
GGATGCTGGTGTTCTCCCTGGGGAGGCACATGAGGATGATGAGGGCTGAAACCAGAGGCTCCCGGGACCCCAGCCTGGAG
GCTCACACCCGGGCGCTCAGGTCTCTCGTCTCTTTCTTCTGCCTGTATGTGCTGTCACTCTCTGCTGCCTTAGTCTCGAT
GCCGTTGCTGACACTGTGGCACAGCAAGGTTGGGGTGATGGCCTGCATAGGGATAATGGCAGCCTGTCCCTCAGGACATG
CAGTCATCCTGATCTCAGGGAATGCCAAGCTGAGGAGGGCTGTGGACTCCATTCTGCTTTGGGCAAAGAGCAGTTTCAGG
GTAAGGATGGACCACAAGGCAGATCCCAGGACACCAGATCTGTGTTGA
>OvmuTAS2R39_HG925611.1:169614-170660
ATGACAAGTGGGAGCTATCACAGACCCGCACATCAAGCGCTAAGGAGCCGTTTTCCTCCAGACATCAAAGAAGAGCAACC
ACTCAGGATGATCCAAACCTGCAGTTCCTCAGAAAAGGATCTGTCACCATCTCTTGTCACTTTGATGTTAATAATTATCG
GCACGGAATGCATCCTTGGTATTCTCGCAAATGGGTTCATTGCAGCGATAAACACAGCTGAATGGATTCACAATAAGGTA
CTCTCCACTAGTGGCAAGATCTTGCTTTTCCTGGGTGTATCCAGAATAGTTCTACAAAGCTTCATGATGCTAGAACTTAC
CTTAAGCTCAACATCCCCACAGTTTTATAATGACGACATCACGTATCACACATTCAGAGGATGTTTCATGTTCTTAAATC
ATTGCAGCCTCTGGTTTGCTGCCTGGCTCAGTGTCTTCTATTTCGTGAAGGTGGCGAATTTCTCCTACCCCCTTTTCCTC
AAGCTGAAGTGGAGAATTTCCAGACTGATGCCCTGGCTTCTGCAGCTTTCAGTGTTTGTTTCCTTGGGCCAGAGTGTGCT
CTTCTTCCAAAACATCTATACTATGAATTGTAACAATCTTTTTTCTCTCCCCTCCTTCAACTCCACTAAGAAAAAGTCCT
TCTCGGAGGCCACTGTGATCAACCTGGTTCTTTTCCTTAACCTGGGGATCTTCATCCCTCTGATCATGTTTATCCTGGCA
GCCACCCTGCTGATCATCTCTCTCAAAAGACACATCTTCCACATGAAAAGCAATGCCACTGGCTCCAGAGATCCCAGCAC
GGAGGCTCACCTGGGGGCCATCAGAGCTATCAGCTACTTTCTCATTCTCTATATTTTCCAAGTACTTGCTCTTTTTCTCT
ACATGTCCAACTTCTTTGACATCAATAGTCCCTTGAATATTTTGTGCAAAATCATCATGGCTACCTACCCTGTGGCCCAT
TCCATTCTACTGATTCAGGACAACCCTGGGCTGAAAAGAGCCTGGAAGAGGCTTCAGGCTCAAGTCCACCTTTATTTTAA
AAAGTAG
>OvmuTAS2R41_HG925611.1:474244-475179
ATGCACCCAGAATTCACAGTCCTCTTCATGCTGCTCTTTGTCCTGCTGTGTATCCTGGGCCTCCTGGCCAATGGCTTCAT
TGTGCTGGTGCTGAGCAGAGAATGGGTGCGACGTGGGAGGCTGCTCCCCTCTGACCTGATCCTCTTTAGCTTGGGACTCT
CCCGCTTCTGCCTGCAGTGGGTTGGAATGGGGAATAACTTCTACTATTTCCTGCATCTGGTCGACTACTGCAGTGGTCCC
GCCCGGCAGTTCTTCGGTCTACCCTGGGTCTTCCTCAACACCGTCACTTCCTGGTTTGGCTCCTGGCTCAGAGTCCTCTT
CTGCATGAAGATTGCTAACTTTACCCACCCCGCCTTCCTCTGGCTAAAGTGGAGGTTCCCCAGGTGGGTGCCCTGGCTTT
TGCTGGGCTCTCTGCTCACCTCCTTCACTGTCACCCTGCTTTTTTTTTCAGGGAACCACGCTTTGTATAAAGGGTCCTTC
ACTAGAAAACCTTTCAGGAACATGACCTATCATCAATGGAGCAGGATTCTGGAAATGTACTATTTCCTGCCCCTGAAAAT
GATCACTCTTTCAGTTCCTGGCTCTGTTTTTCTGGCCTCGATTGCTCTGTTGATTCACTCTCTGAGGAGACACGCATGGA
GGATGCAGCGCAGTGGTCACAGCCTGCAGGATCCCGGTGGCCAGGCTCACACCAGAGCTCTGAAGTCACTAGTCTCCTTC
CTTGTTCTTTATATTCTGTCTTTCGTGTCCCTGATCGTTGATGCTGCAGGGTTCTGCTCCTCAGACAGTGACTGGTACTG
GCCATGGCAAATTTTAGTCTACTCGTGCACGTCCATCCATCCCTTTATCCTCATCCTTGGCAACCTCAGGCTTCGAGGGG
CATCTGGGCAGCTGATTTTGTTGGCCAGGGGCTTCTGGATGGCCGAGGTGGTGTGA
>OvmuTAS2R60_HG925611.1:445533-446486
ATGAGCGGAGAGGACGTGGTTCCAGGACCTCAGGTGGTTGATAAGACAGCCCTCATCTGCATTGTTATTTTATTCCTTTT
GTTCCTGGTGGCATTGGTAGGTAATGGCTTAATCATCGCGGCACTGGGCAGCGAGTGGCTGCTGCGGAGAACGTTGTCAC
CCTGCGATAAGTTATTGGTCAGCCTGGGGACCTCTCGCTTCTGCCTGCAATGGGTGGTAATCAGTAAGAACATTTACATT
TTCCTGAATCCAGCGACCTTCCTTTATAGCCCTGTGTTCCAGCTCCTGGCCGTTCAGTGGGACTTCTTGAACTCGGCAAC
ACTGTGGTTCTCCACCTGGCTCAGTGTCTTCTACTGTGTGAAAATCGCAACCTTCACCCACCCCGTCTTCCTCTGGCTAA

AGCGGAATGTATCTGGGTTGGTTCCTTGGATGCTACTCAGCTCTCTGGGGTTCTCTACCTTTACCACCGTTCTATTTTTC
ATAGGCAACCAGAGAATGTATCAGAACTATTTAAGGAAGGGTCTGCAATCTTGGAATGTCACTAGGAATGCTGTGAGAAC
GTATAAGAGGTTCTACCTCTTCCCTTTGAAAATTGTTACCTGGACCGTCCCTACTGTTGTCTTTATTGCGGGCACGGTTT
TGCTCATTACATCTCTGGGAAGACACACCAAGAAGGTCTTCTTCTCCATCTCAGGCTTTCACAGTTCCAGTGCCCAGGCA
CACATCAAGGCCCTCTTGGCTTTTATCTCCTTTGCTATCTTCTTCACTTCCTCTTTTCTGTCACTGGTTCTCACTGCCTC
AGGTATGTTTCCCTTTCGGGAGTTCCGGTTCTGGATATGGCAGATTGTGATTTATCTGGGTACAGCAACCCACCCCATTA
TTCTTCTCTTAAGTAACCGCAGGCTGAGAGCTCTGCTAGGGAGGGGCTGCTCCTCAGCACATGGGGCATCTTGA
>OvmuTAS2R67A_HG926401.1:95269-94331
ATGCCATCTGGAATTGAAAATACTTTTCTAGTAGCAACAATAGGAGGATTTGTGATTGGAATGTTGGGGAATGGGTTCAT
TGTACTAGTTAACTGCATTGACCTGGTGAAGAGACAAAAGCTCTCATTAGCTGACTGCATCCTCACAGGCCTGGCTATCT
CCAGAATCAGTCAACTTTGGGCAATACTATGTGACTCATTTTTATTGGTACTATGGCCACACCTATATGCCATTGATAAA
CTAACAAAGGTTGTTAATAGTTTTTGGATACTGTCCAATCACCTAGCTACCTGGTTTGCCACCTGTCTAAGTGTTTTCTA
CTTCTTTAAAGTAGCCAACTTCTCCCACCCCTGCTTCACTTGGCTGCGGTGGCGAATTCGTAGTGTGGTACTGGTGCTTC
TCTTGGGGTCTTTGTCCTTACTGTTTTTGAATTTTGAATTAATATATGTGTTTAGTCGTGTTTGGACTAATGGCTACAAA
ATATATGCAAGAAATTCAACGTGGTCCCCAAATGTAAGTGAAACTCATGATCTTCACCTGTTGATTGTTTTTAACTTCAT
CAACTTAATCCCCTTTCTTCTGTCCCTGACCTCACTGCTCCTCTTAGTCCTCTCCTTGATGAGGCACATCAGGAATTTGC
AGCTCAACCCCAGCTCAAAGGATCTCAGCACAAAGGCCCATAAAAGAGCCATGAAAATGGTGATGTCTTTCCTCTTCCTC
TTCGTCGTTCATGTTTCTTCCATCCTGTTAACAGGTTGGGTTTTCCTTAAACTGCAGGGACGTCTGGCCAAATTGGTGGT
TGTGTTAACTTCGACTGTTTTTCCTTCAAGCCACTCATTTATCCTAATTTTGGGAAATAGCAAGCTGAGACAAAATGCCA
TAGGACTATTGTGGTATCTTAACTGCCGCCTGAAAAGAGTGAAATCTTTAGCTTCATAG
>OvmuTAS2R67B_HG926401.1:86318-85383
ATGCCATCTGGAATTAAAAATACTTTTCTAGTAGTAACAATAGGAGGATTTGTGATTGGAATGTTGAGGAATGGGTTCAT
TGTACTAGTTAACTGCATTGACCTGGTGAAGAGACAAAAGCTCTCATCAGCTGACTGCATCCTCACAGGCCTGGCTATCT
CCAGAATCAGTCAACTTTGGGCAATACTATGTGACTCATTTTTATTGGTACTATGGCCACACCTATATGCCATTGATAAA
CTAACAAAAGTTAGCATTTTTTGGACATTGTCCAATCACCTAGCTACCTGGTTTGCCACCTGTCTAAGTGTTTTCTACTT
CTTTAAAGTAGCCAACTTCTCCCACCCCTGCTTCACTTGGCTGCGGTGGCGAATTCGTAGTGTGGTACTGGTGCTTCTCT
TGGGGTCTTTGTCCTTACTGCTTTTGAACTTTGAATTAATACATGTGTTTAATAGTGTTTGGACTAATGACTACAAAATA
TACGCAAGAAATTCAACGTGGTCCCCAAATGTAAGTGAAACTCATGATCTTCACCAGTTGATTGTTTTTAACTTCATCAA
CTTAATCCCCTTTCTTCTGTCCCTGACCTCACTGCTCCTCTTAGTCCTCTCCTTGATGAGGCACATCAGGAATTTGCAGC
TCAACCCCAGCTCAAAGGATCTCAGCACAGAGGCCCATAAAAGAGTCATGAAAATGGTGATGTCTTTCCTCTTCCTCTTC
GTCATTCATATTTCTTCCGTCCTATTAACAGGTTGGGTTTTCCTTAAACTGCAGGGACGTCTGGCCAAATTGGTGGTTGT
GTTAACTGCAACTGTTTTTCCTTCAAGCCACTCATTTATCCTAATTTTGGGAAATAGCAAGCTGAGACAAAATGCTCTTG
GACTACTGTGGTATCTTAATTGCCACCCCAAAAGAGTGAAATCTTTACCTTCATAG
>OvmuTAS2R5P_HG925097.1:344888-343995
ATGCCGACTTCTATCCCAGGACTGATGATGCTGGTGGCAGTGGCTGAATCTCTCATTGGCCTCACTGGAAATGGAGTTCT
TGTGGTCTGGAGTTTTGGAGAATGTCTTCGAACGTCCAGGGAGTCCTCGTATAACCTCATTGTCCTGGGCCTGGCGGTCT
GTCGGTTGCTTCTACAATGGTTGATTATGGTGGACTCAAGTCTGTTCCTGCTTTTCCAGAGCGGCCATTGGCTTCGCTGT
CTCAGTGTCTTCAGGGTTCTGGTAAGCCAGGCCAGCCTGCGGTTTGTGAATTTTCTCAGTGTCTTTTATTGTAGGAAGAT
CATGACCGTTGAACACCCTGTCTCCTTGTGGCTGAAGCAGAGGGCCTGTTACCTGAGTTTCTGGTGCTTTCTGGTGTACT
TCATGATCCATTTGTTATAGTTAGGGGTAGCTTAGACTTCTCCAGTCCTTCCCAAGGAAACAGCAGCATCTTATTCCCCA
TTTCAAACTGGCACTATGGATGTATATTACAGCTCAATACAGGAAGTATGATGCCTTTCATGATGTTTCCTCTTTCCTCT
GGGCTGCTGATTGTCTCTTTGTATAGACACCACAGGAAGATGAAGGTCCATACAGCTGCGCCGGGGGCCAGCATGAGGAA
TTCCGCCCATGGCAAAGGTCATGAGGAAGGAGACTTGGCATACGCAAAGGCATGATCAAGCCTCAGGAAACCCCCTGTTC
CCGAGCATCTAACCCCAAAACCAGAGTCTGTTTTATGCTCTCACCTACACCTCTGACTTTACGGGGGGTTCTCCCCCATA

ACCGTTTCCCTCGGAGAAGGAGTAAACGTGCAGCTCCAAGGCAATAAAAATTCTTGGGCGTGACAAGACTGTTTCAGCTT
ACGGACTCCTCTGA
>OvmuTAS2R8P_HG926251.1:316186-315258
ATGTTCAGTATAGAAGACCACATCTTTCTGACCATAACGACTGCAGAATTCATCATAGGAATGTTTGTGAATGGATACAT
TGGACTAGTAATATATATTGATTGGATTAAGAAGAAAAAGATCTCCACAACTGACTACATCCTCTCCTATTTAGCTCACT
CCAGAATTTGTTTGCTTTGTGTAATGACACTCAGCGGCACCATACTGGCACTCTACCCAGGTGTTTACGAAAATGAGAAA
ATAAAGGTAGTTCTTAATATCTTCTGGACATTCACCAACTACTTAAGTATGTGGTTTGCCACCTGCCTCAATGTCTTCTG
TCTCTTCAAGATAGCCAGTTTCTTCCACCGACTTTTTCTCTGGCTGAAGTGGAGAATCAAGAGGGTGTTTCACTGGAGCC
TGCTGGGGTCCCTGGCCATTTCCATGTTGATCAGCCTTATACAAGCAACATTAACAAATTCTGATTATGAATTTCTCAAA
ATTGAAAACTTAAAAGAAACGTCACCGAATTGTTCCATGTGAGTAAAATTCAATACTTCAACCCACTGACACTGTTTAAC
TTGTCAGCAATTATTCCATTTACTGTGTCATTGATCTCATTTTTCTTTTTAATTACGTCCCTATGGAGACACAGCAAACA
AGTGAAATCCAGTGTAACAGGTGCCACAGACTCCAGCACAGAGGCCCACGTGGATGCCATGAAAACAGTGACCTCATTTC
TTTTCTTCCTTTCTGTATACTACCTGGCCTGTCTTTTGGCAACATTTAGCTACTTTATGAAAGAAAGCAAGTTAGCTATG
ATGTCTAGAGAGATCATAGCAATTCTTTATCCCTTAGGTCACTCACTGTTTTTAATTGTTGGAAATAACAAGCTGAGGCT
GGCAGCTGTCGGGATGCTGAGATGTGGGAAAACAGTCTGCATGATGTAA
>OvmuTAS2R9P_HG926251.1:318051-317120
ATACCAGGTACAATGGAGGCAATATATATGTTCTTGATTGCTGGTGAGTGGATGATAGGAATTTGGGGAAATGGATTCAT
TGTACCGGTAAACTGCAGTGGCTGGCTCAAAAAGAGAGCTGTCTCCTTGACTGAGGTCATCCTGGTCAGCCTGGCCACCT
CCAGAATCTGTTTTTTGTGTGTGATATATATGGATGGTTTTATTATGGTACTCTTTCCAGATACATACAGGCATGGTGAG
ATGATGAACATTTTGGGTATTTTCTGGACAACTTGCAATCATTCAACTGTCTGGTTTACTTCGTGCCTCAGCGTCTTCTA
TTTACTCAAGATAGCCAGTATATCCCACCCAGTTTTCCTCTGGCTGAAGCTGAAGATGAACAGGGTATCCTTGGGATTCT
TCCTATGTCCTTTCTCATCTCCTCAATTGTCAGTGTTTTACTGAATAATGATTCATTTTATAATGTCAAAATCAATAATG
AAGCAAACATTATTTAGGAATTCAAAGTAAGTAAAATCCCAACTGCTTTCAAATTGATTATCCTGAACCTGGGGGCTATG
GTTCCCTTTATTCTTTGCCTGGTCTCATTTGTCCTTTTATTTTTCTCACTTTAACACACCAAGCAAATGAAACTTCATGC
CACTGGGTCCAGAGACCCTAGCATAGAGGCGCACATGAGGGTCATAAAGACAATAGTCATCTTTCTGGCTCTTTTCATTA
TGTACTATGCAGTTTTTCTCATTGTAACATCTAGCTTTCTGATTCCTCATGGAAAATCGGAGCTGATGTTTGGTGGCCTA
AGAGCTGCCATTTTCCCATTGAGCCATCCATTCATCCTGCTAATGGGAAACAGAAAGCTGAGGGAGGCTTTTCTGAAGGT
GCTGGGGATTTTGAAGGGTTTCCACAAAAGAAGGAAATATTTTGTTCCCCAA
>OvmuTAS2R10CP_HG926251.1:329382-328636
ATGCTGAGAAGGCCTCCTCATTTTTGTAGCAGTTAGTAAGTCAGTACTGGGGGTTTTAGGGGATGGATTTATTGGACTTG
CATACTTCATTGAATGTGTGAAGAACAAGAAGTTTTCTATCAGCTTTATTCTCATGGGCTTAGCTACTTCCAGAATTTGC
CTGATAGGGTTAACAACTGCCGATGGATTTGTGAAGATATTTTTCTCCAGAAATGTGTTCCTGTGGTTACCTAATTCACT
GTATTACTTACTCATAGATAATTCTGAATCCATCAAGTGTCTTTTTTTGCCACTAGCCTCAGCATCTTCTATTTCCTGAA
GATGGCCAATTTTTCCCATCACATTTTTCTCTGGTTGAGGAGTGACATCAAAAGGGTTCTTCTCCTGCTGATGGGATACT
TGCTTATTTCATGGTTAGTTACTTTTCCACTAACTATGAAGATAATTAGTGATACTAGAGCAAAGAATAGAAGTGTAATC
TTTTCAGTTGAAGTGCATAAAGGGGAATTCTTTAGAAACCAGATTTTGCTCAATCTTGGAACCCTTCCCATCTTCATACT
ATGCCTGATTACATGTATCTTATTGCTCATTTCCCTTTGGAGGCACAACCAGAGGATGCTATTGAATACCACAGGATTCA
GAGACCCCAGCACAGAAGCACATATCAAAGCAATGAAAGTTTTGATATCTTTTATCATCCTTTTTATCTTGAATTTTATA
AGCATTATCATAGAAATATCATGCACC
>OvmuTAS2R10DP_HG926251.1:353987-353068
ATGCTAAGTATAACGGAAAGTCTCATTTTTGTAGCAATTAGTGAGTCAATATTGGAACTTTTAGGGAATGGATTTATTGG
ATTGGTAAGCTGCATTGACTGTATGAAAAACAAGATCTCTACTATCAGCTTTATTCTTGCTGGCTTAGCAACTTCCAGAT
TTTGCCTGATATGGACAATAGTTACTGATGGATTTTTAAAGTTATTCTCTTCAGATGTACATTCCTCTGGGAACCTAGTT
GAATATAATGGTTACTTATGGATAGTTATGAATCAATCAAGTATCTGGTTTGCCACCTGCCTCAGCATCTTCTATTTCCT

GAAGATATCCAGTTTTTCTCACCGCATCTTTCTCTGGTTGAAGGGTAGATTCAACATGGTTCTTTTCCTTCTTTGGGGAT
GCTTGCTTATTTCATGATTAGTTACTTTTCCACATTTTGTGAAGATTGTTAATGATAATAGAAAATAGAAACACAGTCTG
ATCAATGGATATGCATAAATGTGAACTCTTTGGAAAACAAATTGGGCTCCATCTTGGTGTCATTCCCCTTTTTATACTAT
GCCTGATTACATGTGTCTTGTTGCTCACTTCTTTTTGGAGACACAACAGGAGGATGCAATTGAATGCCACAGGATTCAGA
GACCCCAGTACAGAAGCACATATCAAAGCAGTGAAAGTCTTGGTGTCTTTTATCACCCTCTTTATCTTGAATTTTGTAGG
TACTGCCATACAAATATCAAGTGTGACAGTGCCTAAAAAACAAACTGCTTTTTATTTTTGATATGACAACCACAGTCCTC
CATCTCTGGGGTCACTTGCTTATCCTAATTCTAGGAAATAGGAACCTCAAGCAAGCCTCTTTGAGGGTACTGAAGCCATT
AAAGTGTGGGGAAAAAGAGAAACTTCTCAGAACTCCTTGG
>OvmuTAS2R13P_HG926401.1:10393-9488
ATGGCAGATTCTTTGGAAAACATCTTTATCATTTTAATAAATTCAGCATTCATAATTGGTATTCTGGGGAATGGATTCAT
AGCACTGGTGAACTGCATTGACTGGATCAAGATGCAAAAGGTCTCCTTGGCTGATCGAATCCTCACTGCTTTGGCAATTT
TCAGAATTGGTCTGATTTTGGTAATGATGGTGAGTTGGTTTACAAAGGAGTCTTATCCATTTTCTTCTTTAGACATAAAG
GGAAATAAAGTCATACTTTTTAGTATTGCTGGGCTCTTGGCCAATTATTTTAGTGTCTGGCTTGCCACAGGCCTCAGCCT
CTTTTATTTCCTCAAGATAGCCAATTTTTCAAATGCTGTTTTTCTTCACCTAAAGTTTAGAATTGGAATGGTATTTATGG
TAATGTTTCTGGGGACATTAGTATTGCTGCCTCTAAGTCTTACTTTGGTGAGCATCTATATTAATATCAAGATACATCCA
TATGAAAGAAATATGACTTTAAGTTCTAAAAGAAGTGACACTGAAACCTTTTCCAAATTGATCATATTCACCATGGGATC
TTTCTCATCCTTTATTATATCCCTGAGTTGTTTTCTCCTGTTAATGTTCTCCCTACGGAATCATGTCTAGAAGATGAGGA
GCCAGGGTTCAAGAGATCCCAGCAGCAAAGCCCACGTCAGAGCCATGATCATGGCGATGCCTTTCCTCATACTACTTGCC
ATTCACTTCCTATCTCATCTCATGACAACTTTTCATCACAATGTGATGCAGAGTGAACTGGCCTTTATGCTTGCTGAAGC
TCTTGGAACTATTTATCCTTCAGTCCACTCATTTGTCTTGATTCTGGGAAATGACAAGCTAAGAAAAGCTTCACTTTTGG
TGCTGTGGCAGTTGAGGTGTGGCTGA
>OvmuTAS2R40P_HG925611.1:229417-230372
ATGGTGACAGTGAACACGGATGCGATAGATAAAGACCCGACCAGGTTCAAGATCATCTTCACCTTGGTGGTCTCTGCAAT
AGAGTGCATCATTGGTATCGTGGGGAACGGCTTCATCACCGTCATCCACGGAGCCGAGCGGGTCAGAGGCAAAAGACTCC
CCATTGGTGACTGCATTCTGCTCATGCTGAGCTTTTCCAGGCTCTTGCTACAGATCTGGATGATGCTGGAAAACACGTAC
AGTCTGCTGTTCTGGGTCATCTACAATGAGAAAAGAGTATACATACTTTTCAAAACCATCGTCATGTTTCTGAACTACTC
CAACCTCTGGCTTGCTGCCTGGCTCAATATCTTCTATTGCCTCAGAATCGCAAGCTTTACTCACCCGTGGTTCTCCGTGA
TGAAGAGGAAGGTCAGGGGCTGATGCCTGGGCTTGTGAGGCTGTCCTTGTTCTTCTCCTTTTGCTCCAGCTTCCCCTTCT
CTAGAGGCATCTTCAATGTGTACGTGAACAATTCCGTCCCCGTCCCCTCTTCCAGCTCCACTGAGAAGGTGTACTTCTCC
GAGACCAACATGGGCGACTTGGTTACCACCCTTTACCTGGGGATCTTCATCCCTCTGATCATGTTTATGCTGGCGGCCAC
CCTGCTGATCATCTCTCTCAAAAGACACACCTTCCACATGAAAAGCAACGCCACTGGCTCCAGGGACCCCAGCATGGAGG
CTCACCTGGGGGCCATCAAAGCCATCAGCTATTTTCTCATCTTCTACATTCTCAATGCAGTTGCTCTGTTTCTTTCCATA
TCCAACATCTTTGCCACCAACAGCTCCTGGAATATTTTGTGCAAAATCATCATGGCTGCCTACCCTGCTGGCCACTCAGT
GCTACTGATCTTAGGCAACCCCAGGCTGAAAAGGGCATGGAAGCGGTTTCAGCACCAAGTTCATCTCTACCTGTAA
>OvmuTAS2R42P_HG926401.1:107396-106466
ATGTTCCCTGGGTTGAGTACAGTCTTTCTGATACTGTCAGGAGTGGAATTCTTAATCGGAATTCTAGGCAATGTGTTCAT
TGGACTGGTACTCTGCTCTGAATGTGTTAAGAACCAAAAGACATCTTTATTTGACTTCATCCTCACTGGCTTGGCTATCT
CCAGAATCAGTCAACTGTTGGTGTTTTTTGTGGAATCACTTATAATAGGACTAGAACCACAGGTATTTGCCATTTTTAAA
CTAGCGAAGCCCATTGCTTTACTTTGGAGAATATCTAATCATTTGACTACCTGGCTTGTCACCTGCCTAAGTATTTTCTA
TCTCCTTAAGATAGCTCATTTCTCCCACTCTCTTTTTTTCTGGCTGAAGTGGAGAATGAACAGCGTCATTCTTGTGATAC
TTGCATTTTCTTTGGTCTTTCTGATTTTGGACATTCTTTTGCTAGAAACATTTAATGATCTCTTCTGGAATTTAATAAAT
GAAGGCAATTTGACTTTAGTTGAAAGTAAAACTCATTATATTAAAAGCGAGAGTCTTCTTAGTTTCTCCTATTTCATTCC
TATTGTTCTGTCCCTGCTCTCATTTTTTTTTTTAATTTCGGTCCTTGGTGAAACACACCAGAAATTTGCATCTCAATTTT
ATGGGTTCCAGGGACTTCAGCACAAAGGCCCATAAAAGAGCCATGAAAATGGTGACGTCATTCCTCCTCCTTATCATGGT

TCATTTTCTTTTTACACAATTGGCAAATTGGATGTTTCATAGGTTTTTGGACAGTAAGTTCACAAAGTTCATCATGTTAG
CACTATATGTCTTTCCTTCAGGCCACTCGTTCATGTTGATTCTGGGAAACAACCAGTTAAGACAGATAGCCTTGAAGGTA
CTGAAGCATCTTAAAAGCTCCTTGAAAAGACAAAATCCATTGGCTTTATAG
>OvmuTAS2R18P_HG926401.1:35117-34144
ATGTCAGTTGGAACAAAGGTCTTTCTAGCGGTGTCAACAGGAGAATTGATCTTAGGAGTACTGGGAAATGGGTTCATTGG
ACTGGTAAACTGCATCCAGTGGATCAAGAATGGAAAGGTTTCATCAGCTGAGTTCATCCTTACTTGCTTGGCTATGGCCA
AAATCATTCAGCTGTGGGTAACACTTTTGGATTCACTTATAGTAGGATTAGCTCCACATCTGTATGCCACTGGTAACTAG
TAAAAGTAGTTATTCTTCTTTGGGCACTAATGAATCACTTAACTATCTGGTTTGCCACCTGCCTAAGCATATTTTACTTC
CTTAAGATAGCCACTTTCTCTCACTTCTTTTTCATGTGGCTGAAATGGAGAATGAACCGAGTGCTTCTTGTGCTTTTCCT
GGCCTCTTTCTTCTTATTAACTTTTGACCTCTTAATGCAAGATGCTCTTGGTGAGTTGTGGATGAACACCTTTAGAGAAC
CTGAAAGGAAACTTTGCATTTAGATGCAAGTAAAATTTTCTATCTTAAAAGTCTGATTCTTCTCAGCTTGACATATGTTA
TCCCTTTCATTCTCTTCATGGCTTCTTTGCTGCCTTTCTTTCTTTTCCTGGTGAGACACATCAAGAATTTCCAAGTCAAC
TTGAACCACCGAGAGATTTCAGCACAGAGGCCGATAAAAGGGCCTTGAAAATGGTGACAACATTTCTCCTCTTCTTTATC
GTTTACTTTATTTCTACTCCAACTGGAAATTGGATCTTCCTTAAGCTACACTGGTATGAGGTCATGATGTTTGTCATGGT
GATTTCAACTCTCTTTTCGTCAGGCCACTCATTTGTTATAATTTTGGGAAACAGCAAGCTAAGGCAGGTTGTCTTCAGAC
TACTGTGGGGTCTTAAGTTCTCTAAAAACTAATCAAAACTTTAGCTTAATAGACATTAAAAAAACTTTCTGTATTATATG
TGGAAATACCTTAA
>OvmuTAS2R408AP_HG926401.1:50114-49201
ATGACAACTTTAGTATCGAGCATTCTTTCCATTCTACTGGTGACAGAATTTGTTTTGGGAAATTTTGTGAATGGTTTCAT
AGCACTGGTGAACTGCAATGACTGGGTCAGGAAACAAAAGATCTCCTCAGCTGATGGGATTCTCACTGCTCTGGCAGTCT
GCAGAATTGTTTTGCTCTGGACAATATTAATAAATTGGTATGCAACTATGTATAATCCAGCTCTATATAGTTTAAGAATT
GTTATCCGTGTTGCCTGGACAGTAAGCAACCATTTTAGTAACTGGCTTGCTACTAGCCTCAGTATATTTTATTTGTTCAA
GATAGCTAATTTCTCCAGCTTAATTTTTCTTCACCTGAAGTGGAGAGTTAAAAGTGTAGTTCTCATGATGATGTTGGGGA
CTTCATTGATTTTGTTTTTTCAGGTTGCAGTGTTAGGTATGAAACTATTCAGACAAGCGAATATGAAAGAAACATCACTG
AGAAGACCAAATTGAGGGACTTTTTACACCTTTCAAATATGACCCTGCTCACACTAACAAACTTCATACCCTTCAGTATG
TCCCTGACATCTTTTCTGCTGCTAATCTTTTTCCTGTGGAAACATCTCAGGAAGATGCAGCTCAACGGCCAAAGATCCCA
AGGTCCCAGCACCAAGGTCCACATAAAAGCCATGCAAACTGTCATCTCCTTTCTTTTCCTGTTTGCCACTTACATCCTGA
CTGTAATTTTAACAATTTGGAATTCTAATGAGCTGCGGAAGGAACCGGTCCAAATGCTTTTCCAGGCCCTTGCAATCACC
TATCCTTCAATGCACTCATTTATCCTGATTTGGACAAACAGGAACTTAACACAGACCTTTCTGTCATTTCTATGGCAGCC
AAGATGCTGGCTAAAAGTAAGAGGAACTAGGTAG
>OvmuTAS2R408BP_HG926401.1:39338-38408
ATGATAGTATTTATGTCAAACATTGTTTCCATTCTATTAATGCAGAATTTGTTCTGGGAAATTTTGCCAGTGGCCTCATA
GCACTGGTGAACTGCAATGACTGGATCAAGAGACCAAAGATCTCAGCTGATGGGATTCTCACTGCTCTGGCATTCTGCAG
AATTGTTATGTTCTGGGCAATGTTAATAAATTGGTATGTAATTGTGAATAATCCAACTCTATATAATTCAAAAGTAAAAA
TTATTGTTCATGTTGCCTGGACAGTAAGCAACCATTTTAGTAACTGGCTTGCTTCTAGCCTCAGTATATTTTATTTGTTG
AAGATAGCCATTTCTCCAGCCTAATTTTTCTTCACCCGAAGTGGAGAGTTAAAAGTGTAGCTCTCATGATGATGTTGGGG
ACATCATTAATCTTGTTTATTCAAGTTGCAGTGTTAAGCATAGGTGAGGCTATTCAGATAAAAGAATATGAAGGAAATGC
CACTCAGAAGACCAAACTAAGGGACATTTTACACCTTTCAAATGTGACTCTGTTCACGCTAACAAACTTCATACCCTTCA
GTATGTCCCTGACATCTTTTCTGCTGCTAATCTTTTCCCTGTGGAAACATCTCAGGAAGATGCAGCTCAATGGTAAATGA
TCCCAAGATCCCAGCACCAAGGTGCATATAAAAGCCATGCAAACTGTCATCTCCTTTCTTTTCCTGTTTGCTGTTTACAT
TCTGGCTCTAATTTTATCAGTTTGGAATTCTAATGAGCTTCAGAAAGAACAAATGCTTTCTGATGTTCTTTTAATCATGT
ATCCTTCAATCCACTCATGTATCTTGATCTGGGGAAACAGGAAATTAACTCAAGCCTTTCTGTCATTTCTGTGTCAGTCA
AGATGCTGGCTGAAGGAAAGGAAATAGGTGGAAACATGTCTTTTAGCATAA
>OvmuTAS2R408DP_HG926401.1:56703-55788

ATGATAACTCTACTATCAACCATTTTTTCCATCCTAGTAATAATACAATTTATTCTGAGAAATTTTGCCAATGGCTTTTT
AGCCCTGGTGAGCTGCATTGACTGGGTTAAGAGACAAAAGATCTCCTCAATTGATGTGATTGTCACTGCTATGGCAGTCT
CCAGAATTGTTTTGCTCTGTGTAATGTTAATACATTGGTATTATATTTTGCTTCATCCAGCTTTATATGGTTTTAAAATA
AGAACTATTGTTCATGTTGCCTGGACAATAAGCAATCATTATAGCACCTGGCTTGCTACTAGCCTCAGTATATTTTATTT
GTCGAAGATAGCCAATTTCTCCAGCCTAACTTTTCTTCACCTGAATTGAGAGTTAAAAGTGTAGTCCTCATGATGCTTCT
GGGAACTTCATTCATTTTGGTTTTACAAGTTGTAGTTAAAAGTATAAGTGGGACTATGCAGAGAAGTGAATTTGAAAGAA
ACTTCACACAGAAGACCAAACTGAGGGATATTTTATGGCTTTCACATGTGACCCTGCTCATTCTAGGAAACCTCACACCC
TTTACTATGTCCTTAATATCTTTTCTGCCACCAATCTCTTCCCTGTGGAATATCTCAGGAAGATGCAGCTCAATGGCAAA
GGATTCCAAGTTCCCAGGACCAAGATCCATACAAAAGCCATGCAAACTGTTATCTCCTTTCTCTTGCTATTTGCCTTTTA
CTTTCTGGTTCTAATCATATCAATCTGGAGTCCTAAAAAGTTGCATGAGGAACCGTTTCTCTTGCTTTTCCCAACAGTTG
AAGTCATCTATCCTTCAGTCCACTCATTTATCCTGATTTGGGGAAACAGAAAGTTAACACAGGCCTTTCTATTGTTTCTG
AGGCAGCTGGGGTGCTGGCTGAAAGACAGGAAATAG
>OvmuTAS2R408CP_HG926401.1:20548-19663
ATGATAACTTGACTACATTTTTTCCATCCTAGTAATAGAATTTATTCTAAGAAATTTTGCCAGTGGTTTCATGTCACTGG
TGAACTGCATTGACTGGTCAAGAGACAAAAAATCTCTTCAGCAGATGGGATTCTCACTGCTCTGGCAGTCTCCAGAATTG
GTCTTCTCTGGGTAACATTAATAAATTGGTATGTAAATGTGTTTAATCCCAGCTTTAGACAATTTAAGAGCAAGAATTAT
TATTATTGCCTGGATAATAAGCAACTATTTTGACAACTGGCTTGCTGCTATCCTCAGCATATTTTATTTGCTCAAGATAG
CCAATTCTCCAATATTATTTTTCTTTACCTAAAATGGAAAATTAAAAATATTCTTCTTGTTCACTTTGTCCTGTTTGGCT
TTATTAATTCATGGTGTAAACATAAATAAGACTATCCAGGCAAATGACTATGAAGGAAACATCACTCAGAAGACCAAGTA
GAGGGACACTTTACACCTTTGAAATATGGCTGTGTTCATGCTAGTAAACTTCACACCCTTTGCAATGTCCCTGACATCTT
TTCTGCTGTTAATCTTTTCCCCGAAGAAACATTTCAGGAAGATGCAGCTCAGTGGTAAAGGACCCCAAGATCCCAGCACC
AAGGTCCATATAAAAGCCATGCAAACTGTCTTTTATTTTCTATCTGCCATTTACTTACTGGTTCTAGTTTTCTGTTTGGA
GTTCTAATAGGCAGTGGAACAACTTGGTTATCATGGCATGCATGCCAGGCTTTTGGAATCATATATCTTTCACTTATCCT
GACATGGGGAAAACGGATACTAAGACAGTCCTTCCTGACATTTCTGCAGCAACTGAGTTGATGGCTAAAAGAAAGGAAAT
AAGTGG
>OvmuTAS2R62P_HG925611.1:435056-435981
ATGTCCCCTTCGCCCACATTGATCTTCAAGGTCACCCTTTTCCTGGAGTCATTGGTTGCCATGCTGCAGAATGGCTTCAT
AGTTACCATGATGAGCGGGGAGTGGCGTGTAGCTGGACTCTGCCCGCCAGTGACATGATTGCGGCCTGCCTGGCTGCCTC
CCGGTTCTGTCTGCATGGGATGGCCCTCCTGAACAACCTCATGGCCTCCTCTGGCTTTTGTTCCAAAATCTACTATTTCA
GCATCCCCTGGGATTTCATCACCTCCCTCAGTTTCTGGCTGACTGCCTGGCTTGCTGTCTTCTACTGCACGAAGATCTCC
CTCTTCTCTCACCTCGTCTTCTTCTGGATAAAGTGGAGGATTTCTCGATTGGTTCTCCAGCTGCTGCTGGGTTCCTTGAT
CTTATCTGGTCTGACTGTCATCTCCTCAGCTGCTGGGAATACAATTCTTGCCCAGATGACGGCTGCCCAGAGTTCCCATG
GAAACGCCCTGGCTGGTAGCATACACGCTGTCTATTTGCACTGTTTTCTACCTCATGTAATTCTCATGAGGTTGGTTCCA
TTCCTCCTGTTCCTGGTGTCCACCTTCTCACTCATGGTCTCGCTGCGCCGGCACCTCGGGCAGATACAGGACCACAGACC
CAGCCCACATGATCCCAGTACCTGGGCTCACACCATGGCCCTGAAGTCACTTGCCTTCTTCCTCGTCTTCTACACCTTGC
ACTTCCTGTCCCTGGTTATCATTGTGTACATCCCAGCCTTCCAGAAACACTGGCCCTGGGCCTGTGAGGTGGTGACCTAA
GCAGGCATCTGTCTGCACTCCAGCATCTTGATGCACAGCATCCCCAAGCTGAGAAAGGCCCTGAAGAAGCAGCTTTGGTG
AGCCCTGGACAAGGACCAGTTTGTCTCCAGTTATCAGTATCAATAG
>OvmuTAS2R372CP_HG926251.1:363761-363230
CAGCAGGTAATAAGAGGAGTTTTTGAGTTCTCCTCAAATCTGAGAAACTCCCGAAGTACTGGATGTGCTGCCTGCCTCAG
CGTCTTCTACTTCCTCAAGCTATCTAGTTTTTCCCACCTCTTCTTTCTCTGGCTAAAGTGCAGAAGAGATAGAGTTTTTT
TCACTATTATGTTGCAATTCTGTCTCTCTTTGATTTTTAATCTTCTGAGCATAAAATTTATACTTTTGTGTTCAGCAAGC
ATTTAGAAAAGGAAAGTCTTAACTTGGAAAAAAGGTTTGCATAAAAATCAGTATCACAGCAGTCAAGTTCTCTTCAGCCT
TGGATCTCTCATTTCCTTGTCTGTTATCACTCATTCTATTTTTCCTGTTACTCTTTTCCTTATGGGGACATACCAAGCAG

ATGACACGCCATAACGCAGACCCCCGGGACTTCAGCACAGGGGTTCTTTGAGAGCTAAAAGTACTTCAGCTCCTTTCATC
ATTTTCTGAGTTGTGCACTATTTGGTTGCTCTTATGTTGTTGTTGTTCAGTC
>OvmuTAS2R372AP_HG926251.1:348632-347761
ATGTCAAATGTCATCCAATATGTTTTTTGATCATTGAAATCTCAGAATTCATAACAGGAATTTGCGGAAATGGATTCAT
TGCACTAGTACTCTGTGCTGACTCTCTCAAAAATGAGAATATCTCCTTGCTTGACTTCATCTTCACATGCTTGGCCATCT
CCAGAATTGGTATGATATTCATACTTCTCCTGGATAGCATTAAAATTATGTTCCATCCAGAAATATTAGATCGTCACCAG
GTAATAGAAGTAACTTTTGATTTCCTCTGGAATCTGAGCAATTCCTTAGGTACCTGGTGTGCTGCCTGCCTCAGCGTCTT
CGACTTCCTCAAGCTATCTAGTTTTTCCCACCCCTTCTTTCTCTGGCTAAAATGGAGAAGAAATAGAGCTGTTTTCACCA
TTATGTTGGGATTCTGTCTCTCTTTGTTTTTAATCTTCTGAACATAAAATTCAATGCTCTCAGGGTCTGTGACCATTTA
GAGATAGAAAACAACTTGACTTGGAAAAAATGCATGCGTAAAACACAGTCTTATAGCAGTCAAATTCTCCTCCACCTGGG
ATCTCTCATCCCCTTGGCTGTGTCACTCATTTATTTTTCCTGTTAATATTTTCCTTATGGAGACATACCAGGCAGATGAC
ACGCTATGCCAAAGGATCCAAAGACCTCAACACAGGAGTTCTTGTGAGAACAAGAAATACGTTGGCCTCTTTCATCATTC
TCCTAGTTGTGCACTATTTGGCTACATTCATGTTAACTTGGTCCTATTTCACACTAGAAAATGACATGACTTTTGTTGCT
ATTCACACTGTAGCATTTCTCTATCCTTCAATTCACCCTTTTATTTTGATTCTGAGGAGCCGGAAACTGAGA
>OvmuTAS2R372BP_HG926251.1:380381-379514
ATGTCAAGTGTAATCAAAAACGTTTTTATAATCACTGAAATCTTAGAACTCATAACAGGAATTTGCAGAAATGAATTCAT
TGCCCTAGTACTCTGTGCTGACTCTCTCAAAAGCAAGAATATCTCCTTCTTTGACTTGATCTTAACATGGTTGGCCATCT
CCAGAACTGGCATGATATTCATAATTCTTGGGTGGTGTTAGAATAGTGATCTACCCAGGAATATTTGAAAGTCATCAGGT
AATAGAAGTAATTTTTTATTTCTTCTGGAATCTGAGCAACTCCTTAGGTACCTGATGTGCTGTCTGCCTCAGCGTCTTCT
ACTTCCTCAAGCTATCTAATTTTTCCCACCCTTTCTTTCTCTGGCTGAAGTGCAGAAGAAATAGAGTTGTTTTTACCATT
CTGCAGGGATTCTGTCTTTCCTTGATTTTAATTTTCTGAGCATAACTTTTCATTCATTTGGGGTCAGTGACCATTTAGAA
ATAGAAAACAACTTGCATTGGAAAAAATATGCATAAAATCCGGTCCTATAGCAGTCAAATTCTCCTCCACCTGGGATCTC
TCATCCCCTTGGCTGTGTCATTCATTTTATTTTTCTTGTTAATCTTTTCCTTATGGAGACATACCAGGCAGATGACAAAT
CATGCCAAAGGATCCAGAGACTTCAACACAGGGATTCTTGTGAGAGCCAGAAATACTTTAACTTCTTTCGTCATTTTCTT
AGTTGTGCACTATTTGGCTACATTCTTGTTAACCTGGTCCTGTTTCACACTAGAAAATGAAATGACTTTTATTGTTATTA
AGTCTGTAGCATTTCTCTATCCTTCAATTTACCGTTTTATTTTGATTCTAGGAAATGGAAAATGGAGA
>AimeTAS2R1_GL192612.1:1151157-1152053
ATGCTAGAGTTTTACCTTATTATCCATTTTCTGTTTTCAGTGATACAATTTTTCATCGGGGTTTTAGCAAATGGCATCAT
TGTGCTTGTGAATGGCGCCGAGTTGATCAAGCAGAGAAAGATGATTCCATTGGCTCTCCTTCTTTCCTGCCTTGCGATGT
CCAGGATTTGTCTGCAGTTGTCCGTCTTCTACATTAATTGGGCAATTGTCTCCTTGATTGAAGTCCCTCTACTTGTTGAG
AGCTTTTTAATTTTCATGTTTGTAAATGAATTGGGACTTTGGTTTGCCTCATGGCTCGGCGTTTTCTACTGCGCCAAGAT
TGCCCCCATAGCTCACCCACTCTTCCTCTGGTTGAAGATGAGGATATCGAAGTTGGTGCCCTGGCTGATCCTCGGGTCCC
TGCTATACACATCCGTCCCTTTTGTTTTCTACAGCAAACGTACATGGCTTCTCTCCCAACAAGTCCTGTTGGGCTTTTTC
TCCCCGAATGCAACAACTCAAATCAAAGAAACGTCTGCTATACAGGTTGCCTTTCTTATGAGGTTATTTTTGCCGTTACT
TATCTTCCTCGCTTCAGCCCTGCTCTTGATATTTTCCCTGGGGAGACACACGTGGCAGATGAGAAACACAGCGATGGGCA
CCAGCGTCCCTAGCACAGGCGTCCACGTGAGATCGCTTCTGTCCGTTCTGTCCTTCCTGGTCCTCTGTGTCTCCCACTAC
ATGACAGCTGCTTTGCTCTCTTCTCAGATTTTTAAACTCAGGAGCCTCATGTTTCTGTTCTGCATCTGGGTGTTTGGGTC
CTATCCCTCTGGACACTCTACGATCTTAATTTTAGGAAATCCTAAACTGAAACAAACTGCAAAGAAGCTCGTCCTCCACG
GAAAGTGCTGCCAGTGA
>AimeTAS2R2_GL193073.1:480785-481699
ATGGTCTCCTCTTTGTCAGCTATTCCTCATGTTATCGTTATGTCAGCAGAATTTATCACAGGGATTACAGTAAATGCATT
TCTTATCATCATCAACTGTAAAGAATTGATCAAAAGCAGAAAGCTAACACCAATGCAACTCCTTTTCATATGTATAGGGA
TGTCGAGATTTGGTCTGCTGATGGTGTTAATGGTACAAGGTTTTTTCTCTGTGTTCTTTCCACTCTTTTATAGGGTAAAA
ATTTATGGTGCAGCAATGCTGTTCTTTTGGATGTTTTTGAGCTCTCTCAGTCTCTGGTTTGCCACCTGCCTTTCTGTATT

TTACTGCCTCAAGATATCAGGCTTCACTCAGTCCTATTTTCTTTGGCTGAAATTCAGGATCTCAAAGTTAATGCTTTGGC
TGCTTCTGGGAAGCTTTCTGGCGTCCATGAGCACTGCAGCTCTGTGTGCTGAGGCAGATTACCCCCAAAACACGGACAGT
GATGATGGTCTCAAGAATGACACACTGAAGAGGACTGAAGCCAAGATAAGGGAAATTAATGAAGTGCTTCTTGTCAACTT
GGCATTACTATTTCCTCTAGCCATATTTGTGATGTGCACTTTTATGTTATTCATTTCTCTCTACAAGCACACTCATCGGA
TGCAAAATGGATTTCATGGTGTTAGAAATGTCAGCACAGAAGCCCATATAAATGCATTAAAAACAGTGATAACATTCTTT
TGCTTCTTTATTTCTTATTTTGCTGCCTTCATGGCAAATATGACATTCAGTATTCCTTATGGAAGTCATTTCTTCTTTCT
ACTAAAGGACATAATGGCCGCATTTCCCTCTGGCCATTCAATTATAATCATCTTGAGTAATTCTAAATTCCAACAACATT
TCAGGAAACTCCTCTGCCTCAAAAAGAATCGATGA
>AimeTAS2R3_GL192847.1:444166-443250
ATGTTCCTGGTTCTGTCTGCCACTGAGTTCATTCTGGGGATGCTGGGGAATGGTTTCATAGTGTTGGTCAATGGCAGCAG
CTGGTTCAAGAACAAGACAGTCTCTTTGTCTGACTTCATCATCACTAACCTGGCTCTCTCCAGGATCGTTCTGCTGTGGA
TTCTCTTGGTTGATGGTGTTTTAATGGTGTTCTCGTCCAAAGTACACGATGAAGGGATAGTGATGCAAATTATTGATATT
TTCTGGACATTTACAAACCACCTGAGCATTTGGCTCGCCACCTGTCTCAGTGTCTTCTACTGTCTGAAAATTGCCAGTTT
CTCCCATCCTACATTCCTCTGGCTCAAGTGGAGAGTTTCCAGGGTGGTCGTACACATGATTTTGGGTGCCCTGTTCTTAT
CGTGTGTCAGTGCCATGTCTCTGATCCAGGAATTTAAGATCTATTCTGTTCTCAGTGGGATCGATGGCACAGGGAATATG
ACCGGGCACTTTAGAAAGAAAAGAAATGAATACAAAGTGATCCATGTTCTTGGGACTCTGTGGAACCTCCCTCCGCTAAT
TGTGTCTCTGGCCGCCTACTTTCTGCTCATCCTGTCCCTGGGGAGACACATGCAGCAGCTGCAGCAAAGCGGTATCAGCT
CCAGAGATCCAAGCACTGAGGCCCACCAGAAAGCCATCAAAATCATCATATCTTTCTTCTTTCTCTTCCTGCTTTACTTT
CTGGCCTTTTTGATCACAACATCCAGTTATTTCATCCCAGGAACCGAGATGGTTAATATAATCGGAGAAGTAGTTACAAT
GTTTTATCCTGCTAGCCACTCATTCATTCTCATTCTGGGAAACAACAAGCTGAAGCAGACGTTTGTGGAGATGCTGTGGT
GTGAGCCTGGCCATCTGAAGCCTGGGTTCAAGGGACCTTTTGCCCCATAG
>AimeTAS2R4_GL192847.1:434322-433423
ATGCTTCAGATACTCTTTTTCTCTGCCATTATTGTCTCAGCAATTTTGAATTTTGCAGGACTCATTGTAAATCTGTTTAT
CGCAGTGGTCAATTATCAGACTTGGCTCAAAAGCCCCAGAATCTCCTCTTCTAATAGGATCCTCTTCAGCTTGGGCATCA
CCAGGTTTCTTATGCTGGGACTGTTTCTACTCAACATCATCTACTTCTTCATCTCCCCAAACGTGGAAAGGTCAGTGCAC
TTATCCACTTTTTTCCTGTTGTGTTGGATGTTTTTGGACTCTAATAGTCTCTGGCTTGTCACCTTGCTCAATGCCTTGTA
CTGCGTGAAGATTACGGACTTCCATCACGCAGTATTTCTCCTGCTGAAACGAAGTCTCTCCTTAAAGATCCCCAGGCTGC
TGCTAGCCTGTGTGCTGATTTCTGCCTTCACCACTCTCCTGTATGTTGTGCTCAGACAGACATCATGCTTTCCTGAATTT
GTGCCGGGGAGAAACGGTACAGGATGTGACATCAATGAGAGCGTCTTGTCTTTGGTGATCTCTTTGGTCTTGCGCTCATT
TCTCCAGTTCATCATTAATGTGACTTCTGCTTCCTTGTTAATACATTCCTTGAGGACACATATACAGAAGATGCAGAAAA
ACGCCACTATTTTTTGGAATCCCCAGACTGAAGCTCATGTGGGGGCTATGAAGCTCATGACCTGTTTCCTCATCCTGTAC
ATTCCTTATTCTGTTGCTACTCTGCTACATTATTTCCCTTTTGTTGGGATGGATTTGGGAGCCAAATCCATCTGCATGGT
TATTTCCACCATTTACCCTCCAGGACATTCTGTTCTCATTATTCTCACACATCCTAAACTGAAAACAAAAGCAAAGGAGA
TTCTTTGTTTCAACAAGTAG
>AimeTAS2R7_GL193194.1:840072-841010
ATGCCAGATAAAGTGGAGACCACCTTAATGATGATAGCAGCTGGAGAGTTTTCAGTGGGGATTTTAGGGAATGCATTCAT
TGGATTGGTAAACTGCATGGGCTGGATCAAGAACAGGAAGATCACCTCTATTGATTTAATCCTCACAAGTCTGGCCATAT
CCAGAATTTGTCTATTATGTATAATATTATTAGATTGTTTTATATTGGTGCTGTATTCAGATGTGTATACCACGGGTAAA
CAAATGAGAATCTTTGACTTCTTCTGGGCACTAACCAACCATTTAAGTGTCTGGTTTGCCACCTGTCTCAGCATTTTCTA
TTTCTTCAAGATCGCAAATTTTTTCCATCCCCTTTTCCTCTGGATGAAGTGGAGAATTGACAGTGTGATTCCGAGGATCC
TACTGGGGTGCTTGGTCCTCTCTGTGTTTATTAGCCTTCTTGTCACTGAGAATTTGAATGATGACTTCAGGTGTTGTGTT
AAGACAAAGGAGAAAACAAACCTAACTGTAAGATGCAGAGTAAATAAAGCTCAATATGCTTCCGTCAAGATTTTCCTCAA
CCTGTTAACGCTATTCCCTTTTTCTGTGTCCCTGATCTCATTTCTCCTTTTGATCCTCTCCCTCTGGAGACATACCAGGC
AGATGAAGCTCAATGCCACAGGGTGCAGAGACTTCAGCAGAGAAGCCCACATGGGAGCCAGGAAAGCTGTCATCTTGTTT

CTCCTCCTTTTCATTGCCTACTGTTTGGCCTTTCTCATAGCCACTTCTAGCTACTTTATGCCAGAGACTGAATTAGCTGT
GATCATTGGTGAGTTGATAGCTTTAATCTATCCCTCAAGCCATTCATTTATCCTAATTCTGGGGAGCAATAAATTAAGAC
AGGCATGCTTAAGGGTGCTTTGGAAAGTAAAGTATATCTTAAACAGAAAAAATTTCTAA
>AimeTAS2R8_GL193194.1:835298-836227
ATGCTCAGTATGGAAGACAACATCTTTGTGATCATTTTAACTGGAGAATTCATAATAGGAATGTTGGGGAATGTATATAT
TGGACTAGTAAACTGGATTGACTGGATTAAGAAGAAAAAGATTTCCTCAGTTGACTATATCCTCACCAGTCTAGCCATCT
CCAGAATTTGTTTGCTCTGTATATTGATACTAAATGGCATCATAATTGTATGCTACCCAGATTTTCATGAAAATGATAAA
CTACAGGCGGTCATTAATATCTTCTGGACACTCACCAACTACTTAAGTACGTGGTTTGCCACCTGCCTCAATGTCTTCTA
TTTGCTCAAGATAGCCAATTTCTCCCATCCGCTTTTTCTCTGGCTAAAGAGAAGAATTGACAGAGTGATTCACTGGGTTC
TGTTGGGTTGTTTGGCCATTTCCTCTTTGATCAGCCTTATACTAGCAACGGCACCAAATTATGATTTTGAATTTCAGAAA
ATTATAAATCATAAAAGAAACTGCACTGAAATGTCCCATGTGAGTAAAAGTCAATACTTCAGCCTGTTGACTCTCTTTAA
CCTGTTGGCAATTGTCCCATGTGCTGTGTCATTGATCTCATTTTTCCTTTTAATTGTGTCCCTAAGGAGACATATCAAGC
AAATGAAAGTCAGTGTTACAGGCTGTGGAGACCCCAGCACAGAGGCCCATGTGGGAGCCATGAAAACTATGACTTCATTT
CTCTTCCTCCTTTTTGTATACTATGGGGCTTCTCTTTTGGCGACTTTTCACTACCTTATGAAAGAAAGCAAGTTAGCTGT
GATGTTAGGAGAAATTATAGCAATTCTTTATCCTTCTGGTCATTCACTTATTTTAATTATTAGAAATAACAAGTTGAGGC
AGGCATCTATTAGGGTGCTGAGATTTGGAAGAACAGTCTGCATGATGTAA
>AimeTAS2R9_GL193194.1:832423-833418
ATGCTAAGTACAATGGAGGTAATATATATGATCTTGATTGCTGGTGAATTGACTATGGGAATTTGGGGAAATGGATTTAT
TGTACTGGTTAATTGCACTGGTTGGCTCAAAAGAAGAGATATCTCCGTGATTGACATCATCCTGGTGAGCTTAGCCATCT
CCAGAATCTGTTTGTTGTGTGTGATATCTTTAGATGGCTTTGTTGTATGTATCTCTGGAGATACATATGCTGGTAGCAAG
CTAATGAGCATTGTGGGTGTTTTCTGGACACTTAGCAATCATTCAAGTGTCTGGTTTACTTCTTGCCTCAGCATCTTCTA
TTTACTGAAGATAGCCAATATATCCCACCCATTTTTCATCTGGTTGAAACTAAAGATAAACAGAGTCGTCCTGGGAATTT
TTCTGATGTCCTTCCTTACCTGTGTAATTATTAGTGTTTCATTGAATGAGGACTTCTGGGATCCCTTCCAAGTTAGTCAT
AAGGAAAACATAACTTGGGAATTCAAAGTGAGTAAAATCCCAAATGGTTTCAAACTGGTTATCCTGAATCTAGGAGCTAT
CATTCCCTTTGTTCTTTGCCTAACTTCATTTCTCTTGTTACTTTTCTCCCTATTTAGACACAGCAGGCAGATGAAACTTT
ATGCCACCAGGTCCAGAGACCCCAGCACAGAGGCTCACATGAGGGCCATAAAGGCAGTGATCATCTTTCTGCTTCTCTTC
GTTATGTACTATGCAGTCTTTCTTGTAGTAACCTCTAGCTTACTAATTCCTCAGGGAAAATTAGTGGTGATGTTTGGTGG
CATGATAGCCGTCATTTTCCCATCAAGCCATTCGTTCATCTTGATAATGGGGAACAGCAAGCTGAGGGAGGCTTTTCTAA
AAGTGCTAAGGATTGTGAAGGGTTTCTGCAAAAGAAGGAAACCTTTTGTTCCACAGAGAATCCTGAATACAAGGAGAAAG
AAATCAACAAAAGACCCTCTCCCTTGTCCCAATTGA
>AimeTAS2R10_GL193194.1:824887-825858
ATGCTAAGCATACTGGAAGGCCTCTTCATTTTTATAGCAGTTAGTGAATCAATACTGGGCGTTTTAGGGAATGGATTTAT
TGGACTTGTCAACTGTATTGACTGTGTGAAGAACAAAAAGTTTTCTATGATTGGCTTTATTCTCACTGGTTTAGCTACTT
CCAGAATTTGTTTGATATTGATAATAATTACAGATGGACTTATAAAGATATTCTTTCCAGATATGTATTCCTCTGGTAAC
CTAATTGATTATATTAGTTACTTATGGGTAATTTTCAATCAATCAAGTATCTGGTTTGCCACCAGCCTCAGTGTCTTCTA
TTTCCTGAAGATAGCAAATTTTTCCCACCATATTTTTCTCTGGTTGAAGGGTAGAATCAATAGGGTTCTTCACCTTCTGA
TGGGATCCTTGTTTATTTCATGGTTATTTACTTTTCCACAAATTGTGGAGATTATTAATGAGAGTAGAATGAAGAGTGGA
AATGCAACTTGGAACCTCCACATGCAGAGAAGTAAATTCTTTACTAAGCAGATTTTGCTCAACCTAGGAGTCATTCTTCT
CTTTACACTGTGCCTGATTACATGTTTCTTGTTAATCATTTCCCTTTGGAGACACAACAGGCACATGCAACTGAATGTCA
CTGGACCCCGAGACCCCAGTACAGAAGCGCATGTGAAAGCAATGAAAGTTTTGATATCTTTTATCATCCTCTTTATCTTG
TATTTTATAGGCATTGCCATAGAAATATCATGTTTCACTCTGCCAGAAAACAAATTGCTGTTTATTTTTGGTATGGTGAC
CACAGTCATCTATCCCTGGGGTCACTCATTTATCCTAATTCTAGGAAACAGCAAGCTAAAGCAAGCCTCTTTGAGGGCCC
TGCAGCGATTCAAGCGCTGTGAGGTGGGGAGACTTCTCACAGCTGCACAGACGCGTGTGAGGAGCAATGGATGTTCTAGG
AGAATGATCTAG

>AimeTAS2R12_GL193194.1:804350-805285

ATGGCGAGCACATTGAAGAATGTACTTACGATGATTTTTGCTGGAGAATTCATAATGGGGCTTTTGGGAAATGGATTCAT
TGTATTGGTTAATTGTGTTGATTGGATCAGGAGCTGGAAGTTCTTCCTGATTGACTTTATTCTTACCTGCTTAGCTATTT
CCAGAATATTTCTGCTGTGCATGATAATTCTAGGCATAGGTGTAGATATAATTTGTGAGGAAATATGGTGCAATGATAAT
CAGCTGATGACTTTTGAAACCATCTGGACAGGATCCAATTATTTCTGCATCACCTGTACCGCCTGCCTCAGTGCCTTCTG
CTTCCTGAAGATAGCCAACTTTTCCAATCCCATTTTCTTCCGGATAAAACAGAGAATTCACGCACTGCTTCTCGTTATTG
TCCTGGGGGCGGTCTTCTCTTTCTGCTTGTCCCTGCTTTTTAAGGATAAAGTATTTAAGAACCTGATCGAAACCAGGGTA
CACACTGAAAGCAATCGGACATCGAATTTCACAGGGAGAAAATATGATTTATTAACTTCTAATATACTCCTGAACATAAT
GTTCGTCATCCCCTTTGGAGTGTCTCTGGCTTCCTTGGTCCTCTTGATCCATTCCTTATGGAACCACGCCAGACGGATGA
AGGGCGCAGGTTCCGGGGATCTTATCACAAAGGCCCATGTGAGAGCCATAAAGTCGATGATTTCATTCCTACTCTTCTTC
TTTGTGTACTATTTGAGCAACATTATAATATATTGGGCCTATGTCATTCTAGACAGTTTGGTGGCAAAAATATTTGCTAA
CATGTTAGCATTTTCCTATCCATCTGGCCATCCATTTCTTCTGATTTTATGGAACAGCAAATTGAAGCAGGCTTCCCTCT
GTGTCCTGAGGAAGTTGAGGTGGTGCAGGAACCTAAGAAAACCCGCAAACCCATAA

>AimeTAS2R408A_GL193194.1:768256-769186

ATGCTAACGTTACTATCTGGCCTTTTTTCCATCCTCGTAATAACAGAATTTGTTCTAGGGAATTTTGCCAATGGCTTCAT
AGCAGTGGTGAACTGCACTGACTGGGTCAAGAGACAAAAGATGTCCTCAGCTGATCGAATTCTGACTGCTCTGGCGATCT
CCAGAATCAGTTTGCTCTGGGCAATGTTAGTGAACTGGTATGCAGTCGTGTTGAATCCTGCTTTATACATCTTCAAAGTA
AGACTTCTTGTTCATGTTGCGTGGACGGCAAGCAATCATTTTAGCATCTGGCTTGCTACTAGCCTCAGCATATTTTATTT
GTTCAAAATAGCCAATTTCTCTAGCCTTATTTTTCTTCGCCTGAAGTGGAGAGTTAAAAGTGTAGTTGTTGTGATCCTGT
TGGAGTCTTTGTTCTTGTTGGTTTTTCAGATTGCAGTGGTAAGCATGTATGAGAAAATTCAGATGAAGGAATGTGAAGGA
AATGTCACTAGGCAGACCAAATTGGGGAGCATTTTACACCTTTCGAGTATGACTATATTCACACTAGCGAACTTTGTACC
CTTTGCTATATCCCTGACATCTTTTCTGCTGTTAATCTTTTCCCTGAGGAAGCTTCTCAAGAGGATGCAATCCAGTGGTA
AAAGATCCCAAGATCCCAGCACCAAGGTCCACATAAGAGCCATGCAGACTGTGATCTCCTTTCTCTTTTTATTAGCTGGA
CACTTCACGACTCTCATTATCACAGTCTGGAGTAGTAATGGGCTGCAGAATGAACTATTCTTCATGATTTGCCAAGCTTT
TGGATTTGTGTATCCTTCAAGCCACTCATTTATCCTGATCTGGGGAAACAAGAAGCTAAAACAGGCTTTTCTGTCTGTTT
TATACCAGGGGAAGTACTGGCTGAAAGAACAGAAACTCTCAACTCCATAG

>AimeTAS2R408B_GL193194.1:750728-751645

ATGCTAACATTACTATCGGGCCTTTTTTCCATCCTCATAATAACAGAATTTGTTCTAGGAAGTTTTGCCAATGGCTTCAT
AGCAGTGGTGAACTGCACTGACTGGGTCAAGAGACAAAAGATGTCCTCAGCTGATCGAATTCTGACTGCTCTGGCGATCT
CCAGAATCAGTTTGCTCTGGGTAATGTTAATGAATTGGTATGCAGCCATGTTGAATCCAGCTTCATTTAGCTTAGAAGCA
AGACTTCTTGTTCATGTTGCGTGGGCGGCAAGCAATCATTTTAGCATCTGGCTTGCTACTAGCCTCAGCATATTTTATTT
GTTCAAAATAGCCAATTTCTCTAGCCTTATTTTTCTTCGCCTGAAGTGGAGAGTTAAAAGTGTAGTTGTTGTGATCCTGT
TGGGGTCTTTGTTCTTTTTGGTTTTTCAGATTGCAGTGGTAAGCATGTATGAGAAAATACAGACGAAGGATTTCAAAGGA
AACGTCACTAGGCAGACCAAATTGGGGAGCATTTTACACCTTTCGAGTATGACTATATTCACACTAGCGAACTTTGTACC
CTTTGCTATATCCCTGACATCTTTTCTGCTGTTAATCTTTTCCCTGAGGAAACATCTCAAGAGGATGCAATCCAGTGGTA
GAAGATCGCAAGATCCCAGCACCAAGGTCCACATAAGAGCCATGCAGACTGTGATCTCCTTTCTCTTTGTATTAGTAGGT
TACTTCCCGGCTCTTATTGTCACCATCTGGAGCTCTAAGTGGTTGCCAAACAAGCTGATCCTCCTGCTTTGCAAGGCTAT
TGGAATCATGTATCCTTCAAGCCACTCATTTATCCTGATTTGGGGAAACAAGAAGCTGAGAAAGGCCCTTCTGTCATTCG
TGTGGCAGCTGAGATGCTGGCTGAAAGAAAGAATATAG

>AimeT2R38_GL192847.1:236458-237462

ATGTTGACTCTGACTCCTGTCATAACTGTGTCCTATGAAGTCAAGAGTGCATTTTTGTTCCTTTCGGCCCTGGAGTTTGC
AGTGGGGATCCTGACCAATGCCTTCATTTTCTTGGTGAATTTTTGGGATGTGGTGAGGAGGCAGCCACTGAGCAACTGTG
ACCTCATCCTTCTGAGTCTCAGCCTCACCCGGCTTTTCCTGCATGGGCTGCTGTTTCTGGACGCCCTCCAGCTTATCTAC
TTCCAGCAGATGAAAGACCCACTGAGCCTCAGCTACCAGATCATCATCATGCTCTGGATGATCACGAACCAAGCTGGGCT

CTGGCTCACCACCTGTCTCAGTCTCCTCTACTGCTCCAAGATTGTCCGTTTCTCTCACACCATCCTGCTTTGCTTGGCAA
GCTGGATCTCCAGGAAGGTCCCCCAGATGCTCCTGGGTGCCATGCTTTTCTCCTCCATCTGCACTCTCCTCTGTTTGGGG
GACTTTTTTAGTAGACCTGACTTCGCATTCACAACTATGCTATTCATTAATAATACAGAACTCAATTTGCAAATTACAGA
ACTCAATTTCTACCATTCCTTCATCTTCTGCATCCTGGGGTCCATCCCTCCTTTCTTGCTTTTTCTGGTTTCTTCTGGGG
TGCTGATTGTCTCTCTGGGGAGACACATGAGGACAATGAAGGTCAAAACCAAGGACTCCTGTGACCCCAGCCTGGAGGCC
CATATCAAAGCACTCAGATCACTCGTCTCCTTTTTCTGCCTCTATGTTGTGTCCTTCTGCGCTGCCCTCATTTCAGTGCC
TTTACTGATGTTGTGGCACAACAAGATCGGGGTAATGATCTGTGTAGGGATCCTAGCAGCTTGTCCCTCAATACATGCAG
CAATCCTGATCTCAAGCAATGCCAAGCTGAGGAGAGCTGTGGAGACCATTTTACTCTGGATTCAGAGCAGCCTAAAGGTA
AGGGCAGACCACAGGGCAGATCCCAGGACTCCAGATCTATGTTGA
>AimeTAS2R39_GL193114.1:514772-515737
ATGACTGAAACCTGCAATCCCCCAGAAAATGAATTGTCACCATTTCACATCCTCTCGATTTTAACAATTATAGGCACTGA
ATGCATCGTTGGTATCGTTGCAAATGGGTTCATTATGGCAATAAATGCAGCTGAATGGATTAAAAATAAGGCAGTTTCCA
CAAGTGGCAGAATCCTGTTTTTCTTGAGCGTATCCAGGATAGCTCTCCAAAGCTTCATGATGCTAGAGATTACCTTCAGC
TCAACATTCCCACGTTTTTATAATGAAGATCTTATAGATGAGATGTTCAAAGTAAGTTTCATGTTCTTAAATCATTGTAG
CCTCTGGTTTGCTACCTGGCTCAGTTTCTTCTACTTTGTGAAGATTGCTGATTTCTCCCACCCCCTTTTCCTCAAGCTGA
AGTGGAGAATTTCCAGATGGATGCCCCAGCTTCTGTGGCTTTCAGTGTTTATTTCTTTGGGCTACAGTGGGCTCTTCTCT
AAAGACATCTACACTGTGTATTGTAACAATTCTTCTATCCCCTCCTCCAACTCCACTAAGAAAAAATACTTCACTGAGAC
CAATATGGTCAACCTGGCTCTTCTCTATAACCTGGGGATCTTCATTCCTCTGACCATGTTCATCTTTGCAGCCACCCTGC
TGATTATCTCTCTCAAGAGACACACCCTACACATGGAAAGCAATGCCACTGGCTCCAGGGACCCCAGCATGGAGGCTCAC
ATGGGGGCCATCAAAGCTACCAGCTACTTTCTCATTCTCTACATTTTCAATGCAGTTGCTCTATTTCTCTATATGTCCAA
CATCTTTGATGTCAACAGCTCCTGGAATATTTTGTGCAGATTCATCATGGCTGCCTACCCTGCTGGACACTCCATTCTGC
TGATTCAGGACAACCCTGGGTTGAGAAGAGCTTGGAAGCGGCTTCAGCCTCAAGTTCATCTTTACCTAAAAGAACAGACT
CCATGA
>AimeTAS2R40_GL193114.1:540832-541788
ATGGCCACAGTGAGCACAGATGCCACGGATAGAGACACATCCAGGTTTAAAATCGTCCTCACCTTGGTGGTCTCTGGAGT
AGAGTGCCTCACTGGCATCGCTGGGAATGGCTTCATCACGGCCATCCATGGTGCCGAGTGGGCCAGAGGCAAAAGGCTCC
CTGTCAGTGACTGCATTCTGCTGATGCTCAGCTTTTCCAGGCTCTTGCTGCAGATCTGGGTGATGCTGGAGAATACTTAC
AGTCTACTGTTCCGGGTCACTTACAACCAAAACACAGTGTTCATACTCTTCAAAGTCATCGTCATGTTCCTGAACTATTT
CAGCCTTTGGCTTGCTGCCTGGCTCAACATCTTCTATTGTCTTCGAACTGCCAACTTTGCTCACCCTTTATTCTCCATGA
TGAAGAGGAAAATCACAGGGCTGATGCCTTGTCTTCTGGGACTGTCACTGCTCATCTCCTTATGCTTCAGCTTTCCCTTC
TCTACAGATGTCTTCAATGTGTATGTAAATAGTTCCACTCCCATCCCCTCCTCCAACACCATTGAGAAGAAGTACTTCTC
TGAGACCAATGTGGTCAACCTGGCTCTTCTCTATAACCTGGGGATCTTCATTCCTCTGACCATGTTCATCTTTGCAGCCA
CCCTGCTGATTATCTCTCTCAAGAGACACACCCTACACATGGAAAGCAATGCCACTGGCTCCAGGGACCCCAGCATGGAA
GCTCATGTGGGGGCCATCAAAGCTATCAGCTGCTTTCTCATTCTCTACATTTTCAATGCAGTTGCTTTATTTATTTCTAT
GTCCAACATCTTCGACATCAACAGTTCCGGGAATATTTTGTGCAAAATCATCATGGCCACTTACCCAGCTGGCCACTCAG
TGCTACTGATCTTGGGTAACCCTGGGCTGAGGAAAGCCTGGAAGAGGTTTCAGCACCATGTTCATCTTCACCTGTAA
>AimeTAS2R41_GL193114.1:742480-743406
ATGCAGCCAGCACTCTCAGCCTTCTTCATGCTGCTCTTTGTCCTGCTGTGCCTCCTGGGAATCCTGGCCAACGGCTTCAT
TGTGCTGGTGCTGAGCAGGGAAAGGATGCGGCGTGGGAGGCTGCTTCCCTCCGACATGATCCTCATGAGCTTGGGTGCCT
CCCGCTTCTGCCTGCAGTGTGTTGGAATGGTGAACAACTTCTACTGCTTCCTCCACCTGCACGAGTACAGCAGCGGTCCT
GCGCGGCAGTTCATCAGTCTCCACTGGGACTTCCTGAACTCGGCCACCTTCTGGTTGGGCTCTTGGCTCAGTGTCCTGTT
CTGCATGAAGATTGCTAACTTCACCCACCCTACCTTCCTTTGGCTGAAGTGGAGGTTCCCGGGGTCAGTGCCCTGGCTCC
TCATGGCTTCTCTCCTGATCTCTTTCATCGTCACCCTGCTCTTCTTCTGGGGAAACCATGCTGTGTATCAAGGATTCTTA
ATTAGAAAATTTCCTGGGAACATGACCTTCAAGCAGTGGAGCAGGAGGCTGGAAATTCACTATTTCTTGCCCCTGAAACT

TATCACCTTGTCAATTCCCTGCTCTGTCTTTCTGGTCTCAATTGCATTGTTGATTAATTCTCTGAGGCGACACACAGGGA
GAATGCAGCGCAGTGCCCACAGCCCACAGGACCCTAGTGGCCAGGCTCACACCAGAGCTCTGAAGTCGCTCATCTCCTTC
CTCATTCTTTATGCTCTGTCTTTCGCATCCTTGCTCATCGATGCTGCAGGTTTCTTCTCCTCAGAGAGTGACTGGTACTG
GCCGTGGCAGATTTTAATCTACCTGTGCACATCTGTCCATCCCTACATCCTCATCCTCAGCAACCTCCGGCTTCGAGGGG
TATGCAGGGAGTTACTTCTGTTGGCCAGGGTCTTCCGGCTGGCCTAG
>AimeTAS2R42_GL193194.1:698541-699515
ATGTTAGCTGGTTTTGATATAATCTTTCTTACGCTGTCGACAGCAGAATTCATAATTGGAATGTTGGGGAATGTGCTTAT
TGGACTGGTAAACTGCTCTGAATGGGTCAAGAACCGAAAGATCTCTTTAGCTGACCTCATCCTCATGTGCTTGGCTATCT
CCAGAATCACTCAGCTGTTGGTGTTACTGTTTGAATCATTTATGATGGGACTAAATCCACCTTTCTACTCCATTTACAAA
CTAGCAAAACCTGTTACTTTGCTTTGGAGAATAACTAATCATTTGACTATCTGGTTTACTACCTGCTTAAGTATTTTCTA
CCTCCTTAAGATAGCTCGGTTCTCCCATTCCCTTTTCCTCTGCCTGAGGCGGAGAATGAACAGAGTGGTTCTTGCAATTC
TTGTATTTTCTTTGTTGTTTCTACTGTTTGACTTTCTATTGCTAGAAGCATGGAATGATTTCTTCTTGAATATCCATGCA
ATGGATGAAAGTAATCTGACTTTATTTATGAATGAAGGTAAAACTTTCTATATTAAAAGCCTGATTCTTCTCAGTTTTTC
CTATATCGTTCCTATTGCTCTGTCCCTGACCTCATTGCTTCTTTTATTTCTGTCCTTGATAAAACACATCAGGGATCTGC
AGCTCAACTCCATGGGCTCCAGGGACTTCAGCACCCAGGCCCATAAAAAGGCCATTAAAATGGTGGTGTCTTTCCTCTTC
CTTTCCACAGTTCATTTTTTTTCCATACAATTGTCAAATTGGTTGCTTTTTTTATTTTGGAACAAGAAGAACACAAAGTT
TATTATGTTGACTGTATATGTCTTTCCCTCAGGCCACTCACTAATTTTGATTCTGGGAAACAGCAGGCTAAGACAGACAG
CCTTGAAGGTACTGTGGTATCTTAAAAGCTCCCTGAAAAAAGGAAAACCAATTTCATCTTTGCAGATAGACCTTCCAGAG
CCTTTCCAACGATGA
>AimeTAS2R5p_GL192847.1:421454-420587
ATGCTGACTGCTGCCCTAACACTGCTGATGGTGGTGGCAGTGGCCGAATTTTTCATTGGCCTGGTTGGAAATGGAGTCCT
TATGGTCTGGAGTTTTGGAGAATGGGTCAGAAAATTCAACGGGTCCTCATACAACCTCATTGTTTTGGGCCTAGCTGTCT
GCCGATTTCTCCTGCAGTGGCTGATTATGATGGACTTAAGCCTGTTTCTGCTTTTCTAGAGCAGTCATTGGCTTCGCTAT
CTCAGTGTCTTCTGGATCCTGGTAAGCCAGGCCAGCCTCTGGTTTGCCACTTTCCTCAGTGTCTTCTACTGCAGGAAGAT
CACGACCCTTGAACACCCTGTCTGCTTGTGGCTGAAGCAGAGGGCCTATTGCCTGAGTCTCTGGTGCCTTCTGGGGTACC
TCATGATCAGTTTGGTACTTGTAGCCCTATCATCTTTCCCAAGGCAACAGCAGCATTCTGTACCCCCTCAAAAGCTGTCA
CTACCTGTATATATTAAAGCTCAATGCAGGAAGTGGGTTGCCTCTGATGGTATTTCTTGTTTCTTCCGGGATGCTGATTT
TCTCTTTGTATAGACACCACAAGAAGATGAAGGTGCATACAGCTGGTAGGAGAGATGCTTGGGCCAAGGCTCACATCACT
GTCCTGAAGTCCTTGGGCTGCTTCCTTATCCTTCATGTGGTTTACATCCTGGCCAGCCCCTTTTCCATCATCTCCAAGTC
TTCTGCTGATCTCATTGTCTTCATCTCCGAGACAGTCATGGCTGCCTACCCTTCTCTTCATTCTGTCATATTGATCATGG
GGAATCCCAGGGTGAAGCATACTTGTCAGAGAATTCTGTGGAAGACAGTGTGTGCTTGGAGAGCCTAG
>AimeT2R16p_GL192642.1:856169-855268
ATGGTGCCCCTCCAACTCACTGTCTTCTTCATAATCCTCTGGGTGCTTGAATCGTTGACAGCAATTATGCAGAGCAACTT
AATTTTTACAGTGCTGGGCAGAGTGGGTGCCGGCCAGAAGGCTGTCATCATCGATGGACTTGATTCTCACCTGCCTGGGT
ATCTGCTGCTTCTGTCTACAGTGAGTGTCAGTGCGGAACAATTGTTGCTCCAATTTTAACCCTAACTATGTATTTTGGTA
TTTATTGGTCACTTGGGAGTTTACTAATACTCTTACTGGTTAACCAGCTTGCCTGCTGTCTTCCACTGTGTCAAAGTCTC
CTCCCTAACCCACTCCAGCTTCCTCTGGCTGAGGCGGAGAATTTTGAGGTTCGTTCCTTGGCTGTTGCTGGATTATCTGT
TGATTTCTTGTGTGTCAATCCCCTTCTCGGATGTTAGGAATTATATGAATGGGCACTTAGTCACCATGGGGCATTTCTCT
ACGAACAGCATTAGGATTGAGAGGCTTAAGATGTTTCACCTGTATTTTACCATCTCTTACGCAATGGTCACATTGGTTAT
TCCTTTCCTCCTGTTCCTGGCCTGCACCATCTTGGTCATGGCCTCCCTGTTCCAACCCGTGGAGCAGAAGAGCGCCACAG
CACTGGCCACTACAGCTCCAGCATGGAAACGCACGCCACCGCCCTGAGCTCTCTTGCCATCTTTCTCATTTCCTTCACCT
CTTACTTGCTGACCCTACTCATCTCTATTATGAACATCTCATTGGATAAGAGGTCCTGGTTCTGGGCCTGGGAAGCTGTC
ATCTATGCTATAGTCTCAATTCACTCCCGTTCACTAATGCTGAGCAGCCCTAAATTGAAAGAGGTTTTAAAGATAAGGTG
CTGGGGCCTAGAGGCTGCCTGG

>AimeTAS2R67p_GL193194.1:704077-705016

CAGAAATGCCGAGTCGAAAATGCTTTTCTGATAGCAGCAACAGGAGAATTCATAACTGGAATGTTGGGGAACAGTTTCAT
TGTACTAGTTAACTGCATTGACTGGGTGAAGAGTCAAAAGCTGTCATCAGCTGACTTCATCCTCACCAGCCTGGCTCTCT
CCAGAATCGTTCTTCTTTGTACAGTACTATTTGATTCATATTTTACGGTGTTTTGGGCACATCTTTATGCCACTGACAAA
ACTAGCAAAATTCATTAATATTTTTTGGACACTGAGCAATCATCTAGCTACCTGGTTTGCCACCTGTCTAAATGTTTTCT
ATTTCTTTAAAATAGCCAATTTCTCCCACCCCTGTTTCACCTGGCTGAGGTGGAGACTTAGCAGAGTGCTACTTGTACTT
CCACTGGGGTCTTTATTCTTATTGTTTTGCAACTTTGAATTATCAGATATATTTACTAATTTCTGGGTTAATGTCTATCG
AGGATATAAAAGAAACTCAACTTGGTCTCTAGATGTAAGTAAAATGCTGTATTTTAACAGCTTGATTGTTTTCAGTTTCA
TCTACTTAATCCCCTTTCTTCTGTCGCTGGCCTCACTGCTCCTTTTATTTCTTTCCCTGATGAGACACACCAGGAATATG
CAACTGAACTCCGGCTCTAGGGACTTCAGCACAGAGGCCCACAAAAGGGCCATGAAAATGGTGATGTCTTTCCTTCTCCT
CTCCACGGTTCATTTTTCTTTCCTCCTAATAACAGGTTGGACTTTCCTTTTACTGCATAATTCTCAGGTCAGTTTTGTTG
TCATATTATTATCGACTCTTTTTCCTTCGGGCCACTCATTTATTCTGATTTTGGGAAACAGCAAGTTGAGAAAAACTGCT
TTAGGGTTACTGTGGCATCTTAATCGCCACCTGAAAATGGTGAAACCTTTAGCTTCACAG

>AimeT2R60P_GL193114.1:721339-722511

CTGGATGGAGATGACATGGTTCCAGAACCCAGTGACTGACTGATAAGAGAGCCATCACCCTGGTTATCATCTTATTTTAT
TTTTTTTGTTTCTGGGGACGGCAGTGAGCAATGGCTTCATCAATGTAGCACTGAGCATGGAGCGGTTGCTACAGAGAACA
CTGTTAACCTTGTGATAAATTATTAGTCAGCCAGGGCCTCTTGCCTCCATCTGCAGTGGGTGGTGATGAGGAAGAGCATT
TATATTTTCCTGTATCCAGTGGCCTTCCCATACAACCCTGTACTGCAGTTCCTAGCCTTCCAATGGAACTTACGGAACAC
TGCCACCTTATGGTTCTTCACTTAGCTCAGTGCTTTCTATTGGGTGAAGACCGCAACCCTCCCCCTCCCCATCTTCCTTG
GGCTAAAACAGAAGGTGTCTGGGTTGGTTCCATGGATGCTGCTCGGTTCTGTGGGGCTCTCCAGCTTGAGCACCATCCTA
TTTTCATAGGCAACCAGAGCTTATATCTCTACTTTTTTTTTTTTTTTAAAGATTTTTTTATTTATTTATTTGACAGAGA
GAGACAGCCAGCGAGGGAGGTAACACAAGCAGGGGGAGTGGGAGAGGAAGAAGCAGGCTCATAGCAGAGGAGCCTGATGT
GGGGCTCGATCCCATAACGCTGGGATCACGCCCTGAGCCGAAGGCAGATGCTTAACGACTGCACCACCCAGGCACCCCTA
TATCTCTACATTTTAAGGAGAGGGTTGCAATCTTGGAATGCCACTGGGAATACTATATATAAGATCATATGAGAAATCCT
ACTTCTTCTCTTTAAAACTTGTTACCTGTACACTCTTTACTGTTGTCTTCCTTGTTAGCATGGTTTTGCTCATGTCTCTG
TAAAGACACCCTAAGAAGGCCCTGCTGTCCATCGCAGCTTTCGTGACCCCAGTGTCCAGGCGCACATCAAGGTTCTTTTG
GCTCTCATCTCCTTTGCTATTGTCTTCACCCCCTATTTCTGTCACTGGTGCTCAGCGCTGCAGGTGTTTTTCCATCTTG
GAACTTGGCGCTGGGTGTGGCAGGCAGTGATTTATCTGTGCACAGCCATCCACCCTGTCACTGCTCTTGAGCAACCCCAG
GCTGAGAGGTGTGCTGGAGGGGGGCTGCTGTGCACAGTGCTGGGCATCTTGA

>AimeTas2r62P_GL193114.1:715911-716775

ATGCCTTCCTCACCTGCATTGATCTTCATGGTCATCTTCTTCCTGGAGTCGTTGGCTGCAATGTTGCAGAATGGCTTCGT
GGTTACTGTGTTGGGCATGGAGTGGGTGCGACGCCGGATGCTGCCTGCAGGTGACATGATTGTGGCCTCTCTGGCTGCCT
CCCGGTTCTGCCTACATGGGGTGGCCATCCTGAACAACCTCTTGACCTTCTTTGACCTACCAGACCCCCTGGAACTTCAT
CAACACTCTCACTTCCTGGCTCACTGCCTGGCTTGCCATCTTCTACTGTGTGAAGATCGCCCTCTTCTCCCACCCTGTCT
TCTTCTGGCTGAAGTGGAGGATTTCTCGGTCAGTGCCCAGGCTGCTGCTGGGCTCCCTGGTCTTGGCTGGTCTGACAGTC
ATCTCATCAGCCATTGGGACTAGAATTTTTATGCAGATGATTGCATCCCAGAGTTCCCAAGGAAACAGCACCCTGGCTGA
TACAGTACAGTCCTTCTATTGGTGTTTTACTGTACCTCATGCAATGCTTACATTGTCAATCCCATTCCTCCTGTTCTTGG
TGTCCACGTTCTTGCTCATGTTCTCACTGTGCCAGCACTTGAGGCGGATGAGGGACCATAGACTGAGCCCATGTGATCCT
AGTATCCAGGCTCACACCAGGGCCCTGAAGTCACTTGTCTTCTTCCTTGTCTTCTATACATCATATTTCCTGTCTCTGAT
TGTTGTTGTTAGGAAAATCACAATCTTCCAGAGTCACTGGTACTGGGCCTGGGAAGTGGTAACTTATGCAGGTGTCTGTC
TGCACTCCAGCATCCTGGTGGTAAGCAGCCCCAAGCTGAGAAAGGTCCTGAAGACCAGGGTTTGG

>AimeTas2r42P

ATGTGAGTTGGAATGAAGATCTCTTTCTGGTAGTGGCCCCAGGAGAAGTCATCTTAGGAATGCAGGAAAATGGGTTCATT
GGACTGGCAAACTGCAGTGAATGGGTCAAGAACGGGAAAGTCTCATCAACTGATTTCATTCTTACCTGCTTGGCTATGGT

CAGAATCACTCAACAGTGGGTGACACTATTTGATTCATTTCTGGTGGGGCTGTCTCCACATCCCTATGCCATTGGTGAAC
AAATAGCAAAAGCGGTTACTATTCTTTGGGCACTAATCACTGAACTCCCTGGTTTGCCACCTGCCTAACATTTTCTGCTT
CTTGAAGGTAGCCAAGTTCTCCCACTTCTTTTTCATCCGGCGGAGGTGGAGAGAGAACAGAATGGGTCTCGTGCTTTTCC
TGGGGCCTTTGTTCTTATAGTCTGTTAACTTCTGAATGTAGGATGCCCCTGGTGAGTTGTGGATGAGTACCTATGGGGCA
CATGAGAGACTCTGCATTTAGACGTAAGTGTTTGGTCTGAAAAGGCTTCGACTTCCTAGCTTCACCTATGTTAAGCTTTC
TCCCCTTTCTCCTGCACCTGACCTGTTTGCTCCTTTTATTTCTGTTCTTGGTGAGACACCAAGAACCTACAGCTCAACCG
GATGGGCTTGAGGGACTCCCGCAGAGGCCCATACAAGGGCCATGAAAGTGGTGATGACCTTTCTCCTCCTCACCGCCATT
TACTTATTTCCACTCCAGTAGCAAGTTGGATCTTCCTTAGGGTACAGAGATAACAATCAGGCCAAGATGCTTGTCATGGT
GACTTCAACTTCCTTTCACTCAGACCACTCTCTCGTTTTGGGAAACAGCATGCTAGGAGGGATACCTAACACTACTGTGG
CATCTGAAATTCTGCCTGAGAAAATGAATACATTTAGCTTCATAGATAGAATTGAAAGAAGTTTCTGTATTCTAA
>AimeTAS2R408CT_GL235647.1:189-1
AAGCAATCATTTTAGCATCTGGCTTGCTACTAGCCTCAGCATATTTTATTTGTTCAAAATAGCCAATTTCTCTAGCCTTA
TTTTTCTTCGCCTGAAGTGGAGAGTTAAAAGTGTAGTTGTTGTGATCCTGTTGGAGTCTTTGTTCTTGTTGGTTTTTCAG
ATTGCAGTGGTAAGCATGTATGAGAAAAT
>SuscTAS2R3_GL892960.2:34965-35915
ATGTCGGGATTCACCGAGTGGGGCTTTCTCGTTCTGACTGTCACTGAGTTCATTCTGGGAATGCTGGGGAATGGTTTCAT
AGGGTTGGTCAGTGGCAGCAGCTGGTTCAAGAACAAGAGAATCTCTTTGTCTGACTTCATCCTCACTAACTTGGCCCTTT
CCAGGATTGTTCTGCTGTGCATTCTCTTGGTTGATGGTGTTTTAATGGTGTTCTCTCCCAAACTACATGATGAGGGGATA
GTAATGCAAATTATTGATATTTCCTGGACATTTACAAACCATCTGAGCATTTGGCTTGCCACGTGTCTCAGTGTCTTCTA
CTGCCTGAAAGTCGCCAGTTTCTCCCATCCTACATTCCTCTGGCTCAAATGGAGAGTTTCCAGGGTGGTTGTATGGATGC
TGTTGAGTACCCTGCTCTTATCATGTGGCAGTGCCATCTCTCTGATCCGTGAATTTAAGATCTATTTTGCTCTCAGTGGA
ATCGGTGGATCAGGCAACGTGACTGAGCTCGTTAGAAAGAAAAGAGATGAGTATAAACTGATCCATGTTCTTGGGACTCT
GTGGAACCTCCCTCCCCTGATGGTGTCTCTAGCCTCCTACTTTCTGCTCATCCTCTCCCTGGGGAGGCACACGCGGCGGA
TGCAGCAAAACTCTACCGGCTCCGGAGACCCAAGTCCTGAGGCCCACAGGAGGGCCATCAAAATCATCCTCTCCTTCCTC
TTTCTCTTCCTACTTTACTTTCTTTCCTTTTCAATTCTGTCATCCAGTTATTTTCTGCCAGCAACTAAGACGATTATGAG
GATTGCAGAAGTGATTACAATGTTATACCCTGCTGGCCACTCATATGTCCTCATTCTGGGAAACAACAAGCTGAAGCAGA
TGTTTGTGGAGATGCTCTGCTGTGAGCCTGGTCATCTGAAGCCTGGATCCAAGGGGCCCTTTTCTCCATAG
>SuscTAS2R4_GL892960.2:41686-42576
ATGCTTCGGATACTCTTTTTTTCTTCCGTTATTATCTCAGCAATTTTGACGTTTGTAGGACTCATTGTGAATCTGTTCAT
TGCAGTGGTCAATTGTAAGACTTGGGTCAAGAGCCACAGAATCTCCTCTTCTGATAGAATCTTGTTCAGCTTGAGCATCA
CCAGATTTTTTATATTGGGACTGAATACGCTTTTATTCATCATTCCAAATACTGCAAGGTCAGTCTATTTTTCCACGCTT
TTTCTGACCTGTTGGATGTTTTTGGACTCGAACAGTCTTTGGTTTGTAACCTTGCTGAATGCCTTGTACTGTGTGAAGAT
TGCCAACTGCCAACACTCCATGTTTCTCCTGCTGAAACGAAATCTTTCCCCCAAGATGCCCAGGCTGCTGCTGGTCTGTG
TGCTGATTTCTGCCGTCACCACTCTCCTGTACGTGGTGCTCAGACAGGTAGCACCCCTACCTGAATCCGTGAGTGGAAGA
AATGGCACAGTATTCGATATCAACGAGGGCATCTTGTCCTTGCTGACGCCTTTGGTCTTGAGCTCCCTTCTCCAGTTCAT
CCTCAATGTGACTGCTGCTTCCTTGGTCATCAACTCCTTGAGGAGGCACGTACAGAGGATGCACAGAAATGCCACTGTGC
TTTGGAACCCCCAGACTGAAGCTCACCTGGGTGCTATGAAGCTGATGATCTATTTCCTCATACTCTACATTCCGTACTCA
GTTGTTACCCTGCTCCATTATCTCCCTTCTTCTGTGGCAGTGGATTTGGGAGCCAAGTCCATTTATATTATTATTTCCAC
CTTTTACCCTCCAGGACATTCTGTTCTCATTCTTCTCACACATCCTAAACTGAAAACAAAAGCAAAGAAGATTCTTTGTT
GCAATAAATAG
>SuscTAS2R7_chr5:63985142-63986080
ATGTCAGGTAAAGTGGAGGACATTTTAATGCTCTTAGCAGCTGGGGAATTTTCACTGGGGATTTTAGGGAATGCATTCAT
TGGATTGGTAAACTTCATGGACTGGATCAAGAATAGAAAGATCTGTTCCATTGATTTAATCCTCACAAGTCTGGCCATAT
CCAGAATCTCTCTATTATGTATAATACTATTAGACTGTCTAATATTGGTGCTGCATCCAGATGTCTATGCTGCTGGTAAA

CAAATGAGAATTATCGACTACTTATGGACACTAACCAACCATTTAATTGTCTGGTTTGCCACCTGCCTCAGCATTTTCTA
TTTCCTGAAGATAGCTAATTTCATCCATCCCCTCTTCCTCTGGATGAGGTGGAGAATTGACAGTGCAATTCCAAGGATCC
TGCTAGGGTGTTTGGCCCTCTCTGTGTTTATTAGCCTTCCTGTCACTAGGAATTTGGATGATGATTTCAGACGTTGTGTC
AAGATAAAGGTGAAAATGAACTTGACTTTGAGGTGCAGAGTAAATAAAGCTCAATATGCTTCCATCAAGATATATCTCAA
CCTGTTGACACTGCTCCCCTTTTCTGTGTCCCTGATCTCATTTCTGCTCTTGATTCTCTCCCTGTGGAGACACACCAGGC
GGATGCAATTCAATGCCACAGGGTACAGAGACGCCAGCATCGAAGCCCATGTGAAAGCCATGAGGGCTGTCACCTCCTTC
CTCCTCCTTTTCATTGCCTACTACTTGGCCTATCTTGTAGCCACCTCCAGCTACTTTATGCCAGAGACTGAATTAGCTGT
GATAGTTGGGGAGTTGATAGCTCTAATCTGTCCCTCAATCCATTCAGTTATCCTAATTCTAGAAAACAAGAAATTAAGGA
AAGCATGCCTAAAGGTTCTATGGAAAGTAAAGTATATCCTACAAAGAAGGAATTGCTAA
>SuscTAS2R9_chr5:63976739-63977674
ATGCCAAGTGCAATGGAGACAATATATATTATCTTCATAACTGGTGAATTGACTGTGGGAATTTGGGGAAATGGATTCAT
TGTACTGGTTAACTGCACTGACTGGCTCAAAAAGAGAGGTATCTCCTTGATTGACATCATCTTGGTCAGCTTAGCTGTCT
CCAGAATCTGCTTGTTGTCTGTGATGTTTTCAGATGGCTTTGTTATGGTATTCTATCCAGATAGCTATATTAATGGTGAT
GTAATGGGCATCCTTGATAGTCTCTGGACACTTACCAATCATTCAAGTGTCTGGTTTACTTCTTGCCTCAGCATCTTCTA
TTTACTCAAGATAGCCAGTATATCCCACCCATTTTTCCTCTGGCTGAAGCTAAAGATAAACAAGGTCGTCCTTGGGATTC
TTCTTGTGTCCTTTCTCATGTCCTTAATTATTAGTATTTCATTGAATGATGAGTCCTGGTATGATTTCAAGGTCAGTAAT
AAAGACAACGTTACTTGGGAAGACAAAATGAGTAAAATCTCACATGCTTTCAAACAGATTATCTTGAACCTGGCAGCTTT
CATTCCCTTTATTCTGTGCCTGATCTCATTTCTCTTGTTACTTTTCTCCCTATTTAGACACACCAAGCAGATGAAACTTT
ATGCCACAGGGTCCAGAGACCCTAGCACAGAGGCCCACTTGAGGGCCATAAAGGCAGTAATCATTTTTCTCGTCCTTTTC
ATTATGTACTATGCAGTCTTTCTTGTAGTAACCGCTAGCTTTCTGATCCCTCATGGAAAATTAGCGGTGATGTTTGGTGG
CCTAATAACCCTCATTTTCCCAACAAGCCATTCATTCATCCTGATAATGGGGAACAGCAAGCTGAGGGAGGCTTTTCTGA
AGCTGCTGAGGATTGTGAAATGTTTCCACAAAAGAAGGAGACATCTTGTTCCATAG
>SuscTAS2R10_chr5:63965446-63966375
ATGCTAAGTATAGTAGAAAGCCTCCTCATTTTTATATCAGTTAGTCAGTCAATATTGGGGTTTTTAGGGAATGGATTTAT
TGGACTTGTAACCTGCATTGACTGTGTGAAAAACAAGAACATCTCTATGATCAGCTTTATTCTCACTGGCTTAGCTACTT
CAAGAATTTGTCTGATATGGTTAATAATTATAGATGGATTTATAAAGATATTCTTTCCAGATTTATATATTTCTGGTAAA
CTAACTGAATACATTAGTTACTCATGGGTAATTGTCAATCATTCAAGTATCTGGTTTGCCACCAGCCTCAGCATCTTCTA
TTTCCTGAAGATAGCAAATTTTTCCCACCACATTTTTCTTTGGTTGAAGGGTAAAATCAATAGGGTTCTTCTCATTCTGA
TGGGATACTTGTTTATTTCATGGTTATTTACTTTTCCACAAGTTGTGAAGATTATTAGTGACAGTAAAAAGAAGAATGGA
AGTTCATTCTGGCCACTCAACATGCATAAACTTGAATACTTTATGAGCCAGTTTTTGCTCAATCTGGGTGTCATTCTCCT
CTTTATACTATGCATGATTACATGTTTCTTATTGATCATTTCTCTTTGGAGGCACAACAGGCAGATGCAATCGAATGCCA
CTGGATTCAGAGACCCCAGCACAGAAGCACATATTAAAGCAATGAAAATTGTGATATCTTTTATCATCCTCTTTATCTTG
TATTTTATAGGCGTTGCCATAGAAATATCATGTGGTACTCAGCCAGAAAACAAACTGCTGTTTATTTTTGGTATGATAAC
CACAGCCATTTTTCCTTGGGGTCACTCATTTATCCTAATTCTAGGAAACAAGAAGCTAAAGCAAGCCTCTTTGAAGGTAC
TGAAGCAATTAAAGTGCTGGGGAAAAGAGAAACTTCTCAGAACTCCATGA
>SuscTAS2R11_chr5:63950624-63951541
ATGTTGAATGTGTTGGAGAAAGTTTTCATGACTGTAACAGGTGGGGAACTTATAATTGGAATTTTAGGGAATGGATTCAT
TGGACTCACAACTTTCATTGCTTGGATTAGAAATCAGAAGTTATGCTTGGTTGACTTTATTCTCACTAGTTTGGCCTTTG
CCAGAATCAGTCAACTGTGGCTAACAGTTGCCAATTTGTTTTCAGTGCTACTCTATCAAGAAGGCTTTGGTACTGTGGAA
AGAAATTACGTCCTTGCTAGTAGCTTGATACTGGCCAACCACTTGAGCATTTGGTTAGCTACTTGCCTTGTTGTCTTTTA
TTTCCTGAAGATCGCCAGTTTCTCCAATCTCCCTTTCCTCTGGCTAAAACGGAGAATCAAAAAAGTAGTTTTCATACTTC
TGCTATTATCTGTGCCGTTCCTGTTCATGAGCTTTCCTTTCCCATATAGTTTTGATGGTTTCTGGTATCATACCCAAAAC
ATTCTTGAGAGAAATATAACTGAGTTATACAACGTGACCCAAAGTCAAAAATTAAAATTTATGCTTATTTTTACAGCTGG
GTCCATCCCTCCCTTCTCTCTTTCCCTGATTTCCTTTTTCCTATTGCTCTGTTCTTTGTGGAAACACAAGAAGCACATTG

AACTTAGTGTCAGTGATTCCAGAGACGCCAGCATGGAGGCCCATTTCAGAGCCATGAGAACTGTGTTTTTCTTTCTTGTG
TTCTTTGCCCTGTACCACCTTGCTTTTTTCATGACATTTGGGGGGCATTTTTTGCTGCAGAACAAGTTGGTTACGATGTT
TGGTTATATGTTAGGAATTCTCTATCCTTCGGGTCATTCATATGTTATAATTTTTGGAAACAGCCAAATGAGGAAAGCCT
TCTTGGGGATTCTTTGGCATTGGAAGTGTGGGGCCTGA
>SuscTAS2R12_chr5:63940163-63941095
ATGGGAAGCACATTGGAGAATATACTTATGATCATTTATGCTGTAATATTCCTAGTGGGGATTTTGGGAAATGGATTCAT
TTTACTGGTTAACTGTATTGATTGGATCAAGAACAGGAGGTTCTCCCTGATGGATTGTATTCTCACCTGCTTGGCTATTT
CCAGAATATTTATGCTGTGCATAATAATTTTGAGTATAGGCTTACATGTAATCTCTGTGACAATATGGTGCAATAATGAT
CTACTGATAAGTTTGGAAAACCTCTGGATAGGATCCAATTATTTCTGCACAGTCTGCACCACCTGCCTCAGTGTCTTCTA
TTTCCTCAAAATAGCCAACTTTTCTAATTTGCTTTTCCTCTGGATGAAATGGAGAATTCACAAAGTGCTTCTCATTATTG
TTCTGGGGGCAGCCCTCTCCTTTGGCTTGAGCCTTCTTTTTAAGGATACGGTAGTAAAGATCCTGCTCAAAAATCAAATA
GACGCTGAGAACAATGTGACATTGTATTTTTCAGAAAGAAAACATATATTAACCTCACATTTGTTCCTTGGCACAATGTT
CATCATCCCCTTTGTAGTGTCCCTGACCTCCTTTGTTCTTTTAATCCTCTCCTTATGGAGTCACCTCAGGCACATGAAGA
GCACCGTGTCCAGGGATACTAGCACAGAGGCCCATGTGAGAGCCATGAAGTCTATGATCTCATTCCTTCTCCTCTTCATT
CTGTACTATGCAAGCAATATTTTGTTAATGTGGGGCTATGGCAGGCAAGACAATTTTGTGGTAAAAATTTTTTCAAATGT
GCTATTATTCTTCTATCCATCTGGCCATCCATTTCTTATGATTTTATGGAACAGCAAATTGAAACAGGCTTCTCTCCGTA
TCTTGAGGAAACTGAACTGTTCCATGAATCCAAGAAAACCTACACTTCCGTAA
>SuscTAS2R16_chr18:25883452-25884354
ATGATCCCCATCCAACTTTCTGTCTTCTTCATGATCGTCTATGTGCTCGAGTGCTTGGTAATAATTGTGCAGAGCAGCTT
AACTGTTGTCTTGCTGGGCAGAGAGTGGGTGCAAGTCAAGAGGCTGTCTCCTGTGGACGTGATTCTCACCAGCCTGGGCA
TCTGCCGCTTCTGTCAACTGTGGTCATCCATGCTGTTCAATTTTTTCTCCCACTTCCATCCTCACTGTGTATTTTGGTAC
TTCGGGATCGTCTGGGAGTTTACTAACACGCTCTCGTTCTGGCTAACCAGCTGGCTTGCCGTCCTCTACTGCGTCAAAGT
CTCCTCCTTCAGCTGCCCCATCTTCCTCTGGCTGAAGTGGAGAATTGTGAGGTTGTTTCCATGGCTATTGCTGGGTTCTG
TGCTGATTTCGTGTGTGTCAATGATCTTTTCAGCTATTAGGAATCACATCAAGATTCAGTTAAACTCCATGAGGCATTTC
TCTAGAAACAGCACCGTGACGGAGAGGCTTGAGACATTTCTGCAGCAGTATGCCATATATCAAGGGATTATGTTGGCTGT
TCCTTTCCTCCTGTTCCTGGCCTCCACCATCTTGCTCATGGCCTCATTGTCCCAGCACGTGAGGCAGATGAAACAACATC
ACACCAGCCACTCCAACCTTAGCCTGAAAGCTCCCGCTACTGCCCTGAGGTCGCTGGCCATCTTCCTCATCTTCTTCACC
TCTTATTTTCTGACCACAATCATCTCCATCATGGGTGCCCTAGTTAATAAAAAATCCTGGTTCTGGGTCTGGGAAGCTGT
CATCTATGGTATAGTCTCTATTCATTCTACTTCACTGATGCTGAGCAGCCCTAAATTGAAAAGGGTTTTCAAGGTAAGAT
GCTGGGGCTTAGAGGCTGCCTGA
>SuscTAS2R38_chr18:8357518-8358525
ATGGTGCCTCTGACTCCTGTCATAACCGTTTCCTATGAAATGAAGAACGCATTTCTGTTCTTTTCGGTCCTGGAGTTTGC
AGTAGGAATCCTAGTCAATGCCTTCATTGTCTCGGTGAATTTTTGGGACGTGGTGAGGAGGCAGCCACTGAGCAACTGTG
ACCTTGTCCTGCTAAGCCTCAGCCTCACGCGGCTGTTCCTGCACGGGGTGCTCTTTCTGGATGCCGTCCAGCTCACCCAC
TTCCAGAGGATGAAGGACCCACTGAGTCTCAGCTACCAAACCACCATCATGCTCTGGATGATCATAAATCAAGCTGGCCT
GTGGTTCACCACCTGCCTTAGTCTCTTCTACTGCTCCAAGATTGTCCGTTTCTCTCGCACCTTCCTGCTCTGCTTGGCCA
GCTGGATCTCCAGGAAGACACCCCGGATGCTCTTGGGTGCCGTTCTTTTCTCCTGCCTCTGCACTGTTTTTTGCTTATGG
GACTTTTTTAGTAGCTCTCACTTCTCAGTCACAACTAGGTTATCAATGAATAACAATACGGAGCTCAATTTGAAAACTGC
AAACCTCCGTTTCTCTCGTTCCTTCCTCTTCTGCAGCCTGGGGTCCATCCCTCCTTTCTTGCTTTTTCTGGTTTCTTCCG
GGCTGCTGATTGCCTCCCTGGCAAGGCACATGACGACAATGAGGGCCCAAACCAGGGACTCTCGGGATCCCAGCCTGGAG
GCCCATTTTAAAGCGCTCAGAACTCTCGTCTCCTTTTTCTGCCTGTACATGGTGTCCTTCTGTGCCGCCGTAATCTCGGT
GCCTTTACTGATGCTCTGGCACAACAAGATCGGGGTAATGGTCTGTGTAGGGATACTGGCAGCCTGTCCCTCGGGACACG
CAGTCATCCTGATTGCCGGCAATGCCAAGCTGAGGAGAGCAGTGGAGACCCTTCTCCTCTGGGCTCAGAGAAGCCTAAGG
CTAAGGGTAGACCGCAAGGCAGATCCCAGGACACCAGATCTAAGCTGA

>SuscTAS2R39_chr18:7358812-7359855
ATGAATTGTGGGAGCTATCCCAGATCTGCCCATCAAATGCTAGGGAGACTTTTTTCTCCAGACAACAAAGAAGAGAAACG
ACTCAGGATGATCAAAACCAGCAGTCCCCCAGAAAATGAATTATCATCTCACATCATTTTAATTTTAATCATTGTAAGCG
CTGAATGCATCATTGGTATCATTGCAAATGGGTTCATTGCAGCTATAAATACAGCGGAATGGATTCAGAATAAGGAAATT
TCCACAAATGGCAAGATCCTGCTTTTCCTGAGTGTGTCCAGAATAGCTCTACAAAGCTTCATGATGCTAGAAATTAACTT
CAGTTCAACATCCCCAAGCTTTTATAACCAAGACACTGTGTATGATACATTCAAAGGAGGTTTCATGTTCTTCAATTACT
GTGGCCTCTGGTTTGCTGCCTGGCTCGGCTTCTTCTACTTTGTGAAGATTGCTGATTTCTCCCACCCCCTTTTCCTTAAG
CTGAAGTGGAGAATTTCTGGATTGATGCCCTGGTTTCTGTGGTTGTCAGTGTTTATTTCCTTGGGCCATAGTGGACTCTT
CCTCAAAAACGTCTACACTGTGCATTGTAACAATTCTTCTGCTATCCCCTCCTTCAACTCCACGAAGAAAAATTACTTCA
CTGAAACCAATGTGACCAACCTGGTTCTTGTCTTTAACACGGGGATCATCATCCCTCTGAGCATGTTCATCCTCGCAGCC
ACTCTGCTGATCATCTCTCTCAAGAGACACACCCAGCACATGGAGAGCAATACCACTGGCTCCAGGGACCCCAGCATGGA
GGCTCACATGGGGGCCATCAAAGCCATCAGCTACTTTCTCATTCTCTACATTTTCAATGCTGTCGCTCTTCTTCTCTATA
TGTCCAACGTCTTTGACATCAACAGTTTCTGGAATGTTTTATGCAAAGTCATCCTGGCTACCTACCCTGCTGGCCACTCC
ATCCTACTGATTCGGGACAACCCTGGGTTGAGAAGAGCTTGGAAGCGGCTTCAGGCTCGAGTTCATCTTTATTTAAAAGA
GTAG
>SuscTAS2R41_chr18:7019729-7018806
ATGCACCTAGCCTTCACAGTCCTCTTCGTGCTGCTCTTTGTCCTGCTGTGCGTCCTGGGATTCCTGGCCAATGGTTTCAT
TGTGCTGGCGCTCAGCCGGGAATGGGCGCGCCACGGGCGGCTGCTCCCCTCGGACCTGATCCTCATCAGCCTGGGTGCCT
CCCGCTTCTGCCTGCAGTGGGTTGGCATGGGGAACAACTTCTACTACTTCTTCCACTTGGTCGAGTACTGCAGGGGGCCC
GCCCGGCAGTACTTTGGGCTCCCCTGGGACTTCCTGAACTCGGTCACCTTCTGGTTTGGCTCCTGGCTCAGCGTCCTCTT
CTGCGTGAAGATCGCTAACTTCTCCCACCCCGCTTTCCTTTGGCTGAAGTGGAGGTTCCCAGGGTCCGTGCCCTGGCTTC
TGCTGGGCTCTCTGCTCATCTCCTTCATCGTGGCCCTGCTGTTCTTTTGGGGGAACCACGCTTTGTACAAAGGATACTTG
ATTAGAAGGTATTCTGGGAACATGACCTGCAAGCAGTGGAGCAGGAGGCTGGAAACGCACTATTTCCTGCCCCTGAAACT
GATCACCCTCTCAATTCCTTGCTCTGTTTTTCTGGTCTCCATTGCTCTGTTGATTAATTCTCTGAGGAGACACTCGTGGA
GGATGCAGCGCAGTGCCCACACCCCACAGGACCCCAGCGCCCAGGCACACATCAGAGCCCTGAAGTCACTCATCTCCTTC
CTGGTGCTTTATGCTCTGTCCTTCGTGTCCCTGGTCATCGATGCTGCGGGGATCTTCTCCGAGAGCGACTGGTACTGGCC
GTGGCAAATTTTAGTCTACTTGTGCACATCCGTCCATCCCTTTATCCTCATCCTCAGCAACCTCAGGCTTCGAGGGGTGT
TTGGGCAGCTAACTCTGTTGGCCAGGGGCTTCCGGGTGGCCTAG
>SuscTAS2R42_chr5:63867091-63868041
ATGTTCCCTATGTTGAATACAATCTTTCTGATACTGTCAGTAGTGGAATTCATAATTGGAGCGTTGGGCAATGCATTCAT
TGGACTGGTAAACTGCTCTGAATGTATCAAGAATCAAAAGATCTCTTTAGTTAACTTCATCCTCACCAGCTTGGCTATCT
CCAATGTCAGTCAACTGTTGGTGGCTTTGTTGGACTCATTTATAATGGGACTACCTCCATGTTTATTGCTCACTAACAAA
CTAGCAAAACCTATTACTTTGCTTTGGAGAATAACTAATCACTTGATTACCTGGCTTTCCACCTGCTTAAGCATTTTCTA
CTTCCTTAAGATAGCTCACTTCTCCCACTGCCTTTTCCTCTGGCTGAAGCGGAGAATGAACAAAGTCATTCCTGGGATAC
TTGTATTTTCTTCGGTCTTTCTGGTTTTTGACTTTCTACTGCTAGAAACATTTAATGATCTTTTCCAGAATATAGCATCT
GAAAGTAATCTGACTTTAGATAAAAATCTCTATTTTAAAAGCCTGAGTCTCCTTAGCTTGACCTTTTTCTTTCCTGTTAT
TCTGTCCCTGATGTCATTGCTTTTTTTGTTTCTGTCCTTGGTGAAACACACAAGAAATTTGAAGCTTAACTTTGTGTGCT
CGAGGGACCTCAGCACACAGGCCCATAAAACAGCCATGAAAATGGTGATGTCAGTCTTCCTCCTCATCATGGTTCATTTT
TTTTCCGTACAATTGGTAAATTGGTTGTTATTTGTGTACTCTGACAACGAGTTCATAAAGTTCCTCATGTTAGCAGTATA
TGTCTTTCCCTCCGGCCACTCATTTATTTTGATTCTGGGAAGCAACAAACTAAGACAGAGAGCCTTGAAGGTACTGAGGC
ATCAAAAAGCTTTGAAAAGAGAAAATCCATTGCCTTTACAGATACGCTTTCAAGAGTCTTTTCAAAGATAA
>SuscTAS2R408A_chr5:63904140-63905054
ATGAAAACTTTAACACTGAGTTTTTCCATCATAGTAATAACAGAGTTTGTCCTCGGAAATTTTGCCAATGGTTTCATAGC
ACTGGTGAACTGTATTGACTGGGCCAAGAGAAAAAAGATCTCTTCAGCTGATGGAATTCTCACTGCTCTGGTGGTCTCCA

GAATTGGTTTGCTCTGGATTATATTAATAAATTGGTATTTAACTGTGCTTAATCCAGCTTTATGTAGTTTAAAAGTGAGA
ATTATTGTTCATATTGCCTGGGCAATAAGCAACCATTATAACATCTGGCTTGCTACTAGCCTCAGCATATTTTATTTGTT
CAAGATAGCCAATTTCTCCAGCCTAGTTTTTCTTCATCTAAAGTGGAGAGTTAAGCATGTACTTTTCATGATACTTCTGG
GAGCTTCGTTCTTCTTGGCTTTTCAAGTTGCAGTGGTGAGCTTAAAAGACAGTATCCAGAGAAATGAATATGGAGGAAAC
ATCACTCAGAAAACCAAATTGAGGGACATTTTACAGCTTTCACATGTGACTCTGATCACTCTAGCAAATCTCATACCCTT
TACTATGTCCTTGATATCTTTTCTGCTGCTAATCTTTTCCCTGTGGAAACATCTCAAGAAGATGAAGTGCAATGGCAAAG
GATCCCAAAATCCCAGCACCAAGGTCCATATAAAAGCCATGCAAACTGTGATCTCCTTTCTTTTGCTACTTGCCATTTAC
TTCCTGACTTTAATTAGTTCTGTTTGGAGTTCCAAGAGACAGCAGAATGAACAGGTCCTCGTGCTTTTGCAGGCCTTTGG
AATCCTCCAGCCTTCAGTCCACTCATTTATACTGATTTGGGCAAACAGGAAGTTAACAAAAGCCTTTCTGCCCTTTCTGT
GGCCACTGAGGTGCTGGCTGAAAGAAAGGAAATAA
>SuscTAS2R408B_JH118443.1:205120-204206
ATGAAAACTTTAACACTGAGTTTTTCCATCATAGTAATAACAGAGTTTGTCCTCGGAAATTTTGCCAATGGTTTCATAGC
ACTGGTGAACTGTATTGACTGGGCCAAGAGAAAAAAGATCTCTTCAGCTGATGGAATTCTCACTGCTCTGGTGGTCTCCA
GAATTGGTTTGCTCTGGATTATATTAATAAATTGGTATTTAACTGTGCTTAATCCAGCTTTATGTAGTTTAAAAGTGAGA
ATTATTGTTCATATTGCCTGGGCAATAAGCAACCATTATAACATCTGGCTTGCTACTAGCCTCAGCATATTTTATTTGTT
CAAGATAGCCAATTTCTCCAGCCTAGTTTTTCTTCATCTAAAGTGGAGAGTTAAGCATGTACTTTTCATGATACTTCTGG
GAGCTTCGTTCTTCTTGGCTTTTCAAGTTGCAGTGGTGAGCTTAAAAGACAGTATCCAGAGAAATGAATATGGAGGAAAC
ATCACTCAGAAAACCAAATTGAGGGACATTTTACAGCTTTCACATGTGACTCTGATCACTCTAGCAAATCTCATACCCTT
TACTATGTCCTTGATATCTTTTCTGCTGCTAATCTTTTCCCTGTGGAAACATCTCAAGAAGATGAAGTGCAATGGCAAAG
GATCCCAAAATCCCAGCACCAAGGTCCATATAAAAGCCATGCAAACTGTGATCTCCTTTCTTTTGCTACTTGCCATTTAC
TTCCTGACTTTAATTAGTTCTGTTTGGAGTTCCAAGAGACAGCAGAATGAACAGGTCCTCGTGCTTTTGCAGGCCTTTGG
AATCCTCCAGCCTTCAGTCCACTCATTTATACTGATTTGGGCAAACAGGAAGTTAACAAAAGCCTTTCTGCCCTTTCTGT
GGCCACTGAGGTGCTGGCTGAAAGAAAGGAAATAA
>SuscTAS2R60_chr18:7046597-7045653
ATGAGCGCAGAGGGTGTGGCTCCAGGACTTCAGGTGGCTGATAAGAGAGCCACCATCTTCATTATCATCTTGCTCCTTTT
GTGCCTGGTGGCATTGGTGGGTAACGGCTTAATCACTGCAGCGCTGGGCATGGAGTGGTTGCTGCGTAGAACACTGTCAC
CCTGCAATAAGTTACTGGTCAGCCTGGGGGCCTCTCGCTTCTGTCTTCAATGGGTGATGATGAGCAAGAACCTTTATATT
TTCCTGCATCCGATGGCCTTCCCGTACGACCCGGTATTCCAGTTCCTAGCCTTTCAGTGGGACTTCTTGAATGCTGCCAC
GCTCTGGTTCTCCACCTGGCTCAGCGTCTTCTACTGTGTGAAAATCGCCACCTTCACCCATCCTGCCTTCCTCTGGCTGA
AACGGAATGTGTCGGGGTTGGTCCCATGGATGCTGCTCAGCTCTGTGGGTTTCTCCAGCCTCAGCACCATTCTTTTCTTC
ATAGGCAACCACAGAATATATGAGAACTATTTAAAGAGGGGTCTGCCATCCTGGAATGTCACTGGAAACGCTCTGAGGAG
ATCCTATGAGAGATTCTACTTCTTCCCTTTGAAAATTGCTACCTGGACAGTCCCCAGCAGTGTCTTCGTCGCCAGCATGG
TGCTGCTCCTTACATCGCTGGGGAGACACACCAAGAAGGTCTTCCTGTCCATCTCAGGCCTTCGTGATACCAGCGCCCAG
GCACACGTCAAGGCTCTCCTGGCTCTCGTCTCCTTTGCCGTCCTCTTCATCTCCTACTTTCTGTCACTGGTGCTCAATGC
GGTGGGTGTGTTTCCATCCCAGGAGCTCAGGTACTGGATTGGGCAAACTCTGATTTATCTGTGCACAGCCGTCTACCCCA
TCGTTCTGCTCTTGAGTAACCGCAGGCTGAGAGCTGTGCTAGCGAGGGGCTGCTCCCCCGTATAG
>SuscTAS2R62A_chr18:5877487-5876579
ATGCTGGTCTTCCTGGTCGTCTTTGTCCTGGAGTCGCTGGCTGCGCTGCTGCAGAATGGCTTCATAGTTGCCGTGCTGGG
CAGGGAGTGGGGGCGACGCCGGACGCTGCCCGCAGGCGACATGATCGTGGCCGGCCTGGCCGCCTCGCGGTTCTGCCTGC
ACGGGATGGCGCTGCTGAACAACCTCTTGACCTTCTTTGGGCTCTCTTTCAAAATATATTTCAACACCCCCTGGGACTTC
CTCAACACCCTCACCTTCTGGCTGACCGCCTGGCTCGCTGTGTTCTACTGCATGAAGATCGCCTTCTTCTCCCACCCCGC
CTTCCTCTGGCTGAAGTGGAGGGTTTCCTGCTCGGTGCCCAGGATGCTGCTGGGCTCCCTGCTCCTGTCTGGCGTGAGCA
CCGTCGCGTTCACCGCCGAGCATGCAGCGCAGCTGAGGCCCGCCCGGCGTTCCCATGGCAACGACACCCTGGCCGACAGG
TGGCAGAGCAGCTCTTGGTACTTTTTCTGGCTCGGGTCGTTCTCCTGTGGTGCGTCCCGTTCCTCCTGTTCCTGGCGTC

CACCCTCCTGCTCATGTTCTCGCTGCAGCGGCACCTGGGCCAGATGAGGGACCGCAGTGCTGGCCTGCGTGATGCCAGCA
CCCAGGCTCACACCATGGCCCTGAAGTCGCTGGCCTTCTTCCTCGTCTTCCACACGTCGTATTTTCTGTTCCTGATTGTC
GTGATTCGGAACAGCGTGGCCTCCCAGAACCGCTGGCGCTGGGCCTGGGAAGTGGTGACTTACACGGGCATGTGTCTCCA
CTCCAGCATCCTGGTGCAGAGCAGCCCCAAGCTGAGAAAGGCCCTAAAGGAGAGGCTTCGGAGAGCCCCGGGCAAGGAGC
TGTCCGGCTCGAGGTATCAGAGTCAGTAG
>SuscTAS2R2P_chr9:90211641-90212556
GTGGCCTCCTCTTTGTCGACTATTCTTCATGTTATCATCATGTCAGCAGAATTTATTACAGGATTACAGTAAATGAATTT
CTTATAATCATCAACTATAATGAATTGGTCAAAAGCAGAAAGCTAACACCAGTGCAACTCCTTTTCATATGTATAGGGAT
ATCTAGACTTGATTTGCAGATCCTGTTAATGGTACAAAATTTTTTTCTCTATGTTCTTTCCACTCTTTTATAGAGTAAA
AATTCATGGTAGAGCAATGATTTTTTTTTTTTGATGTTTTTTAGCTCTGTCAGTCTCTGGTTTTGCCACTTGTCTCTCTG
TATTTTACTGCCTCAAAATAACAGGCTTCAACCAGTCCTATTTTCTTTGGCTGAAACTCAGGATCTCAAAGTTAATACCT
TGGATGCTTCTGGGAAGCCTGCTGATCTCTGCGAGCACTGCAGCTCTGTATGTCAAGGTGAACTACCCTAAAAACATGGC
TATTAATGTCCCCAGGAATGGCACACTACAGAGGACTAAGCTAAAAACAAAGCACATTAATGAAGCACTTCTTGTCAACT
TGGTATTACTATTTGCTCTGACCATATTTGTATCGTGCACTGATTTGTTATTCATTTCTCTCTATAAGCACACTCTTTGG
ATACAAAATGAATCACTTGGTTTTAGAAATGCCAGGACAGAAGCCCATATTAATACATTAAGAACAGTTATAATATCCTT
TTGCTTCTTTGTTTCTTATTTTGCCACCTTCATGGCAAATATCATGTTCACTATTCCTTATGGAAGTCAATGTTTCTTTG
TGTTGAAGGACATAATGGCAGCATAGCCCTCTGGTGTTCTGGTTAAAATGATCTTGAGCCATTCTAAGTTCCAACAGCCA
TTCAGGAGAATTTTCTGCCTCAAAAAGAATCAATGA
>SuscTAS2R8P_chr5:63979853-63980778
ATGCTCAGTACAGAAGGCAACATCTTTATGATCATGATGATTGGAGAATTCATAATAGGAATATTTGCAAATGGATACAT
TGGACTAGTAGTATGGATTGACTGGATTAAGAAGAAAAAAACCTCCCCAGTTGACTGCATCCTCGCCAGTTTGGCAATCT
CCAGAATGTGTTTGCTCTGTATAACGATACCAAATGGCATCATACTGGTACTCTGCCCAGAGGTTTATCTAAACAATAAA
ACAAAGGGAGTCATTAGGATCTTCCAGACACTCATCACTATTTAAGTATGCGGTTTTCCACCTGCCTCAGTGTCTTCTAT
CTCTTCAAGACAGCCAATTTCTCCCACCCACTTTTGCCCTGGTTTAAGTGGAGAATCAGCAGGGTGGTTCGTTGGAGCCA
GCTGGGGTCTTTGGCCATTTCCTCACTGATCAGCCTTATGCTTGCAATGTTACCAAATCATGATCATGAATGTCTTAAAA
CTGCAGAACATAAAAGAAACTTCACTGAATTTTTCCATGTGAGTAAAATTCAATACTTCACCCTGTTGACATTCTTTAAC
CTGTTGGCAATTGTTCCATTTACTGTGTCATTGATCTCACTTTTCCTTTTAATTATGTCCCTTTGGAAACATACTACACA
AATGAAATCCCATGTTACAGGTTGAAGAGACTCTAGCACAAAGGCCCTCATGAGGGCCATTAAAACTGTGACTTCATTTC
TCTTCCTCCTTTTTGTTTACTACCTGGCCTGTCTTTCGGCAATATTTAGCTACCTTATGAAAGAAAGCAAGTTAGCTATG
ATATCTGGAGAGATTATGGCAAGTCTCTATTCCTTAGGTAATTCATTTTTTTAATCGTTGGAAATAATAAGCTGAGGCCA
GCATTTGCCAGGATGCTGAGATGTGGAAAAAGCCAGCATGATGTAA
>SuscTAS2R40P_chr18:7267764-7266810
ATGGCGATAGTGAACAGAGATGCTATGGCTGAAGACACCACCGGGTTTAAAATCGTCTTCACCTTAGTGGTCTCCGGAAT
AGAGTGCATCACTGCCATTCCTGGGAACAGCTTCATCACAGCCATCCGTGGAGCCAAGGAGGCCAGAGACAAAATCCTCC
CTGTTGGTGACTGCAATTCTGTTGATGCTGAGTTTTTCCAGGCTCTTGCTACAGCTTTGGATCATGCTAGAAAATACCTA
CATTATACTATTCTGGGTCACTTATAACCGAAAAACCATGTATACGCTTTTTAAAACCGTCACCATGTTTCTAAACTATG
CCAACCTCTGGCTTGCTGCCTGGCTCAGTATCTTCTATTGTCTTAGAATTGCAAACTTTACTCACCCTTTGATCGCTGCG
ATGAAGGGGATAATGCCGGTGCTGTTGCCTTGGCTCTTGAGGCTGTCGTTGTTCTCCTTCTGCTCTAGTTTTCCCTTCTC
TACAGATATCGTCAATGTGCACGTAAAGAACTCCATCCCTAGCCCCTCCTCCAACTCCACTGAGAAGCTGTACTTCTCTG
GGACCAACGTGGTCAACTTGATTCTCACGCTTTACCTGGGTATCTTCATCCCTCTGAGCATGTTCATCCTTGCAGCCACC
CTGCTGATGGTCTCTCTCAAGAGACACCCCCAATGGATGGAGAGCAATGCCACTGGCTTCAGGGGCCCCAGCATGGAGGC
TCACATGAGGGCCATCAAAGCCATCAGCTGCTTCCTCAGTTTTTACACTTTCAGCACAGTTGCTCTATTTCTTTCCATGT
GCAGCATCTTCAATGCCAACAGTTCTTGGAATATTCTGTGTGAAATCATCACGGCTGCCTGCCTAGCTGGCCACTCAGTG
CTATTGATCCTGGGCAACCCTGGGCTGAAAGGAGCCTGGACACGGTCTCAGCACCAAGTTCATCTTTACCTGTAA

>SuscTAS2R62BP_chr18:7054537-7053620
ATGCTCCCCTTACCCACGCTGATCTTCATGGTCGTCTTTGTCCTGCAGTCACTGGCTGCAATGGCTACTGAATGACTTCA
AAGTTACTGTGCTGAGCTGGGAGTGGGGGCGAGGCTGGACATTGCCCGCGGGTGCCATGATTGTGGCCAGCCTGGCCGCC
GCCTGGTTCTGTCTGCATGGGAAGTCCCTCCTGAACAACCTCCTGGCTTCCTTTGACTCTTGTTCCAAAGATTATTTCAA
CCTCGCCCGGGATTTCCTCAGCACTCGCTCTTGCGGGCTGACTGCATTGCTCGCTCTTCTCTGCTCTGGGAAGACCTCCC
GTTCCCACTCCGTCTTCCTCTGCTGAAAAGGAGGCGTTCTCCATCAGCACCCAGGCTGCTGCTGGGCTCCCCGATTGTAT
CGGGACTGACCCTTCCTCCCATCGGCTATAGAGAACGCAAGTCTTTTGCAGATGATGGCTGCCCAGAACTCCCACGACAG
CGGCACCCTGGCTGATGGACTTCAGCCCATTGGTACTTTATCCCACCTCGGGTAGTTCTCCTGGGGTGGAGTCCACTCTT
CCTGCTCCTGGCGTCCACTCTCTTGCTCATGTTCTTGCTGGACTGGCACTTGGGGCAGATGAGGGACCCTAGACCCCGAT
GGGGGACCTCATGATCCTAGCACTCCACCCAGGCGCGTACCGTGGCCCCAAAGTCCCTTGCCTTCTTCCGCATCTCTACA
CATCACATTTCTTGTCCCCGATAATTGTTGCTCTGAATGGCATATGGCTCTGGCACCACTGGCCCTGGGCCTGGGAAGTG
GTGACCCACGCAGGCATCTGTCTCCACTCCAGGATCTTGGGGCAAAGCAGCCCCAAGCTGAGAAGGGCCCTAAAGAAGAG
GCCTTGGTGAGCTCTGGACAAAGAGTCAGTATCAATAA
>SuscTAS2R372P_chr5:63956499-63956974
CCATTCTGTTGGGGTTCTTTCTCTCTTTGTTTTTAATCTTCTGAGCATAAACTTTGACACTTTTGAGGTCAGTGACCAT
TTAGAAACAGAAAGGAACTTGACTTGGAAGAAACATACACATAAAAATCAGTATTATGGCATTCGGATTCTCCTCAACAT
GGGATCTCTCATCCCCTTGGTTGTGTCACTTATTTCATTTTTCCGGTTCATCTTTTCTTTATGGTGACATACCAGGCAAA
TGACATGTCATGCTGAAGGATCTGGAGACCTCAACGCAGTGGTTCTTTTGAGAGCTAGAAATACTTTGAATTATTTCATC
ATTTTCTTAGCTGTCCACTCTTGGGCTACTCTCAAGTTAATATGATCTTATTACAGATTAGACAGTGTAACGACTTTTAT
TATTATTGAGAATGTAGCATTTCTCTATCCTTCAATTCACCCTTTTATTTTGATTCTGGGGAACCAGAAATTGAGA
>SuscTAS2R1T_GL893464.1:29033-28052
ATGCTGGCGTCTCACCACACTGGCTACCTTCTGTTGGCAGTGATACAGTTTCTCATCGGGGTTTTAGTCAATGGTACCAT
TGTGGTTGTGAACGGCATTGACCTGATCCAACGGAGAAAGCTGAACCCGCTGGCTCTCCTTATCTCCTGCCTGGCGATTT
CCAGGATTGGTCTTCAGTTTGTCCTTTTTTGCGCTACCCTGGCTGGTCTTTCCTTGATCGGATTCCTCCAACTTGCTGAG
AGGCCTGCAATTTTCCTCTTTGTCAATGAATCGGGACTTTGGTTTGCCACATGGCTGAGTGTTTTCTACTGTGCCAAGAT
TGCCACCATCCCTCACCCAATCTTCTCATGGTTGAAGATGAGGATATCCAGGTTGGTCCCTTGGCTGATTCTGGGGTCCC
TGGTATATGCATTTAGCATATCTGTTTTCCAGAGCAAACGTAAATGGATATTTTCCAAAGAAGACCTCCTGGCCCTTCTG
TCCCCAAATGCAACAAATCCCTTCAAAGAAATGCCTCCTTTAAAGTTGGCCTTTCTTTCCATTGAGCTTGGCTTGCCATT
ACTTATCTTCCTTATTTCTGTTGTGCTCTTAATATATTCCCTGGGGAGACACACGCAGCAGATGAGAAACACAGCGATGN
NNNNNTCCCGTGTGCACGTCAGCGCCCTTCTCTCCATCCTGGTCTTCCTGGGTCTCTACGTCTGCCACTACACGGCAGCA
GTTTTGTTCTTTTTTCAAATTTTCAAACTTTCAAGCCTCAGATATTTCTTCTGCCTCTTGGTGATTGGTTCCTACCACAC
TGGACACTCTGTTAACTTAATTTTAGGAAATCCTAAACTGAAGCAGATTGGGAAGAAGTTGCTCCTGCACAGGAAGTGCT
GTCAGTGA
>SuscTAS2R4BT_chr18:8191729-8191933
GATCTATTTCCTCATACTCTACATTCCGTACTCAGTTGTTACCCTGCTCCATTATCTCCCTTCTTCTGTGGCAGTGGATT
TGGGAGCCAAGTCCATTTATATTATTATTTCCACCTTTTACCCTCCAGGACATTCTGTTCTCATTCTTCTCACACATCCT
AAACTGAAAACAAAAGCAAAGAAGATTCTTTGTTGCAATAAATAG
>UrmaTAS2R1_NW_007907110.1:8687358-8688254
ATGCTGGAGTTTTACCTTATTATCCATTTTCTGTTTTCAGTGATACAATTTTTCATCGGGGTTTTAGCAAACAGCATCAT
TGTGCTTGTGAATGGCGCTGAGTTGATCAAGCAGAGAAAGATGATTCCATTGGCTCTCCTTCTTTCCTGCCTCGCGATGT
CCAGGATTTGTCTGCAGTTGTCCGTCTTCTACATTAATTGGGCAATTGTCTCCTTGATTGAAGTCCCTCTACTTGTTGAG
AATTTTTTAATTTTCATGTTTGTAAATGAATTGGGACTTTGGTTTGCCTCGTGGCTCGGCGTTTTCTACTGCGCCAAGAT
TGCCCCCATAGCTCACCCACTCTTCCTCTGGTTGAAGATGAGGATATCGAAGTTGGTGCCCTGGCTGATCCTCGGGTCCC
TGCTATACACATCCGTCCCTTTTGTTTTCTACAGCGAACGTACATGGCTTCTCTCCCAACAAGTCCTGTTGGGCTTTTTC

TACCCAAATGCAACAACTCAAATCAAAGAAACATCTGCTATACAAGTTGCCTTTTTTATGAGGTTATTTTTGCCGTTACT
TATCTTCCTCGCTTCAGCCCTGCTCTTGATATTTTCCCTGGGGAGACACGCGTGGCAGATGAGAAACACAGCGATGGGCA
CCAGCGTCCCTAGCACAGGTGTCCACATGAGATCGCTTCTGTCCGTTCTGTCCTTCCTGGTCCTCTGTGTCTCCCACTAC
ATGACAGCTGCTTTGCTCTCTTCTCAGATTTTTAAACCCAGGAGCCTCATGTTTCTGTTCTGCATCTTGGTGTTTGGGTC
CTATCCCTCTGGACACTCTACGATCTTAATTTTAGGAAATCCTAAACTGAAACAAATTGCAAAGAAGCTCGTCCTCCACG
GAAATTGCTGCCAGTGA
>UrmaTAS2R2_NW_007907068.1:3837087-3838001
ATGGTCTCCTCTTTGTCAGCTATTCCTCATGTTATCGTTATGTCAGCAGAATTTATCACAGGGATTACAGTAAATGGATT
TCTTATCATCATCAACTGTAAAGAATTGATCAAAAGCAGAAAGCTAACACCAATGCAACTCCTTTTCATATGTATAGGGA
TGTCGAGATTTGGTCTGCTGATGGTGTTAATGGTACAAGGTTTTTTCTCTGTGTTCTTTCCACTCTTTTATAGGGTAAAA
ATTTATGGTGCAGCAATGTTGTTCTTTTGGATGTTTTTGAGCTCTCTCAGTCTGTGGTTTGCCACCTGCCTTTCTGTATT
TTACTGCCTCAAGATATCAGGCTTCACTCAGTCCTATTTTCTTTGGCTGAAATTCAGGATCTCAAAGTTAATGCTTTGGC
TGCTTCTGGGAAGCTTTCTGGCCTCCATGAGCACTGCAGCTCTGTGTGTTGAGGCAGATTACCCCCAAAACACGGACAGT
GATGATGGTCTCAAGAATGACACACTGAAGAGGACTGAAGCCAAGATAAGGCAAATTAATGAAGTGCTTCTTGTCAACTT
GGCATTACTATTTCCTCTAGCCATATTTGTGATGTGCACTTTTATGTTATTCATTTCTCTCTACAAGCACACTCATCGGA
TGCAAAATGGATTTCATGGTGTTAGAAATGCCAGCACAGAAGCCCATATAAATGCGTTAAAAACAGTGATAACATTCTTT
TGCTTCTTTATTTCTTATTTTGCTGCCTTCATGGCAAATATGACATTCAGTATTCCTTATGGAAGTCATTGCTTCTTTGT
ACTAAAGGACATAATGGCCGCATTTCCCTCTGGCCATTCAATTATAATCATCTTGAGTAATTCTAAATTCCAACAACATT
TCAGGAAACTCCTCTGCCTCAAAAAGAATCGATGA
>UrmaTAS2R4_NW_007907159.1:6177389-6176487
ATGCTTCAGATACTCTTTTTCTCTGCCATTATTGTCTCAGCAATTTTGAATTTTGCAGGACTCATTGTAAATCTGTTTAT
CGCAGTGGTCAATTATCGGACTTGGCTCAAAAGCTCCAGAATCTCCTCTTCTAATAGGATCCTCTTCAGCTTGGGCATCA
CCAGGTTTCTTATGCTGGGACTGTTTCTACTCAACATCATCTACTTCTTAATCTCCCCAAATGTGGAAAGGTCAGTGCAC
TTATCCACTTTTTTCCTGATGTGTTGGATGTTTTTGGACTCTAATAGTCTCTGGCTTGTCACCTTGCTCAATGCCTTGTA
CTGTGTGAAGATTACGGACTTCCATCACGCACTATTTCCCCTGCTGAAACGAAGTCTCTCCCTAAAGATCCCCAGGCTGC
TGCTAGCCTGTGTGCTGATTTCTGCCTTCACCACTCTCCTGTATGTTGTGCTCAGACAGACATCATGCTTTCCTGAATTT
GTGCCGGGGAGAAACGGTACAGGATGTGACATCAGTGAGAGCGTCTTGTCTTTGGTGATCTCTTTGGTCTTGCGCTCATT
TCTCCAGTTCATCATTAATGTGACTTCTGCTTCCTTGTTAATACATTCCTTGAGGACACACATACAGAAGATGCAGAGAA
ACGCCACGATTTTTTGGAATCCCCAGACTGAAGCTCATGTGGGCGCTATGAAACTCATGACCTGTTTCCTCATCCTGTAC
ATTCCCTATTCAGTTGCTACTCTGCTACATTATTTCCCTTTTGTTGGGATGGATTTGGGAGCCAAATCCATCTGCATGGT
TATTTCCACCATTTACCCTCCAGGACATTCTGTTCTCATTATTCTCACACATCCTAAACTGAAGACAAAAGCAAAGGAGA
TTCTTTGTTTCAACAAGTGGTAG
>UrmaTAS2R5_NW_007907159.1:6164785-6163898
ATGCTGACTGCTGCCCTAACACTGCTGATGGTGGTGGCAGTGGCCGAATTTCTCATTGGCCTGGTTGGAAATGGAGTCCT
TATGGTCTGGAGTTTTGGAGAATGGGTCAGAAAATTCAACGGGTCCTCATACAACCTCATTGTTCTGGGCCTGGCTGTCT
GCCGATTTCTCCTGCAGTGGCTGATTATGATGGACTTAAGCCTGTTTCTGCTTTTCCAGAGCAGTCATTGGCTTCACAAT
CTCAGTGTCTTCTGGATCCTGGTAAGCCAGGCCAGCCTCTGGTTTGCCACTTTCCTCAGTGTCTTCTACTGCAGGAAGAT
CACGACCCTTGAACACCCTGTCTGCTTGTGGCTGAAGCAGAGGGCCTATTGCCTGAGTCTCTGGTGCCTTCTGGGGTACC
TCATGATCAGTTTGGTAGCTGTAGCCCGCATTGGCTTAAAGCCCTATCATCTTTCCCAAGGCAACAGCAGCATTCTGTAC
CCCCTCAAAAGCTGTCACTACCTGTATATATTAAAGCTCAATGCAGGAAGTGGGTTGCCTCTGATGGTATTTCTTGTTTC
TTCCGGGATGCTGATTGTCTCTTTGTATAGACACCACAAGAAGATGAAGGTGCATACAGCTGGTAGGAGAGATGCTCGGG
CCAAGGCTCACATCACTGTCCTGAAGTCCTTGGGCTGCTTCCTTATCCTTCATGTGGTTTACATCCTGGCCAGCCCCTTT
TCCATCGTCTCCAAGTCTTCTGCTGATGTCCTCATTGTCTTCATCTCCGAGACAGTCATGGCTGCCTACCCTTCTCTTCA
TTCTGTCATATTGATCATGGGGAATCCCAGGGTGAAGCAGACTTGTCAGAGAATTCTGTGGAAGACAGTGTGTGCTTGGA

GAGCCTAG
>UrmaTAS2R7_NW_007907282.1:45407-44469
ATGCCGGATAAAGTGGAGACCACCTTAATGCTGATAGCAGCTGGAGAGTTTTCAGTGGGGATTTTAGGGAATGCATTCAT
TGGATTGGTAAACTGCATGGGCTGGATCAAGAATAGGAAGATCGCCTCTATTGATTTAATCCTCACAAGTCTGGCCATAT
CCAGAATTTGTCTATTATGTATAATATTATTAGATTGTTTTATATTGGTGCTGTATCCAGATGTGTATACCACGGGTAAA
CAAATGAGAATCATTGACTTCTTCTGGACACTAACCAACCATTTAAGTGTCTGGTTTGCCACCTGTCTCAGCATTTTCTA
TTTCTTCAAGATCGCAAATTTTTTCCATCCCCTTTTCCTCTGGATGAAGTGGAGAATTGACAGTGTGATTCCGAGGATCC
TGCTGGGGTGCTTGGTCCTCTCTGTGTTTATTAGCCTTCTTGTCACTGAGAATTTGAATGATGACTTCAGGTATTGTGTT
AAGACAAAGAAGAAAACAAACCTAACTGTGAGATGCAGAGTAAATAAAGCTCAATATGCTTCCATCAAGATTTTCCTCAA
CCTGTTAACGCTATTCCCTTTTTCTGTGTCCCTGATCTCATTTCTCCTCTTGATCCTCTCCCTCTGGAGACATACCAGGC
AGATGAAGCTCAATGCCACAGGGTGCAGAGACTTCAGCATAGAAGCCCACATGGGAGCCATGAAAGCTGTCATCTCGTTT
CTCCTCCTTTTCATTGTCTACTGTTTGGCCTTTCTTGTAGCCACTTCTAGCTACTTTATGCCAGAGACTGAATTAGCTGT
GATCATTGGTGAGTTGATAGCTTTAATCTATCCCTCAAGCCATTCATTTATCCTAATTCTGGGGAGCAATAAATTAAGAC
AGGAATGCTTAAGGGTGCTTTGGAAAGTAAAGTATATCCTAAACAGAAAAAATTTCTAA
>UrmaTAS2R8_NW_007907282.1:50174-49245
ATGCTCAGTATGGAAGACAACATCTTTGTGATCATTTTAACTGGAGAATTCATAATAGGAATGTTGGGGAATGTATATAT
TGGACTAGTAAACTGGATTGACTGGATTAAGAAGAAAAAGATCTCCTCAGTTGACTATATCCTCACCAGTCTAGCCATCT
CCAGAATTTGTTTGCTCTGTATATTGATACTAAATGGCATCATAATGGTATGCTACCCAGATTTTTATGAAAATGGTAAA
CTACAGGCGGTCATTAATATCTTCTGGACACTCACCAACTACTTAAGTACATGGTTTGCCACCTGCTTCAATGTCTTCTA
TTTGCTCAAGATAGCCAATTTCTCCCATCCGCTTTTTCTCTGGCTAAAGAGAAGAATTGACAGAGTGATTCACTGGGTTC
TGCTGGGTTGTTTGGCCATTTCCTCTTTGATCAGCCTTATACTAGCAACGGCACCAAATTATGATTTTGAAATTCAGAAA
ATTATAAATCATAAAAGAAACTGCACTGAAATGTTCTATGTGAGTAAAAGTCAATACTTCAGCCTGTTGACTCTCTTTAA
CCTGTTGGCAATTGTCCCATGTGCTGTGTCATTGATCTCATTTTTCCTTTTAATTATGTCCCTAAGGAGACATATCAAGC
AAATGAAAGTCAGTGTTACAGGCTGTGGAGACCCCAGCACAGAGGCCCATGTGGGAGCCATGAAAACTATGACTTCATTT
CTCTTCCTCCTTTTTGTATACTATGGGGCTTCTCTTTTGGCGACTTTTCACTACCTTATGAAAGAAAGCAAGTTAGCTGT
GATGTTAGGAGAAATTATAGCAATTCTTTATCCTTCTGGTCATTCACTTATTTTGATTATTAGAAATAACAAGCTGAGGC
AGGCATCTATCAGGGTGCTGAGATTTGGAAGAACAGTCTGCATTATGTAA
>UrmaTAS2R9_NW_007907282.1:53044-52019
ATGCTAAGTACAATGGAGGTAATATACATGATCTTGATCGCTGGTGAATTGACTATGGGAATTTGGGGAAATGGATTTAT
TGTACTGGTTAACTGCACTGGCTGGCTCAAAAGAAGAGATGTCTCCGTGATTGACATCATCCTGGTGAGCTTAGCCATCT
CCAGAATTTGTTTGTTGTTTGTGATATCTTTAGATGGCTTTGTTATATGTATTTCTGGAGATACATATGCTGGTAGCAAG
CTAATGAGCATTGTGGATGTTTTCTGGACACTTAGCAATCATTCAAGTGTCTGGTTTACTTCTTGCCTCAGCATCTTCTA
TTTACTGAAGATAGCCAATATATCCCACCCATTTTTCATCTGGTTGAAACTAAAGATAAACAGAGTCGTCCTGGGAATTT
TTCTGATGTCCTTCCTTACCTGTGTAATTATTAGTGTTTCATTGAATGAGGACTTCTGGGATCCCTTCCAAGTCAGTCAT
AAGGAAAACATAACTTGGGAATTCAAAGTGAGTAAAATCCCCAATGGTTTCAAACTGGTTATCCTGAACCTAGGGGCTAT
CATTCCTTTTGGTCTTTGCCTAACTTCATTTCTCTTGTTACTTTTCTCCCTATTTAGACACACCAGGCAGATGAAACTTT
ATGCCATCGGGTCCAGAGACCCCAGCACAGAGGCTCACATGAGGGCCATAAAGGCAGTGATCATCTTTCTGCTTCTCTTC
GTTATGTACTATGCAGTCTTTCTTGTAGTAACCTCTAGCTTACTGATTCCTCAGGGAAAATTAGTGGTGATGTTTGGTGG
CATGATAGCCGTCATTTTCCCATCAAGCCATTCGTTCATCCTGATAATGGGGAACAGCAAACTGAGGGAGGCTTTTCTGA
AAGTGCTAAGGATTGTGAAGGGTTTCTGCAAAAGAAGGAAACCTTTTGTTCCACAGAGAATCCTGAGTACAAGGAGAAAG
AAATCAACAAAAGACCCTCTCCCTTGTCCCAATTGTTTACATTCATTTTTTAATACATGCTATTAG
>UrmaTAS2R10_NW_007907282.1:66011-65040
ATGCTAAGCATACTGGAAGGCCTCTTCATTTTTATAGCAGTTAGTGAATCAATACTGGGCGTTTTAGGGAATGGATTTAT
TGAACTTGTCAACTGTATTGACTGTGTGAAGAACAAAAAGTTTTCTATGATTGGCTTTATTCTCACTGGTTTAGCTACTT

CCAGAATTTGTTTGATATTGATAATAATTACAGATGGACTTACAAAGATATTCTTTCCAGATATGTATTCCTCTGGTAAC
CTAATTGATTATATTAGTTACTTATGGGTAATTTTCAATCAATCAAGTATCTGGTTTGCCACCAGCCTCAGTGTCTTCTA
TTTCCTGAAGATAGCAAATTTTTCCCACCATATTTTTCTCTGGTTGAAGGGTAGAATCAATAAGGTTCTTCACCTTCTGA
TGGGATCCTTGTTTATTTCATGGTTATTTACTTTTCCACAAATTGTGGAGATTATTAATGAGAGTAGAATGAAGAGTGGA
AATGCAACTTGGAACCTCCACATGCAGAGAAGTAAATTCTTTACTAAGCAGATTTTGCTCAACCTAGGAGTCATTCTTCT
CTTTACACTGTGCCTGATTACATGTTTCTTGTTAATCATTTCCCTTTGGAGACACAACAGGAGCATGCAACTGAATGTCA
CTGGACCCCGAGACCCCAGTACAGAAGCGCATGTGAAAGCAATGAAAGTTTTGATATCTTTTATCATCCTCTTTATCTTG
TATTTTATAGGCATTGCCATAGAAATATCATGTTTCACTCTGCCAGAAAACAAATTGCTGTTTATTTCTGGTATGGTGAC
CACAGCCATCTATCCCTGGGGTCACTCATTTATCCTAATTCTAGGAAACAGCAAGCTAAAGCAAGCCTCTTTGAGGGCCC
TGCAGCGATTCAAGTGCTGTGAGGTGGGGAGACTTCTCACAGCTGCACAGACACGTGTGGGGAGAAATGGATGTTCTAGG
AGAATAATCTAG
>UrmaTAS2R408A_NW_007907282.1:123323-122394
ATGCTAACGTTACTATCTGGCCTTTTTTCCATCCTCATAGTAACAGAATTTGTTCTAGGAACTTTTGCCAATGGCTTCAT
AGCAGTGGTGAACTGCACTGACTGGGTCAAGAGACAAAAGATGTCCTCAGCTGATCGAATTCTGACTGCTCTGGCGATCT
CCAGAATCAGTTTCCTCTGGGTAATGTTAGTGAACTGGTATGCAGCCGTGTTGAATCCAGCTTTATACATCTTCGAAGTA
AGACTTCTTGTTTATGTTGCGTGGACGGCAAGCAATCATTTTAGCGTCTGGCTTGCTACTAGCCTCAGTGTATTTTATTT
GTTCAAAATAGCCAATTTCTCTAGCCTTATTTTTCTTCGCCTGAAGTGGAGAGTTAAAAGTGCAGTTGTTGTGATCCTGT
TGGGGTCTTTGTTCTTTTTGGTTTTTCAGATTGCAGGGGTAAGCATGTATGAGAAAATACAGATGAAGGAACATGAAGGA
AACGTCACCAGGCAGACCAATTTGGGGGGCATTTTACACCTTTCGAGTTTGACTATATTCACACTAGCGAACTTTGTACC
CTTTGCTATATCCCTGACATCTTTTCTGCTGTTAATCTTTTCCCTGAGGAAGCATCTCAAGAGGATGCAATCCAGTGGTA
AAAGATCCCAAGATCCCAGCACCAAGGTCCACATAAGAGCCATGCAGACTGTGATCTCCTTTCTCTTTTTATTAGCTGGA
CACTTCACGACTCTCATTGTCACAGTCTGGAGTAGTAGTGGGCTGCAGAATGAACTATTCTTCATGCTTTTCCAAGCTTT
TGGATTCGTGTATCCTTCAAGCCACTCATTTATCCTGATCTGGGGAAACAAGAAGCTAAAACAGGCCTTTCTGTCTGTTT
TATACCAGGGGAAGTACTGGCTGAAAGAACAGAAACTCTCAACTCCATAG
>UrmaTAS2R38_NW_007907159.1:5977396-5978400
ATGTTGACTCTGACTCCTGTCATAACTGTGTCCTATGAAGTCAAGAGTGCATTTTTGTTCCTTTCGGCCCTGGAGTTTGC
AGTGGGGATCCTGACCAATGCCTTCATTTTCTTGGTGAATTTTTGGGATGTGGTGAGGAGGCAGCCACTGAGCAACTGTG
ACCTCATCCTTCTGAGTCTCAGCCTCACCCGGCTTTTCCTGCATGGGCTGCTGTTTCTGGACGCCCTCGAGCTTATCTAC
TTCCAGCAGATGAAAGACCCACTGAGCCTGAGCTACCAGGTCATCATCATGCTCTGGATGATCACAAACCAAGCTGGGCT
CTGGCTCACCACCTGTCTCAGTCTTCTCTACTGCTCCAAGATTGTCCGTTTCTCTCACACCATCCTGCTCTGCTTGGCAA
GCTGGATCTCCAGGAAGGTCCCCCAGATGCTCCTGGGTGCCATGTTCTTCTCCTCCATCTGCACTCTCCTCTGTTTGGGG
GACTTTTTTAGTAGACCTGACTTTGCATTCACAACTATGCTATTCATTAATAATACAGAGCCCAATTTGCAAATTGCAGA
ACTCAATTTCTATCATTCCTTCATCTTCTGCATCCTGGGGTCCATCCCTCCTTTCTTGCTTTTTCTAGTTTCTTCTGGGG
TGCTGATTGTCTCTCTGGGGAGGCACATGAGGACAATGAAGGTCAAAACCAAGGACTCCTGTGACCCCAGCCTGGAGGCC
CATATCAAAGCACTCAGATCACTCGTCTCCTTTTTCTGCCTCTATGTTGTGTCATTCTGTGCTGCCCTCATTTCAGTGCC
TTTACTGATGTTGTGGCACAACAAGATTGGGGTAATGATCTGTGTAGGGATCCTAGCAGCTTGCCCCTCAATACATGCAG
CAATCCTGATCTCAAGCAATGCCAAGCTGAGGAGAGCTGTGGAGACCACTTTACTCTGGGTTCAGAGCAGCCTAAAGGTA
AGGGCAGACCACAAGGCAGATCCCAGGACTCCAGATCTATGTTGA
>UrmaTAS2R39_NW_007907159.1:5202446-5201481
ATGACTGAAACCTGCAATCCCCCAGAAAATGAATTGTCACCATTTTACATCCTCTCGATTTTAACAATTATAGGAACTGA
ATGCATCGTTGGTATCATTGCAAATGGGTTCATTATGGCAATAAATGCAGCTGAATGGATTGAAAATAAGGCAGTTTCCA
CAAGTGGCAGAATCCTGTTTTTCTTGAGTTTATCCAGAATAGCTCTCCAAAGCTTCATGATGCTAGAGATTACCTTCAGC
TCAACATGCCCACGTTTTTATAATGAAGAACTTATATATGACATGTTCAAAGTAAGTTTCATGTTCTTAAATCATTGTAG
CCTCTGGTTTGCTGCCTGGCTCAGTTTCTTCTACTTTGTGAAGATTGCTGATTTCTCCCACCCCCTTTTCCTCAAGCTGA

AGTGGAGAATTTCCAGATGGATGCCCCAGCTTCTGTGGCTTTCAGTGTTTATTTCCTTGGGCTACAGTGCGCTCTTCTCT
AAAGACATCTACACTGTGTATTGTAACAATTCTTCTATCCCCTCCTCCAACTCCACTAAGAAAAAATACTTCACTGAGAC
CAATATGGTCAACCTGGCTCTTCTCTATAACCTGGGGATCTTCATTCCTTTGACCATGTTCATCTTTGCAGCCACCCTAC
TGATCATCTCTCTCAAGAGACACACCCTACACATGGAAAGCAATGCCACTGGCTCAAGGGACCCCAACATGGAGGCTCAC
ATGGGGGCCATCAAAGCTACCAGCTACTTTCTCATTCTCTACATTTTCAATGCAGTTGCTCTATTTCTCTATATGTCCAA
CATCTTTGACGTCAACAGCTTCTGGAATATTTTGTGCAGATTCATCATGGCTGCCTACCCTGCTGGACACTCCATTCTGC
TGATTCAGGGCAACCCTGGGTTGAGAAGAGCTTGGAAGCGGCTTCAGCCTCAAGTTCATCTTTACCTAAAAGAACAGACT
CCATGA

>UrmaTAS2R40_NW_007907159.1:5178836-5177880

ATGGCCACGGTGCGCACAGATGCTACGGAGAGAGACACATCCAGGTTTAAAATTGTCCTCACCTTGGTGGTCTCTGGAGT
AGAGTGCCTCACTGGCATCGCTGGGAATGGCTTCATCACGGCCACCCATGGCGCTGAGTGGGCCAGAGGCAAAAGGCTCC
CTGTCAGTGACTGCATTCTGCTGATGCTCAGTTTTTCCAGGCTCTTGCTGCAGATCCGGATGATGCTGGAGAATACTTAC
AGTCTACTGTTCTGGGTCACTTACAACCAAAACACAGTGTTCATACTCTTCAAAGTCACCGTCATGTTCCTGAACTATTT
CAACCTTTGGCTTGCTGCCTGGCTCAACATCTTCTATTGTCTTCGAATTGCCAGCTTTGCTCACCCTTTGTTCTCCATGA
TGAAGAGGAAAATCACAGGGCTGATGCCTTGTCTTCTGGGACTGTCACTGCTCATCTCCTTATGCTTCAGCTTTCCCTTC
TCTACAGATATCTTCAATGTGTACGTAAATAGTTCCATTCCCATCCCCTCCTCCAACACCACCGAGAAGAAGTACTTCTC
TGAGACCAATGTGGTCAACCTGGCTCTTCTCTATAACCTGGGGATCTTCATTCCTCTGACCATGTTCATCTTTGCAGCCA
CCCTGCTGATCATCTCTCTCAAGAGACACACCCAACACATGGAAAGCAATGCCACTGGCTCCAGGGACCCCAGCACGGAG
GCTCACGTGGGGGCCATCAAAGCTATCAGCTACTTTCTCATTCTCTACATTTCCAATGCAGTTGCTCTATTTATTTCTAT
GTCCAACATCTTCGACATCAACAGTTCCGGGAATATTTTGTGCAAAATCGTCATGGCCGCTTACCCAGCTGGCCACTCAG
TGCTACTGATCTTGGGTAACCCTGGGCTGAGGAAAGCCTGGAAGAGGTTTCAGCACTATGTTCATCTTCACCTGTAA

>UrmaTAS2R42_NW_007907282.1:194178-193231

ATGTTAGCTGGTTTTGATATAATCTTTCTTACACTGTCGACAGCAGAATTCATAATTGGAATGTTGGGGAATGTGCTTAT
TGGACTGGTAAACTGCTCTGAATGGGTCAAGAACCGAAAGATCTCTTTAGCTGACCTCATCCTCACCTGCTTGGCTATCT
CCAGAATCACTCAGCTGTTGGTGTCACTGTTTGAATCATTTATGATGGGACTAAATCCACCTTTCTACTCCATTTACAAA
CTAGCGAAACCTGTTACTTTGCTTTGGAGAATAACTAATCATTTGACTATCTGGTTTACTACGTGCTTAAGTATTTTCTA
CCTCCTTAAGATAGCTCAGTTCTCCCATTCCCTTTTCCTCTGGCTGAGGCGGAGAATGAACAGAGTGATTCTTGCAATTC
TTGTATTTTCTTTGTTGTTTCTACTGTTTGACTTTCTATTGCTAGAAGCATGGAACGATTTCTTCTTGAATATCCATGCA
ATGGATGAAAGTAATCTGACTTTATTTATGAACGAAAGTAAAACTTTCTATATTAAAAGCCTGATTCTTCTCAGTTTTTC
CTATATCGTTCCTATTGCTCTGTCCATGACCTCATTGCTTCTTTTATTTCTGTCCTTGATAAAACACATCAGGAATCTGC
AACTCAACTCCATGGGCTCCAGGGACTTCAGCACCCAGGCCCATAAGAGGGCCATTAAAATGGTGGTGTCTTTCCTCTTC
CTTTTCACAGTTCATTTTTTTTCCATACAATTGTCAAATTGGTTGCTTTTTTTATTTTGGAACAAGAAGAACACAAAGTT
TATTATGTTGACTGTATATGTCTTTCCCTCAGGCCACTCACTAATTTTGATTCTGGGAAACAGCAGGCTAAGACAGACAG
CCTTGAAGGTACTGTGGCATCTTAAAAGCTCCCTGAAAAAGGAAAACCAATTTCATCTTTACAGATAG

>UrmaTAS2R62_NW_007907159.1:5004106-5003186

ATGCCCTCCTCACCTGCATTGATCTTCATGGTCATCTTCTTCCTGGAGTCATTGGCTGCAATGTTGCAGAATGGCTTCAT
GGTTACTGTGTTGGGCATGGAGTGGGTGCGACGCCGGATGCTGCCCGCAGGTGACATGATTGTGGCCTCTCTGGCCGCCT
CCCGGTTCTGCCTACATGGGGTGGCCATCCTGAACAACCTCTTGACCTTCTTTGAAATGGACTATTACCAGACCCCCTGG
AACTTCATCAACACTCTCACTTCCTGGCTCACTGCCTGGCTTGCCATCTTCTACTGTGTGAAGATCGCCCTCTTCTCCCA
CCCTGTCTTCTTCTGGCTGAAGTGGAGGATTTCTCGGTCAGTGCCCAGGCTGCTGCTGGGCTCCCTGGTCTTGGCTGGTC
TGACAGTCATCTCATCAGCCATTGGGACTAGAATTTTTATGCAGATGATTGCATCCCAGAGTTCCCAAGGAAACAGCACC
CTGGCTGATACAGTACAGTCCTTCTATTGGTGTTTTACTGTACCTCATACAATGCTTACGTTGTCAATCCCATTCCTCCT
GTTCTTGGTGTCCACGTTCTTGCTCATGTTCTCACTGTGCCAGCACTTGAGGCGGATGAGGGACCATAGACTGAGCCCAT
GTGATCCTAGTATCCAGGCTCACACCAGGGCCCTGAAGTCACTTGTCTTCTTCCTTGTCTTCTATACATCATATTTCCTG

TCTCTGATTGTTGTTGTTATGAAAATCACAATCTTCCAGAGTCACTGGTACTGGGCCTGGGAAATGGTAACCTATGCAGG
CATCTGTCTGCACTCCAGCATCCTGGTGGTAAGCAGCCCCAAGCTGAGAAAGGTCCTGAAGACCAGGCTTTGGAAAGCTC
TGGACAAAGGATGGTCTGTCTCAAGTTATCAGTATCAATAA
>UrmaTAS2R3P_NW_007907159.1:6186723-6185753
ATGCCGGGGCTGGAGAAATGCATATTCCTGGTTCTGTCTGCCACTGAGTTCATTCTGGGGATGCTGGGGAATGGTTTCAT
AGTGTTGGTCAATGGCAGCAGCTGGTTCAAGAACAAGACAGTCTCTTTGTCTGACTTCATCATCACTAACCTGGCTCTCT
CCAGGATCGTTCTGCTGTGGATTCTCTTGGTTGATGGTGTTTTAATGGTGTTCTCTTCCAAAGTACATGATGAAGGGATA
GTGATGCAAATTATTGATATTTTCTGGACATTTACAAACCACCTGAGCATTTGGCTCGCCACCTGTCTCAGTGTCCTCTA
CTGCCTGAAAATCGCCAGTTTCTCCCATCCTACATTCCTCTGGCTCAAGTGGAGAGTTTCCAGGGTGGTCGTACACATGA
TTTTGGGTGCCCTGTTCTTATCGTGTGTCAGTGCCATGTCTCTGATCCAGGAATTTAAGATCTATTCTGTTCTCAGTGGG
ATCGATGGCACAGGGAATGTGACTGGGCACTTTAGAAAGAAAAGAAATGAATACAAAGTGATCCATGTTCTTGGGACTCT
GTGGAACCTCCCTCCGCTAATTGTGTCTCTGGCCGCCTACTTTCTGCTCATCCTGTCCCTGGGGAGACACATGCAGCAGC
TGCAGCAAAGCGGCATCAGCTGCAGCAAAGCGGCATCAGCTCCAGAGATCCAAGCACTGAGGCCCACCAGAAAGCCATCA
AAATCATCATCTCTTTCCTCTTTCTCTTCCTGCTTTACTTTCTGGCCTTTTTGATTACAACATCCAGTTATTTCATCCCA
GGAACCGAGATGGTTAATATAATCGGAGAAGTAGTTACAATGTTTTATCCTGCTAGCCACTCATTCATTCTCATTCTGGG
AAACAACAAGCTGAAGCAGACGTTTGTGGAGATGCTGTGGTGTGAGCCTGGCCATCTGAAGCCTGGGTTCAAGGGACCTT
TTGCCCCATAG
>UrmaTAS2R12P_NW_007907282.1:87980-87035
ATGGCGAGCACATTGAAGAATGTACTTCTGATGATTTTTGCTGGAGAATTCATAATGGGGTTTTGGGAAATGGATTCATT
GTATTGGTTAATTGTATTGATTGGATCAGGAGCTGGAAGTTCTTCCTGATTGACTTTATTCTTACCTGCTTAGCTATTTC
CAGAATATTTCTGCTGTGCATGATAATTCTAGGCATAGGTGTGGATATAATTTGTGAGGAAATACGGTACAATGATAATC
AGCTGATGGCTTTTGAAATCGTCTGGACAGGATGCAATTATTTCTGCACAACCTGTACGGCCTGCCTCAGTGTCTTCTAC
TTCCTTAAGATAGCCAACTTTTCCAATCCCATTTTCTTCTGGATAAAACAGAGAATTCACGGACTGCTTCTCGTTATTGT
CCTGGGGGCGGTCTTCTCTTTCTGCTTGTCCCTGCTTTTTAAGGATATAGTATTTAAGAACCTGACCGAAACCAGGGTAA
ACATTGAAAGCAATCGGACATCGAATTTCACAGGGAGAAAATATGATTTATTAACTTCTAATATACTCCTGAACATAATG
TTCGTCATCCCCTTTGGAGTGTCTTTGGCTTCCTTTGTCCTTTCGATCCATTCCTTATGGAAACACACCAGACAGATGAA
GGGCGCAGGTTCTGGGGATCTTGTCACAAAGGCCCATGTGAGAGCCATAAAGTCGATGATTTCATTCCTACTCTTCTTCT
TTATGTACTATTTGAGCAACATTATCATATATTTGGCCTATGTCATTCTAGACAGTTTGGTGGCAAAAATATTTGCTAGT
ATGTTAGCATTTTCCTATCCATCTGGCCATCCATTTCTTCTGATTTTATGGAACAGCAAATTGAAGCAGGCTTCTCTCTG
TGTCCTGGGGAAGTTGAGGCGGTGCAGGAACCTAAGGAAGCCCGCAAACCCATAAAACGTGCCTGA
>UrmaTAS2R16P_NW_007907257.1:6151107-6150206
ATGGTGCCCCTCCAACTCACTGGCTTCTTCGTAATCCTCTGGGTGCTTGAATCGTTGACAGCAATTGTGCAGAGCAACTC
AATTTTTGCAGTGCTGGGCAGAGTGGGTGCCGGCCAGAGGGCTGTCATCATCGATGGACTTGACTCTCACCTGCCTGGGT
ATCTGCTGCTTCTGTCTACAGTGGGTGTCCGTGCGGAACAACTGTTGCTCCAATTTTAACCCTAACTATGTATTTTGTTA
TTTATTGGTCACCTGGGAGTTTACTAATACTCTTACTGGTTAACCAGCTTGCCTGCTGTCTTCCACTGTGTCAAAGTCTC
CTCCCTAACCCACCCCAGCTTCCTCTGGCTGAGGCGGAGAATTTTGAGGTTCGTTCCTTGGCTGTTGCTGGATTCTCTGT
TGATTTCTTGTGTGTCAATCCCCTTCTCAGCTGTTAGGAATTATATGAATGGGCACTTAGTCACCATGGGGCATTTCTCT
ACAAACAGCACTAGGGTGGAGAGACTTAAGACGTTTCACCTGTATTTTACCATCTCTTATGCAATGGTCACATTGGTTAT
TCCTTTCCTCCTGTTCCTGGCCTCCACCGTCTTGCTCATGGCCTCCCTGTTCCAACCCGTGGAGCAGAAGAGAGCCACAG
CACTGGCCACTACAGCTCCAGTGTGGAAACACACGCCACCGCCCTGAGCTCTCTTGCCATCTTCCTCATTTCCTTCACCT
CTTACTTGCTGACCCTACTCATCTCTATTATGAACATCTCATTGGATAAAAGGTCCTGGTTCTGGGCCTGGGAAGCTGTC
ATCTATGCTATAGTCTCAATTCACTCCCGTTCACTAATGCTGAGCAGCCCTAAATTGAAAGAGGTTTTAAAGATAAGGGG
CTGGGGCCTAGAGGCTGCCTGG
>UrmaTAS2R408BP_NW_007907282.1:141235-140192

ATGCTAACATTACTATCGGGCCTTTTTCTCATCCTCATAATAACAGTATTTGTTCTAGGAAATTTTGCCAATGGCTTCAT
AGCAGTGGTGAACTGCACTGACTGGGTCAAGAGACAAAAGATGTCCTCAGCTGATCGAATTCTGACTGCTCTGGCGATCT
CCAGAATCAGTTTCCTCTGGGTAATGTTAGTGAACTGGTATGCAGCCGTGTTGAATCCAGCTTCATTTAGCTTAGAAGCA
AGACTTCTTGTTCATGTTGCGTGGGCGGCAAGCAATCATTTTAGCGTCTGGCTTGCTACTAGCCTCAGTGTATTTTATTT
GTTCAGAATAGCCAATTTCTCTAGCCTTATTTTTCTTCGCCTAAAGTGGAGAGTTAAAAGTGTAGTTGTTGTGATCCTGT
TGGGGTCTTTGTTCTTTTTGGTTTTTCAGATTGCTGTGGTAAGCATGTATGTTAAAATTCAGATGAAGGAATATGAAGGA
AACGTCACCAGGCAGACCAAATTTTACACCTTTCAAGTATGACTATATTCACACTAGCGAACTTTGTACCCTTTGCTATA
TCCCTGACATCTTTTCTGCTATTAATCTTTTCCCTGAGGAAACATCTCAAGAGGATGCAATCCAGTGGTAGAAGATCGCA
AGATCCCAGCACTAAGATCCACATAAGAGCCATGCAGACTGTGATCTCCTTTCTCTTCGTATTAGTAGGTTACTTCCTGG
CTCTCATTGTCACCATCTGGAGCTCTAAGTGGTTGCCAAACAAGTTGATCTTCCTGCTTTGCAAGGCTATTGGAATCATG
TATCCCTCAAGCCACTCATTTATCCTGATTTGGGGAAACAAGAAGCTGAGAGAGGCCCTTCTGTCATTCCTGTGGCAGCT
GAGGTGCTGGCTGAACGAAAGAATATAAGAGGAGCCTTTTGTGTCTTCTAGGAGAAAACAAGTGGATGGAGTCTATAACA
TTTTATACTTTTTTTACCTCTTTTTCTAATGTGTATATAATTGAGTAATTTCTAAAGGTTTACCTAGAAAAGTCTTTTAC
CTAA
>UrmaTAS2R60P_NW_007907159.1:4998639-4997685
ATGGATGGAGATGACATGGTTCCAGAACCTCCAGTGACTGACTGATAAGAGAGCCATCACCCCGGTTATCATCTTATTTT
ATTTTATTTTTTGTTTCTGGGGACGGCAGTGAGCAATGGCTTCATCAATGTAGCACTGAGCATGGAACGGTTGCTACAGA
GAACACTGTTAACCTTGTGATAAATTATTAGTCAGCCAGGGCCTCTTGCCTCCATCTGCAGTGGGTGGTGATGAGGAAGA
GCATTTATATTTTCCTGTATCCAGTGGCCTTCCCATACAACTCTGTACTGCAGTTCCTAGCCTTCCAATGGAACTTACCG
AACACCGCCACCTTATGGTTCTTCACCTAGCTCAGTGCTTTGTGTTGCGTGAAGACCGCAACCCTCACCCTCCCCATCTT
CCTCGGGCTAAAACAGAAGATGTCTGGGTTGGTTCCATGGATGCTGCTCGGTTCTGTGGGGCTCTCCAGCTTGAGCACCA
TCCTATTTTCATAGGCAACCAGAGCTTATATCTCTACTTTTTAAGGAGAGGGTTGCAATCTTGGAATGCCATTAGGAATA
CTATAAGATCACATGAGAAATCCTACTTCTTCTCTTTAAAACTTGTTACCTGTACAGTCTTTACTGTTGTCTTCCTTGTC
GGCATGGTTTTGCTCATGTCTCTGGAAAGACACTAAGAAGGCCCTGCTGTCCATCGCAGCTTTCGTGACTCCAGTGTCCA
GGCGCACATCAAGATTCTTTTGGCTCTCATCTCCTTTGCTATTGTCTTCACCTCCTATTTTCTGTCACCGGTGCTCAGCG
CCGCAGGTGTTTTTCCATCTTGGAACTTAGGCGCTGGGTGTGGCAGGCAGTGATTTATTTGTGCACAGCCACCCACCCCG
TCACTGCTCTTGAGCAACCCCAGGCTGAGAGGTGTGCTGGAGGGGGGCTGCTGTGCACAGTGCTGGGCATCTTGA
>UrmaTAS2R67P_NW_007907282.1:190409-189470
ATGCCATCTGGAGTTGAAAATGCTTTTCTGATGGTGGCAACAGAATTCATAACTGGAATGTTGGGGAACAGTTTCATTGT
ACTAGTTAACTGCATTGACTGGGTGAAGATTCAAAAGCTCTCATCAGCTGACTGCATCCTCACCAGCCTGGCTCTCTCCA
GAATCGTTCTTCTTTGTACAGTACTATTTGATTAGTATTTTACGGTGTTTTGGGCACATCTTTATGCCACTGACAAACTA
GCAAAATTCATTAATATTTTTTGGACACTGAGCAATCATCTAGCTATCTGGTTTGCCACCTGTCTAAGTGTTTTCTACTT
CTTTAAAATAGCCAATTTCTCCCACCCCTGTTTCACCTGGCTGAGGTGGAGACTTACCAGAGTGCTACTTGTACTTCCAC
TGGGGTCTTTATTCTTATTGTTTTGCAACTTTGAATTATCAGATATATTTACTAATTTCTGGGTTAATGTCTATCGAGGA
TATAAAAGAAACTCAACTTGGTCTCTAGATGTAAGTAAAACTCTGTATTTTAACAGCTTGATTGTTTTCAGTTTCATCTA
CTTAATCCCCTTTCTTCTGTCGCTGGCCTCACTGCTCCTTTTCTTTCTTTCCCTGATGAGAGAGACACACCAGGAATATG
CAACTGAACTCCGGCTCTAGGGACTTCAGCACAGAGGCCCACAAAAGGGCCATAAAAATGGTGATGTCTTTCCTTCTCCT
CTCCACGGTTCATTTTTCTTTCCTCCTAATAACAGGTTGGACTTTCCTTTTACTGCATAATTCTCAGGTCAATTTTGTTA
TCATATTATTATCGACTCTTTTTCCTTCAGGCCACTCATTTATTCTGATTTTGGGAAACAGCAAGTTGAGAAAAACTGCT
TTAGGATTACTGTGGCATCTTAATAGCCACCTGAAAATGGTGAAACCTTTAGCTTCATAG
>UrmaTAS2R18P_NW_007907282.1:176023-175099
ATGGAAATGTGAGTTGGAATGAAGATCTCTTTCTGGTAGTGGCATTCTGGTAGGAATGCAGGGAAATGGGTTCATTGGAC
TGGCAAACTGCAGTGAATGGTTCAAGAATGGGAAAGTCTCATCAACTGATTTCATCCTTACCAGCTTGGCTATGGTCAGA
ATCACTCAACAGTGGGTGACACTATTCGATTCATTTCTGGTGGGACTGTCTCCACATCCCTATGCCATTGGTAAACAACT

AGCAAAAGCGGTTACTATTCTTTGGGCACTAATCACTGAACTCCCTGGTTTGCTACCTGCCTAACATTTTCTGCTTCTTG
AAGGTAGCCAAGTTCTCCCACTTCTTTTTCATCCGGCGGAGGTGGAGAGAGAACAGAATGGGTCTCGTGCTTTTCCTGGG
GACTTTGTTCTTATAGTCTGTTAACTTCTGAATGTAGGATGCCCCTGGTGAGTTGTGGATGAGTACCTATGGGATACATG
AGAGACTTTGCATTTAGACATACGTGTTTGGTCTTAAAAGGCTTCTACTTCCTAGCTTCACCTATGTTAAGCTTTCTCCC
CTTTCTCCTGTCCCTGACCTGTTTGCTCCTTTTATTTCTGTTCTTGGTGAGACACCAAGAACCTACAGCTCAACCGGATG
GGCTTGAGGGACTCCAGCAGAGGCCCACACAAGGGCCGTGAAAGTGGTGACGACCTTCCTCCTCCTCACCACCATTTACT
TATTTCCACTCCAGTAGCAAGTTGGATCTTCCTTAAGGTACAGAGATAACAATCAGGCCAAGATGCTTGTCATGGTGACT
TCAGCTTCCTTTCGCTCATAGCACTCTCTAGTTTTGGGAAACAGCATGCTAGGAGGGATACCTGAGACTACGGTGGCATC
TGAAATTCTGTCTGAGAAAATGAATACATTTAGCTTCATAGATAG
>UrmaTAS2R41T_NW_007907159.1:4977957-4977032
ATGCAGCCAGCACTCTCAGCCTTCTTCATGCTGCTCTTTGTCCTGCTATGCCTCCTGGGAATCCTGGCCAACGGCTTCAT
TGTGCTGGTGCTGAGCAGGGAAAGGATGCGGCGTGGGAGGCTGCTTCCCTCTGACATGATCCTCATGAGCTTGGGTGCCT
CCCGCTTCTGCCTGCAGTGTGTTGGAATGGTGAACAACTTCTACTACTACCTCCACCTGAACGAGTACAGCAGCGGTCCT
GCGCGGCAGTTCATCAGTCTCCACTGGGACTTCCTGAACTCGGCCACCTTCTGGTTGGGCTCTTGGCTCAGTGTCCTCTT
CTGCATGAAGATTGCTAACTTCACCCACCCTACCTTCCTTTGGCTGAAGTGGAGGTTCCCAGGGTCAGTGCCCTGGCTCC
TCATGGCTTCTCTCCTGATCTCTTTCATCGTCACCCTGCTCTTCTTCTGGGGAAACCATGCTTGTATCAAGGATTCTTAA
TTAGAAAATTTCCTGGGAATATGACCTTCAAGCAGTGGAGCAGGAGGCTGGAAATTCACTATTTCTTGCCCCTGAAACTT
ATCACCTTGTCAATTCCCTGCTCTGTCTTTCTGGTCTCAATTGCACTGTTGATTAATTCTCTGAGGCGACACACAGGGAG
AATGCAGCGCAGTGCCCACAGCCCACAGGACCCTAGCGGCCAGGCTCACACCAGAGCTCTGAAGTCGCTCATCTCCTTCC
TCATTCTTTATGCTCTGTCTTTCGCGTCCTTGGTCATCGATGCTGCGGGTTTCTTCTCCTCAGAGAGTGACTGGTACTGG
CCGTGGCAGATTTTAATCTACCTGTGCACATCTGTCCATCCCTACATCCTCATCCTCAGCAACCTCCGGCTTCGCGGGGT
GTGCAGGCAGCTACTTCTGTTGGCCAGGGTCTTCCGGCTGGCCTAG
>LeweTAS2R2_NW_006383293.1:242174-241260
ATGGCCTCCTCTTTGTCAGCTATTCCTCATGTTATCATCATGTCAGCAGAATTTATCACAGGGATTACAGTAAATGGATT
TCTTATCATCATCAACTGTAAAGAATTGATCAAAAGCAGAAAGCTAACAACAGTGCAACTCCTTTTCATATGTATAGGTA
TGTCGAGATTTGGTCTGCTGATGGTGTTAATGGTACAAAGTTTTTTCTCTGTGTTCTTTCCATTCTTTTATAGGGTAAAA
GTTTATGGTGCAGCAATGTTGTTCTTTTGGATGTTTTTAAACTCTGTCAGTCTCTGGTTTGCCACCAGCCTTTCTGCATT
TTACTGCCTCAAGATATCAGGCTTCACTCAGCCCTATTTTCTTTGGCTGAAAGTCAGGATCTCAAAGTTAATGCCTTGGC
TGCTTCTGGGAAGCTTTCTGGCCTCCATGAGCACTGCAGCTCTGTGTATTGAGGCAGATTACCCTAAAAATATGGACAGT
GATGATGTCCTCAAGAATGCCACGCTGAAGAGGACTGAACCCAAGATAAGGCAAATTAATGAAGTGCTTCTTGTCAACTT
GGCATTACTATTTCCTCTAGCCGTATTTGTGATGTGCACTCTTATGTTATTCATTTCTCTCTACAAGCACACTCATCGGA
TGCAAAATGGATCTCATGGTGTTAGAAATGCCAGCACAGAAGCCCATATAAATGCATTAAAAACAGTGATAACATTCTTC
TGCTTCTTTATTTCTTATTTTGCTGCCTTCATGGCAAATATGACATTCAGTGTTCCTTATGGAAGTCATTGCTTCTTTGT
ACTCAAGGACATAATGGCAGCATTTCCCTCTGGCCATTCAATTATAATCATCTTGAGTAATTCTAAATTCCAGCAGCCTT
TCAGGAGATTTCTCTGCCTCAAAAAGAATCAATGA
>LeweTAS2R408_NW_006383381.1:1783908-1782952
ATGGTAACTTTACTACCGGGCATTTTTTCCATCCTAGTAATAACAGAATTTGTTCTAGGAAATTTTGCCAGTGGCTTCAA
AGCAGTGGTGAACCGCATTGACTGGGTCAAGAGACGAAAGATGTCCTCAGCTGATCAAATTCTCACTGCTCTGGCGGTCT
CCAGAATCGGTTTGCTCTGGGTAATGTTAATAAACTGGTATGCAACTGTGTTGAATCCAGCTTTATATAGCTTAGAAGTA
AGACTTCTTGTTCATATTGCGTGGACGGTAAACAATCATTTTAACATCTGGCTTGCTACTAGCCTCAGCGTATTTTATTT
GTTCAAAATAGCCAATTTCTCTAGCCTTATTTTTCTTCGCCTGAAGTGGAGAGTTAAAAGTGTAGTTCTTGTGATACTGT
TGGGGTCTTTGTTCTTTTTGGTTTTTCACGTTGCAGTGGTAAGCATATGTGAGAAAAGCATATATGAGAAAAATTCCTTC
ATAAGCAAGATGAAGGAATATGAAGGAAACATCACTAGGCAGACCAAATTGGGGAACATTGTAAGACTTTTGAATATGAC
TGTATTCACGCTAACAAACTTTGTGCCCTTTGCTATATCCCTGACATCTTTCCTGCTGTTGATCTTTTCCCTGTGGAAAC

ATCTCAAGAAGATGCAATCCAGTGGAAGGAGATCCCAAGATCCCAGCACCAAGGTCCACATAAGAGCCATGCAGACTGTG
ATCTCTTTTCTCTTGTTATTAGCTGGTCACTTCCTGACTCTAATTGTCACAGTCTGGAGTTCTAATGGGCTGCAGAACAA
ACTATTCTTCATGCTTTACCAAGCTTTTGGATTCTTGTATCCTTCAAGCCACTCATTTATCCTGATCTGGGGAAACAAGA
AGCTCAATCAGGCCTTTCTGTCTGTTTTATACCAGGGGATGTGCTGGCTGAAAGAACAGAAACTCTCAACTCCATAG
>LeweTAS2R1P_NW_006385676.1:194588-195480
ATGCTAGAGTTTTACCTTATTATCCATTTTCTTTTTGCAGTGTTACAATTTCTCATCGGGGTTTTAGCAAACGGCATCAT
TGTGGTTGTGAATGACACTGAGTTGACCAAGCAGAGAAAGATGATTCCATTGGCTCTCCTTCTTTCCTGCCTGGGGATTT
CCAGGATTTGTCTGCAGTCAGTCATCTACATTAATCGGGCTAATGTCTCTTTGATTGAAGTCCCTCTACTTGTTGAGAAT
TTTGTAATTTTCATGTTTGTAAATGAATTGGGACTTTGGTTTGCCTCATGGCTCTGTGTTTTCTACTGCGCCAGGATTGC
CCCCGTAGCTCACCCACTCTTCTTCTGGTTGAAGATGAGGATATCGAAGTTGGTGCCATGGCTGATCCTCAGGTCCCTGC
TGTATGCATCCATCCCCTCTATTTCTACAACAAATATACATGGGTTCTTTCCCAACAACTCTTGTTGGGCTTTTTCTCC
TCCAACGCAACAACTCAAATCAAAGAAACATCTGCTTTACAGATCGTCTTTCTTGTGAGGTTATTATCGCCGTTATTTAT
CTTCCTTGCTTCTGCCCTGCTCTTGATGTTTTCCCTGGGGAGACACACCTGGCAGATGAGAAACGCGGCGGTGGGCACCA
GGGTCCCTAGCACAGCTGTCCATGTGAGGTCGCTTCTGTCTGTCTGTCCTTCCTGGTCCTCTGCCTCTCCCACTGCATGA
CAGCTGCTTTGCTCTCTTCACAGATTTTTAAGCTCAGGAGCCTCATGTTTCTGTTCTGTATCTGGGCGTTTGGGTCATAT
CCCTATGGACACGCTACGATCTTAATTTTAGGAAATCCTAAACTGAAGCAAAATGCAAGGAAGCTCCTCCTCCATGGGAA
GTGCTGCCAGTGA
>LeweTAS2R3P_NW_006384816.1:534183-535137
ATGTCAGGGCTGGAGAAATGGATGTTCCTGGTTCTGTTTGCCACTGAGTTCATTCTGGGGATGCTGGGGAATTGTTTCAT
AGTACTGGGCAATGGCAGCAGCTGGTTCAAGAACAAGACGGTCTCTTTGTCTGACTTCATCGTCACTAACCTGGCTCTCT
CTAGGATCGTTCTGCTGTGGATTCTCTTGGTTGATGGTGTTTTAATGGTGTTCTCGTCCAAAATACATGATGAAGGGATA
GTGATGCAGATTATTGATATTTTCTGGACATCTACAAAGCACCTGAGCATTTGGCTTGCCACCTGTCTCAGTGTCCTCTA
TTGCCTGAAAATTGCCAGTTTCTCCCATCCTACATGCCTCTGGCTCAAGTGGAGAGTTTCCAGGGTGGTCATACACATGA
TTTTGGGTGCCCTGTTCTTATCGTGTGTCCGTGCCATATCTCTGATCCAGGAACTTAAGATCTATTCTGTTCTCAGTGGG
ATCGAAGGCACAGGGAATGTGACCGAGCACTTTAGAAAGAAGAGAAATGAATACAAAGTGATCCATGTTCTTGTGACTCT
GTGGAACCTCCCTCCTCTAATTGTGTCTCTGGCCACCTACTTTCTGCTCATCCTTTCTCTGGGGAGACACATGCAGCAGC
TGCAGCAAGGTGGCGTCAGCTCCAGAGATCCAAGCACTGAGGCCCACCAGAGAGCCATCAAAATCATCATCTCTTTCTTC
TTTCTCTTCCTGCTTTACTTTCTGGCCTTTTTGATTACATCATCCAGTTATTTCATACCAGGATCTGAGATGGTTAATAT
AATCGGAAAAGTAGTTAGTTACAGTGTTTTATCCTGCTAGCCACTCATTCATTCTCATTCTGGGAAACAACAAGCTGAAG
CAGACATTTGTGGAGATGCTGTGGTGTGAGCCTGGCCATCGGAAGCCTGGATTCAAGGGACCTTTTGCCCCATA
>LeweTAS2R4P_NW_006386560.1:45932-46833
ATGCTTCAGATATTCTTTTTTCTCTGCCATTATTGTCTCAGCAGTTTTGAATTTTGCAGGACTCATTGTGAATCTGTTTA
TCGCAGTGGCCAATTATCAGACTTGGCTCAAAAGCCCCAGAATCTCCTCTTCTAATAGGATCCTCTTCAGCTTGGGCATC
ACCAGGTTTCTTATGCTGGGACTGTTTCTACTCAACATCATCTACTTCTTCATCTCTCCAAATGTGGAAAGGTCAGTGCA
CTTATCTACTTTTTTCCTGTTGTGTTGGATGTTTTTGGACTCTAAATTAGTCTATGGCTTGTCACCTTGCTCAATGCCTT
GTACTGCATCAAGATTATGGACTTCCAACACGCAGTATTTCTCCTGCTGAAATGAAATCTTTCCCCAAAGATCCCCAGGC
TGCTGCTAGCCTGTGGGCTGATTTCTGCCTTCACCACTCTCCCGTATGTTGTGCTCAGACAGACATCATGCTTTCCTGAA
TTTGTGCCTGGGAGAAATGGTACAGGATGTGACATCAATAAGAGCGTCTTGTGTTTGGTGATCTCTTTGGTCTTGTGCTC
ATTTCTCCAGTTCTTCATTAATGTGACTTCTGCTTCCTTGTTAATACATTCCTTGAGGACACATATACAGAAGATGCAGA
AAAATGCCACTATTTTTTGGAATCCCCAGACTGAAGCTCATGTGGGTGCTATGAAGCTCCTGATCTATTTCCTCATCCTG
TATATTCCTATTCAGTTGCTACTCTGCTACATTATTTCCCTTTTGTTGGGATGGATTCGGGAGCCAGATCCATCTGCATG
GTTATTTCCACCATTTACCCTCCAGGACATTCTGTTCTCATTATTCTCACACATCCTAAACTGAAAACAAAAGCAAAGGA
GATTCTTTGTTTCAACAAGTAG
>LeweTAS2R8P_NW_006383381.1:1716069-1715211

ATGCTCAGTACAGAAGACAACATCTTTGTGATCATTGTAACTGGAGAATTCATAATAGGAATGTTGGGGAATGTATACAT
TGGACTAGTAAACTGGATTGACTGGATTAAGAAGAAAAAGATCTCCTCAATTGACTATATCCTCACCAGTCTAGCCATCT
CCAGAATTTGTTTGCTCTGTGTACTGATACTAAATGGCATCATAATGGTATGCTACCCAGATTTTTATGAAAATGATAAG
CTACAGGCAGTCATTAGTATCTTCTGGACACCCACCAACTACTTAAGTACGTGGTTTGCCACCTGCCTCAATGTTCAAGA
TAGCCAATTTCTCCCATCCACTTTTTCTCTGGCTAAAGAGAAGAACTGACAGAGTGATTCACTGGATTCTGCTGGGTTGT
TTGGCCATTTCCTCTTTTGATCAGCCTTATACTAGCAACAACACCAAATTATGATTATGAGTTTCATAAAATTATAAATC
ATAAAAGAAACTGCACTGAAATGTTCCATGTGAGTAAAAGTCAATACTTCAACCCTTTGACTCTCTTTAACCTGTTGGCA
ATTGTCCCATGTACTGTATCATTGATCTCATTTTTCCTTTTAATTATGTCCCTATGGAGACATATCAAGCAAATGAAACT
CAGTGTTACAGGCTGTGGAGACCCCAGCACAGAGGCCCATGTGAGAGCCATGAAAACTATGACTTCATTTCTCTTCCTCC
TTTTTGTATACTATGGGGCTTCTCTTTTGGCGACTTTTAGCTACCCTATGAAAGAAAGCAAGTTAGCTGTGATGTTAGGA
GAAATTATAGCAATTCTCTATCCTTCCGGTCATTCACTTATTTTATTTTATTTTTTTAA
>LeweTAS2R12P_NW_006383381.1:1733157-1732169
ATGGCAAGCACATTTAAGAATGTACTTATGATGATTTTTGCTGGAGAATTCATAATGGGGCTTTTGGGAAATGGATTCAT
TATATTGGTTAACTATATTGATTGGATCAGGAGCTGGAAGTTCTTCCTGATTGACTTTATTCTTACCTGCTTAGCTCTTT
CCAGAATATTTCTGCTGTGCGTAACAATGCTAGGCATAGGTGTAGACATAATTTGTGAGGAAATATGGTACAATGATAAT
CAACTGATGACTTTTGAAATCCTCTGGACAGGATCCAATTATTTCCACACAACCTGTACGGCCTGCCTCAGTGTCTTCTA
CTTTCTCCAGATAGCCAACTTTTCCAATCCCATTTTCTTCTGGATCAAACAGAGAATTCACAGCCTGCTTCTCATTATTG
TCCTGGGGGTGGTCTTCTCTTTCTGCTTGTCCTTGCTTTTTAAGGATACAGTATTTAAGAACCTGATCAAAACCAGGGTA
AACACTTCAGTGTTATTAATATATAATGTTATATTAGTTTCAGGTGTACAATATAATCGGACATCGAATTTCACAGCAAG
AAAATATGATTTATTAACTTCTAGTATAGTCCTGAACATAAAGTTCATCATGCCCTTTGGAGTGTCTCTGGCTTCCTTTG
TCCTTTTGATAGATTCTTTATGGAACCACGCCAGGCAGAGGAAGGGCACAGGTTCTGGGGATCTTATCACAAAGGCCCAT
GCGCAAGCCATGAAGTCTGTGATTTCATTCCTACTCTTCTTCTTTATGTACTACTCGAGCAATGTTATAATATATTTGGC
CTATATCAGTCTAGACAGTTTGGTGGCAAAAAAAGTTTGCTAATATGTTAGTATTTTCCTATCCATCTGGCCATCCATTT
CTTCTGATTTTATGGAACAGCAAATTGAAACAGCTTCTCTCTGTGTCCTGAGGAAGCTGAGGTGGTGCAGGAATCTAAGG
AAACCCACATACCCATAAAACATGCCTGA
>LeweTAS2R16P_NW_006383099.1:1469073-1469523
ATGCCCCTCCAACTCCTTCATAATCATCTATGTGCTCGAATCCTTGACAGTAACTATACAGAGCAACTTAATTTTTGCAG
TGCTGGGCAGGGAGGCTGTCATTGATGGACTTGATTCTTACCTGCCTGGGCATCTGCTGCTTCTGTCTATAGTGGGTGTC
GGTGCTGAACAATTTTTGCTCCTATTTTAACCCTAACTATGTATTTGGGTACTTATTAATCACCTGGGAATTTACTAATA
CTCTTACTTACTGGTTAACCAGCTTACTTGCTGTCTTCCACTGGGTCAGAGTCTCCTCCTTTACCTGCCCCATCCTCCTC
TGGCCAAGGTAGAGAATTTTGAGGTTTGTTCCTTGGCTGTTGCTGGATTCTCTGTTGAATTCTTGTGTGTCAATTGCCTT
TTCAGCTATTAGGAATTATATGAATATTCACTTAATCACCATGGGGCATTT
>LeweTAS2R38D_NW_006385316.1:89124-88123
ATATTGACCCTGACTCCTGTCATAACTGTGTCCTATGAAGTCAAGGGTGCATTTCTATTCCTTTCAGTCCTGGAGTTTGC
AGTGGGGATCCTGACAAATGCCTTCATTTTCTTTATGAATTTTTGGGATGTGGTGAGGAGGCAGCCACTGAGCAACTGTG
ATCTTATCCCTCTGAGTCTCAGCCTCACCCAGCTTTTCCTGCATGCGCTGCTGTTTCTGGATGCCATCCAGCTTATATAC
TTCCAGCGGATGAAGGACCCACTGAGCCTCAGCTACCAGACCATCATCATGCTCTGGATGATCACAAACCAAGCTGGGCT
CTGGCTCACCACCTGTCTCAGTCTTCTCTACTGCTCCAAGATTATCTGTTTCTCTCACACCATCCTGCTCTGCTTGGCAA
GCTGGGTCTCCAGGAAGGTCCCCCAGATGCTCCTGGGTGCCATGCTTTTCTCTTCCATCTGCACTCTCCTCTGTTTGGGG
GACTTTTTTAGTAAATCTGGCTTTGCATTCACAACTATGCTATTCATGAATAATACAGAACTCAATCTGCAAATTGCAAA
ACTCAATTTCTATCATTCCTTCATCTTCTGCACCCTGGAGTCCATTCCTCCTTTCTTACTTTTTCTGGTTTCTTCTGGGG
TGCTGACTGTCTCTCTGAGGAGGCACACGAGGATAATGAAAGCCAAAACCAAGGACTCCCGTGACCCCAGCCTGGAGGCC
CATATCAAAGTACTCAGATCTCTTGTCTCCTCTCTCTGCTTCTATGTGGTGTCATTCTGTGCTGCCCTCATTTCAGTGCC
TTTCCTGATGCTGTGGCACAAGATCAGGGTAATGATCTGTGTAGGGATCCTAGCAGCTTGTCCCTCAATACATGCAGCAA

TCCTGATCTCAAGCAATGCCAAGCTAAAGAGAGCTGTGGAGACCATTCTACTCTGGGTTCAGAGCAGCCTAAAGGTAAGG
GCAGATCACAGGGAGGATCCCAGAATTCCAGATCTATGTTGA
>LeweTAS2R39P_NW_006386364.1:108746-109709
ATGACCGAAACCTGCAATCCCCCAGAAAATGATTTGTCACCATTTCACATCCTCTGGATTTTAATAATTATAGGCACTGA
ATGCATCGTTGGTATCATTGCAAATGGGTTCATTATGGCTATAAATGCAGCTGAATGGATTAAAAATAAGGCAGTTTCCA
CAAGTGGCAGAATCCTGTTTTTCTTGAGTGTATCCAGAATAGCTCTCCAAAGCTTCATGATGCTAGAGATTACCTTCAGC
TCAACATTCCCACGTTTTTATAATGAAGACCATATATATGACATGTTCAAAGTAAGTTTCATGTTCTTAAATCATTGTAG
CCTCTGGTTTGCTGCCTGGCTGTTTCTTCTACTTCGTGAAGATTGCTGATTTCTCCCACCCCCTTTTCCTCAAGCTGAAG
TGGAGAATTTCCAGATGGATGCCCTGGCTTCTGTGGCTTTCGGTGTTTATTTCCTTGGGCTACAGTGGGCTCTTCTCTAA
AGACATCTACACTGTGTATTGTAACAATTCTTTTATCCCCTCCTCCAACTCCACTAAGAAAAAATACTTCACTGAGACCA
ATATGGTCAACCTGGTTCTTCTCTATAACCTGGGGATCTTCATTCCTCTGCTCATGTTCATCCTTTCAGCTACCCTGCTG
ATCATCTCCCTCAAGAGACACACCCTCCATATGGAGAGCAAAGCCACTGGTTCCAGGGACCCCAGCATGGAGGCTCACAT
GGGGGCCATCAAAGCTACCAGCTGCTTTCTCGTTCTCTACATTTTCAATGCAGTTGCTCTATTTCTCTATATGTCCAACA
CCTTTGATGTCAACAGTTCCTGGAATATTGTGTGCAGATTCATCATGGCTGCCTACCTTGCTGGACACTCCATTCTACTG
ATTCAGGACAACCCTGGGTTGAGAAGAGCTTGGAAGCAGCTTCAGCCTCAAGTTCATCTTTACCTAAAAGAGCAGACTCC
ATGA
>LeweTAS2R40P_NW_006386364.1:132921-133888
ATGGCCACGGTGAGCACAGATGCCACGGACAGAGACACATGCAGGTTTAAAATCGTCCTCACCTTGGTGGCCTCTGGAAT
AGAGTGCATCACTGGCATCGTTGGGAATGGCTTCATCATGGCCATCCATGGGGCCGAGCGGGCCAGAGGCAAAAGACTCC
CTGTCAGTGACTGCATTCTGCTGATGCTCAGCCTTTCCAGGCTCTTGCTGCAGATCTGGATGATGCTGGAGAATACTTAC
AGTCTACTGTTCCGGGTCACTTATAACCAAAACACAGTGTTTATACTCTTCAAAGTCGTCATCATGTTTCTGAACTATTT
CAACCTCTGGCTTGAGCTGCCTGGCTCAACATCTTCTATTGTCTTAGAATTGCAAACTTTGCTCACCCTTTGTTCTTCTT
GATGAAGAGGAAAATCACAGCGCTGATGCCTTGGCTTCTGGGACTGTCACTGTTCATCTCCTTATGCTTCAGCTTTCCCT
TCTCTACAGATATCTTCAATGTATATGTAAATAGTTCCATTCCCATCCCCTGCTCCAACACCCTGGAGAAGTACTTCTCT
GAGACCAATGTGGTCAACCTGGTTCTTCTCTATAACCTGGGGATCTTCATTCCTCTGCTCATGTTCATCCTTTCAGCCAC
CCTGCTGATCATCTCTCTCAAGAGACACACCCTCCACATGGAGAGCAATGCCACTGGTTCCAGGGACCCCAGCATGGAGG
CTCACATGGGGGCCATCAAAGCTACCAGCTGCTTTCTCATTCTCTACATTTTCAATGCAGTTGCTCTATTTATTTCCATG
TCCAACATCTTCGACATCAACAGTTCCTGGAATATTGTGTGCAAAATCGTCATGGCCGCTTACCCGGCTGGCCACTCAGC
GCTGATCTTGGGGAACCCTGGGCTGAGAAGAGCATGGAAGAGGTTTCAGCACCATGTTCATCTTCACCTGTAAGAGCAGA
CTCCATGA
>LeweTAS2R42P_NW_006383381.1:1831883-1831011
ATGTTAGCTGGATTGGATATAATCTTTCCTACACTGTCGACAGCAGAATTCATAATTGGAATGTTGGGGAATGTGTTTAT
TGGACTGGTAAACTGCTCTGAATGGGTCAAGAACCAAAAAATCTCTTTAGCTGACTTCATCCTCACCTGCTTGGCTATCT
CCAGAATCACTCAGCTATTGGTGTCATTGTTTGAATCATTTATGATGGGATTAAATCCACCTTTCTATTCCATTTATAAA
CTAGCAAAACCTGTTACTTTGCTTTGGAGAATAACTAACTATTTGGCCATCTGGTTTACTACCTGCCTAAGGATTTTCTA
CCTCCTTAAGATAGCTCAGTTCTCCTATTCCCTTTTCCTCTGGCTGAGGTGGAGAATGAACAGAGTGGTTCTTGCAATTC
TTGTATTTGCTTTGTTCTTTCTACTGTTTGACTTTCTATTGCTAGAAACATTTAATGACCAGGATCATGACCGGAGCCGA
AGGCAGTCGCTTAACCAACTGAGCCACCCAGGCGCCCTTAGGGTTTACCTTTGATGTATTCTTTAGATTGTGTTAAAGAA
ACAGAAAGTTGTAATTAAATGATGATAAGTGTGTGGGCTGTAAATTAATCTTGATGGGGCCATAGGTATGGGCTCTGCTC
TAGGGCTGTTCCCTGGAGCTGAGAAGGATGTTAGAATTATTAACATTTTCCCCGTCCTCTCTCACTGTATGTAGAAAACT
AAACTGCAAACACATCTAGGCTATGTGGTGCTTCTGTGTATCAATAACAATAACGACGTGTATAATGGTTTTTTTTTTTT
TTTTAAAGATTTTATTTATTTATTTGACAGAGAGAGACATAGCGAGAGCAGGAACACAAGCAGGGGGAGTGGG
>LeweTAS2R60P_NW_006385740.1:142925-143543
ATGAATGGAGATGACATGGTTCCAGAACCTCCAGTGACTGACTGATAAGAGAGCCATCACCCCGGCTATCATTTCATTTC

ATTTTCTTTTTTTGTTCCTGGGGACAGCAGTGAGCAACGGCTTCTTCATCACTGTAACACTGAGCATGGAGTGGTTGCTA
CAGTGAACACTGTTAACCTTGTGATAAATTATTAGTCAGCCTGGGGGCCTCTTGCCTCTGTCTGCAGTGGGTGGTGATGA
GGAAGAGCATTTATATTTCCTGTATCCAATGGCCTTCCCATACAACCCTGTACTGCAGTTCCTAGCCTTCCAGTGGGAC
TTACTGAACACTGCCACCTTATGGTTCTTCACCTAGCTCAGCGCTTTCTGTTGCGTGAAGACCGCAACCTACACCCTCCC
CATCTTCCTCTGGCTAAAACAGCAGGTGTCTGGGTTGGTTCCATGGATGCTGCTTAGTTCCTTGGGACTCTCCAGCTTGA
GCACCATCCTCTTTTCATAGGCAACCAGAGCTTATATTACTACTTTTTAAGGAGAGGGTTGCAATCTTGGAATGCCACTG
GGAATACTATAAGATCATATGAGAAATCCTACTTCTCTTTAAAACTTGTTACCTGGACA
>LeweTAS2R62P_NW_006385740.1:137522-138402
ATGCCCTCCTCACCTGCATTGATCTTCATGGTCATCTTCTTCCTGGAGTCGTTGGCTGCAGTGCTGCAGAATGGCTTCAT
AGTTACTGTGTTGGGCAGGGAGTGGGTGTGACGCCAGGTGCTGCCCGCAGGTGACATGATTATGGCCTTCCTGGCCGCCC
GGTTCTGCCTGGATGGGGTGGCCATCCTGAACAACCTCTTGACCTTCTTTGAAATGGACTATTACCACATCTCCTGGAAC
TTCATCAACACTCTTGCTTCCTGGCTCACTGCCTGGCTTGCCGTCTTCTACTGCGTGAAGATCACTGTCTTCTCCTACCC
TGTCTTCTTCTGGCTGAAGTGGAGGATTTCTCGGTCAGTGCCCAGGCTGCTGCTGGGCTCCCTGGTCTTCGCTGGTCTGA
CAGTCATCTCATCAGCCACTGGGACTAGAATTCTTATGGCTCCCCGAGTTCCCAAGGAAACAGCATCCTGGCTGATACAG
TACAGCCCTTCTATTGGTGTTTTATTCTACCTCGTGCAATGCTTACGTGGTCAGTCCCATTCCTCCTGTTCTTGGTGTCC
ATGCTCTTGCTCATGTTCTCACTGTGCCAGCACTTGGGGCAGATAAGGGACCATAGACTGGGCCCACGTGATCCTAGCAC
ACAGGCTCACACCATGGCCCTGAAGTCACTTGTCTTCTTCCTTGTCTTCTATACCTCATATTTCCTGTCTCTGATTGTTG
TTTCTATGAACATCACAGCCTGTCAGAGTCACTGGTACTGGGCCTGGGAAGTGGTGACCTATGCAGGCATCTGTCTGTAC
TCCAGCATCCTGGTGCTAAGCACCCCTAAGCTGAGAAAGGTCCTGAAGACCAGGCTTTGGAAAGCTCTGGACAAAGGCTG
>LeweTAS2R67P_NW_006383381.1:1824656-1823729
ATGCCATCTGGAATTAAAAATGCTTTTCTGATAGCAACAGGAGAATTCATAGCTGGAATGTTGGGGAACAGCTTCATTGT
ACTGGTTAACTGCATTGACTGGGTCAAGAGTCAAAAGCTCTCATCAGCTAACTGCATTCTCACCAGCCTGGCTATCTCCA
GAATTGTTCTTCTTTGCGTAACACTATTCAATTCATTTTTAACGGTGTTCTGGCCACATCTTTATGCCATTGATAAACTA
GCAAGATTCGTTAGCATTCTTTGGACACTGAGCAATCACCTAGCTACCTGGTTTGTCACCTAAGTGTTTTCTACTTCTTT
AAAATAGCCAATTTCTCCCACCCCCGTTTCATCTGGCTGAGGTGGAGAATTAACAGAGTGCTACTTGTGCTCCCAGTGGG
GTCTTTATTCTTATTGTCTTGCAACTTTGAATTGTTAGATACATTTACTAATTTCTGGGTTAATGTCTATCAAAGATATG
AAAGAAACTCAACTTGGTCCCTAGATGTAAGTAAAACTCTGTATCTTAACAGCTTGATTGTTTTCAGTTTTATCTACTTA
ATCCCCTTTCTTCTGTCCCTGACCTCACTGCTCCTTTTCTTTCCCTGAGGAGACATACCAGGAATGTGCAACTGAACTCC
GGCTCTAGGGACTTCAGCAGAGAGGCCCATAAAAAGGCCATGAAAATGGTGATGTCTTTCCTTCTCCTCTCCACGGTTCA
TTTTCTTCCATCCTATTAACAGGTTGGATTTTCCTTTTACTGCAGAATTGTCAGGTCAATTTGGTTGTCATGTTATTAA
CAACACTTTTTCCTCCAGGCCACTCATTTATTCTGATTTTGGGAAACAGCAAGTTGAGAAAAACTGCTTTAGGACTACTG
TGGCATCTTAATCACCACTTGAAAATGGTGAAATCTTTAGCTTCATAG
>LeweTAS2R7AT_NW_006396373.1:1394-618
ATGCCGGATAAAGTGGAGACCACCTTAATGCTCATGGCAGCTGGAGAGTTTTCAATGGGGATTTTAGGAAATGCATTCAT
TGGATTGGTGAACTGCATGGGTTGGATCGAGAATAGGAAGATTGCCTCCGTTGATTTAATCCTCACAAGTCTGGCCATAT
CCAGAATTTGTCTATTATGTATAATACTATTAGATTGTTTTATATTGGTGCTGTATCCAGACGTGTATACTACCGGTAAA
CAAATGAGAATCATTGATTTTTTCTGGACACTAACCAACCATTTAAGTGTCTGGTTTGCCACCTGTCTCAGCATTTTCTA
TTTCCTCAAGATTGCGAATTTCTTCCATCCTCTTTTCCTCTGGATGAAGAGGAGAATTGACAGTGTGATTCCTAGGATCC
TGCTGGGGTGCTTGGTCCTCTCTGTGTTTGTTAGCCTTTTTGTCACTGAGAATTTGAATGATGATTTCAGGTATTGTGTT
AAGACAAAGAAGAAAACAAACTTAACTGTGAGATGCAGAGTAAATAAAGCTCAATATGCTTCTATCAAGATTTGTGTCAA
CCTGTTAACGCTATTCCCCTTTTCTGTGTCCCTGATCTCATTTCTCCTCTTGATCCTCTCCCTCTGGAGACATACCAGGC
AGATGAAACTCAATGCCACAGGGTGCAGAGACTTCAGCATAGAAGCCCACGTGGGAGCCATGAAAGCTGTCATCTCCTTT
CTCCTCCTTTTCATTGCCTACTGTTTGGCCTTTCTCGTAGCCACCTCTAGCTACTTT
>LeweTAS2R7BT_NW_006383381.1:1701481-1701303

CCACCTCTAGCTACTTTATGCCAGAGACTGAATTAGCTGTGATCACTGGTGAGTTGATAGCTTTAATCTATCCCTCAAGC
CATTCATTTACCTTAATTCTGGGGAGCAATAAATTAAGACAGGCATCCCTAAGGGTGCTATGGAAAGTAAAGTATATCCT
AAACAGGAGAAATTTCTAA
>LeweTAS2R9T_NW_006383381.1:1718793-1718011
TCCCGAGATAGATATGCCGGTAGCAAGCTAATGAGCATTGTGGATGTTTTCTGGACACTTAGCAATCATTCAAGTGTCTG
GTTTACTTCTTGCCTCAGCATGTTCTATTTACTGAAGATAGCCAATATATCCCACCCATTTTTCCTCTGGCTGAAACTAA
AGATTAACAGAGTCGTCCTGGGGATTTTTCTGATGTCCTTCCTTACCTGTATAATTATTAGTGTTTCATTGAATGAGGAC
TTCTGGGATCCCTTCAAAGGCAATCATAAGGAAAACATAACTTGGGAATTCGAAGTGAGTAAAATCCCAAGTGCTTTCAA
ACTGGTTATCCTGAACCTGGAGGCTATCATTCCCCTTGTTCTTTGCCTAACCTCATTTCTCTTGTTACTTTTCTCCCTAT
TTAAACATACCAAGCAGATGAAGCTTAATGCCACAGGGTCCAGAGACCCCAGCACAGAGGCCCACATGAGGGACATAAAG
GCAGTGATCATCTTTCTGCTTCTCTTCATTATGTACTATGCAGTCTTTCTTGTAGTAACCTCTAGCTTGCTGATTCCTCA
GGGAAAATTAGTGGTGATGTTTGGTGGCATGATCGCTGTCATTTTCCCATCAAGCCATTTGTTCATTCTGATAATGAGGA
ACAGCAAACTGAGGGAGGCTTTTCTGAAAGTGCTAAGGATTGTGAAGAGTTTCCACAGAAGAAGGGAACCTTTCGTTCCA
CAGAGAATCCTGAATACAAGGAGAAAGAAATCAACAAAAGACCTTCTCCCTTCTCCCAATTGA
>LeweTAS2R41T_NW_006385740.1:168341-168827
ATGCAGCCAGCACTCTCAGCCTTCTTCATGCTGCTCTTTGTCCTGCTGTGTCTCCTGGGAATCCTGGCCAACGGCTTCAT
TGTGCTGGTGCTGAGCAGAGAAAGGATGCGGCGTGGGAGGCTGCTTCCCTCCGACATGATCCTCATTAGCTTGGGTGCCT
CCCACTTCTGCCTGCAGTGGGTTGGAATGGTGAACAACTTCTACTACTTCCTCCACCTGAACGAGTACAGCCAAGGTCCT
GCCTGGCAACTCACTGGTCTCCACTGGGACTTCCTGAACTCGGCCACCTTCTGGCTTGGCTCTTGGCTCAGTGTCCCCTT
CTGCATGAAGATTGCCAACTTCACCCACCCCACCTTCCTCTGGCTGAGGTGGAGGTTCCCAGGGTCAGTGCCCTGGCTCC
TCATGGCTTCTCTCCTGATCTCTTTCATCGTCACCCTGCTCTTCTGGGGAAACCATGCTGTGTATCAAGGATTCTTAATT
AGAAAATNNNNTTCCTTGGAACATGACCTTCAAGCAGTGGAGCCGGAGGCTGGAAATTCACTCTTTCTTGCCCCTGAAAC
TTACCACTTGTCAGTTCCCTGCTCTGCCTTTCTGTTCTCAATTGCACTGTTGATTAATTCTCTGAGGCGACACACAGGGA
GGATGCAGCGCAGCACCCACAGCCCGCAGGACCCCAGCGGCCAGGCTCATACCAGAGCTCTGAAGTCACTCGTCTCCTTC
CTCATTCTTCATGCTCTGTCCTTCGCATCCCTGGTCATCGATGCTGCGGGTTTCTTCTCCTCAGAGAGTGACTGGTACTG
GCCGTGGCAGATTTTAATCTACCTGTGCACATCTGTCCATCCCTACATCCTCATCCTCAGCATCCTCCGGCTTCAAGGGG
TGTGCGGTCAGCTACTTCTGTTGGCCAGGGGCTTCTGGCTGGCCTAG
>OvarTAS2R1_chr16:63380301-63381191
ATGCTGGAGTGTCACCTTGTCAGCCACCTTGTTTTGACAGTGATACAATCTCTCTTTGGGATTTTAGTAAATGGCATCAT
TTTGATTGTGAACGGTACTGACTTGATCAAGCAGAGAAAGTTGATTCCACTGGATCTCCTTGTTTCCTGCTTGGCGATTT
CCAGGATGGGAATTCAGCTGGCCTTCTTCTACATTAACCTGGCTCTTCTTTCCTTGGTCAAATTCCCTCAGGTTACTGAG
AAGCTTGTAGTTTTCACATTTGTAAATGATTTGGGACTTTGGTTTGCCACCTGGCTCAGTGTCTACTACTGCACCAAGAT
TGCTACCATCGCTCACCCTCTCTTATTCTGGTTGAAGATGAAGATCTCCAAGCTGGTTCCTTGGCTGATTCTTGGGTCCC
TGCTGTATGCATGTAGTACTTCTGCTGTGCATGTCAAATATAAGTGGGCATTTTATGGAGAAGGCTTCCTGGACCTTTTC
TTCCCAAATGTAACAACTCACATCAAAGTAACCCCTACTTTACAGTCTGCCTTTCTGCTTGCTGAGTTTGCATTGCCGTT
TTTCATCTTCCTGATTTCTTCTCTGCTCTTGATATTTTCCTTGGGGAGACACACCTGGCAGGTGAGAAACACATGGACAG
GCCCCAGAAACTCTCACACACGTGCATACATCAGGGCCTTTCACTCCATCCTGTCCTTCCTGGCCCTCTATCTCTGCCAC
TACCTGATCGTTGCTTTGATCTTTTTTCAAATTTTTAACCTTAGAAGCTTCCTATTTCTGTTCTGCACCTTCATGGTTGG
TTCATACCACTCCATCCACTCTATTACTTTAATTTTAGGAAACCCAAAAATGAAACAAAATGCAAAGGCATTGCTCCTTC
TCAGAAAGTGA
>OvarTAS2R2_chr4:20568440-20569348
ATGATCTCTTTGTCAGTTATTCCACATGTTATCATCATGTCTGCAGAATTTATTACAGGGGTTACAGTAAATGGATTTCT
TATAATCATCAACTGTAATGAATTGGTCAAAAGCAGAAAGCTAACACCCATGCAACTCCTGTTCGTATGTATCGGGATGT
CTAGATTTGGTCTACAGACTGTGTTAATGGTACAAGGTTTTTTCTCAGTGTTCTTTCCACGCTTTTATAGCACAAAAATC

TATGGCACACCAATGCTGCTTTTTTGGATGTTTTTCAGCTCTGTCAGTCTCTGGTTTGCCACCTGTCTCTCTTTATTTTA
CTGCCTCAAGGTAACAGGCTTCACCCAGTGCTGTTTTCTTTGGCTGAAAGTCAGGATCTCAAAGTTAATGCCTTGGATGC
TCCTGGGAAGCCTGCTGACCTCTATGAACATTGCAGCACTGTGTGTCAAGGTGGATTACCCTAAAATTGTGGATATTGAT
GTCCTCGGGAATGTCACAGCTAAGAGGATTAAACTCAACACAAAGCAAATTAATGAAGTTCTTCTCGTCAACTTGGCATT
ACTATTTCCTCTGACCATATTTATAATATGCACTGTTATATTATTCATTTCTCTCTACAAGCACACTCATCGGATGCAAA
ATGGACCTCCTGGTTTCAGAAACACCAGGACTGAAGCCCATATTAATGCATTAAGAACAGTGATAACATTCTTTTGCTTC
TTTATTTCTTACTTTTGTGCCTTCATGGCAAATATGACATTCAATATTCCTTATGGAAGTCATTGCTTCTTTGTGGTGAA
GGATATTATGGCAGCATATCCCTCTGGTCATTCGGTTATAATGATCTGCAGTAATTCCAAGTTCCAGCAACCAATCAGGA
GACTTCTCTGCCTAAGAAGGAGTCAGTGA
>OvarTAS2R3_chr4:104791789-104792739
ATGTTGAGACTCAGCAATTTGGGGTTTCTGGTTCTGACCGCCATTCAGTTCATCCTGGGAATGCTGGGGAATGGTTTCAT
AGGGTGGGTCAATGGCAGCAGCTGGTTCAAGAGCAAGAGGATCTCTTTGCATGACTTCATTATCACTAACCTGGCTGTCT
CCAGGATTGTTTTGCTGTGGATTCTCTTGATCGATGGTGTTTTACTGGTGTTCTCTCCCAAACTACATGATGAAGGGATA
ATCATGCAAATTATTGATGTGTTCTGGACATTTACAAACCATCTGAGCATTTGGCTTACCACCTGTGTCAGTGTCTTCTA
CTGCCTGAAAGTGGCCAGTTTCTCCCATCCTATGTTCCTCTGGCTCAAATGGAGAGTTTCCAGGGTGGTTGTATGGATGC
TGTTGAGTACCCTGCTGTTATCATGTGGCAGTGCCATCTCTCTGATCCGAGAATTTAAGATCTATTCTGTTCTCGGTGGA
ATTGATAGAACCGGGAATATGACTGAGCTCTTTAGAAGGAAGGAAAAAGAATATAAACTGATCCATGTTCTTGGGACTCT
GTGGGACCTCCCTCCCCTAGTCGTATCTCTGATCTCCTACTTTCTGCTTATCCTCTCCCTGGGGAGGCACGTGCGGCAGA
TGCATCAAGACTGTGGCAGCTCCAGAGATCCCAGTACCGAGGCCCACAGGAGGGCCATCAGAGTCATCCTCTCCTTCCTC
TTCCTCTTCCTACTCTACTATCTTTCCTTTTCTGTTTTAACATCCAGTTATTTCCTACCAGCAACTAAGATGATTGCGAA
GATCGGAGAAGTAATTACAATGTTCTATCTTGCTGGCCACTCCTATGTTCTCATTCTGGGAAATAGCAAGCTGAAGCAGA
TGTTTGTGGCGATGCTCCGGTGTGAGCCTGGTTGTCTGAAGCCTGGATCCAAGGGATCTGTTTATCCATAG
>OvarTAS2R4_chr4:104805255-104806145
ATGCTTCGGATAGTCTTTTTTTCTTCTATCGTTGTCTCTGAAATTATAACTTTTGTAGGACTCATTGTGAATCTCTTCAT
TGTAGTGGTCAGTTACAAGACTTGCATCAAAAGCCACAGGATCTCTTCTTCTGACAGACTCCTGTTCAGTTTGGGCATCA
CCAGATTTTTTATACTGTTACTGAATGTTGTTGTCATCATCTCTCCAAATGTGGAAAGGTCAGTCTCCTTATCCTCTTTC
TTCCTGTCATGTTGGATGTTTTTGGACTCTAGTAGTCTTTGGTTTGTAACCTTGCTCAACGTCTTGTATTGTGTGAAGAT
TGCTAACTACCAACACTCCGTGTTTCTCCTGCTGAAACGAAATCTCTCCACCAGGATGCCCCGGCTGCTGCTGGTCTGTA
TGCTCCTTTCTGTCTTCACCACTCTCCTGTATGTTATGCTCAGACAGTTGGCACCCCGTCTTGAATTTGTGACTGTGAGA
AATGGCACAGTATTTGACATCAATGAGGGACTCCTGTCTTTGGTGACTCCTTTGGTCTTGAGCTCATTTCTCCAATTCAT
CATTAATGTGACTTCTGCTTCTTTGTTGATCAATTCCTTGAAGAGACATGTACGGAAGATGCAGAGAAGCGCCACTGTTC
TTTGGAATCCCCAGACTGAAGCTCATGTGGGTGCTATGAAGCTGATGATCTGTTTCCTCATACTCTACATTCCATATTCA
GTTGCTACCTTGCTCCATTATCTCCCTTCTTCTATAGGGATGGATTTGAGAACCAAGTCTATTTATGTTATTATGTCCAC
CATTTACCCTCCAGGACATTCTCTTCTTATTATTCTCACACATCCTAAACTGAAAACAAAAGCAAAGAATATTCTTTGTT
TCAGTAAATAG
>OvarTAS2R7_chr3:203666621-203667549
ATGTCAAGTGAAGTGCAGGGTATCTTAATGCTCATAGCAGCTGGGGAATTTTCACTGGGGATCTTAGGGAACGCATTCAT
TGGATTGGTAAACTGTGTGGACTGGATCAAGCACAAGAAGATTGCCTCCATTGATTTAATCCTCACAAGCTTGGCCATCT
CCAGAATTTCTCTCTTATGTATAATACTATTGGATTGTAATATATTGGTCCTGTACCCAGATGTCTATACTGGTGGTAAA
CAAATGAGAATCATTGACTACTTCTGGACATTAACCAACCATTTAAGTGTCTGGTTTGCCACCTGCCTCAGCATTTTCTA
TTTCCTCAAGATAGCAAATTTCTTCCATCCATTTTTCCTCTGGATGAAGTGGAGACTTGACAGTGCAACTCCTAGGATCC
TGCTGGGGTGTTTGGTCTTCTCGGTGTTCATTAGCCTTCCTGTCATTAACAATTTGGATGATGATTTCAGGCATTGTGTC
AAGATGAAGTTGAAAACAAATATAAGTCGGAGATGCAGAGTACATAAAGCTCAGCATGCTTCCATCAAGATACGTCTCAA
TCTGTTGACACTATTTCCCTTTTCTGTGTCTCTGGTTTCATTTCTCCTCCTGATCCTCTCCCTGTTCAGACACACCAGAC

GAATGCAGCTCCGTGCCCCGGGGAGCAGAGATCCCAGCACGGAAGCTCACGTGAGAGCCATGAAGGCTGTCATCTCCTTC
CTCCTCCTTTTCATTGCCTACTACTTGGCCTATCTTGTGGCCACGTCCAGCTACTTTATGCCAGAGACTGAATTAGCTGT
GATCGTTGGTGAGTTGATAGCTTTAATCTGTCCATCAAGCCATTCACTCTTCCTAATTCTAGAGAACAAAAAATTAAGAC
AAGCATCTCTAAGGGTGCTATGGAAGGTAAAATATATCCTACAAAGAAGGAATTGCTAA
>OvarTAS2R10B_chr3:203620374-203621303
ATGCTGAGTACAGTGGAAGGCCTCCTAATTTATGTAGCAGTTAGTGAATCAGTATTGGGGGTCTTAGGGAATGGATTTAT
TGGAGTTGTAAGCTGCATTGATTGTGTGAAAAGCAAGAAGATCCCTACTGTCAGCCTTATTCTCACTGGCTTAGCTTCTT
CCAGATTTTGCCTGATATGGATAATAATTACAGATGCATATGTGAGGATGTTTTTTCCAGATACATATTTGTCTGGTAAT
CTAAGTCAAAATATAGCTCACTTTTGGATAATTATGAATCAGTCAAGTATCTGGTTTGCCACCAGCCTCAACATCTTCTA
TTTCCTGAAGATAGCCAATTATTCCCACTGCATTTTTCTCTGGCTGAAGGGTCACATCAACAGGGTTCTTCTCCTTTTCA
TGGGGTCTTTGCTTATTTCATGGTTATTTGCTTTTCCAAGCATTGCAAAGCCTAGTATTAATGATATTATGAAGAACAGA
AGCTCAACCTGGCTGATCGCCCTGCATAAAAGGGAATACTTGACAAATCATATTCTGCTCAATATTGGAGTCATTCTTGT
CTTTGTGCTATGCCTGATTACATGTTTCTTATTAATCACTTCCCTTTGGAGACACAACAGAAAGATGCAATTGAATGCCA
CAGGATTCAGAGATCCCAGCACTGAAGCACATATCAAAGCAATGAAGACTTTGGTGTCTTTTATCATCCTCTTTATCTTG
TATTTTGTAGGCACTGCCATACAAATATCAGGTAGTACTATGCCTGAAAACAACCTGTTGCTCATTATTGGTATAACAAC
CAGACTCCTCTATCCCTGTGGACACTCGTTGATCCTAATTCTAGGAAACAGGAAGCTGAAGCAAGACTTTTTGAGGGTAC
TGAAGCCATTAAAGTGCTGGGGAAAAGAGAAACTTCTTAGAATTCCATGA
>OvarTAS2R11_chr3:203589330-203590274
ATGTTGAATATATTGGAGAAAGTTTTCATGGTTGTGACTGGTGTGGAATTTATAATAGGAATTTTAGGGAATGGATTTAT
TGGACTCACAATTTGCATTGCTTGGATTAGAAATCAGAAGTTGAGCTTGGTTGACTTCATTCTTACTAGTTTGGCCTTTG
CCAGAATCAGTCAATTATGGATAACCACTGTCATGTTTTTTTCAGTGATGTTCTATCAGGCAGGCTTTGGTACTGTGGGA
AGAAAATATATATTTTTTTGTATCTGGATACTGGCCAGTCACTCAAGCACTTGGCTTGCTACTTGCCTTGCTGTCTTTTA
TTTCCTGAAGATCGCCAGTTTCTCCCATCCTCTTTTCCTTTGGCTAAAATGGAGAATTAACAAGGTTGTTTTTATGTTTC
CACTGTTATCTGTGCCCTTCCTAGTCATAAGTTTTCCTTGGCCATATACTGTTGATGTCTTCTGGTGTTATGTCCAAAAG
ATGCATGAGAGAAATATGACTGAATTATGCAATGTGAATGAATATAAAAATTTAAATTTTATTATTATCTACACTGTGGT
GTCCCTCCCACCCTTCTTCCTTTCCCTGATTTCCTTTCTCCTGTTGCTCCATTCTTTGTGGAAACACAAGAAGAACATTG
CACACACTGTCAGGGATTCCAGAGACCCCCATGTGGAGGCCCATTTCAGAGCCATGAAAACTGTGTTTTTCTTTCTCATG
CTCTTTGTCCTGTACCAATTTGGCCTTTTCATGACATTTGGGGGACATGTTTTCCTACAGAACAAGCTGGTTGTGATGTT
TGGTTATATGTTAGAAATGCTGTATCCTTCAAGTCATTCATATGTTTTAATTTTTGGAAACAGCCAAATGAGGAAATTCT
TCTTGGTGATTCTTAGGCACCTGAAGTGTGGCCTGAAAGGAAGGCACTGTTGGCTGCGTAGGTAG
>OvarAST2R12_chr3:203576199-203577110
ATGGAGAGAACACTGAACAATATACTTATGATCATTTCTGCTGGAGAATTCTTACTGGGTATTTTGGGAAATGGATTCAT
TGTCCTGGTTAACTGTATTGATTGGATCAGGAGCAGGAAGTTCTCCCTGATTGACTTTATTCTCACCTGCTTGGCTATTT
CCAGAATATTTGTGCTGTGCATAATGATTTCAAGTACAGGTTTATATGTAATCTCTGAGGAAATACAGTACAACAAGAAT
CTCCTGATAAATTTGGGGTTCCTCTGGACAGGATCCAATTATTTCTCCATAGCCTGCACCACCTGCATCAGTGTCTTCTA
TCTCCTCAGAATAGCTAACTTTTCTAATTTCCTTTTCCTCTGGATGAAACGGAGAATTCACAAGGTGCTTCTCATTATTG
CACTGGGGGCTGTCTTCTCTTTCTGCTTGTGCCTTCTTCAAAAGAATATGGCAGTTGAAATCCTGTTCCAAAACCAGGTA
AACAGCAAAAAAAATGTGACATTGGACTTTCTAATGATAAGATACGATTTGTTCTTTGCCATAATGTTCCTCATCCCCTT
TGTAGTGTCCCTGGCCTCCTTTCTCCTTTTAATCCTCTCCTTATGTGGTCATCTCAGGCGTATGAAGGGTGTAGACTGTA
GCTCGGAAGCCCATGTGAGAGCCCTGAAGGCTATGATTTCATTCCTACTCCTCTTCATTCTACACTATTTGAGCAATATT
ATGACAGTGTGGGCCAATCACATTCTCGGTAGTTTTGTGGCAAAGATTTTTGTGAACATGCTGTTATTTTTCTGTCCTTC
TGGCCACCCTTTGCTTCTGATTTTGTGGAACAGCAAATTGAAAAAGGCTTCACTCTGTGTCCTAAGGAAGCTGAGGGGTT
ACATGAATCTAAGAAAACTCTTCCAAAAATAA
>OvarTAS2R16_chr4:87416330-87415425

ATGATAACCAGCCAACTCTCTGTCTTCTTCATGCTCATCTATATGCTCGAGTTCTTGACAATAACTGGGCAGAGCAGCCT
GATTGTCATAGTGCTGGGCAGAGAGTGGGTGCAGACTCAAAGGCTGCCGCCTGTGGACATGATTCTCACCAGCCTGGGCA
TCTGCCGCTTCTGTCAACTGTGGTCATCGATGCTGCACAACTTCGGCTCCCACTTCCACCTGAATTACAATTTTTGGTGT
TTTGGGATCATCTGGCAGTTTACTAACATCCTTTCCTTCTGGTTAACCAGCTTGCTTGCTGTCTTCTACTGTGTCAAAGT
CTCCTTCTTCAGCCACCCCATCTTCCTCTGGATGAAGTGGAGAATTGTGAGATGGGTTCCTCAGCTGCTGCTGGGCTCTC
TGCTGGTTTCTTGTGTGTCTACCATCTTTTCAGCGACTAGTTATTACATCATCATTCAATTCATCTCCATGAAGGATTTC
CCTAGGAACAGCACCATGCTTGAGAGACTGGAGGCGTTCCTGTGGGATTTTTCCACACTGCGGAAAGTGGTTGTATTGGT
TATTCCTTTCCTCCTGTTCCTGGCCTCCACAGTCTTGCTCATGGCCTTATTATCCCGACATCTGAAGCAGATGAAAGACC
TTCACACAGGCCGCCCCATCTCCAGCCGGGAAGCTCACTCTGCCGCCCTGAGGTCTCTTGGCATCTTCCTCATCTTGTTC
ACCTTTTATTTCTGACCGTGCTCGTCTCCATCTTGGATGTCCTATTTAATAAAGAGTCTTGGTTCTGGGCCTGGGAAGC
TATCATCTATGCATTAGTCTCTATTCATTCTACTTTACTAATGCTGAGCAGTGCCAAACTGAAAAGAGTTTTAAAGGCAA
GGTGCTGGAGCCTAGAAGCTGCCTGA
>OvarTAS2R38_chr4:104955978-104954977
ATGGTGACTCACATCGCATCTGTGCCCTCTGAAGTCAGGAACGCATTTCTGTTCTTTTCAGTCCTGGAGTTTGCAGTAGG
GATCCTGGTCAACGCCTTCATTTTCTTGGTGAATTTCCGGGACCTGGTGAGGAGGCAGCCACTGAGCCACTGTGATCTTG
TCCTGTTGAGTCTCAGCCTCACCCGGCTTGTCCTGCACGGGCTGCTCTTTCTGAAGGCCATCCAGCTTACTCATTTCCAG
CGAATAAGAGACCCACTGAGCTTCAGCTACCAGACCATCATCGTGCTCTGGATGATCGTCCACCAAGCCGGCCTCTGGCT
CACCACGTGCCTTAGTCTCCTTTACTGCTCCAAGATTGTCCATTTCTCTCACGCCTTCCTGCTCCGTGCAGCAAGCTGGA
TCTCCAGAAAGATCCCCCAGATGCTTCTGGGTGCTGTGGTTCTCTCCTGTGTCTGCACTCTTCTCTGCTTATGGGACTTT
TTTAGTGGATCTCGTTTCTCAGCTGTAACTAGGCTACTGACGAATAACAGTACTGAACTCAATTTGAACATTGCAAAACT
CAGTTTCTTTCATTCCTTCCTCTTCTGCAGCCTGGCGTTCATCCCTTCTTTCTTGCTTTTCCTGGTTTCCTCTGGGATGC
TGGTGTTCTCCCTGGGGAGGCACATGAGGATGATGAGGGCTGAAACCAGAGGCTCCCGGGACCCCAGCCTGGAGGCTCAC
ACCCGGGCGCTCAGGTCTCTCGTCTCTTTCTTCTGCCTGTATGTGCTGTCACTCTCCGCTGCCTTAGTCTCGATGCCGTT
GCTGACGCTGTGGCACAGCAAGGTTGGGGTGATGGTCTGCATAGGGATAATGGCAGCCTGTCCCTCGGGACATGCAGTCA
TCCTGATCTCAGGGAATGCCAAGCTGAGGAGGGCTGTGGACTCCATTCTGCTTTGGGCAAAGAGCAGCTTCAGGGTAAGG
ATGGACCACAAGGCAGATCCCAGGACACCAGATCTGTGTTGA
>OvarTAS2R39_chr4:106006335-106007381
ATGACAAGTGGGAGCTATCACAGACCCGCACATCAAGCGCTAAGGAGCCGTTTTCCTCCAGACATCAAAGAAGAGCAACC
ACTCAGGATGATCCAAACCTGCAGTTCCTCAGAAAAGGATCTGTCACCATCTCTTGTCACTTTGATGTTAATAATTATCG
GCACGGAATGCATCCTTGGTATTCTCGCAAATGGGTTCATTGCAGCGATAAACACAGCTGAATGGATTCACAATAAGGTA
CTCTCCACCAGTGGCAAGATCTTGCTTTTCCTGGGTGTATCCAGAATAGTTCTACAAAGCTTCATGATGCTAGAACTTAC
CTTAAGCTCAACATCCCCACAGTTTTATAATGACGACATCACGTATCACACATTCAGAGGATGTTTCATGTTCTTAAATC
ACTGCAGCCTCTGGTTTGCTGCCTGGCTCAGTGTCTTCTACTTCGTGAAGGTGGCGGATTTCTCCTACCCCCTTTTCCTC
AAGCTGAAGTGGAGAATTTCCAGACTGATGCCCTGGCTTCTGCAGCTTTCAGTGTTTGTTTCCTTGGGCCAGAGTGTGCT
CTTCTTCCAAAACATCTATACTATGAATTGTAACAATCTTTTTCTCTCCCCTCCTTCAACTCCACTAAGAAAAAGTCCT
TCTCGGAGGCCACTGTGATCAACCTGGTTCTTTTCCTTAACCTGGGGATCTTCATCCCTCTGATCATGTTTATCCTGGCA
GCCACCCTGCTGATCATCTCTCTCAAAAGACACATCTTCCACATGAAAAGCAATGCCACTGGCTCCAGAGATCCCAGCAC
GGAGGCTCACCTGGGGGCCATCAGAGCTATCAGCTACTTTCTCATTCTCTATATTTTCCAAGTACTTGCTCTTTTTCTCT
ACATGTCCAACTTCTTTGACATCAATAGTCCCTTGAATATTTTGTGCAAAATCATCATGGCTACCTACCCTGTGGCCCAT
TCCATTCTACTGATTCAGGACAACCCTGGGCTGAAAAGAGCCTGGAAGAGGCTTCAGGCTCAAGTCCACCTTTATTTTAA
AAAGTAG
>OvarTAS2R41_chr4:106311662-106312597
ATGCACCCAGAATTCACAGTCCTCTTCATGCTGCTCTTTGTCCTGCTGTGTATCCTGGGCCTCCTGGCCAATGGCTTCAT
TGTGCTGGTGCTGAGCAGAGAATGGGTGCGAGGTGGGAGGCTGCTCCCCTCTGACCTGATCCTCTTTAGCTTGGGACTCT

CCCGCTTCTGCCTGCAGTGGGTTGGAATGGGGAATAACTTCTACTATTTCCTGCATCTGGTCGACTACTGCAGTGGTCCC
GCCCGGCAGTTCTTCGGTCTACCCTGGGTCTTCCTCAACACCGTCACTTCCTGGTTTGGCTCCTGGCTCAGCGTCCTCTT
CTGCATGAAGATTGCTAACTTTACCCACCCCGCCTTCCTCTGGCTAAAGTGGAGGTTCCCCAGGTGGGTGCCCTGGCTTT
TGCTGGGCTCTCTGCTCACCTCCTTCACTGTCACCCTGCTTTTTTTTTCAGGGAACCACGCTTTGTATAAAGGGTCCTTC
ACTAGAAAACCTTTCAGGAACATGACCTATCATCAATGGAGCAGGATTCTGGAAATGTACTATTTCCTGCCCCTGAAAAT
GATCACTCTTTCAGTTCCTGGCTCTGTTTTTCTGGCCTCGATTGCTCTGTTGATTCACTCTCTGAGGAGACACGCATGGA
GGATGCAGCGCAGTGGTCACAGCCTGCAGGATCCCGGTGGCCAGGCTCACACCAGAGCTCTGAAGTCACTAGTCTCCTTC
CTTGTTCTTTATATTCTGTCTTTCGTGTCCCTGATCGTTGATGCTGCAGGGTTCTGCTCCTCAGACAGTGACTGGTACTG
GCCATGGCAAATTTTAGTCTACTCGTGCACGTCCATCCATCCCTTTATCCTCATCCTTGGCAACCTCAGGCTTCGAGGGG
CATCTGGGCAGCTGATTTTGTTGGCCAGGGGCTTCTGGATGGCCGAGGTGGTGTGA
>OvarTAS2R60_chr4:106282857-106283810
ATGAGCGGAGAGGACGTGGTTCCAGGACCTCAGGTGGTTGATAAGACAGCCCTCATCTGCATTGTTATTTATTCCTTTT
GTTCCTGGTGGCATTGGTAGGTAATGGCTTAATCATCGCGGCACTGGGCAGTGAGTGGCTGCTGCGGAGAACGTTGTCAC
CCTGCGATAAGTTATTGGTCAGCCTGGGGACCTCTCGCTTCTGCCTGCAATGGGTGGTAATCAGTAAGAACATTTACATT
TTCCTGAATCCAGCGACCTTCCTTTATAGCCCTGTGTTCCAGCTCCTGGCCGTTCAGTGGGACTTCTTGAACTCGGCAAC
ACTGTGGTTCTCCACCTGGCTCAGTGTCTTCTACTGTGTGAAAATCGCAACCTTCACCCACCCCGTCTTCCTCTGGCTAA
AGCGGAATGTATCTGGGTTGGTTCCTTGGATGCTACTCAGCTCTCTGGGGTTCTCTGCCTTTACCACCATTCTATTTGTC
ATAGGCAACCAGAGAATGTATCAGAACTATTTAAGGAGGGGTCTGCAATCTTGGAATGTCACTAGGAATGCTGTGAGAAT
GTATGAGAGGTTCTACCTCTTCCCTTTGAAAATTGTTACCTGGACCGTCCCTACTGTTGTCTTTATTGCGGGCACGGTTT
TGCTCATTACATCTCTGGGAAGACACACCAAGAAGGTCTTCTTCTCCATCTCAGGCTTTCACAGTTCCAGTGCCCAGGCA
CACATCAAGGCCCTCTTGGCTTTTATCTCCTTTGCTATCTTCTTCACTTCCTCTTTTCTGTCACTGGTTCTCACTGCCTC
AGGTATGTTTCCCTTTCGGGAGTTCCGGTTCTGGATATGGCAGATTGTGATTTATCTGGGTACAGCAACCCACCCCATTA
TTCTTCTCTTAAGTAACCGCAGGCTGAGAGCTCTGCTAGGGAGGGGCTGCTCCTCAGCACATGGGGCATCTTGA
>OvarTAS2R67A_chr3:203469549-203470487
ATGCCATCTGGAATTGAAAATACTTTTCTAGTAGTAACAATAGGAGGATTTGTGATTGGAATGTTGGGGAATGGGTTCAT
TGTACTAGTTAACTGCATTGACCTGGTGAAGAGACAAAAGCTCTCATCAGCTGACTGCATCCTCACAGGCCTGGCTATCT
CCAGAATCAGTCAACTTTGGGCAATACTATGTGACTCATTTTTATTGGTACTATGGCCACACCTATATGCCATTGATAAA
CTAACAAAGGTTGTTAATAGTTTTTGGATACTGTCCAATCACCTAGCTACCTGGTTTGCCACCTGTCTAAGTGTTTTCTA
CTTCTTTAAAGTAGCCAACTTCTCCCACCCCTGCTTCACTTGGCTGCGGTGGCGAATTCGTAGTGTGGTACTGGTGCTTC
TCTTGGGGTCTTTGTCCTTACTGTTTTTGAATTCTGAATCAATATATATGTTTAGTCATATCTCAACTAACAGCTACAAA
ATATATGCAAGAAACTCAACGTGGTCCCCAAATGTAAGTGAAACTCATTATCTTCACCAGTTGATTGTTTTTAACTTCAT
CAACTTAATCCCCTTTCTTCTGTCCCTGACCTCACTGCTCCTCTTAGTCCTCTCCTTGATGAGGCACATCAGGAATTTGC
AGCTCAACCCCAGCTCAAAGGATCTCAGCACAGAGGCCCATAAAAGAGCCATGAAAATGGTGATGTCTTTCCTCTTCCTC
TTCGTCATTCATGTTTCTTCCGTCCTATTAACAGGTTGGGTTTTCCTTAAACTGCAGGGACGTCTGGCCAAATTGGTGGT
TGTGTTAACTTCGACTGTTTTTCCTTCAAGCCACTCATTTATCCTAATTTTGGGAAATAGCAAGCTGAGACAAAATGCCA
TAGGACTACTGTGGTATCTTAACTGCCGCCTGAAAAGAGTGAAATCTTTAGCTTCATAG
>OvarTAS2R67B_chr3:203477157-203478092
ATGCCATCTGGAATTGAAAATACTTTTCTAGTAGCAACAATAGGAGGATTTGTGATTGGAATGTTGGGGAATGGGTTCAT
TGTACTAGTTAACTGCATTGACCTGGTGAAGAGACAAAAGCTCTCATCAGCTGACTGCATCCTCACAGGCCTGGCTATCT
CCAGAATCAGTCAACTTTGGGCAATACTATGTGACTCATTTTTATTGGTACTATGGCCACACCTATATGCCATTGATAAA
CTAACAAAAGTTAGCATTTTTTGGACATTGTCCAATCACCTAGCTACCTGGTTTGCCACCTGTCTAAGTGTTTTCTACTT
CTTTAAAGTAGCCAACTTCTCCCACCCCTGCTTCACTTGGCTGCGGTGGCGAATTCGTAGTGTGGTACTGGTGCTTCTCT
TGGGGTCTTTGTCCTTACTGTTTTTGAACTTTGAATTAATACATGTGTTTAATAGTGTTTGGACTAATGACTACAAAATA
TACGCAAGAAATTCAACGTGGTCCCCAAATGTAAGTGAAACTCATTATCTTCACCAGTTGATTGTTTTTAACTTCATCAA

CTTAATCCCCTTTCTTCTGTCCCTGACCTCACTGCTCCTCTTAGTCCTCTCCTTGATGAGGCACATCAGGAATTTGCAGC
TCAACCCCAGCTCAAAGGATCTCAGCACAGAGGCCCATAAAAGAGCCATGAAAATGGTGATGTCTTTCCTCTTCCTCTTC
GTCATTCATGTTTCTTCCGTCCTATTAACAGGTTGGGTTTTCCTTAAACTGCAGGGACGTCTGGCCAAATTGGTGGTTGT
GTTAACTTCGACTGTTTTTCCTTCAAGCCACTCATTTATCCTAATTTTGGGAAATAGCAAGCTGAGACAAAATGCCATAG
GACTACTGTGGTATCTTAACTGCCGCCTGAAAAGAGTGAAATCTTTACCTTCATAG
>OvarTAS2R5PT_AMGL01124268.1:2457-3106
ATGCCGACTTCTATCCCAGGACTGATGATGCTAGTGGCAGTGGCTGAATCTCTCATTGGCCTCACTGGAAATGGAGTTCT
TGTGGTCTGGAGTTTTGGAGAATGTCTTCGAACGTCCAGGGAGTCCTCGTATAACCTCATTGTCCTGGGCCTGGCGGTCT
GTCGGTTGCTTCTACAATGGTTGATTATGGTGGACTCAAGTCTGTTCCTGCTTTTCCAGAGCGGCCATTGGCTTCGCTGT
CTCAGTGTCTTCAGGGTTCTGGTAAGCCAGGCCAGCCTGCGGTTTGTGAATTTTCTCAGTGTCTTTTATTGTAGGAAGAT
CATGACCGTTGAACACCCTGTCTCCTTGTGGCTGAAGCAGAGGGCCTGTTACCTGAGTTTCTGGTGCTTTCTGGTGTACT
TCATGATCCATTTGTTATAGTTAGGGGTAGCTTAGACTTCTCCAGTCCTTCCCAAGGAAACAGCAGCATCTTATTCCCCA
TTTCAAACTGGCACTATGGATGTATATTACAGCTCAATACAGGAAGTATGATGCCTTTCATGATGTTTCCTCTTTCCTCT
GGGCTGCTGATTGTCTCTTTGTATAGACACCACAGGAAGATGAAGGTCCATACAGCTGCTCCGGGGGCCAGCATGAGGAA
TTCCGCCCAT
>OvarTAS2R8P_chr3:203661111-203662039
ATGTTCAGTATAGAAGACCACATCTTTCTGACCATAACGACTGCAGAATTCATCATAGGAATGTTTGTGAATGGATACAT
TGGACTAGTAATATATATTGATTGGATTAAGAAGAAAAAGATCTCCACAACTGACTACATCCTCTCCAATTTAGCTCACT
CCAGAATTTGTTTGCTTTGTGTAATGACACTCAGCGGCACCATACTGGCACTCTACCCAGGTGTTTACGAAAATGAGAAA
ATAAAGGTAGTTCTTAATATCTTCTGGACATTCACCAACTACTTAAGTATGTGGTTTGCCACCTGCCTCAATGTCTTCTG
TCTCTTCAAGATAGCCAGTTTCTTCCACCGACTTTTTCTCTGGCTGAAGTGGAGAATCAAGAGGGTGTTTCACTGGAGCC
TGCTGGGGTCCCTGGCCATTTCCATGTTGATCAGCCTTATACAAGCAACATTAACAAATTCTGATTATGAATTTCTCAAA
ATTGAAAACATAAAAGAAACGTCACCGAATTGTTCCATGTGAGTAAAATTCAATACTTCAACCCACTGACACTGTTTAAC
TTGTCAGCAATTATTCCATTTACTGTGTCATTGATCTCATTTTTCTTTTTAATTACGTCCCTATGGAGACACAGCAAACA
AGTGAAATCCAGTGTAACAGGTGCCACAGACTCCAGCACAGAGGCCCACGTGGATGCCATGAAAACAGTGACCTCATTTC
TTTTCTTCCTTTCTGTATACTACCTGGCCTGTCTTTTGGCAACATTTAGCTACTTTATGAAAGAAAGCAAGTTAGCTATG
ATGTCTAGAGAGATTATAGCAATTCTTTATCCCTTAGGTCACTCACTGTTTTTAATTATTGGAAATAACAAGCTGAGGCT
GGCAGCTGTCGGGATGCTGAGATGTGGGAAAACAGTCTGCATGATGTAA
>OvarTAS2R9P_chr3:203659246-203660177
ATACCAGGTACAATGGAGGCAATATATATGTTCTTGATTGCTGGTGAGTGGATGATAGGAATTTGGGGAAATGGATTCAT
TGTACCGGTAAACTGCAGTGGCTGGCTCAAAAAGAGAGCTGTCTCCTTGACTGAGGTCATCCTGGTCAGCCTGGCCACCT
CCAGAATCTGTTTTTTGTGTGTGATATATATGGATGGTTTTATTATGGTACTCTTTCCAGATACATACAGGCATGGTGAG
ATGATGAACATTTTGGATATTTTCTGGACAACTTGCAATCATTCAACTGTCTGGTTTACTTCGTGCCTCAATGTCTTCTA
TTTACTCAAGATAGCCAGTATATCCCACCCAGTTTTCCTCTGGCTGAAGCTGAAGATGAACAGGGTATCCTTGGGATTCT
TCCTATGTCCTTTCTCATCTCCTCAATTGTCAGTGTTTTACTGAATAATGATTCATTTTATAACATCAAAATCAATAATG
AAGCAAACATTATTTAGGAATTCAAAGTAAGTAAAATCCCAACTGCTTTCAAATTGATTATCCTGAACCTGGGGGCTATG
GTTCCCTTTATTCTTTGCCTGGTCTCATTTGTCCTTTTATTTTTCTCACTTTAACACACCAAGCAAATGAAACTTCGTGC
CACTGGGTCCAGAGACCCTAGCATAGAGGCCCACATGAGGGTCATAAAGACAATAGTCATCTTTCTGGCTCTTTTCATTA
TGTACTATGCAGTTTTTCTCATTGTAACATCTATCTTTCTGATTCCTCATGGAAAATCGGAGCTGATGTTTGGTGGCCTA
AGAGCTGCCATTTTCCCATTGAGCCATCCATTCATCCTGCTAATGGGAAACAGAAAGCTGAGGGAGGCTTTTCTGAAGGT
GCTGGGGATTGTGAAGGGTTTCCACAAAAGAAGGAAATATTTTGTTCCCCAA
>OvarTAS2R10CP_JH922343.1:8764-8018
ATGCTGAGAAGGCCTCCTCATTTTTGTAGCAGTTAGTAAGTCAGTACTGGGGGTTTTAGGGGATGGATTTATTGGACTTG
CATACTTCACTGAATGTGTGAAGAACAAGAAGTTTTCTATCAGCTTTATTCTCATGGGCTTAGCTACTTCCAGAATTTGC

CTGATAGGGTTAACAACTGCCGATGGATTTGTGAAGATATTTTTCTCCAGAAGTGTGTTCCTGTGGTTACCTAATTCACT
GTATTACTTACTCATAGATAATTCTGAATCCATCAAGTGTCTTTTTTTGCCACTAGCCTCAGCATCTTCTATTTCCTGAA
GATGGCCAATTTTTCCCATCACATTTTTCTCTGGTTGAGGAGTGACATCAAAAGGGTTCTTCTCCTGCTGATGGGATACT
TGCTTATTTCATGGTTAGTTACTTTTCCACTAACTATGAAGATAATTAGTGATACTAGAGCAAAGAATAGAAGCGTAATC
TTTTCAGTTGAAGTGCATAAAGGGGAATTCTTTAGAAACCAGATTTTGCTCAATCTTGGAACCCTTCCCATCTTCATACT
ATGCCTGATTACATGTATCTTATTGCTCATTTCCCTTTGGAGGCACAACCAGAGGATGCTATTGAATACCACAGGATTCA
GAGACCCCAGCACAGAAGCACATATCAAAGCAATGAAAGTTTTGATATCTTTTATCATCCTTTTTATCTTGAATTTTATA
AGCATTATCATAGAAATATCATGCACC
>OvarTAS2R10DP_chr3:203636535-203637454
ATGCTAAGTATAACGGAAAGTCTCATTTTTGTAGCAATTAGTGAGTCAATATTGGAACTTTTAGGGAATGGATTTATTGG
ATTGGTAAGCTGCATTGACTGTATGAAAAACAAGATCTCTATTATCAGCTTTATTCTTGCTGGCTTAGCAACTTCCAGAT
TTTGCCTGATATGGACAATAGTTACTGATGGATTTTTAAAGTTATTCTCTTCAGATGTACATTCCTCTGGGAACCTAGTT
GAATATAATGGTTACTTATGGATAGTTATGAATCAATCAAGTATCTGGTTTGCCACCTGCCTCAGCATCTTCTATTTCCT
GAAGATATCCAGTTTTTCTCACCGCATCTTTCTCTGGTTGAAGGGTAGACTCAACATGGTTCTTTTCCTTCTTTGGGGAT
GCTTGCTTATTTCATGATTAGTTACTTTTCCACATTTTGTGAAGATTGTTAATGATAATAGAAAATAGAAACACAGTCTG
ATCAATGGATATGCATAAATGTGAACTCTTTGGAAAACAAATTGGGCTCCATCTTGGTGTCATTCCCCTTTTTATACTAT
GCCTGATTACATGTGTCTTGTTGCTCACTTCTTTTTGGAGACACAACAGGAGGATGCAATTGAATGCCACAGGATTCAGA
GACCCCAGTACAGAAGCACATATCAAAGCAGTGAAAGTCTTGGTGTCTTTTATCACCCTCTTTATCTTGAATTTTGTAGG
TACTGCCATACAAATATCAAGTGTGACAGTGCCTAAAAAACAAACTGCTTTGTATTTTTGGTATGACAACCACAGTCCTC
CATCTCTGGGGTCACTTGCTTATCCTAATTCTAGGAAATAGGAACCTCAAGCAAGCCTCTTTGAGGGTACTGAAGCCATT
AAAGTGTGGGGAAAAAGAGAAACTTCTCAGAACTCCTTGG
>OvarTAS2R13P_chr3:203553781-203554686
ATGGCAGATTCTTTGGAAAACATCTTTATCATTTTAATAAATTCAGCATTCATAATTGGTATTCTGGGGAATGGATTCAT
AGCACTGGTGAACTGCATTGACTGGATCAAGATGCAAAAGGTCTCCTTGGCTGATCGAATCCTCACTGCTTTGGCAATTT
TCAGAATTGGTCTGATTTTGGTAATGATGGTGAGTTGGTTTACAAAGGAGTCTTATCCATTTTCTTCTTTAGACATAAAG
GGAAATAAAGTCATACTTTTTAGTATTGCTGGGCTCTTGGCCAATTATTTTAGTGTCTGGCTTGCCACAGGCCTCAGCCT
CTTTTATTTCCTCAAGATAGCCAATTTTTCAAATGCTGTTTTTCTTCACCTAAAGTTTAGAATTGGAATGGTATTTATGG
TAATGTTTCTGGGGACATTAGTATTGCTGCCTCTAAGTCTTACTTTGGTGAGCATCTATATTAATATCAAGATACATCCA
TATGAAAGAAATATGACTTTAAGTTCTAAAAGAAGTGACACTGAAACCTTTTCCAAATTGATCATATTCACCATGGGATC
TTTCTCATCCTTTATTATATCCCTGAGTTGTTTTCTCCTGTTAATGTTCTCCCTACGGAATCATGTCTAGAAGATGAGGA
GCCAGGGTTCAAGAGATCCCAGCAGCAAAGCCCACGTCAGAGCCATGATCATGGCGATGCCTTTCCTCATACTACTTGCC
ATTCACTTCCTATCTCATCTCATGACAACTTTTCATCACAATGTGATGCAGAGTGAACTGGCCTTTATGCTTGCTGAAGC
TCTTGGAACTATTTATCCTTCAGTCCACTCATTTGTCTTGATTCTGGGAAATGACAAGCTAAGAAAAGCTTCACTTTTGG
TGCTGTGGCAGTTGAGGTGTGGCTGA
>OvarTAS2R408AP_chr3:203513457-203514370
ATGACAACTTTAGTATCGAGCATTCTTTCCATTCTACTGGTGACAGAATTTGTTTTGGGAAATTTTGTGAATGGTTTCAT
AGCACTGGTGAACTGCAATGACTGGGTCAGGAAACAAAAGATCTCCTCAGCTGATGGGATTCTCACTGCTCTGGCAGTCT
GCAGAATTGTTTTGCTCTGGACAATATTAATAAATTGGTATGCAACTATGTATAATCCAGCTCTATATAGTTTAAGAATT
GTTATCCGTGTTGCCTGGACAGTAAGCAACCATTTTAGTAACTGGCTTGCTACTAGCCTCAGTATATTTTATTTGTTCAA
GATAGCTAATTTCTCCAGCTTAATTTTTCTTCACCTGAAGTGGAGAGTTAAAAGTGTAGTTCTCATGATGATGTTGGGGA
CTTCATTGATTTTGTTTTTTCAGGTTGCAGTGTTAGGTATGAAACTATTCAGACAAGCGAATATGAAAGAAACATCACTG
AGAAGACCAAATTGAGGGACTTTTTACACCTTTCAAATATGACCCTGCTCACACTAACAAACTTCATACCCTTCAGTATG
TCCCTGACATCTTTTCTGCTGCTAATCTTTTTCCTGTGGAAACATCTCAGGAAGATGCAGCTCAATGGTAAATGATCCCA
AGATCCCAGCACCAAGGTCCACATAAAAGCCATGCAAACTGTCATCTCCTTTCTTTTCCTGTTTGCCACTTACATCCTGA

CTGTAATTTTAACAATTTGGAATTCTAATGAGCTGCGGAAGGAACCGGTCCAAATGCTTTTCCAGGCCCTTGCAATCACC
TATCCTTCAATGCACTCATTTATCCTGATTTGGACAAACAGGAACTTAACACAGACCTTTCTGTCATTTCTATGGCAGCC
AAGATGCTGGCTAAAAGTAAGAGGAACTAGGTAG
>OvarTAS2R40P_chr4:106066189-106067144
ATGGTGACAGTGAACACGGATGCGATGGATAAAGACCCAACCAGGTTCAAGATCATCTTCACCTTGGTGGTCTCTGCAAT
AGAGTGCATCATTGGTATCGTGGGGAACGGCTTCATCACCGTCATCCACAGAGCAGAGTGGGTCAGAGGCAAAAGACTCC
CCATTGGTGACTGCATTCTGCTCATGCTGAGCTTTTCCAGGCTCTTGCTACAGATCTGGATGATGCTGGAAAACACGTAC
AGTCTGCTGTTCTGGGTCATCTACAATGAGAAAAGAGTATACATACTTTTCAAAACCATCGTCATGTTTCTGAACTACTC
CAACCTCTGGCTTGCTGCCTGGCTCAATATCTTCTATTGCCTCAGAATCGCAAGCTTTACTCACCCGTGGTTCTCCGTGA
TGAAGAGGAAGGTCAGGGGCTGATGCCTGGGCTTGTGAGGCTGTCCTTGTTCTTCTCCTTTTGCTCCAGCTTCCCCTTCT
CTAGAGGCATCTTCAATGTGTACGTGAACAATTCCGTCCCCGTCCCCTCTTCCAGCTCCACTGAGAAGGTGTACTTCTCC
GAGACCAACATGGGCGACTTGGTTACCACCCTTTATCTGGGGATCTTCATCCCTCTGATCATGTTTATGCTGGCGGCCAC
CCTGCTGATCATCTCTCTCAAAAGACACACCTTCCACATGAAAAGCAACGCCACTGGCTCCAGGGACCCCAGCATGGAGG
CTCACCTGGGGGCCATCAAAGCCATCAGCTATTTTCTCATCTTCTACATTCTCAATGCAGTTGCTCTGTTTCTTTCCATA
TCCAACATCTTTGCCGCCAACAGCTCCTGGAATATTTTGTGCAAAATCATCATGGCTGCCTACCCTGCTGGCCACTCAGT
GCTACTGATCTTAGGCAACCCTGGGCTGAAAAGGGCATGGAAGCGGTTTCAGCACCAAGTTCATCTCTACCTGTAA
>OvarTAS2R42P_chr3:203456653-203457583
ATGTTCCCTGGGTTGAGTACAGTCTTTCTGATACTGTCAGGAGTGGAATTCTTAATCGGAATTCTAGGCAATGTGTTCAT
TGGACTGGTACTCTGCTCTGAATGTGTTAAGAACCAAAAGACATCTTTATTTGACTTCATCCTCACTGGCTTGGCTATCT
CCAGAATCAGTCAACTGTTGGTGTTTTTTGTGGAATCACTTATAATAGGACTAGAACCACAGGTATTTGCCATTTTTAAA
CTAGCGAAGCCCATTGCTTTACTTTGGAGAATATCTAATCATTTGACTACCTGGCTTGTCACCTGCCTGAGTATTTTCTA
TCTCCTTAAGATAGCTCATTTCTCCCACTCTCTTTTTTTCTGGCTGAAGTGGAGAATGAACAGCGTCATTCTTGTGATAC
TTGCATTTTCTTTGGTCTTTCTGATTTTGGACATTCTTTTGCTAGAAACATTTAATGATCTCTTCTGGAATTTAATAAAT
GAAGGCAATTTGACTTTAGTTGAAAGTAAAACTCATTATATTAAAAGCGAGAGTCTTCTTAGTTTCTCCTATTTCATTCC
TATTGTTCTGTCCCTGCTCTCATTGTTTTTTTTTATTTCGGTCCTTGGTGAAACACTCCAGAAATTTGCATCTCAATTTT
ATGGGTTCCAGGGACTTCAGCACAAAGGCCCATAAAAGAGCCATGAAAATGGTGACGTCATTCCTCCTCCTTATCATGGT
TCATTTTCTTTTTACACAATTGGCAAATTGGATGTTTCATAGGTTTTTGGACAATAAGTTCACAAAGTTCATCATGTTAG
CACTATATGTCTTTCCTTCAGGCCACTCGTTCATGTTGATTCTGGGAAACAACCAGTTAAGACAGATAGCCTTGAAGGTA
CTGAAGCATCTTAAAAGCTCCTTGAAAAGACAAAATCCATTGGCTTTATAG
>OvarTAS2R408BP_chr3:203524428-203525358
ATGATAGTATTTATGTCAAACATTGTTTCCATTCTATTAATGCAGAATTTGTTCTGGGAAATTTTGCCAGTGGCCTCATA
GCACTGGTGAACTGCAATGACTGGATCAAGAGACCAAAGATCTCAGCTGATGGGATTCTCACTGCTCTGGCATTCTGCAG
AATTGTTATGTTCTGGGCAATGTTAATAAATTGGTATGTAATTGTGAATAATCCAACTCTATATAATTCAAAAGTAAAAA
TTATTGTTCATGTTGCCTGGACAGTAAGCAACCATTTTAGTAACTGGCTTGCTTCTAGCCTCAGTATATTTTATTTGTTG
AAGATAGCCATTTCTCCAGCCTAATTTTTCTTCACCCGAAGTGGAGAGTTAAAAGTGTAGCTCTCATGATGATGTTGGGG
ACATCATTAATCTTGTTTATTCAAGTTGCAGTGTTAAGCATAGGTGAGGCTATTCAGATAAAAGAATATGAAGGAAATGC
CACTCAGAAGACCAAACTAAGGGACATTTTACACCTTTCAAATGTGACTCTGTTCACGCTAACAAACTTCATACCCTTCA
GTATGTCCCTGACATCTTTTCTGCTGCTAATCTTTTCCCTGTGGAAACATCTCAGGAAGATGCAGCTCAATGGTAAATGA
TCCCAAGATCCCAGCACCAAGGTGCATATAAAAGCCATGCAAACTGTCATCTCCTTTCTTTTCCTGTTTGCTGTTTACAT
TCTGGCTCTAATTTTATCAGTTTGGAATTCTAATGAGCTTCAGAAAGAACAAATGCTTTCTGATGTTCTTTTAATCATGT
ATCCTTCAATCCACTCATGTATCTTGATCTGGGGAAACAGGAAATTAACTCAAGCCTTTCTGTCATTTCTGTGTCAGTCA
AGATGCTGGCTGAAGGAAAGGAAATAGGTGGAAACATGTCTTTTAGCATAA
>OvarTAS2R408DP_chr3:203506723-203507622
ATGATAACTCTACTATCAACCATTTTTTCCATCCTAGTAATAATACAATTTATTCTGAGAAATTTTGCCAATGGCTTTTT

AGCCCTGGTGAGCTGCATTGACTGGGTTAAGAGACAAAAGATCTCCTCAATTGATGTGATTGTCACTGCTATGGCAGTCT
CCAGAATTGTTTTGCTCTGTGTAATGTTAATACATTGGTATTATATTTTGCTTCATCCAGCTTTATATGGTTTTAAAATA
AGAACTATTGTTCATGTTGCCTGGACAATAAGCAATCATTATAGCACCTGGCTTGCTGAAGCCAATTGAAGATAGCCAAT
TTCTCCAGCCTAACTTTTCTTCACCTGAATTGAGAGTTAAAAGTGTAGTCCTCATGATGCTTCTGGGAACTTCATTCATT
TTGGTTTTACAAGTTGTAGTTAAAAGTATAAGTGGGACTATGCAGAGAAGTGAATTTGAAAGAAACTTCACACAGAAGAC
CAAACTGAGGGATATTTTATGGCTTTCACATGTGACCCTGCTCATTCTAGGAAACCTCACACCCTTTACTATGTCCTTAA
TATCTTTTCTGCTACCAATCTCTTCCCTGTGGAACATCTCAGGAAGATGCAGCTCAATGGCAAAGGATTCCAAGTTCCCA
GGACCAAGATCCATACAAAAGCCATGCAAACTGTTATCTCCTATCTCTTGCTATTTGCCTTTTACTTTCTGGTTCTAATC
ATATCAATCTGGAGTCCTAAAAAGTTGCATGAGGAACCGTTTCTCTTGCTTTTCCCAACAGTTGAAGTCATCTATCCTTC
AGTCCACTCATTTATCCTGATTTGGGGAAACAGAAAGTTAACACAGGCCTTTCTATTGTTTCTGAGGCAACTGGGGTGCT
GGCTGAAAGACAGGAAATAG
>OvarTAS2R408EP_chr3:203543088-203543969
ATGATAACTTTACTACATTTTTTCCATCCTAGTAATAGAATTTATTCTAAGAAATTTTGCCAGTGGCTTCATGTCACTGG
TGAACTGCATTGACTGGTCAAGAGACAAAAAATCTCTTCAGCAGATGGGATTCTCACTGCTCTGGCAGTCTCCAGAATTG
GTCTTCTCTGGGTAACATTAATAAATTGGTATGTAAATGTGTTTAATCCCAGCTTTAGACAATTTAAGAGCAAGAATTAT
TATTATTGCCTGGATAATAAGCAACTATTTTGACAACTGGCTTGCTGCTATCCTCAGCATATTTTATTGCTCAAGATAG
CCAATTCTCCAATATTATTTTTCTTTACCTAAAATGGAAAATTAAAAATATTCTTCTTGTTCACTTTGTCCTGTTTGGCT
TTATTAATTCATGGTGTAAACATAAATAAGACTATCCAGGCAAATGACTATGAAGGAAACATCACTCAGAAGACCAAGTA
GAGGGACACTTTACACCTTTGAAATATGGCTGTGTTCATGCTAGTAAACTTCACACCCTTTGCAATGTCCCTGACATCTT
TTCTGCTGTTAATCTTTTCCCCGAAGAAACATTTCAGGAAGATGCAGCTCAGTGGTAAAGGACCCCAAGATCCCAGCACC
AAGGTCCATATAAAAGCCATGCAAACTGTCTTTTATTTTCTATTTGCCATTTACTTACTGGTTCTAGTTTTCTGTTTGGA
GTTCTAATAGGCAGTGGAACAACTTGGTTATCATGGCATGCATGCCAGGCTTTTGGAATCATATATCTTTCACTTATCCT
GACATGGGGAAAACGGATACTAAGACAGTCCTTCCTGTCATTTCTGCAGCAACTGAGTTGATGGCTAAAAGAAAGGAAAT
AA
>OvarTAS2R62P_chr4:106272421-106273346
ATGTCCCCTTCGCCCACATTGATCTTCAAGGTCACCCTTTTCCTGGAGTCATTGGTTGCCATGCTGCAGAATGGCTTCAT
AGTTACCATGATGAGCGGCGAGTGGCGTGTAGCTGGACTCTGCCCGCCAGTGACATGATTGCGGCCTGCCTGGCTGCCTC
CCGGTTCTGTCTGCATGGGATGGCCCTCCTGAACAACCTCATGGCCTCCTCTGGCTTTTGTTCCAAAATCTACTATTTCA
GCATCCCCTGGGATTTCATCACCTCCCTCAGTTTCTGGCTGACTGCCTGGCTTGCTGTCTTCTACTGCACGAAGATCTCC
CTCTTCTCTCACCTCGTCTTCTTCTGGATAAAGTGGAGGATTTCTCGATCAGTTCTCCAGCTGCTGCTGGGTTCCTTGAT
CTTATCTGGTCTGACTGTCATCTCCTCAGCTGCTGGGAATACAATTCTTGCCCAGATGACGGCTGCCCAGAGTTCCCATG
GAAACGCCCTGGCTGGTAGCATACACGCTGTCTATTTGCACTGTTTTCTACCTCATGTAATTCTCATGAGGTTGGTTCCA
TTCCTCCTGTTCCTGGTGTCCACCTTCTCACTCATGGTCTCGCTGCGCTGGCACCTCGGGCAGATACAGGACCACAGACC
CAGCCCACGTGATCCCAGTACCTGGGCTCACACCATGGCCCTGAAGTCACTTGCCTTCTTCCTCATCTTCTACACCTTGC
ACTTCCTGTCCCTGGTTATCATTGTGTACATCCCAGCCTTCCAGAAACACTGGCACTGGGCCTGTGAGGTGGTGACCTAA
GCAGGCATCTGTCTGCACTCCAGCATCTTGATGCACAGCATCCCCAAGCTGAGAAAGGCCCTGAAGAAGAAGCTTTGGTG
AGCCCTAGACAAGGACCAGTTTGTCTCCAGTTATTAGTATCAATAG
>OvarTAS2R18P_chr3:203528650-203529626
ATGTCAGTTGGAACAAAGGTCTTCTTTCTAGCGGTGTCAACAGGAGAATTGATCTTAGGAGTACTGGGAAATGGGTTCAT
TGGACTGGTAAACTGCATCGAGTGGGTCAAGAATGGAAAGGTTTCATCAGCTGAGTTCATCCTTACTTGCTTGGCTATGG
CCAAAATCATTCAGCTGTGGGTAACACTTTTGGATTCACTTATAGTAGGATTAGCTCCACATCTGTATGCCACTGGTAAC
TAGTAAAAGTAGTTATTCTTCTTTGGGCACTAATGAATCACTTAACTATCTGGTTTGCCACCTGCCTAAGCATATTTTAC
TTCCTTAAGATAGCCACTTTCTCTCACTTCTTTTTCATGTGGCTGAAATGGAGAATGAACCGAGTGCTTCTTGTGCTTTT
CCTGGCCTCTTTCTTCTTATTAACTTTTGACCTCTTAATGCAAGATGCTCTTGGTGAGTTGTGGATGAACACCTTTAGAG

AACCTGAAAGGAAACTTTGCATTTAGATGCAAGTAAAATTTTCTATCTTAAAAGTCTGATTCTTCTCAGCTTGACATATG
TTATCCCTTTCATTCTCTTCATGGCTTCTTTGCTGCCTTTCTTTCTTTTCCTGGTGAGACACATCAAGAATTTCCAAGTC
AACTTGAACCACCGAGAGATTTCAGCACAGAGGCCGATAAAAGGGCCTTGAAAATGGTGACAACATTTCTCCTCTTCTTT
ATCGTTTACTTTATTTCTACTCCATCTGGAAATTGGATCTTCCTTAAGCTACACTGGTATGAGGTCATGATGTTTGTCAT
GGTGATTTCAACTCTCTTTTCGTCAGGCCACTCATTTGTTATAATTTTGGGAAACAGCAAGCTAAGGCAGGTTGTCTTCA
GACTACTGTGGGGTCTTAAGTTCTCTAAAAACTAATCAAAACTTTAGCTTAATAGACATTAAAAAAACTTTCTGTATTAT
ATGTGGAAATACCTTAA
>OvarTAS2R372CP_chr3:203626738-203627254
CAGCAGGTAATAAGAGGAGTTTTTGAGTTCTCCTGGAATCTGAGAAACTCCTTAACTACTGGATATGCTGCCTGCCTCAG
CGTCTTCTACTTCCTCAAGCTATCTAGTTTGTCCCACCCCTTCTTTCTCTGGCTAAAGTGCAGAAGAGATAGAGTTTTTT
TCACCATTATGTTGCAATTCTGTCTCTCTTTGATTTTTAATCTTCTGAGCATAAAATTTATACTTTTGTGTTCAGCAAGC
ATTTAGAAAAGGAAAGTCTTAACTTGAAAAAAAGGTTTGCATAAAAATCAGTATCACATAAAAAAAATAAAAATAAATAA
TAAAAATAATAAAAAAAATAAAAATAAATAAAAATTAAAAAATCAGTATCACAGCAGTCAAGTTCTCTTCAGCCTTGGAT
CTCTCATTCCCTGGTCTGTATCACTCATTATATTTTTCCTGTTACTCTTTTCCTTATGGGGACATACCAAGCAGATGACA
CGCCATAACGCAGACCCCCGGGACTTCAGCACAGGGG
>OvarTAS2R372AP_chr3:203641892-203642746
ATGTCAAATGTCATCCAATATGTTTTTTTGATCATTGAAATCTCAGAATTCATAACAGGAATTTGCGGAAATGGATTCAT
TGCACTAGTACTCTGTGCTGACTCTCTCAAAAACGAGAATATCTCCTTGCTTGACTTCATCTTCACATGCTTGGCCATCT
CCAGAATTGGTATGATATTCATACTTCTCCTGGATAGCATTAAAATTATGTTCCATCCAGAAATATTAGATCGTCACCAG
GTAATAGAAGTAACTTTTGATTTCCTCTGGAATCTGAGCAATTCCTTAGGTACCTGGTGTGCTGCCTGCCTCAGCGTCTT
CGACTTCCTCAAGCTATCTAGTTTTTCCCACCCCTTCTTTCTCTGGCTAAAATGGAGAAGAAATAGAGCTGTTTTCACCA
TTATGTTGGGATTCTGTCTCTCTTTGTTTTTTAATCTTCTGAACATAAAATTCAATGCTCTCAGGGTCTGTGACCATTTA
GAGATAGAAAACAACTTGACTTGGAAAAAATGCATGCGTAAAACACAGTCTTATAGCAGTCAAATTCTCCTCCACCTGGG
ATCTCTCATCCCCTTGGCTGTGTCACTCATTTATTTTTCCTGTTAATATTTTCCTTATGGAGACATACCAGGCAGATGAC
ACGCTATGCCAAAGGATCCAAAGACCTCAACACAGGAGTTCTTGTGAGAACAAGAAATACGTTGGCCTCTTTCATCATTC
TCCTAGTTGTGCACTATTTGGCTACATTCATGTTAACTTGGTCCTATTTCACACTAGAAAATGACATGACTTTTGTTGCT
ATTCACACTGTAGCATTTCTCTATCCTTCAATTCACCCTTTTATTTTGATTCTGA
>OvarTAS2R372BP_chr3:203610142-203610992
ATGTCAAGTGTAATCAAAAAAGTTTTTATAATCACTGAAATCTTAGAACTCATAACAGGAATTTGCAGAAATGAATTCAT
TGCCCTAGTACTCTGTGCTGACTCTCTCAAAAGCAAGAATATCTCCTTCTTTGACTTGATCTTAACATGGTTGGCCATCT
CCAGAACTGGCATGATATTCATAATTCTTGGGTGGTGTTAGAATAGTGATCTACCCAGGAATATTTGAAAGTCATCAGGT
AATAGAAGTGATTTTTTATTTCTTCTGGAATCTGAGCAACTCCTTAGGTACCTGGTGTGCTGTCTGCCTCAGCGTCTTCT
ACTTCCTCAAGCTATCTAATTTTTCCCACCCTTTCTTTCTCTGGCTGAAGTGCAGAAGAAATAGAGTTGTTTTTACCATT
CTGCAGGGATTCTGTCTTTCTTTGATTTTAATTTTCTGAGCATAACTTTTCATACATTTGGGGTCAGTGACCATTTAGAA
ATAGAAAACAACTTGCATTGGAAAAAATATGCATAAAATCCGGTCCTATAGCAGTCAAATTCTCCGCCACCTAGGATCTC
TCATCCCCTTGGCTGTGTCATTCATTTTATTTTTCTTGTTAATCTTTTCCTTATGGAAACATACCAGGCAGATGACAAAT
CATGCCAAAGGATCCAGAGACTTCAACACAGGGATTCTTGTGAGAGCCAGAAATACTTTAACTTCTTTCGTCATTTTCTT
AGTTGTGCACTATTTGGCTACATTCTTGTTAACCTGGTCCTGTTTCACACTAGAAAATGAAATGACTTTTATTGTTATTA
AGTCTGTAGCATTTCTCTATCCTTCAATTCACCCTTTTATTTTGATTCTAG
>OvarTAS2R10AT_chr3:203649607-203650170
ATGCTGAGTATAGTAGAAGGCCTCCTCCTTTTTGTAGCAATTAGTGAGTCAGTATTGGGGGTTTTAGGGAATGGGTTTAT
TGGACTAGTAAACTGCATTAACTGTGTGAAAAATAAGAAGATCTCTACACTCAGCCTTATTCTCACTGGCTTAGCCTCTT
CCAGATTTTGCTTGATATGGATAATAACTACAGATGCATATGTGAGATTGTTTTCTCCAGATATGTATTTGTCTGGTGAT
CTAAGTCAATATATAGCTTACTTATGGATAATTATGAATCAATCAAATGTCTGGTTTGCCACCAGCCTCAGCATCTTCTA

CTTCCTGAAGATAGCCAACTTTTCCCACTGCATTTTTCTCTGGCTGAAGGGTCACATCAATAAGATCCTTCTTCTTCTAA
TGGGATGTCTGCCCATTTCATGGTTATTTACTTTCCCAAACATTACAATGCCTTTTATTAATAATATTATGAAGAACAGA
AACACAACCGGGTTGATCACCATGCAGAAAAGTGAATACTTTATAAATCAGATTTTCTTCAGTATTGGAACACTTCTTGT
CTTT
>SoarTAS2R1_NW_004545871.1:27490064-27490996
ATGCCACCATCTGACCTCATTCCCAATGTTATTTTGCCATGTTACAACTTCTTTTGGGCTCTTGGCAAATGGTACCAT
TGTGGTTGTCAATGGCATGGATTGGGCCAAACGCAGAAAGTTGGCTCCCCTGGACCTCCTCCTCTCCTGCCTGGCCCTTT
CCAGGCTGTGCATGCAGTCATTCGTCTTCTACATTCACCTGGGTGTTCTTTCCTTGATCGAAGTGAAGGTAGTTGCTGAG
GGCTTCGTAATGTTCCTTTTTGTAAATTTCTCAACGACTTGGTTTGCCACATGGCTCAGCGTTTTCTACTGCATGAAGAT
TGCCACCTTTGCCCACCCTCTTTTCTCCTGGTTGAAGACGAGGATATCCAGGATGGTCCCTTGGTTGGTCCTTGGGACCC
TGCTCTATGCCTCCATCACTTCTGTTTTCCATGGCAAACATTCGTGGCAGATTTGCAAAGAGTTATGGTTGAGCATTTTC
TCCACAAATGCAACATATCAAATCCATGAGGAACCTGTGTTATTTTTTGTCCTACTTGGCGCTGAGCTCTTTCTTCCATT
TTTAATCTTCCTCCTTTTTGCCCTGCTCTCGATATTGTCCCTGCTGAAACACAGCCGGCAGGTAAGAAACACAGTGGGGG
GACCCAGGGCCTGTAGCTCCTACCTCAGAGCGCTTCTGTCAATCCTCTCCTTCTTTGTCCTCTACTTCTGTCATTGCATG
GCAGCTACCTTGCTCTCTATGAAGATTTTCACATTCAGAAGCTTCTTATTTTATTTTGGCATCTTTGTCATTGGCTCATA
CCCCTCTGGACACTCCATCATCTTAATTTTAGGAAATTATAAACTAAAGCAAAATGCAAAGAAGTTCCTTCTTCACAGTA
AGTGCCAGCTGAAAAGGGATATTGATCCCAGCCAAGAATCTGTGGCCCAGTGA
>SoarTAS2R2_NW_004545860.1:43835595-43836494
ATGGCTTCTTTATCAGCTGTTCCTCATGTTATCATCATGCTGGCAGCATTTATCACAGGGATTATAGCAAATGGATTTCT
TATAATCGTCAATGGTTATGAATTGATCAAAAGCAGAAAGTTAACAACAACGCAACTCCTTTTCGTCTGTATAGGGATGT
CTAGAATTGGTCTACAGTTGGTGTTAATGGCACAAAGTTTTTTCTCTATGTTTTTTCCATTTTTTTACCGAGTAAAGATT
TATGGTGCAGGACTGTTATTCTTTTGGATGTTTTTCAGCTCGGTCAGTCTCTGGTTTGCCACCTGCCTTTCTGTATTTTA
CTGCCTCAAGATATCAGGCTTTACCCAGTCCTGTTTTACTTGGTTGAAATTCAGAATCTCAAAGTTAGTGACATGGCTGA
TTCTGGGAAGTCTGGTAGCCTCCCTGGGCACTGCAGTACTGTGCATCACAGTAGATTACCCTAAAAACGTGGATGATGCC
GTCATCAGGAATGCCACACTACATGGAACTAGCCTCAATTTAAAGCGAATTAATGAAGTGTTCCTTGTCAATTTGGGGTT
AATATTTCCTCTGTCCATATTTGTGATGTGTACTTTTATGTTGTTCATTTCTCTCTCTAAGCATACTCAGCGAATGCAAA
ACAGAACTCATGGTTTTCAAAATGCCAGCACAAAAGCTCATGTAAATGCATTAAGAACAGTAATAACATTCTTTTGCTTC
TTTATATCTTATTTTTCCGTGTTTATGATTAGTCTGACATTTAGTATTTCTTATGGAAGTCTGTGTTTTTTCATGGTGAA
AGACATTATGGCAATATATCCTTCTGGTCATTCAGTTGTAATAATTCTGAGTAATTCTAAGTTTCAGCAACCATTCAGGA
GATTTCTCTGCCTTAAATAG
>SoarTAS2R3A_NW_004545887.1:7250905-7249919
ATGTTGGACCCCCCAGAGCAGGTCTTCCTGGTTCTGATTCTCATTCAGTTCCTCCTGGGAATGCTGAGCAATGGTTTCAT
CGGGTTGGTCAATGGCATCAGCTGGTTCAAGAGCAAGAGACTCTCTTTGTCTGACTTCATCATCACGATGCTGGCCCTCT
CTAGGATGATCATCTTGTGGATTCTCCTGACCGATGGGCTTTTAATGTTATTTTTCTATAGCGTACATGAAGATGGCTTA
GGGATGCAAATGATCGATATTTCCTGGACATTTACAAACCATCTGAGCATTTGGCTTGCCACCTGCCTCAGTGTCTTCTA
CTGCCTGAAAATCGCCAACTTCTCCCACCCGACCTTCCTCTGGCTCAAGTGGAGAGTGTCCAGGATGGTGGTGGGGATGC
TGCTGGCTGCAGTGCTCCTGTCCTGCGCCAGCGCCTTGTCTCTGATCCACGAATTCAAGATCTATGCCCTTCTCCGTGAA
CCTGACGGCAGAGGGAATGTGACTGAGCACTTCAGGATGAAAAGCCATTATGAATTGTTCCATATTCTTGGGAATCTGTG
GGGCGTGCCTCCCCTCCTCGTGTCTCTGGCCTCCTACCTCCTGCTCCTGCTCTCGCTGGGGAGGCACACGCGGCAGATGC
AGCAGCTCAGCGGCCGCCCCAGGGACCCGAGCACTGAGGCCCACAAGAAGGCCATCAGGATCATCCTCTCCTTCTGTGCT
CTCTTCCTGCTCTATTATCTTTCCTTTTTGACCACGTATTCCAGTCATTTCCTACCGGCAACGAAGATGAGCGCGATGGT
CGGAGAAGCAATTACAATGTTTTATCCGGCTGGCCACTCAGTTGTTCTGATCATGAGCAATAGCAAACTGAAGCAGACGT
TGGTCCAGATGCTCTGGTCTGAGCCGGGCCCCCGGAAGCCTGGCTCCAAGGGATGCCCTGCCCCCTCGAGGCGTTACGGC
GGAGATCCCGAGAGTCTTTCAGACTGA

>SoarTAS2R3B_NW_004545887.1:7231680-7230733
ATGTTGGAACTCACAGAGCAGGTCTTCCTGATTCTCGTTATCATTCCGTTTCTCCTAGGTATTCTGGGCAATGGTTTCAT
TGCGTTGGTCAATGGTGGCGGCTGGTTCAAGAACAAGACACTTTCTCTGTCGGACTTTATCATCACTCTCCTGGCTCTTT
CTAGGATCATTTTGCTATGGATTCTCCTGATAGATAGTGTTTTATTGGTGTTGCCTTCCAAGGTACAAGATGATATGATT
TTAAGGGAAATGCTGGGAATTTTCTGGACACTTACAAACCATCTGAGCATTTGGCTTGCCACCTGCCTCAGTGTCCTCTA
CTGCCTGAAAATCGCCAGTTTCTCCCACCCGACCTTCCTCTGGCTCAAGTGGAGAGTGTTCAGGGTGGTCTTAGGGATGC
TGCTGGCTGCAGTGCTCCTGTCCTGCGCCAGCGCCTTGTCTCGGATCCACGAATTTAAGATCTGTGCTGCTTTTAGTGAA
ATCAGCGGCACAGGAAATGTGTCTGAGCACGTTAGGAAAAAAAGTAAATATGATTTGGTCCATGCACTTGGAATTCTTTG
GGACCTCCCTTCCCTAATAGTAATGCTGGTCTCCTACTTTCTGCTCCTTGTCTCCTTGGGGAGGCACATTCAGCGGATGC
AGCTACATGGAATCAACTCCAGAGATACAACTACACAGGCCCACAGGAAAGCCACCAAAATCATTCTCTCCTTTCTCTTT
TTCTTTCTACTGTATTACATTTGCATTTTACTTTCATTTTTCAGATTTTTCCTACCCGGACCAACAACGGTTAAGATGAT
CGGAGAAGTATTTTCTGTGTTTTATTCAGCTGGCCACTCATTTATTATTATTTTAGGAAGCAACAAACTGAAACAGACAT
TTATAAAGATGCTTTGGTGTGAGACTTGTCAACTTCAGATTGGATCCAAGGAATCCCTTGCCCCTTAG
>SoarTAS2R3C_NW_004545887.1:7187584-7186670
ATGTTGGACTTCAACAAGTGTTTGTTCTTGATTCTGTATTTCATCCAATTCATCCTTGGAACACTGGTGAATGGTTTCAT
AGGATTGGTCAATGGTTGTGGCTGGTTCAAGAGCAAGAGGCTCTCTTTGTTCGACCTCATCATCACAATCCTGGCTCTCT
CTCGGATTGTTCTCCTGTGGATTCTCTTGATTGATGGTATTTCAATGGTGCCTTCCAAAGCATATGAACACAATATAGGA
AAGCAAATTCTTGAAATTATCTGGACATTTACAAATGATCTAAGCATTTGGCTTGTCACCTGCCTCAGTGTCCTCTACTG
CCTGAAAATCGCCAATTTCTCCCACCCGACCTTCCTCTGGCTCAAACGGAGAGTCTCCGGGATGGTAGTGTGGACGCTGC
TGAGTGCAGTGCTCCTGTCCTGTGCCAGTGCCTTGTTTCTGATCCATGAATTTAAGATCATTGTTGTTACCTCTGGAACC
AATGATACAGGGAATATCTCTGAGTACTGCAGAAAGAAGAGTTACTATGAATTGGTCCATGGTCTTGGGATGCTTTGGAA
CCTCCCTCCCCTAATAGTGTCCTTGGCGTCCAACATTCTGCTCGTCCTCTCCCTGGGAAGGCACATGCAACAGATGAAGA
AAAGTGGTACTAACTGTAGAGATTCAACCACGGAGGCCCATAAAAGGGCCATCAAACTCATCCTCTCTTTCCTCTTTCTA
TTTCTACTTTATTATCTTGCTCTTTTGATGGTATCATCCAGCCATTTCCTACCAGGAACTAAGATGATTAAGATGACTGG
GATAATAATTAAATTATTTTATCCTTCTTGCCACTCATTTATTCTCATTCTGGGACATAATAGACTGAAGCAGGCATTTG
TACAAATGCTCTGGCATAAATCTTGTCCTCTGTAG
>SoarTAS2R4A_NW_004545887.1:7242823-7241912
ATGAGTCAAGACGATTCCTATACTGTTTACTCTGTTCTCATTGTCGCCACAGTTTTTCATTTTGTGGGGCTCACTGCAAA
TCTGTTTATTGCAGTTTTCAGTTGTAAGAACTGGATGGAAAATCAAAGAATTGTTTCTTCCGACAAGATCCTCTTCAGCT
TGGGCATTGCCCGGTTTCTTATGATGGGACTGTTTCTCTTGAACGTTTTCTGCCTCTTCATCTCCCCTGCTCTTCTCAGG
TCAGTCCACGTATCTATTTTCTTCCTGGTGTGCTGGATGTTTCTGGACTCCTGTAGTCTGTGGTTTGTGACCTTGCTGAA
CACCTTGTACTGTGTGAAGATTTCTAACTTCCAACACTCAGTGTTTCTCCTGCTGAAGCAAAATCTCTCCCCAAAGATCC
CTGGGCTGTTGGTGGCCTGTGTGCTGATTTCTGCCTTTACCTCCCTCCTATATGTTGTGTTCAGACAGACTTCACCCACC
CCAGGACTCGTGGTTGGAAAGAATGACACAGAGTTAGACCTGAAAGAGGACGTTCTGTTTTTGCTGACCTCTTTTATCCT
GAGCTCATCTCTGCAGTTCATCGTGAATGTGACGTCGGCTTCCTTGCTAATCCATTCCTTGAGGAGACACATACAGAAGA
TGCAGAGAAATGCCTCTGGCTTTTGGAATCCCCAGACGGAAGCTCATGTGGGCGCCATGAAGCTGATGATCTGTTTCCTG
GTCCTCTATATTCCCTACTCTGCTGCCACCCTGTTCCTCTACTTACCTTTTCCTGTTCAGATGCCTATGGTATCCAAATT
AATTTGTATGATTATTTCTACACTTTACCATCCAGGACATTCTGTTCTCATTATTCTCATGCACCCAAAACTGAAGACCA
AGGCAAAGCAGATTCTTTGTTTCAAAAAGTAG
>SoarTAS2R4B_NW_004545887.1:7227787-7226855
ATGTGTCACGACAATTATTCGTGTACATTTTTATGTGTTGTTATTATTGTCATAATTTTTGATTTTGTGGGACTCACTGC
AAACCTGTTTATTGCAGTTTTCATTTGTAAGAATTGGATGAAAAGACATAGAATTGTTTCTTCCGACAAGATCCTCTTCA
GCTTGGGCATTGCCCGGTTTCTTATGATGGGACTGTTTTTCTTGAACATCTTCTGCCTCTCCATCACTCCTGATATTCTC
AGGTCAGTCAATGCATCTATTTTCTTCTTGGTGTGCTGGATGCTTTTGGACTTCTGTATTCTTTGTTTTGTGACATTACT

GAACACCTTGTACTGCGTGAAGATTTCTAACTTCCAACACTCAGTGTTTCTCCTGCTGAAACAAAATCTCTCCCCAAAGA
TCCCTGGGCTGCTGGTGGCCTGTGTGCTGATTTCTGCCCTTGTCACTCTTCCCTCTGTCGTGTTCAGACTGATTTCACCC
TCCCCAATAGTTGTGGTTAGGAAAAATGGCACAGAGTTAGACCTGAAAGAGGACATTTTGTTTTTGCTGACCTCTTACGT
CTTGAGCTCATTTCTGCAGTTCATCGTGAACGTGATATTGGCTTCCTTGCTAATCCATTCCTTGAGGAGACACATACAGA
AGATGCAGAGAAATGCTACCAAGTTTTGGAATCCCCAGACGGAAGCTCATGTGGGCGCCATGAAGCTGATGATCTGTTTC
CTGGTCCTCTACATCCCTTACTCGATTGTCACCCTGCTCCTTTATTTACCCTCCTTTTTGGGGATGACTATGAAAATGAG
AATCATTTGCTCGGCATTTACTATCCTTTACCATCCAGCACATTCTGTTCTCATTATTCTCACACACCCTAACCTGAAAA
GCCAAGCCAAGCAGATTCTTTGTTTCAACAAACAGAAGTTCAGGGGTCAGTAG
>SoarTAS2R4C_NW_004545887.1:7176801-7175905
ATGCCTCCTAGAGTGTTTTTATCTGCTTCAATTGTTGCCACCATTTTTGATTTTCTGGGACTCACTGCAAGTCTGTTTGT
TGTAGTTTTCAATGGTAAGAATTGGATGGAAAGTCGCCGAATCGCCTCTTCTGACAGACTCCTGCTCGGCTTGGGCGTGG
TGCGGTGTTTCCTGATGACTCTGTTTCTCCTAAAAAGGATCTGCTTCATTGTCTCTCCAGCTCTTGTCAGGTCAGCCCAA
ATATCCATTTCTTTCCTGGTGTGCTGGATGTTTCTGGACTCCGTTTGTCTGTGGTTTGTGACCTTGCTGAACACCTTGTA
CTGTGTGAAGATTTCTAACTTCCAACACTCAGTGTTTCTCCTACTGAAGCAAAATCTCTCCCCAAAGATCCCTGGGCTGC
TGGTGGCTGGTGTGCTGATTTCTGCCTTTATCACCCTCCTGTACATTGCACTCAGACTGACACTGTATCCTCCAGAACCT
GTGAGTGGGAAAAACTCCACAGAATTCGATGCGAAGAACATCTTGTCTTCAGTGGCCTTTTTCATCCTGGGCTCATCTCT
GCAGTTTATCGTGAATGTGACGTCGGCTTCCTTGCTAATCCATTCCTTGAGGAGACACATACAGAAGATGCGGAGAAATG
CCTCTGGCTTTTGGAATCCCCAGACGGAAGCTCATGTGGGCGCCATGAAGCTGATGATCTGTTTCCTGGTCCTCTATATT
CCCTACTCTGCTGCCACGATGCTCTATTATTTACGCTCCTTTGCAAGGCTAGGGGTGGGCGTCAGAGCCTTATGTATGAT
AGTTTCCTCTATTTACCACCCGGGACATTCTGTTCTCATTATTCTGACACATCCTAAACTGAAGGCAAAAGCAAAGAGGA
TTCTTTGTTTCAACTAA
>SoarTAS2R5_NW_004545887.1:7172387-7171488
ATGTTGAGTACTGCGCTGAGACTCCTGATGCTGGTGGCAGTGGCCGAATTTCTCATTGGCCTGGTTGGAAATGGAATCTT
GGCTGTCTGGAGCTTTAGCGAGTGGACCAGGTCACTCAAGGGGTCCTTGTACAACCCCATTGTCTTGGGCCTGGCTGTCT
TCCGATTTCTCCTGCAGTGGTTGATCATGATGGACTTGAGTCTGTTTCCACTGTTCCAGCGCAGCCTCTGGCTTCGTTAC
GTTAACGTTTCCTGGGTTCTGGTGAGCCAGGCCAGCTTGTGGTTTGCAACTTTCCTCAGCATCTTCTACTGCAGGAAGAT
CACCACCTTTGAACACCCTGTCTACGTGTGGCTGAAACGAAGGGCTTATTGCCTGAGTCTCGGGTGCCTCCTGGGGTACC
TCATGATCAGTTGCTTGCTGATAGCCCACATTAGCTTAAAGTACTTCGTTCCTAGTCAAGGAAACAGCAGCATTGTGATC
CCTCTTTCAAACTGGCACTATATGGGCATATTGAAGCTCAGTGCAGGAAGTGGCTTGCCTTTCATGGTGTTTCTTGTTTC
CTCGGGGATGCTGATTGTCTCTTTGTTTAGACATCACAGGAAGATGCGCGTCCACACAGCTGGTAGGAAGGATCCTCGGT
CCAAGGCGCACATCACTGTCCTGAAGTCCTTGGGCTGCTTCCTGCTGCTCCACGTGATTTATATCTTGGCGACCCCGATT
TCCATCACCTCCAAGTCTTTTGAATCTAATCTCTCTACAGTCTTCATCTCTGAGATAATCATGGGTGCTTATCCTGCTCT
TCATTCTGTCGTATTGATCATGGGGAATCCCAGGGTGAAGCAGACTTGTCAGAGAATCCTGTGGAAGACAATTTGTGCTT
GGAGATCTTGGGATCAGTGA
>SoarTAS2R7A_NW_004545902.1:15804813-15805745
ATGTCAGGTGAGGTGAACAGCACCTTAATGATCATAGCAGCTGGAGAATTCTCCATGGGAATCTTAGGGAACGCCTTCAT
TGCACTGGTAAACTGCTTGGACTGGATGAAGAAAAGGAAGGTGGCCTCCATTGATTTAATTCTCACATGTCTGGCTACTT
CCAGAATCTGGCTACTGTGCATAATAATGCTAGACTGCTCTTTGTTGGCACAGTATCCAAATGGCTATGCTGCCGGAACA
CAAGTGAAAGTCATTGACTTCTTCTGGACACTCACCAACCACCTCAGTGTGTGGTTTGCCACCTGCCTCAGTCTCTTCTA
CTTCCTCAAGATAGCAAATTTCACGCATCCCCTTTTCCTCTGGATGAAGTGGAGAATCGACCGGGTGGTTCTTAGGATGC
TGCTGGGGTGCGTGGTCCTCTCTGTGCTGATCAGCCTTGCTGTCACTGAGAATTTGAATGACTTCCAGTACTGTATCAAG
GTAAAGAAAGAAAGAAACGGAACATTAAAGTACTGCGTAAATGTAGCTCAATATGAGTCCACCAAGATATTTCTCAACCT
TTTAACAATATTTCCTGTTTCTATATCACTGGTCTCCTTTTGCCTCTTGATCCTCTCCCTGTGGAAACACACCAGGAAGA
TGCAGCTCAGTGCCAGCGGACACAGAGACCCAAGCAGAGATGCCCACTTGGGAGCCATGAAAACTGTCATCACCTTCCTC

CTCCTTTTCATTGTCTACTGTTTGGCCTTTCTCATAGCCACTTCCAACTACTTTATATCAGAGACTGAACTAGCTGTGTT
GGTTGGGGAGCTGATAGCTCTAATTTATCCTTCAACCCATTCATTTATTCTAATCCTAGGAAACAATAAACTGAGAGAAG
CGTCTCTAAGGGTGCTATGGAAGATAAAGGCAATTCTAAGCAGAGATTTCTAA
>SoarTAS2R7B_NW_004545902.1:15743372-15744328
ATGTCAGATGAGGTGAACAGCACCTTAATGATCATAGCAGCTGGAGAATTCTCCATGGGAATCTTAGGGAACGCCTTCAT
TGCACTGGTAAACTGCTTGGGCTGGATGAAGAAAAGGAAGATGGCCTCCATTGACTTCATTCTCACGTGTCTGGCTACTT
CCAGAATCTGGCTACTCTGCGCAATAATGCTAGACTGCTTTATGTTGGTGCAGTATCCAAACGTCTACACTGCCGGTAAA
CAAATGAGAGTCGTTGACTTCTTCTGGACACTCACCAACCACCTCAGTGTGTGGTTTGCCACCTGCCTCAGTCTCTTCTA
CTTCCTCAAGATAGCAAATTTCACGCATCCCCTTTTCCTCTGGATGAAGTGGAGAATCGACCGGGTGGTGCTTAGGATCC
TGCTGGGGTGCGTGGCCCTCTCTGTGTTGATCAGCCTTGCTGTCACTGAGAATTTGAATGATGATTTCAGGTACTGTATC
AAGGCAAAGAAGAGAAGAAACGGAACATTGAAGTGCAGTGTAAATAAAGCTCAATATGAGTCCACCAAGATATTTCTCAA
CCTTTTAACAATATTTCCTGTTTCTATGTCACTGGTCTCCTTTTGCCTCTTAATCCTCTCCCTGTGGAAGCACACCAGGA
AGATGCAGCTCAGTGCCAGCGGACACAGAGACCCAAGCAGAGATGCCCACTTGGGAGCCATGAAAACTGTCATCACCTTC
CTCCTCCTTTTCATTGTCTACTGTTTGGCCTTTCTCATAGCCACCTCCAGCTACTTTATACCAGAGAGTGAACTAGCTGT
GTTGGTTGGGGAGCTGATAGCTCTAATTTATCCTTCAACCCATTCATTTATTCTAATCCTAGGAAACAATAAACTGAGAG
AAGCATCTCTAAGGGTGCTATGGAAGATAAAGACGATTCTAACAACAGAAGGAGATTTGAAGAGTCTCAATTTCTAG
>SoarTAS2R8A_NW_004545902.1:15810637-15811548
ATGTTCACTAAAGAAGATATCGTCTTGGCTATTATAATGGCTGGAGAATCTATGATAGGCATGTTGGCGAATGGGTACAT
TGGACTAATGTTCTGGATCGACTCTGTTAAGAAGAAAAAGACACCCTCACTAAACTACATCCTCACAAGTCTGGCTATTT
CCAGAATTTGTCTGCTCTTTATAATAGTAGTAGATGACATTGTGCTGATACTCTACCCAGAGCTTTATGGAGAGGATAAA
CTAGTGATAGTATTCAGTGCTGCCTGGGTGTCCATCAACTACTCGAGTTTGTGGTTTGCCACCTGCCTCAACGTCTTCTA
TTTACTGAAGATAGCGAATTTCTCCAGTCCACTTTTTCTCTGGTTAAAATGGAGGATTGACAGAGCGATTTTCTGGATGT
TGCTGGCAGGCTTGGCCATTTGCTCCTTGAGTGGCCTTCTGACAGCAAAACTGAATTATTATGAACACAGTTATAGATTT
ACAAAACATCAAAGAAACATCACTGAAATATTTCATGTGAGTGAAATTAAATATTTCAACCCATCAGTCCTCTCTAACCT
TTTGGCAGTTGTCCCAGTGACTGTGTCACTGATTTCTTTTTTCCTGTTAATTTTGTCTCTGTGGAGACATACCAAGCACA
TGAAACTCAATGTTACGGGCTGGAGAGACCCCAGCACAGAAGCGCATATTACAGCCATGAAAAATGTGACTTCTTTTCTC
TTCCTCCTTTTCGTATACTATGCGGCTTGTCTTTTAGTAACTTACAGTGACTTAATGAAAGGAAAGTTAGTTTCTGGGCT
TGGAAAGATTATAGTGATTCTCTACCCCTCTGGCCACTCCCTTATTTTAATTATTGGAAATAGCAAGCTGAAACAAGCAT
TTGTCAGGTTATTGAGATGTGGTGGGGGTTAG
>SoarTAS2R8C_NW_004545902.1:15786328-15787248
ATGTTCACTAAAGAAGATATCGTCTTGAGTGTTGTAGTGGCTGGAGAATCTATGATAGGCATCTTGGCAAATGGGTACAT
TGGAATAATGTCCTGGATTGACTCTATTCAGAAAAATAAAATCCCCTCACTTAACTACATCCTCACCAGTCTGGCTATTT
CCAGAATTTGTTTGCTCTTCATAATAGTAGTAGGTGACATTATAGTGAAACTCTATCCAGATTTTTATAAAGATGTTAAA
ATAGTGATAGTATTCAGTGCCTTCTGGATACTTTTCAACTACTCAAGTTTGTGGTGTGCCACCTGCCTCAACGTCTTCTA
TTTACTGAAGATAGCGAATTTCTCCAGTCCACTTTTCCTCTGGCTAAAATGGAGGATTGACAGGGCGATTTACTGGATCC
TGCCGGTGGGCTTGACCATTTGCTCCTTGAGCAGCCTTCTGACAGCAATGCCGAATTATTATGAACACATTTATAGATTT
ACAAAACATCAAAGAAACATCACCGAAATGTTCAATGTGAGTGAAATTAAATATTTCAACCCATCGACCCTCCCTAACCT
TTTGGCAGTTGTCCCAGTTACTATGTCATTGATTTCATTTTTCCTGTTAATTTTGTCTCTGTGGAGACATACCAAGCAAA
TGAAACTCAATGTTACAGGCTGGAGAGACCCCAGCACAGAAGCCCATATTACAGCCATGAAAAATGTGACTTCTTTTCTC
CTCCTCCTTTTCGTGTACTATGCGACTTGTCTTTTACTAACTTACCTAATGAAAGGAAAGTTAGCTTCTGGGCTTGGAAA
GATTATAGTGATTCTCTACCCCTCTGGCCATTCCCTTATTTTAATTATTGGAAATAGCAGGCTGAAACAAGCATTTGTCA
GGATATTGAGAAGTGTTGGGGGTTTGTGGGGAACAGCTTAA
>SoarTAS2R8E_NW_004545902.1:15769339-15770274
ATGTTCACTAAAGAAGATATCGTCTTTGCTATTATAATGGCTGGGGAATCTATGATAGGCATCTTGGCAAATGGGTATGT

TGGAATAATGTCCTGGATGGACTCTGTTAAGAAGAATAGAACCCCCTCACTTAACTACATCCTCACCAGTCTGGCTATTT
CCAGAATTTGTTTGCTCTTTATGATAATAATAGGTGACATTATTCAGAAACTCCATCCAGATTGCTATGAAGATGTCAAA
CTAATAATAGTATACAGTGTCTTCTGGGTACTTATCAACTACTCAAGTTTGTGGATTGCCACCTGTCTCAACGTCTTCTA
TTTACTGAAGATAGCGAATTTCTCCAGTCCACTTTTCCTCTGGCTAAAATGGAGGATTGACAGGGCGGTTTACTGGATCT
TGCTGGCAGGCTTGGCCATTTGCTCCCTGAGCAACCTTCTGACAGCAATGCCGAATTATTATGAACACATTTATAGATTT
AAAAAACATCAAAGAAACATCACTGAAATGTTCCATGTGAGTGAAATTAAGTATTTCAACCCATCAGTCCTCTCTAACCT
TTTGGCAGTTGTCCCAATTACTGTGTCATTAATTTCATTTTTCCTGTTAATTTTGTCTCTGTGGAGACACACCAAGCACA
TGAAACTCAATGTTACAGGCTGGAGAGACCCCAGCACAGAAGCCCATATTACAGCCATGAAAAATGTGACTTTTTTTCTC
TTCCTCCTTTCCATATACTATGCAGCTTGTCTTTTAGTAAATTACAGTGACATAATGAATGCAAAGTTAGTTTCTGGGCT
TGGAAAGATTGTATTAATTCTCTACCCCTCTGGCCACTCCCTTATTTTAATTATTGGAAATAGCAGGCTGAAACAAGCAT
TTGTCAGGATATTGAGAAGTGTTGGGGGCTTGGGGGGCACAGCCTACCTAATGTAA
>SoarTAS2R8G_NW_004545902.1:15752515-15753450
ATGATCACTAAAGAAGATATCGTCTTTACTATTATAGTGGCTGGAGAATCTATGATAGGCATCTTGGCAAATGGGTACGT
TGGAATAATGTCCTGGATCGACTCTATTCAGAAGAATAAAACCCCCTCACTTAACTACATCCTCACAAGCCTGGCTATTT
CCAGAATATGTTTGCTCTTTATGATAATAATAGGTGACATTATTCAGAAACTCCATCCAGATTGTTATGAAGATGTCAAA
CTAGTAATAGTATACAGTGTTTTCTGGATACTTATCAACTACTCAAGTTTGTGGTTTGCCACCTGTTTCAACGTCTTCTA
TTTACTAAAGATAGCGAATTTCTCCAGTCCACTTTTCCTCTGGCTAAAATGGAGGATTGACAGGGCGATTTACTGGATCC
TGCCGGTGGGCTTGACCATTTGCTCCCTGAGCAGCCTTCTGACAGCAATGCCGAATTATTATGAACACATTTATAGATTT
ACAAAACATCAAAGAAACGTCACTGAAATTTTCCATGTGAGTGAAATTAAATATTTCAACCCATCAACTCTCCCTAACCT
TTTGGCAGTTGTCCCAGTTACTGTGTCATTGATTTCATTTTTCCTGTTAATTTTGTCTCTGTGGAGACATACCAAGCACA
TGAAACTCAATGTTGGCTGGAGAGACCCCAGCACAGAAGCCCATATTTCCGCCATGAAAAATGTGACTTCTTTTCTCTTC
CTCCTTTTCGTATACTATGCGACTTGTCTTTTACTTACTTACAGTGATATAATGAAAGAAAAGTTAGTTTCTGGGCTTGG
AAAAATTATATTGATTCTCTACCCCTCTGGCCATTCCCTTATTTTAATTATTGGAAATAGCAGGCTGAAACAAGGATTTG
TCAGGATATTGAGAAGTGTTGGGGGTTGGGGGAACAGCCTGGCTAATGTAAATTAG
>SoarTAS2R8I_NW_004545902.1:15740080-15740982
ATGATCACTAAAGAAGATGTCATCTTGATTATTATGGCTGGGGAATCTATGATAGGCATGTTGGCGAATGGGTACATTGG
ACTAATGTCCTGGATCGACTCTATTCAGAAGAATAAAACCCCCTCACTTAACTGCATCCTCACAAGTCTGGCTATTTCCA
GAATATGTTTGCTCATCAGAATAATAGTAAGTGACATTATAAAGATAGTCTATCCAGATTTTAATAAAGATGTTAAACTA
TTGATAGTACTCAGTGCTTTCTGGATACTCTTCAACTACTCAAGTTTGTGGTTTGCCACCTGCCTCAATGTCTTCTATTT
ACTGAAGATAGCGAATTTCTCCAGTCCACTTTTTCTCTGGCTAAAATGGAGGATTGACAGGGTCTTGCTGGTGGGCTTGA
CCATTTGCTCCTTGAGCAGCCTTCTGACAGCAATACCGATTTATTATAAAGGCAGTTACAGATTTTCAGAACATCAAAGA
AACATCACTGAAATGTTCCATGTGAGTGAAATTAAATATTTCAACCCATCAACTCTCCCTAACCTTTTGGCACTTGTCCC
AGTTACTGTGTCATTGATTTCATTTTTCCTGTTAATTTTGTCTCTGTGGAGACATACCAAGCACATGAAACTCAATTTTA
CAGGCTGGAGAGACCCCAGCACAGAAGCCCATATTACAGCCATGAAAAATGTGACTTCTTTTCTCCTCCTCCTTTTTGTA
TATTATGGGGCTTGTCTTTTAGTAACTTATTGCCACCTAATAGAAGAAGAAAGATTAGCTTGTATGCTTGGAAAGATTTT
AATGATTCTCTACCCCTCTGGCCACTCCCTTATTTTAATTATTGGAAATAGCAAGCTGAAACAAGGATTTGTCAGGATAT
TAAGGTACAAGGGGGGAAAATAA
>SoarTAS2R10_NW_004545902.1:15727352-15728281
ATGCTCAGTATAGGAGAAAGCCTTCTGCTTTTTGCAGCAATCAGTGAATCAATACTGGGGACTATAGGGAATGGATTTAT
TGGAATTGTGTATTGCATTGACTATGTGAAGAACAAGAAACTCTCTCTGATTGGCTTTATTCTCACTGGCTTATCTACCT
CCAGAATTTGTCTGACATGGTTAATAATTACAGATGGGTTAATGAAGATCTTCTCTCCAGATATCTATATCTTGAGTGGT
TCAATTGATTGTGTTAGTTACTTATGGGTAATTTTTAATCAATCGAGTATCTTATTTGCCACCAGCCTCGGCATTTTCTA
TTTCCTAAAGATAGCACATTTTTCCCACCACATTTTCCTCTGGTTAAAGGGAAGAATCAATAGGGTTCTTCCTCTTTTGA
TGGGCTTCTTGCTCATTTCCTGGTTACTGATTTTCCCACAAATTGTGAAGATTCTGAATAATAAAAGCACGGAAAATAAA

AACATCACCTCGTATCTTTCCAAGTCGCTCCGTGAATTCGTGATCCACCAGGTCCTGTTCAACCTGGGAAGCATTCTTTT
CTTTACCCTAACCCTGATGACCGGCGTCTTGTTGATAATCTCCCTCTGGAGGCACCGAAGGCAGATGCAAGCCAACTTCA
CCGGATCCAGGGACTCGAGCACGGCAGCACATATGAGAGCCATGAAAATTGTGTTATCTTTCATCATCCTCTTCGTCTTG
TATTTTATAGGCATTGCTATAGAATTATCATGTCCGACTATACCCCAGAACAAACTGCTCTTTATTTTTGGCATGACCAC
TGCAGCTGTCTATCCATGCGGGCACGCCTTTATTTTAATTCTAGGAAACCGTCAGCTGAAGCAGGTCTCGTGGAGGCTCC
TACAGCAGCTAACGTGCCAGGAGGAGAGAAAATTTCCCACAACTCCATAG
>SoarTAS2R11A_NW_004545902.1:15722554-15723498
ATGTTGAGTATGCTGGAGACAGTTTTCATGATTTTAACATCTGGGGAACTTGTAGTAGGAATTTTAGGGAATGGATTCAT
TGGACTCACAAACTTCACTGCCTGTATTAAAAAGCAGAAGTTATACTTGGTTGACTTCATTCTTATGAGTTTGGCGTGGG
CCAGATTCACTCAATTGTGCCTAATGCACATCAGTGTGTGGGTATTGAAGTTCTCTTGGGAAATTATGGATATTTTGGAA
GATAACAAAGTCCTTACAAGCATGTGGATGCTGATCAATCATTTGTGTACTTGGTTTTCTACCTGTCTGGCTGTGTTTTA
CTTCCTAAAAATTGCCAGTTTTCCCCACCCTCTTTTCCAGTGTCTGAAACGGAGCATTAACAAGGTCATTGTCACGCTTC
TGGTGGTATCTGTGCCTTTCCTACTCATGGTTCTTCCTTTTCCTTACAACATTCATATCATTAGATATCAAGTCGATCTA
AAATATGAAAGAAATATGACAGGGTTTAACTATGTGACTTTAAAGAACAAGTTAAATTATATGACTATCTTCACAATAAC
CTCCCTGCTTCCTTTCTGTCTTTCCTTGATTTCCTTTTTTCTTCTGCTTCTTTCTATGCGGAGACACACAAAGCACATGG
AGCTCAATATCAGGGGTCGCGGAGATCCCAGTCTGGAGGTCCATTTCCGAGCCATGAGAAGCGTGTTTTTCTTTCTTGTG
CTCTTTCTGCTGTTCCACATGGCCCTTTTGCTAATGGCTACTGCATGTTACTCTCAATACCGAAAAGTGATTATGATGTT
AGGCTATTTATTAGGTCTTCTGTATCCTTCCGCTCACTCTTATGTGGTGATTTTTGGCAACAGTCAAATGAAGAAAGCCT
TTTTTGGGGATTTTGTGGACCTGGAAACATTGCCTGAAAGCAAGTTTCTACAGAGATATACATAG
>SoarTAS2R11B_NW_004545902.1:15717592-15716657
ATGTCAAGTACACTGGAGAAAATTTTTATAATTGTAGCCGGTGGGGAATTTGTAGTAGGACTTTTTGGGAATGGATTTAT
TGGAGTCAAAAATTGTATCATGTGGATTAGAAAGCAGAAGTTGTACTTGGTTGACTTCATTCTTACTAGTTTGGCCTGGG
CCAGATTCAGCCAACTGTGTTTCTCAATTTTCCATTTCCGCTTGGTGCTACCATCTCAGGATGTCCAGGTGGCTATGAAA
AGAACCCAAGTCCTTTCTAGTTTATGGATAGTAACCAATCATCTGAGCACTTGGTTTGCTACTTGTCTGGCTGTATGTTA
CTTCTTGAAGATTGCCAATTTTTCCCATCCCCTTTTCCTTTGGCTGAAACAGAGAACTGACACCACCATTTTTGTGCTTC
TACTGGTATCTGTGCCTTTCCTGTTCGTGACCTTGCCTTTGCCTTACAGTGATCACCTCTTCAGGTCTCCAGTCCTTCCT
CAATATGAAAGAAACGTGACAGGATTAGATCATGGAAGTGAAACGATTGTAATGATTATATTCACAATGGCGTCCCTTCT
TCCTTTCTGTCTTTCCTTGATTTCCTTTCTCTTGCTCCTCTTTTCTTTGTGGAGACACACAAAGCATATTGAGCTCAATA
CCGGGAATGCCAGAGACCCCAGTTTGGAGGCTCATTTCAGAGCCATGAAAAACATCTTTTTCTTTCTGGTATTGTTTGTA
CTGTACCACTTTGCCCTTCCAATTACATGCGGGGCATATCAAATGATACGCAACAAGCAGGTTGTTATATTTGGATTTGT
ATTAGGGCTTCTGTGTCCTTCGGCTCACTCTTTTGTGTTGATTTTTGGAAATAACCAATTGAAGAAAGCTTTCTTTGGAG
TATTTTGGCACCTGAGTTATTGCTTGAAAGGACAGATTCCTCAGCAACATAAGTAG
>SoarTAS2R11C_NW_004545902.1:15668807-15669748
ATGTTGAGCACCCTGGAGAAAGTTTTTGTGATTGTAGCTGGTGGGGAATTTATAGTAGGACTTTTGGGGAATGGATTCAT
TGGACTCACAAATTGTACTGCCGGCCTTAGGAAGCAGAAATTATACGTCGTTGACTTAATTCTGACTAGTTTGGCCTGGG
CCAGATTCATTCAACTGTGGCAAACCATTCTCTCTCTCAGTTTGGTGCTAACTTGTAAGGATATCCAAATTGCTTCAAAA
AGAACCCAAGTCTTATCTAACACCTGGATATTGAACAACCATTTGTGTACCTGGTTTGCCACCTGTCTGGCTGTGTTTTA
CTTCCTGAAGATCGCCAATTTTTCACATCCTCTTTTCCTTTGGCTGAAATGGAGAATCAACAAGGTCATTTTCATGCTTC
TAGTGGTATCTGTGCCTTTTATGATCATAATTTTTCCTATGCCTCATAGTGTTGGCATCTTTGGTCATCAAACCTGTCCC
GAATGTGAAATAAATAGGACAGAGCTATTCCATGTGAATAAAACAAATTTAAATTATATGTTTATCTTCGCAATAGCATG
CCTTCCCCCTTTCTGTCTTTCCTTAATTTCATTTCTCTTATTGCTCTTCTCTTTGTGGAGACATAAGAAACATATTGAAC
TCAACAGCACGGATTCCAGAGATCCCAGTATGGAGGCCCATTTCAGGGCCATAAAAAGCGTGTTTTTCTTTCTGGTCCTG
TTTGTACTGAACCACTTTGCCCTTTTCATCACAGGTGGGTCGTATTATATATTACATAACAAGCAGGCTCTGATTTTTGG
CTATGTATTAGGACTTCTATATCCGTCGGCTCACTCATATGTGGTGATTTTTGGAAACAGTCAAATGAAGAAAGCTTTCT

TGGGACATTTTTGTTACCTGAGGCATTACCTGAAAGGAAAATGTCCTTTGCGACATAGATAA
>SoarTAS2R11D_NW_004545902.1:15666136-15665159
ATGACACTCCAAACTTCACATCAGAATTTTCTCATTGCACTCCTGTTGAATATGATGGAGAAAGTTTTTCTGATTGTAGC
TGGCGGGGAATTTATAATAGGACTCCTAGGGAATGGATTTATTGGATTCACGAAATGCACTTCCTGGATTAGAACGCAGA
AGTTATACTTGGTTGACCTCATTCTTACTAGTTTGGCCTGGGCTAGATTCAGCCAGTTGTTGCTTATTATTGTCAATATC
TGGACAGTGCAATTCTCTCAGGAAGTTTGTGATGTAACAGAGTATAACAAAGTCTTTAGTAGTATTTGGATACTGAACAA
CCATTTGTGCGTTTGGTTTGCTACTTGTCTGGCTATATTTTACCTACTGAAGATTGCCAATTTTTCCCATCCCCTTTTCC
TTTGGCTGAAGTGGAGAATTAATAAGGTCATTTTCATACTTCTCGTGGTATCTGTCCCTTTTCTGTGTATAACTTTTCCT
ATGCCTTATGGTTTTGATATCTTCAGTTATAAAATCCTCTCAAAGTACAAAAGAAATATGACACGATTAAAAACTGTATG
TGACAAGAACAATTTAAACTACATGCTTGTCTTCATGATAGCTTACCTCCCTCCTTTCTGTCTTTCCCTGTTTTCCTTTT
TCCTGTTGCTCCTTTCTTTGCAGAGACACAAAAAGCACATAGCACTCAGCATGAGAAATTTCAGAGACCGCAGCGTGGAA
GCCCATATCAAGGCCATGAAAAGCGTATTTTGCTTTCTGTTCCTGTTTTCACTGCACCACGTTTCCCTCTTACTGACATT
CGGAGCATATTATATACTACGTAACAGACTGCTTTTGCTTTTCTCCTATATCCTGTGTCTTCTGTATCCTTCGATTCACT
CATATGTGGTGATTTTTGGAAATAGCCAAATGAAAAAGGACTTCTGTGGAATTTTTTCACATCTGAAACACTACCTGAAA
AGAAAATTTCTGCAGTAG
>SoarTAS2R13_NW_004545902.1:15712520-15713422
ATGTTAAGTGCCTTGGAGAATATCATTGCTACTTTACTCCATGTAGAAATCATACTTGGTATTTTGGGGAATGGATTTAT
AACACTGGTGAACTGCATTGATTGGTTTAAGAAGCGAAAGCTCTCCTGGACTGATGGAATCCTCACTGCTTTGGCGATTG
CCAGACTTTTTCTTATTTTGACAATGATGGTTCGGTTTATGATGGATTTTTATGTAGTTTTGCATCTTAGCAGCCAAAAA
ATTATATTGATTACCATTGCAGTGACCATTGGCAATCATTTTAGTGTCTGGCTTGCTGCAGGCCTCAGTATCTTTTACTT
TTTCAAGATAGTCAATTTTTCAAATCCTGTTTTTATTTACCTAAAGCACAGAGTTGAAATGGTGGTTTTGGTAACCATGC
TGGCAGCATTAGTATTTTTGCCTTTTATTCTTGCCATGACCAGCAGATATCTTAGCATCCGGATACAGTCAGATGGAAAA
AATATGACTTTAAGTTCCAAATGGAATGACAATGAAAAATTTGTAAAACTGATTTTATTCATCGTTGAAGCCTTTGTACC
ATTTTTCATATCCCTGAAGTTTTTACTTCTGTTAGTCTTCTTCCTCTGGAAACATCTCATGAATGTGAAGACCAGCACAA
CAAAATTCAGAAATTCCAGCTTGAAGATTCACGTAACAGCCATGAAAATCATGACGTCTTTCTTCTTCCTGTTTGCTACT
TACTTCCTGACTTGTCTTGTAACAACTTTCTATCATGAGGAGTTGAAGAGCAAATTGGTCCTGTTGTTGGTTCTGACAAT
TGCAAATGCTTGTCCTTCAGTTCACTCGTGGATCCTGATTCTGGGGAGCCATAAGCTGAGAAAGGCTTCTCTTTGCCTGA
TGTGGCAGTTGCGATGTGACTGA
>SoarTAS2R14B_NW_004545902.1:15675930-15674992
ATGGTCAGCATCACAGAAATTACAATTCTAATCATTCTAAGTGCGGTATCCTTTGTAGGAATTCTGGGAAATAGCTTCAT
AGTGCTGATTACCTGCATGGACTGGATCAAGAGAAGAAAGATGTCTCCAGTAGACCAGGTGCTCACTGCTCTGGCCATCT
CCAGAATGGCTATGCTCTCAGTATTACTAATAAATTCATATGCACTCGTCTTTGCCTCAACTATATACGTGACTGAAGAA
ACGTTAAAGGTGATTGCTTATGCCTGGACAGTCACCAATCATTTCAGCATCTGGCTTGCTACAAGTCTCAGCATCTATTA
TTTTCTCAAGATAGCCAGTTTTCCCAACTCCATCTTTCTCTACCTAAAGTGGAGAGTGTCAAAGGTGGTTTCCGTGGTCC
TGCTGGTGACCTTGACCTTCTTGATCATTAATATGACCTTGGTGGACATGCTTATTGACGACTGGTTCCATGATTCTCAA
AGGAACATGACTCACAGTTCCCGAATGAGCGACTTCCCACAATTTTCCAAACGTATTATATTCACTAACACCATGTTCAC
TCTTGGACCCTTTTCCGTGACCCTGACTATTTTTCTCCTGCTGATCTTCTCCCTGTGGCGACATCTCAGGAGGGTCCTGC
ACAGTGGCAAAGACTGCCGAGATGCCAGCACCAAGGCACACATCAAGGCCATGCAGAGTGTCATTGCCTTCCTTGTGCTC
TATGCCGCTTTCTTTCTGTCCCTTTTAATGTCATTTTGCAACTCTGACATGCTAGGGAAAAACCAGAACACACTGTTGTG
CCAGACCACTGGAAGTGTTTACCCCACGGGACACTCGTGTGCCTTGATTCTGGGCAATCGTAAGTTGAGACAGGCCTGTC
TCTCTGTGCTCTGGTGGTTGGGGGTCCGAGTCAGAGGTGGAGCACCCTCTCGCTCATAG
>SoarTAS2R18_NW_004545902.1:15518147-15519055
ATGTCACTAGGAATGAAAGCATTATTTCTGTCAGTGATAACAGGGGAATTCATTTTAGGAATGCTGGGAAATGGATTAAT
TGCACTGGTCAATTGCATGGATTGGGTGAAGAAGGGGAAGATCTCATCAGCTGATTTCATCCTTTTCAATTTGGCAACGG

CCAGAATCATTCAACTGTGGGTAACACTATTGGGTTCCTTTATGTGGCTATTACCACAAAATTATCACAATGGCAAGCTA
CTATTGGTATTTGCTTTTCTTTGGACACTCAGTCATCACCTTACCATCTGGTTTACCACTTGCTTAAGCATCTTTTACTT
TCTCAAAATAGCCACTTTCTCCCACTCCCTCTTCACCTGGCTGAAGTGGAGAGTGAAGAGGGTGGTTCTCGTGATTTTCC
TGGGCTCTTTCTTCTTACTGTCTGTTGACCTGGCGAAGAAGGGTACTGTTAGTGACCTGTGGATAAATGCTTACAGTGTC
CCTGGAAGAAACTGGACTCTGCATTTATATGCGAGTACAATTTTCCGTCAGGATTACATTATTCTTTCCTTGATCTTTGT
TATCCCATTTCTCCTGTCCTTGACTTCCTTGCTTCTTTTATTCCTTTCCTTGTTGAGGCACACAAAGAACTGGCAGCTAA
ACTCTCCAAACCCCAGGGACTCATGCACAGAGGCCCATGAAAGAGCCATGAAGATGGTAGCCATTTTCCTTCTCCTCTTC
ATGATTTACTGTATTTCCATTCTATCAGCCAGTTGGATCTTCGTTACGGGAGAAATGTTTCAGGCCAATTTGGTGGCCAT
GATGCTGTCTGCCATCTTTCTCTCAGGCCACTCATTTGTGATAATTTTTGGAAACAGAAAGTTCAGGCAGGTTCTCCTGA
GATTGCTGTGTTATCTTAAACGCTCTTGA
>SoarTAS2R38_NW_004545887.1:7103636-7104640
ATGGTGAGACTCTCTCCTGTTGTCACTGTGTCCTATGAAGTCAAGGGTGCATTTCTGTTCATTTCCTTTCTGCAATTCAT
TGCGGGAACCCTTGCCAATGCCTTCATTGTCTTGGTGAATTTGAGGGACGTGGTGAGGAGGCAGCCACTGACCAACTCTG
ACCTTGTCCTGCTGTGCCTCAGCCTCACCCGGCTGTTCCTGCACGGGCTGCTGCTTCTGGATGCCATACAGCTGACCCAC
TTTCAGCAGATGAAAGACCCGCTGAACCTCAGCTACCAAATCATCATCATCCTCTGGATGATTACCAAGCTAATTGGCCT
CTGGTTGGCCACCTGCCTCAGTCTCCTCTATTGCTCCAAGATTATCCGTTTCTCTCACCCTCTCCTGGTTGCCTTGGCTT
TCTGGATCCCCAGGAAGATCCGGCAAATGCTCCTGGGCAGTCTCTTCTTCTCCTTTGTCTGCACCACTTTCTGTTTGGGC
AGCTACTTTGGTCAACCTGGCTTCGCCACAACTGCGCAATTCGTGAATAGCAGCATGAAACTCAACTTGCAAATTGAAAA
ACTTCATTTATTCCATTCATTACTCTACTGCAGCCTGGGGTCCGTCCCCCCTTTCCTGATTTTTTTGCTTTCTTCTGGCA
TGTTGATTGTGTCCTTAGGGAAGCACCTGGGGACAATGAGGGCCATATGTAGGAACACTCAGGACCCCAGCCTGGAGGCT
CACATCAGAGCACTCAAATCCCTCGTCTCCTTTCTCTGTTTTTTTGTGCTGTCGTTCTGTGCTGCCTTCACGTCGGTGCC
TCTGCTAATGCTGTGGCACAATAAAATCGGGGTGATGATCTGCGTGGGGATCATGGCAACTTGTCCCTTGGGACATGCAA
TTATCTTGATCTCAAGCAATGCTAAGCTGAGGAAAGCAGTGGAAACCATTTTTCTCTGGGCTCAGAGCAACCAAAAGGTA
AGGGCAGGCCACGAGGCAGATGCCAGGACACCAAATCTCTGTTGA
>SoarTAS2R39_NW_004545887.1:5652695-5651736
ATGGCCAAAACCTACAACACCCCTCAAAACGAATTATCGCCATTTCCTACCATCTTAATTTTTACCATTCTAGGTGTAGA
ATGCATCACGGGCATCATGGCAAACGGCTTCATTGCGGCGATCCATGTGGCCGAGAGGATTCAGAACAAGGCATTTTCCA
CAAGTGGCCGGATCCTATTTTTCCTGAGTGTATCAAGAACAGTTCTTCAGAGCTTCATGATGCTAGAAATTACCTTAAGC
TCACTCTCCCCACAATTTTATCATGAAGAAATTGTGTATAGTGCAGTCAAAGTAAGTTGCATATTCTTAAATTATTGCAG
CCTGTGGTTTGCCGCCTGGCTTAGCTTCTTCTACTTTGTGAAAATTGCTGATTTCACCCACCCTCTTTTCCTCAAACTGA
AGTGGAGGATCTCGAGGCTGATGCCTCAGCTCCTGTGGCTCTCGCTGTTCATTTGCTTGGGCTACAGCACCCTCTTCTTC
AATGATGTCTACGATGTGTATTGTAACGACTGCTTCTCCATCCATGCCTGCAACTCCACCAAGAAGACGTACTCCACGGA
GACCAACGTGGACATTCTGCTTCTTCTCTACAGCCTGGGGATCTTCCTTCCTCTCTCCCTGGTCATCCTCACAGCCATGC
TCCTGATCCTCTCTCTCAAGAAGCACACCCTGCACATGAGAAGCAAAGCCACTGGCTCCAGGGACCCCAGCATGGAAGCT
CACCTGGGGGCCATCAAAGCGACCAGCTACTTCCTCGTTCTCTACATTTTCAACGCCATTGCATTATTTCTCTATCTGTC
CAACATCTTTAAGAGCCACAGCTTCTGGAATATTTTGTGCAAAAGCATTATGGCGGGCTACCCCGCTGGCCACTCAGTGC
TACTGATCTTGAGCAACCCGGGGCTGAGAAGAGCCTGGAAGAAGATTCAGCACCAATTTTATATTTACCTGAAACAGTAG
>SoarTAS2R40_NW_004545887.1:5611126-5610194
ATGTCCGTGGTGAGCTCAGACGTGCCCGACCGAGAGCAGCCCACTTTCCAGACCATCTTCATCCTGCTCATCTCCGGGCT
CGAGTGCGCCGTGGGCGTCATCGGGAACGGTTTCATCGCCGCCATCCATGGGACGGCCTGGGCCCGGCGCAGGAGGGTCC
CCCTGTGCGACCGCATCCTGCTCTTCCTGAGCGTCTCCAGGATCCTGCTGCAGATCTGGATGATGGTGGAGAACACGTAC
AGTCTCCTGCTCTGGCGCGCCTACAGCCAAAACACGGTGTACACGCTGTTCAAGGTCATCCTGATGTTTCTGAACTACGC
CAACCTCTGGCTGGCCGCCTGGCTCAACGTCTTCTACTGCCTGCGGATCGCCCACTTCACCCACCGCCTGTTCTCCCTGC
TGCGGAGGAAGGTGGTGGCGCTCATGCCCTGGCTCCTGCGGCTGTCGCTGCTGGCGTGCCTGTGCTTCAGCCTGCCCTTC

TCCCGGGACATCTTCCAGGTCTACGCCAACAGCTCCGTGCCCGTCCCCACCCTCAACGCCACCGAGAAGAAGTTCTTCTA
CGAGACCAACCTGGCCGGCCTGGTTCTCCTCATGTACCTGGGCATCTGTGTTCCGCTCGTCCTGTTCGTGCTGGCGGCCG
CCCTGCTGATCCTCTCGCTCAGGCGACACTCCCGCCTCATGAGGAGCAAAGCCTCGGGCTCCAGGGACCCCAGCATGGAG
GCTCACCTGGGGGCCATCAAAGCGCTCAGCTATTTCCTCGTTCTCTACATCCTCAACGCCATCGCGCTGTTTCTTTCCAT
GTCCAACCTCCTGGGCCCCACCAGCACCTGGAATATTGTCTGCAAAGTCATCATGGCGGCCTACCCCGCCGGCCACTCCG
TGCTGCTGATCTTGAGCAACCCGGGGCTGAGAAGAGCCTGGAAGAGGTGCTAA
>SoarTAS2R41_NW_004545887.1:5347506-5346586
ATGCAACCAGCACTCACGTTCTTCTTCATGCTGCTCTTTGTCCTTCTGTGTCTCCTGGGACTTGTGGCCAACGGTTTCAT
TGTGCTGGCTCTGGGCAGAGAGCGGGTGTGCCGCGGGAGGCTGCTCCCCTCGGACCTGATCCTCCTGTGTCTGGGCGCCA
GCCGCTTCTGCCTGCAGTGGGTTGGCATGGCCAACAGCTTCTACTACTTCCTGCACCTCATGGAGTACAGACCCGCCCGC
CAGTTCTTCGGGCTGCACTGGGACTTCTTGAACACGGCCACCTACTGGTTCGGCACCTGGCTCAGCGTCCTCTTCTGCCT
GAAGGTTGCCAACGTCTCCCATCCCGCCTTCCTCTGGCTGAAGTGGAGGTTTCTGGGGTCCGTCCCCTGGTTGCTGCTGG
GCTCCCTCCTCGTCTCCTGCCTGGTCACCCTGCTGTTCTTTTGGGGGAACCGTAAAGTGCATCAAGGATTCCTCGCTAGG
AGACTTTCTGACAACGTGACCTACGGGGAGTGGAGCAGGAGGCTGGATCTATCCTACTTCCTGCCCCTCAAGCTGGTCAC
CTTGTCCATCCCCTGCTCCATTTTCCTGCTGTCCATCCTGCTGTTGATTGGGTCGCTGAGGAACCACGCGGGCAGGATGC
AGCATCACATCCGCAGCCCACGGGACCCCCGCGCCCAGGCACACACCCGGGCTCTGAAGTCACTCGTCTCCTTCCTCTTC
CTCTATGCTCTCTCCTTCCTGTCCCTGATCATCGACATCCTCGGGTTCTTCTCTGCGGAGAGTGACTGGTACTGGCCCTG
GCAAAATGTGATCTACCTGTGCACGTCTGTCCACCCCTTGGTCCTCATCCTCAGCAATCCCCGGCTGCAAGGGGTGTGTA
GGCACCTCGTTCTGTGGGTCAGGGGCCTCCAGGAGTCCTAG
>SoarTAS2R42_NW_004545902.1:15482960-15483949
ATGGAACTGCTCACTAGTTTGGAAACTCTCTTCCTGACACTAGCAATGATGGAATTCATCATTGGAATGTTGGGCAATGT
TTTCATTGGACTGGTAAACTGCTATGGATGGGTCAAGACCCGAAAGATCTCTTTCTTTGACCTCATCCTCACCGGCTTGG
CTGTCTCCAGAATCAGTCAACTATTGGTCTATTTTCTTGAGTCGCTTGTAATGGGACCATACCCAGAATTATATATCACT
TATAAACTTGCCAAACCTTGCAGTTTTCTTTGGAGAATAACTAATCACTTGACTACCTGGCTTGCTACCTGCCTTAGTGT
TGTCTACTTTCTTAAAATTGCTCAGTACTCCCATCCCCTTTTCCTCTGGCTCAAGAGGAGAATGAACAGAGTGGTTCTGG
TGACGCTTGCGTTTTCTTTGCTTTTTGTGATTTTTGACTTTTTATTGCTAGAAACATTTAATGATGCCTTCCAGGATTAC
CCTACAAGACACACACTCAATCTTACTTCATTTTCAGATAGAAGAAAATCTTTGCATGTTCAGTCCCTGATTTTTCTCAG
TTTGACATATTGCATCCCTATTGCGGTAACCCTGATCTCCTTGTTATTTCTGTTTCGGTCCTTGGTGAGACACACCAGAA
ATTTGCAACTCAACTCCTTGGGTCCCAGGGACACTAGCACAGAGGCTCATAAAAAGGCCATGAAAATGATGATGTCTTTT
CTCTTCCTCGTCATGGTTCACTCCATTTCTACGATCTCGTCAAACTGGATATTTTATATGTTTTGGAGTAGTAAGTTCAT
TAGGCTTCTCATGCTAGCAGTCTACATCTTTCCTTTAGGCCATTCGTTAATTTTGATTCTGGGAAACAGCAAGCTAAGAC
GGACAGCCTTGAAGGGACTATGTCATCTTCAAAACTTCTTTATGACTACATTTGGGCTTCTTAGAAGGACTCTCCACAGT
CTTTTCAGAAATAAGGACTTAAAGAAGTGA
>SoarTAS2R408F_NW_004545902.1:15695391-15694501
ATGGCTTTACTACTCTGGATTCTTTCCATCCTAGGAACTGCAACATTTATCCTAGGGAATTTCGCAAATGGCTTCATAGT
GCTGGTGAACTGTATAGACTGGAAAAAGAGACGGAAGTTCTCCTTAGTGGATCAGATACTCACTGCCTTGGCAGTCTCCA
GAATTGGTTTACTTTGGGTAATATTAATAAATTGGCAGGCAATTGTGCTCAATTCAGATTTATTTAATTTGAAGGTAAGA
ATGTTTGCTCATCTTGCCTGGATATTAATCAATCATTTCAGCAACTGGCTGACTACAAGTCTCGGTGTATTTTACTTACT
AAAGATAGCCAATTTCTCTAGCCTTCTATTTCTGTACCTAAAATGGAGAATCAGACAGGTACTTCTTGGAATAATATTAG
GAAGCTTGCCCTTTCTGATGTTCCACTTTGCCGTGGGGAGCATATATGACACTGTGCAGATAAATTTTGAAAGAAACACG
ACTTTGAGTACCAAATTGGTACAGGTTGTAAACATTTCAAATATGACTGCTGAGACCCTAGCCAATTTAATTCCCTTTGC
TCTGTCCCTCACATCTCTTGTCCTGTCCGTCTTCTCCCTGTGGAAACATCTCAAGAAGCAGCCGCTCAATGATAAAGGAT
CCCAGGATCCCAGCATCAAGGTCCACATTAGAGCCATACAAACTGTGATCTCCTTCCTTCTGCTGTTTGTCATTTTCTTC
CTGTGTTTGGTCATCTCAGTCTGGAGTTCTGTGAGGGTAGAGAAGAAAGCTATTGCTGTGTTCTGCATGGCTCTGGGAAC

CTTGTATCCTTCAGGCCACTCATTTATCCTGATTTGGGGAAACAAGAAGCTGAAACAGACCTTTGTGGCACTGTGGCACA
GTTTCAACTGA
>SoarTAS2R408G_NW_004545902.1:15679572-15678658
ATGACGGCACGCATTCTTTTCATCCTAGTAACCATACAATTTGTTCTTGGGAATCTTGCCAGTGGCTTCATAGTGCTGGT
GAATTGCATGGACTGGGTCAAGAGACAAAAGATCACCTTAGTGGATCGGATGCTCACAGCTCTGGCAATCTCCAGAATTG
CTTTGCTCTGGGTAACATTGCTAAATTTCTATGCAAATATGTTTAATACGGCAATCTATACTTTAAAAATTAAAATTATT
TGTTGTATTGCTTGGTCTTTAAGCAACCATTTCAGCACCTGGCTGGCTGCAAGCCTCAGCGTGATTTATTTGTTGAAGAT
AGCCAATTTCTCTAATCTTCTGTTTCTTTACCTGAAGTGGAGAGTTAAATGTATAGTAGTCACGATACTTTTGGGAACTC
TGGTAATTTTGGTTTTGAACCTTGCAGTGGCAACCACTGATGAATATATATGGATAGATGAGTGGAAAGAAAACGTGACT
AGGGAGAACAAATGGAAGGATTTTATACGGCTCTCCAACATGACCATCTTCACTCTACAGAATCTTACCCCTTTCATCAT
CTCCCTGACATCTCTTGTGCTGTTAATCATTTCCCTGTGGAAACATCTCAAGAAGAGGCAGTTCAAAGTATCCCAAGATC
CTAGCACCAAAGTCCATATCAGAGCCATGAAAACTTTGGTTTCCTTCCTCCTGGTCTTTGCCACGCACTCTCTAACTCTA
ATCATCGTAGCTTGGAATTCCAATAAGCTGCAGAACAAACGGATTCTCATGCTTTCACAGATTTGGGGCATCATATGTTC
TTCAAGCCACTCATATGTGCTTATTTGGGGAAACATGAAGCTAAAACATGCCTTTTTTTCAGTTTTATGGAAAGCAAAAA
CCTGGCTGAAGAACTGGAAACCATCAAGACACTAG
>SoarTAS2R408J_NW_004545902.1:15607375-15608367
ATGATCACTTTACTATCAAGCATTCTTACCATCCTACTAATGGCAGAATTTGTTCTAGGAAATTTTGCCAATGGCTTCAT
CGTGCTGGTGAACTACAATGACTGGGTCAAGACACGAAAGTTCTCCTTAGCAGATCAAATGATCGTGGCTTTGGCCATCT
CCAGGATTGGTTTTCTCTGTGTATTTTTAATAGCTTGGTATGCAGTGTTTTTTGATGTATTTTCATATAGTTTAGAAGTA
TTCATTATTATGAATGTGGCTTGGGTTGTAAGCTACCATGCTAGCATCTGGCTTACAACCAGCCTTAGCATATTCTATTT
GCTCAAGATCGCCAATTTTTCTAGCCTCCTATTTCTTTACTTGAAGAGGAGAGTTAAAAGAGTAGTTCTCATAATACTCA
TGGGCAGTTTGGTGTTTTCCATGTTTAATCTTTTCTTGGCAATCTTGGATGAGAATGTGTGGGTCAATCAACATCGTGGG
AACAGGACAGTGAGGAGCAAGAGGGAGAAAATTATTCGCCTTTCCAATATGTCTGTCTTCACCCTGACAACCTTCACAGC
CTTCATGATGTCCCTGACTTCGTTTGTTCTGCTGATTTTTTCCCTGTGGAAACATCTCAGGAACATGCAAGCAGAGGGCA
AAGAATCCCAGGATCCCAGCACCAAGGTCCACATCAGAGCCATCAAAACGATGACTTCCTACCTCCTGCTGTTGGCTAGT
TATATTGTGTCTCTGCTCATCTCAGTTTGGAGTTCTAACAGGATGCGGAACATAACCGTCATGTATGTCTTCCAGAGTTC
TTTAGGACTGTATCCTTCAATTCACTCATTTTTTTTAATCTGGGGAAATAAGAAACTGCAACAGTCTTTTCTTTCCTTTT
TGTGTCAGCTGAGGTTGAGGCTGACAGAAAGGAAAAGGACACTGTTTGCCTCACATAGAAAACAAAACGATGAATCTCCT
ACCATATCTAGTCCTATCACTTTTTCAATGTGA
>SoarTAS2R408K_NW_004545902.1:15599340-15600332
ATGATCACTTTACTATCAAGCATTCTTACCTTCCTACTAATGGCAGAATTTGTTCTAGGAAATTTTGCCAATGGCTTCAT
CGTGCTGGTGAACTACAATGACTGGGTCAAGACACGAAAGTTCTCCTTAGCAGATCAAATCATGGTAGCTTTGGCCATCT
CCAGGATTGGTTTTCTCTGTGTATTTTTAATAGTTTGGTATGCATTGTTTTTTGATGTATCTTCATATAGTTTAGAAGTA
TTCATTATTCTTAATATGGCTTGGGTTGTAAGCTACCATTCTAGCATGTGGCTTGCAACCAGCCTTAGCATATTCTATTT
GCTCAAGATCGCCAATTTTTCTAGCCTCCTATTTCTTTACTTGAAGAGGAGAGTTAAAAGAGTAGTTCTCATAATACTCA
TGGGAAGTTTGGTGTTTTTCATGTTTAATCTTCTCCTGTCAATCTTGGATGAGAATGAGTGGGTGAGTCAACATCGTGGG
AACAGGACAGTGAGGAACAAGAGGGAGAAAATTATTCACCTTTCCAATATGTCTGTCTTCACTCTGACAACCTTCACAGC
CTTTGTGATGTCCCTGACTTCGTTTGTTCTGCTGATTTTTTCCCTGTGGAAACATCTCAGGAACATGCAAGCAGAGGGCA
AAGAATCGCAGGATCCCAGCACCGAGGTCCACATCAGAGCCATCAAAACGATGACTTCCTACCTCCTGCTGTCGGCTTGT
TACTTTGTGTCTCTACTCATCTCAGTTTGGAGTTCTAACAGGATCCAGAACAGAACCGTCCTCCATGTCTTGCAGAGTTA
TTTAGGACTGTATCCTTCAATCCACTCATTTTTCTTAATTTGGGGAAATAAGAAGCTGCAACAGTCTTTCCTTTCCTTTT
TGTGTCAGCTGAGGTTGAGGCTGACAGAAAGGAAAAGGACATTGTTTGCCTCAGGCAGAAAACAAACTGATGAGTCTCCT
ACCCTGTCTAGTCCTACTGCTTTTTCAATGTGA
>SoarTAS2R408L_NW_004545902.1:15592102-15593100

ATGATCACTTTACTATCAAGCATTCTTACCTTCCTACTAATGGCAGAATTTGTTCTAGGAAATTTTGCCAATGGCTTCAT
CGTGCTGGTGAACTACAATGACTGGGTCAAGACACGAAAGTTCTCCTTAGCAGATCAGATGATCGTGGCTTTGGCCATCT
CCAGGATTGGTTTTTTCTGTGTATTATTAATAGTTTGGTATACAGTGTTTTTTGATGTATCTTCATATAGTTTAGAAGTA
TTCATTATCCTTAATATGGCTTGGGTTGTAAGCTACCATTCTAGCATCTGGCTTGCAACCAGCCTTAGCATATTCTATTT
GCTCAAGATCGCCAATTTTTCTAACCTCCTATTTCTTTACTTGAAGAGAAGAGTTAAAAGAGTAGTTCTCATAATACTCA
TGGGAAGTTTGGTGATTTTCATGTTTAATCTTATCCTGGCAATCTTGGATGAGAATGTGTGGGTCAATCAACATCGTGGG
AACAGGACAGTGAGGAGCAAGAGGGAGAAAATTATTCGCATTTCCAATATGTCTGTCTTCACTCTGACAACCTTCACAGC
CTTTGTGATGTCCCTGACTTCGTTTGTTCTGCTGATTTTTTCCCTGTGGAAACATCTCAGGAACATGCAAGCAGAGGGCA
AAGAATCCCAGGATCCCAGCACCGAGGTCCACATCAGAGCCATCAAAACGATGACTTCCTACCTCCTGCTGTCGGCTTGT
TACTTTGTGTCTCTACTCATCCCAGTTTGGAGTTCTAACAGGATGCGGAACAGAACCATCCTCCTAGTCTTCCAGAGTTC
TTTAGGACTGTATCCTTCAATCCACTCATTTTTCTTAATTTGGGGAAATAAGAAGCTGCAACAGTCTTTTCTTTCCTTTT
TGTGTCAGCTGAGGTTGAGGCTGACAGAAAGGAAGAGGACATTGTTTGCCTCATGTAGGAAACAAACTGATGAGTCTCCT
ACCTTATCTAGTTCTATTGCTTTCTCAATGCGTATATAA
>SoarTAS2R408M_NW_004545902.1:15576127-15577125
ATGATCACTTTACTATCAAGCATTCTTACCATCTTAGTAATGGCAGAATTTGTTCTGGGAAATTTTGCCAATGGCTTCAT
CGTGCTGGTGAACTACAATGATTGGGTCAAGACACGAACGTTCTCCTTAGCAGATCAAATGATCGTGGCTTTGGCCATCT
CCAGGATTGGTTTTCTCTGTGTATTTTTAATAGCTTGGTATACAGTGTTTCTTGATGAATCTTTATATAGTTTAGAATTA
TTCATTATTATTAATATGGCTTGGCTTGTAAGCTACCATGCTAGCATCTGGCTTGCAACCTGCCTTACCATATTCTATTT
GCTCAAGATCGCCAATTTCTCGAACCTCCTATTTCTTTACTTGAAGAGGAGAGTTAAAAGAGTAGTTCTCATAATACTCA
TGGGCAGTTTGGTGATTTTCATGTCTAATCTTCTACTGGTAATTTTCGATGAGAAATTGAGGATGAATCAATATCTTGGA
AACAGGACACTGAAGAGCAAGAGGGAGAAAATTATTCACCTTTCCAATATGTCTGTCTTCACTCTGACAATCTTCACAGC
CTTTGTGATGTCCCTGACCTCATTTGTGTTGCTGATTTTTTCCCTGTGGAAACATCTCAGGAACATGCAAGCGGATGGCA
AAGAAGATCAGGATGCCAGCACCGAGGTCCACTTCAGAGCTGTGAAAACTAAGACTTCCTACCTCCTGCTATTGGCTTGT
TACTTTGTGCCTCTACTCATCCGAGTCTGGAGTTCTAACAGGATTCGGAACAGAACCATCCTCCAAGTCTTCCAGAGTTC
TTTAGGACTGTATCCTTCAATCCACTCATTTTTCTTAATCTGGGGAAATAAGAAGCTGCAACAGTCTTTTCATTCCTTTT
TGTGTCAGCTGAGGTTGAGGCTGACAGAAAGGAAGAGGACATTGCTTGCTTCACGTAGAAAACAAACTGATGATTCTCCT
ACCTTACCTAGTTCTACTGCTTTTTCAATACGTATATGA
>SoarTAS2R408H_NW_004545902.1:15555230-15556156
ATGATAAGTCTAAGAACAGACGTTCTTTACATCCTTTTTCTGACAGAATTTATTCTAGGAAATTTTGCCAATGGCTTCAT
AGTGGTGGTGAACTGCTTTGACTGGATCAAGAGACAGAAGATTTCCTTAGTGAACCAAATGCTTGTGGCTTTGTCGGTCT
CCAGAATTGGTTTGCTCTGGATAACTTTAATGAGTCGGTGCGCAATATTGAAAGCAGATTTGTTTGGTTTAAGATTATTC
ATGATTGCTAATATAGTTTGGTTATTAAGCAACCATTTTAGCGTCTGGCTCACTGCTAGTCTCAGCGTATTCTACTTGCT
CAAGATAGCCATATTCTCTAGGGCTTTCTTTATTTATTTGAAGAGAAGAGTTAGAAGTGTGGTTCACATAATATTGCTGG
GGAGTTTGCCATTTTTGCTTTACATTATTACAGTGACAGGCATAGATGAAAATGCATGGGTGAATAGATATGAAGGAAAT
ATGACTTGCAAGACCAAATCAATGGACACGATGCACTTTTCAAATATGATTGTCTTCACACTAGCAAACTTCATCCCCTT
CGCCATGTCCCTGGCATCTCTTGTGCTATTAATCTATTCCTTGTGGAATCATCTCAGGAAGATGAAACTCAGTGGCACAG
AATTCCAAGATCCCAGAGTCAAGGTTCACCTCAGAGCCATGCAAACTGTGATCTCCTTTCTCCTGCTATTGATCAGTTAC
TTTGTAGCTATAATTATCTTATTCTGGCATCCCAAAACGGTGTACAACAAATGGGTCATTTTCTTCTGCCAGACTCTTCT
AGGGCTATGTCCTTCAATCCACTCACTTTTCCTTATTTGGGGAAACAAGAAGCTGCAACGGGTCTTTTTGTCATTTCTGT
GGAAGCTGAGGTTCAGACTGAAAGGAAGAAAGGAAGTGGAATGGTGA
>SoarTAS2R408A_NW_004545902.1:15545935-15546837
ATGATAACTTTACTGACAATTGTTCTTTCCATCCTATTAGTAGCAGAATTTGTTCTGGGAAACTTTGTCAATTGCTCTAT
AGTGCTGGTGAACTGCAAGGACTGGATTGAGAGACAGAGGATCTCTTTCGTGGATCAAATACTCATGGCTTTGGCCATCT
CCAGATTGGTTTTGCTCTGGGTAATAACATCAAACTGGATTACAGCTATGTTTTTTGAAGACTCATATACTTCAGAAGCA

TATATTATTTTTGATATTACATGGACAGTAAGCAACCATTCTAGCACCTGGCTTGCTACAAGCCTCAGCATATTCTTTTT
GCTCAAGATCGCCAGTTTCTCTAGCCCTTTATTTCATTATTTAAAAAGAAGCATTAAAAAAGTCCTTCTCATAATGGCAT
TGGGATCTTTGGCAATTTTAACTCTTACTATTGCAGCAGCCAGCAAAGCTGACCGTGTGTGGACACCTATATCTAGAAGA
AACACAACTCGGGACATCGAAAGGAAGGAGATAATATGCCTTCCTGTTATGATCGCTTTCACAGCCACACAGCTCATCCC
TTTTACTATGTCTCTGACATCTTTCGTGCTGTTACTCTTTTCCCTGGACAAACATCTCAAAAGGATGAAAAGGAATGGAG
AAGGATCCCGAGATCCCAGCACCACGGTCCACATCAGAGCCATGCAAATGGTCATCTCCTTTCTCTTGCTTTATGCTTGT
TACTTCGGGGCCACAATTGCCTCAATTTGGGGTATAAACAGGCAACTGAATAATACAGTCCACCAGGTTTGTGAAATTAT
TTTAGCACTGTATCCTTCCAGCCATTCATTGATTTTGATTTGGGGAAATAAGAAGCTCAAACAAGCCTTTATGTCATTTT
TGTGGCAGTGGAGGATGCAGTGA
>SoarTAS2R408B_NW_004545902.1:15534612-15535529
ATGATAACCTTTCTGACCATTGTTTTTTCCATCCTAACAATGACAGAATTTATTCTAGGAAATTTTGCCAATGGCTTCAT
AGTGCTGGTGAACTGTATTGACTGGGTCAAGAGTCGAAAGGTCTCCTGTGTAGATCAAATACTCACAGTTTTGGCCATCT
CCAGGATGGGTTTGCTCTGGGTAATAGAAATATATTGGATTGCAACCATGTTTAATGCAGCTTCATGTAATTTAAAAGTA
TTTATTATCGGTAATATCATCTGGATCGTGAGCAACCATTTTAGCATCTGGCTTGTTATTAGTCTCAGCCTACTCTATGT
GCTCAAGATAGTCAGTTTTTCTAGCGTTCTATTTTTTTATTTAAAAAGAAGAGTTCATAGAGTTATCCTTGTCTTACTGC
TGGGGAGTTTGCCCTCTCTGATCTTTCATCTCGTAAAGGTAAGCGCAGATGAAAATGTGTGGAAGAATGAATGCGGTGGA
AACGTGACTCGGAAGACCAAGTGGAATGACATTGTACGACTTTCCAGCATGTCTGTCTTCCTAGTATCAAACTTTATACC
TTTCACTATGTCTCTGATATCTTTTCTGCTGTTAATCTTTTCCCTGTGGAAACATTTCAAGAAGATGAAAAAGAATGGGC
AAGAACCTCCAGATCTCAGCTCCAAGGTCCACATCCGAGCCCTGCAGACTGTGACCTCCTTTCTCTTGCTCTATACCAGC
TACTTTGTGACGGTAATCATCTCAATCTGGAATTCAGAAATGTTGAAAAACGAACCATTCTTTCATGTTTGCCAGATTCT
TTTAGCCCTGTATCCTTCAAGTCATTCATTTATCCTGATTTTGGGAAATAAGAAACTAAAAGAAGTCTTTCTGTCACTTC
TGTGGCAGCAGAGATTCCAGTCAAGAGGAAGGAAATAA
>SoarTAS2R408D_NW_004545902.1:15502192-15503112
ATGATAACTTTACTATGGTACATTCTCACCGTCCTAGTAGTTGTGCAGTTTGTTCTAGGAAGTTTTGCCAATGGCTTCAT
AGTGCTGGTGAACTGCACAGACTGGATCAAGAGACGAAAGTTCTCCTTAGTGGATCAGGTTGTCACAGCACTGGCGATCT
TCAGAGTTAGTTTTCTCTGGGTAATATTAATAAATTGGTACTCCGTTGCAAGCAATCCGGCTTTAAGTAGCTTAACAGTC
TTTCGAATCATCATTCATATCTCATGGACAGTGAACACCCATTTTAACACCTGGCTTGCTACCAGCCTCAGCATACTTTA
CTTGCTGAAGATAGCCAATTTCTCTAGCCTTCTGTTTTTCTACCTAAAGCGGAGAGTTAAAAGTGTCCTTCTTGTAATCC
TGGTGGGGAGTTCGGTCTTTTTGGCTTCTCAGCTTGTATCGGTAAGCAACCACGAGATTGTGCTCTCTAATGAAAAGGAA
GGAAACGTGACGTGGAAGACTAAAGAGAGGCACTTGATACTGGTTTCAAATTCAGCAGTGTTCTTGCTAGCGAACCTCAT
CCCCTTCACCATGTCCTTGGCGTCTCTTGTGCTGTTAATCTATTCCCTGCGGAAACATCTCAAGAAGAGGCAGCTCAGTG
GCCAAGGATCCCAAGATCCCAGCGCCAGGGTCCACATCAGAGCCTTGCAGACCATGGTCTCCTTCCTCCTGCTGTTTGCC
ATTTACTTCTTTGATACGCTGGTCTCATTTTTGAATTTTAATAAGCTGATGAACAAACTGGTCCTCATGTTTTGCAATGC
TCTATTAGTCCTGTACCCGTCAAGTCACTCACTCATCCTGATTTGGGGAAACAAGAAGCTGACACGGGCCTTCCTGACAT
ATTTGCGGCAACTGAGATCCGGGCTCCGAGACAGGGAGTAA
>SoarTAS2R408E_NW_004545902.1:15510085-15510996
ATGGCAACTTTGAATTTTATTTCCTTCCTAGTAATAGCAGAATTTCTTCTGGGGAATCTCGCCAATGGATTCATAGTGCT
GGTGAATTGCAATGACTGGATCAAGAGACAAAAGATCTCCTTAGTGGATCAAATGCTCACTGCTTTGGCTGTCTCCAGAA
TTAGTTTGCTGTGGGTAGTTTTAAGTTATTGGCATTCAACTGTATTTAATCCAGCTTTATTTCATTTTAAAAACGTAATT
ATTGTTCATATTGCCTGGACATTGAGCACCCATTTTAACATCTGGTTTACTACAGGTCTGAGTATATTTTATTTACTGAA
GGTAGTCAATTTCTCTAGTTTTCTATTTCTTTACCTAAAGCAGAGGGTTAAAACTGTACTTCTCAAAATACTGTTGGGGA
GTTTGATCTTTTTGATTACGCAACTTGCATCTTTAAGCATTCATGAGATTATGATAATAAATGGTAGTGAAGGAAACGTG
ACTTGGAAGGCTGAATTCAGACATATTGTAAAGGTTTCACATTGGACTATACATCTACTAGCTAAATTAGTACCCTTTAC
CATGTCTCTGACATCTTTTGTGCTGCTTATCTTTTCTCTGTGGAAACATCTCAAGAAGGTACAGCTCAGAGGCCAAGGAT

GGCAAGATGCCAGCACGCAGGTCCATGTCAGAGCCTTGAAAACCTTGGTCTCCTTTCTCCTTCTCTATGTAATTTACTTC
CTGGAAGTAATCTTCTCAATCTTGACTATTGATAAGCTCCTGAACAAAACGATTCAAATGATTTACACTACTCTCTTCAT
CCTCTATCCTTCAGGTCACTCGGTTTTTCTGATTTGGGGAAACAAGAAGTTGAAACAGGCTTTTCTGTTATTTCTGTGGT
GGCTTAGGTTCCCTCCGAGAGGAAGGGAGTAA
>SoarTAS2R408C_NW_004545902.1:15621335-15622273
ATGATAACTCTAAAACCAAGCCTTCCTTACATTCTTTTCATGACAGAATTTGTTCTAGGAAACTTTGCCAACGTCTTCAT
AGTGCTGGTGAATTGCATTGACTACATCAAGAAGCAAAAGATTTCTTTAGTGGATCAAATGTTCACAGGTCTGGCACTCT
CCAGAATTGCTCTGCTCTGGGTAATACTAATAAGTTGGTTTACAATATGGCTTGATAAAACTGCATATAATTTAGAAGCA
GATACTGTTGTTTATATTGTCTGGGCAATAAGTAGCCATTGTAGTATTTGGCTTGCTACTAGCCTTAGTGTTTTCTATTT
GCTCAAGATAGCCAGTATTTCTAGCCTTCTATTTGCTTATTTTAAAAACAGAGTAAATAGAACAGTTCCCCTAATACTCC
TTGGAAGTTTTCCCTCTTTGGTTTTTCATCTAGTGGTGGCAAACACAAGTCACAAGGTATGGATGAATGAATACAATGGA
AATATGACTTGGGAGAACATAAGGAAAGATATTGTGTACTTTTCTAGTATACTTGCCCTCATCATAGCAAACTTCATACC
CTTTGCTATGTCCTTTATATCTTTTGTGCTGTTACTCTTTTCACTGTGGAAACATCTCAAGAAGATGCAGCTCAATGACA
AAGGATGCCAAGACCACAGCACGAAGGTCCACATCAGAGCCATGCAAACAGTCATCTCCTTTCTTTTGCTACTTGTCAGT
TACTTTGTGGCTTTAATCATTATGTTCTTGGGTACAGCAGTGCTATCAAACGGGCCCATTATCTCCTATTGCCAGACTCT
TCTGGGACTCTATCCTTCAGGACACTCAATTATCCTGATTTGGGGGAACAAGAAAATGAACCTGGCCTTTCTGGCGTTTT
TGCGGCAGCTGAAGTTCCTGCTGAAAGTGGGAAGGTGTGTGTGTTCACGTAGAAAATGA
>SoarTAS2R408I_NW_004545902.1:15689560-15688652
ATGGCTTTACTACATTGTATTTTTCTATCTTAATCACAGCAGAATTTGTTCTGGGAAATTTCGCCAACGGTTTCATAGT
GCTGGTGAATGGAATTGACTGGATCAAGAGACAAAAGATCTCCTCAGTGGATCAAATACTCACAGCTCTGGCAGTCTCCA
GAATTGGTTTGCTGTGGGGACTATTATTAAATTGGTATGCAAGTGTGCTCAAGTTGGCTTCCTGTAATTCAGAAGTAAGA
CTTTTTACTCAAATTGTCTGGATAGTAAACAATCATTTTAGTGTCTGGCTTGCTACTAGTCTCAGCATATTTTATTTGCT
CAAAATAGCCAATTTCTCGAGCTTTCTATTTCTTTACCTCAAAAGGAGGGTTAACAGTGTGCTGAGAGTAGTTTTGCTAG
GGAGTTGGCTCTTTTTGTTGTCACACTTTGGAATAGCGAGTATCTATAAGATTATTCCAAAAAATAGAGGAAATGAGACT
TGTAAGACTGAAGTAATGGAAGTTGAGCAGCTTCCCAATTTGGTTGTCTTCACGCTGGCAAACTTCGTCCCCTTCACCAT
GTCGCTGGTGTCCCTAGTGCTGCTGATCTCTTCCCTGTTGAAGCACCTCAAGAAGATGAACGTGAATAGCAAAGGATCCC
AAGATCCAAGCACCAAGGTCCACATCAGAGCCATGCAGACCGTGACCTCCTTTCTCTTCTTGTTTGCTGGTTATATACTA
GCTCTAACCATCACGGTTTGGATTTCGGACAACCCCCAGAGCAAGCTAGCCTTTATGTTTTGCCAAATGCTTGGAATCCA
GTATCCTTCAAAGCACTCGTTTGTGCTTATATGGGGGAACAAGAAGCTGAAACAGATGTTTCTGTCATTCCTGTGGCATC
TAAGGTACCAACTGAGAGGAAAGAAGTAA
>SoarTAS2R62_NW_004545887.1:5390843-5389947
ATGCCCTCCTTACCCACTTTGGTCTTCATGGCTTTCTTTTTCTTGGGGTCCTTGGTTGCCATGTTCCAGAATGGCTTCAT
GGTCACCGTGCTGGGGAGGGAATGGGCCCGAGGCCGGTCGCTGCCCACCGGTGACATCATTGTGACCTGCCTGGCTGCCT
CCCGGTTCTGCCTGCATGGCATGTCGGTCTTGAACAACCTGCTGTTCGTCTTTGGGTTTTGTCCCCAAGTGAATGCTTTC
AACATCCCCTGGGACTTCGCCAACACGCTCACCTTCTGGTTCACTGGCTGGCTGGCGTTCTTCTACTGCGTGAAGATCTC
CTCCTTCTCCCATCCCGTCTTCCTCTGGCTCAAGTGGAGGATTTCTCAGTCCATCCCCAGGCTGCTCCTGGGCTCCCTGG
TCATCGCGGGGGTGTCAGTGAGCTCATCCATTGTGGGGAATTCAAAGCTTGGACCCCTGATTGCTCTCCAGATATCCAGC
GGAAACGACACCTTGGCTGATAGAATCCTGGTCGCCCACCGGCTCATCTTCCTACCTCACTTACTGCTTGTGTTGTCTGT
CCCCTTCGTCCTCTTTCTGGTGTCAACTCTCCTGCTCATGTTCTCACTGCACCGGCATTTGGGGAGGATGAAGGAGAGCA
GGCTGGGCCAGAGAGACCCCAGCACCCAGGCACACACCATGGCCCTGAAGTCACTCGCCTTCTTCCTCGTCTTCTGCACA
TGGTACTTCCTGTCCATTGTTGTTATACTCATGAAGGGCGTAAACTTCCCGAACCTCTGGCACTGGGTCTGGGAAGTGGT
AACCTACGCGGGTGTCTGTCTACACTCCAGCATCCTGATACTCAGCAGTCCAAAGCTGAGAAAAGCCCTGAAGATGAGTC
TTAGGAAACCCTGCTGA
>SoarTAS2R67_NW_004545887.1:15491783-15492781

ATGCCGTCAGGAATGGAAAATGTTTTTCTGATAGTGACAATGGGAGAGTTCCTAGTCGGAAAGGTGGGGAATGGGTTCAT
CGTACTGGTGAACTGCATTGACTGGGGGAAGGGCCGGAAGCTCTCGTCCGTAGACCATCTCCTCAGCAGCCTGGCCCTCT
CCAGAATCAGTCTTCTTTGGGTAATGCTATTTGAATCAATTGCACTGGTGTTATGGCCACATCTATACACCAGTAACAAA
ATAACAGCATTCATTAGTACTTCTTGGGCCCTGAACAATCACTTTTCTATCTGGATTGCAACCTGTCTGAGTGTTTTCTA
CTTTTTAAGAATAGCCAATCTCTCCCACCCCTCCTTCCACTGGCTGAGGCAGAGAATCAGCCGAGTGTTAGCTGTGCTTC
TCCTCGGCTCTTCACTCTTACTGTCTGTCAACTTTGCGTTGGTAGATACGTTTATTGATTTCTGGACTAACTGGCACAGG
ACACATCAAGGAAACTCAAGCTGGCCCTCGGGTTTGACGATAACTCTGTACGTTAACAGCTTGGTGGCGTATAACTTGAT
CTACCTGATCCCCCTGCTCCTCTCGCTGGGCTCCCTGGTGCTTCTGTTTGTCTCCTTGAAGAGACACACCAGGAACCTGC
AGATGAGCGCCAGCTGCTCCGACGCCAGCACTGAGGCCCATAGAAGGGCCATGCAGATGGTGATGTCCTTCCTCGCCCTG
TTCCTGGTTCACTTGGCTACCACCATATTAGTTGGCTGGGCTTTCTTCATGCCATTCAAATATCAAGCCCAACTGGCTTT
CTCCGTGACAATGAATCTGTTTCCTGCCGGCCACTCACTGATCCTGATTAGGAGCCACAGCAAACTGCGACAGACTGCCT
TGTGGCTCCTGGGGCATCTTGAATGCAGCCTGAGAAGGAAAACAGGGCGAAACCTTTTCTTCACAGCCCTTTGGCAGAAT
TTTCTCTCTCTCAGGAGAATTAAGTTAACGAGACAGTGA
>SoarTAS2R372A_NW_004545902.1:15660369-15659443
ATGCCAAGTATCATTGAAAGCATTTTTATCACCATCGAAGTTTTAGCATTCGTAACGGCTATCTGGGGAAATGGATTCAT
TGTTCTAGTGATTGGTGCTAACTGGGTCAAAACCAAGAAAATCTCCTGGTGTGACTTCCTGTTCCTAAGCTTTGGCATCT
CCAGAATTGGCATGTTATGTATGATAATTGAAGAGGGCATTAGATTAGTGTTCTATCCAGAAATATATGACAATGACAGA
ACAAAAGCAACCATCATTGATGTCTTATGGCAACTGAACATCTCCTTTAGTACTTGGTGTACGACCTGCCTTGGCATATT
TTATTTCTTCAAGCTGTCCAGCTTTTCCCACCCTCTCTTTCTTTGGCTGAAATGGAGAAGAAACAGAATTTTTCTTGTCA
TTCAATTGGTGTTCCTTCTCTCTCTGATTGGTAATATTATGAGCATAATATACAATCAAACTTGGGCATCTGAGTATTTA
AAAATGGAAAGAAACTTGACTTTGCAAGAATGGAGGTATCAAAAACAATTCTTCAAGAAACATGTACTCCTCAACCTGGG
ATCCCTCACTGTATTGGCATTGTCACTTATTTCCCTTTTTTTGTTAACCTTTTCCTTAGCAAGACATGTCCAGAGGATGA
TGCATCATGCTGACGGATCTGGAGACCTCAGCACAGGGATTCCTGTGAGAGCCCGAAATACGATGGCTTCTTTTCTCATC
CTCTTCATCATACAGTTTGCGTCCACTTTCTTAGTAATTTGGATTTATGCCACAATGGAAAACCTGATTGGTGTGCTTGT
TATTGAGACTCTCATTGCATTTTATTCTTCAGTTCATTCCTTTACTATGATTCAAGGGAACAGAAAACTGAAGCAATCTT
TTGTGAGCTTAAGGGAAAAAATTGAATTTTGCATCAAGGGCATATAG
>SoarTAS2R372C_NW_004545902.1:15649729-15648794
ATGCTAAGTATCATTGAAAGCATCTTTATCACCATCGAAGCTTTAGCATCCATAACAGCTATCTGGGGAAATGGATTCAT
TGTTCTAGTGATTGGTGCTGACTGGGTCAAGACCAAGAAAATCTCCTGGTATGATTTCCTGTTTCTAAACTTTGGCATCT
CCAAAATTGGCATGATATGCACAGTAATGGAAGATAGCATTAGAGTAGTTTTCTACCCAGAAGTATATGGCGATGATAGA
ATAGCATCAGTCATTATTGACTTCTTAAAGAATCTGAGCAACTCCTTTAATACTTGGTGTACAACCTGCCTCGGCATATT
TTATTTCTTCAAGCTGTCCAGCTTTTCCCACCCTCTCTTTCTTTGGCTGAAGTGGAGAAGAGACAAAATTTTCCTTGTCA
TTCAATTGGGGTTTCTTCTCTCTCTGATTTGTAATATTATGAGCATAAAATATGGTCATATTCGGGTCTCTGAGTATTTT
AAAATGGAAATAAATGTGACTTGGCAAGAATGGAGGCATCAAAAACAACACTTCAGAAAATATATACTCCTCAACCTGGG
ATCCTTCATTTCCTTGACATTGTCATTTATTTCATTTTTTTTGTTAACCTTTTCCTTAGCAAGGCATGTCCGGAGGATGA
TGCATCATGCCAATGGATCTGGAGACACAAACACAGAGATTCCTGTGAGAGCCAGAAATACGATGGTTTCCTTTCTCATT
CTCCTCATTATACACTACGTGACAACTTTATTAATAATTTGGATTTATACCACAATGGAAAACCTGATCGGTGTGCTTGT
TATTGAGACTCTCATTGCATTTTATTCTTCAGTTCATTCCTTTACTATGATTCAAGGGAACAGAAAACTGAAGCAATCTT
TTGTGAGTTTAGGAAAAAAGTTAAATTTTGCATCAAGGGAATATAGTTCTTCATAG
>SoarTAS2R7CP_NW_004545902.1:15812211-15813309
ATGCCAGGTGAGGGGAACAGCACGTTAGTGATCATAGCAGCTGGAGAATTCTCCATGAGAATCTTAGGGAACGCCTTTAT
TGCACTGGTAAACTGTTTGGGCTGGATGAAGGAAAGTAAGATGGCCTCCATTGATTTAATTCTCACGTGTCTGGCTATTT
CCAGAATCTGGCTACTGTGAGTAATAATGTTAGACCGCTTTATGTTGGTGCAGTATCCAAATGTCTACACTGCCAGTAGA
CAAATGGATTCATTGACTTCTTCTGGACACTCACCAACCATCTCAGTGTGGGGTTTGCCACCTGCCTCAGTCTCTTCTAT

TTCCTTATCATTGTATCAGTCATCCCATGGTTCATCGATTTACTTGAGTGAGCTCCAGTAAAGTCTCCACTTGTCCCGGT
CCTGAGATTTTAGCAGCCTCTCCTTACCCACTTTTCCCAAGGATTGGAGGAAATTTTCAGCGTCAGGGAATTGAGACCTG
TTATTGTTACTGTATTTGGCATATCCAATATGCCACAGGGAGCTTGCTACGCTCTTCTGTGCAATGACAAATACAAATAT
TTCCTCAAGATAGCTAATTTCACGCATTCCCTTTTCCACTGGATGAAGTGGAGAATCGACCGGGTGGTTCTTAGGATCCT
GCTGGAGTGCGTGGCCCTCTCTGTGTTGATCAGCCTTGCTGTCACTGAGAAGTTTAGTGATGATTTTAGGTACTGTATCA
AGGCAAAGAAGAGAAGAAACAGAACATTGAAGTGCAGTGTAAATAAAGCTCAATATGAGTCCACCAAGATATTTCTCAAC
CTTTTAACAATATTTCCTGTTTCTATGTCACTGGTCTCCTTTTGCCTCTTGATCCTCTCCCTGTGGAAGCACACCAGGAA
GATGCAGCTCAGTGCCAGCGGACACAGAGACCCAAGCAGAGATGCCCACTTGGGAGCCATGAAAACTGTCATCACCTTCC
TCCTCCTGTTCATTGTCTACTGTTTGGCCTTTCTCATAGCCACCTCCAGCTGCTTTATGCCAGAGAGTGAACTAGCTGTG
TTGGTTGGGGAGCTGATAGCTCTAATTTATCCTCCAACCCATTCATTTATTCTAATCCT
>SoarTAS2R7DP_NW_004545902.1:15790555-15791550
ATGTCAGGTGACGTGAACAGCACCTTAATGATCATAGCAGCTGGAGACTTCTCCATGGGAATCTTAGGGAACGCCTTCAT
TGCACTGGTAAACTGCTTGGGCTGGATGAAGAAAAGGAAGATGGCCTCCATTGATTTCATTCTCTCGTGTCTGGCTATTT
CCAGAATCTGGCTACTGTACACAATAATGCTAGACTGCTTTATGTTGGTGCAGTATCCAAATGTCTATACTGCCGGTACA
CAAATGAGAGTCACTGACTTCTTCTGGACACTCACCAACCACCTCAGTGTGTGGTTTGCCACCTGCCTCAGTCTCTTATA
CTTCCTATCTTCTACTTATTCTTCTAAAATTAGCTATCTTCTACTTCAAGATAGCTAATTTCACACATCCCCTTTTTCTC
TGGATGAAGTGGAGAATCGACCGGGTGGTTCTTAGGATCCTGCTGGGGGGCGTGGCCCTCTCTGTGTTGATCAGCCTTGC
TGTCACTGAGAATCTGAGTGATGATTTGAGGTACTGTATCAAGGTAAAGAAGAGAAGAAATAGAACATCTGAGTGCAGTC
TATAAAGCTCAATATGAGTCCACCAAGATATTTCTCAACCTTTTGACAGTGTTTCCATGTTCTGCATCACTGGTCTCCTT
TTGCTTTTTGATCCTCTCCCTGTGGAAACACACCAGGAAGATGCAGCTCAGTGCCAGCGGACACAGAGACCCAAGCAGAG
ATGCCCACTTGAGAGCCATGAAACCTGTTATCACCTTCCTCCTCCTTTTCATTGCCTATGGTCTGACCTTTCTCATAGTC
ACCTACAGCTACTTTATACCAGAGAGTGAACTAGCTGTGTTGGTTGGGGAGTTGATAGCTCTAATTTATCCTTCAACCCA
CTCATTTATTCTAATCCTAGGAAACAATAAACTGAGAAAAAGCATCTCTAATGGTGGCATGAAAGATGAAGACAATTCTA
ACAACAGAAGGATATTTGAAGAGTCTCAATTTCTAG
>SoarTAS2R7EP_NW_004545902.1:15756695-15757645
ATGTCAGGTGAGGTGAACAGCACGTTAGTGATCATAGCAGCTGGAGAATTCTCCATGGGAATCTTAGGGAACACCTTCAT
TGCACTGGTAAACTGCTTGGGCTGGATGAAGAAAAGGAAGGTGGCCTCCATTGACTTCATTCTCACGTGTCTGGCTACTT
CCAGAATCTGGCTACTCTGCGCAATAATGCTAGACTGCTTTATGTTGGTGCAGTATCCAAATGTCTACACTGCCGGTACA
CAAATGAGAGTCACTGACTTCTTCTGGACACTCACCAACCACCTCAGTGTGTGGTTTGCCACCTGCCTCAGTCTCTTCTA
CTTCAAGGTAGCTAATTTCACGCATCCCCTTTTCCTCTGGATGAAGTGGAAAATCGACTGGGTGGTGCTAGGATCCTGCT
GGGGTGCGTGGCCCTCTCTGTGTTGATCAGCCTTGCTGTCACTGAGAATCTGAGTGATGATTTGAGGTACTGTATCAAGA
TAAATAAGAGAAGAAATAGAACATCTGAGTGCAGTCTATAAAGCTCAATATGAGTCCATTAAATTATTTCTCAACCTTTT
GACAGTGTTTCCCTGTTCTGTATCACTGGTCTCCTTTTGCCTCTTGATCCTCTCCCTGTGGAAGCACACCAGGAAGATGC
AGCTCAGTGCCAGCGGACACAGAGACCCAAGCAGAGATGCCCACTTGGGAGCCATGAAAACTGTCATCACCTTCCTCCTC
CTGTTCATTGTCTACTGTTTGGCCTTTCTCACAGCCACCTCCAGCTGCTTTATACCAGAGAGTGAACTAGCTGTGTTGGT
TGGGGAGCTGATAGCTCTAATTTATCCTTCAACCCATTCATTTATTCTAATCCTAGGAAACAATAAACTGAGAGAAGCGT
CTCTAAGGGTGCTATGGAAGATAAAGACGATTCTAACAACAAAAGGAGATTTGAAGAGTCTCAATTTCTAG
>SoarTAS2R8BP_NW_004545902.1:15801505-15802407
ATGATAGCAGGAAGCCTTGCAAATTCATGAAATGCGATCTTTTAAAATTTGCATTAGAGCAGATTTTAGCTCAACATTTC
AGTTTTCACTGACTAAATCCTGGATCGACTCTATTCAGAAGAACAAAACCTCCTCACTTAACTACATCCTCACAAGTCTG
ACTATTTCCAGAACATGTTTGCTCTTCCTAATAGTAGTAGGTGACACTGTAGTGATACTCTATCCAGATTTTTACGAAGA
TGTGAAACTAGTGGGAGTACTCAGTGCTTCCTGGATACTTATTAACTCAAGTTTGTGGTTTGCCACCTGCCTCAACATCT
TCTATTTACTGAAAATAGCGAATTTCTCCAGTCCACCTTTTCTCTGGTAAAGTGGAGGATTGACAGGGCGACATACTGAA
TCTTGCCGGCAGGCTTGACCATTTGCTCCTTGAGCAGCTTTCTGACAGCAATGCCGAATTATTATAAAGACAAAATATCA

AAGAAACATCACTGAAATGTTCCATGTGAGTGAAATTAAACATTCCAACCCATCAACTCTCCCTAACCTTTTGGCAGTTG
TTCCAATTACTGTGTCATTGATTTCATTTTCCCTGTTAATTTTGTCTCTGTGGAGACACACCAAGCAAATGAAACTCAAT
GTTACAGGCTGGAGAGACCCCAGCACAGAAGCCCATATTACAGCCATGAAAAATGTGACTTCTTTTCTCTTCCTCCTTTT
GTATACTACGTGGCTTGTCTTTTAGTAACTTATTGCCACCTTCTTCTATTGAAGAAGAAAGTTTAGCTTGTAGGCTTGGA
AAGATTTTAATGATTCTCTACCCGTCTGCCCACTCCCTTATTTAAATATTTGGAAATAGCAAGCTGAGGCAAGCACCTGT
CAGGATATTAACACACAAGGGTG
>SoarTAS2R8DP_NW_004545902.1:15779812-15780703
ATGTTCACTAAAGAAGATTCATAATAATTATTATGGCTGGGGTATATCTATGGTAGGCATGTTGGAAAATGGGTACATTG
GACTAATGTCCTGGATCGACTATTTTCAGAAGAATAAAAGCTCCTCACTTAACTACATCCATACAAGTCTGACTAGTTCC
AGAACATGTTTGCTCTTCCTAATAGTAGTAGGTGACACTGTAGTGATACTCTATCCATATTTTTACGAAGATGTTAAACT
AGTGGTAGTACTCAGTGCTTTCTGGATACTTATTAACTACTCAAGTTTGTGGTTTGCCACCTGCCTCAACATCTCCTATT
TACTGAAGATAGCGAATTTCTCCAGTCCACCTTTTCTCTGGTAAAGTGGAGGATTGACAGAGCGACATACTGAATCTTGC
CTGTGGGCTTGACCATTTGCTCCTTGAGCAGCTTTCTGACAGCAATACCGAATTACTATAAAGACAAAACATCAAAGAAA
CATCACTGAAATTTTCCATGTGAGTGAAATTAAACATTTCAACCCATCAGCCCTCCCTAACCTATTGGCACTTGTTCCAA
TTACTGTGCCATTGATTTCATTTTTCCTGTTAATTTTGTCTCTGTGGAGACATACCAAGCAAATGAAACTCAATGTTACA
GGCTGGAGAGACCCCAGCACAGAAGCCCATATTACAGCCATGAAAAATGTGACTTCTTTTTTCTTCCTCCTTTTGTATAC
TACGTGGCTTGTCTTTTAGTAACTTATTGCCACCAAATAGAAGAAGAAAGTTTAGCTTGTGGGCTTGGAAAGATTTTAAT
GATTTTCTACCCGTCTGGCCACTCCCTTATTTTAATATTTGGAAATAGCAAGCTGAGGCAAGCACCTGTCAGGATATTAA
GATACAAGGGCG
>SoarTAS2R9P_NW_004545902.1:15796943-15797550
ATGCCACATGCAGTGGAGACCATCTATATGATTTTCATTGTTAGAGAATTGACTCTAGGAATCTGGGGAAATGGGTCCAT
TGCAACGGTGAGCTACATAGGTTGGGTCCAAAGCAGAGCTATTGCCTTGACTAGCATCCTGGTCACTGTAGCCACCTCTA
GAATCTGTTTGTTGTGTGAAACGGCCTTTGATGGCTTTGTCATGCTGCTTTCTCCAGATACATGTGCCCACGGTGAGCTA
ATGAATATTTTGGATATGTTCTGGACCCTTGGCAATCATTTAAGTATCTGGTTTGCTTCTTGCCTCAGCATCTTGCATTT
ACCCAAGGTAGTAAATATACCTCATCCAGTCTTCCTTGGCTTAAGCTAAAGATGACCAAGATCATCTCTGGGATTACTTG
GGTGTCCTTTCCCATCCCCTTAGTTATTAGTTTCTCACTGATGATGAGTCCTGGTGTGACTTTAAGTTCCACCATGAAGA
AACAAAACTTGGGAATCTAAAGTGAGTAAAATTCCCAATGCTTTCAAACAGATTGTTCTGAACATAGGGGCTGTTGTTCG
CTTTAGTCTTTTCCTGATCTCATTTTCTTACAACTTTTGTCAGAACTT
>SoarTAS2R16P_NW_004545860.1:773561-774403
AAGGGTGTTATCTATCCAGTTGTCTTCTTTATGGTCACCTGTGTACTCAAATTCATGTTCAATGTGAGAGCAGCTTTCTT
ATTGCAAAGCTAGGCAGAGAGTGGGCGCAGGTTCAGAGCTTGTTATCTTTGAAAATGATTTTAACCAACTTGAGCATCTG
CCGCTTCTGTATAAGGTAGACAAGTCTGCTGCACTACTTGTGTTTCTGTTTCAACCCTAATTTTGCATTTGGACACTTTT
CTATCTTTTAGAAATGAACTAACACTCTTACATTCTGGCTAATCAGCTGGATTACTATCTTCTACTGTATCAAAATATAT
CTCTCCTTGATGAAGTATATGATTTAAAAATTTCTTCCCTGGATATTTCTGGACTCATTGCTTATATCTACTGTGTCGAT
CATCCATTAGGCTATTACAAATCACATTCAGATTTTCTTAATTACCATGCAACATTTTTCTACAAACAGCTTCATAATGG
AGAAACTGAAGATGTATCAACATTGTTATTTACCTCAGCAGATATTCTCTTGGTTACTCCTTTCCTCCTGTCCTCTACAT
CTTCTCATGGTTTCACTGTCTCAGAGGATAGAGCAGATGCACCATCACAACACTGATCACTGCAACTCCACCCTGAAAGT
TCATGCAGTGATATCTCTTGCCATCTTCCTCATCTCCCACCCTCTTACTTTTCTAGTCACATCACCATATTATGAGTAGG
CTATTTGTTAAGTCATGGTTTGAAGTTTGGGAAGCTGCCACTTATGCTACGTCATCTATTCATTCTGTTTATGTAATTTT
GTAGCCATCCTACAATGGGTAATGTTAGATGGTAAGATTCTAG
>SoarTAS2R372DP_NW_004545902.1:15634859-15634330
ATGCCAAGTGTCACTGAAAGCATTTTTATCACCATCGAAGCTTTAGCATCCATGACAGCTATCTGGGGAAATGGATTCAT
TGTTCTAGTGATTGGTGCTGACTGGGTGAAGACCAAGAAAATCTCCTGGTATGACTTCATGTTCCTAAGCTTTGGCATCT
CCAGAATTGGCATGCTATTCATGATAATTAAAGATGGCATTAGATTAGTGTTCTACCCAGAAACAGATGACAATGACAGA

ACAGAAGCAACCATCATTGATGCCTTATGGAATCTGAGCATCTCCTTTAGTACTTGGTGTACAACCTGCCTTGGCATATT
TTATTTCTTCAAACTGTCCAGCTTTTCCCACCCTCCTTTTCTTTGGCTGAAGTGAAGAGACAGAATTTTTTTTGTCATTC
AATTGGAGTTCCTTCTCTCTCTGATTGGTAATGTTATGAGCATAATATACAATCAAACTTGGGCATGTGAGTATTTAAAA
ATGAAAAGAAATGTGACCTGGCAAGAATGGAGGTATCAAAAACAATTCTT
>SoarTAS2R372EP_NW_004545902.1:15633659-15633268
GGAAACATGTACTCCTCAACCTGGGATCCCTCATTGTATTAGCATTTGTCACTTATTTCCCCTTTTTTTGTTAACCTTTT
CCTTAGCAAGACATGTCTGGAGGATGATGTATCATGCCAATGGATCTGGAGACACAAACACAGATTCCTGTGACAGCCAG
AAATACAACAGTTTCCTTTCTCATTCTCCTCATTAGACACGACGTGACAACTTTCTTAGCAATTTGGGTTTATTCCACAG
TGGAAAACCTGATCGGTGTGCTTATTATTGAGACTATCACTGTACTTTATTCTTTAGTTCATCCCTTTACTATGATTCAA
GGGAACAGAAAACTGAAGCAATCTTTTATGAACTTGGCAAGGAAAGTTGAATTTTGCATCAAGGGAATATAG
>SoarTAS2R8HT_NW_004545902.1:15750915-15751410
ATGATCACTAAAGAAGATATCGTCTTTACTATTATAGTGGCTGGAGAATCTATGATAGGCATCTTGGCAAATGGGTACGT
TGGAATAATGTCCTGGATCGACTCTATTCAGAAGAATAAAACCCCCTCACTTAACTACATCCTCACAAGCCTGGCTATTT
CCAGAATATGTTTGCTCTTTATGATAATAATAGGTGACATTATTCAGAAACTCCATCCAGATTGTTATGAAGATGTCAAA
CTAGTAATAGTATACAGTGTTTTCTGGATACTTATCAACTACTCAAGTTTGTGGTTTGCCACCTGTTTCAACGTCTTCTA
TTTACTAAAGATAGCGAATTTCTCCAGTCCACTTTTCCTCTGGCTAAAATGGAGGATTGACAGGGCGATTTACTGGATCC
TGCCGGTGGGCTTGACCATTTGCTCCCTGAGCAGCCTTCTGACAGCAATGCCGAATTATTATGAACACATTTATAGATTT
ACAAAACATCAAAGAAGA
>SoarTAS2R8FT_NW_004545902.1:15759819-15760428
GAAGATAGCGAATTTCTCCAGTCCACTTTTCCTCTGGCTAAAATGGAGGATTGACAGGGCGGTTTACTGGATCTTGCTGG
CAGGCTTGGCCATTTGCTCCCTGAGCAACCTTCTGACAGCAATGCCGAATTATTATGAACACATTTATAGATTTAAAAAA
CATCAAAGAAACATCACTGAAATGTTCCATGTGAGTGAAATTAAGTATTTCAACCCATCAGTCCTCTCTAACCTTTTGGC
AGTTGTCCCAATTACTGTGTCATTAATTTCATTTTTCCTGTTAATTTTGTCTCTGTGGAGACACACCAAGCACATGAAAC
TCAATGTTACAGGCTGGAGAGACCCCAGCACAGAAGCCCATATTACAGCCATGAAAAATGTGACTTTTTTTCTCTTCCTC
CTTTCCATATACTATGCAGCTTGTCTTTTAGTAAATTACAGTGACATAATGAATGCAAAGTTAGTTTCTGGGCTTGGAAA
GATTGTATTAATTCTCTACCCCTCTGGCCACTCCCTTATTTTAATTATTGGAAATAGCAGGCTGAAACAAGCATTTGTCA
GGATATTGAGAAGTGTTGGGGGCTTGGGGGGCACAGCCTACCTAATGTAA
>SoarTAS2R408NT_NW_004545902.1:15582423-15582799
ATGATCACTTTACTATCAAGCATTCTTACCATCCTACTAATGGCAGAATTTGTTGTAGGAAGTTTTGCCAATGGCTTCAT
CGTGCTGGTGAACTACAATGACTGGGTCAAGACACAAAAGTTCTCCTTAGCAGATCAAATGATTGTGGCTTTGGCCATCT
CCAGGATTGTTTTTTTCTGTGTATTTTTAATAGTTTGGTATGCAGTGTTTTTTGATGTATCTTCATATAGTTTAGAAGTA
TTCATTATTATTAATATGGCTTGGGTTGTAAGCTACCATGTTAGCATCTGGCTTGCAACCAGCCTTAGCATATTCTATTT
GCTCAAGATCGCCAATTTTTCTAGCCTCCTATTTCTTTACTTGAAGAGGAGAGTTGA
>SoarTAS2R408OT_NW_004545902.1:15583293-15583451
TGGGGAAATAAGAAGCTGCAACAGTCTTTTCTTTCCTTTTTGTGTCAGCTGAGGTTGAGGCTGACAGAAAGGAAGAGGAC
ATTGTTTGCTTCACATAGAAAACAAACTGATGAGTCTCCTACCTTATCTAGTTCTACTGCTTTTTCAAAGCGTATATGA
>SoarTAS2R408PT_NW_004545902.1:15569628-15570004
ATGATCTCTTTATTACCAAGCATTCTTACCATCCTAGTAATGGCAGAATTTGTTCTAGGAAGTTTTGCCAATGGCTTCAT
CCTGCTGGTAAACTACAATGATTGGGTCAAGACACGAAAGTTCTCCTTAGCAGATCAAATGATCGTGGCTTTGGCAATCT
CCAGGATTGTTTTTTTCTGTGTATTTCTAATAGTTTGGTATATAGGGATTTTTGATGCATCTTTGAATAGTTTTGAAGTT
TTCATTATTATTAATATGGCTTGGGTTGTAAGCTACCATTTTAGCATCTGGCTTGCAACCAGCCTTAGCATATTCTATTT
GCTCAAGATCGCCAATTTTTCTAGCCTCCTATTTCTTTACTTGAAGAGGAGAGTTGA
>SoarTAS2R408QT_NW_004545902.1:15570178-15570639
TTCACTCTGACAATCTTCACAGCCTTTGTGATGTCCCTGACCTCATTTGTGTTGCTGATTTTTTCCCTGTGGAAACATCT

CAGGAACATGCAAGCAGAGGGCAAAGAATCCCAGGATCCCAGCACCGAGGTCCACATCAGAGCCATCAAAACGATGACTT
CCTACCTCCTGCTGTTTGCTTGTTACTTTGTGTCTCTACTCATCCCAATTTGGAGTTCTAACAGGATGCGGAACAGAACC
GTCCTCCAAGTCTTCCAGAGTTCTTTAGGACTGTATCCTTCAATCCACTCATTTATCTTAATCTGGGGAAATAAGAAGCT
GCAACAGTCTTTTCTTTCCTTTTTGTGTCAGGTGAGGCTGAGGCTGACAGAAAGGAAAAGGACATTGTCTACCTCAGGCA
GAAAACAAACTGATGAATCTGCTACTTTATCTAGTCCTGCTGCTTTTTCAATGTGTATATAA
>SoarTAS2R372BT_NW_004545902.1:15657136-15656846
ATGCCAAGTGTCATTGAAAGCATTTTTATCACCATGGAAGCTTTAGCATCCATGACAGCTATCTGGGGAAATGGATTCAT
TGTTCTAGTGATTGGTGCTGACTGGGTCAAAACCAAGAAAATCTCCTGGTATGACTTCATGTTCCTAAGCTTTGGCATCT
CCAGAATTGGCATGCTATTCATGATAATTAAAGATGGCATTAGATTAGTGTTCTACCCAGAAACAGATGACAATGACAGA
ACAGAAGCAACCATCAATGATGTCTTATGGAATCTGAGCATCTCCTTTAAT
>PhmaTAS2R1P_NW_006712848.1:538121-537228
CTGGAGTCTCACCTCATTAGCCACCTTTGTTTGGCAGCGATACAGTTGCTCGTTGGGGTTTTAGTAAATGGCATCATTGT
GGTTGTGAATGGCACTCACTTGATCAAGCAGAGAAAGATGATTCCGTTGGATCTCCTTGTTTCCTGCCTGGCGATTTCCA
GGATTTGTCTGCAGCTAGCCATCTTCTACGTTAACCTGGCTATTCTTTCCTTGATTGAATTCCCTCAGCTTGCTGAGAAG
TTCGTAATTCTCACATTTATAAATGAATCGGGACTTTGATTTGCCACATGGCTCGGCCGTTTCTACTCTGCCAAGATTGC
CACCATTGCTCACCCACTCTTCTGGTTGAAGATGAGGATATCCAAGTTGGTTCCTTGGCTGATACTTGAGTCCCTGCTAT
ATGCATCTAGCATGGCTCTCTTCCACAGCAAACATAGATGGATATCTTCCAAAGAACACTTCCTGGGCCTTTTCTCCCCA
AATGCAACCACTCAAATCAAAGAAATACCTGCTTTACAGTTTGCCCTTCTTTTTGCTGAGTTCTCATTGCCATTACTTAT
CTTCCTTATTTCTTCTCTGATCTTGATATTTTCCCTGGGGAGACACACCTGACAGATGAGAAGCACAGCAACAGGCCCCA
GGAACCCTCACACATGTGTGCACATCAGCACTCTTCTCTCCATCCTGTCCTTTCTGGTCCTCTATCTCTGCCACTCCGTG
ACAGCTGCTTTGCTCTTTTCTCAAATTTTCAACTTCAGAAGCTTCATATTTCTGCTCTGCATCTTGGGGGTTGGTTCATA
CCACTCTGGACACTCTATTGTGTTAATTTTAGGAAATCCTAAAATGAAACAAAATGCAAAGAAATTGCTCCTCCGCAGAA
AGTGCTGTCAGTGA
>PhmaTAS2R2P_NW_006714444.1:120957-120049
ATGGCCTCCTCTTTGTCAGCTCGTCTTCATGTTATCCTCATGTCAGCAGAATTTATCACAGGGATTACAGTAAATGGATT
TCTTACAATCATCAACTGTAATGAATTGGTCAAAAGCAGAAAGCTAACACCAGTGCAACTCCTTTTCATATGCATAGGGA
TATCTAGATTTGGTTTGCAGATGGTGTTAATGGTAAAGTTTTTTCTCATGTTCTTTCCACTCTTTTATAGAGTAAAAATT
TATGGTACAGCGATGATTTTTTTGGATGTTTTTCAGCTCTGTCAGTCTCTGGTTTGCCACCTGTCTTTCTGTATTTTACT
GCCTCAAGATAACACACTTCACCCAGTACTGTTTTCTTTGGCTGAAATTCAGGATCTCAAAGTTAATGCCTTGACTGCTT
CTGGGAAGCCTGCTGACCTCCGTGAGCATTGCAGCTCTGTGTGTCAAGGTGGATTACCCTAAAAATGTGGATATTGATGT
CCTCAGGGATGCCATGCTAAAGAGCACTAAACTCAAGACAAAGCAAATTAATGAAGTGCTTCTTGTCAACTTGGCATTAA
TATTTCCTCTGGCCATATCTGTGATGTGAACTGTTATGTTACTCATTTCTCTCTATAAGCACGCTCATCGGATGCAAAAT
GGACCTCTTGGTTTTAGAAACGCCAGCACTGAAGCCCATATTAATACATTAAAATCAGTGATAACATTCTTTTGCTTCTT
TATTTCTTATTCTGCTGCCTTCATGGCAAATATGACATTCAGTATTCCTTATGGGAGTCAGTGCTTCTTTGTGGTGAAGG
ACATAATGGTAGCATATATCCCTCTGGCCATTCGGTTATAATTATCTTGAGTAATTCTAAGTTCCAACAACCAATCAGGA
GACTTCTCTGCCTCAGAAAGAATCAGTGA
>PhmaTAS2R3P_NW_006713057.1:233037-233988
ATGCTGGGACTCACTGAGTGCGGGTTTCTGGTTCTGACTGCCACTCAGTTCATTCTGGGAATGCCGGGGAATAGTTTCAT
GGGTTGGTCAGTGGTAGCAGCTGGTTCAAGAACAAGAGAACCTCTTTGTCTGACTTCATCATCACTAACCTGGGTCTCTC
CAGGATTGTTCTGCTGTGGATTCTGTTGATTGATGGTGTTCTAATGGTGTTCTCTTCCAAACTCCACGATGAATAATTTG
CAATCATGCAAATTAGTGATATTTTCTGGACATTTACAAACCATCTGAGCATTTGGCTTGCCACCTGTCTCAGTGTCGTC
TACTGCTTGAATGTCGCCAGTTTCTCCTATCCTACGTTCCTCTGGCTCAAGTGGAGAGTTTCCAGGTTGGTTGTATGGAT
GCTCTGGAGTACCCTGCTCTTATCATGTAGCCGTGCCATCTCTCTGATCCATGAATTTAAGATGTATTCTGTTCTCAGTG
GAATTGATGGAACAGGGAATGTGACTGAACCGTTTAGAAAGAAAAGAAATGAATATAAACTGATCCATGTTCTTGGGACT

CTGTGGGACCTCCCTCCCTTAATTTATCTCTAGCTTCCTACTTTCTGCTCATCCTCTCCCCTGGGGAGGCATATGCAGC
AGATGAAGCAAAACTTTACCAGCTCCAGAGATCCAAGTACTGAGGCCCACAAGAGGGCCATTAAATCATCCTTTCCTTTC
TCTTTCTCTTCCTACTTTACTTACTTTTCTTTTCAATTTTGACATCCAGTTATTTCTACCAGCAACTGAGGTGATTATGA
TGACTGGAGAAGTAATTACAATGTTATATCCTGCTGGCCGCTCATATATTCTCATTCTGGGAAATAATAAGTTGAAGCAG
ATGTTCATGGAGATGCTTTAGTGTGAGCCTGGTCATCTGAAGCCTGGATCCAAGGAACCCGTTTTTCCATAG
>PhmaTAS2R4P_NW_006713057.1:242431-243328
ACACTTCAGACATTCTTTTCTTTTTCTGTTATTGTCTCAGTGATTTTGACTTTTGTAGGACTCATTGTGAATCTCTTTAT
TGTAGTAGTCAGTTACAATACTTGGGTCAAAAGCCACAGAATCTCCTCTTCTGACAGACTCCTGTTCAGCTGGGGCGTCA
CCCGATTTCTTATACTGGGACTGAATGCTGTTTTCTTCATCTCTACAAATATGGAAAGGTCAGTCTACATATCTATTTTT
TTTTTTCCTGTCGTGATTTCTAGGATGTTTTTGGACTCTAATAGTTTTTGGTTTGTAACCTTGCTCCATGCCTTGTACTG
TGTGAAGATTGCTAACTACCAACACTCACTGTTTCTCCTGCTGAAACAAAATCTCTCCCCCAAGATGCCCTGGCTGCTGC
TGGTCTGTATGCTGATTTCTGTCTTCACCACTCTCCTGTATGTTGTGCTCAGACAGAAAGCACTCCCTTCTGAATTTGTG
GCTGGGAGAAATGGCACAGTATTTGACATCAATGAGGGAGTCTTGTCTTTGGTGACCCCTTTGGTCTTGAGCTCATTTCT
CCAATTCATCATTAGTGTGACTTCTGCTTCTTTGTTAATCAGTTCCTTGAGGAGACGTATACAGGCGATGCAGAGGAATG
CCACTGTTCTTTGCAATCCCCAGACGGAGGCTCATGTGGTGCTCTGAAGCTGATGATATATTTCCTCATACTCTACATTC
TGTATTCAATTGCTGCCCTGCTCCATTATCTCCCTTCTTCTGTAGGGATGGATTTGGGAGCCAAGTCCATTTATACTGTC
ATTTCCTCCATTTACCCTCCGGGACATTCTGTTCTCATTATTCTCACACATCCTAAACTGAAAACAAAGCAAAGAAGATT
CTTTGTTTCAATAAATAG
>PhmaTAS2R5P_NW_006713057.1:253058-253968
ATGCTGACTGCTGTCCTAGGACTGTTGATGCTGGTGGCAGTGGCTGAATTTCTCATTGGCCTGGTTGGAAATGGAGTCCT
TGTGGTCCGGAGTTTTGGAGAATGGCTCAGAAAATTCAAGGGGTCCTCATATAACCTCATTGTCCTGGGCCTGGCTGTCT
GTTGATTTCTTCTGCAGTGGTTGATTATGGTGGACTTAAGTCTGTTTCCACTTTTCCAGAGCAGCCATTGGCTTCGCTAT
CTCAATGTCTTCTGGGTCTTAGTAAGCCAGACCAGCCTGTGGTTTGCCACTTTTCTCAGTGTCTTCTACTGCAGGAAGAT
CATGACCTTTGAGCACCCTGTCTACTTGTGGCTGAAGCAGAGGGCCTGTTGCCTGAGTCACTGGTGCCTTCTGGTGTACT
TCATGATCAGTTTGTTACTTACAGTCCAGGGTAGCGTAGAGTTCTCCGATCCTTCCCGAGGAGACAGCAGCATTTTATAC
CCCCCTTCAAACTGGCACTAGCTGTATATATTATGGCTCAATACAGGAAGTATAACGCCTTTCATGGTGTTGCTTATTTC
CTCTGGGATGCTGATTGTCTCTCTGTGTAGGCACCACAGGAAGATGAATGTCCACACAGCCGGCAGGAGAGATGCTCAGG
CCAAGGCTCACATCACTGTCCTGAAGTCCTTGGGCTGTTTCCTTATACTTTACATAGTTTACATCCTGGCCAGCCCCTTC
TCCATCACCTCCAAGTCTTTTCCCGCTGATCTCACCACTCTCTTCATCTCTGAGACACTCATGGCTGCCTCTTCATCTCT
GAGACACTCATGGCTGCCTACCCTTCTCTTCATTCTGTCATATTGATCATAGGGAATCCCAGGATGAAGCAGACTTGTCA
GTGAATCCTGTGGAAGACAGTATATACTTGG
>PhmaTAS2R16P_NW_006724430.1:567500-566607
ATGATAACCATCCAACTCTCTGTCTTCTTCATGATCATCTATATGCTCAAGTTCTTGACAATAATTGTGCAGAGCAGCTT
AACTGCTGTAGTGCTGGGCACAGAGTGGGTGAGGTTCCAAAGGCTGTCACCCGTGCAAATGGCCCTCACTGGCCTGGGCG
TCTGCTGCTTCTGCCAACTGTGCTCATCGACGCTGTACAACTTTGCTCCCACTTCCTCCCTAATTACGAATTTTGCTACT
TCAGGATCATCTGGGAATTTACCAACTTCTTTCATTCTGGTTGACCAGCATGCTTGCTGTCCTCTACCGTGTCAAAGTCT
CCTCCTTCAGCCACCCCATCTTCTGGCTGAAGTGGAGAATTGTGAGGTTGGTTCCTCGGCTGTTGCTGGGCTCTCTGCTG
ATTTCTTGTGTGTCTATCATCTTTGCAGCTGTTGGGCATTACAGCAAGATTCAACTAATCTCCATGAGGCATTTCCCTAG
AAACAGCACCATGACTGAGAGACGAGATATTCCTGTGGGATTTTTCCATGTGCCAGCCAGTGGTTGTGTTGATTATTCCT
TTCCTCCTGTTCCTGCTTCCACCGTCTTGCTCATGGCCTTATTATTCCAACACCTGAGGCAGATGAAAGATCATCACACC
AGCCACTCTCCAGCCTGGAAGCTCACTCTACTGCCCTGAGGTCTCTTGCCATCTTTCTCATTTTCTTCACCTCTTATTTT
CTGACTCTACTAACCTCCATCTGGGGTGTCCTTTTTAATAAGGGGTCCTGGTTCTGGGCCTGGGAAGCTATCATCTATGC
TCTAGTCTCTATTCATTTGACTTCACTGATGCTGAGCAGCCCTAAATTGAAAAGGGTTTTAAAGGTAAGGTGCTGGGGCC
TAGAGGCTGCCTGA

>PhmaTAS2R38P_NW_006713057.1:305001-304003
ATGGTGACTCTGACTGCCGTCGTAACTGTGCCCTGCGAAGTCAGGAATGCATTTCTGTTCTTCTCAGTCCTGGAGTTTGC
AGTAGGGATCCTGGTCAGTGCCTTCATTTTCGTGATGAATTTTTGGGTCGTGGTGAGGAGGTGGCCACTGAGCAACTGTG
ATCTTGTCCTGCTGAATCTCAGCCTCACCTGGCTTCTCCTGCACGGGCTGCTCTTTCTGGATGCCATCCAGCTTACCCAC
TTCCAGTGGATAAAAGACCCACTGAGCCTCTGCTACCAGACCATCCTCATGCTCTGGAGGCTCGTAAATCAAGCTGGCCT
CTGGCTCCCCACTCGCCTTAGTCTCCTCTACTGCTCCAAGACTGTCCATTTCTTTCACACCTTCCTCCTCCGCTTGGCAA
GCTGGATCTCCAGGAAGATCCCCCAGATGCTCCTGGGTGCTATTTTTTCCTCCTGTGTCTGCATTGTTCTCTATCTGTGG
GACTGTTTCAGTAGATCTCACTTCTCAGTTGCAACCATGCTACTCATGAATAACAATACAGAACTCAATTGAGAAAACTC
AATTTCTTTCATTCCTTCCTCTTCTGCAGCCTGGGGTCCATCCCTTCTTTCTTGCTTTTTCTGGTTTCTTCTGGGGTGCT
GATTGTCTCCCTGGAGAGGCACATGAGGACAAGGAGGGCCAAAACCAGAGACTCTCGGGACCCCAGCCTGGAGGCCCACA
TCAAAGCACTCAGGTCTCGTCTCTTTCTTCTGCCTGTATGTGGTGTCCTTCTGCGCTGCGTTCATCTCGGTGCCTTTGCT
GATGCTGTGGCACAACAAGATCGGGGTCATGGTCTGTGCAGGGATACTGGCAGCCTGCCCCTCGGGGCACACAGTCTTGA
TGATCTCAGGCAATGCCAAACTGAAGAGAGCTGTGGAGACCATTCTTCTCTGGGCTCAGAGCAGCCTAAAGGTAAGGGCG
GACTGCAAGGCAGATCCCAGGATGCCAGATCTATGTTGA
>PhmaTAS2R39P_NW_006714005.1:289465-288408
AACAGTGGGAGCTATCACAGATCTGCACATCACACGCTAGGGAGACATTTTCCTCCAGACACCAAAGAGAAGCAACAACT
CAGGATGACTGAAACCTGCAATCCCCCAGAAAATCAACTGTCACCATCTCGCATCATTTTGATGTGAATAATTATAGGCA
CTGAATGCGTCCTTGGTATCACTGCAAATGGGATCATTGTGGCTATAAATACAGCAGAATGGATTCACAACAAGGCAGTT
TCCACAGGTGGCAAGATCCTGCTCTTCCTGAGTGTATCCAGAAGAGCGCTACAAAGCTTCATGATGCTAGAACTCACCTT
CAGTTCAACATGCCCACATTTTTATAATCAAGACATTCATTGTATATGATACATTCAAAGGAAGTTTCATGTTCATAAAT
TATTGTAGCCTCTGGTTTGCTGCCTGGCTGAGATTCTTCTACTTCGTGAAGACTGCGGATTTCTCCTACCCCCTTTTCCT
CAAGCTGAAGTAGAGAATTTCTGGTTTGATGCCCTGGCTTCTGTGACTATCAGTGTTTGTTTCCTTGGGCCACAGTGTGT
TCTTCCTCAAAAACGTCTACACTGTGCATTGCAACCATCCTTTTTCTAGCCCCTCCTTCAACTCCACTAAGAAAAATTAC
TTCACTCAGACCAGTGTGATCAGCCTGGTTCTTTTCTTTAACACGGGAATCTTCGTTCCTCTGATCACTTTCATCCTGGC
TGCCACCCTGCTGATCACCTCTCTCAAGAGACACACCCTACACATGAAAAGCAATGCCACTGGCTCCAGGGACCCCAGCA
TGGAGGCTCATGTGGGGACCATCAAAGCTGTCAGCTATTTTCTCATTTTCTAAATTTTCAATGCAGATGCTCTATTTCTT
TCCATGTCCAACATCTTTGATATCAACAGTTCCTAGAATATTTTGTGCAAAATCATCATGGCTGCCTACCCTGCTGGCCA
CTCCATTCTACTGATACAGGACAAACCCTGGGTTGAGAAGAGCCCGGAAGCGGCTTCAGGCTCACGTTCACCTTTACTTA
AAAGAGTAGACTCTATGA
>PhmaTAS2R41P_NW_006714005.1:45018-44125
ATGCTGCTCTGTGTCCTGGGACTCCTGGCCAATGGCTTCGTTGTGCTGGTGCTGAGCAGAGAATGGGTGTCACCAGGGAG
GCTGCTCCCCTCCAGCAGGAGCCTCTTTAGCTTGGGTGTCTCCTGCTTCTGCCTGCAGTGGGTTGCAGTGGCAACTTCTA
CTACTTCCTCCATCTGGTCGAGTACTGCGGGGGTCCCGCCTGGCAGTTCTTTGGTCTACGCTGAGACTTCCTGAACTCGT
TCATCTTCTGGTTTGGCTCCTGGCTCAGCAACCTCTTCTTCATGGAGATTGCTAACTTCACCCACCCCACCTTCCTCTGG
CAGAAGTAGAGGTTCCCAGGGTTAGTGCTCTGGCTTCTGCTGGGCTCTCTCCTCATCTCCCTTATCATCACCCTGTAGTT
CTTTTGGGGGAACCACGATTTGTATAAAGGTTTCTTTATTAGCAAATTTTCTGGTAACATGACCTACAATCAGCAGAGCA
GGGGGGCTGGAAATTCACCATTTTCTACCCCTGAAACTGGTCACCCTTTCAATTCCTTGCTCTCTTTTTCTGGTCTCGAT
TGCTCTGTTGATTAACTCTCTGAGGAGACACATGTGGAGGAGGCGGCACAATGCCCACAGCCTGCAGGAGCCCAGCGCCC
AGGCTCACACCAGAGCTCTGAAGTCACTCATCTCCTGCCTCGTTCTTTATGCTCTGTCCTTCGTGTCCCTGAGCATCAAT
GCTGCAGCGTTCTACTCCTCAGAAAGTGACTGGTACTGGCCGTGGCAAATTTTAACCTACTCGTGCACATCCATCCATCC
CTTTATCCTCATCCTCAGCAACCTCAGGCTTTGAGGGGTGTTCAGGCAGCAAATTTTGTTGGCCAGGGGCTTCTGGGTGG
CCCAAGTGGCATGA
>PhmaTAS2R60P_NW_006714005.1:75413-74480
ATGGTTCCAGGACCTCAGCTGGCTGATAAGATAGCCTTTATCTTTGCTACCATTTTATTCCTTTTGTGCTTGGTGGCAGT

GGTGGGTAATGGCTTAATCACCATGGCACTGGGCATGGAGTGGTTGCTGCAGAGAACTTTGTCACCCTGCAATAAGTTAT
TGGTCAGCCTGGGAGCCTCTAGCTTCTGTCTGTGATGGGGGTGATGAGTAAGAACATTTATATTTTCCTGAATCCAATAG
CCTTCCTATACAACCCTGTATTCCAGTTCCTAGCCTTTCAGTGGGACTTCTTGAATGCTGTCACGTTATGGTTCTCCACC
TGGCTCAGTTTCTTCTACTGTGTGAAAATCGCAACCTTCACCCACCCTGTCTTCCTCTGGCTGAAGCAGATGGTGTTTGC
ATTGGTTCCATGGATGCTGCTCAGCTCTGTGGGGTTCTCCAGCTTTAGCCCCATTCTGTTTTTCATAGGCAACCAGAGAA
TATGTCAGAACTATTTAAAGAGAGCTCTGCAATCTTGGAATGTCACTGGGGATGCTGTGAGAACATATGAGAGACTCGAC
TTCTTCCCTTTGAAATTGTTACCTGGACAGTCCCTGCTGTCGTCTTCATCGCTGGCGTGGCTTTGCTCATTACATCTCTG
GGAAGACACACCAAGAAGGTCTCCCTGTCCATCTCAGGCTCTCATGATGCCAGCACGCAGGCACACATCAAGACTCTCCT
GGCTCTCATCTCCTTTGCTGTCCTCTTCGTTTCCTGTTTTCTGTCACTGGTGCTCAGTGCCTCAGGTGTGTTTCCATCAC
GGGAACTCAGGTACTGGGTGTGGCAGGCTGGATTTATCTGTGCACAGTGGTCCCCCCATTGTTCTTCTCTTGAGTAACCG
CGGGCTGAGAGCTGCGCTGGAGAGGGGCCGCTCCTCAGGGCATGGGCATCTTGA
>PhmaTAS2R62P_NW_006724183.1:817687-818612
GTGCCCCGCTCACCCACGTTGATCTGCATGGTCATCTTTTTCCTGGAGTCGGTGGCTGCCATACGCCGCAGAACGGAGTC
ACAGTTACCGTGCTGGGCCGGAGCCGGACGCTGGATGATGCTGGACGCTGCCCGCAGGCGACATGATCGTGGCCTGCCCG
GCCGCCCCCCGGTTCTGTCTGCAAGGGATGGCCCTCCAGAACAACCTCCTGACTTCCTTTGCTTTGGGTTCCAAATTTTA
TTTCAAACTCTCCTGGAGCTTCACCAACACTCTCACTTTCTGGCTGACCACCTGGCTTGCTGTCTTCTCCTGTGTGGAGG
ACAGCATCCTTCTCTCCCCGCATCTTCTTCCGGCTGAAGTGGAGGATTTCTCGGTCAGGCCCCAGCTGCTGCTGGGCCCC
CTGATCCTGTCTGGTCTGACATCATCATCAGCCTCCGAGAAGTCCATTCTTGTGCAGATGGTTGCCACCCAGGGTTCCCA
TGGAAACGACACCCCGGCTGGTAGAATACAGACTGTCTCTCTGCGCTTTTTTCTACCTCATGCAATTATCATGTGGTCAG
TTCCACTCCTCCTGTTCCTGGGGTCCACCCTCTCACCCGTGTTCTCGCTGCGCCGGCACTTGGGGCAGAGGGGGGCCCAC
AGACCCGGCCCGAGTGGCCCAGCACCCGGGCTCACACCATGGCCCTGAAGTCACTTGGCCTTCTTCCTCGTCGTATTTCC
TGTGCCTGATTATTGTTGCTGTGAACGTCCCAACCCTCCGGAAGCACCGGCGCTGGGCCTGGGAAGCGGTGACCTGTGCC
GGCATCTGTCTGCCCTCCAGCACCTTGGTGCACAGCAGCCCCAAGCCGAGAGAGGCCCTGGAGCAGAGGCTTTGGCGAGC
CCTGGAGCAGAGGGAGCAGTTTGTCTTGAGTTATCAGTATCAATAA
>PhmaTAS2R67P_NW_006714620.1:702224-703146
ATGCCATCTGGAGTTGAAAATACTTTTCTGGCAGAGGTAATAGGAGAGTTCATGACTGGAATGCTGGGGAATGGGTTCAT
TGTACTAGTTAACTGCATTGACTGGGTGAAGAGACCAAAGTTCTCATCAGCTGACTGCATCCTCACCGGCCTGGCAAGCT
CCAGAATCAGTCAACTTTGGAAAATGCTATTTGACTCGTTTGTACTGGTGTTATGGGCACATCTATATGCCATTGATAAA
CTAGCAAAAAATTTTTGGACCCTGTCCAATCACCTAGCTACCTGGTTTGCTACCTAAGTGTTTTCTGCCTCTTTAAAGTA
GCCAGTTCCTCCCACCCCTGCTTCACCTGGCTGCGGTGGCAAATTCATAGAGTGGTACTTGTGCTTCTGATGGGGTCTTT
GTTCTTACTGTTTTTGAACTTTGAATTAGTAGATGCGTTTAATGGTGTCTGGACTAATGTCTACAAAATATATGAAAGAA
ACTCAACGTGGCCCTCAGATGTAAGTAAAACTCTTTATCTTGACATGTTGATTTTCACCTTCATCTGTTTAATCCCCTTT
CTTCTGTCCCTGACCTCATTGCTCTGTTTATTCCTCTCCTTGATGAGACACACCAGGAATTTGCAGCTCAGCCCCAGCTC
AAAGGACTTCAGCATAGAGGCCCATAAAAGAGCCATGAAAATGGTGATGTCTTTCCTCCTCCTCTTCATGGTTCACGTTT
CTTCTATCCTATTAACAGGTTGGGTTTTCCTTAAACTGCAGAAACATCAAGCCAATTTGGTTGTCATGTTAACTTTGACT
CTTTTTCCTTCAGGCCACTCATTTATCTTAATTTTGGCAAACAGCAAGCTGAGACAAAATGCCTTAGGACTACTGTGGTA
TCTTAACTGCCACCTGAAAAAAGTGAAACCTTTAGCTTCATAG
>PatiTAS2R1_NW_006712067.1:2562830-2563726
ATGCTAGACTTTTACCTCATTATCCATTTTCTTCTTCCAGTGATACAATGTCTCATCGGAGTTTTAGCAAATGGCATCAT
TGTGATTGTGAATGGCATTGAGTTGATCAAGCAGAGAAAGATGATTCCGTTGGATCTCCTTCTTTCCTGCCTGGCGATTT
CCAGGATTTGTCTGCAGTCATTTATCTTCTACATTAATCTGGTTACTCTCTCCTTGATCGACTTCCTTCCACTTGTTAAG
AATTTTGCGGTATTCATGTTTGTAAATGAAATGGGACTTTGGCTGGCCACATGGCTCGGCGTTTTCTACTGCGCCAAGAT
CTCCCCCATCGCTCACCCACTCTTCTTCTGGTTGAAGATGAGGATATCCAAGTTGGTGCCATGGCTGATCATCGGGTCTC
TGCTTTTTGCCTCCGTCCCTTTGGTTTTCTACAGCAAGCATACGTGGGTTCTTTCCCAAGAAGGCTTGTTGAGACTTTTC

TCCCCAAATGCAACAACTCAAATCAAAGAAACATCTGATTTACAGATTGTCTTTCTTGCTAGGTTTTCACCGCCGTTCGT
TATCTTCCTCATTTCTACTCTGCTCCTGGTGTTTTCTCTGGGGAGACATACCTGGCAGATGAGAAACACAGCGACGGGCA
CCAGGGACGGTAGCACAGGTGTCCATGTGAGTGCGCTTCTGTCCATTCTGTCCTTCTTGGTCCTCTATCTCTCCCACTAC
ATGACGGCTGCTTTGCTGTCTTCTCACATTTTTGAGCTCAGAAGCTTCATGTTTCTGTTCTGTATCTTGGTGTTTGGGTC
CTACCCTTCGGGACACTCTATTATCTTAATTTCGGGAAATCCTAAACTGAAACAAAACGCGAAGAAGTTCCTCCTCCATG
GGCAGTGCTGCCAGTGA
>PatiTAS2R2_NW_006711983.1:13180508-13179604
ATGGCCTCCTCTTTGTCAGCGATTCCTCACCTTATCATCATGTCAGCAGAATTTATCACAGGGATTACAGTAAATGGATT
TCTTGTAATCATCAACGGTAAAGAATTGATCAAAAGCAGAAAGCTAACACCAATGCAACTCCTTTGCATATGTATAGGGA
TATCGAGATTTGGTTTGTTGATGGTGTTAATGGTACAAAGTTTTTCTCTGTGTTCTTTCCACTCTTTTATAGGGTAAAA
ATTTATGGTGCAGCAATGTTGTTCTTTTGGATGTTTTTAGCTCTGTCAGTCTTTGGTTTGCCACCTGCCTTTCTGTGTT
TTACTGCCTCAAGATATCAGGCTTCACTCAATCCTATTTTCTTTGGCTGAAATTCAGGATCTCAAAGTTAATGCCTTGGC
TGCTTCTGGGAAGCCTGCTGGCCTCCATGAGCATTGCCGCTGTGTGTTTGGATGTAGGTTACCCTAAAAACACGAACAAT
AATGATTTCCTCAAGAATGCCACGCTGAAGAAGACTGAACTCAAGATAAGACCAATTAATGGAGTGCTTCTTGTCAACTT
GGCATTACTATTTCCACTAGCCATATTTGTGATGTGTACTTTTATGTTATTCATTTCTCTCTATAGGCACACTCATCGGA
TGCAAAACAGATCTCATGGTGTTAGAAATGCCAGCACAGAAGCCCATATAAATGCATTAAAAACAGTGATAACATTCTTT
TGCTTCTTTATTTCTTATTTTGCTGCCTTCATGGCCAATATGACATTCAGTATTCCTTACGGAAGTCAGTGCTTCTTTGT
GGTAAAGGACATAATGGCAGCATTTCCCTCTGGACATTCAGTTATAATCATATTGAATAATTCTAAATTCCAACAACCTT
TCAGGAGACTTCTCTGCCTCAAAAAGAATCAATGA
>PatiTAS2R3_NW_006712036.1:454135-455085
ATGTCAGGGCTCCACAAGTGGGTGTTTCTGGTTCTGTCTGCCACTCAGTTCATTCTGGGGATGCTGGGGAATGGTTTCAT
AGTGTTGGTCAATGGCAGCAGTTGGTTTAAGAATAAGACAATCTCTTTGTCTGACTTCATCATCGCTAACCTGGCTCTCT
CCAGGATCGTTCTGCTGTGGATTCTTTTGGTTGATGGTGTTTTAATTGTGTTCTCTTCCAAAGTGCATGATGAAGGGATA
ATAATGCAAATTATTGATATTTTCTGGACATTTACAAACCACCTGAGCATTTGGCTTGCCACCTGTCTCAGTGTCCTCTA
CTGCCTGAAAATTGCCAGTTTCTCTCACCCTACATTCCTCTGGCTCAAGTGGAGAGTTTCCAGGATGGTCGTACAGATGA
TCTTGGGTGCGCTGGTCTTATCGTGCGCCAGTGCCCTGTCCCTGATCCATGAATTTAAGATGTATTCTATTCTCGGTGGG
ATCGATGGCACAGGGAATGTGACTGAGCACTTTAGAAAGAAAAGAAATGAATATAAATTGATCCATGTTCTTGGGACTCT
GTGGAACCTGCCTCCTCTGATTGTGTCTCTGGCCTCCTACTTTCTGCTCATCCTCTCTCTGGGGAGGCACACACAGCGGA
TGGAGCAAAGCGGCACCAGCTCCAGAGATCCAAGCACTGAGGCCCACAAGAGGGCCATCAAAATCATCCTCTCCTTCCTC
CTTCTCTTCCTGCTTTACTTTCTTGCCTTTTTAATTACATCATCCAGTTATTTCATACCAGGAACTGAGATGGTGAAGAT
AATTGGAGAACTCATTACCATGTTTTATCCTGCTAGCCACTCATTCATTCTCATTCTGGGAAACAGCAAGCTGAAGCATA
TGTTTGTGGGGATGCTGCGGTGTGAGTCTGGTCATCTGAAGCCTGGATCCAAAGGACCTGTTTCCCTGTAG
>PatiTAS2R7_NW_006711765.1:2500952-2501887
ATGCTGGATAAAGTGGAGAGCGCCTTGATGCTCATAGCCGCTGGAGAATTTGCAATGGGGATTTTAGGGAATGCATTCAT
TGGATTGGTAAACTGCATGAACTGGATCAAGAATAGGAAGATTGCCTCCATTGATTTAATCCTCACAAGTCTGGCCATAT
CCAGAATTTGTCTATTATGTATCATACTATTAGACTATTTTATACTGGGGCTGTATCCAGATGTCTATACTACCGGTAAA
GAAATGAGAATCATTGACTTCTTCTGGACGCTCACCAACCATTTAAATGTCTGGTTTGCCACCTGCCTCAGCATTTTCTA
TTTCCTCAAGATCGCGAATTTCTTCCATCCCCTTTTCCTCTGGATGAAGTGGAAAATTGACAGTGCGATTCCTAGGATCC
TGCAGGGATGCTTGGCCTTCTCTGTGTTTATTAGCCTTGTTGTCTCTGAGAATCTGAACGATGATTTCAGGTCTTGTGTT
AAGGTAAAGAAGAAAACAAACATAACTGTGAAATGCAGAGTAAATAAAGCCCAATACGCTTCCGTCAAGATTTGCCTCAA
CCTGTTGACGCTATTCCCCTTTTCCGTGTCCGTGATCTCATTTCTCCTCTTGCTCCTCTCCCTGTGGAGACATACCAGGC
AGATGAAGGTCAGTGCCACGGGGTGCAGGGACACCAGCATAGAAGCCCATGTGGGAGCCATGAAAGCTGTCATCTCCTTC
CTCCTCCTTTTCATTGCTTACTATTTGGCTTTTCTTGTAGCCACCTCCAGCTACTTTATGCCAGAGACTGAATTAGCTGT
GATGATTGGTGAGTTGATAGCTCTCATCTATCCAAGCCATTCATTGATTCTAATTCTGGGGAACAATAAATTACGGCAGG

CGTCTCTAAGGGTGCTGTGGAAAGTAAAGTGTATCCTAAAAAGAAGAAATCACTAA
>PatiTAS2R9_NW_006711765.1:2493021-2494034
ATGCCAAGTGCAGTGGAGGTAATGTATATGGTCTTGATTGCTGGTGAATTGACTATAGGAATCTGGGGAAATGGATTTAT
TGTACTGGTTAACTGCACTGGTTGGCTCCAAAGGCGAGATAGCTCCGTGATTGACATCATCCTGGTGAGTTTGGCCATCT
CCAGAATCTGTGTGTTGTGTGTGGTATCTGCAGATGGCTTTGTTCTGCTGCTCTCTCCACATGCGTATGCTCAAAATGAG
ACAGTAAACACCTTGGATGCTTTCTGGACACTGAGCAACCATTCAAGTGTCTGGTTCACTGCTTGCCTCAGCATTTTCTA
CTTACTGAAGATAGCCAACATATCCCACCCGGTGTTCCTCTGGCTGAAGCTAAACGTTACCAGAGTCGTCCTGGGGCTTT
TTCTGGCGTCCTTCCTCACCTCCATAATTATTAGTGTCTTTTTGAAAGAGGGATCCTGGGGTCACGTCGAAGTCAATCAT
GAGGAAAACATAACTTGGGAATTCAGAGTGAGTAAAGCCCCAAGCGCTTTCAAACTGATTATCCTGAACCTGGGGGCTCT
AGTTCCCTTTGCTCTGTGCCTAATCTCCTTTGTCTTGTTACTTTTCTCCCTCTTTAGACACACTAAGCAGATGAAACTTT
ACGCCACCGGGTCCAGGGACTCTAGCACAGAGGCACACATGAGGGCCATAAAGGCAGTGACCATCTTTCTGCTTCTCTTC
ATCATGTACTATGCAGTCTTTCTTGTAGTCACTTCTAGCTTCCTGATTCCTCAAGGGCGGTTAGTGTTGATGTTTGGTGG
CATAGTCACTGTCATTTTCCCATCAAGCCATTCGTTCATCCTGATCATGGGCAACAACAAACTGAGGGAGGCCTTTCTGA
AGGTGCTAAGGTGTGTGAAGGGCTTCCACAAAAGAAGGAAACCTCTTGTTTCACAGAGAATCCTGAATATGGGGAGAAAG
AAATCAACAAAAGACTGTCTCCCTTCTCCCCGGGGATTACATTCATTTGCTTAA
>PatiTAS2R12_NW_006711765.1:2453225-2454154
ATGGCAAGCATATTGAAGAATGTATTTATGATACTGTTTGCTGGAGAATTCATAATGGGGATTCTGGGAAATGGATTCAT
TATATTGGTTAACCGTATTGACTGGATCAGGAACTGGAAATTCTTCGTAATTGACTTTATTAGTACCTGCCTAGCTATTT
CCAGAATAGTTCTGTTGTGCATAATAATTTTAGGCATAGGTGTAGATGTACCTTGTGAAGAAATATGGAACAAGAAAAAT
CAACTAATAAGTTTTGAAATCCTCTGGACAGGATCCAATTATTTCTGCACAACCTGTACCACCTGCCTCAGTGTCTTCTA
TTTCTTCAAGATAGCCAACTTTTCCAACCCTATTTTCCTCTGGCTAAAATGGAGAATTCACAAAGTGCTTCTCATTATTG
TACTGGCTGCAGTCTTCTCTTTCTGCTTGTCTCTTCCCTTTAAGGATACAGTGTTTATGAGTCTGATCAAAAGCAAGGTA
AACGCGGAAAGAAATCGGACAGTGAGTTTCACAATGAGAACATATGAGTTATTTTTGTCTCATATGCTCCTGAACATAAT
GTTCATCATCCCCTTTGCAGTGTCTCTGGCTTCCTTTGTCCTTTTGATCCGTTCCTTATGGAGCCACACCAGGCAGGTGA
AGGACAGAGGTGGGGATCCTATCACAAAAGTTCACATGAGAGCCATGAAGTCTATGATTTCATTCCTACTCTTCTTCTTT
ATGTACTATTTGAGCACTATTATGATGAATTTGGCCTATGTCATCCTAGATAGTTTGGTGGCAAAGATTTTTGCTAATAC
ACTAGTATTTTTATATCCATCTGGCCATACATTTCTTCTGATTTTATGGACCAGCAAATTGAAACAGGCTTCTCTCTGTG
TCCTGAAGAAGCTGAAGTGCCTGCATCTAAGGAAACCCACACACCCATAA
>PatiTAS2R408A_NW_006711765.1:2417709-2418635
ATGGTAACCGTACTACTGAGCATTTTTTCCATCGTAGTAATAATAGAATTTCTTCTAGGAAATTTTGCCAATGGCTTCAT
AGCACTGGTGAACTTCATTGACTGGACCAAGAGACAAAAGATCTCCTCGGTTGATCACATTCTCGCTGCTCTGGCTGTCT
CCAGAATTGGTTTGCTCTGGGTAATGATAATAAATTGGTATGCAACTTTGTTCAATCCAGATTTCTATAGCTTAGAAGTA
AGAATTATTTTTCAAATTGCCTGGGCAGTAAGCAATCATTTTAGCATCTGGCTGGCTACTAGCCTCAGCATATTTTATTT
GTTCAAAATAGCCAACTTCTCCAGCTGTATTTTCCTTCGCCTCAAGTGGAGAGTTAAAAGCGTCGTGCTTGTGATGCTGC
TGGGGTCTTTGTTCTTATTGTTTTCTCATGTTGCAGCAGTGAGCATATATGAGAAAGTGCAGACTAAGGCATACGAAGGG
AATGTCACTTGGAGGACCAAATGGACGGACATGGCACACCTCTCAAATATGACTGTATTCACACTAGCAAACTTCATACC
CTTTGGTATGTCCCTGACGTCTTTTGTGCTGTTGATCTTTTCCCTCTGGAAACATCTCAAGCGGATGCAGCTCTGTGGCA
AGGGATCCCAAGATCCCAGCACCAAGGTCCACATAAGAGCCATGCAGACGGTCGTCTCCTTTCTCTTGTTCTTTGCCGGT
TACATTCTGAATCTAATTGTTACAGTTTGGAGTTTTAACGGGCTGCAGAAGGAACTGTTCATGTTTTGCCAGGTACTTGC
CTTCGTGTATCCTTCGATCCACTCGCTGATGTTGATTTGGGGAAACAAGAAGCTAAAACAGGCCTTTCTGTCTGTTTTAT
ACCAGGAGAAGTACTGGCTGAAAGAACAGAAACACTCAACTCCATAG
>PatiTAS2R408B_NW_006711765.1:2396835-2397734
ATGGTAACCGCACTACCGAGCATTTTTTCCATCATGGTAATAATAGAATTTCTCCTAGGAAATTTTGCCAATGGCTTCAT
AGCACTGGTGAACTTCATTGACTGGACCAAGAGACAAAAGATCTCCTCGGTTGATCACATTCTCGCTGCTCTGGCTGTCT

CCAGAATTGGTTTGCTCTGGGTAATGATAATAAATTGGTATGCAACTTTGTTCAATCCAGATTTCTATAGCTTAGGAGTA
AGAATTATTTTTCAAATTGCCTGGACAGTAAGCAATCATTTTAGCATCTGGCTGGCTACTAGCCTCAGCATATTTTATTT
GTTCAAAATAGCCAACTTCTCCAGCTGTATTTTCCTTCGCCTCAAGTGGAGAGTTAAAAGCATAGTTCTTGTGATTCTGT
TGGGGTCCTTGTTCTTTTTGGTTTTTCATGTTGTGGTGGTGAGCGTACATGAGAAAGTGCAGACTGAGGTATATGAAGGA
AACGGCACTAGGAAGACCAAATTGAGGGACATTTTACAGCTCTCAAATAGGACTATATTCACACTAGCAAACTTCATACC
CTTTGGTATGTCCCTGATGTCTTTTGTGCTGTTGATCTTTTCCCTCTGGAAACATCTCAAGAGGATGCAGCTCTGTGATA
AGGGATCCCAAGATCCCAGCACCAAGGTCCACATAAGAGCCATGCAGACTGTGGTCTCCTTTCTCTTGTTCTTTGCCGGT
TACTTCTTTACTCTGACGATCACAATTTGGAGTTCTAATTGGCTGCAGAACGAGTTCGGCTTTCCCCCTTTCCCAGTGAT
TGGAATCCTATATCCTTCAATCCACTCGTTGATGCTGATTTGGGGAAACAAGAAGCTAAGACAGGCCTTTCTGTCATTTC
TGTGGCAGCTGAAGTGCTGA
>PatiTAS2R38_NW_006712036.1:624766-623762
ATGTTGGCTCTGACTCCTGTCATAACTGTGTCCTATGAAGTCAAGAGTGCATTTCTATTCCTTTCAATCCTGGAATTTAC
AGTGGGGGTCCTGGCCAATGCCTTCATTTTCCTGGTGAATTTTTGGGATGTGGTGAGGAAGCAGCCACTGAGCAACTGTG
ATCTTATTCTTCTGAGTCTCAGCCTCACCCGGCTTTTCCTGCATGGGCTGCTGTTTCTGGATGCCATCCAGCTTACATAC
TTCCAGAGGATGAAAGATCCGCTGAGCCTCAGCTACCAGACCATCATCATGCTCTGGATGATCACAAACCAAGTTGGGCT
CTGGCTCACCACCTGCCTCAGTCTTCTCTACTGCTCCAAGATTGCCCGTTTCTCTCACACCTTCCTGCACTGTGTGGCAA
GCTGGGTCTCCCGGAAGGTCCCCCAGATGCTCCTGGGTGCAATGTTTTTCTCTTGTATCTGCACCGCCATCTGTTTGGGG
GACTTTTTTAGCAGATCTGGCTTCACATTCACAACTATGCTATTCGTGAATAATACAGAATTCAATTTGCAAATTGCAAA
ACTCAATTTCTATCACTCCTTCATCTTCTGCACACTGGTGTCCATCCCGTCATTGTTATTTTTTCTGGTTTCTTCTGGGG
TGCTGATTGTCTCCCTGGGGAGGCACATGAGGACAATGAGGGCCAAAACCAAAGACTCCCGCGACCCCAGCCTGGAAGCC
CATATCAAAGCCCTCAGATCTCTTGTCTCCTTTCTCTGCCTCTATGTGGTGTCATTCTGTGCTGCCCTCGTTTCAGTGCC
TTTACTGATGCTGTGGCACAACAAGATCGGGGTAATGATCTGTGTGGGGATCCTAGCAGCTTGTCCCTCGATACATGCAG
CAATCCTGATCTCAGGCAATGCCAAGCTGAGGAGAGCTGTGGAGACCATTCTACTCTGGGTTCAGAACAGCCTAAAGATA
GGGGCAGACCACAAGGCAGATCCCAGGACTCCAGACCTATGTTGA
>PatiTAS2R42_NW_006711765.1:2350564-2351532
ATGTTAGCCGGACTGGATAAAATCTTTCTTACGCTGTCAACAGCAGAATTCATAATTGGAATGTCGGGGAATGTGTTCGT
TGGAGTGGTGAACTGCTCTGAATGGATCAAGAACCAAAAAATCTCTTTTGTTGACTTCATCCTCACCTGCTTGGCTCTCT
CCCGAATCACTCAGCTGCTGGTGTCATTGTGGCAATCATTTGTAATGACACTATCTCCGCCTTTCTATTCCACTTGGAAA
TCAGCAAAACTTATTACTTTGCTTTGGAGAATAACTAATCACTGGACTACCTGGTTTACCACCTGCCTGAGCATTTTCTA
CCTCCTTAAAATAGCTCACTTCTCCCACTCTTTCTTCCTCTGGCTGAAGTGGAGAACGAACAGAGTGGTTCTTGCCATTC
TTGTCCTTTCTTTGCCCTTTCTGCTGTTTGACTTCCTGGTGCTAGAATCATTGAATGATTTCTTCTTAAACGTCTATGTG
ATGGATGAAAGTAATCTGACATTACATATAAATGACAGTAAAAGCCTTTACATTAAAACCCTGATTCTTCTTAGTTTTTC
CTATACCATTCCTATTGTTCTGTCCCTGACCTCACTGGTCCTATTGTTTCTGTCCTTGGTAAGACACATCAGAAATTTGC
AGCTCAAGTTCATGGGCTCCAGGGACCCCAGCACACAGGCCCATAAGGGGGCCATTAAAATGGTTATGTCTTTCCTCCTC
CTCTTCACAGTTCATTTTTTTTCCATCCAATTGACAAACTGGATGCTTTTGATATTTTGGAACAACAAGTTCACTAAGTT
TATCATGTTGGCCATATATGTCTTTCCCTCGGGCCACTCATTAATTTTGATTCTGGGAAACAGCAAACTGAGACAGACAG
CCTTGAAGGTACTGCGGCATCTTAAAAGCACCTTGAAAAGAGAAAAAACAGTTTCGTCTTTACAGATAGACGTTCCAGGG
TCTTTCTAA
>PatiTAS2R67_NW_006711765.1:2358081-2359019
ATGCCATCTGGAATCGAAAATACCTTTCTGACAGCAGCAGTAGGAGCATTCATGATTGGAATGTTGGGGAATGGTTTCAT
CGTACTCGTCAACTGCATTGACTGGGTGAAGCATCGAAAGCTCTCGCCAGCTGACTGCATCCTCACCAGCCTGGCTGTCT
CCAGAATCATTCTTCTTTGGATGATACTATTCGATTCGCTTGTAATGGTGTTTTGGCCACATCTATATAACATTGAGAAA
CTAGCTACCGCTGTTAGTATCTGTTGGACAGTGACCAATCACCTAGCTACCTGGTTTGCCACCTGCTTGAGTGTTTTCTA
TTTCTTTAGGATAGCCAATTTCTCCCACCGCTGTTTCACCTGGCTGAGGCAAAGAATTAGCAGGGTGCTCCTTGTGCTTC

CTCTGGGGTCTTTATTCTTACTGGTTTTCAACTACAAATTATTAGTTGGATTTTCTGATCTCTGGGCTACCATCTACCAC
AACTATGAAAGAAACTCAACTCGGCCCCTAGATGTAAGTAAAACTGTGTATCTTAACAGCTTGGTTATTCTCAGTTTCAT
CTACTTAATCCCCTTCCTTCTGTCCCTGACCTCACTGCTCCTTTTATTTCTCTCCTTGATGAGACATACCAGGAACATGC
AACTGAACTCCAGCTCGAGGGACTTCAGCACAGAGGCCCATAGAAGGGCCATGAAAATGGTGATGTCTTTCCTCCTCCTC
TCCACGGTTCATTTTTTTTCCATCCAGTTAACAGGTTGGATTTTCCTTTTACTGAAGAAACATCATGCCAATTTGGTGGT
CACGTTGACATCGGCTCTTTTTCCTTCAGGCCACTCATTGATCCTCATTTTTGGAAACAGCAAACTGAGACAAACTGCTT
TAGGACTACTGTGGCATCTCAATTGCCACCTGAAAATGGTGAAACCTTTAGCTTCATAG
>PatiTAS2R5P_NW_006712036.1:477423-478309
ATACTGTCTGCCGCCCTAGCACTGCTGATGGTGGTGAGAGTGGCTGGATTTCTCATTGGCCTGGTTGGAAATGGAGTCCT
TCTGGTTTGGAGTTTTGGAGAACGGGTCAGAAAATTCAACGGGTCCTTATACAACCTCATTGTCCCGGGCCTGGCCGTTT
GCCGATTTCTCCTGCGGTGGCTGATTATGGTGGACTTAACCCTGTTTCCGCTTTTCCAGAGCAGCCGTGGGCTTCGCTAC
CTCAGTGTCTTCTGGATCCTGGTAAGCCAGGCCAGCTTGTGGTTTGCCACTTTCCTCAGTGTCTTCTACTGTAGGAAGAT
CACAGCCCTTGAACACCCTGTCTATGGCTGAAGCAGAGGGCCTATCGCCTGAGTCTCTGGTGCCTTCTGGGGGTACCTCA
TGATAAATTTGTTACTTGTGGCCCACAGTGGCTTAATGTCCAAAATCCTTCCCAAGGCAACAACAGCATTATGTGTACCC
TTTCAAACTGGTACTACATGCATATATTAAAGCTCACTGCAGGAAGCGGGTTGCCTTTCATGGTGTTTCTTGTTCCTTCT
GGGATGCTGATTGATTGTCTCTTTGTGTAGACACCACAAGAAGATGAAGGTGCATACAGCTGTAAGAGAGATGCTTGGGC
CCAGCCTCACATCACTGCCCTGAAGCCCTTGACCTGCTTCCTTATCCTTCGCGTGGGTTATATCCTGGCCAGCCCCTTTC
CCATCACCTCCAAGTATTCTGCTAATCTCCCCGTTGTCTTCATTTCCAAGACCCTCATGGCTGCCTATCCTTCTCTTCAT
TCTGTCATATTGATCATGGGGAATCCCAGGATGAAGCAGACTTGTTAGAGAATCCTGTGGAAGATAGTGTATGCTTGGAG
ATCCTGA
>PatiTAS2R8P_NW_006711765.1:2495894-2496822
ATGCTCTGTACAGAAGGCAACATCTTCATGATCATAATAACTGGAAAATTCATAATAGGAATTTTGGGGAATGTATACAC
TGGACTGGTAAACTGGATTGACTGGATTAAGAAGAAAAAGATCTCCTCAACTGACTATATCCTCCCCAGTCTAGCCATCT
CCAGAATGTGTTTGCTCTGTATAATGATAGTAAATATCATCATAATGATATCTCTACCCAGATTTTTATGAAAATGCTAA
ACTAAAAAGCCATCATGCATATCATCTGGACACTTGCCAACTACTTAAGTATATGGTTTGCCACCTGCCTCAATGTTTTC
TATTTCCTCAAGATAGCCAATTTCTCCCACCCACTTTTTTCTCTGGCTGAAGAGGGAGAATTGACAGAGTGATTCACTGG
ATTCTGCTGGGCTGTTTGGCCATCTCCTCCTTGGTCAGCCTTCTACTGGCAATGACACCAAATTATGATTAGGAGTTTCA
TAAAATTGCAGAACATAAAAGTAACTGCACTGGAATGTTCCATGTAAGTAAGAGTCAATTCTTCAACCCGCTGACCCTCT
TTAACCTGTTGGCAATTGTCCCATGGACTGTGTCATTGATCTCACTTTTTCTTTATGTCCCTACAGAGACATGTCAAGCA
AATGAAACCCAGTGTTACAGGCTGTGGAGGTCCCAGCACAGAGGCCCAGGCAGGAACCATGAAAACTATGACTTCATTTC
TCTTCCTCCTTTTTGTGTGTTATGAGGTTTCCCTTTTGGCGACTTTTAGCCACCTTATGAAAGAAAGCAAATTCGCTGTG
ATGTTTGGAGAAGCTATACCAATTCTCTATCCTTCTGGTCATTCACTCATTTTAATTATTGGGAATAACAAGCTGAGGCA
GGCATCTATCAGGATGCCAAGATATGGCAAAACAGCCTGGATGATGTAA
>PatiTAS2R10DP_NW_006711765.1:2477932-2478899
ATCGTAAGCATAGTGGAAGGCCTTCTCATTTTTATAGCAGTTAGTGAATCAGTACTGGGGGTTTTAGGGAATGGATTTAT
TGGACTTGTAAACTGTATGGACTGTGTGAAGAACAAAAAGTTTTCTATGATTGGCTTCATCCTCACCGGCTTAGCTACTT
CCAGAATTTGTCTGATATTGATAGTAATTGTAGATGGATTTATAAAGATATTCTCTCCAGATATGTACTCTTCTGGTCAC
CTAATTGATTATATTAGTTACTTATGGATAATTATCAATCCATCAAACATCTGGTTTGCCACCAGCCTCAGCACCTTCTA
CTTCCTGAAGATAGCAAATTTTTCCCACCACACGTTTCTCTGGTTGAAGGGTAGAATCAATTGGGTTCTTCCCCTTCTGA
TGGGATCCTTGTTTATTTCATGGCTCTTTACGTTCCCTCAAATTGTGAAGATTCTTAGTGATAGTAAAGTGGGGAATGGA
AACGCAACCTGGCAGCTCAACATGCCGAAGAGTGAGTTCTTTACTAAGCAGATTTTGGTCAATGTAGGAGTCCTTCTCCT
CTTCACGCTATTCCTGATTACATGTTTCCTGTTAATCATTTCCCTTTGGAGACACAGCAGGCGGATGCAATTGAATGTCA
CTGGATTCCAAGACCCCAGTACAGAAGCGCATATGAAAGCCATGAAAGTTTTGATATCTTTTCTCATCCTCTTTATCTTG
CATTTTATAGGCCTGGCCATAGAAATAGCATGCTTCACAATGCCAGAAAAAAAATTGCTGTTTATTTTTGGTATGATGAC

CACAGTCTTGTACCCCTGGGGTCACTCATTTATCCTCATTCTCGGAAACAGCAAGCTAAAGCAAGCCTCTCTGAGAGCAT
TGCAGCAGGTCAAGTGCTGTTAAGACAGGGCGACTGCTCCCAGCCGCAGAGACTCATGTGGGGAGAAATGGATGTCCTAG
AATGTTCTAG
>PatiTAS2R16P_NW_006711986.1:11728676-11727811
ATGATGCCCCTCCGGCTCACTATCTTCTTCATAACCATCTATGTGCTCAAATCCTTGAGAGTAATTATGCAGAGGAGCTT
AATGTTTGCAGTGCTGGGCAGAGAGTGGGTGCAGGCCAAAAGGCTGTCATCGGTGGACTTGAGTCTCATATGCCTGGGTA
TTTGCTGCTTCTGTCTACAGTGGGCATCTGTCCTGTACAATTTTTGCTCCTATTTTAACCCTAACTATGTATTTTGGTAC
TTATCAATCACCTGAGAATTTACTAATACTCTTACTTTCTGGTTAACCAGCTTGCTTGCTGTCTTCTACTGTGTCAAAGT
CTCTTCCTTTACCAGTCCACCTCTTCTAGCTGAGGTGGAGAATGTTGAGCCAAGGTTTGTTCCTTGGCTGTTGCTGGGTT
CTTTGTTGGTTTTTTGTGTGTCAATCATCTTTTCAACTATTAGGAATTGTGTCAATGTTCGCTTAATCACCGTGGGGCGT
TTCTCTACAAACAGCACTATGGTTAAGGGACTTAAGACATTCCATTTGTTTACCATATCTCATCTAATGGTTGCATTGGT
TATTCCTTTCCTCCTGTTCCTGGCCTCCACCATCCTGCTCATGGCCTCACTGTTCCAACACATGGAGCAGATGCAACACC
ATAGCACTGGTCACTGCATGAAAGCTCACACCACTGCCCTGACGTCTCTCACCATCTTCCTCGTCCTCTTCACCTCTTAC
TTGCTAACCCTACTCATCTCTATTATGAGTATCTCATTGGATAAGAGGTCTTGGTTCGGGGCTTGGGGAGCTATCATCTG
TGCTATAGTCTCTATTCACGCCGCTTAACTAATGCCGAGCATCCCTAAATTGAAAAGGTTTTCAAGATAACGTGCTGGGG
ACTAGAGACTGACTGA
>PatiTAS2R39P_NW_006712036.1:1411521-1412561
ATGAATAGTGGGAGCTACTGAGGATCTGCCTATCAAACGCTAGAGAGACATTTTCCCCCAGACACCAAACAGGGGTAACA
ACTCAGGATGACAAAAACCTGCAATCCTGCAGATAATGAATTTTCACCATTTCACATCCTCTCAATTTTAACAATTATAG
GCACTGAATGCATCATTGGTATCGTTGCAAATGGGTTCATCATGGCTATAAATACAGCTGAATGGATTAAAAATAAGGCA
GTTTCCAATAAGCAGGATCCTGTTTTTCTTGAGTGTATCCAGAATACCTCTCCAAAGCTTCATGATGATAAAAATTACCT
TCAGCTCAACATCCCCACATTTTTATAATGAAGATGTTATATATGGTACATTCAAAGTAACTTTCATGTTCTTAAATCAT
TGTAGCCTCTGGTTTGCTGCCTGGCTCAGCTTCTACTTCGTGAAGATCGCTGATTTCTCCCACCCCCTTTTCCTCAAGCT
GAAGTAGAGAATTTCCGGGACGGATGCCCTGGCTCTGTGGCTATCAATGTTTATTTCCTTAGGCTACAGTGTGCTCTTCT
CCAATGACATCAACACCATGTATTGTAACAATTCTTCTATCCCTCTCCCAACTCCTCTAAGAAAAAATACTTCACTCAGA
CCAATGTGGTCAACCTGGTTCTTCTCTATAACCTGGGGATCTTCATTCCTCCGATCGTGTTCATCTTTGCAGCCACCCTG
CTGATCATCTCTCTCAAAAGACACACCCTACACATGGAAAGCAATGCCACTGGCTCACATGGGGACCATCAAAGTTACCA
GCTACTTTCTCATTCTCTACATTTCCAATGCAGTTGCTCTATTTCTTTATATGTCCAATATCTGTGACGCCAACAGTTCC
TGGATTATTTTGTGCAAATTCATCATGGCTGCCTACCCTGTTGGTCACTCCATTCTGCTGATTCAGGACAACCCTGGGTT
GAGAAGAGCTTGGAAGCAGCTTCAGCCTCAAATTCATCTTCACCTAAAAGAGAAC
>PatiTAS2R40P_NW_006712036.1:1435627__1436604
ATGGCGACAGTGAACACAGATGCCATGGATAGAGACACGTCCAGGTTTAAAATCGTCCTCACCGGTGCTCTCTGGAGTAG
AGTGCATGACTGGCATGATTGGGAATGGCTTCATCACTGCCATCCATGGGGCTGAGTGGGCCAGAGGCAAAAGGCTCCCT
GTGAGTGACTGTACCCTGTTGAAGCTGAGCTTCTCCAGGCTTTTGCTGCAGATCTGGATGATGCTGGAGAATATTTACAG
CCTATTCTTCCTGGTCACTTATAACCAAAACACAGTGTTTATAACCTTCAAAGTCATCACCATGTTTCTGAACTATTCCA
ACCTCTGGCTTGCTGCCTGGCTCAACATCGTCTCTTGCCTTCAAATTGCAAACTTTGCTCACTGTTTGTTCTCCATGATG
AAGAGGAAAATCAGAGAGCTGATGCCTCGGCTTCTGGGGCTGTCACTGTTCATCTCCTTACGCTTCACCTTTTGCTTCTC
TAAAGATATCTTCAATGTGTACATAAATAGTTCCGTTCCTATCCCCTCCTCCAACACCACTGAGAAGAAATACTTCTCTG
AGACTAACATGGTCAACCTGGTTCTTCTCTATAACCTGGGGATCTTCATTCCTCTGATCATGCTCATCTTTGCAGCCACT
CTGCTGATCATCTCTCTCAAGAGATACACCCTACACAAGAGATACACCCTACACATGCCACTGGCTTCAGGGACCCCAGC
ATGGAGGCTCACATGGGGGCGATCAAAGCTATCAGCTACTTTCTCATTTTCTATATTTTCAATGCAGTTGCTCTATTCAT
TTCCATGTCCAACATCTTTGACATCAACAGTTCCTGGAATATTTTGTGCAAAATCGTCATGGCTGCTTACCCAGCTGGCC
ACTCAGTGCTACTGATCTTGGGCAACCCTGGGCTGAGAAGAGCCTGGAAGAGGTTTCAGCACTGTGTTCATCTTCATCTA
TAAGAGCAGACCCTGTGA

>PatiTAS2R41P_NW_006712036.1:1659551-1660470
ACGCAGCCAGGGCTCTCAGCCTTCTTCATGCTGCTCTTCGTCCTGCCGTGCCTTCTGGGAATCCTGGCCAGTGGCTCATT
GTGCTGGTGCTGGGCAGGGAAAGGCTGCAGCGGGGGAGGCTGCCTCCCTCTGGCATGATCCTCCTGAGCTTGGGCGCCTC
CCGCTTCTGCCTGCAGTGGGTTGGAACGGTGAACAGCTTCTACTGGCCGAGTACAGGAGAGGTCCTGCACGGCAGTTCTT
TGGTCTCCATTGGGACTTCCTGAACTCAGCCACCTTCTGGTTCAGCTCCTGGCACAGTGTCCTCTTCTGCATGAAGATCA
CTAACTTCACCCACCCTACCTTCCTCTGGGTCAAGTGGAGGTTCCCAGGGTCAGTGCCCTGGCTCCTCATGGCTCCTCTC
CTGATCTCCTTCATCGTCACTCTGCTCTTCTTTTGGGGAAACCGTGCTGTGTGTGAAGGATTCTTAATTAGAAAGTTTCC
TGGGAACATGACCTTCAGACAGTGGAGCAGGACACTGGAAGTTCACTACTTCTTGCCCCTGAAGCTGATCACCTTGTTAG
TTCCTTGCTCTGTTTTTCTGGTCTCAGTCGCGCTGTTGATTAATTCTCTGAGGAGGCACACCAGGAGGATGCGGCTCAGC
GCCCACAGGCCACAGGACCCCAGTGCCCAGGCTCACACCAGAGCTCTGAAGTCGCTCGTCTCCTTCCTCATTCTTGATGC
TCTGTCCCTCGCATCCCTGGTTATCGATGCTGCAGGTTTCTTCTCAGAGAGTGACTGGTACTGGCCGTACTGGCCGTGGC
AGATTTTAATCTACCTGTGCATGTCTGTCCATCCGTTTTTCCTCATCTCCAGCAACCTCAGGCTTCGAGGGGTGTGCAGA
CAGCTGCTGCTGCTGGCCAGGGGCTTCTGGGTGGCCTAG
>PatiTAS2R62P_NW_006712036.1:1628519-1629441
ATGCCCTCACCTGCATTGATCTTCACGGTCATCTTCTTCCTGGAGTCACTGGCTGAAATGTTGCAGAATGGCTTCATGGT
TACTGTGCTGGGCAGGGAGTGGGTGCGATGCCGGTTGCTGCCCGCGAGTGACATGATTGTGGCCTCCTTGGCCGCCTCCC
GGTTCTGCCTGCATGGGTGGCCATCCTGAACAACCTCTTCACCTTCTTTGATTTTCGTTCCATAATGGACTATTTCAGCA
TCCTCTGGAGCTTCTTCAACACTCTCTCTTCCTGGCTCACCGCCTGGCTTGCTGTCTTCTGTGTGAAGACCTCCATCTTC
TCCCACCCTGTCTTCTCCTGGCTGAGGTGGAGGATTTCTCAGTCAGTGCCCAGGCTGCTGCTGGGCTCCCTGCTCATGGC
TGGTCTGGTGGTCATCTCATCAACCATTGGGACTACATTTCCTGTGCAGGTGACTGCCTCCCAGAATTCCCAAGGAAACA
GGACCCTGGCTGATAGACTACAGAGCTATTGGCGCTTTTTTCATCCTCATGCAATGCTTATGTGGTTGATCCCATTCCTT
CTGTTCTTGGCGTCCACGCTTTTGCTCATGTGCTCCCTGCATCGGCACTTGGGGCAGATGAGGGATCATAGACTGGGTCC
ATGTGATCCCAGCACCCAGGCTCACATCATGGCCCTGAAGTCACTTTCCTTCTTCCTTGTCTTCTATACATCATATTTTC
TGTCCCTGATTGTCGTTACTATGAAAATCGCAACCTTCCAGGGTCACTGGCGCTGGGCCTGGGAAGTGGTGACCTACACA
GGTATCTGTCTGCACTCCAGCATCCTGATGCTAAGCAGCCCCAAGCTGAGAAAGGCCCTGAGGACCAGGCTTTGGAGAGC
TCTGGACAAAGGCGGGTTTATCTCAAGTTATCAGTATCAATT
>PatiTAS2R4T_NW_006712036.1:463545-464446
ATGCTTCAGATACTCTTCTTATCTGCTCTTACTGTCTCAGCAATTTTGAATTTTGTAGGACTCGTTGTAAATCTGTTTAT
CATAGTGGTCAACTACAGGACTTGGGTCCAAAGCCACAGAATCTCCTCTTCTAATAGGATCCTGTTCAGCTTGGGCGTCA
CCAGATTTATTATGCTAGGACTGTTTCTCCTGAACATTATCTACCTGTTCACCTCTCCACATGTCGAAAGGTCAGTCCAC
CTATCCACTTTTTTCCTGTTGTGTTGGATGTTTTTGGAGTCTACTAGTCTCTGGCTTGTAACCTTGCTCAATGCCTTGTA
CTGCGTGAAGATTACTGACTTCCAACACTCATTTTTCCTCCTGCTGNGATCCCCAGGCTGTTGCTGGCCTGCGTGCTGAT
CTCTGCCTTCTCCACTCTCCTGTATGTTGTGCTCACACAGACATCACCCTTTCCTGAGTTTCTGACTGGGAGCAATGGTA
CAGTATGTGACATCAATAAGAGCATCTTGTCTTTGGTGACCTCCTTGGTCCTGAGCTCCTTTCTCCAGTTCATCATGAAT
GTGACTTCCGCTTCCTTGTTAATACATTCCTTGAGGAGACATATACAGAAGATGCAGAAAAACGCCACTGATTTTTGGAA
TCCCCAGACTGAAGCTCATATGGCTGCTATGAAGCTAATGATCTATTTCCTCATCCTCTACATTCCATATTCACTTGCTA
CCCTGCTACAGTATCTCCCTTCCGTACGGATGGATTTGGGAGCCACATCCATCTGTATGATTATTTCCACCTTTTATCCT
CCAGGGCACTCTGTTCTCATTATTCTCACACATCCTAAACTGAAAACAAAAGCAAAGAAGATTCTTTGTTTCAACATATG
GTGGAATTTCAGTAGTAAATAG
>OdroTAS2R2_NW_004450305.1:4534248-4535162
ATGGCCTCCTCTTTGTCAGCTATTCCTCATGTTATCATCATGTCAGCAGAATTTATCACAGGGATTACAGTAAATGGATT
TCTTATCATCGTCAACTGTAAAGAATTAATCAAAAGCAGAAAGCTAACACCAATGCAACTCCTTTTCATATGTATAGGGA
TGTCGAGATTTGGTCTGCTGATGGTGTTAATGGTACAAAGTTTTTCTCTGTGTTCTTTCCATTCTTTTATAAGGTAAAA
GTTTATGGTGCAGCAATGTTGTTCTTTTGGATGTTTTTAGCTCTGTCAGTCTCTGGTTTGCCACCTGCCTTTCTGCATT

TTACTGCCTCAAGATATCAGGCTTCACTCAGCCCTATTTTCTTTGGCTGAAATTCAGGATCTCAAAGTTAATGCCTTGGC
TGCTTCTGGGAAGCTTTCTGGCCTCCATGAGCACTGCAGCTCTGGGTATTGAGGCAGATTACCCTAAAAACATAGACAGT
GATGATGTCCTCAAGAATGCCACGCTGAAGAGGACTGGACCCAAGATAAGGCAAATTAATGAAGTGCTTCTTGTCAACTT
GGCATTACTATTTCCTCTAGCCGTATTTGTGATGTGCACTTTTATGTTATTCATTTCTCTCTACAAGCACACTCATCGGA
TGCAAAATGGATCTCATGGTGTTAGAAATGCCAGCACAGAAGCCCATATAAATGCATTAAAAACAGTGATAACATTCTTT
TGCTTCTTTATTTCTTATTTTGCTGCCTTCATGGCAAATATGACATTCAGTGTTCCTTATGGAAGTCATTGTTTTTTTGT
ACTCAAGGACATAATGGCAGCATTTCCCTCTGGCCATTCAATTATAATCATCTTGAGTAATTCTAAATTCCAGCAATCTT
TCAGGAGACTTCTCTGCCTCAAAAAGAATCAGTGA
>OdroTAS2R7_NW_004450790.1:233604-232666
ATGCCGGATAAAGTGGAGACCACCTTAATGCTCATGGCAGCTGGAGAGTTTTCAATGGGGATTTTAGGAAATGCATTCAT
TGGACTGGTGAACTGCATGGGTTGGATCAAGAATAGGAAGATCGCCTCCATTGATTTAATCCTCACAAGTCTGGCCATAT
CCAGAATTTGTCTATTATGTACAATACTATTAGATTGTTTTATATTGGTGCTGTATCCAGACGTGTATGCTACCGGTAAA
CAAATGAGAATCATTGACTTTTTCTGGACACTAACCAACCATTTAAGTGTCTGGTTTGCCACCTGTCTCAGCATTTTCTA
TTTCCTCAAGATTGCGAATTTCTTCCATCCTCTTTTCCTCTGGATGAAGAAGAGAATTGACAGTGTGATTCCTAGGATCC
TGCTGGGGTGCTTGGTCCTCTCTGTGTTTATTAGCTTTTTTGTCACTGAGAATTTGAATGATGATTTCAGGTATTGTGTT
AAGACAAAGAAGAAAACAAACTTAACTGTGAGATGCAGAGTAAATAAAGCTCAATATGCTTCTATCAAGATTTGCCTCAA
CCTGTTAACGCTATTCCCCTTTTCTGTGTCTCTGATCTCATTTCTCTTCTTGATCCTCTCCCTCTGGAGACATACCAGGC
AGATGAAACTCAGTGCCACAGGGTGCAGAGACTTCAGCATAGAAGCCCACGTGGGAGCCATGAAAGCTGTCATCTCCTTT
CTCCTCCTTTTCATTGCCTACTGTTTGGCCTTTCTCGTAGCCACCTCTAGCTACTTTATGCCAGAGACTGAATTAGCTGT
GATCACTGGTGAGTTGATAGCTTTAATCTATCCCTCAAGCCATTCATTTATCTTAATTCTGGGGAGCAATAAATTAAGAC
AGGCATCCCTAAGGGTGCTATGGAAAGTAAAGTATATCCTAAACAGGAGAAATTTCTAA
>OdroTAS2R42_NW_004450790.1:381601-380627
ATGTCAGCTGGATTGGATATAATCTTTCTTACACTGTCGACAGCAGAATTCATAATTGGAATGTTGGGGAATGTGTTTAT
TGGACTGGTAAACTGCTCTGAATGGGTCAAGAACCGAAAGATCTCTTTAGCTGACTTCATCCTCACCTGCTTGGCTATCT
CCAAAATCACTCAGCTATTGGTGTCATTGTTTGAGTCATTTATGATGGGATTAAATCCACCTTTCTATTCCACTTATAAA
CTAGCAAAACCTGTTGCTTTGCTTTGGAGAATAACTAATCATTTGGCCATCTGGTTTACTACCTGCCTAAGCATTTTCTA
CCTCCTTAAGATAGCTCAGTTCGCCCATTCCCTTTTCCTCTGGCTGAGGTGGAGAATGAACAGAGTGGTTCTTGCAATTC
TTGTATTTTCTTTGTTCTTTCTGCTGTTTGACTTTCTATTGCTAGAAACATTTAATGATCTCTTCTTGAATATCTATGCA
ATGGATGAAAGTAATCTGACTTTATATATAAATGAAAGTAAAACTTTTTATATTAAAACCCCGATTCTTCTTAGTTTTTC
CTATATCATTCCTATTGTTCTGTCCCTGACCTCATTGCTTCTTTTATTTCTGTCCTTGGTAAAACACATCAGGAATCTGC
ATCTCAACTCCATGGGCTCCAGGGACTTCAGCGCACAGGCTCATAAAAAGGCCATTAAAATGGTGGTGTCTTTCCTCTTC
CTTTTCACAGTTCATTTTTTTTCCATACAATTGTCAAATTGGATGCTTTTTTTACTTTGGAACAACAGGAGCACAAAGTT
TATCATGTTGGCCATATGTGTCTTTCCCTCAGGCCACTCATTAATTTTGATTCTGGGAAACAGCAGGCTAAGACAGACAG
CCTTGAAGGTACTATGGCATCTTAAAAGCTCCCTGAAAAGAGAAAAACCAATTTCCTCTTTACAGAAAGACCTTCCAAAG
CCTTTCTAA
>OdroTAS2R62_NW_004451143.1:736616-737533
ATGCCCTCCTCACCTGCATTGATCTTCATGGTCATCTTCTTCCTGGAGTTGTTGGCTGCAATGCTGCAGAATGGCTTCAT
AGTTACTGTGTTGGGCAGGGAGTGGGTGCGACGCCGGGTGCTGCCCGCAGGTGACATGATTATGGCCTTCCTGGCCGCCC
GGTTCTGCCTGGACAGGGTGGCCATCCTGAACAACCTCTTGACCTTCTTTGAAATGGACTATTACCACATACCCTGGAAC
TTCATCAACACTCTCGCTTCCTGGCTCACTGCCTGGCTTGCCGTCTTCTGTTGTGTGAAGATCACTGTCTTCTCCTACCC
TGTTTTCTTCTGGCTGAAGTGGAGGATTTCTCGGTCAGTGCCCAGGCTGCTGCTGGGCTCCCTGGTCTTCGCTGGTCTGA
CAGTCATCTCATCAGCCACTGGGACTAGAATTCTTATGCAGATGATTGCCTCCCCGAGTTCCCAAGGAAACAGCATCCTG
GCTGATACAGTACAGCCCTTCTATTGGTGTTTTATTCTACCTCATGCAATGCTTACATGGTCAGTCCCATTCCTCCTGTT
CTTGGTGTCCACGCTCTTGCTCATGTTCTCACTGTGCCAGCACTTGGGGCGGATGAGGGACCATAGACTGGGCCCATGTG

ATCCTAGTACACAGGCTCACACCATGGCCTTGAAGTCACCTGTCTTCTTCCTTGTCTTCTATACATCATATTTCCTGTCT
CTGATTGTTGTTTCTATGAAAATCACAGCCTTCCAGAGTCACTGGTACTGGGCCTGGGAAGTGGTGACCTATGCAGGCAT
CTGTCTGTACTCCAGCATCCTGGTGCTAAGCAGCCCTAAGCTGAGAAAGGTCCTGAAGACCAGGCTTTGGAAAGCTCTGG
ACAAAGGCTGGTCTGTCTCAAGTTCTCAGTATCAATAA
>OdroTAS2R1P_NW_004451176.1:759649-758760
ATGCTAGAGTTTTACCTTGTTATCCATTTTCTTTTTTCAGTGTTACAATTTCTCATCGGGGTTTTAACAAATGGCATCAT
TGTGGTTGTGAACGGCACTGAGTTGATCAAGCAGAGAAAGATGATTCCATTGGCTCTCCTTCTTTCCTGCCTGGGGATTT
CCAAGATTTGTCTGCAGTCGGTCATCTTCTCCATTAATCTGGCTATTGTCTCCTTGATTGAAGTCCCTCTACCTGTTGAG
AATTTTGTAATTTTCGTGTTTGTAAAAGAATTGGGACTTTGGTTTGCCTCGTGGCTCGGTGTTTTCTACTGCGCCAGGAT
TGCCCCCATAGTTCACCCACTCTTCTTCTGGTTGAAGATGAGGATATCGAAGTTGGTGCCATGGCGGATCCTCGGGTCCC
TGTTGTATGCTTCCATCCCTTCTATTTTCTACAACAAATATACATGGGTTCTTTCCCAGCAACTCTTGTTGGGCTTTTTC
TCCCCCAACGCAACAACTCAAATCAAAGAAACATCTGCTTTACAGATTGTCTTTCTTGTGAGGTTATTATTGCCGTTATT
TATCTCCCTCGCTTCTGCTCTGCTCTTGGTATTTTCCCTGGGGAGACACACCTGGCAGATGAGAAACGCGGCACCAGGGT
CCCTAGCACAGGTGTCCACGTGAGATCGCTTCTGTCCGTTCTGTCCCTCCTGGTCTTCTGCCTCTCCCACTACATGACAG
CTGCTTTGCTCTCTTCTCAGATTTTTAAGCTCAGGAGCCTCAAGTTTATGTTCTGTATCTGTGTGTTTGGGTCATATCCT
TCTGGACACTCTACGATCTTAATTTTAGGAAATCCTAAACTGAAGCAAAATGCAAGGAAGCTCCTCCTCCATGGGAAGTG
CTGCCATTGA
>OdroTAS2R3P_NW_004451203.1:148727-147767
ATGTCAGGGCTGGAGAAATGGATGTTCCCGGTTCTGTTTGCCACTGAGTTCATTCTGGGGATGCTGGGGAATGGTTTCAT
AGTATTGGCCAATGGCAGCAGCTGGTTCAAGAACAAGACAGTCTCTTTGTCTGACTTCATCGTCACTAACCTGGCTCTCT
CCAGCATCGTTCTGCTGTGGATTCTCTTGGTTGATGGTGTTTTAATGGTGTTCTCTTCCAAAATACATGATGAAGGGATA
GTGATGCAAATTATTGATATTTTCTGGACATTTACAAACCACCTGAGCATTTGGCTTGTCACCTGTCTCAGTGTCCTCTA
CTGCCTGAAAATTGCCAGTTTCTCCCCTCCTACATTCCTCTGGCTCAAGTGGAGAGTTTCCAGGGTGGTCGTACACATGA
TTTTGGGTGCCGTGTTCTTATCGTGTGTCAGTGCCATGTCTCTGATCCAGGAACTTAAGATCTATTCTTAAGTTCCCACT
ATTCCTCAGTGGGATCAAAGGCACAGGGAATGTGACCGAGCACTTTAGAAGGAAAAGAAATGAATACAAAGTGATCCATG
TTCTTGGGACTCTGTGGAACCTCCCTCCCCTAATTGTGTCTCTGGCCACCTACTTTCTGCTCATCCTTTCCCTGGGGAGA
CACGTGCAGCAGCTGCAGCAAGGCGGCATCAGCTGCAGAGATCCAAGCACTGAGGCCCACCAGAGAGCCATCAAAATCAT
CATCTCTTTCTTCTTTCTCTTCCTGCTTTACTTTCTGGCCTTTTTGATTACATCATCCAGTTATTTCATACCAGGATCTG
AGATGGTTAATATAATCGGGGAAGTAGTTACAATGTTTTATCCTGCTAGCCACTCATTCATTCTCATTCTGGGAAATAAG
CTGAAGCAGACGTTTGTGGAGATGCTGTGGTGTGAGCCTGGCCATTGGAAGCCTGGATTCAAGGGACCTTTTGCCCCATA
G
>OdroTAS2R4P_NW_004451203.1:139187-138290
ATGCTTCAGATATTCTTCTTCCCTGCCATTATTGTCTCAGCAGTTTTGAATTTTGCAGGACTCATTGTGAATCTGTTTAT
TGCAGTGGTTAATTATCAGACTTGGCTCAAAATCTCCAGAATCTCCTCTTCTAATAGGATCCTCTTCAGCTTGGGCATCA
CCAGATTTCTTATGCTGGGACTGTTTCACTCAACATCATCTACTTCTTCATCTCTCCAAATGTGGAAAGGTCAGTGCACT
TATCTACTTTTTTCCTGTTGTGTTGGATGTTTTTGGACTCTAATAGTCTCTGGCTTGTCACCTTGCTCAATGCCTTGTAC
TGTGTCAAGATTACGGACTTCCAACATGCAGTATTTCTCCTGCTGAAACGAAATCTCTCCCCAAAGATCCCCAGGCTGCT
GCTAGCCTGTGCTGATTTCTGTCTTCACCACTCTCCTGTATGTTGTGCTCAGACAGACATCATGCTTTCCTGAATTTGTG
CCTGGGAGAAATGGTACAGGATGTGACATCAATGAGAGCGTCTTGTCTTTGGTGATCTCTTTGGTCTTGTGCTCATTTCT
CCAGTTCTTCATTAATGTGACTTCTGCTTCCTTGTTAATACATTCCTTGAGGACACATATACAGAAGATGCAGAAAAACG
CCACTATTTTTTTGGAATCCCCAGACTGAAGCTCATGTGGGTGCTATGAAGCTCATGGTCTATTCCCTCATCCTGTACAT
TCCGTATTCAGTTCCTACTCTGCTACATTATTTCCCTTTTGTTGGGATGGATTTGGGAGCCAGATCCATCTGCATGGTTA
TTTCCACCATTTACCCTCCAGGACATACTGTTCTCATTATTCTCACACATCCTAAACTGAAAACAAAAGCAAAGGAGATT
CTTTGTTTCAACAAGTAG

>OdroTAS2R5P_NW_004451203.1:120282-119399
ATGCTGACCCCTGCCCTAGCACTGCTGTTGGCGGTGGCGGTGGCCGAATTTCTCATTGGCCTGGTAGGAAATGGAGTCCT
TATGGTCTGGAGTTTTGGAAAATGGGTCAGAAAATTCAACAGGTCCTCATACAACCTCATTGTCCTGGGCCTGGCTGTCT
GTCGATTTCTCCTGCAGTGGCTGATTATGATGGACTTAAGCCTGTTTCCACTTTTCCAGAGCAGCCATTGGCTTTGCTAT
CTCAGTTTCTTCCGGATCCTGGTAAGCCAGGCCAGCCTGTGATTTGCCACTTTCCTCAGTGTCTTCTACTGCAGGAAGAT
CATGACCCTTGAGCACCCTGTCTGCTTGTGGCTGAAGCAGAGGGCCTATTGTCTGAGTCTCTGGTGCCTTCTGGGGTACC
TTATGATCAGTTTGTTACTTGTAGCCCGCATTGGCTTAAAGCCCTATAATCCTTTCCAAGGCAACGGCAGCATTCTGTAC
CCTGTTAAAAGCTGGCACTACCTTATATATTAAAGCTCAATGCAGGAAGTGGGTTGCCTCTGATGGTATTTCTTGTTTCT
TCCGGGATGCTGATTGTCTCCTTGTATAGACACCACAAGAAGATGAAGGTGCATACAGCTGGTAGGAGAGATGCTCGGGC
CAAGGCTCACATCACTGTCCTAAAGTCCTTGGGCTGCTTCCTTATCCTTCATGTGGTTTACATCCTGGCCAGCCCTTTTT
CCATCACCTCCAAGTCCTCTGCTAATCTTATTGTCTTCATCTCTGAGACAGTCATGACTGCCTATCCTTCTCTTCATTCT
GTCATATTGATCCTGGGGAATCCCAGGGTGAAGCGGACTTGTCAGAGAATTCTGTGGAAGACAGTGTGTGCTTGGAGATC
CTAG
>OdroTAS2R8P_NW_004450790.1:238187-237258
ATGCTCAGTACAGAAGACAACATCTTTGTGATCATTATAACTAGAGAATTCATAATAGGAATGTTGGGGGAATGTATACA
TTGGACTAGTAAACTGGATTGACTGGATTAAGAAGAAGAAGATCTCCTCAATTGACTATATCCTCACCAGTCTAGCCATC
TCCAGAATTTGTTTGCTCTGTGTACTGATACTAAATGGCATCATAGTGGTATGCTACCCAGATTTTTATGAAAATGATAA
ACTACAGGCAGTCATTAATATCTTCTGGACACTCACCAACTACTTAAGTACATGGTTTGCCACCTGGCTCAATGTCTTCT
ATTTGCTCAAGATAGCCAATTTCTCCCATCTGCTTTTTCTCTGGCTAAAGAGAAGAATTGACAGAGTGATTCACTGGATT
CTGCTGGGTTGTTTGGCCATTTCCTCTTTGATCAGCCTTATACCAGCAACACCACCAAATTATGATTATGAGTTTCATAA
AATTATAAATCATAAAAGAAACTGCACTGAAATGTTCCATGTGAGTAAAAGTCAATACTTCAACCCTTTGACTCTCTTTA
ACCTGTTGGAATTGTCCCATGTACTGTATCATTGGTCTCATTTTTCCTTTTAATTATGTCCCTAAGGAAAGATATCAAGC
AAGTGAAACTCAGTGTTACAGGCTGTGGAGACCCCAGCACAGAGGCCCATGTGAGAGCCATGAAAACTATGACTTCATTT
CTCTTCCTCCTTTTTGTATACTATGGGGCTTCTCTTTTGGCGACTTTTAACTATCTTATGAAAGAAAGCAAGTTAGCTGT
GATGTTAGGAGAAATTATAGCAGCTCTCTATCCTTCTGGTCATTCACTTATTTTAATTATTAGAAATAACAAGCTGAGGC
AGACATATATCAGGAGGCTGAGATACGAGACAACAGTCTGCATGATGTAA
>OdroTAS2R9P_NW_004450790.1:241173-240097
ATGACAGACCTATACACCATTCTTCACTGGTCAGCTGAAAGCCAATTCATTGTCAGTGATATCCCAAATCAGAACTCTGC
TGACAGGCTAAGTACAACAGAGGTAATATATACCATCCTGATCGCTGGTGAATTGACTATAGGAATTTGGGAAAATGGAT
TTATTGTACTGGTTAACTGCACTGGCTGGCTCAAAAGGAGAGATACCTCCATGATTGACATTATCTTGGTGAGCTTAGCC
ATCTCCAGAATCTGTTTGTTGTGTGTGATATCTTTAGATGGCTTTATTAGATGTATCTCCCGGGATAGATATGCCAGTAG
CAAGCTGATGAGCATTGTGGATGTTTTCTGGACACTTAGCAATCATTCAAGTGTCTGGTTTACTTCTTGCCTCAGCATCT
TCTAGTTACTGAAGATAGCCAATATATCCCACCCGTTTTTCCTCTGGTTGAAACTAAAGATTAACAAAGTCGTCCTGGGG
ATTTTTCTGATGTCCTTCCTTACCTGTATAATTATTAGTGTTTCACTGAATGAGGACTTCTGGGATCCCTTCAAAGGCAA
TCATAAGGAAAACATAACTTGGGAATTCAAAGTGAGTAAAATCCCAAGTGCTTTCAAACTGGTTATCCTGAACCTGGGGG
CTATCATTCCCTTTGTTCTTTGCCTAACCTCATTTCTCTTGTTACTTTTCTCCCTATTTAAACACACCAAGCAGATGAAA
CTTTATGCCACAGGGTCCAGAGACCCCAGCACAGAGGCCCACATGAGGGCCATAAAGGCAGTGATCATCTTTCTGCTTCT
CTTCATTATGTACTATGCAGTCTTTCTTGTAGTAACCTCTAGCTTACTGATTCCTCAGGGAAAATTAGTGGTGATGTTTG
GTGGTATGATAGCTGTCATTTTCCCAACAAGCCATTTGTTCATCCTGATAATGGGGAACAGCAAACTGAGGGAGGCTTTT
CTTAAAGTGCTAAGGATTGTGAAGAGTTTCCACAAAAGAAGGAAACCTTTCGTTCCACAGAGAATCCTGAATACAAGGAG
AAAGAAATCAACAAAAGACCTTCTCTCTTCTCCCAATTGA
>OdroTAS2R10P_NW_004450790.1:254521-253551
ATGCTAAGCATACTGGAAGGCCTCCTCATTTTTATAGCAGTTAGTGAATCAATACTGGGAGTTTTAGGGAGTGGATTTAT
TGGCCTTGTCAACTGTATTGACTGTGTGAAGAACAAAAAGTTTTCTATGATTGCCTTTATTCTCACTGGCTTAGCTACTT

CCAGAATTTGTCTGATATTGATAATAATTACAGATGGATTTATAAAGATATTCTCTCCAGATATGTATTACTCTTGTAAC
CTAATTGATTATATTAGTTACTTATGTGTAATTATCTATCAATCAAGTATTTGGTTTGCCACCAGTCTCAGCATCTTCTA
TTTTCTGAAGATAGCAAATTTTTCCCACCATATTTTCTCTGGTTGAAGGACAGAATCAATAGGGTTCTTCACCTTCTGAT
GGGGACCTTATTTATTTCATGGTTATTTACTTTTCCACAAATTGTGGAGATGATTAATGATAGTAGAATGAAGAGTGGAA
ATACAACCTGGAACCTCAACATGCAGAAAAGTAAATTCTTTACTAAGCAGATTTTGCTCAATCTAGGAGTCATTCTTCTC
TTTACACTATGCCTGATTACATGTTTCTTGTTAATCATTTCCCTTTGGAGACACAACAGGCGCATGCAATTGAATGTCAC
CGGACCCCAAGACCCCAGTACAGAAGCACATGTGAAAGCAATGAAAGTTTTGATATCTTTTATCATCCTCTTTATCTTGT
ATTTTATAGGCATTGCCATAGAAATATCATGTTTCACTCTGCCAGAAAACAAATTGCTGTTTATTTTTGGTATGATGACC
ACAGCCATCTATCCCTGGGGCCACTCATTTATCCTAATTCTAGGAAACAGCAAGCTAAAGCAAGCCTCTCTGAGGGCCCT
GTGGCAATTCAAGTGCTCTGAGGCGGGGAGACTGCTCACAGCTGCATAAACCTGCGTGGGGAGAAATGGAAGTTCTAGGA
GAATAATCTAG
>OdroTAS2R12P_NW_004450790.1:274770-274497
ATGGCAAGCACATTTAAGAATATACTTACGATGATTTTTGCTGAAGAATTCATAATAGGGCTTTTGGGAAATGGATTCAT
TATACTGATTAACTATATTGATTGGATCAGGAGCTGGAAGTTCCTCCTGATTGACTTTATTCTTACCTGCTTAGCTCTTT
CCAGAATATTTCTGCTGTGCATAATAATGCTAGGCATAGGTGTAGATATAATTTGTGAGGAAATATGGTACAATGATAAT
CAACTGATGACTTTTTTGAAATCCTCCGGACAGGGCGCCTGGGTGGCTCAGTCGGTTAGGCGGCTGCCTTCGGCTCAGGT
CATGATCCCAGAGTCCTGGGATCGAGTCCCGCATCAGGCTCCCTGCTTGGCGGGGAGCCTGCTTCTCCCTCTCCCTCCAC
CTGCCTCTCTGCCTACTTGTGCTCTCTATCTCTCTGTCAAATAAATAAATAAAATCTTTAAAAAAAAAAATCCTCCGGAC
AGGATCCAATTATTTCTGCACAACCTGCACCACCTGCCTCAGGGTCTTCTACTTTCTCAAGATAGCCAACTTTTCCAATC
CCATTTTCTTCTGGATAAAACAGAGGACTCATAGACTGCTTAAATTATTGTCCTGGGGGTGCTTTTCTCTTTCTGCTGTC
CCTGCTTTTTAAGGATACAGTATTTAAGAACCTGATCAAAACCAGGGTAAACACTGAAAGCAATCGGACATTGAATTTCA
CAATGAGAAAATATGATTTATTAACTTCAAATATAGTCCTGAACATAATGTTCATCATCCCCTTTGGAGTGTCTCTGGCT
TCCTTTGTCCTTTTGATCGATTCTTTATGGAACCACGCCAGGTGGAGGAAGGGCACAGGTTCTGGGGATCTTATCACAGA
GGCCCATGTGCAAGCCATGAAGTCTGTGATTTCATTCCTACTCTTCTTTATGTACTACTTGAGCAATATTATAATATATT
TGGCCGATATCAGTCTAGACAGTTTGGTGGCAAAGTTTTGCTAATATGTTAGTATTTTCCTGTCCATGTGGCCATCCATT
TCTTCTGATTTTATGGAACAGCAAAGTGAGGGGGGCTTCTCTCTGTGCCCTGAGGAAGCTGAGGTGGTGCAGAAATCTAA
GGAAACCCACATACGCATAAAACATACCTGA
>OdroTAS2R16P_NW_004450478.1:3047894-3047452
CTTCATAATCATCTATGTGATCGAATCCTTGACAGTAACTGTACCGAGCAACTTAATTTTTGCAGTGCTGGGCAGGAAGG
CTGTCATCGATGGACTTGAGTCTTACCTGCCTGGGCATCTGCTGCTTCTGTCTACAGTGGGTGTCGGTGCTGAACAATTT
TTGCTCCTATTTTAACCCTAACTATGTATTTGGGTACTTATCGATCACCTGGGAATTTACTAATACTCTTACTTACTGGT
TAACCAGCTTACTTGCTGTCTTCCACTGGGTCAGAGTCTCCTCCTTTACCTGCCCCATCCTCGTCTGGCCAAGGTAGAGA
ATTTTGAGGTTTGTTCCTTGGCTGTTGCTGGATTCTCTGTTGATTTCTTTTGTGTCAATTGACACAAAAGCCTTTTCAGC
TATTAGGAATTATATGAATATTCACTTAATCACCATGGGGCAT
>OdroTAS2R38P_NW_004451520.1:289460-290429
ATGAAGTCAAGAGTGCATTTCTATTCTTTTAAGCCCTGGAGTTTGCAGTAGGGATCCTGACAAATGCCTTCATTTTCTTT
ATGAATTTTTGGGATGTGGTGAGGAGGCAGCCACTGAGCAACAGTGATCTTAACCTTCTGAGTCTCAGCCTCACCCAGCT
TTTCCTGCATGGGCTGCTGTTTTTGGATGCCATCCAGCTTATATACTTCCAGAGGATGAAGGACCCACTGAGCCTCAGCT
ACCAGACCATCATCATGCTCTGGATGATCACAAACCAAGCTGGGCTCTGGCTCACCACCTGTCTCAGTCTTCTCTACTGC
TCCAAGATTATCTGTTTCTCTCACACCATCTTGCTCTGCTTGGCAAGCTGGATCTCCAGGAAGGTCCCCCAGATGCTCCT
GGGTGCCATGCTTTTCTCTTCCATCTGCACTCTCCTCTGTTTGGGGGACTTTTTAGTAAATCTGGCTTTGCATTCACAA
CTATGCTATTCATGAATAATACAGAACTCAATTTGCAAATTGCAAAACTCAATTCCTATCATTCCTTCATCTTCTGCACC
CTGGAGTCCATCCCTCCTTTCTTGCTTTTTCTGGTTTCTTCTGGAGTGCTGACTGTCTCTCTGGGGAGGCACACAAGGAT
AATGACGGCCAAAACCAAGGACTCCCGTGACCCCAGCCTGGAGGCCCATAGCAAAGTACTCAGATCTCTTGTCTCCTTTC

TCTGCCTCTATGTGGTGTCATTCTGTGCTGCCCTCATTTCAGTGCCTTTCCTGATGCTGTGGCACAACAAGATCAGGGTA
ATGATCTGTGTAGGGATCCTAGCAGCTTGTCCCTCGATACATGCAGCAATCCTGTCTCAGGCAATGCCAAGCTAAGAAGA
CCTGTGGAGATCATTCTACTCTGGGTTCAGAGCAGCCTAAAGGTAAGGGCAGACCACAGGGAAGATCCCAGGATTCCAGA
TCTATGTTGA
>OdroTAS2R39P_NW_004451143.1:535325-536283
ATGACTGAAACCTGCAATCCCCCAGAAAATGAATTGTCACCATCTCTCGATTTTAACTATTATGGGCACTGAATGCATCA
TTGGTATCATTGCAAATGGGTTCATTATGGCTATAAATGCAGCTGAATGGACTAAAAATAAGGCAGTTTCCACAAGTGGC
AGAATCCTGTTTTTCTTGAGTGTATCCAGAATAGCTCTCCAAAGCTTCATGATGCTACAGATTACCTTCAGCTCAACATT
CCCACGTTCTTATAATGAAGACCATATATATGACATGTTCAAAGTAAGTTTCATGTTCTTAAATCATTGTAGCCTCTGGT
TTGCTGCCTGGCTCAGTTTCTTCTACTTCGTGAAGATTGTTGATTTCTCCCACCCCCTTTTCCTCAAGCTGAAGTGGAGA
ATTTCCAGATGGATGCCCCAGCTTCTGTGGCTTTCAGTGTTTATTTCCTGGGGCTACAGTGGGCTCTTCTCTAAAGACAT
CTACACTGTGTATTGTAACAATTCTTCTATCCCCTCCTCCAACTCCACTAAGAAAAAATACTTCACCTGAGACCAGTATG
GTCAACCTGGTTCTTCTCTATAACCTGGGGATCTTCATTCCTCTGATCATGTTCATCCTTTCAGCTACCTTGCTGATCAT
CTCTCTCAAGAGACACACCCTCCACATGGAGAGCAATGCCACTGGCTCCAGGGACCCCAGCATGGAGGCTCACATGGGGG
CCATCAAAGCTACCAGCTGCTTTCTCGTTCTCTACATTTTCAATGCAGTTGCTCTATTTCTCTATATGTCCAACACCTTT
GATGTCAACAGTTCCTGGAATATTTTGTGCAGATTCATCATGGCTGCCTACCCTGCTGGACACTCCGTTCTACTGATTCA
GGACAACCCTGGATTGAGAAGAGCTTGGAAGCGGCTTCAGCCTCAAGTTCATCTTTACCTAAAAGAGCAGACTCCATGA
>OdroTAS2R40P_NW_004451143.1:559184-560100
ATGGCCACGGTGAGCACAGATGCCACAGATAGAGACACATCCAGGTTTAAAATCGTCCTCACCTTGGTGGCCTCCGGAAT
AGAGTGCATCAATGGCTTCATCATGGCCATCCATGGAGCCGAGTGGGCCAGAGGCAAAAGACTCCCTGTCAGTGACTGCA
TTCTGATGATGCTCAGCTTTTCCAGGCTCTTGCTGCAGATCTGGATGATGCTGGAGAATACTTACAGTCTACTGTTCTGG
GTCACTTATAACCAAAACACAGTGTTTACACTCTATTTCAACCTCTGGCTTGCTGCCTGGCTCAAGATCTTATATTGTCT
TAGAATTGCAAACTTTGCTCACCCTTTGTTCTTCTTGATGAAGAGGAAAATCACAGGGCTGATGCCTTGGCTTCTGGGAC
TGTCACTGTTCATCTTCTTATGCTTCAGCTTTCCCTTCTCTACAAATATCTTCAACGTGTATGTAAATAATTCCATTCCC
ATCCCCTGCTCCAACACCACGGAGAAGTACTTCTCTGAGACCAACCTGGTTCTTCTCTATAACCTGGGGATCTTCATTCC
TCTGATCATGTTCATCCTTTCAGCCACCCTGCTGATCATCTCTCTCAAGAGACACACCCTCCACATGGAGAGCAATGCCC
CTGGCTCCAGCATGGAGGCTCACATGGGGGCCATCAAAGCTACCAGCTGCTTTCTCGTTCTCTACATTTTCAATGCAGTT
GCTCTATTTATTTCCATGTCCAACATCTTCGACGTCAACAGTTCCTGGAATATTGTGTGCAAAATCGTCACGGCTGCTTA
CCCGGCTGGCCACTCAGAGCTACTGATCTTGGGGAACCCTGGGCTGAGAAGAGCCTGGAAGAGGTTTCAGCACCATGTTC
ATCTTCACCTGTAAGAGCAGACTCCATGACTGGAACC
>OdroTAS2R41P_NW_004451143.1:768259-769157
ATGCAGCCGGCACGCTCAGTCTTCATGCTGCTCTTTGTCCTGCTGTGTCTCCTGGAAATCCTGGCCAATGGCTTCATTGT
GCTGGTGCTGAGCAGAGAAAGGATGCGGCGTGGGAGGCTGCTTCCCTCTGACATGATCCTCATTAGCTTGGGTGCCTCCC
GCTTCTGCCTGCAGTGGGTTGGAATGGTGAACAACTTCTGCTACATCCTCCACCTGAACGAGTACAGCCAAGGACCTGCC
CGGCAGTTCATTGGTCTCCACTGGGACTTCCTGAACTCGGCCACCTTCTGGTTTGGCTCCTGGCTCAGTGTCCTCTTCTG
CATGAAGATTGCCAACTTCACCCACCCCACCTTCCTCTGGCTGAAGTGGAGGTTCCCAGGGTCAGTGCCCTGGCTCCTCA
TGGCTTCTCTCCTGATCTCTTTCATTGTCACCCTGCTCTTCTTTTGGGGAAACCAGGCTGTGTATCAAGGATTCTTAATT
AGAAAATTTCCTGGGAACATGACCTTCAAGCAGTGGAGCCAGAGGCTGGAAATTCACTATTTCTTGCCCCTGAAACTTAT
CACCTTGTCAGTTCCCTGGCTCTGTCTTTCTGCTCTCAATTGCACTGTTGATTAATTCTCTGAGGCGACACACAGGGAGA
ATGCAACGCAGCACCCACAGCCTGCAGGACCCCAGCGGCCAGGCTCATACCAGAGCTCTGAAGTCACTCATCTCCTTCTT
CATTCTTTATGCTCTGTCCTTCGCATCCCTGGTCAGAAAGTGACTGGTACTGGCCATGGCAGATTTTAATCTACCTGTGC
ACATCTGTCCATCCCTACATCCTCATCCTCAGCAACCTCCAGCTTCGAGGGGTGTGCAGTCAGCTACTTCTGTTGGCCAA
GGGCTTCCAGCTGGCCTAG
>OdroTAS2R408P_NW_004450790.1:311925-310993

ATGGTAACTTTACCACAGGGCATTTTTTTCCATCCTAGTAATAACAGAATTTGTTCTAGGAAATTTTGCCAGTGGCTTCA
TAGCAGTGGTGAACTGCATTGACTGGGTCAAGAGACAAAAGACGTCCTCAGCTGATCAAATTCTCACTGCTCTGGTGGTC
TCCAGAATCGGTTTGCTCTGGGTAATGTTAATAAACTGGTATGCAACTGTGTTGAATCCAGCTTTATATAGTTTAGAAGT
AAGATTTCTTGTTCATTTGCATGGACAGTAAACAATCATTTTAGCATCTGGCTTGCTACTAGCCTCAGCATATTTTATTT
GTTCAAAATAGCCAATTTCTCTAACCTTATTTTTCTTCGCCTGAAGTGGAGAGTTAAAAGTGTAGTTCTTGCGATACTGT
TGGGGTCTTTGTTCTTTTTGGTTTTTCATGTTGCAGTGGTAAACATATGTGAAAAAAGCATACATGAGAAAAATTCCTTC
ATAAGCAAGATGAAGGAATATGAAGGAAACATCACTAAGCAGACAAAATTGGGGAACACTGTAAGCCTTTTGAATAGGAC
TGTATTCACGCTAGCAAACTTTGTGCCCTTTGCTATATCCCTGACATCTTTCCTGCTCTTAATCTTTTCCCTGTGGAAAC
ATCTCAAGAAGATGCAATCCAGGGGTAGAAGATCCCAAGATCCCAGCACCAAGGTCCACATAAGAGCCATGCAGACTGTG
ATCTCCTTTCTCTTGTTATTAGCTGGTCACTTCCTGACTCTAATTGTCACAGTCTGGAGTTCTAATGGGCTGCAGAACAA
ACTATTCTTCATGCTTTGCCAAGCTTTTGGATTCTTGTATCCTTCAAGCCACTCATTTATCCTGATCTGGGGAAACAAGA
AGCTCAAACAGGTCTTTCTGTCTGTTTTATACCAGGGGACGTGCTGGCTGAAA
>OdroTAS2R67_NW_004450790.1:373121-372187
ATGCCATCTGGAATTAAAAATGCTTTTCTGGTAGCAGCAACAGGAGAATTCATAGCTGGAATGTTGGGGAACAGCTTCAT
TGTACTGGTTAATTGCATTGACTGGGCGAAGAATCAAAAGCTCTCATCAGCTAACTGCATTCTCACCAGCCTGGCTATCT
CCAGAATTGTTCTTCTTTGCATAACACTATTCAATTCATTTTTAATGGTGTTCTGGCCACATCTTTATGCCATTGATAAA
CTAGCAAGATTCGTTAGTATTTTTTGGACACTGAGCGATCACCTAGCTACCTGGTTTGCCACCTAAGTGTTTTCTACTTC
TTTAAAATAGCCAATTTCTCCCATCCCTGTTTCATCTGGCTGAGGTGGAGAATTAACAGAGTGCTACTTGTGCTTCCACT
GGGGTCTTTATTCTCACTGTCTTGCAACTTTGAATTATTAGATACATTTACTAATTTCTGGGTAAATGTCTATCAAAGAT
ATGAAAGAAACTCAACTTGGTCCCTAGATATAAGGAAAACTCTGTATCTTAACAGCTTGATTGTTTTCAGTTTCATCTAC
TTAATCCCCTTTCTTCTGTCCCTGACCTCACTGCTCCTTTTATTTCTTTCCCTGATGAGACATACCAGGAATGTGCAACT
GAACTCTGGCTCTAGGGACTTCAGCAGAGAGGCCCATAAAAAGGCCATGAAAATGGTGATGTCTTTCCTTCTCCTCTCCA
CGGTTCATTTTTCTACCGTCCTATTGACAGGTTGGGTTTTCCTTTTACTGCAGAATTGTCAGGTCAATTTGGTTGTCATG
TTATTATCAACTCTTTTTCCTTCAGGCCACTCATTTATTCTGATTTTGGGAAACAGCAAGTTGAGAAAAACTGCTTTAGG
ACTACTGTGGCATCTTAATCGCCACTTGAAAATGGTGAAACCTTTATCTTCATAG
>BubuTAS2R1_NW_005784622.1:1265060-1264170
ATGCTGGAGTCTCACCTTGTTAGTCACCTTGTTTTGACAGTGGTACACCTTCTCTTTGGGATTTTAGTAAATGGCATCAT
TGTGATTGTGAACGGTACTGACTTCATCAAGCAGAGAAAGTTGATCCCACTGAATCTCCTTGTTTCCTGCTTGGCGATTT
CCAGGATGGGAATTCAGCTGGCCTTCTTCTACACTAACCTGGCTCTTCTTTCCTTGATCAAATTCCCTCAATTTACTGAG
ACGCTTGTAGTCTTCACATTTGTAAATGATTTGGGACTTTGGTTTGCCACCTGGCTCAGTGTCTACTACTGCACCAAGAT
TGCTACCATCGCTCACCCTCTCTCGTTCTGGTTGAAGATGAAGATCTCCAAGTTGGTTCCTTGGCTGATTCTTGCGTCCC
TGCTGTATGCATGTAGTACTTCTGCTGTGCATGTCAAATATAAGTGGGTATTTTACGGAGAAGACTTCCTGGGCCTTTTC
TTCCCAAATGTAACAACTCACATCAAAGTAACCCCTACTTTACAGTTTGCCTTTCTGTTTGCTGAGTTTGCATTGCCACT
GCTCATCTTCCTGATTTCTTCTCTGCCCTTGATATTTTCCTTGGGAAGACATGCCTGGCAGGTGAGAAACACATGGACAG
GCCCCAGAAACCCTCACACACGTGCATACATCAGGGCCTTTCTCTCCATCCTGTCCTTCTTGGCCCTCTATCTCTGCCAC
TACCTGATAATTGCTTTGATCTTTTTTCAAATTTTTAACCTTAGAAGCTTTCTATTTCTGTTCTTCACCTTCGTGGTTGG
TTCATACCACTCCGTCCACTCTATTACTTTAATTTTAGGAAACCCAAAAATGAAACAAAATGCAAAGACATTGTTCCTCC
TCAGAAAGTGA
>BubuTAS2R4_NW_005784086.1:45143-44253
ATGCTTCGGATAGTCTTTTTTTCTTCTGTCGTTGTCTCTGAAATTTTAACTTTTGTAGGACTCATTGTGAATCTCTTCAT
TGTAGTGGTCAGTTACAAGACTTGCATCAAAAGCCACGGGATCTCTTCTTCTGACAGACTCCTGTTCAGTTTGGGCATCA
CCAGATTTTTTATACTGTTACTGAATGTTGTTGTCATCATCTCTCCAAATGTGGAAAGGTCAGTCTCCTTATCCTATTTT
TTCCTGTCATGTTGGATGTTTTTGGACTCTAGTAGTCTTTGGTTTGTAACCTTGCTCAACGTCTTGTATTGTGTGAAGAT
TGCTAACTACCAACACTCAGTGTTTCTCCTGCTGAAACGAAATCTCTCCACCAAGATGCCCCGGCTGCTGCTGGTCTGTA

TGCTACTTTCTGTCTTCACCACTCTCCTGTATGTTATGCTCAGAAAGTTGGCACCCTCTCTTGAATTTGTGACTATGAGA
AATGGCACAGTATTTGACATCAATGAGGGACTCTTGTCTTTGGTGACTCCTTTGGTCTTGAGCTCATTTCTCCAATTCAT
CATTAATGTGACTTCTGCTTCTTTGTTAATCAATTCCTTGAAGAGACATATACAGAAGATGCAGAGAAGTGCCACTGTTC
TTTGGAATCCCCAGACTGAAGCTCATGTGGGTGCTATGAAGCTGATGATCTGTTTCCTCATTCTCTACATTCCATATTCA
GTTGCTACTCTGCTCCATTATCTCCCTCCTTCTATAGGGATGGATTTGAGAACCAAGTCTATTTATGTTATTACGTCCAC
CATTTACCCTCCAGGACATTCTCTTCTTATTATTCTCACACATCCTAAACTGAAAACAAAAGCAAAGAATATTCTTTGTT
TCAATAAATAG
>BubuTAS2R7_NW_005785690.1:1016461-1015523
ATGTCAAGTGAAGGGCAGAGTATCTCAATGCTCATAGCAGCTGGGGAATTTTCACTGGGGATCTTAGGGAACGCATTCAT
TGGACTGGTAAACTGTGTGGACTGGATCAAGCACAAGAAGATTGCCTCCATTGATTTAATCCTCACCAGCCTGGCCATCT
CCAGAATTTCTCTCTTATGTATAATACTATTGGATTGTTACATATTGGTCCTGTACCCAGATGTCTATACTGGTGGTAAA
CAAATGAGAATCATTGACTACTTCTGGACACTAACCAACCATTTAAGTGTCTGGTTTGCCACCTGCCTCAGCATTTTCTA
TTTCCTCAAGATAGCAAATTTCTTCCATCCCTTTTTCCTCTGGATGAAGTGGAGAATTGACAGTGCAATTCCTAGGATCC
TGCTGGGGTGTTTGGTCTTCTCAGTGTTCATTAGCCTTCCTGTCATTAACAATTTGGATGATGATTTCAGGCATTGTGTC
AAGATGAAGTTGAAAATAAATTTAAGTCGAAGATGCAGAGTACATAAAGCTCAGCATGAGTCCATCAAGATACGTCTCAA
TCTGTTGACACTACTTCCCTTTTCTGTGTCCCTGATCTCATTTCTCCTCCTGATCCTCTCCCTGTGCAGACACACCAGGC
GAATGCAGCTCCGTGCCCCAGGGAGCAGAGATCCCAGCACGGAAGCTCACGTGAGAGCCATGAAGGCTGTCATCTCCTTC
CTCCTCCTTTTCATTGCCTACTACTTGGCCTATCTTGTGGCCACGTCCAGCTACTTTATGCCAGAGACTGAATTAGCTGT
GATCATTGGTGAGTTGATAGCTTTAATCTGTCCATCAAGCCATTCACTCTTCCTAATTCTAGAGAACAAAAAATTAAGAC
AAGCATCTCTAAGGGTGCTTTGGAAGGTAAAATGTATCCTACGAAGAAGGAATTGCTAA
>BubuTAS2R8_NW_005785690.1:1022147-1021248
ATGTTCAGTATAGAAGACCACATCTTTCTGACCATAACGACTGCAGAATCCATCATAGGAATGTTTGTGAATGGATACAT
TGGACTAGTAATATGTGTTGATTGGATTAAGAAGAAAAAGATCTCCACAGCTGACTACATCCTCACCAGTTTAGCTCCCT
CCAGAATGTATTTGCTTTGTGTAATGACACTCAACGGCACCATACTGGCACTCTACCCAGGTGTTTATGAAAATGAGAAA
ATAAAGGTAGTTCTTAGTATCTTCTGGACATTCGCCAACTACTTAAGTATGTGGTTTGCCACCTGCCTCAATGTCTTCTG
TCTCTTCGAGATAGCCAATTTCTCCCACCGACTTTTTATCTGGCTAAAGTGGAGAATCGAGAGGATGGTTCACTGGAGCC
TACTGGGGTCCCTGGCCATTTCCATGTTGATCAGCCTTATACAAGCAATGTTAACAAATTCTGATTATGATTTTCTTAAA
ATTGCAAAACATAAAAGAAATGTCACTGAATTGTTCCATGTGAGTAAAATTCAATACTTCGACCCATTGACACTGTTTAA
CCTGTTTGCTATTATTCCATTTACTGTGTCATTGATCTCATTTTTCTTTTTAATTACATCCCTGTGGAGATACAGTAAAC
AAATGAAATCCAGTGTTACAGGCTCCAGAGACTCCAGCACAGAGGTCCACGTGGAGGCCAGGAAAACAGTGACCTCATTT
CTTTTCTTCCTTTTTGTATACTACCTGGCCTGTCTTTTGGCAACATTTAGCGACTTTATGAAAGAAAGCAAGTTAGCTAT
GATGTCTGGAGAGATTATAGCAATTCTTTATCCCTTAGGTCACTCACTGTTTTTAACTGTTGGAAATAACAAGCTAGGCT
GGCATCTGTCAGGACGCTGA
>BubuTAS2R10A_NW_005785690.1:1047016-1046117
ATGCTGAGTATAGTAGAAGGCCTTCTCCTTTTTGTAGCAGTTAATGAGTCAGTATTGGGGGTTTTAGGAAATGGGTTTAT
TGGACTAGTAAACTGCATTAACTGTGTGAAAAATAAGAAGATCTCTACACTCAGCCTTATTCTCACTGGCTTAGCCTCTT
CCAGATTTTGCCTGATATGGATAATAACTACAGATGCATACGTGAGAGTGTTTTCTCCAGATACGTATTTGTCTGGTAAT
CTAAGTCAATATATAGCTTACTTATGGATAATTATGAATCAATCAAGTGTCTGGTTTACCACCAGCCTCAGCATCTTCTA
CTTCCTGAAAATAGCCAACTTTTCCCACTGCATTTTTCTCTGGCTGAAGGGTCACATCACTAAGATTCTTCTTCTTCTAA
TGGGATGTTTGCCCATTTCATGGTTATTTACTTTTCCAAACATTACAGTGCCTTTTATTAATAATATTATGAAGAACAGA
AGCACAACTGGGTTGGTCACCATGCAGAAAAGTGAATACTTTATAAGTCAGATTTTGTTCAATATTGGAACATTTCTTGT
CTTTATACTATGCCTGATTACATGTTTCTTAATAATCACTTCCCTTTGGAGGCACAACAGGAGGATGCAATTGAATGCCA
CAGGATTCAGAGACCCCAGTACAGAAGCACATATCAAAGCAATGAAGATCTTGGTGTCTTTTATCATCCTTTTTATCCTG
TATTTTGTAGGCACTGCCATACAAATATTAAGTGTGACAGTGCCTGAAAACAAACTGCTGTTTATTTTCGGTATGACAAC

CACCATCCTCTATCCCTGTGGACACTCATTTATCCTAATTCTTGGAAACAGCAAGCTTAAGCAAGCCTCTTTGAGGGTAC
TGAAGCTATTAAAGTGCTAG

>BubuTAS2R10B_NW_005785690.1:1078613-1077681

ATGCTGAATATAGTGGAAGGCCTCCTCATTTATGTAGCAGTCAGTGAATCAGTATTGGGGGTCTTAGGGAATGGATTTAT
TGGAGTTGTAAGCTGTGTTGACTGTGTGAAAAGCAAGAAGATCTCTACTGTCAGCCTTATTCTCACTGGCTTAGCCTCTT
CCAGATTTTGCCTGATATGGATGATAATTACAGATGCATATATAAGGATGTTTTTCCAGATATATATTTGTCTGGTAAT
ATAAGTCAATATATAGTTCACTTATGGATAATTATGAATCAATCAAGTATCTGGTTTGCCACCAGCCTCAGCATCTTCTA
TTTCCTGAAGATAGCCAATTATTCCCACTGCATTTTTCTCTGGCTGAAGTGTCACATCAACAGGGTTCTTCTCCTTTTCA
TGGGGTCCTTGCTTATTTCATGGTTATTTGCTTTTCCAAGCATTGCAAAGCCTAGTACTAATAATATTATGAAGAACAGA
AGCACAACCTGGCTGATCACCATGCATAAAAGTGAATACTTGACAAATCAGATTCTGCTCAATATTGGAGTCATTCTTGT
CTTTGTACTATGCCTGATTACATGTTTCTTATTAATCACTTCCCTTTGGAGACACAACAGAAAGATGCGATTGAATGCCA
CAGGATTCAGAGATCCCAGCACTGAAGCACATATCAAAGCAATGAAGATTTTGGTGTCTTTTGTCATCCTCTTTATCTTG
TATTTTGTAGGCACTGCCATACAAATATCAGGTAGTAGTACTATGCCTGAAAACAAACTGTTTTTCATTATTGGTCTAAC
AACCAGACTCCTCTATCCCTGGGGACACTCATTGATTCTAATGCTAGGAAACAGGAAGCTGAAGCAAGACTCTTTGAGGG
TACTGAAGCCATTAAAGTGCTGGGAAAAAGAGAAACTTCTTAGAATTCCATGA

>BubuAST2R12_NW_005785690.1:1106092-1105163

ATGGAGAGAACATTGAACAATATACTTACAATCATTTCTGCTGGAGAATTCTTACTGGGTATTTTGGGAAATGGATTCAT
TGTTCTGGTTAACTGTATTGATTGGATCAGGAGCAGGAAGGTCTCCCTGATTGACTTTATTCTCACCTGCTTGGCTACTT
CCAGAATATTTGTGCTGTGCATAATGATTTCAAATACAGGTTTATATGTAATCTCTGAGGAAATACAGTACAACAAGAAT
CTCCTGATAAATTTGGAGTTCCTCTGGACAGGATCCAGTTATTTCTTCGTAGCCTGCACCACCTGCATCAGTGTCTTCTA
TCTCCTCAGAATAGCCAACTTTTCTAATTTCCTTTTCCTGTGGATGAAATGGAGAATTCACAAGGTGCTTCTCATTATTG
CACTGGGGGCTGTCTTCTCTTTCTGCTTGTGCCTTCTTCAAAAGAATATGGTAGTTGAAATCCTGTCCCAAAACCAGGTA
AATAGCAAAAAAAATATGACATTGCACTTTCTAATGATAAAATACAATTTGTTCCTTACCATAATGTTCCTCATCCCCTT
TGTAGTGTCCCTGGCCTCCTTTCTCCTTTTAATCCTCTCCTTATGTGGTCATCTCAGGCGTATGAAGGGTGTAGACTGTA
GCTCGGAGGCCCACGTGATAGCCCTGAAGGCTATGATTTCATTCCTATTCCTCTATGTTCTATACTATTTGAGCAATATT
ATAACAGGGTGGGCCAGTCACATTCTGGACAGTTTTGTGGCAAAGATTTTTGTGAACATGCTGTTATTTTTCTGTCCTTC
TGGCCACCCTTTGCTTCTGATTTTGTGGAACAGCAAATTGAAACAGGCTTCACTCTGTGTCCTAAGGAAGCTGAAGGGTT
ACATGAATCTAAGAAAACCTACTCTTCCAAAAATAACCCTGAAGCGATGA

>BubuTAS2R16_NW_005784472.1:180453-181358

ATGACAACCAGCCAACTCTCTGTCTTCTTCATGATCATCTATATGCTTGAGTTCTTGACAATAACTGGGCAGAGCAGCCT
GATTGTTGTAGCGCTGGGCAGAGAGTGGGTGCAGACTCAAAGGCTGCCACCTGTGGACATGATTCTCACCAGCCTGGGCA
TCTGCCGCTTCTGTCAACTGTGGTCATCGATGCTGTACAACTTTGGCTCCCACTTCTACCCTAATTACAATTTTTGGTAT
TTTGGAATCATCTGGGAATTTACTAACATCCTTTCCTTCTGGTTAACCAGCTTGCTTGCTGTCTTCTACTGTGTCAAAGT
CTCCTTCTTCAGCCACCCCATCTTCCTCTGGCTGAAGTGGAGAATTGTGAGATGGGTTCCTCGGCTGTTGCTGGGCTCTC
TGCTGATTTCTTGTGCATCTACCATCTTTCCAGCTACTAGTTATTACATTGATATTCAATTCATCTCGATGAAGCATTTC
CCTAGAAACAGCACCATGCTTGAGAGACTTGAGGCGTTCCTGTGGGATTTTACCACACTGTACAAAGTAGTTGTGTTGGT
TATTCCTTTCCTCCTGTTCCTGGCCTCCACAATCTTGTTCATGGCCTTATTATCCCGACATCTGAAGCAGATGAAAGACC
TTCACACAGGCTGCTCCAACTCCAGCCTGGAAGCTCACTCTGCCGCCCTGAGGTCCCTTGCCATCTTCCTCATCTTGTTC
ACCTTTTACTTTCTGACCGTGCTCCTCTCCATATTGGATGTCCTATTTAATAAAGAGTCCTGGTTCTGGGCCTGGGAAGC
TATCATCTATGCATTAGTCTCTATTCATTCTACTTTACTAATGCTGAGCAGTGCCAAACTGAAAAGAGTTTTAAAGGCAA
GGTGCTGGAGCCTAGAAGCTGCCTGA

>BubuTAS2R38_NW_005784422.1:524874-525866

ATGGTGACTCTGACTCACATCGCATCTGTGCCCTCTGAAGTCAGGAATGCATTTCTGTTCTTTTCAGTCCTGGAGTTTGC
AGTAGGGATCCTGCTCAACGCCTTCATTTTCTTGGTCAATTTCCGGGACCTGGTGAGGAGGCAGCCACTGAGCCACTGTG

ATCTTGTCCTATTGAGTCTCAGCCTTACCCGGCTTGTCCTACATGGGCTGCTCCTTCTGAAGGCTGTCCAGCTTACTCAT
TTCCAGCGGATGAAAGACCCGCTGAGCTTCAGCTACCAGACCATCATCGTACTCTGGATGATCGTCCACCAAGCCGGCCT
CTGGCTCGCCACGTGCCTTAGTCTCCTCTACTGCTCCAAGATTGTCCGTTTCTCTCACGCCTTCCTGTTCCGTGCAGCAA
GCTGGATCTCCAGAAAGATCCCCCAGATGCTTCTGGGTGCTGTGGTTCTCTCCTGTGTCTGCACTCTTCTCTGCTTATGG
GACTTTTTTAGTGGATCTCATTTCTCAGCTGTAACTAGGCTACTCATGAATAACAGTACTGAACTCAATTTGAACATTGC
AAAACTCAGTTTCTTTCATTCCTTCCTCTTCTGCAGCCTGGCATCCATCCCTTCTTTCTTGCTTTTCCTAGTTTCCTCTG
GGATGCTGGTGTTCTCCCTGGGGAGGCACAAGAGGATGATGAGGGCCAAAACCAGAGGCTCTGGGGACCCCAGCATGGAG
GCTCACACCCGGGCGCTCAGGTCTCTTGTCTCTTTCTTCTGCCTATATGTGTTGTCACTCTGTGCTGCCTTATTCTTGAT
GCCGTTGCTGACGCTGTGGCACAGCAAGGTCGGGGTTATGGTCTGCGTAGGGATAATGGCAGCCTGTCCCTCAGGACATG
CAGTCATCCTGATCTCAGGGAATGCCAAGCTGAGGAGGGCTGTGGACACCATTCTGCTTTGGGCAAAGAGCAGCTTCAAG
GTAAGGGTGGACCACAAGGCAGATCTGTGTTGA
>BubuTAS2R39_NW_005784386.1:27063-26017
ATGACGAGTGGGAGCTATCACAGACCCGCACATCAAGCGCTAAGGAGACATTTTCCTCCAGACATTGAAGAAAAGCAACC
ACTCAGGATGATCCAAACCTGCAGTTCCTCAGAAAATGATCTGTCACCATCTCTTGTCACTTTGATGTTAATAATTATCG
GCACAGAATGCATCCTTGGTATCCTCGCAAATGGGTTCATTGCAGCGATAAACACAGCTGAATGGATTCACAGTAAGGTA
CTCTCCACCAGTGGCAAGATCCTGCTTTTCCTGGGTGTATCCAGAATAGTTCTACAAAGCTTCATGATGCTAGAACTTAC
CTTAAGCTCAACATCCCCACAGTTTTATAATGATGACATCATGTATCACACATTCAGAGGATGTTTCATGTTCTTAAATC
ATTGCAGCCTCTGGTTTGCTGCCTGGCTCAGTGTCTTCTACTTCGTGAAGGTGGCAGATTTCTCCTACCCCCTTTTCCTC
AAGCTGAAGTGGAGAATTTCCGGGCTGATGCCCTGGCTTCTGCAGCTATCAGTGTTTGTTTCCTTGGGCCAGAGTGTGCT
CTTCTTCCAAAACAACTATACTATGAATTGTAACAATCTTTTTTCTCTCCCCTCCTTCAACTCCACTAAGAAAAAGTCCT
TCGCGGAGGCCACTGTGATCAACCTGTTTCTTTTCCTTAACCTGGGGATCTTCATCCCTCTGATCATATTTATGCTGGCG
GCCACCCTGCTGATCATCTCTCTCAAAAGACACATCTTCCACATGAAAAGCAACGCCACTGGCTGCAGAGACCCCAGCAT
GGAGGCTCACCTGGGGGCCATCAGAGCCATCAGCTATTTTCTCATTCTCTATATTTTCAAAGTACTTGCTCTCTTTCTCT
ACATGTCCAACTTCTTTGACATCAATAGTCCCTTGAATATTTTGTGCAAAATCATCATAGCTACCTACCCTGTGGGCCAT
TCCATTCTACTGATTCAGGACAATCCTGGGCTAAAAAGAGCCTGGAAGAGGCTTCAGGCTCAAGTCCACCTTTATTTTAA
AAAGTAG
>BubuTAS2R40_NW_005785465.1:834180-833224
ATGGTGACAGTGAACACAGATGCGATGGATAAAGACACGACCAGGTTTAAGATCGTCTTCACCTGGGTGGTCTCTGCAAT
AGAGTGCCTCATTGGCATTGCTGGGAATGGCTTCATCACCATCATCCATGGAGCCGAGTGGGTCAGAGGCAAAAGACTCC
CCATTGGTGACTGCATTCTGCTCATGCTGAGCTTTTCCAGGCTCTTGCTACAGATTTGGATGATGCTGGAAAACACGTAC
AGTCTGCTGTTCTGGGTCATCTACAATGAAAAAAGAGTATACATACTTTTCAAAACCATCGTCATGTTTCTGAACTACTC
CAACCTCTGGCTTGCTGCCTGGCTCAATATCTTCTATTGTCTTAGAATCGCAAGCTTTACTCACCCGTGGTTCTCCGTGA
TGAAAAGGAAGGTCATGGGGCTGATGCCTGGGCTTGTGAGGCTGTCCTTGTTCTTCTCCTTTTGCTCCAGCTTTCCCTTC
TCTAAAGGTATCTTCAACGTGTACGTGAACAATTCTGGCCCCGGCCCCTCCTCCAACTCCACTGAGAAGGTGTACTTCTC
CGAGACCAACATGGGCAACTTGGTTACCACCCTTTACCTGGGGATCTTCATCCCTCTGATCATGTTTATGCTGGCGGCCA
CCCTGCTGATCATCTCTCTCAAAAGACACACCTTCCACATGAAGAGCAACGCCACTGGCTCCAGGGACCCCAGCATGGAG
GCTCACCTGGGGGCCATCAGAGCCATCAGCTATTTTCTCATTTTCTACATTCTCAATGCAGTTGCTCTGTTTCTTTCCAT
ATCCAACATCTTTGCCGCCAACAGCTCCTGGAATATTTTGTGCAAAATCATCATGGCTGCCTACCCTGCTGGCCACTCAG
TGCTACTAATCTTGGGCAACCCTGGGCTGAAAAGGGGATGGAAGTGGTTTCAGCACCAAGTTCGTCTCTACCTGTAA
>BubuTAS2R41_NW_005785465.1:585391-584474
ATGCATCCAGCATTCACAGTCCTCTTCATGCTGCTCTTTGTCCTGCTGTGTATCCTGGGCCTCCTGGCCAATGGCTTCAT
TGTGCTGGTGCTGAGCAGAGAATGGGTGCGACGTGGGAGGCTGCTCCCCTCTGACCTGATCCTCTTTAGCTTGGGACTCT
CCCGCTTCTGCCTGCAGTGGGTTGGAATGGGGAATAACTTCTACTATTTCCTGCATCTGGTCGACTACTGCAGTGGTCCC
GCCCGGCAGTTCTTTGGTCTACCCTGGGACTTCCTTAACTCCGTCACCGCCTGGTTTGGCTCCTGGCTCAGCGTCCTCTT

CTGCATGAAGGTTGCTAACTTCACCCACCCTGGCTTCCTCTGGCTAAAGTGGAGGTTCCCCAGGTCAGTGCCCTGGCTTT
TGCTGGGCTCTCTCCTCACCTCCTTCATTGTCACCCTACTGTTTTTTTGGGGGAACCACGCTTTGTATAAAGAGTCCTTC
ACTAGAAAACCTTTCGGGAACATGACCTACCATCAGTGGAACAGGATTCTGGAAATGTACTATTTCCTGCCCCTGAAACT
GATCACTTTTTCAATTCCTGGCTCTGTTTTTCTGGTCTCGATTGCTCTGTTGATTGACTCTCTGAGGAGACACGCATGGA
GGATGCAGCACAGTGCTCACAGCCTGCAGGATCTCAGTGGCCAGGCTCACACCAGAGCTCTGAAGTCACTAGTCTCCTTC
CTTGTTCTTTATACTCTGTCTTTCATTTCCCTGATCATCGATGGTGAAGGGTTCTGCTCCTCAGAGAGTGACTGGTACTG
GCCATGGCAAATTTTAACCTACTCATGCACATCCATCCATCCCTTTATCCTCATCCTTGGCAACCTCAGACTTCGGGGGG
CATTTGGGCAGCTGATTTTGTTGGCCAGGGGCTTCTAG
>BubuTAS2R42_NW_005785690.1:1208982-1208053
ATGTTCCCTGGGTTGAGTACAGTCTTTCTGATACTATCAGGAGTGGAATTCTTAATCGGAATTCTAGGCAATGTGTTCAT
TGGACTGGTACTCTGCTCTGAATGCATTAAGAACCAAAAGACATCTTTATTTGACTTCATTCTCACTGGCTTGGCTATCT
CCAGAATCAGTCAACTGTTGGTGTTTTTTGTGGAGTCACTTGTGATGGGACTAGATTCACAGGTATTTGCCATTTTTAAA
CTAGCAAAACCCATTACTTTACTTTGGAGAATATCTAATCATTTGACTACCTGGCTCGTCACCTGTCTAAGTATTTTCTA
TCTCCTTAAGATAGCTCATTTCTCCCATTCTCTTTTTTTCTGGCTGAAGTGGAGAATGAACAGAGTCATTCTTGCGATGC
TTGCATTTTCTTTGGTCTTTCTGATTTTGGATATTCTTTTGCTAGAAACATTTAATGATCTCTTCTGGAATTTAATAAAT
GAAGACAATTGGACTTTAGTTGAAAGTAAAACTCATTATATTAAAAGTGAGAGTCTTCTTAGTTTCTCCTATTTCATTCC
TATTGTTCTGTCCCTGCTCTCATTGTTTATTTTATTTTGGTCCTTGGTGAAACACACCAGAAATTTGCAGCTCAATTTTA
TGGGTTCCAGGGACTTCAGCACAAAGGCCCATAAAAGAGCCATGAAAATGGTGACATCATTCCTCCTCCTTATCATGGTT
CATTTTCTTTTTACACAATTGGCAAATTGGATGTTTCATAGGTTTTTGGACAATAAGTTCACAAAGTTCATCATATTAGC
ACTATATGTCTTTCCTTCAGGCCACTCGTTCATGTTGATTCTGGGAAATAGCCAGTTAAGACAGATAGCCTTGAAGGTAC
TGAAGCATCTTAAAAGCTCCTTGAAAAGACAAAATCCATTGGCTTTATAG
>BubuTAS2R408A_NW_005785690.1:1168165-1167248
ATGATAGCTCTACTATCAACCGTTTTTTCCATCCTAGTAATAATACAATTTGTTCTGGGAAATTTTGCCAGTGGCTTCAT
AGCCCTGGTGAACTGCATTGCTTGGGTCAAGAGACAAAAGATCTCCTCAACTGATGCGATTGTCACTGCTATGGCAGTCT
CCAGAATTGTTTTGCTCTGTGTAATGTTAATACATTGGTATTATATTTTGCTTCATCCAGCTTTATATAGTTTAAAAGTA
AGAACTATTGTTCATGTTGCCTGGACAATAAGCAATCATTATAGCACCTGGCTTGCTACTAGCCTCAGTATATTTTATTT
GTTGAAGATAGCCAATTTCTCCAGCCTAACTTTTCTTCACCTGAAGTGGAGAGTTAAAAGTGTAGTTCTCATGATGCTTC
TGGGAACTTCATTCATTTTGGTTTTACAAGTTGTAGTTATAAGTGTAAGTGGGACTATGCAGAGAAGTGAATTTGAAGGA
AACTTCACACGGAAGGCTAAACTGAGGGATATTTTATGGCTTTCACAAGTGACCCTGCTCATTCTAGGAAACCTCACACC
CTTTACTATGTCCTTAATATCTTTTCTGCTACCAATCTTTTCCCTGTGGAAACATCTCATGAAGATGCAGCTCAATGGCA
AAGGATTCCAAGATCCCTGTACGAAGGTCCATATAAAAGCCATGCAAACTGTCGTCTCCTTTCTCTTGCTATTTGCCTTT
TACTTTCTGGTTCTAATCATATCAATCTGGAGGCCTAAAAAACTGCATGAGGAACCATTTCTCTTGCTTTTCCCAACAGT
TGAAGTCATCTATCCTTCAGTCCACTCATTTATCCTGATTTGGGGAAACAGAAAGTTAACACAGGCCTTTCTGTTGTTTC
TGTGGCAGCTGGGGTGCTGGCTGAAAGATAGGAAATAG
>BubuTAS2R60_NW_005785465.1:615539-614601
ATGGTTCCTGGAACTCAGTTGGTTGATAAGACAGCCCTCATCTGCATTATTATTTTATTCCTTTTGTTCCTGGTGGCATT
GGTAGGTAATGGCTTAATCATCGCGGCACTGGGCAGCGAGTGGCAGCTGCGGAGAATGTTGTCGCCCTGCGATAAGTTAT
TGGTCAGCCTGGGGGCCTCTCGCTTCTGTCTGCAATGGGTGGTGATTAGTAAGAACATTTACATTTTCCTGAATCCCACG
GCCTTTCCATACAACCCCGTGTTCCAGCTCCTGGCCGTTCAGTGGGACTTCTGGAACTCTGCAACACTGTGGTTCTCCAC
CTGGCTCAGTGTCTTCTACTGTGTGAAAATTGCCACCTTCACCCACCCCATCTTCCTCTGGCTAAAGCGGAATGTATCTG
GGTTGGTTCCTTGGATGCTACTCAGCTCTCTGGGGTTCTCTACCTTTACCACCGTTCTATTTTTCATAAGCAACCAGAAA
ATATATCAGAACTATTTAAAGAAGGGTCTGCAACCTTGGAATGTCACTAGGAATGCTGTGAGAACATATGAGAGGTTCTG
CCTCTTCCCTTTGAAAATTGTTACCTGGACCGTCCCTACTGTTATCTTTATTGTGGGCACGGTTTTGCTCATTACATCTC
TGGGAAGACACACCAAGAAGGTCTTCTTCTCCATCTCAGGCTTTCACAGTTCCAGTGTCCAGGCACACATCAAGGCTCTC

TTGGCTTTTATCTCCTTTGCTATCTTCTTCACTTCCTCTTTTCTGTCACTGGTTCTCACTGCCTCAGGTATGTTTCCTTT
TGGGGAATTCCGGTTCTGGATATGGCAGACTGTGATTTATCTGGGTACAGCAATCCACCCCATTATTCTTCTCTTGAGTA
ACCACAGGCTGAGAGCTGTGCTAGGGAGGGGCTGCTCCTCAGCACATGGGGCATCTTGA
>BubuTAS2R67_NW_005785690.1:1198370-1197432
ATGCCATCTGGAATTGAAAATACTTTTCTAGCAGCAACAATAGGAGGATTCCTGATTGGAATGTTGGGGAATGGGTTCAT
TGTACTAGTTAACTGCATTGACTTGGTGAAGAGACAAAAGCTCTCATCAGCTGACTGCATCCTCACAGGCCTGGCTCTCT
CCAGAATCAGTCAACTTTGGGTAATACTATGCGATTCATTTTTATTGGTACTATGGCCACACTTATATGCCATTGATAAA
CTAACAAAAGTTGTTAGTATTTTTTGGATATTGTCCAATCACCTAGCTACCTGGTTTGCCACCTGTCTAAGTGTTTTCTA
CTTCTTTAAAGTAGCCAACCTCTCCCACCCCTGCTTCACTTGGCTGCGGTGGCGAATTCGTAGTGTGGTACTGGTGCTTC
TCTTGGGGTCTTTGTCCTTACTGTTTTTGAATTCTGAATCAATATATACATTTAGTCATATCTCAACTAACAGCTACAAA
GTATACACAAGAAACTCAACGTGGTCCTCAGATATAAGTGAAACTCATTATCTTCACCAGTTGATTGTTTTTAACTTCAT
CAACTTAATCCCCTTTCTTCTGTCCCTGACCTCACTGCTCCTCTTAGTTCTCTCCTTGATGAGACACATCAAGAATTTGC
AGCTCAACTCCAGCTCAAAGGATCTCAGCACAGAGGCCCATAAAAGAGCCATGAAAATGGTGATGTCTTTCCTCTTCCTC
TTCGTCATTCATGTTTCTTCCATCCTATTAACAGCTTGGGTTTTCCTTAAACTGCAGGGATGTCTGGCCAAATTGGTGGT
TGTATTAACTTCGACTGTTTTTCCTTCAAGCCACTCCTTTATCCTAATTTTGGGAAATAGCAAGCTGAGACAGAATGCCT
TAGGACTACTGTGGTATCCTAACTGCCACCCAAAAAGAGTGAAATCTTTAGCTTCATAG
>BubuTAS2R372A_NW_005785690.1:1058063-1057137
ATGTCAAATGTCATCAAATATGTTTTTTTGATCATTGAAATCTCAGAATTCATAACAGGAATTTGCGGAAATGGATTCAT
TGCACTAGTACTTTGTGCTGACTCTCTCAAAAGCAAGACTATCTCCTTGCTTGACTTCATCTTCACATGCTTGGCCATCT
CCAGAATTGGTATGGTATTCATACTTCTCCTGGATGGCATTAGAATAGTGTTCCATCCAGAAATACTAGATAGTCACCAG
GTAATAGAAGTAACTTTTGATTTCTTCTGGAATCTGAGCAATTCCTTAGCTACCTGGTGTGCTGCCTGTCTCAGCATCTT
CTACTTCCTCAAGCTATCTAGTTTTTCCCACCCCTTCTTTCTCTGGCTAAAATGGAGAAGAAATAGGGTTGTTTTCACCA
TTATGTTGGGATTCTGTCTCTCTTTGTTTTTTAATCTTCTGAACATAAAATTCAATACTCTCAGGGTCAGTGACCATTTA
GAAATAGAAAACAACTTGACTTGGGAAAAAATGCATGCCTAAAACACAGTACTATAGCAGTCAAATTCTCCTCCACCTGGG
ATCTCTCATCCCCTTGGCTGTGTCACTCATTTTATTTTTCCTGTTAATATTTTCCTTATGGAGACATACCAGGCAGATGA
CACGTCATGCCAAAGGATCCAAAGACCTCAACACAGGAGTTCTTGTGAGAACAAGAAATACTTTGACTTCTTTCATCATT
CTCTTAGTTGTGCACTATTTGGCTACATTCATGTTAACTTGGTTCTATTTCACACTAGAAAATGACGTGACTTTTATTGC
TGCTCAAACTGTAGCATTTCTCTATCCTGCAATTCACCCTTTTATTTTGATTCTGGGGAGCAGGAAACTGAGACAGATTT
CTGTAAATCTGCTAAGGCAAATTGAATCCTGTGTCAAGCGATTGTAA
>BubuTAS2R2P_NW_005785493.1:125494-124587
ATGATCTCTTTGTCAGGTATTCCTCATGTTATCATCATGTCAGCAGAATTTATCACAGGGGTTACAGTAAATGGATTTCT
TATAATCATCAACTGTAATGAATTGGTCAAAAGCAGAAAGCTAACACCAATGCAACTCCTGTTCGTATGTATAGGGATAT
CTAGATTTGGTCTACAGACGGTGTTAATGGTACAAGGTTTTTTCTCAGTGTTCTTTCCACTCTTTTATAGCACAAAAATT
TATGGTACACCAATGCTGTTTTTTTGGATGTTTTTCAGCTCTGTCAGTCTCTGGTTTGCCACCTGTCTCTCTCTATTTTA
CTGCCTCAAGGTTACAGGCTTTACCCAGTCCTGTTTTCTTTGGCTGAAAGTCAGGATCTCAAAGTTAATGCCTTGGATGC
TTCTGGGAAGCCTGCTGACCTCTGTGAGCATTGCAGCTCTGTGTGTCGAGGTGGATTACCCTAAAATTGTGGATATTGAT
GTCCTCAGGAATGCCACAGCTAAGAGGACTAAACTCAACACAAAGCAAATTAATGAAGTTCTTCTCGTCAACTTGGCATT
AATATTTCCTCTGACCATATTTATAATATGCACTGTTATATTATTCATTTCTCTCTACAAGCACACTCATCGGATGCAAA
ATGGACCTCTTGGTTTTAGAAACACCAGGACTGAAGCCCATATTAATGCATTAAGAACAGTGATAACATTCTTTTGCTTC
TTTATTTCTTACAGGGTGCCTTCATGGCAAATATGACATTCAGTATTCCTTATGGAAGTCATTGCTTCTTTGTGGTGAAG
GATATTATGGCAGCATATCCCTCTGGTCACTCGGTTATAATGATCTGGAGTAATTCTAAGTTCCAGCAACCAATCAGGAG
ACTTCTCTGCCTAAGAAGGAGTCAATGA
>BubuTAS2R3P_NW_005784086.1:58470-57515
ATGTTGAGACTCAGCCATATGGGGTTTCTGGTTCTGACCGCCATTCAGTTCATCCTGGGAATGCTGGGGAATGGTTTCAT

AGGGTGGGTCAATGGCAGCAGCTGGTTCAAGAGCAAGAGGATCTCTTTGCATGACTTCGTTATCACTAACCTGGCTGTCT
CCAGGATTGTTTTGCTGTGGATTCTCTTGATCGATGGTGTTTTACTGGTGTTCTCTCCCAAACTACATGATGAAGGGATA
ATCATGCAAATTATTGATGTTTTCTGGACATGTACAAACCATCTGAGCATTTGGCTTACCACCTGTCTCAATGTCTTCTA
CTGCCTGAAAGTGGCCAGTTTCTCCCATCCTATGTTCCTCTGGCTCAAATGGAGAGTTTCCAGGGTGGTTGTGTGGATGC
TGTTGAGTACTTTGCTGTTATCATGTTGCAGTGCCATCTCTCTGATCTGGGAATTTAAGATCTATTCTGTTCTTGGTGGA
ACTGATAGAACCGGGAATATGACTGAGCTCTTTAGAAAGAAAGAAAAAGAATATAAACTGATCCATGTTCTTGGGACTCT
GTGGGACCTCCCTCCCCTAGTCATATCGTTAATCTCCTACTTTCTGCTTATCCTCTCCCTGGGGAGGCATATGCGGCAGA
TGCATCAAGACTGTGCCAGTTCCAGAGATCTCAGTACCGAGGCCCACAGGAGGGCCATCTGAGTCATCCTCTCCTTCCTC
TTTCTCTTCCTACTCTACTATCTTTCCTTTTCTGTTTTAACATCCAGTTATTTCTTACCAGCAACTAAAATGATTGCAAA
GATTGGAGAAGTAATTGCAATGTTATATCTTGCTGGCCACTCCTATGTTTAACACTCATTCTGGGAAATAGCAAGCTGAA
GCAGATGTTTGTGGCGATGCTCCGGTGTGAGCCTGGTTGTCTGAAGTCTGGATCCAAGGGATCTGTTTATCCATAG
>BubuTAS2R5P_NW_005784086.1:33600-32721
ATGCCCTCTTCTATCCTAGGACTGCTGATGCTGGAGGCAGTAGCTGAATCTCTCATTGGCCTCATTGGAAATGGAGTTCT
TGTGGTCTGGAGTTTCGGAGAATGTCTCCGAACGTTCAGGGCATCCTTGTATAACCTCATTGTCCTGGGCCTGGTGGTCT
GTTGGTTGCTTTTACAATGGTTGATTATGGTGGACTCAAGTCTGTTCCTGCTTTTCCAGAGCAGCCATTGGCTTCATTGG
CTCAGTGTCTTCAGGGTTCTGGTAAGCCAGGCCAGCCTGTGGTTTGCGAGTTTTCTCAGTGTCTTCTACTGTAGGAAGAT
CATGACCGTTGAACACCCTGTCTCTCTATGGCTGAAGCAGAAGGCCTGTTAGCTGAGTTGCTGGTGCTTTCTGGTGTACT
TCATGATCCATTTGTTACTTATAGTCAGGGGTAGCTTAGACTTCTCCAGTCCTTCCCAAGGAAACAGCAGCATCTTATTC
CCCATTTCAAACTGGCACTATATATGTATATTACAGCTCAATACAGAAAGTATGATGCCTTTCACGATGTTTCCTGTTTC
CTCTGGGCTGCTGTGTCTCTTTGTAGAGACACTACAGGAAGATGAAGGTCCATACAGCCGGCAGAAGAGATGCTCAGGCC
AAGGCTCATATCACTGTCCTGAAGTCCTTGGGCTGTTTCCTTGTACTTTACATGGTCTACATCCTGGCCAGCCCCTTCTC
CATCAGCTCCAAGACTTTTCCTGCAGATCTCTTCACTCTCTTCATCTCTGAGACACTCATGGCCGCCTACCCTTTTCTTC
ATTCTGTCATACTGATCATGGGGAACCCCAGGATGAAGCAGGCATGTCAGAGAATCCTGTGGAAGACTGTATGTGCTTGA
>BubuTAS2R9P_NW_005785690.1:1023997-1023079
ATACCAGGTACAATGGAGGCAATATATATGCTCTTGATTACTGGCGAGTGGATGATGGGAATTTGGGGAAATGAATTCAT
TGTACCGGTAAACTGCAGTGGCTGGTTCAAAAAGAGAGCTATCTCCTTGACTGACGTCATCCTGGTCAGCCTGGCCACCT
CCAGAATCTGTTTTTTGTGTGTTATATATATGGATGGTTTTATTATGGTACTCTTTCCAGATACATACAGGCATGGTGAG
ATGATGAACATTTTGGATATTTTCTGGACAACTTGCAATCATTCAACTGTCTGGTTTACTTTGTGTCTCAGTATCTTCTA
TTTACTCAAGATAGCCAGTATATCCCACCCAGTTTTCCTCTGGCTGAAGCTGAAGATGAACAGGGTATCCTTGGGATTCT
TCCGATGTCCTTTCTCATCTCCTCGATTATTAGTGCTTTACTGAATAATGATTCATTTTATGACTTCAGAATCAATAATG
AAGCAAACATTACTTAGGAATTCAAAGTAAGTAAAATCCCAACTGCTTTCAAATAGATTATCCTGAACCTGGAGGCTATG
GTTCCCTTTATTCTTTGCCTGGTCTCATTTGTCCTTTTATTTTTCTCCTTACTTCGACACACCAAGCAGATGAAACTTCA
TGACACAGGGTCTAGAGACCCTAGCATAGAGGCCCACATGAGGGCCATAAAGACAATAGTCATCTTTCTGGCTCTTTTCA
TTATGTACTATGTAGTTTTTCTCATTGTAACATCTCGCTTTCTGAATCCTCATGGAAAATTGGAGTTGATGTTTGGTGGC
CTAACAGCTGTCATTTTCCCAATGAGCCATTCGTTCATCCTGCTAATGGGAAGCAGCAAGCTGAGGGAGGCTTTTCTGAA
GGTGCGGGGGATTGTGAAGGGTTTCCACAAAAGAAAGAA
>BubuTAS2R10CP_NW_005785690.1:1035570-1034641
ATGCTGAGTGTACTGGAAGGCCTCCTCATTTTTGTAGCAGTTAGTGAGTCAATATTGGGGGTTTTAGGGGATGGATTTAT
TGGACTTGCATACTTCATTGAATGTGTGAAGAACAAGAAGTTTTCTACTATCAGCTTTATTCTCATGGGCTTAGCTACTT
CCAGAATTTGCCTGATAGGGTTAATAACTACCTATGGATTTGTGAAGATTTTTTCTCCAGAAATGTATTCCTCTGGTTAC
CTAATTGACTGTATTACTTACTCATGGGCAATTCTGAATCCAACAAGTGTCTTTTTTGCCACCAGCCTCAGCATCTTCTA
TTTCCTGAAGATAGCCAATTTTTCCCACCACATTTTTCTCTGGTTGAGGAGTGACATCAAAAGGGTTCTTCTCCTTCTGA
TGGGATACTTGCTTATTTCATGGTTAGTTACTTTTCCACTAACTATGAAGATAATTAGTGATTCTAGAGCAAAGAACAGA
AGCGTAGTCTTTTCAGTTGAAGTGTAGAAAGGTGAATTCTTTAGAAACCAGATTTTGCTCAATCTTGGAACCCTTACCAT

CTTCATACTATGCCTGATTACATGTATCTTATTGCTCATTTCCCTTCGGAGGCACAACCAGAGGATGCTACTGAATGCCA
CAGGATTCAGAGACCCAAACACAGAAGCACATATCAAAGCAATGAAAGTTTTGATATCTTTTATCATCCTTTTTATATTG
TATTTTATAGGCATTATCATAGAAATATCATGCACTACTATGTCAGAAAGCAAGCTGTTGTTTATTTTTGGTCTGACCAT
CACCACCCTGTATCCCTGGGGACACTCATTTATCCTAATTCTAGGAAACAACAAGCTAAAGCAAGTTTTTTTGAGAGTAC
TGAAGCAATTAAAATGCTGGAAGAAAGAGAAGCTCCTCAGAACTCCTTGA
>BubuTAS2R10DP_NW_005785690.1:1063847-1062799
ATGCTAAGTATAACAGAACGTCTCCTCATTTTTGTAGCAGTTAAGTGAGTCAATATTGGGACTTTTAGGGAATGGATTTA
TTGGATTTGTAAGCTGCATTGATGGTATGAAAAACAAGAAGATCTCTACTATCAGCTTTATTCTCGCTGGCTTAGCAACT
TCCAGATTTTGCCTGATATGGACAATAGTTATTGATGGATTTTTTAAGTTATTCTCTCCAGATGTGCATTCCTCTGGGAA
CCTAATTGAATATAATGGTTACTTGTGGATAGTGATGAATCAATCAAGTATCTGGTTTGCCACCTGCCTCAGCATCTTCT
ATTTCCTGAAGATATCTACTTTTTCCCACCGCATCTTTCTCTGGTTGAAGGGTAGACTCAACATGGTTCTTTTCCTTCTT
TTGGGATGCTTGCTTATTTCATGCTTAGTTACTTTCCCACATTTTGTGGAGATTGTTAATGATAATAAAAGGAAAATAAA
AACACAGTCTGCTCAATGGATATGCATAAAGAAGAACTCTCTGGAAAACAAATTTGGCTCCATCTTGGTGTCATTCTCTT
TTTTATACTATGCCTGATTACATGTGTCTTGTTGCTCACTTCTCTTTGGAGACACAACTGCTGCTGCTAAGTCACTTCAG
TTGTGTCCGACTCTGTGCGACCCCATAGACGGCAGCCCAACAGGCTCCCCTGTCCCTGGGATTCTCCAGGCAAGAACACT
GGAGTGGGTGGAGACACAACAGGAGGATGCAATTGAATGCCACAGGATTCAGAGACCCCAGTACAGAAGCACATATCAAA
GCAATGAAAGTCTTGTCTTTTATCATCCTCTTTATCTTGAATTTTGTAGGTACTGCCATACAAATATCAAGTGTGACAGT
GCCTGAAAACAAACTGCTTTTTATTTTTGGTATGATAACCATAGTCCTCTATCTCTTTGGTCACTTGCTTATCCTAATTC
TAGGAAATAGGAAGCTCAAGTAAGCCTCTTTGAGGGTACTGAAGTCATTAAAGTGCTGGGAAAAAGAGAAACTTCTCAGA
ACTCCTTGA
>BubuTAS2R11P_NW_005785690.1:1093605-1092711
ATGTTGAATATATTGGAGAAGATTTTCATGGTTGTGACTGGTGGGGAATTTATAATAGGAATTTTAGGGAATGGATTTAT
TGAACTCACAACTTGCATTGCCTGGATTAGAAATCAGAAGTTGAGCTTGGTTGACTTCATTCTTACTAGTTTGGCCTTTT
CCAGAATCAGTCAATTATGGATAACCACTGTCATGTTCTTTTCAATGATGTTCTATCAGGCAGGCTTTGGTACTGTGGGA
AGAAAATATATCTTCTTTTGTATCTGGATACTGGCCAGTCACTCAAGCACTTGGCTTGCTACTTGCCTTGCTGTCTTTTA
TTTCCTGAAGATTGCCAGTTTCTCCCATCCTCCTTTCCTTTGGCTAAAATTGAGAATTAACAAGGTTGTTTTCATGCTTC
CACTGGTATCTGTGCCCTTCCTAGTTATAAGTTTTCCTTAGCCATATAATGTTGATGTCTTCTGGTGTCATGTCCAAAAG
ATGCATGAGAGAAATATGACTGAGTTATGCAATGTGAATGAATATCAAAATTTAAATTTTATTATTATCTTCACTATGGA
GTCCCTCCCCCCTTCTTCCTTTCCCTGATTTCCTTTATCCTGTTGCTCCATTCTTTGTGGAAACACAAGAAGAACATTGC
ACACACTGTCAGGGATTCCAGAGACCCTCGTGTGGAGGCCCATTTCAGAGCCATGAAAACTGTGTTTTTCTTTCTCATGC
TCTTTGTCCTGTACCAATTTGGCCTTTTCATGACATTTGGGGGGCATTTTTTCCTACAGAACAAGCTGGTTGTGATGTTT
GGTTATATGTTAGGAATGCTGTATCCTTCAAGTCACTCATATGTTTTAATTTTTGGAAACAGTCAAATGAGGAAATTCTT
ATTGGTGATTCTTAG
>BubuTAS2R13P_NW_005785690.1:1120831-1119930
ATGGAGGATTCCTTGGAAAACATCTTTATCATTTTAATAAATTCAGAATTCATAATTGGCATTCTGGGGAATGGATTCAT
AGCACTGGTGAACTGCATTGACTGGATCAAGGTGCAAAAGGTCTCCTTGGCTGATCAAATCCTCACTGCTTTGGCAATTT
CCAGAATTGGTCTGATTTTGGTAATGATGGTGAGTTGGTTTACAAAGGAGTCTTATCCATCTTCATCTTTAGACATAAAG
GGAAATAAAGTCATACTTTTTAGTATTGTTGGGCTCTTGGCCAACCATTTTAGTGTCTGGCTTGACACAGGCCTCAGCCT
CTTTTATTTCCTCAAGATAGCCAATTTTTCAAATGCTGTTTTTCTTCACCTATTGGAATGGTAGTTATGGTAATGTTTCT
GGGGACATTAGTATTGCTGCCTTTAAGTCTTACTCTGGTGAGCAGCTATATTAATATCAAGATACATTCATATGAAAGAA
ATATGACTTTAAATTCTAAAAGGAGTGACACTGAAACCTTTTCCAAATTGATTATATTCACCGTGGAATTTTGCTTACCC
TTTATTATATCCCTGAGTTGTTTTCTCCTGTTAATGTTCTCCCTACTGAAACATGTCAAGAAGATGAGGAGCCATGCAAC
AGGGTTCAGAGATCCCAGCAGCAAAGCCTACGTCAAAGCCATGATCATGGTGATATCTTTTCTCATACTACTTGCCATTC
ACTTCCTATCTCATCTCATGACAACTTTTCATCACAATGTGATACAGAGTGAACTGGCCTTTATGCTTGCTGAAGCTCTT

GGAACTATTTACCCTTCATTCCACTCATTTGTCCTGATTCTGGGAAATGACAAGCTAAGAAAAGCTTCACTTTTGGTGCT
GTGGCAGTTGAGGTGTGGCTGA
>BubuTAS2R408BP_NW_005785690.1:1160184-1159275
ATGATAACTTTAGTATCGAGCATTATTTCCATTCTAATGGTGACAGAATTTGTTCTGGGAAATTTTGTGAATGGTTTCAT
AGCACTGGTGAACTGCAATGACTGAACAAAAACTCTCCTCAGCTGATGGGATTCTCACTGCTCTGGCAGTCTGCAGAATT
GTTTTGCTTTGGACAATATTAATAAATTGGTATGCAACTAGGTATAATCCAGCTCTATATAGTTTAAGAATTGTTATCTG
TGTTGCCTGGACAGTAAGCAACCATTTTAGTAACTGGCTTGCTACTAGCCTCAGTATATTTTATTTGTTCAAAATAGCTA
ATTTCTCCAGCCTAATTTTTCTTCACCTGAAGTGGAGAGTTAAAAGTGTAGTTCTCATGATGATGTTGGGGACTTCAGCG
ATTTGTTTTTTCAAGTTGCAGTGTTAAGTATAGATGAGACTATTCAGACAAGTGAATATGAAAGAAACATCACTGAGAA
GACCAAATTAAGGGACATTTTACACCTTTCAAATATGACCCTGCTCACACTTACAAACTTCATACCCTTCACTATGTCCC
TGATATCTTTTCTGCTGCTAATCTTTTTCCTGTGGAAACATCTCAGGAAGATGCAGCTCAACGGCAAAAGATCCCAAGAT
CCCAGCACCAAAGTCCACATGAAAGCGATGCAAACTGTTATCTCCTTTCTTTTCCTGTTTGCCACTTACACGCTGACTGT
AATTTTAACAATTTGGAATTCTAATGAGCTGCAGAAGGAACTGGTCCAAATGCTTTTCCAGGCTCTTGCAATCACCTATC
CTTCAATACACTCATTTATCCTGATTTGGACAAACAGGAAATTAACACAGACCTTTCTGTCGTTTCTGTGGCAGCCAAGA
TGCTGGCTAAAAGTAAAAGGAACTAGGTAG
>BubuTAS2R408CP_NW_005785690.1:1134412-1133472
AAGATAATGTTTATGTCAAACATTGTTTCCATTCTATTAATGACAGAATTTGTTCTGGGAAATTTTGCCAATGGCCTCAT
AGCACTGGTGAACTGCAATGACTGGACCAAGAGACCAAAGATCTCAGCTGATGGGATTCTCACTGCTCTGGCATTTTGCA
GAATTGTTATGCTCTGGGCAATGTTAATAAATTGGTATGTAATTGTGTATAATCTAACTCTATATAATTCAAAAGTAAAA
ATTATTGTTCATGTTGCCTGGGCAGTAAGCAACCATTTTAGTAACTGGCTTGCTACTAGCCTCAGTATATTTTATTTGTT
GAAGATAGCCAATTTCTCCAGCCTAATTTTTCTTCACCTGAAGTGGAGAGTTAAAAGTGTAGTTCTAATGATGATGTTGG
GGACGTCATTGTTCTTGTTTTTTCAAGTTGCAGTGTTAAGCATGGATGAGGCTATTCAGACATATGAATATGAAGGAAAT
ACCACTCAGAAGACCAAACTAAGGGACACTTTACACCTTTCAAATGTGACTCTGTTCACACTAACAAACTTTATACACTT
CACTATGTCCTTGACATCTTTTCTGCTGCTAATCTTTTCCCTGTGGAAACATCTCAGGCAGATGCAGCTCAATGGTAAAG
AATCCCAATTTCCCAGCACCAAGGTCCACATAAAAGCCATGCAAACTGTCATCTCCTTTCTTTTCCTGTTTGTTATTTAC
ATTCTGGCTCTAATTTTATCAGTTTGGAATTCTAATCAGCTGCAGAAAGAACGAGTCTAAATGCTTTATGATGTTGTTTT
AATCATGTATCCTTCAATTCACTCATGTATCTTGATCTGGGGAAACGGAAATTAACTCAAGCCTTTCTGTCATTTCTGT
GGCAGTCAAGATGCTGGCTGAAGGAAAGGAAATAGGTGGAAACATATGTCTTTTAGCATAA
>BubuTAS2R408DP_NW_005785690.1:1176203-1175289
ATGATAACTTTATTATGGACCATTTTTTCCATCCTAGTATTAACAGAATTTGTTCTAGGAAATTTTGCCCATGGCCTCAC
AGCACTGGTGAACTGCATTGATTGGGTCAAGAGACAAAAGATCTCCTCAGCTGATGGGATTCTCACTGCTCTGGCAGTCT
GCAGAATTGTTTTGCTCTGGGTAATGTTAATGAATTGGTACTTAGTTGTGTTGAATCCAGTTCTATATAGTTTAAAAGTA
AGAATTATTGTTCATATTGCCTGGATAGTAAGCAGCCATTATAGCACCTGGCTTGCTACTAGTCTCAGCATATTTTACTT
GTTGAAGATAGCCATTTCTCCAGCCTAATTTTTATTCACCTGAAGTGGAGTTAAAAGTGTCATGCACATAATACTTCTGG
GAACTTCATTCTCCTTGGTTTTTCATGTTGCAGTGATATACAATGATGAGGCTATCCAGACAAATGAATACAAAGGAAAC
ATTCCTCAGAAGAACATATTGAGGGGCAGTTTATGGCTTCCACACGTGACTCTGCTTATGCTAGGAAACCTCATATGCTT
TACTATGTCCCTGACATGTTTTCTGCTATTAACTGTTTCCCTGTGGAAACATCTCAAGAAGATGCAGCTCAGTGGTAAAG
GATCTCCAGATTCTAGCACCAAAGTCCATATAAAAGCCATGTAAACTCTGATATCCTTTCTCTTGCTGTTTGGCATTCAT
TTCCTGGCTCTAATGGGATCCATTTGGAGTTTTAAAAGGCAGCAGAAGGAAACTGTCTTTTTGTTCTTTGAGGCTCTTGG
ATTCCTCTATCCTTCAAACCACTCATGTATCCTGATTTGGGGAAACAGGAAGTTAACAAAGGCATTTCTGTCATTTCTGT
GGCAGCTAAGGTGCTGACTGAGAGAAAAGAAATAG
>BubuTAS2R408EP_NW_005785690.1:1127188-1126306
ATGATAACTTTACTACATTTTTTCCATCCTAGTAATAGAATTTATTCTAAGAAATTTTGCCAGTGGTTTCATGTCACTGG
TGAACTGCATTGACTGGTCAAGAGACAAAAAATCTCTTCAGCAGATGGGATTCTCACTGCTCTGGCAGTCTCCAGAATTG

GTTTGCTCTGAGTAACACTAATAAATTGGTATGTAAATGTGTTTAGTCCCAGCTTTAGACAATTTAAGAGCAAGAATTAT
TATTATTGCCTGGATAATAAGCAACTATTTTGGCATCTGGCTTGCTGTTATCCTCAGCATATTTTATTTGCTCAAGATAG
CCAATTCTCTGATACTATTTTTCTTTACCTAAAATAGAGAATTAAAAATGTTCTTCTTGTTCACTTTGTCCTGTTTGGTT
TTATTAATTCATGGTGTAAACGTAAATAAGACTATCCAGACAAATGACTATGAAGGAAACATCACTCAGAAGACCAAGCT
GAGGGACATTTTACACCTTTGAAATATGACTCTGTTCATGCTAGTAAACTTCACACCCTTTGCTATGTCCCTGAAGTCTT
TTCTGCTGTTAATCTTTCCAGCATGAAATGGAAACATTTCAGGAAGATGCAGCTCAGTGGTAAAGGACCCCAAGATCCCA
GCGCCAAGGTCCATATAAAAGCCATGCAAACTGTCATCTGTTTTCTATTTCCCATTTGCTTCCTGGTTCTAATTTTCTGT
TTGAAGTTCTAATAGGCAGTGGAACAACTTGGTTATCATGGCATGCCAGGCTTTTGGAATCATATGTCCTTCACTTATCC
TGACATGGGGAGAGCAGATACTAAGAGAGTACTTCCTGTCATTTCTGCAGCAGCTGAGTTGATGGCTAAAAGAAAGGAAA
TAA
>BubuTAS2R62AP_NW_005785465.1:625452-624542
ATGTTGATATTCAAGGCCATCTTTTTCCTGGAGTCATTGGTTGCTGTGCTGCAGAATGGCTTCATAGTTACTGTGTTGAG
TGGGGAGTGGGTGCGAAACCGGATGCTGCCCGCCGGTGACACGATTGTGGCCTGCCTGGCTGCCTCCTGGTTCTGTCTGC
ACGGGATGGCCCTCCTGAACAACATCATGGCCTCTTTTGGCTTTTGTTCCAAAATCAACTATTTCAGCATCCCCTGGGAT
TTCATCAACTGCCTCAGTTTCTGGCTGACTGCCTGGTTTGCTGTCTTCTACTGCGCGAAGATCTCCCTCTTCTCTTATCC
CGTCTTCTTCTGGATAAAGTGGAGGATTTCTCGGTCGGTTCCCCAGCTGGTGCTGGGCTCCTTGATCTTATCTGGTCTGT
CCGTCATCTCAGCTGCTGGGAATACAATTCTTGCCCAGATGACAGCTGCCCATATTTCCCATGGAAACGACACTCTGGCT
GATAGAATACATGCTACCTATTTGCACTTTTTCTACCTCATATAATTATCATGTGGTTGGTTCCATTCCTCCTGTTCCTG
GTGTCCACCCTCTTGCTCATGTTCTCACTGCTCCGGCACCTCTGGCAGATGCAGGACCACAGACCCAGCCCACGTGATCT
CAGTACCTGGGCTCACACCATGGCCCTGAAGTCACTTGCCTTCTTCCTCATCTTCTACACCTTGTACTTCCTGTCCCTGG
TTATCATTATGTACATCCCAGCCCTCTGGGAACACTGGCACTGGGCCTATAAGGTGGTGACCTACACTGGCATCTGTCTG
CACTCCAGCATCTTGGTGCACAGCAGCCCCAAGCTGAGAAAGGCCCTGAAGAAGAGGCTTTGGCGAGCCCTGGACAAGGA
CCAGTTTGTCTCCAGTTATCAGTATCAATAG
>BubuTAS2R62BP_NW_005487871.1:471-1381
ATGTTGATATTCAAGGCCATCTTTTTCCTGGAGTCATTGGTTGCTGTGCTGCAGAATGGCTTCATAGTTACTGTGTTGAG
TGGGGAGTGGGTGCGAAACCGGATGCTGCCCGCCGGTGACACGATTGTGGCCTGCCTGGCTGCCTCCTGGTTCTGTCTGC
ACGGGATGGCCCTCCTGAACAACATCATGGCCTCTTTTGGCTTTTGTTCCAAAATCAACTATTTCAGCATCCCCTGGGAT
TTCATCAACTGCCTCAGTTTCTGGCTGACTGCCTGGTTTGCTGTCTTCTACTGCGCGAAGATCTCCCTCTTCTCTTATCC
CGTCTTCTTCTGGATAAAGTGGAGGATTTCTCGGTCGGTTCCCCAGCTGGTGCTGGGCTCCTTGATCTTATCTGGTCTGT
CCGTCATCTCAGCTGCTGGGAATACAATTCTTGCCCAGATGACAGCTGCCCATATTTCCCATGGAAACGACACTCTGGCT
GATAGAATACATGCTACCTATTTGCACTTTTTCTACCTCATATAATTATCATGTGGTTGGTTCCATTCCTCCTGTTCCTG
GTGTCCACCCTCTTGCTCATGTTCTCACTGCTCCGGCACCTCTGGCAGATGCAGGACCACAGACCCAGCCCACGTGATCT
CAGTACCTGGGCTCACACCATGGCCCTGAAGTCACTTGCCTTCTTCCTCATCTTCTACACCTTGTACTTCCTGTCCCTGG
TTATCATTATGTACATCCCAGCCCTCTGGGAACACTGGCACTGGGCCTATAAGGTGGTGACCTACACTGGCATCTGTCTG
CACTCCAGCATCTTGGTGCACAGCAGCCCCAAGCTGAGAAAGGCCCTGAAGAAGAGGCTTTGGCGAGCCCTGGACAAGGA
CCAGTTTGTCTCCAGTTATCAGTATCAATAG
>BubuTAS2R18BP_NW_005785690.1:1155317-1154375
ATGTCAGTTGGAATGAAGGGCTCTTTCTACTAGTGGCAACAGGAGAACTCATCTTAGGAGTGCTGAGAAATGGGTCAAGA
ACAGGAAAGTCTCATCAGCTGGTTTCATCCTTACCTGCTTAGCTGTGGACAGAATCATTCAAATGTGGGTAACACTATTG
GGTTCATTTACAGCGGGGCTATTTTCACATCTGTATGCTACCAGCAAACTAGCAAAGGTGATTACTCTTTTTTGGGCACT
AATGAATCATTTAACTACCTGGTTTGCTAACCCCTAAGTGTGTTCCATTTCTTTAAGATAGCTAATTTCTCTAATTTCTT
CTTCACATGGCTGGAGTGGAGAAGGAACAGAGTGATTCTTATACTTTTCCTGGGCTCTTTGCTCTAACTGTTTATTAACC
TCTTAATGCTCTTGGTGAATTGTGAATGAATAGCTATAGAGAGCCTGAAAGAAACACAGCTTTGCATTTAGATGCAAATA
AAATTTTCTATCTTAGACGCCTTATTCTTCTTAGCTTGACCTATGTTATCCATTTTCATCTCTCTCTGGCCTCTTTGTGC

TTTCATTTTTCTCCTTGGTGAGACACACCAAGAATTTCCAACTCAACCTGAATGGCTCAGAAGACACCAGCACAGAGGCC
CATAAAAAGACCATGAAAAGGGTGACAACCTTCCTCTTCCTGTTCATCATTTACTTTTTCCACTCTATTAGGGAGCTGGA
TCTTTCTTAAGGTACAGCAGTATCAAGCCATGATGTTTGTCATGAAGACTTCAACTGTCTTCATCTTGGGTCCTCTTATG
TTTTAATTTTGGGAATTAGCAAGCTAAGAAAGATCACCTTGAGTTTAATTTTGAATCTTATATTCTCTTTGAGAAAATCA
AGATCATTAGTTTCATGGACAGAATTTAAATGTACTTTATGTATTCTAGAGAAAATGCCTTAA
>BubuTAS2R372BP_NW_005785690.1:1072743-1072079
TCAGCAGTGACCAAAGTAACCAGGATTTCACTTCTACAGTCATGTCCAGTGTAATCAAAAGATTTTTATGATCATTGAAA
TGTTATAATTCATAACTGGAATAACTGTGCTGACTTTGTCAAAAGCAAGAATAGTGCCTTGTTTGACTTCATCTTCACAT
GGATTAGCATAATGTTCATACTTCTCCTAGATTGCGTTAAACTAGTGTTCCATCTAGAAATAGTAGATGGTCACCAGATA
ATAAGAGGAGTTTTTGCGTTCTCCTGGAGTCTGAGAAACTCCTTAAGTACTGGATGTGCTGTCTGCCTCAGTGTCTTCTA
CTTCCTCAAGCTATCTAGTTTTTCCTCACCCCTTCTTTCTCTGGCTGAAGTGCAGAAGAGATAGAGTTGTTTTCACCATT
ATGTTGGGATTCTGTCTCTCTTTGATTTTTAACCTCCTGAGCATAAAATTTATACTTTTGTGTTCAGCAAGCATTTAGAA
AAGGAAAGACTTAACTTGGAAAAAAGATGTGCATAAAAATCAGTATTATAACAGTCAAGTTCTCTTCAGCCTTGGATCTC
TCATCCCCTTGTCTGTATCACTCATTATATTTTTCCTATTAATCTTTTCCTATGGGGACATACCAAGCAGATGACAAGCC
ATAACGCAGATCCCAGGGACTTCAA
>CesiTAS2R1_NW_004454243.1:8209727-8208828
ATGCCAGAGTCTTACCTCATTATCCATCTTCTTTTTGCAGTGATACAATTCCTCACCGGAGTTTTAGCAAATGGCATCAT
TGTGGTTGTGAATGGCACTGACTTGATCAAGCAGAGAAAGATGATTCCATCAGATCTCCTTCTTTCCTGCCTGGCAATTT
CTAGGATTTGTCTGCACTTGGCCTTCTTCTGCATTAATCTGGCTGGTCTCTCCTGGATTGAATTCACTCCATTTTCTGAG
ACTTTTGCAATTCTCGTGTTTGTAAATGAATCGGGACTTTGGTTTGCCGCATGGCTCAGCGTTTTCTACTGTGGCAAGAT
TGCCCCCATCGCTCACCCACTGTTCTTCTGGTTGAAGATGAGGATAGCCAAGTTGGTGCCATGGCTGATCTTCGGATCCC
TGCTATATACATCTATCATTTCTGTTTTCCATAGCAAACATACATGGACTCTTTCCCAAAAAGTCTGGTTGGATCTTTCC
TCCAATAATGCAACAACTCAAATCAAAGAACTATCTGTTTTACAGTGTGCCTTTATTGGCATTGAGCTTTCACTGCCATT
ACTTATTTTCCTTATTTCTGCTCTGCTCTTGATATTTTTCCTGGGGAGACACACTCGGCAGATGAGAAACACAGCGATGG
GCACCAGGAACCCTAGCACGAGTATCCACACCCGTGCATTTCTGTCCATCCTGTCCTTCCTGGTCCTCTACCTCTCCTAC
TATGCGATGATTGCTTTGCTCCTTTCGCAAATTTTCAAGCTCAGAAACTTCATCTTTCGGTTCTGCATCTTGATGATTGG
TTCATACTCCTCTGGACACTCTATTATCTTAGTTTTAGGAAATTGTAAACTGAAACAAAATGCAAAGAAGTTCCTCCTCC
ACAGTAAGTGCTGTCAGTGA
>CesiTAS2R2_NW_004454191.1:19658418-19659326
ATGGCCTCCTCTTTATCAGCTATTCTTCACGTTCTCATCATGTCGGCAGAATTTATCACAGGGATTACAGTAAATGGATT
TCTTATAATCATCAACTGTCATGAATTGATCAAAAGCAGAAAGCTAACACCAATGCAACTCCTTTTTGTATGTATAGGGA
CGTCTAGATTTGGTCTGCAGATAGTGTTAATGCTACAAAGTTTTTTCCCTATATTCTTTCCACTTTTTTATGCTTTAAAA
ATTCATGGTCCAGCGATGATGTTCCTTTGGATGTTTTTTAGCTCTGTCAGTCTCTGGTTTGCCACCTGCCTTTCTGTATT
TTACTGCCTCAAGATATCAGGCTTCACTCAGTCTTATTTTCTTTGGCTGAAATTCAGAATCTCAAAGTTAATGCCTTGGC
TGCTTCTGGGAAGCCTGCTGGCATCCGTGAGCATTGCAGCTCTGTGTATCAAGGTAGATTACCCTAAAATTGTGAATGAT
GTCCTCAGGAACGCCACGCTAAAGAGAACTGAACTCAAGATAAAGCAAATTAGTGAAGTGCTTCTTGTCAACTTGGCATT
AACATTTCCTCTAGCCATATTTGTGATGTGTACTTTTATGTTATTCATTTCTCTCTACAAGCACACTCTTCAGATACAAA
ACGGACTTCATGGTTGTAGAAATGCTAGCAGAGAAGCCCATATAAATGCATTAAGAACAGTGATAACATTCTTTTGCTTC
TTTATTTCTTATTTTGCTGCCTTCATGACAAATATAACGTTCAGTGCTCCTTACAGAAGTCAGAGCTTCTTGGTGGTGAA
GTCTATAATGGCAGCATATCCCTCTGGCCATTCAGTTATAATAATCTTGAGTAATTCGAAGTTCCAACAACCATTCAGGA
GACTTCTCTGCCTCAAAAAGAATCAATGA
>CesiTAS2R3_NW_004454187.1:14623818-14622868
ATGTTGGGACTCGCCGAGTGGGTGTTTCTGGTTCTCTCTACCACTCAGTTTATTCTAGGAATGCTGGGGAATGGTTTCAT
AGAGTTGGTCAATGGAAGCAGCTGGTTCAAGAACAAGAGAATCTCTTTGTCTGACTTCATCATCACTAACCTGGCTCTCT

CCAGGATCGTTGTGCTGTGGGTTCTCTTGGTTGATGGTGTTTTAATGGTGTTCTATTCCAAAGTACGTGATGAAGGGATA
GTAATGCAAATTATTGATATTTTCTGGACATTTACAAATCATCTGAGTATTTGGCTTGCCACCTGTCTCAGTGTCCTCTA
CTGCCTGAAAATTGCCAGTTTCTCCCACCCTACATTCCTCTGGCTCAAGTGGAGAGTTTCCAGGGTGGTTGTATGGATGT
TATTGGGTGCACTGCTCTTATCATGTGGCAGTGCCATATCTCTGATCCGTGAATTTAAGATCTATTCTGTTTTCTGTGGA
ATTATTGGCACAGGGAATGTGACTGAGCACATTAGAAAGAAAAGAAGTGAATATGGACTGATCCATGTTCTTGGGACTCT
GTGGAACCTCCCTCCCCTAATTGTGTCTCTGGCCTCCTACGTTCTGCTCATCCTGTCCCTGCGGAGGCACGTGCGGCAGA
TGCGGCAAAATGGTACCAGCTCCAGAGATCCAAGCACTGAGGCCCACAAGAGGGCCATCAAAATCATTCTCTCCTTCCTC
TTTTTCTTCCTATTTTACTTTCTTGCCTTTGTAATTACAACGTCCGGTCATATCCTACCAGGAACTAAGATGGTTAAGAT
GACTGGAGAAATAATTACAATGTTTTATCCTGCTGGCCACTCCTTTATTCTCATTCTGGGAAACAATAAGCTGAAGCAGA
TGTTTGTGGAGATGCTCTGGTGTAAGCCTGGTCATCTGAAGTCTGGATCCAAGGGATCTTTTTCCCCATAG
>CesiTAS2R4_NW_004454187.1:14612275-14611373
ATGCTTCAGATATTCTTTATCTCTTCTGTTACTGTCTCAGTAATTTTGACTTTTGTAGGACTCATTGTGAATCTGTTTAT
TGCAGTAGTCAATTACAAGACTTGGGTCAATAGCCACAGAATCTCTTCTTCCGATAGGATCCTGTTCAGCTTGGGCATCA
CCAGATTTCTTATGCTGGGAATGTTGCTACTGCATATTATTTACGTCAGCTCTCTAAATGTTGAAAGGTCAGTCTACTTA
CCCACTTTTTTCCAGTCGTGTTGGATGTTTTTGGACTCTAATAGTCTCTGGTGTGTAACCTTGCTCAACGCCTTGTACTG
TGTGAAGATTACTAACTTCCAACACTCAGTGTTTCTCCTGCTGAAACGAAATCTCTCCCCAAAGATCCCCAGGCTGCTGC
TGGCCTGTGTGTTGATTTCTGCCTTCACCACTCTCCTGTACCTTGTGCTCCAACAGACATCACCCATCGCTGAATTTGTG
ACTGGGAGAAATGGCACAGTATTTAACATCAATGAGGGTGCCTTGTTTTTGGTGACCTCTTTGACCTTGAGCTCGTTTCT
CCAGTTCAGCATTAATGTGACTTCTGCTTCCTTGTTAATAAATTCCTTGAGGAGACATATACAGAAGATGCAGAGAAATG
CCACTGGTTTTTGGAATCCCCAGACTGAAGCTCTTGTGGGTGCGATGAAGCTGATGATCTGTTTCCTCATCCTCTACATT
CCATATTCAGTTGCTGCCCTGCTCCTTTATCTCCCTTCTCCTGTAGGGATGAATTTAGAAGCCAGGTGCATGTGTATGAT
TATTTCTACATTTTACCCTCCAGGACACTCTGTTCTCATTATTCTCACTCATCCTAAACTGAAAACAAAAGCAAAGAAGA
TTCTTTGTTGCAAGAAATGGTAG
>CesiTAS2R7_NW_004454209.1:602037-601090
ATGCCGGAGAGCATGTTAAATGTGGAGAGCACTTTAATGATGATAGCAACTGGAGAGTTCTCAATGGGGGTCTTAGGAAA
TGCATTCATTGGACTGGTAAACTGCATGGACTGGATCAAGAATAGGAAGATTTCCTCCATTGATTTAATCCTCACAAGTC
TGGCAATATCCAGAATTTGTCTATTGTGTATAATACTATTAGACTGTTTTATATTGGTGCTGTATCCGGATGTCTATACC
ACTGGTAAACAAATGAGAATCATTGACTTCTTCTGGACGCTAACCAACCATTTAAGTGTGTGGTTTGCCACCTGTCTCAG
CATTTTCTATTTCCTCAAGATAGCTAATTTCTTCCATCCCCTTTTCCTCTGGATGAAGTGGAGAATTGACAGTGTGGTTC
CTAGGATCCTGCTGGGGTGCTTGGCCTTCTCTGTGTTTATTAGCCTTCCTGTCACCGAGAATCTGGACGATGATTTCAGG
CGTTGTGTCAAGGCAAAGTGGAAAACAAACTTAACTTTGAGACGCAGAGATAATAAAGCTCAATATGCTTTCATCAAGCT
ATTTCTCAACCTGTTAACACTATTCCCCCTTTCTGTGTCCCTGATCTCATTTCTCCTCTTGATTCTTTCCTTGTGGAGAC
ACACCAGGAAGATGCAACTCAATGCCACAGGGTGCAGAGACCCCAGCGTGGAAGCCCACATGGGAGCCATGAAAGCTGTC
ATCTCCTTCCTCCTCCTTTTCATTGCCTACTATTTGGCCTTTCTCGTGGCCACCTCGAGCTACTTCATGCCAGAGACTGA
ATTAGCTGTGGTGATTGGTGAGGTGATAGCTTTAATCTATCCCTCAAGCCATTCATTTATCCTAATTCTGGGGAACAACA
AATTAAGACAAGCATCGTTAAAGGTGCTATGGAAGGTAAAGTGTGTCCTAACAAGAAGAAATTTCTAA
>CesiTAS2R8_NW_004454209.1:607940-607020
ATGCTCAGTACAGAAGACAGCATCTTCCTGGTCATAATTATCAGCAAATTCATATTAGGAATGTTGGGGAATGGATACAT
TGGACTAGTAAACTGGATTGACTGGATTAAGAAGAAAAAGACCTCCTCAATTGACTACATCTTCACCAGTTTAGCTATCT
CCAGAATTTGTTTGATTTGTGTAATGGTACTGAATGCCATCATAATAGTATTCTACCCAGATGTTCATGAAAATGGTAAA
CTAAAGATAGTCAGTATCTTCTGGACACTCACCAACTACCTAAGTATGTGGATTGCCACCTGCCTCAATGTCTTCTATTT
CCTCAAGATAGCTAATTTCTCCCACCCGCTTTTTCTCTGGCTGAAGTGGAGAATTGACAGGGTGGTTCACTGGATCCTGC
TGGGGTGCTTGGCCATTTCCTTGTTGATCAGTTCTATATTTGCAATGATACCAAATTGTGAGATTCCTAAAGTTACAAAA
CGTAAAAGAAACTTCACTGAATCTTTCCATGTGAGTAAAGTTCAATACTTCAGCCCAGTGACACTCTTTAGCCTGCTGGC

AATTGTCCCATTTACTGTGTCATTGATCTCATTTCTCCTTTTAATTGTGTCCCTATGGAGACATATTAAGCAAATGAAAC
CCAATGTTACAGGCTGCAGAGACCCCAGCACGAAGGCGCACGTGAGAGCTATGAAAACTGTGACTTCATTTCTCTTTCTC
CTTTTTGTATACTATCTGGCTTCTCTTTTAATGACATTTAGCTACCTTATAAAAGAAAGCAAGTTAGCTGTGATGTTTGG
AGAGGTTATAGCATTTTTCTATCCCTCAGGTCACTCACTTATTTTAATTATTGTAAATAACAAACTGAGGCAGGCATTTG
TCAGGATGCTGAGATGTGGCAAAACAGCCTCCGTGATGTAA
>CesiTAS2R9_NW_004454209.1:610889-609954
ATGCCAAGTACAATGGAGACAATATATATGATCTTGATTGCTGGTGAATTTACTATAGGAGTTTGGGGAAATGGATTCAT
TGTACTGGTTAACTGCACTGGCTGGTTCAAAAGGAGAGATATCTCCTTGATTGACATCATCCTGGTCACCTTAGCCATCT
CTAGAATCTGTTTGCTGTGTGTAATAACTTTAGATGGCTTTGTTATGCTGCTCTCTCCAGATATATATGCCCATGGTGAG
CTAATGAACATTTTGGATGTTTTGTGGACATTTAGCAATCATTCAAGTGTCTGGTTTACTTCTTGCCTCAGCATCTTCTA
CTTACTCAAGATAGCCAATATATCTCACCCGTTTTTCCTCTGGCTGAAGCTAGAGATTAATAGGGTCATCCTTGGGATTC
TTCTGGTGTCCTTTCTCCTCTCCTTAATTATTAGTGTCGCATTGAAGGAGGATTCCTGGTTTAACTTCAAGGTCAATCAT
GAAGAAAACATAACTTGGGAATTCAAAGTGAGTAAAATCTCAAATGCTTTCAAACAGATTATCCTGAACCTGGGGGCAAT
AGTTCCCTTTAGTCTTTGCCTGATCTCATTTTTCTTGTTACTTTTCTCCCTATTTAAACACATCAAACAGATGAAATTTC
ATGCCACAGGGTCCCGAGACCCCAGCACAGAGGCCCACATGAGGGCCATAAAGGCAGTGATCATCTTTCTGCTCCTCTTC
CTTATGTACTATGCAGTCTTTCTTGTGATAACCTCCAGCTATCTGATTCCTCAGAGAAAATTAGTGGTGATGTTTGGTGG
CATAATAACTGTCATTTTCCCCTCAAGCCATTCATTCATCCTAATAAGGGGGAACAGCAAGCTGAGGGAGTCTTTTCTGA
AGGTGCTAAGGAGTGCAAAGTGTTTCCACAAAAGAAGGAACTCTTTTGTTCCATAG
>CesiTAS2R301AP_NW_004454209.1:1014540-1013621
ATGTGAAGTGCACAGGACAACATCTCTTTGGTTGACTTAACCATGGAATTCATAATAGGTATTTGGGGAAATGGATTGAT
TGGAATAATCAACTACATCAACTGGGTAAAACACAGGAAAATCACCTTCATTAACCTAATCCTCACCAGTTTATCCATAA
GCAGAATATGCTTCTTATTGATGATACCGATTGATTTATTACTACTGGTGCTGTCTGCAGACCCACATAGCATTGGTCAA
ATTGGAAAAACTAATATACCATTTTGCGTGATGATTAACTACTTAAGTGTTTGGTTTGACATATGTCTCAGCATCTTGCA
TTTCCTGAAGATTGCTACTTTTTTTCCCAACCTCCTGTTCTTTGATTACAGTGGAGAATTAACATGGTGGTTAATATATT
TCTGGGGTCTTTGCTCTTTTTTCTTTTTAGCATCCTGGTGATAGTCCACATAATTCCTGATTTTTATATCTAGAAACAAA
CAAGACTTGAAGGATTAATACCTGTGAAAATAAACCATTATTTTCTTAGTGCTCATCGGTGTTGAGATCACCATGCCCAT
AACTATTTCCATAGCATCGGTTCTTCTGTTACTTCTTTCTCTTTGGAGGCATATCCTGAGGATGAAGTTCAGTATCACAA
GGTCCCAAGATCCCAGCAGTGAAGCTCTCGGAAAAGCCGTGAGAACGGTGATTTCTTTCTTCTTTCTATTTTTGATTTAT
ATTGCATTGATTCTTATAATAGTTTACAGCCATTTTTTGTCCCCTAACAAGCTGATATGGATATTTGGTAAATTAATAGC
ATCTGCCCATCCTTTAGGCCATTCATTTATCTAATTATGAAAAAACAGCAAGCTGAAGCAAACTGCTCTCTTAGGATTTG
GGGGCAGCTGAAGTGCTTTGTAAAATGAAAGAAATCATAA
>CesiTAS2R301BP_NW_004454209.1:947318-946398
ATGTTAAGTGCATAGGACAACGTGTCTTTGGTTGTCTTCGCCATGGAATTCATAATAGGTATTTGGGGAAATGGATTGAT
TGGAATAATCAACTACATAGACTGGGTAAGACACAGGAAAATTAGCTTCATTACTCTCATCCTCACCAGCTTATCCACAA
GCAGAGTATGTTTCTTGATCATGACACTGATTGATTCATTTCTGCCGGTGCTGTCTGTAGATCCACATAGCATTGGTCAA
ATTGGAAAAACTAATATTCAGTTTTGCATGATGATCAACCACTTAAATGTCTGGTTTGACATCTCTCTCAGCATCTTCTA
TTTCCTGAAAGTAGCTACACTTTTTCCATCCTGTTTTTCTTTGATTAAAGTGGAGAATTAGCGGTGTGGTTATTATTATT
TGTCAGAGGTTTTTGTTCTTTTTTCTTTTTAGCATCGCAGTAATGGACCAAATAATTCCTGATATTTAAAATATAGAAAC
AAACAAGACTTGGAAGAATAACATGTATGAAACACAAACTATTATTTTCTTAGTGCTCATCAGTGTTGAGATCACCTTGC
CCGTAACTATTTCCATAGCATCGCTTCTTCTGTTACTTTTTTCTCTTTGGAGGCATATCTAGAGGATGAAGTTCAGTATC
ACAGGGTCCCAAGATCCCAGCAGTGAAGCTCATGGAAAAGCCACGAGAATGGTGACCTGTTTCTTTTCTCTTTTTGGTTT
CTATTGTGTTGATTCTTATAATAGTTTACAGCCATTAATTGTCCCCTAACAGGCTGATTGGGACATTTGGTAAATTAATA
GTATCTGCCCATCCTTTAGGCTATGCATTTATCCTAATTTTGAAAAAACAGCAAGCTGAAGCCAGCTGCTGTTAGTATTT
GGGGGCAAGTGAAGTACTTGCTAAAAGAAAGGAAAACTTAA

>CesiTAS2R301CP_NW_004454209.1:929926-929008
ATGTCTAGTTCACAGGACAACATCTTTTTGGTTCTCTTAACTATGGGATTCATACTTGGTGCATGGGGAAAAGGATTGAT
TGGAGTAATGAACTATATCTTCTAGGCAAGACACAGGAAAATCGGCTTCATTGACCTCATCCTCATCAGCTTATCCACAA
GCAGAGTACATTTCTTGATCATGACACTGATTGATTCATTTCTACCGGTGCTGTCTGTGGATCCACATAGCGTTGGTCAA
ATTGGAAAAACTAATATTCTGTTTTGCACGATGATCAACCACTTAAATGTCCGGTTTGACATCTCTCTCAGCATCTTCTA
TTTCCTGAAAGTAGCTACATTTTTTTGATCCTGTTTTTCTTAGATTAAAGTGGAGAATTAGCAGCGTGGTTATTATTATT
TTTCTGAGGTCTTTCTTTTTTCTTTTTAGCATCACAGTGATAGACCAAATAATTCCTGATATTTAAAGTATAGAAACAAA
CAAGACTTGGAAGATTAATATGTACGAAACACAAACTGTTATTTTCTTAGTGCTCATCAGTGTTGAGATCACCTTGCCCG
TAACAATTTCCATAGCATCGCTTCTTCTGTTACTTCTTTCTCTTTGGAGGCATATCTAGAGGATGAAGTTCAATATCACA
GGGTCCCAAGATCCCAGCAGTGAAGCTCATGGAAAAGCCATGAGAATGGTGACTTGTTTCTTCTTTCTCTTTTTGATTTC
TATTGTGTTGATTCTTATAATAGTTTACAGCCATTTATTGTCCCCTAACAGGCTGATGTGGACATTTGGTAAATTAATAG
CATCTGCCCACCCTTTAGGCCATTCATTTATCTAATTATGAAAAAACAGCAAGCTGAAGCAAGCTGCTTTTATGATTAGA
GGGCAGCTGAAGTGCTTTCTAAAAGAAAGGAAAACTTAA
>CesiTAS2R301DP_NW_004454209.1:894376-893471
ATCTCAAGCGCACAGGACAACATCTTTTGGGTTGTCTTAACTGTGGAGTTCATACTTGGTACTTAGGGAAATGGATTGAT
TGGAATAATGAACTACATCATCTGGGTAAGACACAAGAAAATCAGCTTCATCACGCTCATCCTCACCAGCTTATCCATAA
GCAGGATGTGTTTCTTGATCATGATAGTGATTGACTCATTTCTACCGGTGCTGTCTGCAGATCCACACAGCACTGGTCAA
ATTAGAAAGATAATATTCTGTTTTGCGTAATGATCAACCACTTAAGCGTCTGGTTTGACACCTGTCTCAGCATCGTCTAT
TCCCTGAAGATAGCTATGTTTTTTCCATCCTCTTTTTCTTTGATTAAAGGTGGTTATTATTATTTTTCTGAGATCTTTGC
TCTTTTTTCTTTTTAGCATCCCAGTGATAGACCAAGTAATTCCTGATATTTTAAATCTAGAAACAAACGAGACTTGAAGG
ATTAACATGGATGAAACACGAACTCTTATTTTCTTAGTGCTCATCGGTGTTGAGATCACCATGCCCATAACTATTTCCAT
AGCATTGGTTCTTCTGTTACTTCTTTCTCTTTGGAGGCATATCCTGAGGATGAAGTTCAGTATTACAGGGTCCCAAGATC
CCAACAGTGAAGCTCATGGAAAAGCCATGAGAATGGTGACTTCTTTTTTCTTTCTTTTTTTGATTTCTATTGTGTTGATT
CTTATAATAGTTCATGGCCATTTATTGTCCCCTAAGAAGCTGATGTAGACATTTGGTAAATTAATAGTATCTGCCCATCC
TTTAGGCCATTCATTTATCCCAATTTTGAAAAACAGCCAGCTGAAGCAAGCTGTTCTTAGGATTCGGGGGCAGCTGAAGT
GCTTTTTAAAAGGAAGGAAAACTTAA
>CesiTAS2R11A_NW_004454209.1:781787-780837
ATGTTGGAGAAAGTTTTCATGATTGTAACAGGTGGAGAATTTATAATAGGAATTTTAGGGAATGGATTTATTGGACTCAC
AAATTGCATCACCTGGATTAGAAATCAGAAGTTATGCTTGGTTGACCTCATCCATACTAGTTTGGCCTTCGCCAGAATCA
GTGAATTATGCATAACAACTATCAGTTTGTTTTCAGTGCTGCTCTATCAGAAAGTCCCTGATGCTACAAAAGGAAACAGG
ATCCTTACTGGTAGCTGGATACTGGCCAACCACTTGAGCACTTGGTTTGCTACTGGTCTCGCAGTCTTTTATTTTTTGAA
GATTGCCAATTTCTCCTATCCCCTCTTCCTTTGGCTAAAATGGAGAATTAACAAGGTAATGTTCATGCTTCTGCTGTTCT
CTGTGCCCTTCCTGCTCATGAAGGAAAACTATTTGGCAAAATTTCCTTTGTCATTTAGTTTGGATGTCTTCCAGTATCAT
GTACAAAACAAGCATGAAAGAAATATGACTAGGTTATTCAATGTGAGTAAAAATAATGATTTAAATTACGTGATGCTCTG
CATTACTTGGACCCTAACTCCTTTCTCTCTTTCCTTGATGTCCTTTGTCCTGTTGCTCCTTTCTCTGTGGAGACACACAC
AGCACAGTGAGCTCAGCATCAAGGATTCCAGAGACCCCAGAATGGAGGCCCACTTCAGAGCCATGAAAACTGTGTTATTC
TTTCTTGTGCTCTTTGCTCTGTACCAATTTGCCTTTTTCATGATGTTTTTAGGTTATTTTTCATTACACAACAAGCTGGT
TGTGATGTTTGGTTATGTGATAGGAATTATATATCCTTCAGGTCACTCATATGTGGTGATTTGGGGAAACAGTCAAATGA
GGAAAGCCTTCTTGAGGATACTTTGGCCCCTGAAGTGTGCCCTGAAAGGAAAGGCACTCTCAGCTGCATAG
>CesiTAS2R11D_NW_004454209.1:663873-662941
ATGTTGAATATGTTGGAGAAAGTTTTCATGATTGTAACAGGTGGGGAATTTGTAATAGGAATTTTAGGGAATGGATTTAT
TGGACTCACAAATTGCATCGCCTGGATTAGAAATCAGAAGTTATGCTTGGTTGACTTCATCCATACTAGTCTGGCCTTCG
CCAGAATCGGTGTATTATGCCTAACAACTATCAGCTTGTTTTCAATGCTGTTCTATCATGAAGTCCCTGATGCTATGAAA
GGAAACATTATCCTTACTGGTAGTGCGATGCTGGCCAACCACTTGAGCACTTGGTTTGCTACTTGTCTCGCTGTCTTTTA

TTTTCTGAAGATCGCCACTTTTTCCCATCCTCTTTTCCTTTGGCTAAAATGGAGAATTAACAAGGTAATTTTCATGCTTC
TGCTGTTCTCTGTGCCCTTCTTGTTCATGAACTTTCCATTGCCATATAGTTTGGATGTCTTCCAGTATCATGTCCAAGAA
AACTATGAAAGAAATATGACTGAGTTATTCAATGTGAGTAAAAATAAACATTTAAGTTCCATGAGAGTCTTCATTATTGG
GTCCCTAACTCCTTTTTCTCTTTCCTTGATGTCCTTTCTGCTGTTGCTCCTTTCTCTGTGGAGACACACGAAGCACAACT
TGCTCAAAGTCAAGGATTCCAGAGACCCCAGAATGGAGGCCCACTTCAGAGCCATGAAAACTGTGTTATTCTTTCTTTTG
CTCTTTGCCCTGTACCAATTTTCCTTTTTCATTATGTTTTTGGGGTATTTTTCACTACAGAACAAGCTGGTTCTGATGTT
TGGTTATGTGATAGGAATTCTGTATCCCTCATGTCACTCGTATGTCGTGATATTTGGAAACAGCCAAATGAAGAAAGCCT
TCTTGGGGATTCTCTGGCAGCTGAAGTGTGTTCTGAAAGGAAAGGCACTCTCAGCTGCATAG
>CesiTAS2R11E_NW_004454209.1:632256-631315
ATGTTGAATACGTTGGAGAAAGTTTTCATGATTGTAGCAGGTGGGGAATTTATAATAGGAATTTTAGGGAATGGATTTAT
TGGACTCTCAAATTGCATCGCCTGGATTAGAAATCAGAAGTTACGCTTGGTTGACTTCATTCTTACTAGTTTGGCCTTTT
CCAGAATCAGTCAATTATGGCTAACAATTGTGAGTTTGTTTTCAGTGCTGCTCTATCAAGAAATGCCTGATACTATGAGA
GGAAACAGTATCTTTACTGGCATCTGGATACTGACCAACCACTTGAGCACTTGGTTTGCTACTTGTCTCGCTGTCTTCTA
TTTCCTGAAGATCGCCAATTTCTCCTGTCCCCTCTTCCTTTGGCTAAAATGGAGAATTAACAAGGTAGTTTTCATGCTTC
TGCTGTTCTCTGTCCCCTTCCTGTTCATGGATTTTTCTGTGCCATATAGTTTGGATGTCTTCTGGTATCGTGTACAAAAA
AAATATGAAAGAAATATGACTGTTTACTCAATGTGGGTAAAAGCAAAGATTTAAATTACGTGATAATCTTCATTATTGG
GTCCCTCCCTCCTTTCTCTCTTTCCTTGATGTCCTTCCTCCTGTTGCTCCTTTCTTTGTGGAGACACACACAGCACAGTG
AGCTCAGCGTCAAGGATTCCAGAGACCCCAGAATGGAGGCCCATTTCAGAGCCATGCAAACTGTGTTATTCTTTCTCCTG
CTCTTTGCTCTGTACCAATTTTCCTTTTTCATGACATTTTTGGGTTATTTTTCACTACAGAACAAACTGGTTGTGATGTT
TGGTTATACGATAGGAATTCTATATCCTTTAGGTCACTCATATGTCGTGATTTTTGGAAACAGCCAAATGAGGAAAACCT
TCTTGGGGATTCTCTGGCACCTGAAGTGTGGCCTGAAAGAAAAGGCGCTCTCAGCTGCATAG
>CesiTAS2R12A_NW_004454209.1:796839-795904
ATGGCAAGCACATTGAAGAAGATATTTATGATCCTTTATGCTGGAGAATTCATGATGGGCATTTTGGGAAATGGATTCAT
TGTATTGGTTAACTGTATTGACTGGATCAGGAGCTGGAAGTTCTCCCTGATTGACTTTATTCTCACCTGCTTGGCGATTT
CCAGAATATTTCTGCTGTGCATAATAATTTCATTTATAGGCTTAAAATCAATCAGTGAGGAAATATGGTACAATAATAAT
CTACTGAGAAGTTTGGAAATCCTCTGGACAAGATCCAATCATTTCTGTGTGATCTGTACCACCTGCCTCGGTGTCTTCTA
TTTCCTCAAGATAGCCAACTTTTCTAATCCCGTTTTCCTCTGGATGAAGTGGAGAATTCACAAGGTGCTTCTCATTCTTG
TGCTGGGGGCAGCCCTCTCTTTCTGTTTGCACCTTTTCTTTAAGGAGACAGTAGTTAGGAGCCTGATCAAAAATCAGGGA
AACACTGAACAAAATGTGACCTGGAACTTCACAGTGAGAAAATATGATTTATTAACTTCTCAAATGCTTTTTGACATAAT
GTTCATCATCCCCTTTGTAGTGCCACTGGCCTCCCTGCTCCTTTTAATCCTGTCCTTATGGAGCCACACCAGGCAGATGA
AGGGCACAGGTTCTAGGGATTCTAGCACAGAGGCCCATGTGAGAGCCATGAAGTGTATGATTTCATTCCTACTCCTCTTC
TTCTTGTACTGTTTGACTCATATTATGATAAATTCAGCCTATGCCGTTCTAGACAGTTTTGTGGCAAAGATTCTTGCTAA
TGTGCTTGTGTTTTTCTATCCATCTGGCCACCCATTTCTTCTGATTTTATGGAACAGCAAATTGAAACAGGCTTCCCTCA
GTGTCCTGAGGAAGCTGAAGTGTTGCCTGAATCTAAGGAAACCTACGTTCCCGTAA
>CesiTAS2R12B_NW_004454209.1:759676-758738
ATGACAAGCACATTGAAGAAGATATTTATGATCCTTTATGCTGGAGAATTCATGATGGGTATTTTGGGAAATGGATTCAT
TGTATTGGTTAACTGTATTGACTGGATCAGGAGCTGGAAGTTCTCCCTGATTGACTTTATTCTCACCTGCTTGGCGATTT
CCAGAATATTTCTGCTGTGCATATTAATTTTATTTATAGGCTTAGAGGTAAACTATGGCAAAATACGGTACGACAATAAT
AATCTAATGATAAGTTTGGAAATCCTCTGGACAGGATCCAATTACTTCTGCACGGCCTGTACCACCTGCCTCAGTGTCTT
CTATTTCCTCAAGATAGCCAACTTCTCTAATCCCATTTTCGTCTGGATGAAATGGAGAATTCACAAGGTGCTTCTCATTC
TTGTGCTGGGGGAAGCCCTCTCTTTCTGTTTGCGCCTTCTCTTTAAGGAGACAGTAGTTAGGAGCCTGATCAAAAACCAG
GGAAACACTGAACGAAATTTGACGTGGAACTTCACAGTGAGAAAATATGATTTATTAACTTCTCAAATGCTCCTTAACAT
AATGTTCCTCATCCCTTTTGTAGTGTCACTGGCCTCCCTACTCCTTTTAATCCTGTCCTTATGGAGCCACACCAGGCAGA
TGAAAGGCACAGGTTCTAGGGATTCTAGCACAAAGGCCCATGTGAGAGCCATGAAGTGTATGATTTCATTCCTACTCCTC

TTCTTCATCTACTATTTGAGAAATAGTATAATAAATTCAGCCTCTGCCATTCTAGACAGTTTTGTGGCAAAGATTTTTGC
TAATGTGCTCGTATTTTTCTATCCATCTGGCCATCCATTTCTTCTGATTTTGTGGAACAGCAAATTGAAACAGGCGTCTC
TCAGTGTCCTCAGGAAGCTGAAGGGTTGCTTGAATCTAAGGAAACCTACATCCCCATAA
>CesiTAS2R12C_NW_004454209.1:719213-718275
ATGACAAGCACACTGAAGAAAATATTTATGATCCTCTATTCTGGAGAATTCATGATGGGGATTTTGGGAAATGGATTTAT
TGTACTGGTTAACTATATTGACTGGATCAGGAGCAGGAAGTACTCCCTGATTGACTTTATTCTCACCTGCCTGGCGATTT
CCAGAATATTTCTGCTGTGCATATTAATTTTGTTTATAGGCTTAGAGGTAAACTATGGCAAAATACGGTACAACAATAAT
AATCTAATGATAAGTTTGGAAATCCTCTGGACAGGATCCAATTACTTCTGCACGGCCTGTACCACCTGCCTCAGTGTCTT
CTATTTCCTCAAGATAGCCAACTTTTCTAATCCCATTTTCCTCTGGATGAAATGGAGAATTCACAAGGTGCTTCTCATTC
TTGTGCTGGGGGAAGCCCTCTCTTTCTGTTTGCGCCTTCTCTTTAAGGAGACAGTAGTTAGGAGCCTGATCAAAAACCAG
GAAAACACTGAACGAAATTTGACGTGGAACTTCACAGTGAGAAAATATGATTTATTAACTTCTCAAATGCTCTTTGACAT
AATGTTCCTCATCCCCTTTGTAGTGTCACTGGCCTCCCTACTCCTTTTAATCCTGTCCTTATGGAGCCACACCAGGCAGA
TGAAGGGCACAGGTTCTAGGGATTCTAGCACAGAGGCCCATGTGAGAGCCATGAAGTGTATGATTTCATTCCTATTCTGC
TTCTTTATATACTACCTGAGCCATATTTTGATGGACTCAGCCTATGTCATTCTAGACAGTTTTGTGGCAAAGGTTTTTTC
TAATGTGCTCGTATTTTTTGATCCATCTGGCCACCCATTTCTTCTGATTTTATGGAACAGCAAATTGAAACAGGCTTCTC
TCAGTGTCCTGAGGAAGCTGGAGTGTTGCCTGAATCTAAGGAAACCTACGTTGCCATAA
>CesiTAS2R12D_NW_004454209.1:679247-678309
ATGGCAAGCACATTGAAGAAGATATTTGTGATCCTTTATGCTGGAGAATTCATGATGGGGATTTTGGGAAATGGATTCAT
TGTATTGGTTAACTGTATTGACTGGATCAGGAGCTGGAAGTTCTCCTTAATTGACTTTATTCTCACCTGCTTAGCTATTT
CCAGAATACTTCTGCTGTGCATAGTAATTTTATTTATAGGCTTAGATGTAAAATATGAGAAAATACAGTACAATGATGAT
AATCTACTGAGAAGTTTGGAAATCCTCTGGACAGGATCCAATTACTTCTGCACAGCCTGTACCACCTGCCTCAGTGTCTT
CTATTTCCTCAAGATAGCCAACTTTTCTAATCCCATTTTCCTCTGGATGAAATGGAGAATTCACAAGGTGCTTCTCATTC
TTGTGCTGGGGGAAGCCTTCTCTTTCTGCTTGTGCCTTCTCTTTAAGGAGACAGTAGTTAGGAGCCTGATCAAAAACCAG
GAAAACACTGAACGAAATTTGACGTGGGACTTCACAGTGAGAAAATATGATTTATTAACTTCTCAAATGCTCCTTAACAT
AATGTTCCTCATCCCCTTTGTAGTGTCACTGGCCTCTCTACTCCTTTCAATCCTGTCCTTATGGAGCCACACCAGGCAGA
TGAAGGGCACAGGTTCTAGGGATTCTAGCACAGAGGCCCATGTGAGAGCCATGAAGTGTATGATTTCATTCCTACTCCTC
TTCTTCATCTACTATTTGACTAACAGTATAATAAAATCAGCCTCTGCCATTCTAGACAGTTTTGTGGCAAAGATTTTTGC
CTATGTGCTCGTATTTTTCTATCCATCTTTTCATCCATTCCTTCTGATTTTATGGAACAGCAAATTGAAACAGGCATCTC
TCAGTGTCCTGAGGAAGCTGAAGTGTTGTATGAATCTAAGGAAAGCTACATCCCCATAA
>CesiTAS2R16_NW_004454157.1:6065473-6066378
ATGATACCCGTCCAACTCACTGTCTTCTTCATGATCGTCTATGTGCTCGAGTCCTTGACAATAATTCTGCAGAGCGGCTT
ACTTGTTGCAGTGCTGGGCAGAGAGTGGGTGCAGGTCAAAAGGCTGTCAGCTGTGGACATGATTCTCACCAGCCTGGGCA
TCTGCCACTTCTGTCTACAGTGGGCATCGATGCTGAACAATTTTTACTCCTATTTCAATCCTAACTATGTATTTTGGTCC
TTATCGATCGCCTGGGACTTTTTAAATACTCTTTCATTCTGGTTAACCAGCTTGCTTGCTGTCGTCTACTGTGTCAAAAT
CTCTTCCTTCACCCACCCTATCTTCCTCTGGCTGAGGTGGAGAATTTTGAGGTTGGTTCCCCGGCTGTTGCTGGGTTCTC
TGCTGCTTTCTTGTGTGACTCTCATCTGTTCAGCTGTTAGGAATCACATCAAGATAAAGATGATCTCTATGGAGCATTTC
CCTAGAAACAACACTATGATCGAGAGACTTAAGATGCTTCTGCAGAAATTTACCTTATCTCAGCCAATGGTTGTGATGAT
TATTCCTTTCCTTCTGTTCCTGGCCTCCACCATCTTGCTCATGGCCTCCTTGTCCCAACACTTGGGGCAGATGCAACATC
ACAAAGCTGGCCACAGCAACTCCAGCACGAAAGCTCATTCCACTGCCCTGAGGTCTCTTGCCGTCTTCCTCATCTTCTTC
ACCTCTTACTTTCTGACCATAGTCATCTCCTTTATGAGCATCCTATATGATAAGAGCTCCTGGTACTGGGTCTGGGAAGC
TGTTATCTATGCTATAGTCTCTGTTCAATCCACCTTACTAATGCTCAGCAGCCCTACGTTGAAAAAGGTTTTAAAGCTAA
GGTGCTGGGTCCTAGAAACTGCCTGA
>CesiTAS2R408A_NW_004454209.1:1010198-1009299
ATGTTAACTTTACTACCAAGCATTTTTTGCATCCTAATAACGACAGAATTTACTCTGGGAAATTTTGCCAATGGCTACAT

AGCACTGGTGAACTGCATTGACCGGGTCAAGAGACAAAAGATGTCCTCAGCTGATCAAATTCTCATGGCTCTGGCGGTCT
CCAGAATTGGGTTGCTCTGGGCAATATTAATAAATTGGTATAGAGCTGTGCTAACTCCAGTTTTATATAGTTTAGAAGTA
AGAATTATTGTTTATATTGCCTGGGCAGTAAGCGACCATTTTACCACCTGGCTTGCTACTAGCCTCAGCATATTTTATTT
GCTCAACATAACTAATTTCTCTAGGCTTATATTTCTTTACCTGAAGTGGAGAGTTAAAAGGGTACTTCTCATAACGCTGT
TGGGGTCTTTGGTCTTTTTGGTTTCTTATCTTGCAGTGCTGTGCATAGATGAGAATATGCAGACTAATGAGTATGAAGGA
AACATTACTAGGAAGACCAAATTGAAGGATATTGTACGCCTTTCAAATATGACTCTATTCACAATAACAAACTTCACATC
TTTTACTATGTCCCTGACATCTTTTCTGTTGTTAATCATTTCCCTGTGGAAACATCTCAAGAAGATGCAGCTCAACGGCC
AAGGATCCCAAGATCCCAGCACCAAAGTCCATATAAGAGCCGTGCAAACTGTGGTCTCCTCTCTCTTGCTACATGCCAGT
CTCTTCCCGGCTCTAGTTATCTCAATTTGGAATCCAAGTAGGCTGCAGAATAAACTGGTTATTATGCTTTGCCAGGCTCT
TGGAATGCTGTATCCTTTAAACCACTCATTTATCCTGATTTGGGGAAACAAGAAGCTAAGACAAGCCTTAAAAAATATAA
GAAAGACTCTAAGATTTCCACACCATTAA
>CesiTAS2R408B_NW_004454209.1:1000217-999306
ATGATAAGGTTACTACCAAGCATTTTTTCCATCCTAATAACGACAGAATTTATTCTGGGAAATTTTGCCAGTGGCTTCAT
AGCACTGGTGAACTGCATTGACTGGATCAAGAGACAAAAGTTGTCCTCAGCTGATCAAATTCTCATGGCTCTGGCGGTCT
CCAGAATTGGTTTCCTCTGTGTAATATTAATAAATTGGTATACAACTGTGCTCCATTCAGTTTTATATAGTTTAAAAGTA
AGAATTATTTTTCGTATTGCCTGGACAGTAAGCAACCATTTTAGCATCTGGCTTGCTACTAACCTCAGCATATTTTACTT
GGTCAAGATAGCTAATTTCTCTAGCCTTATATTTCTTTACCTAAAGCAGAGAGTTAAAAGCGTACTTCTTGTAATGCTGC
TGTTGGGGTCTTTGGTCTTTTTGGTTTCTTATCTTGCAGTGCTATTTGTAGATGATAATATGCAGACTACTGAGTATGAA
GAAAACATCACTCAGAAGACCAAATTGAGGGACATGTTACACCTTTCAAATATGACTCTATTCATGGTAGTAAACTTCAT
ACCTTTTACTATGTCCCTGACATCTTTTCTGCGGTTAATCATTTCTCTGTGGAAACATCTCAAGAAGATGCAGCTCAACG
GCAAAGGATCCCAAGATCCCAGCACCAAAGTCCACGTAAGAGCAGTACTAAGTGTGGTCTCCTTTCTCTTGCTGTATGCC
TGTCACTTCCTGACTATAGTTATCTTCATTTGGGCTTCTGTCAGGCCGCAGAATGAACTGGTTCTCATGCTTTGCGAGGC
TCTGGCAATGCTGTATCCTTTAAGCCATTCATTTATCCTCATTTGGGGAAACAAGAAGCTAAGACAGGCCTTCAAAAATA
TAAGAAAGATTATAAGATTAACAGATCATTAA
>CesiTAS2R408E_NW_004454209.1:926068-925160
ATGACGACTTTACTACCAAGCATTTTTTCCATCCTAATAACGACAGAATTTATTCTGGGAAATTTTGCCAATGGCTTCAT
AGCGCTGGTGAACTGCGTTGACTGGGTCAAGAGACAAAAGATGTCCTCAGCTGATCAAATTCTCATGGCTCTGGCGGTCT
CCAGAATTGGTTTCCTCTGGGTAGTATTAATAAATTGGTATACAACTGTGCTTCCTCCAGTTTTATATAGTTTAGAATTA
AGAATTGTTCTTCGTATTGCCTGGGCAGTAAGCAACCATTTTAGCATCTGGCTTGCTACTAGCCTCAGCATATTTTACTT
GGTCAAGATAGCTAATTTCTCTAGCCTTATATTTCTTTACCTAAAGCGGAGAGTTAAAAGTGTACTTCTCGTAATACTGT
TGGGGACTTTGGTCTTTTGGGTTTCTCATCTTGTAGTGCTGTGCATAGATGAGAATATGAAGACTAATGAATATGAAGGA
AACATCACTCAGAAGACCAAATTGAGGGACATTCTACGCCTTTCAAATATAACTCTATTCACGCTACTATACTTCATACC
CTTTACTCTGTTCCTGACATCTTTTCTGCTGTTAATCATTTCCTTGTGGAAACATCTCAAGAGGATGCAGCTCAACGGCC
AAGGATCCCAAGATCCCAGCACCAAAGTCCACATAAGAGCTATGCAAACTGTGGTCTCCTTTCTCTTGCTATATGCCAGT
TACTTCCTGGCCATAGTTATCTCAGTTTGGACTTCTATTAGGTTGCAGAATGAACTGCTTCTCATGCTTCCCGAGGGTCT
CATAATGCTCTATCCTTCAAGCCACTCGTTTATCCTGATTTGGGGAAACAAGAAGCTAAGACAGACCTTGAAAAACGTAA
GAAAGATGATAAGATTACCATACCATTAA
>CesiTAS2R408G_NW_004454209.1:890272-889364
ATGATAAGTTTACTACCAAGCATTTTTTCCATCCTAATAACGACTGAATTTATTCTGGGAAATTTTGCCAATGGCTTCAT
AGCGCCCGTGAACTGCATCGACTGGGTCAAGAGAAAAAAGATGTCCTCAGCTGATCAAATTCTCACAGCTCTGGCGGTCT
CCAGAATTGGATTGCTCTGGGTAATATTAATAAATTGGTATACAACTGTACTTACTCCAGTTTTCCATAATTTAGAAGTA
AGAATTATTTTTCATATAGCCTGGGCAGTAAGCAACCATTTCAGCCTCCGGCTGGGTACTAGCCTCGGCATATTTTATTT
GCTCAAGATAGCTAATTTCTCCAGCCTTATATTTCTTTACCTAAAGCAGAGAGTTAAAAGGGTACTTCTCGTAATACTGT
TGGGGAGTTTTGTCTTTTTGGCTTCTTATCTTGCAGTGCTATGCATAGATGATAATATGCAAACCAATGAATATGAAGGA

AACATAACTGGGAACACTAAATTGAGTGACATTTTACGACTTTCAAATATGACTCTATTCACACTGATAAACTTCACACC
CTTTACTATGTCCCTGACATCTTTTCTTCTGTTAATCATTTCCCTGTGGAAACATCTCAAGAAGATGCAGCTCAATGGCA
AAGGATCCCAAGATATCAGCACCCAAGTCCACATAAGAGCCATGCAAACTGTGGTCTCCTTTCTCTTGCTATATGCCGGT
TACTTCCTGGCTCTAATCATCTCATATTGGAGTTCTGATAGGCTGCAGAATGAACCAGTTTTCATGCTTTGCCAAGTTCT
TAGAATGCTGTATCCTTCAAGCCACTCATTTATTCTGATTTGGGGAAACAAAAGGCTAAGACAGGTGTCGAAAAATATAA
GAAAGATTATAAGATTACCATACTGTTAA
>CesiTAS2R38_NW_004454187．1:14459685-14460692
ATGTTGACTCTGCCTTCCATCATAACTGTGCCTTGTGAAGTCAAGAATGTATTTCTATTCCTTTCAGTTCTGGAGTTTGC
AGCAGGAATCCTGGCCAATGCCTTCATTTTCTTGGTTAATTTTTGGGACGTGGTGAAGAGGCAGCCACTGAGCAACTGTG
ATCTTATCCTACTGTGTCTCAGCGTCAGCCGGCTTTTCCTGCATGGGCTGCTGTTTCTGGATGCCATCCAGCTTAGCCAC
TTCCAGCGGATGAAAGACCCACTGAGCCTCAGCTACCAAACCACCATCATGCTCTGGATGATCACAAATCAAGCTGGCCT
CTGGCTCGCCACCTGCCTCAGTCTCCTCTACTGCTCCAAGATTGTCCGTTTCTCTCACACCTTCCTGCTCTGCTTGGGAA
GGTGGATCTCCAGGAAGATCCCCAACATGCTCCTGGGTGCTGTTCTTTTCTCCTGTGTCTGTACTGCCATCTGTTTGTGG
GACTTTTCAAGTAGATCTCGCTTCACAGTCACAACTATGCTATTCATGAATAACAATACAGAACTCAGTTTGCAAATTAC
AAAACTCAATTTCTTTCAGTCCTTCCTCTTTTGCAGCCTGGGGTCCATCCCTCCTTTCTTATTATTTCTGGTTTCTTCTG
GGGTGCTAATTGTCTCCCTGGGGAGGCACATGAGGACAATGAGGGCCAAAACCAGAGAGTCTTGCGATCCCAGCCTGGAG
GCTCATATCAAAGCACTCAAATATCTCGTCTCCTTTCTCTGCCTCTATGTGGTGTCATTCTGTGCTGCCCTCATCTCAGT
GCCTTTACTGATGCTGTGGCACAACAAGATTGGAGTAATGGTCTGTGTGGGGATAATGGCAGCTTGTCCCTCGGGACATG
CAGCCATTCTGATCTCAAGCAATACCAAGCTGAGGAGAGCTGTGGAGGCCATTCTGCTCTGGGCTCGGAACAGCCTAAAG
GTAAGGGCAGTTTACAAGGCAGATCCCAGGAGGTCAGGTCTATGTTGA
>CesiTAS2R39_NW_004454187．1:13100873-13099914
ATGATAGAAACCTGCAATCCCCCAGAAAGTAAATTGTCACTGTTTTGGATCGCCTTAATTTGCACAATTATAGGCACTGA
ATGCATCATCGGTATCTTTGCGAATGGGTTCATTGAGGCTATAAATGCAGCTGAATGGATTAAGAATAAGGCAGTCTCCA
CAAGTGGCAGGATCCTGTTTTTCCTGAGTGTATCCAGATTAGCTTTCCAAAGCCTCATGGTGCTAGAACTTACCTTCAGC
TCAACATCCCCAAGTTTTTATTATGAAGATGTTGTATATGATATATTCAAAGCCAGTTTCATGTTCTTAAATTCTTGTAG
CCTATGGTTTGCTGCCTGGCTCAATTTCTTCTACTTCGTGAAGATTGCTGATTTCTCCTACCCCCTTTTCCTCAAGCTGA
AGTGGAGAATTTCTGGATTGATGCCCTGGCTTCTGTACCTATCAACGTTTATTTCCTTGGGTAACAGTGTGCTCTTATCC
AATAACATCTACACTGTATATTGTAACAATTCTTTACCTATCCCCTTCTCCAACTCCACTGAGAAAAAATACTTCACTGA
AACCAATGTGATCAACCTGCTTCTTCTCTATTACCTGGGGATCCTCATTCCTCTGATCATGTTCATCCTTGCAGCCACCC
TGCTGATCATCTCTCTCAAGAGACACACCCTACACATGGAAAGCAATGCCACTGGCTACAGGGACCCCAGCATGGAGGCT
CACCTGGGGGCCATCAAAGCTACCAGCTACTTTCTCATTTTCTACGTTTTCAATGCAGTTGCTCTATTTCTCTATATGTC
CAACATCTTTGACATCAACAATTTCTGGAATATTTTGTGCAAAATCATCATGGCTGCCTACCCTGCTGGTCACTCCATCC
TGCTGATTCAGGACAACCGTGGGCTGAGAAGAGCCTGGAAGCGGCTTCAGCTTCGAGTTCATCTTTACCTAAAAGACTAG
>CesiTAS2R40_NW_004454187．1:13070805-13069834
ATGGCGATGGTGATCACAGATGCCACAGATAAAGACACGTCCAGGTTTAAAACTGTCTTCACCTTGGTAGTCTCCGGAAT
AGAGTGCATCACTGGCATCATTGGGAACGGCTTCATCACAGCCATCCATGGGGCCGAGTGGGCCAGAGGCAAAAGACTCC
CTGTTGGTGACTGCATTCTCTTGATGCTGAGCTTTTCCAGGCTCTTGCTACAAATTTGGATAATGCTAGAGAATACTTAC
AGTCTACTATTCCAGGTCATTTATAAACAAAACACAGTGTATATACTTTTCAAAGTCATCATCATGTTTCTGAACTATTC
CAACCTCTGGCTTGCTGCCTGGCTCAATGTCTTCTATTGTCTTAGAATTGCAAACTTTACTCACCATTTGTTCTCCATGA
TGAAGAGGAAAATCATGGTGCTGATGCCTTGGCTTCTGAGGCTGTCACTGTTCATCTCCGTATGCTTCAGCTTTCCCTTC
TTTAAAGATATCTTCAATGTGTACGTGAATAGTTCCATTCCTATCCCCTCCTCCAACTCTACTGAGAAGAAGTACTTCAC
TGAGACCAATGTCTTCAACCTGGTTCTTCTCTATTACCTGGGGATCCTCATTCCTCTGATCATGTTCATCCTTGCAGCCA
CCCTGCTGATCATCTCTCTCAAGAGACACACCCTACACATGGAAAGCAATGCCACTGGCTTCAGAGACCCCAGCATGGAA
GCTCACATGGGGGCCATCAAAGCTATCAGCTACTTTCTCATCCTCTACACTTTCAATGCAGTTGCTCTATTTCTTTCTAT

GTCCAACATCTTTGCTGTCAACAGTTCCTGGAATATTTTGTGCAAAATCATCGTGGCTGCCTACCCAGCTGGCCACTCAG
TGCTACTGATCTTGGGAAATCCTAGGCTGAGAAGAGCCTGGAAGCGGTTTCAGCACCAAGTTCATCTTTACCTATATGGG
CAGACTCTGTGA
>CesiTAS2R42_NW_004454209.1:1049029-1048049
ATGTTCATTGTATTGGATAAAATCTTTCTGATACTGGCAGCAGTGGAATTCATAATCGGAATGTTCGGGAATGTGTTCAT
TGGACTGGTAAACTGCTCCGAATGGATCAAGAACCAAAAGATTTCCTTAGCTGACTTCATCCTTACGTGCTTGGCTATCT
CCAGAATCACTCAGCTGTTGGTTTTATTGTTTGAATCATTTATGCTGGGACTACCTTCGTATTTATATGGCATTTATAAA
CTAGCAAAACCCATTAGTTTGCTCTGGAGAATGACTAATCACTTCACTGCCTGGTTTGCTACCTGCCTAAGTGTTTTCTA
CCTCCTTAAGATAGCTCACTTCTCCCACTCCCTTTTCCTCTGGCTGAAGTGGAGGATGAACAGAGTGGTTCTTGTGATTC
TTGTATTTTCTTTGTTGTTTCTGATTTTTGACTTTCTATTGCTAGAAACATTAAATGATTTCTTCTTGAATATCTGTAAA
ATAGATAAAAGTAATCTGACTTTATATTTAGATGAAAGGAAAACTCTCCATGTTCAAACCCTGATTCTTCTTAGCCTGAC
CTATTTCGTCCCTATTGTTTTGTCTCTGACCTCATTGCTCCTTTTATTTCTGTCCTTGGTAAGACACACCAGAAATTTGC
GGCTCAACTTGATGGGCTCGAGGGACTCCAGCATACAGGCCCATAAAAGGGCCATGAAAATGATGATGTCTTTCCTCTTC
CTTTTCATAGTTCATTGTTTTTCACACAATTGACAAATTGGACACTTTTTATATTTTGGAAGAACAGGTTCACAAAGTT
TGTCATGTTAGCAGTATATACCTTTCCTTCAGGTCACTCATTTATTTTGATTCTGGGAAACAACAAGCTAAGACAGACAG
CCTTGAAGGTACTGTGGCATCTTAAAAGCTCCTTAAGAAGAGAAAATTCGTTAGCTTTACGGAGTAGACTTTCCAGAGCC
TTTTCAAAGATAATAACTTAA
>CesiTAS2R62A_NW_004454187.1:12897480-12896548
ATGCCCTCCTCACCCACATTGATCTTCATGGTCATCTTTTTCCTGGAGTCATTCGCTGCAATGCTGCAGAATGTCTTTAT
GGTTGCTGTGCTGGGAAGGAAGTGGGTGCGATGCCACACACTGCCTGCAGGTGACATGATTGTGGCCTGCCTGGCTGCCT
CCAGGTTCTGCCTGCATGGGATGGCCCTCCTGAACAACCTCCTGGTCTCCATTAATTTTTCTTCTATAGTTTACTATTTC
AACATCCCCTGGGAGTTTATCAACATTCTCACTTACTGGCTTACTGCCTTGCTTGCTGTCTTCTACTGTGTGAAGATCTC
ATCCTTCTCTCATGCCATCTTCTTCTGGCTGAAGTGGAGGATTTCTCAGTCAGTGCCCAGGTTGCTGCTGGGCTCCCTGA
TCATATCTGGTGTGACAGTCATCCCAGTAGCCACTGGGAATTTAATTCTTCAGCAGATGATTGCCTCCCAGAGTTCCCAT
GGAAACTGCACTCTGGCGGATAGAACACAAACCATCTATAGGTACTTTCTTCTGCCTAATGTAGTGCTTGTGTGGTTGAT
TCCCTTCCTCCTGTTCCTGGTGTCCACCCTCTTGCTCATGTTCTCACTGCACAGGCACTTGCGGCAGATGAGGGACCACA
GACCTGGCCCATGTGATCCCAGCACCCGGGCTCACACTGTGGCTCTGAAGTCACTTGCCTTCTTCTTTGTCTTCTACACA
TCGTATTTCCTGTCCCTGATTATTGCTGTTAATAAAATTGCAACACTGCGGGATCAGTGGCACTGGGCCTGGGAAGTGGT
GACCTATGCAGGTATCTGTCTGCACTCCAGCATCCTGGTGCTAAGCAGCCCCAAGCTGAGAAAGTTTGGAAAGATGATGG
TTTGGAAAGCCCCGGAGAAAAGGCTATTCATCTCGAGTTGTCAAAATCAGTAA
>CesiTAS2R62B_NW_004454187.1:12854139-12853207
ATGCCCTCTTCACTCACGTTGTTCTTCATAGCCATCTTTTGCCTGGAGTCATTGGCTGCAATGCTACAGAATGGCTTTAT
TGTTGCTGTGTTGGGCAGGGAGTGGATGCAATGCCTCAGACTGCCCTCTGGTGATATGATTGTGGCCTGCCTGGCTACCT
CCAGGTTCTGCCTGCATGGGATGGCCCTCCTGAACAACCTCCTGGCTTCCTTTAATTTTTGTTCCATAGTTTACTATTTC
AACATCCCATGGGACTTGATCAACACTCTCACTTACTGGCTTACTGCCTGGCTTGCTGTCTTCTACTGTGTGAAGATCTC
ATCCCTCTCTCATCCCATCTTCTTCTGGCTGAAGTGGAGGATTTCTAGGTCAGTGCCCAAGCTGCTGCTGGGCTCCCTGA
TTATATCTGGTGTGACAGTCATCCCAGTAGCCACTGGGAATTTAATTCTTCAGCAGATGATTGCCTCCCAGAGTTCCCAT
GGAAACTGTACTCTGGCAGATAGAACACAAACCTTCTATAGGTACTTTCTTCTGCCTAATGTAGTGCTTGTGTGGTTGAT
TCCCTTCCTCCTGTTCCTGGTGTCCACCCTCTTGCTCATGTTCTCACTGCACAGGCACTTGCAGCAGATGAGGGACCACA
GACCTGGCCCATGTGATCCCAGCACCCAGGCTCACACCATGGCCCTGAGGTCACTTGCCTTCTTCCTCATCTTCTACACA
TCGTATTTCCTGTCCCTGATTATTGCTGTTAAGAAAATCACAACACTTCAGAATCTGTGGCACTGGGCCTGGGAAGTGGT
GACCTATGCAGGCATCTGTCTGCACTCCAGCATCCTGGTGCTAAGCAGTCCCAAGCTGAGAAAGTCCCTGAAGATGATGG
TCTGGAAAGCCATGGAGAAAAGGCAATTCATCTCGAGTTGTCAATATCAATAA
>CesiTAS2R62D_NW_004454187.1:12818654-12817722

ATGCCCTCCTCACTCACATTGATCTTCATTGTCATATTTTTCCTGGAGTCGTTGGCTGCAATGTTGCAGAATGGCTTTAT
GGTTGCTGTGCTGGGCAGGGAATGGATGCGATGCCACACACTGCCATCAGGTGACATGATTGTGGCCTGCCTGGCTGCCT
CCCGGTTCTGCCTGCATGGGATGGCCCTCCTGAACAACTTCCTTGGCTTCTTTGATTTTTGTTTCAAAGCTTACTATTTC
AACATTTCCTGGGGCTTCATCAACGCTATCACTTTCTGGCTCACTGCCTTGCTTGCTGTCTTCTACTTTGTGAAGATCTC
TTCCTTCTCACATCCTATCTTCTACTGGCTGAAGTGGAGGATTTCTCAGTCAGTGCCCAGGCTGCTTCTGGGCTCCCTGA
TCACATCTGGTGTCACAATCATTGCAGTAGCCACTGGGAATAGCATTCTTGTGCAGATGAGTGCCTCCCAGAGCTCCCAT
GGAAACAGCACCCTGGTTGAAAGAATACAGACCATCTATTGCCATTTTTTTCTGCCTAATGAAGTGCTCTTGTTGTCCAT
TCCCTTCCTCTTGTTCCTGGTGTCCACACTTTTGCTCATGTTCTCACTGCACCAGCACTTTCAGCAGATGAGGAACCACA
GACCCAACCAAGGCGGTTCCAGCACCCAGGCTCACATCATGGCCCTGAGGTCACTTGGTTTCTTCCTCATCTTCTACACA
TCATATTTTCTGTCCCTGATTATTGTTTCTATGCATATCACAACCCTGCAGAATCAGTGGCACTGGGCCTGGGAAGTGGT
GACCTATGCCGGTATCTGTCTGCACTCCAGCATCTTGGTGCTAAGCAGCCCCAAGTTGAGAAAGACCCTGAAGATAATGC
TTTGGAAAGCCCTGGACAAAAGTTGGTTCATCTCAAGTTATCAGTATCAATAA
>CesiTAS2R62F_NW_004454187.1:12786795-12785863
ATGTCCTCCTCATCCACATTGATCTTCATGGTCATCTTTTTCCTGGAGTCATTGGCTGCAATGTTGCAGAATGGCTTTAT
GGTTGCTGTGCTGGGCAGGGAGTGGATGCGATGCCACACACTGCCCTCAGGTGACATGATTGTGGCCTGCCTGGCTGCCT
CCCAGTTCTGCCTGCATGGGATGGCCCTCCTGAACAACCTCATGGGCTCCTTTGGTTTTCGTTCCAGAGTTAACTATTTC
AACATTTCCTGGGTCTTTATCAATACACTCACTTTTTGGCTTACTGCCTGGCTTGCTACCTTTTACTGTGTGAAGATCTC
ATCCTTCTTTCATCCTATCTTCTACTGGCTGAAGTGGAGGATTTCTCAGTCAGTGCCCAGGCTTCTGCTGGGCTCCCTGA
TCATATCTGGTGTGACAGTCATCTCATTAGCCAGTGGGCATAGCACTCTTGTGCAGATGAGTGTCTCTCAGAGTTCCCAT
GGAAACAGCACCCTGGCTGAAAGAATACAGACCATCTATCACAACTTTTTTCTGCCTAATAAAGTGCTTGTGTTGTTAAT
TCCCTTCCTCCTGTTTCTGGTGTCCACGCTCTTGCTCATATTCTCACTACACTGGCATTTGCAGCAGATGAGGAGCCACA
GACCCGACCGACATGATCCCAGCATCCAGGCTCATATTGTGGCCCTGAGGTCACTTGCTTTCTTCCTCATCTTCTACACA
TTATATTTCCTGTACCTGATTATTGTTTCTATGCATATCACAACCCTGCAGAATCAGTGGCACTGGGCCTGGGATGTGGT
GACCTATGCAGGCATCTGTCTGCACTCCAGCATCCTGTTGCTAAGCAGCCCCAAACTGAGAAAGGCCCTGAAGATGATGC
TTTGGAAAACCCTGGAGAAATGGTGGTTGATCTCAAGTTATCAGTATCAATAA
>CesiTAS2R5P_NW_004454187.1:14604791-14603924
ATGGTGCCTGCTGCCCTAGGACTGCTGATGCTGGTGGCAGTGCCTGAATTTTTAATTGGCCTGGTTGGAAATGGAGTCCT
TGTGGTCTGGAGTTTTAGAGAATGGGTCAGAAAATTCAAGGGGTCCTTATACAACCTCATTGTCCTGGGCCTGGCTGTCT
GCTGATTTCTCCTGCGTTGGTTGATTATAGTGGATTTAAGCCTGTTTCTGCTTTTCCAGAACAGCCATTGGCTTTGCTAT
CTCTGGGTCCTGGTAAGCCAGGCCAGCCTGTGGTTGCCACTTTACTCGGTGTCTTCTACTGCAAGAAAATCACGACCTTC
GACCTTGTCTACTTGTGGCTGAAGCAGAGGGCCTATTGCCTGAGTCTCTGGGGTACCTCATGATCAGTTTGTTACTTGTA
GCCCACATTGGCTTAAAGCCCTGCAATCCTTCCCGAGGAAACAGCAGCATTCTATATCCCTTTTCAAACTGGCACTCTCT
GTATATATTACGTCTCAGTGCAGGAAGTAGGGTGCCTTTCACGGTGTTTCTTGTTTCCTCTGGGATGCTCATTGTCTCTT
TGTATAGACACCACAGGAAGATGAAGGTCCATACAGCTGGTAGGAGGGATGCTCGGGTCAAGGCTCACATCAGTGTCCTT
CAGTCCTTGGGCTTCTTCCTTGTATTTTACATGGTTTACGTCCTGGCTAGCCCCTTCTTCATCACCTCCAGGTATTCTCC
TGCTAATCTCACCACTGTCTTCATCTCTGAGACACTCATGGCTGCCTATCCTTCTCTTCATTCTGTCATATTGATCATGA
GGAATCCCAGTGTGCAGCAGATTTGTCAGAGAATCTTGTAGAAGACAGTGTGCGCTTGGAGATCCTGG
>CesiTAS2R11BP_NW_004454209.1:743204-742248
ATGTTGAATACGTTGGAGAAAGTTTTCATGATTGTAACAGGTGGAGAATTTGTAATAGGAATTTTAGGGAATGGATTTAT
TGGACTCACAAATTGCATCAGCTGGATTAGAAATCAGAGGTTATGCTTGGTTGCCTTCATTCTTACTACTTTGGCCTTTG
CCAGAATCAGTGAATTATGCCTAACAACTGTCAATTTGTTTTCAGCGTTGCTCTATCAGAAAGTCCTTGGTACTATGAAA
GGAAACAATATCTTTACTAGTATCTGAATATTGGCCAACCACTTGAGCACTTGGTTTGCTACTTGTCTCGCTGTCTTTTA
TTTCCTGAAGATCGCCAATTTCTCCTGTCCCCCTTCCTTTGGCTAAAATGGAGAATTAACAAGGTAGTTTTCTGCTTCTG
CTGTTCTCTGTGCCCTTCCTGCTAATGAAGGAAAACTATATGGCAAACTTTCCTTTGTCATATAGTTTTCATGTCTTCCG

GTATTACGTCCACGAAAACTATGAAAGAAATATGACTGGGTTATTCAATGTGAGTAAAAATAAACATTAAATTACATGAT
AATCTTCATTATTGGGCCCCTCCCTCCTTTCTCTTTTTCCTTAATGTCCTATCTCCTGTTGCTCCTTTCTCTGTGGAGAC
ACATGAAGCACAGTGAGCTCAGCGTCAAGGATTCCAGAGACCCCAGAATGGAGGCCCACTTCAGAGCCTTGAAAACTGTG
TTATTCTTTCTTCTGCTCTTTGCTCTGTACCAAGTTTCCTTTATCATCACAATTTTGAGTTATTTTTCACTACAGAACAA
GCTGTTTGTGATGTTTGGTTATGTGATAGAAATTGTATATCCTTCGGGTTACTCATATGTTGTGATTTTGGGAAACAGCC
AAATGAGGAAAGCCTTCTTGGGGAAACATTGGCACTTGAAGTGTGGCCTGAAAGGAAAGGCGCTCTCAGCTGCATAG
>CesiTAS2R11CP_NW_004454209.1:704811-703856
ATGTTGAATACGTTGGAGAAAGTTTTCATGATTGTAACAGATGGAGAATTTGTAGTAGGCATTTTAGGGAATGGATTTAT
TAGACTCACAAACTGCGTTACCTGGATTAGAAATCAGAAGTTATGCTTGGTTGACTTCATCCTTACCAGTTTGGCCTTCG
CCAGAATCAGTGAATTATGCCTAACAAGTGCCAATTTGCTTTCAGTGCTGGTCCATCAGGAAGTCCTTGATACTATGAAA
AGAAACAATATGTTTACTGGCATCTGGATATTGGGCAATCACTTGAGCACTTGGTTTGCTACTTGTCTTGCTGTCTTTTA
TATCCTGAAGATCAGCAATTGCTCCTATCCCCCTTCCTTTGGCTAAAATGGAGAATTAACAAGGTAGTTTTCATGCTTCT
GCTGTTCTCTGCGCCCTTCCTGCTAATGAAGGAAAACTATAAGGCAAACTTTCCTTTGTCATATAGTTTGGATGTCTTCC
AGTATCATGTACAAAATAAGTATGAAAGAAATATGACAGATTACTCAATGTAAGTAAAAATAATGATTTAAATTACATGA
TACCCTGAATTACTTGGACGCTCCCTCCTTTCTCTCTTTCCTTGATGTCCTATCTCCTGTTTCTCCTTTCTTTGTGGAGA
CACACAAAGCACAGTGAGCTCAGCGTCAAGGATTCCAGAGACCCCAGAATGGAGGCCCGTTTCAGAGCCATGAAATCTGT
GTTTTTCTTTCTTGTGTTCTTTGGTCTGTACCAATTTTCTTTTTTCTTGACATTTTTGCATTATTTTTCACTACAGAACG
AGATGGTTGTGATGTTTGGTTATTCGATAGGAATTCTATATACTTCGGGTCACTCATGTCATGATTTTTGGAGACAGCCA
AATGAGGAAAGCCTTCTTAGGGATTCTCTGGCACCTGAAGTGTGTCCTGAAAGGAAAGGCACTCTCAGCTGTATAG
>CesiTAS2R12EP_NW_004454209.1:651440-650610
ATGGCAAGCACATTGAAGAAGATATTTATGATCCTTTATGCTGGAGAATTCATGATGGGGATTTTGGGAAATGGATTCAT
TGTATTAGTTAACTGTATTGACGGGATCAAGAGCTGGAAGTTCTCCCTCATTGACTTTATTCTCACCTGCTTGGTGATTT
CCAGAATATTTCTGCTGTGCATATTAATTTTATAGATACTGCATCAGTGTCTTCTATTTCCTCAAGATAGCCAACTTTTC
TAATCTTATTTTCCTCTGGATGAAATGGAGAATTCACAAGGTGCTTCTCATTCTTGTGCTGGGGGAAGCCCTCTCTTTCT
GCTTGCTCCTTCTCTTTTAGGAGACAGTAGTTAGGAGCCTGATCAAAAACCAGGGAAACACTGAATGAAATTTGACATGG
AACTCACAGTGAGAAAATATGATTTATTAACTTCTCAAATGCTCCTTAACATAATGTTCCTCATCCCCTTTGTAGTGTCA
CTGGCCTCCCTGCTCCTTTTAATCCTGTCCTCATGGAGCCACACCAGGCAGATGAAGGGCACAGGTTCTAGGGATTCTAG
CACAGAGGCCCATGTGAGAGCCATGAAGTGTATGATTTCACTCCTACTCCTCTTCTTCTTGTACTGTTTGACTCATATTA
TGATAAATTCAGCCTATGCCATTCTAGACACTTTCACAGCAAAGATATTTGTTAATGTGCTAATATTTTTCAGTCCATCT
GGCCATCCATTTCTTCTGTTTTTATGGAACAGCAAATTGAAACAGGCTTCTCTCAGTGTCCTGAGGAAGCTGAAGTGTTG
CCTGAATCTAAGGAAACCTATATCCCCATAA
>CesiTAS2R13P_NW_004454209.1:844578-843673
ATGGTAAGCTCCTTGCATAGCTTCATCTTCATTTTAGTAAATATAGAAATCATAATCGGTATTTTGGGGAATGGATTTAT
AACACTGGTGAACTGTATTGATTGGCTCAAGAAACGAAAGATCTCCTTGGCAGATTGAATCATCACTGCTTTGGCAATCT
CCAGAATTTGTCTGATTTTCATAACAATGGTGATGTGGTTTATAAGGGAGTTTTATCCATCTTTATATGTGAATAGAAAG
AAAACTATACTTATTAGTATTGCCTGGACCGTGGTCCATCATGTTAGCATTTGGCTTGCCACAGGCCTCAGCCTCTTTTA
TTTTCTTAAGATAGCCAATTTTTCAAATCCTGTTTTTCTTTACCTAAAACATAGAGTTGAAATGGTAATTCTGGTATTGT
TTCTGGGAACATTAGTATTCTTGCCTTTAAATCTTGCTATGGTAATGGTACATGTTATTATCCACATACATCCATATGAA
AGAAATATGACGTTAAGTTCTAAAAGGAGTGACATTGAAAACTGTTCAAACTTGATTACATTCATTGTGGGATCCTTCAT
ACCCTTTTCTATATCCCTGAATTTTTTTCTCCTATTAATCTTCTCCCTATGGAAACATCTCAAGAATGTGAAGCACAATG
CAATGGGATTCAAAGACTCCAGCGTCAAGGCCCATATAAGAGCCATGAAAAATGTGATATCTTTCCTCTTACTATATGCT
ATTCATTTCCTGTCTCTTCTTATAACAATTTTCCATGCTGAGATCATGCAGAACAAACTAGCCATTATGCTAGGTCAGGC
TTTTGCACATGTTTATCCTTCAGTCCACTCAGTTATCCTGATTCTGGGAAACGGTAAACTAAGAAAGGCTTCACTTTCTG
TGCTGTGGCAGCTGAGGTGTGGTTGA

>CesiTAS2R14P_NW_004454209.1:861183-860214
ATGGTCAGTGTCTTACAGAGCACACTTACAATCATTCTAAGTGCCGAATTCGTAATTGGAAATTTAGGAAATGGATTCAT
AGCACTCGTGAACTGCGTTGACTGGGTCAAGAGAAAAGAGATCTCTTCAGCTGATCAAATCCTCACTGCTTTGGCAATCT
CCAGAATTGGTCTGCTCTGGTTAGTATCCATAAATTGGTATATATCTGTGTTTTGTAGAATTTTACTCCTGCCTGGAAAA
ACATTAAGAATAAGCAGTATTGGCTGGACAGTGACCAATCATTTCAACAACTGGCTTGCTACAATCCTCAGCATCTTTTA
TTTTCTCAAGATAGCCAATTTTTCTAACTCTATTTTTCTTTACCTAAAGTGGAGAGTTAAAGAGGTGATTTCAGTGATAC
TGCTGGTGACCTCAGTCCTCTTGATGTTTAATATTGCACTGATCAACATGCATATTAATGTCTGGATCAGTGAACATAAA
ATAAACATGACTTGCAGTTCTAGGATGAGTAACTTTGCACAACTTTCCACTCTTACTTTATTCACTAACACTCTGTTTAC
TTTCATACCCTTTGCTGTGTCCCTGGTGATTTTTCTTCTGTTAATCTTCTCCTTGTGGAAACATCTCAAGAAGATGCAGC
ACAATGCCAAAGGCTCCAGAGATGCAAGCATCGAGGCCCACATAAAAGCCATGAAAAGTGTGATTGCTTTCCTGCTACTA
TTTGCCATTTTCTTTCTGTCTCTTTTTGCGTCAATATGGAACTTGACATTTAATTAAAATTAAAAAGTATTGTGTTGCAT
ATTTTAAAACTACATTGGCAAATACAATTTATTTCCAAATATGAAGTATATTTTCCTAATAATGATAAATATAATTCAAG
AATTCTTGTATGTGAAACTAATAGTATAGAAAGTGTAATTGAAATAAAGCAACTTCATAATTCTGAGGAATTTCTAACAA
TTATTTGTAA
>CesiTAS2R18AP_NW_004454209.1:1006252-1005315
ATGTCAGTTGGAATGAAGGTCTCCTTTCTAGTCATGACAATAGGAGAACTCATCTTAGGAATGCTGGGAAAGGGGTTCAT
TGGACTGGTAAACTGCATCAAATGGGTCAAGAATGGGAAGATCTCATCAGGTGATTTCACCTTTACCAGCTTGGCTATGG
CCAGAATCATTCAACTGTTGGTAACACTGTTGGTTCATTTATAATGGGGCTATCTCCACATCTGTATGCCACCAGTAAAT
GAGCAAAAGCGGTTACTATTCTTTGGGTACTAACAGATTGGTTAACTACCTGGTTTGCCGCCTGCCTAAGCATTTTGTTC
TTCCTCAAGATAGCCAATTTCTTCCACTTCTTTTTCATCTGGCTAAAGTGGACAGTGAAGAGAGTGATTCTTGTACATTT
CCTGGAATCTTTCTTCTCATTGTCTGTTAACCTCTTAATGCAGGATGCTCTTAGTGAGTTGTGGATGAATACCCATAGAC
TACATGAAAGACACATGACTTTGCATTTAGATGTAAATAAAATTTCCTATTTTAAAAGCCTTCTTCTTAGCTTGACTTAT
GTTACCCCTTTTCTTCTATCCATCACCTCTTTGATCCTTTTATTTCTGTCCTTGATGAGATACACCAAGAATTTGCAGCT
CAACCTGATGGGAACAGGGGACTCCAGCACAGAGACCCACAAAAGGGCCATGAAAATGGTGACAACCTTCTTCCTCCTCT
TCATCATTGACTTTATTTCCACTCTAATAGCAAGGTGGATCTTCCTTAAGGTACACAGGTATCAGGTTATGATGTTTGTC
ATGGTGATTTCAACTACCTTTCCCTCAGACCACTCATTTATCATAATCTTGAGCAACAGCAAGCTAAGATAGATCGCCTT
AGACTGCTGTAGCATCTTAAATTATCACTTGAGAAAAGCAAAACTTTTAGATTCATAG
>CesiTAS2R18BP_NW_004454209.1:922387-921453
ATGTCATTTCAAACGAAAGTCTCCTTTCTGGTCGTAGCAACAAGCAAACTCATCTTAAGAATGCTTGGAAATCGGTTCAT
TGGGCTACTAAACTGCATTGAATGGATCAAGAATGGGAAGGTCTCTTCAGCTGATTTCATCCTCACCAGCTTGGCTATGG
CCAGAATCATTCAACTGTGGATAACACTATTTGGTTCATTTATAATGGGGCTATCTCCACATCTGCATGCCACCAGTAAA
CTAACAAAAGTGGTTACTATTCTTTGGGCACTAACTGATCACTTTACCTGGTTTGTCACTTACCTAAGCATTTTCTGCTT
CCTTAAGATAGCCAGTTTCTCCTACTTCTTTTTCATCTGGTTGAAGTGGAGAGTGAACGGAGTGGTTCTTGTGCTTTTCC
TGAGTCTTTCTTCTTCTTGTCTGTTAACCTTTTAATGCAGGATGCTCTTAGTGAGTCGTGGATGAATACCCATAGAGTAC
ATGAAAGACATGTGACTTGGCATTTAGATGTAGATAAAATTTTCCCTCTCAAAAGCCTTCTTCTTCCTAGCTTGACCTGT
GTCATCCCCTTTCCTCTGTCCCTGACCTCTCTGCTCCTTTTATATCTGTCTTTGCTGAGACACCAAGAATTTGCAGCTCA
ACCTGATGGGTGTGAGGGCCTCCAGCACAGAGGCCCATAACAGGGCCATGAAAATGATGACAACCTTCCTCCTCCTCTTC
GTCATTTACTTTATTTCCATTCTAGTAATAAGTTGGATCTTCCATAAGGTAGAGAGGTATTAGGTTATGATGTTGGTCAC
GGTGATTTTAACTATCTTTCCCTCAGGCTGCTCATTTACTATAATTTTGGGAAACAGTAAGCTAAGATAGATCACCTGAG
ACTACTGTAGCATCTTAAATTCTCTCTGAGAAAAGCAAAACTTTCAGCTTCATAG
>CesiTAS2R18CP_NW_004454209.1:886112-885170
ATGTCAATAGGAATGAAGGTCTCCTTTCTGGCCGTGGCAGCAGGAGAACTTGTCTTAGGAATACTGGGGAAATGGGTTCA
TTGGACTGGTAAACTGCATCGAAAGTGTCAAGAATGGGAAGATCTCATCAGCTGATTTCACCTTTACCAGCTTGGCTATG
GCCAATGTCATTCAACTGTGGATAACACTATTTGGTTCATTTATAATGGGGCTGTCTCCACATCTGTACCCCACCAGGAA

ACTAGCAAAAGCGGTTACTCTTCTTTGGGCACTAACTGATCATTTAACTACCTGTTTTGCCACCTGCCTAAGCGTTTTCT
GCTTCCTGTCTTAAGACAACCAGTTTCTTCTACTTCTTTTTCATCTGGCTGAAGTGGAGAGTGAACAGAGTGGTTCTTGT
GCTTTTCCTGGGTCTTCCTTCTTATTTCTGTTTACTTCTTAATGCAGGATGCTCTTCGTGAGTTGAGGATGAATACCTA
TAGGGTACATGAAAGACATGTGACTTTGCATTTAGATGTAAATAAAATTTTCTATCTCAAAAGCCTTCTTCTTCTTAGCT
TGACCTGTGTCATCCCCTTTCCTCTGTCCCTGACTTCCTTGCTCCTTTTATGTCCGTCTTTGCTGAGACACCAAGAATTT
GCAGCTCAACCTGATGAGTGTGAGGGCCTCCAGCACAGAGGCCCATAACAGGGCCGTGAAAATGGTGACAACATTCTTCC
TTCTCTTTGTCACTTACTTTACTTCCATTCTAATAACAAGTTGGATCTCCCATAAGGTAGAGAGGTGTTAGGTTATGATG
TCTGTCATGGTGATTTCAACTTTCTTTCTCTCAGGCCACTCATTTATTATAATTTTTGGAAACAGTAAGCTAAGACAGAT
CACCTGAGACTACTGTAGCACCTTAAATTCTCTCTAAGAAAACCAAAATTTTTAGCTTTGTAG
>CesiTAS2R408CP_NW_004454209.1:961187-960169
ATGATAAGTTTCGTACAAAGCATTTTTTTAATCCTCTAATGACAGAATTCCTTCTGGGAAATTTTGCCAATGGCTTCCTA
GCCCTGGTGAACTACATAGACTGCGTCGAGACAAAAGATGTCCTCAGCTGATCAAATTCTCACAGCTCTGGCTGTCTGCA
GAATTGGTTTGCTCTGGGTAATATTAATAAATTGGTATACAACTGTGTTTAATTCAATTTTATACAGTTTAGAAGTAGGA
ACTGTTGTTTATATTGCCTGGACAACAGCCAACCATTTTAGCATTTGGCTTGCTACTAGCCTCAGCATATTTTATTTGCT
CAAGATAGCCAATATCTCCAGCCTTATATTTCTTTACTTGAAGTGGAGAGTTAAAGTGTAGTTCTCATGATACTGTTGGA
GACTTTGTTCTCTTTGGTTTTTCTTGGTACAGTGGTAAGCATAGATGATAATATGCAGAGAAAAGAATATGACTCTTTGG
TTTTTCTTGGTACAGTGGTAAGCATAGATGATAATATGCAGAGAAAAGAACATGAAGTAAACATCACTTGGAAGATCAAA
TTGAGCGACATTATACAACTTTCACATTTGACTGTACTCACCCTAGCAAACTTCTTACCCTTTACTAGGTCCCTGACATC
TTCTCTGCTGTTAATCTTTTCCCTGTGGAAAACTCTCAAGAAGATGCAGCTCAATGGTAAAGGATCCCTAGATCCCAGCA
CCAAGGTCCACCTAAGAGCCATGCAAACTGTGGTCTCCTTTCTCTTGCTATTTGCCGTTTACTTCTGGCTCTAATCATCT
CATTATGGAGTTCTAATAGGTTGCGGAATGAGCTGGCTCTCATGCTTTGCCAGGCTCTTGGAATGCTGTATCCTTCAAGC
CCCTGATTTACCCTGATTTGGGCAAATAGGAAGCTAAAACAGGGTTTTCTGACAGTTTTATGACAGGTGAAGTGCTGGCT
GAAAGAATGGAAACCCTCACCTCCACAGATCAATAAGAGGGGTGTTGTGTATGTTCTAG
>CesiTAS2R408DP_NW_004454209.1:943184-942228
ATGATAAGTTTAAGTTTATTACAAAAATATTAATCCTAATAATGACAGAATTTATTCTGCGAAATTTTGTCAGTGGCCTC
ATAGCAATGGTGAACCACATTAACTGGGTCAAGAGACAAAAGATATCCTCAGCTGATCAAATTCTCACTGCTCTGGGGTC
TGCAGAATTTGATTTCTCAGAGTAATATTAATAAATTGGTATACAACTGTTTAATCCAATTTTATATACTTCAGAAGTAA
GAACTGTTGTTTGTATTGCCTGGACAATAACTGACAATTTTAGCATTTGGCTTGCTACTAGCATCAGCATATTTTATTTG
CTCAAAGATAGCCAATATCTCCAGCCTTATATTTCTTTACCTGAAGTGGAGAGTTAAAGTGTAGTTCTCATGACACTGTT
GAAGCCTATGTTCTCTTTGGTTTTTCATGTTGCAGTGGTAAGCACAGATGATAATACGCAGAAAAATGAAGATGAAGGAA
ACATCACTTGGAGGATCAAATTGAGGGACATTGTGCAACTTTCACATTTGACTGTAGTCACCCTGGCAAACTTCCTACCC
TTGCCTACATCCCTGACATCTTCTCTGTTGTTAATCTGTTCCCCGTGGAAGGCTCTCAAGAAGATGCAGGTGAATGTCAA
AGTATCCCAAGATCCCAGCTCCAATGTGCATATAAGAGCCGTGCAAACTCTCATCTCCTTTCTCTTGCTATTTGCTGTTT
ACTTCCTGGCTCTACTCATCTCATTTTGGAGTTCTAACATGCTGCAGAATGAGCTGGTTCTCATGCTTTGTGAGGGTCTT
GGAATCTGTATCCTTCAAGCCACTTGTTTATCCTGATTTAGGCAAAGAAGAAGCTAAAATGACTTTCTGTCAGTTTTATG
GCAGGTGAAATGCTGGCTGAAAAAATGGAAACCCTCAACTCCATAGATCGATAAGAGGGACTTTGTGTATGTTCTAG
>CesiTAS2R408FP_NW_004454209.1:906174-905226
GTGATAACTTTCCTAAGAAGAATTTTTTCCACCCCAATAACAACAGGATTTATTCTGGGAAATTTTGCCAGTGGCTTCAT
AGCACTGATGAACTGCATTGACTGGGTCAAGAGACAAAAGATGTCCTCAGCTGATCAAATTCTCATGGCTTTGGCAGTCT
GCAGAGATGGATTGCTCTGAGTAATATTAGTAAATTGGTATACAACTGTGTGTAATCCAATTTTATATAGTTTAGAAGTA
AGAACTGTTGTTTATATTGTCTGGGTAATAATCAACCATTTTAGCGTTTGGCTTGCTACTAGCCTCAGCATATTTTATTT
GCTCAAGATAGCCAATATCTCCAGCCTTATTATCTTTACCTGAAGTGGCGAGTTAAAAGTGTGGTTCTCATGATACTGTT
GGAGACTGTGCTCTCTTCGGTTTTTCATGGTGCGGTGGTAAGCATAGATGATAATATTCAGAAAAATGAATAAGAAGGAA
ACATCACTTGGAAGATCGAGTAGAGGGACACTGTACAACTTACAGATTCACTCTAGCAAACTTCATATCTTTTCCTATGT

CACTGACATCTTCTCTGCTGTTAATCTTTTCCCTGTGGAAACATCTCAAGAAGATGCAGCTCAATGGTAAAGGATTCCGA
GATCCAGCACCAAGGTCCATATAAGGGCCATGCAAACTGTGATCTCCTTTCTCTTGCTATTTGCCATTTACTTCCTGGCT
CTAATCATTTCATTTTGGAGTACTAACATGCTGAAGAATGACCTGATTCTCATGGTTTGCCAGCCTCTTAGCATGCTATA
CCCTTTAAGCCACGCATTTATCCTGATTTGGGCAAACAAGAAGCTAAAACAGGACTTACTGTCACTTTTATGGCAGGTGA
AATGCTGACTGAAAGAATGGGAACCCTCAGCTCCATAGATCAATAAGAGGGGCATTGTGTATGTTCTAG
>CesiTAS2R408HP_NW_004454209.1:867278-866325
ATGAGAACTTTACTACAAAGCATTTTTTCTATCCTAATAATGACAGAATTTATTTTGGGAAATTTTGCCAATGGCTTCAT
AGCACTGGTGAACTGCATTGACTGGGTCAAGAGACAAAAGATGTTCTCAGCTGATCAAATTCTCACAGCTCTGGCCGTCT
CCAGAATTTGATTGTTCTGAGTAAAATTAATAAATTGGTATATGATTGTGTTTAATCCAATTTTATATAGTTTAGAAGTA
AGAACTATTATTTATATTGCCTGGACAACAGCCAACCATTTTAGCATTTGGCTTGCTACTAGCCTCAGCATATTTTATTT
ACTCAAGATAGCCAATATCTCCAGCCTCATATTTCTTTACCTGAAGTGGAGAGTTAAAAGTGTAGTTCTCATGATACTGT
TGGAGACTTTGTTCTTTTTGATTTTTCAGGTTGCAGTGGTAAGCATAGACAACAATATGCAGAAAAATGAATATGAAGGA
AACATCACTTGGAAGATAAAATTGAGGGACATTGTACAAATTTCACATTTGATTGTACTCACCCTAGCAAACTTCATACA
TTTTACTACATCCCTGATGTCTTCTCTGCTGTTAATCTTTTCCCTGTGGAAACATCTCAAGAAGATGCAGCTCAAGGATC
CCAAGATCCCAGCCCCAAGTTCCACATAAGAGCCATGCAAACTGTGGTCTCCTTTCTCTTGCTATTTACCATTTACTTCC
TGGCTCTAATCACCTCATTTTGGAGTTCTAACAGGCTGTAGAATGAACTGCTTCTCATGCTTTATGAGGCTCTTGGAATG
CTGTATCCTTCAAGCCTCTCATTTGTTCTGATTTGGGCAAATAAAATGCTAAAATAGGACTTTCTGTCAGTTTTACGGCA
AGTGAAGTGCAGGCTGAAAGAATGGAAACCCTCAGCTCCATAGATCAATAAGAGGGGCATTGTGTACGTTCTAG
>CesiTAS2R408IP_NW_004454209.1:852157-851203
ATGATAAGTTTACTAAGAGCATTTTTTCCAACCTAGTAGTTGCAGAATTTGTTCTAGGAAATTTTGCCAATGGCTTCATA
GCGCTAGTGAATTGCATTGACTGAGTCAAGAGGCAAAAGATGTCCTCAGCTGATTGAATTCTAACTGCCCTGGTTGTCTC
CAGAATTGGTTTGCTCTGGGGGATATTAGTAAATTAGCATTCAATTGTATTTAACCCAGTTTTATATAGTTTAGAAGCAA
GAACTATTAATATCGCCTGGACAATAACCAACCATTTTGGCATCTGGCTTGCTACTAGCCTCAGCATATTTTATTTGCTA
AAGATAGCCAATATCTCCAGTTTTATTTTTCTTCACCTAAAGTGGAAAACTAAAAGTGTAGTTCTCATGATGCTGTTGAG
GACTTTGGTCTTTGGTTTTTCATCTGGTATTGGTAAGCATAGATGACAGTATGTGCATGAATGACTATGAAAGAAACATC
ACTCGGAAGACCAAATTGAGGGACATTTTATACCTTTCAAATACGACTATATTCACAGTAGCAAACTTCATACTCTTTAC
TGCATGCCTGACATCTTTTCTGCTGTTAATCTTTTCCCTATGGAAACATCTCAAGAAGATGCAGCTCAATGGCAAAGAAT
CCGAAGATCCCAGCACCAAGGTCCATGTAAGAACCATGCAAACCGTGGTCTCCTTTCTCTTGCTATTTGCCAGTTAATCC
CTGGCTCTGGTCGTCTCAATTTGGAGTCCTAATAGGCTGCAGAACAGACTGGTGCTCATGCTTTGCCAGGCTATTGGAAT
CATATGTCCTTCAAACCACTCATTTATCCTGATTTGGGGAAATAAGAAGCTAAGACAGGCCTTTCCGTCATTTCTGTGGC
AGCTGAGGTGCTGGCTGAAAAACAAGACATAAGTGGAGCATTATGTGTCTTCCAGCAGAAAACAAACTATGAGTC
>CesiTAS2R41P_NW_004454187.1:12741721-12740792
ATGCAGCCAGGACTCACAGCCCTCTTCATGTTGCTCTTTTCCCTTCTGTTTCTCCTGGGAATCCTGACCAACGGCTTTAT
TGTGCTGGTGCAGAGCAGAGAATGGATGCGGAGTGGGAGGCTGCTTCCCTCTGACGTGATCCTCGTTAGCTTGGGTGCCT
CCCGCTTCTGCCTGCAGTGGGTTGGAATGGTGAAAAACGTCTACTACTTCCTCCATCTGGTCCAGTACTGCAGGGGGTAC
CGCACGGCAGTTCTTTGGTCTACACCGGGACTTCCTGAACTCGGCCACCTTCTGGTTCGGCACCTGGCTCAGTGGCCTCT
TTTGTGGGAAGATTGCTACCTTCACCCACCCCACCTTCCTCTGGCTGAAGTGGAAGTTCCTGGGGTGAGTGCCATGGCTC
CTCCTGGGCTCTCTTCTGATTGCCTACATCGTCATCGTGCTCTTCTTTTGGGGAAACAACGGTATGTATCAAGGATTCTT
CATTAGAAAATTTTCTGGGAACATGACCTACAAGGAGTGGAGCAGGAGGCTGGAAATTTACTATTTCCTGCCCCTGAAAC
TTGTCACGTTGTCACCTCCTTGCTCTATTTTTCTGATCTCGATTGCACTGTTGATTACTTTTCCGAGGAGACACACACAA
AGAATGTGGCACAATGGCCACAGCCTGTAGGACCCCAGCACCCAGGCTCTCACCAGAGAGCTCTGAAGTCAATCATCGCC
TTCCTTGTTCTTTATGCTGTGTCCTTCGTGTCACTAGCCATCGATGCTGCAGTGTTCTTATCCTCAGAGAGTGACTGGAA
CTGGCCATGGCAAATTTTAACTTACCTGTGCATGTCTGTCCATTCCTTTAACCTCATCCTCAGCAACCTCAGGCTTCGAG
GGGCGTTCAGGCAGTTACTTCTGTTGGCCAGAGGCTTTTGGGTGGCCCAG

>CesiTAS2R60P_NW_004454187.1:12772374-12771432
ATGAATGGAGACAACCTGGTTCCAGGACCTCCGGTGACTGATAAGAGAGCCATCACCTTGGCTATCATTTTATTCCTTTT
GTGCCTGGTGGCAATGGTGGGCAATGGCTTCATCACTGCAGCACTGGGCATGGAGTGGTTGCTATGGAGAATGTTATCAC
CTTGTCATGTTATTGGTCAGCCTGTGGGCCTCTTGCTTCTGTCTACGGTGGGTGGTGATGAGTAAGAGCATTTATGTTTT
TCTGTGTCCACTGGCCCTCCCATACTACCCTGTACTTCAGTTCCTAGCCTTCCAGTGGGACTTTTTGAACGCTGTCACCT
TATGGTTCTCCACTTGGTTCAGTGTCTTCTATTGTGCGAAAATTGCAACCTTCACCCACCCTGTCTTCCTCTGAATAAAG
CAGAAGGTGTCTGGGTTGGTTCCATGGATGCTGCTCAGCTCTGTGGGGTTCTCCATCTTGAGCACCATCCTATTTTTCAT
AGGCAACCAGAACTTTTTAAAGAGAGGTCTGCAATCTTGGAATGTTATCAGGAATCCTATAAGGAGATCACAGGAGAAAT
TCCACTTCTTCCGTTTAAAACTTGTTACCTGGGCAGTTCTTACTGTTGTCTTCCTCACTGGCATAGTTTTGTTCATCACA
TCCCCAAGAAGACACACCAAGAAGGCCTTCCTGACTGTCTCAGGCTTTTGCCATCCCAGTGCCCAGGCACACATAAAGGT
TCTCCTGGCTCTCATCCCCCTTGCTAACTTCTTCAATCCCTACTTTCTGTCACTTGTGCTCAATGTTGCAGGTATTTTTC
CGTCTCAGGAATTTAGGTACTGTGTGTGGCAGGCAGTGATTTATCTGTGCTCAGCAGTTCACCCCACCATTATACTCTTG
AGCAACACAAAGCTGAGAGTGGTGCTGGAGAGGGGCTGCTCCTCAAGGCGTGGGGCATCTTGA
>CesiTAS2R62CP_NW_004454187.1:12839067-12838151
ATGCCCTCCTCACCCACGTTGATCTTCACAGTCATCTTTTTCCTGGAGTCGTTGGGTGCAATGTTGCAGAATGGCTTTAT
AGTTGCTGTGCTGGGCAAGGAGTGGGTGCAATGCCGCACACTGCCGCAGGTGACATGACTGTGGCCTGCCTGGCCACCTC
GCCATTCTGCCTGCTTCAGAGACACCACATGGGCTCCTTTAATTTTTATTCTAAACATCCTATTTTAACATCCCCTGGGA
CTTTATCAACACTATCACTTTCTGGCTTACTGCCTGGCTTGCTATCTTCTACTGTGTGAATATTTCATCCTTCTTTCATC
CCATATTCTACTGGCTGAAGTGGAGGATTTCTGGGTCAGTGCCCTGGCTGCTGCTGGGCTCCCTGATCATATCTGGTATG
ACAGTCATCTAATCAGCTGCTGGGCATAGCATTCTTGTGCAGATGATTGCCTCCCAGAGTTCCCATGGAAACTGCATTCT
GGCTGATAGAATATAGACCTACACTGGCAATTTTTTCTGTCTAATATAGTGCTTGTAATGTTGATTCCCTTCCTCCTTTT
CCTAGTGCCTACTCTCTTTCTTAAGTTCTCACTGCCCCGGCACTTGGGGTAGATGAGGGTCCACAGACCTGGCCCATATA
ATCTCAGCACCCAGCCTCACACTATGGCCCTGAAGTCATTTGCCTTCTTCCTTGTCTTCTACCCATTACATTTTCTGTCC
CTGTTTATTGCTGTTAATAAAATCACAACCCTGTGGGATCAGTGGCACTGGGCCTGGGAAGTGGTGGCCTATGCAGGCAT
CTGTCTGCACTCCAGAATCCTGATGCTAAGCAGCCCCAATCTGAGAAAGGACCTGAAGACAATCCTTTGGAAAGCCCTGG
GCAAAAGATGGGTCACCACAAGTATCAATATCAAAAA
>CesiTAS2R62EP_NW_004454187.1:12809689-12808768
ATGTCCTCCTCACTCACATTGATCTTCATGGTCATCTGTTTTGCTTGGGGTCAGTGGCTGCAATGTTGCAGAATGGCGTA
TAGTTGCTGTGCTGGGTAAGGAGTGTGTATGATGCCACACACTGCCAGCAGGTGACGTGATTCTGGCCTGTTTATCTGCC
TACTGGTTCTGACTTCATAGGATGAACTCTCCCGAATAACCTTCTAGCCTCTTATAATTTTTGTTCAAAAATTTACTATT
TCAACATTCCCTGGGACTTTGTCAACATTTTCACTTACTGGCTTACTGCCTGGCTTGCTGACTGCTACCATGTGAAGATT
TCATCCTTCTTTCATCCCATCTTCTACTGGCCAATGTGGAGGATTTCTGTGTCGGTGCCCAGGCTGCTGCTGGGCTCCCT
GATCATATCTCATCTGACAGTCATCCCAGCAGCCATTGGGGATATAATTCTTATGCATACAATTGCCTCCCAGAGTTCCC
ATGGAAACTGCACTCTGGCTGATAGAACACAGACCTTCTATTGGTACATTTTGCTGCCTAATATACTGCTTGTGGTATTG
ATTCCCTCCCTCCTGTTCCCAGTGTCCACCCTCTTGCTCATGTTCTCGCTGCACAGGCACTTGCAGCAGATGAGGGACAA
CAGACCCGGCCCACGTGACCCCACCACCCAGGACCCCACCATGGCCCTAAAGTCATTTGCCTTCCTCTTTGTCTGGTACA
CATTGCATTTCTTGCTCCTGATTATTGTTATTATGAATACGACAACTCTTGACAGTCACTGACACTGTGACTATGTAGGT
GTCTGTCTGCATTCCAGCATCCTGGTGCTAAGCAGCCCCAAGCTGAGAAGTGTCCTGAAATGATGATGCTTTGGAAATCC
CTGGACAAAAGGCAGTTCATTTTGAGTTATCAGTATCTGTAA
>CesiTAS2R62GP_NW_004454187.1:12777800-12776885
ATGCCCTCCCCACCCGTATTGATCTTCATGGTTATCTTTTTCCTGGAGTCACTGCTGCAATGCTGCAGAATGGCTTCATT
GTTGCTGTGCTGGGCAGGGTGTGGGTGCGATGCCGCACATTGCCCACAGGTGATATGATTGTGGCCTATCTGGCTGCCTC
CCAGTTCTGCCTGCATGGGATGGCCCTCCTGAACAACCCCACGGGCTCCCTTGATTTTTGTTCCAAAGTTTACTATTTCA
ACATCCCGTGGGACTTTATCACCAGTCTCAGTTTCTGGCTTACTGCCTGGCTTGCTCTCTTCTACTGTGTCAAAATTTCA

TCCTTCTCTCATCCCATCTTCTTCTGGCTGAAGTGGAGGATTTCTCAATCAGTGCCCAGGCTGCTGCTGGGATCCCTGAT
CATATCTGGTGTGACAGTCATCTCATCTGTCACTGGGCATAGCATTGTTCCCAGAGTTCCCATGGAAATCACACTCTGGC
TGATAGAATATGGACCTTCTCTCAGCCCTTTTTCTATCTGAAGAGGTGTTTGTGTTGTCTATTCCCTTCCTCCTGATCC
TGGTGTCCACCCTCTTGCTCATGTTCTCACTGCGCCGGCACTTGTGGCAGATGAGGGACCACAAACCTGGTCCACGTGAT
CCCAGCACCCAGGCTCACACCATGGCCCTGAAGTCACTTGCCTTCCTCCTTGTCTTCTACACATCACATTTCCTGTCCCT
TATTATTGCTGTTATGAAAATCACAAGCCTTCAGAATCACTGGCACTGGGCCTGGGAAGTGGTGACCTATGCAGGCATCT
GTCTGCACTCCAGCATCCTGGTGCTAAGCAGCCACAAGTTGAGAAAGGCTCTGAAGATGCTGTCTTGGAAAGCCCTGGAG
AAAAGGTGGTTCATCTCAAGTTATCAGTATCATTAA
>CesiTAS2R67P_NW_004454209.1:1041109-1040178
GTAGAATAGAAAATTGAAAATACTTTTCTGATAGTAGCAATAGGAGAATTCCTAATTGGAATGTTGGGGAATGGGTTCAC
TGTACTAGTTAACTGCATTGACTGGGTGAAGAGTCAGAAGCTCTCATCAGCTGACTGCATCCTCACCAGCCTGGCTATCT
CCAGGATCAGTCATCTTTGGATAACACTATTTCATTAATTCGTAATGGTGTTACGGCCACATCTATATGCCATTGATAAC
TAGCAAAATTTGTTGGTATTTTTTGGATATTGACCAATCACCTAGCTACCCAGTTTGCCATCTGTTTTCTACTTCTTTGA
AATAGCCAATTTCTCCCACCCCTGCTTCACCTGGCTCAAGGGGATAATTAGCAGAGTGCTACTTGTGCTTCCACTGGAGT
CTTTATTCCTCCTGTGTTTCAACCTTGAATTAATAGATACATCAAATGGTTTCTGGATTCATGTCTATAAAAGATATAAC
AGAAACTCAGCTTGGTCCTCAGATGTAAGTAAAACTCTGTATCTTAATAGCTTGATTGTTTTCAATTTAATCTACTTATA
TCCCCTTTCTTCTGTCCCTTACCTCAGTGTTCCTTTTACATCTCTCTTTGATGAGACATAGCAGGAATTTGCAGTTGAAC
TCCAGCTCTTAGGGACCTTAGCTCAGAGGCCCAAAAAAAGAACATGAAAATGGTAATGTCTTTCCTCCTCCTCTTCATGG
TTCACTTTTCTTCCACCCTATTAACAGGTTAGGTTTTCCCTATACTGAAAAAACATCAGGACAATTTGGTTGTCATGTTA
ACGTCAACTCTTTTTCCTTCAAGACACTCATTTATCCTAATTTCAGGAAAACTCAAGCTGAAACAAACTGCCTTAGGACT
TCTGTGGTATCTTAATTGCCACCTGAAAAGGGTGAAACCTTTAGCTTCATAG
>CaaeTAS2R1_HG918332.1:2765776-2766666
ATGCTGGAGTGTCACCTTGTCAGCCACCTTGTTTTGACAGTGATACAATCTCTCTTTGGGATTTTAGTAAACGGCATCAT
TCTGATTGTGAACGGTACTGACTTGATCAAGCAGAGAAAGTTGATCCCACTGGATCTCCTTGTTTCCTGCTTGGCGATTT
CCAGGATGGGAATTCAGCTGGTCTTCTTCTACATTAACCTGGCTCTTCTTTCCTTGGTCAAATTCCCTCCAGTTATTGAG
AAGCTTGTAGTTTTCACATTTGTAAATGATTTGGGACTTTGGTTTGCCACCTGGCTCAGTGTCTACTACTGCATCAAGAT
TGCTACCATCGCTCACCCTCTCTTATTCTGGTTGAAGATGAAGATCTCCAAGCTGGTTCCTTGGCTGATTCTTGTGTCCC
TGCTGTATGCATGTAGTACTTCTGCTGTGCATGTCAAATATAAGTGGGCATTTTATGGAGAAGGCTTCCTGGACCTTTTC
TTCCCAAATGTAACAACTCACATCAAACTAACCCCTACTTTACAGTCTGCCTTTCTGCTTGCTGAGTTTGCATTGCCGTT
TTTCATCTTCCTGATTTCTTCTCTGCTCTTGATATTTTCCCTGGGGAGACATGCCTGGCAGGTGAGAAACACATGGACAG
GCCCCAGAAACCCTCACACACATGCATACCTCAGGGCCTTTCACTCCATCCTGTCCTTCCTGGCCCTCTATCTCTGCCAC
TACCTGATCATTGCTTTGATCTTTTTTCAAATTTTTAACCTTAGAAGCTTCCTATTTCTGTTCTGCACCTTCATGGTTGG
TTCATACCACTCCATCCACTCTATTACTTTAATTTTAGGAAACCCCAAAATGAAACAAAATGCAAAGGCGTTGCTCCTCC
TCAGAAAGTGA
>CaaeTAS2R2_HG919394.1:421090-421998
ATGATCTCTTTGTCAGTTATTCCACATGTTATCATCATGTCTGCAGAATTTATTACAGGGGTTACAGTAAATGGATTTCT
TATAATCATCAACTGTAATGAATTGGTCAAAAGCAGAAAGCTAACACCCATGCAACTCCTGTTCATATGTATCGGGATGT
CTAGATTTGGTCTACAGACTGTGTTAATGGTACAAGGTTTTTCTCAGTGTTCTTTCCATGCTTTTATAGCACAAAAATC
TATGGCACACCAATGCTGCTCTTTTGGATGTTTTTCAGCTCTGTCAGTCTCTGGTTTGCCACCTGTCTCTCTTTATTTTA
CTGCCTCAAGGTAACAGGCTTCACCCAGTGCTGTTTTCTTTGGCTGAAAGTCAGGATCTCAAAGTTAATGCCTTGGATGC
TCCTGGGAAGCCTGCTGACCTCTATGAACATTGCAGCTCTGTGTGTCAAGGTGGATTACCCTAAAATTGTGGATATTGAT
GTCCTCGGGAATGCCACAGCTAAGAGGATTAAACTCAACACAAAGCAAATTAATGAAGTTCTTCTCGTCAACTTGGCATT
ACTATTTCCTCTGACCATATTTATAATATGCACTGTTATATTATTCATTTCTCTCTACAAGCACACTCATCGGATGCAAA
ATGGACCTCTTGGTTTCAGAAACACCAGGACTGAAGCCCATATTAATGCATTAAGAACAGTGATAACATTCTTTTGCTTC

TTTATTTCTTACTTTGGTGCCTTCATGGCAAATATGACATTCAATATTCCTTATGGAAGTCATTGCTTCTTTGTGGTGAA
GGATATTATGGCAGCATATCCCTCTGGTCATTCGGTTATAATGATCTGGAGTAATTCTAAGTTCCAGCAACCAATCAGGA
GACTTCTCTGCCTAAGAAGGAGTCAGTGA
>CaaeTAS2R3_HG918414.1:2650575-2651525
ATGTTGAGACTCAGCAATTTGGGGTTTCTGGTTCTGACCGCCATTCAGTTCATCCTGGGAATGCTGGGGAATGGTTTCAT
AGGGTGGGTCAATGGCAGCAGCTGGTTCAAGAGCAAGAGGATCTCTTTGCATGACTTCATTATCACTAACCTGGCTGTCT
CCAGGATTGTTTTGCTGTGGATTCTCTTGATTGATGGTGTTTTACTGGTGTTCTCTCCCAAACTACACGATGAAGGGATA
ATCATGCAAATTATTGATGTGTTCTGGACATTTACAAACCATCTGAGCATTTGGCTTACCACCTGTGTCAGTGTCTTCTA
CTGCCTGAAAGTGGCCAGTTTCTCCCATCCTATGTTCCTCTGGCTCAAATGGAGAGTTTCCAGGGTGGTTGTATGGATGC
TGTTGAGTACCCTGCTGTTATCATGTTGCAGTGCCATCTCTCTGATCCGAGAATTTAAGATCTATTCTGTTCTCGGTGGA
ATTGATAGAACCAGGAATATGACTGAGCTCTTTAGAAGGAAGGAAAAAGAATACAAACTGATCCATGTTCTTGGGACTCT
GTGGGACCTCCCTCCCCTAGTCGTATCTCTGATCTCCTACTTTCTGCTTATCCTCTCCCTCGGGAGGCACGTGCGGCAGA
TGCATCAAGACTGTGGCAGCTCCAGAGATCCCAGTACCGAGGCCCACAGGAGGGCCATCAGAGTCATCCTCTCCTTCCTC
TTCCTCTTCCTACTCTACTATCTTTCCTTTTCTGTTTTAACATCCAGTTATTTCCTACCAGCAACTAAGATGATTGCGAA
GATTGGAGAAGTAATTACAATGTTCTATCTTGCTGGCCACTCTTATGTTCTCATTCTGGGAAATAGCAAGCTGAAGCAGA
TGTTTGTGGCGATGCTTCGGTGTGAGCCTGGTTGTCTGAAGCCTGGATCCAAGGGATCTGTTTATCCATAG
>CaaeTAS2R4_HG918414.1:2663932-2664822
ATGCTTCGGATAGTCTTTTTTTCTTCTATCGTTGTCTCTGAAATTTTAACTTTTGTAGGACTCATTGTGAATCTCTTCAT
TGTAGTGGTCAGTTACAAGACTTGCATCAAAAGCCACAGGATCTCTTCTTCTGACAGACTCCTGTTCAGTTTGGGCATCA
CCAGATTTTTTATACTGTTGCTGAATGTTGTTGTCATCATCTCTCCAAATGTGGAAAGGTCAGTCTCCTTATCCTCTTTC
TTCCTGTCATGTTGGATGTTTTTGGACTCTAGTAGTCTTTGGTTTGTAACCTTGCTCAACGTCTTGTATTGTGTGAAGAT
TGCTAACTACCAACACTCCGTGTTTCTCCTGCTGAAACGAAATCTCTCCACCAGGATGCCCCGGCTGCTGCTGGTCTGTA
TGCTCCTTTCTGTCTTCACCACTCTCCTGTATGTTATGCTCAGACAGTTGGCACCCCGTCTTGAATTTGTGACTGTGAGA
AATGGCACAGTATTTGACATCAATGAGGGACTCCTGTCTTTGGTGACTCCTTTGGTTTTGAGCTCATTTCTCCAGTTCAT
CATTAATGTGACCTCTGCTTCTTTGTTAATCAATTCCTTGAAGAGACATATACGGAAGATGCAGAGAAGTGCCACTGTTC
TTTGGAATCCCCAGACTGAAGCTCATGTGGGTGCAATGAAGCTGATGATCTGTTTCCTCATACTCTACATTCCATATTCA
GTTGCTACCTTGCTCCATTATCTCCCTTCTTCTATAGGGATGGATTTGAGAACCAAGTCTATTTATGTTATTATGTCCAC
CATTTACCCTCCAGGACATTCTCTTCTTATTATTCTCACACATCCTAAACTGAAAACAAAAGCAAAGAATATTCTTTGTT
TCAGTAAATAG
>CaaeTAS2R7_HG918378.1:3041736-3042674
ATGTCAAGTGAAGTGCAGGGTATCTTAATGCTCATAGCAGCTGGGGAATTTTCACTGGGGATCTTAGGGAACGCATTCAT
TGGACTGGTAAACTGTGTGGACTGGATCAAGCACAAGAAGATTGCCTCCATTGATTTAATCCTCACAAGCCTGGCCATCT
CCAGAATTTCTCTCTTATGCATAATACTATTGGATTGTAATATATTGGTCCTGTACCCAGATGTCTATACTGGTGGTAAA
CAAATGAGAATCATTGACTACTTCTGGACATTAACCAACCATTTAAGTGTCTGGTTTGCCACCTGCCTCAGCATTTTCTA
TTTCCTCAAGATAGCAAATTTCTTCCATCCATTTTTCCTCTGGATGAAGTGGAGACTTGACAGTGCAATTCCTAGGATCC
TGCTGGGGTGTTTGGTCTTCTCGGTGTTCATTAGCCTTCCTGTCATTAACAATTTGGATGATGATTTCAGGCATTGTGTC
AAGATGAAATTGAAAACAAATATAAGTCGGAGATGCAGAGTACATAAAGCTCAGCATGCTTCCATCAAGATACGTCTCAA
TCTGTTGACACTATTTCCCTTTTCTGTGTCTCTGATTTCATTTCTCCTCCTGATCCTCTCCCTGTTCAGACACACCAGAC
GAATGCAGCTCCGTGCCCCGGGGAGCAGAGATCCCAGCACGGAAGCTCACGTGAGAGCCATGAAGGCTGTCATCTCCTTC
CTCCTCCTTTTCATTGCCTACTACTTGGCCTATCTTGTGGCCACGTCCAGCTACTTTATGCCAGAGACTGAATTAGCTGT
GATCGTTGGTGAGTTGATAGCTTTAATCTGTCCATCAAGCCATTCACTCTTCCTAATTCTAGAGAACAAAAAATTAAGAC
AAGCATCTCTAAGGGTGCTATGGAAGGTAAAATATATCCTACGAAGAAGGAATTGCTAA
>CaaeTAS2R10A_HG918378.1:3012038-3012937
ATGCTGAGTATAGTAGAAGGCCTCCTCCTTTTTGTAGCAGTTAGTGAGTCAGTATTGGGGGTTTTAGGGAATGGGTTTAT

TGGACTAGTAAACTGCATTAACTGTGTGAAAAATAAGAAGATCTCTACACTCAGCCTTATTCTCACTGGCTTAGCCTCTT
CCAGATTTTGCCTGATATGGATAATAACTACAGATGCATATGTGAGATTGTTTTCTCCAGATATGTATTTGTCTGGTGAT
CTAAGTCAATATATAGCTTACTTATGGATAATTATGAATCAATCAAGTGTCTGGTTTGCCACCAGCCTCAGCATCTTCTA
CTTCCTGAAGATAGCCAACTTTTCCCACTGCATTTTTCTCTGGCTGAAGGGTCACATCAATAAGATCCTTCTTCTTCTAA
TGGGATGTTTGCCCATTTCATGGTTATTTACTTTTCCAAACATTACAATGCCTTTTATTAATAATATTATGAAGAACAGA
AACACAACCGGGTTGATCACCATGCAGAAAAGTGAATACTTTATAAATCAGATTTTGTTCAGTATTGGAACACTCCTTGT
CTTTATACTGTGCCTGATTACATGTTTCTTATTAATCACTTCCCTTTGGAAGCACAACAGGAGGATGCAATTGAATGGCA
CAGGATTCAGAGACCCCAGTAGAGAAGCACATATCAAAGCAATGAAGATCTTGGTGTCTTTTATCATCCTCTTTATCCTG
TATTTTGTAGGCACTGCCATACAAATATCAATTGATACTATGCCTAAAAACAAACTGCTGTATATTTTTGGTATGACAAC
CACTATCCTCTATCCCTGTGGACACTCATTTATCCTAATTCTTGGAAACAGCAAGCTTAAGCAAGCCTCTCTGAGGGTAC
TGAAGCTATTAAAGTGCTAG
>CaaeTAS2R10B_HG918378.1:2980382-2981311
ATGCTGAGTATAGTGGAAGGCCTCCTCATTTATGTAGCAGTTAGTGAATCAGTATTGGGGGTCTTAGGGAATGGATTTAT
TGGAGTTGTAAGCTGCATTGATTGTGTGAAATGCAAGAAGATCCCTACTGTCAGCCTTATTCTCACTGGCTTAGCTTCTT
CCAGATTTTGCCTGATATGGATAATAATTACAGATGCATATGTGAGGATGTTTTTTCCAGATACATATTTGTCTGGTAAT
CTAAGTCAAAATATAGCTCACTTTTGGATAATTATGAATCAATCAAGTATCTGGTTTGCCACCAGCCTCAACATCTTCTA
TTTCCTGAAGATAGCCAATTATTCCCACTGCATTTTTCTCTGGCTGAAGGGTCACATCAACAGGGTTCTTCTCCTTTTCA
TGGGGTCCTTGCTTATTTCATGGTTATTTGCTTTTCCAAGCATTGCAAAGCCTAGTATTAATGATATTATGAAGAACAGA
AGCTCAACCTGGCTGATTGCCCTGCATAAAAAGGAATACTTGACAAATCATATTCTGCTCAATATTGGAGTCATTCTTGT
CTTTGTGCTATGCCTGATTACATGTTTCTTATTAATCACTTCCCTTTGGAGACACAACAGAAAGATGCAATTGAATGCCA
CAGGATTCAGAGATCCCAGCACTGAAGCACATATCAAAGCAATGAAGACTTTGGTGTCTTTTATCATCCTCTTTATCTTG
TATTTTGTAGGCACTGCCATACAAATATCAGGTAGTACTATACCTGAAAACAACCTGTTGCTCATTATTGGTATAACAAC
CAGACTCCTCTATCCCTGTGGACACTCATTGATCCTAATTCTAGGAAACAGGAAGCTGAAGCAAGACTTTTTGAGGGTAC
TGAAGCCATTAAAGTGCTGGGGAAAAGAGAAACTTCTTAGAATTCCATGA
>CaaeTAS2R11_HG918378.1:2948997-2949941
ATGTTGAATATATTGGAGAAAGTTTTCATGGTTGTGACTGGTGTGGAATTTATAATAGGAATTTTAGGGAATGGATTTAT
TGGACTCACAATTTGCATTGCTTGGATTAGAAATCAGAAGTTGAGCTTGGTTGACTTCATTCTTACTAGTTTGGCCTTTG
CCAGAATCAGTCAATTATGGATAACCGCTGTCATGTTTTTTTCAATGATGTTCTATCAGGCAGGCTTTGGTACTGTGGGA
AGAAAATATATCTTTTTTTGTATCTGGATACTGGCCAGTCACTCAAGCACTTGGCTTGCTACTTGCCTTGCTGTCTTTTA
TTTCCTGAAGATCGCCAGTTTCTCCCATCCTCTTTTCCTTTGGCTAAAATGGAGAATTAACAAGGTTGTTTTTATGTTTC
CACTGGTATCTGTGCCCTTCCTAGTCATAAGTTTTCCTTGGCCATACACTGTTGATATCTTCTGGTGTCATGTCCGAAAG
ATGCATGAGAGAAATATGACTGAATTATGCAATGTGAATGAATATAAAAATTTAAATTTTATGATTATCTACAGTGTGGT
GTCCCTCCCACCCTTCTTCCTTTCCCTGATTTCCTTTCTCCTGTTGCTCCATTCTTTGTGGAAACACAAGAAGAACATTG
CACACACTGCCAGGGATTCCAGAGACCCCCATGTGGAGGCCCATTTCAGAGCCATGAAAACTGTGTTTTTCTTTCTCATG
CTCTTTGTCCTGTACCAATTTGGCCTTTTCATGACATTTGGGGGACATATTTTCCTACAGAACAAGCTGGTTGTGATGTT
TGGTTATCTGTTAGAAATGCTGTATCCTTCAAGTCATTCATATGTTTTAATTTTTGGAAACAGCCAAATGAGGACATTCT
TCTTGGTGATTCTTAGGCACCTGAAGTGTGGCCTGAAAGGAAGGCACTGTTGGCTGCGTAGGTAG
>CaaeAST2R12_HG918378.1:2936346-2937257
ATGGAGAGAACACTGAACAATATACTTATGATCATTTCTGCTGGAGAATTCTTACTGGGTATTTTGGGAAATGGATTCAT
TGTTCTGGTTAACTGTATTGATTGGATCAGGAGCAGGAAGTTCTCCCTGATTGACTTTATTCTCACCTGCTTGGCTATTT
CCAGAATATTTGTGCTGTGCATAATGATTTCAAGTACAGGTTTATATGTAATCTCTGAGGAAATACAGTACAACAAGAAT
CTCCTGATAAATTTGGGGTTCCTCTGGACAGGATCCAATTATTTCTCCATAGCCTGCACCACCTGCATCAGTGTCTTCTA
TCTCCTCAGAATAGCTAACTTTTCTAATTTCCTTTTCCTCTGGATGAAACGGAGAATTCACAAGGTGCTTCTCATTATTG
CACTGGGGGCTGTCTTCTCTTTCTGCTTGTGCCTTCTTCAAAAGAATATGGCAGTTGAAATCCTGTTCCAAAACCAGGTA

AACAGCAAAAAAAATGTGACATTGGACTTTCTAATGATAAGATACGATTTGTTCCTTACCATAATGTTCCTCATCCCCTT
TGTAGTGTCCCTGGCCTCCTTTCTCCTTTTAATCCTCTCCTTATGTGGTCATCTCAGGCATATGAAGGGTGTAGACTGTA
GCTCGGAAGCCCATGTGAGAGCCCTGAAGGCTATGATTTCATTCTTGCTCCTCTTCATTCTACACTATTTGAGCAATATT
ATGACAATGTGGGCCAATCACATTCTCGGTAGTTTTGTGGCAAAGATTTTTGTGAACATGCTGTTATTTTTCTGTCCTTC
TGGCCACCCTTTGCTTCTGATTTTGTGGAACAGCAAATTGAAAAAGGCTTCACTCTGTGTCCTAAGGAAGCTGAGGGGTT
ACATGAATCTAAGAAAACTCTTCCAAAAATAA
>CaaeTAS2R16_HG919291.1:695187-694282
ATGATAACCAGCCAACTCTCTGTCTTCTTCATGCTCATCTATATGCTCGAGTTCTTGACAATAACTGGGCAGAGCAGCCT
GATTGTCATAGTGCTGGGCAGAGAGTGGGTGCAGACTCAAAGGCTGCCGCCTGTGGACATGATTCTCGCCAGCCTGGGCA
TCTGCCGCTTCTGTCAACTGTGGTCATCGATGCTGCACAACTTCGGCTCCCACTTCCACCTTAATTACAATTTTTGGTGT
TTCGGGATCATCTGGCAATTTACTAACATCCTTTCCTTCTGGTTGACCAGCTTGCTTGCTGTCTTCTACTGTGTCAAAGT
CTCCTTCTTCAGCCACCCCATCTTCCTCTGGATGAAGTGGAGAATTGTGAGATGGGTTCCTCGGCTGCTGCTGGGCTCCC
TGCTGGTTTCTTGTGTGTCTACCATCTTTTCAGCGACTAGTTATTACATCATCATTCAATTCATCTCCATGAAGGATTTC
CCTAGAAACAGCACCATGCTTGAGAGACTGGAGGCGTTCCTGTGGGATTTTTCCACACTGCGGAAAGTGGTCGTATTGGT
TATTCCTTTCCTCCTGTTCCTGGCCTCCACAGTCTTGCTCATGGCCTTATTATCCCGACATCTGAAGCAGATGAAAGACC
TTCACACAGGCCGCCCCATCTCCAGCCCGGAAGCTCACTCTGCCGCCCTGAGGTCTCTTGGCATCTTCCTCATCTTGTTC
ACCTTTTATTTTCTGACCGTGCTCGTCTCCATCTTGGATGTCCTATTTAATAAAGAGTCTTGGTTCTGGGCCTGGGAAGC
TATCATCTATGCATTAGTCTCTATTCATTCTACTTTACTAATGCTGAGCAGTGCCAAACTGAAAAGAGTTTTAAAGGCAA
GGTGCTGGAGCCTAGAAGCTGCCTGA
>CaaeTAS2R408A_HG918378.1:2881321-2882238
ATGACAACTTTAGTATCGAGCATTCTTTCCATTCTAACGGTGACAGAATTTGTTTTGGGAAATTTTGTGAATGGTTTCAT
AGCACTGGTGAACTGCAATGACTGGGTCAGGAAACAAAAGATCTCCTCAGCTGATGGGATTCTCACTGCTCTGGCAGTCT
GCAGAATTGTTTTGCTCTGGACAATATTAATAAATTGGTATGCAACTATGTATAATCCAGCTCTATATAGTTTAAGAACT
GTTATCCGTGTTGCCTGGACAGTAAGCAACCATTTTAGTAACTGGCTTGCTACTAGCCTCAGTATATTTTACTTGTTCAA
GATAGCTAATTTCTCCAGCTTAATTTTTCTTCACCTGAAGTGGAGAGTTAAAAGTGTAGTTCTCATGATGATGTTGGGGA
CTTCAGTGATTTTGATTTTTCAGGTTGCTGTGTTAGGTATAGATGAGACTATTCAGACAAGTGAATATGAAAGAAACATC
ACTGAGAAGACCAAATTAAGGGACTTTTTACACCTTTCAAATATGACCCTGCTCACACTAACAAACTTCATACCCTTCAC
TATGTCCCTGACATCTTTTCTGCTGCTAATCTTTTTCCTGTGGAAACATCTCAGGAAGATGCAGCTCAACGGCAAAAGAT
CCCAAGATGCCAGCACCAAGGTCCACACAAAAGCCATGCAAACTGTCATCTCCTTTCTTTTCCTGTTTGCCACTTATATC
CTGACTGTAATTTTAACAATTTGGAATTCTAATGAGCTGCGGAAGGAACCGGTCCAAATGCTTTTCCAGGCCCTTGCAAT
CACCTATCCTTCAATGCACTCATTTATCCTGATTTGGACAAACAGGAACTTAACACAGACCTTTCTGTCATTTCTATGGC
AGCCAAGATGCTGGCTAAAAGTAAGAGGAACTAGGTAG
>CaaeTAS2R38_HG918414.1:2822029-2821022
ATGGTGACTCTGACTCACATCGCATCTGTGCCCTCTGAAGTCAGGAATGCATTTCTGTTCTTTTCAGTCCTGGAGTTTGC
AGTAGGGATCCTGGTCAACGCCTTCATTTTCTTGGTGAATTTCCGGGACCTGGTGAGGAGGCAGCCACTGAGCCACTGTG
ATCTTGTCCTGTTGAGTCTCAGCCTCACCCGGCTTGTCCTGCACGGGCTGCTCTTTCTGAAGGCCATCCAGCTTACTCAT
TTCCAGCGAATAAGAGACCCACTGAGCTTCAGCTACCAGACCATCATCGTGCTCTGGATGATCGTCCACCAAGCCGGCCT
CTGGCTCACCACGTGCCTTAGTCTCCTTTACTGCTCCAAGATTGTCCGTTTCTCTCACGCCTTCCTGCTCCGTGCAGCAA
GCTGGATCTCCAGAAAGATCCCCCAGATGCTTCTGGGTGCTGTGGTTCTCTCCTGTGTCTGCACTCTTCTCTGCTTATGG
AACTTTTTTAGTGGATCTCGTTTCTCAGCTGTAACTAGGCTACTCACGAATAACAGTACTGAACTCAATTTGAACATTGC
AAAACTCAGTTTCTTTCATTCCTTCCTCTTCTGCAGCCTGGCGTCCATCCCTTCTTTCTTGCTTTTCCTGGTTTCCTCTG
GGATGCTAGTGTTCTCCCTGGGGAGGCACATGAGGATGATGAGGGCTGAAACCAGAGGCTCTCGGGACCCCAGCCTGGAG
GCTCACACCCGGGCACTCAGGTCTCTCGTCTCTTTCTTCTGCCTGTATGTGCTGTCACTCTCCGCTGCCTTAGTCTCGGT
GCCGTTGCTGACGCTGTGGCACAGCAAGGTTGGGGTGATGGTCTGCATAGGGATAATGGCAGCCTGTCCCTCGGGACATG

CAGTCATCCTGATCTCAGGGAATGCCAAGCTGAGGAGGGCTATGGACACCATTCTGCTTTGGGCAAAGAGCAGTTTCAGG
GTAAGGATGGACCACAAGGCAGATCCGAGGACACCAGATCTGTGTTGA
>CaaeTAS2R39_HG919221.1:167786-168832
ATGACAAGTGGGAGCTATCACAGACCCGCACATCAAGCGCTAAGGAGCCGTTTTCCTCCAGACATCGAAGAAGAGCAACC
ACTCAGGATGATCCAAACCTGCAGTTCCTCAGAAAAGGATCTGTCACCATCTCTTGTCACTTTGATGTTAATAATTATCG
GCACGGAATGCATCCTTGGTATTCTCGCAAATGGGTTCATTGCAGCGATAAACACAGCTGAATGGATTCACAATAAGGTA
CTCTCCACCAGTGGCAAGATCTTGCTTTTCCTGGGTGTATCCAGAATAGTTCTACAAAGCTTCATGATGCTAGAACTTAC
CTTAAGCTCAACATCCCCACAGTTTTATAATGACGACATCACGTATCACACATTCAGAGGATGTTTCATGTTCTTAAATC
ATTGCAGCCTCTGGTTTGCTGCCTGGCTCAGTGTCTTCTACTTCGTGAAGGTGGCGGATTTCTCCTACCCCCTTTTCCTC
AAGCTGAAGTGGAGAATTTCCGGACTGATGCCCTGGCTCCTGCAGCTTTCAGTGTTTGTTTCCTTGGGCCAGAGTGTGCT
CTTCTTCCAAAACATCTATACTATGAATTGTAACAATCTTTTTCTCTCCCCTCCTTCAACTCCACTAAGAAAAAGTCCT
TCTCGGAGGCCACTGTGATCAACCTGGTTCTTTTCCTTAACCTGGGGATCTTCATCCCTCTGATCATGTTTATCCTGGCA
GCCACCCTGCTGATCATCTCTCTCAAAAGACACATCTTCCACATGAAAAGCAATGCCACTGGCTCCAGAGACCCCAGCAC
GGAGGCTCACCTGGGGGCCATCAGAGCTATCAGCTACTTTCTCATTCTCTATATTTTCCAAGTACTTGCTCTCTTTCTCT
ACATGTCCAACTTCTTTGACATCAATAGTCCCTTGAATATTTTGTGCAAAATCATCATGGCTACCTACCCTGTGGCCCAT
TCCATTCTACTGATTCAGGACAACCCTGGGCTGAAAAGAGCCTGGAAGAGGCTTCAGGCTCAAGTCCACCTTTATTTTAA
AAAGTAG
>CaaeTAS2R41_HG919221.1:472424-473359
ATGCACCCAGAATTCACAGTCCTCTTCATGCTGCTCTTTGTCCTGCTGTGTATCCTGGGCCTTCTGGCCAATGGCTTCAT
TGTGCTGGTGCTGAGCAGAGAATGGGTGCAACGTGGGAGGCTGCTCCCCTCTGATCTGATCCTCTTTAGCTTGGGACTCT
CCCGCTTCTGCCTGCAGTGGGTTGGAATGGGGAATAACTTCTACTATTTCCTGCATCTGGTCGACTACTGCAGCGGTCCC
GCCGGGCAGTTCTTCGGTCTACCCTGGGTCTTCCTCAACTCCGTCACTTCCTGGTTTGGCTCCTGGCTCAGCGTCCTCTT
CTGCATGAAGATTGCTAACTTTACCCACCCTGCCTTCCTCTGGCCAAAGTGGAGGTTCCCCAGGTGGGTGCCCTGGCTTT
TGCTGGGCTCTCTCCTCACCTCCTTCACTGTCACCCTGCTTTTTTTTTCAGGGAACCACGCTTTGTATAAAGGGTCCTTC
ACTAGAAAACCTTTCAGGAACATGACCTATCATCAATGGAGCAGGATTCTGGAAATGTACTATTTCCTGCCCCTGAAAAT
GATCACTCTTTCAGTTCCTGGCTCTGTTTTTCTGGTCTCGATTGCTCTGTTGATTCACTCTCTGAGGAGACACGCATGGA
GGATGCAGCACAGTGGTCACAGCCTGCAGGATCCCAGTGGCCAGGCTCACACCAGAGCTCTGAAGTCACTAGTCTGCTTC
CTTGTTCTTTATATTCTGTCTTTCGTGTCCCTGATCATTGATGCTGCAGGGTTCTGCTCCTCAGAGAGTGACTGGTACTG
GCCATGGCAAATTTTAGTCTACTCGTGCACGTCCATCCATCCCTTTATCCTCATCCTTGGCAACCTCAGGCTTCGAGGGG
CATTTGGGCAGCTGATTTTGTTGGCCAGGGGCTTCTGGATGGCTGAGGTGGTGTGA
>CaaeTAS2R42_HG918378.1:2822237-2823166
ATGTTCCCTGGGTTGAGTACAGTATTTCTGATACTGTCAGGAGTGGAATTCTTAATCGGAATTCTAGGCAATGTGTTCAT
TGGACTGGTACTCTGCTCTGAATGTGTTAAGAACCAAAAGACATCTTTATTTGACTTCATCCTCACTGGCTTGGCTGTCT
CCAGAATCAGTCAACTGTTGGTGTTTTTTGTGGAATCACTTATAATGGGACTAGAACCACAGGTATTTGCCATTTTTAAA
CTAGCAAAGCCCATTGCTTTACTTTGGAGAATATCTAATCATTTGACTACCTGGCTTGTCACCTGCCTAAGTATTTTCTA
TCTCCTTAAGATAGCTCATTTCTCCCACTCTCTTTTTTTCTGGCTGAAGTGGAGAATGAACAGCGTCATCCTTGTGATAC
TTGCATTTTCTTTGGTCTTTCTGATTTTGGACATTCTTTTGCTAGAAACATTTAATGATCTCTTCTGGAATTTAATAAAT
GAAGGCAATTTGACTTTAGTTGAAAGTAAAACTCATTATATTAAAAGCGAGAGTCTTCTTAGTTTCTCCTATTTCATTCC
TATTGTTCTGTCCCTGCTCTCATTGTTTTTTTTATTTCTGTCCTTGGTGAAACACACCAGAAATTTGCATCTCAATTTTA
TGGGTTCCAGGGACTTCAGCACAAAGGCCCATAAAAGAGCCATGAAAATGGTGACGTCATTCCTGCTCCTTATCATGGTT
CATTTTCTTTTTACACAATTGGCAAATTGGATGTTTCATAGGTTTTTGGACAATAAGTTCACAAAGTTCATCATGTTAGC
ACTATATGTCTTTCCTTCAGGCCACTCGTTCATGTTGATTCTGGGAAACAACCAGTTAAGACAGATAGCCTTGAAGGTAC
TGAAGCATCTTAAAAGCTCCTTGAAAAGACAAAATCCATTGGCTTTATAG
>CaaeTAS2R60_HG919221.1:443842-444795

ATGAGGGGAGAGGACGTGGTTCCAGGACCTCAGGTGGTTGATAAGACAGCCCTCATCTGCGTTGTTATTTTATTCCTTTT
GTTCCTGGTGGCATTGGTAGGTAATGGCTTAATCATCGCGGCACTGGGCAGCGAGTGGCTGCTGCGGAGAACGTTGTCAC
CCTGCGATAAGTTATTGGTCAGCCTGGGGACCTCTCGCTTCTGCCTGCAATGGGTGGTAATTAGTAAGAACATTTACATT
TTCCTGAATCCAACGACCTTCCTTTATAGCCCTGTGTTCCAGCTCCTGGCCGTTCAGTGGGACTTCTTGAACTCGGCAAC
ACTGTGGTTCTCCACCTGGCTCAGTGTCTTCTACTGTGTGAAAATCGCAACCTTCACCCACCCTGTCTTCCTCTGGCTAA
AGCGGAATGTATCTGGGTTGGTTCCTTGGATGCTACTCAGCTCTCTGGGGTTCTCTACCTTTACCACCGTTCTATTTTTC
ATAGGCAACCAGAGAATGTATCAGAACTATTTAAGGAGGGGTCTGCAATCTTGGAATGTCACTAGGAATGCTGTGAGAAT
GTATGAGAGGTTCTACCTCTTCCCTTTGAAAATTGTTACCTGGACCGTCCCTACTGTTGTCTTTATTGCGGGCACGGTTT
TGCTCATTACATCTCTGGGAAGACACACCAAGAAGGTCTTCTTCTCCATCTCAGGCTTTCACAGTTCTAGTGCCCAGGCA
CACATCAAGGCCCTCTTGGCTTTTCTCTCCTTTGCTATCTTCTTCACTTCCTCTTTTCTGTCACTGGTTCTCACTGCCTC
AGGTATGTTTCCCTTTCGGGAGTTCGGGTTCTGGATATGGCAGATTGTGATTTATCTGGGTACAGCAATCCACCCCATTA
TTCTTCTCTTAAGTAACCGCAGGCTGAGAGCTCTGCTAGGGAGGGGCTGCTCCTCAGCACATGGGGCATCTTGA
>CaaeTAS2R62_HG919221.1:433340-434263
ATGTCCCCTTCGCCCACATTGATCTTCAAGGTCATCTTTTTCCTGGAGTCATTGGTTGCCATGCTGCAGAATGGCTTCAT
AGTTACCACGATGAGCGGGGAGTGGGCGTGTAGCCGGACTTTGCCCGCCAGTGACATGATTGTGGCCTGCCTGGCTGCCT
CCCGGTTCTGTCTGCATGGGATGGCCCTCCTGAACAACCTCATGGCCTCCTCTGGCTTTTGTTCCAAAATCTACTATTTC
GGCATCCCCTGGGATTTCATCACCTCCCTCAGTTTCTGGCTGACTGCCTGGCTTGCTGTCTTCTACTGCAGGAAGATCTC
CCTCTTCTCTCACCTCGTCTTCTGGATAAAGTGGAGGATTTCTCGATCGGTTCTCCAGCTGCTGCTGGGCTCCTTGATCT
TATCTGGTCTGACTGTCGTCTCCTCAGCTGCTGGGCATACAATTCTTGCCCAGATGACGGCTGCCCAGAGTTCCCATGGA
AACACCCTGGCTGGTAGCATACACGCCGTCTATTTGCACTGTTTTCTACCTCATGTAATTCTCATGAGGTTGGTTCCATT
CCTCCTGTTCCTGGTGTCCACCTTCTCGCTCATGGTCTCGCTGCGCCGGCACCTCGGGCAGATACAGGACCGCAGACCCA
GCCCACGTGATCCCAGTACCTGGGCTCACACCATGGCCCTGAAGTCACTTGCCTTCTTCCTCATCTTCTACACCTTGCAC
TTCCTGTCCCTGGTTATCATTGTGTACATCCCAGCCTTCTGGAAACACTGGCACTGGGCCTGTGAGGTGGTGACCTATGC
AGGCATCTGTCTGCCCTCCAGCATCTTGATGCACAGCAGCCCCAAGCTGAGAAAGGCCCTGAAGAAGAAGCTTTGGCGAG
CTCTGGACAAGGACCAGTTTGTCTCCACTTATCAGTATCAATAG
>CaaeTAS2R67A_HG918378.1:2834502-2835440
ATGCCATCTGGAATTGAAAATACTTTTCTAGTAGCAACAATAGGAGGATTTGTGATTGGAATGTTGGGGAATGGGTTCAT
TGTACTAGTTAACTGCATTGACCTGGTGAAGAGACAAAAGCTCTCATCAGCTGACTGCATCCTCACAGGCCTGGCTATCT
CCAGAATCAGTCAACTTTGGGCAATACTATGTGACTCATTTTTATTGGTACTATGGCCACACCTATATGCCATTGATAAA
CTAACAAAAATTGTTAACAGTTTTTGGACACTGTCCAATCACCTAGCTACCTGGTTTGCCACCTGTCTAAGTGTTTTCTA
CTTCTTTAAAGTAGCCAACTTCTCCCACCCCTGCTTCACTTGGCTGCGGTGGCGAATTCGTAGTGTGGTACTGGTGCTTC
TCTTGGGGTCTTTGCCCTTACTGTTTTTGAATTCTGAATCAATATATATGTTTAGTCATATCTCAACTAACAGCTACAAA
ATATATGCAAGAAACTCAACGCGGTCCCCAAATGTAAGTGAAACTCATGATCTTCACCAGTTGATTGTTTTTAACTTCAT
CAGCTTAATCCCCTTTCTTCTGTCCCTGACCTCGCTGCTCCTCTTAGTCCTCTCCTTGATGAGGCACATCAGGAATTTGC
AGCTCAACCCCAGCTCAAAGGATCTCAGCACAGAGGCCCATAAAAGAGCCATGAAAATGGTGATGTCTTTCCTCTTCCTC
TTCATCATTCATGTTTCTTCCGTCCTATTAACAGGTTGGGTTTTCCTTAAACTGCAGGGACGTCTGGCCAAATTGGTGGT
TGTGTTAACTTCGACTGTTTTTCCTTCAAGCCACTCGTTTATCCTAATTTTGGGAACTAGCAAGCTGAGACAAAATGCCA
TAGGACTACCGTGGTATCTTAACTGCCGCCTGAAAAGAGTGAAATCTTTAGCTTCATAG
>CaaeTAS2R372A_HG918378.1:3003963-3004889
ATGTCAAATGTCATCACATATGTTTTTTTGATCATTGAAATCTCAGAATTCATAACAGGAATTTGCGGAAATGGATTCAT
TGCACTAGTACTTTGTGCTGACTCTCTCAAAAGCAAGAATATCTCCTTGCTTGACTTCATCTTCACATGCTTGGCCATCT
CCAGAATTGGTATGATATTCATACTTCTCCTGGATAGCATTAAAATTTTGTTCCATCCAGAAATATTAGATCGTCACCAG
GTAATAGAAGTAACTTTTGATTTCCTCTGGAATCTGAGCAACTCCTTAGGTACCTGGTGTGCTGCCTGCCTCAGCGTCTT
CTACTTCCTCAAGCTATCTAGTTTTTCCCACCCCTTCTTTCTCTGGCTAAAATGGAGAAGAAATAGAGTTGTTTTCACCA

TTATGTTGGGATTCTGCCTCTCTTTGTTTTTAATCTTCTGAACATAAAATTCAATGCTCTCAGGGTCTGTGACCATTTA
GAAATAGAAAACAAGTTGACTTGGAAAAAATGCATGCGTAAAACACAGTCCTATAGCAGTCAAATTCTCCTCCAGCTGGG
ATCTCTCATCCCCTTGGCTCTGTCACTCGTTTTATTTTTCCTGTTAATATTTTCCTTATGGAGACATAGCAGGCAGATGA
CACGCTATGCCAAAGGATCCAAAGACCTCAACACAGGAGTTCTTGTGAGAACAAGAAATACGTTGGCCTCTTTCATCCTT
CTCCTAGTTGTGCACTATTTGGCTGCTTTCATGTTAACTCGGTCCTATTTCACACTAGAAAATGACATGACTTTTATTGC
TATTCACACTGTAGCATTTCTCTATCCTTCAATTCACCCTTTTATCTTGATTCTGAGGAGCCGGAAACTGAGACAGATTT
CTGTGAATCTGCTAAGGCAAATTGAATCCTGTATCAAAGGACTGTAA
>CaaeTAS2R5P_HG918414.1:2677686-2678560
ATGCTGACTTCTATCCCAGGACTGCTGATGCTGGTGGCAGTGGCTGAATCTCTCATTGGCCTCACTGGAAATGGAGTTCT
TGTGGTCTGGAGTTTTGGAGAATGTCTTCGAACGTCCAGGGAGTCCTCGTATAACCTCATTGTCCTGGGCCTGGCGGTCT
GTCGGTTGCTTCTACAATGGTTGATTATGGTGGACTCAAGTCTGTTCCTGCTTTTCCAGAGCAGCCATTGGCTTCGCTGT
CTCAGTGTCTTCAGGGTTCTGGTAAGCCAGGCCAGCCTGTGGTTTGTGAGTTTTCTCAGTATCTTCTATTGTAGGAAGAT
CATGACCGTTGAACACCCTGTCTCCTTGTGGCTGAAGCAGAGGGCCTGTTACCTGAGTTTCTGGTGCTTTCTGGTGTACT
TCATGATCCATTTGTTACTTATAGTCAGGGGTAGCTTAGACTTCTCCAGTCCTTCCCAAGGAAACAGCATCTTATTCCCC
ATTTCAAACTGGCACTATATATGTATATTACAGCTCAATACAGTATGATGCCTTTCATGACGTTTCCTCTTTCCTCTGGG
CTGCTGATTGTCTCTTTATATAGACACGACAGGAAGATGAAGGTCCATACAGCTGGCAGAAGAGATGCTCAGGCCAAGGC
TCGCATCACTGTCCTCAAGTCCTTGGGCTGTTTCCTTGTACTTTACATGGTCTATACCCTGGCCAGCCCCTTCTCCATCA
GCTCCAAGACTTTTCCTGCAGATCTCATCACTCTCTTCATCTCTGAGACACTCATAGCTGCCTGCCCTTCTCTTCATTCT
GTCATTCTGATCATGGGGAATCCCAGGATGAAGCAGACATGTCAGAGAATCCTGCGGAAGACTGTATGTGCTTGA
>CaaeTAS2R8P_HG918378.1:3036645-3037571
ACATTCAGTATAGAAGACCACATCTTTCTGACCATAACGACTGCAGAATTCATCATAGGAATGTTTGTGAATGGATACAT
GGGACTAGTAATATATATTGATTGGATTAAGAAGAAAAAGATCTCCACAACTGACTACATCCTCTCCAATTTAGCTCACT
CCAGAATTTGTTTGCTTTGTGTAATGACACTCAACGGCACCATACTGGCACTCTACCCAGGTGTTTATGAAAATGAGAAA
ATAAAGGTAGTTCTTAATATCTTCTGGACATTCACCAACTACTTACATATGTGGTTTGCCACCTGCCTCAATGTCTTCTG
TCTCTTCAAGATAGCCAATTTCTTCCACCGACTGTTTCTCTGGCTGAAGTGGAGAATCGAGAGGGTGTTTCACTGGATTC
TGCTGGGGTCCCTGGCCATTTCCATGTTGATCAGCCTTATACAAGCAACGTTAACAAATTCTGATTATGAATTTCTTAAA
ATTGGAGACATAAAAGAAACATCACCAAATTGTTCCATGTGAGTAAAATTCAATACTTCAACCCATTGACACTGTTTAAC
TTGTTAGCAATTATTCCATTTACTGTCATTGATCTCATTTTTCTTTTTAATTACATCCCTGTGGAGACACAGCAAACAAG
TGAAATCCAGTGTTACAGGTGCCACAAACTCCAGCACAGAGGCCCACGTGGATACCATGAAAACAGTGACCTCATTTCTT
TTCTTCCTTTTTGTATACTACCTGGCCTGTCTTTTGGCAACATTTAGCTACTTTATGAAAGAAAGCAAGTTAGCTATGAT
GTCTAGAGAGATTATAGCAATTCTTTATCCCTTAGGTCACTCACTGTTTTTAGTTGTTGGAAATAACAAGCTGAGGCTGG
CAGCTGTCGGGATGCTGAGATGTGGGAAAACAGTCTGCATGACGTAA
>CaaeTAS2R9P_HG918378.1:3034782-3035713
ATACCAGGTACAATGGAGGCAATATATATGTTCTTGATTACTGGTGAGTGGATGATAGGAATTTGGGGAAATGGATTCAT
TGTACCGGTAAACTGCAGTGGCTGGCTCAAAAAGAGAGCTGTCTCCTTGACTGAGGTCATCCTGGTCAGCCTGGCCACCT
CCAGAATCTGTTTTTTGTATGTGATATATATGGATGGTTTTATTATGGTACTCTTTCCAGATACATACAGGCATGGTGAG
ATGATGAACATTTTGGATATTTTCTGGACAACTTGCAATCATTCAACTGTCTGGTTTACTTCGTGCCTCAGCATCTTCTA
TTTACTCAAGATAGCCAGTATATCCCACCCAGTTTTCCTCTGGCTGACGCTGAAGATGAACAGGGTATCCTTGGGATTCT
TCCGATGTCCTTTCTCATCTCCTCAATTGTCAGTGTTTTACTGAATAATGATTCATTTTGTGACGTCAGAATCAATAATG
AAGCAAACATTATTTAGGAATTCAAAGTAAGTAAAATCCCAACTGCTTTCAAATTGATTATCCTGAACCTGGGGGCTATG
GTTCCCTTTATTCTTTGCCTGGTCTCAATTGTCCTTTTATTTTTCTCACTTTAACACACCAAGCAAATGAAACTTCATGC
CACTGGGTCCAGAGACCCTAGCATAGAGGCCCACATGAGGGTCATAAAGACAATAGTCATCTTTCTGGCTCTTTTCATTA
TGTACTATGCAGTTTTTCTCATTGTAACATCTAGCTTTCTGATTCCTCATGGAAAATTGGAGTTGATGTTTGATGGCCTA
AGAGCTGCCATTTTCCCATTGAGCCATCCATTCATCCTGCTAATGGGAAACAGAAAGCTGAGGGAGGCTTTTCTGAAGGT

GCTGGGGATTGTGAAGGGTTTTCACAAAAGAAGGAAATATTTTATTCCCCAG
>CaaeTAS2R10CP_HG918378.1:3023580-3024337
CTGGCATATATGCTGAGAAGGCCTCCTCATTTTTGTAGCAGTTAGTGAGTCAGTACTGGGGGTTTTAGGTGATGGATTTA
TTGGACTTGCATACTTCATTGAATGTGTGAAGAACAAGAAGTTTTCTATCAGCTTTATTCTCATGGGCTTAGCTACTTCC
AGAATTTGCCTGATAGGGTTAACAACTACAGATGGATTTGTGAAGACTTTTTTCTCCAGAAATGTATTCCTGTGGTTACC
TAATTCACTGTATTACTTACTCATGGATAATTCTGAGTCCATCAAGTGTCTTTTTTTGCCACTAGCCTCAGCATCTTCTA
TTTCCTGAAGATAGCCAATTTTTCCCACCGCATTTTTCTCTGGTTGAGGAGTGATATCAAAAGGGTTCTTCTCCTTCTGA
TGGGATACTTGCTTATTTCACGGTTAGTTACTTTTCCACTAACTATGAAGATAATTAGTGATACTAGAGCAAAGAATAGA
AGCGTAATCTTTTCAGTTGAAGTGCATAAAGGGGAATTCTTTAGAAACCAGATTTTGCTCAATCTTGGAACCCTTCCCAT
CTTCATACTATGCCTGATTACATGTATCTTATTGCTCATTTCCCTTTGGAGGCACAACCAGAGGATGCTATTGAATACCA
CAGGATTCAGAGACCCCAGCACAGAAGCACATATCAAAGCAATGAAAGTTGTGATATCTTTTATCATCCTTTTATCTTGA
ATTTTATAAGCATTATCATAGAAATATCATGCACCTAG
>CaaeTAS2R10DP_HG918378.1:2998959-2999881
ATGCTAAGTATAATGGAAAGTCTCCTCATTTTTGTAGCAATCACTGAGTCAATATTGGAACTTTTAGGTAATGGATTTAT
TGGATTGGTAAGCTGCATTGACTGTAAGAAAAACAAGATCTCTACTATAAGCTTTATTCTTGCTGGCTTAGCAACTTCCA
GATTTTGCCTGATATGGACAATAGTTACTGATGGATTTTTAAAGTTATACTCTTCAATGTACATTCCTCTGGGAACCTAG
TTGAATATAATGGTTACTTATGGATAGTTATGAATCAATCAAGTATCTGGTTTGCCACTTGCCTCAGCATCTTCTATTTC
CTGAAGATATCCAGTTTTTCTCACCGCATCTTTCTCTGGTTGAAGGGTAGACTCAACATGGTTCTTTTCCTTCTTTTGGG
ATGCTTGCTTATTTCATGATTAGTTACTTTTCCACATTTTGTGAAGATTGTTAATGATAATAAAAGGAAAATAGAAACAC
AGTCTGATCAATGGATATGCATAAACGTGAACTCTTTGGAAAACAAATTGGGCTCCATCTTGGTGTCATTCTCCTTTTTA
TACTATGCCTGATTACATGTGTCTTGTTGCTCACTTCTTTTTGGAGACACAGCAGGAGGATGCAATTGAATGCCACAGGA
TTCAGAGACCCCAGTACAGAGGCACATATCAAAGCAGTGAAAGTCTTGGTGTCTTTTATCATCCTCTTTATCTTGAATTT
TGTAGGTACTGCCATACAAATATCAAGTGTGACAGTGCCTGAAAACAGACTGCTTTTTATTTTTGGTATGACAACCACAG
TCCTCTATCTCTGGGGTCACTTGCTTATCCTAATTCTAGGAAATAGGAAGCTCAAGCAAGCCTCTTTGAGGGTACTGAAG
CAATTAAAGTGTGGGGAAAAAGAAACTTCTCAGAACTCCTTGA
>CaaeTAS2R13P_HG918378.1:2920876-2921781
ATGGCAGATTCCTTGGAAAACATCTTTATCATTTTAATAAATTCAGCATTCATAATTGGTATTCTGGGGAATGGATTCAT
AACACTGGTGAACTGGATTGACTGGATCAAGATGCAAAAGGTCTCCTTGGCTGATTGAATCCTCACTGCTTTGGCAATTT
CCAGGATTCGTCTGATTTTGGTAATGATGGTGAGTTGGTTTACAAAGGAGTCTTATCCATTTTCTTCTTTAGACATAAAG
GGAAATAAAGTCATACTTTTTAGTATTGCTGGGCTCTTGGCCAATTATTTTAGTGTCTGGCTTGCCACAGGCCTCAGCCT
CTTTTATTTCCTCAAGATAGCCAATTTTTCAAATGCTGTTTTTCTTCACCTAAAGTTTAGAATTGGAATGGTATTTATGG
TAATGTTTCTGGGGACATTAGTATTGCTGCCTCTAAGTCTTACTTTGGTGAGCATCTATATTAATATCAAGATACATCCA
TATGAAAGAAATATGACTGTAAGTTCTAAAAGAAGTGACACTGAAACCTTTTCCAAATTGATCATATTCACCATGGGATC
TTTCTCACCCTTTATTATATCCCTGAGTTGTTTTCTCCTGTTAATGTTCTCCCTACTGAATCATGTCTAGAAGATGAGGA
GCCAGGGTTCAAGAGATCCCAGCAGCAAAGCCCACGTCAGAGCCATGATCATGGCGATGCCTTTCCTCATACTACTTGCC
ATTCACTTCCTATCTCATCTCATGACAACTTTTCATCACAATGTGATGCAGAGTGAACTGGCCTTTATGCTTGCTGAAGC
TCTTGGAACTATTTATCCTTCAGTCCACTCATTTGTCTTGATTCTGGGAAATGACAAGCTAAGAAAAGCTTCACTTTTGG
TGCTGTGGCAGTTGAGGTGTGGCTGA
>CaaeTAS2R40P_HG919221.1:227875-228828
ATGGTGACAGTGAACACGGATGCAATGGATAAAGACACGACCAGGTTCAAGATCGTCTTCACCTTGGTGGTCTCTGCAAT
AGCGTGCATCATTGGCATCGCGGGGAACGACCTCATCACCATCAACCACGGAGCCGAGTGGGTCAGAGGCGAAAGACTCC
CCATTGGTGACTGCATTCTGCTCATGCTGAGCTTTTCCAGGCTCTTGCTACAGATCTGGATGATGCTGGAAAACACGTAC
TGTCTGCTATTCTGGGTCATCTACAATGAGAAAAGAGTATACATACTTTTCAAAACCATCGTCATGTTTCTGAACTACTC
CAACCTCTGGCTTGCTGCCTGGCTCAATATCTTCTATTGTCTCAGAATCGCAAGCTTTACTCACCCGTAGTTCTCCGTGA

TGAAGAGGAAGGTCATGGGACTGATGCCTGGGCTTGTGAGGCTGTCCTTGTTCTTCTCCTTTTGCTCCAGCTTCCCCTTC
TCTAGAGGCATCTTCAATGTGTACGTGAACAATTCCGTCCCCGTCCCCTCTTCCAACTCCACTGAGAAGGTGTACTTCTC
CGAGACCAACGGCAACTTGGTTACCACCCTTTACCTGGGGATCTTCATCCCTCTGATCATGTTTATGCTGGCGGCCACCT
TGCTGATCATCTCTCTCAAAAGACACACCTTCCACATGAAAAGCAACGCCACTGGCTCCAGGGACCCCAGCATGGAGGCT
CACCTGGGGGCCATCAAAGCCATCAGCTATTTTCTCATCTTCTACATTCTCAACGCAGTTGCTCTGTTTCTTTCCATATC
CAACATCTTTGCCGCCAACAGCTCCTGGAATATTTTGTACAAAATCATCATGGCTGCCTACCCTGCTGGCCACTCAGTGC
TACTGATCTTAGGCAACCCTGGGCTGAAAAGGGCATGGAAGCGGTTTCAGCACCAAGTTCATCTCTACCTGTAA

>CaaeTAS2R408BP_HG918378.1:2891502-2892400

ATGATAATATTTAGGTCAAACATTATTTCCATTCTATTAATGACAGAATTTGTTCTGGGAAATTTTGCCAGTGGCCTCAT
AGCACTGGTGAACTGCAATGACTGGATCAAGAGACCAAAGATCTCAGCTGATGGGATTCTCACTGCTCTGGCATCTGCAG
AATTGTTATGCTCTGGACAATGTTAATAAATTGGTATGTAATTGTGCATAATCCAACTCTATATAATTCAAAAGTAAAAA
TTATTGTTCATGTTGCCTGGACAGTAAGCAACCATTTTAGTAACTGGCTTGCTACTAGCCTCAGTATATTTTATTTGTTG
AAGATAGCCAACTTCTCCAGCCGAATTTTTCTTCACCTGAAGTGGAGAGTTAAAAGTGTAGTTCTCATGATGATGTTGGG
GGCATCATTGTTTTTGTTTTTTCAAGTTGCAGTGTTAAGCATAGGTGAGGCTATTCAGACAAATGAATATGAAGGAAATA
CCACTCAGAAGACCAAACTAAGGGACATTTTACACCTTTCAAATGTGACTCTGTTCACGCTAACTTCATACCCTTCACTA
TGTCCTAGACATCTTTTCTGCTGCTAATCTTTTCCCTGTGGAAACATCTCAGGAAGATGCAGCTCAATGGTAAAGGATCC
CAGCACCAAGGTCCACATAAAAGCCATGCAACTGTCATCTCCTTTCTTTTCCTGTTTGCTGTTTACATTCTGGCTCCAGT
TTTATCAGTTTGGAATTCTAATGAGCTCCAGAAAGCTTTATGATGTTCTTTTAATCATGTATCCTTCAATCCACTCATGT
ATCTTGATCTGGGGAAACAGAAAATTAACTCAAGCCTTTCTGTCATTTCTGTGGCAGTCAAGATGCTGGCTGAAGGAAAG
GTGCCGGGGGCCAGCGTGA

>CaaeTAS2R408CP_HG918378.1:2864798-2865981

ATGATAACTTTACTATTGACCATTTTTTCCATCCTAGTATTAACAGAGTTTGTTCTAGGAAATTTTGCCGGTGGCCTCAT
AGCACGGATGAATGGCATTGATTGGGTCAAGAGACAAAACATCTCCACAGCTGATGAGATTCTCACTGCTCTGGCAGTCT
GCAGAATTGTTTTGCTCTGGGTAATGTTAATGAATTGGTACTTAGTTGTGTTGAATCCAGTTCTATATAGTTTAAAAGCA
AGAATTATAGTTCATATTGCCTGGATAGTAAGCAACCATTATAGCACCTGGCTTGCTGCTAGTCTCAGTATATTTTATTT
GTTGAAGATAGCCATTTCTCCAGCCTAATTTTCTTCACCTGAAGTGGAAAGTTAAAAGTGTCATGCACATAATACTTCTG
GGAACTTCATTCTTCTTGGTTTTTCATGTTGCAGTGAATTAGACAATGAGGCTATCCAGACAAATGAATACGAAGGAAAC
ATTCCTCAGAAGACCACATTGAGGGGCTTCCACGTGTGACTCTGCTTACGCTGCTGCTGCTGCTGCTAAGTCGCTTCAGT
CGTGTCCGACTCTGCGTGACCCCATAGACAGCAGCCCACCAGGTCCCCCATCCCTGGGATTCTCCAGGCAAGAACACTGG
AGTGGGTTGCCATTTCCTTCTCCAATGCATGAAAGTGAAAAGTCAAAGTGAAGTCTCTCAGTCGTATCTGACTCTTCACA
ACTCCATGAACTGCAGCCTACTAGACTCCTCCGTCCATGGGATTTTCCAAACAAGAGTACTGGAGTGGGTTGCCATTGCC
TTCTCTGCTGCTTATGCTGGGAAATCTCATACTATGTCCCTGATATGTTTTCTGCTATTAATTGTTTCCCTGTGGAAACA
TCTCAAGAAGATGCAGCTCAGTGGTAAAGGATCTCCAGATCCTAGCACCAAAGTCCATATAAAAGCCATGCAAACTGTGA
TATCCTTTCTCTTGCTGTTTGCCATTCATTTCCTGGCTCTAATGGGATCCATTCAGAGTTTTAAAAGGCAGCAGAAGGAA
CCTGCCTTTTTGTTCTTTGAGGCTCTTGGATTCCTCTGTCCTTCAAATGATTCATGTACGCTGTTTTGGGGAAACAGGAA
GTTAACAAAAGCATTTCTGTCATTTCTTTGGCAGCTAAGGTGCTGACTGAGAGAAAAGAAATAG

>CaaeTAS2R408EP_HG918378.1:2911774-2912650

ATGGTAACTTTACTACATTTTTTCCATCCTAGTAATAGAATTTATTCTAAGAAATTTTTCCAGTGGTTTCATGTCACTGG
TGAACTGCATTGACTGGTCAAGAGACAAAAAATCTCTTCAGCAGATGGGATTCTCACTGCTCTGGCAGTCTCCAGAATTG
GTCTGCTCTGGGTAACATTAATATATTGATATGTAAATGTGTTTAATCCCAGCTTTAGACAATTTAAGAGCAAGAATTAT
TATTATTGCCTGGATAATAAGCAACTATTTTGGCATCTGGCTTGCTGCTATCCTCAGCATATTTTATTTGCTCAAGATAG
CCAATTCTCCAATATTATTTTTCTTTACCTAAAATGGAAAATTAAAAATATTCTTCTTGTTCACTTTGTCCTGTTTGGCT
TTATTAACTCATGGTGTAAACATAAATAAGACTATCCAGGCAAATGACTATGAAGGAAACATCACTCAGAAGACCAAGCA
GAGGGACACTTTACACCTTTGAAATATGACTTTGTTCATGTTAGTAAACTTCACACCCTTTGCAATGTCCCTGACGTCTT

TTCTGCTGTTAATCTTTTCCCCGTGGAAACATTTCAGGAAGATGCAGCTCAGTGGTAAAGGACCCCAAGATCCCAGCCCA
AGGTCCATATAAAAGCCATGCAAACTGTCTTCTATTTTCTATTTGCCATTTACTTCCTGGTTCTAGTTTTCTGTTTGGAG
TTCTAATAGGCAGTGGAACAACTTGGTTATCATGGCATGCCAGGCTTTTGGAATCATATGTCTTTCACTTATCCTGACAT
GGGGAAAACAGATACTAAGACAGTCCTTCCTGTCATTTCTGCAGCAACTGAGTTGATGGCTAAAAGAAAGGAAATAA
>CaaeTAS2R18P_HG918378.1:2897316-2898227
ATGTCGGTTGGAACAAAGGTCTTCTTCCTAGCGGTGTCAACAGGAGAATTGATCTTAGGAGTACTGGGTTCATTGGACTG
GTAAACTGCATCGAGTGGGTCAAGAATGGAGAGGTCTCATCAGCTGAGTTCATCCTTACTTGCTTGGCTGTGGCCAGAAT
CATTCAGCTGTGGGTAACACTTTTGGATTCACTTATAGTAGGATTAGCTCCACATCTGTATGCCACTGGTAACTAGTAAA
AGTAGTTATTCTTCTTTGGGCACTAATGAATCACTTAACTACCTGGTTTGCCACCTTCCAAAGCATATTCTGCTTCCTTA
AGATAGTCAATTTCTCTCACTGCTTTTTCACGTGGCTGAAATGGAGAATGAACCGAGTGCCTCTTGCGCTTTTCCTGGCC
TCTTTCTTCTTATTATCTTTTGACCTCTTCATGCAAGATGCTCTTGGTGAGCTGTGGATGAACACCTTTAGAGAACCTGA
AAGGAACACGACTTTGCATTTAGGTGCAAGTAAAATTTTCTATCTTAAAAGCCTGATTCTTCTCAGCTTGACATATGTTA
TCCCTTTCATTCTCTTCATGGCTTCTTTGCTGCCTTTCTTTCTTTTCCTGGTGAGACACATCAAGAATTTCCAAGTCAAC
TTGAACCACCGAGAGATTTCAGCACAGAGGCCGATAAAAGGGCCTTGAAAATGGTGACAACATTTCTCCTCCTCTTCATC
GTTTACTTTATTTCTACTCCAACTGGAAATTGGATCTTCCTTAAGCTACACTGGTATGAGGTCATGATGTTCGTCATGGT
GATTTCAACTCTCTTTTGGTCAGGCCACTCATTTGTTATAATTTTGGGGAACAGCAAGCTAAGGCAGGTTGTCTTCAGAC
TACTGTGGGGTCTTAAGTTCTCTAAAAACTAA
>CaaeTAS2R372BP_HG918378.1:2970815-2971736
ATGTCAAGTGTAATCAAAACAGTTTTTATAATCATTAAAATCTTAGAATTCATAACAGGAATTTGCAGAAAAGAATTCGT
TGCACTAGTACTCTGTGCTGACTCTCTCAAAAGCAAGAATATCTCCTTGTTTGACTTGATCTTAACATGGTTGGCCGTCT
CCAGAACTGGCATGATATTCATAATTCTTGGGTGGTGTTAGAATAGTGATCTACCCAGGAATATTTGAAAGTCATCAGGT
AATAGAAGTAATTTTTTATTTCTTCTGGAATCTGAGCAACTCCTTAGGTACCTGGTGTGCTGTCTGCCTCAGCGTCTTCT
ACTTCCTCAAGCTATCTAATTTTTCCCACCCTTTCTTTCTCTGGCTGAAATGCAGAAGAAAGAGAGTTGTTTTTACCATT
CTTCTGGGATTCTGTCTTTCTTTGATTTTAATTTTCTGAGCATAACTTTTCATACATTTGGGGTCAGTGACCATTTAGAA
ATAGAAAACAACTTGCATTGGAAAAAATATGCATAAAATCCGGTCCTCGAGCAGTCAAATTCTCCTCCACCTGGGATCTC
TCATCCCCTTGGCTGTGTCACTCAGTTTATTTTTCTTGTTAATCTTTTCCTTATGGAAACATACCAGGCAGATGACAAAT
CATGCCAAAGGATCCAGAGACTTCAACACAGGGATTCTTGTGAGAGCCAGAAATACTTTAACTTCCTTCGTCATTTTCTT
AGTTGTGCACTATTTGGCCACATTCTTGTTAACCTGGTCCTGTTTCACACTAGAAAATGAAATGACTTTTATTGTTATTA
AGTCTGTAGCATTTCTCTATCCTTCAATTCACCCTTTTATTTTGATTCTAGGAAATGGAAAACAGAGAGAGACTTCTGTG
AATCTACTAAGGAAAATTGAATCTTGCATCAAGAGAATGTAA
>CaaeTAS2R372CP_HG918378.1:2988125-2988570
CAGCAGGTAATAAGAGGAGTTTTTGAGTTCTCCTCGAATCTGAGAACTCCTTAAGTACTGGATGTGCTGCCTGCCTCAGC
GTCTTCTACTTCCTCAAGCTATCTAGTTTTTCCCACCCCTTCTTTCTCTGGCTAAAGTGCAGAAGAGATAGAGGTTTTTT
TCACCATTATGTTGCAATTCTGTCTCTCTTTGATTTTTAATCTTCTGAGCATAAAATTTATACTTTTGTGTTCAGCAAGC
ATTTAGAAAAGGAAAGTCTTAACTTGGAAAAAAGGTTTGCATAAAAGTCAGTATCACAGCAGTCAGGTTCTCCTCAGCCT
TGGATCTCTCATTTCCTTGTCTGTTATCACTCATTCTATTTTTCCTGTTACTCTTTTCCTTATGGGGACATACCAAGCAG
ATGACACGCCATAACGCAGACCCCCGGGACTTCAGCACAGGGGTTC
>CaaeTAS2R67BT_HG918378.1:2842468-2842918
AAGAAACTCAACGCGGTCCCCAAATGTAAGTGAAACTCATGATCTTCACCAGTTGATTGTTTTTAACTTCATCAGCTTAA
TCCCCTTTCTTCTGTCCCTGACCTCGCTGCTCCTCTTAGTCCTCTCCTTGATGAGGCACATCAGGAATTTGCAGCTCAAC
CCCAGCTCAAAGGATCTCAGCACAGAGGCCCATAAAGGAGCCATGAAAATGGTGATGTCTTTCCTCTTCCTCTTCGTCAT
TCATGTTTCTTCCGTCCTATTAACAGGTTGGGTTTTCCTTAAACTGCAGGGACGTCTGGCCAAATTGGTGGTTGTGTTAA
CTGCAACTGTTTTTCCTTCAAGCCACTCATTTATCCTAATTTTGGGAAATAGCAAGCTGAGATAAAATGCTATTGGACTA
CTGTGGTATCTTAATTGCCACCCCAAAAGAGTGAAATCTTTACCTTCATAG

>EqprTAS2R1_NW_007673174.1:144221-145120
ATGCTTGAGACTTACCTTTTTGTCTATCTTCTTTCTGCAGTAATTCAATTGCTCGTTGGAGTTTTAGCAAATGGCATCAT
TGTGGTTGTGAATGGCACTGGCTTGATCAAGCAGAGAAAGATGATTCCATTGGACCTCCTTCTTTCCTGCCTGGCGATTT
CTAGAATTTGTCTGCAGTTGGTCATCTTCTGCATTAATCTGAATGTTCTCTCCTTGACTGAATTTACTATATTTCCGGAC
AATTTTGCAATTTTCACGTTTGTAAATGAATTGGGACTTTGGTTTGCCGCATGGCTCAGCGTTTTCTACTGTGCCAAGAT
TGCCTGCATCGCTCACCCACTCTTCTTCTGGTTGAAGATGAGGATAGCCAAGTTGGTGCCATGGCTGATCTTCGGGTCCC
TGCTATATGCATCGATCATTTCTGTTTTGCATAGCAAACATACAGGGATTCTGTTCCAAAAAATCTGGTTGGACCTTTTC
TCCAATAACACAACAGCTCAAATCAGAGAACTATCTGTTTTACAGCGTTCCTTTCTTGTCATTGAGTTCTCATTACCGTT
TCTTATCTTCCTTTTTTCTACTCTGCTCTTGATATTTTTCCTGGGGAGACACACCTGGCAGATGAGAAACACAGTGACAG
GCACCAGGAACGCTAGCATGCGTATCCACATCAGTGCACTTCTGTCCATCCTGTCCTTCCTGGTCCTCTACCTCGCCTAC
TATGTGATGCCTGCTTTGTTCTTTTCTCAAATTTTTAAGCTCAGGAACCCCATCTTTCTATTCTGCCTCTTTGTGGTTGG
ATCATACCCCGGTGGACACTCTGTTATCTTAATTTTAGGAAATCCTAAACTGAAACAAAATGTGAAGAAGACCCTCCTTC
ACAGTAAGTACTGTCAGTGA
>EqprTAS2R2_NW_007675307.1:62194-61289
ATGGTCTCCCCTTTATCAGCTATTCTTCATGTTCTCATCATGTCAGCAGAATTTATCACAGGGATTACAGTAAATGGATT
TCTTATAATCATCAACTGTCATGAATTGATCAAAAGCAGAAAGCTAACACCAATGCAACTCCTTTTTGTATGTATAGGGA
CGTCTAGATTTGGTCTGCAGATAGTGTTAATGGTACAGAGTTTCTTCTCTATCTTCTTCCCACTCTTATATGCTGTAAAA
ATTTATGGTCCAGTGATGATCTTCCTTTGGATGTTTTTTAGCTCTGTCAGTCTCTGGTTTGCCACCTGCTTGTCTGTATT
TTACTGCCTCAAGATAGCAGGCTTCACTCAGTCCTATTTTCTTTGGCTGAAATTCAGAATCTCAAAGTTAATGCCTTGGC
TGCTTCTGGGAAGCCTGCTGGCCTCCGTGAGCATTGCAGCTCTGTGTACGGAGGTAGATTACCCTCTACACGTGAGTGAT
ATCCTCAGGAACACCACGCTAAAGAGAACGGAACTCAAGATAAAGCAAATTAGTGAAGTGCTTCTTGTCAACTTGGCATT
GATATTTCCTCTGGCCATATTTGTGATGTGCACTTTTGTGTTATTCATTTCTCTCTATAAGCACACTCGTCGGATGCAAA
AAGGACCTCATGGTTTCAGCGATGCCAGCACAGCAGCCCATATAAATGCATTAAGAACAGTAATAACATTCTTTTGCTTC
TTTATTTCTTATTTTGCTGCCTTCATGACAAATATAACATTCAGTGTTCCTTACAGAAGTCAGAACTTCTTTGTGTTGAA
GTTCATAATGGCAGCATATCCCTCTGGCCATTCGGTTATTATAATCTTAAGTCATTCTAAGTTCCAACAACCATTCAGGA
GACTTCTCTGCCTCAAAAAGAATTAA
>EqprTAS2R3_NW_007682172.1:4600-5547
ATGTTGGGACTCACTGAGTGGGTGTTTCTGGTTCTATCTGCCACTCAGTTCCTTCTGGGAATGCTGGGGAATGGTTTTAT
AGAGTTGGTCAATGGCAGCAGCTGGTTCAAGAACAAGAGAATCTCTTTGTCTGACTTCATCATCACTAACCTGGCTCTCT
CCAGGATCGTTCTGCTGTGGGTTCTCTTGGTTGATGGTGTTTTAATGGTGTTCTCTTCCAAAGTACGTGAGGAAAGGATA
GTAATGCAAATTATTTGTGTTTCCTGGACATTTACAAACCATCTGAGTATTTGGCTTGCCACCTGTCTCAGTGTCCTCTA
CTGCCTGAAAATTGCCAGTTTCTCCCACCCTACATTCCTCTGGCTCAAGTGGAGAGTTTCCAGAGTCGTCATATGGATGC
TCTTGGGTGCGCTGCTCTTATCATGTGGCAGTGCCGTGTCTCTGACCCATGGATTTAAGATTTATTCTGTTTTCCGTGGA
ATCAATGGCACAAGGAATGTGACTGAGCACTTTAAAAAGAGAAATGAATATGGATTGATCCATGTTCTTTGGACTCTGTG
GAACCTCCCTCCGTTAATTGTGTCTCTGGCCTCCTACTTTCTGCTCATCCTCTCCCTGGGAAGGCACATGTGGCAGATGC
AGCAAAATGGTACCAGTGCCGGAAATCTAAGCACTGAAGCTCACAAGAGGGCCATCAAAATCATCCTCTCCTTCCTCTTT
CTCTTCCTACTTTACTTTCTTGCCTTTATAATTACAACAGCCAGTGATTTCCTACCAGGAACTAAGATGGTTAAGATGAT
TGGAGAAATAATTACAATGTTTTATCCTGCTGGCCACTCATTTATTCTCATTCTGGGAAACAGCAAGCTGAAGCAGATGT
TTGTGGAGATGCTCTGGTGTAAGCCTGGTCATCTGAAGTCTGGATCCAAGGGCTCCTTTTCCCCATAG
>EqprTAS2R4_NW_007682172.1:10082-11002
ATGCTTCGGATATTTTTTATCTGTTCTGTTATTGTCTCAGTAATTTTGACCTGTGTAGGACTCATTGTGAACCTGTTTAT
TGCAGTAGTCAATTATAAGACTTGGGTCAAAAGCCACAGAATCTCCTCTTCTGATAGGATCCTGTTCAGCTTGGGCATCA
CCAGATTTCTTATGCTGGGAATTCTTCTACTGAATAATGTCTACATCAACTCTCTGAAAGTTGAAAGGTCAGTCTACTTA
TCCACTTTTTTCCTGTTGTGTTGGATATTTTTGGACTCTAATAGTCTCTGGTCTGTAACCTTGCTCAATGCCTTGTACTG

TGTGAAGATTACTAACTTCCAACACTCCATGTTTCTCCTGCTGAAACGAAATCTCTCCCCAATGATCCCCAGGCTACAGC
TGGCCTGTGTGCTGATTTCTGCCTTCACCACTCTCCTGTATGTTTTGCTCAGACAGACATCACCCGTCCCTGAATTTGTG
ACTGGGAGAAACAGCACAGTATTTAACATCAATGAGGGCGTCTTGTTTTTGGTGACCTCTTTGGTCTCGAGCTCATTTCT
CCAGTTCATGATTAATGTGACGTCTGCTTCCTTGTTAATAAATTCCTTGAGGAGACATATACAGAAGATGCAGAGAAATG
CCACTGGCTTTTGGAATCCCCAGACTGAAGCTCATGTGGGTGCAATGAAGCTGATGATCTGTTTCCTCATCCTCTACATT
CCATATTCAGTTGCTGCCCTACTCCATTATGTCCCTTCTTCTGTAGGGATGAATTTAGGAGCCAGATCCATTTGTGTGAT
TGTTTCCACCTTATACCCTCCAGGACATTCTGTTCTTATTATTCTCACACATCGTAAACTGAAAACAAAAGCAAAGAAGA
TTCTTTGTTTCAACAAATGGTGGAATTTCAGTAGTAAATAG
>EqprTAS2R7_NW_007674042.1:293540-292602
ATGACAATTGACATGAAGAGCACCTTAATGATCATAGCAGCTGGAGAGTTCTCGATGGGGATCTTAGGGAATGCATTCAT
TGGATTGGTGAACTGTATGGACTGGATCAAGAACAGAAAGATTGCCTCCATTGATATAATCCTCACGAGTTTGGCCATAT
CCAGAATTTGTCTATTGTGTATTATACTATTAGATTGTTTTATATTGGTGCTGTATCCAGATGTCTATACCAGTGGTAAA
CAAATGAGAATCATTGATTTCTTCTGGACACTAACCAACCATTTAAATGTCTGGTTTGCCACCTGCCTCAGCATTTTCTA
TTTCCTCAAGATAGCAAATTTCTTCCATCCCCTTTTCCTCTGGATGAAGTGGAGAATTAACAGTATGATTCCTAGGATTC
TGCTGGGATGTTTGGCCCTCTCTGTGTTTATTAGCCTTCCTGTCCCTGAGAATCTGAATGATGATTTCAGGAGCTGTGTC
AAGAGAAAGTGGAAAACAAACTTAACTTTGAGATGCAGAGTAAATAAAGCTCAATATGCTTTCATAAAGCTATTTCTCAA
CCTGTTAACACTATTCCCCTTTTCTGTGTCCCTGATCTCATTTTTCCTCTTGATTCTTTCCCTGTGGAGACACACCAGGC
GGATGCAACTCAATGCCACAGGGAGCAGAGACCCCAGCATGGAAGCCCACGTGGGAGCCATGAAAGCTGTCATCTCCTTC
CTCCTCCTTTTCATTGCCTACTATCTGGCCTTTCTTGTGGCCACCTCCAGCTACTTCATGCCAGAGACTGAATTAGTTGT
TATGGTTGGTGAGGTGATAGCTTTAATCTATCCCTCAAGCCATTCATTTATCCTAATTCTGGGGAACAACAAATTAAGAC
AAGCATCTCTAAGGGTGCTATGGAAAGTAAAGTATCTTCTAAAAAGAAGAAATTTCTGA
>EqprTAS2R8_NW_007674042.1:298155-297235
ATGCTCAGTACAGAAGAAAACATCTTTATGGTCATAATAACTGGTGAATTCACAGTTGGAATGTTGGGGAATGGATACAT
TGGACTGGTAAACTGGATTGACTGGATTAAGAAGAAAGAGACCTCTTCAATTGACTACATCTTCACCAGTTTAGCTATCT
CCAGAATTTGTTTGATTTGTGCAATGGTGCTGAATGCCCTTATAATAGTATTCTACCCAGAGGTTCATGAAAATGATAAA
ATAAAGATAGTCAACATCTTCTGGACACTCACCAACTACTTAAGTATGTGGTTTGCCACCTGCCTCAATGTCTTCTATTT
CCTCAAGGTAGCTAACTTCTCCCATCCACTTTTTCTCCAGCTGAAGTGGAGAATTGACAGGGTGGTTCACTGGATCCTGC
TGGGGTGCTTGGCCATTTCCTTGTTGATCAGCCTTATATTTGCAATGACCCCAAAATATGAGTTGCTGAAAATTGCAAAA
CATAAAAGAAACTTCACTGAATCATTTCATGTGAGTAAAATTCAATACTTCAGCCCAGTGACAATCTTTAGCCTGTTGGC
AACTGTCCTATTTACTGTGTCATTGATTTCACTTTTCCTTTTAATTATGTCCCTATGGAAACATATTAAGCAAATGAAAC
TCAATGTTACAGGCTGCAGAGACCCCAGCACAGAGGCCCATGTGAGACCCATGAAAACTGTGACTTCATTTCTCTTTCTT
CTTTTTGTATACTATCTGGCCTCTCTTTTAATGACGTTTAGCTACCTGATGAAAGAAAGAAAGTTAGCTGTGATGTTTGA
AGAGGTTATAGCAATTTTCTATCCCTCAGGTCACTCACTTATTTTAATTATTGGAAACAACAAGCTGAGGCAGGCATTTG
TCAGAATGCTGAGATGTGGAAAAACAGCCTGCATGATGTAA
>EqprTAS2R9_NW_007674042.1:301359-300424
ATGCCAAATACAATGGAGACAATATACATGATCCTGATTGCTAGCGAATTGAGTATAGGAATTTGGGGAAATGGATTTAT
TGTACTGGTTAACTGCACTGGCTGTTTCAAAAGGAGCATTATCTCCTTGATTGACATCATCCTGGTCCCCTTAGCCATCT
CCAGAATCTGTTTGTTGTGTGTGATATCTTTAGATGGCTATGTTATGCTGCTCTCTCCAGATACATATGACCATGGGGAG
CTAATGAACATTTTGGATGTTTTGTGGACATTTAGCAATCATTCAAGTGTCTGGTTTACTTCTTGCCTCAGCATCTTCTA
CTTACTCAAGATAGCCAATATAGCCCACCCATTTTTCCTCTGGCTGAAGCTAAAGATTAACAGGGTCATCCTTGGGATTC
TTCTGGCATCCTCTCTCCTCTCCTTAATTATTAGTGTTGCACTGAATGAAGATTCCTGGTATAACTTCAAGGTCAATCAT
GAAGGAAATATAACTTTGAAATTCAAAGTGAGTAAAGTCTCAAATGCTTTCAAACAGATTATCCTGAACCTGGGGGCGAT
TGTTCCCTTTATACTCTGCCTGATATCATTTCTGTTGCTGCTTTTCTCCCTATTTAGACACACCAAGCAGATGAAATTTC
ATGTCACAGGGTCCAGAGACACCAGCACAGAGGCCCACATGAGGGCCATAAAGACAGTGCTGATCTTTCTGCTCCTCTTC

ATTGTGTACTATGCCGTCTTTCTTGTAATGACCTCTAGCTATCTGATTCCTCAGAGAAAATTACGGATAATGTTTGGTGG
CATAGTAGCTGTCATTTTCCCATCTAGCCATTCTTTTATCCTGATAATGGGGAACAGCAAGCTGAGGGAGGCTTTTCTGA
AGGTGTTAAGGACTGTAAAGGGTTTCCACCAAAAAAGGAAATCTTTTGTTCTCTAG
>EqprTAS2R11B_NW_007674042.1:407634-406693
ATGTTGAATACGTTGGAGAAAATTTTCATGATTGTAACAGGTGGAGAATTTATAATAGGAATTTTAGGGAATGGATTTAT
TGGACTCACAAATTGCATTGCCTTGATTAGAAATCGGAAGTTATGCTTGGTTGACTTCATTCTTACCAGTTTGGCCTTCG
CCAGAATCAGTAAATTATGGCTAACAATTGTCAATTTGGTTTTAGTGCTGGTCTATCAGGAAATCCCTGAAACTATGAAA
ACAAACAATATCCTTACCAGCATCTGGATACTGGTCAACCACTTGACCACTTGGTTGGCTGCTTGTCTCGCTGTCTTTTA
TTTCCTGAAGATCTCCAGTTTCTCCCATCCTCTTTTCCTTTGGCTGAAACGGAGAATTAACAAGGTAATTTACATGGTTC
TGCTGTCATCTTTGCCCTTCCTGTTGATCAACTTTCCTTTGCCAGTTAATATTGATGTCGTCTGGTATCATGTCCAAAAG
AAATATGAAAGAAATATGACTGGGTTAGTCAATGTGAGTAAAAGCAAACATTTAAGAGTCATGGTAGTCTTCATTATTGG
GTCCTTCCCTCCTTTCTCTCTTTCTTTGATTTCCTTTTTCCTTTTGCTCTTTTCTTTGTGGAGACACACGAAACACAATT
TGCTCAACTTCAAGGATTCCAGAGACCCCAGTATGGAGGCCCATGTCAGAGCCATGAAAACTGTATTTCTCTTTCTTGTC
CTCTTTGCTGTGTACCAGTTATCTATTTTCATGACATTTTGGGGGTATTTTTCACTACAGAACAAGCTGGTTGTGATGTT
TGCTTATATGATAGAAATTCTCTATCCTTCTGGTCACTCATATGTTGTGATTTTTGGAAATAGTCAAATGAGGAAAGCCT
TCTTGGGGTTTCTTTGTCACTTGAAGTGTGACTTGAAAGGAAAGGCACTGTCAGCTGCATAG
>EqprTAS2R11C_NW_007674042.1:363811-362870
ATGCCGAATACATGGGAGAAAGTTTTCATGATTGTAACAGGTGGAGAATTTATAATAGGAATTTTAGGGAATGGATTTAT
TGGACTCACAAATTGCATTGCCTGGATTAGAAATCGGAAGTTGTGCTTGGTTGACTTCATTCTTACCAGTTTGACCTTGG
CCAGAATCAGTCAATTATGCCTAACAATTATCAATTTGTTTTTAGCACTGGTCTATCAGAAAATCCCTGAAACTATGAAA
ACAAACAATATCCTTACCATCATGGGGATACTGATCAACCACTTGACCACTTGGTTGACTGCTTGTCTCACTGTCTTTTA
TTTCCTGAAGATCTCCAGTTTCTCCCATCCTTTTTTCCTTTGGCTGAAGTGGAGAATTAACAAGGTAGTTTACATGGTTC
TGCTGTCATCTTTGCCCTTCCTGTTGATCAACTTTCCTTTGCCACTTAATTTTGATGTCTTCTGGTATCATGTCCAAAAG
AAACATCAAAGAAATATGACGGGATTAGTCAATGTGAGTAAAAGCAAACATTTAAGTGCCAGGACCGTCTTCATTATTGG
ATCCATCCTTCCTTTCTCTCTTTCCTTGATTTCCTTTTTCCTTTTGCTCTTTTCCTTGCGGAGACACATGAAACGCAATT
TGCTCAACTTCAAGGATTCCAGAGACCCCAGCATGGAGGCCCATGTCAGAGCCATGAAAACTGTGCTTCTCTTTCTTGTG
CTCTTTGCTCTGTACCAATTATCATTTTTCATGACATTTTGGGGATATTTTTCACTACAGAACAAGCTGGTTGTGATGTT
TGCTTATATGATAGGAATTCTCTATCCTTCCGCTCACTCATATGTTGTGATTTTTGGAAATAGTCAAATGAGGAAAGCCT
TCTTGGGGCTTCTTTGTCACGTGAAGTGTGTCCTGAAAGGAAGAGCAATCTCAGCTGCATAG
>EqprTAS2R12A_NW_007674042.1:496273-495302
ATGGAAAGCACATTGAAGAATATATTTATGATCATTTTGGCTGGAGAATTGTTAATGGGGATTTTGGGAAATGGATTCAT
TGTGCTGGTTAACTGTATTGATAATGGATTCATTGTACTGATTAACTGTATTGACTGGATCAGGAGCTGGAAGTTCTCCC
TGATTGACTTTATTCTCACCTGCTTGGCTAGTTCCAGAATATTTCTGCTGTGCATAATAATTTTAGGTATAGCTTTAGAT
GTAAACTTTGAGGAAATATGGTACAATAATAATAATCTACTGAGAAGTTTGGAAATCCTCTGGACGGGATCCAATTATTT
CTGCATGATCTCTACCACCTGCCTCTGTGTCTTCTATTTCCTCAAGATAGCCAACTTCTCTAATTCCATTTTCTTCTGGA
TGAAGTGGAGAATTCACAAGATTCTTCTCATTATTGTGTTGGGGGCAACCCTCTCTTTCTGCTTGTGCATTCTTTTTAAG
GATACATTAGTTAGGAGCCTGATCAAATACCAGGTAAACGCTGAAAGAAATTTTACATGTAACTTCATAGAGAGAAAATA
TGATTTATTAACTCCTCAAAAGCTCATTAACATAATGTTCATCATCCCCTTTTTAGTGTCTCTGGCCTCCTTAGTCCTTT
TAATCCTCTCTTTATGGAGTCATGCCAGGCAGATGGAAGACACAGGTTCTAGGGATCCTAGCAGAGAGGCCCATGTGAGA
GCTATGAAGTCTATGATTTCATTCCTACTCCTCTTCTTCATATACTATTTGAGCCATATTATAATAAATTCAGCCAATAC
CACTCTAAACACTTTTGTGGCAAAGATTTTTGCTCACGTGCTGTTATATTTGTACCCATCTGGCCATCCGTTTCTTCTGA
TTTTGTGGAACAGCAAATTGAAACAGGCTTCCCTCAGTGTCCTGAAGAAGCTGAAGAGTTGCATGAACCTAAGGAAACCT
ACAATCCCATAA
>EqprTAS2R14_NW_007674042.1:565692-564730

ATGGTCAGTGTCGTACAGAGCACACTTACAATCATTCTAAGTGCTGAATTCATAATTGGAAATTTAGGAAATGGATTCAT
AGCACTCGTGAACTGCATTGACTGGGTCAAGAGGAGAGAGATCTCTTCAGCTGATCAAATCCTCACTGGTTTGGCAATCT
CCAGAATTGGTCTGCTCTGGTTAGTATCTATAAACTGGTATATATCTGTGTTTTTTACAGTTTTACTTGTGCCTGGAAAA
CTGTTAAGAGTGAATAGTATTGGCTGGACAGTGACCAATCATTTCAGCAACTGGCTTGCTACAAGCCTCAGCATCTTTTA
TTTTCTCAAGATAGCCAGTTTTTCTAACTCTATTTTTCTTTACCTAAAGTGGAGAGTTAAAAAGGTGATTTCAATGATAC
TGCTGGTGACCTTGGTCCTCTTCATTTTTAATATTGCACTGATGAACATGCATATTAATGTCTGGATCAATGAACATAAA
GTAAACATGACCGGCACTTCTAGGATGAGCAACTTTGTACAACTTTCCACTCGTACTTTATTCATTAACACTCTGTTCAC
TATCATACCCTTTGCTGTGTCCCTGATAATTTTTCTTCTGCTTATCTTCTCCTTGTGGAAACACCTCAAGAAGATACAGC
ACAATGCCAAAGACTCCAGAGATGCCAGCACCGAGGCCCACATAAAAGCCATGAAAAGCATGATTGCTTTCCTCCTACTA
TTTGCCATTTACTTTCTGTCTCTTTTTGTGTCAATTTGGAGCTTTAAATTTCCAGAGAGAAAGCAGATCATTATGTTTTG
CCAGGTTATCGGAATTATCTATCCTGCAGGTCACCCATATGTCCCGATTCTGGGATACAATAAACTGAGACAAGCCTTTC
TTTCAGTGCTGTGCTGGCTTAGGTCGAAGATGGAGAATCTTCAGGCCCGTAGACCATTTAGAGATTCATCTTGCATATCC
TAG
>EqprTAS2R16_NW_007673219.1:110163-111068
ATGATTCCCATCCAACTCACTGTCTTCTTCATGGTCATCTATGTGGTCGAGTCCTTGACAATAATTATGCAGAGTGGCTT
AATTGTTGCAGTGCTGGGCAGAGAGTGGGTGCAGGTAAAGAGGCTGTCACCTGTGGACGTGATTCTCACCAGCCTGGGCA
TCTGCCGCTTCTGTCTACAGTGGGCATCGATGCTGTATAATTTTTGCTCCTATTTCAACCCTAACTATGTATTTTGGTAC
TACTCGATCACCTGGGACTTTTTAAATACTCTTACATTCTGGTTAACCAGCTTACTTGCTGTCATCTACTGCGTCAAAGT
CTCTTCCTTCACCCACCCCGCCTTCCTCTGGTTGAGGTGGAGAATTTTGAGGTTGGTTCCCTGGCTGTTACTGGCTTCTC
TGCTGATTTCTTGTGTGACTCTTATCTCTTCAGCTATTAGGAATCACATGAAGATACAGATAATCTCCATGGAGCATTTC
CCTAGAAACAACACTATGGTTGAAAGACTTAAGATGCTTCTGAAGAAATTTACCTTATCTCAGCCAGTGGTTGTGTTGAT
TATTCCTTTCCTCCTGTTCCTGGCCTCCACCATCTTGCTCATGACCTCATTGTCCCAACACTTGGAGCAGATGCAACATC
ACAACACTGACCACAGCAACTCCAGCGTGAACGCTCACTCCACTGCCCTGAGGTCTCTTGCTATCTTCCTTCTCTTCTTC
ACCTCTTATTTTCTGACCATACTCATCTCCATTCTGAGCATCCTATATGATAAGAGATCCTGGTTCTGGGTCTGGGAAGC
TGTTATCTATGCTATAGTCTCTGTTCAATCCACTTCACTAATGCTGAGCAGCCCTACATTGAAAAAGGTTTTAAAGGTAA
GGTGCTGGGGCCTGGAGGCTGCCTGA
>EqprTAS2R39_NW_007675651.1:24186-25199
ATGATGAAAACCTGCGGTTCCCCAGAAAATAAATTGTCACCTTTTTGGATCACCTTAGTTTTCACAATTATAGGCGCTGA
ATGCATCATTGGTATCTTTGCAAATGGATTCATCGTGGCTATAAATGCAGCTGAATGGATTCAGAATAAGGCAGTTTCCA
CAAGTGGCAGGATCCTGCTTTTCCTGAGCGTATCCCGAATAGCTCTCCAAAGCTTCCTGATGCTAGAACTTACCTTCAGC
TCAACATCCCCAAGTTTTTATAATGAAGATGTTGTATATGACACATTCAAAGTAAGTTTCATGTTCTTAAATTATTGTAG
CCTCTGGTTTGCTGCCTGGCTCAGTTTCTTCTACTTTGTGAAGATTGCTGATTTCTCCTACCCCGTTTTCCTCAAGCTGA
AGTGGAGAATTTCTGGATTGATGCCCTGGCTTTTATGGCTATCAATGCTTATTTCCTTGGGCAACAGTGTGGTCTTCTTC
AAAGACATCTACACCGTGTATTCTAACAATTCTTTTCCTATCCCCTCCTCCAACTCCACTGAGAAAAAATACTTCACTGA
GACAAATGTGTTCAATCTGGTTCTTTTCTATTACCTGGGGATCCTCATTCCTCTGATCATGTTCATCCTTGCAGCCACCC
TGCTGATCATCTCTCTCAAGAAACACACCCTACACATGGAGAGTGCAGCCACCCTGCTGATCATCTCTCTCAAGAAACAC
ACCCTACACATGGAAAGCAATGCCACTGGCTTCAGGGACCCCAGCATGGAGGCTCACATGGGGGCCATCAAAGCTACCAG
CTACTTTCTCATTCTCTACGTTTTCAATGCAATTGCTCTGTTTCTCTATATGTCCAACATCTTTGACATCAACAGTTCCT
GGAACATTTCGTGCAAAATCATCATGGCGGCCTACCCTGCTGGTCACTCCATTCTACTGATTCAGGACAACCCTGGGTTG
AGAAGAGCCTTGAAGAGGCTTCAGGCTCGAGTTCATCTTTACCTAAAAGACTAG
>EqprTAS2R40_NW_007675651.1:51889-52845
ATGGCGACAGTGAACACAGATGCCATGGATAAAGACACGTCCAGGTTTAAAATCATCTTTACCTTGCTGGTCTCCGGAAT
AGAGTGCACCATTGGCATCGTTGGGAATGGCTTCATCACAGCTATCTATGGGGCGGAGTGGGCCAGAGGCAAAAGACTAC
CCATTGGTGACCGCATCCTTTTGATGCTGAGCTTTTCCAGGTTCTTGCTACAGATCTGGATGATGCTGGAGAATACTTAC

AGTCTACTATTGCGGGTCATTTATACCCAAAACGCAGTGTATAAACTTTTCAAAGTCATCATCATGTTTCTGAACTATTC
CAACCTCTGGCTTGCTGCCTGGCTCAATGTCTTCTATTGTCTTAGAATTGCAAGCTTTATTCATCCTTTGTTCTCCGTGA
TGAAGAGGAAAATCATGGTGCTGATGCCTTGGCTTCTGAGGCTGTCACTCCTCATCTCTTTATGCTTCAGCTTTCCCTTC
TTTAAAGATATCTTCACTGTGTATGTGAATAGTTCCATTCCTATCCCCTTCTCCAACTCCACTGAGAAGAAATACTTCAC
TGAGACCAATGTCTTCAATCTGATTCTTCTTTATTATCTGGGGATCCTCATTCCTCTGATCATGTTCATCCTTGCAGCCA
CCCTGCTGATCATCTCTCTCAAGAGACACACCCTACACATGGAAAGCAATGCCACTGGCTTCAGGGACCCCAGCATGGAA
GCTCACATGGGGGCCATCAAAGCTATCAGCTACTTTCTCATTCTCTACACTTTCAATGCAGTTGCTCTATTTCTTTCTAT
GTCCAACATCTTTAATGCTGATAGTTCCTGGAATATTTTGTGCAAAATCATCATGGCTGCCTACCCAGCTGGCCACTCAG
TGCTACTGATCTTGGGAAATCCTGGCCTGAGAAGAGCCTGGAAGAGGTTTCAGAACCGAGTTCATCTTTACCTATAA
>EqprTAS2R41_NW_007676765.1:112992-112066
ATGAAGCCAGGGCTCACAGCCTTCTTTATACTGCTTTTTGCCCTGCTCTGTGTCCTGGGAATCCTGGCCAATGGCTTCAT
TGTGCTGGTGCTGAGCAGAGAATGGATGCGGAGTGGGAGGCTGCTTCCCTCTGACATGATCCTTATTAGCTTGGGTGCCT
CCCGCTTCTGCCTGCAGTGGGTTGGAATGGTGAGAAACTTCTACTTCTTCCTCCATCTGGTCGAGTACTGCAGGGGTACC
GCATGGCAGTTCTTTGGTCTACACTGGGACTTCCTGAACTCAGCCACCTTCTGGTTCGGCACCTGGCTCAGTGTCCTCTT
CTGCGTGAAGATTGCTACCTTCACCCACCCCACCTTCCTCTGGCTGAAGTGGAGGTTCCCAGGGTCAGTGCCCTGGCTCC
TCTTGGGCTCTCTCCTGGTCGCTTTCATTGTCACCCTGCTCTTCTTTTGGGGGAACTACAGTATGTATCAAGGATTCTTC
ATTAGAAAATTTTCTGGAAACATGACCTACGAGCAATGGAGCAGGAGGCTGGAAATTTACTATTTCCTACCCTTGAAATT
TATCACGTTGTCAATTCCTTGCTCTATTTTCCTGATCTCAATTGCACTGTTGATTACTTTTCTGAGGAGACACACACGGA
GAATGTGGCATAATGCCCACAGCCTGCAGGACTCTAGCACCCAGGCTCTCACCAGCGCTCTGAGGTCAATCATCTCCTTC
CTTGTTCTTTATGTGATGTCCTTTGTGTCACTGGTCATCGATGCTGCACTGTTTTTGTCCTCAGACAGTGATTGGTACTG
GCCATGGCAAATTTTAACTTACCTGGGCACATCTGTCCATTCCTTTATTCTCATCCTCAGCAACCTCAGGCTTCGAAGGG
TGTTCAGGCAGTTACTTCTGTTGGCCAGGGGCTTCTGGGTGGCCTAG
>EqprTAS2R42_NW_007673179.1:1230131-1231111
ATGTTCACTGTATTGGATAAAATCTTTCTGACGCTGGCAGGAGTGGAATTCATAATCGAAATGTTAGGGAATGTGTTCAT
GGGGCTGGTAACCTTCTCTGAATGGGTCAAGAACCAAAAGATTTCCTTAGCTGACTTCATTCTTACCTGCTTGGCTATCT
CCAGAATCACTCAACTGTTGGTTTTATTGTTTGAATCATTTATGCTGGGACAACCTTCGTGTTTCTATGCCACTTATAAA
CTAGCAAAACCTATCACTTTGCTTTGGAGAATGACTAATCACTTAACCACATGGTTTGCTACCTGCCTAAGCATTTTCTA
CCTCCTTAAGATAGCTCACTTCTCCCATTCCCTTTTCCTCTGGCTGAAGTGGAGAATGAACAGAGTGGTTCTTGTGATTT
TTGTATTTTCTTTGTTGTTTCTGATTTTTGACTTTCTATTGCTAGAAACATTTAACGATCTCTTCTTCAATGTCTGTAAA
ATAGATCAAAGTAATCTGACTTTATATTTAGATGAAAGAAAAATTCTTTATGTTAAAACCCAGATTCTTCTTAGCTTGAC
CTATTTCATACCTATTGCTCTGTCTCTGATTTCGTTGCTCCTTTTATTTCTGTCCTTGGGAAGACACAGCAGAAATTTGA
AGCTCAACTCCATGGGCTCAAGAGATTCCAGTACAGAGGCCCACAAAAGGGCCATGAAAATGGTGATGTCCTTCCTCTTC
CTTTTCGTAGTTCATTGTTTTTTCACACAATTGACACATTGGATAATTATGTTTTGTAACAGCAAATTCACAAAGTTTGT
CTTATTAGCATTATATGTCTTTCCTTCAGGCCACGCATTTATTTTGATTCTGGGAAATAACAAGCTAAGACAGACAGCCT
TGAAGGTACTGTGGCATCTTAAAAGCTCCTTAAAAAGAGAAAATCCATTAGCTTTACAGGATAGACTTTCCAGAGCCTTT
TCAAAGATAGTAACTCAGTGA
>EqprTAS2R408B_NW_007673179.1:1425606-1426514
ATGGTAAATTTACTACCAAGCATTTTTTCTGTCTTAATAACGACGGAATTTATTCTGGGAAATTTTGCCAATGGCATCAT
AGCACTGGTGAATTGCATTGACTGGGTCAAGAGACATAAGATGTCCTCAGCTGATCAAATTCTCACTGCTCTGGCGGTCT
CCAGAATTGTTTTGCTCTGGGTACTATTAATGAATTGGTATACAGTTGTGCTCCATCCGGGTTTATATAGTTTGGAAGTA
AGAATTTTTGTTCGTATTGCCTTGACAGTAAGCAACCATTTTAACATCTGGTTTGCTGCTAGCCTCAGCATATTTTATTT
GCTCAAGGTAGCTAATTTCTCTAGCTTTATATTTCTTTACCTAAAGCGGAGAGTTAAAAGTGTACTTCTCATAATACTGT
TGGGGACTCTGGTCTTTTTGGTTCCTCATCTTGCAATTCTATGCATATATGAGAATATTCAGACTAATGAGTATGAAAGA
AACATCACTCAGAAGACCAAATTGAGGGACATTTTTCACTTCTCATATATGAGTCTATTCATGCTAGTAAACTTCGCACC

ATTTTCTATGTCGCTGACATCTTTTCTGCTGTTAATCATTTCCCTGTGGAATCATCTCAAGAAGATGCAGCTCAGTGGCA
AAGGATCCCAAGATATCAGCACCAAGGTCCACATAAGAGCCATGCAAACTGTGGTCTCCTTTCTCTTCTTGTATGTCAGT
TACTTCCTAGCTCTGGTTACTTTAGTTGAGAGTTATAATAGGCTGCATAATACACTGCTTGTCATGCTTTCTGAGGCTCT
TGCAATGCTCTATCCTTTAAGCCACTCATTTATCCTGATTTGGGGAAACAAGAAGCTAAGACAGGCCTTGAAAACCATAA
GAAAGATGATAAGATTCCCATACCATTAG
>EqprTAS2R408E_NW_007673179.1:1331902-1332810
ATGGTAGCTTTACTACCAAACATTTTTCTGTTCTAATAATGACAGAATTTATTCTGGGAAATTTTGCCAATGGCTTCAT
AGCACTGGTAAACTGCATTGACTGGGTCAAGAGACAAAGGATGTCCTCAGCTGATCAAATTCTCACAGCTCTGGCGATCT
CCAGAATTGGTTTGCTCTGGGTAATATTAATAAATTGGTATACAGCTGTGCTCCGTACAGGTTTATATAGTTTAGAAGTA
AGAACTGTTGTTCATGTTGCCTGGGCAGTAAGCAACCATTTTAACATCTGGTTTGCTACTAGCCTCAGCATATTTTATTT
GGTCAAGATAGCTAATTTCTCTAGCTTTATATTTCTTTACCTAAAGCAGAGAGTTAAAAGTATACTTCTCGTAATAATCT
TGGGGACTCCAGTCTTTTTGGTTCCTCATATTACAGTGCTATGCTTAGATGAGAATATGCGGACTAATGAATATGAAGGA
AACATCACTCAGAAGACCAAATTGAAGGGCGTTTTTCACCTTTCATTTATGACTCTATTCACACTAGTAAACTTCATACC
ATTTTCTATTTCCCTGACATCTTGTCTGCTGTTAATCATTTCCCTGTGGAAACATCTCAAGAAAATTCAGCTGAATCTAA
AAGGATCCCAAGATCTCAGCACCAGGGTCCACATAAGAGCCATGCAAACTGTGGTTTCCTTTCTCCTGCTACATGTCAGT
TACTTCCTGGCTCTAATTATCTCAGTTTGGAATTCTGAAAGGCTGAAGAATCAACTATTTGTCATGCTTTGTGAGGTTCT
TGCAATGTTGTATCCTCTAAGCCACTCATTTATCCTGATTTGGAAAAACAAGAAGCTAAGATCATACTTGAAAAACATAA
GAAAGATTATAAGGTTACCATACCATTAA
>EqprTAS2R408F_NW_007673179.1:1326803-1327711
ATGATAACTTTACTACCAAGCATTTTTCCATCCTAATAACAACAGAATTTTTTCTTGGCAATTTTGCCAATGGCTTCAT
AGCACTGGTGAACTGCATTGACCGGGTCAAGAGACAAAAGTCGTCCTCAGCTGATCAAATTCTCACAGCTCTGGCGGTCT
CCAGAATTGGTTTACTCTGGGTAATATTAATAAATTGGTATGTGACTGTGCTTCCTTCAGTTTTTTGTAGTTTAGAAGTA
AGAATTATTGTTTGTGTTGCCTGGACAGTAAGCAACCATTTTAGCATCTGGCTTGCTACTAGCCTCAGCATATTTTATCT
GCTCAAGATAGCTAATTTCTCTTGCTGTATATTTCTTTACCTAAAGTGGAGAGTTAATAGTTTACTTCTTGTAATACTGT
TGGCAACTTTGGTCTTTTTGGTTCCTCACTTTGCAGTGCTGTGCGTAGATGAGACTATGCAGACTAAAGAGTATGAAGGA
AACGTCACTCGGAAGACCAAATTGAGAGATGTTGTAGGCCTTTCAAATATGACTCTATTCACGCTAATACACTTCATACC
CTTTACTATGTCCCTGACATCTTTTCTGCTGTTAATCGTTTCCCTATGGAAACATCTCAAGAAGATGCAGCTTAATGGCA
AAGGATACCAAGATCCTAGCACCAAGGTCCACATAAGAGCCATGCAAACTGTGCTCTCTTTTCTCCTGCTATATTCCGGT
TACTTCTTGGCTCTAGTTATCTCAGTTTGGAGTTCTAATCGACTGAAGAATGAACTAGTTCTCATGATTTGCCAGGCTCT
TGGAATGCTGTATCCTTCAAGCCACTCATTCATCCTGATTTGGGGAAACCAGAAGCTAAGAAAGGCCTTGAAAAATGTAA
GACAGATTATAAGATTACCACACGATTAA
>EqprTAS2R62A_NW_007675651.1:255901-256857
ATGCCTTCCTCACTCACATTGATTTTCATGGTCATCTTTCTTCTAGAGTCCTTGGCTGCAATGTCGCAGAATGGCTTCAT
TGTTGTTGTGCTAGGCAGGGAGTGGGTGCGATGCCGCACACTGCCCTCAGGTGACATGATTGTGTCCTGCCTGGCTGCCT
CCCGGTTCTGCCTGCATGGGATGGCTCTCCTAAGCAACTTTATGTCGTTATTTAATTTTTGTTCCCAAGTTTCCTATTTG
GGCGCTCTTTGGGAGTTTATCAACAGTCTCACTTTCTGGCTTACTGCCTGGCTTGCTGCCTTCTACTGTGTGAAGATCTC
ATCCTTCTCTCATCCCATCTTCTTCTGGCTGAAGTGGAGACTTTCTCGGTCAGTGCCCAGGCTGCTGCTGGGCTCCCTGA
TCATATCTGTTGTGACAGGCATCTCATCAGTCAGTGGGAATATAATTCTTATGCAGATGATTGCCTCCCTGAGGTCCCAT
GGAAACTGCACTCTGGCTGATAGAATAAGGACCTTCTCTTGGCACTTTTTCTACCTCAAGATGTGCTTGCAATGTCAAT
TCCCTTCCTCCTGTTCCTGGTATCCACTCTCTTGCTCATGTTCTCACTGCGCCGGCACTTGCGGCAGATGAGGGCCCATA
GACCCGGCCCACATGATCCCAGCACCCAGGCTCACACCATGGCCCTTAAGTCACTTACCCTCTTCCTCGTGTTCTACACA
TCATATTTCCTGTCCCTGATTATTACTGTTATGAAAATCACAACTCTGCAGGATCAGTGGCACTGGGCCTGGGAAGTGGT
GACCTATGCAGGCATCTGTTTGCACTCCAGCATTCTTGTGCTAAGCAGCCCCAAGCTGAGAAAGGCCCTGAAGATGAGAC
TTTGGAAAGCCCCGGAGAAAAGGTGGTTCATCGCAAGTATCAATATCAATAATCAGTATCAACAGACAAGCCCTTAA

>EqprTAS2R62C_NW_007675651.1:229129-230061
ATGCCCTCCTCATCCACCCTGATCTTCGTGGTTATCTTTTTTCTGGAGACCTTGGCTGCAATGTTGCAGAACGGCTTCAT
GGTTGCTGTGCTGGGCAGGGAGTGGATGCGATGCTGCACACTGCCTGCAGGTGACATGATTGTGGCCTGCCTGGCTGCCT
CCAGGTTCTGCCTGCATGGGATGGCCCTCCTGAACAACCTCCTGGACTCCTTTAATTTTCGTTCCATCGTTTCCTATTTC
AACATCCCTTGGAACTTTATCAACACTCTCACTTACTGGCTGACTGCCTGGCTTGCTGTCTTCTACTGTGTGAAGATCTC
ATCCTTCTCTCATCCCATCTTCTGCTGGCTGAAGTGGAGGATTTCTCGGTCAGTGCTCAGGCTGCTGCTGGGTTCCCTGA
TCATATCTGGTGTGACAGTCATCCCAGCAGTCACCAGAGATATAATTCTTATACAGATGATTGCCTCCCAGAGTTCCCAT
GGAAACTGCACTCTGGCTGATAGAATAAGGACCTTCCATAGGTATTTTTTGATGCCTATTATAGTTCTGGTATTGTCGAT
TCCCTTCCTCCTGTTCCTGGTGTCCACCCTTTTGCTCATGTTCTCACTGCACTGGCACTTGCGGCAGATGAGGGCCCACA
GATCCAGCCCACGTGATCCCAGCACCCAGGCTCACACCATGGCCCTGAAGTCACTTACCTTCTTCCTCGTGTTCTACACA
TCATATTTCCTGTCCCTGATTATTGCTTTTATGAAAATCACAACCCTGCAGGATCAGTGGCACTGGGCCTGGGAAGTGGT
GACCTATGCAGGCATCTGTTTGCACTCCAGCATCCTGGTGCTAAGCAGCCCCAAGTTGAGAAAGGCCCTGAAGATGAGGC
TTTGGAAAGCCCTGGACAAAAAGCAGTTTATTTTGAGTTATCAGTATCAATAA
>EqprTAS2R62E_NW_007676765.1:152440-151484
ATGCCCTCTTCACTCATGTTGATCTTCATGGCCACTTTTTCCCTGGAGACGTTGATTGCAATGTTGCAGAATGGCTTCAT
TGTTGCTGTACTGGGCAGGGAGTGGGTACAAGGCTGCACACCCCCCTCGGGGGATGTGATTGTGGCCTGCCTGGCTGCGT
CCCGGTTCTGCCTGCATGGGCTGGCCCTCCTGAACAGCTTCCTGGGCTTCTTTAAGTTTTCTTCCAAAATTTACTATTTC
AGCATCCCCTGGGACTTTATCAACACTCTCAATTTCTGGCTGACTGCCTGGCTTGCTGTCTTCTACTGTGTGAAGATCTC
AACCTTCTCTCATCCCACCTTCCTCTGGCTGAAGTGGAGGATTTCTCGGTCAGTGCCCAGGCTTCTGCTGGGCTCCCTGA
TCATATCTGGTGTGACAGTCATCTCATCAGCTACTGGGAATAGCATCGCTGTGCTGAGGAGTACCTCCCAGAGTTCCCCT
GGAAACCACACTTTGGCTGATAGAATAAGCCCCTTCTTTCGGCACTTTTTTCTGAGTCAAGAGCTACTTGTGTTGTTGCT
TCCTTTTCTCCTGTTCCTGGTGTCCACCCTCTTGCTCATGTTCTCACTGCACCAGCACCTGCAGCAGATGAGGGCCCACA
GACCCAGCCCACATGATCCCAGCACCCAGGTTCACATCACGGCCCTGAAGTCACTTTCCTTCTTCTTTGTGTTCTACACA
TCATATTTCCTGTCCCTGATTATTGTTTCTATGCAAATCACAGCCCTGCAGCATCAGTGGCACTGGGCCTGGGAAGTGGT
GACCTATGCAGGCATTTGTCTGCATTCCAGCATCCTGGTGCTAAGCAGCCCCAAGCTGAGAAAGGCCCTGAAGACAATCT
TTGGAAAGCCCTTGACAAAAGATGCTTCATCTCAAGTTATCAGTATCAATAACCAGTATCAATGGACAAGCCCATGA
>EqprTAS2R62F_NW_007676765.1:143552-142647
ATGCCCTTCTCACCCATGTTGATCTTCGTGGTCATCTTTTTCCTGGAGACCTTGGCTGCGATGTTGCAGAATGGCTTCAT
GGTTGCTGTGCTGGGCAGGGAGTGGGTGAGATGCCTCACACTGCCTGCATGTGACATGATTGTGGCCTGTCTAGCTGCCT
CTAGGTTCTGCCTGCATGGGCTGGCCCTCCTAAACAACCTCATTGACTCCTTTAACTTTTGTTCCAAAGTTTACTATTTC
AACATCCTCTGGAACTTTATCAACATTCTCACTTACTGGCTTACTGCCTGGCTTGCTGTCTTCTACTGTGTGAAGATCTC
ATCCTTCTCTCATCCCATCTTCTTCTGGCTGAAGTGGAGGATTTCTCGGTCAGTGCCCAGGCTGCTGCTGGGCTCCCTGA
TCATCTCTGTTGTGACAGTCATTCCAGCAGCAGCTAGCAATGTAATTCTTATACAGATGATTGCCTCGCAGAGTTCCCAT
GGAAACCACACTCTGGCTGATAGAGCACAGACCTTCCATAGGTACTTTTCTCTGTCTAATTTAGTGCTTGTATTGTTGAT
TCCCTTCCTGCTGTTCCTGGTATCCACTCTTTTGCTCATATTCTCACTGCACCAGCACTTGGGACAGATGAGGGCCCGCA
GACCCGGCTCATGTGATCCCAGCACCCAGGCTCACATCATGGCCTTGAAGTCACTTACCTTCTTTCTTGTGTCCTACGCA
TCGTATTTCCTGTCCCTGATTATTGCGTTTATGAAAATCACAGCCCTGCGGCGTCAGTGGCACTGGGCCTGGGAAGTGGT
GACCTATGCAGGCATCTGTCTGCACTCCAGCATCCTGGTGCTAAGCAGCCCTAAGCTGAGAAATACCCTGAAGACAAAGC
TTTGGAAAGCCCTGGACAAAAGGTGA
>EqprTAS2R5P_NW_007690259.1:924-1818
ATGCTGATTGCTGCCCTAAGACTGCTGATGCTGGTGGCAGTGGCTGAATTTCTCATTGGCCTGGTTGGAAATGGAATTCT
TGTGGTATGGAGTTTTGGAGAATGGGTCAGAAAATCCAAGGGGTCCTCATACAACCTCATTGTGCTGGGCCTGGCTGTTT
GCCGATTTCTCCTGCAGTGGTTGATTATGATGGACTTAATCCTGTTTCCGCTTTTCCAGAGCAGCTGTTGGCATCGCTAT
CTCAGTGTCTTCTGGGTTCTGGTAAGCCAGGACAGCCTGTGGTTTGCCACTTTCCTCAGATTCTTCTACTGCAGGAAGAT

CACGACCTTTGAACACCCCATTTACTTGTGGCTGAAGCAGAGGGCCTATTGCCTGAGTCTCTGGTCTGGGGTACCTCATG
ATCAGTTTGTGACTTGTGGTCCACATTGGCTTAAAGCCTTGCAATCCTTCCCATGGAAACAGCAGCATTCTATACCCCTT
TTCAAACTGGCACTATCTGTGTATTTTACATCTCAGTGCAGGAAGTGTGGTGCCTTTCATGGTGTTTCTGGTTTCTTCTG
GGATGCTGATCGTCTCTTTGTATAGACACCACAGGAAGATGAAGGCCCATACAGCTGGTAGGAGGGATGCTCGGGCCCAG
GCTCACATCACTGTCCTGAAGTCCTTGGGTTGCTTCCTTGTACTTTACGTGGTTTATGTTCTGGCCAGCCCCTTCTCCAT
CACCTCCAAGTATTCTCCTGCTAATCTCACCACTGTCTTCATCTCTGAGACACTCATGGCTGCCTATCCTTCTCTTCATT
CTGTCATATTGATCATGGAGAATCCCAGGGTGAAGCAGATTTGTCAGAGAATTTTGTGGAAGATAATGTGTGCTTGGAGA
TCTTGGGGCCTGTGA

>EqprTAS2R11AP_NW_007674042.1:488054-487143

ATGTTGAATACATTGGAGAAAGTTTTCATGCTGTAGTAGGTGAGGAATTTATAACAGGAATTTTAGGGAATGGGTTTATT
GCACTCACAAATTGCATTGCCTGCACTAGAAATCAGAAGTTATGCTTGGTTAACTTCATTCTTACTAGTTTTTCCTTTGC
CATAATTAGTCAATGATGGCTAACAATTATGTGTCTTAATAAATCTCTGATACTATGAAAAGAAACCATATCCATACTGG
TATCTGGATATTGGACAATCACTTGAGCACTTGGTTTCCTACTTGTCTCACTGTCTTTTATTTCCTGAAGATCACCAGTT
TCTCGTTCCCTTTTTCCTTTGACTAAAATGGAGAATTAATGAGATAATTTTCATACTTCTGCTGTTATCTGTGCCCTTCC
TGTTCATCAACTTTCCTTTGCCATATAGTTTTGATGTCTTCCAGTATCGTGCCCCCCCAAAAATATGAAAGTAATATGAC
TGGGTTATTCAATGTGAGAAAAAAATAAATATTTAAAAGCCATGATACTCTTCATTACTGGGTCTCTCCCTCCTTTCTCTC
CTTCCTTGATTTCCTGTTTCCTTTTCTTTGTGGAGACACACGAGACACAATTTGCTCAGCATCCAGGATTCCAGAGACTC
CAGTACGGATGCCAGTTTCAGAGCCATGAAAATTCTTTTTTGCTTTTGCGCTTTTTGCTCCGTACCAGCTTTCATTTTTC
CTGACATTGTTGAGATATTTTTCACTACAGAACAAGCTGGCTGTGATGTTTGCGTATATGATACAAATTCTCTATCCTTC
AGGTCACTCATATGTTGTGATTTTTGAAAGCAGCCTAATGAGGAAAGCCTTCTTGGGGATTCTCTGGCACCTGAAGTGTG
GCCTGAAAGGAAAGGTAGTCTTGGCTGCACAG

>EqprTAS2R11DP_NW_007674042.1:331364-330437

GTGTTGAAAACATTGGAGAAAGTTTTCATGATTGTAGCAGGTGGGGAATTTATAGCAGGAATTTTAGGGAATGGATTTAT
TGGACTCACAAATTGCATTGCCTGGATTAGAAATCGGAAGTTATGTTTGGTTGGCTTCATTCTTACCAGTTCGGCCTTCA
CCAGAATCAGTCAATTACGGCTAACAATTGTCAATTTGTTTTCAGTGGTGGTCTATCAGGAAATCCCTGATACTAAGAAA
AGAAATCATATACATACTGGTATCTGGATACTGGCCAACCACTTGAGCACTTGGTTTGCTACTTGTCTCACTGTCTTTAA
TTTTCTGAAGATCAACAATTTCTCCTATCCCCTTTTCCTTTGGCTGAAATGGAGAATTAATCAGGTAGTTTTCATGCTTC
TGCTGTTACCTGTGCCCTTCCTGTTCATCAACTTTCCTTTCCCATACAGTTTTGATGTTTTCTGGTGTCATGTCCAAAAA
AATATAAAAGAGATATGACTGGGTTATTCAATGTGAGTAAAAATAAACATGTAAGTGCCATGACAGTCTTCATTACTGGG
TCCCTCCCTCCTTTCTCTCTTTCCTTGATTTCCTTTCTCCTGTTGCTCCTTTTCTTTGTGGAGACACATGAAACACAATT
TGCTCAAAGTCAGGGTTTCCAAGGACCCCAGTATGGAGGCCCATGTCAGAGCCATGTTTTCTTTCTTGAGCTCTTTTTTC
TGTACCAGTTTGCCTTTTCCTGATATTTTTGGGTTATTTTTCACTACAGAACAAGCTGGTTGTGATGTTTGGTTACATGT
TAAGAATTCTATATCTTTGGGGTCACTCAAATGTCATGATTTTCCTCAGCCAAGTGAGGAAAGCCTTCTTGGGGATTCTC
TGGCACCTCAAGTGTAGCCTCAAAGGAAAGGCACTCTCAGCTGCATAG

>EqprTAS2R12BP_NW_007674042.1:461105-460109

ATGGCAAGCACACTGAAGAATATATTTATGATAATTTCTGTTGGAGCATTCACAATGGGGATTTTGGAGCATTCACAATG
GGGACATTCACAATGGGGATTTTGGGAAACGGATTCATTGTACTGATTAACTGTATTGACTGGATCAGGAGCTGGAAGTT
CTCCCTGATTGACTTTATTCTCACCTGCTTGGCTATTTCCAGAATATTTCTGCTGTGCATAACAATTTTAGGTATAGGCT
TACATTTCATCAATGAGGAAATATGGTACAATGATAATAATCTACTGAGAAGTTTGAGAATTCTCTGGACAGGATCCGAT
TATTTCTGCATGACCTGTACCACCTGCCTCTGTGTCTTTTATTTCCTCAAGATAGCCAACTTTTCTAATCCCATTTTCCT
CTGGATGAAGTGGAGAATTCACAAGGTGCTTCTCATTATTGTGCTCGGGGCATCCGTCTCTTTCTGCTTGTGTATTTTTT
TAAGGATACAGAAACTAGGAGCCTGATCAAAAACCAGGTAAACACGGAACAAAATTTGACATGGAATATTGCAGTGAGAA
AATATAATTTATCAACTTCTCATATGCTTATTAACATAATGTTCATCATCCCCTTTTTAGTGTCACTGGCCTCCTTACTC
CTTTTAATTCTCTCTTTATGGAGCCACACTAGGCAGATGAAGGGCACAGGTTCTAGGGATCCTAGCACAGAGGCCCACGT

GAGGGCCACGAAGTCTATGATTTCATTCCTACTCTTCTTCTTCTTGTACTATTTGAGTAATATTATGCTAAAGTCAATCT
ATGCCAATCTAGACAGTTTTACAGTAAAGATTTTTGCTAATGTGCTAGTGTTTTTCTTTCTATCTGGCCATCCATTTCTT
CTGATTTTGTGGAATAGCAAATTGAAAAAGGCTTCTCTCAGTGTCCTGAGGAAGCTGAAGTGTTGCATGAATCTAAGGAA
ACCTACAATCCCGTAAACATGCCTGAAAGGATTGTGA
>EqprTAS2R12CP_NW_007674042.1:428603-427643
ATGGCAAGCACATTGAAGAATATATTTGTGATCCTTTACACTGGAGCATTCACAATGGGGATTTTGGGAAATGGATTCAT
TGTACTGATTAACTGTATTGACTGGATCAGGAGCTGGAAGTTCTCCCTGATTGACTTTATTCTCACCTGCTTGGCTATTT
CCAGAATATTTCTGCTGTGCATAATAATTTTAGGTATAGGCTTACATTTCATCAATGAGGAAATATGGTACAATGATAAT
AATCTACTGAGAAGTTTGAGAATGCTCTGGATAGGATCCAATTATTTCTGCATGACCTGTACCACCTGCCTCAGTGTCTT
CTATTTTCTCAAGATAGCCAACTTTTCTAATTCCATTTTCCTCTGGATGAAGTGGAGGATTCACAAGGTGCTTCTCATTA
TTGTACTAGGGGCAGCCATTTCTTTCTGCTTGTGCCTTTTTTTTTAAGGAGACAGTATTTAAGAGCCTGATCAAAAACCA
GGCAAACACTGAAAGAAATTTGACATATAACTTATTAGTGAGAAAATATCATTTATTAACTCCTCGTATGCTCATTAACG
TAATGTTCATCATCCCCTTTGTGGTGTCGCTGGCCTCCTTACTCCTTTTAGCTCTCTCCTTATGGAGCCATACCAGGCAG
ATGAAGGGCACCGGTTCTAGGGATCTCAGCGCAGAGGCCCATGTGAGGGCCATGAAGTGTACGATTTCATTCCTACTCCT
CTTCTTCTTGTACTATTTGAGTAACATTATGCTAAATTCAGCCTATGCTATTCTAGACAGTTTTACGGCAAAGATTTTCG
CTAATGTGCTAGTGTTTTTCTATCCATCTGGCCATCCATTTCTTCTGATTTTGTACAACAGCAAATTGAAACAGGCTTCT
CTCAGTGTCCTGGAGAAGCTGAAGAGTTGCATGAATCTAAGGAAACCTACATTCCCATAAATATGACTGAAAGGATTGTG
A
>EqprTAS2R12DP_NW_007674042.1:423448-422493
ATGGCAAGCACACTCAGCAATATATTTGTGATCCTTTATGCTGGAGCATTCACAATGGGGATTTTGGGAAACGGATTCAT
TGTACTGATTAACTGTATTGACTGGATCAGGAGCTGGAAGTTCTCCCTGATTGGCTTTATTCTCACCTGCTTGGCTATTT
CCAGAATATTTCTGCTGTGCATAACAATTTTAGGTATAGGCTTAGATGTAAACTTTGAGAAAATATTGTACACTAATAAT
AATCTACTGATAAATTTGAAAAACCTCTGGATAGGATCCAATTATTTCCGCATGACCTGTACCACCTGCCTCAGTGTCTT
CTATTTCCTCAAGATAGCAACTTTTCTAATCCCGTTTTCCTCTGGATGAAATGGAGAATTCACAAGATCCTTCTCATTAT
TGTGTTGGGAGCAACCTTCTCTTTCTGCTTGTGCCTGATTTTTAAGGAGGCAGTATTTAAGAGCCTGATCAAAAACAAGG
TAAATGCTGAAAGAAATTTGACATGGAACTTCACAATGAGAAAATATTCATTAACTTCTCAAATGCCCTTTGACATAATG
TTCATCATTCCCTTTTTAGTGTCACTGGCCTCTTTATTTCCTTTAATCCTCTCCTTGTGGCGCCATATCAAGAAGATGAA
GGGCACAGGTTCTAGGGATCCCAGGACAGAGGCCCATGTGAGGGCCATGAAGTGTATGATTTCATTCCTACTCCTCTTCT
TCTTGTACTATTTGAGCCATATCATAAAATATTCTGCCGATGCGGTTGTAGATACTTTTGTAGCAAAGATATTTGGTAAT
GTGCTAATATTTTTGGATCTGTCTGGCCATCCATTTCTTCTGATTTTGTATAACAGCAAATTGAAACAGGCTTCTCTCAG
TGTCCTGGAGAAGCTGAAGTGTTGCATGAATCTAAGGAAACCTACAATCCCATAAACATGCCTGAAGAGACTGTAA
>EqprTAS2R12EP_NW_007674042.1:376665-375710
ATGGCAAGCACACTCAGCAATATATTTATGATCCTTTATGCTGGAGCATTCACAATGGGGATTTTGGGAAATGGATTCAT
TGTGCTGGTTAACTGTATTGACTGGATCAGGAGCTGGAAGTTCTCCCTGATTGACTTTATTCTCACCTGCTTGGCTATTT
CCAGAATATGTCTGCTGTGCATAATAATTTTAAGAATAGCCGTAGTTGTAAACTATGAGAAAACATTGTACACTAATAAT
AATCTACTGATAAGTTTGGAAACCCTCTGGACAGGATCCAATTATTTCTGCATGACCTGTACCACCTGCCTCAGTGTCTT
CTATTTCCTCAAGATAGCAACTTTTCTAATCCCGTTTTCCTCTGGATGAAATGGAGAATTCACAAGATGCTTCTCATCAT
TGTACTGGGAGCAACCTTCTCTTTCTGCTTGTGCCTTCTTTTTAAGAATACACTAGTTAAGAGCCTGATCGTAAACCAGG
TAAATGCTGAAAGAAATTTGACTTGGAACTTCACAGTGAGAAAATATTTATTAACTTCTCAAATTCTCCTTGACATAATG
TTCATCACCCCCTTTGTAGTGTCACTGGCCTCCTTACTTCCTTTAATCCTCTCCTTATGGAGCCATACTAAGCAGATGAA
AGGTACAGGTTCTAGGGATACTAGGACAGAGGCCCACGTGAGAGCCATGAAGTGTATGATTTCATTCCTACTCCTCTTCT
TTATGTACTATTTTAACCATATTATAAAATATTCAGTCTATGCTGTTGTAGATACTTTTGTAGTAAACACTTTTGCTAAT
GTGCTAGTGTTTTTTGATCCATCTGGCCATCCATTTCTTCTGATTTTGTGGAACACCAAATTGAAACAGGCTTTTCTCAG
TGTCCTGGAGAAGCTGAAGTGTTGCGTGAATCTAAGGAAACCTACAATCCCATAAACGTGCCTGAAAAGATTGTGA

>EqprTAS2R12FP_NW_007674042.1:346227-345313
ATGGCAAGCACATTGAAGAATATATTTAAGATCCTTTATGCTGCAGCATTCACAATGGGGTTTTGGGAAACGGATTCATT
GTACTGATTAACTGTATTGACTGGATCAGTGCTTGGCTATTTCCAGAATATTTCTGCTGTGCATAACAATTTTAGGTATC
AGCTTAGATGTAAACTCTGAGAAAATATTGTACACTAATAATAATTGACTGATAAGTTTGGAAACCCTCCGGGTAGGATC
CAATTATTTCTGCATGACCTGTACCACCTGCCTCAGTTTCTTCTGTTTCCTCAAGATAGCCAACTTTTCTAATCCCATTT
TCCTCTGGATGACATGGAGAATTCATAAGGTGATTCTCAGTGCTGTGCTGGGGGAAGCCCACTCTTTCTGCTTGTGCCTT
TTTTTAAGGATACAGTACTTAAGAGCCTGATCATAAACCAGGTAAATACTGAAAGAAATTTGATATGGAACTTCACAGTG
AGAAAATATTCATTAACTTCTCAAATGCACTTTGACATAACGTTCATCACCCCCTTTGTAGTGTCACTGGCCTCCTTACT
TCCTTTAATCCTCTCCTTATGGAGCCGTACTAAGCGGATGAAGAGTACAGGTTCTAGGGATCCCAGGACAGAGGCCCATG
TGAGGGCCATGAAGTGTATGATTTCATTCCTACTCTTCTTCTTGTACTATTTGAACCATCTGATAATAAATCCAGCCTGT
GCCCTTCTAGACACTTTTGTGGCAAAGACTTATGCTAATGTGCTAGTATTTTTTGATCCATCTGGCCATCCATTTCTTCT
GATTTTGTGGAACAGCAAGTTGAAACAGGCTTCTCTCAGTGTCCTGGAGAAACTAAAGTGTTGCATGAATCTAAGGAAAC
CTACATTCCTATCAAGATGCCTGAAAACACTGTGA
>EqprTAS2R18AP_NW_007673179.1:1439556-1440488
ATGTCACCTGGAATGAAGGTCTCCTTTCTTGTCGTGGCAATAGGATAACTCATCTAAGGAATGCTAGGAAATGGGTTCAT
TGGACTGGTAAACGTCATCGAATGGATCAAGAATGGAAAGGTCTCATCAGGTGATTTCATCCTTTCCAACTTGGCTGTGG
CCAAAGTCATTCAACTGTGGGTAACTCTATTTGGTTCATTTAATGTGGGTCTATCTCCACATCTGTATGCCACCAGGAAA
CTAGCAAAAACGCGGTCACATTCTTTGGACACTTACTGATCACTTTACCTGGTTTGCAACCTGCCTAAGCATTTTCTGCT
TCCTTAAGTTGGCCAATTTCTCCCACTTCTTTTTCATCTGGCTGAAGTGGAGAGTGAACAGAGTGGTTCTTGCAATTTTC
CTGAGGTCTTTTTTCTTACTGTTAATCTCTTAATGCAGGATGCTCTTAGTGAGTTGTGGGTGAATACCTGTAAAGTGCAG
GAAAGAAATATGACTTTGTACTTAGACAGTAATAAAATGTTCTGTCTCAAAAGCCTGTTCTTCTCAGGTTGACCTATGTT
TTCCCCTTTCTTCTGTCTCTGACCTCTTTGTTCCTTTTATTTCTGTCCTTGCTGAGACACACCAAGAATTTGCTGCTCAA
CCTGATGGGTATGAGGGACTCCAGCATAGCGGCCCGTAAAAGGGCTGTGAAGATGGTGACAACCTTCTTCCTCCTCTTCA
TCATTTACTTTGTTTTCACTCTACTAGCAAGTTGGATCTTCCTTAAGGTATAAAGTATCAGGTTATGATGTTTCTCATCG
TAATTTCAACCATCTTTCCCTCAGGCCACTCATTAATTATAATTTTGGGAAACAACGAGCTAAGACAGATGACCTGAGAC
TACTGTAGCATCTTACATTCTCTCTGGGAAAAGCAAAACCTTTATCTTCATAG
>EqprTAS2R18BP_NW_007673179.1:1429301-1430229
ATGTCGATTGTAATGAGGGCTTCCTTTCTGCTCATGGCAACAGGAGAACTCATCTTAGGAATGCTGGGAAATGGGTTCAT
TGGGCTGGTAACCCGCATCAAATGGATCAAGAATGGGAAGAGCTCATCAGCTGATTTCATCCTACCAGCTTGGCTATGGC
CAGAGTCATCCAACAGTAGGTAACACTATTTGGTTCATTGAATGTGGAGCTATCTCCACATCTGTATGCCACCAGGAAAC
TAGCAAAAGCAGTTACTATCCTTTGAGCACTAACTGATCACTTAACTACCTGGTTTACAACCTGCCTAAGCATCTTCTGC
TTCCTTAAGATCGCCAATTTCTCCCACTTCTTTTTCATCTGGCTGAAGTGGAGAGAGAATAGAATGGTTCTTGTGCTTTT
CCTGGGGTCTTTCTTCTGATTGTCTGTTAACCTTTTAATGCAGGATGCTCTTAGTGAGTTGTGGATGAATATCCATAGAG
GACGTGAAAGAAACATGACTTTGCATTAAGTGAAAATAAAATTTTCTATCTTAAATGCCTTCTTCTTCTTACTTGACCTA
TATCATCCCCTTTCTTCTGTTCCTGACCTCTTTGTTCCTTTGTTTCTGTCCGTGATGAGACACACCGAGAATTTGTACCT
AAACCTGATGGATATAAGGGACTCCAGCACAGAGGTCCATAAAAGGACCGTGAAAATGGTGACAACCTTCTTCCTCCTCT
TCATCATTTACTTTATTTCCACTCTAAGAAAAAGTTAGATCTTCCTTAAGGTACAGAGGTTTCAGGTTATGATGTTTGTT
ATGGTGATTTCAACAATCTTTCCTTCAGGCTACACATTAATTATAAATTTGGGAAATAGCAATCTAAGAAAAATCATCTG
AGATTACTGTAAATTCTCTGTGAGAGAAGCAAAAGTTTTATCATCATAG
>EqprTAS2R18CP_NW_007673179.1:1387826-1388770
ATGTCAGTTGGAATGAAGGTCTACTTTCTGATGTGGCAGCAGGAGAACTCATTTTAGGAATGCTGGGAAATGGGTTCATT
GGAGTGATAAACACTATCAGATGGGTCAAGAATGGGAAAGTCTCATCAGCTGACTTCAACCTTACTAGCTTGGCTCTGCC
TAGAATCATTCAACTGTGGTTAACACTATTTGATTCAGTTATAATGGGGCTCTCTCCACATCTGTATGCCACCAATAAAC
TAGCAAAAGTGGTTAGTATTCTTTAGGCACTAACTCATCACTTAACTACCTGGTTTACCACCTGCCTAAGCATTTTCTGC

TTCCTTAAGATAACCAGTTTCTCCCACTTCTTTTTCATCCTGCTGAAGTGCAGAGTGAACTGAGTGGTTTTTGTGCTTTT
CCTGGGATCTTTCTTCTTGTTGTCTGTTACCTCTTAATGCAGGATCAGGAAGCTCTTAATGAGTGGTGGATGAACACCTC
TAGAGCATATGAAAGAAACATGACTTTGTATTTAGATGTAAATAAAATTTTCTATCTTACAAACCTTCTTCTTCTTAATT
TGACCTATGTTATCACCTTTCTTCTGTCCCTGACCTCTTTGTTGGTTTTATTTCTGTCCTTGGTGAGGCCCACCAAGAAT
TTATAGCTCAGCCTGATGGGCATGAGGGACTCCAGCACAGAGGCCCATAAAAGACCATGAAAATGGTGACAACCTTCTTC
CTCCTGTTCATCATTTACTTTATTTCCACTCTAATAGCAAGCTGGATCTTCCTTAAGGGACATAAGTATTAGGTTATGAT
GTTTGTCATGGTGATTTCAACCATCTTTCTCTCGGGCCACTCATTTATTGTAATTTTGGGAAATAGCAAGGTAAGACAGA
TAACGTGAGACTACTGTGGTGTCTAAAATTCTCTCTCGAGATATGCAAAACTTTCAGCTTCATAG
>EqprTAS2R18DP_NW_007673179.1:1368865-1369784
ATGTCAATTGGAATGAAGGTCTCCCTTTTTGTTGTAGCAACAGGAGAACTCATTTTAGAAATGCAGGGAAATGGGTTCAT
TGGACTGGTGAACTCATCAAATGCATCAACAATGGGAAGATCCTATCAGCTGATTTCAACCTTACCAGCATGCTATGACC
AGAATCATTCAACTGTGGGTAACAGCATTTGGTTTATTTAATGTGAGGGCTCTTTCCACATCTGTAAGCCACCAGTAAAT
CAGCAAAAGCAGTTACTATCCTTTGGGCACTAACCAATCACTTAACTACCTGGTTTGCCACCTGCCTAAGCATTTTCTGC
TTCCTTAAGATAACCAGTTTCTCCCACTTGTTCATCTGGCTGAAGTGGAGAGTGAACAGAATGTTTCTTCTGCTTTTTCT
GGGGTCTTCCTTCTTGATGTCTGTTAACCTCTTAATGGAAGATGCTCTTAATGAGTTGTTGATTAGTACCTATTGCATAT
ATGAAAGGAACATGATTTCACTTTTATTTAGATGTAAATAAATTTTCTATATCAAAAGACTTCTTCTTCTTAGCTTGACC
TATGTCCTTTCTTCTGTCACTGACCTCTTTGCTGCTTTGCTGGTGAGACACACCAAGAATTTTCAGCTCAATCAGATGGG
CCGGAGGGACTCCAGCACAGCAACCCATAAAAGGGCCATGAAAATGTTGACAACCTTCTTTCTCCTCTTCAGCATTTGCT
TTATTTTCACTCTAATAACAAGTAGGATCTTCCTTAAGGTACAGAGGTATCAGGTTATGGTGTTTATCATGGTGATTTCA
CCCTTCTTTCCCTCAGGCCACTCATTCATTATAATTTTTGGAAACAGTGAGCTAAGACAGATCACTTGAGACTACTGTAG
CATTTTAAATTCTAAGAAAAGAAAACTTTTATCTTCAGAG
>EqprTAS2R18EP_NW_007673179.1:1335571-1336504
ATATGAGTTGGAATGAAGGTCTCCTTTCTTGTCATGGCAACAGGAGAACTCATCTTAGGAATACTGGGAAATGGGTTCAT
TGGACTGGTAAACTGCATCGAATGGGTCAAGAATGGGAAGGTCTCATCAGCTGATTTCATCCTTACCAGCTTGGTATGGC
CAGAATCACTCAACCGTGGGTGACACTATCTGATTCATTTATAATGGCACTGTCTTCACATCTGTATGCCACCAGGAAAC
CAATAAAAGTGGTTACTATTCTTTGGGCACTAACTGATCACTGAACTACCTGGTTTGCCACCTGCCTAAGCATCTTCTGC
TTCCTTAAGATGGCCAGTTTCGCCCACTTGTTTTTCATCTAGCTGTAGTGGAGAGTGAACAAAGTGGTTCTTATGCTTTT
CCTGGGAACTTTCTTCTTGTTGTCTGTTAACCTCTTAATGCAGGATGCTCTTAGGAGTTGTGGATGAATATCTGTAGAGT
ACACGAAAGAAACATGACTTTGCATTTAGATGTAAATAAAATGCTCTATCTTAAAAGCTTTCTTCTTAGTTTGACCTATG
TTATCCCCTTTCTTCTGTCCCTGACTTCTTTGCTCCTTTTGCTCCTGTCCTTGATGAGACACACCAAGAATTTGCAGGTC
AACTTGATGGGCATGAGGGACTCCAGCACAGAGGCCATAAAAGGGCCATAAAAATGGTGAAAACCTTATTCCCCCTCTTG
ATAATTTGCTTTATTTCCACTCTAATAGCAAGTTGGATCTTCCTTAAGGTACAGAGGTATCATGTTATGATGTTTGTCAT
GGTGATTTCAACCATCTTTCCCTCAGGCCACTCATTAATTATAATTTTGGGAAACAGTGAGCTGAGACAGATCATCTGAG
ACTACAGTAGCATCTTAAATTCTCTATGGGAAAAGCCAAACTTTTTCTCCATAG
>EqprTAS2R18FP_NW_007673179.1:1302774-1303713
ATGTCAGTTGGGATGAAGATCTACTTTCTGATCATGGTAACAGGAGAACTCATTTCAGGAAAGCTTGGAAATCAGTTCAT
TGGACTAGTAATCTGCATTGAATGAGTCAAGAATGGGAAGATCTCAACAAGTGATTTCATCCTTACCAGATGATTTCATC
CTTACCAGGGTGGCCATGGGCAGAATCATTCAACTGTGGGTAACACTATTTGATTCATTTATAATGTGGCTATCTCCACA
TCTGTATGCCACCAGTAAACCAGCAAAAGTGGTTACTATTCTTTGGGCACTAATCGATTACTTAACTACCTGGTTTGCCA
TCTGCCTAAGCGTTTTCTGCTTCCTTGAGATAGCCAATTTCTCCCACTTCTTTTTCATCTGGCTGAAGTGGAGAGTGAAC
AGAGTGGTTCTTGTGCTTTTCCTGGTGTCTTTGTCTGTTAACCTCTTAATGTAGAATGCTCTTAAACAGTTGCGAATGAA
TACCTATAGAGTACATGAAAGAAGCATGACTTTGCATTTAGATGTAAATAAAATTTTCTATCTTAAAAGCCGTCTTCTTC
TTAGCTTGACCTATGTTATCACCTTTCTTCTGTCCCTGACCTCTTTGCTCCTTTTGTTTCTGTACTTAACGAAACACAGC
AAGAATTTGCAGCTCAGCCTGTAGAAAGGAGGGACTCCAGTACAGAGGCCCAGAAAAGACCATGGAAGTGAGGACAACCT

TCTTCCTCTTCTTCATCATTTACTTCATTTCCACTCTAATAGCAATTTGGATTTTCCTTAAGGTCCAGAGGTATCAGTTT
GTCATCGTGATTTCATTCATCTTTCCCTCAGTCCACTCATTAATTATAATTTTGGGAAACAGCTAGATAAGACAGGCCAC
CTGAGACTACAGTAGCATTTTAAATTCTCTCTGAGAAAAGCCAAACTTTTGTCTTCACAG
>EqprTAS2R18GP_NW_007673179.1:1275568-1276492
ATGTCAGTTGGAATTAAGGTCTCCTTTCTGGTCATGGCAACAGGAGAACTCATCTTACGAATGCTGGGAAATGGGTTCAT
TGGACTGGTAAACTGCATCGAATGGGTCAAGAATGGGAAAATCTCATCAGCTGATTTCATCCTTACCAGCTTGGTATGGC
CAGAATCATTCAACTGTGGATAACACTATTTGGTTCATTTAATGTAGGGCTATTTCCACATCTGAATGCCACTAGTAAAT
TAGCAAAAGCGATTACTATTCTTTGGGCACTAACTAATCACTTGCTTGCCACCTGCCTAAGCATTTTCTGCTTCCTTAAG
ATTGCCGGTTTCTCCCACTTCTTGTTCATCTGGCTGAAGTGGAGAGTGAACAGAGTGGGTCTTGTACTTTTCCTGAGGTC
TTTCTTCTTATCTCCTGCTAGTCTCTTAATGCAGGATGCTCTTATTGAGTTGTGGATGAATACGTATGGAGTACATGAAA
GAAACATGACTTTGCATTTACACGTAAATAAAATGTTCTATCTTAAAAGCCTTCTTCTTAGTTGACCTATGTTATCCCCT
TTCTTCTGTCCCTGACCTTTTTGCTCCTTTTATTTCTGTCTTTGGTGATACACACCAGGAATTTGTAGCTCAACCTGAAG
GGCATGAGGGACTCCAGCACAAAGGCTCTTAAAGGGTCATGAAGATGGTGACAACCTTCTTCCTCCTCTTCATCATTTAC
TTTATTTCCACTCTAATAGCAAGTTAGATCTTCCTTAAGGTACAAAGGTTTCAGGTTATGATGTTGTTGTGGTGTTTTCC
ACCATCTTTCCCTCAGGCCACTCATTTATTATAATTTAGGAAACAGCAAGCTAAGATAGATCACTAGAGATTACTGTAGC
ATCTTAAATTCTCTCTTGAGAAAAGCAAAACTTTTAGCTTCATAG
>EqprTAS2R38P_NW_007674652.1:224898-225899
ATGTTGACTCTGCCTTCCATCATAATTGTGTCCTACAAAGTCAAGAATGCATTTCTATTACTTTCATTCCTGGAGTTTGC
TCCGGGAATCCTGGCCAATGCCTTTATTTTCTTGGTGAATTTTTGGTGAGGAAGCAGCCACTAAGCAACTGTGATCTTAT
CCTGCTATGTCTCAACCTCTCTTGGCTTTTCCTGCATGGGCTCTGTTTCTGGGTGCCATACAGCTTACCCATTTCCAGCA
GATGGAAGACCCACTGAGCCTCAGCTACCAAACTATCATCATGCTCTGAATGATCACAAATCAAGCTGGCATCTGGCTTG
CCACTTGCCTCAGTCTCCTCTACTGCTCCAAGATTGTCCATTTCTCTCACACCTTCCTGCTCTGCTTGGCAAGCTGCATC
TCCAGGGAGATCCCCAAGATGCTCCTGGGTGCTATTCTTTTCGCCTGTGTCTGCACTGTCCTCTGTTTGCGGGACTTTTT
CAGTACATCTCACTCCACAGTCTCAACTATGCTATTCATGAATAACAATTCACAACTCAATTTGCAAATTGCAAAACTCA
ATTTCTTTCATTCCTTCCTCTTCCACAGCCTGGCGTCCATCCCACCTTTCTTATTTTTTTCTGTTTCTTCTGGTATGTTA
ATTATCTTCCTGGGGAGGCACATGAGGACAATGAGGGCCAAAACTAGAGAGCCTTGCCATCCCAGCCTGGAGGCCCAGAT
CAAAGCACTCAAATATCTCTCATCTCCTTTCTCTGCCTCTATGTGGTGTCGTTCTGCGCTGTCCTCATCTCAATACCTCT
ACTGATGCTGTGACATAGCAAGATTGTCGTAATGGTCTCTGCATGGATAATGGCAGTTTGTCCCTCGGGACATGCAGCCA
TCCTGATCTCAGGCAATGTTAAGCTGAGGGGAGCTGTGGAGACCATTCCACTCTAGGCTCAGAGCAGCCTAAAGGTAAGG
GCAGACTACAAGGCAGATCCCAGGACACCAGATCTATGTTGA
>EqprTAS2R408AP_NW_007673179.1:1384195-1385048
ATGAGAAGTTTACTACAAAGCATTTTTTTTTTATCCTAATAATGACAGAATTTATTCTGGGAAATTTTGCCAGTGGCTTC
ATAGCACTGGTGAACTGCACTGGCTGAGTCAAGAGATGAAAGATGTCCTCAGCTGATCAAATTCTCACTGCTCTTGTGGT
CTTGAGAATTGGTTTGCTCTGGGTAATATCAATAAATTGGTATACAACTGTACTTACTCCAGTTTTATATTTTAGAAGTA
AGAATTATTGTTTATATTGCCTTCACAGTAAGCAGCCATTTTAGCATGTGGCTTGCTACTAGGCTCAGCATATTTTATTT
GCTCAAGATAGCTAATTTCTCTAGCTTGAGAGTTAAAATTTTATTTCTCAGAATACTGCTGGGGAGTTTGGTCTTTTTCT
TATCTTGCAGTGCTATTCATAGATGAGAATACACAGACTAATGCACATGAAGGAAACATCACTGGGAAGACCAAATAGAG
GGATTTTGTAGGCCTTTCAAATATGACTCTACTCACGCTAGTAAACTTGATACCCTTTACCATGTCCCTGACATCTTTTC
TGCTGTTAATCATTTCCCTCAATGGCAAAGGATCCCAAGATGCCAGGACCAAGGTCCACATAAGAGCCATGCAAACTGTG
GTCTCCTTTCTCTTGCCATATGCCAGTTACTTCCTGGCTCTAGTTATTTCAGTTTGGAACCTAACAGGTGGCAGAATGAA
CCAGTTTTTATGCTTTGCCAAGCTCTTAGAATGCTGTATCCTTGAAGCTACTTATTTATACTGATTTGGGGAAACAAAAG
TTAAGACAGGCCCTGAAAAATATGAGATGGATTATAAGATTACCATATCATTAA
>EqprTAS2R408CP_NW_007673179.1:1365222-1366130
ACAATAACTTTACTACCAATCATTTTTTCCGTCCTGAAAATGACAGAATTTATTCTAGGAAATTTTGCCAATGGCTTCAT

AGCACTGGTGAACTGCATTTACGGGGTCAAGAGATGAAAGATGTCCTCAGCTGATCAAATTCTCATGGCTCTTTCCATCT
CCAGAACTGGTTTGCTGTGGCTCATGTTAATAAATTGGCATGCAACTGTACTTACTCCAGTTTTATATAGTTTAGAAGTA
AGAATGATTGTTCGTATTGCCTGGGCAGTGAGCAACCATTTTAGCATGTGGCTTGCTACTAGCCTCAGCATATTTTATTT
GCTAAAGATAGCTAATTTCTCAAGCTGTATATTTCTTTACTTAAAGTGGAGAGTTAAAAGTGTACTTCTCGGAATACTGT
TGGGGACATCGGTCTTTTTGGTTTCTTATCTTCCAGTGCTATGCATAGATGAGAATATACAGACTAATGAGTATGAAGGA
AAGATCACTTGGAAGACCAAATTGAGGCACACGGTATACCTTTCAAATATGACTCTATTCATGCTAATAAACTTTGTACC
CTTTGTTATGTCCTTGACATGTTTTCTGCTGTTAATTATTTCCCTATGGAAACATCTCAGGAAGATGCAGCTCAATGACA
AAGGATCCCAAGATCCCAGCACCAAGGTCCATCTAAGAGCCATGCAAACTGTGGTCTCCTTTCTCTTCCTATTGTCCTGT
TACTTCCTGACTCTAGTTATCTCAGTTTGGAATTCTAATAGGCTCCAGAATGAATGGCTTCTCATGCTTTGCCAGGCTCT
TGGAATGTTGCATCCTTCAAGCCATTCGTTTATCCTGATTTGGGGAAACAAGAAGCTAAGACAGGCCTTGAAAAATATAA
GAAAGATGATAAGATTACCATACCATTAA
>EqprTAS2R408DP_NW_007673179.1:1299096-1300005
ATGGTAACTTTACTACCCAGCATTTTTTCCGTCATAGAAATGACAGAATTTATTCTGGGAAATTTCGCTAATGGCTTCAT
AGCACCGCTGAACTTCATTGCCTGGGTCAGGAGACGAAAGATGTCCTCAGCTGATCAAATTCTCATGGCTCTGGCGGTCT
CCAGAATTGGTTTGCACTGGGTAATATTAATAAATTGGTATACAACTGTGCTTCATCCAGTTTTATATAGTTTTGAAGTA
AGAATTTTTGTTCATATTATCTGGGCAGTAAGCAACCATTTTAACATCTGGCTTGCTACTAGCCTCAGCATATTTTATCT
GCTCAAGATCGCTAATTTCTCTAGCCTTATATTTCTTTACCTAAAGTGGAGAGTTAAAAGTGTAATTCTGGTAACACTAT
TAGGGACTTTGGTCTTTTTGGTTTTTTTATTTTGCAGTGATATGCATAGATGAGAAAACACAGACTAATGTGTATGAAGG
AAACATCACTGGGAAGACCAAACTGAGGGATATTTTACGTGTTTCAAATATGACTGTATTCGTGCTAGTACGCTTATTAC
CATTTTCTACGTCCCTGACATGTTTTCTGCTGTTAATCATTTCTCTATGGAAACATCTCAAGAAGATGCAGCTTAATGGC
AAAGGATACCAAGATCCTAGCACCAAGGTCCACATAAGAGCCATGCAAACTGTGTTCTCCTTTCTCTTGCTATATGCCAG
TTACTTACTGGCTCTAGTTATCTCAGTTTGGAGTTCTAATAGGCTGCAGAATGAACTGCTTCTCATGCTTTGTGAGGTTC
TTGCAGTACTGCATCCATTAAGCCACTTGTTTATCCTGATTTGGGGAAACAAGAAGCTAAGACAGGCCTTGAAAAACATA
AGAAAGATTACAAGATTACCATACCATTAA
>EqprTAS2R408GP_NW_007673179.1:1271618-1272774
ATGATAACTTTACTACCAAGCATTTTTTCTGTCCTAATAACGACAGAATTTGTTCTGGGAAATTTCGCCAATGGCTTCAT
AGCACTGGTGAACTGCATTGACTGGGTCAAGAGACAAAAGATGTCCTCAGCTGATCAAATTCTCACAGCTCTGGCGGTCT
CCAGAATTGGTTTGCTCTGGGTAATCTTTTTTTTTTTTTTTTTAAGATTTTATTTTTTCCTTTTTCTCCCCAAAGCCCCC
CGGTACATAGTTGTGTATTCTTCGTTGTGGGTTCTTCTAGTTGTGGCATGTGGGACGCTGCCTCAGTGTGGTCTGATGAG
CAGTGCCATGTCCGCGCCCAGGATTTGAACTGACGAAACACTGGGCCGCCTGCAGCGGAGTGCGCGAACTTAACCACTCG
GCCACGGGGCCAGCCCCTGCTCTGGGTAATCTTAATAAATTGGTATATGACTGTGCTTCCTTCAGTTTTTCATAGTTTAG
AAGTAAGAATTATTGTTTTTATTGCCTGGACAGTAAGCAACCATCTTAACATCTGGCTTGCTACTAGCCTCAGCATATTT
TTTTACTTGCTAAAGATAGCTAATTTCTCTAGCCTTATATTTCTTTACCTAAAGTGGAGAGTTAAAAGTGTACTTCTCGT
AATACTGTTGGGCGCTTCGGTCTTTTGGGTTTCTCATCTTGCAGTGCTATGGGTAAATAATAATGTGCAGACTAATGAAT
TTGAAGGAAACATCACTCAGAAGACCAAATTGAGGGATATTGTAGCTCTTTCGAATTTGACTCTATTCACGCTAGTAAAC
TTCATACACTTTTCTATGTCTCTGACATGTTTTCTGCTGTTAATCATTTCCCTGTGGAAACATCTCAAGAAGATTCAGCT
TAATGGCAAAGGATCCCATGATCCCAGCACCAAGGTCCACATAAGAGCCATGCAAACTGTGGTCTCCTTTCTCTTGCTAT
ATGCTGTTTACTTCTTGGCTCTAGTTATCTTAGTTTGGAGTTCTAATAGGCTGGAGAGTCAACTGCTTGTCATGCTTTGC
CGGGCTTTTGAAATACTCTATCCTTCAAGCCATTCATTTATTCTGATTTGGGGAAACAAGAAGCTAAGACAGGCCTTGCA
AAATATACGAAATATTATAAGATTACCATACTATTAA
>EqprTAS2R408HP_NW_007673179.1:1435365-1436389
GTGGCAACTTTACCACCAAGCATTTTTTCTGTCCTAATAACGACAGAATTTATTCTGGGAAATTTTGTCAAGGCTTCAT
AGCACTGGTGAACTGCATTGACTGGGTCAAGAGACAAAAGATGTCCTCAGCTGATCAAATATTCACACTCTGGTGGTCTC
CAGAATTGGTTTGCTCTGGGTACTATCAATCACTTGGTATACAACTGTGCTCCTTCAAGTTTTATATAGTTTAGAAGTAA

AAATTATTGCTTGTATTGACTGGACAGTGAGCAACCATTTTGATGTCTGGCTTGCTACTAGTCCCAGCATACATTTTTTT
TTTTTTTTTAAAGATTTTATTTTTTTCCTTTTTCTCCCCAAAGCTCCCCCGGTACATAGTTGTATATTCTTAGTTGTGGG
TCCGTGAGGTGTGGTATGTGGGATGCCGCCTCAGCGTGGCTCAATGAATGGTGCCACGTCCGCGCCCAGGATTCGAACTG
ACGAAACACTGGGCCGCCTGCAGCGAGCGCTCTAACTTAACCACTCAGGCAAAGGGCCAGCCCCCTTGTGATAAGAACTT
TTAAGATTTACTCTAGTCCCAGCATACATTTTTGTTCAAGATAGATAATTTCTCTAGCTTTGTATTTCTTTACCTAAAGT
AGAGAGTTAAAAGTGTACTTCTCGTTATGTTGTTAGGGACTTTGGTCTTTTTGGTTCCTCATCTTGCAGTGCTATGCATG
GATAATAATATGCAGACTAATGAGCATGAAGGAAACATCACTCAGAAGGCCAAATTGAGGGACATTTTATACCTTCCAAA
TGTCACTCTATTCACACTAATACACTTCATACCCTTTACTATGTCCCTGACATCTTTTCTGCTGTTAATCATTTCCCTGT
GGAAACATCTCAAGAACATGCAGCTCAATGGCAAAAGATTCCAAGATCCCAGCACCAAGGTCCACATAAGAGCCATGTAA
ACTGTGCTCTCCTTTCTCTTTCTATATGCTGGTTACTTCCTGATTCTAGTTATCTCTCTTTTTTT
>EqprTAS2R408IP_NW_007674042.1:556449-555521
ATGATAAGTTCACTACGGAGCATTTTTTCCAACTAGCAACTGAAGAATTTGTTCTAGGAAATTTGGCCAATGTCTTCATA
GCATGGGTGCATAGAACTGCATTGACTGAATGAAGAGACAAAAGATGTCCTCAGCTGATTAAATTCTAACTTCTCTGACA
GTCTCCAGGATTGGTCTACTTTGGGTAATATTAATAAATTGGTATTCAATTGTTTAACCCAGCTTTACATAGTTTAGAAG
CAAGACTATTTATATTGCCTGGTCAATAACCAACCATTTTGGCATCTGGCTTGCCACTAGCCTCAGTATATTTTATTTGT
TCAAGATAGCAAATATCTCCAGCTTTATTTTTCTTCACTTAAAGTGGAAAGTTAAAAGAGTGGTTCCCGTGATACTGTTG
AGGACTTGGATCCTTTGGTTTTTCATCTAGTAGTGGTAAGCATAGATAAAAGTATGTGCATGAATGACTACAAAGGATGC
ATCACTCGGAAGACCAAATTGAGGGACATTTTACGCCTTTCCAATAACTTCATACCCTTTATATGTCACTGACATCTTTT
CTGCTGTTAATATTTTATCTATGGAAACATCTTAAGAAGATGCAGCTCAATGGCAACGATCCCAACATCCCAGTACCAAG
GTCCACAAAAGAGCCATGCAAACTGCAGTCTCCTTTCTCTTGCTATATGCTGGTTACTTCCTGGCTCTAGTTATCTCAGT
TTGGAGTTCTAATAGGCTGCATAACAGACCACTCATTTATCTTGATTTCAGGAAAGGAGAAGATAAGACAGGCCTTTCTG
TCATTTCTGTGGCAGCTGAGGTGCTGGCTGAAAAAAAGAGAAGTAAGTGGGCTAGTATGTGTCTTCTAGCAAGAAACAAA
CTATCAGTCTTTATAAGCTTTATATATATGTGACAATAAATAATAATGA
>EqprTAS2R60P_NW_007676765.1:137839-136882
ATGAATGGACATGACATGGTTTCAGGATGTTGGTGATTGATAGGAGAGCCATCACCTTGGCTATCATTTTATTCCTTTTG
TGCCTGGTGGCAGAGATGGGCAATGGCTTCATCACTGTGGCACTGGGCATGGAGTGGTTACTATGGAGAACATTGTCACC
TTGTGATGTTATTGGTCAGCCTGGGAGTCTCTTGCTTCTATGTGCAGTGGGTGGTGATGAGTAAGAACATTTACGCTAGA
ATTGTGTGTCCACTGGCCCTTCCATACAACCCTGTACTATAGTTTCTAGCCTTATAGTGGGACTTCTTGAACACTACCAC
CTTTTGGTTCTCTACCTGGCTCAGTGTCCTCTATTGCAGGAAAATTTCAACCTTCACTCACCCTGTCTTCCTCTGGCTAA
AACAGAAGGTGTCTGGGTTGGTTCCCTTGATGCTGCTCAGCTCCATGGGGTTCTCTAGCTTGAGCACCATCCTATTTTTC
ACAGGCAACCAGAGCCAATATCAGAACTTTTTAAGGAGAGGTCTGCAATATTGGAATATCATCAGGAATCCTATAAGGAG
ATCACATGAGAAATTCTACTTCTTTTGTTTAAAATTCGTTACTTAGGCAGTTCCTGCTGTTGTCTCCCTCACTGGTAGGA
TTTTAGCTCATCATGTCTCTCGGAAGACACACCAATAAGCCCTTTCTGTCTGTCTCGGGCTTTTGCCATCCTAGTGCCCA
GGCACACATCAGGACTTTCCTGGCTCTCATCTCCTTTGCTATCTTCTTCAATTCCTATTTTCTGTCACTGGTGCTCAGTG
CTGCAGTTATTTTTCCATCTTAGGAATTTAGGTACTGGGTGTGGCAGACAGTGATTTATCGGTGCACAGTAGTCCACCCC
ATCAGTCTACTCTTGAGCAACACCAGGCTGAGAGTGGTGCCGGAGAGGAGCTGCTCCTCAAGGCATGGGGCATCTTGA
>EqprTAS2R62BP_NW_007675651.1:240791-241692
ATGTCCTCCTCACCCATGTTGATCTTCATGGTCATCTTTTTCTTGGAGTCCTTGGCTGCAAATTTGCAGAATGGCTTCAT
GGTTGCTGTACTGGGCAGGGATTGGATGCAGTGCCACACATTGCTCACAGATGACATGACTGTGGCCTGCTTAGCTGCCT
CCAGGTTCTTCCTGCATTGGATGATTCCCCTGAATAAACTCCTAGCCTCCTTTGATTTTTGCTCCAAAATTCACTATTTC
AACATCCCTTGGAGCTTCATCAACACACTTTCTGCCTCCCTGCCTGGCGTGCTGTCTTCTACTGTGTGAAGATCTCGTCC
TTCTCTCATCCAGTCTTCTTCTTGCTGAAGTGGAGGATTTCTCAGTCACTGCCAAGGCTGCTGCTGGGTTCCCTGATAAT
ATCTGGTGTGACAGTCATCTCATTAGCCACTGGGCATAGCATTCTTGTGTAGATGGTTGCCTGCCATATTTCCTATGGAA
ACAGCACTGTGGCTGATATAACAGAGCACATCTCTTTGTACATTTTTCTGGCTAAGATACTGCTTGTGTTATTGATTTCC

TTCCTCCTGTTCCTGGTGTTCACTCTCTTGCTCATGTTCTCACTTCACCGGTCCTTGAGGCAGATGAGGGATCATAGACC
TGGCCCACACGATCCCAGCACCCAGGCTCACACTATGGCCCCGAAGTCACTTGCTGTCTCTCTCGTCTTCTACACATAAT
ATTTCTTGTCCCTCATTATTGTTTCTATGCATATCACAACACTTCATAATCACTGGAACTGGGTCTAGGATGTGGTGATC
TATGCAGGCATCTGTCTATGCTCCAGCATCCTGGTACAAAGCAGCCTCAAGCTGAGAAAGGCCCTGAAGATGATGTTGTG
GAAATCCCTGGACAAAAGGTGA
>EqprTAS2R62DP_NW_007676765.1:159744-158792
ATGCCCTCCTCATCCATGTTGATCTTCATGGTCATCTTTTTCATGGAGACCTTGGCTACAATGTTGCAGAATGGCTTCAT
TGTTGCTGTACTGGGCAGGGAGTGGGTGAGATGCTGCACACTGCCTTCAGGTGACATGATGGTATCCTGCCTGGCTGCCT
CCTTATTCTGCCTGCATGGGATGGCCCTCCTAAACAATCTCATTGACTCCTTTAACTTTTGTTCCAAAGTTTACTATTTC
AACACTTCCTGGGGCTTTATCAACGCTCTTACTTTCTGGCTTACTGCCTAGCTTGCTGCCTTCTACTGTGTGAAGATCTC
ATCCTTCTTTCATCCTGTCTTCCTCTGGCTAAAGTGGAGGATTTCTCGGTCAGTGCCCAGGCTGCTGCTGGGCTCCATGA
TTATATCTATCGTGCCAGTCATTCCATTAGCCACTTGGAATAGCATTCTTGTGCAGCTGAGTGCCTCCCAGAGTTCCCAT
GGAAACGGCACCCTATGTGAAAGAATACAGACCATCTCTCTCTACTCTTTTTTGCCTACAGTGCTTATGTTGTTGATTCC
CTTTCTCCTGTTCCTTGTGTCCACCCTCTTGCTCATATTCTCACTGTACAGGCACTTGGGGAAAATGAGGGACCACAGAT
CCGGCCCATGTGATCCCAGCACCCAGTCTCACACCATGGCCCCGAAGTCATTTACTGTCTTCCTTGTCTTCTACACATCA
TATTTCCTCTCCCTGATTATTGCTTTTATGAAAATCACAACACTGAAGAATCAGAGCCATGGGGCCTGGGAAGTGGTGAC
CTATGCAGGCATCTGTCTGCATTCTAGCATCCTGGTGCTAAGCAGCCCAAAGCTGAGAAAGGCCCTGAAGAGAATCCTTT
GCAAGTCCCTGGACAAAAGATGGTTCATCACAAGTATCAATATCAATAATCAGTATCAATAGATAAGCCCTGA
>EqprTAS2R67P_NW_007673179.1:1238287-1239263
CCGCCATCTGGAATTGAAAGCACTTTTCTGATAGTGGCAATAGGAGAATTCATAACTGGAATGTTGGGGAATGCGTGCAT
TGTACTGGTTAACTGCATTGACTAGGTGAAGAGTCAGCAGCTCTCATTAGCCGACTGCATCCTCACCAGCCTGGCTATCT
CCAGAACCAGTCATCTTTGGGTAATACTACTTGGTTAATTTGTCATATTGCCACATCTATATGCCAATGATAACTAGCAA
ACTTGGTTGGTATATTTTGGACACTGACCAATCACCTAGCTACCTGGTTTGCCGTCTATCTAAGCTTTTTCTACTTCTTT
AAAATAGCCAGTTTCTCTCACCCCTGCTTCGCCTGGCTGAGGTGGAGAATTAGCATAGTGTTACTTGTGCTTCCACTGGA
GTCTTTGTTCTGTTTTTCAACCTTGAACTGAAAGGTACAATTAATGGTTTCTCAATTTAGTCCTCAGATGTAAGTGAACT
TCTGTATCTTAACAGCTCGTTTGTTGTCAGTTTGATCTACTTAATCCCCCTTCTTCTGTCCCTGAACTCACTGCTCCTTT
TATTTCTCTCCTTGATGAGACATACCAGGAATTTGCCGCTGGACTCTAGCTCTTAGGGACCTCAGCTCAGAGACCCATAA
AAGGGCCACGAAAATGGTGATGTCTTTCCTCCTCCTCCTCATGGTTCACTTTTCTTCCACTCTATTAACAGGGTGGGTTT
TCCTTATACTGAAGAAACATTGGGCCAATTTGGTTAATGTCAACTCTTTTTCCTTCAGGCCACACATTTATCCTAATTTT
AGGAAACAGCAAGCTGAGGCAAAATGCCTTAGGACTATTGTGGCATCTTAATTGTCACTGGAAAGGGTGAAACTTTTAG
>BogrTAS2R1_NW_005395147.1:630609-629719
ATGCTGGAGTCTCACCTTGTTAGCCACCTTGTTTTGGCAGTGGTACACCTTCTCTTGGGGATTTTAGTAAATGGCATCAT
TGTGATTGTGAACAGTACTGACTTCATCAAGCAGAGAAAGTTGATCCCACTGGATCTCCTTGTTTCCTGCTTGGCGATTT
CCAGGATGGGAATTCAGCTGGCCTTCTTCTACACTAACCTGGCTCTTCTTTCCTTGATCAAATTCCCTCAATTTACTGAG
ATGCTTGTAGTTTTCACATTTGTAAATGATTTGGGACTTTGGTTTGCCACCTGGCTCAGTGTCTACTACTGCACCAAGAT
TGCTACCATCGCTCACCCGCTCTCGTTCTGGTTGAAGATGAAGATCTCCAAGTTGGTTCCTTGGCTGATTCTTGTGTCCC
TGCTGTATGCATGTAGTACTTCTGCTATGCATGTCAAATATAAGTGGGTATTTTACGGAGAAGACTTCCTGGGCCTTTTC
TTCCCAAATGTAACAACTCACATCAAAGTAACCCCTACCTTACAGTTTGCCTTTCTGTTTGCTGAGTTTGCATTGCCATT
GTTCATCTTCCTGATTTCTTCTCTGCCCTTGATATTTTCCTTGGGAAGACATGCCTGGCAGGTGAGAAACACATGGACAG
GCCCCAGAAACCCTCACACACGTGCGTACATCAGGGCCTTTCTCTCCATCCTGTCCTTCTTGGCCCTCTATCTCTGCCAC
TACCTGATCATTGCTTTGATCTTTTTTCAAATTTTTAACCTCAGAAGCTTTCTATTTCTGTTCTGCACCTTCGTGGTTGG
TTCATACCACTCCGTCCACTCTATTACTTTAATTTTAGGAAACCCGAAAATGAAACAAAATGCAAAGGCATTGCTCCTCC
TCAGAAAGTGA
>BogrTAS2R2_NW_005395799.1:338033-337125

ATGATCTCTTTGTCAGGTATTCCTCATGTTATCATCATGTCAGCAGAATTTATCACAGGGGTTACAGTAAATGGATTTCT
TATAATCATCAACAGCAATGAATTGGTCAAAAGCAGAAAGCTAACACCAATGCAACTCCTGTTCGTATGTATAGGGATAT
CTAGATTTGGTCTACAGACGGTGTTAATGGTACAAGGTTTTTCTCAGTGTTCTTTCCACTCTTTTATAGCGCAAAAATT
TATGGTACACCAATGCTGTTTTTTTGGATGTTTTTCAGCTCTGTCAGTCTCTGGTTTGCCACCTGTCTCTCTTTATTTTA
CTGCCTCAAGGTTACAGGCTTTACCCAGTCCTGTTTTCTTTGGCTGAAAGTCAGGATCTCAAAGTTAATGCCTTGGATGC
TTCTGGGAAGCCTGCTGACCTCTGTGAGCATTGCAGCTCTGTGTGTCAAGGTGGATTACCCTAAAATTGTGGATATTGAT
GTCCTCGGGAATGCCACAGCTAAGAGGACTAAACTCAACACAAAGCAAATTAATGAAGTTCTTCTCATCAACTTGGCATT
AATATTTCCTCTGACTATATTTATAATATGCACTGTTATATTATTAATTTCTCTCTACAAGCACACTCATCGGATGCAAA
ATGGACCTCTTGGTTTTAGAAACACCAGGACTGAAGCCCATATTAATGCATTAAGAACAGTGATAACATTCTTTTGCTTC
TTTATTTCTTACTTTGGTGCCTTCATGGCAAATATGACATTCAATATTCCTTATGGAAGTCATTGCTTCTTTGTGGTGAA
GGATATTATGGCAGCATATCCCTCTGGTCATTCAGTTATAATGATTTGGAGTAATTCTAAGTTCCAGCAACCAATCAGGA
GACTTCTCTGCCTAAGAAGGAGTCAATGA

>BogrTAS2R3_NW_005394292.1:207661-206711

ATGTTGAGACTCAGCAATATGGGGTTTCTGGTTCTGACCACCATTCAGTTCATCCTGGGAATGCTGGGGAATGGTTTCAT
AGGGTGGGTCAATGGCAGCAGCTGGTTCAAGAGCAAGAGGATCTCTTTGCATGACTTCGTTATCACTAACCTGGCTGTCT
CCAGGATTGTTTTGCTGTGGATTCTCTTGATCGATGGTATTTTACTGGTGTTCTCTCCCAAACTACATGATGAAGGGATA
ATCATGCAAATTATTGATGTTTTCTGGACATTTACAAACCATCTGAACATTTGGCTTACCACCTGTCTCAGTGTCTTCTA
CTGCCTGAAAGTGGCCAGTTTCTCCCATCCTATGTTCCTGTGGCTCAAATGGAGAGTTTCCAGGGTGGTTGTATGGATGC
TGTTGAGTACCCTGCTGTTATCGTGTTGCAGTGCCATCTCTCTGATCCGGGAATTTAAGATCTATTCTGTTCTTGGTGGA
ATTGATAGAACCGGGAATATGACTGAACTTTTTACAAAGAAAGAAAAAGAATATAAACTGATCCATGTTCTTGGGACTCT
GTGGGACCTCCCTCCCCTAGTCATATCGGTAATCTCCTACTTTCTGCTTATCCTCTCCCTGGGGAGGCATATGCGGCAGA
TGCATCAAGACTGTGCCAGCTCCAGAGATCTCAGTACCGAGGCCCACAGGAGGGCCATCAGAGTCATCCTCTCCTTCCTC
TTTCTCTTCCTACTCTACTATCTTTCCTTTTATGTTTTAACATCCAGTTATTTCTTACCAGCAACTAAGATGATTGCAAA
GATTGGAGAAGTAATTGCAGTGTTATATCTTGCTGGCCACTCCTATGTTCTCATTCTGGGAAATAGTAAGCTGAAGCAGA
TGTTTGTGGCGATGCTCCGGTGTGAGCCTGGTTGTCTGAAGCCTGGATCCAAGGGATCTGTTTATCCATAG

>BogrTAS2R7_NW_005396436.1:295949-295011

ATGTCAAGTGAAGGGCAGAGTATCTTAATGCTCATAGCAGCTGGGGAATTTTCACTGGGGATCTTAGGGAATGCATTCAT
TGGACTGGTAAACTGTGTGGACTGGATCAAGCACAAGAAGATTGCCTCCATTGATTTAATCCTCACAAGCCTGGCCATCT
CCAGAATTTCTCTCTTATGTATAATACTATTGGATTGTTACATATTGGTCCTGTACCCAGATGTCTATACTGGTGGTAAA
CAAATGAGAATCATTGACTACTTCTGGACACTAACCAACCATTTAAGTGTCTGGTTTGCCACCTGCCTCAGCATTTTCTA
TTTCCTCAAGATAGCAAATTTCTTCCATCCCTTTTTCCTCTGGATGAAGTGGAGAATTGACAGCGCAATTCCTAGGATCC
TGCTGGGGTGTTTGGTCTTCTCGGTGTTCATTAGCCTTCCTGTCATTAACAATTTGGATGATGATTTCAGGCATTGTGTC
AAGATGAAGTTGAAAACAAATTTAAGTCGAAGATGCAGAGTACATAAAGCTCAGCATGCGTCTATCAAGATACGTCTCAA
TCTGTTGACACTACTTCCCTTTTCTGTGTCCCTGATCTCATTTCTCCTCCTGATCCTCTCCCTGTGCAGACACACCAGGC
GAATGCAGCTCCGTGCCCCAGGGAGCAGAGATCCCAGCACGGAAGCTCACGTGAGCGCCATGAAGGCTGTCATCTCCTTC
CTCCTCCTTTTCATTGCCTACTACTTGGCCTATCTTGTGGCCACGTCCAGCTACTTTATGCCAGAGACTGAATTAGCTGT
GATCGTTGGTGAGTTGATAGCTTTAATCTGTCCATCAAGCCATTCACTCTTCCTAATTCTAGAGAACAAAAAATTAAGAC
AAGCATCTCTAAGGGTGCTTTGGAAGGTAAAATGTATCCTACGAAGAAGGAATTGCTAA

>BogrTAS2R10A_NW_005393569.1:350370-351269

ATGCTGAGTATAGTAGAAGGCCTCCTCCTTTTTGTAGCAGTTAATGAGTCAGTATTGGGGGTTTTAGGGAATGGGTTTAT
TGGACTAGTAAACTGCATTAACTGTGTGAAAAATAAGAAGATCTCTACACTCAGCCTTATTCTCACTGGCTTAGCCTCTT
CTAGATTTTGTCTGATATGGATAATAACTACAGATGCATATGTGAGAGTGTTTTCTCCAGATATTTATTTGTCTGGTAAT
CTAAGTCAATATATAGCTTACTTATGGATAATTATGAATCAATCAAGTGTCTGGTTTACCACTAGCCTCAGCATCTTCTA
CTTCCTGAAAATAGCCAACTTTTCCCACTGCATTTTTCTCTGGCTGAAGGGTCACATCACTGAGATTCTTCTTCTTCTAA

TGGGATGTTTGCCCATTTCATGGTTATTTACTTTTCCAAACATTACAATGCCTTTTATTAATAATATTATGAAGAACAGA
AGCACAAACGGGTTGGTCACCATGCATAAAAGTGAATACTTTATAAATCAGATTTTGTTCAATCTTGGAACATTTCTTGT
CTTTGTACTATGCCTGATTACATGTTTCTTAATAATCGCTTCCCTTTGGAGGCACAACAGGAGGATGCAATTGAATGCCA
CAGGATTCAGAGACCCCAGTACAGAAGCACACATCAAAGCAATGAAGATTTTGGTGTCTTTTATCATCCTCTTTATCCTG
TATTTTGTAGGCACTGCCATACAAATATTAAGTGTGACAGTGCCTGAAAACAAACTGCTATTTATTTTTGGTATGACAAC
CACCATCCTCTATCCCTGTGGACACTCATTTATCCTAATTCTTGGAAACAGCAAGCTTAACCAAGCCTCTTTGAGGGTAC
TGAAGCTATTAAAGTGCTAG
>BogrTAS2R10B_NW_005393569.1:311107-312039
ATGCTGAATATAGTGGAAGGCCTCCTCATTTATGTAGCAGTCAGTGAATCAGTATTGGGGGTCTTAGGGAATGGATTTAT
TGGAGTTGTAAGCTGCATTGACTGTGTGAAAAGCAAGAACATCTCTACTGTCAGCCTTATTCTCACTGGCTTAGCCTCTT
CCAGATTTTGCCTGATATGGATGATAATTACAGATGCATATATAAGGATGTTTTTTCCAGATATATATTTGTCTGGTAAT
ATAAGTCAATATATAGTTTACTTAAGGATAATTATGAATCAATCAAGTACCTGGTTTGCCACCAGCCTCAGCATCTTCTA
TTTCCTGAAGATAGCCAATTATTCCCACTGCATTTTTCTCTGGCTGAAGTGTCACATCAACAGGGTTCTTCTCCTTTTCA
TGGGGTCCTTGCTTATTTCATGGTTATTTGCTTTTCCAAGCATTGCAAAGCCTAGTACCAATAATATTATGAAGAACAGA
AGCACAACCTGGCTGATCACCATGCATAAAAGTGAATACTTGACAAATCAGATTCTGCTCAATATTGGAGTCATTCTTGT
CTTTGTACTATGCCTGATTACATGTTTCTTATTAATCACTTCCCTTTGGAGACACAACAGAAAGATGCGATTGAGTGCCA
CAGGATTCAGAGATCCCAGCACTGAAGCACATATCAAAGCAATGAAGATTTTGGTGTCTTTTATCATCCTCTTTATCTTG
TATTTTGTAGGCACTGCCATACAAATATCAGGTAGTAGTACTATGCCTGAAAACAAACTGTTGTTCATTATTGGTATAAC
AACCAGACTCCTCTATCCCTGGGGACACTCATTGATTCTAATGCTAGGAAACAGGAAGCTGAAGCAAGACTCTTTGAGGG
TACTGAAGCCATTAAAGTGCTGGGAAAAAGAGAAACTTCTTAGAATTCCATGA
>BogrTAS2R10C_NW_005393569.1:361925-362854
ATGCTGAGTGTACTGGAAGGCCTCCTCATTTTTGTAGCAGTTAGTGAGTCAATATTGGGGGTTTTAGGGGATGGATTTAT
TGGACTTGCATACTTCATTGAATGTGTGAAGAACAAGAAGTTTTCTACTATCAGCTTTATTCTCATGGGATTGGCTACTT
CCAGAATTTTCCTGATAGGGTTAATAACTACCGATGGATTTGTGAAGATTTTTTCTCCAGAAATGTATTCCTCTGGTTAC
CTAATTGACTGTATTACTTACTCATGGGTAATTCTGAATCCAGCAAGTGTCTTTTTTGCCACCAGCCTCAGCATCTTCTA
TTTCCTGAAGATAGCCAATTTTTCCCACCACATTTTTCTCTGGTTGAGGAGTGACGTCAAAAGGGTTCTTCTCCTTCTGA
TGGGATACTTGCTTATTTCATGGTTAGTTACTTTCCCACTAACTATGAAGATAATTAGTGATTCTAGAGCAAAGAATAGA
AGTGTAGTCTTTTCAGTTGAAGTGCATAAAGGTGAATTCTTTAGAAACCAGATTTTGCTCAATCTTGGAACCCTTACCAT
CTTCATACTATGCCTGATTACATGTATCTTATTGTTCATTTCCCTTCGGAGGCACAACCAGAGGATGCTACTGAATGCCA
CAGGATTCAGAGACCCCAGCACAGAAGCACATATCAAAGCAATGAAAGTTTTGATATCTTTTATCATCCTTTTTATTTTG
TATTTTATAGGCATTACCATAGAAATATCATGCACTACTATGTCAGAAAGCAAGCTGTTGTTTATTTTTGGTCTGACCAT
CACTGCCCTCTATCCCTGGGGACACTCATTTATCCTAATTCTAGGAAACAACAAGCTAAAGCAAGTTTTTTTGAGAGTAC
AGAAGCAATTAAAATGCTGGAAGAAAGAGAAGCTCCTCAGAACTCCTTGA
>BogrAST2R12_NW_005393569.1:263962-264891
ATGGAGAGAACATTGAACAATATACTTACGATCATTTATGCTGGAGAGTTCTTACTGGGTATTTTGGGAAATGGATTCAT
TGTTCTGGTTAACTGTATTGATTGGATCAGGAGTAGGAAGTTCTCCCTGATTGACTTTATTCTCACCTGCTTGGCTATTT
CCAGAATATGTGTGCTGTGCATAATGATTTCAAGTACAAGTTTATATGTAATCTCTAAGGAAATACAGTACAATAAGAAT
CTCCTGATAAATTTGAGGTTCCTCTGGACAGGATCCAATTATTTCTCCATAGTCTGCACCACCTGCATCAGTGTCTTCTA
TCTCCTCAGAATAGCCAACTTTTCGAATTTCCTTTTCCTCTGGATGAAACGGAGAATTCACAAGGTGCTTCTCATTATTG
CACTGGGGGCTGTCTTCTCTTTCTGCTTGTGCCTTCTTCAAAAGGATGTGGTAGTTGAAAGCCGGCTCCAAAACCAGGTA
AACAGCGAAAACAATGTGACGTTGGACTTTCTAATGATAAAATATGATTTGTTCCTTACCATAATGTTCCTCATCCCCTT
TGTAGTGTCCCTGGCCTCCTTTCTCCTTTTAATCCTCTCCTTATGTGGTCATCTCAGGCGTATGAACGGTGTAGACTGTA
GCTCGGAGGCCCATGTGAGAGCCCTGAAGGCTATGATTTCATTCCTACTCCTCTTCGTTCTATACTATTTGAGCAATATT
ATAACTGTGTGGGCCAATCACATTCTAGGTAGTTTCGTGGCAAAGATTTTTGTGAACATGCTGTTATTTTTCTGTCCTTC

TGGCCATACTTTGCTTCTGATTTTGTGGAACAGCAAATTGAAACAGGCTTCACTCTGTGTCCTAAGGAAGCTGAAGGGTT
ACATGAATCTAAGAAAACCTGCTCTTCCAAAAAGAAGCCTGAAGCGATGA
>BogrTAS2R13_NW_005393569.1:246378-247289
ATGGAAGATTCCTTGGAAAACATCTTTATCATTTTAATAAATTCAGAATTCATAACTGGCATTCTGGGGAATGGGTTCAT
AACACTGGTGAACTGCATTGACTGGATCAAGATGCAAAAGGTCTCCTTGGCTGATCAAATCCTCACTGCTTTGGCAATTT
CCAGAATTGGTCTGATTTTGGTAATAATGGTGAGTTTGTTTACAAAGGAGTCTTATCCATCTTCATCTTTAGACATAAAG
GGAAATAAAGTCATACTTTTTAGTATTGCTGGGCTCTTGGCCAATCATTTTAGTGTCTGGCTTGCCACAGGCCTCAGCCT
CTTCTATTTCCTCAAGATAGTCAATTTTTCAAATGCTGTTTTTCTTCACCTAAAGTTTAGAATTGGAATGGTAGTTATGG
TAATGTTTCTGGGGACATTAGTATTGCTGCCTTTAAGTCTTACTCTGGTGAGCAGCTATATTAATATCAAGATACATCTG
TATGAAAGAAATACGACTTTAAATTCTAAAAGGCGTGACACTGAAACCTTTTCCAAATTAATTATATTCACCGTAGGATC
TTTCTTACCTTTTATTATATCCCTGAGTTGTTTTCTCCTGTTAATGTTCTCCCTACTGAAACATGTCAAGAAGATGAGGA
GCCATGCAACAGGATTCAGAGATCCCAGCAGCAAAGCCTACGTCAGAGCCATGATCATGGTGATATCTTTTCTCATACTA
CTTGCCATTCACTTCCTATCTCATCTCATGACAACTTTTCATCACAATGTGATACAGAGTGAACTGGCCTTTATGCTTGC
TGAAACTCTTGGAACTATTTACCCTTCAGTTCACTCATTTGTCCTGATTCTGGGAAATGACAAGCTAAGAAAAGCTTCAC
TTTTGGTGCTGTGGCAGTTGAGGTGTGGCTGA
>BogrTAS2R16_NW_005393954.1:362622-363527
ATGACAACCAGCCAACTCTCTGTCTTCTTCATGATCATCTATATGCTCGAGTTCTTGACAATAACTGGGCAGAGCAGCCT
GATTGTTGTAGCACTGGGCAGAGAGTGGGTGCAGACTCAAAGGCTGCCACCTGCGGACATGATTCTCATCAGCCTGGGCA
TCTGTCGCTTCTGTCAACTGTGGTCATCGATGCTGTACAACTTTGGTTCCCACTTCCACCCTAATTACAATTTTTGGTAT
TTCGGGATCATCTGGGAATTTACTAACATCCTTTCCTTCTGGTTGACCAGCTTGCTTGCTGTCTTCTATTGTGTCAAAGT
CTCCTTCTTCAGCCACCCCATCTTCCTCTGGCTGAAGTGGAGAATTGTGAGATGGGTTCCTCGGCTGTTGCTGGGCTCTC
TGCTGATTTCTTGTGTGTCTACCATATTTCCAGCTACTAGTTATTACATTGATATTCAATTCATCGCCATGAAGCATTTC
CCTAGAAACAGCACCATGCTTGAGAGACTTGAGGCGTTCCTGTGGGATTTTTCCACACTGCACAAAGTAGTTGTGTTGGT
TATTCCTTTCCTCCTGTTCCTGGCCTCCACAGTCTTGCTCATGGCCTTATTATCCCGACATCTGAAGCAGATGAAAGACC
TTCACACAGGCTGCTCCAACTCCAGCCCGGAAGCCCACTCTGCCGCCCTGAGGTCCCTTGCCATCGTCCTCATCTTGTTC
ACCTTTTATTTTCTCACCGTGCTCCTCTCCATATTGGATGTCCTATTTAATAAAGAGTCCTGGTTCTGGGCCTGGGAAGC
TATCATCTATGCATTCGTCTCTATTCATTCTACTTTACTAATGCTGAGCAGTGTCAAACTGAAAAGAGTTTTAAAGGCAA
GGTGCTGGAGCCTAGAAGCTGCCTGA
>BogrTAS2R408B_NW_005393569.1:197651-198568
ATGATAACTTTAGTATCGAGCATTATTTCCATTCTAATGGTGGCAGAATTTGTTCTGGGAAATTTTGTGAATGGTTTCAT
AGCACTGGTGAACTGCAATGACTGGCTCAGGAAACAAAAGTTCTCCTTAGCTGATGGGATTCTCACTGCTCTGGCAGTCT
GCAGAATTGTTTTGCTCTGGACAATATTAATAAATTGGTATGCAACTATGTATAATCCAGCTCTATATAGTTTAAGAATT
GTTATCCGTGTTGCCTGGACAGTAAGCAACCATTTTAGTAACTGGCTTGCTACTAGCCTCAGTATATTTTATTTGTTCAA
GATAGCTAATTTCTCCAGCCTAATTTTTCTTCACCTGAAGTGGAGAGTTAAAAGTGTAGTTCTCATGATGATTTTGGGGA
CTTCAGTGATTTTGTTTTTTCAAGTTGCAGTGTTAAGTATAGATGAGACTATTCAGACAAGTGAATATGAAAGAAACATC
ACTGAGAAGACCAAATTAAGGGACGTTTTACACCTTTCAAATATGACCCTGCTCACACTAACAAACTTCATACCCTTCAC
TATGTCTCTGGTATCTTTTCTGCTGCTAATCTTTTTCCTGTGGAAACATCTCAGGAAGATGCAGCTCAACGGCAAAAGAT
CCCAAGATCCCAGCACCAAGGTCCACATAAAAGCCATGCAAACTGTCATCTCCTTTCTTTTCCTGTTTGCCACTTACATG
CTGACTGTAATTTTAACAATTTGGAATTCTAATGAGCTGCAGAAGGAACTGGTCCAAATGCTTTTCCAGGCTCTTGCAAT
CACATATCCTTCAATACACTCATTTATCCTGATTTGGACAAACAGGAAATTAACACAGACCTTTCTGTCATTTCTGTGGC
AGCCAAGATGCTGGCTAAAAGTAAAAGGAACTAGGTAG
>BogrTAS2R38_NW_005396514.1:17559-18566
ATGGTGACTCTGACTCACATCGTATCTGTGCCCTCTGAAGTCAGGAATGCATTTCTGTTCTTTTCAGTCCTGGAGTTTGC
AGTAGGGATCCTACTCAACGCCTTCATTTTCTTGGTCAATTTCCGGGACCTGGTGAGGAGGCAGCCACTGAGCCACTGTG

ATCTTGTCCTATTGAGTCTCAGCCTCACCCGGCTTGTCCTACACGGGCTGCTCTTTCTGAAGGCCATCCAGCTTACTCAT
TTCCAGCGGATGAAAGACCCGCTGAGCTTCAGCTACCAGACCATCATCGTACTCTGGATGATCGTCCACCAAGCCGGACT
CTGGCTCACCATGTGCCTTAGTCTCCTCTACTGCTCCAAGATTGTCCGTTTCTCTCATGCCTTCCTGCTCCATGCAGCAA
GCTGGATCTCCAGAAAGATCCCCCAGATGCTTCTGGGTGCTATGGTTCTCTCCTGTGTCTGCACTCTTCTCTGCTTATGG
GACTTTTTTAGTGGATCTCATTTATCAGCTGTAACTAGGCTACTCATGAATAACAGTACTGAACTCAATTTGAACATTGC
AAAACTCAGTTTCTTTCATTCCTTCCTCTTCTGCAGCCTGGCATCCATCCCTTCTTTCTTGCTTTTCCTGGTTTCCTCTG
GGATGCTGGTGTTCTCCCTGGGGAGGCATATGAGGATGATGAGGGCCAAAACCAGAGGCTCTGGGGAACCCAGCCTGGAG
GCTCACACACGGGTGCTCAGGTCTCTTGTCTCTTTCTTCTGCCTGTATGTGCTGTCACTCTGTGCTGCCTTATGCTCGAT
ACCGTTGCTGACGCTGTGGCACAGCAAGGTCGGGGTGATGGTCTGCATAGGGATAATGGCAGCCTGTCCCTCAGGACACG
CAGTCATTCTGATCTCAGGGAATGCCAAGCTGAGGAGGGCTGTGGACACCATTCTGCTTTGGGCAAAGAGCAGCTTCAAG
GTAAGGGTGGACCACAAGGCAGATCCCAGGACGCCAGATCTGTGTTGA
>BogrTAS2R39_NW_005392839.1:179587-180630
ATGAGTGGGAGCTATCACAGACCAGCACACCAAGTGCTAAGGAGACATTTTCCTCTAGACATTGAAGAAAAGCAACCACT
CAGGATGATCCAAACCTGCAGTTCCTCAGAAAATGATCTGTCACCATCTCTTGTCACTTTGATGTTAATAATTATCGGCA
CGGAATGCATCCTTGGTATCCTCGCAAATGGGTTCATTGCAGGGATAAACACAGCTGAATGGATTCACAGTAAGGTACTC
TCCACCAGTGGCAAGATCCTGCTTTTCCTGGGTGTATCCAGAATAGTTCTACAAAGCTTCATGATGCTAGAACTTACCTT
AAGCTCAACATCCCCACAGTTTTATAATGATGACATCATGTATCACACATTCAGAGGATGTTTCATGTTCTTAAATCACT
GCAGCCTCTGGTTTGCTGCCTGGCTCAGTGTCTTCTACTTCGTGAAGGTGGCGGATTTCTCCTACCCCCTTTTCCTCAAG
CTGAAGTGGAGAATTTCCGGACTGATGCCCTGGCTTCTGCAGCTATCAGTGTTTGTTTCCTTGGGCCAGAGTGTGCTCTT
CTTCCAAAACAACTATACTATGAATTGTAACAATCTTTTTTCTCTCCCGTCCTTCAACTCTACTAAGAAAAAGTCCTTCG
CGGAGTCCACTGTGATCAACCTGGTTCTTTTCCTTAACCTGGGGATCTTCATCCCTCTGATCATGTTTATGCTGGCGGCC
ACCCTGCTGATCATCTCTCTCAAAAGACACATCTTCCACATGAAAAGCAACGCCACTGGCTCCAGAGACCCCAGCATGGA
GGCTCACCTGGGGGCCATCAGAGCCATCAGCTATTTTCTCATTCTCTATATTTTCAAAGTACTTGCTCTCTTTCTCTACA
TGTCCAACTTCTTTGACATCAATAGTCCCTTGAATATTTTGTGCAAAATCATCATGGCTACCTACTCTGTGGGCCATTCC
ATTCTACTGATTCAGGACAATCCTGGGCTGAAAAGAGCCTGGAAGAGGCTTCAGACTCAAGTTCACCTTTATTTTAAAAA
GTAG
>BogrTAS2R41_NW_005392839.1:480598-481515
ATGCATCCAGCATTCACAGTCCTCTTCATGCTGCTCTTTGTCCTGCTGTGTATCCTGGGCCTCCTGGCCAATGGCTTCAT
TGTGCTGGTGCTGAGCAGAGAATGGGTGCGACGTGGGAGGCTGCTCCCCTCTGACCTGATCCTCTTTAGCTTGGGACTCT
CCCGCTTCTGCCTGCAGTGGGTTGGAATGGGGAATAACTTCTACTATTTCCTGCATCTGGTCGACTACTGCAGTGGTCCC
GCCCGGCAGTTCTTTGGTCTACCCTGGGACTTCCTCAACTCTGTCACCGCCTGGTTTGGCTCCTGGCTCAGCGTCCTCTT
CTGCATGAAGGTTGCTAACTTCACCCACCCTGGCTTCCTCTGGCTAAAGTGGAGGTTCCCCAGGTCAGTGCCCTGGCTTT
TGCTGGGCTCTCTCCTCACCTCCTTCATTGTCACCCTACTGTTTTTTGGGGGGAACCACGCTTTGTATAAAGAGTCCTTC
ACTAGAAAACCTTTCGGGAATATGACCTACTATCAGTGGAACAGGATTCTGGAAATGTACTATTTCCTGCCCCTGAAACT
GATCACTTTTTCAATTCCTGGCTCTGTTTTTCTGGTCTCGATTGCTCTGTTGATTGACTCTCTGAGGAGACACGCATGGA
GGATGCAGCACAGTGCTCACAGCCTGCAGGATCCCAGTGGCCAGGCTCACACCAGAGCTCTGAAGTCACTAGTCTCCTTC
CTTGTTCTTTATACTCTGTCTTTCATGTCCCTGATCATCGATGGTGAAGGGTTCTGCTCCTCAGAGAGTGACTGGTACTG
GCCATGGCAAATTTTAACCTACTCGTGCACATCCATCCATCCCTTTATCCTCATCCTTGGCAACCTCAGGCTTCGGGGGG
CATTTGGGCAGCTGATTTTGTTGGCCAGGGGCTTCTAG
>BogrTAS2R408C_NW_005393569.1:223461-224303
ATGATAATGTTTATGTCAAACATTGTTTCCATTCTATTAATGACAGAATTTGTTCTGGGAAATTTTGCCAATGTCCTCTT
AGCACTGGTGAACTGCAATGACTGGACCAAGAGACCAAAGATCTCAGCTGATGGGATTCTCACTGCTCTGGCATTCTGCA
GAATTGTTATGTTCTGGGCAATGTTAATAAATTGGTATGTAATTGTGTATAATTTAACTCTATATAATTCAGAAGTAAAA
ATGATTGTTCATGTTGCCTTGACAGTAAGCAACCATTTTAGTAACTGGCTTGCTACTAGCCTCAGTATATTTTATTTGTT

GAAGATAGCCAATTTCTCCAGCCTAATTTTTCTTCACCTGAAGTGGAGAGTTAAAAGTGTAGTTCTCATGATGATGTTGG
GGACGTCATTGTTCTTATTTTTTCAAGTTGCAGTGTTAAGCATGGATGAGGCTATTCAGACAAATGAATATGAAGGAAAC
ACCACTCAGAAGATCAAACTAAGGGACACTTTACACCTTTCAAATGTGACTCTGTTCACACTAACAAACTTTATACCCTT
CACTATGTCCTTGACATCTTTTCTGCTGCTAATCTTTTCCCTGTGGAAACATCTTAGGCAGATGCAGCTCAATGGTAAAG
GATCCCAAGATCCCAGCACCAAGGTCCACATAAAAGCCATGCAAACTGTCATCTCCTTTCTTTTCCTGTTTGTTATTTAC
ATTCTGGCTCTAATTTTATCAGTTTGGAATTCTAATCAGCTGCAGAAAGAACCAGTCCAAATGCTTTATGATGTCATTTT
AATCATGTATCCTTCAATCCACTCATGTATCTTGATCTGGTGA
>BogrTAS2R408A_NW_005393569.1:191024-191941
ATGATAACTCTACTATCAACCATTTTTTCCATCCTAGGAATAATACAATTTGTTCTGGGAAATTTTGCCAATGGCTTCAT
AGCCCTGGTGAACTGCATTGACTGGGTCAAGAGACAAAAGATCTCCTCAACTGATGTGATTGTCACTGCTATGGCAGTCT
CCAGAATTGTTTTGTTCTGTGTAATGTTAATACATTGGTATTATATTTTGCTTCATCCAGCTTTATATAGTTTAAAAGTA
AGAACTATTTTTCATGTTGCCTGGACAATAAGCAATCATTATAGCACCTGGCTTGCTACTAGCCTCAGTATATTTTATTT
GTTGAAGATAGTCAATTTCTCCAGCCTAACTTTTCTTCACCTGAAGTGGAGAGTTAAAAGTGTAGTTCTCATGATGCTTC
TGGGAACTTCATTCATTTTGGTTTTACAAGTTGTAGTTATAAGCGTAAGTGGGACTATGCAGAGAAGTGAATTTGAAGGA
AACTTCACACAGAAGACCAAACTAAGGGATATTTTATGGCTTTCACATGTGACCCTGCTCATTCTAGGAAACCTCACACC
CTTTACTATGTTCTTAATATCTTTTCTGCTACCAGTCTTTTCCCTGTGGAAACATCTCAGGAAGATGCAGCTCAATGGCA
AAGGATTCCAAGATCCCTGTACGAAGGTCCACATAAAAGCCATGCAAACTGTCATCTCCTTTCTCTTGCTATTTGCCTTT
TACTTTCTGGTTCTAATCATATCAATCTGGAGGCCTAAAAAACTGCATGAGGAACCATTTCTCTTGCTTTTCCCAACAAT
CGAAGTCATCTATCCTTCAGTCCACTCATTTATCCTGATTTGGGGAAACAGAAAGTTAACACAGGCCTTTCTGTTGTTTC
TGTGGCAGCTGGGGTGCTGGCTGAAAGAGAGGAAATAG
>BogrTAS2R60_NW_005392839.1:450189-451136
ATGGTTCCTGGACCTCAGTTGGTTGATAAGACAGCCCTTGTCTGCATTATTATTTTATTCCTTTTGTTCCTGGTGGCATT
GGTAGGTAATGGCTTAATCATCATGGCACTGGGCAGCGAGTGGCTGCTGCAGAGAACGTTGTTGCCTTGCGATAAGTTAT
TGGTCAGCCTGGGGGCCTCTCGCTTCTGTCTGCAATGGGTGGTGATTAGTAAGAACATTTACATTTTCCTGAATCCCACG
GCCTTCCCATACAACCCCGTGTTCCAGCTCCTGGCCGTTCAGTGGGACTTCTGGAACTCTGCAACACTGTGGTTCTCCAC
CTGGCTCAGTGTCTTCTACTGTGTGAAAATTGCCACCTTCACCCACCCCGTCTTCCTCTGGCTAAAGCGGAATGTATCTG
GGTTGGTTCCTTGGATGCTACTCAGCTCTCTGGGGTTCTCTACCTTTACCACCGTTCTATTTTTCATAGGCAACCATAGA
ATGTATCAGAACTATTTAAAGAAGGGTCTGCAACCTTGGAATGTCACTAGGAATGCTGTGAGAACATATGAGAGGTTCTG
CCTCTTCCCTTTGAGAATTGTTACCTGGACCGTCCCTACTGTTATCTTTATTGTGGGCACAGTTTTGCTCATTACATCTC
TGGGAAGACACACCAAGAAGGTCTTCTTCTCTATCTCAGGCTTTCACAGTTCCAGCGCCCAGGCACACATCAAGGCTCTC
TTGGCTTTTATCTCCTTTGCTATCTTCTTCACTTCCTCTTTTCTGTCACTGGTTCTCACTGCCTCAGGTATGTTTCCTTT
TGGGGAATTCCGGTTCTGGATATGGCAGACTGTGATTTATCTGGGTACAGCAATCCACCCCCTTATTCTTCTCTTGAGTA
ACCGCAGGCTGAGAGCTCTGCTAGGGAGGGGAGGGAGGGGCTGCTCCTCAGCACATGGGGCATCTTGA
>BogrTAS2R67_NW_005393569.1:160661-161599
ATGCCATCTGGAATTGAAAACACTTTTCTAGCAGCAACAATAGGAGGATTCCTGATTGGAATTTTGGGGAATGGGTTCAT
TGTACTAGTTAACTGCATTGACCTGGTGAAGAGACAAAAGCTCTCATCAGCTGACTGCATCCTCACAGGCCTGGCTATCT
CCAGAATCAGTCAACTTTGGGTAATACTATGTGACTCATTTTTATTGGTACTATGGCCACACCTATATGCCATTGATAAA
CTAACAAAAGTTGTTAGTAGTTTTTGGATATTGTCCAATCACCTAGCTACCTGGTTTGCCACCTGTCTAAGTGTTTTCTA
CTTCTTTAAAGTAGCCAACTTCTCCCACCCCTGCTTCACTTGGCTGCGGTGGCGAATTCGTAGTGTGGTACTGGTGCTTC
TCTTGGGGTCTTTGTCCTTACTGTTTTTGAATTATGAATCAATATATACACTTAGTCATATCTTAACTAACAGCTACAAA
ATATATGTAAGAAACTCAACGTGGTCCTCAGATGTTAGTGAAACTCATTATCTTCACCAGTTGATTGTTTTTAACTTCAT
CAACTTAATCCCCTTTCTTCTGTCCCTGACCTCACTGCTCCTCTTAGTTCTCTCCTTGATGAGACACATCAGGAATTTGC
AGTTCAACCCCAGCTCAAAGGATCTCAGCACAGAGGCCCATAAAAGAGCCATGAAAATTGTGATGTCTTTCCTCTTCCTC
TTCATCATTCATGTCTCTTCCATCCTATTAATAGGTTGGGTTTTCCTTAAACTACAGGGACGTCTGGCCCAATTGGTGGT

TGTATTAACTTCGACTGTTTTTCCTTCAAGCCACTCCTTTATCCTAATTTTGGGAAATAGCAAGCTGAGACAGAATGCCT
TAGGACTACTGTGGTATCTTAACTGCCACCCGAAAAGAGTGAAATCTTTAGCTTCATAG
>BogrTAS2R372A_NW_005393569.1:335362-336288
ATGTCAAATATCATCAAATATGTTTTTTTGATCATTGAAATCTCAGAATTCATAACAGGAATTTGCGGAAATGGATTCAT
TGCACTAGTACTTTGTGCTGACTCTCTCAAAAGCAAGACTATCTCCTTGCTTGACTTCATCTTCACATGCTTGGCCATCT
CCAGAATTGGTATGGTATTCATACTTCTCCTGGATGGCATTAGAATAGTGTTCCATCCAGAAATATTAGATAGTCACCAG
GTAATAGAAGTAACTTTTGATTTCTTCTGGAATATGAGCAATTCCTTAGCTACCTGGTGTGCTGCCTGCCTCAGCATCTT
CTACTTCCTCAAGCTATCTAATTTTTCCCACCCCTTCTTTCTCTGGCTAAAATGGAGAAGAAATAGAGTTGTTTTCACCA
TTATGTTGGGATTCTGTCTCTCTTTGTTTTTTAATCTTCTGAACATAAAGTTCAATACTCTCAGGGTCAGTGACCATTTA
GAAATAGAAAACAACTTGACTTGGGAAAAATGCATGCCTAAAACACAGTACTACAGCAGTCAAATTCTCCTCCACCTGGG
ATCTCTCATCCCCTTGGCTGTGTCACTCATTTTATTTTTCCTGTTAATCTTTTCCTTATGGAGACATACCAGGCAGATGA
CACATCATGCCAAAGGATCCAAAGACCTCAACACAGGAGTTCTTGTGAGAACAAGAAATACTTTGACTTCTTTCATCATT
CTCTTAGTTGTGCACTATTTGGCTACATTCATGTTAACTTGGTTCTATTTCACACTAGAAAATGACATGACTTTTATTGC
TGCTCAGACTGTAGCATTTCTCTATCCTGCAATTCACCCTTTTATTCTGATTCTGGGGAGCAGGAAACTGAGACAGATTT
CTGTGAATCTGCTAAGGCAAATTGAATCCTGTGTCAAGCGATTGTAA
>BogrTAS2R4P_NW_005394292.1:194265-193376
ATGCTTCGAATAGTCTTTTTTTCTTCTGTCGTTGTCTCTGAAATTTTAACTTTTGTAGGACTCATTGTGAATCTCTTCAT
TGTAGTGGTCAGTTACAAGACTTGCATCAAAAGCCACAGGATCTCTTCTTCTGACAGACTCCTGTTCAGTTTGGGCATCA
CCAGATTTTTTATACTGTTACTGAATGTTGTTGTCATCATCTCTCCAAATGTGGAAAGGTCAGTCTCCTTATCCTATTTT
TTTCTGTCATGTTGGATGTTTTTGGACTGTAGTAGTCTTTGGTTTGTAACCTTGCTCAACGTCTTGTATTGTGTGAAGAT
TGCTAACTACCAACACTCAGTGTTTCTCTGCTGAAACGAAATCTCTCCACCAAGATGCCCCGGCTGCTGCTGGTCTGTAT
GCTGCTTTCTGTCTTCACCACTCTCCTGTATGTTATGCTCAGACAGTTGGCACCCTCTCTTGAATTTGTGACTATGAGAA
ATGGCACAGTATTTGACATCAATGAGGGACTCTTGTCTTTGGTGACTCCTTTGGTCTTGAGCTCATTTCTCCAATTCATC
ATTAATGTGACTTCTGCTTCTTTGTTAATCAATTCCTTGAAGAGACATATACAGAAGATGCAGAGAAGTGCCACTGTTCT
TTGGAATCCCCAGACTGAAGCTCATGTGGGTGCTATGAAGCTGATGATCTGTTTCCTCGTACTCTACATTCCATATTCAG
TTGCTACCCTGGTCCATTATCTCCCTCCTTCTATAGGGATGGATTTGAGAACCAAGTCTATTTATGTTATTATGTCCACC
ATTTACCCTCCAGGACATTCTCTTCTTATTATTCTCACACATCCTAAACTGAAAACAAAAGCAAAGAATATTCTTTGTTT
CAGTAAATAG
>BogrTAS2R5P_NW_005394292.1:182424-181545
ATGCCCTCTTCTATCCTAGGACTGCTGATGCTGGTGGCAGTAGCTGAATCTCTCATTGGCCTCATTGGAAATGGAGTTCT
TGTGGTCTGGAGTTTCGGAGAATGTCTCCAAACGTTCAGGGCGTCCTCGTATAACCTCATTGTCCTGGGCCTGGCGGTCT
GTCGGTTGCTTCTACAATGGTTGATTATGGTGGACTCAAGTCTGTTCGTGCTTTTCCAGAGCAGCCATTGGCTTCGCTGG
CTCAGTGTCTTCAGGGTTCTGGTAAGCCAGGTCAGCCTGTGGTTTGCGAGTTTTCTCAGTGTCTTCTATTGTAGGAAGAT
CATGACCGTTGAACACCCTGTCTCTTTGTGGCTGAAGCAGAAGGCCTGTTACCTGAGTTGCTGGTGCTTTCTGGTGTACT
TCACGATCCATTTGTTACTTACAGTCAGGGGTAGCTTAGACTTCTCCAGTCCTTCCCAAGGAAACAGCAACATCTTATTC
CCCATTTCAAACTGGCACTATATATGTATATTACAGCTCAATACAGAAAGTATGATGCCTTTCACGATGTTTCCTGTTTC
CTCTGGGCTGCTGTGTCTCTTTGTATAGACACTACAGGAAGATGAAGGTCCATACAGCCGGCAGAAGAGATGCTCAGGCC
AAGGCTCATATCACTGTCCTGAAGTCCTTGGGCTGTTTCCTTGTACTTTACATGGTCTACATCCTGGCCAGCCCCTTCTC
CATCAGCTCCAAGACTTTTCCTGCAGATCTCTTCACTGTCTTCATCTCTGAGACACTCATGGCCACCTACCCTTTTCTTC
ATTCTGTCATACTGATCATGGGGAACCCCAGGATGAAGCAGGCATGTCAGAGAATCCTGTGGAAGACTCTATGTGCTTGA
>BogrTAS2R8P_NW_005393569.1:375278-376054
ATGTTCAGTATAGAAGACCACATCTTTCTGACCATAACGACTGCATAATTCATCATAGGAATGTTTGTGAATGGATGCAT
TGGACTAGTAATATGTGTTGATTGGATTAAGAAGAAAAAGATCTCCATAGCTGACTACATCCTCACCAGTTTAGCTCTCT
CCAGAATGTATTTGCTTTGTGTAATGACACTCAACGGCACCATACTGGCACTCTACCCAGGTGTTTATGAAAATGAGAAA

ATAAAGGTAGTTCTTAATATCTTCTGGACATTCACCAACTACTTAAGTATGTGGTTTGCCACCTGCCTCAATGTCTTCTG
TCTCTTCGAGATAGCCAATTTCTCCCACCGACTTTTTCTCTGGCTGAAGTGGAGAATTGAGAGGGTGGTTCACTGGAGCC
TACTGGGGTCCCTGGCCATTTCCATGTTGATCAGCCTTATACAAGCAACGTTAACAAATTCTGAACTCATTCTGAACAGT
GAACTCATTTCTTTTCTTCCTTTTTGTTATGATTTTCTTAAAATTGCAAAACATAAAAGAAACGTCACCGAATTGTTCCA
TGTGAGTAAAATTCAATACTTCGACCCATTGACATTGTTTAACCTGTTTGCTATTATTCCATTTACTGTGTCATTGATCT
CATTTTTCTTTTTAATTACATCCCTGTGGAGATACAGTAAACAAATGAAATCCAGTGTTACAGGCTCCAGAGACTCCATC
ACAGAGGCCCACGTGGAGGCCAGGAAAACAGTGAACTCATTTCTTTTCTTCCTTTTT
>BogrTAS2R9P_NW_005393569．1:373395-374331
ATACCAGGTACAATGGAGGCAATATATATGCTCTTGATTACTGGCTCGTGGATGATAGGAATTTGGGGAAATCGATTCAT
TGTACTGGTAAACTACAGTGGCTGGCTCAAAAAGAGAGCTGTTTCCTTGACTGATGTCATCCTGGTCAGCCTGGCCACCT
CCAGAATCTGTTTTTTTGGTGTGTTATATATATGGATGGTTTTATTATGGTACTCTTTCCAGATACATACAGGCATGGTG
AGATGATGAACATTTTGGATATTTTCTGGACAACTTGCAATCATTCAACTGTCTGGTTTACTTTTTGCCTCAGCATCTTC
TATTTACTCAAGATAGCCAGTATATCCCACCCAGTTTTCCTCTGGCTGAAGCTGAAGATGAACAGGGTATCCTTGGGATT
CTTCTGATGTCCTTTCTCATCTCCTCAATTATTAGTGCTTTACTGAATAATGATTCATTTTATGACTTCAGAATCAATAA
TGAAGCAAACATTACGTAGGAATTCAAAGTAAGTAAAATCCCAACTGCTTTCAAATAGATTATCCTGAACCTGGAGGCTA
TGGTTCCCTTTATTCTTTGCCTGGTCTCATTTGTCCTTTTATTTTTCTCCTTACTTCGACACACCAAGCAGATGAAACTT
CATGCCACAGGGTCTAGAGACCCTAGCATAGAGGCCCACATGAGGGCCATAAAGACAATAGTCATCTTTCTGGCTGTTTT
CATTATGTACTATGTAGTTTTTCTCTTCGTAACATCTCGCTTTCTGAATCCTCATGGAAAATTGGAGTTGATGTTTGGTG
GCCTAACAGCTGTCATTTTCCCATTGAGCCATTTGTTCATCCTGCTAATGGGAAACAGCAAGCTGAGGGAGGCTTTTCTG
AAGGTTCTGGGGATTGTGAAGGGTTTCCACAAAAGAAGGAAATATTCTGTTCCCCAG
>BogrTAS2R10DP_NW_005393569．1:330026-330939
ATGGACTGGTTGGATCTCCTTGCAGTTAGTGAGTCAATATTGGGACTTTAAGGGAATGGATTTATTGGATTTATAAGCTG
CATTGATGGTATGAAAAACAAGAAGATCTCTACTATCAGCTTTATTCTCGCTGGCTTAGCAACTGCCAGAGTTTGCCTGA
TATGGACAATAGTTACTGATGGATTTTTAAAGTTATGCTCTCCAGATGTACATTCCTCTGGGGACCTAATTGAATATAAT
GGTTACTTGTGGATAGTGATGAATCAATCAAGTATCTGGTTTGCTACCTGCCTCAGCATCTTCTATTTCCTGAAGATATC
TAGTTTTTCCCACTGCATCTTTCTCTGGTTGAAGGGTAGACTCAACATGGTTGTTTTCCTTCTTTTGGGATGCTTGCTTA
TTTCATGGTTAGTTACTTTTCCACATTTTGTGAAGATTGTTAATGATGATAAAAGGAAAATAAAAACACAGTCTGGTCAA
TGGATATGCATAAAGGTGAACTCTTTGGAAAACAAATTTGGCTGCATCTTGGTGTCATTCTCCTTTTTATACAATACCTG
ATTATATGTGTCTTGTTGCTCACTTCTCTTTGGAGACACAACAGGAGGATGCAATCGAATGCCACAGGATTCAGTGACCC
CAGTACAGAAGCACATATCAAAGCGATGAAAGTCTTGGTGTCTTTTATCATCCTCTTTATCTTGAATTTTGTAGGTACTG
CCATACAAATATCAAGTGTGACAGTGCCTGAAAACAAACTGCTTTTTATTTTTGGTATGACAACCACAGTCCTCTATCTC
TGGGGTCACTCGCTTATCCTAATTCTAGGAAATAGGAAGCTCAAGCAAGCCTCTTTGAGAGTACTGAAGTCATTAAAGTG
CTGGGAAAAAGAGAAACTTCTCAGAACTCCTTGA
>BogrTAS2R11P_NW_005393569．1:276426-277319
ATGTTGAATATATTGGAGAAGATTTTCATGGTTGTGACTGGTGGGGAATTTATAATAGGAATTTTAGGGAATGGATTTAT
TGGACTCATAACTTGCATTGCTTGGATTAGAAATCAGAAGTTGAGCTTGCTTGACTTCATTCTTACTAGTTTGGCCTTTG
CCAGAATCAGTCAATTATGGATAACCACTGTCATGTTCTTTTCAATGATGTTCTATCAGGCAGGCTTTGGTACTGTGGGA
AGAAAATATATCTTTTTTTGTATCTGGATACTGACCAGTCACTCAAGCACTTGGCTTGCTACTTGCCTTGCTGTCTTTTA
TTTCCTGAAGATTGCCAGTTTCTCCCATCCTCCTTTCCTTTGGCTAAAATGGAGAATTAACAAGGTTGTTTTCATGCTTC
CACTGGTAACTGTGCCCTTCCTAGTCATAAGTTTTCCTTGGCCATATAATGTTGATGTCTTCTGGTGTCATGTCCAAAAG
ATGCATGAGAGAAATATGACTGAGTTATGCAATGTGAATGAATATCAAAATTTAAATTTTATTATTATCTTCACTATGGA
GTCCCTCCCCCCTTCTTTCTTTCCCTGATTTCCTTTCTCCTGTTGCTCCATTCTTTGTGGAAACACAAGAAGAACATTGC
ACAAACTGTCAGGGATTCCAGAGACCCCCGTGTTGAGGTCCATTCAGAGCCATGAAAACTGTGTTTTTCTTTCTCATGCT
CTTTGTCCTGTACCAATTTGGCCTTTTCATGACATTTGGGGGGCATTTTTTCCTACAGAACAAGCTGGTTGTGATGTTTG

GTTATATGTTAGGAATGCTGTATCCTTCAAGTCACTCATATGTTTTAATTTTTGGAAACAGTCAAATGAGGAAATTCTTC
TTGGTGATTCTTAG
>BogrTAS2R40P_NW_005392839.1:231383-232338
ATGGTGACGGTGAACACAGATGCGATGGATAAAAACACGACCAGGTTTAAGATCGTCTTCACCTTGGTGGTCTCTGCAAT
AGAGTGCCTCATTGGCATTGCTGGGAATGGCCTCATCACCGTCATCCATGGAGCCGAGTGGGTCAGAGGCAAAAGACTCC
CCATTGGACTGCATTCTGCTCATGCTGAGCTTTTCCAGGCTCTTGCTACAGATTTGGATGATGCTGGAAAACACGTACAG
TCTGCTGTTCTGGGTCATCTACAATGAAAAAAGAGTATACATACTTTTCAAAACCATCATCATGTTTCTGAACTACTCCA
ACCTCTGGCTTGCTGCCTGGCTCAATATCTTCTATTGTCTTAGAATCGCAAGCTTTACTCACCCGTGGTTCTCCGTGATG
AAAAGGAAGGTCATGTGGCTGATGCCTGGGCTTGTGAGGCTGTCCTTGTTCTTCTCCTTTTGCTCCAGCTTTCCCTTCTC
TAAAGGTATATTCAACGTGTATGTGAACAATTCCGTCCCCATCCCCTCCTCCAACTCCACTGAGAAGGTGTACTTCTCCG
AGACCAACATGGGCAACTTGGTTACCACCCTTTACCTGGGGATCTTCATCCCTCTGATCATGTCTATGCTGGTGGCCACC
CTGCTGATCATCTCTCTCAAAAGACACACCTTCCACATGAAAAGCAATGCCACTGGCTCCAGGGACCCCAGCATGGAGGC
TCACCTGGGGGCCATCAGAGCCATCAGCTATTTTCTCATTTTCTACATTCTCAATGCAGTTGCTCTGTTTTTTTTCCATA
TCCAACATCTTTGCCGCCAACAGCTCCTGGAATATTTTATGAAAAATCATCATGGCTGCCTACCCTGCTGGCCACTCAGT
GCTACTGATCTTGGGCAACCCTGGGCTGAAAAGGGCATGGAAGCAGTTTCAGCACCAAGTTCATCTCTACCTGTAA
>BogrTAS2R42P_NW_005393569.1:151341-152266
ATGTTCCCTGGGTTGAGTACCATCTTTCTGATACTATCAGGAGTGGAATTCTTAATCGGAATTCTAGGCAATGTGTTCAT
TGGACTGGTACTCTGCTCTGAATGCGTTAAGAATCAAAAGACATCTTTATTTGACTTCATTCTCACTGGCTTGGCTATCT
CCAGAATCAGTCAACTGTTGGTGTTTTTTGTGGAGTCACTTATGATGGGACTAGATTCACAGGTATTTGCCATTTTTAAA
CTAGCAAAACCCATTACTTTACTTTGGAGAATATCTAATCATTTGACTACCTGGCTTGTCACCTGTCTAAGTATTTTCTA
TCTCCTTAAGATAGCTCATTTCTCCCACTCTCTTTTTTTCTGGCTGAAGTGGAGAATGAACAGAGTCATTCTTGCGATGC
TTGCATTTTCTTTGGTCTTTCTGATTTTGGATATTCTTTTGCTAGAAACATTTAATGATCTCTTCTGGAATTTAATAAAT
GAAGGCAACTGGACTTTAGTTGAAAGTAAAACTCATTATATTAAAAGCGAGAGTCTTCTTAGTTTCTCCTATTTCATTCC
TATTGCTCTGTCCCTGCTCTCATTTTTTTATTTTGGTCCTTGGTGAAACACACCAGAAATCTGCAGCTCAATTTTATGGG
TTCCAGGGACTTCAGCACAAAGGCCCATAAAAGAGCCATGAAAATGGTGACATCATTCCTCCTCCTTATCATGGTTCATT
TTCTTTTTACACAATTGGCAAATTGGATGTTTCATAGGTTTTTGGACAAGAAGTTCACAAAGTTCATCATCTTAGCACTA
TATGTCTTTCCTTCAGGCCACTCGTTCATGTTGATTCTGGGAAATAGCCAGTTAAGACAGATAGCCTTGAAGGTACTGAA
GCATCTTAAAAGCTCCTTGAAAAGACAAAATCCATTGGCTTTATAG
>BogrTAS2R408DP_NW_005393569.1:182650-183588
ATGATATCTTTACTATGGACCATTTTTTCCATCCTAGTATTAACAGAATTTGTTCTAGGAAATTTTGCCCATGGCCTCAC
AGCACTGGTGAACTGCATTGATTGGGTCAAGAGACAAAAGATCTCCTCAGCTGATGGGATTCTCACTGCTCTGGCAGTCT
GCAGAATTGTTTTGCTCTGGGTAACGTTAATGAATTGGTACTTAGTTGTGTTGAATCCAGTTCTATATAGTTTAAAAGTA
AGAATTATTGTTCATATTGCCTGGATAGTAAGCAACCATTATAGCACCTGGCTTGCTACTAGTCTCAGCATATTTTATTT
GTTGAAGATAGCCATTTCTCCAGCCTAATTTTTCTTCACCTGAAATAGAGTTAAAAGTGTCATGCACATAATACTTTTGG
GAACTTCATTCTCCTTGGTTTTTCATGTTGCAGTGATATACAACGATGAGGCTATCCAGACAAATGAATACAAAGGAAAC
ATTCCTCAGAAGACCATATTGAGGGGCAGTTTATGGCTTCCACATATGACTCTGCTTATGCCAGGAAATCTCATATGCTT
TACTATTTTAATCTCATATGCTTTACTATGTCCCTGACATGTTTTCTGCTATTAACTGTTTCCCTGTGGAAACATCTCAA
GAAGATGCAGCTCAGTGGTAAAGGATCTCCAGATTCTAGCACCAAAGTCCATATAAAAGCCATGCAAACTGTGATATCCT
TTCTCTTGCTGTTTGCCATTCATTTCCTGGCTCTAAATGGGATCCATTTGGAGTTTTAAAAGGCAGCAGAAGGAAACTGT
CTTTTTGTTCTTTGAGGCTCTTGGATTCCTCTATCCTTCAAACCACTCATGTATCCTGATTTGGGGAAACAGGAAGTTAA
CAAAGGCATTTCTGTCATTTCTGTGGCAGCTAAGGTGCTGACTGAGAGAAAAGAAATAG
>BogrTAS2R408EP_NW_005393569.1:239989-240866
ATGACAACTTTACTACATTTTTTCCATCCTAGTAATAGAATTTATTCTAAGAAATTTTGCCAGTGGTTTCATGTCACTGG
TGAACTGCATTGACTGGTCAAGAGACAAAAAATCTCTTCAGCAGATGGGATTCTCACTGCTCTGGCAGTCTCCAGAATTG

GTTTGCTCTGAGTAACATTAATACATTGGTGTGAAAATGTGTTTAATCCCAGCTTTAGACAATTTAAGAGCAAGAATTAT
TATTATTGCCTGGATAATAAGCAACTGTTTTGGTACCTGGCTTGCTGCTATCCTCAGCATATTTTATTTGCTCAAGATAG
CCAATTTTCCAATATTATTTTTCTTTACCTAAAATGGAGAATTAAAAATGTTCTTCTTGTTCACTTTGTCCTGTTTGGTT
TTATTAATTCATGGTGTAAATGTAAACAAGACTATCCAGACAAATGACTATGAAGGAAACATCACTCAGAAGACCAAGCT
GAGGGACATTTTACACCTTTGAAATATGACTCTGTTCGTGCTAGTAAACTTCACACCCTTTGCTATGTCCCTGAGTTCTT
TTCTGCTGTTTATCTTTTCCCCATGGAAACATTTCAGGAAGATGCAGCTCAGTGGTAAAGGACCCCAAGATCCCAGCACC
AAGGTCCATATAAAAGCCATGCAAACTGTCATCTGTTTTCTATTTGCCGTTTGCTTCCGGGTTCTAATTTTCTGTTTGAA
GTTCTAATAGGCAGTGGAACAACTTGGTTATCATGGCATGCCAGGCTTTTGGAATCATATGTCCTTCACTTATCCTGACA
TGGGGAAAGCAGACACTAGGACAGTCCTTCCTGTCATTTCTGCAGCAGCTGAGTTGATGGCTAAAAGAAAGGAAATAA
>BogrTAS2R62P_NW_005392839.1:440602-441512
ATGTTGATATTCAAGGTCATCTTTTTCCTGGAGTCATTGGTTGCTGTGCTGCAGAATGGCTTCATAGTTACTGTGTTGAG
CGGGGAGTGGGTGTGAAGCCAGATGCTGCCCGCTGGTGACATGATTGTGACCTGCCTGGCTGCCTCCTGGTTCTGTCTGC
ATGGGATGGCCCTCCTGAACAACATCATGGCCTCTTCTGGCTTTTGTTCCAAAATCGACTATTTCAGCATCCCCTGGGAT
TTCATCAACTGCCTCAGTTTCTGGCTGACTGCCTGGTTTGCTGTCTTCTACTGCGCGAAGATCTCCCTCTTCTCTCATCC
CCTCTTCTTCTGGATAAAGTGGAGGATTTCTCGGTCGGTTCCCCAGCTGGTGCTGGGCTCCTTGATCTTATCTGGTCTGT
CCGTCATCTCAGCTGCTGGGAATACAATTCTTGCCCAGATGACGGCTGCCCAGATTTCCCATGGAAACGACACCCTGGCT
GGTAGAATACATGCTACCTATTTGCACTTTTCCTACCTCATGTAATTCTCATGTGGTTGGTTCCATTCTTCCTGTTCCTG
GTGTCCACCCTCTCGCTCATGTTCTCACTGCGCCGGCACCTCTGGCAGATGCAGGACCACAGACCCAGCCCACGTGATCC
CAGTACCTGGGCTCATACCATGGCCCTGACGTCACTTGCCTTCTTCCTCATCTTCTACACCTTGTACTTCCTGTCCCTGG
TTATCATTATGTACATCCCAGCCCTCCGGGAACACTGGCACTGGGCCTGTAAGGTGGTGACCTACACGGGCATCTGTCTG
CACTCCAGCATCTTGGTGCACAGCAGCCCCAAGATGAGAAAGGGCCTGAAGAAGAGGCTTTGGCGAGCCCTGGACAAGGA
CCAGTTTGTCTCCAGTTATCAGTATCAATAG
>BogrTAS2R18AP_NW_005393569.1:227779-228666
GTGTCCGACTCTGTGCGACCCCATAGATGGCAGCCCACCAGGCTCCCCCGTCCCTGGGATTCTCCAGGCAAGAACACTGG
AGTGGGTTGCCATTTCCTCCAACAATGCATGAAAGTGAAAAGTGAAAGTGAAGTCGCTCAGTCGTGTCCGACTCTTACCG
ATGCCATGGACTACAGCCTACCAGGCTCCTCTGTCCATGGGATTTTCCAGGCAAGAGTACTGGAGTGGGGTTGGGCACTA
ACAAATCACTTAACTATCTGGTTTGCCATCTGCCTAAGCGTATTCTACTTCCTTAAGATAGCCAATTTCTCTCACTTCTT
TTCCATGTGGCTGAAATGGAGAATGAACCGAGTGCTTCTGGTGCTTTTCCTGGCCTTTTTCTTTTTATTATCTTTTGACC
TCTTAATGCAGGATGCTCTTGGTGAGCTGTGGATGAACACCTTTAGAGAACCTGAAAGGAACATGACTTTGCATTTAGAT
GCAAGTAAAATTTTCTATCTTAAAAGCCTTATTCTTCTCAGATTGACATATGTTATCCCTTTTCTTCTCTCCTTGGCTTC
TTTCCTGCTTTTCTTTCTTTCCCTGGTGAGACACATCAAGAATTTCCAAGTCAACTTGAACCACTTGAGAGACTTCAGCA
CAGAGGCCGATAAAAGGGCCAAGAAAATGGTGACAACATTTCTCCACCTTGTCATCGTTTACTTTATTTCCATTCTAATT
GGAAATTGGATCTTCTTTAAGCTACACTGGTATGAGGTCATGATGTTTGTCATGGTGATTCCAACTCTCTTTTCATCCGG
CCACTCGTTTGTTATAATTTTGGGAAACAGCAAGCTAAGGCAGATTGCTTTCAGACTATTGTGGGGTCTTAAGTTCTCTA
AAAACTAA
>BogrTAS2R18BP_NW_005393569.1:202709-203651
ATGTCAGTTGGAATGAAGGGCTCTTTCTACTAGTGGCAACAGGAGAACTCATCTTAGGAGTGCTGAGAAATGGGTCAAGA
ATAGGAAAGTCTCATCAGCTGGTTTCATCCTTACCTGCTTAGCTGTGGAGAGAATCATTCAAATGTGGGTAACACTATTG
GGTTCATTTACAGCGGGGCTATTTTCACATCTGTATGCTACCAGCAAACTAGCAGAGGTGATTACTCTTTTTTGGGCACT
AACGAATCACTTAACTACCTGGTTTGCTAACCCCTAAGTGTGTTCCATTTCTTTAAGATAGCCAATTTCTCTCATTTCTT
CTTCACATGGCTGGAGTGGAGAAGGAACAGAGTGATTCTTATACTTTTCCTGGGCTCTTTGCTCTAACTGTCTATTAACC
TCTTAATGCTCTTGGTGAGTTGTGGATGAGTAGCTATAGAGAGCCTGAAAGAAACACAGCTTTGCATTTAGATGCAAATA
AAATTTTCTATCTTAGATGCCTTATTCTTCTTAGCTTGACCTATGTTATCCATTTTCATCTCTCCCTGGCCTCTTTGTGC
TTTTATTTTTCTCCTTGGTGAGACACACCAAGAATTTCCAACTCAACCTGAATGGCTCAGAAGACACCAGCTCAGAGGCC

CATAAAAGGACCGTGAAAAGGGTGACAACCTTCCTCTTCCTGTTCATCATTTACTTTTTCCACTCCATTAGGGAGCTGGA
TCTTTCTTAAGGTACAGCAGTATCAGGCCATGATGTTTGTCATGAAGATTTCAACTGTCTTCACTTTGGGTCCTCTTATG
TTTTAATTTCGGGGATTAGCAAGCTAAGAAAGATCACCTTGAGTTTAATTTTGAATCTTATATTCTCTTTGAGAAAACCA
AGATCATTAGTTTCATGGACAGAATTTAAATGTACTTTATGTATTCTGGAGAAAATGCCTTAA
>BogrTAS2R372AP_NW_005393569.1:317008-317958
TCAGCAGTGACCAAAGTAACCAGGATTTCACTTCTGCAGTCATGTCCAGTGTAATCAAAAGATTTTTATGATCATTGAAA
TTTTATAATTCATAACAGGAATAACTGTGCTGACTTTGTCAAAAGCAAGAATAGTGCCTTGTTTGACTTCATCTTCACAT
GGATCAGCGTGATGTTCATACTTCTCCTAGATTGCATTAAACTAGTGTTCCATCTAGAAATATTAGATGGTCACCAGGTA
ATAAGAGGAGTTTTTGAGTTCTCCTGGAATCTGAGAAACTCATTAAGTACTGGATGTGCTGCCTGCCTCAGTGTCTTCTA
CTTCCTCAAGCTATCTAGTTTTTCTCACCCCTTCTTTCTCTGGCTGAAGTGCAGAAGAGATAGAGTTGTTTTCATCATTA
TGTTGGGATTCTGTCTCTTTTTGATTTTTAACCTTCTGAGCATAAAATTTATACTTGTGTGTTCAGCAAGCATTTAGAAA
AGGAAAGACTTAACTTGGAAAAAAGATATGCATAAAAATCAGTATTATAACAGTCAAGTTCTCTTCAGCCTTGGATCTCT
CATCCCCTTGTCTGTATCACTCATTATATTTTTCCTGTTAATCTTTTCCTATGGGGACATACCAAGCAGATGACATGCCA
TAACACAGATCCCAGGGACTTCAATGCCGGGAGCCACCACGGGAGATCCCACCCATGACAAAGGTCATGCGGAAGAGACC
TGACAGGCAAAGGAGGATCAGGCCTCAAGGGACCCCCTGAATCTTCTTGAGCATCTACCCCAAAACCAAAATCTGTCTAC
TGTTTATTATATTATGCCTTTCACCAACTCTTCTGTCATTAACAGGGGGCTATCCCCAACTAACCTCAGATATGCAGATG
ACACCACCCTTATGGCAGAAAGTGAAGAGGAACTCAAAAGCCTCTTGATGAAAGTGAAAGAGGAGAGTGAA
>BogrTAS2R372BP_NW_005393569.1:296308-297044
ATGTCAAGTGTAATCAAAAAAAGTTTTTATAATCATTGAAATCTTAGAATTCATAACAGGAATTTGCAGAAATGAATTCA
TTGCACTAGTACTCTGTGCTGACTCTCTCAAAAGCAAGAATGTCTCCTTGTTTGACTTGATCTTAACTTGCTTGGCCGTC
TCCAGAACTGGCATGATATTCATAATTTTCTTGGATGGCATTAGAATAGTGTTCTATCCAGGAATATTTGAAAGTCATCA
GATAATAGATGTAATTTTTTATTTCCCATTCTGTTGGGATTCTGTATTTCTTTGATTTTAATTTTCTGAGCATAAGTTTT
CATACATTTGGGGTCAGTGACCATTTAGAAATAGAAAACAACTTGACTTGGGAAAAATATGCATAAAATCCGATCCTATA
GCAGTCAAATTCTCCTCCACCTGGGATCTCTCATCCCCTTGGCTGTGTCACTCATTTTATTTTTCCTGTTAATCTTTTCC
TTATGGAAACATACCAGGCAGATGACACGTCATGTCAAAGGATCCAGAGACCTCAACACAGGAGTTCTTGTGAGAGCCAG
AAATACATGACTTCTTTCATCATTTTCTTAGTTGTGCACTATTTGGCTACATTCTTGTTAACTTGATCCTGTTTCACACT
AGAAAATGAAGTGACTTTTATTGTTATTAAGTCTGTAGCATTTCTCTATCCTTCAATTCACCCTTTTATTTTGATTCTAG
GAAACGGAAAACTGAGA
